# Gene Signature of Human Oral Mucosa Fibroblasts: Comparison with Dermal Fibroblasts and Induced Pluripotent Stem Cells

**DOI:** 10.1155/2015/121575

**Published:** 2015-08-03

**Authors:** Keiko Miyoshi, Taigo Horiguchi, Ayako Tanimura, Hiroko Hagita, Takafumi Noma

**Affiliations:** Department of Molecular Biology, Institute of Health Biosciences, The University of Tokushima Graduate School, 3-18-15 Kuramoto-cho, Tokushima 770-8504, Japan

## Abstract

Oral mucosa is a useful material for regeneration therapy with the advantages of its accessibility and versatility regardless of age and gender. However, little is known about the molecular characteristics of oral mucosa. Here we report the first comparative profiles of the gene signatures of human oral mucosa fibroblasts (hOFs), human dermal fibroblasts (hDFs), and hOF-derived induced pluripotent stem cells (hOF-iPSCs), linking these with biological roles by functional annotation and pathway analyses. As a common feature of fibroblasts, both hOFs and hDFs expressed glycolipid metabolism-related genes at higher levels compared with hOF-iPSCs. Distinct characteristics of hOFs compared with hDFs included a high expression of glycoprotein genes, involved in signaling, extracellular matrix, membrane, and receptor proteins, besides a low expression of HOX genes, the hDFs-markers. The results of the pathway analyses indicated that tissue-reconstructive, proliferative, and signaling pathways are active, whereas senescence-related genes in p53 pathway are inactive in hOFs. Furthermore, more than half of hOF-specific genes were similarly expressed to those of hOF-iPSC genes and might be controlled by WNT signaling. Our findings demonstrated that hOFs have unique cellular characteristics in specificity and plasticity. These data may provide useful insight into application of oral fibroblasts for direct reprograming.

## 1. Introduction

Oral mucosa is a convenient cell source for regenerative medicine, having the following advantages: (1) simple operation, (2) no cosmetic and functional problems after operation, (3) fast wound healing without scar formation [1], (4) nonkeratinizing epithelia, and (5) no need to consider age and gender differences. Practically, epithelial cell-sheets of human oral mucosa have been used as the grafting material for corneal and esophageal mucosal reconstructions after surgically removing damaged mucosal tissue in regeneration therapy [2, 3]. However, few studies have focused on human oral mucosa fibroblasts (hOFs) as material for regenerative medicine, and little is known about the molecular basis of their characteristics.

Recently, induced pluripotent stem cell (iPSC) technology has shown remarkable progress and has been applied to personalized medicine for diagnostics, drug screening, and regenerative therapy [4]. We also generated human iPSCs from oral mucosa fibroblasts (hOFs-iPSCs), and the excised area of the buccal mucosa was completely healed within a week without any scar formation, as expected [5]. So far, scarless healing is well recognized in fetal, but not adult skin [6]. Therefore, molecular events of the healing process have been studied by comparing postnatal (adult) and fetal skin tissues [7–12]. The differences between fetal and adult healing are strongly related to the production of inflammatory-triggered extracellular matrix (ECM), activation of growth factor signaling, and induction of epithelial-mesenchymal transition (EMT) [1, 10–12]. For example, fibronectin, type III collagen, and hyaluronic acid are more abundant in the fetal skin than in adult skin [1, 8, 11, 13–15]. Furthermore, antifibrotic tumor growth factor-beta3 (TGF-beta3) is highly expressed during fetal wound healing, whereas profibrotic TGF-beta1 and TGF-beta2 are low or absent [1, 7, 11]. These results suggest that skin fibroblasts are deeply involved in ECM deposition and remodeling. In the case of hOFs, higher activity of matrix metalloproteinase-2 (MMP-2) combined with decreased production and activation of tissue inhibitors of metalloproteinases have been demonstrated by comparing hOFs with skin fibroblasts during ECM remodeling [14].

So far, two comprehensive transcriptome studies have been reported using oral mucosa. One included the comparison of the expression profiles between skin and oral mucosal tissue derived from wound healing mouse models [16]. In this report, oral mucosa epithelial cells produced far less amounts of proinflammatory cytokines compared with skin epithelial cells. The other study compared cultured age-matched human skin fibroblasts with hOFs, showing that wounding stimuli induced cell proliferation and reorganization of collagenous environments in hOFs to a greater extent than in skin fibroblasts [17]. Based on these previous studies, we hypothesized that the sensitivity and plasticity of hOFs may explain their uniqueness and hiPSCs can be used as the alternative for fetal skin fibroblasts to compare the gene profiles.

Additionally, we previously found that endogenous* Krüppel-like factor 4 (KLF4)* and* v-myc avian myelocytomatosis viral oncogene homolog (c-MYC)*, which are the reprogramming factors for generating iPSCs, and* maternally expressed gene 3* (*MEG3*), which is an imprinted gene and long noncoding RNA, were highly expressed in hOFs [5].* Meg3/Gtl2* is located within the* delta-like 1 homolog 1 (Dlk1)-deiodinase, iodothyronine type III (Dio3)* region and the activation of this region is associated with the level of pluripotency in iPSCs or ESCs [18]. These findings may exhibit a part of plasticity in hOFs.

In this study, we performed comparative analyses of gene profiles of hOFs, hDFs, and hOF-iPSCs to understand the molecular characteristics of hOFs. We chose hOFs derived from the buccal region, not other regions of oral mucosa (gingiva, palate, and tongue) because of its superior accessibility as a cell source appropriate for future regenerative medicine. hOF-iPSCs were used as not only the alternative for fetal skin fibroblasts, but also pluripotent stem cells to find out the specificity in the gene signature of hOFs.

## 2. Materials and Methods

### 2.1. Human Fibroblasts

hOFs were isolated from individually collected buccal mucosal tissues obtained from four healthy volunteers (26–35 years old) after receiving written agreement including an informed consent at the Tokushima University Medical and Dental Hospital. Approval from the Institutional Research Ethics Committee of the University of Tokushima was obtained (Project number 708). Details on hOFs isolation have been described previously [5]. After isolation, hOFs were individually designated as hOF1 to hOF4. Among them, we failed to establish primary cell culture from hOF1, so we used three successful cell lines, hOF2, hOF3, and hOF4, for further experiments.

hOFs (hOF2, hOF3, and hOF4) were cultured in Dulbecco's Modified Eagle Medium (DMEM; Nissui, Tokyo, Japan) supplemented with 10% FBS (Nichirei Biosciences, Tokyo, Japan). Three types of hDFs derived from individuals aged 33–36 years old were purchased from the Health Science Research Resources Bank (TIG110, TIG111, and TIG114; Osaka, Japan). hDFs were cultured in Eagle's MEM (EMEM; Nissui) supplemented with 10% FBS (Nichirei Biosciences).

### 2.2. Generation of hOF-iPSCs

hOF-iPSCs were generated as shown previously [5]. Briefly, mouse* solute carrier family 7, member 1 (mslc7a1)*, was introduced into hOFs using lentiviral infection. Then, four reprogramming factors,* POU class 5 homeobox 1/octamer-binding transcription factor 4 (POU5F1/OCT4)*,* KLF4*,* SRY (sex determining region Y)-box 2 (SOX2)*, and* v-myc avian myelocytomatosis viral oncogene homolog c-MYC*, were transduced by retroviral infection. Generated hOF-iPSCs were maintained in human ES medium (ReproCELL, Tokyo, Japan) supplemented with 5 ng/mL of basic fibroblast growth factor (bFGF) on SNL feeder cells. The pluripotency of hOF-iPSCs was confirmed by the expression of the pluripotent cell markers and by* in vitro* differentiation through embryoid body formation.

### 2.3. RNA Isolation

RNA samples were prepared from three individual samples in each group (hOFs, hDFs, and hOF-iPSCs; a total of nine samples). Total RNA was isolated using TRI Reagent (Molecular Research Center, Cincinnati, OH, USA), according to the manufacturer's protocol.

### 2.4. Microarray Analyses

Microarray analysis was performed as previously described [19]. In brief, GeneChip Human Gene 1.0 ST Arrays (Affymetrix, Santa Clara, CA, USA) containing 28,869 oligonucleotide probes for known and unknown genes were used to define gene signatures. First-strand cDNA was synthesized with 400 ng of total RNA from hOFs and hDFs or with 220 ng from hOFs-iPSCs using a WT Expression Kit (Affymetrix), according to the manufacturer's instructions, modified with additional ethanol precipitation. With cRNA obtained from the first-strand cDNA, the second-cycle cDNA reaction was performed. Resulting cDNA was end-labeled with a GeneChip WT Terminal Labeling Kit (Affymetrix). Approximately 5.5 *μ*g of labeled DNA target was hybridized to the array for 17 h at 45°C on the GeneChip Hybridization Oven 640 (Affymetrix). After washing, arrays were stained on a GeneChip Fluidics Station 450 and scanned with a GeneChip Scanner 3000 7G (Affymetrix). A CEL file was generated for each array. All microarray data from the three groups (nine samples in total) have been deposited in Gene expression Omnibus (GEO, http://www.ncbi.nlm.nih.gov/geo/) under GEO Accession number GSE56805.

### 2.5. *In Silico* Data Analyses

The data were analyzed with GeneSpring GX12.0 (Agilent Technologies, Santa Clara, CA, USA). The normalization and summarization of CEL files were performed by “Exon RMA 16” algorithm. After that, the signal values of probe sets were transformed to the value of log_2._ For the technological variability, we checked several quality controls including Hybridization Controls (provided by Affymetrix), Histogram, Profile Plot, Matrix Plot, 3D PCA, Pearson's correlation coefficient, and hierarchical clustering analyses following the standard protocols provided by the manufacturers. Among them, the results of Hybridization controls and Pearson's correlation coefficient were shown in Supplementary Figures S1B and S1C in Supplementary Materials available online at http://dx.doi.org/10.1155/2015/121575, respectively. Each value of Pearson's correlation coefficient is indicated as follows: 1 indicates perfect positive correlation between two samples, 0.80 to 1.0 indicates very strong correlation, and 0.60 to 0.79 indicates strong correlation. Expressed genes that showed a fluorescence intensity greater than 100 were further analyzed. Average gene expression level was calculated for three samples in each group and used for the comparison. To make the stringent criteria, several statistical analyses were performed. First, the data obtained from the differently expressed genes between the 2 groups were analyzed by one-way ANOVA and cut off with the corrected *p-*value (*p* < 0.05) according to Benjamini-Hochberg (BH) method. Furthermore, Tukey's honestly significant difference (HSD) test was used as the post hoc test, and the differently expressed genes between the 2 groups were extracted. Among 28,869 gene probes, 12,713 gene probes were left after one-way ANOVA and BH analyses (all data was *p* < 0.05, Supplementary Table S1). From these 12,713 gene probes, more than 2-fold differentially expressed gene probes were selected between the two paired groups.

Functional analyses were performed using the Database for Annotation, Visualization and Integrated Discovery (DAVID) v6.7 (http://david.abcc.ncifcrf.gov/) [20, 21]. Major biological significance and importance were evaluated by functional annotation clustering (FAC) tool in DAVID. To obtain enrichment clusters of functionally significant and important genes, FAC analysis was performed with the enrichment scores below medium stringency. Pathway analyses were conducted using Kyoto Encyclopedia of Genes and Genomes (KEGG, http://www.genome.jp/kegg/) pathway tools.

## 3. Results

### 3.1. Gene Profiles of Microarray Data

**Figure 1:**
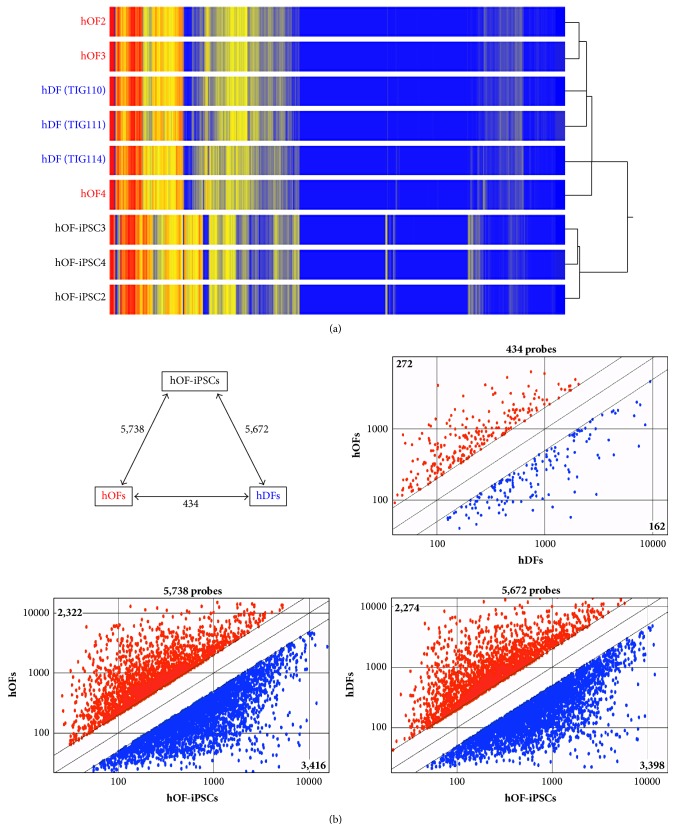
Gene expression signatures in hOFs, hDFs, and hOF-iPSCs. (a) Heat map and hierarchical clustering of whole microarray probes for each of the nine samples. Three individual samples were prepared from each of three types of cells, hOFs, hDFs, and hOF-iPSCs. (b) Comparisons of average signal values among the three types of cells, hOFs, hDFs, and hOF-iPSCs. The number indicates differentially expressed genes (*p* < 0.05, ≥2-fold change; upper panel, left). Scatter plots comparing the average signal values of three samples are shown and the number of differentially expressed probes at more than 2-fold levels is indicated as follows: hOFs* versus* hDFs (upper panel, right), hOFs* versus* hOF-iPSCs (lower panel, left), and hDFs* versus* hOF-iPSCs (lower panel, right).

To analyze the molecular profile of hOFs, we prepared three types of cells, hOFs, hDFs, and hOF-iPSCs. Three independent cell lines from different donors were chosen to obtain accurate results from each cell type. Heat map and hierarchical clustering analysis revealed that the gene expression pattern in each group was conserved, except for hDF3 (TIG114) and hOF4, for which intermediate patterns between fibroblasts and hOF-iPSCs were identified (Figure 1(a)). Notably, 56% of probes were expressed at the similar levels (16,156 out of 28,869 probes; less than 2-fold difference) among hOFs, hDFs, and OF-iPSCs. While our samples were not exact age- and gender-matched samples, we observed the strong correlation among the samples by Pearson's correlation coefficient analysis (Supplementary Figure S1A). Each correlation coefficient value among the samples in each group, and also that between hOFs and hDFs, was within the range between 0.9 and 1.0. Furthermore, each correlation coefficient value between either hOFs or hDFs and hOF-iPSCs was within the range between 0.7 and 0.8. These results indicated that our data may, at least in part, exclude the issues about age and gender difference with the strong correlation among the samples. The reliability of microarray hybridization techniques were confirmed by the company-supplied hybrydization control (Figure S1B).

Next, average gene expression signal values in each group were calculated and used for further comparative analyses. Figure S1C shows the scattered plot of gene profile comparison between hOFs and hDFs. Each gene expression of samples was indicated as a spot, and most of them were exhibited within 2-fold line (green line). Therefore, threshold can be set at 2-fold to find the difference of gene expression profile between hOFs and hDFs.

Out of 12,713 probes, we found that 5,738 probes and 5,672 probes (45%) were more than 2-fold differently expressed in hOFs and hDFs, respectively, compared with those in hOF-iPSCs (Figure 1(b), upper panel, left). Approximately 2,300 probes were highly expressed, whereas the expression of 3,400 probes was lower in both hOFs and hDFs than in hiPSCs (Figure 1(b), lower panel). In contrast, only 3.4% (434/12,713 probes) of differentially expressed probes were observed between hOFs and hDFs (Figure 1(b), upper, right). Among these, 272 probes had a high expression and 162 probes had a low expression in hOFs compared with the expression in hDFs (Figure 1(b), upper panel, right).

### 3.2. Enriched Pathways in Fibroblasts

**Figure 2:**
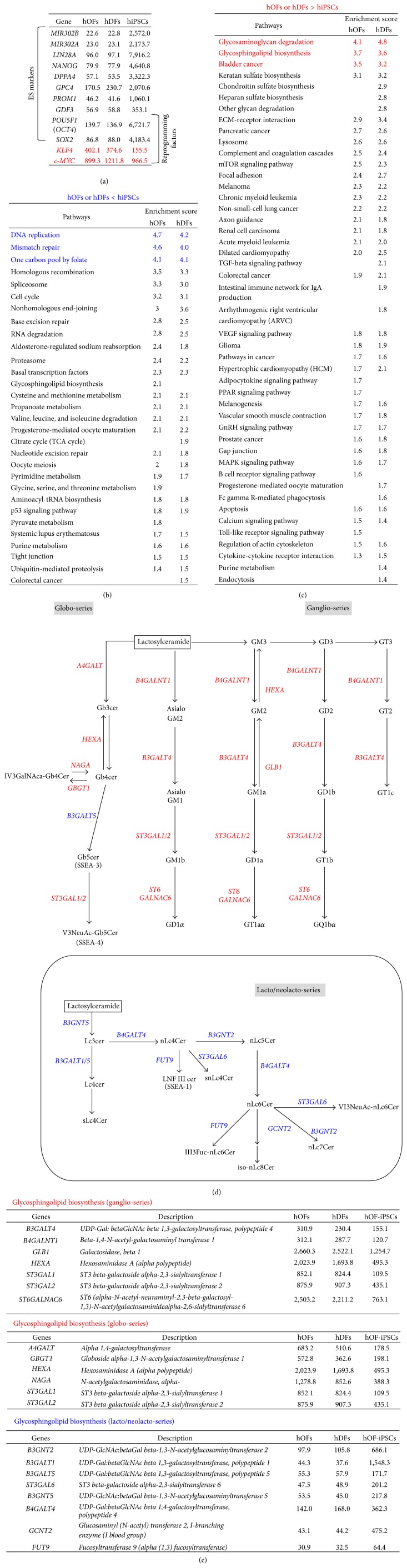
Pathway analysis of human fibroblasts and hOF-iPSCs. (a) Gene expression of ESCs markers and reprogramming factors. The numbers indicate average signal values in each cell type. Red: highly expressed genes in hOFs and hDFs compared with hiPSCs. (b) and (c) Pathways with low (b) and high (c) expression in human fibroblasts compared with those in hOF-iPSCs. Numbers indicate enrichment scores provided by DAVID. The top three clusters are colored. Blanks indicate “not listed” in the samples. The top three clusters are highlighted in blue (b) and in red (c), respectively. (d) A diagram of various GSL-biosynthetic pathways. Red and blue colors indicate genes with high and low expressions in human fibroblasts, respectively. cer: ceramide; Gb with subscript: globoside with the number of carbohydrates; G with subscript: ganglioside with subclass; Lc with subscript: lacto- with the number of carbohydrates; nLc-: neolacto-; Fuc: fucose; GalNAc: N-acetylgalactosamine; NeuAc: N-acetylneuraminic acid. (e) Individual gene-expression levels of each GSL-biosynthetic pathway in hOFs, hDFs, and hOF-iPSCs. Red and Blue indicate the same as in (d).

At the beginning, we confirmed the expression levels of several embryonic stem cells (ESCs) markers and reprogramming factors that had been generally observed in iPSCs (Figure 2(a)). As expected, hOF-iPSCs highly expressed all pluripotent markers tested for, that is,* micro RNA302a (MIR302A)*,* MIR302B*,* lin-28 homolog A (LIN28A)*,* Nanog homeobox (NANOG)*,* developmental pluripotency associated 4 (DPPA4)*,* glypican 4 (GPC4)*,* prominin 1 (PROM1)*,* growth differentiation factor 3 (GDF3)*,* POU5F1/OCT4*, and* SOX2*. We also found that the reprogramming factors* KLF4* and* c-MYC* were highly expressed in hOFs and hDFs than in hOF-iPSCs. These results were consistent with previous observations [5].

To elucidate the characteristics of hOFs, we first compared the gene profiles of fibroblasts (hOFs or hDFs) with hOF-iPSCs in steady-state condition. For prediction of the biological function of respective gene profiles, we matched functionally related gene groups to the known pathways by pathway analysis using DAVID linked with KEGG. Genes in thirty pathways were expressed at lower levels in hOFs and hDFs than in hOF-iPSCs, suggesting that these pathways are functionally active in hOF-iPSCs (Figure 2(b)). High expression groups in hOF-iPSCs represented pathways of energy metabolism (glycolysis and tricarboxylic acid (TCA) cycle), nucleotide metabolism (DNA replication, DNA repair, and spliceosome), cell cycle metabolism, and membrane lipid metabolism (Figure 2(b) and Supplementary Figure S2).

Conversely, 46 pathways were enriched among the highly expressed genes in hOFs and hDFs compared with those in hOF-iPSCs (Figure 2(c)). We found that the pathways of glycosaminoglycan (GAG) degradation, glycosphingolipid (GSL) biosynthesis, keratan and heparan sulfate biosynthesis, and lysosome metabolism were highly enriched in hOFs and hDFs. Among them, glycosyltransferases (GTases) in the globo- and ganglio-series of GSL biosynthesis pathways, but not GTases in the lacto- or neolacto-series of the GSL synthetic pathway, were highly expressed in hOFs and hDFs (Figures 2(d) and 2(e)). In addition to these, other signaling components, such as ECM-receptor interaction, complement and coagulation, mammalian target of rapamycin (mTOR) signaling pathway, focal adhesion, and signaling pathways of TGF-beta, mitogen-activated protein kinase (MAPK), vascular endothelial growth factor (VEGF), and calcium were enriched to a greater extent in hOFs and hDFs than in hOF-iPSCs (Figure 2(c)).

### 3.3. Characterization of hOFs in Comparison with hDFs

**Figure 3:**
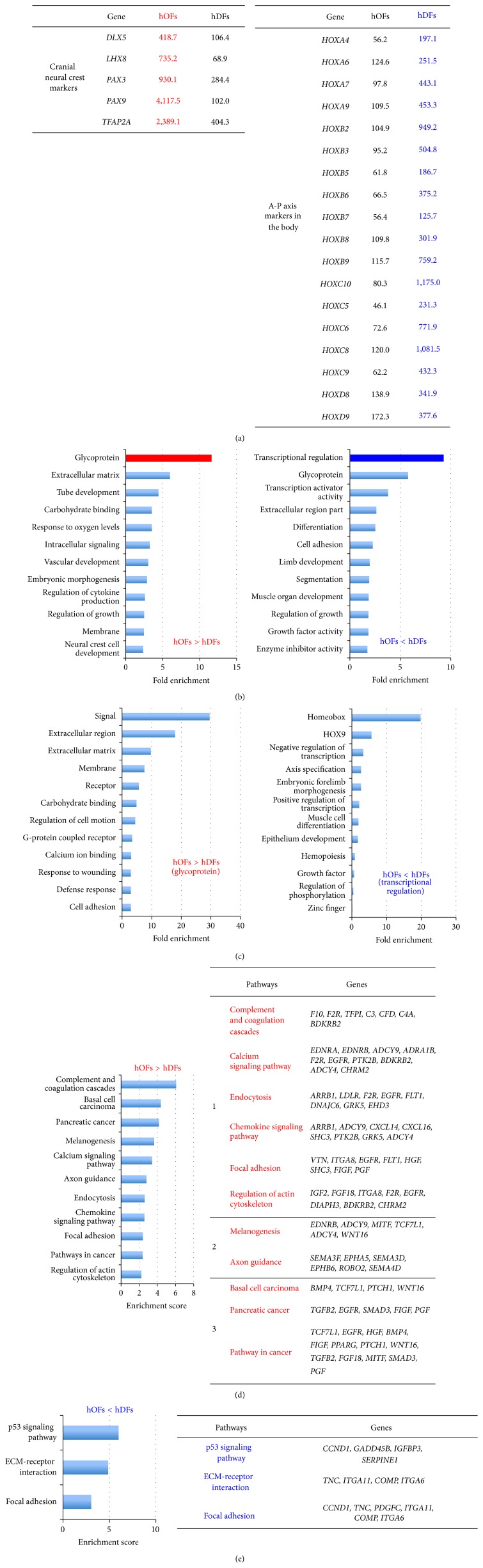
Comparison of gene profiles in hOFs and hDFs by functional annotation clustering (FAC) and pathway analysis. (a) The positional signatures of hOFs and hDFs as an internal validation. Gene expression of cranial neural crest markers for hOFs (left). Gene expression of anterior-posterior (A-P) axis markers in the body for hDFs (right). Numbers indicate the average signal values in hOFs and hDFs. (b) The top 12 clusters of FAC result in hOFs compared with hDFs. Red bar: the highest enriched cluster in hOFs > hDFs; blue bar: the highest enriched cluster in hOFs < hDFs. (c) The top 12 clusters of FAC result in the individual components of glycoproteins and transcriptional regulation in (b). (d) and (e) Pathway analysis results in genes with high (d) and low (e) expression in hOFs compared with hDFs. Indicated numbers in (b) to (e) represent enrichment scores by DAVID. The number in (d) indicates the three groups categorized in the text. The full names of each gene listed in (d) and (e) are shown in Supplementary Tables S2 and S3, respectively.

Since some of the expressed genes in both hOFs and hDFs must be shared in the biological pathways to display “fibroblastic” characteristics compared with those expressed in hOF-iPSCs, we next tried to elucidate the specificity between hOFs and hDFs. For this purpose, we analyzed a number of genes that were differentially expressed between hOFs and hDFs using microarray analysis, for which overlapping probes were designed and arranged within the same gene to obtain accurate results. Compared with hDFs, 232 genes were overexpressed in hOFs “hOFs > hDFs,” whereas 152 genes were underexpressed “hOFs < hDFs.” Cranial neural crest markers especially, such as* distal-less homeobox 5 (DLX5)*,* LIM homeobox 8 (LHX8)*,* paired box 3 (PAX3)*,* PAX9*, and* transcription factor AP-2 alpha (TFAP2A)*, were expressed at a remarkably high level in hOFs (Figure 3(a), left). On the other hand, hDFs expressed homeobox (HOX) cluster genes (Figure 3(a), right) to preserve their positional information as expected [22].

To understand the biological roles of highly expressed genes in hOFs, we performed FAC analysis using DAVID. One hundred and five clusters in hOFs > hDFs and 64 clusters in hOFs < hDFs were observed. The top 12 clusters are shown in Figure 3(b). The top three clusters in hOFs > hDFs were glycoprotein (103 genes), ECM (21 genes), and tube development/embryonic morphogenesis (32 genes). In the glycoprotein cluster, genes related to signaling molecules, extracellular component and matrix, membrane components, and receptors were enriched (Figure 3(c), left), being involved in receiving the extracellular signals. Conversely, transcriptional regulation (20 genes), glycoprotein (63 genes), and transcription activator activity (7 genes) were enriched in hOFs < hDFs. Most of the genes highly enriched in the cluster of transcriptional regulation were HOX genes (Figure 3(c), right), which were shown in Figure 3(a).

Next, we performed pathway analysis to understand the intracellular events in hOFs > hDFs and hOFs < hDFs. In the group of hOFs > hDFs, eleven pathways were enriched (Figure 3(d), Supplementary Table S2). These were categorized into three groups, including (1) tissue-reconstructive pathways (such as complement and coagulation cascades, calcium signaling pathway, endocytosis, chemokine signaling, focal adhesion, and regulation of actin cytoskeleton); (2) differentiation pathways of cranial neural crest lineages (melanogenesis, axon guidance); and (3) growth- and differentiation-inducing factors. The third group comprised three cancer-related pathways (basal cell carcinoma, pancreatic cancer, and pathway in cancer) comprising mainly cytokines, growth factors, and signaling molecules, not oncogenes. In addition, melanogenesis and axon guidance pathways were only detected in hOFs, consistent with hOFs being derived from cranial neural crest cells. In addition, TGF-beta signaling was not enriched independently. Because* TGF-beta3* is expressed higher than* TGF-beta1 *and* TGF-beta2 *in embryonic skin fibroblasts and opposed to adult skin fibroblasts during wound healing [11], we analyzed TGF-beta signaling pathway-related genes by KEGG program. We found that* TGF-beta2*,* SMAD2* and* SMAD3* were highly expressed, but not* TGF-beta3* (data not shown).

Conversely, only three pathways (p53 signaling, ECM-receptor, and focal adhesion pathways) were enriched in hOFs < hDFs (Figure 3(e), Supplementary Table S3). p53 is known as a tumor suppressor [23], and the expression of* p53* itself showed no difference between hOFs and hDFs (data not shown). However, the downstream genes,* cyclin D1 (CCND1)*,* growth arrest *and* DNA-damage-inducible beta (GADD45B)*,* serpin peptidase inhibitor*,* clade E*,* member 1/plasminogen activator inhibitor type 1 (SERPINE1/PAI-1)*, and* insulin-like growth factor binding protein 3 (IGFBP3)* were downregulated in hOFs. These molecules regulate cell cycle, DNA repair, antiangiogenesis, and the anti-insulin-like growth factor 1 (IGF-1) pathway [23].* Tenascin C (TNC)*,* integrin*,* alpha 1 (ITGA1)*,* cartilage oligomeric matrix protein (COMP)*, and* ITGA6* were identified and seen to overlap in ECM-receptor and focal adhesion pathways.

### 3.4. Plasticity and Specificity of hOFs

**Figure 4:**
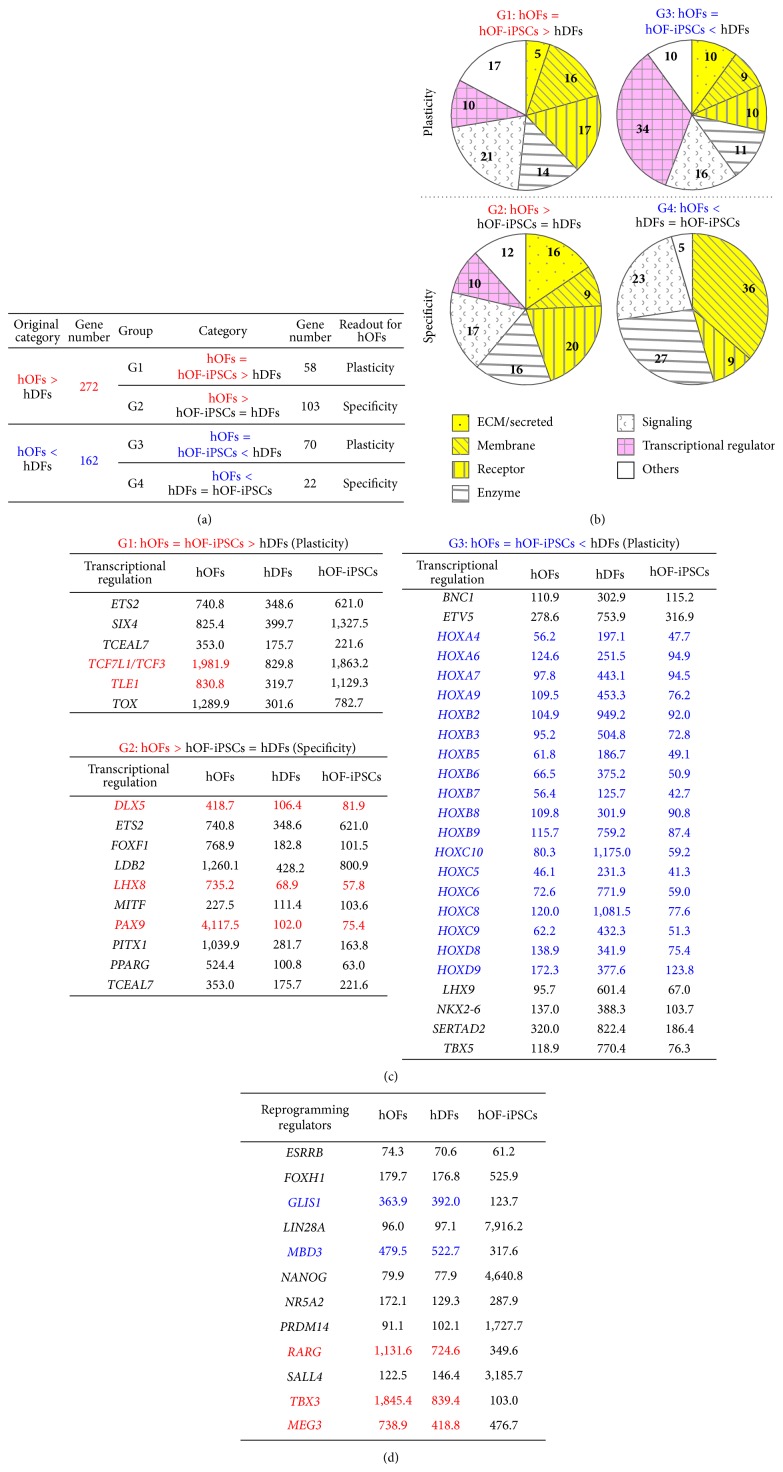
Characterization of hOFs in comparison with hDFs and hOF-iPSCs. (a) Strategy to define the characteristics of hOFs. The differentially expressed gene groups between hOFs and hDFs were rearranged by the expression similarity with hOF-iPSCs. The readout for hOFs indicates the characteristics of hOFs. Each gene in G1–G4 is listed in Supplementary Tables S4–S7, respectively. (b) The characterization of each gene group categorized in (a). Numbers indicate the percentage of gene numbers in the individual categories compared with total numbers. The groups colored in yellow show the molecules receiving environmental stimuli. The groups colored in pink represent molecules involved in controlling cell fate. (c) The list of individual transcriptional regulators found in (b). Each indicated number is the average signal value in each cell type. (d) Expression levels of reprograming enhancers among the three cell types. Each indicated number is the average signal value in each cell type. Red: hOFs > hDFs > hOF-iPSCs; blue: hOFs = hDFs > hOF-iPSCs. The full names of each gene listed in (d) are shown in Supplementary Table S8.

To further define the characteristics of hOFs, gene groups in hOFs > hDFs and hOFs < hDFs were filtered by similarity in gene-expression level to hOF-iPSCs (Figure 4(a)).

First, we found that 58 genes in hOFs were shared with the similar expression levels in hOF-iPSCs and with the expression levels higher than that in hDFs (hOFs = hiPSCs > hDFs; group G1), suggesting that the genes reflect the plasticity or undifferentiated property of multipotent hOFs by enhancement. Second, 103 genes were highly expressed in hOFs compared with those in hDFs and hiPSCs (hOFs > hiPSCs = hDFs; group G2). The genes in G2 were highly expressed in hOFs but may be kept at low or absent expression levels in hDFs. Therefore, it was suggested that the genes in G2 can exhibit the specificity or differentiated property of hOFs. Third, 70 genes in hOFs had expression levels similar to hOF-iPSCs but were expressed at lower levels than in hDFs (hOFs = hiPSCs < hDFs; group G3). The genes in G3 are defined as the specificity of hDFs; however, these genes could be also involved in the plasticity of hOFs by being suppressed. Twenty-two genes in hOFs were expressed at lower levels than in hDFs that showed expression levels similar to those of hOF-iPSCs (hOFs < hDFs = hiPSCs; group G4), suggesting specificity or differentiated property of hOFs by suppression and, reciprocally, plasticity or undifferentiated property of hDFs.

We further analyzed the individual components in the G1–G4 groups, and we categorized them into seven groups, such as ECM/secreted, membrane, receptor, enzyme, signaling, transcriptional regulator, and others (Figure 4(b)). The genes in each group are listed in Supplementary Tables S4–S7. Based on this classification, we found that approximately 30%–40% of the genes in all groups comprised ECM/secreted proteins, membrane proteins, and receptors/transporters, which are highlighted in yellow color in Figure 4(b). These molecular groups are all located at the interface between the cell surface and the extracellular environment, and they may function as a gate of chemical substances and signals (Supplementary Tables S4–S7). Therefore, it is suggested that both fibroblasts are sensitive to environmental factors or cues.

Then we observed that the transcriptional regulator accounted for 10% in both G1 and G2, 34% in G3, and 0% in G4, highlighted by pink color in Figure 4(b) (the gene list, Supplementary Tables S4–S7). This finding is quite important because transcriptional regulators can influence cell fate [24].  Figure 4(c) shows the lists of transcriptional regulators in G1, G2, and G3. The listed genes in G2 and G3 were mostly overlapping with the genes listed in Figures 3(b) and 3(c), which are associated with the fibroblastic specificity of hOFs and hDFs, respectively. The transcriptional regulators in G1 are supposed to represent the gene group related to the plasticity of hOFs because* transcription factor 7-like 1 (T-cell specific, HMG-box)/T-cell factor-3 (TCF7L1/TCF3)* and* transducin-like enhancer of split 1 (E (sp1) homolog, Drosophila *Groucho) (*TLE1*) are involved in controlling ESCs status by functioning as components of wingless-type MMTV integration site family (WNT) signaling. The transcriptional regulators in G2 are involved in the early developmental regulation, and they are also recognized as markers of the cranial neural crest. The transcriptional regulators in G3 are rich in* HOX *genes, which are involved in determining localization and morphology.

Lastly, we surveyed expression levels of reprogramming regulators because these can support plasticity in fibroblasts. Compared with hOF-iPSCs, the higher expression of reprogramming enhancers, such as* Gli-similar 1 (GLIS1)*,* methyl-CpG-binding domain protein 3 (MBD3)*,* retinoic acid receptor, gamma (RARG)*, and* T-box 3 (TBX3)*, was detected in hOFs and hDFs (Figure 4(d), Supplementary Table S8). Notably, hOFs expressed* RARG* and* TBX3* at the highest level among the three types of cells. Conversely,* LIN28A* was expressed only to a limited extent in hOFs and hDFs.* MEG3*, a human homolog of mouse* Meg3/gene trap locus 2 (Meg3/Gtl2)*, was expressed at the higher level in hOFs than in hDFs and hOF-iPSCs.

## 4. Discussion

**Figure 5:**
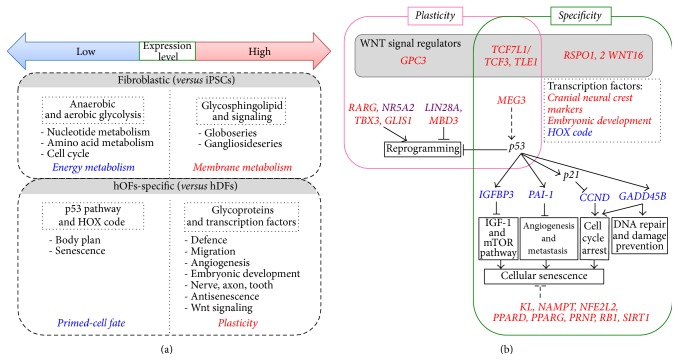
Summary of gene signatures in hOFs. (a) Overview of gene profiles in oral mucosal fibroblasts. Dotted-lined box indicates the category of genes. Red- and blue-colored words in italics show the biological function of gene categories with high and low expressions, respectively. (b) A proposed possible gene network in oral mucosal fibroblasts. Gene names in the different color are indicated as follows. Red: high expression in hOFs compared with that in hDFs (hOFs > hDFs); blue: low expression in hOFs compared with that in hDFs (hOFs < hDFs); purple: similar expression in hOFs and hDFs, but higher than hiPSCs (*hOFs = hDF > hiPSCs*). The box colors indicate biological characteristics or functions as follows. Pink box: possible “plastic” characteristics; green box: possible “specific” characteristics of hOFs; gray box: WNT signal regulators; lined box: biological function; dotted-lined box: the known key transcription factors. Dotted bar indicates indirect effect. Detailed explanations of (a) and (b) are described in the text.

In this study, we elucidated the unique characteristics of hOFs through comparative analyses of gene expression profiles among hOFs, hDFs, and hOF-iPSCs. In Figure 5(a), we categorized the characteristic gene profile in hOFs that the common fibroblastic features as observed in hOFs and hDFs compared with hOF-iPSCs (upper box) and the specific characteristics of “hOFs” can be demonstrated by comparing with hDFs (lower box). Based on these findings, we developed the possible gene network in hOFs as shown in Figure 5(b).

### 4.1. Unique Metabolic Pathways in Human Fibroblasts Compared with iPSCs

First, we noted activated GSL metabolism in both hOFs and hDFs compared with hOF-iPSCs (Figures 2 and 5(a)). GSLs are important for membrane organization, signaling interface to ECM, cell-cell adhesion, and cell recognition [25–27]. Furthermore, some GSLs function as sensors in cellular differentiation and tissue patterning [27, 28]. GSLs are basically categorized into three major groups: (1) the ganglio-series and isoganglio-series, (2) the lacto-series and neolacto-series, and (3) the globo-series and isoglobo-series [25, 27]. The ganglio-series and isoganglio-series GSLs are abundant in the brain and are also detected in ESCs of embryoid bodies, neural lineage cells, macrophages, and B cells. The ganglio-series GSLs are functionally involved in cell adhesion and molecular recognition, forming the “glycosynapse” [29, 30]. For example, monosialodihexosylganglioside (GM3) is involved in integrin regulation, epidermal growth factor (EGF) receptor signaling [31], and lipid raft localization [32]. Conversely, lacto-series and neolacto-series GSLs were originally found in erythrocytes as blood group antigen and in tumors as Lewis X (Le^*x*^) GSL antigen. Stage-specific embryonic antigen-1 (SSEA-1), a marker for both mouse ESCs and embryonic carcinoma cells (ECCs), is also included in this group, and it contains Le^*x*^ and mediates homotypic adhesion related to compaction or autoaggregation [25]. Furthermore, the absence of lactotriaosylceramide (Lc3cer) synthase, as shown in* UDP-GlcNAc:betaGal beta-1,3-N-acetylglucosaminyltransferase 5- (B3GNT5-)* deficient mice, has been reported to cause preimplantation lethality [33] or multiple postnatal defects [34]. The globo-series and isoglobo-series GSLs were originally found in human erythrocytes as the major component. Both SSEA-3 and SSEA-4 are common markers for human ESCs and iPSCs [35, 36].

In our profiles, GSL-related GTs in the globo-series and ganglio-series GSL biosynthetic pathways were highly expressed in both hOFs and hDFs compared with their expression in hOF-iPSCs, whereas GTs in the lacto-series/neolacto-series GSL biosynthetic pathways were less expressed (Figure 2(d)). GSL expression has been demonstrated to be strictly controlled during both mouse embryonic development* in vivo* [25] and differentiation of human ESCs* in vitro *[37, 38]. Globo-series and lacto-series of GSLs are highly expressed in stem cells, whereas gangliosides are contained in further differentiated cells such as embryoid bodies and neuronal cells [37, 38]. Based on these findings, it is suggested that both hOFs and hDFs have the characteristics of differentiated cells except for the high expression of GTs in the globo-series. However, we found that* UDP-Gal:betaGlcNAc beta 1,3-galactosyltransferase, polypeptide 5 (B3GALT5)*, which catalyzes the conversion from globotetraosylceramide (Gb4cer) to globopentaosylceramide (Gb5cer) (Figures 2(d) and 2(e)), was lower expressed in both hOFs and hDFs than in hOF-iPSCs. Lower expression of* B3GALT5* may cause the accumulation of Gb4cer or globotriaosylceramide (Gb3cer). Because Gb3cer and other glycosphingolipids are also involved in caveolar-1 oligomerization [39], their accumulation may affect the sorting and trafficking of caveolae in the membrane, resulting in the function of signaling in fibroblasts. Taken together, the expression profiles of GSLs-GTs suggested their possible roles in “the environmental sensor” in fibroblasts through membrane metabolism.

Another unique “fibroblastic” feature is the underexpression of aerobic and anaerobic glycolysis-related genes in hOFs and hDFs (Supplementary Figure S2). This finding suggested that hOFs and hDFs are bioenergetically less active than hOF-iPSCs. A recent study reported that the metabolic switching of energy metabolism is linked with cell fate decision [40], consistent with the change from oxidative phosphorylation in mouse embryonic fibroblasts (MEFs) to glycolysis in iPSCs during reprogramming [41]. In addition, it was also demonstrated that active hypoxia inducible factor 1, alpha subunit (HIF1*α*), and cytochrome c oxidase (COX) could regulate the metabolic transition from aerobic glycolysis in mouse ESCs to anaerobic glycolysis in mouse epiblast stem cells (EpiSCs) and human ESCs [42]. However, we observed that expression levels of* HIF1α* and* COX* were similar among hOFs, hDFs, and hOF-iPSCs in our profiling data (data not shown). Collectively, hOFs and hDFs appear to exhibit the bioenergetically intermediate phenotype between stem cells and terminally differentiated cells, showing the potential to select cell fate, together with membrane sensing of GSLs.

### 4.2. Gene Signatures Unique to hOFs Compared with hDFs

Next, we elucidated the differences between hOFs and hDFs by comparative* in silico* analyses. The glycoprotein group was highly enriched in hOFs compared with hDFs (Figure 3(b), left); ECM and membrane components, cell motion, adhesion, and defense responses, which are linked with responses to stimuli from outside the cell, were also sequentially enriched in hOFs (Figure 3(c)). Corresponding pathway analysis revealed that the pathways of tissue reconstruction and differentiation and induction of growth and differentiation factors were active in hOFs (Figure 3(d)). The combination of these highly enriched groups in hOFs may enhance the potential of responding to invasive events or inflammation [43], the advantages of differentiating into melanocytes and neurons (axons) [44, 45], and accessibility of signaling molecules that maintain cell growth or differentiation. These characteristics indicated that hOFs may have the flexibility or plasticity as shown in Figure 5(a).

On the other hand, “transcriptional regulation” was highly enriched in the underexpression group in hOFs (Figure 3(b), right). The components of this group especially were* HOX* genes, conversely representing the specificity of hDFs. Fibroblasts derived from the various anatomical positions in the body have been demonstrated to keep HOX code and position-specified gene signatures to achieve their molecular specification of site-specific variations in fibroblasts [22]. HOX genes are known to regulate anterior-posterior axis, patterning, and timing through development [46]. Although both hOFs and hDFs express their positional information, hOFs might have some plasticity due to low expression of clustered* HOXA* to* HOXD* groups of homeobox genes that tightly control body axis formation (Figure 5(a)).

In addition, we found a low gene expression related to the p53 signaling pathway in hOFs compared with hDFs (Figure 3(e)). p53 is a tumor suppressor gene and its activation regulates multiple events including cell cycle arrest, apoptosis, angiogenesis and metastasis inhibition, DNA repair, IGF-1/mTOR pathway inhibition, reprogramming suppression, and cellular senescence [23, 47, 48]. Although the expression level of* p53* itself was similar in hOFs and hDFs, the downstream genes* CCND1*,* IGFBP3*, and* SERPINE1/PAI-1*, which are involved in p53-induced or stress-induced senescence [49–51], were underexpressed in hOFs. Supportively, we confirmed that the expression of some antisenescence regulators [52] was expressed higher in hOFs compared to those in hDFs (Supplementary Figure S3):* klotho *(*KL*; a membrane protein and suppressor of aging [53]),* nicotinamide phosphoribosyltransferase *(*NAMPT*; a converting enzyme for NAD^+^ biosynthesis to increase intracellular NAD^+^ levels [54]),* nuclear factor (erythroid derived 2) related factor 2 *(*Nrf2*; a transcription factor and induction of antioxidant enzymes [55]),* peroxisome proliferator-activated receptor gamma* (*PPARG*; a transcription factor, antiaging and reduction of physiological stress [56]),* PPAR delta *(*PPARD*, a transcription factor, inhibition of ROS generation [57]),* prion protein* (*PRNP*; a membrane anchored glycoprotein and antioxidant activity [58]),* retinoblastoma 1* (*RB1*; a tumor suppressor protein [59]), and* sirtuin 1* (*SIRT1*; NAD^+^ dependent deacetylase and a mammalian longevity protein [60]). These findings suggested that hOFs exhibit not only higher plasticity but also greater longevity compared to hDFs (Figure 5(b)).

### 4.3. Specificity and Plasticity in hOFs Predicted by the Profiles of Transcription Factors

We performed comparative analyses between hOFs and hOF-iPSCs, which were generated from parental hOFs (Figure 4) to elucidate hOF plasticity and specificity. Focused on the transcription regulators that control cell fate, we developed a plausible gene network to characterize hOFs (Figure 5(b)).

The plasticity of hOFs could be regulated by the high expression of* TCF7L1/TCF3 *and* TLE1*, the negative regulators of canonical WNT signaling. In human ESCs, canonical WNT signaling actively regulates pluripotency. However, to differentiate into specific cell types of mesodermal and endodermal lineages, WNT signals need to be transiently downregulated by* TCF7L1/TCF3 *and* TLE1* [61–66]. TCF7L1/TCF3 is also defined as a mouse ESC marker [67], and downregulation of TCF7L1/TCF3 has been observed when mouse ESCs differentiate into EpiSCs [68]. Furthermore,* Tcf7l1/Tcf3 *regulates stage-specific WNT signaling during the reprogramming of fibroblasts into iPSCs [69], neural stem cell status [70], or epidermal progenitor status [71]. Recently, a new role for* TCF7L1/TCF3* in skin wound healing was reported by demonstrating that* TCF7L1/TCF3 *was upregulated in epithelial cells at the site of injury, accelerating wound healing* in vivo* through lipocalin-2 (Lcn2) induction [72]. Another molecule, TLE1, is a transcriptional repressor essential in hematopoiesis and neuronal and epithelial differentiation [73]. Recently, it was reported that TLE1 binds to TCF3 and TCF4 but not to LEF1 and TCF1 and that TCF-TLE1 complexes bind directly to heterochromatin in a specific manner to control transcriptional activation [74]. Furthermore, we found that some positive regulators of WNT signaling were highly expressed by hOFs, for example, a proteoglycan* glypican-3 (GPC3)* [75–77], a secreted protein* R-spondin 1, 2 (RSPO1, 2)* [78, 79], and* WNT16* [80] (Figure 5(b), Supplementary Tables S4 and S5). GPC3 is expressed in pluripotent cells and cancer cells [75–77].* RSPO1* has been demonstrated to commit to the specification of germ cells, and* RSPO2* plays a role in craniofacial, limb, and branching development [78, 79].* WNT16* is involved in the specification of hematopoietic stem cells [80]. Taken together, the characteristics of hOFs can be controlled by WNT signaling, and our data is the first report to reveal this by transcriptome profiles. In addition, the cranial neural crest markers were classified into highly expressed gene group of hOFs (Figures 3(a) and 4(c)), and* HOX* genes were repeatedly categorized into the underexpressed gene group of hOFs (Figures 3(b) and 4(c)). These findings suggested that hOFs are differently primed from dermal fibroblasts, but they preserve flexibility or plasticity.

The specificity of hOFs is mainly characterized by a high expression of cranial neural crest markers [44].* Forkhead box F1 (FOXF1)* (lung),* LIM homeobox 8 (LHX8)* (nerve),* microphthalmia-associated transcription factor (MITF)* (melanogenesis),* PAX9* (tooth, palate, and limb) [81], and* PPARG* (adipocyte) [82, 83] are all involved in embryonic development (Figure 5(b)). These findings suggested that hOFs have some advantage in differentiating into neural crest-derived lineages.

### 4.4. Plasticity in hOFs is Predicted by Reprogramming Regulators

When we surveyed the detailed hOF gene signatures, we found several important genes associated with plasticity. Recent development of iPSCs technology demonstrated that the cellular plasticity can be acquired by reprogramming with not only four transcription factors, such as Pou5f1/Oct4, Sox2, KLF4, and c-myc [84], but also with additional reprogramming regulators. Among them, we found that two transcription factors,* RARG* [85] and* TBX3* [86], are quite highly expressed in hOFs compared with hDFs (Figure 4(d)). RARG, a nuclear receptor, can form heterodimers with* nuclear receptor subfamily 5*,* group A*,* member 2/liver receptor homolog 1 (NR5A2/LRH-1)* [87], and directly activate* Oct* transcription [88, 89], and the combination with reprogramming factors increased reprogramming efficiency of MEFs into mouse iPSCs [85]. Recently, *Rarg* and *Nr5a2* combined with achaete-scute complex homolog 1 (*Ascl1*),* POU domain*,* class 3*,* transcription factor 2 (Pou3f2/Brn2)*, and* neurogenin 2 (Ngn2)* enhanced the efficiency of transdifferentiation from MEFs to functional neurons [90]. Conversely,* TBX3* is necessary to maintain pluripotency of mouse ESCs and also to regulate differentiation, proliferation, and signaling [86, 91, 92], although* TBX3* in hESC regulates proliferation and differentiation [93]. Although the roles of* RARG* and* TBX3 *in hOFs are not fully understood, it might be possible for these to regulate the plasticity of hOFs (Figure 5(b)).

In the other transcription factors,* GLIS1*, [94] was highly expressed, whereas* NR5A2/LRH-1* was underexpressed in both hOFs and hDFs.* LIN28A*, a miRNA and a reprogramming repressor controlling cell plasticity [95, 96], was also expressed at quite low levels in both hOFs and hDFs. Although* MBD3*, the suppression of which can increase reprogramming efficiency [97], was highly expressed in both hOFs and hDFs, these results suggested that both types of fibroblasts might have a similar advantage of reprogramming both cell fate and plasticity. Indeed, in hDFs, less factors or only exogenous* POU5F1/OCT4 *can introduce reprogramming [98, 99]. Furthermore, direct induction of transdifferentiation has been reported from hDFs to the other cell lineage without iPSC formation [100–103]. Transdifferentiation has been induced by a combination of specific media and supplements, for example, addition of FGF2 to the culture changed transcriptional profiles in hDFs and promoted regeneration capability [104]. Recently, it was demonstrated that mouse DFs are not a terminally differentiated cell type but can be further differentiated into several different types of fibroblasts to form the dermal structure during skin development and wound healing steps [105]. Since DFs have the plasticity to adapt to the environmental changes* in vitro* and* in vivo* [100–105], hOFs might have similar properties. Further investigations are required to confirm this hypothesis.

In addition,* MEG3* was expressed at a quite high level compared to those of hDFs and hOF-iPSCs (Figure 4(d)). Because* MEG3* is located within the imprinted* DLK1-DIO3* gene cluster on chromosome 14q32, we further examined the additional imprinted genes (Supplementary Figure S4). Interestingly, hOFs highly expressed both paternal imprinted genes,* DIRAS3* and* IGF2*, and maternal imprinted genes,* H19 *and* MEG3,* compared to those in hOF-iPSCs. Furthermore, the expression of* DLK1* and* DIO3*, which are paternally expressed genes and located within the same region as* MEG3*, was lower than that of* MEG3* in hOFs. We found a similar expression pattern within 14q32 in hOF-iPSCs.* MEG8*, known as* Rian *in mouse, is also located within 14q32 and maternally expressed, long noncoding RNAs were not on the lists of gene profiles. The expression of* DIRAS3*,* PEG10*, and* IGF2 *was reciprocally observed between hOFs and hOF-iPSCs. Furthermore, although* H19* and* IGF2* exhibit maternal and paternal expressions that are located in the same region, both genes were highly expressed in hOFs. At this moment, we do not know the biological meaning of their expression patterns. Further analyses will be required.* MEG3* is also known as a tumor suppressor via p53 activation [106, 107]. Because the underexpression of p53-downstream genes was observed along with the high expression level of* p53 *in hOFs,* MEG3* could also be involved in the specificity of hOFs by controlling p53 signaling as shown in Figure 5(b).

## 5. Conclusions

We elucidated the fibroblastic plasticity and specificity by analyzing transcriptome profiles of GSL metabolism in hOFs and hDFs. The uniqueness of hOFs is defined as partly primed cells committed to the neural crest cell lineage with plasticity and longevity controlled by* WNT* and* p53 *gene network as shown in Figure 5(b). Further analyses are required to prove this hypothesis, but, importantly, our findings in the present study provide a novel basis for discussing the potential application of hOFs in regenerative medicine.

## Supplementary Material

The reliability of microarray hybridization techniques were confirmed by the company-supplied hybrydization control.
