# Supplementary material for: Gene Signature of Human Oral Mucosa Fibroblasts: Comparison with Dermal Fibroblasts and Induced Pluripotent Stem Cells
Source: Biomed Res Int. 2015 Aug 3;2015:121575. doi: 10.1155/2015/121575 (PMC4538314; doi:10.1155/2015/121575)
Supplement: Supplementary file 1 — The reliability of microarray hybridization techniques were confirmed by the company-supplied hybrydization control. [file 121575.f1.pdf]

Supplementary Figure S1

A

Array Name (sample name)	hOF2	hOF3	hOF4	hDF (TIG110)	hDF (TIG111)	hDF (TIG114)	hOF-iPSC3	hOF-iPSC2	hOF-iPSC4
hOF2	1	0.99	0.96	0.97	0.97	0.94	0.77	0.78	0.77
hOF3	0.99	1	0.97	0.97	0.97	0.95	0.77	0.78	0.77
hOF4	0.96	0.97	1	0.95	0.92	0.96	0.78	0.79	0.78
hDF (TIG110)	0.97	0.97	0.95	1	0.97	0.97	0.78	0.79	0.78
hDF (TIG111)	0.97	0.97	0.92	0.97	1	0.94	0.77	0.78	0.76
hDF (TIG114)	0.94	0.95	0.96	0.97	0.94	1	0.78	0.78	0.79
hOF-iPSC3	0.77	0.77	0.78	0.78	0.77	0.78	1	0.99	0.99
hOF-iPSC2	0.78	0.78	0.79	0.79	0.78	0.78	0.99	1	0.99
hOF-iPSC4	0.77	0.77	0.78	0.78	0.76	0.79	0.99	0.99	1

0.8	0.83	0.85	0.88	0.9	0.93	0.95	0.98	1
0.8				0.9				1

B

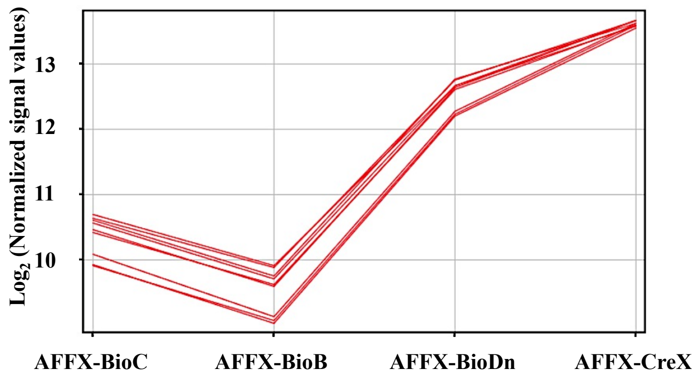

C

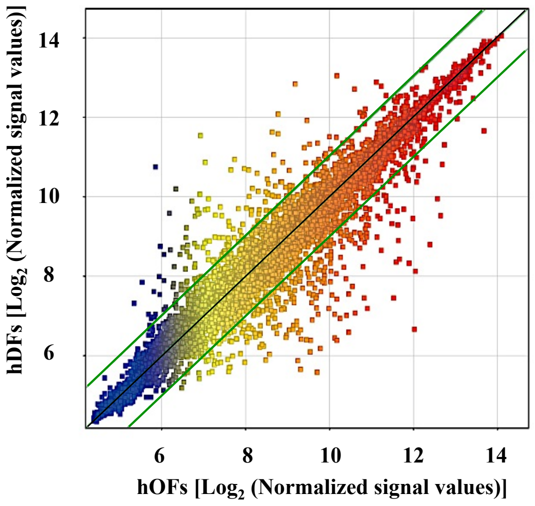

Supplementary Figure S2

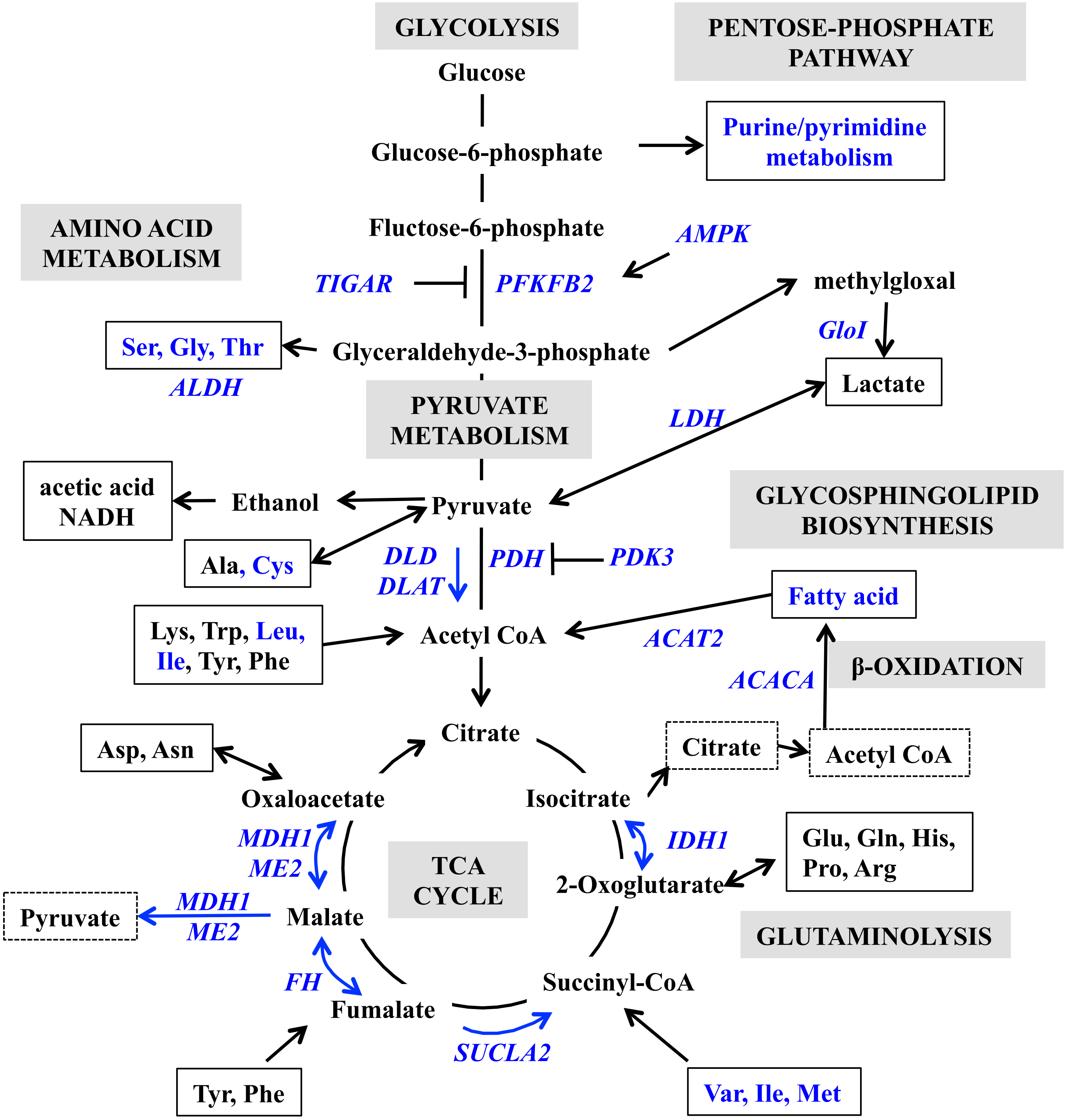

Supplementary Figure S3

Entrez GeneID	*Gene Symbol*	*Gene Name*	hODs	hDFs	hOF-iPSCs
644	*BLVRA*	*biliverdin reductase A*	223.9	259.3	253.0
3162	*HMOX1*	*heme oxygenase 1*	1474.2	2698.0	452.3
9365	*KL*	*klotho*	54.1	44.8	36.5
10135	*NAMPT*	*nicotinamide phosphoribosyltransferase*	827.7	626.2	2999.8
4780	*NFE2L2*	*nuclear factor (erythroid-derived 2) related factor 2*	2134.5	1331.7	1006.2
5467	*PPARD*	*peroxisome proliferator-activated receptor delta*	1534.6	1352.9	330.8
5468	*PPARG*	*peroxisome proliferator-activated receptor gamma*	524.4	100.8	63.0
5621	*PRNP*	*prion protein*	4059.0	3134.1	1065.5
5925	*RB1*	*retinoblastoma 1*	882.9	805.8	739.3
23411	*SIRT1*	*sirtuin 1*	231.5	216.8	1676.0
7157	*TP53*	*tumor protein p53*	2107.7	2197.0	2149.1

Supplementary Figure S4

Entrez Gene ID	Expressed Allele	Location	*Gene Symbol*	*Gene Name*	hOFs	hDFs	hOF-iPSCs
9077	Paternal	1p31 AS	*DIRAS3*	*DIRAS family, GTP-binding RAS-like 3*	254.9	140.7	72.4
23089	Paternal	7q21	*PEG10*	*paternally expressed 10*	257.1	374.8	1841.9
283120	Maternal	11p15.5 AS	*H19*	*H19, imprinted maternally expressed transcript (non-protein coding)*	1304.4	801.3	559.8
3481	Paternal	11p15.5 AS	*IGF2*	*insulin-like growth factor 2 (somatomedin A)*	1060.0	184.8	82.6
3784	Maternal	11p15.5	*KCNQ1*	*potassium voltage-gated channel, KQT-like subfamily, member 1*	150.2	147.2	107.9
55384	Maternal	14q32	*MEG3*	*maternally expressed 3 (non-protein coding)*	738.9	418.8	476.7
8788	Paternal	14q32	*DLK1*	*delta-like 1 homolog*	161.4	182.5	173.5
388015	Paternal	14q32.31 AS	*RTL1*	*retrotransposon-like 1*	78.9	79.0	68.7
1735	Paternal	14q32	*Dio3*	*deiodinase, iodothyronine, type III*	76.5	84.5	68.6
79104	Maternal	14q32.31	*MEG8*	*maternally expressed 8 (non-protein coding)*	n.d.	n.d.	n.d.
4692	Paternal	15q11.2-q12 AS	*NDN*	*necdin homolog (mouse)*	147.2	122.5	98.9
5178	Paternal	19q13.4 AS	*PEG3*	*paternally expressed 3*	104.5	107.8	86.7

Supplementary Table S1. 12,713 gene probe list after differential expression analysis with One-way ANOVA (corrected *p* < 0.05).

Transcripts Cluster Id	Entrez Gene	*Gene description*	*Gene symbol*	hOFs	hDFs	hOF-iPSCs	*p*-value	corrected *p-value* (BH)
7980908	10516	*fibulin 5*	*FBLN5*	13.59	13.13	7.99	1.86E-11	5.37E-07
7899167	79727	*lin-28 homolog A (C. elegans)*	*LIN28A*	6.59	6.60	12.95	6.80E-11	9.81E-07
8173414	84889	*solute carrier family 7 (cationic amino acid transporter, y+ system), member 3*	*SLC7A3*	5.97	6.02	12.94	1.98E-10	9.88E-07
7969533	122060	*SLAIN motif family, member 1*	*SLAIN1*	5.61	5.41	10.02	2.05E-10	9.88E-07
8102404	407028	*microRNA 302a*	*MIR302A*	4.52	4.53	11.09	1.22E-10	9.88E-07
8178470	5460, 642559, 645682, 5462	*POU class 5 homeobox 1*	*POU5F1*	7.34	7.30	13.19	1.67E-10	9.88E-07
8170648	633	*biglycan*	*BGN*	13.48	13.57	7.57	2.53E-10	1.04E-06
8041853	4072	*epithelial cell adhesion molecule*	*EPCAM*	5.42	5.50	12.02	7.89E-10	2.85E-06
8052940	400961	*poly(A) binding protein interacting protein 2B*	*PAIP2B*	6.37	6.36	9.97	9.95E-10	2.87E-06
8102406	442894	*microRNA 302b*	*MIR302B*	4.50	4.51	11.33	9.53E-10	2.87E-06
7970989	728591, 54937	*chromosome 13 open reading frame 38 | spermatogenesis and oogenesis specific basic helix-loop-helix 2*	*C13orf38 |SOHLH2*	6.04	6.20	10.83	1.14E-09	3.00E-06
8169385	6998	*teratocarcinoma-derived growth factor 3, pseudogene*	*TDGF3*	5.97	6.12	10.61	1.57E-09	3.50E-06
8124889	5460, 642559, 645682, 5462	*POU class 5 homeobox 1*	*POU5F1*	7.13	7.10	12.71	1.62E-09	3.50E-06
8179719	5460, 642559, 645682, 5462	*POU class 5 homeobox 1*	*POU5F1*	7.11	7.09	12.71	1.70E-09	3.50E-06
8089438	151871	*developmental pluripotency associated 2*	*DPPA2*	4.97	4.94	9.12	2.27E-09	4.37E-06
8072015	157	*adrenergic, beta, receptor kinase 2*	*ADRBK2*	5.91	5.86	9.25	2.93E-09	4.46E-06
7962455	4753	*NEL-like 2 (chicken)*	*NELL2*	5.35	5.29	9.28	2.79E-09	4.46E-06

7944667	6653	*sortilin-related receptor, L(DLR class) A repeats-containing*	*SORL1*	6.32	6.44	10.00	2.55E-09	4.46E-06
7953675	79923, 404635	*Nanog homeobox | Nanog homeobox pseudogene 1*	*NANOG |NANOGP 1*	6.32	6.28	12.18	2.88E-09	4.46E-06
7996837	999	*cadherin 1, type 1, E-cadherin (epithelial)*	*CDH1*	6.22	6.22	11.45	3.09E-09	4.46E-06
8162652	1515	*cathepsin L2*	*CTSL2*	5.30	5.36	9.06	3.44E-09	4.73E-06
8077366	57633	*leucine rich repeat neuronal 1*	*LRRN1*	5.04	5.06	11.62	4.09E-09	5.19E-06
8044878				4.48	4.48	5.20	4.13E-09	5.19E-06
7986822	2562	*gamma-aminobutyric acid (GABA) A receptor, beta 3*	*GABRB3*	7.12	7.17	10.87	4.95E-09	5.50E-06
8160637	2683	*UDP-Gal:betaGlcNAc beta 1,4- galactosyltransferase, polypeptide 1*	*B4GALT1*	10.85	11.37	8.04	4.92E-09	5.50E-06
7932109	22929	*selenophosphate synthetase 1*	*SEPHS1*	10.69	10.36	13.17	4.87E-09	5.50E-06
8098745	654254	*zinc finger protein 732*	*ZNF732*	5.57	5.51	8.65	5.97E-09	6.16E-06
7921713	50848, 100131187	*F11 receptor | thiosulfate sulfurtransferase (rhodanese)-like domain containing 1*	*F11R|TST D1*	6.79	6.81	11.09	5.86E-09	6.16E-06
8089448	55211	*developmental pluripotency associated 4*	*DPPA4*	5.84	5.74	11.70	6.30E-09	6.27E-06
7984813	3671	*immunoglobulin superfamily containing leucine-rich repeat*	*ISLR*	12.42	12.64	6.40	7.29E-09	6.52E-06
8135774	5803	*protein tyrosine phosphatase, receptor-type, Z polypeptide 1*	*PTPRZ1*	5.28	5.24	11.23	7.01E-09	6.52E-06
8100464	10874	*neuromedin U*	*NMU*	5.54	5.61	8.93	6.98E-09	6.52E-06
8148040	114569	*mal, T-cell differentiation protein 2*	*MAL2*	6.11	6.04	12.90	7.46E-09	6.52E-06
8134263	1278	*collagen, type I, alpha 2*	*COL1A2*	13.26	13.37	8.83	7.95E-09	6.75E-06
7965565	84101	*ubiquitin specific peptidase 44*	*USP44*	5.54	5.86	11.63	8.35E-09	6.89E-06
8121251	389421	*lin-28 homolog B (C. elegans)*	*LIN28B*	4.78	4.68	9.34	9.02E-09	7.23E-06
7923582				6.21	6.69	9.27	1.00E-08	7.83E-06
8069668	116159	*cysteine/tyrosine-rich 1*	*CYYR1*	6.27	6.29	10.23	1.14E-08	8.65E-06
8016646	1277	*collagen, type I, alpha 1*	*COL1A1*	13.68	13.78	9.54	1.31E-08	9.24E-06
8020779	1829	*desmoglein 2*	*DSG2*	5.12	5.06	10.83	1.36E-08	9.24E-06
8129783	9053	*microtubule-associated protein 7*	*MAP7*	7.03	6.90	10.13	1.40E-08	9.24E-06

8133976	10926		*DBF4*	4.75	4.91	6.91	1.42E-08	9.24E-06
7927631	22943	*dickkopf homolog 1 (Xenopus laevis)*	*DKK1*	10.71	12.13	6.02	1.44E-08	9.24E-06
8111524	167127	*UDP glycosyltransferase 3 family, polypeptide A2*	*UGT3A2*	6.48	6.19	10.34	1.30E-08	9.24E-06
7987365	79923, 404635	*Nanog homeobox | Nanog homeobox pseudogene 1*	*NANOG|NANOGP1*	6.54	6.40	11.82	1.37E-08	9.24E-06
8035855	148213	*zinc finger protein 681*	*ZNF681*	5.59	5.40	8.69	1.54E-08	9.64E-06
8046003	25801	*grancalcin, EF-hand calcium binding protein*	*GCA*	5.19	4.81	8.60	1.65E-08	1.01E-05
7995681	4313	*matrix metallopeptidase 2 (gelatinase A, 72kDa gelatinase, 72kDa type IV collagenase)*	*MMP2*	13.34	13.54	9.11	1.71E-08	1.02E-05
8167185	7076	*TIMP metallopeptidase inhibitor 1*	*TIMP1*	13.81	13.86	11.29	1.75E-08	1.02E-05
8133983	53616	*ADAM metallopeptidase domain 22*	*ADAM22*	5.14	5.29	7.19	1.77E-08	1.02E-05
8022666	83539	*carbohydrate (N-acetylgalactosamine 4-0) sulfotransferase 9*	*CHST9*	5.32	5.29	8.17	1.84E-08	1.03E-05
8089988	131076	*coiled-coil domain containing 58*	*CCDC58*	7.11	7.39	9.63	1.86E-08	1.03E-05
7955869	3224	*homeobox C8*	*HOXC8*	6.91	10.08	6.28	1.93E-08	1.05E-05
8033767	147741	*zinc finger protein 560*	*ZNF560*	6.54	5.55	10.02	2.03E-08	1.08E-05
8071920	6634, 83606	*small nuclear ribonucleoprotein D3 polypeptide 18kDa | chromosome 22 open reading frame 13*	*SNRPD3|C22orf13*	8.14	8.16	10.18	2.05E-08	1.08E-05
8179041	3105	*major histocompatibility complex, class I, A*	*HLA-A*	13.34	13.17	11.36	2.17E-08	1.10E-05
8000574	26471	*nuclear protein, transcriptional regulator, 1*	*NUPR1*	12.59	12.48	7.18	2.16E-08	1.10E-05
8046078	8708	*UDP-Gal:betaGlcNAc beta 1,3-galactosyltransferase, polypeptide 1*	*B3GALT1*	5.47	5.23	10.60	2.45E-08	1.20E-05
8020806	54941	*ring finger protein 125*	*RNF125*	5.46	5.45	9.19	2.45E-08	1.20E-05
8115691	6586	*slit homolog 3 (Drosophila)*	*SLIT3*	12.61	12.72	6.58	2.53E-08	1.22E-05
8147351	54845	*epithelial splicing regulatory protein 1*	*ESRP1*	5.66	5.90	11.38	2.60E-08	1.23E-05
8045330	441666	*zinc finger protein 91 pseudogene*	*LOC441666*	4.64	4.60	6.56	2.67E-08	1.25E-05
8171359	2824	*glycoprotein M6B*	*GPM6B*	5.48	5.49	9.47	3.17E-08	1.38E-05
7980152	4053	*latent transforming growth factor beta binding protein 2*	*LTBP2*	11.85	12.51	6.74	3.25E-08	1.38E-05
8026139	4784	*nuclear factor I/X (CCAAT-binding transcription factor)*	*NFIX*	11.54	11.60	7.05	3.10E-08	1.38E-05

8097017	7368	*UDP glycosyltransferase 8*	*UGT8*	5.89	5.78	9.96	3.08E-08	1.38E-05
7998898	9074	*claudin 6*	*CLDN6*	6.29	6.34	12.57	3.21E-08	1.38E-05
8146967	83690	*cysteine-rich secretory protein LCCL domain containing 1*	*CRISPLD1*	5.45	5.31	9.12	3.16E-08	1.38E-05
8138805	54504	*carboxypeptidase, vitellogenic-like*	*CPVL*	6.08	5.98	8.52	3.33E-08	1.39E-05
8108217	7045	*transforming growth factor, beta-induced, 68kDa*	*TGFBI*	13.46	13.68	8.32	3.39E-08	1.40E-05
7996819	1001	*cadherin 3, type 1, P-cadherin (placental)*	*CDH3*	6.23	6.24	11.23	3.46E-08	1.41E-05
7960744	715	*complement component 1, r subcomponent*	*C1R*	12.84	12.14	7.67	3.69E-08	1.46E-05
8006187	79915	*ATPase family, AAA domain containing 5*	*ATAD5*	5.42	5.42	8.62	3.69E-08	1.46E-05
8124901	3107, 3106	*major histocompatibility complex, class I, C | major histocompatibility complex, class I, B*	*HLA-C|HLA-B*	12.78	12.55	10.89	3.90E-08	1.52E-05
8170200	7547	*Zic family member 3 (odd-paired homolog, Drosophila)*	*ZIC3*	5.76	5.94	10.18	3.96E-08	1.53E-05
8121515	117247	*solute carrier family 16, member 10 (aromatic amino acid transporter)*	*SLC16A10*	6.31	6.27	10.36	4.10E-08	1.56E-05
8067955	1525	*coxsackie virus and adenovirus receptor*	*CXADR*	6.58	6.51	12.49	4.29E-08	1.59E-05
8041867	4436	*mutS homolog 2, colon cancer, nonpolyposis type 1 (E. coli)*	*MSH2*	6.55	6.44	10.10	4.43E-08	1.59E-05
8076094	11144		*DMC1*	5.67	5.54	6.74	4.45E-08	1.59E-05
8173812	79366, 6451	*high-mobility group nucleosome binding domain 5 | SH3 domain binding glutamic acid-rich protein like*	*HMGN5|SH3BGRL*	6.11	6.12	8.71	4.34E-08	1.59E-05
7915563				6.05	6.24	8.04	4.37E-08	1.59E-05
7912670	7388, 440567	*ubiquinol-cytochrome c reductase hinge protein | ubiquinol-cytochrome c reductase hinge protein-like*	*UQCRH|UQCRHL*	8.87	9.21	11.36	4.87E-08	1.71E-05
8097920	9227	*lecithin retinol acyltransferase (phosphatidylcholine--retinol O-acyltransferase)*	*LRAT*	5.85	5.93	9.90	5.43E-08	1.89E-05
8096440	2895	*glutamate receptor, ionotropic, delta 2*	*GRID2*	5.53	5.71	9.98	5.72E-08	1.97E-05
7954899	1272	*contactin 1*	*CNTN1*	5.13	5.12	8.21	5.96E-08	2.00E-05
8148315	5462	*POU class 5 homeobox 1B*	*POU5F1B*	6.93	6.94	11.50	5.94E-08	2.00E-05
7901860	54596	*LINE-1 type transposase domain containing 1*	*L1TD1*	4.94	4.89	9.26	6.38E-08	2.10E-05
7980891	123036	*tandem C2 domains, nuclear*	*TC2N*	5.74	5.69	9.58	6.39E-08	2.10E-05
8178489	3107	*major histocompatibility complex, class I, C*	*HLA-C*	12.79	12.54	10.89	6.72E-08	2.18E-05

7938528	55742	*parvin, alpha*	*PARVA*	12.34	12.32	9.43	7.05E-08	2.26E-05
7897441	9563	*hexose-6-phosphate dehydrogenase (glucose 1-dehydrogenase)*	*H6PD*	10.54	10.24	7.27	7.22E-08	2.29E-05
7991335	290	*alanyl (membrane) aminopeptidase*	*ANPEP*	12.50	12.38	6.63	7.38E-08	2.32E-05
8122279	57221		*KIAA1244*	6.39	6.62	8.59	7.68E-08	2.38E-05
8112649	26049	*family with sequence similarity 169, member A*	*FAM169A*	5.57	5.34	9.74	7.80E-08	2.40E-05
8148435	8840	*WNT1 inducible signaling pathway protein 1*	*WISP1*	8.02	9.72	6.37	8.06E-08	2.42E-05
8117890	3133, 3106	*major histocompatibility complex, class I, E | major histocompatibility complex, class I, B*	*HLA-E|HLA-B*	11.82	11.36	9.43	8.02E-08	2.42E-05
8149330	1508	*cathepsin B*	*CTSB*	12.96	12.74	9.47	8.36E-08	2.49E-05
8046922	1281	*collagen, type III, alpha 1*	*COL3A1*	11.74	12.09	6.94	8.77E-08	2.58E-05
7921076	54865	*G patch domain containing 4*	*GPATCH4*	6.69	6.87	8.65	8.89E-08	2.59E-05
8061746	1789	*DNA (cytosine-5-)-methyltransferase 3 beta*	*DNMT3B*	6.98	7.24	12.98	9.78E-08	2.60E-05
8077731	2177	*Fanconi anemia, complementation group D2*	*FANCD2*	5.98	6.18	10.31	9.60E-08	2.60E-05
7949412	4054	*latent transforming growth factor beta binding protein 3*	*LTBP3*	11.16	10.98	7.80	1.00E-07	2.60E-05
7901123	4678	*nuclear autoantigenic sperm protein (histone-binding)*	*NASP*	6.81	7.10	10.29	9.53E-08	2.60E-05
8144036	7516	*X-ray repair complementing defective repair in Chinese hamster cells 2*	*XRCC2*	6.32	6.43	11.45	9.99E-08	2.60E-05
7951910	23621	*beta-site APP-cleaving enzyme 1*	*BACE1*	10.93	11.11	7.86	9.73E-08	2.60E-05
7962203	51067	*tyrosyl-tRNA synthetase 2, mitochondrial*	*YARS2*	7.28	7.44	8.79	9.19E-08	2.60E-05
7932765	143098	*membrane protein, palmitoylated 7 (MAGUK p55 subfamily member 7)*	*MPP7*	5.51	5.89	8.05	9.90E-08	2.60E-05
8123678	221749	*chromosome 6 open reading frame 145*	*C6orf145*	11.33	11.32	6.80	9.91E-08	2.60E-05
8138258	221806	*von Willebrand factor D and EGF domains*	*VWDE*	5.34	5.43	8.44	9.54E-08	2.60E-05
8117800	3105, 3106	*major histocompatibility complex, class I, A | major histocompatibility complex, class I, B*	*HLA-A|HLA-B*	13.26	13.06	11.30	9.26E-08	2.60E-05
8167449	5355, 4007	*proteolipid protein 2 (colonic epithelium-enriched) | prickle homolog 3 (Drosophila)*	*PLP2|PRICKLE3*	12.54	12.77	9.57	9.63E-08	2.60E-05
7961381	2842	*G protein-coupled receptor 19*	*GPR19*	6.33	6.25	9.14	1.04E-07	2.63E-05
8084165	6657	*SRY (sex determining region Y)-box 2*	*SOX2*	6.44	6.46	12.03	1.06E-07	2.63E-05

8173506	54821	*excision repair cross-complementing rodent repair deficiency, complementation group 6-like*	*ERCC6L*	5.02	5.00	7.75	1.06E-07	2.63E-05
8067087	57167	*sal-like 4 (Drosophila)*	*SALL4*	6.94	7.19	11.64	1.04E-07	2.63E-05
7982712	90417	*chromosome 15 open reading frame 23*	*C15orf23*	7.90	8.54	10.80	1.03E-07	2.63E-05
7966122	338773	*transmembrane protein 119*	*TMEM119*	12.40	11.93	6.80	1.07E-07	2.63E-05
7983718	29106	*secretogranin III*	*SCG3*	4.86	4.87	8.47	1.09E-07	2.66E-05
8098439	4072	*epithelial cell adhesion molecule*	*EPCAM*	5.09	5.08	11.78	1.10E-07	2.66E-05
8059905	1293	*collagen, type VI, alpha 3*	*COL6A3*	11.55	12.23	6.24	1.15E-07	2.72E-05
8013341	4239	*microfibrillar-associated protein 4*	*MFAP4*	12.94	12.74	7.46	1.17E-07	2.72E-05
7916316	55706	*transmembrane protein 48*	*TMEM48*	7.85	8.02	11.00	1.15E-07	2.72E-05
8117777	3136, 3135, 3105, 3106	*major histocompatibility complex, class I, H (pseudogene) | major histocompatibility complex, class I, G | major histocompatibility complex, class I, A | major histocompatibility complex, class I, B*	*HLA-H|HLA-G|HLA-A|HLA-B*	12.23	11.97	10.34	1.16E-07	2.72E-05
7913450	3339, 401944	*heparan sulfate proteoglycan 2 | low density lipoprotein receptor class A domain containing 2*	*HSPG2|LDLRAD2*	11.97	11.95	8.28	1.16E-07	2.72E-05
7953321	4055	*lymphotoxin beta receptor (TNFR superfamily, member 3)*	*LTBR*	11.10	10.95	7.04	1.25E-07	2.82E-05
8024623	4782	*nuclear factor I/C (CCAAT-binding transcription factor)*	*NFIC*	11.66	11.55	7.61	1.27E-07	2.82E-05
8173755	9452	*integral membrane protein 2A*	*ITM2A*	5.46	5.76	8.29	1.29E-07	2.82E-05
8147796	9699	*regulating synaptic membrane exocytosis 2*	*RIMS2*	5.94	6.19	8.57	1.26E-07	2.82E-05
8106820	10622	*polymerase (RNA) III (DNA directed) polypeptide G (32kD)*	*POLR3G*	6.96	6.78	11.08	1.27E-07	2.82E-05
7901010	11004	*kinesin family member 2C*	*KIF2C*	6.77	7.32	9.64	1.25E-07	2.82E-05
8065011	79727	*lin-28 homolog A (C. elegans)*	*LIN28A*	6.04	6.09	11.30	1.22E-07	2.82E-05
8142585	93664	*Ca++-dependent secretion activator 2*	*CADPS2*	6.61	5.97	9.41	1.30E-07	2.82E-05
7922412	692197	*small nucleolar RNA, C/D box 77*	*SNORD77*	4.70	4.68	6.48	1.31E-07	2.82E-05
7982663	701, 56924	*budding uninhibited by benzimidazoles 1 homolog beta (yeast) | p21 protein (Cdc42/Rac)-activated kinase 6*	*BUB1B|PAK6*	6.27	6.61	10.86	1.30E-07	2.82E-05
7963970	6490	*silver homolog (mouse)*	*SILV*	6.56	6.50	10.71	1.34E-07	2.83E-05
8128123	58528	*Ras-related GTP binding D*	*RRAGD*	5.49	5.59	9.15	1.34E-07	2.83E-05

7992293	79652	*transmembrane protein 204*	*TMEM204*	11.21	11.38	7.41	1.32E-07	2.83E-05
8095043	65997	*RAS-like, family 11, member B*	*RASL11B*	8.00	6.50	10.97	1.36E-07	2.84E-05
8083146	5357	*plastin 1*	*PLS1*	5.53	5.71	8.64	1.38E-07	2.85E-05
8136631	6742	*single-stranded DNA binding protein 1*	*SSBP1*	8.81	9.08	10.73	1.38E-07	2.85E-05
8152491	2131	*exostosin 1*	*EXT1*	11.52	11.58	9.64	1.41E-07	2.88E-05
8157193	158399	*zinc finger protein 483*	*ZNF483*	6.13	5.75	10.04	1.45E-07	2.91E-05
7980828	440193	*coiled-coil domain containing 88C*	*CCDC88C*	4.77	5.20	9.01	1.44E-07	2.91E-05
8097801	729830	*family with sequence similarity 160, member A1*	*FAM160A1*	6.12	6.17	10.06	1.43E-07	2.91E-05
8041048	2355	*FOS-like antigen 2*	*FOSL2*	10.88	11.05	7.68	1.49E-07	2.95E-05
8168589	7552	*zinc finger protein 711*	*ZNF711*	5.24	5.22	7.66	1.50E-07	2.95E-05
7957032	8089	*YEATS domain containing 4*	*YEATS4*	7.91	8.37	10.21	1.50E-07	2.95E-05
8106354	10788	*IQ motif containing GTPase activating protein 2*	*IQGAP2*	5.22	5.06	9.27	1.52E-07	2.97E-05
8079422	6997	*teratocarcinoma-derived growth factor 1*	*TDGF1*	4.78	4.82	12.23	1.58E-07	3.07E-05
8103769	3248	*hydroxyprostaglandin dehydrogenase 15-(NAD)*	*HPGD*	5.10	5.15	7.22	1.61E-07	3.11E-05
7962146	58516	*family with sequence similarity 60, member A*	*FAM60A*	5.74	6.23	11.29	1.63E-07	3.13E-05
8177732	3105, 3106	*major histocompatibility complex, class I, A | major histocompatibility complex, class I, B*	*HLA-A|HLA-B*	13.24	13.03	11.18	1.65E-07	3.14E-05
8155673	8395	*phosphatidylinositol-4-phosphate 5-kinase, type I, beta*	*PIP5K1B*	5.34	5.37	6.32	1.69E-07	3.16E-05
8146934	23643	*lymphocyte antigen 96*	*LY96*	8.71	8.48	4.68	1.68E-07	3.16E-05
8168517	2846	*lysophosphatidic acid receptor 4*	*LPAR4*	5.77	5.67	9.87	1.70E-07	3.17E-05
7997726	2294	*forkhead box F1*	*FOXF1*	9.59	7.51	6.67	1.74E-07	3.19E-05
8115099	5159	*platelet-derived growth factor receptor, beta polypeptide*	*PDGFRB*	12.38	11.90	7.47	1.77E-07	3.19E-05
7986049	64784	*CREB regulated transcription coactivator 3*	*CRTC3*	10.95	10.85	7.04	1.76E-07	3.19E-05
8177788	3133, 3106	*major histocompatibility complex, class I, E | major histocompatibility complex, class I, B*	*HLA-E|HLA-B*	11.84	11.39	9.44	1.73E-07	3.19E-05
8179103	3133, 3106	*major histocompatibility complex, class I, E | major histocompatibility complex, class I, B*	*HLA-E|HLA-B*	11.84	11.39	9.44	1.77E-07	3.19E-05
8056572	57405		*SPC25*	5.33	5.72	8.95	1.82E-07	3.26E-05

7934553	84858, 100131213	*zinc finger protein 503 | non-protein coding RNA 245*	*ZNF503|NCRNA00245*	11.16	10.63	7.24	1.84E-07	3.28E-05
7969640	9071, 22873	*claudin 10 | DAZ interacting protein 1*	*CLDN10|DZIP1*	5.58	5.53	7.70	1.86E-07	3.30E-05
7985213	1138	*cholinergic receptor, nicotinic, alpha 5*	*CHRNA5*	5.60	5.66	9.84	1.94E-07	3.41E-05
8171848	9468	*phosphate cytidylyltransferase 1, choline, beta*	*PCYT1B*	6.19	6.15	8.47	1.97E-07	3.42E-05
8123819	9521	*eukaryotic translation elongation factor 1 epsilon 1*	*EEF1E1*	7.38	7.90	9.91	1.97E-07	3.42E-05
8138857	79017	*gamma-glutamylcyclotransferase*	*GGCT*	7.79	8.26	10.14	1.98E-07	3.43E-05
8117888	5901		*RAN*	10.68	11.12	12.36	2.02E-07	3.44E-05
8170420	10046	*mastermind-like domain containing 1*	*MAMLD1*	9.76	10.87	6.56	2.01E-07	3.44E-05
7948908	9302, 23642	*small nucleolar RNA, C/D box 26 | small nucleolar RNA host gene 1 (non-protein coding)*	*SNORD26|SNHG1*	7.91	7.91	11.36	2.10E-07	3.56E-05
8171248	3730	*Kallmann syndrome 1 sequence*	*KAL1*	5.54	5.50	9.21	2.12E-07	3.58E-05
8080562	55540	*interleukin 17 receptor B*	*IL17RB*	5.56	5.42	7.10	2.17E-07	3.64E-05
8020411	6632	*small nuclear ribonucleoprotein D1 polypeptide 16kDa*	*SNRPD1*	7.37	7.72	9.85	2.26E-07	3.77E-05
8083360	116931	*mediator complex subunit 12-like*	*MED12L*	5.92	5.87	8.46	2.27E-07	3.77E-05
8078999	253639	*zinc finger protein 620*	*ZNF620*	6.66	6.95	8.96	2.32E-07	3.82E-05
7943577	54734		*RAB39*	5.79	6.00	7.78	2.40E-07	3.92E-05
7983239	548596, 1159	*creatine kinase, mitochondrial 1A | creatine kinase, mitochondrial 1B*	*CKMT1A|CKMT1B*	6.40	6.33	10.39	2.40E-07	3.92E-05
8109712	3161	*hyaluronan-mediated motility receptor (RHAMM)*	*HMMR*	5.47	5.83	9.17	2.42E-07	3.93E-05
7983256	548596, 1159	*creatine kinase, mitochondrial 1A | creatine kinase, mitochondrial 1B*	*CKMT1A|CKMT1B*	6.39	6.33	10.39	2.45E-07	3.95E-05
8124498	7754	*zinc finger protein 204, pseudogene*	*ZNF204P*	4.89	4.88	8.27	2.52E-07	3.98E-05
8173059	65267	*WNK lysine deficient protein kinase 3*	*WNK3*	5.26	5.28	9.24	2.52E-07	3.98E-05
8105899	153562	*MARVEL domain containing 2*	*MARVELD2*	5.68	5.82	9.07	2.51E-07	3.98E-05
8177498	153562	*MARVEL domain containing 2*	*MARVELD2*	5.69	5.83	9.06	2.52E-07	3.98E-05
8151334	9242	*musculin*	*MSC*	11.45	11.04	6.98	2.55E-07	4.00E-05
7961693	3945	*lactate dehydrogenase B*	*LDHB*	11.52	11.21	13.50	2.58E-07	4.01E-05

8133089	168374	*zinc finger protein 92*	*ZNF92*	6.77	6.81	9.89	2.59E-07	4.01E-05
8116835	2651	*glucosaminyl (N-acetyl) transferase 2, I-branching enzyme (I blood group)*	*GCNT2*	5.43	5.47	8.89	2.70E-07	4.13E-05
8009040	9902	*mannose receptor, C type 2*	*MRC2*	11.94	11.79	7.98	2.68E-07	4.13E-05
8147697	79977	*grainyhead-like 2 (Drosophila)*	*GRHL2*	5.85	6.00	9.05	2.70E-07	4.13E-05
7927186	83937	*Ras association (RalGDS/AF-6) domain family member 4*	*RASSF4*	9.72	7.06	7.49	2.74E-07	4.17E-05
8058498	7855	*frizzled homolog 5 (Drosophila)*	*FZD5*	7.04	6.79	10.85	2.81E-07	4.24E-05
7923189	9928	*kinesin family member 14*	*KIF14*	5.65	5.63	9.02	2.84E-07	4.27E-05
8097829	85462	*FH2 domain containing 1*	*FHDC1*	7.32	5.83	9.01	2.91E-07	4.35E-05
8069494	441666	*zinc finger protein 91 pseudogene*	*LOC441666*	5.07	5.06	7.23	2.96E-07	4.41E-05
7990657	23102	*TBC1 domain family, member 2B*	*TBC1D2B*	10.26	9.34	7.46	3.04E-07	4.48E-05
7981998	100033436	*small nucleolar RNA, C/D box 116-25*	*SNORD116-25*	7.36	7.61	10.29	3.04E-07	4.48E-05
7959882	5901		*RAN*	9.86	10.26	11.45	3.15E-07	4.62E-05
8022902	125476	*INO80 complex subunit C*	*INO80C*	5.89	6.03	7.69	3.20E-07	4.67E-05
8171205	57502	*neuroligin 4, X-linked*	*NLGN4X*	6.21	6.15	10.79	3.24E-07	4.68E-05
8085754	151648	*shugoshin-like 1 (S. pombe)*	*SGOL1*	5.82	6.26	9.96	3.23E-07	4.68E-05
7962212	5318	*plakophilin 2*	*PKP2*	7.05	7.16	11.05	3.35E-07	4.76E-05
8068633	10317	*UDP-Gal:betaGlcNAc beta 1,3-galactosyltransferase, polypeptide 5*	*B3GALT5*	5.79	5.86	7.42	3.34E-07	4.76E-05
8036172				7.47	7.61	6.96	3.33E-07	4.76E-05
8096130	1040	*CDP-diacylglycerol synthase (phosphatidate cytidylyltransferase) 1*	*CDS1*	5.68	5.67	7.80	3.39E-07	4.77E-05
8036351	342892	*zinc finger protein 850*	*ZNF850*	6.54	6.45	10.86	3.38E-07	4.77E-05
8101762	6622	*synuclein, alpha (non A4 component of amyloid precursor)*	*SNCA*	7.02	6.68	8.76	3.41E-07	4.78E-05
8083471	26084	*Rho guanine nucleotide exchange factor (GEF) 26*	*ARHGEF26*	5.26	5.13	7.97	3.44E-07	4.78E-05
8144378	55326	*1-acylglycerol-3-phosphate O-acyltransferase 5 (lysophosphatidic acid acyltransferase, epsilon)*	*AGPAT5*	6.71	7.04	10.41	3.43E-07	4.78E-05
8139488	3486	*insulin-like growth factor binding protein 3*	*IGFBP3*	11.10	12.89	8.01	3.48E-07	4.78E-05

7909628	28982	*feline leukemia virus subgroup C cellular receptor 1*	*FLVCR1*	6.69	6.74	10.76	3.48E-07	4.78E-05
8165794	4267		*CD99*	11.90	11.98	8.01	3.58E-07	4.87E-05
8034712	79883	*podocan-like 1*	*PODNL1*	10.69	10.90	6.71	3.59E-07	4.87E-05
7977440	9834, 29064	*KIAA0125 | family with sequence similarity 30, member A*	*KIAA0125| FAM30A*	7.44	7.68	6.87	3.58E-07	4.87E-05
8146092	3620	*indoleamine 2,3-dioxygenase 1*	*IDO1*	6.02	5.44	10.76	3.61E-07	4.87E-05
8176360	4267		*CD99*	11.90	11.99	8.01	3.71E-07	4.91E-05
8158250	51148	*cerebral endothelial cell adhesion molecule*	*CERCAM*	10.68	10.99	6.10	3.70E-07	4.91E-05
8164013	55342	*spermatid perinuclear RNA binding protein*	*STRBP*	5.70	5.61	9.63	3.70E-07	4.91E-05
8146794	80243	*phosphatidylinositol-3,4,5-trisphosphate-dependent Rac exchange factor 2*	*PREX2*	5.74	5.74	9.08	3.67E-07	4.91E-05
8043203	10791	*vesicle-associated membrane protein 5 (myobrevin)*	*VAMP5*	11.70	11.82	8.55	3.76E-07	4.95E-05
8079237	56992	*kinesin family member 15*	*KIF15*	5.70	5.82	8.76	3.78E-07	4.96E-05
8105842	64946	*centromere protein H*	*CENPH*	5.92	5.87	9.66	3.84E-07	4.99E-05
7982006	100033821	*small nucleolar RNA, C/D box 116-29*	*SNORD11 6-29*	6.47	6.71	9.56	3.84E-07	4.99E-05
7902861	23507	*leucine rich repeat containing 8 family, member B*	*LRRC8B*	5.94	5.87	9.41	3.91E-07	5.06E-05
7979307	9787	*discs, large (Drosophila) homolog-associated protein 5*	*DLGAP5*	6.80	6.93	11.24	3.96E-07	5.10E-05
8104107	205860	*tripartite motif family-like 2*	*TRIML2*	5.89	6.06	9.26	3.98E-07	5.10E-05
8078805	93	*activin A receptor, type IIB*	*ACVR2B*	6.68	6.58	8.84	4.08E-07	5.18E-05
7963567	3856	*keratin 8*	*KRT8*	7.81	7.94	11.54	4.07E-07	5.18E-05
7987636	11339	*Opa interacting protein 5*	*OIP5*	6.57	6.79	10.32	4.26E-07	5.39E-05
8042283	29094	*galectin-related protein*	*HSPC159*	8.53	8.03	11.51	4.29E-07	5.40E-05
8076481	1727	*cytochrome b5 reductase 3*	*CYB5R3*	12.86	12.82	10.50	4.34E-07	5.45E-05
7915733	5052	*peroxiredoxin 1*	*PRDX1*	11.42	11.26	12.77	4.41E-07	5.47E-05
7981945	6638	*small nuclear ribonucleoprotein polypeptide N*	*SNRPN*	4.74	4.61	9.48	4.40E-07	5.47E-05
8109407	55568	*UDP-N-acetyl-alpha-D-galactosamine:polypeptide N-acetylgalactosaminyltransferase 10 (GalNAc-T10)*	*GALNT10*	9.81	10.53	8.22	4.41E-07	5.47E-05
7956593	10956	*osteosarcoma amplified 9, endoplasmic reticulum lectin*	*OS9*	12.14	12.02	10.10	4.45E-07	5.47E-05
8102389	55345	*chromosome 4 open reading frame 21*	*C4orf21*	5.53	5.70	8.65	4.48E-07	5.47E-05

7938951	203859	*anoctamin 5*	*ANO5*	4.99	4.99	7.44	4.46E-07	5.47E-05
8027363	730087	*zinc finger protein 726*	*ZNF726*	4.97	4.86	6.41	4.53E-07	5.52E-05
8102076	1062	*centromere protein E, 312kDa*	*CENPE*	5.78	6.01	8.69	4.91E-07	5.76E-05
8145005	2039	*erythrocyte membrane protein band 4.9 (dematin)*	*EPB49*	8.28	7.06	6.28	4.81E-07	5.76E-05
8081657	4345		*CD200*	6.78	6.91	10.97	4.90E-07	5.76E-05
8144786	6542	*solute carrier family 7 (cationic amino acid transporter, y+ system), member 2*	*SLC7A2*	5.79	5.68	10.18	4.83E-07	5.76E-05
8145611	7976	*frizzled homolog 3 (Drosophila)*	*FZD3*	5.66	5.76	10.05	4.75E-07	5.76E-05
7948332	9404	*leupaxin*	*LPXN*	9.05	10.17	7.06	4.96E-07	5.76E-05
8138566	10643	*insulin-like growth factor 2 mRNA binding protein 3*	*IGF2BP3*	7.33	6.36	11.07	4.97E-07	5.76E-05
7942064	51083	*galanin prepropeptide*	*GAL*	7.63	7.89	12.54	4.77E-07	5.76E-05
8096361	51191	*hect domain and RLD 5*	*HERC5*	5.34	5.42	8.58	4.96E-07	5.76E-05
7926259	55388	*minichromosome maintenance complex component 10*	*MCM10*	6.32	6.49	9.73	4.95E-07	5.76E-05
8112260	55789	*DEP domain containing 1B*	*DEPDC1B*	6.22	6.12	10.23	4.84E-07	5.76E-05
8022420	162655	*zinc finger protein 519*	*ZNF519*	5.87	5.67	7.54	4.94E-07	5.76E-05
8027254	7643, 81931	*zinc finger protein 90 | zinc finger protein 93*	*ZNF90|ZNF93*	6.94	7.09	11.18	4.99E-07	5.76E-05
7973709	4776	*nuclear factor of activated T-cells, cytoplasmic, calcineurin-dependent 4*	*NFATC4*	10.38	9.80	7.55	5.05E-07	5.79E-05
8095751	25849	*prostate androgen-regulated mucin-like protein 1*	*PARM1*	5.71	5.82	8.49	5.04E-07	5.79E-05
7953040	775	*calcium channel, voltage-dependent, L type, alpha 1C subunit*	*CACNA1C*	10.08	9.43	6.36	5.10E-07	5.80E-05
7948900	9299	*small nucleolar RNA, C/D box 30*	*SNORD30*	7.76	7.76	10.76	5.10E-07	5.80E-05
8098690	132625	*zinc finger protein 42 homolog (mouse)*	*ZFP42*	5.72	5.72	11.49	5.15E-07	5.83E-05
7910190	339500	*zinc finger protein 678*	*ZNF678*	5.45	5.40	8.26	5.25E-07	5.93E-05
8104394	108	*adenylate cyclase 2 (brain)*	*ADCY2*	7.00	6.92	11.05	5.37E-07	6.03E-05
8052882	119	*adducin 2 (beta)*	*ADD2*	6.53	7.00	10.40	5.42E-07	6.04E-05
8098576	291	*solute carrier family 25 (mitochondrial carrier; adenine nucleotide translocator), member 4*	*SLC25A4*	8.78	8.93	11.05	5.52E-07	6.04E-05
8007100	3487	*insulin-like growth factor binding protein 4*	*IGFBP4*	13.79	13.28	10.77	5.47E-07	6.04E-05

8152512	4982	*tumor necrosis factor receptor superfamily, member 11b*	*TNFRSF11B*	11.91	11.27	6.03	5.53E-07	6.04E-05
8106730	7518	*X-ray repair complementing defective repair in Chinese hamster cells 4*	*XRCC4*	7.27	7.71	9.14	5.57E-07	6.04E-05
8099476	8842	*prominin 1*	*PROM1*	5.53	5.38	10.05	5.55E-07	6.04E-05
8057554	27101	*calcyclin binding protein*	*CACYBP*	8.74	9.04	11.10	5.42E-07	6.04E-05
8071086	27443	*cat eye syndrome chromosome region, candidate 2*	*CECR2*	6.22	6.14	9.59	5.51E-07	6.04E-05
8125919	2289, 285847	*FK506 binding protein 5 | hypothetical LOC285847*	*FKBP5|LOC285847*	6.22	6.25	9.07	5.55E-07	6.04E-05
7937330	10581	*interferon induced transmembrane protein 2 (1-8D)*	*IFITM2*	12.59	12.48	9.84	5.59E-07	6.04E-05
8039928	728833, 729533, 653820, 554282	*family with sequence similarity 72, member D | family with sequence similarity 72, member A | family with sequence similarity 72, member B | family with sequence similarity 72, member C*	*FAM72D|FAM72A|FAM72B|FAM72C*	6.75	6.95	10.39	5.62E-07	6.05E-05
8164269	2022	*endoglin*	*ENG*	12.15	12.15	7.38	5.69E-07	6.09E-05
7929078	9585	*kinesin family member 20B*	*KIF20B*	5.83	6.21	9.26	5.70E-07	6.09E-05
8113666	57556	*sema domain, transmembrane domain (TM), and cytoplasmic domain, (semaphorin) 6A*	*SEMA6A*	6.96	6.17	12.31	5.86E-07	6.23E-05
7965760	120863	*DEP domain containing 4*	*DEPDC4*	4.99	5.14	7.21	5.87E-07	6.23E-05
8126058	51645	*peptidylprolyl isomerase (cyclophilin)-like 1*	*PPIL1*	7.89	8.34	10.41	6.00E-07	6.32E-05
8158771	83543	*allograft inflammatory factor 1-like*	*AIF1L*	6.30	6.47	9.33	5.98E-07	6.32E-05
8099967	54502	*RNA binding motif protein 47*	*RBM47*	6.92	6.92	9.56	6.12E-07	6.43E-05
8160024	2731	*glycine dehydrogenase (decarboxylating)*	*GLDC*	7.05	7.22	10.67	6.20E-07	6.45E-05
7981976	100033426	*small nucleolar RNA, C/D box 116-14*	*SNORD116-14*	12.14	12.25	13.46	6.17E-07	6.45E-05
7904452	728833, 729533, 653820, 554282	*family with sequence similarity 72, member D | family with sequence similarity 72, member A | family with sequence similarity 72, member B | family with sequence similarity 72, member C*	*FAM72D|FAM72A|FAM72B|FAM72C*	6.81	7.05	10.54	6.21E-07	6.45E-05
7985159	1381	*cellular retinoic acid binding protein 1*	*CRABP1*	7.01	6.84	10.87	6.27E-07	6.49E-05
7948906	9301, 23642	*small nucleolar RNA, C/D box 27 | small nucleolar RNA host gene 1 (non-protein coding)*	*SNORD27|SNHG1*	6.08	5.99	9.10	6.33E-07	6.53E-05
8102643	890	*cyclin A2*	*CCNA2*	8.05	8.86	11.67	6.37E-07	6.55E-05

7946957	113174	*serum amyloid A-like 1*	*SAAL1*	6.92	6.98	9.39	6.40E-07	6.56E-05
7965410	1634	*decorin*	*DCN*	12.54	12.38	5.52	6.51E-07	6.60E-05
8063078	5476	*cathepsin A*	*CTSA*	13.17	12.98	11.57	6.48E-07	6.60E-05
8043835	150590, 51263	*chromosome 2 open reading frame 15 | mitochondrial ribosomal protein L30*	*C2orf15|MRPL30*	5.07	5.18	6.95	6.51E-07	6.60E-05
8084717	6480	*ST6 beta-galactosamide alpha-2,6-sialyltranferase 1*	*ST6GAL1*	6.44	6.44	11.39	6.61E-07	6.67E-05
8146448	29088	*mitochondrial ribosomal protein L15*	*MRPL15*	8.17	8.67	10.14	6.63E-07	6.67E-05
7908407	6233	*ribosomal protein S27a*	*RPS27A*	10.22	10.36	11.18	6.67E-07	6.68E-05
7985224	23102, 646938	*TBC1 domain family, member 2B | TBC1 domain family member 2B pseudogene*	*TBC1D2B|LOC646938*	10.10	9.23	7.04	6.69E-07	6.68E-05
7924733	142	*poly (ADP-ribose) polymerase 1*	*PARP1*	9.02	8.78	11.61	6.97E-07	6.87E-05
8022711	1824	*desmocollin 2*	*DSC2*	5.49	5.63	9.61	6.93E-07	6.87E-05
7929438	3070	*helicase, lymphoid-specific*	*HELLS*	5.62	5.54	10.10	6.98E-07	6.87E-05
8078435	131405	*tripartite motif-containing 71*	*TRIM71*	7.85	7.88	11.30	7.00E-07	6.87E-05
7919591	728833, 729533, 653820, 554282	*family with sequence similarity 72, member D | family with sequence similarity 72, member A | family with sequence similarity 72, member B | family with sequence similarity 72, member C*	*FAM72D|FAM72A|FAM72B|FAM72C*	6.31	6.22	9.96	6.92E-07	6.87E-05
8053315	80059	*leucine rich repeat transmembrane neuronal 4*	*LRRTM4*	5.96	6.04	9.53	7.06E-07	6.91E-05
8038913	65251	*zinc finger protein 649*	*ZNF649*	6.63	6.86	10.69	7.12E-07	6.92E-05
7969576	407975, 406952, 406979, 406980, 406982, 407048, 406953	*MIR17 host gene (non-protein coding) | microRNA 17 | microRNA 19a | microRNA 19b-1 | microRNA 20a | microRNA 92a-1 | microRNA 18a*	*MIR17HG|MIR19A|MIR19B1|MIR20A|MIR92A1|MIR18A*	5.97	6.25	9.50	7.11E-07	6.92E-05
7946340	79608	*resistance to inhibitors of cholinesterase 3 homolog (C. elegans)*	*RIC3*	5.31	5.39	7.43	7.21E-07	6.99E-05
8033635	81794	*ADAM metallopeptidase with thrombospondin type 1 motif, 10*	*ADAMTS10*	8.69	8.79	7.53	7.26E-07	6.99E-05
7958211	121053	*chromosome 12 open reading frame 45*	*C12orf45*	6.46	6.50	8.54	7.24E-07	6.99E-05

8077993	79188	*transmembrane protein 43*	*TMEM43*	11.74	11.82	9.80	7.37E-07	7.07E-05
8099107	55646	*Ly1 antibody reactive homolog (mouse)*	*LYAR*	6.22	6.32	9.06	7.40E-07	7.08E-05
8174047	1678	*translocase of inner mitochondrial membrane 8 homolog A (yeast)*	*TIMM8A*	6.84	7.36	9.85	7.46E-07	7.10E-05
7982098	338428, 338429	*small nucleolar RNA, C/D box 109A | small nucleolar RNA, C/D box 109B*	*SNORD109A|SNORD109B*	6.32	6.02	10.61	7.57E-07	7.17E-05
8064844	5111, 100302739	*proliferating cell nuclear antigen | PCNA antisense RNA (non-protein coding)*	*PCNA|PCNA-AS*	7.79	8.20	10.82	7.57E-07	7.17E-05
7981947	338428, 338429	*small nucleolar RNA, C/D box 109A | small nucleolar RNA, C/D box 109B*	*SNORD109A|SNORD109B*	6.31	6.03	10.60	7.60E-07	7.17E-05
7955694	3489	*insulin-like growth factor binding protein 6*	*IGFBP6*	12.22	12.26	8.42	7.69E-07	7.23E-05
8130438	51106, 26230	*transcription factor B1, mitochondrial | T-cell lymphoma invasion and metastasis 2*	*TFB1M|TIAM2*	6.69	6.97	9.25	7.77E-07	7.28E-05
7983867	84952	*cingulin-like 1*	*CGNL1*	6.11	6.17	8.68	7.99E-07	7.44E-05
8139421	94239	*H2A histone family, member V*	*H2AFV*	9.36	9.52	10.90	7.98E-07	7.44E-05
8034084	10053	*adaptor-related protein complex 1, mu 2 subunit*	*AP1M2*	6.40	6.49	9.60	8.06E-07	7.48E-05
8155707	9414	*tight junction protein 2 (zona occludens 2)*	*TJP2*	8.53	7.54	10.69	8.14E-07	7.53E-05
7968637	8900	*cyclin A1*	*CCNA1*	6.75	6.58	8.92	8.18E-07	7.54E-05
7909146	728833, 729533, 653820, 554282	*family with sequence similarity 72, member D | family with sequence similarity 72, member A | family with sequence similarity 72, member B | family with sequence similarity 72, member C*	*FAM72D|FAM72A|FAM72B|FAM72C*	6.74	6.99	10.50	8.20E-07	7.54E-05
8151475	7163	*tumor protein D52*	*TPD52*	5.07	5.13	7.32	8.24E-07	7.55E-05
8002760	100132346	*10 kDa heat shock protein, mitochondrial-like*	*LOC100132346*	5.39	5.60	7.51	8.28E-07	7.57E-05
7949503	30008	*EGF-containing fibulin-like extracellular matrix protein 2*	*EFEMP2*	12.38	12.23	8.39	8.33E-07	7.59E-05
7949588	57124		*CD248*	12.15	11.98	6.45	8.36E-07	7.59E-05
8142497	83992	*cortactin binding protein 2*	*CTTNBP2*	5.64	5.69	7.69	8.39E-07	7.59E-05
7978766	2287	*FK506 binding protein 3, 25kDa*	*FKBP3*	6.87	7.04	8.59	8.45E-07	7.62E-05
8029950	30846	*EH-domain containing 2*	*EHD2*	12.22	12.23	8.21	8.51E-07	7.64E-05

8175250	406981	*microRNA 19b-2*	*MIR19B2*	4.62	4.60	6.59	8.52E-07	7.64E-05
8091600	23007	*phospholipase C, eta 1*	*PLCH1*	6.06	5.91	8.91	8.66E-07	7.74E-05
8157650	5742	*prostaglandin-endoperoxide synthase 1 (prostaglandin G/H synthase and cyclooxygenase)*	*PTGS1*	11.48	9.33	6.81	8.78E-07	7.82E-05
7967486	80212	*coiled-coil domain containing 92*	*CCDC92*	11.00	11.04	5.63	8.86E-07	7.87E-05
7913852	79000	*chromosome 1 open reading frame 135*	*C1orf135*	6.04	6.28	9.14	8.99E-07	7.96E-05
8095221	10606	*phosphoribosylaminoimidazole carboxylase, phosphoribosylaminoimidazole succinocarboxamide synthetase*	*PAICS*	8.94	9.51	11.45	9.04E-07	7.98E-05
8065403	1471	*cystatin C*	*CST3*	12.85	12.90	10.11	9.31E-07	8.19E-05
8121510	84154	*ribosome production factor 2 homolog (S. cerevisiae)*	*RPF2*	7.31	7.89	9.48	9.41E-07	8.26E-05
8153002	10397	*N-myc downstream regulated 1*	*NDRG1*	11.96	12.19	8.04	9.47E-07	8.28E-05
7957338	6857	*synaptotagmin I*	*SYT1*	5.97	6.54	10.43	9.54E-07	8.29E-05
8027268	7617	*zinc finger protein 66, pseudogene*	*ZNF66P*	6.70	6.65	9.83	9.51E-07	8.29E-05
7957649	6636	*small nuclear ribonucleoprotein polypeptide F*	*SNRPF*	6.44	6.75	8.36	9.64E-07	8.33E-05
7946365	65975	*serine/threonine kinase 33*	*STK33*	5.48	5.24	7.86	9.62E-07	8.33E-05
8160459	1993	*ELAV (embryonic lethal, abnormal vision, Drosophila)-like 2 (Hu antigen B)*	*ELAVL2*	5.63	5.57	8.36	9.73E-07	8.37E-05
7919715	81611	*acidic (leucine-rich) nuclear phosphoprotein 32 family, member E*	*ANP32E*	6.34	6.30	9.03	9.75E-07	8.37E-05
8037251	5676, 5669, 5672, 440533	*pregnancy specific beta-1-glycoprotein 7 (gene/pseudogene) | pregnancy specific beta-1-glycoprotein 1 | pregnancy specific beta-1-glycoprotein 4 | pregnancy specific beta-1-glycoprotein 8*	*PSG7|PSG1|PSG4|PSG8*	10.83	10.39	6.26	9.77E-07	8.37E-05
7973336	4323	*matrix metallopeptidase 14 (membrane-inserted)*	*MMP14*	11.50	11.57	8.95	9.84E-07	8.38E-05
7939642	90993	*cAMP responsive element binding protein 3-like 1*	*CREB3L1*	12.08	11.83	7.41	9.81E-07	8.38E-05
7925048	54583	*egl nine homolog 1 (C. elegans)*	*EGLN1*	8.79	9.15	7.61	9.87E-07	8.38E-05
8006005	51268	*pipecolic acid oxidase*	*PIPOX*	6.86	6.78	10.38	9.90E-07	8.38E-05
7909841	4139	*MAP/microtubule affinity-regulating kinase 1*	*MARK1*	5.65	5.83	8.64	1.00E-06	8.44E-05
8067869	54033	*RNA binding motif protein 11*	*RBM11*	4.89	4.81	6.27	1.00E-06	8.44E-05
7982002	100033439	*small nucleolar RNA, C/D box 116-27*	*SNORD116-27*	7.10	7.28	9.89	1.01E-06	8.47E-05

7981953	100033415, 100033421, 100033417, 100033419, 100033420	*small nucleolar RNA, C/D box 116-3 | small nucleolar RNA, C/D box 116-9 | small nucleolar RNA, C/D box 116-5 | small nucleolar RNA, C/D box 116-7 | small nucleolar RNA, C/D box 116-8*	*SNORD116-3|SNORD116-9|SNORD116-5|SNORD116-7|SNORD116-8*	11.50	11.71	12.64	1.02E-06	8.47E-05
7981966	100033415, 100033421, 100033417, 100033419, 100033420	*small nucleolar RNA, C/D box 116-3 | small nucleolar RNA, C/D box 116-9 | small nucleolar RNA, C/D box 116-5 | small nucleolar RNA, C/D box 116-7 | small nucleolar RNA, C/D box 116-8*	*SNORD116-3|SNORD116-9|SNORD116-5|SNORD116-7|SNORD116-8*	11.50	11.71	12.64	1.01E-06	8.47E-05
8046186	151230, 493911	*kelch-like 23 (Drosophila) | phosphatase, orphan 2*	*KLHL23|PHOSPHO2*	6.28	6.03	8.40	1.03E-06	8.53E-05
7922756	23057	*nicotinamide nucleotide adenylyltransferase 2*	*NMNAT2*	6.53	6.88	8.94	1.06E-06	8.76E-05
8020164	2774	*guanine nucleotide binding protein (G protein), alpha activating activity polypeptide, olfactory type*	*GNAL*	6.53	6.41	8.68	1.06E-06	8.78E-05
7898057	10630	*podoplanin*	*PDPN*	7.60	8.82	11.50	1.06E-06	8.78E-05
8162179	2619	*growth arrest-specific 1*	*GAS1*	10.30	10.03	8.19	1.08E-06	8.84E-05
8005064	9912	*Rho GTPase activating protein 44*	*ARHGAP44*	6.60	6.51	8.20	1.08E-06	8.84E-05
8141107	10165	*solute carrier family 25, member 13 (citrin)*	*SLC25A13*	7.19	7.58	10.94	1.08E-06	8.84E-05
7901765	51361	*hook homolog 1 (Drosophila)*	*HOOK1*	4.87	4.82	8.02	1.08E-06	8.84E-05
8072160	84133	*zinc and ring finger 3*	*ZNRF3*	7.28	7.52	8.90	1.09E-06	8.87E-05
8114536	340061	*transmembrane protein 173*	*TMEM173*	11.23	10.93	7.02	1.09E-06	8.87E-05
7915500	149466	*chromosome 1 open reading frame 210*	*C1orf210*	6.57	6.62	9.05	1.10E-06	8.88E-05

8153021	6482	*ST3 beta-galactoside alpha-2,3-sialyltransferase 1*	*ST3GAL1*	9.73	9.69	6.77	1.11E-06	8.92E-05
8131944	9603	*nuclear factor (erythroid-derived 2)-like 3*	*NFE2L3*	7.71	7.34	10.36	1.11E-06	8.92E-05
8147503	55353	*lysosomal protein transmembrane 4 beta*	*LAPTM4B*	9.90	9.86	12.75	1.11E-06	8.92E-05
7918157	10451	*vav 3 guanine nucleotide exchange factor*	*VAV3*	4.98	5.01	6.95	1.12E-06	8.92E-05
7995354	23594	*origin recognition complex, subunit 6*	*ORC6*	5.74	6.18	8.68	1.12E-06	8.92E-05
7945045	29118	*DEAD (Asp-Glu-Ala-Asp) box polypeptide 25*	*DDX25*	5.56	5.64	9.04	1.12E-06	8.92E-05
7902913	8317	*cell division cycle 7 homolog (S. cerevisiae)*	*CDC7*	5.78	5.92	9.12	1.13E-06	8.99E-05
8179049	3137, 3105	*major histocompatibility complex, class I, J (pseudogene) | major histocompatibility complex, class I, A*	*HLA-J|HLA-A*	12.87	12.73	10.71	1.14E-06	9.01E-05
7963946	4327	*matrix metallopeptidase 19*	*MMP19*	9.70	9.18	6.57	1.15E-06	9.02E-05
8062211	84154	*ribosome production factor 2 homolog (S. cerevisiae)*	*RPF2*	9.02	9.59	11.36	1.15E-06	9.02E-05
7902353	431707	*LIM homeobox 8*	*LHX8*	9.52	6.11	5.85	1.15E-06	9.02E-05
7934026	1763	*DNA replication helicase 2 homolog (yeast)*	*DNA2*	5.78	5.88	9.11	1.17E-06	9.18E-05
8041781	2034	*endothelial PAS domain protein 1*	*EPAS1*	12.65	12.24	7.42	1.20E-06	9.36E-05
8005877	6201	*ribosomal protein S7*	*RPS7*	8.36	8.47	9.33	1.20E-06	9.37E-05
8170538	55879	*gamma-aminobutyric acid (GABA) receptor, theta*	*GABRQ*	6.10	6.40	8.87	1.21E-06	9.41E-05
8139680	23242	*cordon-bleu homolog (mouse)*	*COBL*	6.14	6.29	9.25	1.23E-06	9.54E-05
8160521	79817	*MOB1, Mps One Binder kinase activator-like 2B (yeast)*	*MOBKL2B*	6.41	6.49	8.34	1.26E-06	9.69E-05
8102371	55345	*chromosome 4 open reading frame 21*	*C4orf21*	6.18	6.13	8.52	1.26E-06	9.72E-05
7923086	259266	*asp (abnormal spindle) homolog, microcephaly associated (Drosophila)*	*ASPM*	6.48	6.63	9.53	1.29E-06	9.87E-05
8088285	8820	*HESX homeobox 1*	*HESX1*	5.11	5.19	6.30	1.30E-06	9.96E-05
8069301	1292	*collagen, type VI, alpha 2*	*COL6A2*	12.02	12.04	8.52	1.33E-06	1.02E-04
8116848	55003	*PAK1 interacting protein 1*	*PAK1IP1*	7.74	8.46	10.67	1.34E-06	1.02E-04
8171561	10389	*sex comb on midleg-like 2 (Drosophila)*	*SCML2*	5.82	5.77	8.68	1.36E-06	1.03E-04
7955873	3223, 3221, 3222	*homeobox C6 | homeobox C4 | homeobox C5*	*HOXC6|HOXC4|HOXC5*	6.18	9.59	5.88	1.36E-06	1.03E-04
8001007	5652	*protease, serine, 8*	*PRSS8*	6.72	6.59	9.50	1.38E-06	1.04E-04

8145418	157313	*cell division cycle associated 2*	*CDCA2*	6.80	6.92	9.75	1.39E-06	1.05E-04
8121043	23595	*origin recognition complex, subunit 3*	*ORC3*	9.30	9.23	11.09	1.42E-06	1.07E-04
7962274	55605	*kinesin family member 21A*	*KIF21A*	5.69	5.81	8.50	1.43E-06	1.07E-04
8084891	131583	*family with sequence similarity 43, member A*	*FAM43A*	10.84	9.20	7.31	1.42E-06	1.07E-04
7956867	8091	*high mobility group AT-hook 2*	*HMGA2*	7.31	7.16	9.98	1.46E-06	1.09E-04
8069269	1291	*collagen, type VI, alpha 1*	*COL6A1*	12.98	12.96	8.99	1.48E-06	1.10E-04
7981992	100033433	*small nucleolar RNA, C/D box 116-22*	*SNORD116-22*	6.40	6.56	9.52	1.48E-06	1.10E-04
7981964	100033420, 100033415, 100033421	*small nucleolar RNA, C/D box 116-8 | small nucleolar RNA, C/D box 116-3 | small nucleolar RNA, C/D box 116-9*	*SNORD116-8|SNORD116-3|SNORD116-9*	10.96	11.16	12.10	1.49E-06	1.10E-04
8104234	9319	*thyroid hormone receptor interactor 13*	*TRIP13*	7.65	8.34	10.59	1.49E-06	1.10E-04
8138277	221806	*von Willebrand factor D and EGF domains*	*VWDE*	5.69	5.70	9.14	1.50E-06	1.10E-04
8087337	3913	*laminin, beta 2 (laminin S)*	*LAMB2*	12.07	11.87	8.70	1.52E-06	1.10E-04
7934997	5507	*protein phosphatase 1, regulatory (inhibitor) subunit 3C*	*PPP1R3C*	10.01	11.21	6.32	1.52E-06	1.10E-04
8018966	7077	*TIMP metallopeptidase inhibitor 2*	*TIMP2*	13.04	13.04	9.78	1.53E-06	1.10E-04
8028991	29785	*cytochrome P450, family 2, subfamily S, polypeptide 1*	*CYP2S1*	8.87	7.68	11.98	1.51E-06	1.10E-04
8131957	29887	*sorting nexin 10*	*SNX10*	5.98	5.83	8.19	1.53E-06	1.10E-04
8021349	51046	*ST8 alpha-N-acetyl-neuraminide alpha-2,8-sialyltransferase 3*	*ST8SIA3*	5.31	5.24	7.03	1.53E-06	1.10E-04
8146517	79145	*coiled-coil-helix-coiled-coil-helix domain containing 7*	*CHCHD7*	5.96	5.88	7.44	1.51E-06	1.10E-04
7968678	341640	*FRAS1 related extracellular matrix protein 2*	*FREM2*	5.31	5.24	8.94	1.53E-06	1.10E-04
7999903	400506	*chromosome 16 open reading frame 88*	*C16orf88*	7.40	7.38	9.71	1.53E-06	1.10E-04
7924712	286826	*lin-9 homolog (C. elegans)*	*LIN9*	6.33	6.55	8.64	1.55E-06	1.11E-04
8027348	100129543	*zinc finger protein 730*	*ZNF730*	6.34	6.26	11.19	1.55E-06	1.11E-04
8012126	1366	*claudin 7*	*CLDN7*	7.78	7.38	11.43	1.59E-06	1.12E-04
7903786	1435	*colony stimulating factor 1 (macrophage)*	*CSF1*	11.07	11.10	7.44	1.59E-06	1.12E-04

7936734	2263	*fibroblast growth factor receptor 2*	*FGFR2*	6.27	6.58	10.48	1.60E-06	1.12E-04
7964119	6773	*signal transducer and activator of transcription 2, 113kDa*	*STAT2*	11.10	10.49	8.46	1.60E-06	1.12E-04
8081219	10402	*ST3 beta-galactoside alpha-2,3-sialyltransferase 6*	*ST3GAL6*	5.57	5.61	7.65	1.57E-06	1.12E-04
7971838	11061	*leukocyte cell derived chemotaxin 1*	*LECT1*	5.62	5.59	9.43	1.58E-06	1.12E-04
8081343	54931	*RNA (guanine-9-) methyltransferase domain containing 1*	*RG9MTD1*	7.63	7.79	9.83	1.61E-06	1.12E-04
8096091	79725	*THAP domain containing 9*	*THAP9*	6.28	5.91	8.33	1.58E-06	1.12E-04
8117813	3137, 3105	*major histocompatibility complex, class I, J (pseudogene) | major histocompatibility complex, class I, A*	*HLA-J|HLA-A*	12.83	12.67	10.85	1.59E-06	1.12E-04
8040334				13.20	13.17	11.73	1.60E-06	1.12E-04
8043155				7.22	7.37	6.61	1.61E-06	1.12E-04
8099172	1400	*collapsin response mediator protein 1*	*CRMP1*	7.49	7.63	9.95	1.61E-06	1.12E-04
8022473	114799	*establishment of cohesion 1 homolog 1 (S. cerevisiae)*	*ESCO1*	6.45	6.55	8.83	1.62E-06	1.13E-04
8112274	79993	*ELOVL family member 7, elongation of long chain fatty acids (yeast)*	*ELOVL7*	5.47	5.61	8.06	1.64E-06	1.13E-04
8054580	699	*budding uninhibited by benzimidazoles 1 homolog (yeast)*	*BUB1*	6.64	6.80	10.89	1.68E-06	1.14E-04
7979505	6495	*SIX homeobox 1*	*SIX1*	11.64	8.91	6.73	1.68E-06	1.14E-04
8134117	8321	*frizzled homolog 1 (Drosophila)*	*FZD1*	8.32	8.69	7.27	1.68E-06	1.14E-04
8146945	54332	*ganglioside-induced differentiation-associated protein 1*	*GDAP1*	6.52	6.27	8.99	1.66E-06	1.14E-04
8036079	93099	*dermokine*	*DMKN*	7.72	7.89	10.79	1.67E-06	1.14E-04
8027233	94039	*zinc finger protein 101*	*ZNF101*	7.98	7.86	9.53	1.67E-06	1.14E-04
7958253	387882	*chromosome 12 open reading frame 75*	*C12orf75*	11.31	11.96	9.86	1.68E-06	1.14E-04

7981958	100033417, 100033419, 100033415, 100033421	*small nucleolar RNA, C/D box 116-5 | small nucleolar RNA, C/D box 116-7 | small nucleolar RNA, C/D box 116-3 | small nucleolar RNA, C/D box 116-9*	*SNORD116-5|SNORD116-7|SNORD116-3|SNORD116-9*	10.57	10.83	12.10	1.69E-06	1.15E-04
7981962	100033417, 100033419, 100033415, 100033421	*small nucleolar RNA, C/D box 116-5 | small nucleolar RNA, C/D box 116-7 | small nucleolar RNA, C/D box 116-3 | small nucleolar RNA, C/D box 116-9*	*SNORD116-5|SNORD116-7|SNORD116-3|SNORD116-9*	10.57	10.83	12.10	1.69E-06	1.15E-04
8177222	100133941		*CD24*	5.42	5.37	12.15	1.70E-06	1.15E-04
7937772	3481, 723961	*insulin-like growth factor 2 (somatomedin A) | INS-IGF2 readthrough transcript*	*IGF2|INS-IGF2*	10.05	7.53	6.37	1.70E-06	1.15E-04
8115327	6678	*secreted protein, acidic, cysteine-rich (osteonectin)*	*SPARC*	13.74	13.83	12.32	1.72E-06	1.16E-04
8154381	286343	*chromosome 9 open reading frame 150*	*C9orf150*	9.89	11.09	6.60	1.77E-06	1.19E-04
8105181	10884	*mitochondrial ribosomal protein S30*	*MRPS30*	9.11	8.97	10.56	1.77E-06	1.19E-04
8112121	153129	*solute carrier family 38, member 9*	*SLC38A9*	8.26	8.17	9.94	1.79E-06	1.20E-04
8147049	2171, 220832	*fatty acid binding protein 5 (psoriasis-associated) | fatty acid binding protein 5 pseudogene 3*	*FABP5|FABP5P3*	5.98	5.96	10.08	1.79E-06	1.20E-04
8151423	56704	*junctophilin 1*	*JPH1*	6.20	6.37	9.14	1.81E-06	1.21E-04
8106403	2150	*coagulation factor II (thrombin) receptor-like 1*	*F2RL1*	6.80	7.09	9.40	1.83E-06	1.21E-04
8096301	6696	*secreted phosphoprotein 1*	*SPP1*	5.95	6.29	12.22	1.83E-06	1.21E-04
8028254	390927	*zinc finger protein 793*	*ZNF793*	6.63	6.58	7.99	1.83E-06	1.21E-04
8121850	23493	*hairy/enhancer-of-split related with YRPW motif 2*	*HEY2*	5.84	5.94	10.56	1.84E-06	1.21E-04
7948904	9300	*small nucleolar RNA, C/D box 28*	*SNORD28*	7.89	7.82	11.45	1.85E-06	1.21E-04
8128383	51805	*coenzyme Q3 homolog, methyltransferase (S. cerevisiae)*	*COQ3*	6.81	7.00	9.45	1.85E-06	1.21E-04

8093332	642280	*zinc finger protein 876, pseudogene*	*ZNF876P*	6.77	6.67	9.81	1.86E-06	1.22E-04
8020702	6875	*TAF4b RNA polymerase II, TATA box binding protein (TBP)-associated factor, 105kDa*	*TAF4B*	5.74	6.05	8.40	1.88E-06	1.23E-04
8129045	3066	*histone deacetylase 2*	*HDAC2*	9.36	9.64	11.75	1.91E-06	1.24E-04
8126347	25862	*ubiquitin specific peptidase 49*	*USP49*	7.16	7.13	8.13	1.91E-06	1.24E-04
7975268	384, 10490	*arginase, type II | vesicle transport through interaction with t-SNAREs homolog 1B (yeast)*	*ARG2|VTI 1B*	6.30	7.30	10.06	1.91E-06	1.24E-04
8004510	968		*CD68*	11.58	11.96	7.74	1.93E-06	1.24E-04
8075126	4330	*meningioma (disrupted in balanced translocation) 1*	*MN1*	10.80	9.99	7.33	1.92E-06	1.24E-04
8027272	7639	*zinc finger protein 85*	*ZNF85*	6.99	7.13	9.17	1.93E-06	1.24E-04
7955865	3225	*homeobox C9*	*HOXC9*	5.96	8.76	5.68	1.95E-06	1.25E-04
7952914	84318	*coiled-coil domain containing 77*	*CCDC77*	6.58	6.90	9.10	1.95E-06	1.25E-04
8179731	3106	*major histocompatibility complex, class I, B*	*HLA-B*	13.20	13.06	11.02	1.97E-06	1.25E-04
8169984	3251	*hypoxanthine phosphoribosyltransferase 1*	*HPRT1*	8.60	8.94	10.51	1.97E-06	1.25E-04
8058869	7145	*tensin 1*	*TNS1*	10.59	10.83	7.07	1.97E-06	1.25E-04
7983512	58472	*sulfide quinone reductase-like (yeast)*	*SQRDL*	9.63	9.11	5.90	1.97E-06	1.25E-04
8112841	9456	*homer homolog 1 (Drosophila)*	*HOMER1*	6.37	6.87	9.75	1.99E-06	1.26E-04
8100578	2044	*EPH receptor A5*	*EPHA5*	7.61	5.71	5.54	2.03E-06	1.27E-04
8040430	7447	*visinin-like 1*	*VSNL1*	5.98	6.24	11.68	2.03E-06	1.27E-04
8051298	79623	*UDP-N-acetyl-alpha-D-galactosamine:polypeptide N-acetylgalactosaminyltransferase 14 (GalNAc-T14)*	*GALNT14*	10.89	7.20	8.46	2.03E-06	1.27E-04
8106827	84059	*G protein-coupled receptor 98*	*GPR98*	5.48	5.58	8.30	2.01E-06	1.27E-04
8152812	157638	*family with sequence similarity 84, member B*	*FAM84B*	6.11	6.30	8.12	2.02E-06	1.27E-04
8121911	387103	*centromere protein W*	*CENPW*	5.30	5.43	8.26	2.02E-06	1.27E-04
7953812	1911, 4074	*polyhomeotic homolog 1 (Drosophila) | mannose-6-phosphate receptor (cation dependent)*	*PHC1|M6 PR*	9.39	9.16	12.24	2.02E-06	1.27E-04
7965224				4.74	4.80	6.31	2.04E-06	1.27E-04
8163257	1902	*lysophosphatidic acid receptor 1*	*LPAR1*	10.90	10.10	6.78	2.10E-06	1.30E-04
8134869	5118	*procollagen C-endopeptidase enhancer*	*PCOLCE*	13.60	13.38	10.78	2.11E-06	1.30E-04
7985873	90381	*chromosome 15 open reading frame 42*	*C15orf42*	6.96	7.63	10.50	2.10E-06	1.30E-04

7922008	117143	*transcriptional adaptor 1*	*TADA1*	7.56	7.56	9.45	2.11E-06	1.30E-04
8145865	25960, 55290	*G protein-coupled receptor 124 | BRF2, subunit of RNA polymerase III transcription initiation factor, BRF1-like*	*GPR124|BRF2*	11.77	11.70	7.42	2.09E-06	1.30E-04
8146285	138050	*heparan-alpha-glucosaminide N-acetyltransferase*	*HGSNAT*	11.28	11.05	7.52	2.12E-06	1.30E-04
8047223	3336	*heat shock 10kDa protein 1 (chaperonin 10)*	*HSPE1*	7.34	7.64	9.79	2.12E-06	1.30E-04
7942832	220042	*chromosome 11 open reading frame 82*	*C11orf82*	5.64	6.11	10.21	2.14E-06	1.31E-04
8001818	7084	*thymidine kinase 2, mitochondrial*	*TK2*	9.61	9.61	7.00	2.14E-06	1.31E-04
7956120	2065	*v-erb-b2 erythroblastic leukemia viral oncogene homolog 3 (avian)*	*ERBB3*	6.50	6.37	8.85	2.18E-06	1.33E-04
8106761				8.39	6.29	5.87	2.20E-06	1.34E-04
8072626	7078	*TIMP metallopeptidase inhibitor 3*	*TIMP3*	12.19	13.20	8.66	2.23E-06	1.34E-04
8172471	11040	*pim-2 oncogene*	*PIM2*	7.45	7.39	12.08	2.22E-06	1.34E-04
7989985	22801	*integrin, alpha 11*	*ITGA11*	10.16	13.05	6.20	2.22E-06	1.34E-04
8093916	23324	*mannosidase, alpha, class 2B, member 2*	*MAN2B2*	10.44	10.15	7.19	2.23E-06	1.34E-04
8103932	79682	*MLF1 interacting protein*	*MLF1IP*	6.34	6.82	10.56	2.23E-06	1.34E-04
8097011				5.48	5.63	6.88	2.23E-06	1.34E-04
7978956	79944	*L-2-hydroxyglutarate dehydrogenase*	*L2HGDH*	6.54	6.46	8.51	2.24E-06	1.34E-04
7973352	26020	*low density lipoprotein receptor-related protein 10*	*LRP10*	12.26	12.12	8.14	2.25E-06	1.35E-04
8020377	729863, 100505852	*putative zinc finger protein ENSP00000328166-like | putative zinc finger protein 834-like*	*LOC729863|LOC100505852*	4.99	4.95	7.28	2.26E-06	1.35E-04
8087485	7318	*ubiquitin-like modifier activating enzyme 7*	*UBA7*	10.33	9.47	5.91	2.30E-06	1.37E-04
7906079	57111		*RAB25*	5.64	5.74	8.57	2.30E-06	1.37E-04
8126259	221443	*chromosome 6 open reading frame 130*	*C6orf130*	6.52	6.62	8.70	2.29E-06	1.37E-04
7963869	1911, 4074	*polyhomeotic homolog 1 (Drosophila) | mannose-6-phosphate receptor (cation dependent)*	*PHC1|M6PR*	9.30	9.07	12.15	2.31E-06	1.37E-04
8045637	3800	*kinesin family member 5C*	*KIF5C*	6.19	6.24	9.83	2.37E-06	1.40E-04
8008530	51096		*UTP18*	7.75	8.00	10.16	2.38E-06	1.40E-04
8000480				9.68	7.21	6.18	2.37E-06	1.40E-04
8106931	84250	*ankyrin repeat domain 32*	*ANKRD32*	5.50	5.50	7.85	2.40E-06	1.41E-04

8083887	5010	*claudin 11*	*CLDN11*	12.41	12.23	8.07	2.41E-06	1.41E-04
8086517	64866	*CUB domain containing protein 1*	*CDCP1*	6.95	7.29	9.13	2.43E-06	1.41E-04
8096663	493856, 150159	*CDGSH iron sulfur domain 2 | Na+/H+ exchanger domain containing 1*	*CISD2|NHEDC1*	9.51	10.11	11.00	2.42E-06	1.41E-04
7988444	50804, 283652	*myelin expression factor 2 | solute carrier family 24, member 5*	*MYEF2|SLC24A5*	5.89	5.74	9.86	2.42E-06	1.41E-04
8081686	91653	*Boc homolog (mouse)*	*BOC*	10.42	9.54	6.37	2.47E-06	1.43E-04
8046804	129401	*nucleoporin 35kDa*	*NUP35*	7.03	7.06	9.51	2.46E-06	1.43E-04
8116272	9509	*ADAM metallopeptidase with thrombospondin type 1 motif, 2*	*ADAMTS2*	10.94	11.28	7.71	2.50E-06	1.45E-04
7993167	80063	*activating transcription factor 7 interacting protein 2*	*ATF7IP2*	5.25	5.05	7.60	2.52E-06	1.46E-04
8162236	10507	*sema domain, immunoglobulin domain (Ig), transmembrane domain (TM) and short cytoplasmic domain, (semaphorin) 4D*	*SEMA4D*	8.33	6.53	9.25	2.54E-06	1.47E-04
7910997	9156	*exonuclease 1*	*EXO1*	6.55	6.50	9.80	2.55E-06	1.47E-04
7915926	6491	*SCL/TAL1 interrupting locus*	*STIL*	6.60	6.81	9.66	2.56E-06	1.47E-04
8094372	6649	*superoxide dismutase 3, extracellular*	*SOD3*	11.97	9.46	7.57	2.57E-06	1.47E-04
8155734	9413	*family with sequence similarity 189, member A2*	*FAM189A2*	6.13	6.33	8.64	2.57E-06	1.47E-04
8145055	649	*bone morphogenetic protein 1*	*BMP1*	10.17	10.11	7.29	2.61E-06	1.49E-04
8029056	641649	*transmembrane protein 91*	*TMEM91*	9.40	8.76	7.94	2.63E-06	1.50E-04
7963721	57658	*calcium binding and coiled-coil domain 1*	*CALCOCO1*	10.75	10.55	7.91	2.64E-06	1.50E-04
8060134	547	*kinesin family member 1A*	*KIF1A*	6.77	6.88	10.03	2.66E-06	1.51E-04
8042211	10678	*UDP-GlcNAc:betaGal beta-1,3-N-acetylglucosaminyltransferase 2*	*B3GNT2*	6.61	6.73	9.42	2.67E-06	1.52E-04
7973797	1690	*coagulation factor C homolog, cochlin (Limulus polyphemus)*	*COCH*	6.65	6.52	8.95	2.75E-06	1.56E-04
8138728	3201	*homeobox A4*	*HOXA4*	5.81	7.62	5.58	2.78E-06	1.57E-04
8107722	84466	*multiple EGF-like-domains 10*	*MEGF10*	6.01	5.99	8.55	2.80E-06	1.58E-04
8107823	171019	*ADAM metallopeptidase with thrombospondin type 1 motif, 19*	*ADAMTS19*	5.60	5.74	8.10	2.80E-06	1.58E-04
8154163	10171	*RNA terminal phosphate cyclase-like 1*	*RCL1*	7.61	7.85	9.60	2.85E-06	1.60E-04

7927915	219736	*storkhead box 1*	*STOX1*	4.88	4.86	6.46	2.86E-06	1.60E-04
8176230	116442		*RAB39B*	6.16	6.26	7.15	2.86E-06	1.60E-04
7909782	51018	*ribosomal RNA processing 15 homolog (S. cerevisiae)*	*RRP15*	6.52	6.69	8.43	2.87E-06	1.60E-04
8001782	6233	*ribosomal protein S27a*	*RPS27A*	9.49	9.69	10.55	2.89E-06	1.61E-04
8053901	55654	*transmembrane protein 127*	*TMEM127*	11.67	11.58	9.78	2.90E-06	1.61E-04
7955858	3226	*homeobox C10*	*HOXC10*	6.33	10.20	5.89	2.92E-06	1.62E-04
8113709	4015	*lysyl oxidase*	*LOX*	12.58	12.70	6.48	2.95E-06	1.63E-04
8149485	9108	*myotubularin related protein 7*	*MTMR7*	5.88	5.78	8.23	2.95E-06	1.63E-04
7969243	26586	*cytoskeleton associated protein 2*	*CKAP2*	7.99	8.20	10.65	2.96E-06	1.63E-04
7948420	2171, 220832	*fatty acid binding protein 5 (psoriasis-associated) | fatty acid binding protein 5 pseudogene 3*	*FABP5|FABP5P3*	5.96	5.91	10.14	2.96E-06	1.63E-04
8143575	2041	*EPH receptor A1*	*EPHA1*	6.95	6.95	9.90	2.97E-06	1.63E-04
7988414	2628	*glycine amidinotransferase (L-arginine:glycine amidinotransferase)*	*GATM*	5.68	5.69	9.29	2.97E-06	1.63E-04
7955663	23371	*tensin like C1 domain containing phosphatase (tensin 2)*	*TENC1*	10.91	10.61	6.81	2.99E-06	1.63E-04
8148263	55039	*tRNA methyltransferase 12 homolog (S. cerevisiae)*	*TRMT12*	7.00	7.06	8.40	2.99E-06	1.63E-04
8000323	4706	*NADH dehydrogenase (ubiquinone) 1, alpha/beta subcomplex, 1, 8kDa*	*NDUFAB1*	8.23	8.42	9.79	3.02E-06	1.64E-04
8120961	112609	*melanocortin 2 receptor accessory protein 2*	*MRAP2*	5.76	6.53	9.46	3.02E-06	1.64E-04
8151367				5.34	5.14	6.88	3.03E-06	1.64E-04
7966321	51184	*GPN-loop GTPase 3*	*GPN3*	7.58	8.00	10.12	3.03E-06	1.65E-04
8160531	203228	*chromosome 9 open reading frame 72*	*C9orf72*	5.79	5.91	8.87	3.04E-06	1.65E-04
7984771	4016	*lysyl oxidase-like 1*	*LOXL1*	11.71	12.17	9.07	3.05E-06	1.65E-04
8103859	1635	*dCMP deaminase*	*DCTD*	11.94	11.77	10.72	3.06E-06	1.65E-04
7922130	1805	*dermatopontin*	*DPT*	8.62	11.63	6.04	3.06E-06	1.65E-04
7979357	5015	*orthodenticle homeobox 2*	*OTX2*	7.05	7.17	11.23	3.07E-06	1.65E-04
8175647	83692	*CD99 molecule-like 2*	*CD99L2*	10.39	10.31	7.61	3.09E-06	1.66E-04
8028200	163081	*zinc finger protein 567*	*ZNF567*	5.86	5.79	7.25	3.09E-06	1.66E-04
8089372	57650		*KIAA1524*	6.56	6.71	9.83	3.11E-06	1.66E-04

7903032	22823	*metal response element binding transcription factor 2*	*MTF2*	6.92	6.99	11.13	3.12E-06	1.67E-04
7949916	1119	*choline kinase alpha*	*CHKA*	8.12	8.00	10.55	3.13E-06	1.67E-04
7918716	148281	*synaptotagmin VI*	*SYT6*	6.41	6.31	9.28	3.15E-06	1.67E-04
8029465	602	*B-cell CLL/lymphoma 3*	*BCL3*	11.48	11.34	7.74	3.17E-06	1.68E-04
8177635	6880		*TAF9*	7.66	7.72	8.98	3.17E-06	1.68E-04
8080226	25981	*dynein, axonemal, heavy chain 1*	*DNAH1*	7.65	7.43	6.41	3.16E-06	1.68E-04
7916898	55635	*DEP domain containing 1*	*DEPDC1*	5.55	5.85	8.88	3.19E-06	1.68E-04
7938890	10196	*protein arginine methyltransferase 3*	*PRMT3*	6.64	6.62	8.80	3.23E-06	1.70E-04
7992967	114990	*vasorin*	*VASN*	11.48	11.13	7.83	3.23E-06	1.70E-04
7981949	100033413	*small nucleolar RNA, C/D box 116-1*	*SNORD116-1*	9.89	10.14	11.63	3.23E-06	1.70E-04
8127854	4199	*malic enzyme 1, NADP(+)-dependent, cytosolic*	*ME1*	9.82	8.93	8.27	3.30E-06	1.73E-04
8151240	63978	*PR domain containing 14*	*PRDM14*	6.51	6.67	10.75	3.30E-06	1.73E-04
8073775	2192	*fibulin 1*	*FBLN1*	12.52	11.84	9.49	3.34E-06	1.74E-04
8136388	55281	*transmembrane protein 140*	*TMEM140*	8.63	8.06	6.17	3.33E-06	1.74E-04
8146579	55636	*chromodomain helicase DNA binding protein 7*	*CHD7*	5.65	5.88	8.79	3.34E-06	1.74E-04
8093256				10.08	10.17	11.00	3.35E-06	1.74E-04
7932069	11164	*nudix (nucleoside diphosphate linked moiety X)-type motif 5*	*NUDT5*	9.27	9.20	10.76	3.38E-06	1.74E-04
8035838	440519	*zinc finger protein 724, pseudogene*	*ZNF724P*	5.71	6.13	10.86	3.37E-06	1.74E-04
8122182	9519, 154091	*TBP-like 1 | solute carrier family 2 (facilitated glucose transporter), member 12*	*TBPL1|SLC2A12*	8.62	8.38	10.25	3.37E-06	1.74E-04
8112458	6880		*TAF9*	8.06	8.12	9.42	3.39E-06	1.75E-04
8089875	10721	*polymerase (DNA directed), theta*	*POLQ*	5.59	5.61	7.79	3.40E-06	1.75E-04
7917276	23566	*lysophosphatidic acid receptor 3*	*LPAR3*	6.65	6.29	9.32	3.41E-06	1.75E-04
7982597	7057	*thrombospondin 1*	*THBS1*	13.63	13.83	10.14	3.42E-06	1.75E-04
8100015	389206	*BEN domain containing 4*	*BEND4*	6.67	6.71	9.34	3.43E-06	1.75E-04
8083839	26996	*G protein-coupled receptor 160*	*GPR160*	4.83	4.83	7.47	3.46E-06	1.77E-04
7976239				7.09	7.18	7.85	3.52E-06	1.80E-04
7919382				5.57	5.74	7.39	3.53E-06	1.80E-04

8166525	5422	*polymerase (DNA directed), alpha 1, catalytic subunit*	*POLA1*	6.24	6.32	9.10	3.54E-06	1.80E-04
7948898	9298, 23642	*small nucleolar RNA, C/D box 31 | small nucleolar RNA host gene 1 (non-protein coding)*	*SNORD31| SNHG1*	6.86	6.96	9.98	3.54E-06	1.80E-04
8150138	56154	*testis expressed 15*	*TEX15*	5.57	5.48	8.32	3.55E-06	1.80E-04
7937508	977		*CD151*	13.20	13.12	11.20	3.57E-06	1.80E-04
7902448	6139	*ribosomal protein L17*	*RPL17*	11.49	11.64	12.51	3.57E-06	1.80E-04
8060835				4.48	4.44	5.77	3.57E-06	1.80E-04
7971077	10631	*periostin, osteoblast specific factor*	*POSTN*	11.49	10.91	5.81	3.59E-06	1.80E-04
8068810	54020	*solute carrier family 37 (glycerol-3-phosphate transporter), member 1*	*SLC37A1*	7.24	6.47	8.69	3.61E-06	1.81E-04
8129763	113115	*family with sequence similarity 54, member A*	*FAM54A*	5.85	5.95	8.43	3.62E-06	1.81E-04
8047187	284992	*coiled-coil domain containing 150*	*CCDC150*	5.44	5.45	7.12	3.63E-06	1.82E-04
7981990	100033432, 692236	*small nucleolar RNA, C/D box 116-21 | small nucleolar RNA, C/D box 116 cluster*	*SNORD11 6-21|SNORD 116@*	8.22	8.02	12.13	3.65E-06	1.82E-04
7902527	5737	*prostaglandin F receptor (FP)*	*PTGFR*	8.51	8.42	5.89	3.66E-06	1.82E-04
8030362	26818, 23521	*small nucleolar RNA, C/D box 33 | ribosomal protein L13a*	*SNORD33| RPL13A*	13.22	13.36	12.93	3.68E-06	1.83E-04
8083673	654502, 100505385, 29970	*IQ motif containing J | IQ motif containing J-schwannomin interacting protein 1 read-through transcript | schwannomin interacting protein 1*	*IQCJ|IQCJ -SCHIP1*	5.79	5.78	5.59	3.77E-06	1.87E-04
8114425	995	*cell division cycle 25 homolog C (S. pombe)*	*CDC25C*	6.26	6.63	9.08	3.80E-06	1.88E-04
8094278	64151, 254251	*non-SMC condensin I complex, subunit G | ligand dependent nuclear receptor corepressor-like*	*NCAPG|L CORL*	6.68	6.67	9.79	3.79E-06	1.88E-04
7973974	5083	*paired box 9*	*PAX9*	12.01	6.67	6.24	3.83E-06	1.89E-04
8043902	79031	*phosducin-like 3*	*PDCL3*	6.70	7.14	8.57	3.83E-06	1.89E-04
8075462	140606	*selenoprotein M*	*SELM*	12.40	12.32	9.03	3.84E-06	1.89E-04
8150592	1052	*CCAAT/enhancer binding protein (C/EBP), delta*	*CEBPD*	11.65	11.72	8.74	3.85E-06	1.89E-04
8095303	23284	*latrophilin 3*	*LPHN3*	5.30	5.39	8.32	3.85E-06	1.89E-04
7933772	288	*ankyrin 3, node of Ranvier (ankyrin G)*	*ANK3*	5.84	5.70	8.54	3.86E-06	1.89E-04
8148973	157695	*chromosome 8 open reading frame 42*	*C8orf42*	7.23	7.11	9.50	3.87E-06	1.89E-04

8123609	5272	*serpin peptidase inhibitor, clade B (ovalbumin), member 9*	*SERPINB9*	8.54	6.83	11.92	3.91E-06	1.90E-04
7910923	56776	*formin 2*	*FMN2*	9.71	9.21	6.16	3.91E-06	1.90E-04
8068952	56894	*1-acylglycerol-3-phosphate O-acyltransferase 3*	*AGPAT3*	9.43	9.36	7.47	3.90E-06	1.90E-04
8150002	157574, 55893	*F-box protein 16 | zinc finger protein 395*	*FBXO16|ZNF395*	5.73	5.56	8.46	3.89E-06	1.90E-04
7933149	221002	*RasGEF domain family, member 1A*	*RASGEF1A*	7.10	7.33	9.25	3.92E-06	1.90E-04
8107706	4001	*lamin B1*	*LMNB1*	6.42	6.63	9.52	3.99E-06	1.93E-04
8134351	55607	*protein phosphatase 1, regulatory (inhibitor) subunit 9A*	*PPP1R9A*	5.67	6.02	7.84	4.03E-06	1.94E-04
8157246	158405		*KIAA1958*	6.62	7.10	8.88	4.02E-06	1.94E-04
8113369	353189	*solute carrier organic anion transporter family, member 4C1*	*SLCO4C1*	5.16	5.24	8.12	4.05E-06	1.95E-04
7920057	11022	*tudor and KH domain containing*	*TDRKH*	6.17	6.23	8.42	4.09E-06	1.96E-04
8140967	54809	*sterile alpha motif domain containing 9*	*SAMD9*	8.77	8.43	5.02	4.10E-06	1.96E-04
7975989	81892	*chromosome 14 open reading frame 156*	*C14orf156*	11.89	12.12	13.38	4.08E-06	1.96E-04
8081779	254887	*zinc finger, DHHC-type containing 23*	*ZDHHC23*	6.84	6.88	8.55	4.10E-06	1.96E-04
8160033	6635	*small nuclear ribonucleoprotein polypeptide E*	*SNRPE*	8.84	9.18	10.72	4.12E-06	1.97E-04
7936777	54780	*non-SMC element 4 homolog A (S. cerevisiae)*	*NSMCE4A*	8.30	8.36	10.40	4.15E-06	1.98E-04
7953835	10219	*killer cell lectin-like receptor subfamily G, member 1*	*KLRG1*	5.72	5.73	7.28	4.17E-06	1.99E-04
7911529	54587	*matrix-remodelling associated 8*	*MXRA8*	13.34	13.31	9.95	4.19E-06	1.99E-04
7947590	57586	*synaptotagmin XIII*	*SYT13*	5.93	6.06	7.21	4.19E-06	1.99E-04
8031807	90233	*zinc finger protein 551*	*ZNF551*	6.35	6.35	8.68	4.18E-06	1.99E-04
7909730	3776	*potassium channel, subfamily K, member 2*	*KCNK2*	9.46	11.92	6.04	4.20E-06	1.99E-04
8120880	7162	*trophoblast glycoprotein*	*TPBG*	11.27	11.26	9.52	4.22E-06	1.99E-04
8162880	54534	*mitochondrial ribosomal protein L50*	*MRPL50*	9.36	9.67	11.25	4.24E-06	2.00E-04
7959893	283383	*G protein-coupled receptor 133*	*GPR133*	11.78	10.75	6.29	4.25E-06	2.00E-04
8179034	3135	*major histocompatibility complex, class I, G*	*HLA-G*	12.37	12.27	10.37	4.26E-06	2.00E-04
8069933	54069	*chromosome 21 open reading frame 45*	*C21orf45*	6.64	6.99	10.20	4.29E-06	2.01E-04

7950683	79731	*asparaginyl-tRNA synthetase 2, mitochondrial (putative)*	*NARS2*	8.28	8.35	11.06	4.29E-06	2.01E-04
7982000	100033438	*small nucleolar RNA, C/D box 116-26*	*SNORD116-26*	7.75	8.05	10.48	4.29E-06	2.01E-04
7932453	10529	*nebulette*	*NEBL*	6.21	6.18	8.28	4.31E-06	2.01E-04
7957052	10576	*chaperonin containing TCP1, subunit 2 (beta)*	*CCT2*	8.84	9.23	11.06	4.33E-06	2.01E-04
8092839	131578	*leucine rich repeat containing 15*	*LRRC15*	10.77	12.02	7.51	4.32E-06	2.01E-04
8044278	165055	*coiled-coil domain containing 138*	*CCDC138*	5.67	5.80	8.31	4.34E-06	2.01E-04
7985829	55215, 5428	*Fanconi anemia, complementation group I | polymerase (DNA directed), gamma*	*FANCI|POLG*	6.18	6.24	9.96	4.32E-06	2.01E-04
8177725	3135	*major histocompatibility complex, class I, G*	*HLA-G*	12.37	12.27	10.37	4.37E-06	2.02E-04
7959408	9735	*kinetochore associated 1*	*KNTC1*	6.37	6.62	9.51	4.36E-06	2.02E-04
7970513	221150, 78988	*spindle and kinetochore associated complex subunit 3 | mitochondrial ribosomal protein 63*	*SKA3|MRP63*	7.62	7.79	11.15	4.38E-06	2.03E-04
8142524	23554	*tetraspanin 12*	*TSPAN12*	5.94	5.94	8.20	4.40E-06	2.03E-04
7963786	3678	*integrin, alpha 5 (fibronectin receptor, alpha polypeptide)*	*ITGA5*	12.04	12.58	9.02	4.44E-06	2.05E-04
8051528	10153	*CCAAT/enhancer binding protein (C/EBP), zeta*	*CEBPZ*	6.82	6.78	9.23	4.45E-06	2.05E-04
8121489	262	*adenosylmethionine decarboxylase 1*	*AMD1*	8.16	8.01	10.92	4.48E-06	2.05E-04
8112376	64105	*centromere protein K*	*CENPK*	6.49	6.92	10.18	4.47E-06	2.05E-04
8080911	84541	*kelch repeat and BTB (POZ) domain containing 8*	*KBTBD8*	5.89	5.77	8.23	4.49E-06	2.05E-04
7996377	283848	*carboxylesterase 4A*	*CES4A*	8.10	7.49	6.49	4.48E-06	2.05E-04
7967870				6.55	6.23	9.89	4.53E-06	2.06E-04
7902425	256435	*ST6 (alpha-N-acetyl-neuraminyl-2,3-beta-galactosyl-1,3)-N-acetylgalactosaminide alpha-2,6-sialyltransferase 3*	*ST6GALNAC3*	6.51	6.14	8.49	4.53E-06	2.06E-04
7916727	23421	*integrin beta 3 binding protein (beta3-endonexin)*	*ITGB3BP*	7.04	7.46	9.36	4.55E-06	2.07E-04
7966690	6926	*T-box 3*	*TBX3*	10.85	9.71	6.69	4.57E-06	2.07E-04
8052004	285051	*chromosome 2 open reading frame 61*	*C2orf61*	6.18	6.23	5.53	4.56E-06	2.07E-04
7986214	28232	*solute carrier organic anion transporter family, member 3A1*	*SLCO3A1*	10.87	9.30	9.42	4.58E-06	2.07E-04
7982889	51203	*nucleolar and spindle associated protein 1*	*NUSAP1*	7.34	7.58	11.01	4.60E-06	2.08E-04

8015769	672	*breast cancer 1, early onset*	*BRCA1*	5.93	5.97	8.54	4.61E-06	2.08E-04
8055426	4175	*minichromosome maintenance complex component 6*	*MCM6*	6.92	7.14	10.55	4.63E-06	2.09E-04
8166355	22866	*connector enhancer of kinase suppressor of Ras 2*	*CNKSR2*	6.70	6.77	9.12	4.64E-06	2.09E-04
7955562	94	*activin A receptor type II-like 1*	*ACVRL1*	9.12	8.98	6.75	4.67E-06	2.09E-04
8100834	2926	*G-rich RNA sequence binding factor 1*	*GRSF1*	9.01	8.94	10.10	4.67E-06	2.09E-04
8055711	4703	*nebulin*	*NEB*	5.80	5.83	6.99	4.67E-06	2.09E-04
8079074	51188	*synovial sarcoma translocation gene on chromosome 18-like 2*	*SS18L2*	8.60	8.74	9.84	4.67E-06	2.09E-04
7923043	343450	*potassium channel, subfamily T, member 2*	*KCNT2*	5.70	5.81	8.62	4.66E-06	2.09E-04
8111772	1601	*disabled homolog 2, mitogen-responsive phosphoprotein (Drosophila)*	*DAB2*	11.97	12.71	8.35	4.70E-06	2.09E-04
8150419	79698	*zinc finger, matrin-type 4*	*ZMAT4*	5.93	6.43	9.94	4.69E-06	2.09E-04
7926319	79723	*suppressor of variegation 3-9 homolog 2 (Drosophila)*	*SUV39H2*	7.18	7.33	10.51	4.77E-06	2.12E-04
8138108	11014	*KDEL (Lys-Asp-Glu-Leu) endoplasmic reticulum protein retention receptor 2*	*KDELR2*	11.90	12.18	11.06	4.79E-06	2.12E-04
8122334	51554	*chemokine (C-C motif) receptor-like 1*	*CCRL1*	11.53	11.25	5.86	4.85E-06	2.15E-04
8131927	51678	*membrane protein, palmitoylated 6 (MAGUK p55 subfamily member 6)*	*MPP6*	7.54	7.67	9.54	4.86E-06	2.15E-04
7993588	79905	*transmembrane channel-like 7*	*TMC7*	6.03	6.53	8.29	4.88E-06	2.15E-04
7957126	27345	*potassium large conductance calcium-activated channel, subfamily M, beta member 4*	*KCNMB4*	6.65	7.25	9.67	4.89E-06	2.16E-04
8096081	58478	*enolase-phosphatase 1*	*ENOPH1*	7.71	8.09	10.28	4.91E-06	2.16E-04
7908988	6635	*small nuclear ribonucleoprotein polypeptide E*	*SNRPE*	5.27	5.36	6.25	4.95E-06	2.17E-04
7960518	7132	*tumor necrosis factor receptor superfamily, member 1A*	*TNFRSF1 A*	11.93	11.62	9.23	4.95E-06	2.17E-04
8023575	147372	*collagen and calcium binding EGF domains 1*	*CCBE1*	8.78	9.28	6.12	4.96E-06	2.17E-04
8106999	202299	*chromosome 5 open reading frame 27*	*C5orf27*	6.07	6.24	5.66	4.97E-06	2.17E-04
7968484	675	*breast cancer 2, early onset*	*BRCA2*	5.97	6.14	8.87	5.01E-06	2.18E-04
7981960	100033418	*small nucleolar RNA, C/D box 116-6*	*SNORD11 6-6*	8.32	8.42	10.60	5.00E-06	2.18E-04
7956908				7.34	6.97	8.90	5.00E-06	2.18E-04
8009476	5608	*mitogen-activated protein kinase kinase 6*	*MAP2K6*	6.27	6.45	8.46	5.05E-06	2.19E-04

8159142	1289	*collagen, type V, alpha 1*	*COL5A1*	10.22	10.51	6.92	5.05E-06	2.19E-04
8178498	3106, 3107	*major histocompatibility complex, class I, B | major histocompatibility complex, class I, C*	*HLA-B|HLA-C*	13.10	12.91	10.73	5.06E-06	2.20E-04
7975361	9766		*KIAA0247*	9.35	9.16	7.23	5.08E-06	2.20E-04
8053248	130951	*chromosome 2 open reading frame 65*	*C2orf65*	6.76	7.02	8.02	5.12E-06	2.21E-04
7924096	4751	*NIMA (never in mitosis gene a)-related kinase 2*	*NEK2*	6.67	6.94	9.44	5.14E-06	2.21E-04
8043602	23397	*non-SMC condensin I complex, subunit H*	*NCAPH*	6.98	7.53	10.29	5.13E-06	2.21E-04
8073612	706	*translocator protein (18kDa)*	*TSPO*	11.71	11.70	7.97	5.21E-06	2.24E-04
7945245	50863	*neurotrimin*	*NTM*	9.98	9.75	7.90	5.20E-06	2.24E-04
8097857	84057	*meiotic nuclear divisions 1 homolog (S. cerevisiae)*	*MND1*	5.70	5.99	8.90	5.23E-06	2.24E-04
8101893	126	*alcohol dehydrogenase 1C (class I), gamma polypeptide*	*ADH1C*	7.62	6.37	5.45	5.24E-06	2.24E-04
8104901	3575	*interleukin 7 receptor*	*IL7R*	7.59	8.89	5.20	5.26E-06	2.25E-04
8169061	5354	*proteolipid protein 1*	*PLP1*	6.40	6.36	10.28	5.26E-06	2.25E-04
8005839	27346	*transmembrane protein 97*	*TMEM97*	10.60	8.83	11.61	5.28E-06	2.25E-04
8168762	1478	*cleavage stimulation factor, 3' pre-RNA, subunit 2, 64kDa*	*CSTF2*	7.45	7.74	9.15	5.32E-06	2.26E-04
7995783	4502	*metallothionein 2A*	*MT2A*	13.80	13.92	10.89	5.36E-06	2.27E-04
8030782	27180	*sialic acid binding Ig-like lectin 9*	*SIGLEC9*	6.56	6.80	6.18	5.37E-06	2.27E-04
7909877	64757	*MOCO sulphurase C-terminal domain containing 1*	*MOSC1*	6.93	6.98	9.50	5.37E-06	2.27E-04
8081645	79669	*chromosome 3 open reading frame 52*	*C3orf52*	6.27	6.28	7.57	5.38E-06	2.27E-04
8111960	375444	*chromosome 5 open reading frame 34*	*C5orf34*	5.62	5.89	7.98	5.37E-06	2.27E-04
7925250	2786	*guanine nucleotide binding protein (G protein), gamma 4*	*GNG4*	7.03	6.92	9.92	5.39E-06	2.27E-04
7979281	11169	*WD repeat and HMG-box DNA binding protein 1*	*WDHD1*	6.30	6.55	10.67	5.40E-06	2.28E-04
7976350	57578		*KIAA1409*	5.72	5.89	6.96	5.41E-06	2.28E-04
8101324	3184	*heterogeneous nuclear ribonucleoprotein D (AU-rich element RNA binding protein 1, 37kDa)*	*HNRNPD*	10.78	10.85	12.73	5.43E-06	2.28E-04
7951284	4314	*matrix metallopeptidase 3 (stromelysin 1, progelatinase)*	*MMP3*	7.64	10.69	5.59	5.44E-06	2.28E-04
7926728	53904	*myosin IIIA*	*MYO3A*	5.23	5.29	6.78	5.47E-06	2.29E-04

8129963	134637	*adenosine deaminase, tRNA-specific 2, TAD2 homolog (S. cerevisiae)*	*ADAT2*	6.58	6.51	9.06	5.50E-06	2.30E-04
8077970	2199	*fibulin 2*	*FBLN2*	11.32	11.96	7.66	5.52E-06	2.30E-04
8068833	5152	*phosphodiesterase 9A*	*PDE9A*	7.60	7.48	9.19	5.51E-06	2.30E-04
8005512	5636	*phosphoribosyl pyrophosphate synthetase-associated protein 2*	*PRPSAP2*	7.30	7.52	9.69	5.53E-06	2.30E-04
7934916	9023	*cholesterol 25-hydroxylase*	*CH25H*	7.03	10.88	7.33	5.53E-06	2.30E-04
7999553	55313	*calcineurin-like phosphoesterase domain containing 1*	*CPPED1*	9.53	9.62	8.31	5.54E-06	2.30E-04
8173600	4674	*nucleosome assembly protein 1-like 2*	*NAP1L2*	6.08	5.66	7.46	5.55E-06	2.30E-04
8062119	140851	*metallothionein 1 pseudogene 3*	*MT1P3*	9.16	9.15	6.86	5.56E-06	2.30E-04
8146357	4173	*minichromosome maintenance complex component 4*	*MCM4*	7.76	8.26	11.00	5.59E-06	2.31E-04
8078380	344787	*zinc finger protein 860*	*ZNF860*	5.60	5.39	8.46	5.61E-06	2.32E-04
8147101	1875	*E2F transcription factor 5, p130-binding*	*E2F5*	7.23	7.48	10.91	5.64E-06	2.32E-04
8034974	79852	*epoxide hydrolase 3*	*EPHX3*	6.57	6.75	9.94	5.73E-06	2.36E-04
8124911	3106, 3107	*major histocompatibility complex, class I, B | major histocompatibility complex, class I, C*	*HLA-B|HLA-C*	13.09	12.90	10.67	5.74E-06	2.36E-04
8007154	60681	*FK506 binding protein 10, 65 kDa*	*FKBP10*	12.89	12.86	11.21	5.79E-06	2.38E-04
8097356	10733	*polo-like kinase 4*	*PLK4*	6.01	6.29	10.13	5.81E-06	2.38E-04
8035782	91120	*zinc finger protein 682*	*ZNF682*	6.16	5.92	9.26	5.84E-06	2.39E-04
8096704	255743	*nephronectin*	*NPNT*	6.07	6.18	6.81	5.85E-06	2.39E-04
7958031	55010	*chromosome 12 open reading frame 48*	*C12orf48*	5.91	5.97	8.48	5.87E-06	2.39E-04
8011884	22861, 728392	*NLR family, pyrin domain containing 1 | hypothetical protein LOC728392*	*NLRP1|LOC728392*	8.54	8.94	6.94	5.87E-06	2.39E-04
8104625				5.70	5.73	8.19	5.87E-06	2.39E-04
8007799	401884, 1394	*hypothetical LOC401884 | corticotropin releasing hormone receptor 1*	*MGC57346|CRHR1*	10.10	9.47	11.37	5.90E-06	2.40E-04
8047243	92935	*methionyl-tRNA synthetase 2, mitochondrial*	*MARS2*	7.16	7.39	8.72	5.91E-06	2.40E-04
7964271	5557	*primase, DNA, polypeptide 1 (49kDa)*	*PRIM1*	7.18	7.53	11.01	5.92E-06	2.40E-04
7915861	148932, 8569	*MOB1, Mps One Binder kinase activator-like 2C (yeast) | MAP kinase interacting serine/threonine kinase 1*	*MOBKL2C|MKNK1*	7.82	7.65	6.62	5.93E-06	2.40E-04

8151788	389677	*RNA binding motif protein 12B*	*RBM12B*	7.27	6.91	9.24	5.95E-06	2.41E-04
8163637	3371	*tenascin C*	*TNC*	9.17	12.85	7.75	6.02E-06	2.42E-04
8100495	5471	*phosphoribosyl pyrophosphate amidotransferase*	*PPAT*	6.56	6.65	10.65	6.03E-06	2.42E-04
8139057	9844	*engulfment and cell motility 1*	*ELMO1*	6.06	5.81	7.53	5.99E-06	2.42E-04
7901535	127435	*podocan*	*PODN*	11.62	11.25	7.06	6.01E-06	2.42E-04
8051785	170850	*potassium voltage-gated channel, subfamily G, member 3*	*KCNG3*	6.03	6.18	8.99	6.01E-06	2.42E-04
8159519	203235	*chromosome 9 open reading frame 141*	*C9orf141*	8.82	9.07	7.96	6.03E-06	2.42E-04
7933582	100287932, 653252	*translocase of inner mitochondrial membrane 23 homolog (yeast) | translocase of inner mitochondrial membrane 23 homolog B (yeast)*	*TIMM23|TIMM23B*	11.09	11.42	12.11	6.04E-06	2.42E-04
7976292	79890	*Ras and Rab interactor 3*	*RIN3*	10.21	9.85	6.92	6.05E-06	2.42E-04
8027247	81931	*zinc finger protein 93*	*ZNF93*	6.63	7.16	10.84	6.07E-06	2.42E-04
8036395	148266	*zinc finger protein 569*	*ZNF569*	6.26	6.42	7.63	6.07E-06	2.42E-04
8012896	5376	*peripheral myelin protein 22*	*PMP22*	12.38	12.19	7.97	6.10E-06	2.42E-04
8005132	4213, 56917, 257468	*Meis homeobox 3 pseudogene 1 | Meis homeobox 3 pseudogene 2*	*MEIS3P1|MEIS3P2*	10.25	10.11	8.23	6.09E-06	2.42E-04
7899192	6195	*ribosomal protein S6 kinase, 90kDa, polypeptide 1*	*RPS6KA1*	7.09	7.32	9.54	6.11E-06	2.43E-04
8013671	10615, 124923	*sperm associated antigen 5 | uncharacterized serine/threonine-protein kinase SgK494*	*SPAG5|SGK494*	6.69	7.23	10.17	6.15E-06	2.44E-04
7940028	710	*serpin peptidase inhibitor, clade G (C1 inhibitor), member 1*	*SERPING1*	12.88	12.45	9.87	6.19E-06	2.45E-04
8028652	7538	*zinc finger protein 36, C3H type, homolog (mouse)*	*ZFP36*	10.29	10.31	7.55	6.26E-06	2.47E-04
7947512	25891	*peptidase domain containing associated with muscle regeneration 1*	*PAMR1*	11.46	11.30	7.10	6.27E-06	2.47E-04
8102342	79071	*ELOVL family member 6, elongation of long chain fatty acids (FEN1/Elo2, SUR4/Elo3-like, yeast)*	*ELOVL6*	6.81	6.63	10.54	6.26E-06	2.47E-04
8035789	664701	*zinc finger protein 826, pseudogene*	*ZNF826P*	6.62	6.16	10.58	6.27E-06	2.47E-04
7907183	57147, 55732	*SCY1-like 3 (S. cerevisiae) | chromosome 1 open reading frame 112*	*SCYL3|C1orf112*	6.29	6.55	8.96	6.28E-06	2.47E-04
8030383	57333	*reticulocalbin 3, EF-hand calcium binding domain*	*RCN3*	12.18	12.26	8.92	6.33E-06	2.49E-04
7936100	51063	*calcium homeostasis modulator 2*	*CALHM2*	10.96	11.09	7.34	6.34E-06	2.49E-04

8108697	26167	*protocadherin beta 5*	*PCDHB5*	7.55	7.22	10.99	6.38E-06	2.49E-04
7926821	84930	*microtubule associated serine/threonine kinase-like*	*MASTL*	7.34	7.90	10.06	6.37E-06	2.49E-04
7947189	91057	*coiled-coil domain containing 34*	*CCDC34*	7.14	7.39	9.33	6.40E-06	2.50E-04
8080028	1795	*dedicator of cytokinesis 3*	*DOCK3*	6.40	6.51	8.14	6.48E-06	2.51E-04
8138202	3382	*islet cell autoantigen 1, 69kDa*	*ICA1*	5.35	5.51	7.84	6.47E-06	2.51E-04
7931097	5654	*HtrA serine peptidase 1*	*HTRA1*	11.85	12.28	8.65	6.45E-06	2.51E-04
8061471	9837	*GINS complex subunit 1 (Psf1 homolog)*	*GINS1*	7.03	7.64	11.90	6.44E-06	2.51E-04
7995258	10308	*zinc finger protein 267*	*ZNF267*	6.37	6.38	9.11	6.47E-06	2.51E-04
8175860	57595	*PDZ domain containing 4*	*PDZD4*	6.74	6.84	9.42	6.45E-06	2.51E-04
7998921	84891	*zinc finger and SCAN domain containing 10*	*ZSCAN10*	7.20	7.41	10.54	6.47E-06	2.51E-04
7906904	51478, 158160	*hydroxysteroid (17-beta) dehydrogenase 7 | hydroxysteroid (17-beta) dehydrogenase 7 pseudogene 2*	*HSD17B7|HSD17B7P2*	5.86	5.54	8.02	6.53E-06	2.52E-04
7928534	83938	*chromosome 10 open reading frame 11*	*C10orf11*	8.34	6.76	5.91	6.58E-06	2.53E-04
8155634	441666	*zinc finger protein 91 pseudogene*	*LOC441666*	4.59	4.55	6.41	6.57E-06	2.53E-04
7965022	11103, 11010	*KRR1, small subunit (SSU) processome component, homolog (yeast) | GLI pathogenesis-related 1*	*KRR1|GLIPR1*	8.22	8.32	9.46	6.58E-06	2.53E-04
8101143	53371	*nucleoporin 54kDa*	*NUP54*	8.04	8.07	10.11	6.62E-06	2.54E-04
7978544	112399	*egl nine homolog 3 (C. elegans)*	*EGLN3*	7.13	7.15	10.46	6.61E-06	2.54E-04
8123342	11116, 1235	*FGFR1 oncogene partner | chemokine (C-C motif) receptor 6*	*FGFR1OP|CCR6*	6.57	6.58	8.37	6.63E-06	2.54E-04
8151252				5.80	5.98	7.34	6.63E-06	2.54E-04
8131600	27075	*tetraspanin 13*	*TSPAN13*	6.81	7.62	8.81	6.67E-06	2.55E-04
8107408	3781	*potassium intermediate/small conductance calcium-activated channel, subfamily N, member 2*	*KCNN2*	6.69	6.82	8.75	6.68E-06	2.55E-04
8145914	9530, 27257	*BCL2-associated athanogene 4 | LSM1 homolog, U6 small nuclear RNA associated (S. cerevisiae)*	*BAG4|LSM1*	8.60	8.30	9.77	6.71E-06	2.56E-04
7929258	3832	*kinesin family member 11*	*KIF11*	6.47	6.46	10.06	6.73E-06	2.56E-04
7917676	11146	*glomulin, FKBP associated protein*	*GLMN*	6.69	6.57	9.04	6.73E-06	2.56E-04
8122336	58527	*chromosome 6 open reading frame 115*	*C6orf115*	7.00	7.36	9.90	6.76E-06	2.56E-04

8005695	4213, 56917, 257468	*Meis homeobox 3 pseudogene 1 | Meis homeobox 3 pseudogene 2*	*MEIS3P1|MEIS3P2*	10.28	10.21	8.28	6.75E-06	2.56E-04
7979133	22795	*nidogen 2 (osteonidogen)*	*NID2*	11.76	9.70	6.77	6.78E-06	2.57E-04
7988876	55930	*myosin VC*	*MYO5C*	5.54	5.59	7.68	6.86E-06	2.60E-04
8152582	79075	*defective in sister chromatid cohesion 1 homolog (S. cerevisiae)*	*DSCC1*	6.16	6.28	9.28	6.88E-06	2.60E-04
7977075	677811	*small nucleolar RNA, H/ACA box 28*	*SNORA28*	5.85	6.16	6.83	6.88E-06	2.60E-04
8059538	80704	*solute carrier family 19, member 3*	*SLC19A3*	6.98	6.46	8.60	6.91E-06	2.60E-04
8171823	79135, 254158	*apolipoprotein O | chromosome X open reading frame 58*	*APOO|CXorf58*	9.33	9.75	11.56	6.94E-06	2.61E-04
7942879	55863	*transmembrane protein 126B*	*TMEM126B*	6.72	7.05	8.49	6.96E-06	2.61E-04
8103728	3148	*high-mobility group box 2*	*HMGB2*	6.97	7.32	9.41	6.97E-06	2.62E-04
8100026	10396	*ATPase, aminophospholipid transporter (APLT), class I, type 8A, member 1*	*ATP8A1*	5.48	5.69	7.56	6.99E-06	2.62E-04
8112007	133418	*embigin*	*EMB*	5.71	6.66	9.53	6.99E-06	2.62E-04
8135943				12.20	12.30	10.34	7.05E-06	2.64E-04
8122818	79624	*chromosome 6 open reading frame 211*	*C6orf211*	7.84	7.98	10.14	7.07E-06	2.64E-04
8059350	130340	*adaptor-related protein complex 1, sigma 3 subunit*	*AP1S3*	5.89	5.73	8.04	7.06E-06	2.64E-04
7957629				4.51	4.50	5.21	7.13E-06	2.66E-04
8009366	25926	*nucleolar protein 11*	*NOL11*	7.44	7.48	9.56	7.24E-06	2.69E-04
8171760	100158262	*small Cajal body-specific RNA 9-like (retrotransposed)*	*SCARNA9L*	5.55	6.07	6.80	7.35E-06	2.73E-04
7981943	8123, 347686	*Prader-Willi/Angelman syndrome-5 | small nucleolar RNA, C/D box 64*	*PAR5|SNORD64*	5.58	5.40	11.56	7.35E-06	2.73E-04
7975702	55237	*vertebrae development homolog (pig)*	*VRTN*	6.13	6.35	9.59	7.44E-06	2.76E-04
8034344	90589, 7568	*zinc finger protein 625 | zinc finger protein 20*	*ZNF625|ZNF20*	6.30	6.47	8.16	7.48E-06	2.77E-04
7936968	8038	*ADAM metallopeptidase domain 12*	*ADAM12*	9.70	11.35	5.96	7.50E-06	2.77E-04
8022747	9331	*UDP-Gal:betaGlcNAc beta 1,4- galactosyltransferase, polypeptide 6*	*B4GALT6*	6.57	6.72	10.22	7.51E-06	2.77E-04

7951325	84259	*DCN1, defective in cullin neddylation 1, domain containing 5 (S. cerevisiae)*	*DCUN1D5*	7.90	8.13	10.60	7.49E-06	2.77E-04
7897803	5351	*procollagen-lysine 1, 2-oxoglutarate 5-dioxygenase 1*	*PLOD1*	12.24	12.49	9.62	7.55E-06	2.78E-04
8082965	22808	*muscle RAS oncogene homolog*	*MRAS*	9.77	10.12	7.93	7.57E-06	2.78E-04
7935188	10580	*sorbin and SH3 domain containing 1*	*SORBS1*	5.59	6.04	8.52	7.59E-06	2.79E-04
8169352	55916	*nuclear transport factor 2-like export factor 2*	*NXT2*	8.80	8.88	10.33	7.64E-06	2.80E-04
8016438	3212	*homeobox B2*	*HOXB2*	6.71	9.89	6.52	7.66E-06	2.80E-04
8101675	9429	*ATP-binding cassette, sub-family G (WHITE), member 2*	*ABCG2*	5.67	5.54	7.15	7.65E-06	2.80E-04
8094165	391634	*heat shock protein 90kDa alpha (cytosolic), class B member 2 (pseudogene)*	*HSP90AB2P*	6.98	7.28	8.26	7.69E-06	2.81E-04
7903162	148534	*transmembrane protein 56*	*TMEM56*	5.71	5.62	7.64	7.74E-06	2.82E-04
8102789	7013, 646359	*telomeric repeat binding factor (NIMA-interacting) 1 | telomeric repeat binding factor (NIMA-interacting) 1 pseudogene 2*	*TERF1|TERF1P2*	8.39	7.98	11.51	7.74E-06	2.82E-04
7903582	6139	*ribosomal protein L17*	*RPL17*	11.88	12.03	12.88	7.76E-06	2.83E-04
7973414	25983	*neuroguidin, EIF4E binding protein*	*NGDN*	7.53	7.63	9.46	7.77E-06	2.83E-04
7932254	8516	*integrin, alpha 8*	*ITGA8*	10.60	7.08	5.23	7.82E-06	2.84E-04
8038407	6237	*related RAS viral (r-ras) oncogene homolog*	*RRAS*	13.21	13.10	10.90	7.87E-06	2.85E-04
7916862	79971	*wntless homolog (Drosophila)*	*WLS*	11.88	8.34	7.11	7.86E-06	2.85E-04
7938100	6609	*sphingomyelin phosphodiesterase 1, acid lysosomal*	*SMPD1*	10.76	10.42	6.72	7.88E-06	2.85E-04
8031768	284307	*zinc finger protein interacting with K protein 1 homolog (mouse)*	*ZIK1*	7.12	7.17	9.94	7.90E-06	2.85E-04
7920642	4582	*mucin 1, cell surface associated*	*MUC1*	9.09	8.99	6.58	7.93E-06	2.86E-04
7935553	84171	*lysyl oxidase-like 4*	*LOXL4*	9.42	10.05	6.72	7.93E-06	2.86E-04
7933750	220963	*solute carrier family 16, member 9 (monocarboxylic acid transporter 9)*	*SLC16A9*	5.33	5.49	8.42	7.93E-06	2.86E-04
8020814	51444	*ring finger protein 138*	*RNF138*	6.37	6.47	8.71	8.00E-06	2.87E-04
8052654	57162	*pellino homolog 1 (Drosophila)*	*PELI1*	6.78	6.58	10.23	8.02E-06	2.88E-04
7953697				6.30	6.46	8.84	8.04E-06	2.88E-04
7994131	5579	*protein kinase C, beta*	*PRKCB*	6.35	6.50	8.82	8.10E-06	2.90E-04

7925823	23560	*GTP binding protein 4*	*GTPBP4*	6.92	7.35	8.78	8.12E-06	2.90E-04
7927519	100287932, 653252	*translocase of inner mitochondrial membrane 23 homolog (yeast) | translocase of inner mitochondrial membrane 23 homolog B (yeast)*	*TIMM23|TIMM23B*	11.24	11.50	12.21	8.11E-06	2.90E-04
7954090	2012	*epithelial membrane protein 1*	*EMP1*	11.21	11.86	6.04	8.15E-06	2.91E-04
8142981	5420	*podocalyxin-like*	*PODXL*	8.31	9.58	13.54	8.17E-06	2.91E-04
7978838	55172	*chromosome 14 open reading frame 104*	*C14orf104*	8.27	8.50	10.74	8.17E-06	2.91E-04
8151730	793	*calbindin 1, 28kDa*	*CALB1*	5.44	5.59	9.31	8.19E-06	2.91E-04
8109639	9232	*pituitary tumor-transforming 1*	*PTTG1*	7.31	7.57	10.29	8.20E-06	2.91E-04
8138749	3205, 3206	*homeobox A9 | homeobox A10*	*HOXA9|HOXA10*	6.77	8.82	6.25	8.23E-06	2.92E-04
8035808	163227	*zinc finger protein 100*	*ZNF100*	5.85	5.99	9.36	8.24E-06	2.92E-04
8046906	51454	*GULP, engulfment adaptor PTB domain containing 1*	*GULP1*	9.58	6.90	11.40	8.27E-06	2.92E-04
8061129	92667	*chromosome 20 open reading frame 72*	*C20orf72*	7.00	6.87	9.93	8.28E-06	2.92E-04
8097773	10586	*mab-21-like 2 (C. elegans)*	*MAB21L2*	10.11	6.45	5.87	8.38E-06	2.96E-04
8177717	3134	*major histocompatibility complex, class I, F*	*HLA-F*	10.29	10.03	8.25	8.43E-06	2.97E-04
8121712	222553	*solute carrier family 35, member F1*	*SLC35F1*	6.30	6.45	8.37	8.43E-06	2.97E-04
8016476	3219	*homeobox B9*	*HOXB9*	6.85	9.57	6.45	8.51E-06	2.97E-04
8090162	3693	*integrin, beta 5*	*ITGB5*	11.71	12.23	10.72	8.57E-06	2.97E-04
8095362	4502	*metallothionein 2A*	*MT2A*	13.74	13.79	11.37	8.52E-06	2.97E-04
8145440	5520	*protein phosphatase 2, regulatory subunit B, alpha*	*PPP2R2A*	8.76	8.99	10.31	8.53E-06	2.97E-04
8120838	7272		*TTK*	5.62	6.28	9.77	8.58E-06	2.97E-04
7983969	9133	*cyclin B2*	*CCNB2*	7.41	8.07	11.38	8.59E-06	2.97E-04
8142061	54517	*pseudouridylate synthase 7 homolog (S. cerevisiae)*	*PUS7*	6.65	6.99	9.16	8.56E-06	2.97E-04
8120279	55227	*leucine rich repeat containing 1*	*LRRC1*	6.89	6.60	8.48	8.54E-06	2.97E-04
8109752	57451	*odz, odd Oz/ten-m homolog 2 (Drosophila)*	*ODZ2*	6.92	11.55	6.36	8.53E-06	2.97E-04
8078173	92106	*oxidoreductase NAD-binding domain containing 1*	*OXNAD1*	7.97	8.29	9.64	8.51E-06	2.97E-04
8064336	128637	*TBC1 domain family, member 20*	*TBC1D20*	10.76	10.90	8.83	8.54E-06	2.97E-04
8136315	136332	*leucine-rich repeats and guanylate kinase domain containing*	*LRGUK*	5.89	5.82	7.19	8.48E-06	2.97E-04

7943749	143903	*layilin*	*LAYN*	11.13	11.30	8.63	8.56E-06	2.97E-04
7981970	100033423	*small nucleolar RNA, C/D box 116-11*	*SNORD116-11*	7.02	7.03	10.38	8.54E-06	2.97E-04
7981974	100033425	*small nucleolar RNA, C/D box 116-13*	*SNORD116-13*	6.62	6.54	10.51	8.51E-06	2.97E-04
8093166	80235	*phosphatidylinositol glycan anchor biosynthesis, class Z*	*PIGZ*	9.38	9.15	6.24	8.60E-06	2.97E-04
8117415	8353	*histone cluster 1, H3e*	*HIST1H3E*	5.71	5.99	7.71	8.64E-06	2.98E-04
7901447	84950	*PRP38 pre-mRNA processing factor 38 (yeast) domain containing A*	*PRPF38A*	9.55	9.53	11.33	8.65E-06	2.98E-04
8016832	23531	*monocyte to macrophage differentiation-associated*	*MMD*	7.23	7.45	10.12	8.68E-06	2.98E-04
7932214	414149	*acyl-CoA binding domain containing 7*	*ACBD7*	5.70	5.68	8.95	8.67E-06	2.98E-04
7939341	960		*CD44*	13.07	12.61	9.63	8.70E-06	2.98E-04
7906564	8682	*phosphoprotein enriched in astrocytes 15*	*PEA15*	13.00	13.03	11.35	8.71E-06	2.98E-04
8035847	171392	*zinc finger protein 675*	*ZNF675*	6.15	6.01	9.56	8.69E-06	2.98E-04
7986463	79705	*leucine-rich repeat kinase 1*	*LRRK1*	9.40	9.18	6.96	8.73E-06	2.98E-04
8118607	3116	*major histocompatibility complex, class II, DP beta 2 (pseudogene)*	*HLA-DPB2*	5.83	5.80	8.60	8.84E-06	3.02E-04
7927669	7019	*transcription factor A, mitochondrial*	*TFAM*	6.69	6.78	9.89	8.83E-06	3.02E-04
8075164	11200	*CHK2 checkpoint homolog (S. pombe)*	*CHEK2*	5.94	5.82	8.62	8.89E-06	3.02E-04
8073548	55964	*septin 3*	*3-Sep*	7.39	6.87	10.37	8.89E-06	3.02E-04
8146533	90362	*family with sequence similarity 110, member B*	*FAM110B*	8.53	9.16	7.39	8.90E-06	3.02E-04
7968236	387496	*RAS-like, family 11, member A*	*RASL11A*	9.13	6.77	6.79	8.89E-06	3.02E-04
8007323	8506	*contactin associated protein 1*	*CNTNAP1*	10.26	9.35	6.62	8.93E-06	3.03E-04
8147244	64168	*N-terminal EF-hand calcium binding protein 1*	*NECAB1*	5.57	5.71	8.82	8.93E-06	3.03E-04
7981994	100033434	*small nucleolar RNA, C/D box 116-23*	*SNORD116-23*	9.13	9.38	11.12	8.96E-06	3.03E-04
7928551	6229	*ribosomal protein S24*	*RPS24*	9.58	9.88	11.48	8.97E-06	3.03E-04
8155214	9833	*maternal embryonic leucine zipper kinase*	*MELK*	6.40	6.78	9.51	8.99E-06	3.03E-04
8132458	64983	*mitochondrial ribosomal protein L32*	*MRPL32*	9.15	9.37	10.38	8.98E-06	3.03E-04
7915363	22955	*sex comb on midleg homolog 1 (Drosophila)*	*SCMH1*	10.34	10.54	9.10	9.09E-06	3.06E-04

8133049	51427	*zinc finger protein 107*	*ZNF107*	6.88	6.85	9.74	9.09E-06	3.06E-04
8052382	55120, 7444	*Fanconi anemia, complementation group L | vaccinia related kinase 2*	*FANCL|VRK2*	6.69	6.73	9.21	9.09E-06	3.06E-04
7951977	53826	*FXYD domain containing ion transport regulator 6*	*FXYD6*	7.31	7.25	9.20	9.12E-06	3.06E-04
7930714	26033	*attractin-like 1*	*ATRNL1*	5.55	5.43	7.29	9.20E-06	3.08E-04
8105828	891	*cyclin B1*	*CCNB1*	7.10	7.37	10.59	9.24E-06	3.09E-04
7965486	51134	*coiled-coil domain containing 41*	*CCDC41*	5.62	5.56	7.63	9.23E-06	3.09E-04
7988260	84978	*FERM domain containing 5*	*FRMD5*	6.59	6.66	8.18	9.23E-06	3.09E-04
8008768	22843	*protein phosphatase, Mg2+/Mn2+ dependent, 1E*	*PPM1E*	5.80	5.95	7.68	9.27E-06	3.09E-04
8129937	10370	*Cbp/p300-interacting transactivator, with Glu/Asp-rich carboxy-terminal domain, 2*	*CITED2*	12.99	13.34	11.21	9.29E-06	3.10E-04
8115666	134492	*NudC domain containing 2*	*NUDCD2*	6.85	7.19	8.42	9.30E-06	3.10E-04
7982829	6692	*serine peptidase inhibitor, Kunitz type 1*	*SPINT1*	7.50	7.44	10.06	9.31E-06	3.10E-04
8143028	54927	*coiled-coil-helix-coiled-coil-helix domain containing 3*	*CHCHD3*	9.86	10.05	11.73	9.36E-06	3.11E-04
7932407	338596	*ST8 alpha-N-acetyl-neuraminide alpha-2,8-sialyltransferase 6*	*ST8SIA6*	4.97	5.11	6.88	9.37E-06	3.11E-04
8007141	10209	*eukaryotic translation initiation factor 1*	*EIF1*	13.59	13.62	13.21	9.39E-06	3.11E-04
8130867	7058	*thrombospondin 2*	*THBS2*	13.10	12.39	8.53	9.43E-06	3.12E-04
8037272	5673, 5671	*pregnancy specific beta-1-glycoprotein 5 | pregnancy specific beta-1-glycoprotein 3*	*PSG5|PSG3*	11.18	10.72	5.50	9.51E-06	3.14E-04
8017850	55062	*WD repeat domain, phosphoinositide interacting 1*	*WIPI1*	11.63	11.86	9.75	9.58E-06	3.16E-04
8096457				5.83	5.69	9.42	9.58E-06	3.16E-04
8151824	25788	*RAD54 homolog B (S. cerevisiae)*	*RAD54B*	5.97	6.28	8.77	9.60E-06	3.16E-04
7903959	128344	*chromosome 1 open reading frame 88*	*C1orf88*	5.85	5.71	7.52	9.60E-06	3.16E-04
8100393	3791	*kinase insert domain receptor (a type III receptor tyrosine kinase)*	*KDR*	6.10	6.06	9.99	9.64E-06	3.17E-04
8126531	401262	*cysteine-rich protein 3*	*CRIP3*	7.65	7.76	9.74	9.67E-06	3.18E-04
8017143	51651	*peptidyl-tRNA hydrolase 2*	*PTRH2*	6.84	7.16	8.58	9.70E-06	3.18E-04
8137091	57541	*zinc finger protein 398*	*ZNF398*	8.10	8.19	10.26	9.70E-06	3.18E-04
8168729				5.55	5.72	8.02	9.77E-06	3.20E-04
7995797	4493	*metallothionein 1E*	*MT1E*	10.74	10.85	8.50	9.89E-06	3.23E-04

7972180	79596	*ring finger protein 219*	*RNF219*	6.95	6.77	8.89	9.91E-06	3.24E-04
7920317	3608	*interleukin enhancer binding factor 2, 45kDa*	*ILF2*	12.18	12.27	13.25	9.94E-06	3.24E-04
8164200	23452	*angiopoietin-like 2*	*ANGPTL2*	12.07	11.00	8.15	9.96E-06	3.25E-04
8075635	7078	*TIMP metallopeptidase inhibitor 3*	*TIMP3*	9.87	11.03	7.05	1.00E-05	3.26E-04
8030002	163071	*zinc finger protein 114*	*ZNF114*	5.17	5.13	7.54	1.00E-05	3.26E-04
7990080	55323	*La ribonucleoprotein domain family, member 6*	*LARP6*	11.51	11.80	9.63	1.01E-05	3.29E-04
8165438	29952	*dipeptidyl-peptidase 7*	*DPP7*	11.10	11.21	8.35	1.02E-05	3.30E-04
8080144	10039	*poly (ADP-ribose) polymerase family, member 3*	*PARP3*	10.48	10.57	6.89	1.02E-05	3.30E-04
7953218	10635	*RAD51 associated protein 1*	*RAD51AP 1*	5.43	5.93	8.95	1.02E-05	3.30E-04
8095376	4502	*metallothionein 2A*	*MT2A*	13.48	13.53	11.13	1.02E-05	3.31E-04
7983928	3990	*lipase, hepatic*	*LIPC*	6.91	6.18	5.29	1.03E-05	3.32E-04
8133057	7697	*zinc finger protein 138*	*ZNF138*	6.55	6.49	9.74	1.03E-05	3.32E-04
7924172	25936		*NSL1*	9.62	9.58	10.86	1.03E-05	3.32E-04
8128371	84553	*chromosome 6 open reading frame 168*	*C6orf168*	7.25	7.31	9.42	1.03E-05	3.32E-04
7925452	64388	*gremlin 2*	*GREM2*	11.83	10.47	6.29	1.03E-05	3.32E-04
7909708	1063	*centromere protein F, 350/400kDa (mitosin)*	*CENPF*	6.93	7.10	11.13	1.04E-05	3.33E-04
8179019	3134	*major histocompatibility complex, class I, F*	*HLA-F*	9.44	9.19	7.47	1.04E-05	3.34E-04
7973936	5687	*proteasome (prosome, macropain) subunit, alpha type, 6*	*PSMA6*	10.48	10.76	12.30	1.05E-05	3.34E-04
7960529	6337	*sodium channel, nonvoltage-gated 1 alpha*	*SCNN1A*	7.15	7.02	9.96	1.04E-05	3.34E-04
8088491	8618	*Ca++-dependent secretion activator*	*CADPS*	5.73	6.25	8.12	1.05E-05	3.34E-04
8130374	26271	*F-box protein 5*	*FBXO5*	7.74	8.25	10.94	1.05E-05	3.34E-04
8002218	80004	*epithelial splicing regulatory protein 2*	*ESRP2*	6.86	6.90	9.11	1.05E-05	3.34E-04
8168984	80823	*basic helix-loop-helix domain containing, class B, 9*	*BHLHB9*	7.01	6.98	9.09	1.05E-05	3.34E-04
8128316	81491	*G protein-coupled receptor 63*	*GPR63*	7.40	5.83	9.48	1.05E-05	3.34E-04
8175256	84848		*MGC1612 1*	10.22	10.61	7.87	1.04E-05	3.34E-04
8147079	85444	*leucine rich repeat and coiled-coil domain containing 1*	*LRRCC1*	6.49	6.29	8.80	1.05E-05	3.34E-04

7958989	196463	*phospholipase B domain containing 2*	*PLBD2*	10.74	10.97	8.56	1.05E-05	3.34E-04
8047356	60491, 100129888	*NIF3 NGG1 interacting factor 3-like 1 (S. pombe) | hypothetical LOC100129888*	*NIF3L1|LOC100129888*	7.14	7.42	8.92	1.05E-05	3.34E-04
8040419	4613	*v-myc myelocytomatosis viral related oncogene, neuroblastoma derived (avian)*	*MYCN*	7.16	7.04	10.46	1.06E-05	3.36E-04
8057377	285025	*coiled-coil domain containing 141*	*CCDC141*	5.32	5.34	7.42	1.06E-05	3.36E-04
8042270	7360	*UDP-glucose pyrophosphorylase 2*	*UGP2*	9.34	9.18	11.63	1.08E-05	3.40E-04
7899394	9473	*chromosome 1 open reading frame 38*	*C1orf38*	6.99	7.30	8.45	1.08E-05	3.41E-04
8090433	11343	*monoglyceride lipase*	*MGLL*	9.96	9.77	6.37	1.08E-05	3.41E-04
8086880	993	*cell division cycle 25 homolog A (S. pombe)*	*CDC25A*	6.55	6.71	9.68	1.08E-05	3.41E-04
8148580	286122	*chromosome 8 open reading frame 31*	*C8orf31*	7.96	7.90	5.62	1.08E-05	3.41E-04
7952268	7070	*Thy-1 cell surface antigen*	*THY1*	13.19	12.74	10.82	1.09E-05	3.42E-04
8160321	25769	*solute carrier family 24 (sodium/potassium/calcium exchanger), member 2*	*SLC24A2*	6.13	6.15	7.24	1.09E-05	3.42E-04
8022176	284217	*laminin, alpha 1*	*LAMA1*	6.81	6.81	8.94	1.09E-05	3.42E-04
8025992	388507	*zinc finger family member 788*	*ZNF788*	6.43	6.23	8.65	1.09E-05	3.42E-04
8136473	8805	*tripartite motif-containing 24*	*TRIM24*	7.72	7.54	11.35	1.09E-05	3.42E-04
8115443				4.67	4.72	8.83	1.10E-05	3.42E-04
8046975	84128	*WD repeat domain 75*	*WDR75*	9.06	9.06	11.13	1.11E-05	3.47E-04
7983228	4130	*microtubule-associated protein 1A*	*MAP1A*	10.33	10.28	6.51	1.12E-05	3.47E-04
8050425				7.11	7.03	6.70	1.12E-05	3.47E-04
8064976	23234, 317662	*DnaJ (Hsp40) homolog, subfamily C, member 9 | family with sequence similarity 149, member B1*	*DNAJC9|FAM149B1*	8.02	8.56	9.68	1.12E-05	3.49E-04
8080082				5.35	5.37	7.13	1.12E-05	3.49E-04
7946142	112464	*protein kinase C, delta binding protein*	*PRKCDBP*	11.94	11.88	8.70	1.12E-05	3.49E-04
8136067	340348	*tetraspanin 33*	*TSPAN33*	8.41	7.14	10.19	1.13E-05	3.51E-04
8006788	4302	*myeloid/lymphoid or mixed-lineage leukemia (trithorax homolog, Drosophila); translocated to, 6*	*MLLT6*	9.25	9.08	7.65	1.14E-05	3.52E-04
7934185	64115	*chromosome 10 open reading frame 54*	*C10orf54*	10.59	10.45	6.84	1.14E-05	3.53E-04

8007148	2520	*gastrin*	*GAST*	7.15	7.16	6.46	1.14E-05	3.53E-04
8093320	152687, 255403	*zinc finger protein 595 | zinc finger protein 718*	*ZNF595|ZNF718*	6.44	6.35	8.55	1.15E-05	3.54E-04
7947396	1479	*cleavage stimulation factor, 3' pre-RNA, subunit 3, 77kDa*	*CSTF3*	7.31	7.10	10.03	1.15E-05	3.54E-04
8046380	3655	*integrin, alpha 6*	*ITGA6*	6.60	7.88	10.85	1.15E-05	3.54E-04
8012212	57659	*zinc finger and BTB domain containing 4*	*ZBTB4*	10.34	10.15	7.79	1.15E-05	3.54E-04
8061019	79133	*chromosome 20 open reading frame 7*	*C20orf7*	6.60	6.76	7.62	1.15E-05	3.54E-04
8027285	148206	*zinc finger protein 714*	*ZNF714*	7.19	7.24	9.70	1.15E-05	3.54E-04
8107356	167227		*DCP2*	7.78	7.65	10.89	1.15E-05	3.54E-04
8058373	55759	*WD repeat domain 12*	*WDR12*	7.54	7.91	10.66	1.16E-05	3.54E-04
7997381	55839, 56942	*centromere protein N | chromosome 16 open reading frame 61*	*CENPN|C16orf61*	8.70	9.35	10.71	1.16E-05	3.54E-04
7914878	63967	*claspin*	*CLSPN*	5.57	5.86	8.47	1.16E-05	3.54E-04
7904742	128077	*Lix1 homolog (mouse)-like*	*LIX1L*	10.25	10.42	8.18	1.17E-05	3.56E-04
7969374	79866, 22894	*chromosome 13 open reading frame 34 | DIS3 mitotic control homolog (S. cerevisiae)*	*C13orf34|DIS3*	6.20	6.24	8.56	1.17E-05	3.56E-04
8164087	2649	*nuclear receptor subfamily 6, group A, member 1*	*NR6A1*	6.56	6.59	10.28	1.18E-05	3.58E-04
8155460	79937, 728577, 643792	*contactin associated protein-like 3 | contactin associated protein-like 3B | contactin associated protein-like 3 pseudogene*	*CNTNAP3|CNTNAP3B|LOC643792*	5.57	5.57	8.03	1.18E-05	3.59E-04
7922408	692198	*small nucleolar RNA, C/D box 78*	*SNORD78*	6.85	6.64	9.81	1.18E-05	3.60E-04
7943827	1737	*dihydrolipoamide S-acetyltransferase*	*DLAT*	8.18	8.40	9.99	1.19E-05	3.62E-04
8007620	2896	*granulin*	*GRN*	13.25	13.00	11.34	1.19E-05	3.63E-04
7943998	4837	*nicotinamide N-methyltransferase*	*NNMT*	11.53	12.44	7.30	1.20E-05	3.63E-04
8075483	113791	*phosphoinositide-3-kinase interacting protein 1*	*PIK3IP1*	8.98	8.32	6.81	1.20E-05	3.64E-04
8116998	3720	*jumonji, AT rich interactive domain 2*	*JARID2*	7.76	7.76	12.04	1.21E-05	3.65E-04
7908793	1999	*E74-like factor 3 (ets domain transcription factor, epithelial-specific)*	*ELF3*	6.23	6.32	7.74	1.22E-05	3.68E-04
8010354	2548	*glucosidase, alpha; acid*	*GAA*	9.60	9.62	7.44	1.22E-05	3.68E-04
7985507	6457	*SH3-domain GRB2-like 3*	*SH3GL3*	5.89	6.03	7.79	1.22E-05	3.68E-04

7964360	6778	*signal transducer and activator of transcription 6, interleukin-4 induced*	*STAT6*	11.31	10.92	8.39	1.22E-05	3.68E-04
7930213	6877		*TAF5*	6.95	7.06	8.79	1.23E-05	3.68E-04
8117225	51053	*geminin, DNA replication inhibitor*	*GMNN*	6.64	6.87	10.34	1.22E-05	3.68E-04
7936507	143379	*chromosome 10 open reading frame 82*	*C10orf82*	6.81	6.76	8.78	1.22E-05	3.68E-04
7917946	163404	*lipid phosphate phosphatase-related protein type 5*	*LPPR5*	5.36	5.40	8.50	1.22E-05	3.68E-04
7911114	55083	*kinesin family member 26B*	*KIF26B*	7.84	8.66	6.63	1.23E-05	3.68E-04
8171182	5613	*protein kinase, X-linked*	*PRKX*	8.24	8.32	9.69	1.24E-05	3.71E-04
8053278	84141	*family with sequence similarity 176, member A*	*FAM176A*	7.61	8.53	6.34	1.24E-05	3.72E-04
7905220	1893	*extracellular matrix protein 1*	*ECM1*	11.48	11.32	6.95	1.25E-05	3.73E-04
7932390	1787	*tRNA aspartic acid methyltransferase 1*	*TRDMT1*	6.43	6.65	7.97	1.26E-05	3.75E-04
8040036	6201	*ribosomal protein S7*	*RPS7*	9.39	9.69	10.67	1.26E-05	3.75E-04
7945204	6768	*suppression of tumorigenicity 14 (colon carcinoma)*	*ST14*	6.78	6.91	8.76	1.26E-05	3.75E-04
8175420	27316, 494115	*RNA binding motif protein, X-linked | RNA binding motif protein, X-linked-like 1*	*RBMX|RBMXL1*	9.66	9.73	11.52	1.26E-05	3.75E-04
7973972				8.09	6.47	5.81	1.26E-05	3.76E-04
8097118	402483, 100131884	*hypothetical LOC402483 | hypothetical protein LOC100131884*	*FLJ45340|LOC100131884*	8.25	8.13	7.46	1.27E-05	3.77E-04
7900576	10465	*peptidylprolyl isomerase H (cyclophilin H)*	*PPIH*	8.12	8.24	10.25	1.27E-05	3.78E-04
8052397				8.89	9.01	7.88	1.28E-05	3.78E-04
8054930	84365	*MKI67 (FHA domain) interacting nucleolar phosphoprotein*	*MKI67IP*	10.39	10.79	11.72	1.28E-05	3.79E-04
8161288	79937, 389722	*contactin associated protein-like 3 | similar to cell recognition molecule CASPR3*	*CNTNAP3|CNTNAP3B*	5.75	5.84	8.33	1.28E-05	3.80E-04
8179555	8705	*UDP-Gal:betaGlcNAc beta 1,3-galactosyltransferase, polypeptide 4*	*B3GALT4*	8.28	7.85	7.27	1.29E-05	3.80E-04
7910600	84451	*mixed lineage kinase 4*	*KIAA1804*	6.87	6.98	8.35	1.29E-05	3.80E-04
8062190	6676	*sperm associated antigen 4*	*SPAG4*	7.28	7.62	6.64	1.30E-05	3.85E-04
8044111	64965	*mitochondrial ribosomal protein S9*	*MRPS9*	6.46	6.65	7.95	1.31E-05	3.86E-04
8047288	151246	*shugoshin-like 2 (S. pombe)*	*SGOL2*	6.20	6.52	8.94	1.31E-05	3.86E-04

7943051	10003	*N-acetylated alpha-linked acidic dipeptidase 2*	*NAALAD2*	5.18	5.19	6.53	1.31E-05	3.87E-04
8178264	8705	*UDP-Gal:betaGlcNAc beta 1,3-galactosyltransferase, polypeptide 4*	*B3GALT4*	8.28	7.85	7.27	1.32E-05	3.87E-04
7931268	56647	*BRCA2 and CDKN1A interacting protein*	*BCCIP*	8.07	8.16	9.75	1.32E-05	3.87E-04
8139723	360132	*FK506 binding protein 9-like*	*FKBP9L*	8.06	8.28	5.84	1.32E-05	3.88E-04
8155747	138255	*chromosome 9 open reading frame 135*	*C9orf135*	5.00	5.01	7.51	1.33E-05	3.89E-04
8093878	2121	*Ellis van Creveld syndrome*	*EVC*	9.55	9.52	7.50	1.33E-05	3.89E-04
8046848	55854	*zinc finger CCCH-type containing 15*	*ZC3H15*	9.60	9.60	10.98	1.34E-05	3.91E-04
7913252	65018, 1650	*PTEN induced putative kinase 1 | dolichyl-diphosphooligosaccharide--protein glycosyltransferase*	*PINK1|DDOST*	9.88	9.80	7.81	1.34E-05	3.91E-04
8103822	7424	*vascular endothelial growth factor C*	*VEGFC*	10.10	10.22	7.03	1.35E-05	3.91E-04
8174444	8471	*insulin receptor substrate 4*	*IRS4*	5.61	5.79	7.22	1.35E-05	3.91E-04
8160284	54801	*HAUS augmin-like complex, subunit 6*	*HAUS6*	7.11	7.34	10.59	1.35E-05	3.91E-04
8113602	153733	*coiled-coil domain containing 112*	*CCDC112*	6.02	6.11	7.79	1.34E-05	3.91E-04
8093425	3425	*iduronidase, alpha-L-*	*IDUA*	10.20	10.41	7.47	1.36E-05	3.93E-04
7899407	27293	*sphingomyelin phosphodiesterase, acid-like 3B*	*SMPDL3B*	7.00	6.85	10.78	1.36E-05	3.93E-04
7968563	5983	*replication factor C (activator 1) 3, 38kDa*	*RFC3*	7.06	7.54	11.28	1.36E-05	3.94E-04
8155048	9853	*RUN and SH3 domain containing 2*	*RUSC2*	9.92	10.59	7.34	1.36E-05	3.94E-04
8083749	151742	*protein phosphatase, Mg2+/Mn2+ dependent, 1L*	*PPM1L*	6.01	5.84	8.06	1.36E-05	3.94E-04
7976567	623	*bradykinin receptor B1*	*BDKRB1*	11.32	11.45	6.22	1.37E-05	3.95E-04
8071289	1312	*catechol-O-methyltransferase*	*COMT*	11.92	12.09	8.84	1.37E-05	3.95E-04
7968915	2963	*general transcription factor IIF, polypeptide 2, 30kDa*	*GTF2F2*	6.84	7.21	8.41	1.37E-05	3.95E-04
8072876	3956	*lectin, galactoside-binding, soluble, 1*	*LGALS1*	13.89	13.86	10.08	1.37E-05	3.95E-04
7976621	7443	*vaccinia related kinase 1*	*VRK1*	7.09	7.17	10.50	1.37E-05	3.95E-04
7985053	26263, 692224	*F-box protein 22 | FBXO22 opposite strand (non-protein coding)*	*FBXO22|FBXO22OS*	7.98	8.08	9.37	1.37E-05	3.95E-04
8165345	20	*ATP-binding cassette, sub-family A (ABC1), member 2*	*ABCA2*	9.44	9.48	7.03	1.38E-05	3.96E-04
8042052	6233	*ribosomal protein S27a*	*RPS27A*	9.52	9.72	10.55	1.38E-05	3.97E-04
7974461	3958	*lectin, galactoside-binding, soluble, 3*	*LGALS3*	9.23	9.20	6.78	1.39E-05	3.98E-04
8092640	5984	*replication factor C (activator 1) 4, 37kDa*	*RFC4*	7.64	8.14	10.93	1.39E-05	3.98E-04

8014974	7153	*topoisomerase (DNA) II alpha 170kDa*	*TOP2A*	7.34	7.50	11.02	1.39E-05	3.98E-04
8008969	6909	*T-box 2*	*TBX2*	8.89	8.34	7.39	1.39E-05	3.98E-04
8036151	126393	*heat shock protein, alpha-crystallin-related, B6*	*HSPB6*	11.17	11.71	6.93	1.40E-05	3.99E-04
8066985	140876	*family with sequence similarity 65, member C*	*FAM65C*	7.66	6.87	6.60	1.40E-05	3.99E-04
8075910	5880	*ras-related C3 botulinum toxin substrate 2 (rho family, small GTP binding protein Rac2)*	*RAC2*	9.87	10.54	7.56	1.41E-05	4.03E-04
7975238	57475	*pleckstrin homology domain containing, family H (with MyTH4 domain) member 1*	*PLEKHH1*	6.58	6.60	8.44	1.42E-05	4.03E-04
7993296	729993	*shisa homolog 9 (Xenopus laevis)*	*SHISA9*	8.33	8.27	10.50	1.42E-05	4.03E-04
8109159	406937, 728264	*microRNA 145 | hypothetical LOC728264*	*MIR145|LOC728264*	10.23	10.21	5.41	1.42E-05	4.03E-04
7969192	220108	*family with sequence similarity 124A*	*FAM124A*	6.49	6.24	9.18	1.42E-05	4.03E-04
8156571	407019, 84909	*microRNA 27b | chromosome 9 open reading frame 3*	*MIR27B|C9orf3*	9.02	8.54	5.78	1.42E-05	4.03E-04
8082003	55840	*ELL associated factor 2*	*EAF2*	6.01	5.82	7.69	1.43E-05	4.06E-04
7916167	4998	*origin recognition complex, subunit 1*	*ORC1*	6.48	6.60	9.76	1.44E-05	4.07E-04
7929816	6319	*stearoyl-CoA desaturase (delta-9-desaturase)*	*SCD*	10.70	9.75	12.31	1.44E-05	4.07E-04
8018288	10476	*ATP synthase, H+ transporting, mitochondrial Fo complex, subunit d*	*ATP5H*	11.38	11.47	11.92	1.44E-05	4.07E-04
8021286	162681	*chromosome 18 open reading frame 54*	*C18orf54*	5.84	6.00	9.20	1.44E-05	4.07E-04
7897339	23261	*calmodulin binding transcription activator 1*	*CAMTA1*	6.37	6.35	6.87	1.45E-05	4.10E-04
7906017	645682	*POU class 5 homeobox 1 pseudogene 4*	*POU5F1P4*	6.44	6.78	9.18	1.47E-05	4.14E-04
7970329	2621	*growth arrest-specific 6*	*GAS6*	9.42	10.85	6.80	1.48E-05	4.15E-04
8088180	7474	*wingless-type MMTV integration site family, member 5A*	*WNT5A*	10.28	9.99	5.96	1.48E-05	4.15E-04
8094759	79730	*NOP2/Sun domain family, member 7*	*NSUN7*	5.90	6.00	9.36	1.48E-05	4.15E-04
7969204	115825	*WD repeat and FYVE domain containing 2*	*WDFY2*	7.70	8.35	9.50	1.48E-05	4.15E-04
8113981	8974	*prolyl 4-hydroxylase, alpha polypeptide II*	*P4HA2*	11.21	11.45	7.98	1.49E-05	4.16E-04
8088264	54756	*interleukin 17 receptor D*	*IL17RD*	8.01	7.19	9.94	1.49E-05	4.16E-04
8019857	10403		*NDC80*	6.64	6.80	9.64	1.49E-05	4.17E-04
7974288	145447	*abhydrolase domain containing 12B*	*ABHD12B*	5.29	5.10	6.81	1.49E-05	4.17E-04

7944049	51092	*SID1 transmembrane family, member 2*	*SIDT2*	11.28	11.06	8.55	1.49E-05	4.17E-04
7910030	127602	*dynein, axonemal, heavy chain 14*	*DNAH14*	5.62	5.82	7.19	1.50E-05	4.17E-04
7920697	2629	*glucosidase, beta, acid*	*GBA*	11.49	11.25	8.91	1.50E-05	4.18E-04
8117760	3134	*major histocompatibility complex, class I, F*	*HLA-F*	8.42	8.16	6.53	1.50E-05	4.18E-04
8169949	51765		*MST4*	7.54	6.92	11.48	1.52E-05	4.21E-04
8148694	2907	*glutamate receptor, ionotropic, N-methyl D-aspartate-associated protein 1 (glutamate binding)*	*GRINA*	11.06	10.86	8.81	1.52E-05	4.22E-04
7925918	1645	*aldo-keto reductase family 1, member C1 (dihydrodiol dehydrogenase 1; 20-alpha (3-alpha)-hydroxysteroid dehydrogenase)*	*AKR1C1*	8.22	7.65	6.19	1.52E-05	4.22E-04
8170468	3149	*high-mobility group box 3*	*HMGB3*	7.09	7.59	10.30	1.52E-05	4.22E-04
7905131	23632	*carbonic anhydrase XIV*	*CA14*	6.31	6.23	8.36	1.53E-05	4.23E-04
8015196	81851, 81850	*keratin associated protein 1-1 | keratin associated protein 1-3*	*KRTAP1-1|KRTAP1-3*	8.67	10.02	7.73	1.53E-05	4.24E-04
8156476	5253	*PHD finger protein 2*	*PHF2*	9.86	9.74	7.84	1.55E-05	4.28E-04
7999079	115	*adenylate cyclase 9*	*ADCY9*	11.13	9.94	7.98	1.55E-05	4.28E-04
8088397	8309	*acyl-CoA oxidase 2, branched chain*	*ACOX2*	9.23	8.86	6.77	1.56E-05	4.29E-04
7928395	170384, 386671	*fucosyltransferase 11 (alpha (1,3) fucosyltransferase) | FLJ44715 gene product*	*FUT11|FLJ44715*	9.23	9.48	7.89	1.55E-05	4.29E-04
8142079	60561, 100130771	*RAD50 interactor 1 | EF-hand calcium binding domain 10*	*RINT1|EFCAB10*	5.17	5.20	7.12	1.56E-05	4.30E-04
8016444	3213	*homeobox B3*	*HOXB3*	6.57	8.98	6.19	1.57E-05	4.31E-04
8139820	340252	*zinc finger protein 680*	*ZNF680*	6.30	6.12	8.77	1.57E-05	4.31E-04
7922400				8.06	8.06	10.19	1.57E-05	4.31E-04
8168146	24137	*kinesin family member 4A*	*KIF4A*	6.17	6.17	8.61	1.57E-05	4.31E-04
8174527	827	*calpain 6*	*CAPN6*	6.39	6.43	8.75	1.57E-05	4.32E-04
8106776	1350	*cytochrome c oxidase subunit VIIc*	*COX7C*	9.39	9.33	10.65	1.58E-05	4.32E-04
8016870	51649	*mitochondrial ribosomal protein S23*	*MRPS23*	7.74	7.97	9.08	1.58E-05	4.32E-04
8123137	39, 100129518	*acetyl-CoA acetyltransferase 2 | hypothetical LOC100129518*	*ACAT2|LOC100129518*	7.73	6.98	9.91	1.58E-05	4.32E-04

7956949	57122	*nucleoporin 107kDa*	*NUP107*	6.80	7.00	9.77	1.58E-05	4.32E-04
7907893	3140	*major histocompatibility complex, class I-related*	*MR1*	9.75	9.44	5.65	1.59E-05	4.33E-04
7974198	122769	*peptidylprolyl isomerase (cyclophilin)-like 5*	*PPIL5*	5.96	6.36	8.04	1.59E-05	4.33E-04
7933707	11130	*ZW10 interactor*	*ZWINT*	7.10	7.56	10.04	1.60E-05	4.35E-04
8077160	410	*arylsulfatase A*	*ARSA*	10.29	10.24	6.67	1.60E-05	4.35E-04
8029006	558		*AXL*	12.39	12.45	10.04	1.60E-05	4.36E-04
8117537	8294	*histone cluster 1, H4i*	*HIST1H4I*	6.35	6.41	8.99	1.61E-05	4.36E-04
8079598	51385	*zinc finger protein 589*	*ZNF589*	7.09	7.04	10.62	1.61E-05	4.36E-04
7956301	4035	*low density lipoprotein receptor-related protein 1*	*LRP1*	13.28	13.06	9.85	1.62E-05	4.38E-04
7984364	4088	*SMAD family member 3*	*SMAD3*	11.42	10.39	8.82	1.62E-05	4.38E-04
8118655	8705	*UDP-Gal:betaGlcNAc beta 1,3-galactosyltransferase, polypeptide 4*	*B3GALT4*	8.28	7.85	7.28	1.62E-05	4.38E-04
7982004	100033820	*small nucleolar RNA, C/D box 116-28*	*SNORD116-28*	5.12	4.91	7.08	1.63E-05	4.40E-04
7960878	642559	*POU class 5 homeobox 1 pseudogene 3*	*POU5F1P3*	7.39	7.60	10.63	1.63E-05	4.41E-04
8019578	81851, 81850	*keratin associated protein 1-1 | keratin associated protein 1-3*	*KRTAP1-1|KRTAP1-3*	8.67	10.01	7.73	1.64E-05	4.43E-04
8008454	8714	*ATP-binding cassette, sub-family C (CFTR/MRP), member 3*	*ABCC3*	9.64	9.60	6.59	1.64E-05	4.43E-04
8073015	11015	*KDEL (Lys-Asp-Glu-Leu) endoplasmic reticulum protein retention receptor 3*	*KDELR3*	12.05	12.08	9.14	1.65E-05	4.45E-04
8046346	8520	*histone acetyltransferase 1*	*HAT1*	9.09	9.34	10.99	1.66E-05	4.45E-04
8090678	11222	*mitochondrial ribosomal protein L3*	*MRPL3*	9.72	10.06	11.43	1.66E-05	4.47E-04
8041149	23160	*WD repeat domain 43*	*WDR43*	8.20	8.25	10.66	1.66E-05	4.47E-04
7925320	4811	*nidogen 1*	*NID1*	11.16	11.00	8.29	1.66E-05	4.47E-04
7947248	81930	*kinesin family member 18A*	*KIF18A*	6.25	6.55	9.30	1.67E-05	4.47E-04
7953291	928		*CD9*	10.79	8.24	11.33	1.68E-05	4.48E-04
8039977	54221	*syntrophin, gamma 2*	*SNTG2*	6.42	6.49	7.47	1.68E-05	4.48E-04
8098379	116966	*WD repeat domain 17*	*WDR17*	5.03	5.04	6.51	1.68E-05	4.48E-04

8148619				7.09	6.88	6.52	1.68E-05	4.48E-04
8021154	9811		*KIAA0427*	10.19	10.12	7.29	1.68E-05	4.49E-04
8166447	139411	*patched domain containing 1*	*PTCHD1*	6.33	6.36	7.22	1.69E-05	4.50E-04
7969830	7546	*Zic family member 2 (odd-paired homolog, Drosophila)*	*ZIC2*	7.84	7.93	9.57	1.69E-05	4.51E-04
8173245	55613, 142689	*myotubularin related protein 8 | ankyrin repeat and SOCS box-containing 12*	*MTMR8|ASB12*	5.84	5.81	7.41	1.69E-05	4.51E-04
8147132	760	*carbonic anhydrase II*	*CA2*	5.66	5.80	7.56	1.70E-05	4.51E-04
8044880				4.68	4.55	5.62	1.70E-05	4.52E-04
7922410	26806	*small nucleolar RNA, C/D box 44*	*SNORD44*	7.32	7.28	9.69	1.71E-05	4.53E-04
7971218	84078	*kelch repeat and BTB (POZ) domain containing 7*	*KBTBD7*	8.05	7.31	9.21	1.71E-05	4.53E-04
8155359	79937, 728577	*contactin associated protein-like 3 | contactin associated protein-like 3B*	*CNTNAP3|CNTNAP3B*	5.15	5.14	7.55	1.71E-05	4.54E-04
7975045	4522	*methylenetetrahydrofolate dehydrogenase (NADP+ dependent) 1, methenyltetrahydrofolate cyclohydrolase, formyltetrahydrofolate synthetase*	*MTHFD1*	8.20	8.30	10.96	1.72E-05	4.54E-04
8035819	7757	*zinc finger protein 208*	*ZNF208*	5.12	4.79	9.91	1.73E-05	4.56E-04
8096688	79807	*glutathione S-transferase, C-terminal domain containing*	*GSTCD*	6.23	6.35	8.51	1.73E-05	4.56E-04
7950669	283219, 100289388	*potassium channel tetramerisation domain containing 21 | hypothetical LOC100289388*	*KCTD21|LOC100289388*	9.71	9.50	7.61	1.73E-05	4.56E-04
8011110				6.64	6.44	6.05	1.73E-05	4.56E-04
8127767	6785	*elongation of very long chain fatty acids (FEN1/Elo2, SUR4/Elo3, yeast)-like 4*	*ELOVL4*	6.93	6.92	8.95	1.75E-05	4.60E-04
8124484	8970	*histone cluster 1, H2bj*	*HIST1H2BJ*	6.04	6.25	6.88	1.75E-05	4.60E-04
8121142				5.01	4.98	5.44	1.75E-05	4.60E-04
8127989	692088	*small nucleolar RNA, C/D box 50B*	*SNORD50B*	6.55	6.52	8.50	1.75E-05	4.61E-04
8083941	1894	*epithelial cell transforming sequence 2 oncogene*	*ECT2*	6.92	7.07	9.91	1.77E-05	4.63E-04
8120165	55166	*centromere protein Q*	*CENPQ*	6.21	6.51	8.62	1.77E-05	4.63E-04

8021208	4200	*malic enzyme 2, NAD(+)-dependent, mitochondrial*	*ME2*	8.41	8.61	10.53	1.77E-05	4.64E-04
7928695	84293	*chromosome 10 open reading frame 58*	*C10orf58*	6.17	6.07	7.98	1.77E-05	4.64E-04
7916219	65260	*chromosome 1 open reading frame 163*	*C1orf163*	6.75	7.15	9.33	1.78E-05	4.64E-04
8079662	10425	*ariadne homolog 2 (Drosophila)*	*ARIH2*	9.55	9.58	10.79	1.79E-05	4.68E-04
7899750	84734	*family with sequence similarity 167, member B*	*FAM167B*	9.23	8.31	6.82	1.79E-05	4.68E-04
7902592	10620	*AT rich interactive domain 3B (BRIGHT-like)*	*ARID3B*	7.45	7.59	11.07	1.80E-05	4.68E-04
8173917	4675	*nucleosome assembly protein 1-like 3*	*NAP1L3*	6.54	6.80	10.17	1.80E-05	4.70E-04
8112902	1719	*dihydrofolate reductase*	*DHFR*	9.13	9.07	11.18	1.81E-05	4.72E-04
8085665	23180	*raftlin, lipid raft linker 1*	*RFTN1*	9.44	9.42	7.22	1.83E-05	4.75E-04
8083523	8833	*guanine monphosphate synthetase*	*GMPS*	10.36	10.34	11.89	1.83E-05	4.75E-04
8056323	55137	*fidgetin*	*FIGN*	7.17	6.91	9.75	1.84E-05	4.77E-04
8172043	8406	*sushi-repeat-containing protein, X-linked*	*SRPX*	12.10	11.80	9.44	1.84E-05	4.77E-04
7925662	57116	*zinc finger protein 695*	*ZNF695*	5.79	5.81	7.90	1.84E-05	4.77E-04
7995895	9709	*homocysteine-inducible, endoplasmic reticulum stress-inducible, ubiquitin-like domain member 1*	*HERPUD1*	11.87	12.15	9.18	1.84E-05	4.77E-04
8100338				6.71	6.45	8.24	1.85E-05	4.79E-04
8116591	2296	*forkhead box C1*	*FOXC1*	9.36	9.39	8.24	1.87E-05	4.81E-04
8102560	4085	*MAD2 mitotic arrest deficient-like 1 (yeast)*	*MAD2L1*	6.04	6.25	9.93	1.86E-05	4.81E-04
8059301	5077	*paired box 3*	*PAX3*	9.86	8.15	5.88	1.86E-05	4.81E-04
7977761	6297	*sal-like 2 (Drosophila)*	*SALL2*	7.61	7.28	10.85	1.86E-05	4.81E-04
8158059	6812	*syntaxin binding protein 1*	*STXBP1*	11.51	11.53	10.08	1.87E-05	4.81E-04
8091799	165679	*chromosome 3 open reading frame 57*	*C3orf57*	5.84	5.94	8.01	1.87E-05	4.81E-04
7906878	4921	*discoidin domain receptor tyrosine kinase 2*	*DDR2*	11.61	11.57	7.31	1.87E-05	4.81E-04
7959025	84900	*ring finger protein, transmembrane 2*	*RNFT2*	6.87	6.87	8.76	1.88E-05	4.82E-04
7982326	51621	*Kruppel-like factor 13*	*KLF13*	9.18	9.42	8.41	1.88E-05	4.82E-04
8090988	80321	*centrosomal protein 70kDa*	*CEP70*	6.83	6.07	8.84	1.88E-05	4.83E-04
7986068	641	*Bloom syndrome, RecQ helicase-like*	*BLM*	5.96	6.02	10.16	1.90E-05	4.87E-04
7923578	2331	*fibromodulin*	*FMOD*	11.14	9.52	5.90	1.91E-05	4.88E-04

7942417	9828	*Rho guanine nucleotide exchange factor (GEF) 17*	*ARHGEF17*	9.59	9.67	7.22	1.91E-05	4.88E-04
8002999	2653, 729080, 100329108, 100329109	*glycine cleavage system protein H (aminomethyl carrier) | glycine cleavage system H pseudogene | glycine cleavage system protein H pseudogene*	*GCSH|LOC729080|LOC100329108|LOC100329109*	8.64	9.06	11.05	1.91E-05	4.88E-04
7919578	2946	*glutathione S-transferase mu 2 (muscle)*	*GSTM2*	7.00	7.19	10.99	1.92E-05	4.89E-04
8071332	5902	*RAN binding protein 1*	*RANBP1*	8.27	8.64	10.78	1.93E-05	4.89E-04
7959052	5985	*replication factor C (activator 1) 5, 36.5kDa*	*RFC5*	7.45	7.57	9.51	1.92E-05	4.89E-04
8148124	27085	*Mdm2, transformed 3T3 cell double minute 2, p53 binding protein (mouse) binding protein, 104kDa*	*MTBP*	5.97	5.96	8.10	1.92E-05	4.89E-04
7965200	29080	*coiled-coil domain containing 59*	*CCDC59*	7.78	8.03	9.44	1.92E-05	4.89E-04
8117194	57380		*MRS2*	8.16	8.22	11.67	1.93E-05	4.89E-04
7963923	84324	*SAP domain containing ribonucleoprotein*	*SARNP*	7.95	8.09	8.81	1.92E-05	4.89E-04
8175299	441518	*family with sequence similarity 127, member C*	*FAM127C*	12.12	12.02	9.99	1.92E-05	4.89E-04
8135410	11062, 10466	*dihydrouridine synthase 4-like (S. cerevisiae) | component of oligomeric golgi complex 5*	*DUS4L|COG5*	6.93	6.89	7.96	1.94E-05	4.90E-04
7899943	79830	*zinc finger, MYM-type 1*	*ZMYM1*	7.01	7.03	9.09	1.94E-05	4.91E-04
8174543	1641	*doublecortin*	*DCX*	5.93	5.94	6.80	1.95E-05	4.91E-04
7949465	5970	*v-rel reticuloendotheliosis viral oncogene homolog A (avian)*	*RELA*	11.20	11.32	8.90	1.94E-05	4.91E-04
8117834	6992	*protein phosphatase 1, regulatory (inhibitor) subunit 11*	*PPP1R11*	10.72	10.84	8.97	1.94E-05	4.91E-04
7958455	7374	*uracil-DNA glycosylase*	*UNG*	8.05	8.12	10.52	1.95E-05	4.92E-04
8177744	6992	*protein phosphatase 1, regulatory (inhibitor) subunit 11*	*PPP1R11*	10.72	10.84	8.97	1.95E-05	4.92E-04
7911657	728690		*LOC728690*	7.48	7.65	6.92	1.95E-05	4.92E-04
7984538				6.51	6.46	6.03	1.97E-05	4.95E-04
7934459	8509	*N-deacetylase/N-sulfotransferase (heparan glucosaminyl) 2*	*NDST2*	10.45	10.36	8.40	1.97E-05	4.96E-04

7933192	10151	*heterogeneous nuclear ribonucleoprotein A3 pseudogene 1*	*HNRNPA3P1*	6.02	6.12	7.65	1.98E-05	4.97E-04
8147566	3788	*potassium voltage-gated channel, delayed-rectifier, subfamily S, member 2*	*KCNS2*	8.59	7.30	6.75	1.99E-05	4.99E-04
8040655	85465, 165082	*ethanolaminephosphotransferase 1 (CDP-ethanolamine-specific) | G protein-coupled receptor 113*	*EPT1|GPR113*	6.64	6.64	9.71	1.99E-05	4.99E-04
8178115	629	*complement factor B*	*CFB*	8.65	7.89	6.18	1.99E-05	5.00E-04
8128329	253714	*MMS22-like, DNA repair protein*	*MMS22L*	5.95	6.04	8.87	2.00E-05	5.00E-04
8014956	9572, 7067	*nuclear receptor subfamily 1, group D, member 1 | thyroid hormone receptor, alpha (erythroblastic leukemia viral (v-erb-a) oncogene homolog, avian)*	*NR1D1|THRA*	9.41	10.26	7.75	2.00E-05	5.00E-04
8173647	158866	*zinc finger, DHHC-type containing 15*	*ZDHHC15*	5.86	5.46	7.87	2.03E-05	5.06E-04
7901915	29929	*asparagine-linked glycosylation 6, alpha-1,3-glucosyltransferase homolog (S. cerevisiae)*	*ALG6*	6.31	5.95	8.61	2.03E-05	5.07E-04
8093330				6.65	6.51	8.60	2.03E-05	5.07E-04
7954006	5757	*prothymosin, alpha*	*PTMA*	11.81	11.90	13.07	2.04E-05	5.08E-04
8105111	26272	*F-box protein 4*	*FBXO4*	7.54	7.86	5.69	2.04E-05	5.09E-04
8003773	23108	*RAP1 GTPase activating protein 2*	*RAP1GAP2*	7.18	7.30	9.01	2.05E-05	5.09E-04
7905016	164022, 644591	*peptidylprolyl isomerase A (cyclophilin A)-like 4A | peptidylprolyl isomerase A (cyclophilin A)-like 4G*	*PPIAL4A|PPIAL4G*	7.67	7.65	7.98	2.05E-05	5.10E-04
8138708	3198	*homeobox A1*	*HOXA1*	6.23	6.87	5.66	2.05E-05	5.10E-04
8128429	892	*cyclin C*	*CCNC*	10.12	9.99	11.78	2.05E-05	5.10E-04
8097753	166614	*doublecortin-like kinase 2*	*DCLK2*	8.59	8.47	6.06	2.07E-05	5.14E-04
7914361	1307	*collagen, type XVI, alpha 1*	*COL16A1*	8.55	8.77	7.25	2.08E-05	5.15E-04
8166202	2925	*gastrin-releasing peptide receptor*	*GRPR*	7.03	6.29	11.90	2.08E-05	5.15E-04
8008310	146956	*essential meiotic endonuclease 1 homolog 1 (S. pombe)*	*EME1*	6.46	6.78	8.26	2.08E-05	5.15E-04
8056860	7456	*WAS/WASL interacting protein family, member 1*	*WIPF1*	10.65	10.72	7.71	2.08E-05	5.15E-04
8093336	7700	*zinc finger protein 141*	*ZNF141*	7.88	7.60	10.38	2.09E-05	5.16E-04
7988537	22995	*centrosomal protein 152kDa*	*CEP152*	5.57	5.51	6.84	2.09E-05	5.16E-04
8027650	10054	*ubiquitin-like modifier activating enzyme 2*	*UBA2*	10.07	10.41	11.54	2.10E-05	5.16E-04

7904997	164022, 644591	*peptidylprolyl isomerase A (cyclophilin A)-like 4A | peptidylprolyl isomerase A (cyclophilin A)-like 4G*	*PPIAL4A| PPIAL4G*	7.67	7.65	7.98	2.09E-05	5.16E-04
8021187	220134	*spindle and kinetochore associated complex subunit 1*	*SKA1*	5.84	6.25	8.27	2.10E-05	5.17E-04
8067167	6790	*aurora kinase A*	*AURKA*	7.31	7.63	10.92	2.11E-05	5.18E-04
8041225	30845	*EH-domain containing 3*	*EHD3*	10.75	9.44	8.48	2.11E-05	5.18E-04
8113443				7.23	7.02	6.50	2.11E-05	5.18E-04
8161727	51104	*family with sequence similarity 108, member B1*	*FAM108B1*	7.00	7.16	9.97	2.13E-05	5.22E-04
8175039	2000	*E74-like factor 4 (ets domain transcription factor)*	*ELF4*	9.86	9.89	6.98	2.14E-05	5.23E-04
8070467	7113	*transmembrane protease, serine 2*	*TMPRSS2*	5.97	6.10	7.98	2.14E-05	5.23E-04
8028311	10653	*serine peptidase inhibitor, Kunitz type, 2*	*SPINT2*	7.31	7.12	9.81	2.14E-05	5.23E-04
7978776	55320	*chromosome 14 open reading frame 106*	*C14orf106*	6.86	7.00	9.60	2.14E-05	5.23E-04
8170921	55558	*plexin A3*	*PLXNA3*	9.91	10.09	7.15	2.14E-05	5.23E-04
7914851	63967	*claspin*	*CLSPN*	5.36	5.62	7.20	2.14E-05	5.23E-04
8151074	5150	*phosphodiesterase 7A*	*PDE7A*	6.13	6.36	9.17	2.15E-05	5.24E-04
8161648	687	*Kruppel-like factor 9*	*KLF9*	8.52	8.40	7.28	2.16E-05	5.26E-04
7927710	983	*cyclin-dependent kinase 1*	*CDK1*	5.68	6.52	10.20	2.17E-05	5.26E-04
7995206	7041	*transforming growth factor beta 1 induced transcript 1*	*TGFB1I1*	11.33	11.50	8.03	2.16E-05	5.26E-04
8052562	10575	*chaperonin containing TCP1, subunit 4 (delta)*	*CCT4*	9.60	9.75	11.44	2.17E-05	5.26E-04
8174189	11013	*thymosin beta 15a*	*TMSB15A*	5.66	5.50	8.21	2.17E-05	5.26E-04
8046646	114880	*oxysterol binding protein-like 6*	*OSBPL6*	6.65	7.67	8.05	2.17E-05	5.26E-04
8030991	147804	*tropomyosin 3 pseudogene*	*LOC147804*	10.40	10.60	11.51	2.18E-05	5.27E-04
8045009	2995	*glycophorin C (Gerbich blood group)*	*GYPC*	10.14	9.61	7.02	2.19E-05	5.28E-04
7929550	54619	*cyclin J*	*CCNJ*	8.13	8.11	10.74	2.19E-05	5.28E-04
7989759	54956	*poly (ADP-ribose) polymerase family, member 16*	*PARP16*	9.74	9.06	7.80	2.18E-05	5.28E-04
7977507	85495	*ribonuclease P RNA component H1*	*RPPH1*	11.37	11.51	9.94	2.19E-05	5.28E-04
7907830	5768, 200058	*quiescin Q6 sulfhydryl oxidase 1 | hypothetical protein FLJ23867*	*QSOX1|FLJ23867*	12.25	11.98	9.56	2.19E-05	5.28E-04
7983661				4.57	4.54	5.30	2.18E-05	5.28E-04

7940904	7423	*vascular endothelial growth factor B*	*VEGFB*	10.21	10.30	8.67	2.19E-05	5.29E-04
7940582	7439	*bestrophin 1*	*BEST1*	8.71	8.77	6.42	2.20E-05	5.30E-04
8120552	57579	*family with sequence similarity 135, member A*	*FAM135A*	6.96	6.84	9.33	2.20E-05	5.30E-04
8056693	79675	*FAST kinase domains 1*	*FASTKD1*	6.97	7.02	9.05	2.20E-05	5.30E-04
8043909	4862	*neuronal PAS domain protein 2*	*NPAS2*	9.89	10.62	7.52	2.21E-05	5.30E-04
7911591	728661, 9906	*solute carrier family 35, member E2B | solute carrier family 35, member E2*	*SLC35E2B*	10.31	10.03	8.87	2.21E-05	5.31E-04
7996430	3299, 55336	*heat shock transcription factor 4 | F-box and leucine-rich repeat protein 8*	*HSF4|FBXL8*	7.80	7.71	6.86	2.21E-05	5.31E-04
8049246	3635	*inositol polyphosphate-5-phosphatase, 145kDa*	*INPP5D*	6.60	6.65	7.84	2.22E-05	5.32E-04
8152323	3646	*eukaryotic translation initiation factor 3, subunit E*	*EIF3E*	10.06	9.53	10.87	2.22E-05	5.32E-04
8076998	23654	*plexin B2*	*PLXNB2*	12.13	12.08	9.81	2.23E-05	5.32E-04
8180166	6892	*TAP binding protein (tapasin)*	*TAPBP*	12.15	11.61	9.72	2.23E-05	5.34E-04
7995580				9.44	9.61	10.49	2.24E-05	5.35E-04
8071044	727764, 727768, 100132288, 100233156	*MAFF interacting protein | tektin 4 pseudogene 1 | hypothetical protein LOC100132288 | hypothetical LOC100233156*	*MAFIP|TEKT4P1|LOC100132288|LOC100233156*	9.44	9.72	6.53	2.25E-05	5.37E-04
7994659	9961	*major vault protein*	*MVP*	9.65	9.65	6.60	2.26E-05	5.37E-04
8154654	51198	*chromosome 9 open reading frame 53*	*C9orf53*	6.56	6.55	6.20	2.25E-05	5.37E-04
8052399	53335	*B-cell CLL/lymphoma 11A (zinc finger protein)*	*BCL11A*	7.28	7.47	10.42	2.26E-05	5.39E-04
7990090				9.98	10.14	11.17	2.26E-05	5.39E-04
7926896	1163	*CDC28 protein kinase regulatory subunit 1B*	*CKS1B*	9.74	10.24	12.73	2.27E-05	5.39E-04
8082478	22820	*coatomer protein complex, subunit gamma*	*COPG*	12.11	12.06	10.90	2.27E-05	5.39E-04
8135378	5577	*protein kinase, cAMP-dependent, regulatory, type II, beta*	*PRKAR2B*	7.55	6.82	10.54	2.28E-05	5.40E-04
7984567				6.88	5.57	5.43	2.28E-05	5.41E-04
8150698	6591	*snail homolog 2 (Drosophila)*	*SNAI2*	12.51	12.64	7.73	2.30E-05	5.45E-04
8133062	10793	*zinc finger protein 273*	*ZNF273*	6.85	6.70	10.14	2.31E-05	5.45E-04
8027297	148203	*zinc finger protein 738*	*ZNF738*	7.26	7.48	11.42	2.31E-05	5.45E-04

7948987	11145	*phospholipase A2, group XVI*	*PLA2G16*	7.39	7.15	9.43	2.31E-05	5.46E-04
8052554	84140	*family with sequence similarity 161, member A*	*FAM161A*	5.61	5.49	8.17	2.31E-05	5.46E-04
7903457	55170	*protein arginine methyltransferase 6*	*PRMT6*	6.56	6.94	8.44	2.32E-05	5.47E-04
8157487	5069, 493913	*pregnancy-associated plasma protein A, pappalysin 1 | PAPPA antisense RNA (non-protein coding)*	*PAPPA|PAPPAS*	10.20	10.42	6.97	2.32E-05	5.47E-04
8113073	57561	*arrestin domain containing 3*	*ARRDC3*	11.49	10.96	10.15	2.34E-05	5.50E-04
8125713	6892	*TAP binding protein (tapasin)*	*TAPBP*	11.33	10.85	9.16	2.34E-05	5.50E-04
8155121	4882	*natriuretic peptide receptor B/guanylate cyclase B (atrionatriuretic peptide receptor B)*	*NPR2*	10.57	9.91	8.06	2.35E-05	5.53E-04
8029446	56971	*carcinoembryonic antigen-related cell adhesion molecule 19*	*CEACAM19*	8.11	7.63	6.78	2.35E-05	5.53E-04
7938629	5140	*phosphodiesterase 3B, cGMP-inhibited*	*PDE3B*	5.11	5.12	7.36	2.36E-05	5.54E-04
7936661	10935	*peroxiredoxin 3*	*PRDX3*	10.05	10.21	11.60	2.36E-05	5.54E-04
7931930	5588	*protein kinase C, theta*	*PRKCQ*	6.09	6.12	8.73	2.37E-05	5.55E-04
7918379	2947	*glutathione S-transferase mu 3 (brain)*	*GSTM3*	9.84	9.21	6.01	2.39E-05	5.60E-04
7960165	10795	*zinc finger protein 268*	*ZNF268*	7.37	7.13	8.53	2.39E-05	5.60E-04
7998174	55692	*LUC7-like (S. cerevisiae)*	*LUC7L*	8.11	8.19	9.37	2.40E-05	5.61E-04
8041961	494143	*ChaC, cation transport regulator homolog 2 (E. coli)*	*CHAC2*	6.02	6.27	8.47	2.41E-05	5.63E-04
8094704	3093	*ubiquitin-conjugating enzyme E2K (UBC1 homolog, yeast)*	*UBE2K*	10.36	10.62	11.91	2.42E-05	5.64E-04
7918913	3321	*immunoglobulin superfamily, member 3*	*IGSF3*	6.55	6.71	8.39	2.42E-05	5.64E-04
8018803	6427	*serine/arginine-rich splicing factor 2*	*SRSF2*	9.24	9.47	11.26	2.42E-05	5.65E-04
7997332	283927	*nudix (nucleoside diphosphate linked moiety X)-type motif 7*	*NUDT7*	8.68	7.62	9.79	2.43E-05	5.65E-04
8162183	440173		*LOC440173*	5.90	5.66	7.24	2.43E-05	5.65E-04
8128138	23195		*MDN1*	6.83	6.85	10.05	2.43E-05	5.66E-04
8016468	3217	*homeobox B7*	*HOXB7*	5.82	6.97	5.41	2.45E-05	5.67E-04
8171624	10149	*G protein-coupled receptor 64*	*GPR64*	5.88	5.91	7.90	2.45E-05	5.67E-04
8174715	26779	*small nucleolar RNA, H/ACA box 69*	*SNORA69*	4.74	4.87	6.14	2.44E-05	5.67E-04
8152506	401474	*sterile alpha motif domain containing 12*	*SAMD12*	5.44	5.62	7.08	2.45E-05	5.67E-04

8143627	346528, 402317	*olfactory receptor, family 2, subfamily A, member 1 | olfactory receptor, family 2, subfamily A, member 42*	*OR2A1|OR2A42*	7.73	7.51	6.60	2.45E-05	5.67E-04
7970563				4.76	4.75	5.15	2.45E-05	5.67E-04
8006479	3980	*ligase III, DNA, ATP-dependent*	*LIG3*	8.54	8.72	10.23	2.46E-05	5.68E-04
7922104	2653, 729080, 100329108, 100329109	*glycine cleavage system protein H (aminomethyl carrier) | glycine cleavage system H pseudogene | glycine cleavage system protein H pseudogene*	*GCSH|LOC729080|LOC100329108|LOC100329109*	8.70	9.13	11.11	2.46E-05	5.68E-04
7945864	7748	*zinc finger protein 195*	*ZNF195*	7.79	7.81	10.65	2.47E-05	5.71E-04
8064100	79144	*pancreatic progenitor cell differentiation and proliferation factor homolog (zebrafish)*	*PPDPF*	12.93	12.78	10.79	2.48E-05	5.71E-04
8081786	79691	*queuine tRNA-ribosyltransferase domain containing 1*	*QTRTD1*	6.62	6.58	9.08	2.48E-05	5.71E-04
8027292	170959	*zinc finger protein 431*	*ZNF431*	6.09	6.26	9.81	2.48E-05	5.71E-04
8164535	1384	*carnitine O-acetyltransferase*	*CRAT*	10.86	10.89	7.93	2.49E-05	5.73E-04
8070129	29980	*downstream neighbor of SON*	*DONSON*	8.11	8.19	10.13	2.50E-05	5.75E-04
8119227	60685	*zinc finger, AN1-type domain 3*	*ZFAND3*	10.29	10.26	9.00	2.52E-05	5.78E-04
8082663	152195	*nudix (nucleoside diphosphate linked moiety X)-type motif 16 pseudogene 1*	*NUDT16P1*	8.07	7.76	9.69	2.52E-05	5.79E-04
7964300	9880	*zinc finger and BTB domain containing 39*	*ZBTB39*	9.07	8.91	10.51	2.53E-05	5.79E-04
7989915	54962	*TIMELESS interacting protein*	*TIPIN*	7.50	7.60	10.01	2.53E-05	5.79E-04
8121002	57150	*chromosome 6 open reading frame 162*	*C6orf162*	6.86	6.60	8.33	2.53E-05	5.79E-04
7945040	219844	*hydrolethalus syndrome 1*	*HYLS1*	5.59	5.75	6.64	2.53E-05	5.79E-04
8070330	8624	*proteasome (prosome, macropain) assembly chaperone 1*	*PSMG1*	8.78	9.27	10.32	2.55E-05	5.82E-04
8022640	1719	*dihydrofolate reductase*	*DHFR*	9.17	9.15	11.25	2.56E-05	5.84E-04
7917674	6418		*SET*	8.55	8.64	11.48	2.56E-05	5.84E-04
8003850	124936	*cytochrome b5 domain containing 2*	*CYB5D2*	9.61	9.39	8.13	2.56E-05	5.84E-04
8155540	79937, 728577	*contactin associated protein-like 3 | contactin associated protein-like 3B*	*CNTNAP3|CNTNAP3B*	5.08	5.15	7.50	2.56E-05	5.84E-04

8027368	9534	*zinc finger protein 254*	*ZNF254*	7.47	7.21	9.86	2.58E-05	5.87E-04
8114991	79628	*SH3 domain and tetratricopeptide repeats 2*	*SH3TC2*	6.66	6.37	5.86	2.58E-05	5.87E-04
8087419	275, 84276	*aminomethyltransferase | nicolin 1*	*AMT|NICN1*	7.79	7.56	9.44	2.58E-05	5.87E-04
7981982	100033429, 727708, 100033427, 100033428, 100033430, 100033432, 100033433, 100033426, 100033431, 692236, 6638	*small nucleolar RNA, C/D box 116-17 | small nucleolar RNA, C/D box 116-19 | small nucleolar RNA, C/D box 116-15 | small nucleolar RNA, C/D box 116-16 | small nucleolar RNA, C/D box 116-18 | small nucleolar RNA, C/D box 116-21 | small nucleolar RNA, C/D box 116-22 | small nucleolar RNA, C/D box 116-14 | small nucleolar RNA, C/D box 116-20 | small nucleolar RNA, C/D box 116 cluster | small nuclear ribonucleoprotein polypeptide N*	*SNORD116-17|SNORD116-19|SNORD116-15|SNORD116-16|SNORD116-18|SNORD116-21|SNORD116-22|SNORD116-14|SNORD116-20|SNORD116@|SNRPN*	12.38	12.52	13.06	2.59E-05	5.88E-04
8161460	79937, 728577	*contactin associated protein-like 3 | contactin associated protein-like 3B*	*CNTNAP3|CNTNAP3B*	5.10	5.16	7.49	2.59E-05	5.89E-04
7897044	5590	*protein kinase C, zeta*	*PRKCZ*	7.46	7.24	9.27	2.60E-05	5.89E-04
8145570	157570	*establishment of cohesion 1 homolog 2 (S. cerevisiae)*	*ESCO2*	5.81	6.12	9.49	2.60E-05	5.89E-04
8165682				11.58	11.37	10.50	2.60E-05	5.89E-04
8042917	1796	*docking protein 1, 62kDa (downstream of tyrosine kinase 1)*	*DOK1*	9.48	9.89	7.89	2.61E-05	5.90E-04
8108627	56664	*vault RNA 1-1*	*VTRNA1-1*	8.91	9.32	6.50	2.61E-05	5.90E-04
7954729	121512	*FYVE, RhoGEF and PH domain containing 4*	*FGD4*	6.85	6.08	9.07	2.61E-05	5.90E-04

7922402	26802, 60674	*small nucleolar RNA, C/D box 47 | growth arrest-specific 5 (non-protein coding)*	*SNORD47|GAS5*	9.05	9.36	10.91	2.61E-05	5.90E-04
8163716	1620	*deleted in bladder cancer 1*	*DBC1*	6.40	7.00	8.68	2.62E-05	5.90E-04
8159977	6019	*relaxin 2*	*RLN2*	4.66	4.75	5.87	2.62E-05	5.90E-04
8019061	6448	*N-sulfoglucosamine sulfohydrolase*	*SGSH*	11.12	10.41	8.13	2.62E-05	5.90E-04
7964579	10106	*CTD (carboxy-terminal domain, RNA polymerase II, polypeptide A) small phosphatase 2*	*CTDSP2*	12.05	11.76	10.46	2.62E-05	5.90E-04
8155167	646962	*histidine rich carboxyl terminus 1*	*HRCT1*	7.33	7.76	6.45	2.64E-05	5.93E-04
8169389	5063	*p21 protein (Cdc42/Rac)-activated kinase 3*	*PAK3*	5.98	6.17	7.23	2.64E-05	5.94E-04
7940135	92292	*glycine-N-acyltransferase-like 1*	*GLYATL1*	5.80	5.82	7.63	2.65E-05	5.94E-04
7981986	100033429, 727708, 100033427, 100033428, 100033430, 100033432, 100033433, 100033426, 100033431, 692236, 6638	*small nucleolar RNA, C/D box 116-17 | small nucleolar RNA, C/D box 116-19 | small nucleolar RNA, C/D box 116-15 | small nucleolar RNA, C/D box 116-16 | small nucleolar RNA, C/D box 116-18 | small nucleolar RNA, C/D box 116-21 | small nucleolar RNA, C/D box 116-22 | small nucleolar RNA, C/D box 116-14 | small nucleolar RNA, C/D box 116-20 | small nucleolar RNA, C/D box 116 cluster | small nuclear ribonucleoprotein polypeptide N*	*SNORD116-17|SNORD116-19|SNORD116-15|SNORD116-16|SNORD116-18|SNORD116-21|SNORD116-22|SNORD116-14|SNORD116-20|SNORD116@|SNRPN*	12.38	12.52	13.06	2.65E-05	5.94E-04
8077728	55831, 442075	*transmembrane protein 111 | hypothetical LOC442075*	*TMEM111|LOC442075*	6.58	6.66	8.85	2.66E-05	5.95E-04

8071051	727764, 727768, 441057, 100132288, 100233156	*MAFF interacting protein | tektin 4 pseudogene 1 | hypothetical gene supported by AK096952; AK126241; BC068588 | hypothetical protein LOC100132288 | hypothetical LOC100233156*	*MAFIP|TEKT4P1|FLJ44253|LOC100132288|LOC100233156*	9.62	9.92	6.77	2.66E-05	5.96E-04
8118345	629	*complement factor B*	*CFB*	8.58	7.86	6.21	2.68E-05	5.99E-04
7930139	81603	*tripartite motif-containing 8*	*TRIM8*	11.46	11.61	9.19	2.68E-05	5.99E-04
8096032	56978	*PR domain containing 8*	*PRDM8*	9.20	9.16	7.58	2.69E-05	6.02E-04
7933180	8187	*zinc finger protein 239*	*ZNF239*	6.49	6.48	7.85	2.70E-05	6.02E-04
8016463	3216	*homeobox B6*	*HOXB6*	6.06	8.55	5.67	2.70E-05	6.02E-04
8032755	51341	*zinc finger and BTB domain containing 7A*	*ZBTB7A*	9.83	10.10	8.21	2.70E-05	6.02E-04
8026047	3726	*jun B proto-oncogene*	*JUNB*	10.18	10.43	7.80	2.71E-05	6.04E-04
8161056	7094	*talin 1*	*TLN1*	11.94	12.12	9.49	2.72E-05	6.04E-04
8164008	81571	*non-protein coding RNA 287*	*NCRNA00287*	6.49	6.34	8.63	2.72E-05	6.04E-04
8128052	8732	*RNA guanylyltransferase and 5'-phosphatase*	*RNGTT*	7.72	7.55	9.84	2.72E-05	6.04E-04
7974380	5706	*proteasome (prosome, macropain) 26S subunit, ATPase, 6*	*PSMC6*	8.24	8.41	9.78	2.73E-05	6.06E-04
7962689	7421	*vitamin D (1,25- dihydroxyvitamin D3) receptor*	*VDR*	8.77	9.78	6.08	2.73E-05	6.06E-04
7955887	3222	*homeobox C5*	*HOXC5*	5.53	7.85	5.37	2.74E-05	6.07E-04
8004671	23135	*lysine (K)-specific demethylase 6B*	*KDM6B*	9.07	9.20	7.11	2.75E-05	6.09E-04
8051001	10669	*cell growth regulator with EF-hand domain 1*	*CGREF1*	7.22	7.15	6.37	2.75E-05	6.09E-04
8017621	11232	*polymerase (DNA directed), gamma 2, accessory subunit*	*POLG2*	6.02	5.94	7.94	2.76E-05	6.09E-04
8078008	27258		*LSM3*	10.46	10.62	11.47	2.75E-05	6.09E-04
7968154	51761	*ATPase, aminophospholipid transporter, class I, type 8A, member 2*	*ATP8A2*	5.74	5.89	7.70	2.76E-05	6.09E-04
8080804	200845	*potassium channel tetramerisation domain containing 6*	*KCTD6*	7.18	7.14	8.72	2.76E-05	6.09E-04
8019437	284001	*coiled-coil domain containing 57*	*CCDC57*	7.48	7.44	6.82	2.76E-05	6.09E-04
8070182	1827	*regulator of calcineurin 1*	*RCAN1*	8.66	9.36	7.81	2.77E-05	6.10E-04

7948896	9304, 23642	*small nucleolar RNA, C/D box 22 | small nucleolar RNA host gene 1 (non-protein coding)*	*SNORD22| SNHG1*	6.32	6.32	9.20	2.77E-05	6.10E-04
8124604				5.35	5.55	8.90	2.77E-05	6.10E-04
8053949	55683		*KIAA1310*	9.41	8.98	8.13	2.78E-05	6.12E-04
8105663	57486	*neurolysin (metallopeptidase M3 family)*	*NLN*	7.60	8.03	10.62	2.79E-05	6.12E-04
8145027	64760	*family with sequence similarity 160, member B2*	*FAM160B2*	10.47	10.28	7.57	2.79E-05	6.12E-04
8059244	79586	*chondroitin polymerizing factor*	*CHPF*	10.75	10.87	9.05	2.79E-05	6.12E-04
8110450	3182	*heterogeneous nuclear ribonucleoprotein A/B*	*HNRNPAB*	11.17	11.60	13.13	2.80E-05	6.13E-04
8113542	7905	*receptor accessory protein 5*	*REEP5*	11.91	11.93	11.36	2.80E-05	6.13E-04
8102468	8492	*protease, serine, 12 (neurotrypsin, motopsin)*	*PRSS12*	11.62	9.30	7.78	2.81E-05	6.15E-04
8179326	50854	*chromosome 6 open reading frame 48*	*C6orf48*	11.31	11.78	10.43	2.81E-05	6.15E-04
7901720	5563	*protein kinase, AMP-activated, alpha 2 catalytic subunit*	*PRKAA2*	6.62	6.79	9.78	2.84E-05	6.21E-04
7938669	10944	*chromosome 11 open reading frame 58*	*C11orf58*	10.07	10.02	11.27	2.86E-05	6.24E-04
8117045	221662	*RNA binding motif protein 24*	*RBM24*	7.96	7.22	6.00	2.87E-05	6.26E-04
8162531				13.37	13.43	11.02	2.87E-05	6.26E-04
8179351	629	*complement factor B*	*CFB*	8.53	7.98	6.42	2.88E-05	6.28E-04
8076128	9929	*Josephin domain containing 1*	*JOSD1*	10.46	10.47	8.95	2.89E-05	6.28E-04
8178090	50854	*chromosome 6 open reading frame 48*	*C6orf48*	11.31	11.78	10.43	2.89E-05	6.28E-04
7952805	283174		*LOC28317 4*	6.06	6.15	8.62	2.89E-05	6.28E-04
7945014	1111	*CHK1 checkpoint homolog (S. pombe)*	*CHEK1*	7.81	7.79	10.82	2.90E-05	6.30E-04
8126153	8645	*potassium channel, subfamily K, member 5*	*KCNK5*	6.44	6.54	7.95	2.90E-05	6.30E-04
8029437	5817	*poliovirus receptor*	*PVR*	11.11	10.77	8.84	2.92E-05	6.33E-04
7935002	6726	*signal recognition particle 9kDa*	*SRP9*	10.53	10.66	12.12	2.92E-05	6.34E-04
8125545	3111	*major histocompatibility complex, class II, DO alpha*	*HLA-DOA*	7.37	7.47	8.94	2.93E-05	6.35E-04
7961022	5757	*prothymosin, alpha*	*PTMA*	12.06	12.15	13.32	2.94E-05	6.36E-04
8175121	3547	*immunoglobulin superfamily, member 1*	*IGSF1*	5.84	5.94	7.77	2.95E-05	6.38E-04
8053467	6439	*surfactant protein B*	*SFTPB*	6.40	6.40	5.98	2.95E-05	6.38E-04

8042558	79998	*ankyrin repeat domain 53*	*ANKRD53*	7.73	7.47	6.55	2.96E-05	6.39E-04
7981919	6638, 8926, 347746, 91380	*small nuclear ribonucleoprotein polypeptide N | SNRPN upstream reading frame | paternally expressed transcript PAR-SN | small nucleolar RNA, C/D box 107*	*SNRPN|SNURF|PAR-SN|SNORD107*	8.96	8.99	11.68	2.98E-05	6.42E-04
8119109	221477	*chromosome 6 open reading frame 89*	*C6orf89*	10.05	9.90	7.70	2.99E-05	6.44E-04
7948973	117245	*HRAS-like suppressor family, member 5*	*HRASLS5*	6.89	6.91	9.36	3.00E-05	6.47E-04
7994874	64319	*fibrosin*	*FBRS*	9.83	9.72	7.15	3.00E-05	6.47E-04
8155192	152007	*GLI pathogenesis-related 2*	*GLIPR2*	10.53	11.23	9.52	3.01E-05	6.47E-04
8034416	6134	*ribosomal protein L10*	*RPL10*	13.04	12.99	12.34	3.01E-05	6.48E-04
7980233	5228	*placental growth factor*	*PGF*	10.54	7.75	6.91	3.03E-05	6.50E-04
8112327	1163	*CDC28 protein kinase regulatory subunit 1B*	*CKS1B*	9.85	10.37	12.85	3.03E-05	6.51E-04
7915516	112950	*mediator complex subunit 8*	*MED8*	8.73	8.74	7.91	3.03E-05	6.51E-04
8171747	1964	*eukaryotic translation initiation factor 1A, X-linked*	*EIF1AX*	8.94	9.63	11.09	3.05E-05	6.53E-04
7908758	149345	*shisa homolog 4 (Xenopus laevis)*	*SHISA4*	10.45	10.19	7.81	3.05E-05	6.53E-04
8168749	27286	*sushi-repeat-containing protein, X-linked 2*	*SRPX2*	9.36	10.14	5.97	3.06E-05	6.53E-04
8031825	79818, 730051, 84914	*zinc finger protein 552 | zinc finger protein 814 | zinc finger protein 587*	*ZNF552|ZNF814|ZNF587*	7.58	7.82	10.83	3.05E-05	6.53E-04
7926807	23590	*prenyl (decaprenyl) diphosphate synthase, subunit 1*	*PDSS1*	7.88	8.14	10.29	3.06E-05	6.54E-04
8089544	151887	*coiled-coil domain containing 80*	*CCDC80*	11.81	12.55	7.11	3.06E-05	6.54E-04
7941537	55690	*phosphofurin acidic cluster sorting protein 1*	*PACS1*	11.45	11.43	8.79	3.10E-05	6.62E-04
7970864	10808	*heat shock 105kDa/110kDa protein 1*	*HSPH1*	8.15	8.13	10.83	3.11E-05	6.63E-04
8138647	136895	*chromosome 7 open reading frame 31*	*C7orf31*	8.11	7.39	6.70	3.12E-05	6.65E-04
8160238	11168	*PC4 and SFRS1 interacting protein 1*	*PSIP1*	7.74	7.56	10.62	3.12E-05	6.65E-04
8098328	51809	*UDP-N-acetyl-alpha-D-galactosamine:polypeptide N-acetylgalactosaminyltransferase 7 (GalNAc-T7)*	*GALNT7*	7.15	6.77	9.76	3.13E-05	6.65E-04
8098463				4.86	4.91	6.19	3.13E-05	6.65E-04
8055137				5.68	5.69	5.04	3.14E-05	6.68E-04

7950534	7481	*wingless-type MMTV integration site family, member 11*	*WNT11*	8.12	7.28	6.86	3.15E-05	6.68E-04
8102532	8654	*phosphodiesterase 5A, cGMP-specific*	*PDE5A*	10.24	6.94	8.38	3.16E-05	6.70E-04
7982377	26585	*gremlin 1*	*GREM1*	13.12	12.91	8.20	3.16E-05	6.70E-04
8151917	51001	*MTERF domain containing 1*	*MTERFD1*	6.70	6.83	8.07	3.16E-05	6.70E-04
8018439	3021	*H3 histone, family 3B (H3.3B)*	*H3F3B*	10.47	10.75	11.59	3.17E-05	6.71E-04
8018169	124599	*CD300 molecule-like family member b*	*CD300LB*	6.47	6.40	6.05	3.17E-05	6.71E-04
7986789	57194	*ATPase, class V, type 10A*	*ATP10A*	8.06	8.93	6.84	3.17E-05	6.71E-04
7938687	4925	*nucleobindin 2*	*NUCB2*	9.18	9.23	7.45	3.18E-05	6.72E-04
8166784	7102	*tetraspanin 7*	*TSPAN7*	6.06	5.96	9.34	3.20E-05	6.75E-04
7934690	219654	*zinc finger, CCHC domain containing 24*	*ZCCHC24*	10.43	10.27	8.07	3.20E-05	6.75E-04
7967456	196383	*Rab interacting lysosomal protein-like 2*	*RILPL2*	9.71	8.81	7.53	3.21E-05	6.76E-04
8126750	59084	*ectonucleotide pyrophosphatase/phosphodiesterase 5 (putative)*	*ENPP5*	5.54	6.05	8.37	3.21E-05	6.77E-04
8060418	140885	*signal-regulatory protein alpha*	*SIRPA*	9.39	9.64	7.76	3.23E-05	6.79E-04
7900201	51118	*UTP11-like, U3 small nucleolar ribonucleoprotein, (yeast)*	*UTP11L*	8.33	8.66	9.94	3.23E-05	6.80E-04
7961626	6579	*solute carrier organic anion transporter family, member 1A2*	*SLCO1A2*	5.03	5.01	5.88	3.25E-05	6.82E-04
8007454	8153	*Rho family GTPase 2*	*RND2*	8.08	7.74	11.54	3.24E-05	6.82E-04
7906930	83540		*NUF2*	5.66	6.03	8.84	3.25E-05	6.82E-04
8068353	6526, 64968	*solute carrier family 5 (sodium/myo-inositol cotransporter), member 3 | mitochondrial ribosomal protein S6*	*SLC5A3|MRPS6*	8.71	8.65	10.02	3.26E-05	6.83E-04
8081810	2596	*growth associated protein 43*	*GAP43*	6.36	6.80	9.59	3.27E-05	6.86E-04
7957793	64431	*ARP6 actin-related protein 6 homolog (yeast)*	*ACTR6*	6.09	6.17	7.19	3.29E-05	6.89E-04
8041570	130733	*transmembrane protein 178*	*TMEM178*	8.43	8.38	8.91	3.30E-05	6.89E-04
8123407	4301, 100505818	*myeloid/lymphoid or mixed-lineage leukemia (trithorax homolog, Drosophila); translocated to, 4 | afadin-like*	*MLLT4|LOC100505818*	6.79	6.88	9.74	3.30E-05	6.91E-04
8119080	6428	*serine/arginine-rich splicing factor 3*	*SRSF3*	9.96	10.21	12.22	3.32E-05	6.93E-04
8165808	7499	*Xg blood group*	*XG*	9.84	9.59	6.46	3.32E-05	6.93E-04

8078248	3146, 140690	*high-mobility group box 1 | CCCTC-binding factor (zinc finger protein)-like*	*HMGB1|CTCFL*	10.08	10.31	11.87	3.32E-05	6.93E-04
8056327	2888	*growth factor receptor-bound protein 14*	*GRB14*	5.42	5.49	7.36	3.33E-05	6.94E-04
7962590	79657	*RNA polymerase II associated protein 3*	*RPAP3*	6.67	6.55	8.41	3.34E-05	6.96E-04
8109830	54908	*coiled-coil domain containing 99*	*CCDC99*	7.86	9.15	10.83	3.35E-05	6.97E-04
8162086	23287	*ATP/GTP binding protein 1*	*AGTPBP1*	6.43	6.98	9.71	3.35E-05	6.97E-04
8017555	2081	*endoplasmic reticulum to nucleus signaling 1*	*ERN1*	7.78	8.03	7.30	3.37E-05	7.00E-04
8005847	7126	*tumor necrosis factor, alpha-induced protein 1 (endothelial)*	*TNFAIP1*	11.28	10.72	8.94	3.37E-05	7.00E-04
8122198				9.86	10.32	8.53	3.37E-05	7.00E-04
7921916	8490	*regulator of G-protein signaling 5*	*RGS5*	5.73	5.53	9.53	3.38E-05	7.00E-04
7912852	1964	*eukaryotic translation initiation factor 1A, X-linked*	*EIF1AX*	9.11	9.91	11.47	3.41E-05	7.06E-04
7899534	2035	*erythrocyte membrane protein band 4.1 (elliptocytosis 1, RH-linked)*	*EPB41*	7.06	6.93	9.50	3.43E-05	7.09E-04
8025877	64748	*lipid phosphate phosphatase-related protein type 2*	*LPPR2*	10.97	11.04	7.86	3.43E-05	7.09E-04
8034099	406976	*microRNA 199a-1*	*MIR199A1*	7.90	7.69	5.12	3.44E-05	7.12E-04
7930894	2869	*G protein-coupled receptor kinase 5*	*GRK5*	10.91	9.29	6.82	3.45E-05	7.12E-04
8109490	6444	*sarcoglycan, delta (35kDa dystrophin-associated glycoprotein)*	*SGCD*	9.27	9.84	5.66	3.45E-05	7.13E-04
8104449	22948	*chaperonin containing TCP1, subunit 5 (epsilon)*	*CCT5*	9.76	10.26	12.47	3.45E-05	7.13E-04
7984540	9493	*kinesin family member 23*	*KIF23*	7.25	7.58	10.50	3.47E-05	7.14E-04
7938485	9645	*microtubule associated monoxygenase, calponin and LIM domain containing 2*	*MICAL2*	9.04	10.02	6.18	3.47E-05	7.14E-04
7976451	57718	*protein phosphatase 4, regulatory subunit 4*	*PPP4R4*	6.30	5.77	7.72	3.47E-05	7.14E-04
7992956	84662	*GLIS family zinc finger 2*	*GLIS2*	10.95	10.42	8.68	3.46E-05	7.14E-04
7925672	93474, 57116	*zinc finger protein 670 | zinc finger protein 695*	*ZNF670|ZNF695*	5.55	5.52	7.79	3.47E-05	7.14E-04
8139244	55744	*chromosome 7 open reading frame 44*	*C7orf44*	9.93	9.94	11.97	3.48E-05	7.15E-04
7952361	7753	*zinc finger protein 202*	*ZNF202*	7.12	7.41	8.52	3.49E-05	7.16E-04
8011626	7326	*ubiquitin-conjugating enzyme E2G 1 (UBC7 homolog, yeast)*	*UBE2G1*	11.36	11.53	12.56	3.50E-05	7.18E-04

8032410	126308	*MOB1, Mps One Binder kinase activator-like 2A (yeast)*	*MOBKL2A*	11.15	11.30	8.59	3.51E-05	7.19E-04
7927173	220992	*zinc finger protein 485*	*ZNF485*	6.46	6.53	8.10	3.51E-05	7.19E-04
8170298	389898	*ubiquitin-conjugating enzyme E2N-like*	*UBE2NL*	7.40	7.63	8.56	3.52E-05	7.20E-04
7910416	9816		*URB2*	7.34	7.68	9.30	3.52E-05	7.21E-04
8073816	400931		*LOC400931*	9.11	8.99	6.98	3.53E-05	7.21E-04
7986132	4122	*mannosidase, alpha, class 2A, member 2*	*MAN2A2*	9.17	8.80	7.16	3.54E-05	7.21E-04
7973618	10379	*interferon regulatory factor 9*	*IRF9*	10.21	10.12	7.87	3.54E-05	7.21E-04
7990810	10588	*5,10-methenyltetrahydrofolate synthetase (5-formyltetrahydrofolate cyclo-ligase)*	*MTHFS*	7.96	8.10	9.44	3.54E-05	7.21E-04
8105191	79668	*poly (ADP-ribose) polymerase family, member 8*	*PARP8*	7.48	7.13	9.40	3.54E-05	7.21E-04
8109843	1794, 80005	*dedicator of cytokinesis 2 | dedicator of cytokinesis 5*	*DOCK2|DOCK5*	6.55	6.41	8.02	3.53E-05	7.21E-04
8083075	92370	*acid phosphatase-like 2*	*ACPL2*	6.75	6.79	8.52	3.56E-05	7.25E-04
8144153	54892	*non-SMC condensin II complex, subunit G2*	*NCAPG2*	6.22	6.43	9.59	3.57E-05	7.26E-04
8131709	6671	*Sp4 transcription factor*	*SP4*	7.21	7.09	10.02	3.58E-05	7.28E-04
8070863	728039	*chromosome 21 open reading frame 122*	*C21orf122*	7.36	7.27	6.29	3.58E-05	7.28E-04
8156783	1306	*collagen, type XV, alpha 1*	*COL15A1*	11.16	10.86	6.69	3.60E-05	7.29E-04
7914557	81493	*syncoilin, intermediate filament protein*	*SYNC*	8.47	9.09	6.26	3.59E-05	7.29E-04
8039687	79818	*zinc finger protein 552*	*ZNF552*	5.89	5.78	7.24	3.61E-05	7.31E-04
7917634	164045		*HFM1*	4.80	4.82	5.79	3.61E-05	7.31E-04
7906574	474338	*SUMO1 pseudogene 3*	*SUMO1P3*	5.04	5.00	6.27	3.61E-05	7.31E-04
7914094	10163	*WAS protein family, member 2*	*WASF2*	10.83	10.71	9.13	3.62E-05	7.32E-04
7909027	23114	*neurofascin*	*NFASC*	10.14	9.49	7.12	3.62E-05	7.32E-04
8073733	10762	*nucleoporin 50kDa*	*NUP50*	6.93	6.94	9.12	3.63E-05	7.32E-04
7961208	55110	*mago-nashi homolog B (Drosophila)*	*MAGOHB*	7.91	8.21	9.89	3.63E-05	7.32E-04
7968999	55270, 8803	*nudix (nucleoside diphosphate linked moiety X)-type motif 15 | succinate-CoA ligase, ADP-forming, beta subunit*	*NUDT15|SUCLA2*	6.99	7.25	8.83	3.63E-05	7.33E-04
8023995	10272	*follistatin-like 3 (secreted glycoprotein)*	*FSTL3*	10.21	10.72	7.85	3.64E-05	7.33E-04

8150830	10434	*lysophospholipase I*	*LYPLA1*	9.01	9.19	11.73	3.64E-05	7.33E-04
8030944	147660	*zinc finger protein 578*	*ZNF578*	4.82	4.69	6.72	3.65E-05	7.33E-04
8084947	200933	*F-box protein 45*	*FBXO45*	6.76	7.02	8.95	3.64E-05	7.33E-04
8171879	100508181	*ran-specific GTPase-activating protein-like*	*LOC100508181*	7.42	7.46	9.25	3.65E-05	7.33E-04
8085272	401052		*LOC401052*	7.44	7.24	6.26	3.66E-05	7.35E-04
8127932	9096	*T-box 18*	*TBX18*	7.58	8.97	5.83	3.66E-05	7.35E-04
7899604	51538	*zinc finger, CCHC domain containing 17*	*ZCCHC17*	7.55	8.02	9.40	3.67E-05	7.35E-04
8022803	64762	*family with sequence similarity 59, member A*	*FAM59A*	7.20	7.11	8.53	3.67E-05	7.35E-04
7980051	91748	*chromosome 14 open reading frame 43*	*C14orf43*	9.04	8.98	7.52	3.67E-05	7.35E-04
7921882	25903	*olfactomedin-like 2B*	*OLFML2B*	10.44	9.01	7.26	3.68E-05	7.37E-04
8157761	10783	*NIMA (never in mitosis gene a)-related kinase 6*	*NEK6*	10.46	10.43	8.57	3.69E-05	7.39E-04
7975066	9495	*A kinase (PRKA) anchor protein 5*	*AKAP5*	5.33	5.27	6.41	3.71E-05	7.41E-04
8027241	56242	*zinc finger protein 253*	*ZNF253*	6.94	6.67	10.23	3.71E-05	7.41E-04
8093456	92070	*chromosome 4 open reading frame 42*	*C4orf42*	9.49	9.23	7.99	3.71E-05	7.41E-04
8059838	55355	*Holliday junction recognition protein*	*HJURP*	7.86	8.30	10.79	3.72E-05	7.41E-04
8150204				7.74	8.29	9.55	3.72E-05	7.42E-04
8153550	340371	*nuclear receptor binding protein 2*	*NRBP2*	9.27	9.18	7.28	3.74E-05	7.44E-04
8023063	498	*ATP synthase, H+ transporting, mitochondrial F1 complex, alpha subunit 1, cardiac muscle*	*ATP5A1*	10.07	9.83	11.61	3.75E-05	7.46E-04
7933855	219790	*rhotekin 2*	*RTKN2*	5.44	5.33	8.45	3.75E-05	7.46E-04
8056408	2591	*UDP-N-acetyl-alpha-D-galactosamine:polypeptide N-acetylgalactosaminyltransferase 3 (GalNAc-T3)*	*GALNT3*	5.89	5.26	8.98	3.75E-05	7.46E-04
8002029	29800	*zinc finger, DHHC-type containing 1*	*ZDHHC1*	10.31	10.03	7.37	3.76E-05	7.48E-04
8027402	898	*cyclin E1*	*CCNE1*	7.50	7.46	9.99	3.78E-05	7.51E-04
8137404	54480, 768213	*chondroitin polymerizing factor 2 | microRNA 671*	*CHPF2|MIR671*	10.34	10.19	8.16	3.78E-05	7.51E-04
7899153	83442, 91544	*SH3 domain binding glutamic acid-rich protein like 3 | UBX domain protein 11*	*SH3BGRL3|UBXN11*	9.85	10.11	8.72	3.80E-05	7.53E-04
7930921	9531	*BCL2-associated athanogene 3*	*BAG3*	9.95	9.97	8.05	3.82E-05	7.56E-04

8040490	114818	*kelch-like 29 (Drosophila)*	*KLHL29*	7.29	7.19	6.46	3.84E-05	7.60E-04
8064859	128674	*prokineticin receptor 2*	*PROKR2*	6.08	6.06	7.00	3.85E-05	7.60E-04
7984330	55055, 197021	*Zwilch, kinetochore associated, homolog (Drosophila) | lactase-like*	*ZWILCH|L CTL*	7.44	7.74	10.80	3.84E-05	7.60E-04
8010287	114897	*C1q and tumor necrosis factor related protein 1*	*C1QTNF1*	9.90	8.77	7.35	3.87E-05	7.65E-04
8000329	79728	*partner and localizer of BRCA2*	*PALB2*	7.48	7.51	10.29	3.87E-05	7.65E-04
7975354	400224		*UPF0639*	6.50	6.41	6.00	3.88E-05	7.65E-04
7979813	677	*zinc finger protein 36, C3H type-like 1*	*ZFP36L1*	11.75	12.01	10.30	3.89E-05	7.65E-04
7939524	2132	*exostosin 2*	*EXT2*	11.55	11.47	10.38	3.89E-05	7.65E-04
7947138	2188	*Fanconi anemia, complementation group F*	*FANCF*	7.29	7.41	8.85	3.89E-05	7.65E-04
7982102	2558	*gamma-aminobutyric acid (GABA) A receptor, alpha 5*	*GABRA5*	6.63	7.29	8.52	3.90E-05	7.65E-04
8077899	5468	*peroxisome proliferator-activated receptor gamma*	*PPARG*	9.03	6.66	5.98	3.90E-05	7.65E-04
7910099	6726	*signal recognition particle 9kDa*	*SRP9*	9.87	9.95	11.46	3.90E-05	7.65E-04
8162744	7464	*coronin, actin binding protein, 2A*	*CORO2A*	7.19	6.83	8.67	3.90E-05	7.65E-04
7938183	7762	*zinc finger protein 215*	*ZNF215*	6.10	6.45	8.77	3.89E-05	7.65E-04
8150877	26795	*small nucleolar RNA, C/D box 54*	*SNORD54*	6.25	6.44	7.75	3.88E-05	7.65E-04
8084895	200958	*mucin 20, cell surface associated*	*MUC20*	7.26	7.16	6.62	3.89E-05	7.65E-04
8097679	11157		*LSM6*	7.78	7.73	9.51	3.91E-05	7.66E-04
7935403	84986	*Rho GTPase activating protein 19*	*ARHGAP1 9*	6.75	6.80	9.88	3.92E-05	7.68E-04
8007071	990	*cell division cycle 6 homolog (S. cerevisiae)*	*CDC6*	6.20	6.78	10.35	3.93E-05	7.68E-04
8032465	126306	*junctional sarcoplasmic reticulum protein 1*	*JSRP1*	8.96	8.97	8.18	3.93E-05	7.69E-04
8098006	2743	*glycine receptor, beta*	*GLRB*	6.23	6.69	5.45	3.94E-05	7.69E-04
8051589	64225	*atlastin GTPase 2*	*ATL2*	6.85	7.03	9.49	3.94E-05	7.69E-04
7967386	10198	*M-phase phosphoprotein 9*	*MPHOSP H9*	7.81	7.71	9.60	3.95E-05	7.70E-04
7914326	79570	*Na+/K+ transporting ATPase interacting 1*	*NKAIN1*	6.97	7.18	9.51	3.96E-05	7.70E-04
7943369	83935	*transmembrane protein 133*	*TMEM133*	7.25	5.78	9.63	3.95E-05	7.70E-04
8031047	91663	*myeloid-associated differentiation marker*	*MYADM*	13.19	13.42	11.68	3.95E-05	7.70E-04
7994981	93129	*ORAI calcium release-activated calcium modulator 3*	*ORAI3*	10.86	10.34	8.60	3.96E-05	7.71E-04

8130952	5689	*proteasome (prosome, macropain) subunit, beta type, 1*	*PSMB1*	9.08	9.21	10.34	3.97E-05	7.72E-04
8059376	5270	*serpin peptidase inhibitor, clade E (nexin, plasminogen activator inhibitor type 1), member 2*	*SERPINE2*	11.22	12.79	9.84	3.99E-05	7.74E-04
7971486	80183	*chromosome 13 open reading frame 18*	*C13orf18*	5.77	6.10	7.09	3.99E-05	7.74E-04
8063283	1434	*CSE1 chromosome segregation 1-like (yeast)*	*CSE1L*	9.91	9.88	12.27	3.99E-05	7.75E-04
8049689	57140	*arginyl aminopeptidase (aminopeptidase B)-like 1*	*RNPEPL1*	10.15	10.07	7.60	4.01E-05	7.77E-04
8132214	11328	*FK506 binding protein 9, 63 kDa*	*FKBP9*	9.95	10.07	6.35	4.02E-05	7.78E-04
8177038	6736	*sex determining region Y*	*SRY*	5.51	5.22	6.41	4.02E-05	7.78E-04
8123760	285780	*LY86 antisense RNA (non-protein coding)*	*LY86-AS*	7.56	7.67	6.94	4.02E-05	7.78E-04
8025179	140467	*zinc finger protein 358*	*ZNF358*	10.94	10.86	9.35	4.05E-05	7.82E-04
8016898	6426	*serine/arginine-rich splicing factor 1*	*SRSF1*	10.22	10.15	12.23	4.05E-05	7.83E-04
7965094	144455	*E2F transcription factor 7*	*E2F7*	6.97	7.26	9.27	4.06E-05	7.84E-04
7922406	26770	*small nucleolar RNA, C/D box 79*	*SNORD79*	7.62	7.79	9.90	4.07E-05	7.85E-04
7940147	374393	*family with sequence similarity 111, member B*	*FAM111B*	5.80	6.05	9.18	4.07E-05	7.85E-04
8055645	5000	*origin recognition complex, subunit 4*	*ORC4*	7.28	7.24	9.36	4.08E-05	7.86E-04
7909642	79805	*vasohibin 2*	*VASH2*	6.84	6.77	10.11	4.09E-05	7.86E-04
8080198	132160	*protein phosphatase, Mg2+/Mn2+ dependent, 1M*	*PPM1M*	9.00	8.81	6.82	4.08E-05	7.86E-04
7908409	5997	*regulator of G-protein signaling 2, 24kDa*	*RGS2*	9.24	6.59	8.84	4.11E-05	7.91E-04
7926299	51182	*heat shock 70kDa protein 14*	*HSPA14*	7.08	7.32	8.72	4.12E-05	7.92E-04
7909789	7042	*transforming growth factor, beta 2*	*TGFB2*	9.35	7.71	6.27	4.13E-05	7.93E-04
8046488	83879	*cell division cycle associated 7*	*CDCA7*	6.81	6.67	10.14	4.13E-05	7.93E-04
8178977	6892	*TAP binding protein (tapasin)*	*TAPBP*	12.08	11.57	9.60	4.16E-05	7.97E-04
7910427	2590	*UDP-N-acetyl-alpha-D-galactosamine:polypeptide N-acetylgalactosaminyltransferase 2 (GalNAc-T2)*	*GALNT2*	10.87	10.71	9.78	4.21E-05	8.06E-04
8031931				8.99	9.52	7.07	4.21E-05	8.06E-04
8101945	3015	*H2A histone family, member Z*	*H2AFZ*	9.95	10.28	12.01	4.25E-05	8.13E-04
8096635	4790	*nuclear factor of kappa light polypeptide gene enhancer in B-cells 1*	*NFKB1*	9.79	9.34	7.18	4.25E-05	8.13E-04
8168447	51260	*chromosome X open reading frame 26*	*CXorf26*	7.08	7.16	8.92	4.26E-05	8.15E-04

7948910	9303, 23642	*small nucleolar RNA, C/D box 25 | small nucleolar RNA host gene 1 (non-protein coding)*	*SNORD25|SNHG1*	5.94	5.36	9.32	4.27E-05	8.15E-04
7968232	26771	*small nucleolar RNA, C/D box 102*	*SNORD102*	5.26	5.27	5.98	4.29E-05	8.19E-04
7926345	10557, 221060	*ribonuclease P/MRP 38kDa subunit | chromosome 10 open reading frame 111*	*RPP38|C10orf111*	8.30	8.49	9.32	4.30E-05	8.20E-04
8142663	4698	*NADH dehydrogenase (ubiquinone) 1 alpha subcomplex, 5, 13kDa*	*NDUFA5*	7.38	7.35	8.76	4.31E-05	8.21E-04
8035803	7562	*zinc finger protein 708*	*ZNF708*	7.07	6.94	9.67	4.31E-05	8.21E-04
8105908	4950, 647859	*occludin | occludin pseudogene*	*OCLN|LOC647859*	6.05	5.88	11.39	4.31E-05	8.21E-04
7908072	3918	*laminin, gamma 2*	*LAMC2*	6.11	6.38	8.22	4.32E-05	8.22E-04
7964347	23306	*transmembrane protein 194A*	*TMEM194A*	7.45	7.59	10.57	4.32E-05	8.22E-04
8120833	83699	*SH3 domain binding glutamic acid-rich protein like 2*	*SH3BGRL2*	7.53	6.71	9.99	4.33E-05	8.23E-04
8021101	115106	*HAUS augmin-like complex, subunit 1*	*HAUS1*	8.96	8.78	10.72	4.34E-05	8.23E-04
8092067	200916	*ribosomal protein L22-like 1*	*RPL22L1*	6.47	6.34	9.35	4.34E-05	8.23E-04
7945688	723961, 3481, 3630		*INS-IGF2*	8.10	7.67	6.99	4.35E-05	8.24E-04
8016239	9842	*pleckstrin homology domain containing, family M (with RUN domain) member 1*	*PLEKHM1*	8.85	8.82	6.58	4.37E-05	8.29E-04
7912956	55920	*regulator of chromosome condensation 2*	*RCC2*	10.46	10.73	11.98	4.38E-05	8.30E-04
8174568	340596	*lipoma HMGIC fusion partner-like 1*	*LHFPL1*	5.52	5.72	6.45	4.38E-05	8.30E-04
8040698	54978, 1058	*chromosome 2 open reading frame 18 | centromere protein A*	*C2orf18|CENPA*	11.20	10.75	8.74	4.40E-05	8.32E-04
8136576				6.23	6.24	5.84	4.40E-05	8.32E-04
7958724	100131138		*LOC100131138*	7.62	7.79	9.52	4.42E-05	8.34E-04
8152703	114907	*F-box protein 32*	*FBXO32*	8.29	10.14	5.92	4.42E-05	8.35E-04
8169969	84295	*PHD finger protein 6*	*PHF6*	7.49	7.32	9.49	4.43E-05	8.35E-04
8126324	5225	*progastricsin (pepsinogen C)*	*PGC*	6.09	6.31	5.87	4.44E-05	8.37E-04
7925691	7678	*zinc finger protein 124*	*ZNF124*	6.91	6.71	8.56	4.45E-05	8.38E-04

7981335	3320	*heat shock protein 90kDa alpha (cytosolic), class A member 1*	*HSP90AA1*	11.51	11.42	12.75	4.45E-05	8.39E-04
7967620	57647	*DEAH (Asp-Glu-Ala-His) box polypeptide 37*	*DHX37*	12.81	12.81	11.90	4.46E-05	8.39E-04
8100541	3490	*insulin-like growth factor binding protein 7*	*IGFBP7*	13.41	12.47	9.03	4.46E-05	8.39E-04
8036436	84775	*zinc finger protein 607*	*ZNF607*	6.58	6.45	8.25	4.47E-05	8.39E-04
7910792	6262	*ryanodine receptor 2 (cardiac)*	*RYR2*	5.68	5.65	7.52	4.49E-05	8.42E-04
7946401	6764	*suppression of tumorigenicity 5*	*ST5*	10.28	10.24	7.98	4.51E-05	8.45E-04
8175023	51114	*zinc finger, DHHC-type containing 9*	*ZDHHC9*	12.23	12.10	10.95	4.51E-05	8.45E-04
7959604	57696	*DEAD (Asp-Glu-Ala-Asp) box polypeptide 55*	*DDX55*	6.99	7.10	8.45	4.51E-05	8.45E-04
7992867	283876		*FLJ39639*	7.30	7.18	6.28	4.51E-05	8.45E-04
8029530	348, 3159	*apolipoprotein E | high mobility group AT-hook 1*	*APOE|HMGA1*	7.77	7.88	9.26	4.50E-05	8.45E-04
8166511	5165	*pyruvate dehydrogenase kinase, isozyme 3*	*PDK3*	7.40	7.39	9.82	4.53E-05	8.47E-04
8017810	5718	*proteasome (prosome, macropain) 26S subunit, non-ATPase, 12*	*PSMD12*	7.89	8.00	9.09	4.54E-05	8.49E-04
8107934	441108	*chromosome 5 open reading frame 56*	*C5orf56*	9.10	9.03	7.52	4.54E-05	8.49E-04
8104825	55299, 5810	*BRX1, biogenesis of ribosomes, homolog (S. cerevisiae) | RAD1 homolog (S. pombe)*	*BRIX1|RAD1*	7.67	7.79	9.81	4.55E-05	8.49E-04
7920984	7203	*chaperonin containing TCP1, subunit 3 (gamma)*	*CCT3*	10.41	10.44	11.81	4.58E-05	8.54E-04
7960771	283316	*CD163 molecule-like 1*	*CD163L1*	6.65	6.78	6.18	4.58E-05	8.54E-04
8103922	836	*caspase 3, apoptosis-related cysteine peptidase*	*CASP3*	7.69	7.84	9.56	4.60E-05	8.57E-04
8043848	51263	*mitochondrial ribosomal protein L30*	*MRPL30*	9.63	9.66	10.96	4.60E-05	8.57E-04
7965884	5053	*phenylalanine hydroxylase*	*PAH*	5.87	6.08	7.81	4.61E-05	8.58E-04
8142554	10157	*aminoadipate-semialdehyde synthase*	*AASS*	7.78	7.33	12.60	4.64E-05	8.64E-04
7939595	1408	*cryptochrome 2 (photolyase-like)*	*CRY2*	9.02	8.96	8.03	4.65E-05	8.64E-04
7903022	6083	*small nucleolar RNA, C/D box 21*	*SNORD21*	6.25	6.00	7.98	4.66E-05	8.64E-04
8072817	11135	*CDC42 effector protein (Rho GTPase binding) 1*	*CDC42EP1*	11.46	11.64	8.27	4.65E-05	8.64E-04
8011774	23125	*calmodulin binding transcription activator 2*	*CAMTA2*	9.71	9.84	7.50	4.66E-05	8.64E-04
8104492	83853	*ropporin 1-like*	*ROPN1L*	9.58	7.82	6.95	4.66E-05	8.64E-04
8132557	165	*AE binding protein 1*	*AEBP1*	12.23	11.38	8.49	4.67E-05	8.64E-04

7982757	57082	*cancer susceptibility candidate 5*	*CASC5*	6.97	7.17	9.69	4.67E-05	8.64E-04
8032312	148229	*ATPase, aminophospholipid transporter, class I, type 8B, member 3*	*ATP8B3*	7.62	7.56	7.12	4.67E-05	8.65E-04
8017675	440456, 9842, 100291232	*pleckstrin homology domain containing, family M (with RUN domain) member 1 pseudogene | putative pleckstrin homology domain-containing family M member 1P-like*	*PLEKHM1P|LOC100291232*	10.21	10.09	8.43	4.68E-05	8.65E-04
8112767	6902	*tubulin folding cofactor A*	*TBCA*	9.76	9.90	10.76	4.69E-05	8.67E-04
8004506	652965	*small nucleolar RNA, H/ACA box 48*	*SNORA48*	11.07	11.36	10.07	4.69E-05	8.67E-04
8081036	253559	*cell adhesion molecule 2*	*CADM2*	5.66	5.43	7.86	4.69E-05	8.67E-04
8091485	6478	*seven in absentia homolog 2 (Drosophila)*	*SIAH2*	9.51	9.87	8.78	4.71E-05	8.68E-04
8132523	51619	*ubiquitin-conjugating enzyme E2D 4 (putative)*	*UBE2D4*	11.32	11.18	9.37	4.71E-05	8.68E-04
8012197	57048	*phospholipid scramblase 3*	*PLSCR3*	11.47	11.43	8.97	4.71E-05	8.68E-04
8130505	7430	*ezrin*	*EZR*	8.66	9.25	11.12	4.73E-05	8.70E-04
8002987	56942, 55839	*chromosome 16 open reading frame 61 | centromere protein N*	*C16orf61| CENPN*	10.53	11.27	11.68	4.73E-05	8.70E-04
7951485	54733	*solute carrier family 35, member F2*	*SLC35F2*	6.77	7.94	9.69	4.74E-05	8.72E-04
7979351	100129075	*chromosome 14 open reading frame 33*	*C14orf33*	7.32	7.20	8.33	4.75E-05	8.72E-04
8063410	84612	*par-6 partitioning defective 6 homolog beta (C. elegans)*	*PARD6B*	6.65	6.76	9.97	4.76E-05	8.74E-04
8055992	130399	*activin A receptor, type IC*	*ACVR1C*	6.33	6.48	7.01	4.76E-05	8.74E-04
8155754	256691	*MAM domain containing 2*	*MAMDC2*	7.65	6.37	9.24	4.76E-05	8.74E-04
8036763	9149	*dual-specificity tyrosine-(Y)-phosphorylation regulated kinase 1B*	*DYRK1B*	9.33	9.33	7.92	4.79E-05	8.77E-04
8043036	1720	*dihydrofolate reductase pseudogene*	*LOC1720*	8.87	8.84	11.02	4.79E-05	8.78E-04
8154692	7010		*TEK*	5.92	6.74	9.77	4.80E-05	8.79E-04
7981980	100033428	*small nucleolar RNA, C/D box 116-16*	*SNORD116-16*	5.07	5.21	6.63	4.81E-05	8.80E-04
8036284	1346	*cytochrome c oxidase subunit VIIa polypeptide 1 (muscle)*	*COX7A1*	8.65	8.43	6.42	4.82E-05	8.80E-04
8108995	117156	*secretoglobin, family 3A, member 2*	*SCGB3A2*	5.74	5.90	8.69	4.82E-05	8.80E-04

8108757	56097, 56105, 56106, 5098, 56104, 56098, 56099, 56114, 56113, 56112, 56103, 56108, 56101, 26025, 56110, 56109, 56107, 56102, 56100, 9708, 56111, 8641	*protocadherin gamma subfamily C, 5 | protocadherin gamma subfamily A, 11 | protocadherin gamma subfamily A, 10 | protocadherin gamma subfamily C, 3 | protocadherin gamma subfamily B, 1 | protocadherin gamma subfamily C, 4 | protocadherin gamma subfamily B, 7 | protocadherin gamma subfamily A, 1 | protocadherin gamma subfamily A, 2 | protocadherin gamma subfamily A, 3 | protocadherin gamma subfamily B, 2 | protocadherin gamma subfamily A, 7 | protocadherin gamma subfamily B, 5 | protocadherin gamma subfamily A, 12 | protocadherin gamma subfamily A, 5 | protocadherin gamma subfamily A, 6 | protocadherin gamma subfamily A, 9 | protocadherin gamma subfamily B, 3 | protocadherin gamma subfamily B, 6 | protocadherin gamma subfamily A, 8 | protocadherin gamma subfamily A, 4 | protocadherin gamma subfamily B, 4*	*PCDHGC5|PCDHGA11|PCDHGA10|PCDHGC3|PCDHGB1|PCDHGC4|PCDHGB7|PCDHGA2|PCDHGA3|PCDHGB2|PCDHGA7|PCDHGB5|PCDHGA12|PCDHGA5|PCDHGA6|PCDHGA9|PCDHGB3|PCDHGB6|PCDHGA8|PCDHGA4|PCDHGB4*	8.26	7.96	7.18	4.82E-05	8.80E-04
8092564	6434	*transformer 2 beta homolog (Drosophila)*	*TRA2B*	9.86	10.24	11.87	4.83E-05	8.81E-04
7999834	23204	*ADP-ribosylation factor-like 6 interacting protein 1*	*ARL6IP1*	11.76	11.85	13.11	4.85E-05	8.84E-04
8108403	9782, 724102	*matrin 3 | small nucleolar RNA host gene 4 (non-protein coding)*	*MATR3|SNHG4*	9.04	8.95	10.90	4.85E-05	8.84E-04
7902634	80135	*ribosome production factor 1 homolog (S. cerevisiae)*	*RPF1*	8.35	8.54	9.68	4.88E-05	8.88E-04
8117640	80345	*zinc finger and SCAN domain containing 16*	*ZSCAN16*	5.78	5.77	7.49	4.88E-05	8.88E-04
8089723				6.33	6.39	5.71	4.88E-05	8.88E-04
8058765	2335	*fibronectin 1*	*FN1*	13.66	13.91	11.37	4.90E-05	8.90E-04
8162850	54881	*testis expressed 10*	*TEX10*	8.67	8.82	10.93	4.90E-05	8.90E-04
7901948	79469	*deleted in lymphocytic leukemia 2-like*	*DLEU2L*	4.42	4.53	4.36	4.92E-05	8.94E-04

7898875	6135	*ribosomal protein L11*	*RPL11*	11.46	11.56	11.90	4.94E-05	8.94E-04
8084100	8975	*ubiquitin specific peptidase 13 (isopeptidase T-3)*	*USP13*	7.64	7.70	9.20	4.93E-05	8.94E-04
8105612	10283		*CWC27*	7.20	7.09	9.06	4.93E-05	8.94E-04
7996571	79567	*family with sequence similarity 65, member A*	*FAM65A*	10.60	10.80	7.99	4.95E-05	8.94E-04
7963545	338785	*keratin 79*	*KRT79*	7.07	7.09	6.54	4.95E-05	8.94E-04
7959574	387893	*SET domain containing (lysine methyltransferase) 8*	*SETD8*	10.81	10.58	9.46	4.94E-05	8.94E-04
8167971	407008	*microRNA 223*	*MIR223*	5.45	5.39	5.14	4.94E-05	8.94E-04
8104180	57491, 10016	*aryl-hydrocarbon receptor repressor | programmed cell death 6*	*AHRR|PD CD6*	9.00	8.73	7.65	4.94E-05	8.94E-04
8142405				7.11	7.13	8.27	4.95E-05	8.94E-04
8035517	1311	*cartilage oligomeric matrix protein*	*COMP*	8.68	10.66	6.73	4.96E-05	8.94E-04
8140859	7978	*mitochondrial transcription termination factor*	*MTERF*	6.46	6.31	7.56	4.96E-05	8.95E-04
7940711	374395	*transmembrane protein 179B*	*TMEM179 B*	12.35	12.25	10.39	4.96E-05	8.95E-04
7925130	6894	*TAR (HIV-1) RNA binding protein 1*	*TARBP1*	6.76	7.13	8.26	4.98E-05	8.96E-04
8091941	11235	*programmed cell death 10*	*PDCD10*	6.60	6.94	8.20	4.97E-05	8.96E-04
8148280	6713	*squalene epoxidase*	*SQLE*	9.47	8.74	11.13	4.99E-05	8.97E-04
8002939				6.10	6.29	5.72	4.99E-05	8.97E-04
8149953				5.27	4.83	6.43	4.99E-05	8.97E-04
7940660				7.51	7.17	6.58	5.01E-05	8.99E-04
8115865	91272	*biorientation of chromosomes in cell division 1*	*BOD1*	9.55	9.51	10.80	5.02E-05	9.00E-04
8011850	708	*complement component 1, q subcomponent binding protein*	*C1QBP*	10.22	10.35	11.90	5.02E-05	9.01E-04
8038933	80110	*zinc finger protein 614*	*ZNF614*	6.98	6.87	8.63	5.04E-05	9.03E-04
7954065	9052	*G protein-coupled receptor, family C, group 5, member A*	*GPRC5A*	8.68	8.57	6.42	5.06E-05	9.05E-04
8127743	9324	*high mobility group nucleosomal binding domain 3*	*HMGN3*	7.06	6.69	9.64	5.05E-05	9.05E-04
8126588	57510	*exportin 5*	*XPO5*	8.36	8.74	10.43	5.06E-05	9.05E-04
8091698	6474, 51319	*short stature homeobox 2 | arginine/serine-rich coiled-coil 1*	*SHOX2|RS RC1*	9.65	8.33	6.44	5.06E-05	9.05E-04
8170009	8933	*family with sequence similarity 127, member A*	*FAM127A*	12.79	12.75	11.23	5.08E-05	9.08E-04

8072735	8542	*apolipoprotein L, 1*	*APOL1*	9.48	8.09	6.29	5.09E-05	9.09E-04
7903878	64783	*RNA binding motif protein 15*	*RBM15*	7.66	7.44	9.38	5.11E-05	9.11E-04
8168463	8823	*fibroblast growth factor 16*	*FGF16*	5.61	5.70	7.41	5.12E-05	9.14E-04
7982574	283742	*family with sequence similarity 98, member B*	*FAM98B*	7.12	7.12	8.99	5.14E-05	9.15E-04
8062933	6406	*semenogelin I*	*SEMG1*	5.64	5.66	7.24	5.15E-05	9.17E-04
8017702				5.63	5.65	6.71	5.16E-05	9.18E-04
8060344	57761	*tribbles homolog 3 (Drosophila)*	*TRIB3*	9.91	10.98	6.94	5.17E-05	9.18E-04
8046147	129880	*Bardet-Biedl syndrome 5*	*BBS5*	7.46	7.25	5.91	5.17E-05	9.18E-04
7925413	645745	*metallothionein 1 pseudogene 2*	*MT1P2*	8.54	8.55	7.34	5.17E-05	9.18E-04
7912537	9249	*dehydrogenase/reductase (SDR family) member 3*	*DHRS3*	12.59	12.45	10.24	5.18E-05	9.20E-04
7998381	64788	*lipase maturation factor 1*	*LMF1*	10.52	10.26	7.94	5.21E-05	9.24E-04
7984779	5371	*promyelocytic leukemia*	*PML*	10.01	10.01	7.86	5.22E-05	9.26E-04
8163116	54566	*erythrocyte membrane protein band 4.1 like 4B*	*EPB41L4B*	6.18	6.23	7.50	5.22E-05	9.26E-04
8103485	92345	*nuclear assembly factor 1 homolog (S. cerevisiae)*	*NAF1*	7.91	8.09	9.46	5.25E-05	9.29E-04
8005267	10743	*retinoic acid induced 1*	*RAI1*	9.16	8.99	6.77	5.25E-05	9.30E-04
8141688	8985	*procollagen-lysine, 2-oxoglutarate 5-dioxygenase 3*	*PLOD3*	11.78	12.14	9.21	5.28E-05	9.34E-04
7953699	55810	*forkhead box J2*	*FOXJ2*	10.32	10.38	8.57	5.29E-05	9.36E-04
8124144	7913		*DEK*	9.26	9.18	10.75	5.31E-05	9.38E-04
8153424	642475	*chromosome 8 open reading frame 73*	*C8orf73*	7.80	7.20	5.96	5.31E-05	9.38E-04
8015730	388387		*LOC388387*	7.26	7.33	6.52	5.33E-05	9.40E-04
8021565	23239	*PH domain and leucine rich repeat protein phosphatase 1*	*PHLPP1*	8.12	8.36	9.58	5.33E-05	9.40E-04
7914282	9672	*syndecan 3*	*SDC3*	10.48	10.63	8.03	5.34E-05	9.42E-04
8100298	132299	*OCIA domain containing 2*	*OCIAD2*	7.04	6.82	8.88	5.35E-05	9.42E-04
7980496	145508	*chromosome 14 open reading frame 145*	*C14orf145*	6.35	6.33	7.88	5.37E-05	9.45E-04
7929689	83742, 100270710	*MARVEL domain containing 1 | hypothetical LOC100270710*	*MARVELD1|LOC100270710*	11.33	11.26	9.31	5.40E-05	9.50E-04

7995069	2521	*fused in sarcoma*	*FUS*	9.27	9.89	11.73	5.41E-05	9.51E-04
8131860	10643, 115416	*insulin-like growth factor 2 mRNA binding protein 3 | chromosome 7 open reading frame 30*	*IGF2BP3| C7orf30*	10.47	10.37	11.05	5.42E-05	9.53E-04
8152053	157567	*ankyrin repeat domain 46*	*ANKRD46*	7.45	7.35	8.85	5.43E-05	9.53E-04
8056257	2191	*fibroblast activation protein, alpha*	*FAP*	9.31	9.76	5.02	5.45E-05	9.55E-04
8160040	5789	*protein tyrosine phosphatase, receptor type, D*	*PTPRD*	6.52	6.23	8.98	5.45E-05	9.55E-04
7981290	7453	*tryptophanyl-tRNA synthetase*	*WARS*	12.02	11.85	10.92	5.44E-05	9.55E-04
8046824	401024	*fibrous sheath interacting protein 2*	*FSIP2*	4.94	4.77	6.57	5.45E-05	9.55E-04
8092691	604	*B-cell CLL/lymphoma 6*	*BCL6*	9.26	9.88	7.36	5.47E-05	9.57E-04
8138489	55536	*cell division cycle associated 7-like*	*CDCA7L*	7.23	8.00	11.05	5.48E-05	9.58E-04
8140534	10512	*sema domain, immunoglobulin domain (Ig), short basic domain, secreted, (semaphorin) 3C*	*SEMA3C*	8.47	10.13	5.89	5.48E-05	9.58E-04
7961602				5.56	5.45	5.05	5.48E-05	9.58E-04
8007084	5914	*retinoic acid receptor, alpha*	*RARA*	9.74	9.83	7.67	5.51E-05	9.62E-04
8090591	23129	*plexin D1*	*PLXND1*	10.74	10.61	8.15	5.51E-05	9.62E-04
8064557	64773	*family with sequence similarity 113, member A*	*FAM113A*	10.04	9.69	8.13	5.51E-05	9.62E-04
8033054	2525	*fucosyltransferase 3 (galactoside 3(4)-L-fucosyltransferase, Lewis blood group)*	*FUT3*	6.26	6.17	5.78	5.53E-05	9.62E-04
8122440	84946		*LTV1*	7.78	7.95	10.04	5.52E-05	9.62E-04
8001932	84752, 80262	*UDP-GlcNAc:betaGal beta-1,3-N-acetylglucosaminyltransferase 9 | chromosome 16 open reading frame 70*	*B3GNT9|C 16orf70*	9.49	9.47	7.11	5.53E-05	9.62E-04
8117018				4.71	4.77	7.69	5.53E-05	9.62E-04
7948612	3992	*fatty acid desaturase 1*	*FADS1*	10.48	9.70	11.10	5.54E-05	9.62E-04
7945232	170689	*ADAM metallopeptidase with thrombospondin type 1 motif, 15*	*ADAMTS1 5*	9.97	7.20	6.90	5.54E-05	9.62E-04
8145317	27299	*ADAM-like, decysin 1*	*ADAMDE C1*	5.30	5.31	4.97	5.55E-05	9.63E-04
7907882	57710		*KIAA1614*	7.62	7.61	6.75	5.55E-05	9.63E-04
7926170	55526	*dehydrogenase E1 and transketolase domain containing 1*	*DHTKD1*	8.64	8.63	10.01	5.58E-05	9.68E-04
7906576	23385	*nicastrin*	*NCSTN*	11.73	11.48	9.99	5.59E-05	9.69E-04

8027692	90075	*zinc finger protein 30*	*ZNF30*	5.67	5.46	6.24	5.61E-05	9.72E-04
8129985	5325, 57061	*pleiomorphic adenoma gene-like 1 | hydatidiform mole associated and imprinted (non-protein coding)*	*PLAGL1|HYMAI*	9.56	9.63	6.80	5.62E-05	9.73E-04
8011188	727910	*TLC domain containing 2*	*TLCD2*	8.68	8.69	7.75	5.63E-05	9.75E-04
7997733	2303	*forkhead box C2 (MFH-1, mesenchyme forkhead 1)*	*FOXC2*	8.63	8.39	7.26	5.65E-05	9.77E-04
7909568	51514	*denticleless homolog (Drosophila)*	*DTL*	6.70	7.52	11.58	5.65E-05	9.77E-04
8003484	9605	*chromosome 16 open reading frame 7*	*C16orf7*	8.75	8.76	6.95	5.66E-05	9.78E-04
8032157	22904	*strawberry notch homolog 2 (Drosophila)*	*SBNO2*	9.44	9.26	7.86	5.67E-05	9.78E-04
7934945	53354	*pantothenate kinase 1*	*PANK1*	6.80	6.80	8.51	5.68E-05	9.81E-04
7960828	9573	*growth differentiation factor 3*	*GDF3*	5.83	5.88	8.46	5.70E-05	9.81E-04
8007363	65266	*WNK lysine deficient protein kinase 4*	*WNK4*	7.99	7.85	6.68	5.70E-05	9.81E-04
7957072	117177	*RAB3A interacting protein (rabin3)*	*RAB3IP*	6.06	6.36	8.36	5.70E-05	9.81E-04
8030844	406887	*microRNA let-7e*	*MIRLET7E*	6.67	6.55	5.82	5.70E-05	9.81E-04
8152340	84955	*NudC domain containing 1*	*NUDCD1*	7.88	8.08	9.93	5.71E-05	9.82E-04
8041713	5495	*protein phosphatase, Mg2+/Mn2+ dependent, 1B*	*PPM1B*	7.25	7.26	10.34	5.72E-05	9.83E-04
7917591				6.89	6.60	6.03	5.73E-05	9.85E-04
8142468	7170	*tropomyosin 3*	*TPM3*	11.96	12.10	12.81	5.74E-05	9.86E-04
8092661	5648	*mannan-binding lectin serine peptidase 1 (C4/C2 activating component of Ra-reactive factor)*	*MASP1*	9.59	8.88	6.03	5.76E-05	9.88E-04
7899101	10256	*connector enhancer of kinase suppressor of Ras 1*	*CNKSR1*	6.38	6.49	7.56	5.76E-05	9.88E-04
8001133	79801	*SHC SH2-domain binding protein 1*	*SHCBP1*	5.85	6.31	8.85	5.78E-05	9.92E-04
7916910	55631	*leucine rich repeat containing 40*	*LRRC40*	7.07	7.37	9.05	5.79E-05	9.93E-04
7974533	57161	*pellino homolog 2 (Drosophila)*	*PELI2*	8.25	7.25	9.25	5.80E-05	9.93E-04
8136115	57464	*family with sequence similarity 40, member B*	*FAM40B*	6.25	6.38	7.91	5.81E-05	9.94E-04
8067185	655	*bone morphogenetic protein 7*	*BMP7*	6.17	6.28	7.73	5.82E-05	9.94E-04
8000117	1428	*crystallin, mu*	*CRYM*	6.99	6.98	8.33	5.82E-05	9.94E-04
8072170	83999	*kringle containing transmembrane protein 1*	*KREMEN1*	9.94	9.21	8.07	5.83E-05	9.96E-04
7984405	145853	*chromosome 15 open reading frame 61*	*C15orf61*	8.43	9.30	7.53	5.84E-05	9.96E-04

8120208	442890	*microRNA 133b*	*MIR133B*	7.33	7.45	6.33	5.84E-05	9.96E-04
8149774	4017	*lysyl oxidase-like 2*	*LOXL2*	12.52	12.97	8.94	5.85E-05	9.97E-04
8030871	79898	*zinc finger protein 613*	*ZNF613*	6.58	6.58	9.33	5.85E-05	9.97E-04
8134339	23089	*paternally expressed 10*	*PEG10*	8.01	8.55	10.85	5.88E-05	0.001000884
8035813	7594	*zinc finger protein 43*	*ZNF43*	7.04	6.74	9.70	5.90E-05	0.001003461
7969428	7347	*ubiquitin carboxyl-terminal esterase L3 (ubiquitin thiolesterase)*	*UCHL3*	8.48	8.90	10.29	5.91E-05	0.001005884
8144516	90459	*exoribonuclease 1*	*ERI1*	6.85	6.93	8.75	5.93E-05	0.001007597
7983360	567	*beta-2-microglobulin*	*B2M*	11.24	11.14	8.35	5.93E-05	0.001008112
8046560	3232	*homeobox D3*	*HOXD3*	6.17	6.92	5.80	5.94E-05	0.001008112
7972461	6564	*solute carrier family 15 (oligopeptide transporter), member 1*	*SLC15A1*	6.01	5.95	7.00	5.94E-05	0.001008112
7983650	11001	*solute carrier family 27 (fatty acid transporter), member 2*	*SLC27A2*	5.40	5.52	7.70	5.94E-05	0.001008112
7926084	509	*ATP synthase, H+ transporting, mitochondrial F1 complex, gamma polypeptide 1*	*ATP5C1*	11.38	11.41	12.17	5.98E-05	0.001012324
8017106	4591	*tripartite motif-containing 37*	*TRIM37*	7.91	7.82	10.12	5.98E-05	0.001012324
8102200	27123	*dickkopf homolog 2 (Xenopus laevis)*	*DKK2*	8.07	10.68	6.61	5.98E-05	0.001012324
7987642	51103	*NADH dehydrogenase (ubiquinone) 1 alpha subcomplex, assembly factor 1*	*NDUFAF1*	6.79	7.01	8.31	5.99E-05	0.001012324
8031076	59284	*calcium channel, voltage-dependent, gamma subunit 7*	*CACNG7*	8.05	7.51	9.89	5.98E-05	0.001012324
8036252	25999	*CAP-GLY domain containing linker protein 3*	*CLIP3*	11.42	11.23	8.46	6.00E-05	0.001013597
8001876	8883	*NEDD8 activating enzyme E1 subunit 1*	*NAE1*	8.46	8.44	11.08	6.01E-05	0.001015583
8045075	2840	*G protein-coupled receptor 17*	*GPR17*	6.70	6.64	6.04	6.04E-05	0.001018422
7959807	114795	*transmembrane protein 132B*	*TMEM132 B*	5.60	5.91	7.64	6.04E-05	0.001018422
7973020	390432	*olfactory receptor, family 4, subfamily Q, member 2 (gene/pseudogene)*	*OR4Q2*	5.93	5.78	5.59	6.05E-05	0.001020116
8169022	51186	*WW domain binding protein 5*	*WBP5*	9.11	7.50	7.65	6.08E-05	0.001024768
7997633	9100	*ubiquitin specific peptidase 10*	*USP10*	9.65	10.08	11.60	6.10E-05	0.001027606
8016789	54799	*mbt domain containing 1*	*MBTD1*	7.20	7.20	10.02	6.11E-05	0.001027811
8072206	2130	*Ewing sarcoma breakpoint region 1*	*EWSR1*	8.04	8.09	9.01	6.11E-05	0.001028007

8058108	129450	*chromosome 2 open reading frame 60*	*C2orf60*	6.40	6.45	8.23	6.11E-05	0.001028007
7986520	196968	*dynamin 1 pseudogene*	*C15orf51*	11.97	10.71	8.68	6.13E-05	0.001028879
8172156				7.34	7.61	6.70	6.13E-05	0.001028879
8168179	1741	*discs, large homolog 3 (Drosophila)*	*DLG3*	6.69	6.69	8.00	6.14E-05	0.001030794
8131496	113263	*glucocorticoid induced transcript 1*	*GLCCI1*	8.22	7.88	10.13	6.16E-05	0.001033455
7973924	9692		*KIAA0391*	8.94	9.17	10.46	6.19E-05	0.00103596
8149835	4747, 100129717	*neurofilament, light polypeptide | hypothetical LOC100129717*	*NEFL|LOC100129717*	7.19	7.18	8.36	6.18E-05	0.00103596
8081454				6.87	7.35	6.21	6.18E-05	0.00103596
8127542				7.44	7.53	6.89	6.19E-05	0.001036161
7923347	3898	*ladinin 1*	*LAD1*	7.47	7.61	8.85	6.20E-05	0.001037228
7924603	3930	*lamin B receptor*	*LBR*	7.66	7.33	9.92	6.21E-05	0.001037481
8044212	6819	*sulfotransferase family, cytosolic, 1C, member 2*	*SULT1C2*	5.59	5.58	6.73	6.21E-05	0.001037481
8010766				6.79	6.62	6.40	6.21E-05	0.001037481
7898084	23254	*kazrin*	*KAZ*	8.70	8.84	8.03	6.23E-05	0.001038944
7986517	196968	*dynamin 1 pseudogene*	*C15orf51*	11.99	10.74	8.71	6.23E-05	0.001039169
7970287	3916	*lysosomal-associated membrane protein 1*	*LAMP1*	11.64	11.59	10.31	6.25E-05	0.001039344
8058824	55686	*melanoregulin*	*MREG*	5.74	5.81	7.55	6.24E-05	0.001039344
8014081	55813		*UTP6*	7.17	7.49	9.20	6.24E-05	0.001039344
8039655	162972	*zinc finger protein 550*	*ZNF550*	7.48	7.29	8.56	6.25E-05	0.001039344
7986522	196968	*dynamin 1 pseudogene*	*C15orf51*	11.99	10.74	8.71	6.25E-05	0.001039344
8079099	92999	*zinc finger and BTB domain containing 47*	*ZBTB47*	9.10	9.04	7.41	6.26E-05	0.001039694
8008982	9496	*T-box 4*	*TBX4*	8.18	7.26	6.56	6.27E-05	0.001040521
8126574	25844	*Yip1 domain family, member 3*	*YIPF3*	12.98	12.85	11.67	6.27E-05	0.001040521
8141898	27000	*DnaJ (Hsp40) homolog, subfamily C, member 2*	*DNAJC2*	7.67	7.55	9.32	6.27E-05	0.001040873
8019762	5034	*prolyl 4-hydroxylase, beta polypeptide*	*P4HB*	12.03	12.19	10.29	6.28E-05	0.001040918
8115831	1843	*dual specificity phosphatase 1*	*DUSP1*	11.11	11.49	8.87	6.29E-05	0.001041439
7915045	54955	*chromosome 1 open reading frame 109*	*C1orf109*	6.68	6.83	7.51	6.29E-05	0.001041439

8146216	7419	*voltage-dependent anion channel 3*	*VDAC3*	11.29	11.43	12.64	6.30E-05	0.001041766
8002762	55159	*ring finger and WD repeat domain 3*	*RFWD3*	8.34	8.69	11.28	6.30E-05	0.001041766
7966668	6910	*T-box 5*	*TBX5*	6.89	9.59	6.25	6.31E-05	0.001043906
8091863	22865	*SLIT and NTRK-like family, member 3*	*SLITRK3*	5.75	5.82	7.46	6.32E-05	0.001043906
7976160	55775	*tyrosyl-DNA phosphodiesterase 1*	*TDP1*	8.01	8.59	10.67	6.33E-05	0.001043906
7929012	57559	*STAM binding protein-like 1*	*STAMBPL 1*	10.38	7.95	7.24	6.33E-05	0.001043906
7986509	196968	*dynamin 1 pseudogene*	*C15orf51*	11.97	10.70	8.63	6.33E-05	0.001043906
7986512	196968	*dynamin 1 pseudogene*	*C15orf51*	11.97	10.70	8.63	6.32E-05	0.001043906
7986527				11.97	10.70	8.63	6.34E-05	0.001043906
8112312	27292	*DIM1 dimethyladenosine transferase 1-like (S. cerevisiae)*	*DIMT1L*	9.18	9.64	10.33	6.34E-05	0.001044647
7936242	85450	*inositol 1,4,5-triphosphate receptor interacting protein*	*ITPRIP*	9.06	9.75	8.12	6.36E-05	0.001047434
8004144	79003		*MIS12*	7.04	7.03	9.49	6.37E-05	0.001048301
7920664	7059	*thrombospondin 3*	*THBS3*	9.21	8.79	6.52	6.39E-05	0.001049837
8117675	84547	*piggyBac transposable element derived 1*	*PGBD1*	6.40	6.54	7.47	6.39E-05	0.001049837
8145768				5.75	5.66	6.38	6.39E-05	0.001049866
8086494	285346	*zinc finger protein 852*	*ZNF852*	6.41	6.54	8.14	6.40E-05	0.00105036
8123621	5269	*serpin peptidase inhibitor, clade B (ovalbumin), member 6*	*SERPINB6*	10.39	9.81	8.51	6.42E-05	0.001052422
8152355	55638	*syntabulin (syntaxin-interacting)*	*SYBU*	8.77	8.87	6.32	6.42E-05	0.001052422
8105596	401190	*regulator of G-protein signaling 7 binding protein*	*RGS7BP*	5.78	6.08	7.26	6.42E-05	0.001052422
8152845	51571	*family with sequence similarity 49, member B*	*FAM49B*	7.41	7.27	10.40	6.43E-05	0.001053247
8115886	84321, 728554	*THO complex 3 | THO complex 3 pseudogene*	*THOC3|LOC728554*	7.67	8.07	9.77	6.44E-05	0.001053247
8174338	79710	*MORC family CW-type zinc finger 4*	*MORC4*	9.77	9.67	8.62	6.44E-05	0.001054111
7931899	3601	*interleukin 15 receptor, alpha*	*IL15RA*	9.36	9.13	7.65	6.46E-05	0.001054944
7965471	7334	*ubiquitin-conjugating enzyme E2N (UBC13 homolog, yeast)*	*UBE2N*	8.97	9.20	10.52	6.46E-05	0.001054944
7941639	582, 10072	*Bardet-Biedl syndrome 1 | dipeptidyl-peptidase 3*	*BBS1|DPP 3*	10.14	9.79	8.59	6.46E-05	0.001054944

7936463	3983	*actin binding LIM protein 1*	*ABLIM1*	7.05	6.87	9.76	6.47E-05	0.001055695
7977511	7011	*telomerase-associated protein 1*	*TEP1*	8.44	8.35	7.34	6.48E-05	0.001056812
7970975	54937	*spermatogenesis and oogenesis specific basic helix-loop-helix 2*	*SOHLH2*	6.00	5.89	8.76	6.49E-05	0.00105803
7914525	65108	*MARCKS-like 1*	*MARCKSL 1*	10.41	10.32	10.73	6.49E-05	0.00105803
8016390	51226	*coatomer protein complex, subunit zeta 2*	*COPZ2*	11.96	11.91	7.54	6.51E-05	0.001060644
8058849	57574	*membrane-associated ring finger (C3HC4) 4*	*MARCH4*	9.68	10.72	7.10	6.52E-05	0.001061587
8033789	7675	*zinc finger protein 121*	*ZNF121*	7.81	7.60	11.09	6.53E-05	0.001062701
8071276	6899, 54584	*T-box 1 | guanine nucleotide binding protein (G protein), beta polypeptide 1-like*	*TBX1|GNB 1L*	8.80	8.36	7.29	6.55E-05	0.001064356
8142471	7472	*wingless-type MMTV integration site family member 2*	*WNT2*	9.41	9.71	5.95	6.56E-05	0.001064381
8049752	25992	*sushi, nidogen and EGF-like domains 1*	*SNED1*	9.77	8.92	6.70	6.55E-05	0.001064381
8113348				6.70	6.88	6.10	6.58E-05	0.001067653
8136985	346528, 402317	*olfactory receptor, family 2, subfamily A, member 1 | olfactory receptor, family 2, subfamily A, member 42*	*OR2A1|OR 2A42*	7.74	7.51	6.69	6.63E-05	0.001075695
8133258	64409	*Williams-Beuren syndrome chromosome region 17*	*WBSCR17*	6.00	6.00	7.32	6.65E-05	0.00107755
8171577				5.67	5.98	5.08	6.66E-05	0.001078229
8078450	10491	*cartilage associated protein*	*CRTAP*	12.26	12.08	11.34	6.69E-05	0.001083134
8099633	10891	*peroxisome proliferator-activated receptor gamma, coactivator 1 alpha*	*PPARGC1 A*	5.97	5.98	6.91	6.69E-05	0.001083329
8172531	11230, 11152	*PRA1 domain family, member 2 | WD repeat domain 45*	*PRAF2|W DR45*	11.93	11.99	8.92	6.70E-05	0.001083389
7966127	6404	*selectin P ligand*	*SELPLG*	9.34	9.09	6.41	6.71E-05	0.001083539
8062293	22839	*discs, large (Drosophila) homolog-associated protein 4*	*DLGAP4*	10.41	10.50	8.95	6.70E-05	0.001083539
7961371	80824	*dual specificity phosphatase 16*	*DUSP16*	7.69	7.90	9.55	6.71E-05	0.001083539
8073192	7386	*ubiquinol-cytochrome c reductase, Rieske iron-sulfur polypeptide 1*	*UQCRFS1*	10.33	10.51	11.20	6.75E-05	0.001087723
7956271	8630	*hydroxysteroid (17-beta) dehydrogenase 6 homolog (mouse)*	*HSD17B6*	7.20	5.61	6.85	6.74E-05	0.001087723
7998136	64285	*rhomboid 5 homolog 1 (Drosophila)*	*RHBDF1*	10.51	10.07	8.28	6.75E-05	0.001087723
8113577	55521	*tripartite motif-containing 36*	*TRIM36*	5.65	5.67	6.30	6.76E-05	0.001089696

8124518	8331	*histone cluster 1, H2aj*	*HIST1H2AJ*	4.98	5.29	8.27	6.77E-05	0.001089719
8067017	8813	*dolichyl-phosphate mannosyltransferase polypeptide 1, catalytic subunit*	*DPM1*	10.55	10.77	11.89	6.78E-05	0.00109099
8118544				6.43	6.22	7.11	6.83E-05	0.001098771
8056968	375295		*LOC375295*	7.27	8.99	6.22	6.84E-05	0.001099269
8059852	151507	*male-specific lethal 3-like 2 (Drosophila)*	*MSL3L2*	7.95	7.83	9.59	6.86E-05	0.001101663
8129082	1300	*collagen, type X, alpha 1*	*COL10A1*	6.94	7.25	6.19	6.86E-05	0.00110175
7972055	115207	*potassium channel tetramerisation domain containing 12*	*KCTD12*	10.26	9.06	8.01	6.87E-05	0.001102531
7946428	56673	*chromosome 11 open reading frame 16*	*C11orf16*	6.61	6.90	6.26	6.88E-05	0.001103373
8029728	2696	*gastric inhibitory polypeptide receptor*	*GIPR*	7.28	7.30	6.53	6.89E-05	0.001103695
8001449	79191	*iroquois homeobox 3*	*IRX3*	9.96	10.09	7.23	6.90E-05	0.001105397
7969559	150928	*prothymosin, alpha pseudogene 5*	*PTMAP5*	11.15	11.25	12.14	6.91E-05	0.001107075
8053733	387893	*SET domain containing (lysine methyltransferase) 8*	*SETD8*	11.40	11.15	9.99	6.94E-05	0.001109429
8114778	2653, 729080, 100329108	*glycine cleavage system protein H (aminomethyl carrier) | glycine cleavage system H pseudogene | glycine cleavage system protein H pseudogene*	*GCSH|LOC729080|LOC100329108*	9.33	9.79	11.65	6.93E-05	0.001109429
7979565	112840	*WD repeat domain 89*	*WDR89*	6.89	6.97	8.98	6.95E-05	0.00111051
8017150	51174	*tubulin, delta 1*	*TUBD1*	6.13	6.26	8.28	6.96E-05	0.001111285
7943075	120114	*FAT tumor suppressor homolog 3 (Drosophila)*	*FAT3*	5.85	6.12	8.26	6.96E-05	0.001111285
8137874	84433	*caspase recruitment domain family, member 11*	*CARD11*	7.15	6.80	7.99	6.97E-05	0.00111301
8124606				4.84	4.91	5.77	6.99E-05	0.001114544
7920128	6282	*S100 calcium binding protein A11*	*S100A11*	13.29	13.34	11.24	7.00E-05	0.001115253
8098423	55247	*nei endonuclease VIII-like 3 (E. coli)*	*NEIL3*	7.43	7.03	9.23	7.04E-05	0.001121583
7933488	196740	*chromosome 10 open reading frame 72*	*C10orf72*	10.86	9.57	7.27	7.05E-05	0.001121944
8164217	64855	*family with sequence similarity 129, member B*	*FAM129B*	11.08	10.78	8.09	7.05E-05	0.001122111
7898673				9.09	9.17	7.73	7.06E-05	0.001123267
8147012	5569	*protein kinase (cAMP-dependent, catalytic) inhibitor alpha*	*PKIA*	6.69	6.52	7.80	7.08E-05	0.001125191

8131479	54468	*missing oocyte, meiosis regulator, homolog (Drosophila)*	*MIOS*	7.70	7.65	9.51	7.09E-05	0.001125191
7979158	57544	*thioredoxin domain containing 16*	*TXNDC16*	5.63	5.26	6.98	7.09E-05	0.001125191
7905523	353134	*late cornified envelope 1D*	*LCE1D*	9.11	9.19	8.54	7.09E-05	0.001125191
8059868	401036	*ankyrin repeat and SOCS box-containing 18*	*ASB18*	6.88	6.94	6.27	7.10E-05	0.001125191
7950626	729927	*ribosomal protein S20 pseudogene 27*	*RPS20P27*	7.72	7.60	9.08	7.10E-05	0.001125191
8092265	57129	*mitochondrial ribosomal protein L47*	*MRPL47*	9.12	9.30	10.70	7.11E-05	0.001126096
8160587	4712	*NADH dehydrogenase (ubiquinone) 1 beta subcomplex, 6, 17kDa*	*NDUFB6*	7.84	8.27	8.83	7.13E-05	0.001127915
8029458	388551	*carcinoembryonic antigen-related cell adhesion molecule 16*	*CEACAM16*	7.59	7.58	6.87	7.13E-05	0.001127915
8145772				5.76	5.90	6.64	7.13E-05	0.001127915
8147262	51633	*OTU domain containing 6B*	*OTUD6B*	8.04	8.07	9.92	7.15E-05	0.001130322
7913237	55450	*calcium/calmodulin-dependent protein kinase II inhibitor 1*	*CAMK2N1*	11.80	10.58	8.21	7.18E-05	0.001135001
7956613	6302	*tetraspanin 31*	*TSPAN31*	11.43	11.16	10.01	7.19E-05	0.001136168
7937020	4288	*antigen identified by monoclonal antibody Ki-67*	*MKI67*	7.14	7.24	9.85	7.20E-05	0.001136227
7918606	333926	*protein phosphatase, Mg2+/Mn2+ dependent, 1J*	*PPM1J*	7.39	7.36	8.92	7.20E-05	0.001136227
8026407	85360	*synapse defective 1, Rho GTPase, homolog 1 (C. elegans)*	*SYDE1*	10.46	10.71	7.68	7.22E-05	0.00113806
8109995	401217	*hypothetical LOC401217*	*FLJ40453*	5.77	5.46	5.30	7.22E-05	0.00113806
7986515	196968	*dynamin 1 pseudogene*	*C15orf51*	11.78	10.72	8.58	7.24E-05	0.001139522
7986525	196968	*dynamin 1 pseudogene*	*C15orf51*	11.78	10.72	8.58	7.25E-05	0.001139522
8023526	100132992	*high-mobility group nucleosome binding domain 1 pseudogene*	*LOC100132992*	9.31	9.42	10.95	7.25E-05	0.001139522
7950530				4.49	4.49	4.41	7.25E-05	0.001139522
8020451				4.83	4.83	5.83	7.26E-05	0.001140525
8128322	29078	*NADH dehydrogenase (ubiquinone) 1 alpha subcomplex, assembly factor 4*	*NDUFAF4*	8.93	9.34	11.16	7.27E-05	0.001142346
8144685				8.39	8.45	7.55	7.29E-05	0.001144289
8133360	1364	*claudin 4*	*CLDN4*	6.43	6.42	7.11	7.32E-05	0.001148789
8036923	9253	*numb homolog (Drosophila)-like*	*NUMBL*	10.36	10.11	8.81	7.33E-05	0.0011492

8174717	65109	*UPF3 regulator of nonsense transcripts homolog B (yeast)*	*UPF3B*	6.47	6.21	8.91	7.34E-05	0.001150112
7972570	87769	*AIG2-like domain 1*	*A2LD1*	8.13	8.18	7.16	7.34E-05	0.001150434
8104723				5.05	4.93	5.69	7.37E-05	0.001153361
8015635	284119	*polymerase I and transcript release factor*	*PTRF*	10.99	11.08	8.27	7.41E-05	0.001159216
8117170				5.04	4.99	5.90	7.41E-05	0.00115927
7981046	83982	*interferon, alpha-inducible protein 27-like 2*	*IFI27L2*	10.93	10.69	9.12	7.42E-05	0.001159516
8169836	7512	*X-prolyl aminopeptidase (aminopeptidase P) 2, membrane-bound*	*XPNPEP2*	8.84	8.75	6.35	7.43E-05	0.001159974
7897460	84275	*solute carrier family 25, member 33*	*SLC25A33*	7.28	7.43	8.77	7.43E-05	0.001160291
8171381	2187	*Fanconi anemia, complementation group B*	*FANCB*	6.44	6.50	8.04	7.47E-05	0.001162732
8083616	4291	*myeloid leukemia factor 1*	*MLF1*	5.90	5.88	7.69	7.46E-05	0.001162732
8116348	23061	*TBC1 domain family, member 9B (with GRAM domain)*	*TBC1D9B*	9.82	10.11	8.06	7.46E-05	0.001162732
8042381	56902	*partner of NOB1 homolog (S. cerevisiae)*	*PNO1*	7.67	8.05	10.49	7.46E-05	0.001162732
8004133	84268	*RPA interacting protein*	*RPAIN*	8.37	8.24	9.96	7.46E-05	0.001162732
7988031	6189	*ribosomal protein S3A*	*RPS3A*	10.80	10.87	11.97	7.49E-05	0.001164882
7913566	3352	*5-hydroxytryptamine (serotonin) receptor 1D*	*HTR1D*	6.87	6.92	9.81	7.50E-05	0.001165633
8052331	87178	*polyribonucleotide nucleotidyltransferase 1*	*PNPT1*	6.59	6.54	9.22	7.50E-05	0.001165633
8151942	10247	*heat-responsive protein 12*	*HRSP12*	8.20	8.29	9.73	7.51E-05	0.001165737
8012110	11337	*GABA(A) receptor-associated protein*	*GABARAP*	13.15	13.06	12.31	7.51E-05	0.001165737
8060205	23178	*PAS domain containing serine/threonine kinase*	*PASK*	7.11	6.93	8.31	7.51E-05	0.001165737
7906244	4914	*neurotrophic tyrosine kinase, receptor, type 1*	*NTRK1*	7.60	7.69	6.88	7.52E-05	0.001166212
7938291	619562	*small nucleolar RNA, H/ACA box 3*	*SNORA3*	13.34	13.45	12.89	7.52E-05	0.001166361
7978331	643866	*cerebellin 3 precursor*	*CBLN3*	9.20	9.00	6.88	7.53E-05	0.001166361
8145774				4.45	4.43	5.13	7.55E-05	0.001169229
8060353	10616	*RanBP-type and C3HC4-type zinc finger containing 1*	*RBCK1*	10.91	11.02	8.58	7.57E-05	0.001172243
8088192	26059	*ELKS/RAB6-interacting/CAST family member 2*	*ERC2*	6.16	6.18	7.41	7.59E-05	0.001173128
8064904	55612	*fermitin family member 1*	*FERMT1*	5.95	6.06	7.97	7.59E-05	0.001173128
7903117				5.96	6.10	5.59	7.64E-05	0.001180713

8020653	26256	*calcium binding tyrosine-(Y)-phosphorylation regulated*	*CABYR*	7.67	7.27	9.27	7.65E-05	0.001181037
8044849	5775	*protein tyrosine phosphatase, non-receptor type 4 (megakaryocyte)*	*PTPN4*	6.83	6.71	8.95	7.65E-05	0.001181124
8158625	100129785		*LOC100129785*	7.40	7.41	6.91	7.65E-05	0.001181124
8001394	27324	*TOX high mobility group box family member 3*	*TOX3*	6.90	7.01	9.20	7.66E-05	0.001181371
8042373	54465	*Ewing tumor-associated antigen 1*	*ETAA1*	6.85	6.51	8.25	7.66E-05	0.001181371
8132250	168667	*BMP binding endothelial regulator*	*BMPER*	7.60	9.39	6.41	7.67E-05	0.001181618
8041763	5581	*protein kinase C, epsilon*	*PRKCE*	8.84	8.26	6.62	7.69E-05	0.00118332
8065165	27131	*sorting nexin 5*	*SNX5*	9.41	9.42	10.91	7.69E-05	0.00118353
8097128	5393	*exosome component 9*	*EXOSC9*	7.79	8.32	10.23	7.71E-05	0.001185435
8169115	203447	*Nik related kinase*	*NRK*	5.32	5.42	7.35	7.73E-05	0.001187557
7995492	113	*adenylate cyclase 7*	*ADCY7*	8.45	8.99	7.12	7.76E-05	0.001191675
7960134	7574	*zinc finger protein 26*	*ZNF26*	6.55	6.31	7.64	7.77E-05	0.001193121
8006736	11072	*dual specificity phosphatase 14*	*DUSP14*	8.77	10.01	8.33	7.78E-05	0.001194113
8170364	2334	*AF4/FMR2 family, member 2*	*AFF2*	6.68	7.42	7.98	7.79E-05	0.001194983
7946812	6207	*ribosomal protein S13*	*RPS13*	13.03	13.06	13.53	7.80E-05	0.001195918
8174351	54830	*nucleoporin 62kDa C-terminal like*	*NUP62CL*	5.46	5.59	6.65	7.82E-05	0.001197918
8176671	9085, 253175, 9426, 203611	*chromodomain protein, Y-linked, 1 | chromodomain protein, Y-linked, 1B | chromodomain protein, Y-linked, 2A | chromodomain protein, Y-linked, 2B*	*CDY1|CDY1B|CDY2A|CDY2B*	5.25	5.35	5.13	7.85E-05	0.001202337
7999478	51061	*thioredoxin domain containing 11*	*TXNDC11*	9.30	9.40	8.08	7.87E-05	0.001204628
8165680				12.23	12.11	11.03	7.87E-05	0.001204628
8147469	10404	*plasma glutamate carboxypeptidase*	*PGCP*	10.37	10.80	7.27	7.90E-05	0.001206583
7899595	4146, 100129196	*matrilin 1, cartilage matrix protein | hypothetical LOC100129196*	*MATN1|LOC100129196*	7.10	6.88	6.13	7.89E-05	0.001206583
7953135	7289	*tubby like protein 3*	*TULP3*	10.94	11.15	9.72	7.92E-05	0.001207457
8149258	9258	*malignant fibrous histiocytoma amplified sequence 1*	*MFHAS1*	8.11	7.79	9.87	7.91E-05	0.001207457

8076137	25777	*Sad1 and UNC84 domain containing 2*	*SUN2*	9.73	9.83	7.39	7.91E-05	0.001207457
8056716	29081	*methyltransferase like 5*	*METTL5*	8.80	8.88	10.04	7.93E-05	0.001207457
7906307	55243	*kin of IRRE like (Drosophila)*	*KIRREL*	10.94	10.90	9.02	7.92E-05	0.001207457
7908543	140609	*NIMA (never in mitosis gene a)-related kinase 7*	*NEK7*	10.84	12.21	8.85	7.92E-05	0.001207457
7953603	716	*complement component 1, s subcomponent*	*C1S*	12.48	11.96	8.01	7.95E-05	0.001208407
8128592	9474		*ATG5*	8.44	8.52	9.83	7.95E-05	0.001208407
8016094	10052	*gap junction protein, gamma 1, 45kDa*	*GJC1*	8.55	8.57	10.90	7.94E-05	0.001208407
8128606	84816	*reticulon 4 interacting protein 1*	*RTN4IP1*	6.16	5.96	8.04	7.95E-05	0.001208407
7953622	283314		*LOC283314*	7.88	7.85	6.81	7.94E-05	0.001208407
7978846	5427	*polymerase (DNA directed), epsilon 2 (p59 subunit)*	*POLE2*	6.47	6.82	9.94	7.97E-05	0.001210928
7929511	953	*ectonucleoside triphosphate diphosphohydrolase 1*	*ENTPD1*	9.31	6.43	7.54	7.98E-05	0.001211874
7990941	440297, 114817	*chondroitin sulfate proteoglycan 4 pseudogene | chondroitin sulfate proteoglycan 4 pseudogene 5*	*LOC440297|CSPG4P5*	8.72	8.72	6.43	8.01E-05	0.001215228
8025429	4670	*heterogeneous nuclear ribonucleoprotein M*	*HNRNPM*	8.79	8.80	10.58	8.03E-05	0.001217117
7985259	54469	*zinc finger, AN1-type domain 6*	*ZFAND6*	8.32	8.47	10.07	8.03E-05	0.001217117
7940643	80150	*asparaginase like 1*	*ASRGL1*	6.81	6.79	8.10	8.04E-05	0.001217117
7987369	89978	*ATP binding domain 4*	*ATPBD4*	8.18	7.65	10.70	8.04E-05	0.001217117
8036333	57711	*zinc finger protein 529*	*ZNF529*	6.30	6.19	7.95	8.05E-05	0.001217379
8105545	51194, 3796	*importin 11 | kinesin heavy chain member 2A*	*IPO11|KIF2A*	8.47	8.27	10.72	8.05E-05	0.001217379
8132860	1956	*epidermal growth factor receptor*	*EGFR*	10.02	8.92	6.68	8.07E-05	0.001218916
8063000	10406	*WAP four-disulfide core domain 2*	*WFDC2*	7.19	7.25	8.56	8.08E-05	0.001218916
7966829	55884	*WD repeat and SOCS box-containing 2*	*WSB2*	11.28	11.72	10.66	8.07E-05	0.001218916
7956894	92797	*helicase (DNA) B*	*HELB*	6.53	6.09	8.28	8.07E-05	0.001218916
7981988	100033431, 692236	*small nucleolar RNA, C/D box 116-20 | small nucleolar RNA, C/D box 116 cluster*	*SNORD116-20|SNORD116@*	12.25	12.43	13.05	8.09E-05	0.001219483

8017671	146880		*LOC146880*	8.69	8.64	7.70	8.09E-05	0.001219486
7953200	894	*cyclin D2*	*CCND2*	7.59	7.27	11.51	8.10E-05	0.001219908
7961440	79887	*phospholipase B domain containing 1*	*PLBD1*	6.22	5.74	7.91	8.11E-05	0.001220916
8118945	5467	*peroxisome proliferator-activated receptor delta*	*PPARD*	10.58	10.40	8.37	8.14E-05	0.00122265
7905329	10962	*myeloid/lymphoid or mixed-lineage leukemia (trithorax homolog, Drosophila); translocated to, 11*	*MLLT11*	8.74	9.33	11.20	8.12E-05	0.00122265
8121734	25842	*ASF1 anti-silencing function 1 homolog A (S. cerevisiae)*	*ASF1A*	8.48	8.43	10.57	8.13E-05	0.00122265
8142332	83943	*IMP2 inner mitochondrial membrane peptidase-like (S. cerevisiae)*	*IMMP2L*	5.55	5.48	6.37	8.13E-05	0.00122265
8080162	25864, 95	*abhydrolase domain containing 14A | aminoacylase 1*	*ABHD14A|ACY1*	10.94	10.78	8.02	8.14E-05	0.00122265
7985416	440297, 114817	*chondroitin sulfate proteoglycan 4 pseudogene | chondroitin sulfate proteoglycan 4 pseudogene 5*	*LOC440297|CSPG4P5*	8.72	8.71	6.43	8.14E-05	0.00122265
8154523	10670	*Ras-related GTP binding A*	*RRAGA*	9.28	9.34	8.40	8.15E-05	0.001222976
8013159	146691	*target of myb1-like 2 (chicken)*	*TOM1L2*	10.67	10.68	8.00	8.16E-05	0.001223081
8003410	2588	*galactosamine (N-acetyl)-6-sulfate sulfatase*	*GALNS*	10.92	10.77	8.73	8.17E-05	0.001225248
7985457	440297, 114817	*chondroitin sulfate proteoglycan 4 pseudogene | chondroitin sulfate proteoglycan 4 pseudogene 5*	*LOC440297|CSPG4P5*	8.72	8.72	6.43	8.20E-05	0.001227976
7985202	5685	*proteasome (prosome, macropain) subunit, alpha type, 4*	*PSMA4*	10.12	10.32	11.96	8.22E-05	0.001230484
8099612	166647	*G protein-coupled receptor 125*	*GPR125*	6.91	6.81	9.35	8.22E-05	0.001230484
7908777				12.59	12.63	11.75	8.23E-05	0.001230629
7985560	440297, 114817	*chondroitin sulfate proteoglycan 4 pseudogene | chondroitin sulfate proteoglycan 4 pseudogene 5*	*LOC440297|CSPG4P5*	8.15	8.04	5.93	8.24E-05	0.001232051
8136849	373156	*glutathione S-transferase kappa 1*	*GSTK1*	11.25	10.98	9.45	8.25E-05	0.001232204
8137953	84629	*trinucleotide repeat containing 18*	*TNRC18*	10.25	10.12	8.39	8.26E-05	0.001232949
8063386	1051	*CCAAT/enhancer binding protein (C/EBP), beta*	*CEBPB*	10.96	11.20	8.65	8.26E-05	0.001233276
7951447	143884	*CWF19-like 2, cell cycle control (S. pombe)*	*CWF19L2*	6.10	5.87	7.37	8.27E-05	0.001234499

7985688	440297, 114817	*chondroitin sulfate proteoglycan 4 pseudogene | chondroitin sulfate proteoglycan 4 pseudogene 5*	*LOC440297|CSPG4P5*	8.73	8.72	6.42	8.28E-05	0.001235089
7957458	4922	*neurotensin*	*NTS*	5.01	4.98	7.46	8.30E-05	0.001236467
7958884	4938	*2',5'-oligoadenylate synthetase 1, 40/46kDa*	*OAS1*	7.65	7.15	6.72	8.30E-05	0.001236467
8069753	10694	*chaperonin containing TCP1, subunit 8 (theta)*	*CCT8*	7.83	7.80	9.39	8.35E-05	0.001242832
8170971	1736	*dyskeratosis congenita 1, dyskerin*	*DKC1*	7.47	7.82	10.01	8.36E-05	0.001244063
8109333	2878	*glutathione peroxidase 3 (plasma)*	*GPX3*	12.03	8.13	9.15	8.36E-05	0.001244063
7929334	55165	*centrosomal protein 55kDa*	*CEP55*	6.94	7.08	9.15	8.37E-05	0.001244063
8000284	23062	*golgi-associated, gamma adaptin ear containing, ARF binding protein 2*	*GGA2*	9.49	9.31	10.60	8.38E-05	0.00124453
8012376	6844	*vesicle-associated membrane protein 2 (synaptobrevin 2)*	*VAMP2*	10.52	10.34	8.36	8.39E-05	0.0012451
7970413	55269	*paraspeckle component 1*	*PSPC1*	8.50	8.49	10.42	8.39E-05	0.0012451
8099649	1665	*DEAH (Asp-Glu-Ala-His) box polypeptide 15*	*DHX15*	8.05	7.94	10.70	8.42E-05	0.001247957
7927827	84665	*myopalladin*	*MYPN*	6.26	5.77	5.26	8.42E-05	0.001247957
7992255	84572	*N-acetylglucosamine-1-phosphate transferase, gamma subunit*	*GNPTG*	10.66	10.50	8.73	8.43E-05	0.001249434
7920291	140576	*S100 calcium binding protein A16*	*S100A16*	11.39	11.99	8.68	8.44E-05	0.00124974
8004416	1140, 2256	*cholinergic receptor, nicotinic, beta 1 (muscle) | fibroblast growth factor 11*	*CHRNB1|FGF11*	7.35	7.23	8.49	8.45E-05	0.001250525
8155062	7016, 971	*testis-specific kinase 1 | CD72 molecule*	*TESK1|CD72*	10.24	10.49	8.23	8.46E-05	0.001251675
8032815	80700	*UBX domain protein 6*	*UBXN6*	10.27	10.17	8.19	8.46E-05	0.00125183
7954173	11171	*serine/threonine kinase receptor associated protein*	*STRAP*	9.44	9.93	11.13	8.48E-05	0.001253369
8051622	6432	*serine/arginine-rich splicing factor 7*	*SRSF7*	7.99	8.14	10.60	8.49E-05	0.001254482
7973948	84312	*breast cancer metastasis-suppressor 1-like*	*BRMS1L*	5.50	5.43	6.42	8.50E-05	0.001254482
7995813	326343	*metallothionein 1D (pseudogene)*	*MT1DP*	10.83	10.90	8.81	8.50E-05	0.001254482
7937802	975		*CD81*	13.75	13.66	12.35	8.52E-05	0.001255088
8086810	6599	*SWI/SNF related, matrix associated, actin dependent regulator of chromatin, subfamily c, member 1*	*SMARCC1*	9.50	9.63	11.76	8.52E-05	0.001255088

7916282	7804	*low density lipoprotein receptor-related protein 8, apolipoprotein e receptor*	*LRP8*	8.34	8.23	10.29	8.53E-05	0.001255088
8062023	84557	*microtubule-associated protein 1 light chain 3 alpha*	*MAP1LC3A*	9.09	9.23	7.22	8.52E-05	0.001255088
8024532	126295	*zinc finger protein 57*	*ZNF57*	7.80	7.87	9.61	8.52E-05	0.001255088
8133200				5.71	5.73	5.30	8.53E-05	0.001255088
8163666	58483	*chromosome 9 open reading frame 27*	*C9orf27*	5.41	5.47	5.19	8.60E-05	0.00126483
8013061	23495	*tumor necrosis factor receptor superfamily, member 13B*	*TNFRSF13B*	8.61	8.67	8.03	8.61E-05	0.001265589
8006634	284098	*phosphatidylinositol glycan anchor biosynthesis, class W*	*PIGW*	6.41	6.91	8.73	8.62E-05	0.001265833
7954711	55196	*chromosome 12 open reading frame 35*	*C12orf35*	7.60	7.26	10.76	8.62E-05	0.001266062
8024728	27231	*integrin beta 1 binding protein 3*	*ITGB1BP3*	7.47	7.47	9.01	8.63E-05	0.001266341
8041179	79745	*CAP-GLY domain containing linker protein family, member 4*	*CLIP4*	8.81	7.97	5.50	8.64E-05	0.001267013
7943297	9702	*centrosomal protein 57kDa*	*CEP57*	8.22	8.16	9.84	8.64E-05	0.001267222
7998843	83886	*protease, serine 27*	*PRSS27*	8.66	8.76	8.06	8.65E-05	0.00126793
7959361	22877	*MLX interacting protein*	*MLXIP*	10.02	10.28	8.70	8.66E-05	0.001268051
8011262	4335	*MAX binding protein*	*MNT*	9.21	9.22	7.88	8.68E-05	0.00126952
8069880	7074	*T-cell lymphoma invasion and metastasis 1*	*TIAM1*	6.58	7.03	8.74	8.68E-05	0.00126952
7960850	144195	*solute carrier family 2 (facilitated glucose transporter), member 14*	*SLC2A14*	8.03	8.48	10.40	8.68E-05	0.001269568
8176191	3150	*high-mobility group nucleosome binding domain 1*	*HMGN1*	10.64	10.72	12.08	8.69E-05	0.001269636
8061136	5757	*prothymosin, alpha*	*PTMA*	13.53	13.66	14.23	8.69E-05	0.001270282
8088172				6.89	6.87	6.19	8.70E-05	0.001270308
8008263	5164	*pyruvate dehydrogenase kinase, isozyme 2*	*PDK2*	10.72	10.33	8.51	8.72E-05	0.001271867
7980316	7043	*transforming growth factor, beta 3*	*TGFB3*	7.90	7.73	6.32	8.72E-05	0.001271867
8046515	3020, 440926	*H3 histone, family 3A | H3 histone, family 3A pseudogene*	*H3F3A|LOC440926*	10.83	11.00	12.75	8.73E-05	0.001273322
7935776	6319	*stearoyl-CoA desaturase (delta-9-desaturase)*	*SCD*	5.56	5.36	6.68	8.75E-05	0.001274602
8126212				8.05	8.30	7.50	8.78E-05	0.001278327
8139891	51119	*Shwachman-Bodian-Diamond syndrome*	*SBDS*	11.16	11.49	10.10	8.79E-05	0.001280109

7914141	6118	*replication protein A2, 32kDa*	*RPA2*	6.68	6.87	7.94	8.80E-05	0.001280589
7903478	148545, 653149, 284610	*neuroblastoma breakpoint family, member 4 | neuroblastoma breakpoint family, member 6 | neuroblastoma breakpoint family, member 5*	*NBPF4|NBPF6|NBPF5*	6.29	6.20	5.90	8.82E-05	0.001282336
8112139	3572	*interleukin 6 signal transducer (gp130, oncostatin M receptor)*	*IL6ST*	12.07	10.38	8.12	8.82E-05	0.001282674
8145490	2185		*PTK2B*	10.01	7.87	6.96	8.84E-05	0.001284407
8116579	94234	*forkhead box Q1*	*FOXQ1*	8.10	7.96	7.29	8.85E-05	0.001284625
8093171	4241	*antigen p97 (melanoma associated) identified by monoclonal antibodies 133.2 and 96.5*	*MFI2*	9.26	8.63	7.39	8.87E-05	0.001287462
8124402	3010	*histone cluster 1, H1t*	*HIST1H1T*	5.75	5.78	7.21	8.88E-05	0.001287532
7922908				5.08	5.27	4.82	8.88E-05	0.001287532
7957540	28977	*mitochondrial ribosomal protein L42*	*MRPL42*	8.46	8.32	10.76	8.89E-05	0.001287749
7966368	5501	*protein phosphatase 1, catalytic subunit, gamma isozyme*	*PPP1CC*	11.35	11.02	13.14	8.90E-05	0.001288972
8017262	83990	*BRCA1 interacting protein C-terminal helicase 1*	*BRIP1*	6.68	6.45	9.48	8.90E-05	0.001289259
7897068	6497	*v-ski sarcoma viral oncogene homolog (avian)*	*SKI*	11.26	11.36	9.98	8.93E-05	0.001291659
7929919	81855	*sideroflexin 3*	*SFXN3*	10.20	10.29	7.58	8.93E-05	0.001292197
7909967	824	*calpain 2, (m/II) large subunit*	*CAPN2*	11.36	11.44	8.65	8.98E-05	0.001296496
8073962	54456	*Mov10l1, Moloney leukemia virus 10-like 1, homolog (mouse)*	*MOV10L1*	6.77	6.54	5.97	8.97E-05	0.001296496
8058927	64114	*transmembrane BAX inhibitor motif containing 1*	*TMBIM1*	12.70	12.65	10.52	8.97E-05	0.001296496
8043131	84173	*ELMO/CED-12 domain containing 3*	*ELMOD3*	8.25	8.11	6.84	9.01E-05	0.001300395
8145136	5533	*protein phosphatase 3, catalytic subunit, gamma isozyme*	*PPP3CC*	8.19	8.48	6.93	9.05E-05	0.001303353
7974920	23224	*spectrin repeat containing, nuclear envelope 2*	*SYNE2*	6.77	5.99	9.13	9.04E-05	0.001303353
7947934	23788	*mitochondrial carrier homolog 2 (C. elegans)*	*MTCH2*	10.47	10.71	11.13	9.05E-05	0.001303353
7996654	55815	*translin-associated factor X interacting protein 1*	*TSNAXIP1*	6.51	6.41	6.00	9.05E-05	0.001303353
8051372	51072, 84661	*mediator of cell motility 1 | dpy-30 homolog (C. elegans)*	*MEMO1|DPY30*	7.74	8.11	8.70	9.04E-05	0.001303353
8013157	146691	*target of myb1-like 2 (chicken)*	*TOM1L2*	8.20	8.44	6.31	9.07E-05	0.001305311
7931571	118471	*proline-rich acidic protein 1*	*PRAP1*	7.64	7.65	7.11	9.11E-05	0.001309841

8045946	10213	*proteasome (prosome, macropain) 26S subunit, non-ATPase, 14*	*PSMD14*	8.42	8.80	10.60	9.11E-05	0.001310038
8156116	414328	*chromosome 9 open reading frame 103*	*C9orf103*	8.49	8.21	6.88	9.15E-05	0.001315373
8141222	6189	*ribosomal protein S3A*	*RPS3A*	11.95	12.00	12.98	9.20E-05	0.00132032
8151066	55156	*armadillo repeat containing 1*	*ARMC1*	7.53	7.57	8.90	9.20E-05	0.00132032
8081838	57514	*Rho GTPase activating protein 31*	*ARHGAP31*	8.49	9.49	6.88	9.23E-05	0.00132501
7927814	23411	*sirtuin 1*	*SIRT1*	7.85	7.76	10.71	9.26E-05	0.001328641
8130739	6196	*ribosomal protein S6 kinase, 90kDa, polypeptide 2*	*RPS6KA2*	10.54	10.39	8.05	9.28E-05	0.001330162
8134407	1750	*distal-less homeobox 6*	*DLX6*	7.85	7.77	7.27	9.30E-05	0.001331584
8027323	113835	*zinc finger protein 257*	*ZNF257*	5.01	4.99	8.83	9.30E-05	0.001331584
8132045	84725, 51054	*pleckstrin homology domain containing, family A (phosphoinositide binding specific) member 8 | pleckstrin homology domain containing, family A (phosphoinositide binding specific) member 9*	*PLEKHA8| PLEKHA9*	7.89	7.78	9.29	9.30E-05	0.001331584
8159521	5730	*prostaglandin D2 synthase 21kDa (brain)*	*PTGDS*	8.02	8.51	7.19	9.32E-05	0.001332257
8165277	158062	*lipocalin 6*	*LCN6*	7.20	7.31	6.70	9.31E-05	0.001332257
8001496	11051	*nudix (nucleoside diphosphate linked moiety X)-type motif 21*	*NUDT21*	10.66	10.44	12.62	9.34E-05	0.001334015
8093997	57537	*sortilin-related VPS10 domain containing receptor 2*	*SORCS2*	8.31	8.61	6.82	9.34E-05	0.001334015
8117237				9.17	9.42	10.70	9.34E-05	0.001334015
8124211	2822	*glycosylphosphatidylinositol specific phospholipase D1*	*GPLD1*	5.63	5.56	6.86	9.37E-05	0.001336715
8110265	2264	*fibroblast growth factor receptor 4*	*FGFR4*	7.10	6.95	8.66	9.41E-05	0.00133933
7904761	8515	*integrin, alpha 10*	*ITGA10*	6.24	6.32	5.79	9.41E-05	0.00133933
8065920	80307	*fer-1-like 4 (C. elegans) pseudogene*	*FER1L4*	6.78	7.03	6.42	9.42E-05	0.00133933
8050427	81553	*family with sequence similarity 49, member A*	*FAM49A*	6.17	6.13	7.65	9.41E-05	0.00133933
8021453	90701	*SEC11 homolog C (S. cerevisiae)*	*SEC11C*	7.74	7.73	9.59	9.42E-05	0.00133933
8054702	150468	*cytoskeleton associated protein 2-like*	*CKAP2L*	6.26	6.65	8.61	9.40E-05	0.00133933
7910198	339500	*zinc finger protein 678*	*ZNF678*	6.66	6.40	8.63	9.42E-05	0.00133933
7924307				5.66	5.73	6.89	9.42E-05	0.00133933
8001680				6.76	7.01	6.18	9.43E-05	0.001340313

8020955	55034	*molybdenum cofactor sulfurase*	*MOCOS*	8.81	9.25	7.96	9.44E-05	0.001340519
8098150	55319	*chromosome 4 open reading frame 43*	*C4orf43*	6.75	6.65	9.05	9.47E-05	0.001343799
7997336	57687	*vesicle amine transport protein 1 homolog (T. californica)-like*	*VAT1L*	8.92	7.19	9.98	9.47E-05	0.001343799
7927008	219771	*cyclin Y*	*CCNY*	10.14	10.48	9.99	9.50E-05	0.00134765
8058091	23314	*SATB homeobox 2*	*SATB2*	10.35	10.05	8.07	9.52E-05	0.001348335
7926786	54518	*amyloid beta (A4) precursor protein-binding, family B, member 1 interacting protein*	*APBB1IP*	9.87	8.02	6.16	9.52E-05	0.001348335
8041170				7.07	6.94	9.58	9.52E-05	0.001348335
8137081				6.58	6.68	5.98	9.54E-05	0.001350655
8094228	683	*bone marrow stromal cell antigen 1*	*BST1*	10.45	9.87	7.38	9.56E-05	0.001352067
8070081				7.45	7.42	6.65	9.56E-05	0.001352067
8120022	988	*CDC5 cell division cycle 5-like (S. pombe)*	*CDC5L*	9.31	9.25	10.64	9.59E-05	0.001354101
7899813	5928	*retinoblastoma binding protein 4*	*RBBP4*	10.71	10.71	12.49	9.60E-05	0.001354101
7944913	80071	*coiled-coil domain containing 15*	*CCDC15*	5.68	5.63	6.57	9.60E-05	0.001354101
7910124	3020, 440926	*H3 histone, family 3A | H3 histone, family 3A pseudogene*	*H3F3A|LOC440926*	10.94	11.11	12.81	9.59E-05	0.001354101
7990309	64220	*stimulated by retinoic acid gene 6 homolog (mouse)*	*STRA6*	8.74	7.41	7.16	9.61E-05	0.001354692
8108192	9555	*H2A histone family, member Y*	*H2AFY*	7.54	6.91	6.35	9.61E-05	0.00135522
8110982	1611	*death-associated protein*	*DAP*	12.43	12.35	10.20	9.62E-05	0.001355702
8054439	84620	*ST6 beta-galactosamide alpha-2,6-sialyltranferase 2*	*ST6GAL2*	6.42	6.47	7.86	9.64E-05	0.001357337
8108015	3308	*heat shock 70kDa protein 4*	*HSPA4*	9.03	9.09	11.55	9.65E-05	0.001358087
8097928	166863	*RNA binding motif protein 46*	*RBM46*	5.38	5.40	8.69	9.70E-05	0.001365352
8021470	5366	*phorbol-12-myristate-13-acetate-induced protein 1*	*PMAIP1*	6.83	7.61	11.22	9.75E-05	0.001368336
7927936	9188	*DEAD (Asp-Glu-Ala-Asp) box polypeptide 21*	*DDX21*	7.67	7.72	10.97	9.73E-05	0.001368336
7939805	91252	*solute carrier family 39 (zinc transporter), member 13*	*SLC39A13*	10.51	10.69	8.31	9.74E-05	0.001368336
8177938	170679	*psoriasis susceptibility 1 candidate 1*	*PSORS1C1*	6.92	6.25	5.72	9.74E-05	0.001368336
7919326	51205	*acid phosphatase 6, lysophosphatidic*	*ACP6*	7.80	7.23	8.54	9.76E-05	0.001368896
8145134				9.30	5.67	5.44	9.76E-05	0.001368896

8029907	728	*complement component 5a receptor 1*	*C5AR1*	6.79	6.83	5.96	9.80E-05	0.001372504
8019478	924		*CD7*	9.44	8.94	8.11	9.80E-05	0.001372504
7957850	283431	*growth arrest-specific 2 like 3*	*GAS2L3*	7.63	7.36	9.65	9.80E-05	0.001372504
8018975	3959	*lectin, galactoside-binding, soluble, 3 binding protein*	*LGALS3BP*	11.58	10.87	8.52	9.83E-05	0.001375342
7957737	7112	*thymopoietin*	*TMPO*	8.21	8.86	11.15	9.83E-05	0.001375342
8161558	375719, 364, 100510503	*aquaporin 7 pseudogene 1 | aquaporin-7-like*	*AQP7P1|LOC100510503*	5.97	5.93	5.33	9.87E-05	0.001379848
8046318	80067	*DDB1 and CUL4 associated factor 17*	*DCAF17*	8.02	7.67	9.83	9.89E-05	0.001382177
8021866	4772	*nuclear factor of activated T-cells, cytoplasmic, calcineurin-dependent 1*	*NFATC1*	8.67	8.98	7.44	9.93E-05	0.00138654
7969171	10301	*deleted in lymphocytic leukemia 1 (non-protein coding)*	*DLEU1*	7.14	7.81	9.10	9.93E-05	0.00138654
8030128	23645	*protein phosphatase 1, regulatory (inhibitor) subunit 15A*	*PPP1R15A*	10.28	10.68	8.39	9.92E-05	0.00138654
7917604	84146	*zinc finger protein 644*	*ZNF644*	8.20	8.13	10.21	9.96E-05	0.001387923
7953341	55080, 6843	*TAP binding protein-like | vesicle-associated membrane protein 1 (synaptobrevin 1)*	*TAPBPL|VAMP1*	10.07	9.52	7.02	9.95E-05	0.001387923
8034334	7568, 90589	*zinc finger protein 20 | zinc finger protein 625*	*ZNF20|ZNF625*	6.29	6.19	6.85	9.96E-05	0.001387923
8043100	9168	*thymosin beta 10*	*TMSB10*	13.19	13.32	12.52	9.98E-05	0.001390844
8035980	85415	*rhophilin, Rho GTPase binding protein 2*	*RHPN2*	7.38	7.69	9.06	9.99E-05	0.001391751
8102050	133308	*Na+/H+ exchanger domain containing 2*	*NHEDC2*	8.96	9.18	7.16	1.00E-04	0.001392008
7989647	9768, 53944	*KIAA0101 | casein kinase 1, gamma 1*	*KIAA0101|CSNK1G1*	7.91	7.77	9.98	1.00E-04	0.001392008
7981730	28831, 28442, 3500, 3493	*immunoglobulin lambda joining 3 | immunoglobulin heavy variable 3-23 | immunoglobulin heavy constant gamma 1 (G1m marker) | immunoglobulin heavy constant alpha 1*	*IGLJ3|IGHV3-23|IGHG1|IGHA1*	8.11	8.25	7.53	1.00E-04	0.001396459
8174513	91851	*chordin-like 1*	*CHRDL1*	6.70	6.64	9.31	1.01E-04	0.001398478
8099668				7.07	6.72	9.49	1.01E-04	0.001398478
7922391	91687	*centromere protein L*	*CENPL*	6.01	6.51	7.75	1.01E-04	0.001400219

7976560	624	*bradykinin receptor B2*	*BDKRB2*	9.80	8.37	6.78	1.01E-04	0.001401488
8064766	11237	*ring finger protein 24*	*RNF24*	9.76	10.03	8.36	1.01E-04	0.001401488
8052735	25927	*cannabinoid receptor interacting protein 1*	*CNRIP1*	9.85	9.43	7.85	1.01E-04	0.001402407
7903092	54874	*formin binding protein 1-like*	*FNBP1L*	5.68	7.05	11.10	1.01E-04	0.001402842
8052355	2202	*EGF-containing fibulin-like extracellular matrix protein 1*	*EFEMP1*	12.50	12.91	8.72	1.01E-04	0.001402935
8147756	79870	*brain and acute leukemia, cytoplasmic*	*BAALC*	6.81	7.89	6.47	1.01E-04	0.001402935
8014969	125111	*gap junction protein, delta 3, 31.9kDa*	*GJD3*	9.13	8.85	7.54	1.01E-04	0.001402935
8005110	57335, 729288, 353149	*zinc finger protein 286A | zinc finger protein 286B | TBC1 domain family, member 26*	*ZNF286A| ZNF286B| TBC1D26*	7.44	7.51	10.22	1.01E-04	0.001402935
8142979				8.47	8.53	7.52	1.01E-04	0.001402935
8044295	344558	*SH3 domain containing ring finger 3*	*SH3RF3*	10.51	9.98	8.17	1.02E-04	0.001403841
8017210	8905, 653653	*adaptor-related protein complex 1, sigma 2 subunit | adaptor-related protein complex 1, sigma 2 subunit pseudogene*	*AP1S2|LOC653653*	9.16	8.80	11.31	1.02E-04	0.001403841
8116956	23408	*sirtuin 5*	*SIRT5*	7.32	7.43	8.13	1.02E-04	0.001406875
8158930	11092, 158067	*chromosome 9 open reading frame 9 | chromosome 9 open reading frame 98*	*C9orf9|C9orf98*	8.49	8.34	6.91	1.02E-04	0.001408131
8008388	64847	*spermatogenesis associated 20*	*SPATA20*	11.09	10.57	8.55	1.02E-04	0.001409332
8156290	1164	*CDC28 protein kinase regulatory subunit 2*	*CKS2*	9.52	9.39	12.18	1.02E-04	0.001409644
8117572	346157	*zinc finger protein 391*	*ZNF391*	6.75	6.90	8.86	1.02E-04	0.001409644
8175261	574506	*microRNA 503*	*MIR503*	9.17	9.60	6.89	1.03E-04	0.001413531
8157582	2934	*gelsolin*	*GSN*	11.51	11.22	8.81	1.03E-04	0.001415611
8028872	8425	*latent transforming growth factor beta binding protein 4*	*LTBP4*	10.03	9.32	8.22	1.03E-04	0.001415611
8093665	2868	*G protein-coupled receptor kinase 4*	*GRK4*	6.47	6.53	5.91	1.03E-04	0.001418448
8058161	4999	*origin recognition complex, subunit 2*	*ORC2*	7.36	7.35	9.87	1.04E-04	0.001421917
7939102	26610	*elongation protein 4 homolog (S. cerevisiae)*	*ELP4*	8.36	8.72	9.74	1.04E-04	0.001421917
8124262	51567	*tyrosyl-DNA phosphodiesterase 2*	*TDP2*	8.77	8.54	10.37	1.04E-04	0.001421917
7948037				4.77	4.74	6.05	1.04E-04	0.001421917

8066343				5.53	5.26	4.83	1.04E-04	0.001421917
7930917	2869	*G protein-coupled receptor kinase 5*	*GRK5*	8.64	7.02	5.86	1.04E-04	0.001421931
7951408	114769	*caspase recruitment domain family, member 16*	*CARD16*	6.28	5.52	5.15	1.04E-04	0.001421931
7900216	79647	*akirin 1*	*AKIRIN1*	9.24	9.63	11.92	1.04E-04	0.001424472
8087530	79012	*CaM kinase-like vesicle-associated*	*CAMKV*	6.18	6.25	8.77	1.05E-04	0.001432193
7910630	388753	*chromosome 1 open reading frame 31*	*C1orf31*	7.80	8.05	9.25	1.05E-04	0.00143272
7973756	55632	*G2/M-phase specific E3 ubiquitin protein ligase*	*G2E3*	6.73	6.16	9.14	1.05E-04	0.001435246
7914021	6548	*solute carrier family 9 (sodium/hydrogen exchanger), member 1*	*SLC9A1*	10.07	10.42	8.28	1.05E-04	0.001435568
8008564	10040	*target of myb1 (chicken)-like 1*	*TOM1L1*	6.26	6.05	7.97	1.05E-04	0.001435568
8122242	5191	*peroxisomal biogenesis factor 7*	*PEX7*	7.21	7.39	8.42	1.05E-04	0.001437375
7916582				6.21	6.31	5.95	1.05E-04	0.00143793
7971813	55901	*thrombospondin, type I, domain containing 1*	*THSD1*	7.47	7.86	6.39	1.06E-04	0.001441373
8042962	9801	*mitochondrial ribosomal protein L19*	*MRPL19*	8.31	8.59	9.57	1.06E-04	0.001444059
8124551	222699	*transducer of ERBB2, 2 pseudogene*	*LOC222699*	8.27	8.22	7.42	1.06E-04	0.001444059
7897737	374946	*chromosome 1 open reading frame 187*	*C1orf187*	7.82	7.78	9.10	1.06E-04	0.001444059
8099304	8310	*acyl-CoA oxidase 3, pristanoyl*	*ACOX3*	9.46	9.35	7.93	1.06E-04	0.00144576
8077612	26140, 7862	*tubulin tyrosine ligase-like family, member 3 | bromodomain and PHD finger containing, 1*	*TTLL3|BRPF1*	9.29	8.76	6.64	1.06E-04	0.00144576
8141228	222865	*transmembrane protein 130*	*TMEM130*	10.26	8.49	7.77	1.06E-04	0.001446779
7940775	5920	*retinoic acid receptor responder (tazarotene induced) 3*	*RARRES3*	9.68	7.52	6.94	1.07E-04	0.001449424
7936856	51363	*carbohydrate (N-acetylgalactosamine 4-sulfate 6-O) sulfotransferase 15*	*CHST15*	9.05	7.58	8.27	1.07E-04	0.001449424
8065032	51575		*ESF1*	6.02	6.21	8.03	1.07E-04	0.001449424
8104570	54491	*family with sequence similarity 105, member A*	*FAM105A*	5.63	5.55	7.69	1.07E-04	0.001449424
8042487	64395	*germ cell-less homolog 1 (Drosophila)*	*GMCL1*	7.83	7.90	9.20	1.07E-04	0.001449424
7926545	84898	*plexin domain containing 2*	*PLXDC2*	6.84	9.70	10.68	1.07E-04	0.001449424
8108424	51247	*poly(A) binding protein interacting protein 2*	*PAIP2*	10.77	10.66	12.09	1.07E-04	0.00145518
8115196	91975	*zinc finger protein 300*	*ZNF300*	6.73	6.22	8.78	1.07E-04	0.001455728

8106923	7025	*nuclear receptor subfamily 2, group F, member 1*	*NR2F1*	8.57	9.64	7.46	1.07E-04	0.001456183
8099685	55203	*leucine-rich repeat LGI family, member 2*	*LGI2*	5.94	6.08	7.42	1.08E-04	0.00145638
8101340	9987	*heterogeneous nuclear ribonucleoprotein D-like*	*HNRPDL*	9.02	9.21	10.81	1.08E-04	0.001458082
7989146	55329	*meiosis-specific nuclear structural 1*	*MNS1*	6.82	6.71	7.91	1.08E-04	0.001458082
8156919	347273	*muscle-related coiled-coil protein*	*MURC*	6.02	6.01	7.15	1.08E-04	0.001458082
8149296	54984	*PIN2/TERF1 interacting, telomerase inhibitor 1*	*PINX1*	6.65	7.05	8.35	1.08E-04	0.001462036
8067602	128414	*Na+/K+ transporting ATPase interacting 4*	*NKAIN4*	6.66	6.73	7.86	1.08E-04	0.001462036
8103079	90826	*protein arginine methyltransferase 10 (putative)*	*PRMT10*	7.59	7.23	8.96	1.08E-04	0.001462143
7920100	284486	*thioesterase superfamily member 5*	*THEM5*	6.03	5.92	5.70	1.08E-04	0.001462143
8001477	267	*autocrine motility factor receptor*	*AMFR*	10.93	11.11	9.05	1.09E-04	0.001464462
8172035	6990	*dynein, light chain, Tctex-type 3*	*DYNLT3*	8.82	8.55	7.14	1.09E-04	0.001464513
8039706	10172	*zinc finger protein 256*	*ZNF256*	7.33	7.22	9.26	1.09E-04	0.001464513
7929116	10556	*ribonuclease P/MRP 30kDa subunit*	*RPP30*	8.39	8.66	10.20	1.09E-04	0.001464513
7913593	6920	*transcription elongation factor A (SII), 3*	*TCEA3*	10.18	9.31	6.68	1.09E-04	0.001465726
7945146	3762	*potassium inwardly-rectifying channel, subfamily J, member 5*	*KCNJ5*	6.62	6.63	6.11	1.09E-04	0.001467407
8171284	4935	*G protein-coupled receptor 143*	*GPR143*	6.30	6.39	8.20	1.09E-04	0.001467407
8119722	23113	*cullin 9*	*CUL9*	8.34	8.13	7.00	1.09E-04	0.001467407
7977749	56339	*methyltransferase like 3*	*METTL3*	8.85	8.80	10.68	1.09E-04	0.001467407
7991367	348110, 10239	*chromosome 15 open reading frame 38 | adaptor-related protein complex 3, sigma 2 subunit*	*C15orf38| AP3S2*	9.68	9.77	8.35	1.09E-04	0.001467407
7965855	79023	*nucleoporin 37kDa*	*NUP37*	8.07	8.16	9.68	1.09E-04	0.001468253
7989611	84191	*family with sequence similarity 96, member A*	*FAM96A*	7.76	7.86	9.34	1.09E-04	0.001468975
7939137	10480	*eukaryotic translation initiation factor 3, subunit M*	*EIF3M*	10.84	10.91	12.17	1.10E-04	0.001471082
7995803	4498, 4499	*metallothionein 1J (pseudogene) | metallothionein 1M*	*MT1JP|MT 1M*	8.09	8.15	6.30	1.10E-04	0.00147269
7923034	8707	*UDP-Gal:betaGlcNAc beta 1,3-galactosyltransferase, polypeptide 2*	*B3GALT2*	7.25	8.42	5.11	1.10E-04	0.001472932
8171170				6.39	6.50	5.77	1.10E-04	0.00147635
7917304	55283	*mucolipin 3*	*MCOLN3*	6.34	6.07	8.04	1.10E-04	0.001477519

8147424	137682	*chromosome 8 open reading frame 38*	*C8orf38*	6.31	6.41	6.99	1.10E-04	0.001477519
8062034	58476	*tumor protein p53 inducible nuclear protein 2*	*TP53INP2*	10.11	9.87	8.26	1.10E-04	0.001478485
8166230	55787	*taxilin gamma*	*TXLNG*	7.62	7.58	10.47	1.11E-04	0.001481258
8092970	347	*apolipoprotein D*	*APOD*	11.87	10.42	6.79	1.11E-04	0.001484058
8097782	6189	*ribosomal protein S3A*	*RPS3A*	12.09	12.14	13.08	1.11E-04	0.001484412
8038954	90317	*zinc finger protein 616*	*ZNF616*	6.54	6.53	8.50	1.11E-04	0.001485306
7899870	149076	*zinc finger protein 362*	*ZNF362*	10.81	10.62	9.44	1.11E-04	0.001485306
7943690	1662	*DEAD (Asp-Glu-Ala-Asp) box polypeptide 10*	*DDX10*	6.53	6.64	8.97	1.11E-04	0.001486817
8015240	85290	*keratin associated protein 4-3*	*KRTAP4-3*	6.45	6.56	6.09	1.11E-04	0.001486817
8104592	23194	*F-box and leucine-rich repeat protein 7*	*FBXL7*	9.74	10.56	8.89	1.12E-04	0.001488687
8024062	1675	*complement factor D (adipsin)*	*CFD*	10.52	8.90	7.53	1.12E-04	0.001490037
8047518	51602		*NOP58*	8.46	8.31	10.97	1.12E-04	0.001490037
8121277	202	*absent in melanoma 1*	*AIM1*	6.55	6.84	8.27	1.12E-04	0.001490501
8095773	8615		*USO1*	10.10	9.89	12.20	1.12E-04	0.001493284
7962194	440093	*H3 histone, family 3C*	*H3F3C*	10.92	11.08	11.79	1.12E-04	0.00149347
8131253	221937, 9907	*forkhead box K1 | KIAA0415*	*FOXK1|KIAA0415*	9.85	9.62	7.55	1.12E-04	0.001493931
8032249	339366	*ADAMTS-like 5*	*ADAMTSL5*	9.69	9.73	6.96	1.12E-04	0.00149424
8156199	1612	*death-associated protein kinase 1*	*DAPK1*	8.04	7.78	10.65	1.13E-04	0.001496631
7940191	6809	*syntaxin 3*	*STX3*	7.45	7.70	9.87	1.13E-04	0.001496631
8074845	140883	*zinc finger protein 280B*	*ZNF280B*	6.96	7.14	9.05	1.13E-04	0.001498499
8013015	201161	*centromere protein V*	*CENPV*	8.69	8.09	11.46	1.13E-04	0.001498499
7967149	51433	*anaphase promoting complex subunit 5*	*ANAPC5*	10.33	10.35	10.75	1.13E-04	0.001501859
8155572	548321, 100133121, 100132948	*family with sequence similarity 27, member A | family with sequence similarity 27, member B | family with sequence similarity 27, member C*	*FAM27A|FAM27B|FAM27C*	7.96	8.19	7.16	1.13E-04	0.001501859
8027642	84306	*programmed cell death 2-like*	*PDCD2L*	7.90	7.84	10.00	1.14E-04	0.001503808
8112709	134359		*POC5*	7.59	7.67	8.73	1.14E-04	0.001503808
8080909				6.84	6.98	6.50	1.14E-04	0.001509049

8111670	2668	*glial cell derived neurotrophic factor*	*GDNF*	8.04	7.99	6.67	1.14E-04	0.001509835
8069998	56683	*chromosome 21 open reading frame 59*	*C21orf59*	7.68	7.59	9.13	1.14E-04	0.001509835
7928619	728118, 729262, 283008, 728130	*family with sequence similarity 22, member A | family with sequence similarity 22, member B | family with sequence similarity 22, member E | family with sequence similarity 22, member D*	*FAM22A|FAM22B|FAM22E|FAM22D*	7.52	7.58	6.97	1.14E-04	0.001509835
8100599				5.92	5.76	5.59	1.14E-04	0.001509835
8103368				4.50	4.75	4.45	1.14E-04	0.001509835
7916274	4116	*mago-nashi homolog, proliferation-associated (Drosophila)*	*MAGOH*	7.36	7.56	8.21	1.15E-04	0.001512036
8015257	3883	*keratin 33A*	*KRT33A*	7.25	7.63	6.88	1.15E-04	0.001514641
8067983				5.70	5.95	5.60	1.15E-04	0.001515179
8095341				10.32	10.22	9.23	1.15E-04	0.00151711
7963670	7786	*mitogen-activated protein kinase kinase kinase 12*	*MAP3K12*	9.29	8.77	7.78	1.15E-04	0.001517289
7954077	57613		*KIAA1467*	7.96	8.32	9.22	1.15E-04	0.001518647
7963208				4.56	4.52	4.91	1.15E-04	0.001520015
8058857	3488	*insulin-like growth factor binding protein 5*	*IGFBP5*	13.67	11.66	7.06	1.16E-04	0.001521931
8037283	5672	*pregnancy specific beta-1-glycoprotein 4*	*PSG4*	9.67	9.22	7.98	1.16E-04	0.001524836
7941074	116085	*solute carrier family 22 (organic anion/urate transporter), member 12*	*SLC22A12*	6.84	6.94	6.48	1.16E-04	0.001524836
8058335	7341	*SMT3 suppressor of mif two 3 homolog 1 (S. cerevisiae)*	*SUMO1*	8.42	8.26	9.95	1.16E-04	0.001525595
8161044	7169	*tropomyosin 2 (beta)*	*TPM2*	12.50	12.63	10.50	1.16E-04	0.001528251
8133372	2006	*elastin*	*ELN*	9.69	10.80	7.92	1.16E-04	0.001528739
8102912	23158	*TBC1 domain family, member 9 (with GRAM domain)*	*TBC1D9*	8.60	7.59	6.17	1.17E-04	0.001532315
7915787	8503	*phosphoinositide-3-kinase, regulatory subunit 3 (gamma)*	*PIK3R3*	7.08	6.44	8.53	1.17E-04	0.001534491
7919815	1513	*cathepsin K*	*CTSK*	11.95	11.51	7.08	1.17E-04	0.001537001
8040440	348654	*Gen homolog 1, endonuclease (Drosophila)*	*GEN1*	6.88	6.84	9.31	1.17E-04	0.001537001
7991828				8.00	8.03	7.30	1.17E-04	0.001537363
8135718	54556	*inhibitor of growth family, member 3*	*ING3*	7.68	7.72	8.99	1.18E-04	0.001538788

7980833	55671	*SMEK homolog 1, suppressor of mek1 (Dictyostelium)*	*SMEK1*	7.41	7.38	9.35	1.18E-04	0.001538788
8039273	148170	*CDC42 effector protein (Rho GTPase binding) 5*	*CDC42EP 5*	9.78	9.64	8.08	1.18E-04	0.001539051
8152597	28998	*mitochondrial ribosomal protein L13*	*MRPL13*	6.24	6.25	7.12	1.18E-04	0.001541155
8064879	51605	*tRNA methyltransferase 6 homolog (S. cerevisiae)*	*TRMT6*	7.45	7.80	9.74	1.18E-04	0.001542436
7988348				4.45	4.36	4.40	1.18E-04	0.001543151
7936249	114815	*sortilin-related VPS10 domain containing receptor 1*	*SORCS1*	6.04	5.97	6.47	1.18E-04	0.001544162
8129108	221302	*zinc finger with UFM1-specific peptidase domain*	*ZUFSP*	5.90	5.93	6.72	1.18E-04	0.001544624
7955331	6602	*SWI/SNF related, matrix associated, actin dependent regulator of chromatin, subfamily d, member 1*	*SMARCD1*	9.92	9.98	11.03	1.19E-04	0.001548583
8018816				8.00	8.21	7.14	1.19E-04	0.001549993
8165974	1183	*chloride channel 4*	*CLCN4*	6.31	6.26	6.76	1.19E-04	0.001551988
8138745	3204	*homeobox A7*	*HOXA7*	6.61	8.79	6.56	1.19E-04	0.001551988
7908169	6045	*ring finger protein 2*	*RNF2*	8.81	8.90	10.12	1.19E-04	0.001551988
7983335	51496	*CTD (carboxy-terminal domain, RNA polymerase II, polypeptide A) small phosphatase like 2*	*CTDSPL2*	9.38	9.33	10.97	1.19E-04	0.001551988
7969861	9358	*integrin, beta-like 1 (with EGF-like repeat domains)*	*ITGBL1*	10.09	11.13	6.33	1.19E-04	0.001553305
8040792	11117	*elastin microfibril interfacer 1*	*EMILIN1*	9.36	9.23	7.26	1.20E-04	0.001553305
8050443	79677	*structural maintenance of chromosomes 6*	*SMC6*	7.87	7.83	9.85	1.19E-04	0.001553305
8039586	147948	*zinc finger protein 582*	*ZNF582*	6.55	6.43	7.06	1.20E-04	0.001553305
8135990	2318	*filamin C, gamma*	*FLNC*	11.49	11.91	9.23	1.20E-04	0.001553371
8051066	4358	*MpV17 mitochondrial inner membrane protein*	*MPV17*	13.01	12.90	12.13	1.20E-04	0.001553371
8094938	152519	*NIPA-like domain containing 1*	*NIPAL1*	6.48	6.46	8.05	1.20E-04	0.001553371
7990928	440299, 440297, 440300, 80154, 727849, 100288549	*DNM1 pseudogene 41 | chondroitin sulfate proteoglycan 4 pseudogene | hypothetical LOC80154 | golgin A2 pseudogene | hypothetical LOC100288549*	*DNM1P41|LOC440297|LOC440300|LOC80154|LOC727849|LOC100288549*	7.70	7.70	6.78	1.20E-04	0.001553371
8085676	1618	*deleted in azoospermia-like*	*DAZL*	5.83	5.76	7.24	1.20E-04	0.001554821

8020183	3613	*inositol(myo)-1(or 4)-monophosphatase 2*	*IMPA2*	6.95	7.39	9.03	1.20E-04	0.001554821
7951397	834	*caspase 1, apoptosis-related cysteine peptidase (interleukin 1, beta, convertase)*	*CASP1*	7.12	5.58	4.74	1.20E-04	0.001556879
8103520	391712	*tripartite motif-containing 61*	*TRIM61*	7.73	8.12	8.69	1.20E-04	0.001556879
7932433	221078	*NOP2/Sun domain family, member 6*	*NSUN6*	6.27	6.15	8.50	1.20E-04	0.001557685
8052526	7514	*exportin 1 (CRM1 homolog, yeast)*	*XPO1*	8.73	8.57	11.54	1.21E-04	0.00155913
8155268	64425	*polymerase (RNA) I polypeptide E, 53kDa*	*POLR1E*	8.86	8.82	9.67	1.21E-04	0.001559234
8094719	55728	*NEDD4 binding protein 2*	*N4BP2*	5.76	5.54	8.96	1.21E-04	0.001559834
8059708	654321	*small nucleolar RNA, H/ACA box 75*	*SNORA75*	6.91	6.92	8.98	1.21E-04	0.001560619
8055060	55339	*WD repeat domain 33*	*WDR33*	8.59	8.59	10.14	1.21E-04	0.001561245
7995442	123970	*chromosome 16 open reading frame 78*	*C16orf78*	5.44	5.67	5.21	1.21E-04	0.001561686
7949679	6712	*spectrin, beta, non-erythrocytic 2*	*SPTBN2*	6.76	6.88	8.46	1.21E-04	0.001564558
8141050	6189	*ribosomal protein S3A*	*RPS3A*	10.39	10.41	11.84	1.22E-04	0.001567051
8057689	64172	*O-sialoglycoprotein endopeptidase-like 1*	*OSGEPL1*	6.15	6.14	8.47	1.22E-04	0.001567051
7951662	1410	*crystallin, alpha B*	*CRYAB*	11.77	11.79	8.22	1.22E-04	0.001567055
8055377	84083	*zinc finger, RAN-binding domain containing 3*	*ZRANB3*	6.59	6.40	8.13	1.22E-04	0.001567055
8152522	5168	*ectonucleotide pyrophosphatase/phosphodiesterase 2*	*ENPP2*	10.67	11.69	8.22	1.22E-04	0.001569278
8039340	7138	*troponin T type 1 (skeletal, slow)*	*TNNT1*	7.61	7.47	8.61	1.22E-04	0.001569278
8045776	114805	*UDP-N-acetyl-alpha-D-galactosamine:polypeptide N-acetylgalactosaminyltransferase 13 (GalNAc-T13)*	*GALNT13*	5.42	5.53	7.66	1.22E-04	0.001571485
7926596	23412, 648	*COMM domain containing 3 | BMI1 polycomb ring finger oncogene*	*COMMD3|BMI1*	8.84	9.07	7.26	1.22E-04	0.001571485
7970858	3146	*high-mobility group box 1*	*HMGB1*	6.90	6.50	8.45	1.22E-04	0.001572317
7980304				6.43	6.44	5.53	1.23E-04	0.001582784
7971915	100128202		*FLJ25694*	7.46	7.26	6.63	1.24E-04	0.001585322
7903490	653149, 148545, 284610	*neuroblastoma breakpoint family, member 6 | neuroblastoma breakpoint family, member 4 | neuroblastoma breakpoint family, member 5*	*NBPF6|NBPF4|NBPF5*	6.61	6.49	6.16	1.24E-04	0.001590392
8115476	9443	*mediator complex subunit 7*	*MED7*	7.26	7.45	8.32	1.24E-04	0.001592283
7974603	5684	*proteasome (prosome, macropain) subunit, alpha type, 3*	*PSMA3*	9.20	9.39	10.83	1.24E-04	0.001594549

7999387	2013	*epithelial membrane protein 2*	*EMP2*	10.20	10.67	9.02	1.25E-04	0.001598967
8097417	79960	*PHD finger protein 17*	*PHF17*	8.58	8.51	10.83	1.25E-04	0.001598967
8070046	94104	*GC-rich sequence DNA-binding factor 1*	*GCFC1*	6.73	6.57	9.28	1.25E-04	0.001598967
7985317	57214		*KIAA1199*	11.71	11.05	5.84	1.25E-04	0.001599111
8154151	403313	*phosphatidic acid phosphatase type 2 domain containing 2*	*PPAPDC2*	7.40	7.56	8.34	1.25E-04	0.001602235
8034130	25959	*KN motif and ankyrin repeat domains 2*	*KANK2*	10.36	10.12	8.18	1.25E-04	0.001603065
7945831	114879	*oxysterol binding protein-like 5*	*OSBPL5*	9.23	9.22	6.69	1.26E-04	0.001606278
8109305	11346	*synaptopodin*	*SYNPO*	9.12	9.30	7.14	1.26E-04	0.001611593
7941714	79703, 9986	*chromosome 11 open reading frame 80 | RCE1 homolog, prenyl protein peptidase (S. cerevisiae)*	*C11orf80| RCE1*	7.76	7.86	9.81	1.26E-04	0.001614377
8024557	2767	*guanine nucleotide binding protein (G protein), alpha 11 (Gq class)*	*GNA11*	11.40	11.35	9.36	1.27E-04	0.001615966
8138640	54205	*cytochrome c, somatic*	*CYCS*	7.78	8.12	9.86	1.27E-04	0.00162038
8051882	10128	*leucine-rich PPR-motif containing*	*LRPPRC*	8.89	8.60	11.47	1.27E-04	0.001622001
8038225	57664	*pleckstrin homology domain containing, family A (phosphoinositide binding specific) member 4*	*PLEKHA4*	8.70	8.61	7.75	1.27E-04	0.001623547
8041888	2956	*mutS homolog 6 (E. coli)*	*MSH6*	6.88	6.88	10.14	1.28E-04	0.001625471
8145151	10174	*sorbin and SH3 domain containing 3*	*SORBS3*	10.14	9.85	7.83	1.28E-04	0.00162589
7987385	4212	*Meis homeobox 2*	*MEIS2*	9.10	9.14	6.13	1.28E-04	0.001626793
7901299	51727	*cytidine monophosphate (UMP-CMP) kinase 1, cytosolic*	*CMPK1*	9.92	9.94	8.74	1.29E-04	0.001636925
8006325	23512	*suppressor of zeste 12 homolog (Drosophila)*	*SUZ12*	8.97	8.81	10.32	1.29E-04	0.001640635
8011027	4641	*myosin IC*	*MYO1C*	12.07	11.97	9.92	1.29E-04	0.001640645
7985418	440299, 440297, 440300, 80154, 727849, 100288549	*DNM1 pseudogene 41 | chondroitin sulfate proteoglycan 4 pseudogene | hypothetical LOC80154 | golgin A2 pseudogene | hypothetical LOC100288549*	*DNM1P41|LOC440297|LOC440300|LOC80154|LOC727849|LOC100288549*	7.69	7.70	6.79	1.29E-04	0.001640645
7914015	115572	*family with sequence similarity 46, member B*	*FAM46B*	8.20	8.33	10.73	1.29E-04	0.001642583

8107646	93166	*PR domain containing 6*	*PRDM6*	7.88	6.95	6.42	1.30E-04	0.001644144
7978586	1073	*cofilin 2 (muscle)*	*CFL2*	10.52	10.68	9.30	1.30E-04	0.001644896
8127824	90025	*ubiquitin-conjugating enzyme E2C binding protein*	*UBE2CBP*	6.52	6.72	7.75	1.30E-04	0.001644896
7960158	7556, 10795	*zinc finger protein 10 | zinc finger protein 268*	*ZNF10|ZNF268*	5.37	5.39	6.73	1.30E-04	0.001644896
8090637				7.65	7.27	6.97	1.30E-04	0.001644896
8148029	10584	*collectin sub-family member 10 (C-type lectin)*	*COLEC10*	7.11	5.90	5.59	1.30E-04	0.001645461
7911403	8784	*tumor necrosis factor receptor superfamily, member 18*	*TNFRSF18*	8.13	8.21	7.52	1.30E-04	0.001646021
7940857	10963	*stress-induced-phosphoprotein 1*	*STIP1*	9.58	9.90	11.27	1.30E-04	0.001650233
7900157	6232	*ribosomal protein S27*	*RPS27*	12.90	12.93	12.64	1.30E-04	0.00165025
8168794	2491	*centromere protein I*	*CENPI*	7.08	7.17	9.70	1.31E-04	0.001651064
7915032	284654	*R-spondin homolog (Xenopus laevis)*	*RSPO1*	9.45	8.19	7.22	1.31E-04	0.001651064
7985459	440299, 440297, 440300, 80154, 727849, 100288549	*DNM1 pseudogene 41 | chondroitin sulfate proteoglycan 4 pseudogene | hypothetical LOC80154 | golgin A2 pseudogene | hypothetical LOC100288549*	*DNM1P41|LOC440297|LOC440300|LOC80154|LOC727849|LOC100288549*	7.69	7.70	6.79	1.31E-04	0.001651064
8147654	5440	*polymerase (RNA) II (DNA directed) polypeptide K, 7.0kDa*	*POLR2K*	10.71	11.01	11.62	1.31E-04	0.001656587
7941236	10435	*CDC42 effector protein (Rho GTPase binding) 2*	*CDC42EP2*	10.79	10.44	8.59	1.31E-04	0.001656587
8023392	677819	*small nucleolar RNA, H/ACA box 37*	*SNORA37*	9.26	9.72	8.51	1.31E-04	0.001656587
7985023				7.84	7.88	6.77	1.31E-04	0.001656587
7937774	51214	*insulin-like growth factor 2 antisense*	*IGF2AS*	7.72	7.51	6.79	1.31E-04	0.001656782
7915846	8569	*MAP kinase interacting serine/threonine kinase 1*	*MKNK1*	10.17	9.78	8.96	1.32E-04	0.001659694
8037474	7733	*zinc finger protein 180*	*ZNF180*	6.50	6.46	7.28	1.32E-04	0.001662402
8020847	1837	*dystrobrevin, alpha*	*DTNA*	6.35	6.15	8.76	1.32E-04	0.001666528
7999412	780776	*family with sequence similarity 18, member A*	*FAM18A*	6.76	6.83	7.82	1.32E-04	0.001666528

7978710				5.65	5.56	4.96	1.32E-04	0.001666528
7923426	29089	*ubiquitin-conjugating enzyme E2T (putative)*	*UBE2T*	8.00	8.50	10.87	1.33E-04	0.001667605
8074335	5625	*proline dehydrogenase (oxidase) 1*	*PRODH*	6.48	6.69	8.64	1.33E-04	0.0016685
7933080				7.88	7.71	7.01	1.33E-04	0.0016685
8006940	2886	*growth factor receptor-bound protein 7*	*GRB7*	7.07	7.14	8.68	1.33E-04	0.001676147
7946661	27122	*dickkopf homolog 3 (Xenopus laevis)*	*DKK3*	11.82	12.39	9.34	1.34E-04	0.001676147
8156905	8577	*transmembrane protein with EGF-like and two follistatin-like domains 1*	*TMEFF1*	8.70	8.23	11.85	1.34E-04	0.001676551
8019737	3838	*karyopherin alpha 2 (RAG cohort 1, importin alpha 1)*	*KPNA2*	10.17	10.66	13.14	1.34E-04	0.001678443
8009533	9382, 84923	*component of oligomeric golgi complex 1 | family with sequence similarity 104, member A*	*COG1|FAM104A*	10.30	10.40	9.28	1.34E-04	0.001678443
8150266	27257		*LSM1*	8.63	8.93	9.51	1.34E-04	0.001681764
7917503	2635	*guanylate binding protein 3*	*GBP3*	7.61	7.05	5.31	1.34E-04	0.001682592
8021716	29090	*chromosome 18 open reading frame 55*	*C18orf55*	7.28	7.44	9.02	1.35E-04	0.001692967
8179950	80863	*proline-rich transmembrane protein 1*	*PRRT1*	8.36	7.97	6.95	1.36E-04	0.001698135
8062490	677837	*small nucleolar RNA, H/ACA box 60*	*SNORA60*	7.88	8.16	7.10	1.36E-04	0.001698135
8115847				11.68	11.89	10.28	1.36E-04	0.001698135
8076176	23466	*chromobox homolog 6*	*CBX6*	10.72	10.26	8.47	1.36E-04	0.001701337
7903753	2946, 2948	*glutathione S-transferase mu 2 (muscle) | glutathione S-transferase mu 4*	*GSTM2|GSTM4*	11.15	10.49	8.47	1.36E-04	0.001703704
8102321	81579	*phospholipase A2, group XIIA*	*PLA2G12A*	8.81	8.98	9.82	1.37E-04	0.001704504
8117630	7718	*zinc finger protein 165*	*ZNF165*	6.00	5.95	7.29	1.37E-04	0.001712504
7917875	2152	*coagulation factor III (thromboplastin, tissue factor)*	*F3*	8.52	9.05	7.47	1.37E-04	0.001714467
7956876	84298	*LLP homolog, long-term synaptic facilitation (Aplysia)*	*LLPH*	5.33	5.38	6.67	1.38E-04	0.001714808
8043349	64682	*anaphase promoting complex subunit 1*	*ANAPC1*	6.83	7.06	9.33	1.38E-04	0.001716022
8151223	81796	*solute carrier organic anion transporter family, member 5A1*	*SLCO5A1*	6.05	6.17	7.00	1.38E-04	0.001716341
8174598	3598	*interleukin 13 receptor, alpha 2*	*IL13RA2*	5.42	6.05	4.81	1.38E-04	0.001717221
7931643	1571	*cytochrome P450, family 2, subfamily E, polypeptide 1*	*CYP2E1*	6.79	6.91	6.25	1.38E-04	0.001718772
7903188	58155	*polypyrimidine tract binding protein 2*	*PTBP2*	8.07	7.90	11.21	1.38E-04	0.001718772

7907486	84614	*zinc finger and BTB domain containing 37*	*ZBTB37*	6.61	6.77	8.32	1.38E-04	0.001718772
8143663	2146	*enhancer of zeste homolog 2 (Drosophila)*	*EZH2*	7.05	7.51	9.98	1.39E-04	0.001725789
8130993	56975	*family with sequence similarity 20, member C*	*FAM20C*	12.08	11.74	9.00	1.39E-04	0.001725789
8178754	80863	*proline-rich transmembrane protein 1*	*PRRT1*	8.36	7.97	6.95	1.39E-04	0.001725789
7950990	120103	*solute carrier family 36 (proton/amino acid symporter), member 4*	*SLC36A4*	6.49	6.67	8.51	1.39E-04	0.001725789
8102141	27068	*pyrophosphatase (inorganic) 2*	*PPA2*	7.02	7.10	8.08	1.39E-04	0.001727502
7936494	2674	*GDNF family receptor alpha 1*	*GFRA1*	10.92	9.47	7.65	1.39E-04	0.001729069
8008547	10040, 1353	*target of myb1 (chicken)-like 1 | COX11 cytochrome c oxidase assembly homolog (yeast)*	*TOM1L1|COX11*	9.08	8.12	10.01	1.39E-04	0.001729069
8013305	729288, 57335, 353149	*zinc finger protein 286B | zinc finger protein 286A | TBC1 domain family, member 26*	*ZNF286B| ZNF286A| TBC1D26*	6.03	6.06	8.91	1.39E-04	0.001729069
7944365	338657	*coiled-coil domain containing 84*	*CCDC84*	8.75	8.73	9.99	1.40E-04	0.0017346
7946454	57758	*signal peptide, CUB domain, EGF-like 2*	*SCUBE2*	8.26	7.85	6.22	1.40E-04	0.001736942
8146914	7013	*telomeric repeat binding factor (NIMA-interacting) 1*	*TERF1*	9.59	9.04	12.95	1.41E-04	0.001742356
8164304	30815, 100131355	*ST6 (alpha-N-acetyl-neuraminyl-2,3-beta-galactosyl-1,3)-N-acetylgalactosaminide alpha-2,6-sialyltransferase 6 | hypothetical protein LOC100131355*	*ST6GALNAC6|LOC100131355*	11.29	11.11	9.58	1.41E-04	0.001742356
7961390	50865	*heme binding protein 1*	*HEBP1*	11.50	11.22	9.80	1.42E-04	0.001749779
8027793	51599	*lipolysis stimulated lipoprotein receptor*	*LSR*	8.26	8.22	10.10	1.41E-04	0.001749779
8079707	25915	*NADH dehydrogenase (ubiquinone) 1 alpha subcomplex, assembly factor 3*	*NDUFAF3*	11.17	11.14	9.59	1.42E-04	0.001753409
8158539	414318	*chromosome 9 open reading frame 106*	*C9orf106*	7.69	7.25	6.31	1.42E-04	0.001758193
7997940	606500, 6137	*small nucleolar RNA, C/D box 68 | ribosomal protein L13*	*SNORD68| RPL13*	10.34	10.54	9.59	1.42E-04	0.001758422
7942674	25987	*tsukushi small leucine rich proteoglycan homolog (Xenopus laevis)*	*TSKU*	11.21	11.06	9.83	1.43E-04	0.001761512
8157264	1318	*solute carrier family 31 (copper transporters), member 2*	*SLC31A2*	9.32	9.92	7.49	1.43E-04	0.001762596
8134257	2791	*guanine nucleotide binding protein (G protein), gamma 11*	*GNG11*	11.52	11.04	8.00	1.43E-04	0.001762596

8037005	7040	*transforming growth factor, beta 1*	*TGFB1*	10.78	11.03	8.59	1.43E-04	0.001762596
8132092	11185	*indolethylamine N-methyltransferase*	*INMT*	8.96	7.69	6.77	1.44E-04	0.001768593
8007446	3430	*interferon-induced protein 35*	*IFI35*	10.59	9.86	7.67	1.44E-04	0.001769032
7925550	159	*adenylosuccinate synthase*	*ADSS*	8.04	8.11	10.05	1.44E-04	0.001771632
7940561	2237	*flap structure-specific endonuclease 1*	*FEN1*	7.39	7.59	9.32	1.44E-04	0.001772204
8163962	347169	*olfactory receptor, family 1, subfamily B, member 1*	*OR1B1*	6.19	6.32	5.85	1.44E-04	0.001772204
8086008	51143	*dynein, cytoplasmic 1, light intermediate chain 1*	*DYNC1LI1*	9.06	9.15	10.66	1.44E-04	0.001774629
7966738	79794	*chromosome 12 open reading frame 49*	*C12orf49*	9.68	9.74	10.31	1.44E-04	0.001774629
8114787	10007	*glucosamine-6-phosphate deaminase 1*	*GNPDA1*	9.23	9.07	10.55	1.45E-04	0.001775459
8091354	6596	*helicase-like transcription factor*	*HLTF*	8.09	7.68	9.99	1.45E-04	0.001777038
8168163	54857	*glycerophosphodiester phosphodiesterase domain containing 2*	*GDPD2*	5.67	5.81	7.33	1.45E-04	0.001777038
8097647	6059	*ATP-binding cassette, sub-family E (OABP), member 1*	*ABCE1*	8.16	8.16	10.25	1.45E-04	0.001778111
8127031	4172	*minichromosome maintenance complex component 3*	*MCM3*	8.48	8.93	11.82	1.46E-04	0.001783895
8155442	375719, 364	*aquaporin 7 pseudogene 1*	*AQP7P1*	6.60	6.61	6.07	1.46E-04	0.001783895
8158554	51450	*paired related homeobox 2*	*PRRX2*	9.95	10.12	7.84	1.46E-04	0.001786227
8124798	170954		*KIAA1949*	9.25	9.61	7.23	1.46E-04	0.001788513
8019486	6398	*secreted and transmembrane 1*	*SECTM1*	12.64	9.53	6.68	1.46E-04	0.001788516
7913249	65018	*PTEN induced putative kinase 1*	*PINK1*	7.88	7.89	6.64	1.46E-04	0.001788516
8019507	79415	*chromosome 17 open reading frame 62*	*C17orf62*	9.88	9.62	8.56	1.46E-04	0.001791236
7983828	374618	*testis expressed 9*	*TEX9*	5.84	5.93	7.77	1.46E-04	0.001791264
7993756	6296, 112479	*acyl-CoA synthetase medium-chain family member 3 | ERI1 exoribonuclease family member 2*	*ACSM3|ERI2*	5.80	5.55	7.57	1.47E-04	0.001791498
7909102				6.03	6.18	7.13	1.47E-04	0.001792628
8176133	2539	*glucose-6-phosphate dehydrogenase*	*G6PD*	12.28	10.90	9.49	1.48E-04	0.001803338
8133721	3315	*heat shock 27kDa protein 1*	*HSPB1*	13.61	13.71	12.35	1.48E-04	0.001803338
7951838	8882	*zinc finger protein 259*	*ZNF259*	9.00	9.51	10.14	1.48E-04	0.001803338
7952830	23310	*non-SMC condensin II complex, subunit D3*	*NCAPD3*	7.61	7.94	9.85	1.48E-04	0.001805413
8075695	80833	*apolipoprotein L, 3*	*APOL3*	7.53	7.05	5.91	1.48E-04	0.001806387

8093130	165918	*ring finger protein 168*	*RNF168*	7.33	7.47	9.37	1.49E-04	0.001812961
7951372	837	*caspase 4, apoptosis-related cysteine peptidase*	*CASP4*	9.07	8.83	5.47	1.49E-04	0.001815183
7951271	4312	*matrix metallopeptidase 1 (interstitial collagenase)*	*MMP1*	12.10	10.54	5.99	1.49E-04	0.001815183
7964722	11197	*WNT inhibitory factor 1*	*WIF1*	5.69	5.69	7.66	1.49E-04	0.001815183
8116177	85007	*alanine-glyoxylate aminotransferase 2-like 2*	*AGXT2L2*	9.10	8.90	7.48	1.50E-04	0.001819741
8176935	9081, 442862	*PTPN13-like, Y-linked | PTPN13-like, Y-linked 2*	*PRY|PRY2*	6.01	6.06	5.58	1.50E-04	0.001819741
7974164				5.42	5.21	6.34	1.50E-04	0.001819835
8052091	9378	*neurexin 1*	*NRXN1*	5.75	5.80	7.49	1.50E-04	0.001820223
8141066	5446	*paraoxonase 3*	*PON3*	5.83	5.78	7.60	1.50E-04	0.001821553
8082314	5361	*plexin A1*	*PLXNA1*	11.02	11.14	9.19	1.50E-04	0.001824768
7939590	55343	*solute carrier family 35, member C1*	*SLC35C1*	10.24	9.85	8.02	1.50E-04	0.001824768
8000600				7.79	7.84	7.24	1.50E-04	0.001824768
8041745	79823	*chromosome 2 open reading frame 34*	*C2orf34*	8.45	7.49	9.87	1.51E-04	0.001826253
8082229	7372	*uridine monophosphate synthetase*	*UMPS*	7.65	7.96	10.15	1.51E-04	0.001827825
7898957	11123	*RCAN family member 3*	*RCAN3*	8.24	8.57	9.25	1.51E-04	0.001828594
7951654	91893, 79796	*ferredoxin-fold anticodon binding domain containing 1 | asparagine-linked glycosylation 9, alpha-1,2-mannosyltransferase homolog (S. cerevisiae)*	*FDXACB1|ALG9*	5.90	6.20	6.77	1.51E-04	0.001828594
8008912				6.00	6.15	5.63	1.51E-04	0.001828594
7925954	10276	*neuroepithelial cell transforming 1*	*NET1*	7.34	7.55	9.87	1.51E-04	0.0018291
8103222				12.33	12.41	13.22	1.51E-04	0.0018291
8095806	419	*ADP-ribosyltransferase 3*	*ART3*	5.32	5.10	6.04	1.52E-04	0.001830416
7965523	7181	*nuclear receptor subfamily 2, group C, member 1*	*NR2C1*	8.41	8.33	9.59	1.51E-04	0.001830416
7955119	121273	*chromosome 12 open reading frame 54*	*C12orf54*	5.69	5.76	5.36	1.52E-04	0.001830416
8164464	10444	*zer-1 homolog (C. elegans)*	*ZER1*	9.76	9.76	8.03	1.52E-04	0.001832796
7928531	100131213	*non-protein coding RNA 245*	*NCRNA00245*	7.81	7.65	6.46	1.52E-04	0.001834362
7917516	2633, 2635	*guanylate binding protein 1, interferon-inducible, 67kDa | guanylate binding protein 3*	*GBP1|GBP3*	9.62	9.67	5.61	1.52E-04	0.001834917

8048120	471	*5-aminoimidazole-4-carboxamide ribonucleotide formyltransferase/IMP cyclohydrolase*	*ATIC*	10.22	10.02	11.81	1.52E-04	0.00183688
8121152	10690	*fucosyltransferase 9 (alpha (1,3) fucosyltransferase)*	*FUT9*	4.95	5.02	6.01	1.52E-04	0.00183688
8160849	138715	*AT rich interactive domain 3C (BRIGHT-like)*	*ARID3C*	6.61	6.69	5.85	1.53E-04	0.001837878
7922416	692195	*small nucleolar RNA, C/D box 75*	*SNORD75*	7.06	7.20	9.68	1.53E-04	0.001837878
8043621	10865	*AT rich interactive domain 5A (MRF1-like)*	*ARID5A*	7.94	8.10	6.65	1.53E-04	0.00184082
8127051	9697	*translocation associated membrane protein 2*	*TRAM2*	12.48	12.60	10.25	1.53E-04	0.001842884
7964262	4666	*nascent polypeptide-associated complex alpha subunit*	*NACA*	10.96	10.96	11.69	1.53E-04	0.001843354
7995806	4489	*metallothionein 1A*	*MT1A*	9.28	9.22	7.44	1.54E-04	0.001846905
7945460	406992	*microRNA 210*	*MIR210*	9.06	9.10	8.25	1.54E-04	0.001846905
8160431	554202		*LOC554202*	8.71	10.28	6.12	1.54E-04	0.001846905
7981996	100033435	*small nucleolar RNA, C/D box 116-24*	*SNORD116-24*	11.53	11.70	12.32	1.54E-04	0.001846905
7908694	89796	*neuron navigator 1*	*NAV1*	10.96	11.18	8.70	1.54E-04	0.00185069
8138442	7291	*twist homolog 1 (Drosophila)*	*TWIST1*	11.40	10.99	8.29	1.55E-04	0.001854594
8150870	5706	*proteasome (prosome, macropain) 26S subunit, ATPase, 6*	*PSMC6*	8.25	8.33	9.81	1.55E-04	0.001857025
8073007	23764	*v-maf musculoaponeurotic fibrosarcoma oncogene homolog F (avian)*	*MAFF*	8.31	8.58	7.26	1.55E-04	0.001862783
8141133	7979	*split hand/foot malformation (ectrodactyly) type 1*	*SHFM1*	9.33	9.32	10.53	1.56E-04	0.001863718
7901557	63948	*DMRT-like family B with proline-rich C-terminal, 1*	*DMRTB1*	7.76	7.80	7.22	1.56E-04	0.001863718
8082380	80325	*ankyrin repeat and BTB (POZ) domain containing 1*	*ABTB1*	8.46	8.39	7.06	1.56E-04	0.001863718
7902345	127253	*tRNA-yW synthesizing protein 3 homolog (S. cerevisiae)*	*TYW3*	8.33	8.29	9.70	1.56E-04	0.001863718
8152642	100131726	*HCC-related HCC-C11_v3*	*LOC100131726*	6.34	6.29	5.96	1.56E-04	0.001863718
7961964	55726	*chromosome 12 open reading frame 11*	*C12orf11*	7.85	7.83	9.78	1.56E-04	0.001864485
7909494	255928	*synaptotagmin XIV*	*SYT14*	7.57	5.91	9.31	1.57E-04	0.001871969
8135464	1738	*dihydrolipoamide dehydrogenase*	*DLD*	8.14	8.00	9.92	1.57E-04	0.001874846
7905589	26097	*chromosome 1 open reading frame 77*	*C1orf77*	8.84	9.00	9.77	1.57E-04	0.00187504
8038683	5653	*kallikrein-related peptidase 6*	*KLK6*	6.53	6.59	7.77	1.57E-04	0.001875383

8171758	100158262	*small Cajal body-specific RNA 9-like (retrotransposed)*	*SCARNA9L*	5.31	5.81	7.64	1.57E-04	0.001875383
7913357	1889	*endothelin converting enzyme 1*	*ECE1*	11.71	11.17	9.93	1.57E-04	0.001876893
8024712	85300	*ataxia, cerebellar, Cayman type*	*ATCAY*	7.14	7.17	8.88	1.57E-04	0.001876931
7948718	256364	*echinoderm microtubule associated protein like 3*	*EML3*	9.07	9.03	7.49	1.57E-04	0.001876931
8031748	388567	*zinc finger protein 749*	*ZNF749*	6.07	6.14	8.01	1.58E-04	0.001876931
8039692	730051, 79818, 84914	*zinc finger protein 814 | zinc finger protein 552 | zinc finger protein 587*	*ZNF814|ZNF552|ZNF587*	7.47	7.60	10.11	1.58E-04	0.001876931
7962659	51564	*histone deacetylase 7*	*HDAC7*	9.54	9.42	8.06	1.58E-04	0.001877938
8108954	10915	*transcription elongation regulator 1*	*TCERG1*	8.24	8.21	10.16	1.58E-04	0.001879396
8149069				6.25	6.26	5.66	1.58E-04	0.001879396
8085431	23225	*nucleoporin 210kDa*	*NUP210*	6.51	6.59	8.88	1.58E-04	0.001880462
7985089	5955	*reticulocalbin 2, EF-hand calcium binding domain*	*RCN2*	9.30	9.44	11.37	1.58E-04	0.001880616
7989069	26108	*pygopus homolog 1 (Drosophila)*	*PYGO1*	8.47	8.30	6.48	1.58E-04	0.00188108
8146130	84296	*GINS complex subunit 4 (Sld5 homolog)*	*GINS4*	6.42	6.93	9.42	1.59E-04	0.001882715
7996345	8824	*carboxylesterase 2*	*CES2*	9.48	9.45	7.70	1.59E-04	0.001883299
8136401	23165	*nucleoporin 205kDa*	*NUP205*	7.49	7.75	10.93	1.59E-04	0.001883972
8015846	4356	*membrane protein, palmitoylated 3 (MAGUK p55 subfamily member 3)*	*MPP3*	7.63	7.83	7.13	1.59E-04	0.001887648
8156126	80010		*RMI1*	6.45	6.49	8.80	1.59E-04	0.001887648
8142697	25913	*protection of telomeres 1 homolog (S. pombe)*	*POT1*	7.69	7.58	8.84	1.59E-04	0.001888676
8000310	124454	*glutamyl-tRNA synthetase 2, mitochondrial (putative)*	*EARS2*	8.27	8.35	9.22	1.59E-04	0.001888676
8009417	3838	*karyopherin alpha 2 (RAG cohort 1, importin alpha 1)*	*KPNA2*	10.19	10.69	13.20	1.60E-04	0.001890576
8047788	8745	*ADAM metallopeptidase domain 23*	*ADAM23*	6.92	7.34	8.96	1.60E-04	0.001891435
8098707	3323	*heat shock protein 90kDa alpha (cytosolic), class A member 4 (pseudogene)*	*HSP90AA4P*	6.36	6.36	7.50	1.60E-04	0.001896082
8160839	11258	*dynactin 3 (p22)*	*DCTN3*	11.02	10.74	9.02	1.60E-04	0.001896082
8070826	3689	*integrin, beta 2 (complement component 3 receptor 3 and 4 subunit)*	*ITGB2*	7.09	7.73	6.62	1.61E-04	0.001897758
8133809	222194	*round spermatid basic protein 1-like*	*RSBN1L*	6.77	6.79	8.16	1.61E-04	0.001897758

8172708	55190	*nudix (nucleoside diphosphate linked moiety X)-type motif 11*	*NUDT11*	7.14	7.30	8.86	1.61E-04	0.001900448
8030978	91664	*zinc finger protein 845*	*ZNF845*	6.74	6.60	9.28	1.61E-04	0.001901576
8057441	57703		*CWC22*	7.83	7.70	9.33	1.61E-04	0.001901587
8107307	814	*calcium/calmodulin-dependent protein kinase IV*	*CAMK4*	6.16	6.33	7.38	1.61E-04	0.001904002
8141140	1749	*distal-less homeobox 5*	*DLX5*	8.71	6.73	6.36	1.62E-04	0.00190557
8100179	152518	*nuclear transcription factor, X-box binding-like 1*	*NFXL1*	6.83	6.59	8.98	1.62E-04	0.00190557
8152335	157753	*transmembrane protein 74*	*TMEM74*	6.30	6.30	7.79	1.62E-04	0.00190557
8169740	3020, 440926	*H3 histone, family 3A | H3 histone, family 3A pseudogene*	*H3F3A|LOC440926*	11.00	11.21	12.85	1.62E-04	0.00190557
8127977	10492	*synaptotagmin binding, cytoplasmic RNA interacting protein*	*SYNCRIP*	9.10	9.20	10.89	1.62E-04	0.001909253
8071489	9127	*purinergic receptor P2X, ligand-gated ion channel, 6*	*P2RX6*	7.16	7.34	6.13	1.63E-04	0.001915489
8138912	23658		*LSM5*	7.14	7.48	9.06	1.63E-04	0.001915489
8011861	56919	*DEAH (Asp-Glu-Ala-His) box polypeptide 33*	*DHX33*	7.63	7.86	10.15	1.63E-04	0.001915489
7978553	171546	*chromosome 14 open reading frame 147*	*C14orf147*	7.36	7.11	7.87	1.63E-04	0.001915489
8004464	8742, 407977, 8741	*tumor necrosis factor (ligand) superfamily, member 12 | TNFSF12-TNFSF13 readthrough | tumor necrosis factor (ligand) superfamily, member 13*	*TNFSF12|TNFSF12-TNFSF13*	9.45	9.21	7.72	1.63E-04	0.001915489
7931810	1316	*Kruppel-like factor 6*	*KLF6*	8.95	9.54	8.09	1.64E-04	0.001923747
8122202	4602	*v-myb myeloblastosis viral oncogene homolog (avian)*	*MYB*	5.70	5.81	8.03	1.64E-04	0.001930292
8048304	58190	*CTD (carboxy-terminal domain, RNA polymerase II, polypeptide A) small phosphatase 1*	*CTDSP1*	10.21	10.33	7.83	1.65E-04	0.001930541
7918026	2135	*exostoses (multiple)-like 2*	*EXTL2*	6.73	6.63	9.06	1.65E-04	0.001937301
8130824	100128124		*HGC6.3*	7.29	7.36	6.66	1.65E-04	0.001937301
7965403	4060	*lumican*	*LUM*	10.80	12.10	5.65	1.66E-04	0.001939388
7999909	51704	*G protein-coupled receptor, family C, group 5, member B*	*GPRC5B*	11.58	8.37	12.06	1.65E-04	0.001939388
8006183	440423	*suppressor of zeste 12 homolog pseudogene*	*SUZ12P*	8.90	8.82	10.06	1.66E-04	0.001940778
8150509	5327	*plasminogen activator, tissue*	*PLAT*	10.11	10.82	7.93	1.66E-04	0.00194095
7994058	6340	*sodium channel, nonvoltage-gated 1, gamma*	*SCNN1G*	6.45	6.54	7.76	1.66E-04	0.001943475

8027778	53827, 100127972	*FXYD domain containing ion transport regulator 5 | hypothetical LOC100127972*	*FXYD5|LOC100127972*	12.02	12.10	10.30	1.66E-04	0.001943475
8072141				5.36	5.29	4.97	1.66E-04	0.001946495
7946228	1200	*tripeptidyl peptidase I*	*TPP1*	11.28	11.26	9.26	1.67E-04	0.001946621
8167013	9767	*PHD finger protein 16*	*PHF16*	6.84	6.74	8.63	1.67E-04	0.001946621
8006298	84440	*RAB11 family interacting protein 4 (class II)*	*RAB11FIP4*	6.89	7.10	8.25	1.67E-04	0.001950663
7950578	5058	*p21 protein (Cdc42/Rac)-activated kinase 1*	*PAK1*	9.15	8.96	11.20	1.67E-04	0.001952101
8088915				4.47	4.78	6.49	1.68E-04	0.001955444
8103431	201725	*chromosome 4 open reading frame 46*	*C4orf46*	8.11	8.09	9.57	1.68E-04	0.001955819
8030842	407056	*microRNA 99b*	*MIR99B*	8.08	8.16	7.36	1.68E-04	0.00195758
8085529				4.51	4.55	4.83	1.68E-04	0.00196048
7897620	5226	*phosphogluconate dehydrogenase*	*PGD*	12.19	11.29	12.81	1.68E-04	0.001961545
8150112	2936	*glutathione reductase*	*GSR*	10.94	10.53	11.40	1.69E-04	0.001962437
8145782	84549, 80185	*MAK16 homolog (S. cerevisiae) | chromosome 8 open reading frame 41*	*MAK16|C8orf41*	7.75	7.84	9.84	1.69E-04	0.001967997
8121076	10957	*proline-rich nuclear receptor coactivator 1*	*PNRC1*	11.31	11.10	9.96	1.69E-04	0.001969077
7952869	29087	*thymocyte nuclear protein 1*	*THYN1*	8.80	8.52	10.42	1.70E-04	0.001979
8037290	5678	*pregnancy specific beta-1-glycoprotein 9*	*PSG9*	8.48	8.03	6.80	1.70E-04	0.001979707
7958147	6996	*thymine-DNA glycosylase*	*TDG*	6.81	6.84	7.93	1.70E-04	0.001979852
7988467	2200	*fibrillin 1*	*FBN1*	11.79	11.91	6.37	1.71E-04	0.001980954
8002919	3735	*lysyl-tRNA synthetase*	*KARS*	9.94	9.95	11.24	1.71E-04	0.001980954
8063453	5203	*prefoldin subunit 4*	*PFDN4*	7.38	7.54	8.70	1.71E-04	0.001980954
7955768	5204	*prefoldin subunit 5*	*PFDN5*	9.76	9.37	8.81	1.71E-04	0.001980954
8101099	55153	*SDA1 domain containing 1*	*SDAD1*	7.94	8.02	10.04	1.71E-04	0.001980954
8174361	1831	*TSC22 domain family, member 3*	*TSC22D3*	9.54	9.84	7.90	1.71E-04	0.001981127
8107563	51334	*proline rich 16*	*PRR16*	9.38	10.02	6.86	1.71E-04	0.0019825
8081241	84319	*chromosome 3 open reading frame 26*	*C3orf26*	8.17	9.16	10.48	1.71E-04	0.00198511
7974352	51637	*chromosome 14 open reading frame 166*	*C14orf166*	9.69	9.82	11.12	1.72E-04	0.001989767

8121502	112495	*general transcription factor IIIC, polypeptide 6, alpha 35kDa*	*GTF3C6*	9.28	9.50	10.53	1.72E-04	0.001989767
8151310	2138	*eyes absent homolog 1 (Drosophila)*	*EYA1*	9.44	6.41	6.88	1.73E-04	0.001997927
7952557	6734	*signal recognition particle receptor (docking protein)*	*SRPR*	11.73	11.72	10.76	1.73E-04	0.001997927
8176806	9081, 442862	*PTPN13-like, Y-linked | PTPN13-like, Y-linked 2*	*PRY|PRY2*	5.95	6.01	5.61	1.73E-04	0.001997927
8166402	6611	*spermine synthase*	*SMS*	8.86	8.85	11.02	1.73E-04	0.002000454
8171172	25878	*matrix-remodelling associated 5*	*MXRA5*	10.15	11.50	6.47	1.73E-04	0.002000454
8031884	27300	*zinc finger protein 544*	*ZNF544*	7.35	7.46	8.91	1.73E-04	0.002000454
8161892	9630	*guanine nucleotide binding protein (G protein), alpha 14*	*GNA14*	6.00	6.19	9.49	1.73E-04	0.00200069
8101881	125	*alcohol dehydrogenase 1B (class I), beta polypeptide*	*ADH1B*	8.96	6.53	5.45	1.74E-04	0.002001798
8057056	7273	*titin*	*TTN*	5.70	5.72	7.28	1.74E-04	0.002001798
8109283	3340	*N-deacetylase/N-sulfotransferase (heparan glucosaminyl) 1*	*NDST1*	12.05	12.35	10.46	1.74E-04	0.00200451
8027279	80264	*zinc finger protein 430*	*ZNF430*	5.67	5.74	7.64	1.74E-04	0.002009494
7965842				9.80	9.88	8.45	1.74E-04	0.002009494
8106280	3156	*3-hydroxy-3-methylglutaryl-CoA reductase*	*HMGCR*	8.14	7.52	10.98	1.75E-04	0.002009699
7995820	4490	*metallothionein 1B*	*MT1B*	7.58	7.57	6.84	1.75E-04	0.002009699
7979906	51241		*COX16*	11.44	11.64	12.42	1.75E-04	0.002009699
8150433	157848	*NK6 homeobox 3*	*NKX6-3*	8.79	8.81	8.25	1.75E-04	0.002009699
8098465	403315	*family with sequence similarity 92, member A3*	*FAM92A3*	5.54	5.49	5.10	1.75E-04	0.002009699
8069508				4.78	4.90	5.78	1.75E-04	0.002009699
8037103	2901	*glutamate receptor, ionotropic, kainate 5*	*GRIK5*	8.05	7.38	8.95	1.75E-04	0.002013726
8070194	861	*runt-related transcription factor 1*	*RUNX1*	9.87	10.76	7.46	1.76E-04	0.002015969
8132858				7.31	7.60	6.60	1.76E-04	0.002017066
8098508	3622	*inhibitor of growth family, member 2*	*ING2*	6.99	7.13	7.77	1.76E-04	0.002017133
7979671	4149	*MYC associated factor X*	*MAX*	8.74	8.68	8.05	1.76E-04	0.002019195
7930299	119392	*chromosome 10 open reading frame 78*	*C10orf78*	5.98	5.99	8.02	1.76E-04	0.002019195
8076260	10478	*solute carrier family 25 (mitochondrial carrier; peroxisomal membrane protein, 34kDa), member 17*	*SLC25A17*	8.74	8.87	10.05	1.76E-04	0.002020423

8062873	60598	*potassium channel, subfamily K, member 15*	*KCNK15*	9.85	9.93	8.15	1.76E-04	0.002020423
7970949	4081	*mab-21-like 1 (C. elegans)*	*MAB21L1*	10.09	8.83	5.47	1.77E-04	0.002021537
7898833	23028	*lysine (K)-specific demethylase 1A*	*KDM1A*	9.19	9.10	11.24	1.77E-04	0.002023576
7991478	145814	*pyroglutamyl-peptidase I-like*	*PGPEP1L*	6.92	7.05	6.60	1.77E-04	0.002024539
7994350				7.80	7.82	7.24	1.77E-04	0.002024864
8159854	9933		*KIAA0020*	7.07	6.87	9.20	1.77E-04	0.002025592
7958331	55188	*resistance to inhibitors of cholinesterase 8 homolog B (C. elegans)*	*RIC8B*	7.13	7.19	8.76	1.77E-04	0.002025592
8138067	9265	*cytohesin 3*	*CYTH3*	10.72	10.25	8.59	1.78E-04	0.002028804
8115147	972		*CD74*	7.56	7.01	9.20	1.78E-04	0.002030952
7914127	2537	*interferon, alpha-inducible protein 6*	*IFI6*	11.42	11.09	9.60	1.78E-04	0.002030952
8074157	284942, 118433, 388574, 644128	*ribosomal protein L23a pseudogene 82 | ribosomal protein L23a pseudogene 7 | ribosomal protein L23a pseudogene 53*	*RPL23AP82|RPL23AP7|FLJ43681|RPL23AP53*	9.65	9.95	9.18	1.78E-04	0.002030952
8010897	284207	*meteorin, glial cell differentiation regulator-like*	*METRNL*	9.11	9.39	7.66	1.78E-04	0.002031378
8002865	10428, 284021	*craniofacial development protein 1 | chromosome 17 open reading frame 60*	*CFDP1|C17orf60*	7.37	7.34	8.82	1.78E-04	0.002031378
7937915	6240	*ribonucleotide reductase M1*	*RRM1*	8.41	8.49	10.51	1.78E-04	0.002031414
8136983	401428, 441295	*olfactory receptor, family 2, subfamily A, member 20 pseudogene | olfactory receptor, family 2, subfamily A, member 9 pseudogene*	*OR2A20P|OR2A9P*	9.86	9.58	8.48	1.78E-04	0.002031414
7898227	23207	*pleckstrin homology domain containing, family M (with RUN domain) member 2*	*PLEKHM2*	11.21	11.07	9.62	1.79E-04	0.002035928
7954717	636	*bicaudal D homolog 1 (Drosophila)*	*BICD1*	7.52	7.70	9.52	1.79E-04	0.002036328
8136580	401409		*RAB19*	6.88	7.01	8.96	1.79E-04	0.002036328
7933139	7582	*zinc finger protein 33B*	*ZNF33B*	6.48	6.29	8.63	1.79E-04	0.002039934
8019149	124565	*solute carrier family 38, member 10*	*SLC38A10*	9.68	9.57	8.08	1.79E-04	0.002040669
7981972	100033424	*small nucleolar RNA, C/D box 116-12*	*SNORD116-12*	5.25	5.79	6.90	1.80E-04	0.002044937
8008151	10642	*insulin-like growth factor 2 mRNA binding protein 1*	*IGF2BP1*	7.22	7.47	11.52	1.80E-04	0.002046363

7949948	53838	*chromosome 11 open reading frame 24*	*C11orf24*	11.68	11.77	10.21	1.80E-04	0.002046363
8090448	8607	*RuvB-like 1 (E. coli)*	*RUVBL1*	9.02	8.92	11.00	1.80E-04	0.002047269
8021924	9984	*THO complex 1*	*THOC1*	7.69	7.73	9.82	1.80E-04	0.002047269
8006836	342666		*FLJ43826*	5.96	6.08	5.81	1.81E-04	0.002048366
7975626	91748	*chromosome 14 open reading frame 43*	*C14orf43*	6.91	6.89	5.82	1.81E-04	0.00205033
8132318	54443	*anillin, actin binding protein*	*ANLN*	8.04	8.24	10.27	1.81E-04	0.002052193
7978692	89874	*solute carrier family 25 (mitochondrial oxodicarboxylate carrier), member 21*	*SLC25A21*	5.44	5.43	6.74	1.81E-04	0.002053389
7971184	10240	*mitochondrial ribosomal protein S31*	*MRPS31*	6.92	7.14	8.52	1.82E-04	0.002056328
7979984	53349	*zinc finger, FYVE domain containing 1*	*ZFYVE1*	9.26	9.11	8.17	1.82E-04	0.002056328
8160478	79886	*chromosome 9 open reading frame 82*	*C9orf82*	7.01	7.11	8.43	1.82E-04	0.002058959
8030997	126017	*zinc finger protein 813*	*ZNF813*	5.66	5.52	7.74	1.82E-04	0.002058959
8015187	81851, 81850, 728255	*keratin associated protein 1-1 | keratin associated protein 1-3 | keratin associated protein 1-4*	*KRTAP1-1|KRTAP1-3|KRTAP1-4*	9.98	11.52	8.36	1.82E-04	0.002060131
8145361	4741	*neurofilament, medium polypeptide*	*NEFM*	6.14	6.26	7.15	1.83E-04	0.002064311
7954132	144608, 100125871	*chromosome 12 open reading frame 60 | hypothetical protein LOC100125871*	*C12orf60|LOC100125871*	5.93	5.91	6.65	1.83E-04	0.002066064
8056217	439921	*matrix-remodelling associated 7*	*MXRA7*	10.73	10.97	8.74	1.83E-04	0.002066114
8149142	1670	*defensin, alpha 5, Paneth cell-specific*	*DEFA5*	6.03	6.13	5.48	1.83E-04	0.002068775
7925500	1122	*choroideremia-like (Rab escort protein 2)*	*CHML*	7.55	6.55	10.14	1.83E-04	0.0020703
8075886	3560	*interleukin 2 receptor, beta*	*IL2RB*	7.46	7.50	6.91	1.84E-04	0.002072165
7956930	8445	*dual-specificity tyrosine-(Y)-phosphorylation regulated kinase 2*	*DYRK2*	8.81	8.94	9.57	1.84E-04	0.002072165
8030360	26819, 23521	*small nucleolar RNA, C/D box 32A | ribosomal protein L13a*	*SNORD32A|RPL13A*	12.04	12.32	11.25	1.84E-04	0.002072165
8143629	441295, 401428	*olfactory receptor, family 2, subfamily A, member 9 pseudogene | olfactory receptor, family 2, subfamily A, member 20 pseudogene*	*OR2A9P|OR2A20P*	9.82	9.58	8.54	1.84E-04	0.002072165

7964832				8.80	9.14	9.54	1.84E-04	0.002072165
8121416				6.99	7.09	5.82	1.84E-04	0.002072221
7995787	4499	*metallothionein 1M*	*MT1M*	9.83	9.54	7.42	1.85E-04	0.00207699
8044353	55289	*acyl-CoA oxidase-like*	*ACOXL*	5.66	5.67	7.47	1.85E-04	0.002079571
7991070	50810	*hepatoma-derived growth factor, related protein 3*	*HDGFRP3*	9.25	9.08	10.79	1.86E-04	0.002086656
8067978	5478	*peptidylprolyl isomerase A (cyclophilin A)*	*PPIA*	12.61	12.63	13.07	1.86E-04	0.002087927
7947245	3324	*heat shock protein 90kDa alpha (cytosolic), class A member 2*	*HSP90AA2*	9.09	9.04	10.69	1.86E-04	0.002089256
8143919	6604	*SWI/SNF related, matrix associated, actin dependent regulator of chromatin, subfamily d, member 3*	*SMARCD3*	9.19	8.54	7.33	1.86E-04	0.002090175
7935146	64318	*nucleolar complex associated 3 homolog (S. cerevisiae)*	*NOC3L*	7.03	7.25	9.00	1.86E-04	0.002090175
7968658	11340	*exosome component 8*	*EXOSC8*	8.25	8.21	10.32	1.86E-04	0.002091955
8116582	2295	*forkhead box F2*	*FOXF2*	10.88	9.10	7.61	1.87E-04	0.00209497
8121563	4082	*myristoylated alanine-rich protein kinase C substrate*	*MARCKS*	10.52	11.04	9.61	1.87E-04	0.00209497
7993478	4363	*ATP-binding cassette, sub-family C (CFTR/MRP), member 1*	*ABCC1*	10.95	10.36	9.42	1.87E-04	0.002096132
7979864	2079	*enhancer of rudimentary homolog (Drosophila)*	*ERH*	12.21	12.41	13.29	1.87E-04	0.002096756
8092726	9076	*claudin 1*	*CLDN1*	6.15	6.04	7.46	1.87E-04	0.002096756
8031516	51157, 51545	*zinc finger protein 580 | zinc finger protein 581*	*ZNF580|ZNF581*	9.44	9.31	8.14	1.87E-04	0.002098734
7960933	4074	*mannose-6-phosphate receptor (cation dependent)*	*M6PR*	10.78	10.90	11.62	1.88E-04	0.002099401
8148591	338328	*glycosylphosphatidylinositol anchored high density lipoprotein binding protein 1*	*GPIHBP1*	7.27	7.38	6.89	1.88E-04	0.002099401
8110408	84321, 728554	*THO complex 3 | THO complex 3 pseudogene*	*THOC3|LOC728554*	8.11	8.61	10.27	1.88E-04	0.002103337
8096050	2250	*fibroblast growth factor 5*	*FGF5*	9.83	8.88	6.30	1.88E-04	0.002105801
8124008	63933	*coiled-coil domain containing 90A*	*CCDC90A*	8.11	8.58	9.27	1.89E-04	0.002105801
8135064	81844	*tripartite motif-containing 56*	*TRIM56*	8.03	8.21	7.47	1.89E-04	0.002105801
7967463	353116	*Rab interacting lysosomal protein-like 1*	*RILPL1*	8.95	9.16	7.40	1.89E-04	0.002105801
7987511	388115	*chromosome 15 open reading frame 52*	*C15orf52*	8.21	8.06	6.82	1.88E-04	0.002105801

8172022	83604	*transmembrane protein 47*	*TMEM47*	13.35	12.97	11.98	1.89E-04	0.002106627
7918622	6566	*solute carrier family 16, member 1 (monocarboxylic acid transporter 1)*	*SLC16A1*	8.16	9.07	11.69	1.89E-04	0.002112847
7921014	4209	*myocyte enhancer factor 2D*	*MEF2D*	9.89	10.15	8.16	1.90E-04	0.0021138
7985016	257364	*sorting nexin 33*	*SNX33*	10.06	10.22	8.18	1.90E-04	0.002117034
8033257	718	*complement component 3*	*C3*	10.71	8.72	7.14	1.90E-04	0.002117999
8068254	3588, 3455	*interleukin 10 receptor, beta | interferon (alpha, beta and omega) receptor 2*	*IL10RB|IFNAR2*	10.41	10.30	7.41	1.90E-04	0.002118891
8092321	54165	*DCN1, defective in cullin neddylation 1, domain containing 1 (S. cerevisiae)*	*DCUN1D1*	9.56	9.69	11.16	1.90E-04	0.002120158
7916986	257194	*neuronal growth regulator 1*	*NEGR1*	9.21	8.88	6.35	1.91E-04	0.00212262
8171295				7.64	7.66	6.64	1.91E-04	0.002123639
7943282	55693	*lysine (K)-specific demethylase 4D*	*KDM4D*	6.36	6.52	7.51	1.91E-04	0.00212546
8097792	8944	*small nucleolar RNA, C/D box 73A*	*SNORD73A*	6.83	6.69	8.63	1.91E-04	0.00212621
7996448	8996, 653319	*nucleolar protein 3 (apoptosis repressor with CARD domain) | KIAA0895-like*	*NOL3|KIAA0895L*	9.05	8.95	7.81	1.91E-04	0.002127304
8020468	5932	*retinoblastoma binding protein 8*	*RBBP8*	7.38	8.43	9.84	1.92E-04	0.002128135
8005953	26773	*small nucleolar RNA, C/D box 4A*	*SNORD4A*	7.47	7.62	9.71	1.92E-04	0.002136323
8046169	9360	*peptidylprolyl isomerase G (cyclophilin G)*	*PPIG*	6.83	6.47	8.65	1.93E-04	0.0021387
8079294	23016	*exosome component 7*	*EXOSC7*	7.75	7.64	8.83	1.93E-04	0.0021387
8080991	220988	*heterogeneous nuclear ribonucleoprotein A3*	*HNRNPA3*	8.42	8.54	11.44	1.93E-04	0.002142774
8078196	131096	*potassium voltage-gated channel, subfamily H (eag-related), member 8*	*KCNH8*	5.74	5.83	6.89	1.93E-04	0.002144608
8155554	375719, 364	*aquaporin 7 pseudogene 1*	*AQP7P1*	6.66	6.66	6.15	1.94E-04	0.002144608
7994609	23475	*quinolinate phosphoribosyltransferase*	*QPRT*	9.06	8.82	11.28	1.94E-04	0.002146853
8096335	55008	*hect domain and RLD 6*	*HERC6*	6.54	6.17	8.15	1.94E-04	0.002147195
8007637	2535	*frizzled homolog 2 (Drosophila)*	*FZD2*	10.80	10.47	8.25	1.95E-04	0.002154851
7947570	9537	*tumor protein p53 inducible protein 11*	*TP53I11*	10.60	9.64	8.11	1.95E-04	0.002154851
7986329	7026	*nuclear receptor subfamily 2, group F, member 2*	*NR2F2*	9.84	9.31	7.99	1.95E-04	0.002154954
8047069	3628	*inositol polyphosphate-1-phosphatase*	*INPP1*	7.41	7.68	6.20	1.95E-04	0.002156122

7903980	128346	*chromosome 1 open reading frame 162*	*C1orf162*	6.55	6.77	6.29	1.95E-04	0.002156122
7982514				7.27	7.56	6.26	1.95E-04	0.002156122
8070925				7.98	8.03	7.45	1.95E-04	0.002156122
8161488	375719, 364	*aquaporin 7 pseudogene 1*	*AQP7P1*	6.60	6.62	6.07	1.96E-04	0.002159036
8071655				8.48	8.44	7.89	1.96E-04	0.002160341
7972946	22821	*RAS p21 protein activator 3*	*RASA3*	9.28	9.29	8.24	1.96E-04	0.002160601
7933758				5.69	5.88	6.73	1.96E-04	0.002160889
8102831	84709, 4717	*chromosome 4 open reading frame 49 | NADH dehydrogenase (ubiquinone) 1, subcomplex unknown, 1, 6kDa*	*C4orf49|NDUFC1*	9.79	8.91	7.37	1.96E-04	0.002163309
8036636	22933	*sirtuin 2*	*SIRT2*	10.06	10.07	7.68	1.96E-04	0.002164694
8011114	8578	*scavenger receptor class F, member 1*	*SCARF1*	7.86	7.92	7.16	1.97E-04	0.002172365
7898521	84966	*immunoglobin superfamily, member 21*	*IGSF21*	6.70	6.82	8.11	1.97E-04	0.002172365
8026339	6637, 100130932	*small nuclear ribonucleoprotein polypeptide G | small nuclear ribonucleoprotein G-like protein*	*SNRPG|LOC100130932*	8.67	8.97	10.53	1.97E-04	0.002172365
7902290	1491	*cystathionase (cystathionine gamma-lyase)*	*CTH*	6.70	7.21	8.90	1.98E-04	0.002176633
7973067	4860	*purine nucleoside phosphorylase*	*PNP*	7.90	9.02	11.07	1.98E-04	0.002177845
7957008	11052	*cleavage and polyadenylation specific factor 6, 68kDa*	*CPSF6*	8.48	8.46	10.37	1.98E-04	0.002179002
8138888	5137	*phosphodiesterase 1C, calmodulin-dependent 70kDa*	*PDE1C*	6.84	8.75	5.65	1.98E-04	0.00217981
7939116				6.38	6.46	5.72	1.99E-04	0.002181044
8018864	9021	*suppressor of cytokine signaling 3*	*SOCS3*	9.21	8.58	7.59	1.99E-04	0.002181937
7958600	88455	*ankyrin repeat domain 13A*	*ANKRD13A*	12.00	11.14	9.01	1.99E-04	0.002182946
8139500	64759	*tensin 3*	*TNS3*	10.96	10.16	8.00	1.99E-04	0.002186628
8048411	9654	*tubulin tyrosine ligase-like family, member 4*	*TTLL4*	8.49	8.87	10.48	2.00E-04	0.002191179
8010737	5881	*ras-related C3 botulinum toxin substrate 3 (rho family, small GTP binding protein Rac3)*	*RAC3*	8.46	8.48	10.19	2.00E-04	0.002191225
7970473	64328	*exportin 4*	*XPO4*	7.02	6.68	9.99	2.00E-04	0.002191788
8035886	83636	*chromosome 19 open reading frame 12*	*C19orf12*	9.00	9.31	8.13	2.00E-04	0.002194562

7997642	83716	*cysteine-rich secretory protein LCCL domain containing 2*	*CRISPLD2*	10.34	10.07	7.04	2.00E-04	0.002194562
8011817	7775	*zinc finger protein 232*	*ZNF232*	6.43	6.34	7.81	2.01E-04	0.002198939
8068410	51072, 84661	*mediator of cell motility 1 | dpy-30 homolog (C. elegans)*	*MEMO1|DPY30*	7.95	8.33	8.81	2.01E-04	0.002198939
8105328	112574	*sorting nexin 18*	*SNX18*	8.95	8.76	7.86	2.02E-04	0.002207859
8143448	285962	*hypothetical LOC285962*	*FLJ40852*	5.86	6.15	5.59	2.02E-04	0.002207859
8062687				6.95	7.07	6.70	2.02E-04	0.002207859
8114476	64374		*SIL1*	9.05	8.95	6.85	2.02E-04	0.002208856
8020647	125488	*tetratricopeptide repeat domain 39C*	*TTC39C*	8.82	7.87	8.40	2.03E-04	0.002213402
8177395	9081, 442862	*PTPN13-like, Y-linked | PTPN13-like, Y-linked 2*	*PRY|PRY2*	6.04	6.08	5.61	2.03E-04	0.002213857
7981955	100033416, 6638	*small nucleolar RNA, C/D box 116-4 | small nuclear ribonucleoprotein polypeptide N*	*SNORD116-4|SNRPN*	9.01	9.25	10.42	2.03E-04	0.002215474
7915640	8891	*eukaryotic translation initiation factor 2B, subunit 3 gamma, 58kDa*	*EIF2B3*	7.61	7.59	8.73	2.03E-04	0.002215694
8134454	25798	*brain protein I3*	*BRI3*	10.39	10.55	9.38	2.03E-04	0.002215694
7904953				4.66	4.69	7.31	2.03E-04	0.002215694
7914550	339487	*zinc finger and BTB domain containing 8 opposite strand*	*ZBTB8OS*	7.78	8.16	8.82	2.04E-04	0.002218816
8050846	1838	*dystrobrevin, beta*	*DTNB*	8.55	7.98	9.51	2.04E-04	0.002219414
7957833				5.23	5.34	4.83	2.04E-04	0.002225691
8106473				7.55	7.44	7.88	2.05E-04	0.00222693
7966035	10970	*cytoskeleton-associated protein 4*	*CKAP4*	11.37	11.52	9.86	2.05E-04	0.002227709
8055038	55679	*LIM and senescent cell antigen-like domains 2*	*LIMS2*	9.77	9.86	8.03	2.05E-04	0.002230779
8143209	136306	*SVOP-like*	*SVOPL*	6.29	6.21	7.30	2.05E-04	0.002230779
7913869	3925	*stathmin 1*	*STMN1*	9.15	9.21	11.29	2.05E-04	0.002230943
8107532	3295	*hydroxysteroid (17-beta) dehydrogenase 4*	*HSD17B4*	8.06	7.90	10.88	2.05E-04	0.002231205
8012282	284023		*LOC284023*	7.99	7.95	6.97	2.06E-04	0.002235402
8026381				4.78	4.85	5.60	2.06E-04	0.002235402

8138581	29896	*transformer 2 alpha homolog (Drosophila)*	*TRA2A*	10.47	10.41	12.18	2.06E-04	0.002236906
7975076	3306	*heat shock 70kDa protein 2*	*HSPA2*	7.60	9.27	10.77	2.06E-04	0.002238216
8111629	9631	*nucleoporin 155kDa*	*NUP155*	8.58	8.51	11.27	2.07E-04	0.002238216
7917771	30836	*deoxynucleotidyltransferase, terminal, interacting protein 2*	*DNTTIP2*	8.12	8.13	9.82	2.06E-04	0.002238216
8047174	57181	*solute carrier family 39 (zinc transporter), member 10*	*SLC39A10*	7.95	8.21	11.61	2.06E-04	0.002238216
8120335	222584	*family with sequence similarity 83, member B*	*FAM83B*	6.13	6.04	9.86	2.07E-04	0.002241771
8038078	10945	*KDEL (Lys-Asp-Glu-Leu) endoplasmic reticulum protein retention receptor 1*	*KDELR1*	12.27	11.99	11.13	2.07E-04	0.002242879
7982529	100509022	*splicing factor U2AF 35 kDa subunit-like*	*LOC10050 9022*	8.29	8.47	9.02	2.07E-04	0.002242879
7978312	11035	*receptor-interacting serine-threonine kinase 3*	*RIPK3*	7.53	7.17	6.16	2.07E-04	0.002243738
7974066	5411	*pinin, desmosome associated protein*	*PNN*	7.42	7.39	10.31	2.08E-04	0.002247289
7949754	23529	*cardiotrophin-like cytokine factor 1*	*CLCF1*	9.03	9.54	7.83	2.08E-04	0.002247289
8085608	26061	*2-hydroxyacyl-CoA lyase 1*	*HACL1*	8.21	7.90	10.26	2.08E-04	0.002247289
7949746	57804	*polymerase (DNA-directed), delta 4*	*POLD4*	11.15	11.01	9.20	2.08E-04	0.002247289
7900710	23334, 149469	*KIAA0467 | chromosome 1 open reading frame 84*	*KIAA0467| C1orf84*	8.79	8.68	7.24	2.08E-04	0.002247289
8065254				12.82	12.73	13.08	2.08E-04	0.002247289
8142136	168455		*FLJ36031*	8.96	9.21	7.77	2.08E-04	0.002247517
8008598	58488	*phosphatidylcholine transfer protein*	*PCTP*	8.59	8.50	9.97	2.09E-04	0.002248899
8027260	90649	*zinc finger protein 486*	*ZNF486*	8.50	8.66	11.21	2.09E-04	0.002248899
8070629	90625	*chromosome 21 open reading frame 105*	*C21orf105*	6.24	6.35	10.06	2.09E-04	0.002249308
7922243	92342	*chromosome 1 open reading frame 156*	*C1orf156*	7.23	7.32	8.49	2.09E-04	0.002250257
7981283	123096	*solute carrier family 25, member 29*	*SLC25A29*	9.17	8.95	9.36	2.09E-04	0.002251198
8162562	100128782, 375748	*chromosome 9 open reading frame 130 | chromosome 9 open reading frame 102*	*C9orf130| C9orf102*	7.42	7.08	6.76	2.09E-04	0.00225231
8131844	10457	*glycoprotein (transmembrane) nmb*	*GPNMB*	11.80	11.12	6.61	2.10E-04	0.002255995
8062864	8839	*WNT1 inducible signaling pathway protein 2*	*WISP2*	9.92	10.08	7.76	2.10E-04	0.002262664
8039748	1, 162968	*alpha-1-B glycoprotein | zinc finger protein 497*	*A1BG|ZNF 497*	8.09	8.11	7.09	2.10E-04	0.002262664

8106181	689	*basic transcription factor 3*	*BTF3*	10.02	10.15	10.79	2.11E-04	0.002264747
8151684	4325	*matrix metallopeptidase 16 (membrane-inserted)*	*MMP16*	6.16	6.29	8.47	2.11E-04	0.002265625
8114900	5521	*protein phosphatase 2, regulatory subunit B, beta*	*PPP2R2B*	6.43	6.43	8.73	2.11E-04	0.00226857
7905754	57198	*ATPase, class I, type 8B, member 2*	*ATP8B2*	10.29	10.63	9.06	2.11E-04	0.002270881
8075401	9514	*galactose-3-O-sulfotransferase 1*	*GAL3ST1*	7.02	7.14	7.90	2.12E-04	0.00227223
7947147	258010	*small VCP/p97-interacting protein*	*SVIP*	6.35	6.66	8.07	2.12E-04	0.002274943
8132980	84629	*trinucleotide repeat containing 18*	*TNRC18*	9.16	9.19	7.06	2.12E-04	0.00227496
7967056	51499	*TP53 regulated inhibitor of apoptosis 1*	*TRIAP1*	8.23	8.47	9.61	2.12E-04	0.002278308
8116980	221687	*ring finger protein 182*	*RNF182*	5.84	7.04	8.58	2.13E-04	0.002278662
7960143	7637	*zinc finger protein 84*	*ZNF84*	7.56	7.42	9.33	2.13E-04	0.002279067
8138602	1687	*deafness, autosomal dominant 5*	*DFNA5*	9.88	9.17	8.07	2.13E-04	0.002280078
8044391	10461	*c-mer proto-oncogene tyrosine kinase*	*MERTK*	6.30	6.38	7.53	2.13E-04	0.002280078
8171013	7411	*von Hippel-Lindau binding protein 1*	*VBP1*	6.67	6.66	7.91	2.13E-04	0.002284078
7997164				5.75	5.67	5.97	2.14E-04	0.002286422
7943349	143872	*Rho GTPase activating protein 42*	*ARHGAP42*	8.18	6.66	10.28	2.14E-04	0.002286561
7969815	171425	*citrate lyase beta like*	*CLYBL*	6.02	5.89	7.92	2.15E-04	0.00229461
8038877	8778	*sialic acid binding Ig-like lectin 5*	*SIGLEC5*	6.98	7.15	6.45	2.15E-04	0.00229504
7900531	2981	*guanylate cyclase activator 2B (uroguanylin)*	*GUCA2B*	7.88	7.99	7.18	2.15E-04	0.002295184
8123864	7020	*transcription factor AP-2 alpha (activating enhancer binding protein 2 alpha)*	*TFAP2A*	11.22	8.66	7.38	2.15E-04	0.002295184
8071069	23765	*interleukin 17 receptor A*	*IL17RA*	10.12	10.13	8.15	2.15E-04	0.002295184
8040386	1653	*DEAD (Asp-Glu-Ala-Asp) box polypeptide 1*	*DDX1*	9.38	9.24	10.99	2.15E-04	0.002295975
8068902	23076	*ribosomal RNA processing 1 homolog B (S. cerevisiae)*	*RRP1B*	8.15	8.08	10.23	2.15E-04	0.002295975
8125321	80863	*proline-rich transmembrane protein 1*	*PRRT1*	8.55	8.15	7.13	2.15E-04	0.002295975
8075897	114904	*C1q and tumor necrosis factor related protein 6*	*C1QTNF6*	9.21	8.82	7.38	2.15E-04	0.002295975
7955469	9498	*solute carrier family 4, sodium bicarbonate cotransporter, member 8*	*SLC4A8*	6.73	6.78	7.94	2.16E-04	0.002303203
7999827	6210	*ribosomal protein S15a*	*RPS15A*	12.48	12.50	12.85	2.16E-04	0.002303789
8035177	79939	*solute carrier family 35, member E1*	*SLC35E1*	11.15	11.40	9.92	2.16E-04	0.002304278

7899289	23038	*WD and tetratricopeptide repeats 1*	*WDTC1*	9.41	9.33	7.43	2.17E-04	0.002305883
7952893	440073	*IQ motif and Sec7 domain 3*	*IQSEC3*	9.75	9.90	9.01	2.17E-04	0.002306905
8046590	220988, 10151	*heterogeneous nuclear ribonucleoprotein A3 | heterogeneous nuclear ribonucleoprotein A3 pseudogene 1*	*HNRNPA3|HNRNPA3P1*	7.56	7.63	10.53	2.17E-04	0.002308196
8023267	4645	*myosin VB*	*MYO5B*	5.92	6.03	7.55	2.18E-04	0.002311382
8124540	8336	*histone cluster 1, H2am*	*HIST1H2AM*	6.31	6.47	9.16	2.18E-04	0.002311382
7958960	53373	*two pore segment channel 1*	*TPCN1*	10.33	9.54	8.35	2.18E-04	0.002311382
8083594	5806	*pentraxin 3, long*	*PTX3*	10.57	11.02	5.77	2.18E-04	0.00231154
7904755	8799	*peroxisomal biogenesis factor 11 beta*	*PEX11B*	11.22	11.18	9.97	2.18E-04	0.002314558
8131881	340277	*chromosome 7 open reading frame 46*	*C7orf46*	5.77	5.65	8.43	2.18E-04	0.002316595
8170775	6748	*signal sequence receptor, delta (translocon-associated protein delta)*	*SSR4*	9.45	9.52	8.13	2.19E-04	0.002324633
8165888				5.61	5.47	7.13	2.19E-04	0.002325999
8108420	26821	*small nucleolar RNA, H/ACA box 74A*	*SNORA74A*	6.90	7.06	9.12	2.20E-04	0.002326985
7919436	164022, 644591	*peptidylprolyl isomerase A (cyclophilin A)-like 4A | peptidylprolyl isomerase A (cyclophilin A)-like 4G*	*PPIAL4A|PPIAL4G*	12.88	12.89	13.31	2.20E-04	0.002326985
7965721				6.81	7.33	5.64	2.20E-04	0.002326985
8121225	2898	*glutamate receptor, ionotropic, kainate 2*	*GRIK2*	5.81	6.98	5.91	2.20E-04	0.002328706
8109612	147	*adrenergic, alpha-1B-, receptor*	*ADRA1B*	9.50	7.83	6.82	2.20E-04	0.002331183
8102860	3020, 440926	*H3 histone, family 3A | H3 histone, family 3A pseudogene*	*H3F3A|LOC440926*	11.05	11.27	13.04	2.20E-04	0.002331892
8176026	2316	*filamin A, alpha*	*FLNA*	12.96	13.06	11.53	2.21E-04	0.002340029
7900426	64744	*small ArfGAP2*	*SMAP2*	9.66	9.60	7.37	2.21E-04	0.002340029
8024816	79187	*fibronectin type III and SPRY domain containing 1*	*FSD1*	7.72	7.84	9.09	2.21E-04	0.002340029
7896863	554210	*microRNA 429*	*MIR429*	6.70	6.94	6.25	2.21E-04	0.002340029
7948176	85456	*tankyrase 1 binding protein 1, 182kDa*	*TNKS1BP1*	10.10	10.03	8.55	2.22E-04	0.002342709
8016457	3215	*homeobox B5*	*HOXB5*	5.95	7.54	5.62	2.22E-04	0.002343207

7945420	6050, 84200	*ribonuclease/angiogenin inhibitor 1 | hypothetical protein FLJ23519*	*RNH1|FLJ 23519*	11.06	11.36	8.25	2.22E-04	0.002345475
7925813	414235	*chromosome 10 open reading frame 108*	*C10orf108*	6.70	6.67	6.11	2.22E-04	0.002346471
8104693	23037	*PDZ domain containing 2*	*PDZD2*	6.32	6.43	7.33	2.23E-04	0.002347984
7922656	84320	*acyl-CoA binding domain containing 6*	*ACBD6*	6.83	6.61	7.39	2.24E-04	0.002356765
8131614	196	*aryl hydrocarbon receptor*	*AHR*	11.39	9.63	8.53	2.24E-04	0.002358876
8095894	65008	*mitochondrial ribosomal protein L1*	*MRPL1*	6.64	6.59	7.88	2.24E-04	0.002358876
8074632	84861	*kelch-like 22 (Drosophila)*	*KLHL22*	8.17	7.98	6.90	2.24E-04	0.002362559
8117034	2766	*guanosine monophosphate reductase*	*GMPR*	10.88	8.98	6.90	2.25E-04	0.002364088
7996012	23568	*ADP-ribosylation factor-like 2 binding protein*	*ARL2BP*	11.52	11.49	9.58	2.25E-04	0.002365893
8005628				7.67	7.60	6.81	2.25E-04	0.002365893
8073457	2547	*X-ray repair complementing defective repair in Chinese hamster cells 6*	*XRCC6*	10.83	10.85	11.80	2.26E-04	0.002369343
7936408	9937	*DNA cross-link repair 1A*	*DCLRE1A*	6.36	6.25	8.09	2.26E-04	0.002369343
8068671	25825	*beta-site APP-cleaving enzyme 2*	*BACE2*	10.23	10.87	8.22	2.25E-04	0.002369343
7954591	60488	*mitochondrial ribosomal protein S35*	*MRPS35*	8.13	8.48	9.21	2.25E-04	0.002369343
8025633	5141	*phosphodiesterase 4A, cAMP-specific*	*PDE4A*	9.44	9.16	7.71	2.26E-04	0.002370567
8016452	3214	*homeobox B4*	*HOXB4*	7.08	7.90	6.62	2.26E-04	0.002371115
8040742	22924	*microtubule-associated protein, RP/EB family, member 3*	*MAPRE3*	8.59	8.48	6.25	2.26E-04	0.002371115
8112761				5.15	5.16	4.92	2.26E-04	0.002371115
7986503	196968	*dynamin 1 pseudogene*	*C15orf51*	9.06	8.75	7.98	2.26E-04	0.002372129
7917896	199857	*asparagine-linked glycosylation 14 homolog (S. cerevisiae)*	*ALG14*	8.43	8.60	7.79	2.26E-04	0.002372129
8179713	29113	*chromosome 6 open reading frame 15*	*C6orf15*	7.05	7.03	6.32	2.26E-04	0.002372371
7927876	80312	*tet oncogene 1*	*TET1*	7.43	7.19	10.26	2.28E-04	0.00238657
8058052	3329	*heat shock 60kDa protein 1 (chaperonin)*	*HSPD1*	9.22	9.27	12.08	2.28E-04	0.00238778
8052703	116143, 5534	*WD repeat domain 92 | protein phosphatase 3, regulatory subunit B, alpha*	*WDR92|P PP3R1*	7.10	7.13	8.24	2.28E-04	0.002390479
7899753	3932	*lymphocyte-specific protein tyrosine kinase*	*LCK*	7.20	7.24	8.35	2.29E-04	0.00239163
8117741	7932	*olfactory receptor, family 2, subfamily H, member 2*	*OR2H2*	7.22	7.27	6.57	2.29E-04	0.002394032

7932733	283078	*mohawk homeobox*	*MKX*	9.99	10.13	7.24	2.29E-04	0.00239421
8177120	727764, 727768, 100132288, 100233156	*MAFF interacting protein | tektin 4 pseudogene 1 | hypothetical protein LOC100132288 | hypothetical LOC100233156*	*MAFIP|TEKT4P1|LOC100132288|LOC100233156*	8.24	8.49	6.54	2.29E-04	0.00239421
8033118	4298	*myeloid/lymphoid or mixed-lineage leukemia (trithorax homolog, Drosophila); translocated to, 1*	*MLLT1*	10.58	10.77	8.92	2.29E-04	0.002394731
7909142	64710	*nuclear casein kinase and cyclin-dependent kinase substrate 1*	*NUCKS1*	6.49	6.52	7.83	2.29E-04	0.002395169
7990211	3073	*hexosaminidase A (alpha polypeptide)*	*HEXA*	10.98	10.73	8.95	2.29E-04	0.002395279
8114938	9832	*janus kinase and microtubule interacting protein 2*	*JAKMIP2*	5.23	5.21	8.04	2.30E-04	0.002395548
8103399	56034	*platelet derived growth factor C*	*PDGFC*	7.46	8.97	6.95	2.30E-04	0.002395548
7945648	440021, 338651	*keratin associated protein 5-2 | hypothetical LOC338651*	*KRTAP5-2|LOC338651*	8.00	7.92	7.39	2.30E-04	0.002399068
8037374	5329	*plasminogen activator, urokinase receptor*	*PLAUR*	10.30	10.88	8.24	2.30E-04	0.002401727
8015312	8689	*keratin 36*	*KRT36*	5.49	5.53	5.26	2.30E-04	0.002401779
8007008	7067, 9572	*thyroid hormone receptor, alpha (erythroblastic leukemia viral (v-erb-a) oncogene homolog, avian) | nuclear receptor subfamily 1, group D, member 1*	*THRA|NR1D1*	10.55	10.45	8.33	2.31E-04	0.002402723
8042978	130120	*regenerating islet-derived 3 gamma*	*REG3G*	5.54	5.43	5.07	2.31E-04	0.002403417
7915841	148930	*kinocilin*	*KNCN*	7.94	8.05	7.50	2.31E-04	0.002403417
7950086	4926	*nuclear mitotic apparatus protein 1*	*NUMA1*	10.24	9.84	8.74	2.31E-04	0.002404445
8033956	9294	*sphingosine-1-phosphate receptor 2*	*S1PR2*	9.10	8.95	7.83	2.31E-04	0.002406743
7972692	6555	*solute carrier family 10 (sodium/bile acid cotransporter family), member 2*	*SLC10A2*	6.51	6.41	6.13	2.31E-04	0.002406972
8010590	57597	*BAH domain and coiled-coil containing 1*	*BAHCC1*	7.98	8.01	6.85	2.32E-04	0.002406972
8099504				4.83	4.86	6.92	2.32E-04	0.002407042
7935588	3257	*Hermansky-Pudlak syndrome 1*	*HPS1*	9.50	9.25	7.52	2.32E-04	0.002410895
7931754	3422	*isopentenyl-diphosphate delta isomerase 1*	*IDI1*	8.68	8.09	10.40	2.32E-04	0.002412234
7941985	10312	*T-cell, immune regulator 1, ATPase, H+ transporting, lysosomal V0 subunit A3*	*TCIRG1*	10.00	9.98	8.01	2.33E-04	0.002417758

7973748	387978	*chromosome 14 open reading frame 23*	*C14orf23*	5.97	5.93	5.66	2.33E-04	0.002417758
8109999	57222	*endoplasmic reticulum-golgi intermediate compartment (ERGIC) 1*	*ERGIC1*	11.66	11.51	10.34	2.33E-04	0.002420051
8097809				5.13	5.24	6.99	2.34E-04	0.002421362
7986969	56160	*necdin-like 2*	*NDNL2*	8.31	8.58	7.95	2.34E-04	0.002421978
8056825	100131390	*Sp9 transcription factor homolog (mouse)*	*SP9*	7.61	7.58	6.99	2.34E-04	0.002421978
8079311	23395	*leucyl-tRNA synthetase 2, mitochondrial*	*LARS2*	8.00	7.84	9.54	2.34E-04	0.002423339
8122554	10981		*RAB32*	9.57	9.96	7.64	2.34E-04	0.002423751
8174201	55859	*brain expressed, X-linked 1*	*BEX1*	6.71	6.07	9.81	2.34E-04	0.002423751
8128565	64208	*popeye domain containing 3*	*POPDC3*	9.13	10.00	7.69	2.34E-04	0.002423751
8025382	79603	*LAG1 homolog, ceramide synthase 4*	*LASS4*	8.40	7.69	9.24	2.34E-04	0.002423751
8163916	158135	*tubulin tyrosine ligase-like family, member 11*	*TTLL11*	8.04	8.32	6.87	2.34E-04	0.002423751
7981893				8.29	8.30	7.30	2.34E-04	0.002423751
8037728	339344	*Myb-related transcription factor, partner of profilin*	*MYPOP*	8.89	8.80	7.48	2.35E-04	0.002424829
7973652	283629	*testis-specific serine kinase 4*	*TSSK4*	6.55	6.54	6.02	2.35E-04	0.002425223
8014248	146857	*schlafen family member 13*	*SLFN13*	6.14	6.27	8.34	2.35E-04	0.002428419
8113050	1070	*centrin, EF-hand protein, 3*	*CETN3*	5.71	5.73	7.73	2.36E-04	0.002430317
8071676	9609		*RAB36*	8.87	8.43	7.35	2.36E-04	0.002430317
8055913	55660	*PRP40 pre-mRNA processing factor 40 homolog A (S. cerevisiae)*	*PRPF40A*	8.71	8.62	10.48	2.36E-04	0.002430317
7920258	6277	*S100 calcium binding protein A6*	*S100A6*	8.95	9.05	7.53	2.36E-04	0.002430373
8094574	23216	*TBC1 (tre-2/USP6, BUB2, cdc16) domain family, member 1*	*TBC1D1*	9.35	9.23	10.98	2.36E-04	0.002430373
8029829	400707		*LOC400707*	7.19	7.16	6.30	2.36E-04	0.002430373
8134689	7589	*zinc finger and SCAN domain containing 21*	*ZSCAN21*	7.94	8.06	9.09	2.37E-04	0.002440773
8055476	7531	*tyrosine 3-monooxygenase/tryptophan 5-monooxygenase activation protein, epsilon polypeptide*	*YWHAE*	12.42	12.43	13.32	2.37E-04	0.002441181
8068583	3772	*potassium inwardly-rectifying channel, subfamily J, member 15*	*KCNJ15*	6.29	6.62	5.87	2.37E-04	0.002441417
8173524	4435	*Cbp/p300-interacting transactivator, with Glu/Asp-rich carboxy-terminal domain, 1*	*CITED1*	7.08	7.17	6.56	2.38E-04	0.002447256

7930498	51703	*acyl-CoA synthetase long-chain family member 5*	*ACSL5*	7.22	6.69	5.94	2.38E-04	0.002447256
8173941	7105	*tetraspanin 6*	*TSPAN6*	8.67	9.06	11.15	2.38E-04	0.002449685
8040223	6241	*ribonucleotide reductase M2*	*RRM2*	6.78	7.23	9.54	2.39E-04	0.002451431
8015179	83895, 81850, 81851	*keratin associated protein 1-5 | keratin associated protein 1-3 | keratin associated protein 1-1*	*KRTAP1-5|KRTAP1-3|KRTAP1-1*	9.52	11.32	7.56	2.39E-04	0.002452857
8019588	83895, 81850, 81851	*keratin associated protein 1-5 | keratin associated protein 1-3 | keratin associated protein 1-1*	*KRTAP1-5|KRTAP1-3|KRTAP1-1*	9.52	11.32	7.56	2.39E-04	0.002452857
8036591	3960	*lectin, galactoside-binding, soluble, 4*	*LGALS4*	7.12	7.34	6.65	2.39E-04	0.002454022
8088480	84099, 3398	*inhibitor of DNA binding 2B, dominant negative helix-loop-helix protein (pseudogene) | inhibitor of DNA binding 2, dominant negative helix-loop-helix protein*	*ID2B*	8.03	8.21	7.63	2.39E-04	0.002454022
7984289	8766		*RAB11A*	10.48	10.53	11.17	2.39E-04	0.002455714
8027473	9141	*programmed cell death 5*	*PDCD5*	8.79	9.11	9.64	2.40E-04	0.002455714
8109368	10146	*GTPase activating protein (SH3 domain) binding protein 1*	*G3BP1*	9.84	10.12	12.64	2.40E-04	0.002455714
7969574	693207, 3875	*microRNA 622 | keratin 18*	*MIR622|KRT18*	8.73	8.62	12.16	2.39E-04	0.002455714
7975676	80127, 4329	*chromosome 14 open reading frame 45 | aldehyde dehydrogenase 6 family, member A1*	*C14orf45|ALDH6A1*	6.54	6.68	5.43	2.40E-04	0.002459093
8143441	57189		*KIAA1147*	6.86	6.64	9.01	2.40E-04	0.002459375
8068157	100151643	*keratin associated protein 20-4*	*KRTAP20-4*	6.15	6.13	5.70	2.40E-04	0.002459375
8012823	6341	*SCO cytochrome oxidase deficient homolog 1 (yeast)*	*SCO1*	8.19	8.43	10.33	2.41E-04	0.002463144
7983393	6652	*sorbitol dehydrogenase*	*SORD*	8.67	8.30	9.76	2.41E-04	0.002467204
7920575	57326	*pre-B-cell leukemia homeobox interacting protein 1*	*PBXIP1*	8.94	8.60	7.47	2.41E-04	0.002469421
7913187	255104	*transmembrane and coiled-coil domains 4*	*TMCO4*	8.23	7.59	6.58	2.42E-04	0.002469421
8049536	9208	*leucine rich repeat (in FLII) interacting protein 1*	*LRRFIP1*	9.65	9.73	9.17	2.42E-04	0.002472656

8153201	27161	*eukaryotic translation initiation factor 2C, 2*	*EIF2C2*	8.81	8.71	10.20	2.43E-04	0.002479748
8162777	9568	*gamma-aminobutyric acid (GABA) B receptor, 2*	*GABBR2*	7.58	8.78	7.41	2.43E-04	0.002480861
8103736	11341	*stimulator of chondrogenesis 1*	*SCRG1*	6.05	7.17	5.51	2.43E-04	0.002484764
8051583	1545	*cytochrome P450, family 1, subfamily B, polypeptide 1*	*CYP1B1*	11.49	11.41	7.82	2.45E-04	0.002495654
7899134	64793	*coiled-coil domain containing 21*	*CCDC21*	6.78	6.82	8.50	2.45E-04	0.002495654
8023882	9658	*zinc finger protein 516*	*ZNF516*	8.08	7.99	6.90	2.45E-04	0.002497518
8117561				5.05	5.07	6.66	2.45E-04	0.002499517
7950492	283212	*kelch-like 35 (Drosophila)*	*KLHL35*	8.77	8.74	7.46	2.46E-04	0.002501905
8127544	1915	*eukaryotic translation elongation factor 1 alpha 1*	*EEF1A1*	8.03	8.20	9.57	2.46E-04	0.002506629
8165406	56654	*neural proliferation, differentiation and control, 1*	*NPDC1*	10.25	10.30	8.54	2.46E-04	0.002508245
8149347	613210	*defensin, beta 136*	*DEFB136*	5.18	5.40	4.99	2.47E-04	0.002511485
8070777	386679	*keratin associated protein 10-2*	*KRTAP10-2*	8.55	8.61	7.82	2.47E-04	0.002512228
8073822	406883, 400931	*microRNA let-7a-3 | hypothetical LOC400931*	*MIRLET7A3|LOC400931*	7.63	7.42	6.11	2.47E-04	0.002512456
7962842	112	*adenylate cyclase 6*	*ADCY6*	9.32	8.96	7.72	2.48E-04	0.002515479
7937079	664	*BCL2/adenovirus E1B 19kDa interacting protein 3*	*BNIP3*	11.60	12.39	10.11	2.48E-04	0.002515479
8075728	4627	*myosin, heavy chain 9, non-muscle*	*MYH9*	11.95	12.16	10.35	2.48E-04	0.002515479
7928126	27143		*KIAA1274*	6.86	6.98	7.75	2.48E-04	0.002515479
7901895	84938	*ATG4 autophagy related 4 homolog C (S. cerevisiae)*	*ATG4C*	7.77	7.46	9.90	2.48E-04	0.002515479
7902396	692085	*small nucleolar RNA, C/D box 45C*	*SNORD45C*	6.99	6.84	8.55	2.48E-04	0.002515479
8146685	23212		*RRS1*	7.77	8.40	10.27	2.48E-04	0.002515728
7950899	23682		*RAB38*	8.18	7.02	9.82	2.48E-04	0.002515728
8114443	2107	*eukaryotic translation termination factor 1*	*ETF1*	8.61	8.46	10.38	2.48E-04	0.002517806
8085145	56852		*RAD18*	8.19	8.08	9.97	2.48E-04	0.002517806
7940046	10978		*CLP1*	7.90	7.99	8.83	2.49E-04	0.00251863

7928915	728118, 728130, 729262, 283008	*family with sequence similarity 22, member A | family with sequence similarity 22, member D | family with sequence similarity 22, member B | family with sequence similarity 22, member E*	*FAM22A|FAM22D|FAM22B|FAM22E*	7.55	7.61	6.99	2.49E-04	0.002519636
8127107				7.49	8.20	5.59	2.49E-04	0.002521229
7972973	387893	*SET domain containing (lysine methyltransferase) 8*	*SETD8*	8.05	8.00	7.27	2.49E-04	0.00252172
7952522	83480	*pseudouridylate synthase 3*	*PUS3*	6.74	6.63	8.28	2.49E-04	0.002522022
8060339	80023	*neurensin 2*	*NRSN2*	11.33	11.35	8.69	2.50E-04	0.002523087
7992744	84256	*FLYWCH-type zinc finger 1*	*FLYWCH1*	8.79	8.63	7.64	2.50E-04	0.002523087
8027354	388524	*ribosomal protein SA pseudogene 58*	*RPSAP58*	5.94	5.59	8.34	2.50E-04	0.002523087
8020058	4729	*NADH dehydrogenase (ubiquinone) flavoprotein 2, 24kDa*	*NDUFV2*	9.60	9.74	10.69	2.50E-04	0.002523703
8087685	11070	*transmembrane protein 115*	*TMEM115*	11.10	11.11	9.20	2.50E-04	0.002523703
7933298	83849	*synaptotagmin XV*	*SYT15*	7.93	8.01	7.09	2.50E-04	0.002523703
8157324	5998	*regulator of G-protein signaling 3*	*RGS3*	8.11	8.08	7.09	2.50E-04	0.002524506
7929562	9849	*zinc finger protein 518A*	*ZNF518A*	6.70	6.29	9.03	2.50E-04	0.002527645
8142830	375616	*kielin/chordin-like protein*	*KCP*	8.01	8.08	7.28	2.51E-04	0.002530102
8126784	7941, 221400	*phospholipase A2, group VII (platelet-activating factor acetylhydrolase, plasma) | tudor domain containing 6*	*PLA2G7|TDRD6*	5.62	5.47	7.62	2.51E-04	0.002530192
7897078	11079		*RER1*	10.31	10.34	9.33	2.51E-04	0.002534102
8158372	6418		*SET*	10.85	10.94	12.99	2.52E-04	0.002536821
8088671	151647	*family with sequence similarity 19 (chemokine (C-C motif)-like), member A4*	*FAM19A4*	6.27	6.33	7.38	2.52E-04	0.002537081
8030391				7.93	8.12	6.77	2.52E-04	0.002537081
7961026	408186, 144203	*ovostatin | ovostatin 2*	*OVOS|OVOS2*	4.97	4.97	7.22	2.52E-04	0.002539675
7953965				5.08	5.05	6.01	2.52E-04	0.002539675
8019331	255275	*myeloid-associated differentiation marker-like 2*	*MYADML2*	7.69	7.75	7.11	2.53E-04	0.002540551
8021484	28316	*cadherin 20, type 2*	*CDH20*	6.24	6.56	5.95	2.53E-04	0.00254534
7947991	23279	*nucleoporin 160kDa*	*NUP160*	7.02	6.93	10.12	2.54E-04	0.00254645

7929072	24138	*interferon-induced protein with tetratricopeptide repeats 5*	*IFIT5*	8.46	8.45	6.91	2.53E-04	0.00254645
7989347	79664	*NMDA receptor regulated 2*	*NARG2*	8.23	8.15	9.99	2.54E-04	0.00254645
7964718				6.62	6.55	5.87	2.54E-04	0.00254645
7921099	1382	*cellular retinoic acid binding protein 2*	*CRABP2*	12.09	11.42	9.89	2.54E-04	0.002547467
8153568	5339	*plectin*	*PLEC*	10.76	10.87	8.71	2.54E-04	0.002547467
7981514	113146	*AHNAK nucleoprotein 2*	*AHNAK2*	9.53	10.28	6.92	2.54E-04	0.002547467
8105607	100132916	*family with sequence similarity 159, member B*	*FAM159B*	5.95	5.94	7.20	2.54E-04	0.002547467
8137433				5.44	5.97	5.16	2.54E-04	0.002547467
8122705	5110	*protein-L-isoaspartate (D-aspartate) O-methyltransferase*	*PCMT1*	10.13	10.34	11.37	2.54E-04	0.002547827
7964183	27165	*glutaminase 2 (liver, mitochondrial)*	*GLS2*	6.87	6.65	8.20	2.55E-04	0.002551621
8085784				5.54	5.39	5.02	2.55E-04	0.002552894
8109001	11005	*serine peptidase inhibitor, Kazal type 5*	*SPINK5*	5.74	5.83	7.17	2.55E-04	0.002553073
7934733	311	*annexin A11*	*ANXA11*	12.11	11.78	10.35	2.56E-04	0.002559833
7929768	51076	*cutC copper transporter homolog (E. coli)*	*CUTC*	7.48	7.86	9.62	2.56E-04	0.002560628
8092750	2257	*fibroblast growth factor 12*	*FGF12*	6.02	6.09	7.12	2.56E-04	0.002562348
8013919	116236	*abhydrolase domain containing 15*	*ABHD15*	8.41	8.23	6.86	2.57E-04	0.002566133
8030721	6320	*C-type lectin domain family 11, member A*	*CLEC11A*	9.83	9.67	7.67	2.57E-04	0.002567816
7901867	7398	*ubiquitin specific peptidase 1*	*USP1*	7.54	7.60	9.29	2.57E-04	0.002568609
8151747	169200	*transmembrane protein 64*	*TMEM64*	8.93	8.83	10.27	2.58E-04	0.002571566
8023914	80148	*PQ loop repeat containing 1*	*PQLC1*	9.65	9.70	7.99	2.58E-04	0.0025731
8149161	349196		*LOC349196*	9.74	8.66	7.06	2.58E-04	0.002575233
7997152	10164	*carbohydrate (N-acetylglucosamine 6-O) sulfotransferase 4*	*CHST4*	6.24	6.45	7.82	2.58E-04	0.002577186
8121895	60487	*tRNA methyltransferase 11 homolog (S. cerevisiae)*	*TRMT11*	6.65	6.47	8.43	2.59E-04	0.002582568
8149210	349196		*LOC349196*	9.74	8.66	7.07	2.59E-04	0.002585836
7961142	4973	*oxidized low density lipoprotein (lectin-like) receptor 1*	*OLR1*	6.66	6.13	5.66	2.60E-04	0.002588074
8175052	9131	*apoptosis-inducing factor, mitochondrion-associated, 1*	*AIFM1*	7.68	7.80	9.55	2.60E-04	0.002591885

7918925	80263	*tripartite motif-containing 45*	*TRIM45*	7.26	6.84	7.98	2.60E-04	0.002591885
8051387	84661	*dpy-30 homolog (C. elegans)*	*DPY30*	9.73	9.92	11.57	2.61E-04	0.002591885
8090565	677797	*small nucleolar RNA, H/ACA box 7B*	*SNORA7B*	9.58	9.97	8.48	2.60E-04	0.002591885
8056963	100129455		*LOC100129455*	6.33	7.26	5.76	2.60E-04	0.002591885
8091009	5291	*phosphoinositide-3-kinase, catalytic, beta polypeptide*	*PIK3CB*	7.09	6.78	9.63	2.61E-04	0.00259371
7904314	8458	*transcription termination factor, RNA polymerase II*	*TTF2*	6.75	6.97	9.27	2.61E-04	0.002594375
7979963	8110	*D4, zinc and double PHD fingers, family 3*	*DPF3*	7.34	7.12	6.18	2.62E-04	0.002598963
8137202	643641	*zinc finger protein 862*	*ZNF862*	8.04	7.87	6.95	2.62E-04	0.002598963
8062576	60625	*DEAH (Asp-Glu-Ala-His) box polypeptide 35*	*DHX35*	7.71	7.83	9.31	2.62E-04	0.002600977
7947110	79733	*E2F transcription factor 8*	*E2F8*	6.39	6.48	7.50	2.62E-04	0.002600977
8092009	151827	*leucine rich repeat containing 34*	*LRRC34*	6.24	6.13	6.58	2.62E-04	0.002600977
7981771				8.29	8.30	7.30	2.62E-04	0.002600977
7988327	197135	*protein associated with topoisomerase II homolog 2 (yeast)*	*PATL2*	6.44	6.38	5.83	2.62E-04	0.00260296
8152222	51582	*antizyme inhibitor 1*	*AZIN1*	9.15	9.05	10.95	2.63E-04	0.002604579
7923007	51377	*ubiquitin carboxyl-terminal hydrolase L5*	*UCHL5*	7.46	7.76	9.23	2.63E-04	0.002606925
8154823	441459	*ankyrin repeat domain 18B*	*ANKRD18B*	5.15	5.26	7.44	2.63E-04	0.002606925
8014100	64149	*chromosome 17 open reading frame 75*	*C17orf75*	7.10	7.37	9.60	2.63E-04	0.002607822
8022342	10939	*AFG3 ATPase family gene 3-like 2 (S. cerevisiae)*	*AFG3L2*	8.03	7.95	9.34	2.63E-04	0.002608617
8060722	80025	*pantothenate kinase 2*	*PANK2*	8.50	8.49	9.40	2.64E-04	0.002613436
7949717				5.68	5.75	7.08	2.64E-04	0.002613436
7942957	11098	*protease, serine, 23*	*PRSS23*	8.24	9.09	5.89	2.65E-04	0.002616939
7928411	23053		*KIAA0913*	9.34	9.34	7.66	2.65E-04	0.002616939
8055980	9595	*cytohesin 1 interacting protein*	*CYTIP*	5.51	5.53	5.18	2.65E-04	0.002621014
8071155	11274	*ubiquitin specific peptidase 18*	*USP18*	8.78	7.44	7.17	2.65E-04	0.002621014
8046201	6741	*Sjogren syndrome antigen B (autoantigen La)*	*SSB*	9.88	9.62	11.84	2.66E-04	0.002630479
8140620	27445	*piccolo (presynaptic cytomatrix protein)*	*PCLO*	5.40	5.40	5.96	2.67E-04	0.00263495
7991034	9455	*homer homolog 2 (Drosophila)*	*HOMER2*	7.41	7.88	8.34	2.67E-04	0.002635237

8158714	23404	*exosome component 2*	*EXOSC2*	10.30	10.35	12.09	2.67E-04	0.002636599
7967810	2802	*golgin A3*	*GOLGA3*	9.52	9.56	8.39	2.68E-04	0.002639344
8043840	51601, 129531	*lipoyltransferase 1 | MIT, microtubule interacting and transport, domain containing 1*	*LIPT1|MITD1*	7.07	6.89	7.90	2.68E-04	0.002639344
7974214	122773	*kelch domain containing 1*	*KLHDC1*	7.32	7.00	5.16	2.68E-04	0.002639767
8045339				5.68	5.57	5.25	2.68E-04	0.002642232
8087833	1849	*dual specificity phosphatase 7*	*DUSP7*	11.68	11.66	10.09	2.69E-04	0.002644856
7978166	10548	*transmembrane 9 superfamily member 1*	*TM9SF1*	10.78	10.73	9.23	2.69E-04	0.002644856
7950864	10873	*malic enzyme 3, NADP(+)-dependent, mitochondrial*	*ME3*	8.58	8.57	7.25	2.69E-04	0.002644856
8114320	10949	*heterogeneous nuclear ribonucleoprotein A0*	*HNRNPA0*	9.73	9.88	10.68	2.69E-04	0.002644856
8171837	80311	*kelch-like 15 (Drosophila)*	*KLHL15*	7.76	7.28	8.61	2.69E-04	0.002644856
8132992				7.87	7.82	7.12	2.69E-04	0.002644856
8027304	284443	*zinc finger protein 493*	*ZNF493*	6.32	6.10	8.86	2.69E-04	0.002645935
7938329	677808	*small nucleolar RNA, H/ACA box 23*	*SNORA23*	12.92	13.12	12.61	2.69E-04	0.002645935
8003667	5176	*serpin peptidase inhibitor, clade F (alpha-2 antiplasmin, pigment epithelium derived factor), member 1*	*SERPINF1*	12.46	12.06	9.12	2.70E-04	0.002648881
8154725	3875	*keratin 18*	*KRT18*	8.02	7.95	11.38	2.70E-04	0.002649025
8129649	116843	*chromosome 6 open reading frame 192*	*C6orf192*	7.09	6.15	8.92	2.70E-04	0.002649025
8004237	440400	*ribonuclease, RNase K*	*RNASEK*	13.28	13.22	12.36	2.70E-04	0.002649025
8131815	55975	*kelch-like 7 (Drosophila)*	*KLHL7*	7.49	7.56	9.33	2.70E-04	0.002651922
7963265	5463	*POU class 6 homeobox 1*	*POU6F1*	8.64	8.69	7.40	2.71E-04	0.002653369
8059186	5798	*protein tyrosine phosphatase, receptor type, N*	*PTPRN*	7.39	9.76	7.42	2.71E-04	0.002653419
7934278	5033	*prolyl 4-hydroxylase, alpha polypeptide I*	*P4HA1*	10.11	10.78	8.73	2.71E-04	0.002654363
8131337				7.00	7.21	6.13	2.71E-04	0.002654363
8145702	7486	*Werner syndrome, RecQ helicase-like*	*WRN*	7.25	7.29	9.96	2.71E-04	0.002654984
7972269	23483	*TDP-glucose 4,6-dehydratase*	*TGDS*	6.38	6.47	7.29	2.71E-04	0.00265658
8088535	9861	*proteasome (prosome, macropain) 26S subunit, non-ATPase, 6*	*PSMD6*	7.87	8.17	9.55	2.72E-04	0.002657737
8140070	26608	*transducin (beta)-like 2*	*TBL2*	9.39	9.50	7.93	2.72E-04	0.002657737

8030557	22809	*activating transcription factor 5*	*ATF5*	10.32	10.82	8.46	2.72E-04	0.002658834
7938390	133	*adrenomedullin*	*ADM*	12.84	12.85	10.40	2.73E-04	0.002665741
8115957	3101	*hexokinase 3 (white cell)*	*HK3*	6.41	6.37	6.02	2.73E-04	0.002665741
7923621	3814	*KiSS-1 metastasis-suppressor*	*KISS1*	7.49	7.50	6.66	2.73E-04	0.002665741
7898585	4681	*neuroblastoma, suppression of tumorigenicity 1*	*NBL1*	9.35	9.40	8.03	2.73E-04	0.002665836
8179688	10211	*flotillin 1*	*FLOT1*	10.88	10.71	9.33	2.73E-04	0.002665836
8177323	9081, 442862	*PTPN13-like, Y-linked | PTPN13-like, Y-linked 2*	*PRY|PRY2*	5.95	6.01	5.59	2.73E-04	0.002665836
8138721	3200	*homeobox A3*	*HOXA3*	7.11	7.69	6.51	2.73E-04	0.00266795
7938139	144132	*dynein heavy chain domain 1*	*DNHD1*	6.89	6.97	6.35	2.74E-04	0.002671767
8177186	9085, 253175, 9426, 203611	*chromodomain protein, Y-linked, 1 | chromodomain protein, Y-linked, 1B | chromodomain protein, Y-linked, 2A | chromodomain protein, Y-linked, 2B*	*CDY1|CDY1B|CDY2A|CDY2B*	5.06	5.20	4.97	2.74E-04	0.002671953
8150978	767	*carbonic anhydrase VIII*	*CA8*	5.16	5.31	5.74	2.74E-04	0.002672242
8114215	5307	*paired-like homeodomain 1*	*PITX1*	10.02	8.14	7.36	2.74E-04	0.002672242
8149315				5.57	5.49	6.69	2.74E-04	0.002672242
7906348	911	*CD1c molecule*	*CD1C*	6.01	5.83	5.73	2.75E-04	0.002672905
8139232	5683	*proteasome (prosome, macropain) subunit, alpha type, 2*	*PSMA2*	10.48	10.61	12.17	2.75E-04	0.002674826
8121087	135293	*peptidase M20 domain containing 2*	*PM20D2*	7.27	7.18	9.27	2.75E-04	0.002674826
8093413	84286	*transmembrane protein 175*	*TMEM175*	10.05	10.03	7.76	2.76E-04	0.002680768
8061171	10621, 55184	*polymerase (RNA) III (DNA directed) polypeptide F, 39 kDa | chromosome 20 open reading frame 12*	*POLR3F|C20orf12*	6.70	6.69	8.55	2.76E-04	0.002684061
8043310	64795	*required for meiotic nuclear division 5 homolog A (S. cerevisiae)*	*RMND5A*	7.69	7.64	10.51	2.76E-04	0.002684241
8030954	7696	*zinc finger protein 137, pseudogene*	*ZNF137P*	5.52	5.41	6.96	2.77E-04	0.002690843
7924476	9015	*TATA box binding protein (TBP)-associated factor, RNA polymerase I, A, 48kDa*	*TAF1A*	5.64	5.77	7.28	2.77E-04	0.002690843
7956842	23592	*LEM domain containing 3*	*LEMD3*	7.66	7.49	8.89	2.77E-04	0.002690843
8039933	55629	*proline-rich nuclear receptor coactivator 2*	*PNRC2*	10.36	10.28	12.03	2.77E-04	0.002690843
7901497	440590	*zyg-11 homolog A (C. elegans)*	*ZYG11A*	5.82	5.82	7.48	2.77E-04	0.002690843

8050238				6.33	6.19	5.90	2.77E-04	0.002690843
7964759	23426	*glutamate receptor interacting protein 1*	*GRIP1*	5.75	5.77	7.00	2.78E-04	0.002696361
8029701	147699	*protein phosphatase, Mg2+/Mn2+ dependent, 1N (putative)*	*PPM1N*	7.44	7.21	8.77	2.78E-04	0.002697269
8052866	84908	*family with sequence similarity 136, member A*	*FAM136A*	8.67	8.91	10.67	2.79E-04	0.002698909
7908639	55765	*chromosome 1 open reading frame 106*	*C1orf106*	6.89	6.83	7.70	2.79E-04	0.00270176
8176149	246100, 1485, 30848	*cancer/testis antigen 1A | cancer/testis antigen 1B | cancer/testis antigen 2*	*CTAG1A|CTAG1B|CTAG2*	7.75	7.84	7.25	2.79E-04	0.00270176
7962703	1280	*collagen, type II, alpha 1*	*COL2A1*	7.82	8.01	7.01	2.79E-04	0.002703909
8043251	55037	*Pentatricopeptide repeat domain 3*	*PTCD3*	8.97	8.81	11.57	2.79E-04	0.002703909
7963817	121355	*gametocyte specific factor 1*	*GTSF1*	5.71	5.56	6.66	2.79E-04	0.002703909
8046373	1745	*distal-less homeobox 1*	*DLX1*	8.53	7.78	6.77	2.80E-04	0.002705121
8005957	26772	*small nucleolar RNA, C/D box 4B*	*SNORD4B*	8.34	8.25	10.04	2.80E-04	0.002706936
7986246	145858	*chromosome 15 open reading frame 32*	*C15orf32*	8.75	8.98	10.07	2.81E-04	0.002712882
8074597	729461, 284861	*hypothetical LOC729461 | hypothetical LOC284861*	*LOC729461|LOC284861*	5.71	5.85	5.36	2.81E-04	0.002718757
8178419	10211	*flotillin 1*	*FLOT1*	10.88	10.72	9.33	2.82E-04	0.002719628
8146863	23213	*sulfatase 1*	*SULF1*	12.01	12.00	6.97	2.82E-04	0.002719628
8124828	10211	*flotillin 1*	*FLOT1*	10.88	10.72	9.33	2.82E-04	0.002721741
7916229	55268	*enoyl CoA hydratase domain containing 2*	*ECHDC2*	8.04	7.59	7.02	2.82E-04	0.002721741
8114920	1809	*dihydropyrimidinase-like 3*	*DPYSL3*	10.12	10.62	11.81	2.83E-04	0.002725277
8121118	9994	*caspase 8 associated protein 2*	*CASP8AP2*	7.11	7.14	8.75	2.83E-04	0.00272731
8041617	57504	*metastasis associated 1 family, member 3*	*MTA3*	7.90	7.72	9.61	2.83E-04	0.00272731
7973985	145282	*mirror-image polydactyly 1*	*MIPOL1*	6.76	6.42	7.94	2.83E-04	0.00272731
8037657	1760	*dystrophia myotonica-protein kinase*	*DMPK*	9.95	9.52	7.76	2.83E-04	0.002729274
8136448	1129	*cholinergic receptor, muscarinic 2*	*CHRM2*	9.21	6.87	5.68	2.84E-04	0.002733077
8037614	2828	*G protein-coupled receptor 4*	*GPR4*	8.41	7.39	6.77	2.84E-04	0.002733077
7973732	23351	*KH and NYN domain containing*	*KHNYN*	7.85	7.87	6.96	2.84E-04	0.002733077

7972579	84899	*transmembrane and tetratricopeptide repeat containing 4*	*TMTC4*	8.01	7.85	9.06	2.84E-04	0.002733077
7925184	23029, 51742	*RNA binding motif protein 34 | AT rich interactive domain 4B (RBP1-like)*	*RBM34|ARID4B*	7.59	7.63	8.60	2.84E-04	0.002733077
7897439				6.66	6.59	5.93	2.84E-04	0.002733077
8027938	11045	*uroplakin 1A*	*UPK1A*	8.08	6.76	6.75	2.85E-04	0.002733894
7950983	26973	*cysteine and histidine-rich domain (CHORD)-containing 1*	*CHORDC1*	7.64	7.51	9.73	2.85E-04	0.002733894
8155148	51754	*transmembrane protein 8B*	*TMEM8B*	9.02	8.58	7.76	2.85E-04	0.002733894
7916789	79819	*WD repeat domain 78*	*WDR78*	5.99	5.81	5.15	2.85E-04	0.002733894
8149811	4824	*NK3 homeobox 1*	*NKX3-1*	7.51	7.83	7.27	2.85E-04	0.002739116
7950473	408	*arrestin, beta 1*	*ARRB1*	10.83	8.86	10.05	2.86E-04	0.002745211
7941302	23625	*family with sequence similarity 89, member B*	*FAM89B*	10.93	11.18	8.62	2.87E-04	0.002748095
7920271	6275	*S100 calcium binding protein A4*	*S100A4*	10.41	10.01	7.12	2.87E-04	0.002749328
7971296	94240	*epithelial stromal interaction 1 (breast)*	*EPSTI1*	6.67	6.66	5.61	2.87E-04	0.002749328
8123717	10799	*ribonuclease P/MRP 40kDa subunit*	*RPP40*	7.45	7.59	8.90	2.87E-04	0.00275079
8067203	55544, 100291105	*RNA binding motif protein 38 | hypothetical LOC100291105*	*RBM38|LOC100291105*	6.69	6.82	6.42	2.87E-04	0.00275079
8034401	163050	*zinc finger protein 564*	*ZNF564*	7.57	7.42	8.63	2.87E-04	0.002752225
8174313	79710	*MORC family CW-type zinc finger 4*	*MORC4*	9.11	8.59	6.99	2.88E-04	0.002753784
8173629	22	*ATP-binding cassette, sub-family B (MDR/TAP), member 7*	*ABCB7*	7.94	7.64	9.96	2.89E-04	0.002762183
8006621	9560, 388372, 6351	*chemokine (C-C motif) ligand 4-like 1 | chemokine (C-C motif) ligand 4-like 2 | chemokine (C-C motif) ligand 4*	*CCL4L1|CCL4L2*	5.77	5.79	5.44	2.89E-04	0.002762183
7963911	967		*CD63*	11.40	11.52	9.86	2.89E-04	0.002766681
8086389				5.49	5.55	6.90	2.89E-04	0.002766681
8161654	80036	*transient receptor potential cation channel, subfamily M, member 3*	*TRPM3*	6.85	6.36	7.53	2.90E-04	0.002769867
8126102	266727	*MAM domain containing glycosylphosphatidylinositol anchor 1*	*MDGA1*	8.02	7.79	5.99	2.90E-04	0.002771062

8081073	285237, 8545	*chromosome 3 open reading frame 38 | CGG triplet repeat binding protein 1*	*C3orf38|CGGBP1*	8.25	8.22	9.40	2.91E-04	0.002777648
8172244	139341	*FUN14 domain containing 1*	*FUNDC1*	7.96	7.94	8.69	2.91E-04	0.002778449
8081953	2960	*general transcription factor IIE, polypeptide 1, alpha 56kDa*	*GTF2E1*	7.13	7.16	9.92	2.91E-04	0.002781875
8051605	92906	*heterogeneous nuclear ribonucleoprotein L-like*	*HNRPLL*	7.87	7.96	8.88	2.92E-04	0.002783088
7900438	65243	*zinc finger protein 643*	*ZNF643*	6.64	6.73	7.89	2.92E-04	0.002784152
7926708	79896	*threonine synthase-like 1 (S. cerevisiae)*	*THNSL1*	5.81	5.74	7.64	2.92E-04	0.002784152
8111922	648987		*LOC648987*	6.73	7.39	5.86	2.92E-04	0.002784152
7980069	51004, 145483	*coenzyme Q6 homolog, monooxygenase (S. cerevisiae) | family with sequence similarity 161, member B*	*COQ6|FAM161B*	7.59	7.79	6.62	2.93E-04	0.002787027
8082058	1475	*cystatin A (stefin A)*	*CSTA*	6.35	7.21	5.65	2.93E-04	0.002793952
7952243	114902, 83552	*C1q and tumor necrosis factor related protein 5 | membrane frizzled-related protein*	*C1QTNF5|MFRP*	9.68	9.85	8.02	2.94E-04	0.002795232
8137517	3361	*5-hydroxytryptamine (serotonin) receptor 5A*	*HTR5A*	6.33	6.45	5.97	2.94E-04	0.002799801
7898910	55629	*proline-rich nuclear receptor coactivator 2*	*PNRC2*	10.37	10.28	12.04	2.94E-04	0.002799801
8065990				4.76	4.72	5.20	2.95E-04	0.002802675
8034342	90589, 7568	*zinc finger protein 625 | zinc finger protein 20*	*ZNF625|ZNF20*	7.94	8.09	10.46	2.95E-04	0.002806374
7969693	5911		*RAP2A*	8.18	8.58	9.48	2.95E-04	0.002807885
8147303				9.82	9.85	9.29	2.95E-04	0.002807885
8111941	3157	*3-hydroxy-3-methylglutaryl-CoA synthase 1 (soluble)*	*HMGCS1*	7.56	6.86	10.51	2.96E-04	0.002808598
8016878	404093	*CUE domain containing 1*	*CUEDC1*	9.92	9.72	7.56	2.96E-04	0.002808598
8121095	22881, 57226	*ankyrin repeat domain 6 | LYR motif containing 2*	*ANKRD6|LYRM2*	6.78	6.75	7.58	2.96E-04	0.002808598
7939902	646813	*DEAH (Asp-Glu-Ala-His) box polypeptide 9 pseudogene*	*LOC646813*	7.23	7.17	9.21	2.96E-04	0.002809173
8148512	389690		*FLJ43860*	8.09	8.04	7.42	2.96E-04	0.002811853
7969048				9.36	9.37	8.59	2.97E-04	0.00281699
7980344	283576	*zinc finger, DHHC-type containing 22*	*ZDHHC22*	6.58	6.70	8.16	2.97E-04	0.002817452
7962058	83857	*transmembrane and tetratricopeptide repeat containing 1*	*TMTC1*	10.39	7.02	8.31	2.97E-04	0.002817515

7918294	127003	*chromosome 1 open reading frame 194*	*C1orf194*	6.64	6.66	6.14	2.98E-04	0.002822097
8086372	54986	*unc-51-like kinase 4 (C. elegans)*	*ULK4*	6.07	6.12	7.35	2.98E-04	0.002823365
7919028	6913	*T-box 15*	*TBX15*	9.33	9.04	5.65	2.98E-04	0.002825239
8131067	115330	*G protein-coupled receptor 146*	*GPR146*	7.25	7.96	6.46	2.98E-04	0.002825239
8170965	246100, 1485, 30848	*cancer/testis antigen 1A | cancer/testis antigen 1B | cancer/testis antigen 2*	*CTAG1A|CTAG1B|CTAG2*	7.75	7.84	7.24	2.99E-04	0.002826635
8149399	91694	*LON peptidase N-terminal domain and ring finger 1*	*LONRF1*	7.51	7.60	9.41	3.00E-04	0.002834619
8153390	116447	*topoisomerase (DNA) I, mitochondrial*	*TOP1MT*	8.64	8.16	9.39	3.00E-04	0.002840244
8030374	2217	*Fc fragment of IgG, receptor, transporter, alpha*	*FCGRT*	10.01	9.68	8.47	3.01E-04	0.002841818
8019250	5034	*prolyl 4-hydroxylase, beta polypeptide*	*P4HB*	13.37	13.48	12.48	3.02E-04	0.002849452
7970511				4.98	5.08	5.86	3.02E-04	0.002849452
7918593	389	*ras homolog gene family, member C*	*RHOC*	12.06	12.38	10.22	3.02E-04	0.002849764
7952011	6327	*sodium channel, voltage-gated, type II, beta*	*SCN2B*	6.67	6.62	6.18	3.02E-04	0.002852504
8164580	9536	*prostaglandin E synthase*	*PTGES*	9.46	8.96	7.79	3.02E-04	0.002852504
8153609	84875	*poly (ADP-ribose) polymerase family, member 10*	*PARP10*	8.46	8.34	7.21	3.02E-04	0.002852504
8061579	22974		*TPX2*	7.76	8.35	10.88	3.03E-04	0.00285357
7945283	27034	*acyl-CoA dehydrogenase family, member 8*	*ACAD8*	8.76	8.76	9.96	3.03E-04	0.002857795
8167656	9500	*melanoma antigen family D, 1*	*MAGED1*	12.82	12.61	11.21	3.03E-04	0.002858145
7906264	375033	*platelet endothelial aggregation receptor 1*	*PEAR1*	9.78	8.99	6.98	3.04E-04	0.002864339
8132843	54801	*HAUS augmin-like complex, subunit 6*	*HAUS6*	6.63	6.45	9.55	3.04E-04	0.002867775
8080419	26354	*guanine nucleotide binding protein-like 3 (nucleolar)*	*GNL3*	8.35	8.36	10.48	3.05E-04	0.002871446
8057045	51661	*FK506 binding protein 7*	*FKBP7*	9.43	9.17	7.45	3.05E-04	0.002871446
7947199	55366	*leucine-rich repeat-containing G protein-coupled receptor 4*	*LGR4*	8.44	9.29	10.82	3.05E-04	0.002871446
8149324	83648	*family with sequence similarity 167, member A*	*FAM167A*	8.80	8.54	6.38	3.06E-04	0.002875529
8053231	84695, 27429	*lysyl oxidase-like 3 | HtrA serine peptidase 2*	*LOXL3|HTRA2*	9.99	9.87	7.92	3.06E-04	0.002875529
8160581	10210	*topoisomerase I binding, arginine/serine-rich, E3 ubiquitin protein ligase*	*TOPORS*	7.49	7.50	9.08	3.07E-04	0.002881433

8169882	63035	*BCL6 corepressor-like 1*	*BCORL1*	9.02	8.90	7.48	3.07E-04	0.002881433
8060949	63926	*ankyrin repeat domain 5*	*ANKRD5*	5.91	5.98	7.47	3.07E-04	0.0028823
7965846	51019	*coiled-coil domain containing 53*	*CCDC53*	9.56	9.75	7.99	3.08E-04	0.002889267
8171493	56474	*CTP synthase II*	*CTPS2*	7.97	7.84	10.33	3.08E-04	0.002889267
7975521	58517	*RNA binding motif protein 25*	*RBM25*	7.85	7.65	10.19	3.08E-04	0.002889267
7906372	128368	*olfactory receptor, family 10, subfamily Z, member 1*	*OR10Z1*	5.87	5.74	5.44	3.08E-04	0.002889267
8133114	154807	*vitamin K epoxide reductase complex, subunit 1-like 1*	*VKORC1L 1*	9.43	10.03	10.41	3.08E-04	0.002889267
8074972	25774, 653399, 2952	*glutathione S-transferase theta pseudogene 1 | glutathione S-transferase theta pseudogene 2 | glutathione S-transferase theta 1*	*GSTTP1|G STTP2|GS TT1*	5.70	5.65	5.34	3.08E-04	0.002889267
8171336				7.95	8.09	7.22	3.08E-04	0.002889267
8058940	57695	*ubiquitin specific peptidase 37*	*USP37*	6.33	6.19	8.64	3.09E-04	0.002892005
7954503	9412	*mediator complex subunit 21*	*MED21*	9.60	9.77	11.01	3.10E-04	0.002903892
7910651	6905	*tubulin folding cofactor E*	*TBCE*	7.90	8.21	10.07	3.10E-04	0.002906363
7940473	51524	*transmembrane protein 138*	*TMEM138*	10.13	10.21	9.09	3.10E-04	0.002906363
8102362	92610	*TRAF-interacting protein with forkhead-associated domain*	*TIFA*	6.50	6.96	6.14	3.11E-04	0.002909147
8059319	10056	*phenylalanyl-tRNA synthetase, beta subunit*	*FARSB*	9.56	9.90	11.83	3.12E-04	0.002916783
7966441	51275	*chromosome 12 open reading frame 47*	*C12orf47*	7.36	7.28	8.84	3.13E-04	0.002923308
8114300	26249	*kelch-like 3 (Drosophila)*	*KLHL3*	6.86	6.35	7.56	3.13E-04	0.00292604
8090193	57493	*HEG homolog 1 (zebrafish)*	*HEG1*	12.04	11.74	8.21	3.13E-04	0.002927572
7968789	28984	*chromosome 13 open reading frame 15*	*C13orf15*	10.02	8.33	6.64	3.14E-04	0.002930084
8160981	80256		*KIAA1539*	9.46	10.03	7.73	3.14E-04	0.002931901
8068280	3460	*interferon gamma receptor 2 (interferon gamma transducer 1)*	*IFNGR2*	10.12	10.09	9.21	3.14E-04	0.002932058
7930927	22876	*inositol polyphosphate-5-phosphatase F*	*INPP5F*	6.78	6.88	9.63	3.14E-04	0.002934173
8128620	57673	*BEN domain containing 3*	*BEND3*	7.23	7.49	9.46	3.15E-04	0.002934173
8054762	118433, 284942	*ribosomal protein L23a pseudogene 7 | ribosomal protein L23a pseudogene 82*	*RPL23AP7 |RPL23AP 82*	9.81	10.02	9.52	3.15E-04	0.002934173

8094870	152573	*shisa homolog 3 (Xenopus laevis)*	*SHISA3*	6.43	6.71	7.76	3.15E-04	0.002937401
8068543	257203	*Down syndrome critical region gene 9 (non-protein coding)*	*DSCR9*	5.71	5.76	5.33	3.15E-04	0.002937401
8019651	9560, 388372, 6351	*chemokine (C-C motif) ligand 4-like 1 | chemokine (C-C motif) ligand 4-like 2 | chemokine (C-C motif) ligand 4*	*CCL4L1|CCL4L2*	5.77	5.79	5.45	3.15E-04	0.002937401
8042111				4.88	5.09	4.61	3.15E-04	0.002937401
7982358	9824	*Rho GTPase activating protein 11A*	*ARHGAP11A*	6.40	6.51	9.38	3.16E-04	0.002938585
7903972	515	*ATP synthase, H+ transporting, mitochondrial Fo complex, subunit B1*	*ATP5F1*	7.28	7.34	7.97	3.16E-04	0.002938906
8090469	2624	*GATA binding protein 2*	*GATA2*	8.19	8.23	6.82	3.16E-04	0.002939488
8005661	92521	*sperm antigen with calponin homology and coiled-coil domains 1*	*SPECC1*	8.80	8.47	10.23	3.16E-04	0.002943595
7942964	65084	*transmembrane protein 135*	*TMEM135*	7.89	7.36	9.37	3.17E-04	0.00294533
8124040	6310	*ataxin 1*	*ATXN1*	9.33	9.64	7.69	3.17E-04	0.002946048
8107458	51397	*COMM domain containing 10*	*COMMD10*	7.76	7.81	9.52	3.17E-04	0.002946048
8030950	55762	*zinc finger protein 701*	*ZNF701*	6.31	6.09	7.73	3.17E-04	0.002946048
8143564	9715	*family with sequence similarity 131, member B*	*FAM131B*	7.78	7.74	8.76	3.18E-04	0.002949753
8005043	284034		*FLJ34690*	5.26	5.32	5.01	3.18E-04	0.002950399
8124684	346171	*zinc finger protein 57 homolog (mouse)*	*ZFP57*	6.67	6.62	10.50	3.18E-04	0.002950399
8080158	118442, 57060	*G protein-coupled receptor 62 | poly(rC) binding protein 4*	*GPR62|PCBP4*	7.81	7.79	7.11	3.18E-04	0.002950399
7903049	343099, 50999	*coiled-coil domain containing 18 | transmembrane emp24 protein transport domain containing 5*	*CCDC18|TMED5*	5.34	5.32	6.91	3.18E-04	0.002952716
8148703	375686	*spermatogenesis and centriole associated 1*	*SPATC1*	7.51	7.51	6.72	3.19E-04	0.002955508
8156026	84131	*centrosomal protein 78kDa*	*CEP78*	6.60	6.84	9.44	3.19E-04	0.002957459
7952927	283358	*beta-1,4-N-acetyl-galactosaminyl transferase 3*	*B4GALNT3*	7.20	7.34	8.44	3.19E-04	0.002958375
8082012	6565	*solute carrier family 15 (H+/peptide transporter), member 2*	*SLC15A2*	5.42	5.46	6.15	3.20E-04	0.002960978
8175209	51270	*transcription factor Dp family, member 3*	*TFDP3*	5.78	5.67	5.33	3.20E-04	0.002962609

7981425				7.82	7.81	6.74	3.20E-04	0.002966318
7957890	27340		*UTP20*	6.97	7.00	9.85	3.20E-04	0.002966494
8103005	10393	*anaphase promoting complex subunit 10*	*ANAPC10*	6.94	7.13	7.93	3.21E-04	0.002971903
8083136	483	*ATPase, Na+/K+ transporting, beta 3 polypeptide*	*ATP1B3*	8.96	9.18	10.51	3.22E-04	0.002974054
8107814	51015	*isochorismatase domain containing 1*	*ISOC1*	8.14	8.00	9.51	3.22E-04	0.002974054
8044473	84172	*polymerase (RNA) I polypeptide B, 128kDa*	*POLR1B*	7.25	7.61	9.93	3.22E-04	0.002974054
8080138	389123	*IQ motif containing F2*	*IQCF2*	4.66	4.82	4.57	3.22E-04	0.002975203
8102039	150159	*Na+/H+ exchanger domain containing 1*	*NHEDC1*	5.23	5.06	4.98	3.22E-04	0.002977918
7911287	391211	*olfactory receptor, family 2, subfamily G, member 6*	*OR2G6*	6.14	6.09	5.69	3.23E-04	0.002985454
8118863	23294	*ankyrin repeat and sterile alpha motif domain containing 1A*	*ANKS1A*	9.08	8.54	9.31	3.23E-04	0.002986181
7939587	374387		*DKFZp779 M0652*	8.24	8.16	7.52	3.25E-04	0.003000206
8151572	64089	*sorting nexin 16*	*SNX16*	6.47	6.55	7.63	3.26E-04	0.003006338
8015247	85285, 85291	*keratin associated protein 4-1 | keratin associated protein 4-2*	*KRTAP4-1|KRTAP4-2*	7.26	7.32	6.79	3.26E-04	0.003006375
7965838				5.62	5.55	7.13	3.26E-04	0.003008333
8126760	10231	*regulator of calcineurin 2*	*RCAN2*	9.98	10.30	7.86	3.27E-04	0.003009761
8097146				6.52	6.45	5.95	3.27E-04	0.003013808
8066136	5933	*retinoblastoma-like 1 (p107)*	*RBL1*	6.70	6.73	8.29	3.28E-04	0.003014619
7977621	57447	*NDRG family member 2*	*NDRG2*	7.84	7.61	9.71	3.28E-04	0.003014619
7912511	90231		*KIAA2013*	9.46	9.73	8.53	3.27E-04	0.003014619
7905043	388692, 644634	*hypothetical LOC388692 | UPF0627 protein ENSP00000358171-like*	*LOC388692|LOC644634*	7.32	7.14	6.60	3.28E-04	0.003014619
8039070	342926	*zinc finger protein 677*	*ZNF677*	5.67	5.57	7.02	3.28E-04	0.003019523
8131967				5.18	5.58	4.87	3.28E-04	0.003019523
8119898	7422	*vascular endothelial growth factor A*	*VEGFA*	10.26	11.02	9.11	3.29E-04	0.003022612
7949532	8061	*FOS-like antigen 1*	*FOSL1*	9.93	10.65	9.04	3.29E-04	0.003022812

7965686	56899	*ankyrin repeat and sterile alpha motif domain containing 1B*	*ANKS1B*	5.25	5.37	6.08	3.29E-04	0.003022812
8105487	115827		*RAB3C*	5.64	5.54	7.96	3.29E-04	0.003022812
8030993	388561	*zinc finger protein 761*	*ZNF761*	6.49	6.31	8.29	3.29E-04	0.003022812
7930274				5.49	5.43	4.85	3.29E-04	0.003022812
8111974	10605	*poly(A) binding protein interacting protein 1*	*PAIP1*	9.00	9.26	10.15	3.31E-04	0.00303556
7929882	57715	*sema domain, immunoglobulin domain (Ig), transmembrane domain (TM) and short cytoplasmic domain, (semaphorin) 4G*	*SEMA4G*	8.77	7.95	7.61	3.31E-04	0.003035736
8026300	976		*CD97*	10.37	10.31	7.68	3.32E-04	0.003038717
8001387	6299	*sal-like 1 (Drosophila)*	*SALL1*	8.41	8.47	9.31	3.32E-04	0.003038717
8073943	9889	*zinc finger, BED-type containing 4*	*ZBED4*	7.60	7.89	8.71	3.32E-04	0.003038717
7942527	10714	*polymerase (DNA-directed), delta 3, accessory subunit*	*POLD3*	7.20	7.39	9.50	3.31E-04	0.003038717
8069811	337963	*keratin associated protein 23-1*	*KRTAP23-1*	6.84	6.89	6.24	3.32E-04	0.003038717
8096158				6.93	7.15	6.35	3.32E-04	0.003043465
7962226	341359	*synaptotagmin X*	*SYT10*	6.20	6.39	7.21	3.34E-04	0.003053141
8069987	140290	*t-complex 10 (mouse)-like*	*TCP10L*	6.54	6.76	6.13	3.34E-04	0.003055594
8146198	5423	*polymerase (DNA directed), beta*	*POLB*	8.16	8.21	10.01	3.34E-04	0.003058427
7937485	57104	*patatin-like phospholipase domain containing 2*	*PNPLA2*	11.26	11.13	9.39	3.34E-04	0.003058427
8079198	285349, 10168	*zinc finger protein 660 | zinc finger protein 197*	*ZNF660|ZNF197*	5.75	5.53	7.42	3.35E-04	0.003059026
7991332	55897	*mesoderm posterior 1 homolog (mouse)*	*MESP1*	8.26	8.35	7.50	3.35E-04	0.00305987
7962884	27289	*Rho family GTPase 1*	*RND1*	6.44	6.34	7.70	3.35E-04	0.003061385
8060738				11.35	11.26	10.73	3.35E-04	0.003063957
7950597	1207	*chloride channel, nucleotide-sensitive, 1A*	*CLNS1A*	10.10	9.96	11.23	3.36E-04	0.00307087
8019669	2642	*glucagon receptor*	*GCGR*	7.77	7.78	7.16	3.37E-04	0.00307087
7933237	93550	*AN1, ubiquitin-like, homolog (Xenopus laevis)*	*ANUBL1*	7.21	6.83	8.53	3.36E-04	0.00307087
8036365	374899	*zinc finger protein 829*	*ZNF829*	6.50	6.52	8.07	3.37E-04	0.00307087
8118826	6631	*small nuclear ribonucleoprotein polypeptide C*	*SNRPC*	9.25	9.54	10.39	3.37E-04	0.003072

8107100	285704	*RGM domain family, member B*	*RGMB*	10.34	10.42	8.57	3.37E-04	0.003073556
8168674				7.29	7.09	8.67	3.37E-04	0.003074207
8086024	389102	*hypothetical LOC389102*	*YPLR6490*	6.25	6.19	7.51	3.38E-04	0.00307667
7955142	784	*calcium channel, voltage-dependent, beta 3 subunit*	*CACNB3*	10.51	10.09	8.30	3.38E-04	0.003079907
8112592	2297	*forkhead box D1*	*FOXD1*	9.03	10.66	7.40	3.39E-04	0.003084252
7949400	283130	*solute carrier family 25, member 45*	*SLC25A45*	8.28	8.02	6.66	3.39E-04	0.003084252
8001651	57567	*zinc finger protein 319*	*ZNF319*	8.44	8.38	6.82	3.39E-04	0.00308497
8022380	79959	*centrosomal protein 76kDa*	*CEP76*	6.52	6.62	8.03	3.41E-04	0.003098453
8085062	3568	*interleukin 5 receptor, alpha*	*IL5RA*	5.05	5.11	4.83	3.41E-04	0.003099383
8025697	3609	*interleukin enhancer binding factor 3, 90kDa*	*ILF3*	10.07	10.05	11.36	3.41E-04	0.003100695
8131475	56913	*core 1 synthase, glycoprotein-N-acetylgalactosamine 3-beta-galactosyltransferase, 1*	*C1GALT1*	7.72	8.98	10.58	3.41E-04	0.00310147
7902623	58511	*deoxyribonuclease II beta*	*DNASE2B*	5.19	5.30	5.02	3.42E-04	0.003104714
8136614	55750	*acylglycerol kinase*	*AGK*	8.31	8.40	9.61	3.42E-04	0.003105028
8081503	9666	*DAZ interacting protein 3, zinc finger*	*DZIP3*	6.79	6.32	9.26	3.42E-04	0.003108974
8109528	26999	*cytoplasmic FMR1 interacting protein 2*	*CYFIP2*	8.17	7.63	10.03	3.43E-04	0.003108974
7934161	5551	*perforin 1 (pore forming protein)*	*PRF1*	6.69	6.68	6.12	3.43E-04	0.003109931
8049448	116987	*ArfGAP with GTPase domain, ankyrin repeat and PH domain 1*	*AGAP1*	8.54	8.86	9.08	3.44E-04	0.003118927
7952290	23650	*tripartite motif-containing 29*	*TRIM29*	6.71	6.99	6.27	3.44E-04	0.003120518
8107578	153443	*serum response factor binding protein 1*	*SRFBP1*	7.77	7.86	10.05	3.44E-04	0.00312104
8060379	9491	*proteasome (prosome, macropain) inhibitor subunit 1 (PI31)*	*PSMF1*	11.87	11.80	11.03	3.45E-04	0.003124373
7945275	112936	*vacuolar protein sorting 26 homolog B (S. pombe)*	*VPS26B*	10.47	10.46	8.93	3.45E-04	0.003124643
8085852	55768	*N-glycanase 1*	*NGLY1*	7.38	7.53	9.17	3.45E-04	0.003125387
8137627	10049, 65084	*DnaJ (Hsp40) homolog, subfamily B, member 6 | transmembrane protein 135*	*DNAJB6|TMEM135*	9.13	9.39	11.18	3.45E-04	0.003126515
7918869	4803	*nerve growth factor (beta polypeptide)*	*NGF*	10.32	10.24	8.88	3.46E-04	0.003129607
8039013	399669	*zinc finger protein 321*	*ZNF321*	5.99	5.98	7.42	3.46E-04	0.003130859
8081233				5.04	5.09	6.74	3.46E-04	0.003131172

8165843	416	*arylsulfatase F*	*ARSF*	5.14	5.24	4.93	3.47E-04	0.003136982
7906496	3765	*potassium inwardly-rectifying channel, subfamily J, member 9*	*KCNJ9*	7.07	7.14	6.50	3.47E-04	0.003136982
8109938	64901	*RAN binding protein 17*	*RANBP17*	6.93	7.21	8.83	3.47E-04	0.003136982
8113124	84250	*ankyrin repeat domain 32*	*ANKRD32*	4.92	4.92	6.10	3.47E-04	0.003136982
8013606	7448	*vitronectin*	*VTN*	9.60	7.55	6.39	3.48E-04	0.003139041
8104022	27295	*PDZ and LIM domain 3*	*PDLIM3*	6.39	6.44	7.24	3.48E-04	0.003142994
7966462	80018	*N(alpha)-acetyltransferase 25, NatB auxiliary subunit*	*NAA25*	7.41	7.18	10.18	3.50E-04	0.003157809
8037079	478	*ATPase, Na+/K+ transporting, alpha 3 polypeptide*	*ATP1A3*	7.70	7.20	9.36	3.50E-04	0.003158102
8117543	85235	*histone cluster 1, H2ah*	*HIST1H2AH*	6.09	6.34	8.77	3.50E-04	0.003158102
7971920	220988, 10151	*heterogeneous nuclear ribonucleoprotein A3 | heterogeneous nuclear ribonucleoprotein A3 pseudogene 1*	*HNRNPA3|HNRNPA3P1*	8.39	8.46	11.40	3.50E-04	0.003158102
7904572				7.30	7.36	11.00	3.51E-04	0.003162196
8173174	158880	*ubiquitin specific peptidase 51*	*USP51*	6.83	6.63	7.94	3.51E-04	0.003162937
8010983	29	*active BCR-related gene*	*ABR*	10.47	10.27	8.77	3.52E-04	0.003171678
8151471	28957, 7163	*mitochondrial ribosomal protein S28 | tumor protein D52*	*MRPS28|TPD52*	6.63	6.94	8.07	3.52E-04	0.003171678
7927202	7570, 220979	*zinc finger protein 22 (KOX 15) | chromosome 10 open reading frame 25*	*ZNF22|C10orf25*	7.04	7.30	8.67	3.52E-04	0.003171678
8157818	401551	*WD repeat domain 38*	*WDR38*	7.22	7.32	6.80	3.53E-04	0.003172597
7970624	55835	*centromere protein J*	*CENPJ*	6.50	6.41	8.56	3.53E-04	0.003177362
8154153	55664	*cell division cycle 37 homolog (S. cerevisiae)-like 1*	*CDC37L1*	7.26	7.38	8.31	3.54E-04	0.003180864
8001178	388272	*chromosome 16 open reading frame 87*	*C16orf87*	7.46	7.66	9.11	3.55E-04	0.003194572
7941583	81876		*RAB1B*	12.92	12.96	11.66	3.56E-04	0.003195714
8163509	210	*aminolevulinate dehydratase*	*ALAD*	8.29	8.30	7.30	3.56E-04	0.003197937
8151686	4325	*matrix metallopeptidase 16 (membrane-inserted)*	*MMP16*	6.71	6.87	8.90	3.56E-04	0.003197937
8076417	4700	*NADH dehydrogenase (ubiquinone) 1 alpha subcomplex, 6, 14kDa*	*NDUFA6*	10.77	10.89	11.37	3.56E-04	0.003197937
7948643	5866	*RAB3A interacting protein (rabin3)-like 1*	*RAB3IL1*	8.49	8.52	7.02	3.56E-04	0.003197937

7968004	6445	*sarcoglycan, gamma (35kDa dystrophin-associated glycoprotein)*	*SGCG*	7.70	9.20	5.41	3.56E-04	0.003197937
8076739	55267	*chromosome 22 open reading frame 26*	*C22orf26*	7.54	7.74	6.61	3.56E-04	0.003197937
8072328	23541	*SEC14-like 2 (S. cerevisiae)*	*SEC14L2*	7.58	7.73	6.51	3.58E-04	0.003209341
7961622				7.26	7.36	6.68	3.58E-04	0.003209341
8132943	908	*chaperonin containing TCP1, subunit 6A (zeta 1)*	*CCT6A*	8.51	8.51	10.95	3.59E-04	0.003211977
7964145	8914	*timeless homolog (Drosophila)*	*TIMELESS*	6.88	7.17	9.08	3.59E-04	0.003211977
8154777				5.65	5.54	5.19	3.59E-04	0.003211977
8145532	2053	*epoxide hydrolase 2, cytoplasmic*	*EPHX2*	7.06	5.87	6.50	3.59E-04	0.003213898
8123739	51299	*neuritin 1*	*NRN1*	8.76	11.59	7.32	3.59E-04	0.003215394
8084067	86	*actin-like 6A*	*ACTL6A*	7.62	7.97	9.45	3.59E-04	0.003215589
8022761				4.60	4.62	5.77	3.59E-04	0.003215589
8072488	4733	*developmentally regulated GTP binding protein 1*	*DRG1*	9.76	9.78	10.62	3.60E-04	0.003217715
7952325	3312	*heat shock 70kDa protein 8*	*HSPA8*	13.54	13.64	14.05	3.60E-04	0.003219445
8084323	1857	*dishevelled, dsh homolog 3 (Drosophila)*	*DVL3*	10.75	10.75	9.41	3.61E-04	0.003223722
8034097	147727		*LOC147727*	6.88	6.86	8.21	3.61E-04	0.003223751
7937900	6786	*stromal interaction molecule 1*	*STIM1*	10.04	10.02	7.51	3.61E-04	0.003224745
8021297	284254	*chromosome 18 open reading frame 26*	*C18orf26*	5.15	5.12	6.01	3.61E-04	0.003224745
8151561	79752	*zinc finger, AN1-type domain 1*	*ZFAND1*	8.42	8.30	9.70	3.62E-04	0.003231193
8170390	3423	*iduronate 2-sulfatase*	*IDS*	11.42	11.55	9.83	3.63E-04	0.003240446
7914791	6421	*splicing factor proline/glutamine-rich*	*SFPQ*	9.57	9.56	11.38	3.64E-04	0.00324393
8172876	8243	*structural maintenance of chromosomes 1A*	*SMC1A*	7.34	7.60	9.14	3.64E-04	0.00324393
7921487	8407	*transgelin 2*	*TAGLN2*	11.90	11.97	10.38	3.64E-04	0.00324393
8004360	147040	*potassium channel tetramerisation domain containing 11*	*KCTD11*	9.18	9.62	7.67	3.64E-04	0.00324393
8026787	199786	*family with sequence similarity 129, member C*	*FAM129C*	6.60	6.65	6.03	3.64E-04	0.00324393
8004699	1107	*chromodomain helicase DNA binding protein 3*	*CHD3*	9.65	9.30	7.18	3.65E-04	0.003248083
8005753				8.07	8.09	6.73	3.65E-04	0.00324931

7992887	7627, 100128510	*zinc finger protein 75a | hypothetical protein LOC100128510*	*ZNF75A|LOC100128510*	6.48	6.69	7.47	3.65E-04	0.003253237
7958352	121551	*BTB (POZ) domain containing 11*	*BTBD11*	6.51	6.42	7.56	3.66E-04	0.003255979
8098758	170960, 79963	*zinc finger protein 721 | ATP-binding cassette, sub-family A (ABC1), member 11 (pseudogene)*	*ZNF721|ABCA11P*	7.01	6.69	10.57	3.67E-04	0.003261853
7917728	388650	*family with sequence similarity 69, member A*	*FAM69A*	10.84	10.43	9.22	3.67E-04	0.003264325
8032899	148022	*toll-like receptor adaptor molecule 1*	*TICAM1*	8.95	9.34	7.20	3.67E-04	0.003264676
7898337	55707	*NECAP endocytosis associated 2*	*NECAP2*	11.36	11.44	9.76	3.67E-04	0.003266073
8032909	10226	*perilipin 3*	*PLIN3*	10.67	10.86	9.11	3.68E-04	0.003268983
8169174	79589	*ring finger protein 128*	*RNF128*	5.24	5.28	5.88	3.68E-04	0.003272955
8156982	10592	*structural maintenance of chromosomes 2*	*SMC2*	6.72	6.65	9.23	3.69E-04	0.003274853
7972828	55608	*ankyrin repeat domain 10*	*ANKRD10*	7.89	7.81	9.61	3.69E-04	0.003274853
7951046	4361	*MRE11 meiotic recombination 11 homolog A (S. cerevisiae)*	*MRE11A*	6.37	6.21	8.57	3.70E-04	0.003275101
8067351	5509	*protein phosphatase 1, regulatory (inhibitor) subunit 3D*	*PPP1R3D*	8.85	8.90	8.10	3.70E-04	0.003275101
8090852	51421	*angiomotin like 2*	*AMOTL2*	9.60	9.82	8.14	3.70E-04	0.003275101
8062981	51604	*phosphatidylinositol glycan anchor biosynthesis, class T*	*PIGT*	12.21	12.06	10.81	3.70E-04	0.003275101
7954407	55907	*cytidine monophosphate N-acetylneuraminic acid synthetase*	*CMAS*	7.88	7.82	9.25	3.69E-04	0.003275101
8055941	56475	*reprimo, TP53 dependent G2 arrest mediator candidate*	*RPRM*	6.65	6.80	8.03	3.70E-04	0.003275101
8016628	84687	*protein phosphatase 1, regulatory (inhibitor) subunit 9B*	*PPP1R9B*	8.50	8.39	7.16	3.70E-04	0.003275101
7953775	144568	*alpha-2-macroglobulin-like 1*	*A2ML1*	5.94	5.90	7.17	3.70E-04	0.003275101
8159109	266655	*non-protein coding RNA 94*	*NCRNA00094*	7.48	7.51	6.46	3.69E-04	0.003275101
8078663	407015	*microRNA 26a-1*	*MIR26A1*	5.82	6.05	5.49	3.70E-04	0.003275101
8032972				8.72	8.95	9.99	3.69E-04	0.003275101
8112433				6.49	6.67	6.11	3.69E-04	0.003275101
7945786	4676	*nucleosome assembly protein 1-like 4*	*NAP1L4*	8.38	8.47	9.70	3.70E-04	0.003275391

7931417	282973	*Janus kinase and microtubule interacting protein 3*	*JAKMIP3*	6.62	6.55	6.26	3.71E-04	0.003275391
8002403	92154	*metastasis suppressor 1-like*	*MTSS1L*	11.38	11.43	8.72	3.71E-04	0.003275774
8034696	407018	*microRNA 27a*	*MIR27A*	6.90	7.20	6.37	3.71E-04	0.003275774
7959386	254050	*leucine rich repeat containing 43*	*LRRC43*	6.92	6.77	6.07	3.72E-04	0.003282188
7908766	10440	*translocase of inner mitochondrial membrane 17 homolog A (yeast)*	*TIMM17A*	8.31	8.75	9.31	3.72E-04	0.003284982
7942562	10825	*sialidase 3 (membrane sialidase)*	*NEU3*	8.11	7.83	8.94	3.72E-04	0.003285226
8072577	7533	*tyrosine 3-monooxygenase/tryptophan 5-monooxygenase activation protein, eta polypeptide*	*YWHAH*	10.89	11.44	11.96	3.73E-04	0.00328609
7932082	83643	*coiled-coil domain containing 3*	*CCDC3*	6.30	6.90	7.81	3.73E-04	0.00328609
8000791	83719	*yippee-like 3 (Drosophila)*	*YPEL3*	10.35	10.36	8.62	3.73E-04	0.00328609
8141765				8.07	8.24	7.23	3.73E-04	0.00328609
7995334				8.89	8.95	8.03	3.73E-04	0.003286798
8049435	23677	*SH3-domain binding protein 4*	*SH3BP4*	9.70	10.20	9.04	3.73E-04	0.003287308
8002692	463	*zinc finger homeobox 3*	*ZFHX3*	8.85	8.81	7.27	3.74E-04	0.003289605
7935462	51013	*exosome component 1*	*EXOSC1*	10.46	10.36	11.17	3.74E-04	0.003292558
7937335	8519	*interferon induced transmembrane protein 1 (9-27)*	*IFITM1*	13.01	12.37	12.91	3.74E-04	0.003292955
8178275	282890	*zinc finger protein 311*	*ZNF311*	5.97	5.95	6.52	3.74E-04	0.003292955
8011713	58191	*chemokine (C-X-C motif) ligand 16*	*CXCL16*	8.54	6.97	6.98	3.75E-04	0.003294559
8157905	89853	*family with sequence similarity 125, member B*	*FAM125B*	8.76	8.42	7.40	3.75E-04	0.003294559
8137781	100128653		*LOC100128653*	7.30	7.19	6.02	3.75E-04	0.003294559
8069332	114044, 8888	*MCM3AP antisense RNA (non-protein coding) | minichromosome maintenance complex component 3 associated protein*	*MCM3AP-AS*	6.48	6.36	7.04	3.75E-04	0.003294559
8126135	2739	*glyoxalase I*	*GLO1*	10.25	10.21	11.86	3.75E-04	0.003294853
8113761	57507	*zinc finger protein 608*	*ZNF608*	8.89	7.21	9.67	3.75E-04	0.003294853
8052925	113419	*testis expressed 261*	*TEX261*	11.41	11.24	9.14	3.76E-04	0.003298007
7973056	328	*APEX nuclease (multifunctional DNA repair enzyme) 1*	*APEX1*	12.19	12.17	12.99	3.76E-04	0.003298603
7918345	5686	*proteasome (prosome, macropain) subunit, alpha type, 5*	*PSMA5*	8.53	8.56	9.85	3.76E-04	0.003298603

8125941	6732	*SRSF protein kinase 1*	*SRPK1*	8.53	8.34	11.05	3.76E-04	0.003298603
8149986	55893, 157574	*zinc finger protein 395 | F-box protein 16*	*ZNF395|FBXO16*	9.44	9.73	7.89	3.76E-04	0.003298603
8165021				8.26	8.23	7.51	3.76E-04	0.003298622
8072710	80830	*apolipoprotein L, 6*	*APOL6*	8.71	8.30	5.49	3.77E-04	0.003300825
7922870	81627	*chromosome 1 open reading frame 25*	*C1orf25*	8.39	7.88	9.48	3.78E-04	0.003311893
8162140				7.30	7.37	6.65	3.78E-04	0.003311893
7988350	50506	*dual oxidase 2*	*DUOX2*	6.08	6.12	5.70	3.78E-04	0.003312007
8118076	170679	*psoriasis susceptibility 1 candidate 1*	*PSORS1C1*	6.53	6.01	5.59	3.79E-04	0.003316322
8060103	51281	*ankyrin repeat and MYND domain containing 1*	*ANKMY1*	7.57	7.66	6.96	3.79E-04	0.0033184
8171313	395	*Rho GTPase activating protein 6*	*ARHGAP6*	6.90	6.38	5.71	3.79E-04	0.00331852
8085412	9922	*IQ motif and Sec7 domain 1*	*IQSEC1*	9.66	9.45	8.22	3.80E-04	0.003318958
8062123	10893	*matrix metallopeptidase 24 (membrane-inserted)*	*MMP24*	8.10	8.11	9.65	3.80E-04	0.003318959
8041495	55471	*chromosome 2 open reading frame 56*	*C2orf56*	6.97	7.34	9.54	3.80E-04	0.003320467
8167912	11279	*Kruppel-like factor 8*	*KLF8*	6.63	6.06	7.35	3.80E-04	0.003321173
8040142	51692	*cleavage and polyadenylation specific factor 3, 73kDa*	*CPSF3*	7.74	7.73	10.60	3.80E-04	0.003321173
7906284	149499	*chromosome 1 open reading frame 92*	*C1orf92*	6.55	6.47	6.01	3.81E-04	0.003327054
7981722	3493, 3500	*immunoglobulin heavy constant alpha 1 | immunoglobulin heavy constant gamma 1 (G1m marker)*	*IGHA1|IGHG1*	8.72	8.85	8.19	3.82E-04	0.003336359
8097692	1909	*endothelin receptor type A*	*EDNRA*	9.29	6.66	8.31	3.83E-04	0.00333916
8049670	2817	*glypican 1*	*GPC1*	10.21	10.28	7.97	3.83E-04	0.00333916
7955719	25994	*HIG1 hypoxia inducible domain family, member 1A*	*HIGD1A*	9.14	9.26	10.36	3.83E-04	0.00333916
8138466	346389	*metastasis associated in colon cancer 1*	*MACC1*	5.81	5.53	9.04	3.83E-04	0.00333916
8138440				5.27	5.29	5.04	3.84E-04	0.00334286
7967358	57605	*phosphatidylinositol transfer protein, membrane-associated 2*	*PITPNM2*	7.89	7.86	6.56	3.84E-04	0.003345625
8084838	57110	*HRAS-like suppressor*	*HRASLS*	5.82	5.83	6.64	3.84E-04	0.003346081
8093141	348793	*WD repeat domain 53*	*WDR53*	7.10	7.33	8.51	3.85E-04	0.003356141

8082408	29927	*Sec61 alpha 1 subunit (S. cerevisiae)*	*SEC61A1*	12.73	12.66	11.52	3.86E-04	0.003358922
7897449	80176	*splA/ryanodine receptor domain and SOCS box containing 1*	*SPSB1*	8.92	9.01	7.44	3.86E-04	0.003361647
8030914	162963	*zinc finger protein 610*	*ZNF610*	7.04	6.74	8.06	3.86E-04	0.003361647
8097568	8821, 401157	*inositol polyphosphate-4-phosphatase, type II, 105kDa | FLJ44477 protein*	*INPP4B|FLJ44477*	5.88	5.89	5.36	3.86E-04	0.003361647
8148158	93594	*WD repeat domain 67*	*WDR67*	7.47	7.08	8.70	3.87E-04	0.003367691
7977657	3183	*heterogeneous nuclear ribonucleoprotein C (C1/C2)*	*HNRNPC*	10.98	11.03	12.63	3.88E-04	0.0033695
8148304	10221	*tribbles homolog 1 (Drosophila)*	*TRIB1*	8.36	8.32	10.62	3.88E-04	0.0033695
8003116	83693	*hydroxysteroid dehydrogenase like 1*	*HSDL1*	7.20	7.23	9.12	3.88E-04	0.0033695
8008682	124540	*musashi homolog 2 (Drosophila)*	*MSI2*	6.94	7.26	8.67	3.88E-04	0.0033695
7947332	196294	*IMP1 inner mitochondrial membrane peptidase-like (S. cerevisiae)*	*IMMP1L*	6.20	6.18	7.97	3.88E-04	0.0033695
8131949	11335	*chromobox homolog 3*	*CBX3*	8.82	8.74	9.85	3.88E-04	0.003369609
8003089	8720	*membrane-bound transcription factor peptidase, site 1*	*MBTPS1*	11.15	11.22	9.84	3.89E-04	0.003369951
7933084	10135	*nicotinamide phosphoribosyltransferase*	*NAMPT*	9.69	9.29	11.55	3.88E-04	0.003369951
8104201	11336, 25845	*exocyst complex component 3 | hypothetical LOC25845*	*EXOC3|LOC25845*	9.17	8.86	8.12	3.89E-04	0.003369951
8001784	1006	*cadherin 8, type 2*	*CDH8*	5.90	6.19	7.83	3.89E-04	0.003371265
8115584	79616	*cyclin J-like*	*CCNJL*	8.40	7.99	9.54	3.89E-04	0.003372979
7975787	122953	*Jun dimerization protein 2*	*JDP2*	11.25	11.17	9.56	3.89E-04	0.003372979
8114211				11.40	11.31	10.14	3.90E-04	0.003377104
7954419	55500	*ethanolamine kinase 1*	*ETNK1*	6.86	6.58	8.47	3.91E-04	0.003384223
7927649	55847	*CDGSH iron sulfur domain 1*	*CISD1*	8.56	9.14	9.78	3.91E-04	0.003385526
7924910	58	*actin, alpha 1, skeletal muscle*	*ACTA1*	8.86	9.05	11.45	3.91E-04	0.003385724
8041236	6683	*spastin*	*SPAST*	7.83	7.75	9.53	3.91E-04	0.003386396
7942328	2352	*folate receptor 3 (gamma)*	*FOLR3*	7.29	7.39	6.60	3.92E-04	0.003392353
8138930	441212	*retinitis pigmentosa 9 pseudogene*	*RP9P*	8.13	9.66	6.78	3.93E-04	0.003395031
7940372	79073	*transmembrane protein 109*	*TMEM109*	12.32	12.20	9.99	3.93E-04	0.003395609
8049542	9208	*leucine rich repeat (in FLII) interacting protein 1*	*LRRFIP1*	4.90	4.93	6.41	3.93E-04	0.003396035

7968015	55504	*tumor necrosis factor receptor superfamily, member 19*	*TNFRSF19*	8.86	8.47	6.61	3.93E-04	0.003396035
7978343	1215	*chymase 1, mast cell*	*CMA1*	5.82	5.82	5.36	3.94E-04	0.003401917
8086515				4.62	4.56	4.49	3.94E-04	0.003401917
8138741	3203	*homeobox A6*	*HOXA6*	6.96	7.97	6.57	3.94E-04	0.003403823
7969736	10160	*FERM, RhoGEF (ARHGEF) and pleckstrin domain protein 1 (chondrocyte-derived)*	*FARP1*	11.80	11.90	10.36	3.95E-04	0.003404166
8025968	7620	*zinc finger protein 69*	*ZNF69*	7.15	7.41	8.32	3.95E-04	0.003405134
7982294	161725, 100288637	*OTU domain containing 7A | hypothetical LOC100288637*	*OTUD7A|LOC100288637*	5.66	5.76	6.95	3.95E-04	0.003405134
8070819	754	*pituitary tumor-transforming 1 interacting protein*	*PTTG1IP*	11.76	12.08	10.69	3.95E-04	0.003405912
8178727	1388, 7148	*activating transcription factor 6 beta | tenascin XB*	*ATF6B|TNXB*	11.17	10.89	9.39	3.96E-04	0.003410971
8163892	57000	*chromosome 9 open reading frame 31*	*C9orf31*	6.19	6.23	5.53	3.97E-04	0.00341665
8148276	137209	*zinc finger protein 572*	*ZNF572*	6.12	5.88	7.03	3.97E-04	0.003422329
8138147	81622, 729196	*unc-93 homolog B1 (C. elegans) | unc-93 homolog B5 (C. elegans)*	*UNC93B1|UNC93B5*	8.98	8.94	8.00	3.98E-04	0.00342246
8092836	1370	*carboxypeptidase N, polypeptide 2*	*CPN2*	7.26	7.47	6.46	3.98E-04	0.003422838
8093852	4487	*msh homeobox 1*	*MSX1*	8.53	8.60	7.15	3.98E-04	0.003422838
8017283	57508	*integrator complex subunit 2*	*INTS2*	6.91	6.88	9.24	3.98E-04	0.003422838
7958396	121549	*achaete-scute complex homolog 4 (Drosophila)*	*ASCL4*	6.05	6.14	5.58	3.99E-04	0.00342594
8156058				6.41	6.45	5.50	3.98E-04	0.00342594
8039809	7593	*myeloid zinc finger 1*	*MZF1*	7.72	7.69	7.02	3.99E-04	0.00343197
8069689	11096	*ADAM metallopeptidase with thrombospondin type 1 motif, 5*	*ADAMTS5*	8.74	9.48	6.25	4.00E-04	0.00343197
8059716	151477	*chromosome 2 open reading frame 52*	*C2orf52*	5.82	5.89	6.61	4.00E-04	0.00343197
7899350	199870	*family with sequence similarity 76, member A*	*FAM76A*	7.24	7.52	8.62	4.00E-04	0.00343197
8136918	7791	*zyxin*	*ZYX*	12.16	12.29	10.64	4.00E-04	0.003431988
8082368	50512	*podocalyxin-like 2*	*PODXL2*	7.89	7.94	8.36	4.00E-04	0.003431988
8015242	85291	*keratin associated protein 4-2*	*KRTAP4-2*	8.20	8.49	7.40	4.00E-04	0.003431988

8070655	7307	*U2 small nuclear RNA auxiliary factor 1*	*U2AF1*	10.75	11.01	11.85	4.01E-04	0.003436769
8153359	8581	*lymphocyte antigen 6 complex, locus D*	*LY6D*	8.11	8.15	7.49	4.01E-04	0.003436769
8002194	64174	*dipeptidase 2*	*DPEP2*	6.54	6.57	6.08	4.01E-04	0.003436769
8050689	80304	*chromosome 2 open reading frame 44*	*C2orf44*	7.51	7.59	9.68	4.01E-04	0.003436769
7909214	83593	*Ras association (RalGDS/AF-6) domain family member 5*	*RASSF5*	8.00	7.38	6.54	4.01E-04	0.003436769
8162059	64078	*solute carrier family 28 (sodium-coupled nucleoside transporter), member 3*	*SLC28A3*	6.07	6.14	5.71	4.01E-04	0.00343732
8096938	51574	*La ribonucleoprotein domain family, member 7*	*LARP7*	7.03	7.01	7.85	4.02E-04	0.003443054
8117343	3077	*hemochromatosis*	*HFE*	7.66	7.46	5.91	4.02E-04	0.003443097
8006229	84282	*ring finger protein 135*	*RNF135*	9.21	9.14	7.84	4.03E-04	0.003448881
7947423				8.72	8.78	7.16	4.03E-04	0.00345116
7968234	619499	*small nucleolar RNA, H/ACA box 27*	*SNORA27*	6.21	6.40	7.52	4.04E-04	0.003451875
8064007	54994	*chromosome 20 open reading frame 11*	*C20orf11*	9.56	9.53	10.60	4.04E-04	0.003452712
8085287	55845	*chromosome 3 open reading frame 10*	*C3orf10*	8.39	8.52	7.64	4.04E-04	0.003452712
8054192	129531	*MIT, microtubule interacting and transport, domain containing 1*	*MITD1*	6.16	6.35	7.25	4.04E-04	0.003452712
8156569	407011, 84909	*microRNA 23b | chromosome 9 open reading frame 3*	*MIR23B|C9orf3*	8.40	8.11	7.04	4.05E-04	0.003461034
8135933	346653	*family with sequence similarity 71, member F2*	*FAM71F2*	6.84	6.90	6.22	4.05E-04	0.003461991
7903667	284612	*synaptophysin-like 2*	*SYPL2*	7.14	7.20	6.70	4.06E-04	0.003468287
8086660	29122	*protease, serine, 50*	*PRSS50*	6.66	6.76	6.29	4.07E-04	0.003473413
8009875	80022	*myosin XVB pseudogene*	*MYO15B*	7.11	7.09	6.59	4.07E-04	0.003473413
8012274	84316	*LSM domain containing 1*	*LSMD1*	10.19	10.03	8.66	4.07E-04	0.003473413
8154491	92949	*ADAMTS-like 1*	*ADAMTSL1*	11.04	10.13	6.59	4.07E-04	0.003473413
8020825				5.88	5.88	7.76	4.08E-04	0.003475439
8066279	23051	*zinc fingers and homeoboxes 3*	*ZHX3*	8.55	8.93	6.64	4.08E-04	0.003475497
8165575	375775	*patatin-like phospholipase domain containing 7*	*PNPLA7*	7.33	7.40	6.66	4.08E-04	0.003475497
7955637	3875	*keratin 18*	*KRT18*	8.77	9.11	11.44	4.08E-04	0.003475503
7965541	55785	*FYVE, RhoGEF and PH domain containing 6*	*FGD6*	6.49	7.02	9.62	4.08E-04	0.003475503

8091095				10.65	10.68	10.11	4.08E-04	0.003475565
8081358	27107, 100009676	*zinc finger and BTB domain containing 11 | hypothetical LOC100009676*	*ZBTB11|LOC100009676*	7.96	8.07	7.08	4.09E-04	0.003478187
8012220	6665	*SRY (sex determining region Y)-box 15*	*SOX15*	7.43	7.34	8.70	4.10E-04	0.003484108
7962083				7.40	7.50	6.27	4.10E-04	0.003485442
8113445	64839	*F-box and leucine-rich repeat protein 17*	*FBXL17*	8.51	8.52	7.87	4.10E-04	0.00348599
8177405	9085, 253175, 9426, 203611	*chromodomain protein, Y-linked, 1 | chromodomain protein, Y-linked, 1B | chromodomain protein, Y-linked, 2A | chromodomain protein, Y-linked, 2B*	*CDY1|CDY1B|CDY2A|CDY2B*	5.12	5.20	4.96	4.10E-04	0.00348599
8097116				5.02	4.84	6.40	4.10E-04	0.00348599
8172305				4.97	4.71	5.60	4.10E-04	0.00348599
8147396	55656	*integrator complex subunit 8*	*INTS8*	7.48	7.03	8.76	4.11E-04	0.003492047
8015806	2118	*ets variant 4*	*ETV4*	6.75	7.12	9.48	4.12E-04	0.003496112
7983306	79968	*WD repeat domain 76*	*WDR76*	7.38	7.82	9.69	4.12E-04	0.003498722
8049137	248	*alkaline phosphatase, intestinal*	*ALPI*	7.58	7.61	7.15	4.12E-04	0.003498763
7956395	11247	*neurexophilin 4*	*NXPH4*	9.48	9.23	7.35	4.13E-04	0.003498763
7900999	339541	*chromosome 1 open reading frame 228*	*C1orf228*	7.22	6.71	6.34	4.13E-04	0.003498763
7950391	283209	*phosphoglucomutase 2-like 1*	*PGM2L1*	6.49	6.85	8.98	4.13E-04	0.003500887
7898466	353238	*peptidyl arginine deiminase, type VI*	*PADI6*	6.50	6.36	6.10	4.13E-04	0.003500887
8041826	57217	*tetratricopeptide repeat domain 7A*	*TTC7A*	8.48	8.77	7.42	4.13E-04	0.00350248
7901691	7809	*Bartter syndrome, infantile, with sensorineural deafness (Barttin)*	*BSND*	6.39	6.42	5.94	4.14E-04	0.0035054
7979943	4293	*mitogen-activated protein kinase kinase kinase 9*	*MAP3K9*	6.41	6.58	7.30	4.15E-04	0.003513442
8047262	205327	*chromosome 2 open reading frame 69*	*C2orf69*	7.01	7.25	8.96	4.15E-04	0.003513442
7976852	494326	*microRNA 377*	*MIR377*	5.14	5.01	4.82	4.15E-04	0.003513442
8072659	10043	*target of myb1 (chicken)*	*TOM1*	10.94	10.91	9.40	4.16E-04	0.003513588
7997896	197320	*zinc finger protein 778*	*ZNF778*	7.04	7.09	7.96	4.16E-04	0.003513588
7949021	283248	*REST corepressor 2*	*RCOR2*	7.57	7.29	9.36	4.15E-04	0.003513588

8066407	128486	*fat storage-inducing transmembrane protein 2*	*FITM2*	8.70	8.61	6.87	4.16E-04	0.003515212
8027312	353088	*zinc finger protein 429*	*ZNF429*	6.52	6.04	8.74	4.16E-04	0.003517873
8047635	6136, 645688	*ribosomal protein L12 | ribosomal protein L12 pseudogene 38*	*RPL12|RPL12P38*	12.88	12.84	13.03	4.17E-04	0.003524702
8126450	6152	*ribosomal protein L24*	*RPL24*	11.06	11.25	11.71	4.19E-04	0.003530989
7919669	10903	*myotubularin related protein 11*	*MTMR11*	8.07	7.98	6.78	4.18E-04	0.003530989
8086451	25994	*HIG1 hypoxia inducible domain family, member 1A*	*HIGD1A*	9.02	9.22	10.39	4.18E-04	0.003530989
8069511	391267	*ankyrin repeat domain 20 family, member A3 pseudogene*	*C21orf81*	6.56	6.39	6.04	4.18E-04	0.003530989
8040753	54867	*transmembrane protein 214*	*TMEM214*	12.13	11.99	10.46	4.19E-04	0.003535002
7920971	112770	*chromosome 1 open reading frame 85*	*C1orf85*	11.68	11.19	10.33	4.20E-04	0.003539536
7974316	122786	*FERM domain containing 6*	*FRMD6*	10.03	10.32	7.89	4.20E-04	0.003541773
8073562	164684, 4668	*WBP2 N-terminal like | N-acetylgalactosaminidase, alpha-*	*WBP2NL|NAGA*	5.80	5.68	5.34	4.21E-04	0.003543431
8015268	3885	*keratin 34*	*KRT34*	6.59	8.63	5.64	4.21E-04	0.003544548
8126269	340205	*triggering receptor expressed on myeloid cells-like 1*	*TREML1*	6.66	6.65	6.25	4.21E-04	0.003546963
8118455	720, 721, 100509001	*complement component 4A (Rodgers blood group) | complement component 4B (Chido blood group) | complement C4-B-like*	*C4A|C4B|LOC100509001*	8.39	7.39	6.20	4.22E-04	0.003549874
7973850	9472	*A kinase (PRKA) anchor protein 6*	*AKAP6*	6.59	7.84	6.02	4.22E-04	0.003550684
7978628	55012	*protein phosphatase 2, regulatory subunit B'', gamma*	*PPP2R3C*	8.02	8.19	8.87	4.22E-04	0.003550684
7962487	83956	*Rac GTPase activating protein 1 pseudogene*	*RACGAP1P*	5.72	5.89	6.86	4.22E-04	0.003550684
7948088	84275	*solute carrier family 25, member 33*	*SLC25A33*	7.04	7.33	8.76	4.22E-04	0.003550684
8022338	166647, 85455	*G protein-coupled receptor 125 | dispatched homolog 2 (Drosophila)*	*GPR125|DISP2*	7.42	7.03	10.40	4.22E-04	0.003550684
8176076	1774, 6134	*deoxyribonuclease I-like 1 | ribosomal protein L10*	*DNASE1L1|RPL10*	9.30	8.10	7.04	4.23E-04	0.003552235
8067279	1522	*cathepsin Z*	*CTSZ*	11.10	11.35	8.89	4.23E-04	0.003552601
7970831	5412	*ubiquitin-like 3*	*UBL3*	8.02	8.93	7.72	4.23E-04	0.003553529
8054872	29842	*transcription factor CP2-like 1*	*TFCP2L1*	6.54	6.59	7.80	4.23E-04	0.003554057
7981708	3497	*immunoglobulin heavy constant epsilon*	*IGHE*	7.24	7.27	6.25	4.24E-04	0.003559961

8113641	1036	*cysteine dioxygenase, type I*	*CDO1*	6.51	6.56	8.76	4.24E-04	0.003561073
7934196	5660	*prosaposin*	*PSAP*	12.73	12.67	11.76	4.25E-04	0.003562226
8174076	2717	*galactosidase, alpha*	*GLA*	8.24	8.20	9.81	4.25E-04	0.003562778
7930120	51684	*suppressor of fused homolog (Drosophila)*	*SUFU*	8.63	8.33	7.48	4.25E-04	0.003563326
8060505	57593	*early B-cell factor 4*	*EBF4*	7.99	7.99	6.89	4.25E-04	0.003563326
8179399	720, 721, 8859, 100509001	*complement component 4A (Rodgers blood group) | complement component 4B (Chido blood group) | serine/threonine kinase 19 | complement C4-B-like*	*C4A|C4B|STK19|LOC 100509001*	8.37	7.38	6.20	4.25E-04	0.003563326
8114145	7416	*voltage-dependent anion channel 1*	*VDAC1*	9.14	9.40	9.64	4.26E-04	0.003564667
8140061	9275	*B-cell CLL/lymphoma 7B*	*BCL7B*	10.57	10.94	9.22	4.26E-04	0.003564667
7981326	100302145, 64150	*microRNA 1247 | DIO3 opposite strand (non-protein coding)*	*MIR1247| DIO3-OS*	7.48	7.41	6.75	4.26E-04	0.003564667
7901363	1031	*cyclin-dependent kinase inhibitor 2C (p18, inhibits CDK4)*	*CDKN2C*	6.44	6.38	5.52	4.27E-04	0.003570795
8180111	6257	*retinoid X receptor, beta*	*RXRB*	9.99	9.97	8.45	4.27E-04	0.003570795
8010405	284129	*solute carrier family 26, member 11*	*SLC26A11*	9.01	8.93	7.90	4.27E-04	0.003572542
7964646	57460	*protein phosphatase, Mg2+/Mn2+ dependent, 1H*	*PPM1H*	7.08	7.10	8.40	4.28E-04	0.003576542
8107470	5757, 150928	*prothymosin, alpha | prothymosin, alpha pseudogene 5*	*PTMA|PTMAP5*	13.17	13.21	13.87	4.28E-04	0.003576872
7901272	260293	*cytochrome P450, family 4, subfamily X, polypeptide 1*	*CYP4X1*	5.79	5.80	6.98	4.29E-04	0.003584006
8159670	10811, 377841	*NADPH oxidase activator 1 | ectonucleoside triphosphate diphosphohydrolase 8*	*NOXA1|ENTPD8*	8.40	8.40	7.70	4.29E-04	0.003584006
7923635	22874	*pleckstrin homology domain containing, family A member 6*	*PLEKHA6*	9.50	7.99	7.95	4.30E-04	0.003591656
8118409	720, 721, 8859, 100509001	*complement component 4A (Rodgers blood group) | complement component 4B (Chido blood group) | serine/threonine kinase 19 | complement C4-B-like*	*C4A|C4B|STK19|LOC 100509001*	8.39	7.38	6.20	4.30E-04	0.003591656
7999608	54700		*RRN3*	7.92	8.10	9.53	4.30E-04	0.003591828
8000738	253980	*potassium channel tetramerisation domain containing 13*	*KCTD13*	9.06	9.25	7.80	4.30E-04	0.003591828

8015798	100130581		*LOC100130581*	7.82	7.68	6.49	4.31E-04	0.003595531
7898653	163933	*family with sequence similarity 43, member B*	*FAM43B*	9.18	9.25	8.49	4.31E-04	0.003596171
7918759	10286, 163259	*breast carcinoma amplified sequence 2 | DENN/MADD domain containing 2C*	*BCAS2|DENND2C*	8.63	8.77	10.39	4.31E-04	0.003596429
7991742	4350	*N-methylpurine-DNA glycosylase*	*MPG*	8.77	8.72	7.95	4.32E-04	0.003597595
8100328	64854	*ubiquitin specific peptidase 46*	*USP46*	8.38	7.96	10.05	4.32E-04	0.00359773
8027819	57817	*hepcidin antimicrobial peptide*	*HAMP*	7.73	7.65	7.03	4.33E-04	0.003611073
7901592	127428	*chromosome 1 open reading frame 83*	*C1orf83*	7.27	7.43	8.27	4.34E-04	0.00361141
7937936				6.62	6.64	6.20	4.34E-04	0.00361141
8057620	1290	*collagen, type V, alpha 2*	*COL5A2*	9.13	9.25	6.47	4.34E-04	0.003614953
7931187	9184	*budding uninhibited by benzimidazoles 3 homolog (yeast)*	*BUB3*	8.83	8.83	10.63	4.35E-04	0.003617122
8109677	2566	*gamma-aminobutyric acid (GABA) A receptor, gamma 2*	*GABRG2*	5.31	5.34	6.70	4.35E-04	0.003617973
7920472	7170	*tropomyosin 3*	*TPM3*	6.40	6.47	7.04	4.35E-04	0.003617973
8052994	26056	*RAB11 family interacting protein 5 (class I)*	*RAB11FIP5*	9.52	9.82	7.74	4.35E-04	0.003617973
7902891	284695	*zinc finger protein 326*	*ZNF326*	6.85	6.83	8.66	4.35E-04	0.003617973
8031778	348327	*zinc finger protein 530*	*ZNF530*	6.26	6.26	6.83	4.35E-04	0.003618802
8038393	406942	*microRNA 150*	*MIR150*	7.73	7.58	6.89	4.36E-04	0.003622765
8079964				8.65	8.67	7.37	4.36E-04	0.003624415
8130470				8.34	8.30	7.44	4.37E-04	0.003624415
7973384	8106	*poly(A) binding protein, nuclear 1*	*PABPN1*	11.40	11.23	12.30	4.37E-04	0.003630491
8105504	257415, 728066, 728153	*family with sequence similarity 133, member B | family with sequence similarity 133, member B pseudogene | similar to FAM133B protein*	*FAM133B|LOC728066|LOC728153*	7.30	7.21	9.03	4.38E-04	0.003630898
8168373				6.82	6.99	6.33	4.38E-04	0.003632048
8063177	81031	*solute carrier family 2 (facilitated glucose transporter), member 10*	*SLC2A10*	10.72	10.60	8.30	4.39E-04	0.003637349
8053036	51002	*TP53RK binding protein*	*TPRKB*	6.41	6.96	7.96	4.39E-04	0.003637704

7904737	284615	*ankyrin repeat domain 34A*	*ANKRD34A*	7.33	7.27	6.42	4.39E-04	0.00363816
8107326	654322	*small nucleolar RNA, H/ACA box 13*	*SNORA13*	6.38	6.55	7.61	4.39E-04	0.003640078
8152133	50484	*ribonucleotide reductase M2 B (TP53 inducible)*	*RRM2B*	8.42	8.11	10.34	4.41E-04	0.00365021
8142997	91584	*plexin A4*	*PLXNA4*	6.84	7.25	6.34	4.41E-04	0.003653423
8046815	91752	*zinc finger protein 804A*	*ZNF804A*	6.00	5.77	5.43	4.43E-04	0.003670041
8128247	60468	*BTB and CNC homology 1, basic leucine zipper transcription factor 2*	*BACH2*	6.15	6.30	6.94	4.44E-04	0.003674599
8122045	9465	*A kinase (PRKA) anchor protein 7*	*AKAP7*	5.90	6.07	7.19	4.45E-04	0.003679781
7917976	163786	*spindle assembly 6 homolog (C. elegans)*	*SASS6*	7.16	7.16	9.71	4.45E-04	0.003679781
7966839	54621	*V-set and immunoglobulin domain containing 10*	*VSIG10*	7.71	7.71	9.02	4.45E-04	0.003680285
8022404	125228	*chromosome 18 open reading frame 19*	*C18orf19*	7.53	8.25	8.98	4.45E-04	0.003680285
8175438				7.72	7.83	6.92	4.45E-04	0.003680285
7956826	23329	*TBC1 domain family, member 30*	*TBC1D30*	5.71	5.77	6.69	4.46E-04	0.003680304
7934101	84883	*apoptosis-inducing factor, mitochondrion-associated, 2*	*AIFM2*	7.99	8.12	6.67	4.46E-04	0.003680304
7899043	57190	*selenoprotein N, 1*	*SEPN1*	11.77	11.62	9.82	4.46E-04	0.003681407
8112435				8.05	8.01	7.37	4.46E-04	0.003681407
8146649	9650	*mitochondrial fission regulator 1*	*MTFR1*	8.28	8.70	9.87	4.46E-04	0.003681905
8128043	1268	*cannabinoid receptor 1 (brain)*	*CNR1*	5.36	5.40	5.91	4.46E-04	0.003682393
8003857				8.78	9.01	7.51	4.47E-04	0.003684996
8164426				6.56	6.64	6.12	4.47E-04	0.003685203
8124622	282890	*zinc finger protein 311*	*ZNF311*	5.98	5.95	6.51	4.48E-04	0.003696244
8035351	3718	*Janus kinase 3*	*JAK3*	7.29	6.86	6.31	4.49E-04	0.003697222
8153346	66004	*Ly6/neurotoxin 1*	*LYNX1*	9.78	9.63	8.46	4.49E-04	0.003699508
7927681	80114	*bicaudal C homolog 1 (Drosophila)*	*BICC1*	10.24	9.84	6.03	4.49E-04	0.003699948
8030899	90321	*zinc finger protein 766*	*ZNF766*	7.23	7.18	9.46	4.49E-04	0.003700348
8085581	8292	*collagen-like tail subunit (single strand of homotrimer) of asymmetric acetylcholinesterase*	*COLQ*	7.15	7.24	6.62	4.50E-04	0.003705999
7978754	10944	*chromosome 11 open reading frame 58*	*C11orf58*	10.78	10.73	11.90	4.51E-04	0.003707375
8131155	23288	*IQ motif containing E*	*IQCE*	9.22	9.06	7.78	4.51E-04	0.003707375

8151384	27067	*staufen, RNA binding protein, homolog 2 (Drosophila)*	*STAU2*	8.02	7.63	9.00	4.51E-04	0.003709901
7972157	1910	*endothelin receptor type B*	*EDNRB*	9.70	5.60	9.60	4.52E-04	0.003713527
8125638	6257	*retinoid X receptor, beta*	*RXRB*	10.00	9.99	8.44	4.52E-04	0.003717608
7930226	22984	*programmed cell death 11*	*PDCD11*	8.24	8.39	9.65	4.53E-04	0.003718746
8080983				5.00	4.87	4.68	4.53E-04	0.003722679
8143154	9162	*diacylglycerol kinase, iota*	*DGKI*	6.52	6.74	6.09	4.53E-04	0.003723863
7961320	653247, 5542	*proline-rich protein BstNI subfamily 2 | proline-rich protein BstNI subfamily 1*	*PRB2|PRB 1*	7.47	7.57	7.04	4.54E-04	0.003725149
8002706	388289	*chromosome 16 open reading frame 47*	*C16orf47*	6.44	6.55	6.01	4.54E-04	0.00372833
8162667				6.56	6.57	8.19	4.54E-04	0.003729137
8099887	6133	*ribosomal protein L9*	*RPL9*	11.98	12.07	12.43	4.55E-04	0.003729231
8014749	9349, 619505	*ribosomal protein L23 | small nucleolar RNA, H/ACA box 21*	*RPL23|SN ORA21*	7.74	7.87	9.33	4.55E-04	0.003729231
8001587	3801	*kinesin family member C3*	*KIFC3*	8.63	8.86	7.53	4.55E-04	0.003730119
8003357	9780	*family with sequence similarity 38, member A*	*FAM38A*	10.32	10.64	8.20	4.55E-04	0.003730119
8137942	84629	*trinucleotide repeat containing 18*	*TNRC18*	10.00	9.89	8.16	4.55E-04	0.003730119
7913776	163702	*interleukin 28 receptor, alpha (interferon, lambda receptor)*	*IL28RA*	6.59	6.66	7.34	4.55E-04	0.003730119
8170992	677835	*small nucleolar RNA, H/ACA box 56*	*SNORA56*	6.09	6.24	8.51	4.55E-04	0.003730119
8163525	54107	*polymerase (DNA directed), epsilon 3 (p17 subunit)*	*POLE3*	8.25	8.62	9.28	4.56E-04	0.003730738
8164131	286205	*suppressor of cancer cell invasion*	*SCAI*	7.94	7.16	9.27	4.56E-04	0.003734851
7927854	3189	*heterogeneous nuclear ribonucleoprotein H3 (2H9)*	*HNRNPH3*	7.12	7.10	9.71	4.57E-04	0.00373498
7901804	10207	*InaD-like (Drosophila)*	*INADL*	7.27	6.79	9.51	4.57E-04	0.00373498
8105302	10468	*follistatin*	*FST*	11.61	10.61	8.71	4.57E-04	0.00373498
7948144	219487	*olfactory receptor, family 5, subfamily M, member 11*	*OR5M11*	5.42	5.54	5.03	4.57E-04	0.00373498
8137707	442907	*microRNA 339*	*MIR339*	9.35	9.40	8.58	4.57E-04	0.00373498
8088020	3700, 389125	*inter-alpha (globulin) inhibitor H4 (plasma Kallikrein-sensitive glycoprotein) | musculoskeletal, embryonic nuclear protein 1*	*ITIH4|MU STN1*	7.24	7.09	6.42	4.57E-04	0.00373498

8164554	389792, 4247	*immediate early response 5-like | mannosyl (alpha-1,6-)-glycoprotein beta-1,2-N-acetylglucosaminyltransferase*	*IER5L|MGAT2*	8.96	9.19	7.94	4.57E-04	0.00373498
8145622				6.93	6.99	8.11	4.57E-04	0.00373498
7967586				7.29	7.33	6.91	4.58E-04	0.003736663
8019046	9775	*eukaryotic translation initiation factor 4A3*	*EIF4A3*	10.67	10.58	11.78	4.58E-04	0.003740751
8071466	8216	*leucine-zipper-like transcription regulator 1*	*LZTR1*	9.30	9.27	7.82	4.58E-04	0.003740942
7973865				6.75	6.77	6.29	4.59E-04	0.003742626
8112807	411	*arylsulfatase B*	*ARSB*	9.81	10.20	8.48	4.59E-04	0.003745243
8052581				6.33	7.40	5.15	4.59E-04	0.003745243
8060675	994	*cell division cycle 25 homolog B (S. pombe)*	*CDC25B*	10.59	9.11	7.61	4.60E-04	0.003747921
7932420	9200	*protein tyrosine phosphatase-like (proline instead of catalytic arginine), member A*	*PTPLA*	7.73	8.24	8.52	4.60E-04	0.003747921
7968734	10166	*solute carrier family 25 (mitochondrial carrier; ornithine transporter) member 15*	*SLC25A15*	7.28	7.72	9.06	4.60E-04	0.003747921
8021376	23327	*neural precursor cell expressed, developmentally down-regulated 4-like*	*NEDD4L*	6.94	6.71	10.32	4.61E-04	0.003749945
7986442				6.98	7.11	6.22	4.61E-04	0.003749945
8012000	440400	*ribonuclease, RNase K*	*RNASEK*	9.54	9.66	7.86	4.63E-04	0.003765756
7985690	440297, 440300	*chondroitin sulfate proteoglycan 4 pseudogene*	*LOC440297|LOC440300*	7.86	7.82	6.77	4.63E-04	0.003765961
8099326	56606	*solute carrier family 2 (facilitated glucose transporter), member 9*	*SLC2A9*	7.90	7.22	5.81	4.63E-04	0.003766814
8076339	84844	*PHD finger protein 5A*	*PHF5A*	8.30	8.47	9.71	4.63E-04	0.003766814
8067157	140689	*cerebellin 4 precursor*	*CBLN4*	5.94	5.92	5.60	4.64E-04	0.003767788
7964736	8091, 100129940	*high mobility group AT-hook 2 | hypothetical LOC100129940*	*HMGA2|LOC100129940*	5.94	6.03	5.60	4.64E-04	0.003767788
8049952	285093, 100131763	*chromosome 2 open reading frame 85 | hypothetical LOC100131763*	*C2orf85|LOC100131763*	7.52	7.58	6.97	4.65E-04	0.003774427
8050507	57539	*WD repeat domain 35*	*WDR35*	6.41	5.97	8.36	4.65E-04	0.003774884

8020323	8731	*RNA (guanine-7-) methyltransferase*	*RNMT*	6.84	6.75	8.50	4.65E-04	0.003777451
8178903	6257	*retinoid X receptor, beta*	*RXRB*	9.99	9.97	8.45	4.66E-04	0.003783431
7956488	3798	*kinesin family member 5A*	*KIF5A*	6.70	6.78	8.65	4.66E-04	0.003784705
8082886	5096	*propionyl CoA carboxylase, beta polypeptide*	*PCCB*	9.20	9.11	10.47	4.67E-04	0.003785569
8051963	55133	*S1 RNA binding domain 1*	*SRBD1*	7.63	7.48	9.38	4.67E-04	0.003787976
8030339	2323	*fms-related tyrosine kinase 3 ligand*	*FLT3LG*	10.43	10.22	8.17	4.67E-04	0.003788149
7909890	3142	*H2.0-like homeobox*	*HLX*	8.91	9.39	7.32	4.67E-04	0.003788149
8110158	285598	*ADP-ribosylation factor-like 10*	*ARL10*	7.73	7.88	7.13	4.67E-04	0.003788149
8149151	349196		*LOC349196*	9.73	8.68	7.13	4.68E-04	0.003790447
8061402				6.16	6.20	5.61	4.69E-04	0.003797271
8040070	6664	*SRY (sex determining region Y)-box 11*	*SOX11*	7.34	7.45	8.48	4.70E-04	0.003804409
8149214	349196		*LOC349196*	9.73	8.68	7.13	4.70E-04	0.003805058
7950030				5.43	5.52	8.11	4.71E-04	0.00381224
8125295	1388, 7148	*activating transcription factor 6 beta | tenascin XB*	*ATF6B|TNXB*	11.20	10.95	9.43	4.71E-04	0.003812497
8079217	7584	*zinc finger protein 35*	*ZNF35*	6.80	6.92	8.40	4.72E-04	0.003814816
8081546				5.84	6.10	7.81	4.72E-04	0.003815926
8139887				7.62	7.68	7.15	4.72E-04	0.003818353
8038126	770	*carbonic anhydrase XI*	*CA11*	7.90	7.68	9.56	4.73E-04	0.003826239
8066925	5740	*prostaglandin I2 (prostacyclin) synthase*	*PTGIS*	13.03	11.96	9.80	4.74E-04	0.003826794
7947681	392	*Rho GTPase activating protein 1*	*ARHGAP1*	12.30	12.43	10.94	4.74E-04	0.003829614
8090098	4638	*myosin light chain kinase*	*MYLK*	9.84	10.03	7.39	4.74E-04	0.003829614
7947828	4607	*myosin binding protein C, cardiac*	*MYBPC3*	7.57	7.59	7.13	4.74E-04	0.003830363
8148572	4061	*lymphocyte antigen 6 complex, locus E*	*LY6E*	12.51	11.73	9.98	4.75E-04	0.003832651
8139632	63979	*fidgetin-like 1*	*FIGNL1*	5.99	6.01	7.19	4.75E-04	0.003832651
8080960				7.18	7.18	6.44	4.75E-04	0.003833656
7977397	1397	*cysteine-rich protein 2*	*CRIP2*	10.98	11.14	8.06	4.76E-04	0.003837188
8077989	348825	*tetra-peptide repeat homeobox-like*	*TPRXL*	7.75	7.70	7.25	4.76E-04	0.003837188

7907492	9910	*RAB GTPase activating protein 1-like*	*RABGAP1L*	6.33	6.25	8.18	4.76E-04	0.003838249
7950271	23201	*family with sequence similarity 168, member A*	*FAM168A*	10.15	10.03	8.92	4.77E-04	0.003838249
8106210	64283	*190 kDa guanine nucleotide exchange factor*	*RGNEF*	7.48	6.70	6.25	4.76E-04	0.003838249
8136954	285966	*family with sequence similarity 115, member C*	*FAM115C*	8.34	8.74	6.97	4.78E-04	0.003846986
8071392	51586	*mediator complex subunit 15*	*MED15*	11.65	11.74	10.34	4.78E-04	0.003849397
8021768	10194	*teashirt zinc finger homeobox 1*	*TSHZ1*	8.47	8.43	6.38	4.79E-04	0.003851884
7911413	7293	*tumor necrosis factor receptor superfamily, member 4*	*TNFRSF4*	7.99	8.04	7.19	4.79E-04	0.003856263
8015575	84514	*GH3 domain containing*	*GHDC*	8.46	8.18	7.12	4.79E-04	0.003856636
8035779	56242, 199777, 81931	*zinc finger protein 253 | zinc finger protein 626 | zinc finger protein 93*	*ZNF253|ZNF626|ZNF93*	10.06	10.03	12.50	4.80E-04	0.003860159
7931832	1646	*aldo-keto reductase family 1, member C2 (dihydrodiol dehydrogenase 2; bile acid binding protein; 3-alpha hydroxysteroid dehydrogenase, type III)*	*AKR1C2*	9.42	9.02	5.93	4.81E-04	0.00386833
7948565	220002	*cytochrome b, ascorbate dependent 3*	*CYBASC3*	10.52	10.03	8.08	4.81E-04	0.003868427
7942603	80168	*monoacylglycerol O-acyltransferase 2*	*MOGAT2*	6.17	6.34	5.67	4.82E-04	0.003873102
8156295	79048	*SECIS binding protein 2*	*SECISBP2*	8.22	8.35	10.57	4.82E-04	0.003875096
7903227	54873	*palmdelphin*	*PALMD*	5.89	5.72	5.45	4.83E-04	0.003878831
7971922	5101	*protocadherin 9*	*PCDH9*	6.34	5.62	5.35	4.83E-04	0.003878885
7945579	66005	*chitinase domain containing 1*	*CHID1*	10.77	10.78	9.32	4.84E-04	0.003881442
7938738	3746	*potassium voltage-gated channel, Shaw-related subfamily, member 1*	*KCNC1*	8.03	8.10	7.40	4.84E-04	0.003885029
7950374	283208	*prolyl 4-hydroxylase, alpha polypeptide III*	*P4HA3*	7.49	8.47	6.42	4.86E-04	0.003896429
7906085	4000	*lamin A/C*	*LMNA*	10.92	10.98	8.09	4.86E-04	0.003900843
7957260	11010, 11103	*GLI pathogenesis-related 1 | KRR1, small subunit (SSU) processome component, homolog (yeast)*	*GLIPR1|KRR1*	11.91	12.13	7.98	4.87E-04	0.003902458
8081235	1295	*collagen, type VIII, alpha 1*	*COL8A1*	7.03	7.85	5.61	4.88E-04	0.003903571
8100827	3512	*immunoglobulin J polypeptide, linker protein for immunoglobulin alpha and mu polypeptides*	*IGJ*	6.07	6.74	5.43	4.88E-04	0.003903571
8051949	10736	*SIX homeobox 2*	*SIX2*	10.03	9.19	8.24	4.87E-04	0.003903571
7914467	90853	*SPOC domain containing 1*	*SPOCD1*	8.67	9.54	6.97	4.88E-04	0.003903571

8173503	340526	*retrotransposon gag domain containing 4*	*RGAG4*	8.03	8.10	6.40	4.87E-04	0.003903571
7930857	2018	*empty spiracles homeobox 2*	*EMX2*	8.74	9.44	6.67	4.88E-04	0.0039053
7974047	6751	*somatostatin receptor 1*	*SSTR1*	7.03	7.95	6.59	4.88E-04	0.0039053
8169249	11043	*midline 2*	*MID2*	8.98	8.89	6.71	4.88E-04	0.0039053
8047228	25843	*MOB1, Mps One Binder kinase activator-like 3 (yeast)*	*MOBKL3*	8.79	8.56	10.44	4.88E-04	0.0039053
8071339	29801	*zinc finger, DHHC-type containing 8*	*ZDHHC8*	9.19	9.21	7.59	4.89E-04	0.0039053
8036494	199720	*gametogenetin*	*GGN*	7.81	7.85	7.06	4.89E-04	0.0039053
8144490	349196		*LOC349196*	9.79	8.73	7.10	4.88E-04	0.0039053
8101366	79966	*stearoyl-CoA desaturase 5*	*SCD5*	8.59	8.70	7.02	4.89E-04	0.003909208
8017437	117246	*FtsJ homolog 3 (E. coli)*	*FTSJ3*	7.97	7.95	9.15	4.90E-04	0.003909701
8144492	349196		*LOC349196*	9.79	8.74	7.10	4.90E-04	0.003910321
8144418	349196		*LOC349196*	9.79	8.74	7.10	4.90E-04	0.003913303
8108708	56129	*protocadherin beta 7*	*PCDHB7*	6.78	7.04	6.04	4.91E-04	0.003916726
8079019	6232	*ribosomal protein S27*	*RPS27*	12.93	12.96	12.70	4.91E-04	0.003917929
8100231	7006	*tec protein tyrosine kinase*	*TEC*	6.12	6.11	7.26	4.91E-04	0.003917929
8144416	349196		*LOC349196*	9.79	8.74	7.10	4.92E-04	0.003921761
8114263	3950	*leukocyte cell-derived chemotaxin 2*	*LECT2*	5.74	5.77	5.47	4.92E-04	0.003922899
7998983	197358	*NLR family, CARD domain containing 3*	*NLRC3*	6.69	6.77	6.49	4.92E-04	0.003922899
8022557				4.62	4.55	4.93	4.92E-04	0.003922899
7949645	254359	*zinc finger, DHHC-type containing 24*	*ZDHHC24*	9.68	9.79	8.45	4.93E-04	0.003930121
7898405				6.75	6.21	8.25	4.94E-04	0.003933519
7923442	127833	*synaptotagmin II*	*SYT2*	6.57	6.72	8.29	4.95E-04	0.003936212
8147481				7.68	7.73	7.00	4.95E-04	0.003938108
8053187	84759	*polycomb group ring finger 1*	*PCGF1*	8.52	8.71	7.64	4.96E-04	0.003943901
7956470	114785	*methyl-CpG binding domain protein 6*	*MBD6*	9.62	9.53	7.78	4.96E-04	0.003943901
8055862	26225	*ADP-ribosylation factor-like 5A*	*ARL5A*	9.11	9.23	10.36	4.96E-04	0.003946682

8149153	349196		*LOC349196*	9.73	8.66	7.07	4.97E-04	0.003950276
7917912	1806	*dihydropyrimidine dehydrogenase*	*DPYD*	9.82	9.63	6.53	4.97E-04	0.003950592
7915827	10489	*leucine rich repeat containing 41*	*LRRC41*	11.43	11.37	10.04	4.98E-04	0.003952802
8177410	114758, 84664, 114817	*chondroitin sulfate proteoglycan 4 pseudogene 1, Y-linked | chondroitin sulfate proteoglycan 4 pseudogene 2, Y-linked | chondroitin sulfate proteoglycan 4 pseudogene 5*	*CSPG4P1Y|CSPG4P2Y|CSPG4P5*	7.55	7.64	6.56	4.98E-04	0.003952919
7928645	728118, 729262, 283008, 728130	*family with sequence similarity 22, member A | family with sequence similarity 22, member B | family with sequence similarity 22, member E | family with sequence similarity 22, member D*	*FAM22A|FAM22B|FAM22E|FAM22D*	7.60	7.73	7.03	4.99E-04	0.003961228
7984470	10116	*fem-1 homolog b (C. elegans)*	*FEM1B*	9.45	9.10	10.82	4.99E-04	0.003961691
7945321	89944	*galactosidase, beta 1-like 2*	*GLB1L2*	6.17	6.31	7.81	4.99E-04	0.003961691
8077082	91289	*lipase maturation factor 2*	*LMF2*	10.12	10.38	7.87	4.99E-04	0.003961691
8019576	85294, 81872, 728279, 730755	*keratin associated protein 2-4 | keratin associated protein 2-1 | keratin associated protein 2-2 | keratin associated protein 2-4-like*	*KRTAP2-4|KRTAP2-1|KRTAP2-2|LOC730755*	8.83	10.38	7.67	5.00E-04	0.003961691
7929322	1592	*cytochrome P450, family 26, subfamily A, polypeptide 1*	*CYP26A1*	6.31	6.35	8.45	5.00E-04	0.003965715
8147883	56943	*enhancer of yellow 2 homolog (Drosophila)*	*ENY2*	8.71	8.94	10.05	5.01E-04	0.003974906
7990898	6133	*ribosomal protein L9*	*RPL9*	12.02	12.11	12.47	5.02E-04	0.00397528
8001938	8717	*TNFRSF1A-associated via death domain*	*TRADD*	9.44	9.37	7.82	5.02E-04	0.00397528
8093792	3083	*HGF activator*	*HGFAC*	7.67	7.71	6.88	5.02E-04	0.003976486
8061075	6629	*small nuclear ribonucleoprotein polypeptide B*	*SNRPB2*	9.60	9.76	10.68	5.03E-04	0.003977941
8050766	109, 79172	*adenylate cyclase 3 | centromere protein O*	*ADCY3|CENPO*	11.07	10.09	8.36	5.02E-04	0.003977941

8015210	85294, 81872, 728279, 730755	*keratin associated protein 2-4 | keratin associated protein 2-1 | keratin associated protein 2-2 | keratin associated protein 2-4-like*	*KRTAP2-4|KRTAP2-1|KRTAP2-2|LOC730755*	8.83	10.38	7.67	5.03E-04	0.003980879
7960559	25900	*intermediate filament family orphan 1*	*IFFO1*	8.31	7.94	7.11	5.04E-04	0.003983941
8138454	221830	*TWIST neighbor*	*TWISTNB*	6.98	7.19	8.58	5.05E-04	0.003992976
8135015	4584, 57876, 100509994	*mucin 3A, cell surface associated | mucin 3B, cell surface associated | hypothetical LOC100509994*	*MUC3A|MUC3B|LOC100509994*	6.54	6.66	7.72	5.05E-04	0.003992976
8004024	2811	*glycoprotein Ib (platelet), alpha polypeptide*	*GP1BA*	6.46	6.50	5.99	5.06E-04	0.003993936
8163019	10880	*actin-like 7B*	*ACTL7B*	7.34	7.34	6.86	5.05E-04	0.003993936
7975799	55640	*feline leukemia virus subgroup C cellular receptor family, member 2*	*FLVCR2*	6.88	6.81	7.99	5.06E-04	0.003993936
7951004	56935	*chromosome 11 open reading frame 75*	*C11orf75*	7.79	8.40	9.17	5.06E-04	0.003993936
8112582				5.66	5.44	6.83	5.05E-04	0.003993936
7907160	481	*ATPase, Na+/K+ transporting, beta 1 polypeptide*	*ATP1B1*	7.27	8.35	9.18	5.06E-04	0.003994392
7976037	7253	*thyroid stimulating hormone receptor*	*TSHR*	5.59	5.63	5.22	5.06E-04	0.003994392
8173825	27330	*ribosomal protein S6 kinase, 90kDa, polypeptide 6*	*RPS6KA6*	6.97	6.28	8.91	5.06E-04	0.003994392
8004167	54478	*family with sequence similarity 64, member A*	*FAM64A*	8.13	8.50	11.08	5.06E-04	0.003994392
8149157	349196		*LOC349196*	9.73	8.66	7.07	5.07E-04	0.003994392
8154512	92949	*ADAMTS-like 1*	*ADAMTSL1*	10.11	9.18	6.28	5.07E-04	0.003994884
7948115	338674	*olfactory receptor, family 5, subfamily F, member 1*	*OR5F1*	6.39	6.30	5.83	5.07E-04	0.003994884
8056041				9.19	9.32	8.62	5.07E-04	0.003994884
8109773	23286	*WW and C2 domain containing 1*	*WWC1*	7.25	7.26	8.36	5.08E-04	0.003996841
8030251	54795	*transient receptor potential cation channel, subfamily M, member 4*	*TRPM4*	8.88	8.76	7.43	5.08E-04	0.003996841
8133662	57414	*rhomboid domain containing 2*	*RHBDD2*	12.44	12.44	10.82	5.07E-04	0.003996841

8173156				10.50	10.42	9.53	5.08E-04	0.003996841
8002143	3931, 6560	*lecithin-cholesterol acyltransferase | solute carrier family 12 (potassium/chloride transporters), member 4*	*LCAT|SLC 12A4*	9.59	9.29	7.97	5.08E-04	0.003997352
7913146	22977	*aldo-keto reductase family 7, member A3 (aflatoxin aldehyde reductase)*	*AKR7A3*	7.71	7.87	6.94	5.08E-04	0.00399807
7968297	51371	*proteasome maturation protein*	*POMP*	11.69	11.82	12.14	5.09E-04	0.004001446
7953763				7.09	7.14	6.60	5.09E-04	0.004001446
7954071	93164	*5-hydroxytryptamine (serotonin) receptor 7 pseudogene 1*	*HTR7P1*	8.14	8.23	7.55	5.09E-04	0.004003495
7903688	284613	*cytochrome b-561 domain containing 1*	*CYB561D1*	8.09	8.05	7.09	5.10E-04	0.004004246
8139203	136647	*chromosome 7 open reading frame 11*	*C7orf11*	8.30	8.19	9.04	5.11E-04	0.004015139
7974166	57697	*Fanconi anemia, complementation group M*	*FANCM*	5.51	5.47	6.94	5.11E-04	0.004015432
8027053	23373	*CREB regulated transcription coactivator 1*	*CRTC1*	10.06	9.85	8.76	5.11E-04	0.00401547
8113344				7.56	7.80	6.67	5.12E-04	0.004015577
8006392	5717	*proteasome (prosome, macropain) 26S subunit, non-ATPase, 11*	*PSMD11*	10.19	10.10	11.70	5.12E-04	0.004019283
7907466	55157, 91687	*aspartyl-tRNA synthetase 2, mitochondrial | centromere protein L*	*DARS2|CE NPL*	7.47	7.85	9.70	5.12E-04	0.004019283
8174253	9643	*mortality factor 4 like 2*	*MORF4L2*	9.51	9.67	10.50	5.13E-04	0.004019937
8122720	80328	*UL16 binding protein 2*	*ULBP2*	6.45	6.62	5.74	5.13E-04	0.004019937
7924996	84886	*chromosome 1 open reading frame 198*	*C1orf198*	11.63	11.55	9.47	5.13E-04	0.004019937
8103202				6.17	6.22	5.70	5.14E-04	0.004024428
8009844	3993	*lethal giant larvae homolog 2 (Drosophila)*	*LLGL2*	7.10	7.20	8.11	5.14E-04	0.004026843
8120362	221336	*BEN domain containing 6*	*BEND6*	7.44	7.92	6.35	5.14E-04	0.004026843
8159259	29991, 29989	*odorant binding protein 2A | odorant binding protein 2B*	*OBP2A|O BP2B*	7.61	7.77	6.54	5.14E-04	0.004028087
8074106	113730	*kelch domain containing 7B*	*KLHDC7B*	7.77	8.02	6.89	5.15E-04	0.00402856
7897089	9651	*phospholipase C, eta 2*	*PLCH2*	7.76	7.77	7.08	5.15E-04	0.004029017
7927062	7581	*zinc finger protein 33A*	*ZNF33A*	6.76	6.62	9.22	5.16E-04	0.00403457
7940662	6094	*retinal outer segment membrane protein 1*	*ROM1*	7.42	7.34	6.40	5.18E-04	0.004047554

8055672	27249	*methylmalonic aciduria (cobalamin deficiency) cblD type, with homocystinuria*	*MMADHC*	9.86	9.64	10.51	5.17E-04	0.004047554
8110914				11.51	11.50	11.09	5.19E-04	0.004054755
8088848	23024	*PDZ domain containing ring finger 3*	*PDZRN3*	9.70	9.30	7.81	5.19E-04	0.004055318
8028219	147923	*zinc finger protein 420*	*ZNF420*	6.08	5.89	6.92	5.20E-04	0.004061294
8065353	7056	*thrombomodulin*	*THBD*	7.65	7.79	7.12	5.20E-04	0.004063888
8177750	135644	*tripartite motif-containing 40*	*TRIM40*	6.37	6.45	5.98	5.21E-04	0.004066061
8072113	402055, 24144	*SRR1 domain containing | tuftelin interacting protein 11*	*SRRD|TFIP11*	8.81	8.79	9.87	5.21E-04	0.004066061
8063382	6615	*snail homolog 1 (Drosophila)*	*SNAI1*	9.62	9.85	8.02	5.22E-04	0.004070917
7994804	29895	*myosin light chain, phosphorylatable, fast skeletal muscle*	*MYLPF*	7.06	7.39	6.43	5.22E-04	0.004070917
8132369	54749	*ependymin related protein 1 (zebrafish)*	*EPDR1*	8.28	8.25	7.37	5.22E-04	0.004070917
8088436	200844	*chromosome 3 open reading frame 67*	*C3orf67*	6.38	6.41	7.32	5.22E-04	0.004070917
8177478	5884		*RAD17*	7.18	6.94	8.82	5.22E-04	0.004071868
7981494	207	*v-akt murine thymoma viral oncogene homolog 1*	*AKT1*	11.49	11.45	9.91	5.23E-04	0.004074719
7905028	645166, 654342	*lymphocyte-specific protein 1 pseudogene*	*LOC645166|LOC654342*	10.83	10.99	8.70	5.23E-04	0.004078062
7968126	4046	*lymphocyte-specific protein 1*	*LSP1*	9.21	9.37	7.48	5.25E-04	0.004087815
8053059	388962	*bolA homolog 3 (E. coli)*	*BOLA3*	10.83	11.06	11.95	5.25E-04	0.00409174
8031762	256051	*zinc finger protein 549*	*ZNF549*	6.75	6.58	8.06	5.26E-04	0.0040933
7962427	83448	*pseudouridylate synthase 7 homolog (S. cerevisiae)-like*	*PUS7L*	7.62	7.33	8.98	5.26E-04	0.004093979
7940108	219952	*olfactory receptor, family 6, subfamily Q, member 1*	*OR6Q1*	5.52	5.60	5.27	5.27E-04	0.004097439
8154245	80380	*programmed cell death 1 ligand 2*	*PDCD1LG2*	8.82	9.44	5.68	5.27E-04	0.004100526
7938681				6.97	7.08	6.49	5.28E-04	0.004103873
8038362	113091	*parathyroid hormone 2*	*PTH2*	8.56	8.65	7.83	5.28E-04	0.004106612
8047265	79568, 129450	*chromosome 2 open reading frame 47 | chromosome 2 open reading frame 60*	*C2orf47|C2orf60*	7.46	7.63	8.54	5.29E-04	0.004110826
7971015	4093	*SMAD family member 9*	*SMAD9*	8.65	8.18	6.75	5.29E-04	0.004113442

7945031				4.99	5.00	6.15	5.29E-04	0.004113442
8056102	9936, 4065	*CD302 molecule | lymphocyte antigen 75*	*CD302|LY 75*	10.87	10.19	7.80	5.31E-04	0.004127977
7943231	54851	*ankyrin repeat domain 49*	*ANKRD49*	7.83	7.79	8.69	5.32E-04	0.004129278
8162759	55357	*TBC1 domain family, member 2*	*TBC1D2*	9.11	9.09	7.59	5.32E-04	0.004129278
7973182	554207		*LOC55420 7*	5.40	5.51	5.21	5.32E-04	0.0041293
7996318	123920	*CKLF-like MARVEL transmembrane domain containing 3*	*CMTM3*	10.79	10.93	9.35	5.33E-04	0.004130358
8002492	54768, 100288805	*hydrocephalus inducing homolog (mouse) | hydrocephalus inducing homolog 2 (mouse)*	*HYDIN|HY DIN2*	6.07	6.52	7.18	5.33E-04	0.004130358
8173206				7.32	7.55	9.74	5.33E-04	0.004130358
8070712				5.59	5.75	5.35	5.34E-04	0.004136697
8089145	25890	*ABI family, member 3 (NESH) binding protein*	*ABI3BP*	10.89	10.02	5.61	5.34E-04	0.004139004
8043583	285033		*LOC28503 3*	7.60	7.92	6.44	5.34E-04	0.004139004
8136200	51200	*carboxypeptidase A4*	*CPA4*	8.74	8.58	5.80	5.35E-04	0.004143253
7938000	390059	*olfactory receptor, family 51, subfamily M, member 1*	*OR51M1*	5.64	5.53	5.11	5.35E-04	0.004143253
8039068	4729	*NADH dehydrogenase (ubiquinone) flavoprotein 2, 24kDa*	*NDUFV2*	9.60	9.77	10.68	5.35E-04	0.004145028
7989596	23604	*death-associated protein kinase 2*	*DAPK2*	9.28	8.75	7.52	5.36E-04	0.004149448
8096109	51023, 84142	*mitochondrial ribosomal protein S18C | family with sequence similarity 175, member A*	*MRPS18C| FAM175A*	6.07	6.04	6.82	5.37E-04	0.00415171
8168470	1349	*cytochrome c oxidase subunit VIIb*	*COX7B*	6.53	7.09	8.58	5.37E-04	0.004154229
8002152	6560, 3931	*solute carrier family 12 (potassium/chloride transporters), member 4 | lecithin-cholesterol acyltransferase*	*SLC12A4| LCAT*	10.38	10.45	8.71	5.37E-04	0.004154229
8065252				6.94	6.71	6.06	5.38E-04	0.004156167
8099746	886	*cholecystokinin A receptor*	*CCKAR*	6.76	6.15	5.74	5.39E-04	0.004162707
8019018	8535	*chromobox homolog 4*	*CBX4*	8.93	8.80	7.59	5.40E-04	0.004169214
7954527	56938	*aryl hydrocarbon receptor nuclear translocator-like 2*	*ARNTL2*	6.96	6.88	8.37	5.40E-04	0.004170765
8029541	346	*apolipoprotein C-IV*	*APOC4*	7.35	7.35	6.67	5.41E-04	0.004171653

7933366	2658	*growth differentiation factor 2*	*GDF2*	7.49	7.50	6.72	5.41E-04	0.004171653
7900857	6487	*ST3 beta-galactoside alpha-2,3-sialyltransferase 3*	*ST3GAL3*	8.57	8.82	7.30	5.41E-04	0.004171653
7997158	91862	*MARVEL domain containing 3*	*MARVELD 3*	6.82	6.84	7.85	5.41E-04	0.004171653
7944795	219875	*olfactory receptor, family 4, subfamily D, member 5*	*OR4D5*	6.64	6.62	6.16	5.41E-04	0.004171653
7926531	221079	*ADP-ribosylation factor-like 5B*	*ARL5B*	9.84	9.60	11.66	5.40E-04	0.004171653
8103483				7.56	7.80	6.67	5.41E-04	0.004171653
8176480				5.01	4.85	4.67	5.41E-04	0.004171653
8112865	256987	*serine incorporator 5*	*SERINC5*	8.89	8.69	11.46	5.43E-04	0.004186333
8038598	50944	*SH3 and multiple ankyrin repeat domains 1*	*SHANK1*	8.14	7.99	6.90	5.44E-04	0.004188051
8172056	6103	*retinitis pigmentosa GTPase regulator*	*RPGR*	5.76	5.76	6.56	5.44E-04	0.004188275
8146500	4067	*v-yes-1 Yamaguchi sarcoma viral related oncogene homolog*	*LYN*	7.91	7.88	9.70	5.44E-04	0.004188794
8149228	349196		*LOC34919 6*	9.73	8.72	7.14	5.44E-04	0.004189197
8127158	2729	*glutamate-cysteine ligase, catalytic subunit*	*GCLC*	7.79	7.33	9.82	5.45E-04	0.004190058
7964250	10728	*prostaglandin E synthase 3 (cytosolic)*	*PTGES3*	9.80	9.83	11.16	5.45E-04	0.004190058
8005751				6.89	6.94	6.35	5.45E-04	0.004190058
7913907	84676	*tripartite motif-containing 63*	*TRIM63*	5.97	6.00	5.57	5.45E-04	0.004190922
8176923	114758, 84664, 114817	*chondroitin sulfate proteoglycan 4 pseudogene 1, Y-linked | chondroitin sulfate proteoglycan 4 pseudogene 2, Y-linked | chondroitin sulfate proteoglycan 4 pseudogene 5*	*CSPG4P1Y|CSPG4P2Y|CSPG4P5*	7.54	7.65	6.57	5.45E-04	0.004190922
7945666	1509	*cathepsin D*	*CTSD*	12.84	13.02	11.43	5.46E-04	0.004193119
7946728	5682	*proteasome (prosome, macropain) subunit, alpha type, 1*	*PSMA1*	10.93	10.90	11.90	5.46E-04	0.004193119
8132290				8.09	8.20	7.53	5.46E-04	0.004193119
8062571	81610	*family with sequence similarity 83, member D*	*FAM83D*	7.90	8.10	10.31	5.46E-04	0.004194197
8059565	55022	*phosphotyrosine interaction domain containing 1*	*PID1*	8.64	8.41	6.86	5.47E-04	0.004197048
7944751	79864	*chromosome 11 open reading frame 63*	*C11orf63*	7.80	7.97	6.08	5.47E-04	0.004197071
8147573	116039	*odd-skipped related 2 (Drosophila)*	*OSR2*	12.56	9.95	6.52	5.47E-04	0.004197419

8062312	10398	*myosin, light chain 9, regulatory*	*MYL9*	12.26	12.37	10.56	5.48E-04	0.004201266
8131069	2852	*G protein-coupled estrogen receptor 1*	*GPER*	8.97	10.03	7.23	5.48E-04	0.004203185
7996943				7.17	6.99	6.34	5.48E-04	0.004203185
8013521				8.12	8.11	8.70	5.48E-04	0.004203185
7970232	2155	*coagulation factor VII (serum prothrombin conversion accelerator)*	*F7*	8.15	7.60	7.07	5.49E-04	0.004204813
8054664	84524	*zinc finger CCCH-type containing 8*	*ZC3H8*	6.57	6.62	7.27	5.49E-04	0.004204813
7992121	388199	*proline rich 25*	*PRR25*	6.93	7.02	6.33	5.49E-04	0.004204813
7898693	249	*alkaline phosphatase, liver/bone/kidney*	*ALPL*	8.96	7.35	11.28	5.49E-04	0.004207818
8000003	55623	*THUMP domain containing 1*	*THUMPD 1*	6.79	6.64	8.74	5.50E-04	0.004208909
8025978	284390	*zinc finger protein 763*	*ZNF763*	6.20	6.05	6.57	5.50E-04	0.004208909
7951686	3606	*interleukin 18 (interferon-gamma-inducing factor)*	*IL18*	4.65	4.69	5.36	5.51E-04	0.004212791
7899057	56181	*family with sequence similarity 54, member B*	*FAM54B*	10.48	10.23	9.25	5.51E-04	0.004212791
8114207				7.56	7.79	6.66	5.51E-04	0.004212791
8171587	5256	*phosphorylase kinase, alpha 2 (liver)*	*PHKA2*	8.58	8.14	9.04	5.51E-04	0.004214311
8124446				7.11	7.35	9.11	5.51E-04	0.004214311
8042259	4190	*malate dehydrogenase 1, NAD (soluble)*	*MDH1*	8.65	8.84	10.38	5.52E-04	0.004215394
7934299	25961	*nudix (nucleoside diphosphate linked moiety X)-type motif 13*	*NUDT13*	6.79	6.75	8.11	5.52E-04	0.004215394
7958158	29915	*host cell factor C2*	*HCFC2*	8.27	7.71	6.02	5.52E-04	0.004215394
7920757	2224, 284618	*farnesyl diphosphate synthase | chromosome 1 open reading frame 104*	*FDPS|C1o rf104*	7.52	7.63	6.52	5.52E-04	0.004215394
8010186				5.93	6.07	5.59	5.52E-04	0.004215394
8081959	9515	*syntaxin binding protein 5-like*	*STXBP5L*	5.09	5.06	5.96	5.53E-04	0.004219233
8006999	1440	*colony stimulating factor 3 (granulocyte)*	*CSF3*	8.55	8.64	7.72	5.54E-04	0.004219318
8142120	10135	*nicotinamide phosphoribosyltransferase*	*NAMPT*	9.49	9.10	11.13	5.54E-04	0.004219318
8098263	23022	*palladin, cytoskeletal associated protein*	*PALLD*	9.58	8.91	7.44	5.54E-04	0.004219318
8066231	85449		*KIAA1755*	7.46	7.25	6.53	5.54E-04	0.004219318
8123651	347733	*tubulin, beta 2B*	*TUBB2B*	8.16	8.62	11.52	5.53E-04	0.004219318

7900508				5.93	6.15	5.75	5.54E-04	0.004219318
8043743				9.98	9.90	9.09	5.54E-04	0.004219318
8088526	80145	*THO complex 7 homolog (Drosophila)*	*THOC7*	8.49	8.82	9.52	5.55E-04	0.004222853
7897263	388591	*ring finger protein 207*	*RNF207*	7.89	7.59	6.95	5.55E-04	0.004222853
8067288	514	*ATP synthase, H+ transporting, mitochondrial F1 complex, epsilon subunit*	*ATP5E*	10.05	10.17	9.66	5.55E-04	0.004227054
8084206	84002	*UDP-GlcNAc:betaGal beta-1,3-N-acetylglucosaminyltransferase 5*	*B3GNT5*	5.74	5.49	7.77	5.56E-04	0.004227054
8113790	115123	*membrane-associated ring finger (C3HC4) 3*	*3-Mar*	9.09	9.40	10.28	5.56E-04	0.004227054
7949863	347853	*T-box 10*	*TBX10*	7.51	7.48	6.86	5.57E-04	0.004232794
7961306	5542, 653247	*proline-rich protein BstNI subfamily 1 | proline-rich protein BstNI subfamily 2*	*PRB1|PRB 2*	7.52	7.59	7.03	5.58E-04	0.004239457
8141395	4176	*minichromosome maintenance complex component 7*	*MCM7*	9.04	9.75	11.76	5.60E-04	0.004255067
7993024	146562	*chromosome 16 open reading frame 71*	*C16orf71*	7.13	7.15	6.55	5.60E-04	0.004255067
8122684	387082	*SMT3 suppressor of mif two 3 homolog 4 (S. cerevisiae)*	*SUMO4*	7.34	7.36	8.40	5.61E-04	0.004263845
7971361	26747	*nuclear fragile X mental retardation protein interacting protein 1*	*NUFIP1*	8.13	8.09	10.21	5.62E-04	0.004265281
7968653	5994	*regulatory factor X-associated protein*	*RFXAP*	6.38	6.47	6.97	5.62E-04	0.004267636
8008784	55771	*proline rich 11*	*PRR11*	7.87	8.08	10.71	5.62E-04	0.004269403
7904254	476	*ATPase, Na+/K+ transporting, alpha 1 polypeptide*	*ATP1A1*	11.74	11.49	12.13	5.63E-04	0.004272778
8070900				6.62	6.58	6.20	5.63E-04	0.004272778
8060813	84515	*minichromosome maintenance complex component 8*	*MCM8*	6.76	6.69	10.27	5.65E-04	0.004284215
8053386				6.55	6.70	7.36	5.65E-04	0.004284215
7991540				6.87	7.14	6.16	5.66E-04	0.004291901
8033801	54811	*zinc finger protein 562*	*ZNF562*	6.96	7.09	8.24	5.67E-04	0.004294268
7997168	9798		*KIAA0174*	10.74	10.67	11.56	5.67E-04	0.004299425
8075616	8224	*synapsin III*	*SYN3*	6.90	7.14	8.56	5.68E-04	0.004303365
8170704	215	*ATP-binding cassette, sub-family D (ALD), member 1*	*ABCD1*	9.61	9.39	7.28	5.69E-04	0.004307508
7957092	196446	*chromosome 12 open reading frame 28*	*C12orf28*	5.42	5.53	6.76	5.69E-04	0.004307508
7927645				7.48	7.49	6.69	5.70E-04	0.004315201

7954631	55711	*fatty acyl CoA reductase 2*	*FAR2*	7.21	6.42	9.46	5.70E-04	0.004315953
7910971	128025	*WD repeat domain 64*	*WDR64*	4.75	4.82	4.71	5.70E-04	0.004315953
8065444	84532	*acyl-CoA synthetase short-chain family member 1*	*ACSS1*	7.89	7.60	6.77	5.71E-04	0.004316676
8117382	3017	*histone cluster 1, H2bd*	*HIST1H2BD*	6.76	6.80	8.00	5.72E-04	0.004323361
8137252	170575	*GTPase, IMAP family member 1*	*GIMAP1*	6.42	6.46	6.18	5.72E-04	0.004323361
7908924	5549	*proline/arginine-rich end leucine-rich repeat protein*	*PRELP*	11.25	10.25	7.09	5.73E-04	0.004326684
8148385	7038	*thyroglobulin*	*TG*	6.65	6.72	6.01	5.73E-04	0.004329958
8179060	135644	*tripartite motif-containing 40*	*TRIM40*	6.36	6.45	5.98	5.73E-04	0.0043306
7907404	51430	*chromosome 1 open reading frame 9*	*C1orf9*	7.40	7.42	8.94	5.74E-04	0.004332728
7950641	65987	*potassium channel tetramerisation domain containing 14*	*KCTD14*	8.28	7.66	10.68	5.74E-04	0.004332888
8149218	349196		*LOC349196*	9.77	8.73	7.09	5.74E-04	0.004332888
8149220	349196		*LOC349196*	9.77	8.73	7.09	5.74E-04	0.004332888
7963212	4891	*solute carrier family 11 (proton-coupled divalent metal ion transporters), member 2*	*SLC11A2*	8.37	8.44	9.49	5.75E-04	0.004336109
8101043	9908	*GTPase activating protein (SH3 domain) binding protein 2*	*G3BP2*	9.17	9.09	11.54	5.75E-04	0.004336109
7915485	10969	*EBNA1 binding protein 2*	*EBNA1BP2*	9.00	9.25	10.05	5.76E-04	0.004336109
7957186	64786	*TBC1 domain family, member 15*	*TBC1D15*	9.08	8.92	10.08	5.75E-04	0.004336109
7986325				4.54	4.46	4.44	5.75E-04	0.004336109
8024566				10.99	10.89	8.78	5.76E-04	0.004336109
7936406				7.62	7.56	6.79	5.76E-04	0.004337236
7909992	23219	*F-box protein 28*	*FBXO28*	7.92	7.86	9.68	5.76E-04	0.004337418
8157105	58499	*zinc finger protein 462*	*ZNF462*	8.50	8.23	11.45	5.76E-04	0.004338852
7985025	161753	*outer dense fiber of sperm tails 3-like 1*	*ODF3L1*	6.75	6.69	6.02	5.77E-04	0.004338852
7911422	51150	*stromal cell derived factor 4*	*SDF4*	10.50	10.45	8.85	5.78E-04	0.004345121
8149222	349196		*LOC349196*	9.77	8.73	7.09	5.78E-04	0.004345121

7927082	158160	*hydroxysteroid (17-beta) dehydrogenase 7 pseudogene 2*	*HSD17B7 P2*	6.23	5.86	7.63	5.78E-04	0.004346665
7970455	51084	*crystallin, lambda 1*	*CRYL1*	8.73	8.26	7.16	5.78E-04	0.004348616
8063484	1477	*cleavage stimulation factor, 3' pre-RNA, subunit 1, 50kDa*	*CSTF1*	8.54	8.64	10.22	5.79E-04	0.004349233
7905492	353143	*late cornified envelope 3B*	*LCE3B*	8.92	8.89	8.11	5.79E-04	0.004349233
8106660	5924	*Ras protein-specific guanine nucleotide-releasing factor 2*	*RASGRF2*	7.68	6.00	9.47	5.79E-04	0.004349458
7979574	81537	*sphingosine-1-phosphate phosphatase 1*	*SGPP1*	8.36	8.60	7.54	5.79E-04	0.004349458
8117458	11119	*butyrophilin, subfamily 3, member A1*	*BTN3A1*	9.16	8.58	7.05	5.79E-04	0.004349924
8132151	117	*adenylate cyclase activating polypeptide 1 (pituitary) receptor type I*	*ADCYAP1 R1*	6.15	6.31	7.92	5.80E-04	0.004350626
7962000	5744	*parathyroid hormone-like hormone*	*PTHLH*	7.19	7.28	6.41	5.81E-04	0.004356527
7924445	400804		*LOC40080 4*	5.71	5.59	5.24	5.81E-04	0.00435785
7919763	2029	*endosulfine alpha*	*ENSA*	7.41	7.41	8.72	5.82E-04	0.004362696
8166049	5634	*phosphoribosyl pyrophosphate synthetase 2*	*PRPS2*	9.19	8.87	10.34	5.82E-04	0.004362696
8083183	23350		*SR140*	8.87	8.64	11.07	5.82E-04	0.004362696
8167322	64743	*WD repeat domain 13*	*WDR13*	9.56	9.41	7.88	5.82E-04	0.004362696
8015642	29893, 6945	*PSMC3 interacting protein | MAX-like protein X*	*PSMC3IP| MLX*	6.47	6.68	7.54	5.82E-04	0.004362696
7939934				5.34	5.13	4.94	5.83E-04	0.004364543
7923378	1465	*cysteine and glycine-rich protein 1*	*CSRP1*	12.07	12.76	9.80	5.83E-04	0.004366336
7914648	1912	*polyhomeotic homolog 2 (Drosophila)*	*PHC2*	8.90	8.93	7.20	5.83E-04	0.004366336
8107857				8.68	9.19	7.22	5.83E-04	0.004366336
8113786				4.53	4.58	4.40	5.84E-04	0.004366336
7948101	403253	*olfactory receptor, family 4, subfamily A, member 47*	*OR4A47*	5.14	5.14	4.78	5.85E-04	0.004373395
8131385				6.41	6.42	5.75	5.85E-04	0.004373395
7998700	5310	*polycystic kidney disease 1 (autosomal dominant)*	*PKD1*	9.17	9.25	7.55	5.85E-04	0.004373607
8149224	349196		*LOC34919 6*	9.77	8.73	7.08	5.85E-04	0.004373607
8040473	388	*ras homolog gene family, member B*	*RHOB*	10.17	10.30	8.84	5.85E-04	0.004373959

7901192	8438	*RAD54-like (S. cerevisiae)*	*RAD54L*	6.68	6.94	8.87	5.86E-04	0.00437896
7982309	22909, 54893	*FANCD2/FANCI-associated nuclease 1 | myotubularin related protein 10*	*FAN1|MTMR10*	6.70	6.69	8.33	5.86E-04	0.004379724
8149226	349196		*LOC349196*	9.77	8.73	7.09	5.87E-04	0.004385201
7911754	8764	*tumor necrosis factor receptor superfamily, member 14 (herpesvirus entry mediator)*	*TNFRSF14*	8.64	8.35	6.84	5.88E-04	0.004387176
8027770	53822	*FXYD domain containing ion transport regulator 7*	*FXYD7*	8.12	8.11	8.89	5.88E-04	0.004387176
7903334	8556	*CDC14 cell division cycle 14 homolog A (S. cerevisiae)*	*CDC14A*	6.94	6.96	8.55	5.88E-04	0.004387918
8079613				4.75	4.87	5.50	5.88E-04	0.004387918
8001656				6.57	6.96	5.55	5.89E-04	0.004393457
8000244	57478	*ubiquitin specific peptidase 31*	*USP31*	8.71	8.51	9.53	5.89E-04	0.004394223
7912806				6.74	6.22	8.26	5.91E-04	0.004401771
7956551	115557, 65012	*Rho guanine nucleotide exchange factor (GEF) 25 | solute carrier family 26, member 10*	*ARHGEF25|SLC26A10*	9.60	9.13	7.67	5.91E-04	0.004403249
8057797	8436	*serum deprivation response*	*SDPR*	7.70	7.60	6.22	5.91E-04	0.00440385
7922266				6.18	6.24	5.83	5.91E-04	0.004404951
8170576	158511	*chondrosarcoma associated gene 1*	*CSAG1*	6.48	6.46	5.92	5.92E-04	0.004409086
8049538	9208	*leucine rich repeat (in FLII) interacting protein 1*	*LRRFIP1*	9.60	9.62	8.71	5.93E-04	0.004411027
7963396	9119	*keratin 75*	*KRT75*	6.09	5.99	5.71	5.93E-04	0.00441203
7984132	9960	*ubiquitin specific peptidase 3*	*USP3*	9.54	9.60	7.80	5.94E-04	0.004418703
8011275	79066	*methyltransferase 10 domain containing*	*METT10D*	7.25	7.32	8.91	5.94E-04	0.004418703
7945086	114609	*toll-interleukin 1 receptor (TIR) domain containing adaptor protein*	*TIRAP*	7.84	7.79	6.79	5.94E-04	0.004418703
8165656				11.31	11.20	10.50	5.94E-04	0.004418703
8105353	23517, 8611	*superkiller viralicidic activity 2-like 2 (S. cerevisiae) | phosphatidic acid phosphatase type 2A*	*SKIV2L2|PPAP2A*	9.33	9.10	11.07	5.95E-04	0.004420805
7925161	359948	*interferon regulatory factor 2 binding protein 2*	*IRF2BP2*	9.96	10.34	9.50	5.95E-04	0.004421594
7922162	10560	*solute carrier family 19 (thiamine transporter), member 2*	*SLC19A2*	6.43	6.85	8.70	5.96E-04	0.0044256

8161618	320	*amyloid beta (A4) precursor protein-binding, family A, member 1*	*APBA1*	7.53	7.28	6.68	5.96E-04	0.004427365
8102352	5308	*paired-like homeodomain 2*	*PITX2*	7.13	7.47	6.21	5.96E-04	0.004427365
7897196	148870	*coiled-coil domain containing 27*	*CCDC27*	6.85	6.81	6.32	5.96E-04	0.004427697
7998190	8786	*regulator of G-protein signaling 11*	*RGS11*	8.36	7.89	6.72	5.97E-04	0.004430489
8119435	9436	*natural cytotoxicity triggering receptor 2*	*NCR2*	7.09	7.11	6.61	5.98E-04	0.004435828
8166950	63904	*dual specificity phosphatase 21*	*DUSP21*	5.73	5.77	5.34	5.98E-04	0.004436791
8114489				6.60	6.54	6.14	5.98E-04	0.004437299
7917670				7.77	7.78	6.78	5.99E-04	0.004438532
7905035				7.40	7.30	6.76	5.99E-04	0.004439658
7916489				7.32	7.31	6.61	5.99E-04	0.004439658
7953873	408186, 144203	*ovostatin | ovostatin 2*	*OVOS|OVOS2*	5.04	5.03	7.08	6.00E-04	0.004444957
7961489	51729	*WW domain binding protein 11*	*WBP11*	10.06	10.09	11.33	6.01E-04	0.004451792
8143697	136051	*zinc finger protein 786*	*ZNF786*	7.18	7.17	8.07	6.01E-04	0.004452458
7901744				6.74	6.69	6.25	6.01E-04	0.004452458
8034762	5566	*protein kinase, cAMP-dependent, catalytic, alpha*	*PRKACA*	10.05	10.05	8.24	6.03E-04	0.004458802
8041982	98	*acylphosphatase 2, muscle type*	*ACYP2*	7.30	7.65	6.59	6.03E-04	0.00445923
7968759				7.06	7.34	6.48	6.03E-04	0.004460717
7949383	84447	*synovial apoptosis inhibitor 1, synoviolin*	*SYVN1*	10.23	10.37	8.72	6.03E-04	0.004460818
7910217	89780	*wingless-type MMTV integration site family, member 3A*	*WNT3A*	7.13	7.25	6.73	6.03E-04	0.004460818
8176306	1438	*colony stimulating factor 2 receptor, alpha, low-affinity (granulocyte-macrophage)*	*CSF2RA*	6.27	6.42	6.80	6.04E-04	0.004463025
7963375	3890	*keratin 84*	*KRT84*	6.84	6.95	6.49	6.04E-04	0.004463025
7997414	8139	*gigaxonin*	*GAN*	7.52	7.68	9.74	6.04E-04	0.004463025
8098604	353322, 55325	*ankyrin repeat domain 37 | UFM1-specific peptidase 2*	*ANKRD37|UFSP2*	7.84	8.15	7.04	6.04E-04	0.004463025
8089928				8.15	8.18	7.16	6.04E-04	0.004463025
8148057				13.40	13.42	13.06	6.05E-04	0.004465175
8106193	84135		*UTP15*	6.68	6.93	9.25	6.05E-04	0.004467733

7974404	1033	*cyclin-dependent kinase inhibitor 3*	*CDKN3*	7.02	7.17	9.19	6.06E-04	0.004468677
8174636	6613, 728825	*SMT3 suppressor of mif two 3 homolog 2 (S. cerevisiae) | SMT3 suppressor of mif two 3 homolog 2 (S. cerevisiae) pseudogene*	*SUMO2|LOC728825*	11.20	11.25	12.34	6.06E-04	0.004472352
8161433				9.27	9.00	8.27	6.07E-04	0.004475499
8028186	7705	*zinc finger protein 146*	*ZNF146*	8.36	8.26	10.66	6.09E-04	0.004484955
8010137	677781	*small Cajal body-specific RNA 16*	*SCARNA16*	7.85	7.94	8.84	6.08E-04	0.004484955
7951614	5519	*protein phosphatase 2, regulatory subunit A, beta*	*PPP2R1B*	8.23	8.15	10.52	6.09E-04	0.004487922
8157632	254956	*MORN repeat containing 5*	*MORN5*	6.10	6.07	5.64	6.10E-04	0.004491961
7977609	341799	*olfactory receptor, family 6, subfamily S, member 1*	*OR6S1*	5.43	5.31	5.17	6.10E-04	0.004491961
8061542	81502	*histocompatibility (minor) 13*	*HM13*	11.56	11.63	10.23	6.11E-04	0.004497186
8095800				7.74	7.75	7.16	6.11E-04	0.004497186
7917037	1429	*crystallin, zeta (quinone reductase)*	*CRYZ*	9.34	9.02	10.25	6.11E-04	0.004497445
8156452	23196	*family with sequence similarity 120A*	*FAM120A*	9.34	9.43	8.57	6.12E-04	0.004503048
8095574	1633	*deoxycytidine kinase*	*DCK*	7.26	7.54	8.37	6.12E-04	0.004503085
8020903	2589	*UDP-N-acetyl-alpha-D-galactosamine:polypeptide N-acetylgalactosaminyltransferase 1 (GalNAc-T1)*	*GALNT1*	7.98	7.84	10.05	6.13E-04	0.00450711
8144420	349196		*LOC349196*	9.77	8.67	7.08	6.14E-04	0.00451514
8055978	257415, 728066, 728640	*family with sequence similarity 133, member B | family with sequence similarity 133, member B pseudogene*	*FAM133B|LOC728066|LOC728640*	7.97	7.87	9.61	6.14E-04	0.0045152
8142171	1811	*solute carrier family 26, member 3*	*SLC26A3*	5.44	5.37	5.34	6.15E-04	0.004518961
7925457	6000	*regulator of G-protein signaling 7*	*RGS7*	8.42	6.25	6.70	6.15E-04	0.004520693
8076497	53947	*alpha 1,4-galactosyltransferase*	*A4GALT*	9.42	9.00	7.48	6.16E-04	0.004521022
8103853	90768	*hypothetical LOC90768*	*MGC45800*	7.10	8.43	8.57	6.16E-04	0.004521022
7956522	3798	*kinesin family member 5A*	*KIF5A*	6.47	6.37	9.18	6.16E-04	0.004521412

8155521	548321, 100133121, 100132948, 724094	*family with sequence similarity 27, member A | family with sequence similarity 27, member B | family with sequence similarity 27, member C | family with sequence similarity 27, member D1*	*FAM27A|FAM27B|FAM27C|FAM27D1*	8.49	8.84	7.75	6.16E-04	0.004521412
7925904	83592	*aldo-keto reductase family 1, member E2*	*AKR1E2*	8.32	8.33	7.07	6.16E-04	0.004521745
7988227	619189, 10169	*serine incorporator 4 | small EDRK-rich factor 2*	*SERINC4|SERF2*	6.30	6.18	5.92	6.16E-04	0.004521745
8026806	79709	*glycosyltransferase 25 domain containing 1*	*GLT25D1*	11.04	11.16	9.28	6.17E-04	0.004526828
7987180	79768, 729176	*chromosome 15 open reading frame 29 | chromosome 15 open reading frame 29 pseudogene*	*C15orf29|LOC729176*	7.44	7.66	8.79	6.18E-04	0.004527257
8055089	5433	*polymerase (RNA) II (DNA directed) polypeptide D*	*POLR2D*	8.85	8.86	9.99	6.18E-04	0.004529254
7983490	283651	*Dresden prostate cancer 2*	*C15orf21*	6.22	6.10	5.77	6.20E-04	0.004539655
8117476	10384	*butyrophilin, subfamily 3, member A3*	*BTN3A3*	9.20	8.25	6.19	6.21E-04	0.004545542
8115978	51720	*ubiquitin interaction motif containing 1*	*UIMC1*	6.95	7.19	8.14	6.21E-04	0.004546447
8022326	65258	*metallophosphoesterase 1*	*MPPE1*	7.91	7.93	7.06	6.21E-04	0.004549167
7899029	57134	*mannosidase, alpha, class 1C, member 1*	*MAN1C1*	9.67	8.25	7.69	6.22E-04	0.00455022
8144494	349196		*LOC349196*	9.76	8.67	7.07	6.22E-04	0.004551048
8128939	10758	*TRAF3 interacting protein 2*	*TRAF3IP2*	10.57	10.27	8.74	6.22E-04	0.004551572
7904907	607	*B-cell CLL/lymphoma 9*	*BCL9*	9.29	8.66	9.94	6.23E-04	0.004559454
8044700	57628	*dipeptidyl-peptidase 10 (non-functional)*	*DPP10*	5.28	5.31	7.63	6.24E-04	0.004563965
8035236	93323	*HAUS augmin-like complex, subunit 8*	*HAUS8*	6.46	6.51	7.37	6.25E-04	0.004569559
8089249	6152, 256691	*ribosomal protein L24 | MAM domain containing 2*	*RPL24|MAMDC2*	10.81	10.96	11.51	6.25E-04	0.004569559
7902382	5876	*Rab geranylgeranyltransferase, beta subunit*	*RABGGTB*	8.01	8.05	9.70	6.26E-04	0.004571124
7901052	94163	*small nucleolar RNA, C/D box 38B*	*SNORD38B*	6.44	6.33	7.86	6.26E-04	0.004571124
8144758	51201	*zinc finger, DHHC-type containing 2*	*ZDHHC2*	9.19	8.40	9.24	6.26E-04	0.004574176
8158725	25	*c-abl oncogene 1, non-receptor tyrosine kinase*	*ABL1*	10.81	10.60	9.67	6.27E-04	0.004576583
7995739	2775	*guanine nucleotide binding protein (G protein), alpha activating activity polypeptide O*	*GNAO1*	6.55	6.60	8.39	6.29E-04	0.004591902

8105878	5884		*RAD17*	7.32	7.10	8.87	6.29E-04	0.004591902
8074063	79924	*adrenomedullin 2*	*ADM2*	9.53	9.62	8.10	6.30E-04	0.004592896
8176926	9085, 253175, 9426, 203611	*chromodomain protein, Y-linked, 1 | chromodomain protein, Y-linked, 1B | chromodomain protein, Y-linked, 2A | chromodomain protein, Y-linked, 2B*	*CDY1|CDY1B|CDY2A|CDY2B*	5.13	5.20	4.97	6.30E-04	0.004592896
7927889	55749	*cell division cycle and apoptosis regulator 1*	*CCAR1*	9.67	9.56	11.39	6.31E-04	0.004601484
7968761	79612	*N(alpha)-acetyltransferase 16, NatA auxiliary subunit*	*NAA16*	7.49	6.57	8.97	6.32E-04	0.004606367
7930208	9118	*internexin neuronal intermediate filament protein, alpha*	*INA*	7.86	7.16	10.10	6.32E-04	0.004608219
8020384	80000	*growth regulation by estrogen in breast cancer-like*	*GREB1L*	6.10	7.22	5.62	6.32E-04	0.004608219
8082940	25852	*armadillo repeat containing 8*	*ARMC8*	7.40	7.31	9.29	6.35E-04	0.004622516
8026926	23031	*microtubule associated serine/threonine kinase 3*	*MAST3*	9.65	9.54	7.97	6.35E-04	0.004624362
8023375				5.78	5.75	5.51	6.35E-04	0.004624362
8024429	268, 100423031	*anti-Mullerian hormone | microRNA 4321*	*AMH|MIR4321*	8.59	8.73	7.75	6.36E-04	0.004626093
7975851	112752	*chromosome 14 open reading frame 179*	*C14orf179*	8.58	8.78	7.97	6.36E-04	0.004627681
7996281	146227	*brain expressed, associated with NEDD4, 1*	*BEAN1*	7.28	7.38	6.78	6.36E-04	0.004627681
7942520				6.40	6.48	7.06	6.36E-04	0.004627681
8121561				5.63	5.78	5.51	6.36E-04	0.004628473
8019828	1068	*centrin, EF-hand protein, 1*	*CETN1*	5.81	6.04	5.61	6.38E-04	0.004630558
8057480	4760	*neurogenic differentiation 1*	*NEUROD1*	6.16	6.23	5.56	6.38E-04	0.004630558
8066214	7052	*transglutaminase 2 (C polypeptide, protein-glutamine-gamma-glutamyltransferase)*	*TGM2*	11.92	11.44	7.57	6.37E-04	0.004630558
8054135	11320	*mannosyl (alpha-1,3-)-glycoprotein beta-1,4-N-acetylglucosaminyltransferase, isozyme A*	*MGAT4A*	5.18	5.22	6.30	6.37E-04	0.004630558
8144410	349196		*LOC349196*	9.77	8.68	7.05	6.38E-04	0.004630558
8071312	406961	*microRNA 185*	*MIR185*	8.06	8.16	7.37	6.37E-04	0.004630558
7901746				8.29	8.38	7.81	6.38E-04	0.004630558
8161426	100131997, 100289124	*family with sequence similarity 27, member E3 | family with sequence similarity 27, member E2*	*FAM27E3|FAM27E2*	7.78	7.94	7.03	6.39E-04	0.004635447

8157691	348235, 729012	*spindle and kinetochore associated complex subunit 2 | spindle and kinetochore associated complex subunit 2-like*	*SKA2|SKA2L*	10.44	10.17	12.16	6.39E-04	0.004635447
7944271	143941	*tetratricopeptide repeat domain 36*	*TTC36*	8.43	8.45	7.48	6.39E-04	0.00463658
7993821	400508	*non-protein coding RNA 169*	*NCRNA00169*	6.77	7.18	6.23	6.39E-04	0.004636688
7974303	81542	*thioredoxin-related transmembrane protein 1*	*TMX1*	9.99	9.68	11.59	6.40E-04	0.004639267
8001529				9.10	9.08	8.17	6.40E-04	0.004639267
8179704	8870	*immediate early response 3*	*IER3*	10.31	11.36	8.83	6.41E-04	0.004645473
7900461	127396	*zinc finger protein 684*	*ZNF684*	5.65	5.59	6.21	6.41E-04	0.004646875
7974372	283554	*G protein-coupled receptor 137C*	*GPR137C*	5.89	5.91	7.10	6.42E-04	0.004647884
7982745	113189	*carbohydrate (N-acetylgalactosamine 4-0) sulfotransferase 14*	*CHST14*	9.71	9.60	8.46	6.42E-04	0.004649918
8124848	8870	*immediate early response 3*	*IER3*	10.31	11.36	8.83	6.42E-04	0.004650867
8173732	51616		*TAF9B*	10.34	9.34	11.44	6.43E-04	0.004657094
8149243	377630, 645836, 401447, 392188, 391627	*ubiquitin specific peptidase 17-like 2 | ubiquitin specific peptidase 17-like 3 | ubiquitin specific peptidase 17-like 1 (pseudogene) | ubiquitin specific peptidase 17-like 8 | ubiquitin specific peptidase 17*	*USP17L2|USP17L3|USP17L1P|USP17L8*	7.43	7.37	6.81	6.44E-04	0.00466095
7922590	400797	*non-protein coding RNA 83*	*NCRNA00083*	7.19	7.29	6.76	6.45E-04	0.00466363
8176263	51616		*TAF9B*	10.35	9.34	11.44	6.45E-04	0.004664839
7994961	1489	*cardiotrophin 1*	*CTF1*	9.86	9.95	8.57	6.45E-04	0.004665719
8009561	6169, 118424	*ribosomal protein L38 | ubiquitin-conjugating enzyme E2, J2 (UBC6 homolog, yeast)*	*RPL38|UBE2J2*	12.78	12.79	12.99	6.47E-04	0.004673234
8143957	6009	*Ras homolog enriched in brain*	*RHEB*	9.59	9.95	10.86	6.47E-04	0.004674966

8167573	729428, 729422, 100132399, 729431, 645073, 729442, 729396, 645051, 729447, 645037, 2574, 729408, 2576, 2578, 2577, 26748, 2579, 100008586, 2543	*G antigen 12B | G antigen 12C | G antigen 12D | G antigen 12E | G antigen 12G | G antigen 12H | G antigen 12J | G antigen 13 | G antigen 2A | G antigen 2B | G antigen 2C | G antigen 2D | G antigen 4 | G antigen 6 | G antigen 5 | G antigen 12I | G antigen 7 | G antigen 12F | G antigen 1*	*GAGE12B|GAGE12C|GAGE12D|GAGE12E|GAGE12G|GAGE12H|GAGE12J|GAGE13|GAGE2A|GAGE2B|GAGE2C|GAGE2D|GAGE4|GAGE6|GAGE5|GAGE12I|GAGE7|GAGE12F*	9.14	8.72	8.41	6.48E-04	0.00468096
8096061	255119, 2250	*chromosome 4 open reading frame 22 | fibroblast growth factor 5*	*C4orf22|FGF5*	6.22	5.40	4.96	6.48E-04	0.004681421
7982792	5888		*RAD51*	6.91	7.48	9.87	6.49E-04	0.004686341
8049180	9470	*eukaryotic translation initiation factor 4E family member 2*	*EIF4E2*	9.86	10.04	10.73	6.50E-04	0.004689951
7898609	5322	*phospholipase A2, group V*	*PLA2G5*	6.68	6.85	6.03	6.50E-04	0.0046924
7996891	84916	*cirrhosis, autosomal recessive 1A (cirhin)*	*CIRH1A*	10.94	11.43	12.22	6.50E-04	0.0046924
8132960				5.87	6.04	7.31	6.50E-04	0.0046924
8070102	2618	*phosphoribosylglycinamide formyltransferase, phosphoribosylglycinamide synthetase, phosphoribosylaminoimidazole synthetase*	*GART*	8.02	8.21	10.26	6.52E-04	0.004702142
7961540	85004	*RAS-like, estrogen-regulated, growth inhibitor*	*RERG*	5.73	5.85	6.46	6.52E-04	0.004702142
8051422	151325	*myeloid-associated differentiation marker-like*	*MYADML*	7.04	7.14	6.19	6.52E-04	0.004702142
8154047	10655	*doublesex and mab-3 related transcription factor 2*	*DMRT2*	5.60	5.77	5.39	6.53E-04	0.004702457
8142774	55131	*RNA binding motif protein 28*	*RBM28*	7.31	7.61	8.95	6.53E-04	0.004703952

7929383	159371	*transmembrane protein 20*	*TMEM20*	7.18	6.97	7.96	6.53E-04	0.004703952
8099815				6.24	6.33	5.76	6.53E-04	0.004705361
7939546	3732		*CD82*	9.19	8.29	7.53	6.55E-04	0.004713199
8161437	100131997, 100289124	*family with sequence similarity 27, member E3 | family with sequence similarity 27, member E2*	*FAM27E3|FAM27E2*	7.77	7.93	7.02	6.55E-04	0.004713199
8166580				9.35	9.46	10.96	6.56E-04	0.004722657
7903010	6125, 26782, 388650	*ribosomal protein L5 | small nucleolar RNA, H/ACA box 66 | family with sequence similarity 69, member A*	*RPL5|SNORA66|FAM69A*	8.27	8.22	9.05	6.57E-04	0.004725248
8166876	1654	*DEAD (Asp-Glu-Ala-Asp) box polypeptide 3, X-linked*	*DDX3X*	8.45	8.40	10.47	6.58E-04	0.004726379
7903214	9890	*lipid phosphate phosphatase-related protein type 4*	*LPPR4*	6.44	6.54	7.66	6.58E-04	0.004726379
7915444	64175	*leucine proline-enriched proteoglycan (leprecan) 1*	*LEPRE1*	10.46	10.50	8.50	6.57E-04	0.004726379
8076937	83933	*histone deacetylase 10*	*HDAC10*	8.95	8.66	7.74	6.58E-04	0.004726379
8090872	339855	*kyphoscoliosis peptidase*	*KY*	6.57	6.56	5.92	6.58E-04	0.004726379
8169263	340547	*V-set and immunoglobulin domain containing 1*	*VSIG1*	5.76	5.60	6.74	6.58E-04	0.004726379
8165684				11.64	11.50	10.74	6.59E-04	0.00473168
8045697	55183	*RAP1 interacting factor homolog (yeast)*	*RIF1*	8.21	8.02	11.00	6.60E-04	0.00473295
8025478	84527, 7730	*zinc finger protein 559 | zinc finger protein 177*	*ZNF559|ZNF177*	7.02	6.89	8.50	6.59E-04	0.00473295
8166353				4.47	4.50	4.45	6.60E-04	0.00473295
8155200	881	*calicin*	*CCIN*	6.65	7.22	5.90	6.60E-04	0.004736829
8034565	1777	*deoxyribonuclease II, lysosomal*	*DNASE2*	10.25	10.00	8.58	6.60E-04	0.004736829
8119661	88745	*ribosomal RNA processing 36 homolog (S. cerevisiae)*	*RRP36*	8.06	8.23	8.97	6.61E-04	0.004740811
7968892	387923	*stress-associated endoplasmic reticulum protein family member 2*	*SERP2*	8.01	8.06	6.84	6.61E-04	0.004740811
7991777	201725	*chromosome 4 open reading frame 46*	*C4orf46*	8.36	7.82	10.15	6.62E-04	0.004741619
7897966	93190	*chromosome 1 open reading frame 158*	*C1orf158*	6.19	7.00	5.64	6.62E-04	0.004743115
7909390	1379	*complement component (3b/4b) receptor 1-like*	*CR1L*	4.70	4.83	5.64	6.63E-04	0.004749938
7972021	9882	*TBC1 domain family, member 4*	*TBC1D4*	7.69	7.68	8.99	6.64E-04	0.004750767
8137334	11194	*ATP-binding cassette, sub-family B (MDR/TAP), member 8*	*ABCB8*	7.52	7.54	6.80	6.63E-04	0.004750767

7938066	390083	*olfactory receptor, family 56, subfamily A, member 3*	*OR56A3*	6.61	6.52	6.24	6.64E-04	0.004750767
8040163	285148, 6868	*isoamyl acetate-hydrolyzing esterase 1 homolog (S. cerevisiae) | ADAM metallopeptidase domain 17*	*IAH1|ADAM17*	10.13	10.08	8.43	6.64E-04	0.004750767
8161513	595135, 5239	*phosphoglucomutase 5 pseudogene 2*	*PGM5P2*	7.20	6.78	5.78	6.64E-04	0.004750767
8095331				4.74	4.68	5.32	6.64E-04	0.004750767
7944113	22897	*centrosomal protein 164kDa*	*CEP164*	8.11	8.11	7.40	6.65E-04	0.004757463
8036777	2091	*fibrillarin*	*FBL*	11.19	11.25	12.57	6.67E-04	0.004762399
8160016	26953	*RAN binding protein 6*	*RANBP6*	7.76	7.68	9.39	6.67E-04	0.004762399
8168438	139596	*uracil phosphoribosyltransferase (FUR1) homolog (S. cerevisiae)*	*UPRT*	7.18	7.16	8.67	6.66E-04	0.004762399
8149165	349196		*LOC349196*	9.76	8.70	7.10	6.66E-04	0.004762399
8074748	375133, 728233, 5297	*phosphatidylinositol 4-kinase, catalytic, alpha pseudogene 2 | phosphatidylinositol 4-kinase, catalytic, alpha pseudogene 1*	*PI4KAP2|PI4KAP1*	10.84	10.78	9.78	6.67E-04	0.004762399
8149413	10395	*deleted in liver cancer 1*	*DLC1*	7.76	7.41	6.24	6.67E-04	0.004763432
8107576	94033	*ferritin mitochondrial*	*FTMT*	5.72	5.90	5.31	6.68E-04	0.004766083
7934133	5464	*pyrophosphatase (inorganic) 1*	*PPA1*	8.72	8.99	9.90	6.70E-04	0.004780146
8098995	10608	*MAX dimerization protein 4*	*MXD4*	9.85	9.58	7.84	6.70E-04	0.004780146
8049919	728294	*D-2-hydroxyglutarate dehydrogenase*	*D2HGDH*	8.39	8.35	7.40	6.70E-04	0.004780146
8149167	349196		*LOC349196*	9.76	8.70	7.10	6.72E-04	0.004789482
7936529	57698		*KIAA1598*	7.38	7.13	9.56	6.73E-04	0.004794903
8172220	4693	*Norrie disease (pseudoglioma)*	*NDP*	6.10	6.10	5.77	6.73E-04	0.004796101
8009517	6662	*SRY (sex determining region Y)-box 9*	*SOX9*	8.58	8.99	8.29	6.74E-04	0.004796101
8155569	100131997, 100289124	*family with sequence similarity 27, member E3 | family with sequence similarity 27, member E2*	*FAM27E3|FAM27E2*	7.77	7.94	7.03	6.73E-04	0.004796101
8108422				5.71	5.64	7.08	6.73E-04	0.004796101
8144993				6.09	6.21	5.61	6.73E-04	0.004796101
8160782				6.94	7.09	6.12	6.74E-04	0.004796101
8100478	132949	*aminoadipate-semialdehyde dehydrogenase*	*AASDH*	7.13	6.96	8.32	6.74E-04	0.004796166

7993622	162073	*inositol 1,4,5-triphosphate receptor interacting protein-like 2*	*ITPRIPL2*	8.66	9.04	6.96	6.74E-04	0.004796166
8178322	6613, 728825	*SMT3 suppressor of mif two 3 homolog 2 (S. cerevisiae) | SMT3 suppressor of mif two 3 homolog 2 (S. cerevisiae) pseudogene*	*SUMO2|LOC728825*	10.75	10.79	11.88	6.75E-04	0.004798481
7910915	1131	*cholinergic receptor, muscarinic 3*	*CHRM3*	5.62	5.63	6.64	6.75E-04	0.004800302
8069450	3275	*protein arginine methyltransferase 2*	*PRMT2*	8.10	8.17	7.39	6.75E-04	0.004800302
7939877	403253	*olfactory receptor, family 4, subfamily A, member 47*	*OR4A47*	4.78	4.87	4.62	6.75E-04	0.004800302
8021440				6.09	6.04	5.60	6.75E-04	0.004800302
7927099	84856		*LOC84856*	9.21	8.34	7.44	6.76E-04	0.004802959
8018315	6613, 728825	*SMT3 suppressor of mif two 3 homolog 2 (S. cerevisiae) | SMT3 suppressor of mif two 3 homolog 2 (S. cerevisiae) pseudogene*	*SUMO2|LOC728825*	10.91	10.93	12.04	6.77E-04	0.004806948
7905428	7286	*tuftelin 1*	*TUFT1*	8.81	9.69	8.68	6.77E-04	0.004808256
8116070	9260	*PDZ and LIM domain 7 (enigma)*	*PDLIM7*	10.74	11.11	8.63	6.77E-04	0.004808256
8098782	10815	*complexin 1*	*CPLX1*	7.74	7.70	7.23	6.78E-04	0.004808256
7928401	118487	*coiled-coil-helix-coiled-coil-helix domain containing 1*	*CHCHD1*	7.95	8.09	9.24	6.77E-04	0.004808256
8116585				8.55	8.63	7.78	6.78E-04	0.004808256
8079224	91392	*zinc finger protein 502*	*ZNF502*	6.29	6.00	7.31	6.79E-04	0.004816107
8140709	222223	*KIAA1324-like*	*KIAA1324L*	6.71	7.00	9.82	6.79E-04	0.004816107
8123246	6581	*solute carrier family 22 (extraneuronal monoamine transporter), member 3*	*SLC22A3*	5.97	6.10	7.10	6.80E-04	0.004821498
8145083				6.29	6.46	5.85	6.80E-04	0.004822737
8084524	2049	*EPH receptor B3*	*EPHB3*	8.58	8.68	6.47	6.81E-04	0.004827221
8132998				12.06	12.01	11.17	6.82E-04	0.004829492
7953735	349196		*LOC349196*	9.73	8.82	7.19	6.83E-04	0.004834055
8110932	9037	*sema domain, seven thrombospondin repeats (type 1 and type 1-like), transmembrane domain (TM) and short cytoplasmic domain, (semaphorin) 5A*	*SEMA5A*	11.57	10.29	8.15	6.83E-04	0.004835128
7994858	78994	*proline rich 14*	*PRR14*	9.61	9.50	7.87	6.83E-04	0.004835128

8071444	150209	*apoptosis-inducing factor, mitochondrion-associated, 3*	*AIFM3*	7.13	7.09	6.62	6.83E-04	0.004835216
8084496	8646	*chordin*	*CHRD*	7.55	7.62	6.79	6.84E-04	0.004837713
7928408	23053		*KIAA0913*	8.69	8.66	7.72	6.84E-04	0.004837713
8007212	6776	*signal transducer and activator of transcription 5A*	*STAT5A*	8.82	8.15	7.15	6.85E-04	0.00484472
7965941	83468	*glycosyltransferase 8 domain containing 2*	*GLT8D2*	11.26	11.05	8.25	6.86E-04	0.004847328
8019367	5986		*RFNG*	9.63	9.75	8.16	6.86E-04	0.004852043
8119515	2978	*guanylate cyclase activator 1A (retina)*	*GUCA1A*	6.87	6.68	7.75	6.87E-04	0.004855027
8045808				5.43	5.44	5.01	6.87E-04	0.004855027
8149271				6.16	6.43	5.85	6.87E-04	0.004855027
8109484	285643, 24137	*kinesin family member 4B | kinesin family member 4A*	*KIF4B|KIF 4A*	5.84	5.93	7.01	6.88E-04	0.00486138
7978285	196883	*adenylate cyclase 4*	*ADCY4*	8.63	7.60	6.45	6.89E-04	0.004862108
8048146	7520	*X-ray repair complementing defective repair in Chinese hamster cells 5 (double-strand-break rejoining)*	*XRCC5*	10.07	10.15	11.84	6.89E-04	0.004864757
8058695	580	*BRCA1 associated RING domain 1*	*BARD1*	7.60	7.54	8.67	6.91E-04	0.004877104
7922414	692196, 60674	*small nucleolar RNA, C/D box 76 | growth arrest-specific 5 (non-protein coding)*	*SNORD76| GAS5*	9.56	9.79	11.46	6.91E-04	0.004877104
7928291	9469	*carbohydrate (chondroitin 6) sulfotransferase 3*	*CHST3*	9.29	9.31	7.71	6.93E-04	0.004884675
7923991	5362	*plexin A2*	*PLXNA2*	7.51	7.56	6.42	6.94E-04	0.004892774
7896746				10.87	10.76	9.88	6.94E-04	0.0049
8074274	57553	*microtubule associated monoxygenase, calponin and LIM domain containing 3*	*MICAL3*	8.12	8.08	7.27	6.94E-04	0.004893649
7914938				7.41	7.39	5.59	6.95E-04	0.004895231
8090690	131034	*copine IV*	*CPNE4*	6.11	6.24	7.63	6.95E-04	0.004895487
7974190	131118	*DnaJ (Hsp40) homolog, subfamily C, member 19*	*DNAJC19*	9.11	9.19	10.12	6.95E-04	0.004897004
8006602	6351	*chemokine (C-C motif) ligand 4*	*CCL4*	5.78	5.39	4.95	6.96E-04	0.00489886
8159566	11253	*mannosidase, alpha, class 1B, member 1*	*MAN1B1*	9.91	9.89	8.29	6.96E-04	0.00489886
7997765	84627	*zinc finger protein 469*	*ZNF469*	8.82	8.71	7.58	6.96E-04	0.00489886
7942061				6.09	6.06	9.15	6.96E-04	0.00489886

8012304	58485	*trafficking protein particle complex 1*	*TRAPPC1*	12.52	12.50	11.24	6.97E-04	0.004900815
7950726	220382	*family with sequence similarity 181, member B*	*FAM181B*	8.34	8.32	7.72	6.97E-04	0.004900815
8166094				5.29	5.24	5.00	6.97E-04	0.004900815
8005777	440419	*TBC1 domain family, member 3H pseudogene*	*LOC44041 9*	5.99	5.98	5.53	6.98E-04	0.004909263
7950042	22941	*SH3 and multiple ankyrin repeat domains 2*	*SHANK2*	7.24	7.16	8.70	6.99E-04	0.004911588
8098307	442117	*UDP-N-acetyl-alpha-D-galactosamine:polypeptide N-acetylgalactosaminyltransferase-like 6*	*GALNTL6*	5.88	5.79	5.54	6.99E-04	0.004911588
7929664	112817, 55361	*dihydrodipicolinate synthase-like, mitochondrial | phosphatidylinositol 4-kinase type 2 alpha*	*DHDPSL| PI4K2A*	7.12	7.11	6.39	6.99E-04	0.004911588
7981737				7.88	7.88	7.42	6.99E-04	0.004911588
8047778	26798	*small nucleolar RNA, C/D box 51*	*SNORD51*	5.68	5.65	7.05	7.00E-04	0.004912047
8018786	439921	*matrix-remodelling associated 7*	*MXRA7*	7.76	8.27	6.28	7.00E-04	0.004913513
8097262	166378	*spermatogenesis associated 5*	*SPATA5*	7.39	7.55	9.33	7.00E-04	0.004914333
7989037	9236	*cell cycle progression 1*	*CCPG1*	7.49	8.02	6.23	7.01E-04	0.004915352
8085116	9695	*ER degradation enhancer, mannosidase alpha-like 1*	*EDEM1*	6.92	7.00	6.28	7.01E-04	0.004915975
8094144	81622, 729196	*unc-93 homolog B1 (C. elegans) | unc-93 homolog B5 (C. elegans)*	*UNC93B1| UNC93B5*	8.91	8.93	8.03	7.02E-04	0.004919637
7963157	29127	*Rac GTPase activating protein 1*	*RACGAP1*	7.84	8.21	9.81	7.02E-04	0.004923625
7909285	5208	*6-phosphofructo-2-kinase/fructose-2,6-biphosphatase 2*	*PFKFB2*	6.27	6.18	7.93	7.03E-04	0.004925357
8135763	51384	*wingless-type MMTV integration site family, member 16*	*WNT16*	7.73	6.63	6.13	7.03E-04	0.004925357
8050695	51639	*splicing factor 3B, 14 kDa subunit*	*SF3B14*	9.95	10.23	11.03	7.03E-04	0.004927016
8038899	2357	*formyl peptide receptor 1*	*FPR1*	6.28	6.64	5.69	7.04E-04	0.004928517
7933359	5949	*retinol binding protein 3, interstitial*	*RBP3*	6.51	6.65	6.06	7.04E-04	0.004928517
7944530	220323		*OAF*	10.12	9.85	8.79	7.04E-04	0.004928517
8035465	51477	*inositol-3-phosphate synthase 1*	*ISYNA1*	8.92	8.55	11.00	7.04E-04	0.004929345
8007127	83900	*keratin associated protein 9-3*	*KRTAP9-3*	6.75	6.70	6.19	7.05E-04	0.004930036
8137962	100129484		*LOC10012 9484*	9.47	9.47	8.45	7.05E-04	0.004930036
8093624	6452	*SH3-domain binding protein 2*	*SH3BP2*	8.15	8.10	7.30	7.06E-04	0.004936211

8008933	54828	*breast carcinoma amplified sequence 3*	*BCAS3*	10.10	9.83	9.26	7.06E-04	0.004936612
8173269	81887	*LAS1-like (S. cerevisiae)*	*LAS1L*	8.59	8.72	10.36	7.07E-04	0.004938982
7920278	6274	*S100 calcium binding protein A3*	*S100A3*	8.52	8.88	5.70	7.07E-04	0.004940053
7924058	3664	*interferon regulatory factor 6*	*IRF6*	6.93	6.75	8.27	7.08E-04	0.004942932
8041467	5212	*vitrin*	*VIT*	10.45	7.56	5.75	7.08E-04	0.004942932
8108435	7322	*ubiquitin-conjugating enzyme E2D 2 (UBC4/5 homolog, yeast)*	*UBE2D2*	10.97	11.04	11.40	7.09E-04	0.004942932
8088142	55349	*choline dehydrogenase*	*CHDH*	6.88	6.88	7.79	7.08E-04	0.004942932
8121578	221294	*5'-nucleotidase domain containing 1*	*NT5DC1*	8.22	7.64	9.76	7.08E-04	0.004942932
7981724	3495, 3500	*immunoglobulin heavy constant delta | immunoglobulin heavy constant gamma 1 (G1m marker)*	*IGHD|IGHG1*	7.22	7.33	6.59	7.08E-04	0.004942932
7937228	503542, 92170	*shadow of prion protein homolog (zebrafish) | mitochondrial GTPase 1 homolog (S. cerevisiae)*	*SPRN|MTG1*	7.75	7.82	6.91	7.08E-04	0.004942932
7942914	8726	*embryonic ectoderm development*	*EED*	7.72	7.50	8.69	7.10E-04	0.004949604
7967900	7750	*zinc finger, MYM-type 2*	*ZMYM2*	9.57	9.34	11.70	7.10E-04	0.004951291
8146756	79848	*centrosome and spindle pole associated protein 1*	*CSPP1*	6.88	6.43	7.83	7.10E-04	0.004951291
8104314	79192	*iroquois homeobox 1*	*IRX1*	7.88	8.71	7.23	7.11E-04	0.004954485
8071997	1415	*crystallin, beta B2*	*CRYBB2*	6.98	6.98	6.54	7.11E-04	0.004956268
8097461	25819	*CCR4 carbon catabolite repression 4-like (S. cerevisiae)*	*CCRN4L*	8.51	8.59	9.69	7.11E-04	0.004956268
8137826	29960	*FtsJ homolog 2 (E. coli)*	*FTSJ2*	8.46	8.71	9.40	7.12E-04	0.004958729
8008664	8165	*A kinase (PRKA) anchor protein 1*	*AKAP1*	8.26	8.33	9.41	7.13E-04	0.00496284
8133202	55253	*tRNA-yW synthesizing protein 1 homolog (S. cerevisiae)*	*TYW1*	6.86	7.07	7.58	7.13E-04	0.00496284
7918203	148545, 653149, 285622	*neuroblastoma breakpoint family, member 4 | neuroblastoma breakpoint family, member 6 | neuroblastoma breakpoint family, member 22 (pseudogene)*	*NBPF4|NBPF6|NBPF22P*	6.44	6.36	6.01	7.13E-04	0.004964522
8164607	23048	*formin binding protein 1*	*FNBP1*	10.00	9.79	8.82	7.15E-04	0.004970603
8095539	57050		*UTP3*	6.74	6.98	8.51	7.14E-04	0.004970603

8135069	5054	*serpin peptidase inhibitor, clade E (nexin, plasminogen activator inhibitor type 1), member 1*	*SERPINE1*	10.59	12.71	8.31	7.16E-04	0.004977841
8118324	717, 629	*complement component 2 | complement factor B*	*C2|CFB*	7.82	7.04	5.94	7.16E-04	0.00497885
7990736	11173	*ADAM metallopeptidase with thrombospondin type 1 motif, 7*	*ADAMTS7*	9.90	9.26	8.48	7.16E-04	0.004979208
7912692	27129	*heat shock 27kDa protein family, member 7 (cardiovascular)*	*HSPB7*	8.94	10.25	7.01	7.17E-04	0.004984577
7927425	57705	*WDFY family member 4*	*WDFY4*	6.81	6.81	6.39	7.18E-04	0.004984767
8066820				6.51	6.30	6.07	7.18E-04	0.004984767
7982935				9.12	9.70	10.23	7.18E-04	0.004985853
7900609	114625	*erythroblast membrane-associated protein (Scianna blood group)*	*ERMAP*	8.79	8.77	6.89	7.19E-04	0.004993669
7937518	7106	*tetraspanin 4*	*TSPAN4*	11.39	11.38	9.83	7.21E-04	0.005006295
8085984	114884	*oxysterol binding protein-like 10*	*OSBPL10*	9.24	8.04	11.56	7.22E-04	0.005007513
7915529	81888, 23334	*hydroxypyruvate isomerase (putative) | KIAA0467*	*HYI|KIAA0467*	10.04	10.04	8.53	7.22E-04	0.00500899
8153071	51059	*family with sequence similarity 135, member B*	*FAM135B*	5.72	5.72	5.45	7.22E-04	0.005009192
8031906				6.82	6.39	6.12	7.22E-04	0.005009901
8116297	3187	*heterogeneous nuclear ribonucleoprotein H1 (H)*	*HNRNPH1*	8.46	8.47	10.73	7.23E-04	0.005014421
8036304	57677	*zinc finger protein 14 homolog (mouse)*	*ZFP14*	5.89	5.54	7.25	7.24E-04	0.005015092
7925480	2271	*fumarate hydratase*	*FH*	8.19	7.83	8.84	7.25E-04	0.005024289
7964687	115749	*chromosome 12 open reading frame 56*	*C12orf56*	5.06	5.14	6.38	7.25E-04	0.005024289
8018982	124583	*calcium activated nucleotidase 1*	*CANT1*	10.07	10.01	8.50	7.26E-04	0.005024289
8071554	164592	*coiled-coil domain containing 116*	*CCDC116*	7.38	7.32	6.74	7.25E-04	0.005024289
8167603	1184	*chloride channel 5*	*CLCN5*	6.64	6.55	7.78	7.27E-04	0.005030035
8071717	266747	*ral guanine nucleotide dissociation stimulator-like 4*	*RGL4*	6.83	6.88	6.29	7.27E-04	0.005031001
7968333	10208	*ubiquitin specific peptidase like 1*	*USPL1*	7.71	7.82	9.32	7.28E-04	0.005032763
8102368	63973	*neurogenin 2*	*NEUROG2*	6.19	6.19	5.65	7.28E-04	0.005032763
8047379				7.46	7.66	6.65	7.28E-04	0.005032763
8117739				8.11	8.08	7.51	7.28E-04	0.005032763

8168264	54413	*neuroligin 3*	*NLGN3*	7.98	7.52	8.91	7.29E-04	0.005037352
8109629	2172	*fatty acid binding protein 6, ileal*	*FABP6*	7.22	7.07	7.89	7.29E-04	0.005038797
8048340	9125	*RCD1 required for cell differentiation1 homolog (S. pombe)*	*RQCD1*	10.70	10.64	11.50	7.29E-04	0.005040616
7924558	4931	*nuclear VCP-like*	*NVL*	6.90	6.98	8.65	7.31E-04	0.005047139
7973036	10038	*poly (ADP-ribose) polymerase 2*	*PARP2*	7.50	7.88	9.42	7.31E-04	0.005047139
8149356	377630, 391627, 401447, 645836, 392188	*ubiquitin specific peptidase 17-like 2 | ubiquitin specific peptidase 17 | ubiquitin specific peptidase 17-like 1 (pseudogene) | ubiquitin specific peptidase 17-like 3 | ubiquitin specific peptidase 17-like 8*	*USP17L2|USP17L1P |USP17L3|USP17L8*	7.33	7.24	6.75	7.31E-04	0.005048658
8169210	9075, 92129	*claudin 2 | ripply1 homolog (zebrafish)*	*CLDN2|RIPPLY1*	6.13	6.17	5.82	7.31E-04	0.005049472
8151993	1345	*cytochrome c oxidase subunit VIc*	*COX6C*	8.71	8.56	10.03	7.33E-04	0.005050686
8165735	1438	*colony stimulating factor 2 receptor, alpha, low-affinity (granulocyte-macrophage)*	*CSF2RA*	6.27	6.43	6.80	7.33E-04	0.005050686
8038993	7576	*zinc finger protein 28*	*ZNF28*	6.95	6.89	8.97	7.33E-04	0.005050686
8059712	25826	*small nucleolar RNA, C/D box 82*	*SNORD82*	8.49	8.84	8.28	7.32E-04	0.005050686
7990191	60677	*CUGBP, Elav-like family member 6*	*CELF6*	7.13	7.20	6.60	7.32E-04	0.005050686
8167930	158584	*fatty acid amide hydrolase 2*	*FAAH2*	5.67	5.63	6.72	7.32E-04	0.005050686
8001547	51090	*plasmolipin*	*PLLP*	7.23	7.25	7.72	7.34E-04	0.005060415
8112285	1161	*excision repair cross-complementing rodent repair deficiency, complementation group 8*	*ERCC8*	6.29	6.32	7.26	7.35E-04	0.005061739
8065730	8894	*eukaryotic translation initiation factor 2, subunit 2 beta, 38kDa*	*EIF2S2*	9.91	10.11	11.35	7.35E-04	0.005061739
7971550	29079	*mediator complex subunit 4*	*MED4*	7.23	7.29	8.88	7.35E-04	0.005061739
8176494	728137, 7258, 64591, 728395, 728403	*testis specific protein, Y-linked 3 | testis specific protein, Y-linked 1 | testis specific protein, Y-linked 2 | testis specific protein, Y-linked 4 | testis specific protein, Y-linked 8*	*TSPY3|TSPY1|TSPY2|TSPY4|TSPY8*	6.11	6.10	5.81	7.35E-04	0.005061739
8011193	84981, 407004	*chromosome 17 open reading frame 91 | microRNA 22*	*C17orf91|MIR22*	8.41	8.78	7.22	7.36E-04	0.005061739

8010084				7.58	7.60	6.95	7.35E-04	0.005061739
8107691	51808	*phosphorylated adaptor for RNA export*	*PHAX*	7.72	8.03	9.51	7.36E-04	0.00506376
8116831	7020, 100130275	*transcription factor AP-2 alpha (activating enhancer binding protein 2 alpha) | hypothetical LOC100130275*	*TFAP2A|LOC100130275*	8.48	7.37	6.08	7.36E-04	0.005064273
8029340	7711	*zinc finger protein 155*	*ZNF155*	6.28	6.40	7.08	7.37E-04	0.005068158
8174889	286423	*mitochondrial ribosome recycling factor pseudogene 1*	*MRRFP1*	6.20	6.54	6.85	7.37E-04	0.005070373
8096753	3033	*hydroxyacyl-CoA dehydrogenase*	*HADH*	8.80	8.62	9.68	7.38E-04	0.005072199
8048468	79137	*family with sequence similarity 134, member A*	*FAM134A*	9.48	9.50	7.97	7.38E-04	0.005072199
8162934	138799	*olfactory receptor, family 13, subfamily C, member 5*	*OR13C5*	6.09	5.78	5.21	7.39E-04	0.005074705
8041422	25780	*RAS guanyl releasing protein 3 (calcium and DAG-regulated)*	*RASGRP3*	6.27	5.73	5.06	7.39E-04	0.005076217
7939068	6230	*ribosomal protein S25*	*RPS25*	11.18	11.35	11.97	7.40E-04	0.00508029
7945896	57053	*cholinergic receptor, nicotinic, alpha 10*	*CHRNA10*	6.95	6.99	6.36	7.40E-04	0.00508029
8015262	3884	*keratin 33B*	*KRT33B*	7.06	7.20	6.32	7.41E-04	0.005081884
7926679	56243		*KIAA1217*	6.52	7.10	7.78	7.41E-04	0.005081884
8082767	66000	*transmembrane protein 108*	*TMEM108*	5.73	5.79	7.61	7.41E-04	0.005081884
7981909				8.16	8.33	7.44	7.41E-04	0.005081884
7929344	338557	*G protein-coupled receptor 120*	*GPR120*	6.19	6.26	5.76	7.41E-04	0.005082305
8136940	285966, 154761	*family with sequence similarity 115, member C | hypothetical LOC154761*	*FAM115C|LOC154761*	8.17	8.46	6.84	7.42E-04	0.005084319
7923528	4656	*myogenin (myogenic factor 4)*	*MYOG*	7.65	7.59	7.02	7.42E-04	0.005086637
8101237	152559	*progestin and adipoQ receptor family member III*	*PAQR3*	6.40	6.16	7.63	7.42E-04	0.005086637
8001108				7.93	8.07	7.60	7.42E-04	0.005086637
8078300	54995	*3-oxoacyl-ACP synthase, mitochondrial*	*OXSM*	7.63	7.84	7.03	7.43E-04	0.00508666
8084630	344887	*NmrA-like family domain containing 1 pseudogene*	*LOC344887*	7.93	7.21	5.78	7.43E-04	0.005089465
8117746	692092	*small nucleolar RNA, C/D box 32B*	*SNORD32B*	7.31	7.59	6.61	7.44E-04	0.005092684
8040712	1058	*centromere protein A*	*CENPA*	8.32	8.22	10.18	7.44E-04	0.005094994

8153363	1584	*cytochrome P450, family 11, subfamily B, polypeptide 1*	*CYP11B1*	7.16	7.19	6.53	7.45E-04	0.005096189
8050071	55256	*acireductone dioxygenase 1*	*ADI1*	10.94	10.76	9.64	7.45E-04	0.005096189
8031962	55663	*zinc finger protein 446*	*ZNF446*	8.04	8.04	7.13	7.45E-04	0.005096189
7939620	120071	*glycosyltransferase-like 1B*	*GYLTL1B*	7.50	7.55	9.29	7.46E-04	0.005101444
8162394	54829	*asporin*	*ASPN*	10.11	7.05	4.97	7.47E-04	0.00510335
8095080	5156	*platelet-derived growth factor receptor, alpha polypeptide*	*PDGFRA*	10.67	10.46	6.93	7.47E-04	0.0051049
7989670	348093	*RNA binding protein with multiple splicing 2*	*RBPMS2*	9.03	8.66	12.03	7.47E-04	0.0051049
8001800	1009	*cadherin 11, type 2, OB-cadherin (osteoblast)*	*CDH11*	11.12	11.62	7.83	7.48E-04	0.005106899
8088745	23150	*FERM domain containing 4B*	*FRMD4B*	5.75	5.57	6.48	7.48E-04	0.005106899
7922328	406977	*microRNA 199a-2*	*MIR199A2*	9.51	9.46	6.61	7.48E-04	0.005107657
8009096	1636	*angiotensin I converting enzyme (peptidyl-dipeptidase A) 1*	*ACE*	8.11	7.54	6.82	7.49E-04	0.005110453
8072122				7.83	7.94	8.64	7.49E-04	0.00511107
8157103				6.00	6.11	5.60	7.49E-04	0.00511107
8039484	3589	*interleukin 11*	*IL11*	7.94	8.10	7.07	7.49E-04	0.005112067
8154563	340485	*alkaline ceramidase 2*	*ACER2*	6.63	6.66	7.62	7.50E-04	0.005116112
7982531	8125, 723972	*acidic (leucine-rich) nuclear phosphoprotein 32 family, member A | hepatopoietin PCn127*	*ANP32A|LOC723972*	8.76	9.10	11.61	7.50E-04	0.005116112
7915504	64834	*elongation of very long chain fatty acids (FEN1/Elo2, SUR4/Elo3, yeast)-like 1*	*ELOVL1*	12.12	12.19	10.69	7.52E-04	0.005123071
8045182	26469	*protein tyrosine phosphatase, non-receptor type 18 (brain-derived)*	*PTPN18*	8.23	8.26	6.91	7.52E-04	0.005127665
8072004	91353, 3543	*immunoglobulin lambda-like polypeptide 3, pseudogene | immunoglobulin lambda-like polypeptide 1*	*IGLL3P|IGLL1*	7.81	7.89	7.37	7.53E-04	0.005130405
8042326	23177	*centrosomal protein 68kDa*	*CEP68*	8.25	8.08	8.66	7.53E-04	0.005130611
8110618	10776	*cAMP-regulated phosphoprotein, 19kDa*	*ARPP19*	7.48	8.19	10.28	7.54E-04	0.005134129
8076826	348645	*chromosome 22 open reading frame 34*	*C22orf34*	6.62	6.75	6.27	7.54E-04	0.005134129
7901967				7.10	7.41	6.24	7.54E-04	0.005134161
8014066	2123	*ecotropic viral integration site 2A*	*EVI2A*	5.42	6.18	5.09	7.55E-04	0.005135966

7993335				4.93	5.18	4.58	7.55E-04	0.005135966
8018972	7077	*TIMP metallopeptidase inhibitor 2*	*TIMP2*	8.37	8.18	7.07	7.56E-04	0.005140327
7991904	9091	*phosphatidylinositol glycan anchor biosynthesis, class Q*	*PIGQ*	8.80	8.89	7.34	7.56E-04	0.005143805
7953520	10233	*leucine rich repeat containing 23*	*LRRC23*	7.95	8.08	6.97	7.57E-04	0.00514602
8167654	23708	*G1 to S phase transition 2*	*GSPT2*	8.57	8.45	9.57	7.59E-04	0.005159227
8085293	51738	*ghrelin/obestatin prepropeptide*	*GHRL*	7.17	7.24	6.59	7.60E-04	0.005161884
7909510	55733	*hedgehog acyltransferase*	*HHAT*	7.48	7.38	6.20	7.60E-04	0.005163847
8158890	84628	*netrin G2*	*NTNG2*	7.81	7.83	6.96	7.60E-04	0.005163847
8156521	406888	*microRNA let-7f-1*	*MIRLET7F 1*	5.57	5.59	7.55	7.60E-04	0.005163847
7976571	51527	*chromosome 14 open reading frame 129*	*C14orf129*	7.84	7.96	9.53	7.61E-04	0.005165772
8069574	54149	*chromosome 21 open reading frame 91*	*C21orf91*	6.06	6.18	8.16	7.61E-04	0.005165772
7949067	572	*BCL2-associated agonist of cell death*	*BAD*	9.22	9.20	7.84	7.62E-04	0.005167847
8147548	10940	*processing of precursor 1, ribonuclease P/MRP subunit (S. cerevisiae)*	*POP1*	7.05	7.28	8.40	7.62E-04	0.005167847
8115580	114825	*PWWP domain containing 2A*	*PWWP2A*	8.33	8.30	9.47	7.62E-04	0.005167847
7971537				6.26	6.22	5.58	7.63E-04	0.005176434
8015991	6521	*solute carrier family 4, anion exchanger, member 1 (erythrocyte membrane protein band 3, Diego blood group)*	*SLC4A1*	6.46	6.55	5.95	7.64E-04	0.005179733
7931239	26098	*chromosome 10 open reading frame 137*	*C10orf137*	7.88	7.60	9.62	7.64E-04	0.005180237
7899851	113451	*arginine decarboxylase*	*ADC*	7.38	7.42	6.49	7.65E-04	0.005182272
7968272				9.21	9.33	8.03	7.65E-04	0.005182272
8003068	10200	*M-phase phosphoprotein 6*	*MPHOSP H6*	7.64	6.59	8.57	7.65E-04	0.005183098
8120698	25821	*mitochondrial translation optimization 1 homolog (S. cerevisiae)*	*MTO1*	8.23	8.33	9.20	7.66E-04	0.005183098
7938748				8.17	8.57	7.40	7.66E-04	0.005183098
8022531	4864	*Niemann-Pick disease, type C1*	*NPC1*	9.34	9.89	9.64	7.66E-04	0.005185376
8089527	64422		*ATG3*	10.01	10.16	11.05	7.66E-04	0.005185376

8031475	147744	*transmembrane protein 190*	*TMEM190*	7.25	7.35	6.72	7.66E-04	0.005185376
8142019	5001	*origin recognition complex, subunit 5*	*ORC5*	7.97	8.05	9.50	7.67E-04	0.00518626
8042335	7417	*voltage-dependent anion channel 2*	*VDAC2*	11.89	11.96	12.58	7.67E-04	0.00518626
8005235	23164	*myosin phosphatase Rho interacting protein*	*MPRIP*	8.87	9.09	8.13	7.67E-04	0.00518626
7923798	64710	*nuclear casein kinase and cyclin-dependent kinase substrate 1*	*NUCKS1*	10.46	10.37	12.51	7.67E-04	0.00518626
7914898	79729, 55194	*chromosome 1 open reading frame 113 | family with sequence similarity 176, member B*	*C1orf113|FAM176B*	9.65	9.74	8.31	7.68E-04	0.005189087
7968323	440131		*LOC440131*	6.20	6.24	5.86	7.68E-04	0.005191303
7914194	22826	*DnaJ (Hsp40) homolog, subfamily C, member 8*	*DNAJC8*	9.24	9.49	10.05	7.69E-04	0.005191405
7988426	7782	*solute carrier family 30 (zinc transporter), member 4*	*SLC30A4*	8.45	8.45	6.19	7.70E-04	0.005197611
7979400	145407	*chromosome 14 open reading frame 37*	*C14orf37*	6.45	6.86	8.07	7.70E-04	0.005201075
8065018	55617	*taspase, threonine aspartase, 1*	*TASP1*	6.67	6.83	7.41	7.71E-04	0.005201658
8062427	128434	*V-set and transmembrane domain containing 2 like*	*VSTM2L*	8.27	8.30	7.49	7.71E-04	0.005202917
7954460	144363	*LYR motif containing 5*	*LYRM5*	10.00	8.77	8.18	7.72E-04	0.005204716
8022974				4.87	4.87	6.15	7.71E-04	0.005204716
8139758	51142	*coiled-coil-helix-coiled-coil-helix domain containing 2*	*CHCHD2*	10.20	10.29	7.62	7.72E-04	0.005205263
8018482	23558	*WW domain binding protein 2*	*WBP2*	11.06	10.92	9.76	7.73E-04	0.005212889
8161270	253650	*ankyrin repeat domain 18A*	*ANKRD18A*	5.63	5.73	6.92	7.73E-04	0.005212889
7907537	27101	*calcyclin binding protein*	*CACYBP*	7.92	8.06	8.98	7.75E-04	0.005221851
8021946	81035	*collectin sub-family member 12*	*COLEC12*	10.62	9.86	7.37	7.75E-04	0.005222691
8060101	100130449	*similar to hCG1777210*	*PP14571*	7.11	7.11	6.65	7.76E-04	0.005223676
7952739	29068	*zinc finger and BTB domain containing 44*	*ZBTB44*	8.87	8.97	10.25	7.76E-04	0.005225277
7941822	338692	*ankyrin repeat domain 13 family, member D*	*ANKRD13D*	8.18	8.30	7.28	7.76E-04	0.005225277
7982269				5.98	5.85	7.63	7.76E-04	0.005225277
8049509	51052	*prolactin releasing hormone*	*PRLH*	8.57	8.76	7.76	7.77E-04	0.005230415

8161533	653404, 286380, 100036519, 349334, 2298, 200350, 653427	*forkhead box D4-like 6 | forkhead box D4-like 3 | forkhead box D4-like 2 | forkhead box D4-like 4 | forkhead box D4 | forkhead box D4-like 1 | forkhead box D4-like 5*	*FOXD4L6| FOXD4L3| FOXD4L2| FOXD4L4| FOXD4L1| FOXD4L5*	6.85	6.88	6.50	7.77E-04	0.005230415
8108688	56132	*protocadherin beta 3*	*PCDHB3*	6.10	6.22	8.61	7.78E-04	0.005233416
7996185	4324	*matrix metallopeptidase 15 (membrane-inserted)*	*MMP15*	7.12	7.05	8.30	7.78E-04	0.005234555
8101874	124	*alcohol dehydrogenase 1A (class I), alpha polypeptide*	*ADH1A*	7.26	6.60	5.82	7.79E-04	0.005235989
7932000	22944		*KIN*	7.07	6.85	8.32	7.80E-04	0.005241592
8063524				6.54	6.70	6.10	7.81E-04	0.005247709
8120967	4907	*5'-nucleotidase, ecto (CD73)*	*NT5E*	10.02	9.77	5.80	7.82E-04	0.005250146
8024048	5657	*proteinase 3*	*PRTN3*	8.10	8.08	7.43	7.82E-04	0.005250146
8033233	10382	*tubulin, beta 4*	*TUBB4*	7.30	7.59	9.52	7.82E-04	0.005250146
7966202	83892	*potassium channel tetramerisation domain containing 10*	*KCTD10*	10.29	10.15	9.38	7.82E-04	0.005250146
8107909	6583	*solute carrier family 22 (organic cation/ergothioneine transporter), member 4*	*SLC22A4*	7.57	8.46	5.91	7.82E-04	0.00525097
7912672	729614	*hypothetical LOC729614*	*FLJ37453*	6.86	6.84	6.23	7.82E-04	0.005251008
8035040	58525	*widely interspaced zinc finger motifs*	*WIZ*	8.48	8.66	7.28	7.83E-04	0.005252658
7967240	65082	*vacuolar protein sorting 33 homolog A (S. cerevisiae)*	*VPS33A*	7.91	7.88	9.47	7.83E-04	0.005252658
8077914	80746	*tRNA splicing endonuclease 2 homolog (S. cerevisiae)*	*TSEN2*	7.18	7.16	8.50	7.83E-04	0.005252658
7917255	117178	*synovial sarcoma, X breakpoint 2 interacting protein*	*SSX2IP*	6.83	7.03	8.60	7.83E-04	0.005252658
8152865				5.84	5.59	6.90	7.84E-04	0.005252924
7926207	8872	*cell division cycle 123 homolog (S. cerevisiae)*	*CDC123*	9.30	9.36	11.37	7.84E-04	0.005255258
7905406	57530	*cingulin*	*CGN*	6.46	6.62	7.47	7.84E-04	0.005255258
8050350				9.36	9.49	7.77	7.85E-04	0.005256049
8144866	10	*N-acetyltransferase 2 (arylamine N-acetyltransferase)*	*NAT2*	5.14	5.26	5.92	7.85E-04	0.005257352
7958692	79600	*tectonic family member 1*	*TCTN1*	9.40	9.20	7.51	7.85E-04	0.005257352
7912004	387509	*G protein-coupled receptor 153*	*GPR153*	8.99	8.93	7.82	7.86E-04	0.00526057

8124924				5.76	5.73	6.00	7.87E-04	0.005263302
7946354	4004	*LIM domain only 1 (rhombotin 1)*	*LMO1*	6.91	6.95	6.40	7.87E-04	0.005263467
8079229	115560	*zinc finger protein 501*	*ZNF501*	6.48	6.10	7.38	7.87E-04	0.005263467
7939884	57093, 653111, 283257, 283116, 642612	*tripartite motif-containing 49 | tripartite motif-containing 49-like 2 | tripartite motif-containing protein 49B-like | tripartite motif-containing protein LOC642612-like | tripartite motif-containing protein LOC642612*	*TRIM49|TRIM49L2|TRIM49B|LOC283116|LOC642612*	5.41	5.39	6.10	7.87E-04	0.005263467
8030092	653677	*secretory blood group 1*	*SEC1*	7.41	7.42	6.57	7.88E-04	0.005266794
7904883	9557	*chromodomain helicase DNA binding protein 1-like*	*CHD1L*	6.56	7.14	8.25	7.89E-04	0.00527028
7969271	10910	*SGT1, suppressor of G2 allele of SKP1 (S. cerevisiae)*	*SUGT1*	7.96	8.10	9.50	7.89E-04	0.00527028
8087116	440955	*transmembrane protein 89*	*TMEM89*	8.52	8.58	7.91	7.89E-04	0.00527028
8049540	9208	*leucine rich repeat (in FLII) interacting protein 1*	*LRRFIP1*	6.52	6.52	8.05	7.90E-04	0.005279011
8170208				5.59	5.74	5.32	7.91E-04	0.005280775
8170479	203547		*VMA21*	8.28	8.42	9.16	7.92E-04	0.005283982
7939003				4.65	4.65	4.48	7.92E-04	0.005283982
7953211	57103	*chromosome 12 open reading frame 5*	*C12orf5*	6.56	6.94	8.66	7.92E-04	0.005284426
7900001	339488, 5690	*transcription factor AP-2 epsilon (activating enhancer binding protein 2 epsilon) | proteasome (prosome, macropain) subunit, beta type, 2*	*TFAP2E|PSMB2*	8.52	8.56	7.85	7.92E-04	0.005284426
7924888	92815	*histone cluster 3, H2a*	*HIST3H2A*	6.82	6.74	8.02	7.93E-04	0.005285339
8125289	7146, 7148	*tenascin XA pseudogene | tenascin XB*	*TNXA|TNXB*	10.84	9.72	6.74	7.93E-04	0.005285339
7995324				9.42	9.61	8.55	7.93E-04	0.005285339
7913712	10772	*serine/arginine-rich splicing factor 10*	*SRSF10*	7.23	7.27	9.37	7.93E-04	0.005285384
8108134	9879	*DEAD (Asp-Glu-Ala-Asp) box polypeptide 46*	*DDX46*	7.29	6.92	10.06	7.93E-04	0.005286034
8164729	7270	*transcription termination factor, RNA polymerase I*	*TTF1*	6.19	6.25	7.67	7.94E-04	0.00528814
8164373	25792	*CDKN1A interacting zinc finger protein 1*	*CIZ1*	10.90	10.77	9.38	7.95E-04	0.005295104
8072678	3162	*heme oxygenase (decycling) 1*	*HMOX1*	10.53	11.40	8.82	7.96E-04	0.005295845

8073068	27350	*apolipoprotein B mRNA editing enzyme, catalytic polypeptide-like 3C*	*APOBEC3C*	11.41	10.95	9.73	7.96E-04	0.005295845
8164396	406978	*microRNA 199b*	*MIR199B*	6.80	6.71	5.25	7.96E-04	0.005295845
8018791				7.03	6.63	5.47	7.95E-04	0.005295845
8159900	169792	*GLIS family zinc finger 3*	*GLIS3*	8.25	8.34	5.88	7.96E-04	0.005297171
8092523	1962	*enoyl-CoA, hydratase/3-hydroxyacyl CoA dehydrogenase*	*EHHADH*	6.14	6.20	7.40	7.96E-04	0.005298179
8060698	55317	*chromosome 20 open reading frame 29*	*C20orf29*	9.57	9.50	7.69	7.97E-04	0.005299021
7995926	84166	*NLR family, CARD domain containing 5*	*NLRC5*	7.83	7.89	6.49	7.97E-04	0.005299021
8128991	3910	*laminin, alpha 4*	*LAMA4*	10.80	10.11	6.00	7.98E-04	0.005301077
8078227	8850	*K(lysine) acetyltransferase 2B*	*KAT2B*	8.07	8.01	6.80	7.98E-04	0.005301077
8153322	23237	*activity-regulated cytoskeleton-associated protein*	*ARC*	8.09	8.25	7.54	7.98E-04	0.005301077
8130129	348995	*nucleoporin 43kDa*	*NUP43*	8.49	8.84	11.35	7.98E-04	0.005302477
7918424				5.41	5.15	5.30	7.99E-04	0.005307487
7952129	6230	*ribosomal protein S25*	*RPS25*	12.72	12.71	13.15	8.00E-04	0.005311612
7921332	922	*CD5 molecule-like*	*CD5L*	6.46	6.53	6.12	8.01E-04	0.005312495
8120210	3605	*interleukin 17A*	*IL17A*	7.06	6.95	6.43	8.00E-04	0.005312495
8070411	114041	*chromosome 21 open reading frame 88*	*C21orf88*	6.46	6.86	8.84	8.01E-04	0.005312495
8039698	147686	*zinc finger protein 418*	*ZNF418*	6.78	6.58	7.64	8.01E-04	0.005312495
8148461				6.37	6.29	5.86	8.01E-04	0.005312495
8016947	9256	*benzodiazapine receptor (peripheral) associated protein 1*	*BZRAP1*	7.41	7.53	6.86	8.02E-04	0.00531299
8115210	10318	*TNFAIP3 interacting protein 1*	*TNIP1*	9.18	9.18	7.86	8.01E-04	0.00531299
8038824	89790, 89858	*sialic acid binding Ig-like lectin 10 | sialic acid binding Ig-like lectin 12*	*SIGLEC10 |SIGLEC12*	6.59	6.70	6.27	8.02E-04	0.00531299
8149475	29883	*CCR4-NOT transcription complex, subunit 7*	*CNOT7*	10.06	9.98	11.50	8.02E-04	0.005313285
8038261	2997	*glycogen synthase 1 (muscle)*	*GYS1*	10.44	11.25	9.19	8.03E-04	0.005314642
7905171	9129	*PRP3 pre-mRNA processing factor 3 homolog (S. cerevisiae)*	*PRPF3*	7.94	7.98	9.48	8.02E-04	0.005314642
8138485				6.15	6.35	5.72	8.03E-04	0.005314642

7956287	4665	*NGFI-A binding protein 2 (EGR1 binding protein 2)*	*NAB2*	9.38	9.57	8.04	8.03E-04	0.005316138
7969835	5095	*propionyl CoA carboxylase, alpha polypeptide*	*PCCA*	7.60	7.10	9.11	8.03E-04	0.005316138
8051411				6.03	6.05	5.48	8.03E-04	0.005316138
8097600	8467	*SWI/SNF related, matrix associated, actin dependent regulator of chromatin, subfamily a, member 5*	*SMARCA5*	8.96	8.41	10.82	8.04E-04	0.005317534
7935730	55280	*CWF19-like 1, cell cycle control (S. pombe)*	*CWF19L1*	7.97	8.02	9.15	8.04E-04	0.005317534
8027385	342865	*V-set and transmembrane domain containing 2B*	*VSTM2B*	8.58	8.66	7.96	8.04E-04	0.005317534
8058147	53938	*peptidylprolyl isomerase (cyclophilin)-like 3*	*PPIL3*	9.64	9.67	10.43	8.05E-04	0.005318061
8017259	342538	*nascent polypeptide-associated complex alpha subunit 2*	*NACA2*	4.80	4.89	5.23	8.05E-04	0.005318061
8088167	58515	*selenoprotein K*	*SELK*	9.02	9.41	9.76	8.06E-04	0.00532081
7944275	84866	*transmembrane protein 25*	*TMEM25*	8.63	7.88	7.70	8.05E-04	0.00532081
8059081	151295	*solute carrier family 23 (nucleobase transporters), member 3*	*SLC23A3*	6.71	6.71	5.99	8.06E-04	0.00532081
8050232	192668	*cystin 1*	*CYS1*	9.42	9.42	8.58	8.06E-04	0.00532081
8015206	81872, 728279, 85294, 730755	*keratin associated protein 2-1 | keratin associated protein 2-2 | keratin associated protein 2-4 | keratin associated protein 2-4-like*	*KRTAP2-1|KRTAP2-2|KRTAP2-4|LOC730755*	9.91	10.97	8.93	8.06E-04	0.00532081
8117032				5.85	5.84	5.46	8.06E-04	0.00532081
7950447	143570, 254225	*X-ray radiation resistance associated 1 | ring finger protein 169*	*XRRA1|RNF169*	8.22	7.82	7.21	8.07E-04	0.005328147
8005814	51701	*nemo-like kinase*	*NLK*	9.26	8.64	10.77	8.08E-04	0.005328763
8054758	84771, 100288486	*DEAD/H (Asp-Glu-Ala-Asp/His) box polypeptide 11 like 2 | DEAD/H (Asp-Glu-Ala-Asp/His) box polypeptide 11 like 9*	*DDX11L2|DDX11L9*	6.87	6.92	6.12	8.09E-04	0.005336431
8095986	306	*annexin A3*	*ANXA3*	7.87	6.96	10.61	8.11E-04	0.005338556
8119689	5754		*PTK7*	10.30	10.67	8.87	8.10E-04	0.005338556
7987361	54989	*zinc finger protein 770*	*ZNF770*	7.82	7.17	10.14	8.10E-04	0.005338556
8097373	80167	*chromosome 4 open reading frame 29*	*C4orf29*	7.37	7.27	8.91	8.10E-04	0.005338556

8161829	138199	*chromosome 9 open reading frame 41*	*C9orf41*	7.71	7.70	9.78	8.10E-04	0.005338556
8078567				7.27	7.06	6.41	8.10E-04	0.005338556
8066739	100130934	*zinc finger protein 663*	*ZNF663*	5.84	5.87	6.42	8.12E-04	0.005345677
8003639	124997	*WD repeat domain 81*	*WDR81*	8.56	8.74	7.35	8.12E-04	0.005345726
8076298				9.60	9.76	9.37	8.12E-04	0.005345726
8144395	377630, 401447, 645836, 392188, 391627	*ubiquitin specific peptidase 17-like 2 | ubiquitin specific peptidase 17-like 1 (pseudogene) | ubiquitin specific peptidase 17-like 3 | ubiquitin specific peptidase 17-like 8 | ubiquitin specific peptidase 17*	*USP17L2| USP17L1P |USP17L3| USP17L8*	7.37	7.29	6.76	8.12E-04	0.00534625
7914880	1296	*collagen, type VIII, alpha 2*	*COL8A2*	8.56	9.01	7.76	8.13E-04	0.005346394
7981346	5891	*renal tumor antigen*	*RAGE*	7.21	7.15	5.95	8.13E-04	0.005346729
7904364	10885	*WD repeat domain 3*	*WDR3*	6.46	6.47	9.02	8.14E-04	0.005353812
8169624	203427	*solute carrier family 25, member 43*	*SLC25A43*	8.98	9.26	7.77	8.15E-04	0.005355783
8097480	80155	*N(alpha)-acetyltransferase 15, NatA auxiliary subunit*	*NAA15*	7.60	7.27	10.72	8.16E-04	0.005360406
8176544	728137, 7258, 64591, 728395, 728403	*testis specific protein, Y-linked 3 | testis specific protein, Y-linked 1 | testis specific protein, Y-linked 2 | testis specific protein, Y-linked 4 | testis specific protein, Y-linked 8*	*TSPY3|TSPY1|TSPY2|TSPY4|TSPY8*	6.23	6.21	5.92	8.16E-04	0.005360406
7906400	3428	*interferon, gamma-inducible protein 16*	*IFI16*	10.66	10.19	7.09	8.16E-04	0.005361068
7919950	5298	*phosphatidylinositol 4-kinase, catalytic, beta*	*PI4KB*	10.31	10.08	8.72	8.16E-04	0.005361068
8156341	401541, 64768	*centromere protein P | inositol 1,3,4,5,6-pentakisphosphate 2-kinase*	*CENPP|IPPK*	6.64	6.71	7.51	8.17E-04	0.005362073
8150225	80223	*RAB11 family interacting protein 1 (class I)*	*RAB11FIP1*	7.46	7.07	8.44	8.17E-04	0.00536583
8172028				9.62	9.93	9.15	8.18E-04	0.005368127
7905054				5.29	5.16	6.49	8.18E-04	0.005370165
8098500	55602	*CDKN2A interacting protein*	*CDKN2AIP*	7.57	7.61	8.55	8.20E-04	0.005377722
7906154	128229	*chromosome 1 open reading frame 182*	*C1orf182*	5.77	5.76	6.61	8.20E-04	0.005379627
8144705	57604	*chromosome 8 open reading frame 79*	*C8orf79*	7.67	7.07	6.05	8.21E-04	0.005384882

8108927	54439	*RNA binding motif protein 27*	*RBM27*	8.45	8.37	10.40	8.22E-04	0.00538533
8048752	65080	*mitochondrial ribosomal protein L44*	*MRPL44*	7.58	7.70	9.30	8.22E-04	0.00538533
8038989	162966	*zinc finger protein 600*	*ZNF600*	6.44	6.46	7.47	8.22E-04	0.00538533
7913858	164091	*progestin and adipoQ receptor family member VII*	*PAQR7*	7.23	7.13	6.41	8.22E-04	0.00538533
7981951	100033414	*small nucleolar RNA, C/D box 116-2*	*SNORD116-2*	8.02	7.78	9.53	8.22E-04	0.00538533
7938331	7702	*zinc finger protein 143*	*ZNF143*	6.67	6.44	8.56	8.22E-04	0.005385351
8003844	83903	*germ cell associated 2 (haspin)*	*GSG2*	6.68	7.32	9.00	8.23E-04	0.005386611
8144669	2222	*farnesyl-diphosphate farnesyltransferase 1*	*FDFT1*	12.14	11.66	13.01	8.23E-04	0.005388498
8016128	2670	*glial fibrillary acidic protein*	*GFAP*	7.34	7.43	6.90	8.24E-04	0.005394676
8178435	8870	*immediate early response 3*	*IER3*	10.63	11.73	9.12	8.25E-04	0.005395404
8068684	54097	*family with sequence similarity 3, member B*	*FAM3B*	5.58	5.56	6.19	8.26E-04	0.005402941
8177601	64087, 100510706	*methylcrotonoyl-CoA carboxylase 2 (beta) | methylcrotonoyl-CoA carboxylase beta chain, mitochondrial-like*	*MCCC2|LOC100510706*	8.33	8.41	9.99	8.26E-04	0.005403236
8096296	3327	*heat shock protein 90kDa alpha (cytosolic), class B member 3 (pseudogene)*	*HSP90AB3P*	8.49	8.74	10.56	8.27E-04	0.005406683
7916570	115209, 1600	*OMA1 homolog, zinc metallopeptidase (S. cerevisiae) | disabled homolog 1 (Drosophila)*	*OMA1|DAB1*	7.18	6.96	8.21	8.27E-04	0.005408932
7902687	3491	*cysteine-rich, angiogenic inducer, 61*	*CYR61*	11.28	12.23	10.25	8.29E-04	0.005415556
8021635	5055	*serpin peptidase inhibitor, clade B (ovalbumin), member 2*	*SERPINB2*	8.08	10.01	5.77	8.29E-04	0.005415556
8010770	9123	*solute carrier family 16, member 3 (monocarboxylic acid transporter 4)*	*SLC16A3*	9.92	10.43	8.45	8.29E-04	0.005415556
8083630	85476	*G elongation factor, mitochondrial 1*	*GFM1*	6.92	6.70	8.59	8.29E-04	0.005415556
7976836	574453	*microRNA 495*	*MIR495*	5.20	4.91	4.74	8.30E-04	0.00542178
7968252				7.84	7.81	7.15	8.31E-04	0.005425182
8138504	9771	*Rap guanine nucleotide exchange factor (GEF) 5*	*RAPGEF5*	5.83	5.64	7.30	8.33E-04	0.005435967
7920382	200186	*CREB regulated transcription coactivator 2*	*CRTC2*	9.75	9.78	8.21	8.33E-04	0.005436503
7954036	51202, 81575	*DEAD (Asp-Glu-Ala-Asp) box polypeptide 47 | apolipoprotein L domain containing 1*	*DDX47|APOLD1*	9.28	9.61	10.35	8.33E-04	0.005436503

8106068	64087, 100510706	*methylcrotonoyl-CoA carboxylase 2 (beta) | methylcrotonoyl-CoA carboxylase beta chain, mitochondrial-like*	*MCCC2|LOC100510706*	8.33	8.41	9.99	8.33E-04	0.005436503	
7965048	4673	*nucleosome assembly protein 1-like 1*	*NAP1L1*	10.34	10.12	11.87	8.34E-04	0.005437391	
8140319	3092	*huntingtin interacting protein 1*	*HIP1*	7.65	8.58	9.94	8.34E-04	0.005439322	
7933561	8505, 728404	*poly (ADP-ribose) glycohydrolase | ArfGAP with GTPase domain, ankyrin repeat and PH domain 8*	*PARG|AGAP8*	6.85	6.86	8.19	8.34E-04	0.005439322	
8174556	7224	*transient receptor potential cation channel, subfamily C, member 5*	*TRPC5*	5.87	6.41	5.76	8.35E-04	0.005440642	
8149216	349196		*LOC349196*	9.71	8.68	7.06	8.35E-04	0.005440642	
8015208	81872, 728279, 85294, 730755, 85297	*keratin associated protein 2-1 | keratin associated protein 2-2 | keratin associated protein 2-4 | keratin associated protein 2-4-like | keratin associated protein 2.1B*	*KRTAP2-1|KRTAP2-2|KRTAP2-4|LOC730755|KAP2.1B*	9.93	10.98	8.96	8.36E-04	0.005448477	
8109475	29093	*mitochondrial ribosomal protein L22*	*MRPL22*	7.30	7.40	8.29	8.37E-04	0.005448604	
8005951	26808	*small nucleolar RNA, C/D box 42B*	*SNORD42B*	7.59	7.57	9.13	8.37E-04	0.00544897	
8010082	677848	*small nucleolar RNA, C/D box 1A*	*SNORD1A*	5.21	5.29	7.03	8.37E-04	0.005449182	
8054377	2274	*four and a half LIM domains 2*	*FHL2*	10.06	10.30	8.51	8.37E-04	0.005449746	
8119974	2030	*solute carrier family 29 (nucleoside transporters), member 1*	*SLC29A1*	9.48	9.29	11.33	8.38E-04	0.00545131	
8136140	4899	*nuclear respiratory factor 1*	*NRF1*	7.44	7.52	8.00	8.38E-04	0.00545131	
7919166				5.51	5.33	6.70	8.39E-04	0.005455773	
8047815	22868	*FAST kinase domains 2*	*FASTKD2*	7.13	6.98	8.72	8.39E-04	0.005456398	
7949340	116071	*basic leucine zipper transcription factor, ATF-like 2*	*BATF2*	7.87	7.63	6.90	8.39E-04	0.00545693	
8161242	51010	*exosome component 3*	*EXOSC3*	6.86	6.91	8.90	8.40E-04	0.005458084	
8087806	57060	*poly(rC) binding protein 4*	*PCBP4*	9.96	10.01	7.88	8.40E-04	0.005460995	
8130580	677812	*small nucleolar RNA, H/ACA box 29*	*SNORA29*	5.49	5.46	6.17	8.42E-04	0.005468308	

8145829	80139	*zinc finger protein 703*	*ZNF703*	9.26	9.62	7.80	8.42E-04	0.005469894
8016232	201175	*SH3 domain containing 20*	*SH3D20*	7.01	7.06	6.46	8.42E-04	0.005469894
8093314	4710	*NADH dehydrogenase (ubiquinone) 1 beta subcomplex, 4, 15kDa*	*NDUFB4*	10.59	10.48	11.16	8.43E-04	0.005470585
7956631	25895	*family with sequence similarity 119, member B*	*FAM119B*	8.85	9.03	7.71	8.43E-04	0.00547457
8101031	8999	*cyclin-dependent kinase-like 2 (CDC2-related kinase)*	*CDKL2*	5.33	5.38	6.76	8.44E-04	0.005476018
7990417	10066	*secretory carrier membrane protein 2*	*SCAMP2*	12.07	11.95	10.69	8.44E-04	0.005476018
7975167	10243	*gephyrin*	*GPHN*	7.40	7.36	8.27	8.44E-04	0.005476018
7988132	161497	*stereocilin*	*STRC*	6.47	6.57	6.10	8.44E-04	0.005476018
8171418	5277	*phosphatidylinositol glycan anchor biosynthesis, class A*	*PIGA*	6.80	6.87	8.25	8.45E-04	0.005477785
8046555	3233, 3232	*homeobox D4 | homeobox D3*	*HOXD4|HOXD3*	7.64	8.06	7.06	8.45E-04	0.005480042
7951038	677822, 79101	*small nucleolar RNA, H/ACA box 40 | TATA box binding protein (TBP)-associated factor, RNA polymerase I, D, 41kDa*	*SNORA40|TAF1D*	7.87	8.20	9.61	8.46E-04	0.005483443
8112967	153339	*transmembrane protein 167A*	*TMEM167A*	8.79	8.68	10.72	8.48E-04	0.005491087
8077036	414918	*family with sequence similarity 116, member B*	*FAM116B*	8.09	8.20	7.38	8.48E-04	0.005491541
7953765	57494	*ribosomal modification protein rimK-like family member B*	*RIMKLB*	6.88	7.16	9.90	8.48E-04	0.005492457
8061112				6.03	5.98	5.71	8.48E-04	0.005492457
8040045	78989	*collectin sub-family member 11*	*COLEC11*	7.38	7.46	6.99	8.49E-04	0.005495894
8065363	200261		*LOC200261*	6.32	6.18	5.87	8.50E-04	0.005503415
7967727	50614	*UDP-N-acetyl-alpha-D-galactosamine:polypeptide N-acetylgalactosaminyltransferase 9 (GalNAc-T9)*	*GALNT9*	7.33	7.17	6.75	8.52E-04	0.005510565
8161476				10.11	10.04	8.78	8.53E-04	0.005515803
7980765	8111	*G protein-coupled receptor 68*	*GPR68*	6.76	7.24	6.30	8.53E-04	0.005516365
7899519	51441	*YTH domain family, member 2*	*YTHDF2*	8.88	9.05	10.04	8.54E-04	0.005516365
8137584	64434	*nucleolar protein with MIF4G domain 1*	*NOM1*	8.26	8.50	9.23	8.54E-04	0.005516365
8124583	222696	*zinc finger and SCAN domain containing 23*	*ZSCAN23*	5.76	5.67	6.64	8.54E-04	0.005516365

8049598	339768	*espin-like*	*ESPNL*	7.85	7.83	7.12	8.53E-04	0.005516365
8121255				4.66	4.65	4.51	8.54E-04	0.005516365
7989937	197021, 55055	*lactase-like | Zwilch, kinetochore associated, homolog (Drosophila)*	*LCTL|ZWILCH*	6.09	6.63	7.52	8.56E-04	0.005526877
8001615	1258	*cyclic nucleotide gated channel beta 1*	*CNGB1*	7.34	7.39	6.98	8.56E-04	0.005527954
8071234	5413, 2812	*septin 5 | glycoprotein Ib (platelet), beta polypeptide*	*SEPT5|GP1BB*	8.86	8.75	7.86	8.56E-04	0.005527954
7981427	1152	*creatine kinase, brain*	*CKB*	8.86	9.38	11.45	8.58E-04	0.005534692
8030007	2014	*epithelial membrane protein 3*	*EMP3*	12.30	12.47	10.19	8.59E-04	0.005541557
8126839	27242	*tumor necrosis factor receptor superfamily, member 21*	*TNFRSF21*	8.80	8.84	9.89	8.60E-04	0.005548771
8129706	10767	*HBS1-like (S. cerevisiae)*	*HBS1L*	8.79	8.65	10.20	8.60E-04	0.005549888
8132710	80099	*chromosome 7 open reading frame 69*	*C7orf69*	7.13	6.75	5.08	8.61E-04	0.005549952
7920409	10899	*jumping translocation breakpoint*	*JTB*	11.56	11.26	10.33	8.62E-04	0.005556905
8167287	64840	*porcupine homolog (Drosophila)*	*PORCN*	9.90	10.11	8.50	8.63E-04	0.005561376
7941961	221, 222	*aldehyde dehydrogenase 3 family, member B1 | aldehyde dehydrogenase 3 family, member B2*	*ALDH3B1|ALDH3B2*	10.59	10.51	8.13	8.63E-04	0.005561376
7934411	159195	*ubiquitin specific peptidase 54*	*USP54*	7.26	7.35	8.93	8.64E-04	0.005563214
8081945	4710	*NADH dehydrogenase (ubiquinone) 1 beta subcomplex, 4, 15kDa*	*NDUFB4*	10.59	10.48	11.16	8.65E-04	0.005568705
8162388	4958	*osteomodulin*	*OMD*	9.54	6.39	5.27	8.65E-04	0.005568705
8114898				5.78	5.60	6.67	8.65E-04	0.005568705
8068422	9980	*dopey family member 2*	*DOPEY2*	7.23	7.70	7.21	8.65E-04	0.005570689
8152617	3037	*hyaluronan synthase 2*	*HAS2*	8.26	10.59	7.76	8.68E-04	0.00558009
8046695	3676, 375298	*integrin, alpha 4 (antigen CD49D, alpha 4 subunit of VLA-4 receptor) | ceramide kinase-like*	*ITGA4|CERKL*	7.04	7.88	6.11	8.68E-04	0.00558009
7974314				6.71	6.20	8.46	8.67E-04	0.00558009
8027381				7.66	7.77	6.77	8.68E-04	0.00558009
8063187	2139	*eyes absent homolog 2 (Drosophila)*	*EYA2*	8.03	8.49	6.61	8.69E-04	0.005586274
7933372	2662	*growth differentiation factor 10*	*GDF10*	7.89	7.66	7.01	8.69E-04	0.005588654

7941380				7.00	6.93	6.50	8.70E-04	0.005590094
8137959	84629	*trinucleotide repeat containing 18*	*TNRC18*	9.08	8.99	7.99	8.72E-04	0.005601758
8046530	3237	*homeobox D11*	*HOXD11*	7.10	7.12	6.42	8.72E-04	0.005602539
8165703	51720	*ubiquitin interaction motif containing 1*	*UIMC1*	9.67	9.68	8.79	8.73E-04	0.005602539
8067543	149650		*FLJ32154*	6.95	7.09	6.41	8.73E-04	0.005602539
8179331	717, 629	*complement component 2 | complement factor B*	*C2|CFB*	7.85	7.13	6.11	8.72E-04	0.005602539
8046755				7.51	7.43	6.94	8.74E-04	0.005608216
7997533	29948	*oxidative stress induced growth inhibitor 1*	*OSGIN1*	8.37	8.24	7.34	8.74E-04	0.00560846
8119599	171558	*pre T-cell antigen receptor alpha*	*PTCRA*	8.00	8.05	7.30	8.74E-04	0.00560846
7999596	90231, 728138	*KIAA2013 | KIAA2013 pseudogene*	*KIAA2013| LOC728138*	11.35	11.28	10.16	8.74E-04	0.00560846
8095728	2069	*epiregulin*	*EREG*	6.09	6.07	5.60	8.75E-04	0.005609772
8009008	339175	*methyltransferase like 2A*	*METTL2A*	8.04	8.17	8.99	8.76E-04	0.005613607
8109093	22885	*actin binding LIM protein family, member 3*	*ABLIM3*	8.96	9.00	6.96	8.76E-04	0.00561661
7900957	128209	*Kruppel-like factor 17*	*KLF17*	6.78	6.94	6.20	8.77E-04	0.005617435
7969986	10673	*tumor necrosis factor (ligand) superfamily, member 13b*	*TNFSF13B*	6.92	5.99	5.71	8.77E-04	0.005617572
7971661	406948	*microRNA 15a*	*MIR15A*	7.33	8.08	6.86	8.77E-04	0.005620362
7977270	388022		*LOC388022*	9.86	9.52	7.54	8.78E-04	0.005624086
8027222	148113	*cartilage intermediate layer protein 2*	*CILP2*	7.57	7.61	7.01	8.79E-04	0.005629132
8067932	388815	*chromosome 21 open reading frame 34*	*C21orf34*	5.92	6.71	5.01	8.80E-04	0.005632788
7914042	9064	*mitogen-activated protein kinase kinase kinase 6*	*MAP3K6*	9.34	9.06	7.22	8.80E-04	0.005633698
7943919	54970	*tetratricopeptide repeat domain 12*	*TTC12*	7.91	6.58	7.46	8.81E-04	0.005635408
8035271	79575	*abhydrolase domain containing 8*	*ABHD8*	8.68	8.49	7.74	8.82E-04	0.005641067
8112342	11174	*ADAM metallopeptidase with thrombospondin type 1 motif, 6*	*ADAMTS6*	7.07	7.87	5.81	8.83E-04	0.005644441
8176109	8273	*solute carrier family 10 (sodium/bile acid cotransporter family), member 3*	*SLC10A3*	9.57	9.73	8.11	8.83E-04	0.005644892
7915718	10420	*testis-specific kinase 2*	*TESK2*	7.59	7.47	8.83	8.83E-04	0.005644892

7920659	200185	*keratinocyte associated protein 2*	*KRTCAP2*	11.64	11.74	10.81	8.84E-04	0.005644892
7932023	9712, 439951	*USP6 N-terminal like | hypothetical LOC439951*	*USP6NL|LOC439951*	8.13	8.10	9.95	8.83E-04	0.005644892
7974339				6.94	6.97	6.57	8.84E-04	0.005644892
7960654	51147	*inhibitor of growth family, member 4*	*ING4*	10.00	9.89	9.28	8.84E-04	0.005647618
7942592	6079	*small nucleolar RNA, C/D box 15A*	*SNORD15A*	8.08	8.02	9.92	8.85E-04	0.005650185
7901087	84842	*4-hydroxyphenylpyruvate dioxygenase-like*	*HPDL*	6.34	6.58	8.31	8.86E-04	0.005653171
8113413	83594	*nudix (nucleoside diphosphate linked moiety X)-type motif 12*	*NUDT12*	7.21	6.99	8.23	8.87E-04	0.005662092
8036033	284402	*secretoglobin-like*	*SCGBL*	6.32	6.20	5.86	8.87E-04	0.005662391
8138289	2115	*ets variant 1*	*ETV1*	6.98	7.63	9.93	8.88E-04	0.005665653
7929840	5076	*paired box 2*	*PAX2*	7.21	7.24	6.70	8.89E-04	0.005666104
7929424	23232	*TBC1 domain family, member 12*	*TBC1D12*	7.82	7.82	7.04	8.89E-04	0.005666104
7911343	51720	*ubiquitin interaction motif containing 1*	*UIMC1*	9.68	9.68	8.80	8.89E-04	0.005666104
8064686	80332	*ADAM metallopeptidase domain 33*	*ADAM33*	10.62	9.78	7.82	8.89E-04	0.005666104
8130073	340152	*zinc finger CCCH-type containing 12D*	*ZC3H12D*	8.04	8.09	7.30	8.89E-04	0.005666104
8067754	100128998	*chromosome 20 open reading frame 181*	*C20orf181*	7.80	7.71	7.30	8.89E-04	0.005666104
8150186	79845	*ring finger protein 122*	*RNF122*	7.82	8.88	8.99	8.91E-04	0.005674627
7995128	3687	*integrin, alpha X (complement component 3 receptor 4 subunit)*	*ITGAX*	6.68	6.74	6.21	8.92E-04	0.005678063
8091103	7029	*transcription factor Dp-2 (E2F dimerization partner 2)*	*TFDP2*	9.66	9.50	11.73	8.92E-04	0.005678063
8159086	9719	*ADAMTS-like 2*	*ADAMTSL2*	7.41	7.35	6.85	8.93E-04	0.005678063
7936307	10285	*survival motor neuron domain containing 1*	*SMNDC1*	9.52	9.64	10.37	8.93E-04	0.005678063
7938816	54503	*zinc finger, DHHC-type containing 13*	*ZDHHC13*	8.79	8.83	10.06	8.92E-04	0.005678063
7987025				5.94	5.82	7.56	8.92E-04	0.005678063
7988685				7.10	7.14	6.41	8.92E-04	0.005678063
7906501	477	*ATPase, Na+/K+ transporting, alpha 2 polypeptide*	*ATP1A2*	6.50	6.57	7.51	8.93E-04	0.005679129
7917754	8412	*breast cancer anti-estrogen resistance 3*	*BCAR3*	7.55	8.16	6.88	8.93E-04	0.005679319
8060736				6.69	6.32	8.62	8.94E-04	0.005679319

8110347	6569	*solute carrier family 34 (sodium phosphate), member 1*	*SLC34A1*	7.33	7.44	6.99	8.94E-04	0.005680719
8065880	128876	*family with sequence similarity 83, member C*	*FAM83C*	6.73	6.87	6.31	8.95E-04	0.005686218
7916341				4.72	4.67	4.91	8.95E-04	0.005687415
7993248	116028	*chromosome 16 open reading frame 75*	*C16orf75*	7.11	7.13	7.90	8.96E-04	0.005690136
8141526	81628	*TSC22 domain family, member 4*	*TSC22D4*	8.17	8.24	7.10	8.97E-04	0.005691923
7959249				7.34	7.53	6.63	8.97E-04	0.005691923
8066716	63916	*engulfment and cell motility 2*	*ELMO2*	9.85	9.85	8.87	8.98E-04	0.005696665
7960052	677829	*small nucleolar RNA, H/ACA box 49*	*SNORA49*	8.98	9.34	8.08	8.99E-04	0.00569978
8031260	115653, 3803, 3804, 768329, 3806, 3802, 100132285, 57292	*killer cell immunoglobulin-like receptor, three domains, long cytoplasmic tail, 3 | killer cell immunoglobulin-like receptor, two domains, long cytoplasmic tail, 2 | killer cell immunoglobulin-like receptor, two domains, long cytoplasmic tail, 3 | killer-cell Ig-like receptor | killer cell immunoglobulin-like receptor, two domains, short cytoplasmic tail, 1 | killer cell immunoglobulin-like receptor, two domains, long cytoplasmic tail, 1 | killer cell immunoglobulin-like receptor, two domains, short cytoplasmic tail, 2 | killer cell immunoglobulin-like receptor, two domains, long cytoplasmic tail, 5A*	*KIR3DL3| KIR2DL2| KIR2DL3| KIR3DP1| KIR2DS1| KIR2DL1| KIR2DS2| KIR2DL5A*	6.52	6.47	6.11	8.98E-04	0.00569978
8047565	150864	*family with sequence similarity 117, member B*	*FAM117B*	8.05	7.86	10.08	8.99E-04	0.005702498
8066981				6.55	6.68	6.07	8.99E-04	0.005702498
8038653	606293	*kallikrein pseudogene 1*	*KLKP1*	6.27	6.39	5.88	9.00E-04	0.005705757
7988245	4236	*microfibrillar-associated protein 1*	*MFAP1*	6.88	6.75	8.09	9.00E-04	0.005706126
8161421				9.39	9.59	8.80	9.01E-04	0.005706126
8130071	79768, 729176	*chromosome 15 open reading frame 29 | chromosome 15 open reading frame 29 pseudogene*	*C15orf29| LOC72917 6*	7.57	7.90	9.30	9.01E-04	0.005708579
7944382	55823	*vacuolar protein sorting 11 homolog (S. cerevisiae)*	*VPS11*	10.65	10.48	9.16	9.01E-04	0.005708717
7979516	57570	*TRM5 tRNA methyltransferase 5 homolog (S. cerevisiae)*	*TRMT5*	8.08	7.84	9.34	9.02E-04	0.005708717
8070563	150147, 89766	*chromosome 21 open reading frame 128 | uromodulin-like 1*	*C21orf128| UMODL1*	6.53	6.58	6.25	9.03E-04	0.005714124

7915870	64756, 9813	*ATP synthase mitochondrial F1 complex assembly factor 1 | KIAA0494*	*ATPAF1|KIAA0494*	7.40	7.51	8.33	9.03E-04	0.005714124
8052250	4528	*mitochondrial translational initiation factor 2*	*MTIF2*	7.20	7.02	8.52	9.04E-04	0.005719421
8175570				5.92	5.93	5.48	9.04E-04	0.005719421
8075585	51493	*chromosome 22 open reading frame 28*	*C22orf28*	10.36	10.16	11.48	9.04E-04	0.005720282
8140915	5189	*peroxisomal biogenesis factor 1*	*PEX1*	7.82	7.69	9.70	9.06E-04	0.005728407
8033097	5990	*regulatory factor X, 2 (influences HLA class II expression)*	*RFX2*	8.45	8.72	6.97	9.08E-04	0.005738774
8089128	9868	*translocase of outer mitochondrial membrane 70 homolog A (S. cerevisiae)*	*TOMM70A*	7.98	7.94	9.34	9.09E-04	0.005744597
8039947	10772	*serine/arginine-rich splicing factor 10*	*SRSF10*	7.29	7.32	9.49	9.09E-04	0.005744597
8032650	23396	*phosphatidylinositol-4-phosphate 5-kinase, type I, gamma*	*PIP5K1C*	10.35	10.43	8.55	9.09E-04	0.005745294
8034821	5731	*prostaglandin E receptor 1 (subtype EP1), 42kDa*	*PTGER1*	8.25	8.37	7.46	9.10E-04	0.00574923
7923824	254428	*solute carrier family 41, member 1*	*SLC41A1*	10.22	10.17	9.12	9.11E-04	0.005752978
8176245	8263, 474383, 474384	*coagulation factor VIII-associated (intronic transcript) 1 | coagulation factor VIII-associated (intronic transcript) 2 | coagulation factor VIII-associated (intronic transcript) 3*	*F8A1|F8A2|F8A3*	11.77	11.64	10.42	9.11E-04	0.005754503
8042086	7444	*vaccinia related kinase 2*	*VRK2*	7.87	8.00	8.98	9.12E-04	0.005757493
8014666	440435	*G protein-coupled receptor 179*	*GPR179*	6.81	6.72	6.37	9.14E-04	0.005770882
8176234	1193	*chloride intracellular channel 2*	*CLIC2*	6.18	5.27	4.99	9.16E-04	0.005772952
8022393	5771	*protein tyrosine phosphatase, non-receptor type 2*	*PTPN2*	7.66	7.41	8.98	9.16E-04	0.005772952
8103415	51313	*family with sequence similarity 198, member B*	*FAM198B*	7.92	9.31	6.20	9.15E-04	0.005772952
7919160				5.51	5.34	6.70	9.15E-04	0.005772952
8021714				6.49	6.49	6.16	9.15E-04	0.005772952
7916412	26027, 338094	*acyl-CoA thioesterase 11 | family with sequence similarity 151, member A*	*ACOT11|FAM151A*	6.95	7.16	6.45	9.17E-04	0.005778985
8081001	6092	*roundabout, axon guidance receptor, homolog 2 (Drosophila)*	*ROBO2*	9.77	6.19	6.35	9.18E-04	0.005780289
7978923	79609	*chromosome 14 open reading frame 138*	*C14orf138*	6.07	6.26	7.47	9.17E-04	0.005780289
8102783				8.03	7.98	7.39	9.18E-04	0.005780289

7995306				11.32	11.35	10.86	9.19E-04	0.005785864
7922868				5.41	5.69	4.99	9.19E-04	0.005786313
7989253	79811	*SAFB-like, transcription modulator*	*SLTM*	7.48	7.17	9.90	9.20E-04	0.005790016
8077858	10533		*ATG7*	8.92	8.80	8.20	9.21E-04	0.005794519
8040949	200634	*keratinocyte associated protein 3*	*KRTCAP3*	7.96	8.07	8.61	9.21E-04	0.005794519
8143725	155061	*zinc finger protein 746*	*ZNF746*	10.42	10.49	9.54	9.22E-04	0.00580035
7956417				5.52	5.46	5.21	9.22E-04	0.00580035
8074647	5297	*phosphatidylinositol 4-kinase, catalytic, alpha*	*PI4KA*	10.32	10.16	8.74	9.23E-04	0.005806876
8120247				6.20	6.06	5.66	9.24E-04	0.005809441
7997738				5.90	5.58	5.28	9.24E-04	0.005810689
7928746				7.01	7.09	6.08	9.25E-04	0.005813606
8156274	158046	*nucleoredoxin-like 2*	*NXNL2*	6.97	6.94	6.43	9.25E-04	0.005813951
8090485	23434	*chromosome 3 open reading frame 27*	*C3orf27*	7.34	7.44	6.88	9.27E-04	0.005822396
7900095	55700	*MAP7 domain containing 1*	*MAP7D1*	9.63	9.82	8.12	9.27E-04	0.00582291
8074642				6.71	6.75	6.31	9.28E-04	0.00582375
8146941				7.97	8.09	6.79	9.29E-04	0.005833314
8087852	54106, 11344	*toll-like receptor 9 | twinfilin, actin-binding protein, homolog 2 (Drosophila)*	*TLR9|TWF2*	7.91	7.89	7.08	9.32E-04	0.005848015
8002969	4094	*v-maf musculoaponeurotic fibrosarcoma oncogene homolog (avian)*	*MAF*	7.22	6.76	6.09	9.32E-04	0.005848086
8144412	349196		*LOC349196*	9.87	8.80	7.27	9.34E-04	0.005858291
8073242	158	*adenylosuccinate lyase*	*ADSL*	9.64	9.67	10.93	9.35E-04	0.005863104
7943779	64776	*chromosome 11 open reading frame 1*	*C11orf1*	7.71	7.89	8.40	9.35E-04	0.005863104
7911799	1953	*multiple EGF-like-domains 6*	*MEGF6*	9.33	9.25	7.80	9.35E-04	0.005864495
8107594	9627	*synuclein, alpha interacting protein*	*SNCAIP*	6.76	6.57	7.23	9.36E-04	0.005865334
7936115	9644	*SH3 and PX domains 2A*	*SH3PXD2A*	9.14	9.42	7.33	9.36E-04	0.005865334
8035773	440515	*zinc finger protein 506*	*ZNF506*	7.79	7.72	9.34	9.36E-04	0.005866588
8000013	112479, 6296	*ERI1 exoribonuclease family member 2 | acyl-CoA synthetase medium-chain family member 3*	*ERI2|ACSM3*	6.89	7.00	8.11	9.37E-04	0.005871058

7902023	55225	*ribonucleoprotein, PTB-binding 2*	*RAVER2*	7.38	6.85	9.88	9.38E-04	0.005873189
8124430	3007	*histone cluster 1, H1d*	*HIST1H1D*	7.46	8.19	10.32	9.39E-04	0.005878304
8178095	717, 629	*complement component 2 | complement factor B*	*C2|CFB*	7.84	7.13	6.11	9.39E-04	0.005878304
7999920	2813	*glycoprotein 2 (zymogen granule membrane)*	*GP2*	6.25	6.20	5.82	9.39E-04	0.005879475
7967976	10284	*Sin3A-associated protein, 18kDa*	*SAP18*	9.69	9.84	10.48	9.40E-04	0.005882511
8105463	65056	*GC-rich promoter binding protein 1*	*GPBP1*	8.77	8.51	10.27	9.40E-04	0.005882511
8107868	56990	*CDC42 small effector 2*	*CDC42SE 2*	8.23	7.89	10.14	9.41E-04	0.005884298
8176159	30848	*cancer/testis antigen 2*	*CTAG2*	8.93	8.95	8.31	9.41E-04	0.005884384
7904158	56944	*olfactomedin-like 3*	*OLFML3*	12.60	12.58	11.27	9.42E-04	0.00588671
7924150	55248	*transmembrane protein 206*	*TMEM206*	7.43	7.45	8.61	9.42E-04	0.005886782
8107644				6.78	6.84	6.39	9.42E-04	0.005887727
8098870	10417, 100322895	*spondin 2, extracellular matrix protein | hypothetical LOC100322895*	*SPON2|LO C1001308 72-SPON2*	10.46	8.94	7.11	9.43E-04	0.005888657
8151991				6.43	6.44	5.46	9.45E-04	0.005902213
7985765	407044	*microRNA 7-2*	*MIR7-2*	6.76	6.83	6.20	9.45E-04	0.005902484
8176508	728137, 7258, 64591, 728395, 728403	*testis specific protein, Y-linked 3 | testis specific protein, Y-linked 1 | testis specific protein, Y-linked 2 | testis specific protein, Y-linked 4 | testis specific protein, Y-linked 8*	*TSPY3|TS PY1|TSPY 2|TSPY4|T SPY8*	6.21	6.18	5.89	9.45E-04	0.005902484
8115234	309	*annexin A6*	*ANXA6*	12.80	12.76	11.41	9.46E-04	0.005903203
8081158	84100	*ADP-ribosylation factor-like 6*	*ARL6*	6.08	6.08	7.40	9.46E-04	0.005903203
8147785	25879	*DDB1 and CUL4 associated factor 13*	*DCAF13*	7.91	8.22	8.93	9.46E-04	0.005904852
8110990	1501	*catenin (cadherin-associated protein), delta 2 (neural plakophilin-related arm-repeat protein)*	*CTNND2*	6.94	6.87	7.74	9.47E-04	0.005907691
8144414	349196		*LOC34919 6*	9.87	8.81	7.27	9.47E-04	0.005909088
7978360	2999	*granzyme H (cathepsin G-like 2, protein h-CCPX)*	*GZMH*	6.84	6.82	6.35	9.50E-04	0.005922107

7992197				7.66	7.58	6.99	9.51E-04	0.005925858
7973135	29986	*solute carrier family 39 (zinc transporter), member 2*	*SLC39A2*	5.94	5.96	5.60	9.51E-04	0.005926846
7991406	9055	*protein regulator of cytokinesis 1*	*PRC1*	8.22	8.58	10.44	9.52E-04	0.005930544
7933164	3185	*heterogeneous nuclear ribonucleoprotein F*	*HNRNPF*	9.10	9.02	10.44	9.52E-04	0.005931474
7942793	7069	*thyroid hormone responsive*	*THRSP*	6.58	6.49	6.07	9.53E-04	0.005935636
8105661	80006	*chromosome 5 open reading frame 44*	*C5orf44*	4.91	4.85	5.78	9.53E-04	0.005937356
8102567	11107	*PR domain containing 5*	*PRDM5*	7.49	7.59	8.75	9.57E-04	0.005958612
8159255	3933	*lipocalin 1 (tear prealbumin)*	*LCN1*	8.00	7.97	7.59	9.59E-04	0.005967836
8170998	8263, 474383, 474384	*coagulation factor VIII-associated (intronic transcript) 1 | coagulation factor VIII-associated (intronic transcript) 2 | coagulation factor VIII-associated (intronic transcript) 3*	*F8A1|F8A2|F8A3*	11.68	11.54	10.26	9.59E-04	0.005967836
7915056	284656	*EPH receptor A10*	*EPHA10*	5.74	5.95	5.47	9.60E-04	0.005970622
8050441				8.06	7.97	7.23	9.60E-04	0.00597385
8102212				6.99	7.35	6.69	9.61E-04	0.005974492
7919984	8991	*selenium binding protein 1*	*SELENBP1*	11.44	9.78	9.36	9.62E-04	0.005978948
8150901	5179	*proenkephalin*	*PENK*	11.51	7.45	6.70	9.62E-04	0.005982549
8148149	22882	*zinc fingers and homeoboxes 2*	*ZHX2*	10.39	9.43	8.55	9.63E-04	0.0059849
8175589				5.89	5.94	5.17	9.63E-04	0.005985257
7930031	8729	*golgi brefeldin A resistant guanine nucleotide exchange factor 1*	*GBF1*	10.96	10.59	9.41	9.64E-04	0.005986321
8019807	9097	*ubiquitin specific peptidase 14 (tRNA-guanine transglycosylase)*	*USP14*	9.46	9.80	10.82	9.64E-04	0.005988161
8038571	126119	*Josephin domain containing 2*	*JOSD2*	9.63	9.66	7.96	9.65E-04	0.00599053
8150153				5.89	6.00	5.39	9.65E-04	0.005992804
7919872	55793	*family with sequence similarity 63, member A*	*FAM63A*	8.97	8.21	7.47	9.66E-04	0.005998312
8099091				8.59	8.92	7.61	9.68E-04	0.006007424
8083779	5274	*serpin peptidase inhibitor, clade I (neuroserpin), member 1*	*SERPINI1*	5.58	5.66	7.28	9.69E-04	0.006012467
8111210				12.46	12.47	11.51	9.69E-04	0.006013672

7953547	1822	*atrophin 1*	*ATN1*	10.99	10.76	9.10	9.70E-04	0.006016462
8050352	23620	*neurotensin receptor 2*	*NTSR2*	6.54	6.66	6.13	9.70E-04	0.006017321
8114814	2908	*nuclear receptor subfamily 3, group C, member 1 (glucocorticoid receptor)*	*NR3C1*	9.34	8.99	6.66	9.71E-04	0.006021171
7937847	55539	*KCNQ1 downstream neighbor*	*KCNQ1DN*	7.89	7.83	7.38	9.74E-04	0.006033732
8053073	57835	*solute carrier family 4, sodium bicarbonate cotransporter, member 5*	*SLC4A5*	5.68	5.68	5.43	9.74E-04	0.006033732
8061373	64412	*GDNF-inducible zinc finger protein 1*	*GZF1*	7.58	7.85	8.18	9.74E-04	0.006033732
8102871	100129858		*LOC100129858*	6.10	5.99	5.82	9.75E-04	0.006034682
8065537	100134868		*LOC100134868*	6.83	6.89	9.81	9.75E-04	0.006034682
8050215	10971, 392510	*tyrosine 3-monooxygenase/tryptophan 5-monooxygenase activation protein, theta polypeptide | YWHAQ pseudogene 8*	*YWHAQ|YWHAQP8*	12.65	12.67	13.17	9.75E-04	0.006034682
8039820	115653, 57292, 3804	*killer cell immunoglobulin-like receptor, three domains, long cytoplasmic tail, 3 | killer cell immunoglobulin-like receptor, two domains, long cytoplasmic tail, 5A | killer cell immunoglobulin-like receptor, two domains, long cytoplasmic tail, 3*	*KIR3DL3|KIR2DL5A|KIR2DL3*	6.65	6.63	6.14	9.74E-04	0.006034682
8116867	81853	*transmembrane protein 14B*	*TMEM14B*	12.21	12.25	12.82	9.76E-04	0.006036764
8054870				8.37	9.12	7.39	9.76E-04	0.006036764
7904000	11218	*DEAD (Asp-Glu-Ala-Asp) box polypeptide 20*	*DDX20*	8.08	8.06	9.79	9.77E-04	0.006041289
7930008	9221	*nucleolar and coiled-body phosphoprotein 1*	*NOLC1*	7.98	8.15	10.91	9.77E-04	0.006042922
8165711	55344	*phosphatidylinositol-specific phospholipase C, X domain containing 1*	*PLCXD1*	7.49	7.37	8.70	9.78E-04	0.006047578
8176524	728137, 7258, 64591, 728395, 728403	*testis specific protein, Y-linked 3 | testis specific protein, Y-linked 1 | testis specific protein, Y-linked 4 | testis specific protein, Y-linked 2 | testis specific protein, Y-linked 8*	*TSPY3|TSPY1|TSPY4|TSPY2|TSPY8*	6.26	6.22	5.93	9.78E-04	0.006047907
8130817	653483	*chromosome 6 open reading frame 124*	*C6orf124*	6.32	6.35	6.65	9.79E-04	0.00605061
8060705	57506	*mitochondrial antiviral signaling protein*	*MAVS*	9.82	9.77	8.39	9.80E-04	0.006052552

7906900	4921	*discoidin domain receptor tyrosine kinase 2*	*DDR2*	9.02	8.74	5.17	9.80E-04	0.006054019
7952577	84623	*kin of IRRE like 3 (Drosophila)*	*KIRREL3*	8.00	8.73	6.78	9.80E-04	0.006054019
7996290	113540, 51192	*CKLF-like MARVEL transmembrane domain containing 1 | chemokine-like factor*	*CMTM1|CKLF*	7.26	7.40	6.71	9.80E-04	0.006054019
8103622	84869	*carbonyl reductase 4*	*CBR4*	7.00	6.94	9.13	9.81E-04	0.006054948
7981978	100033427	*small nucleolar RNA, C/D box 116-15*	*SNORD116-15*	13.39	13.49	13.75	9.81E-04	0.006055704
7985266				6.30	6.01	6.94	9.81E-04	0.006055704
8037322	23474	*ethylmalonic encephalopathy 1*	*ETHE1*	11.57	10.59	8.61	9.83E-04	0.006060408
8048703	84298	*LLP homolog, long-term synaptic facilitation (Aplysia)*	*LLPH*	8.74	9.06	10.48	9.83E-04	0.006060408
7919929	29765, 79005	*tropomodulin 4 (muscle) | sodium channel modifier 1*	*TMOD4|SCNM1*	5.99	5.91	5.40	9.83E-04	0.006060408
7983771				6.53	6.62	5.62	9.83E-04	0.006060408
8039212	10288	*leukocyte immunoglobulin-like receptor, subfamily B (with TM and ITIM domains), member 2*	*LILRB2*	6.91	7.04	6.47	9.84E-04	0.006066671
7926021	84991	*RNA binding motif protein 17*	*RBM17*	7.14	7.16	8.39	9.85E-04	0.006070741
8023766	25914	*rotatin*	*RTTN*	6.50	6.94	8.24	9.85E-04	0.006072475
8147156	11059	*WW domain containing E3 ubiquitin protein ligase 1*	*WWP1*	9.48	8.95	10.95	9.86E-04	0.006075672
7927108	9790		*BMS1*	7.66	7.67	9.53	9.87E-04	0.006076844
8159127	6256	*retinoid X receptor, alpha*	*RXRA*	10.22	10.25	8.71	9.87E-04	0.006077953
8001197	81831	*neuropilin (NRP) and tolloid (TLL)-like 2*	*NETO2*	7.23	8.11	9.73	9.88E-04	0.006079956
8026405	126370	*olfactory receptor, family 1, subfamily I, member 1*	*OR1I1*	6.77	6.88	6.37	9.88E-04	0.006079956
8143486	136541	*trypsin X3*	*TRYX3*	5.22	5.26	5.03	9.88E-04	0.006079956
8095364	28983	*transmembrane protease, serine 11E*	*TMPRSS11E*	5.39	5.24	6.70	9.88E-04	0.006081031
8146703	254778	*chromosome 8 open reading frame 46*	*C8orf46*	6.51	6.84	6.21	9.90E-04	0.00608731
7983630	2252	*fibroblast growth factor 7*	*FGF7*	8.80	9.43	5.38	9.91E-04	0.006087432
8118794	3159	*high mobility group AT-hook 1*	*HMGA1*	10.01	10.16	11.46	9.91E-04	0.006087432
7912887	4237	*microfibrillar-associated protein 2*	*MFAP2*	11.54	12.07	10.86	9.91E-04	0.006087432
8064031	79444	*baculoviral IAP repeat-containing 7*	*BIRC7*	7.48	7.60	7.06	9.90E-04	0.006087432
8031632	147947	*zinc finger protein 542*	*ZNF542*	7.73	7.80	9.52	9.90E-04	0.006087432

8170882	537	*ATPase, H+ transporting, lysosomal accessory protein 1*	*ATP6AP1*	11.94	11.75	10.51	9.92E-04	0.006092899
8111892	5019	*3-oxoacid CoA transferase 1*	*OXCT1*	7.79	7.59	9.16	9.92E-04	0.006092899
8013094	8533	*COP9 constitutive photomorphogenic homolog subunit 3 (Arabidopsis)*	*COPS3*	9.92	10.04	11.21	9.92E-04	0.006092899
8125234	7148, 7146	*tenascin XB | tenascin XA pseudogene*	*TNXB|TNXA*	9.19	8.33	6.77	9.92E-04	0.006092899
8003193	339145	*family with sequence similarity 92, member B*	*FAM92B*	7.76	7.83	7.11	9.93E-04	0.006094756
7938420				6.27	6.34	5.89	9.94E-04	0.006097957
8058668				4.74	4.68	4.63	9.94E-04	0.006101986
7945620	54472	*toll interacting protein*	*TOLLIP*	9.64	9.76	8.38	9.95E-04	0.00610314
7930137				6.08	6.13	5.49	9.96E-04	0.006108339
8021774	400658		*FLJ44313*	7.26	7.25	6.73	9.96E-04	0.006110374
8024843	10036	*chromatin assembly factor 1, subunit A (p150)*	*CHAF1A*	8.01	8.28	9.94	9.99E-04	0.006122971
7933498	170370	*family with sequence similarity 170, member B*	*FAM170B*	7.59	7.66	7.01	0.001000082	0.006128585
8043900				6.23	6.39	7.82	0.001000096	0.006128585
7905067	8370, 554313	*histone cluster 2, H4a | histone cluster 2, H4b*	*HIST2H4A|HIST2H4B*	8.11	8.51	9.80	0.001001967	0.006138751
7987701	51332, 100289090	*spectrin, beta, non-erythrocytic 5 | hypothetical protein LOC100289090*	*SPTBN5|LOC100289090*	7.06	7.12	6.64	0.001002941	0.006143413
8116418	9945	*glutamine-fructose-6-phosphate transaminase 2*	*GFPT2*	9.03	9.67	10.86	0.001003981	0.00614848
7986446	220	*aldehyde dehydrogenase 1 family, member A3*	*ALDH1A3*	8.88	9.46	8.15	0.001004472	0.006148876
7910490				6.87	6.97	5.95	0.001004418	0.006148876
8078397	152189	*CKLF-like MARVEL transmembrane domain containing 8*	*CMTM8*	7.06	6.97	8.50	0.001004791	0.006149525
7941087	5526	*protein phosphatase 2, regulatory subunit B', beta*	*PPP2R5B*	9.17	9.75	7.40	0.001005888	0.006152316
8061186	10483	*Sec23 homolog B (S. cerevisiae)*	*SEC23B*	9.13	9.68	10.96	0.00100564	0.006152316
8061497	284802	*FSHD region gene 1 family, member B*	*FRG1B*	9.40	9.03	8.53	0.001006137	0.006152316
7925442				6.24	6.02	5.65	0.001006146	0.006152316
8090936				7.11	7.30	6.63	0.001006312	0.006152316

8059158	79411	*galactosidase, beta 1-like*	*GLB1L*	8.66	7.83	6.86	0.001006931	0.006153488
8056753	79828	*methyltransferase like 8*	*METTL8*	7.14	7.12	7.98	0.001006761	0.006153488
7944216	10632	*ATP synthase, H+ transporting, mitochondrial Fo complex, subunit G*	*ATP5L*	11.38	11.25	12.14	0.001007679	0.006155458
7922596	646976		*LOC646976*	7.12	7.15	6.43	0.001007644	0.006155458
8153342	137797	*LY6/PLAUR domain containing 2*	*LYPD2*	7.73	7.71	6.91	0.001008007	0.006156157
8002882	4166	*carbohydrate (N-acetylglucosamine 6-O) sulfotransferase 6*	*CHST6*	7.12	7.16	8.71	0.001008876	0.006160159
8054831	2019	*engrailed homeobox 1*	*EN1*	7.94	8.92	6.69	0.001009176	0.006160399
8007548	23591	*chromosome 17 open reading frame 88*	*C17orf88*	8.15	8.25	7.57	0.001009342	0.006160399
7950524	4135	*microtubule-associated protein 6*	*MAP6*	7.56	7.40	6.59	0.001009845	0.006162168
8169750	10735	*stromal antigen 2*	*STAG2*	8.96	8.70	10.32	0.001010098	0.006162408
8132964	25870	*sulfatase modifying factor 2*	*SUMF2*	12.25	12.19	11.17	0.001010772	0.00616522
8061725	171023	*additional sex combs like 1 (Drosophila)*	*ASXL1*	9.15	9.17	7.88	0.001011827	0.00617035
8022914	55197	*regulation of nuclear pre-mRNA domain containing 1A*	*RPRD1A*	7.91	7.68	10.23	0.001012137	0.006170933
7955890	3178	*heterogeneous nuclear ribonucleoprotein A1*	*HNRNPA1*	5.73	5.72	7.88	0.001013123	0.006175641
8166613	4114	*melanoma antigen family B, 3*	*MAGEB3*	6.28	6.42	5.92	0.001014462	0.006180919
7993737	123876, 348158	*acyl-CoA synthetase medium-chain family member 2A | acyl-CoA synthetase medium-chain family member 2B*	*ACSM2A|ACSM2B*	5.79	5.80	5.59	0.001014233	0.006180919
8054037				5.69	5.89	5.52	0.001014631	0.006180919
7940323	83661	*membrane-spanning 4-domains, subfamily A, member 8B*	*MS4A8B*	6.05	5.89	5.55	0.001016075	0.006188414
7903694	83873	*G protein-coupled receptor 61*	*GPR61*	7.17	7.26	6.71	0.001016461	0.006189454
8094476	55296	*TBC1 domain family, member 19*	*TBC1D19*	9.39	8.84	7.77	0.001017017	0.006191536
7899480	6080, 8420	*small nucleolar RNA, H/ACA box 73A | small nucleolar RNA host gene 3 (non-protein coding)*	*SNORA73A|SNHG3*	11.78	12.03	11.40	0.001018252	0.006197745
8166243	9185	*RALBP1 associated Eps domain containing 2*	*REPS2*	6.32	6.40	7.13	0.001019521	0.006204162
7898793	712	*complement component 1, q subcomponent, A chain*	*C1QA*	6.05	6.10	5.53	0.001020107	0.006205114
8000978	339105	*protease, serine, 53*	*PRSS53*	7.07	7.18	6.57	0.00101999	0.006205114
8095110	3815	*v-kit Hardy-Zuckerman 4 feline sarcoma viral oncogene homolog*	*KIT*	6.76	6.35	9.53	0.001022341	0.006217395

8109981	8817	*fibroblast growth factor 18*	*FGF18*	10.16	8.08	7.36	0.001023003	0.00621897
7902282	11147	*HERV-H LTR-associating 3*	*HHLA3*	10.48	9.73	8.80	0.001023031	0.00621897
8085531	64432	*mitochondrial ribosomal protein S25*	*MRPS25*	7.90	8.00	9.12	0.001024793	0.006226337
7951032	677792	*small nucleolar RNA, H/ACA box 1*	*SNORA1*	6.90	6.82	8.06	0.00102489	0.006226337
8074909	3543, 91316	*immunoglobulin lambda-like polypeptide 1 | glucuronidase, beta/immunoglobulin lambda-like polypeptide 1 pseudogene*	*IGLL1|LOC91316*	7.94	8.06	7.21	0.001024774	0.006226337
7960359				12.00	12.05	11.34	0.001025216	0.006227008
8159439	389813	*chromosome 9 open reading frame 172*	*C9orf172*	8.17	8.02	7.03	0.00102551	0.006227484
7990020	8125	*acidic (leucine-rich) nuclear phosphoprotein 32 family, member A*	*ANP32A*	8.13	8.38	10.94	0.001026012	0.006229219
8152297	284	*angiopoietin 1*	*ANGPT1*	7.21	8.41	6.17	0.001027181	0.006233694
8175434	83550	*G protein-coupled receptor 101*	*GPR101*	6.62	6.72	6.08	0.001027083	0.006233694
7990436	9377	*cytochrome c oxidase subunit Va*	*COX5A*	11.12	11.10	11.65	0.001027888	0.006236674
8048782	1285	*collagen, type IV, alpha 3 (Goodpasture antigen)*	*COL4A3*	6.69	6.80	6.14	0.001028453	0.006238793
7951679	26521	*translocase of inner mitochondrial membrane 8 homolog B (yeast)*	*TIMM8B*	9.51	9.62	10.53	0.001029025	0.006239641
7900214				5.06	4.78	4.57	0.001028993	0.006239641
8096905	55435	*adaptor-related protein complex 1 associated regulatory protein*	*AP1AR*	7.03	7.38	8.61	0.001029548	0.006241502
8099246	80273	*GrpE-like 1, mitochondrial (E. coli)*	*GRPEL1*	8.62	9.05	9.77	0.001030301	0.006243708
7961453				7.39	7.55	6.92	0.001030345	0.006243708
8000706	10423	*CDP-diacylglycerol--inositol 3-phosphatidyltransferase*	*CDIPT*	11.87	11.80	10.76	0.00103178	0.006249901
8067820	440073	*IQ motif and Sec7 domain 3*	*IQSEC3*	8.66	8.84	7.94	0.0010318	0.006249901
8000811	5595	*mitogen-activated protein kinase 3*	*MAPK3*	12.03	11.75	10.65	0.001032415	0.00625083
8121349	246269	*lactation elevated 1*	*LACE1*	6.63	6.60	7.42	0.001032421	0.00625083
7958948	79039, 387885	*DEAD (Asp-Glu-Ala-Asp) box polypeptide 54 | coiled-coil domain containing 42B*	*DDX54|CCDC42B*	7.88	7.91	7.35	0.001032603	0.00625083
8001046	283933	*zinc finger protein 843*	*ZNF843*	7.79	7.78	6.97	0.001034334	0.006256065
7938010	340980	*olfactory receptor, family 52, subfamily B, member 6*	*OR52B6*	5.88	5.81	5.68	0.001034332	0.006256065

7928547				11.10	11.33	10.31	0.001033795	0.006256065
8133012				7.57	7.37	6.91	0.001033915	0.006256065
7942889	84233	*transmembrane protein 126A*	*TMEM126 A*	7.33	7.43	8.16	0.001035294	0.00626056
7939365	24147	*four jointed box 1 (Drosophila)*	*FJX1*	8.13	9.09	7.54	0.001036497	0.00626652
8119052	285848	*patatin-like phospholipase domain containing 1*	*PNPLA1*	6.48	6.51	5.88	0.001036776	0.006266894
7922474	9674		*KIAA0040*	6.46	5.96	8.51	0.001036998	0.006266925
8045919	64844	*membrane-associated ring finger (C3HC4) 7*	*7-Mar*	9.52	9.33	11.10	0.001037331	0.006267624
7930422	9126	*structural maintenance of chromosomes 3*	*SMC3*	8.18	7.84	10.40	0.0010388	0.006273876
8107798	28965	*solute carrier family 27 (fatty acid transporter), member 6*	*SLC27A6*	5.75	5.59	6.93	0.001038623	0.006273876
8085112				6.05	5.97	5.58	0.001039795	0.006278568
8165046	157922	*calmodulin regulated spectrin-associated protein 1*	*CAMSAP1*	8.42	8.63	9.45	0.001041085	0.006285045
8166956	7403	*lysine (K)-specific demethylase 6A*	*KDM6A*	8.00	8.25	10.30	0.001041687	0.006285277
8009705	92736	*otopetrin 2*	*OTOP2*	6.69	6.76	6.31	0.001041777	0.006285277
8025927	199692	*zinc finger protein 627*	*ZNF627*	8.01	8.07	9.08	0.001041682	0.006285277
7914500	56063	*chromosome 1 open reading frame 91*	*C1orf91*	8.01	8.01	6.86	0.001042573	0.006288243
8176117	60343	*family with sequence similarity 3, member A*	*FAM3A*	9.86	9.92	8.42	0.001042704	0.006288243
8049471	57007	*chemokine (C-X-C motif) receptor 7*	*CXCR7*	6.10	6.86	5.08	0.001043336	0.006290743
7985442				6.39	6.46	6.07	0.001043646	0.006291295
8014037	51379	*cytokine receptor-like factor 3*	*CRLF3*	7.13	7.46	8.98	0.001044672	0.006296165
7906602	57216	*vang-like 2 (van gogh, Drosophila)*	*VANGL2*	6.76	6.86	8.18	0.001045437	0.006298147
8137524				7.01	6.87	6.06	0.001045421	0.006298147
8124502	7738	*zinc finger protein 184*	*ZNF184*	7.43	7.18	8.42	0.001046634	0.006302168
7952086	11181	*trehalase (brush-border membrane glycoprotein)*	*TREH*	6.22	6.17	5.92	0.001046591	0.006302168
8058997	53632	*protein kinase, AMP-activated, gamma 3 non-catalytic subunit*	*PRKAG3*	6.67	6.70	6.17	0.001046958	0.006302168
8107530				7.63	7.38	6.99	0.001046978	0.006302168
8109049	84651	*serine peptidase inhibitor, Kazal type 7 (putative)*	*SPINK7*	4.76	4.72	4.55	0.001048588	0.006310543
7965510	57458	*transmembrane and coiled-coil domain family 3*	*TMCC3*	7.11	7.20	8.20	0.001051341	0.006325793

7950955	57093, 653111, 283257	*tripartite motif-containing 49 | tripartite motif-containing 49-like 2 | tripartite motif-containing protein 49B-like*	*TRIM49|TRIM49L2|TRIM49B*	5.66	5.62	6.09	0.001052075	0.006328895
8113305	1105	*chromodomain helicase DNA binding protein 1*	*CHD1*	7.48	7.34	9.66	0.001052577	0.00633059
7906433				8.46	8.60	6.96	0.001053158	0.006332765
8129901	85021	*RALBP1 associated Eps domain containing 1*	*REPS1*	9.78	9.81	11.40	0.001053562	0.006333876
8010778	1453	*casein kinase 1, delta*	*CSNK1D*	7.70	7.99	6.62	0.001055183	0.006342304
8079165	406929	*microRNA 138-1*	*MIR138-1*	7.37	7.39	6.86	0.001057792	0.006356662
8116316	11282	*mannosyl (alpha-1,3-)-glycoprotein beta-1,4-N-acetylglucosaminyltransferase, isozyme B*	*MGAT4B*	10.01	10.21	8.65	0.001060009	0.006364603
8029642	57787	*MAP/microtubule affinity-regulating kinase 4*	*MARK4*	9.99	10.00	8.72	0.001060071	0.006364603
8142730	168850	*zinc finger protein 800*	*ZNF800*	9.35	9.47	8.19	0.001059791	0.006364603
8005549	400581, 10750	*GRB2-related adaptor protein-like | GRB2-related adaptor protein*	*GRAPL*	7.01	6.92	6.44	0.001060216	0.006364603
8176517	7258, 728137, 64591, 728395, 728403	*testis specific protein, Y-linked 1 | testis specific protein, Y-linked 3 | testis specific protein, Y-linked 2 | testis specific protein, Y-linked 4 | testis specific protein, Y-linked 8*	*TSPY1|TSPY3|TSPY2|TSPY4|TSPY8*	6.35	6.32	6.01	0.00106007	0.006364603
7970031				6.19	6.20	5.70	0.001061818	0.006372892
8169015	56849	*transcription elongation factor A (SII)-like 7*	*TCEAL7*	8.46	7.46	7.79	0.001062085	0.006373172
8047370				4.79	4.76	5.55	0.001062961	0.006377101
7941127	7542	*zinc finger protein-like 1*	*ZFPL1*	10.96	10.90	9.41	0.001064845	0.006382435
8082133	10954	*protein disulfide isomerase family A, member 5*	*PDIA5*	9.47	9.57	8.82	0.001064528	0.006382435
7960757	51279	*complement component 1, r subcomponent-like*	*C1RL*	10.10	9.56	7.88	0.001065176	0.006382435
8091562	170506	*DEAH (Asp-Glu-Ala-His) box polypeptide 36*	*DHX36*	7.47	7.10	9.89	0.001065044	0.006382435
7898357	11209, 4485, 11223	*macrophage stimulating 1 (hepatocyte growth factor-like) pseudogene 2 | macrophage stimulating 1 (hepatocyte growth factor-like) pseudogene 9*	*MST1P2|MST1P9*	7.17	7.05	6.52	0.001065012	0.006382435
7959621				6.93	6.94	6.77	0.001064474	0.006382435

8176484	728137, 7258, 64591, 728395, 728403	*testis specific protein, Y-linked 3 | testis specific protein, Y-linked 1 | testis specific protein, Y-linked 4 | testis specific protein, Y-linked 2 | testis specific protein, Y-linked 8*	*TSPY3|TSPY1|TSPY4|TSPY2|TSPY8*	6.21	6.21	5.90	0.001065837	0.006385069
8154934	3590	*interleukin 11 receptor, alpha*	*IL11RA*	9.27	9.07	7.10	0.001066612	0.006388389
8065334	4821	*NK2 homeobox 2*	*NKX2-2*	6.57	6.90	6.36	0.001066889	0.006388722
7969179	79621	*ribonuclease H2, subunit B*	*RNASEH2B*	8.21	8.67	9.06	0.001067445	0.006389401
8107996				9.27	9.55	8.31	0.001067247	0.006389401
8087691	9254	*calcium channel, voltage-dependent, alpha 2/delta subunit 2*	*CACNA2D2*	6.72	6.79	7.68	0.001068281	0.006393075
8076513				8.46	8.51	7.93	0.001069653	0.00639996
7938416	100129827		*LOC100129827*	7.48	7.38	6.36	0.001070322	0.006402639
8041206	81606	*limb bud and heart development homolog (mouse)*	*LBH*	10.82	11.83	8.94	0.001070775	0.006402695
8100962				8.41	8.50	7.93	0.001070762	0.006402695
8152115				6.73	7.05	6.23	0.001071067	0.006403111
8025672	57153	*solute carrier family 44, member 2*	*SLC44A2*	12.60	11.94	11.17	0.001071755	0.006405897
7916304	148979	*GLIS family zinc finger 1*	*GLIS1*	8.51	8.61	6.95	0.001072116	0.006406729
7970325	6011	*G protein-coupled receptor kinase 1*	*GRK1*	6.98	7.12	6.43	0.001073055	0.006411015
7940673	2785, 26580	*guanine nucleotide binding protein (G protein), gamma 3 | Berardinelli-Seip congenital lipodystrophy 2 (seipin)*	*GNG3|BSCL2*	6.19	6.28	7.08	0.001074469	0.006416804
8098985	79441, 353497	*HAUS augmin-like complex, subunit 3 | polymerase (DNA directed) nu*	*HAUS3|POLN*	6.24	6.30	7.19	0.00107428	0.006416804
7919627	8370, 554313	*histone cluster 2, H4a | histone cluster 2, H4b*	*HIST2H4A|HIST2H4B*	8.10	8.47	9.76	0.001075543	0.006421894
7922343	7292	*tumor necrosis factor (ligand) superfamily, member 4*	*TNFSF4*	6.20	8.67	6.20	0.001075831	0.006422282
8060977	128710	*chromosome 20 open reading frame 94*	*C20orf94*	7.06	6.71	8.44	0.001076141	0.006422808
7931873	2665	*GDP dissociation inhibitor 2*	*GDI2*	11.54	11.48	12.56	0.001076538	0.006423849
8170891	2664	*GDP dissociation inhibitor 1*	*GDI1*	12.86	12.91	11.97	0.001077216	0.006426564

7912481	10459	*MAD2 mitotic arrest deficient-like 2 (yeast)*	*MAD2L2*	9.75	9.81	12.02	0.001077888	0.006427955
8084213				7.02	6.81	7.85	0.001077894	0.006427955
8088813	60675	*prokineticin 2*	*PROK2*	6.95	6.95	7.48	0.001078733	0.006431213
8091118				5.74	5.50	6.79	0.001078886	0.006431213
7943193				6.09	5.94	6.76	0.001080743	0.006439622
8014704				7.84	8.28	7.07	0.001080737	0.006439622
8031682				5.78	5.80	5.37	0.001081758	0.006444339
8180049	5696	*proteasome (prosome, macropain) subunit, beta type, 8 (large multifunctional peptidase 7)*	*PSMB8*	8.09	7.70	6.98	0.001082029	0.006444626
8049722	2859	*G protein-coupled receptor 35*	*GPR35*	6.92	6.97	6.43	0.00108275	0.006447588
8020495	91768	*Cdk5 and Abl enzyme substrate 1*	*CABLES1*	7.36	7.70	8.48	0.001083301	0.006449541
8009227	5705	*proteasome (prosome, macropain) 26S subunit, ATPase, 5*	*PSMC5*	8.54	8.61	9.42	0.001085444	0.006459633
7956573	65012, 2583	*solute carrier family 26, member 10 | beta-1,4-N-acetyl-galactosaminyl transferase 1*	*SLC26A10|B4GALNT1*	6.82	6.76	5.95	0.001085435	0.006459633
7998267	339123	*jumonji domain containing 8*	*JMJD8*	11.40	11.30	10.18	0.001085943	0.006461272
8178855	5696	*proteasome (prosome, macropain) subunit, beta type, 8 (large multifunctional peptidase 7)*	*PSMB8*	8.10	7.70	6.98	0.001087715	0.006469544
8058837	55825	*peroxisomal trans-2-enoyl-CoA reductase*	*PECR*	7.11	7.03	8.23	0.001087782	0.006469544
7992302	57585	*Crm, cramped-like (Drosophila)*	*CRAMP1L*	7.54	7.54	7.17	0.001089141	0.006476292
8052413				6.66	6.94	7.85	0.001089595	0.006477658
8143788				7.29	7.41	6.83	0.001090721	0.006483018
8098721				8.15	8.33	7.21	0.001090951	0.006483054
7949371	399904		*LOC399904*	7.80	8.05	7.52	0.00109155	0.006483087
8023252	6139, 26790	*ribosomal protein L17 | small nucleolar RNA, C/D box 58B*	*RPL17|SNORD58B*	7.52	7.50	8.47	0.001091631	0.006483087
7979127				8.21	8.06	7.64	0.001091603	0.006483087
8080121	51368	*testis expressed 264*	*TEX264*	9.40	9.41	7.99	0.001092985	0.006489795

8023154	728929, 162699, 51224	*transcription elongation factor B polypeptide 3C-like | transcription elongation factor B polypeptide 3C (elongin A3) | transcription elongation factor B polypeptide 3B (elongin A2)*	*TCEB3CL| TCEB3B*	7.33	7.39	6.62	0.001093281	0.006490219
7995330				10.11	10.10	9.27	0.001094531	0.006496301
8029536	341	*apolipoprotein C-I*	*APOC1*	6.48	6.57	7.81	0.001095432	0.006500311
8088602	9223	*membrane associated guanylate kinase, WW and PDZ domain containing 1*	*MAGI1*	8.20	8.52	10.23	0.001096198	0.006502183
8051746	80745	*THUMP domain containing 2*	*THUMPD 2*	6.87	6.84	8.48	0.001096025	0.006502183
7960919	8076	*microfibrillar associated protein 5*	*MFAP5*	11.34	12.55	7.42	0.001096935	0.006504507
7917156	8880	*far upstream element (FUSE) binding protein 1*	*FUBP1*	8.97	8.71	11.65	0.00109704	0.006504507
7933194	6387	*chemokine (C-X-C motif) ligand 12*	*CXCL12*	12.10	11.48	9.81	0.001097997	0.006508848
7953409	5763	*parathymosin*	*PTMS*	11.68	11.99	10.48	0.001098318	0.00650941
7927708				5.12	5.07	5.52	0.001098873	0.006510037
8071817				6.02	6.05	5.30	0.001098875	0.006510037
8116952				8.71	8.75	9.96	0.001099445	0.006512082
8030133	4924	*nucleobindin 1*	*NUCB1*	11.98	11.73	10.28	0.001099911	0.006513501
8049152	1144	*cholinergic receptor, nicotinic, delta*	*CHRND*	6.78	6.89	6.42	0.001100414	0.006515144
8001350	29117	*bromodomain containing 7*	*BRD7*	7.50	7.55	9.57	0.001101323	0.006519188
8153776	51160	*vacuolar protein sorting 28 homolog (S. cerevisiae)*	*VPS28*	11.25	11.32	9.63	0.00110159	0.006519436
7922910				4.91	4.76	4.72	0.001102294	0.006522263
8061847	140683	*chromosome 20 open reading frame 70*	*C20orf70*	6.10	5.94	5.54	0.001103547	0.006527001
7965478				8.36	8.72	7.17	0.001103413	0.006527001
8041902	3344	*forkhead box N2*	*FOXN2*	7.84	8.04	8.85	0.001105144	0.00653288
8066200	9675	*Tel2 interacting protein 1 homolog (S. pombe)*	*TTI1*	7.88	8.12	9.42	0.00110508	0.00653288
8091260	285195	*solute carrier family 9 (sodium/hydrogen exchanger), member 9*	*SLC9A9*	9.02	7.71	6.12	0.00110522	0.00653288
8171029	8263, 474383, 474384	*coagulation factor VIII-associated (intronic transcript) 1 | coagulation factor VIII-associated (intronic transcript) 2 | coagulation factor VIII-associated (intronic transcript) 3*	*F8A1|F8A 2|F8A3*	11.68	11.56	10.25	0.00110598	0.006536034

7956046	1606	*diacylglycerol kinase, alpha 80kDa*	*DGKA*	10.46	10.04	8.85	0.001106224	0.006536138
7965918	51559	*5'-nucleotidase domain containing 3*	*NT5DC3*	7.40	7.26	8.59	0.001107979	0.006545168
7917850	9411	*Rho GTPase activating protein 29*	*ARHGAP29*	9.29	8.02	7.23	0.001108792	0.006546344
7951093	143684	*family with sequence similarity 76, member B*	*FAM76B*	6.62	6.60	7.79	0.001108729	0.006546344
8006608	9560, 388372, 6351	*chemokine (C-C motif) ligand 4-like 1 | chemokine (C-C motif) ligand 4-like 2 | chemokine (C-C motif) ligand 4*	*CCL4L1|CCL4L2*	5.84	5.87	5.53	0.001108858	0.006546344
7928558	57178	*zinc finger, MIZ-type containing 1*	*ZMIZ1*	10.13	10.53	8.91	0.001109884	0.006547046
7944882	64221	*roundabout, axon guidance receptor, homolog 3 (Drosophila)*	*ROBO3*	7.16	7.09	6.41	0.001109824	0.006547046
7988838	123169	*Leo1, Paf1/RNA polymerase II complex component, homolog (S. cerevisiae)*	*LEO1*	7.09	7.15	8.33	0.001109562	0.006547046
7906475	343413	*Fc receptor-like 6*	*FCRL6*	6.24	6.28	5.87	0.001109546	0.006547046
8147864	9694	*tetratricopeptide repeat domain 35*	*TTC35*	9.11	8.35	9.58	0.001112686	0.006559054
8054866	84931		*LOC84931*	5.98	6.15	5.59	0.001112842	0.006559054
8147721	401472		*FLJ45248*	7.47	7.59	6.75	0.001113044	0.006559054
7967656				5.54	5.35	4.94	0.001113056	0.006559054
7969364				5.87	5.13	5.13	0.001112608	0.006559054
8151549	3612	*inositol(myo)-1(or 4)-monophosphatase 1*	*IMPA1*	7.65	7.81	9.10	0.001113414	0.006559825
8154447				5.53	5.82	5.29	0.001113966	0.006561741
7898916	57822	*grainyhead-like 3 (Drosophila)*	*GRHL3*	6.49	6.57	7.69	0.001114712	0.006564796
8121670				5.54	5.55	5.18	0.001115181	0.006566216
8086981	90226	*urocortin 2*	*UCN2*	7.58	7.57	6.51	0.001115875	0.006568962
8155497	100132948, 548321, 100133121	*family with sequence similarity 27, member C | family with sequence similarity 27, member A | family with sequence similarity 27, member B*	*FAM27C|FAM27A|FAM27B*	8.29	8.57	7.55	0.001116684	0.006571556
7906898				6.92	7.00	6.20	0.001116771	0.006571556
7918426	9122	*solute carrier family 16, member 4 (monocarboxylic acid transporter 5)*	*SLC16A4*	9.56	7.34	5.70	0.001118923	0.00658288
7928543				5.83	5.81	5.33	0.001121305	0.006595549
7993165				7.95	7.88	7.05	0.001121914	0.006597788

8068883	1409	*crystallin, alpha A*	*CRYAA*	8.51	8.71	7.60	0.001122546	0.006600159
7914342	2170	*fatty acid binding protein 3, muscle and heart (mammary-derived growth inhibitor)*	*FABP3*	7.51	6.90	8.95	0.001123551	0.006601991
8140151	5982	*replication factor C (activator 1) 2, 40kDa*	*RFC2*	8.71	9.13	11.02	0.001123772	0.006601991
8070689	11077	*heat shock transcription factor 2 binding protein*	*HSF2BP*	7.15	7.06	6.82	0.00112322	0.006601991
7914530	55108	*BSD domain containing 1*	*BSDC1*	9.79	9.96	8.93	0.001123564	0.006601991
8107970	3596	*interleukin 13*	*IL13*	6.63	6.62	5.96	0.001125035	0.006604389
8009334	27092	*calcium channel, voltage-dependent, gamma subunit 4*	*CACNG4*	7.10	7.25	7.76	0.001125096	0.006604389
8101957	51705	*endomucin*	*EMCN*	5.18	5.36	4.99	0.001124786	0.006604389
8177674	730394, 2966, 728340, 653238	*general transcription factor IIH, polypeptide 2D | general transcription factor IIH, polypeptide 2, 44kDa | general transcription factor IIH, polypeptide 2C | general transcription factor IIH, polypeptide 2B*	*GTF2H2D| GTF2H2C| GTF2H2B*	7.68	7.76	9.50	0.001124509	0.006604389
7924977	79605	*piggyBac transposable element derived 5*	*PGBD5*	6.09	6.22	7.15	0.001127442	0.006611442
8046333	79901	*cytochrome b reductase 1*	*CYBRD1*	12.26	11.71	8.55	0.001127217	0.006611442
7951467	91801	*alkB, alkylation repair homolog 8 (E. coli)*	*ALKBH8*	6.35	6.15	7.53	0.001126729	0.006611442
8077103	1890, 9997, 440836	*thymidine phosphorylase | SCO cytochrome oxidase deficient homolog 2 (yeast) | outer dense fiber of sperm tails 3B*	*TYMP|SCO2|ODF3B*	8.66	8.87	7.52	0.001127114	0.006611442
8099253				7.66	7.71	7.08	0.00112694	0.006611442
8114653	6879		*TAF7*	7.53	7.76	8.94	0.001129342	0.006621239
7938396	272	*adenosine monophosphate deaminase 3*	*AMPD3*	7.80	7.87	7.05	0.001129739	0.006622222
8066745	55713	*zinc finger protein 334*	*ZNF334*	6.50	6.19	8.17	0.001132112	0.006634783
8094408	29945	*anaphase promoting complex subunit 4*	*ANAPC4*	8.69	8.31	9.96	0.001132367	0.006634927
7931683	22982	*DIP2 disco-interacting protein 2 homolog C (Drosophila)*	*DIP2C*	8.86	8.96	7.85	0.001133188	0.006638392
7950501	81544	*glycerophosphodiester phosphodiesterase domain containing 5*	*GDPD5*	9.00	9.05	7.81	0.001134826	0.00664664
8161945	158158	*RAS and EF-hand domain containing*	*RASEF*	5.36	5.45	6.10	0.001135976	0.006652024
8153828	8928	*forkhead box H1*	*FOXH1*	7.49	7.47	9.04	0.001136735	0.006655124

8169073	286527, 767811, 286436	*thymosin beta 15B | H2B histone family, member X, pseudogene | H2B histone family, member M*	*TMSB15B| H2BFXP| H2BFM*	5.83	5.79	6.69	0.001137287	0.006657
8096463	56916	*SWI/SNF-related, matrix-associated actin-dependent regulator of chromatin, subfamily a, containing DEAD/H box 1*	*SMARCAD 1*	7.58	7.45	9.87	0.001138362	0.006660591
7990963				6.38	6.47	6.08	0.001138139	0.006660591
8078857	199223	*tetratricopeptide repeat domain 21A*	*TTC21A*	6.43	6.38	5.88	0.001138614	0.006660719
8154365				6.54	6.80	5.91	0.001139058	0.006661967
8086317	64689	*golgi reassembly stacking protein 1, 65kDa*	*GORASP1*	9.19	9.03	7.77	0.001139515	0.006663287
8007594	79089	*transmembrane and ubiquitin-like domain containing 2*	*TMUB2*	9.47	9.32	8.08	0.001140864	0.006668892
7972936	348013	*family with sequence similarity 70, member B*	*FAM70B*	9.32	9.47	7.36	0.001140935	0.006668892
7940187	219986	*olfactory receptor, family 4, subfamily D, member 11*	*OR4D11*	5.82	5.84	5.56	0.001144118	0.006686142
8007250	4669	*N-acetylglucosaminidase, alpha*	*NAGLU*	9.39	9.56	8.28	0.001144652	0.006687908
8007312	27175	*tubulin, gamma 2*	*TUBG2*	10.26	10.36	8.93	0.001145676	0.006692076
8161575	220869, 55871, 644019, 728013, 150472, 653510, 100507355	*COBW domain containing 5 | COBW domain containing 1 | COBW domain containing 6 | COBW domain containing 7 | COBW domain containing 2 | COBW domain-containing protein 5-like | hypothetical LOC100507355*	*CBWD5|CBWD1|CBWD6|CBWD7|CBWD2|LOC653510|LOC100507355*	6.00	6.20	7.65	0.001145975	0.006692076
8023152	728929, 162699, 51224	*transcription elongation factor B polypeptide 3C-like | transcription elongation factor B polypeptide 3C (elongin A3) | transcription elongation factor B polypeptide 3B (elongin A2)*	*TCEB3CL| TCEB3B*	7.12	7.18	6.43	0.001146061	0.006692076
7951734	9183		*ZW10*	6.74	6.82	8.47	0.001146674	0.006693809
8106921				5.88	6.00	5.41	0.001146821	0.006693809
8123364	1235	*chemokine (C-C motif) receptor 6*	*CCR6*	5.78	5.85	5.43	0.001148521	0.006701021
7944763				6.19	6.32	5.58	0.001148473	0.006701021
8131179	80727	*tweety homolog 3 (Drosophila)*	*TTYH3*	11.24	11.20	9.72	0.001149628	0.006706127
8074196	150160	*chaperonin containing TCP1, subunit 8 (theta)-like 2*	*CCT8L2*	7.82	7.64	6.95	0.001150138	0.006707091

8132707				6.77	6.85	6.24	0.001150258	0.006707091
7976412	256369	*chromosome 14 open reading frame 48*	*C14orf48*	6.04	6.18	5.72	0.001152008	0.006713902
8078916	574040	*small nucleolar RNA, H/ACA box 6*	*SNORA6*	7.14	7.29	8.38	0.001152002	0.006713902
7935228				5.01	4.53	6.04	0.001152124	0.006713902
7960150	7699	*zinc finger protein 140*	*ZNF140*	7.66	7.72	8.75	0.001152837	0.006715752
7920877	9181	*Rho/Rac guanine nucleotide exchange factor (GEF) 2*	*ARHGEF2*	10.55	10.91	8.78	0.001152907	0.006715752
7927801	305	*annexin A2 pseudogene 3*	*ANXA2P3*	7.17	7.37	6.31	0.001154116	0.006719081
7930837	6571	*solute carrier family 18 (vesicular monoamine), member 2*	*SLC18A2*	5.96	5.92	6.68	0.001154045	0.006719081
8031511	147807	*zinc finger protein 524*	*ZNF524*	8.27	8.46	7.24	0.001154401	0.006719081
8112182	166968	*mesoderm induction early response 1, family member 3*	*MIER3*	8.08	7.82	9.26	0.001154409	0.006719081
8052376	114800, 100129434	*coiled-coil domain containing 85A | hypothetical LOC100129434*	*CCDC85A| LOC100129434*	6.87	7.13	6.31	0.001155311	0.006722971
8062041	55902	*acyl-CoA synthetase short-chain family member 2*	*ACSS2*	9.34	8.83	7.49	0.001155989	0.006724211
7982593	400360	*chromosome 15 open reading frame 54*	*C15orf54*	5.03	5.27	4.87	0.00115593	0.006724211
8137250	26157	*GTPase, IMAP family member 2*	*GIMAP2*	6.24	5.74	4.99	0.001156779	0.006727449
8022767	22878	*trafficking protein particle complex 8*	*TRAPPC8*	7.19	6.94	8.80	0.001158536	0.006734954
7930194	54805	*cyclin M2*	*CNNM2*	9.28	8.03	8.89	0.001158481	0.006734954
8046283	2571	*glutamate decarboxylase 1 (brain, 67kDa)*	*GAD1*	6.48	6.50	6.16	0.001158874	0.006735563
8035868				4.92	5.11	6.38	0.001160559	0.006743996
8173627				5.30	5.16	6.17	0.001161005	0.00674523
8002802	79726	*WD repeat domain 59*	*WDR59*	8.84	8.66	9.66	0.00116373	0.006759703
8141328	1577	*cytochrome P450, family 3, subfamily A, polypeptide 5*	*CYP3A5*	6.00	5.98	5.55	0.001164802	0.006762372
8130102	11104	*katanin p60 (ATPase-containing) subunit A 1*	*KATNA1*	7.86	7.86	8.53	0.001164946	0.006762372
8105633	23398	*peptidylprolyl isomerase domain and WD repeat containing 1*	*PPWD1*	8.39	8.07	9.81	0.001165127	0.006762372
8108013				7.29	7.35	6.52	0.001165057	0.006762372
8165934	357	*shroom family member 2*	*SHROOM2*	6.95	7.05	7.58	0.001166641	0.006768202

7911578	728642, 984, 100294398, 100133692	*cyclin-dependent kinase 11A | cyclin-dependent kinase 11B | cell division protein kinase 11B-like*	*CDK11A|CDK11B|LOC100294398|LOC100133692*	8.21	7.92	8.53	0.001166834	0.006768202
7979876				8.97	8.92	8.21	0.00116677	0.006768202
7966098	9733	*squamous cell carcinoma antigen recognized by T cells 3*	*SART3*	7.96	8.08	9.63	0.001169685	0.006782472
8147777	115908	*collagen triple helix repeat containing 1*	*CTHRC1*	9.45	9.42	7.99	0.001169999	0.006782472
8170644	139735	*zinc finger protein 92 homolog (mouse)*	*ZFP92*	6.96	6.76	6.07	0.001169906	0.006782472
8166576	347541	*melanoma antigen family B, 5*	*MAGEB5*	5.58	5.60	5.28	0.001170541	0.006784252
8174322	79710	*MORC family CW-type zinc finger 4*	*MORC4*	8.26	7.88	6.33	0.001170785	0.006784302
8163729	406939	*microRNA 147*	*MIR147*	7.16	6.66	6.58	0.00117117	0.006784413
7995263	28424, 100290036	*immunoglobulin heavy variable 3-48 | similar to hCG2038926*	*IGHV3-48|LOC100290036*	8.07	8.25	7.65	0.001171274	0.006784413
8123524	55770	*exocyst complex component 2*	*EXOC2*	8.14	8.05	10.55	0.001171716	0.006785608
8086028	2720	*galactosidase, beta 1*	*GLB1*	11.38	11.30	10.29	0.00117242	0.006786963
8042737	7840	*Alstrom syndrome 1*	*ALMS1*	7.00	6.83	9.54	0.001172354	0.006786963
7913571	10236	*heterogeneous nuclear ribonucleoprotein R*	*HNRNPR*	8.46	8.33	10.70	0.001174485	0.006793472
8070341	54014	*bromodomain and WD repeat domain containing 1*	*BRWD1*	7.65	7.51	9.53	0.001174193	0.006793472
8014233	91607	*schlafen family member 11*	*SLFN11*	8.37	8.07	5.17	0.001174389	0.006793472
8023156	162699, 728929, 51224	*transcription elongation factor B polypeptide 3C (elongin A3) | transcription elongation factor B polypeptide 3C-like | transcription elongation factor B polypeptide 3B (elongin A2)*	*TCEB3C|TCEB3CL|TCEB3B*	7.56	7.58	6.78	0.001174474	0.006793472
7924686	163859	*chromosome 1 open reading frame 55*	*C1orf55*	7.32	7.80	8.68	0.001175531	0.006798157
8169049	9338	*transcription elongation factor A (SII)-like 1*	*TCEAL1*	7.77	7.71	6.96	0.001175994	0.006799474
8065421	1473	*cystatin D*	*CST5*	6.66	6.71	6.17	0.001177872	0.006808968
7898655	978	*cytidine deaminase*	*CDA*	7.07	8.89	7.90	0.001178665	0.006811365
7986385	23336	*synemin, intermediate filament protein*	*SYNM*	7.39	7.35	6.99	0.00117923	0.006811365
8098895	152877	*family with sequence similarity 53, member A*	*FAM53A*	7.90	8.02	7.29	0.001179213	0.006811365

8168841				4.46	4.59	4.50	0.001179022	0.006811365
7950671	9846	*GRB2-associated binding protein 2*	*GAB2*	8.67	8.94	7.91	0.001180406	0.00681679
8170292				4.51	4.47	4.40	0.00118298	0.006830288
8005475	147166, 10626	*tripartite motif-containing 16-like | tripartite motif-containing 16*	*TRIM16L*	11.49	10.67	8.56	0.001183604	0.006832528
8067303				7.88	7.83	7.29	0.001184908	0.006838685
8148049	4856	*nephroblastoma overexpressed gene*	*NOV*	9.38	8.82	7.35	0.001185171	0.006838835
7976055				5.05	5.16	4.84	0.001185424	0.006838928
8059731	5147	*phosphodiesterase 6D, cGMP-specific, rod, delta*	*PDE6D*	9.64	9.90	10.39	0.001188331	0.006854328
7928653	728118, 283008, 729262, 728130	*family with sequence similarity 22, member A | family with sequence similarity 22, member E | family with sequence similarity 22, member B | family with sequence similarity 22, member D*	*FAM22A|FAM22E|FAM22B|FAM22D*	7.60	7.73	7.06	0.001189181	0.006857865
8099406	579	*NK3 homeobox 2*	*NKX3-2*	7.68	7.75	7.06	0.001190315	0.006863032
7977674	11198	*suppressor of Ty 16 homolog (S. cerevisiae)*	*SUPT16H*	7.95	7.87	11.32	0.001191605	0.006868025
8120102	23607	*CD2-associated protein*	*CD2AP*	8.03	8.05	11.17	0.001191657	0.006868025
8016473	3218	*homeobox B8*	*HOXB8*	6.78	8.24	6.50	0.001193774	0.006876374
7928736	10718	*neuregulin 3*	*NRG3*	6.52	6.42	7.70	0.001193617	0.006876374
8022145	91133	*l(3)mbt-like 4 (Drosophila)*	*L3MBTL4*	5.87	5.78	6.33	0.00119382	0.006876374
8142424	54329	*G protein-coupled receptor 85*	*GPR85*	5.42	5.72	5.34	0.001194919	0.006881329
8101437	27235	*coenzyme Q2 homolog, prenyltransferase (yeast)*	*COQ2*	8.91	9.18	9.79	0.001195321	0.006882271
8108706	54661	*protocadherin beta 17 pseudogene*	*PCDHB17*	5.95	6.22	6.41	0.001196911	0.006889494
8040690	339778	*chromosome 2 open reading frame 70*	*C2orf70*	6.88	7.05	6.38	0.001197052	0.006889494
8119993	3326	*heat shock protein 90kDa alpha (cytosolic), class B member 1*	*HSP90AB1*	11.53	11.66	13.16	0.001197959	0.006893338
7901247	127343	*diencephalon/mesencephalon homeobox 1*	*DMBX1*	7.65	7.69	6.97	0.001199296	0.006899302
8008170				6.21	5.89	7.18	0.001199473	0.006899302
8176286	55344	*phosphatidylinositol-specific phospholipase C, X domain containing 1*	*PLCXD1*	7.59	7.48	8.99	0.00120086	0.006905051
8018258	124590	*Usher syndrome 1G (autosomal recessive)*	*USH1G*	7.06	7.07	6.59	0.001201669	0.006905051

8109161	389337	*Rho guanine nucleotide exchange factor (GEF) 37*	*ARHGEF37*	6.87	6.74	6.13	0.001201245	0.006905051
7954243				7.84	7.75	8.41	0.001201173	0.006905051
8071593				8.40	8.54	7.39	0.001201489	0.006905051
8044927	3625	*inhibin, beta B*	*INHBB*	7.95	8.62	7.40	0.001202358	0.006906597
7978909				8.01	7.95	7.13	0.001202416	0.006906597
7955578	160622	*GRP1 (general receptor for phosphoinositides 1)-associated scaffold protein*	*GRASP*	7.75	7.85	6.95	0.001203577	0.00691189
7908022	1660	*DEAH (Asp-Glu-Ala-His) box polypeptide 9*	*DHX9*	8.15	8.19	10.61	0.001207279	0.006929012
8125500	5696	*proteasome (prosome, macropain) subunit, beta type, 8 (large multifunctional peptidase 7)*	*PSMB8*	8.10	7.70	6.99	0.001206825	0.006929012
8151490				4.44	4.47	4.43	0.001207251	0.006929012
8139314	2645	*glucokinase (hexokinase 4)*	*GCK*	7.25	7.15	6.84	0.001210656	0.006940074
7992474	7249	*tuberous sclerosis 2*	*TSC2*	10.53	10.27	8.76	0.001209627	0.006940074
8150036	23303	*kinesin family member 13B*	*KIF13B*	6.91	6.80	7.63	0.001210651	0.006940074
7939559	90139	*tetraspanin 18*	*TSPAN18*	7.73	10.95	9.25	0.001210717	0.006940074
8165038	402381	*spermatogenesis and oogenesis specific basic helix-loop-helix 1*	*SOHLH1*	7.43	7.43	6.93	0.001210747	0.006940074
8162669	79692, 387328	*zinc finger protein 322A | zinc finger protein 322B*	*ZNF322A| ZNF322B*	6.77	6.39	8.32	0.001210889	0.006940074
8167893				7.02	7.29	6.30	0.001209759	0.006940074
8091731				6.15	6.41	5.78	0.001211715	0.006943428
8114780	51294	*protocadherin 12*	*PCDH12*	6.41	6.48	6.07	0.001213179	0.006946304
8122348	51534	*Vps20-associated 1 homolog (S. cerevisiae)*	*VTA1*	9.82	9.89	10.72	0.001212843	0.006946304
8052269	55704	*coiled-coil domain containing 88A*	*CCDC88A*	8.05	7.57	10.09	0.001212642	0.006946304
8157798				7.45	7.61	6.78	0.001213082	0.006946304
7991080	646	*basonuclin 1*	*BNC1*	6.79	8.24	6.85	0.001215154	0.006954111
8026564	10365	*Kruppel-like factor 2 (lung)*	*KLF2*	8.87	9.17	7.83	0.001215265	0.006954111
8152246				4.46	4.46	4.41	0.001214798	0.006954111
8079746	6988	*T-cell leukemia translocation altered gene*	*TCTA*	9.89	9.96	8.00	0.001215782	0.006954313
8009277	8787	*regulator of G-protein signaling 9*	*RGS9*	6.96	6.05	6.96	0.001215705	0.006954313

8020455	2627	*GATA binding protein 6*	*GATA6*	9.80	9.90	8.03	0.001216574	0.006957464
8130622				8.41	8.54	7.39	0.001217315	0.006960323
7980338	64207	*chromosome 14 open reading frame 4*	*C14orf4*	10.19	9.86	9.24	0.001218546	0.006965981
7982810	643338	*chromosome 15 open reading frame 62*	*C15orf62*	7.23	7.28	6.63	0.001220803	0.006976494
8005657				7.83	7.93	6.85	0.001220868	0.006976494
8159876	5991	*regulatory factor X, 3 (influences HLA class II expression)*	*RFX3*	6.87	6.37	8.31	0.001222906	0.006986755
8060594	2797	*gonadotropin-releasing hormone 2*	*GNRH2*	7.37	7.35	6.86	0.001223899	0.006991043
8046546	3234	*homeobox D8*	*HOXD8*	7.12	8.42	6.24	0.00122477	0.006994636
8144267	2055	*ceroid-lipofuscinosis, neuronal 8 (epilepsy, progressive with mental retardation)*	*CLN8*	7.76	7.98	8.54	0.001226762	0.007004624
8086352	54986	*unc-51-like kinase 4 (C. elegans)*	*ULK4*	6.72	6.74	7.67	0.001227135	0.007005369
7999916	124274	*G protein-coupled receptor 139*	*GPR139*	8.55	8.48	8.01	0.001228502	0.007011789
7898602	23252	*OTU domain containing 3*	*OTUD3*	6.41	6.45	6.98	0.001229373	0.007015372
8098822	1609	*diacylglycerol kinase, theta 110kDa*	*DGKQ*	8.22	8.38	7.51	0.001229871	0.007015728
7996593	10664	*CCCTC-binding factor (zinc finger protein)*	*CTCF*	8.55	8.78	10.07	0.001229921	0.007015728
8133442	7462	*linker for activation of T cells family, member 2*	*LAT2*	7.02	7.10	6.56	0.001231254	0.007020556
8104124	448831, 441581, 100288801	*FSHD region gene 2 | FSHD region gene 2 family, member B | FSHD region gene 2 family, member C*	*FRG2|FRG2B|FRG2C*	5.88	5.83	5.47	0.001231088	0.007020556
8167835	7216	*trophinin*	*TRO*	7.78	7.52	8.45	0.001232229	0.007022409
7993624	51760	*synaptotagmin XVII*	*SYT17*	6.94	6.97	8.50	0.001232049	0.007022409
8071355	150197		*LOC150197*	6.89	7.07	6.47	0.001232309	0.007022409
7924969	27097	*TAF5-like RNA polymerase II, p300/CBP-associated factor (PCAF)-associated factor, 65kDa*	*TAF5L*	8.04	8.38	9.14	0.00123402	0.007030245
8162624	195827	*chromosome 9 open reading frame 21*	*C9orf21*	9.44	9.56	7.96	0.001234171	0.007030245
8061357	5075	*paired box 1*	*PAX1*	7.16	7.15	6.58	0.001234933	0.007033195
8119184	9025	*ring finger protein 8*	*RNF8*	6.80	6.88	7.73	0.001236066	0.007038261
8157534	11064	*centrosomal protein 110kDa*	*CEP110*	6.11	5.84	7.10	0.001236746	0.007040747
8159265	5047	*progestagen-associated endometrial protein*	*PAEP*	6.96	7.01	6.29	0.001238011	0.007041005

8011222	23293	*Smg-6 homolog, nonsense mediated mRNA decay factor (C. elegans)*	*SMG6*	9.29	9.17	8.37	0.001237699	0.007041005
8127698	55023	*pleckstrin homology domain interacting protein*	*PHIP*	9.04	8.26	10.61	0.001237484	0.007041005
7990848	342125	*transmembrane channel-like 3*	*TMC3*	5.88	5.85	5.42	0.001237929	0.007041005
7972334				4.72	4.68	4.51	0.001237435	0.007041005
8127778	55603	*family with sequence similarity 46, member A*	*FAM46A*	9.89	9.90	8.29	0.001238286	0.00704118
8085060				5.42	5.48	4.99	0.001238557	0.007041333
8147439	79666	*pleckstrin homology domain containing, family F (with FYVE domain) member 2*	*PLEKHF2*	7.16	7.15	7.89	0.001239272	0.007044009
8179935	7148, 7146	*tenascin XB | tenascin XA pseudogene*	*TNXB|TNXA*	10.26	9.11	6.55	0.001239515	0.007044009
7964460	1649	*DNA-damage-inducible transcript 3*	*DDIT3*	9.64	10.31	7.68	0.001240287	0.007045623
8020919	83608	*chromosome 18 open reading frame 21*	*C18orf21*	7.43	7.66	8.82	0.001240142	0.007045623
8171917	53940	*ferritin, heavy polypeptide-like 17*	*FTHL17*	7.09	7.06	6.31	0.001240916	0.007047808
8067546	28231, 100127888	*solute carrier organic anion transporter family, member 4A1 | hypothetical LOC100127888*	*SLCO4A1|LOC100127888*	7.35	7.28	6.68	0.00124259	0.007055928
7944302	23187	*pleckstrin homology-like domain, family B, member 1*	*PHLDB1*	9.36	9.20	8.29	0.001243835	0.007061606
8095380	28983	*transmembrane protease, serine 11E*	*TMPRSS11E*	5.37	5.23	6.71	0.001244883	0.007065517
8037309	653583	*pleckstrin homology-like domain, family B, member 3*	*PHLDB3*	7.51	7.51	6.75	0.001245013	0.007065517
7919971	5993	*regulatory factor X, 5 (influences HLA class II expression)*	*RFX5*	8.24	8.00	9.09	0.001246256	0.007070651
8101013	25898	*ring finger and CHY zinc finger domain containing 1*	*RCHY1*	6.82	6.83	8.45	0.001246482	0.007070651
8105918	2966, 728340, 653238, 730394	*general transcription factor IIH, polypeptide 2, 44kDa | general transcription factor IIH, polypeptide 2C | general transcription factor IIH, polypeptide 2B | general transcription factor IIH, polypeptide 2D*	*GTF2H2|GTF2H2C|GTF2H2B|GTF2H2D*	7.68	7.74	9.52	0.001246653	0.007070651
7954785				5.58	5.74	5.25	0.001250425	0.007090653
8005141	54902	*tetratricopeptide repeat domain 19*	*TTC19*	7.92	8.20	10.27	0.001251344	0.007092588
8179750	7919, 534	*HLA-B associated transcript 1 | ATPase, H+ transporting, lysosomal 13kDa, V1 subunit G2*	*BAT1|ATP6V1G2*	12.48	12.49	13.41	0.001251503	0.007092588

7899909				7.34	7.18	7.99	0.001251447	0.007092588
8028004	55957	*lin-37 homolog (C. elegans)*	*LIN37*	10.69	10.70	8.57	0.001253733	0.007103832
7992463	9351	*solute carrier family 9 (sodium/hydrogen exchanger), member 3 regulator 2*	*SLC9A3R2*	10.32	10.14	8.29	0.001254567	0.007107162
8168117	407	*arrestin 3, retinal (X-arrestin)*	*ARR3*	5.94	5.97	5.51	0.001254857	0.007107411
7992337	90861, 57585	*hematological and neurological expressed 1-like | Crm, cramped-like (Drosophila)*	*HN1L|CRAMP1L*	9.02	9.13	10.61	0.001255463	0.007109449
8117106	255488	*ring finger protein 144B*	*RNF144B*	7.97	8.00	9.60	0.001255862	0.007110314
7945371	10410	*interferon induced transmembrane protein 3 (1-8U)*	*IFITM3*	13.46	13.42	12.26	0.001257048	0.007115629
8124166	154141	*membrane bound O-acyltransferase domain containing 1*	*MBOAT1*	9.50	7.58	10.09	0.001257559	0.007115731
8067867				7.75	7.70	7.44	0.001257468	0.007115731
8178712	7148, 7146	*tenascin XB | tenascin XA pseudogene*	*TNXB|TNXA*	10.26	9.12	6.55	0.001258803	0.007121378
8165285	138307	*lipocalin 8*	*LCN8*	7.15	7.12	6.44	0.001260842	0.007129357
8080973	151987	*protein phosphatase 4, regulatory subunit 2*	*PPP4R2*	7.70	7.61	9.20	0.001261081	0.007129357
7918900				7.47	6.89	6.84	0.001261202	0.007129357
8144526				6.82	6.86	5.71	0.001260769	0.007129357
8113512	64097	*erythrocyte membrane protein band 4.1 like 4A*	*EPB41L4A*	5.47	5.49	6.75	0.00126174	0.007131006
8067942	407055	*microRNA 99a*	*MIR99A*	6.76	7.19	5.97	0.001262351	0.007133063
8002209				7.15	7.06	6.48	0.00126305	0.007135612
7909689	56950	*SET and MYND domain containing 2*	*SMYD2*	8.19	8.57	7.35	0.001263934	0.007139213
8113691	285605	*DTW domain containing 2*	*DTWD2*	6.89	7.10	8.43	0.001265354	0.007144573
8152863				4.95	4.94	6.08	0.001265378	0.007144573
8169006	282808	*RAB40A, member RAS oncogene family-like*	*RAB40AL*	7.18	7.31	6.78	0.001266159	0.007147584
8079839	389119	*chromosome 3 open reading frame 54*	*C3orf54*	7.24	7.21	6.16	0.001266585	0.007148592
8094460	3516	*recombination signal binding protein for immunoglobulin kappa J region*	*RBPJ*	8.93	8.69	11.26	0.001267262	0.007151013
8174228	90843	*transcription elongation factor A (SII)-like 8*	*TCEAL8*	8.02	7.70	8.48	0.001268197	0.00715489
7990273	9399	*stomatin (EPB72)-like 1*	*STOML1*	7.91	8.13	6.88	0.00126884	0.007157118

7940486	54949	*succinate dehydrogenase complex assembly factor 2*	*SDHAF2*	9.90	9.83	10.65	0.001272524	0.007176498
8140504	9863	*membrane associated guanylate kinase, WW and PDZ domain containing 2*	*MAGI2*	7.98	7.54	8.82	0.001273231	0.007177683
7933413	728053, 399761, 728127, 642517	*BMS1 pseudogene 1 | BMS1 pseudogene 5 | ArfGAP with GTPase domain, ankyrin repeat and PH domain 10 | ArfGAP with GTPase domain, ankyrin repeat and PH domain 9*	*BMS1P1|BMS1P5|AGAP10|AGAP9*	7.16	7.07	8.74	0.001273013	0.007177683
8016932	4353	*myeloperoxidase*	*MPO*	6.35	6.35	5.95	0.001274328	0.007181062
8000906	92595	*zinc finger protein 764*	*ZNF764*	8.62	8.53	7.64	0.001274183	0.007181062
7911078	51029	*PPPDE peptidase domain containing 1*	*PPPDE1*	8.86	8.39	9.89	0.001276116	0.007186996
8066074	79980		*DSN1*	7.71	8.06	10.49	0.001276128	0.007186996
8024100	116444	*glutamate receptor, ionotropic, N-methyl-D-aspartate 3B*	*GRIN3B*	8.03	8.09	7.34	0.001275968	0.007186996
8016847	7706	*tripartite motif-containing 25*	*TRIM25*	10.82	10.47	9.63	0.001276771	0.007189214
7970241	2159	*coagulation factor X*	*F10*	11.60	9.66	7.24	0.001278162	0.007194241
8137232	155038	*GTPase, IMAP family member 8*	*GIMAP8*	5.70	5.70	5.46	0.001278136	0.007194241
8109141	134266	*GrpE-like 2, mitochondrial (E. coli)*	*GRPEL2*	9.36	9.48	11.21	0.001278635	0.0071955
7918134	276, 277, 278, 279, 280	*amylase, alpha 1A (salivary) | amylase, alpha 1B (salivary) | amylase, alpha 1C (salivary) | amylase, alpha 2A (pancreatic) | amylase, alpha 2B (pancreatic)*	*AMY1A|AMY1B|AMY1C|AMY2A|AMY2B*	5.54	5.87	5.41	0.001280424	0.007204165
8093518	2261	*fibroblast growth factor receptor 3*	*FGFR3*	7.70	7.68	8.98	0.001281189	0.007205656
8032863	56005	*chromosome 19 open reading frame 10*	*C19orf10*	10.37	10.72	8.91	0.00128103	0.007205656
8130163	353091	*retinoic acid early transcript 1G*	*RAET1G*	8.48	8.72	7.60	0.001284154	0.00722059
8098421				7.56	7.72	7.14	0.001284344	0.00722059
7983381	145645	*chromosome 15 open reading frame 43*	*C15orf43*	4.67	4.69	4.59	0.00128503	0.007223037
8025347	80131	*leucine rich repeat containing 8 family, member E*	*LRRC8E*	7.29	7.66	6.88	0.001285724	0.007225534
8163825	7185	*TNF receptor-associated factor 1*	*TRAF1*	6.50	6.43	5.85	0.001286744	0.007228452
8003007	114780	*polycystic kidney disease 1-like 2*	*PKD1L2*	6.99	6.82	6.61	0.001286558	0.007228452
8162006	80318	*G kinase anchoring protein 1*	*GKAP1*	4.98	4.94	5.59	0.001288114	0.007233915

7964642	283416	*chromosome 12 open reading frame 61*	*C12orf61*	7.78	7.78	7.10	0.001288218	0.007233915
7977497	57820	*cyclin B1 interacting protein 1*	*CCNB1IP1*	7.43	7.67	9.74	0.001289322	0.007238708
8034698	407010	*microRNA 23a*	*MIR23A*	7.01	7.46	6.49	0.001289842	0.007240219
8030848	147650	*non-protein coding RNA 85*	*NCRNA00 085*	7.53	7.61	6.94	0.001291896	0.007250342
7993281	92017	*sorting nexin 29*	*SNX29*	10.17	9.67	7.71	0.001292481	0.007250805
8149625				5.10	5.04	4.74	0.001292353	0.007250805
7938286	6157	*ribosomal protein L27a*	*RPL27A*	7.49	7.42	8.02	0.001292747	0.007250886
8012116	23399	*CTD nuclear envelope phosphatase 1*	*CTDNEP1*	11.16	11.06	9.87	0.001294137	0.00725727
8057506	2487	*frizzled-related protein*	*FRZB*	6.16	5.76	7.76	0.001294621	0.007258575
8083000	55179	*Fas apoptotic inhibitory molecule*	*FAIM*	7.22	7.27	7.67	0.001296319	0.007265273
7991399				6.12	6.09	5.50	0.001296241	0.007265273
8047038	5378		*PMS1*	8.72	8.78	10.20	0.001297106	0.007268275
7968344	241	*arachidonate 5-lipoxygenase-activating protein*	*ALOX5AP*	6.48	7.12	6.05	0.00129738	0.007268289
8098571	391723	*HES/HEY-like transcription factor*	*HELT*	7.31	7.38	6.56	0.001298116	0.007268289
8167693	9502, 728242	*X antigen family, member 2 | X antigen family, member 2B*	*XAGE2|XA GE2B*	6.08	5.98	5.59	0.00129801	0.007268289
8087780				6.66	7.11	5.64	0.001297741	0.007268289
8029236	1954	*multiple EGF-like-domains 8*	*MEGF8*	8.92	8.74	7.53	0.001298911	0.007271331
7997257	162239	*zinc finger protein 1 homolog (mouse)*	*ZFP1*	7.28	7.21	8.10	0.001299515	0.007273302
8140828	6717	*sorcin*	*SRI*	11.57	11.60	12.08	0.001300146	0.007275426
8052698	10438		*C1D*	8.13	8.52	9.10	0.001302412	0.007286693
8035884				7.17	7.15	6.54	0.001303889	0.007293541
8050176	9270	*integrin beta 1 binding protein 1*	*ITGB1BP1*	8.80	9.19	8.58	0.001305359	0.007300349
8114010	3659	*interferon regulatory factor 1*	*IRF1*	9.17	9.16	7.29	0.001306554	0.00730562
8077768				4.64	4.64	5.49	0.001307739	0.007310831

8176532	728137, 7258, 64591, 728395, 728403	*testis specific protein, Y-linked 3 | testis specific protein, Y-linked 1 | testis specific protein, Y-linked 4 | testis specific protein, Y-linked 2 | testis specific protein, Y-linked 8*	*TSPY3|TSPY1|TSPY4|TSPY2|TSPY8*	5.94	5.92	5.64	0.001308436	0.007313309
8162601	195828	*zinc finger protein 367*	*ZNF367*	6.72	6.89	7.94	0.001309837	0.007319721
7972932	650669	*hypothetical LOC650669*	*FLJ41484*	7.24	6.67	5.84	0.001310283	0.007320799
8029640	388552	*biogenesis of lysosomal organelles complex-1, subunit 3*	*BLOC1S3*	9.74	9.67	8.60	0.001311428	0.007325776
8027184	23383		*MAU2*	9.41	9.43	8.50	0.001312712	0.007331109
8030539	79735	*TBC1 domain family, member 17*	*TBC1D17*	9.87	9.77	8.68	0.001313121	0.007331109
8122634	389432	*sterile alpha motif domain containing 5*	*SAMD5*	6.58	6.34	5.92	0.001313144	0.007331109
8089034	83787	*armadillo repeat containing 10*	*ARMC10*	8.38	8.60	9.54	0.001314784	0.007338842
8078918	6044, 3921	*small nucleolar RNA, H/ACA box 62 | ribosomal protein SA*	*SNORA62|RPSA*	7.76	7.87	9.18	0.001317739	0.007353914
8023593	4160	*melanocortin 4 receptor*	*MC4R*	5.91	6.13	5.44	0.001318938	0.007356343
8093839	27065	*DNA segment on chromosome 4 (unique) 234 expressed sequence*	*D4S234E*	6.36	6.56	7.60	0.001318482	0.007356343
8038477	84335	*AKT1 substrate 1 (proline-rich)*	*AKT1S1*	10.43	10.49	8.87	0.001318863	0.007356343
7941062	55867	*solute carrier family 22 (organic anion/urate transporter), member 11*	*SLC22A11*	7.34	7.41	6.82	0.001319526	0.007358198
7944375	51399	*trafficking protein particle complex 4*	*TRAPPC4*	9.50	9.62	10.39	0.001319921	0.007358979
8101086	27163	*N-acylethanolamine acid amidase*	*NAAA*	9.25	9.10	7.72	0.001321648	0.007367187
8062134	8200, 554250	*growth differentiation factor 5 | growth differentiation factor 5 opposite strand*	*GDF5|GDF5OS*	6.21	6.38	5.80	0.001322758	0.00737195
8112045	11082	*endothelial cell-specific molecule 1*	*ESM1*	5.43	5.57	5.19	0.001323673	0.007375623
8057959	80055	*post-GPI attachment to proteins 1*	*PGAP1*	6.94	6.81	9.40	0.001323944	0.007375711
7962358	10138	*YY1 associated factor 2*	*YAF2*	7.32	7.64	6.22	0.001324437	0.007376717
7903440	276, 277, 278, 279, 280	*amylase, alpha 1A (salivary) | amylase, alpha 1B (salivary) | amylase, alpha 1C (salivary) | amylase, alpha 2A (pancreatic) | amylase, alpha 2B (pancreatic)*	*AMY1A|AMY1B|AMY1C|AMY2A|AMY2B*	5.54	5.85	5.40	0.001324636	0.007376717

7934074	6865	*tachykinin receptor 2*	*TACR2*	7.06	7.04	6.47	0.00132651	0.007385732
8158145				7.80	7.87	7.17	0.001326777	0.007385792
8005157	9487	*phosphatidylinositol glycan anchor biosynthesis, class L*	*PIGL*	7.32	7.17	7.80	0.001327611	0.007389009
8136235	1357	*carboxypeptidase A1 (pancreatic)*	*CPA1*	7.17	7.00	6.27	0.001329156	0.007396183
8063914	128826	*chromosome 20 open reading frame 166*	*C20orf166*	7.60	7.63	7.01	0.001329526	0.007396819
7988687	2553	*GA binding protein transcription factor, beta subunit 1*	*GABPB1*	7.77	7.90	9.05	0.001329797	0.007396899
7975616	83544	*dynein, axonemal, light chain 1*	*DNAL1*	5.79	5.88	6.44	0.00133016	0.007397492
7990914				6.39	6.47	6.09	0.001330532	0.007398139
7934906	59	*actin, alpha 2, smooth muscle, aorta*	*ACTA2*	12.28	13.27	8.91	0.00133219	0.007405932
8043322	64682, 100133898	*anaphase promoting complex subunit 1 | anaphase-promoting complex subunit 1-like*	*ANAPC1|LOC100133898*	7.09	7.08	9.99	0.001332747	0.0074076
8042532	25806	*ventral anterior homeobox 2*	*VAX2*	8.41	8.56	7.60	0.001334296	0.007414783
8082916	53833	*interleukin 20 receptor beta*	*IL20RB*	8.53	10.33	7.09	0.001335223	0.007418503
7941172	387778	*speedy homolog C (Xenopus laevis)*	*SPDYC*	7.82	7.85	7.16	0.001336547	0.007424431
8091922	151790, 5276	*WD repeat domain 49 | serpin peptidase inhibitor, clade I (pancpin), member 2*	*WDR49|SERPINI2*	5.68	5.53	6.15	0.001336886	0.007424888
7965206	55117	*solute carrier family 6 (neutral amino acid transporter), member 15*	*SLC6A15*	5.45	5.52	6.79	0.00133762	0.007425582
8077688	84818	*interleukin 17 receptor C*	*IL17RC*	8.85	8.45	7.52	0.001337761	0.007425582
7961998				7.21	7.28	6.61	0.001337783	0.007425582
8061445				7.27	7.47	6.68	0.001338293	0.007426984
8104141	153478	*pleckstrin homology domain containing, family G (with RhoGef domain) member 4B*	*PLEKHG4B*	7.24	7.14	7.91	0.00133879	0.007428316
8077238	728294	*D-2-hydroxyglutarate dehydrogenase*	*D2HGDH*	8.48	8.45	7.56	0.001339294	0.007429683
8170007	644538		*LOC644538*	7.54	7.60	6.99	0.001340381	0.007434287
8039078	112401	*baculoviral IAP repeat-containing 8*	*BIRC8*	5.47	5.73	5.32	0.001340826	0.007435327
8115543	1879	*early B-cell factor 1*	*EBF1*	9.92	9.79	6.47	0.001342044	0.007439225
8072587	6523	*solute carrier family 5 (sodium/glucose cotransporter), member 1*	*SLC5A1*	5.86	5.88	5.49	0.001341795	0.007439225

7958800	8550	*mitogen-activated protein kinase-activated protein kinase 5*	*MAPKAPK 5*	8.83	9.00	10.26	0.001342711	0.007440281
8037767	55228	*PNMA-like 1*	*PNMAL1*	6.94	7.00	7.50	0.001342751	0.007440281
8021623	8710	*serpin peptidase inhibitor, clade B (ovalbumin), member 7*	*SERPINB7*	5.34	7.33	4.86	0.001344463	0.00744834
8089472	90102, 100128035	*pleckstrin homology-like domain, family B, member 2 | VGSA5840*	*PHLDB2|LOC100128035*	6.71	6.74	6.14	0.001345339	0.007451761
8080781	54899	*PX domain containing serine/threonine kinase*	*PXK*	10.50	10.81	8.07	0.001345717	0.007452426
7955589	3164	*nuclear receptor subfamily 4, group A, member 1*	*NR4A1*	7.93	8.57	7.39	0.0013468	0.007454873
7975926	85457		*KIAA1737*	7.48	7.56	9.39	0.001346543	0.007454873
8099805				5.06	5.09	4.77	0.001346933	0.007454873
7955943	5153	*phosphodiesterase 1B, calmodulin-dependent*	*PDE1B*	6.64	6.71	7.06	0.001348156	0.00746021
7958439	84749	*ubiquitin specific peptidase 30*	*USP30*	9.95	9.78	8.62	0.001348706	0.007461825
8103494	4886	*neuropeptide Y receptor Y1*	*NPY1R*	5.00	5.00	7.11	0.001349398	0.007464218
8064245	6919	*transcription elongation factor A (SII), 2*	*TCEA2*	11.08	11.00	9.67	0.001350663	0.007469785
8054614	64682, 285074	*anaphase promoting complex subunit 1 | anaphase promoting complex subunit 1 pseudogene*	*ANAPC1|LOC285074*	7.91	8.07	10.68	0.001351474	0.007472841
7926916	6935	*zinc finger E-box binding homeobox 1*	*ZEB1*	10.24	9.69	6.18	0.001351875	0.007473628
7928529	84858	*zinc finger protein 503*	*ZNF503*	6.62	6.65	5.92	0.001355568	0.007488455
8092765	151963	*chromosome 3 open reading frame 59*	*C3orf59*	8.83	9.03	10.59	0.001355308	0.007488455
8144488	349196		*LOC349196*	10.03	9.10	7.65	0.001355595	0.007488455
8150032				6.17	6.03	5.69	0.001354963	0.007488455
7944720				6.80	6.96	6.20	0.00135762	0.007498208
8174496	9949	*Alport syndrome, mental retardation, midface hypoplasia and elliptocytosis chromosomal region gene 1*	*AMMECR 1*	8.65	8.36	9.53	0.001358469	0.007498708
8054217	10190	*thioredoxin domain containing 9*	*TXNDC9*	6.99	7.27	8.13	0.001358619	0.007498708
7960553	51258	*mitochondrial ribosomal protein L51*	*MRPL51*	12.36	12.46	12.90	0.001358666	0.007498708
7939052	387758	*fin bud initiation factor homolog (zebrafish)*	*FIBIN*	7.96	10.71	5.90	0.00135875	0.007498708

7943036	57093, 653111, 283257	*tripartite motif-containing 49 | tripartite motif-containing 49-like 2 | tripartite motif-containing protein 49B-like*	*TRIM49|TRIM49L2|TRIM49B*	5.57	5.62	6.17	0.001359173	0.007499612
7950003	116512	*MAS-related GPR, member D*	*MRGPRD*	6.70	7.06	6.36	0.001359563	0.007500331
8135697	56311	*ankyrin repeat domain 7*	*ANKRD7*	4.75	4.77	5.18	0.00136294	0.007517521
7917240	1486	*chitobiase, di-N-acetyl-*	*CTBS*	9.32	9.25	7.64	0.001364417	0.007524233
8166712				8.38	8.41	7.67	0.001365453	0.007528508
7938758	6288	*serum amyloid A1*	*SAA1*	4.99	4.84	4.54	0.001366029	0.007530242
8054166	80705	*testis specific, 10*	*TSGA10*	5.38	5.30	5.79	0.001366792	0.00753257
8056959	344191	*even-skipped homeobox 2*	*EVX2*	7.73	7.83	7.15	0.001366973	0.00753257
8172333	5199	*complement factor properdin*	*CFP*	7.05	7.00	6.60	0.001367409	0.007532633
7912257	22883	*calsyntenin 1*	*CLSTN1*	10.50	10.35	8.99	0.001367506	0.007532633
7986532	390649	*olfactory receptor, family 4, subfamily F, member 15*	*OR4F15*	5.11	5.11	4.92	0.001370454	0.007547434
8124459	79692, 387328	*zinc finger protein 322A | zinc finger protein 322B*	*ZNF322A|ZNF322B*	6.96	6.55	8.47	0.001370829	0.007548055
8103745	9464	*heart and neural crest derivatives expressed 2*	*HAND2*	7.43	7.79	6.46	0.001371787	0.007551875
8105970	2966, 728340, 730394, 653238	*general transcription factor IIH, polypeptide 2, 44kDa | general transcription factor IIH, polypeptide 2C | general transcription factor IIH, polypeptide 2D | general transcription factor IIH, polypeptide 2B*	*GTF2H2|GTF2H2C|GTF2H2D|GTF2H2B*	7.65	7.73	9.56	0.001372307	0.007551875
7914212	677838, 85028	*small nucleolar RNA, H/ACA box 61 | small nucleolar RNA host gene 12 (non-protein coding)*	*SNORA61|SNHG12*	9.95	10.26	11.37	0.001372097	0.007551875
8088425	131177	*family with sequence similarity 3, member D*	*FAM3D*	7.23	7.22	6.58	0.001373694	0.007554365
8027213	374887, 51079	*YjeF N-terminal domain containing 3 | NADH dehydrogenase (ubiquinone) 1 alpha subcomplex, 13*	*YJEFN3|NDUFA13*	8.26	8.23	7.51	0.001373261	0.007554365
8122717	84918, 353091	*low density lipoprotein receptor-related protein 11 | retinoic acid early transcript 1G*	*LRP11|RAET1G*	7.60	7.71	6.74	0.001373614	0.007554365
7902787				6.19	6.37	7.05	0.001373806	0.007554365
8075477	91445	*ring finger protein 185*	*RNF185*	6.59	6.98	5.65	0.00137431	0.007555695
7962579	347902	*adhesion molecule with Ig-like domain 2*	*AMIGO2*	8.19	8.77	5.95	0.001376137	0.007564299
7912515	4878	*natriuretic peptide A*	*NPPA*	6.13	6.09	5.67	0.001376564	0.007565057

8164766	158067	*chromosome 9 open reading frame 98*	*C9orf98*	7.68	7.29	6.79	0.001376799	0.007565057
8145736	3084	*neuregulin 1*	*NRG1*	7.60	8.65	6.95	0.001378366	0.007572226
8018156	283982	*chromosome 17 open reading frame 54*	*C17orf54*	5.91	5.78	5.51	0.001378832	0.007573344
7995007	80270	*hydroxy-delta-5-steroid dehydrogenase, 3 beta- and steroid delta-isomerase 7*	*HSD3B7*	9.61	10.03	8.00	0.001379653	0.007576413
7975793	10538	*basic leucine zipper transcription factor, ATF-like*	*BATF*	7.29	7.41	6.80	0.001380623	0.007580299
7993776	81691	*exonuclease NEF-sp*	*LOC81691*	6.96	6.96	7.74	0.001381067	0.007581291
7947274	744	*metallophosphoesterase domain containing 2*	*MPPED2*	5.74	6.95	9.19	0.001382451	0.00758745
8165255				7.05	7.02	6.57	0.001383916	0.007594047
7923534	4608	*myosin binding protein H*	*MYBPH*	7.51	7.48	7.03	0.001384732	0.007597079
7903425	276, 277, 278, 279, 280	*amylase, alpha 1A (salivary) | amylase, alpha 1B (salivary) | amylase, alpha 1C (salivary) | amylase, alpha 2A (pancreatic) | amylase, alpha 2B (pancreatic)*	*AMY1A|AMY1B|AMY1C|AMY2A|AMY2B*	5.54	5.86	5.40	0.001384999	0.007597101
8178476	7919, 534	*HLA-B associated transcript 1 | ATPase, H+ transporting, lysosomal 13kDa, V1 subunit G2*	*BAT1|ATP6V1G2*	12.45	12.48	13.41	0.001389853	0.007622279
8168622	56062	*kelch-like 4 (Drosophila)*	*KLHL4*	5.09	5.13	6.96	0.001391957	0.007632367
7995668	10265	*iroquois homeobox 5*	*IRX5*	8.59	8.78	7.52	0.001393018	0.007636735
7975095	26030	*pleckstrin homology domain containing, family G (with RhoGef domain) member 3*	*PLEKHG3*	7.14	7.00	7.50	0.00139404	0.007640883
8028172	826	*calpain, small subunit 1*	*CAPNS1*	13.07	13.13	12.30	0.001394358	0.007641178
8164931	389827	*transmembrane protein 8C*	*TMEM8C*	6.75	6.67	6.28	0.0013961	0.007649269
8066579	140686	*WAP four-disulfide core domain 3*	*WFDC3*	6.64	6.42	6.11	0.001396368	0.007649288
8001971	26231	*leucine rich repeat containing 29*	*LRRC29*	7.18	7.19	6.42	0.001398484	0.007659424
7971071	122011	*casein kinase 1, alpha 1-like*	*CSNK1A1L*	7.40	7.48	6.87	0.001398874	0.007660108
8065082				6.19	6.42	5.76	0.001399917	0.007664366
8056792				10.19	10.72	8.98	0.001400202	0.007664475
7917906	6129	*ribosomal protein L7*	*RPL7*	12.29	12.17	12.70	0.001400979	0.007665818
8146379	7336	*ubiquitin-conjugating enzyme E2 variant 2*	*UBE2V2*	7.51	7.50	8.57	0.001400852	0.007665818

8094609	92689	*family with sequence similarity 114, member A1*	*FAM114A1*	10.95	11.04	8.37	0.001403869	0.007680176
8117176	100129307, 390414	*UPF0607 protein ENSP00000383144-like | hypothetical LOC390414*	*LOC100129307|LOC390414*	6.77	6.86	5.95	0.001404423	0.007681752
7907439	9588	*peroxiredoxin 6*	*PRDX6*	11.62	12.51	12.67	0.001404845	0.007682602
7955361	113251	*La ribonucleoprotein domain family, member 4*	*LARP4*	8.75	8.26	11.05	0.001407159	0.007693803
8150214	11160	*ER lipid raft associated 2*	*ERLIN2*	7.64	7.64	7.03	0.001408008	0.007696987
8126086	221468	*transmembrane protein 217*	*TMEM217*	6.20	6.45	5.80	0.001408504	0.007698242
8151281	23471	*translocation associated membrane protein 1*	*TRAM1*	11.71	11.18	10.43	0.001409036	0.007699688
8043572				6.00	6.04	5.67	0.001409354	0.007699967
7985488	83640	*family with sequence similarity 103, member A1*	*FAM103A1*	8.18	8.31	9.69	0.001409825	0.007701084
8104738	10923		*SUB1*	10.55	10.58	11.50	0.001411352	0.007707971
8159815	55871, 150472, 445571, 220869, 644019, 728013, 653510, 100507355	*COBW domain containing 1 | COBW domain containing 2 | COBW domain containing 3 | COBW domain containing 5 | COBW domain containing 6 | COBW domain containing 7 | COBW domain-containing protein 5-like | hypothetical LOC100507355*	*CBWD1|CBWD2|CBWD3|CBWD5|CBWD6|CBWD7|LOC653510|LOC100507355*	7.29	7.44	9.26	0.001411684	0.00770807
8124926	7919, 534	*HLA-B associated transcript 1 | ATPase, H+ transporting, lysosomal 13kDa, V1 subunit G2*	*BAT1|ATP6V1G2*	12.44	12.47	13.41	0.001411905	0.00770807
7947462	25841	*ankyrin repeat and BTB (POZ) domain containing 2*	*ABTB2*	7.55	8.61	7.54	0.001412386	0.007709242
7966259	51228	*glycolipid transfer protein*	*GLTP*	9.31	9.67	8.24	0.001414926	0.007720185
7990452				7.04	7.13	6.75	0.001414861	0.007720185
8167261	6759, 548313	*synovial sarcoma, X breakpoint 4 | synovial sarcoma, X breakpoint 4B*	*SSX4|SSX4B*	5.81	5.85	6.56	0.001415556	0.007722161
7930304	9446	*glutathione S-transferase omega 1*	*GSTO1*	8.90	9.04	8.13	0.001417771	0.007731336
7927723	219621	*chromosome 10 open reading frame 107*	*C10orf107*	7.87	6.06	4.71	0.001417773	0.007731336
7992269				8.22	8.24	7.47	0.001418901	0.007736026

7983704	342035	*gliomedin*	*GLDN*	7.36	6.04	5.78	0.001419212	0.007736262
8170834	8277	*transketolase-like 1*	*TKTL1*	6.29	6.41	6.01	0.00141975	0.007737732
8093643	118	*adducin 1 (alpha)*	*ADD1*	10.87	10.60	9.66	0.0014202	0.007738721
8067155				6.15	6.13	5.68	0.001421124	0.007742295
8050713	375189	*profilin family, member 4*	*PFN4*	6.73	6.65	6.12	0.001422589	0.007748817
8048205	3485	*insulin-like growth factor binding protein 2, 36kDa*	*IGFBP2*	11.65	8.43	10.84	0.001423631	0.007753026
8002878	124491	*transmembrane protein 170A*	*TMEM170 A*	9.48	9.54	11.92	0.001426159	0.007765329
7973377	599	*BCL2-like 2*	*BCL2L2*	7.36	7.70	6.77	0.001428043	0.007774123
8153304	203062	*t-SNARE domain containing 1*	*TSNARE1*	7.65	7.63	7.19	0.001429149	0.007778677
8085276	152302, 63924	*cell death-inducing DFFA-like effector c pseudogene*	*CIDECP*	7.79	8.11	7.08	0.001429906	0.00778133
7968132	9818	*nucleoporin like 1*	*NUPL1*	7.73	7.88	9.94	0.00143025	0.007781735
8084704	1974	*eukaryotic translation initiation factor 4A2*	*EIF4A2*	7.00	7.45	8.87	0.001430541	0.007781853
8180003	3125	*major histocompatibility complex, class II, DR beta 3*	*HLA-DRB3*	6.65	6.56	7.67	0.001431658	0.007786458
7975224	1965	*eukaryotic translation initiation factor 2, subunit 1 alpha, 35kDa*	*EIF2S1*	9.22	9.42	10.60	0.001435058	0.007790274
7997735	2300	*forkhead box L1*	*FOXL1*	7.75	7.52	6.83	0.001434114	0.007790274
7908459	3075	*complement factor H*	*CFH*	8.33	7.08	5.13	0.001434127	0.007790274
8152477	5885		*RAD21*	8.37	8.61	10.25	0.001433109	0.007790274
8098904	7884	*stem-loop binding protein*	*SLBP*	8.98	9.19	9.38	0.001433363	0.007790274
8172538	11152	*WD repeat domain 45*	*WDR45*	9.49	9.46	7.96	0.001434825	0.007790274
7933659	23283	*cleavage stimulation factor, 3' pre-RNA, subunit 2, 64kDa, tau variant*	*CSTF2T*	7.93	8.05	9.22	0.001433595	0.007790274
7905324	54964	*chromosome 1 open reading frame 56*	*C1orf56*	10.05	9.66	8.65	0.001433038	0.007790274
7977296	196872	*hypothetical LOC196872*	*MGC2327 0*	6.81	6.68	6.11	0.0014346	0.007790274
8031514	100133142	*zinc finger protein 865*	*ZNF865*	8.91	9.02	7.92	0.00143495	0.007790274
7966301	51434	*anaphase promoting complex subunit 7*	*ANAPC7*	8.99	8.96	10.86	0.001436757	0.007792562
8009430	440461	*SH3 domain containing 20 pseudogene*	*LOC44046 1*	8.33	8.34	7.59	0.001436661	0.007792562

8039017	125893, 399669	*zinc finger protein 816 | zinc finger protein 321*	*ZNF816|ZNF321*	6.73	6.48	8.00	0.001436055	0.007792562
8075423	150290, 339665	*dual specificity phosphatase 18 | solute carrier family 35, member E4*	*DUSP18|SLC35E4*	8.11	8.26	6.99	0.001436365	0.007792562
8112613				7.08	7.21	6.55	0.001436829	0.007792562
8081820				6.85	6.88	6.36	0.001437638	0.007795486
8049007	130560	*spermatogenesis associated 3*	*SPATA3*	7.15	7.13	6.47	0.001438337	0.007797313
8068397				6.07	6.28	7.41	0.001438515	0.007797313
7964602	121227	*leucine-rich repeats and immunoglobulin-like domains 3*	*LRIG3*	10.25	8.89	7.34	0.001440303	0.007805056
7919606	440689, 337875, 8349	*histone cluster 2, H2bf | histone cluster 2, H2ba | histone cluster 2, H2be*	*HIST2H2BF|HIST2H2BA|HIST2H2BE*	10.13	9.99	11.01	0.001440484	0.007805056
8073309	100288034		*LOC100288034*	8.49	8.46	7.95	0.001442628	0.007815206
8137709	90637	*zinc finger, AN1-type domain 2A*	*ZFAND2A*	10.13	10.71	9.28	0.001443448	0.007818179
8059689	4691	*nucleolin*	*NCL*	9.32	9.51	11.71	0.001445265	0.00782494
8081612	55347	*abhydrolase domain containing 10*	*ABHD10*	8.01	7.66	8.68	0.001445509	0.00782494
8098712				6.88	7.11	6.37	0.001445455	0.00782494
8061416	8530	*cystatin F (leukocystatin)*	*CST7*	7.78	7.78	7.11	0.001446272	0.007826134
7950654	79053	*asparagine-linked glycosylation 8, alpha-1,3-glucosyltransferase homolog (S. cerevisiae)*	*ALG8*	10.04	10.15	11.66	0.001446197	0.007826134
7963577	84926	*SPRY domain containing 3*	*SPRYD3*	9.62	9.86	7.71	0.001448076	0.007832957
8098732	441056, 22947, 728410, 653548, 653545, 653544, 653543, 26583	*double homeobox 4 like 4 | double homeobox 4 like 2 | double homeobox 4 like 3 | double homeobox 4 like 5 | double homeobox 4 like 6 | double homeobox 4 like 7 | double homeobox 2*	*DUX4L4|DUX4L2|DUX4L3|DUX4L5|DUX4L6|DUX4L7|DUX2*	8.54	8.57	7.86	0.001447918	0.007832957
8024485	4616	*growth arrest and DNA-damage-inducible, beta*	*GADD45B*	9.11	10.28	7.81	0.001448597	0.007834308

8144457	140596, 503618	*defensin, beta 104A | defensin, beta 104B*	*DEFB104A|DEFB104B*	5.52	5.60	5.25	0.001449362	0.007836977
7923810				5.09	5.03	5.85	0.001449914	0.007838493
7979551	5529	*protein phosphatase 2, regulatory subunit B', epsilon isoform*	*PPP2R5E*	9.04	9.10	9.99	0.00145065	0.007841005
7946019	119678	*olfactory receptor, family 52, subfamily E, member 2*	*OR52E2*	5.69	5.56	5.41	0.001452554	0.007848361
8025285	199675	*chromosome 19 open reading frame 59*	*C19orf59*	6.69	6.76	6.34	0.001452303	0.007848361
8131831	11097	*nucleoporin like 2*	*NUPL2*	7.33	7.34	8.94	0.001453104	0.007848678
7985402	100133144, 388152, 80154, 727849, 100134869, 388165	*ubiquitin-conjugating enzyme E2Q family member 2 pseudogene 3 | hypothetical LOC388152 | hypothetical LOC80154 | golgin A2 pseudogene | ubiquitin-conjugating enzyme E2Q family member 2 pseudogene 2 | ubiquitin-conjugating enzyme E2Q family member 2 pseudogene 1*	*UBE2Q2P3|LOC388152|LOC80154|LOC727849|UBE2Q2P2|UBE2Q2P1*	6.51	6.74	7.04	0.001453157	0.007848678
7996563	3291	*hydroxysteroid (11-beta) dehydrogenase 2*	*HSD11B2*	6.96	7.11	8.07	0.001453986	0.007850228
8130173	154064	*retinoic acid early transcript 1L*	*RAET1L*	7.41	7.61	6.82	0.001454234	0.007850228
8005865	23098, 147007	*sterile alpha and TIR motif containing 1 | transmembrane protein 199*	*SARM1|TMEM199*	8.61	8.44	7.15	0.00145426	0.007850228
7901460	2882	*glutathione peroxidase 7*	*GPX7*	11.36	11.20	9.61	0.001455918	0.007857708
7897663	23435	*TAR DNA binding protein*	*TARDBP*	8.73	8.77	10.33	0.001456211	0.007857821
8033433	339390	*C-type lectin domain family 4, member G*	*CLEC4G*	7.73	7.82	7.50	0.001457009	0.007860661
8113023	153396	*transmembrane protein 161B*	*TMEM161B*	8.72	8.62	10.28	0.001457888	0.00786393
8110841	79888	*lysophosphatidylcholine acyltransferase 1*	*LPCAT1*	9.47	9.63	10.63	0.001459349	0.007870342
8140942	257415, 728066, 728640	*family with sequence similarity 133, member B | family with sequence similarity 133, member B pseudogene*	*FAM133B|LOC728066|LOC728640*	7.26	7.13	8.97	0.001460523	0.007875202
7920875	677771	*small Cajal body-specific RNA 4*	*SCARNA4*	6.74	7.30	7.63	0.001462386	0.007883775
8028791	23646	*phospholipase D family, member 3*	*PLD3*	12.26	12.20	11.13	0.001462907	0.007884518

8177507	2966, 728340, 653238, 730394	*general transcription factor IIH, polypeptide 2, 44kDa | general transcription factor IIH, polypeptide 2C | general transcription factor IIH, polypeptide 2B | general transcription factor IIH, polypeptide 2D*	*GTF2H2|GTF2H2C|GTF2H2B|GTF2H2D*	7.70	7.73	9.51	0.00146307	0.007884518
8098348	80817, 26269	*KIAA1712 | F-box protein 8*	*KIAA1712|FBXO8*	5.54	5.61	7.05	0.001463412	0.007884891
8161587	445571, 55871, 220869, 150472, 644019, 728013, 653510, 100507355	*COBW domain containing 3 | COBW domain containing 1 | COBW domain containing 5 | COBW domain containing 2 | COBW domain containing 6 | COBW domain containing 7 | COBW domain-containing protein 5-like | hypothetical LOC100507355*	*CBWD3|CBWD1|CBWD5|CBWD2|CBWD6|CBWD7|LOC653510|LOC100507355*	7.37	7.56	9.35	0.001464043	0.007886816
8067206	140690	*CCCTC-binding factor (zinc finger protein)-like*	*CTCFL*	7.12	7.39	8.16	0.001464486	0.007887734
7980001				5.94	5.92	5.56	0.001465137	0.007889766
8021914	22850	*ADNP homeobox 2*	*ADNP2*	7.56	7.52	9.51	0.001466437	0.007895295
8076690	23313	*chromosome 22 open reading frame 9*	*C22orf9*	10.80	10.16	9.41	0.001467796	0.007899665
8065948	80307	*fer-1-like 4 (C. elegans) pseudogene*	*FER1L4*	7.48	7.55	6.63	0.001467648	0.007899665
8049684	285193	*dual specificity phosphatase 28*	*DUSP28*	7.42	7.43	6.72	0.001468625	0.007902652
7996211	64785	*GINS complex subunit 3 (Psf3 homolog)*	*GINS3*	7.26	7.55	8.86	0.00146926	0.007904599
8104350	23379		*KIAA0947*	7.47	7.40	9.47	0.001470517	0.007909888
8077503	859	*caveolin 3*	*CAV3*	7.13	7.27	6.40	0.001471944	0.007916086
8114593	10307	*amyloid beta (A4) precursor protein-binding, family B, member 3*	*APBB3*	9.25	9.10	8.07	0.001473297	0.007921885
8112538	2966, 728340, 653238, 730394	*general transcription factor IIH, polypeptide 2, 44kDa | general transcription factor IIH, polypeptide 2C | general transcription factor IIH, polypeptide 2B | general transcription factor IIH, polypeptide 2D*	*GTF2H2|GTF2H2C|GTF2H2B|GTF2H2D*	7.70	7.73	9.52	0.001473746	0.007922825
7955290	359	*aquaporin 2 (collecting duct)*	*AQP2*	7.31	7.40	6.70	0.001474434	0.007925047
7951612	143903	*layilin*	*LAYN*	7.94	7.73	6.79	0.001475015	0.007926698

8170716	5365	*plexin B3*	*PLXNB3*	7.89	8.23	6.97	0.001478253	0.007940712
8054395	80146	*UDP-glucuronate decarboxylase 1*	*UXS1*	8.40	8.81	10.42	0.001478448	0.007940712
7964577				7.80	7.33	6.25	0.0014784	0.007940712
8092165	2693	*growth hormone secretagogue receptor*	*GHSR*	7.07	7.19	6.52	0.001479103	0.007942251
8029299				6.68	6.75	6.15	0.001479285	0.007942251
7930482	150	*adrenergic, alpha-2A-, receptor*	*ADRA2A*	8.48	9.66	7.99	0.001480344	0.007945669
7974793	4990	*SIX homeobox 6*	*SIX6*	8.08	8.13	7.29	0.001480935	0.007945669
7989968	91860	*calmodulin-like 4*	*CALML4*	6.53	6.56	7.10	0.001481023	0.007945669
8142431	401397		*LOC401397*	8.31	8.60	9.18	0.00148086	0.007945669
8093343	54872	*phosphatidylinositol glycan anchor biosynthesis, class G*	*PIGG*	8.68	8.83	8.22	0.001481875	0.007945814
8116807	154007	*small nuclear ribonucleoprotein 48kDa (U11/U12)*	*SNRNP48*	6.76	6.95	9.06	0.001481368	0.007945814
8018189	342510	*CD300e molecule*	*CD300E*	7.30	7.36	6.76	0.001481745	0.007945814
7955817	5094	*poly(rC) binding protein 2*	*PCBP2*	12.99	12.82	12.64	0.001483681	0.007954016
7898663	65018	*PTEN induced putative kinase 1*	*PINK1*	9.29	9.54	8.09	0.001484266	0.007955679
7915543	6536	*solute carrier family 6 (neurotransmitter transporter, glycine), member 9*	*SLC6A9*	10.87	10.91	8.36	0.00148479	0.007957008
8066189	2691	*growth hormone releasing hormone*	*GHRH*	6.36	6.41	6.00	0.001485161	0.007957519
7945712	7054	*tyrosine hydroxylase*	*TH*	7.09	7.12	6.57	0.001485457	0.007957629
8161520	595135, 5239	*phosphoglucomutase 5 pseudogene 2*	*PGM5P2*	7.23	6.74	5.77	0.001486278	0.007960547
8115939				6.80	6.97	6.39	0.001486896	0.007962383
8161503	548321, 100133121, 100132948	*family with sequence similarity 27, member A | family with sequence similarity 27, member B | family with sequence similarity 27, member C*	*FAM27A|FAM27B|FAM27C*	8.63	8.89	7.91	0.001487775	0.00796561
8107920	6584	*solute carrier family 22 (organic cation/carnitine transporter), member 5*	*SLC22A5*	8.44	7.92	7.38	0.001488762	0.007969417
7960984	5858	*pregnancy-zone protein*	*PZP*	5.63	5.63	5.44	0.001490836	0.007973129
7897416	50651	*solute carrier family 45, member 1*	*SLC45A1*	7.80	7.33	6.78	0.001490128	0.007973129
7906223	51093	*chromosome 1 open reading frame 66*	*C1orf66*	9.32	9.18	8.34	0.00149071	0.007973129

7946001	119695	*olfactory receptor, family 52, subfamily R, member 1*	*OR52R1*	6.44	6.40	5.97	0.001490618	0.007973129
8176419	64591, 728137, 7258, 728395, 728403	*testis specific protein, Y-linked 2 | testis specific protein, Y-linked 3 | testis specific protein, Y-linked 1 | testis specific protein, Y-linked 4 | testis specific protein, Y-linked 8*	*TSPY2|TSPY3|TSPY1|TSPY4|TSPY8*	6.38	6.36	6.08	0.001489839	0.007973129
7972890	79774	*growth hormone regulated TBC protein 1*	*GRTP1*	7.86	7.47	8.65	0.00149151	0.007975255
8105136	7690	*zinc finger protein 131*	*ZNF131*	7.88	7.96	9.22	0.001491793	0.007975291
8141361	81392	*olfactory receptor, family 2, subfamily AE, member 1*	*OR2AE1*	6.28	6.35	5.96	0.001492334	0.007976706
8159373	138311	*family with sequence similarity 69, member B*	*FAM69B*	8.28	8.21	9.18	0.00149316	0.007979644
7992347	23162	*mitogen-activated protein kinase 8 interacting protein 3*	*MAPK8IP3*	8.39	8.22	7.73	0.001494199	0.007983009
8130765	83640	*family with sequence similarity 103, member A1*	*FAM103A1*	8.41	8.56	9.93	0.001494383	0.007983009
8040827	150921	*transcription factor 23*	*TCF23*	7.34	7.35	6.91	0.001494619	0.007983009
8077490	29995	*LIM and cysteine-rich domains 1*	*LMCD1*	8.88	10.88	8.87	0.001495544	0.007986471
8122317	23593	*heme binding protein 2*	*HEBP2*	12.26	12.50	11.59	0.001496684	0.007991079
7950701	26011	*odz, odd Oz/ten-m homolog 4 (Drosophila)*	*ODZ4*	6.79	7.44	6.75	0.00149698	0.00799118
8109222	6129	*ribosomal protein L7*	*RPL7*	12.26	12.14	12.67	0.001498845	0.007995863
8008914	124773	*chromosome 17 open reading frame 64*	*C17orf64*	6.43	6.34	5.89	0.001498965	0.007995863
8040278	130814	*PQ loop repeat containing 3*	*PQLC3*	10.42	10.29	9.34	0.001498709	0.007995863
8133366	135886	*Williams-Beuren syndrome chromosome region 28*	*WBSCR28*	6.98	7.00	6.44	0.00149819	0.007995863
7990949	6133	*ribosomal protein L9*	*RPL9*	12.16	12.28	12.61	0.001500153	0.007997766
8099259	60312	*actin filament associated protein 1*	*AFAP1*	9.88	9.67	7.98	0.001499784	0.007997766
8014702	4302, 100129395	*myeloid/lymphoid or mixed-lineage leukemia (trithorax homolog, Drosophila); translocated to, 6 | NS5ATP13TP1*	*MLLT6|LOC100129395*	6.91	6.75	5.92	0.00150011	0.007997766
8005328	51168	*myosin XVA*	*MYO15A*	6.93	6.91	6.36	0.001501473	0.008003326
8083876	6498	*SKI-like oncogene*	*SKIL*	7.20	7.79	10.28	0.001501897	0.008004108
8125731	9278	*zinc finger and BTB domain containing 22*	*ZBTB22*	8.74	8.79	7.68	0.001502286	0.008004705
8042439	84168	*anthrax toxin receptor 1*	*ANTXR1*	10.81	11.20	9.27	0.00150421	0.008011036

7963459	140807	*keratin 72*	*KRT72*	6.52	6.54	5.97	0.001504307	0.008011036
8072153	150275	*coiled-coil domain containing 117*	*CCDC117*	8.18	8.21	10.16	0.001503843	0.008011036
8063590	5105	*phosphoenolpyruvate carboxykinase 1 (soluble)*	*PCK1*	6.27	6.30	5.88	0.001504993	0.008012059
8166335	5160	*pyruvate dehydrogenase (lipoamide) alpha 1*	*PDHA1*	8.16	8.54	9.84	0.001505331	0.008012059
7945663	402778	*CD225 family protein FLJ76511*	*LOC402778*	8.23	9.17	6.43	0.001505159	0.008012059
8007643	284071	*chromosome 17 open reading frame 104*	*C17orf104*	4.79	4.83	5.52	0.001506068	0.008014504
8025601	3383	*intercellular adhesion molecule 1*	*ICAM1*	9.18	7.95	7.33	0.001506824	0.008015571
8061772	22919	*microtubule-associated protein, RP/EB family, member 1*	*MAPRE1*	8.93	9.72	11.46	0.001506759	0.008015571
8059953				4.77	4.77	4.62	0.001507681	0.008018649
8093386	4636	*myosin, light chain 5, regulatory*	*MYL5*	7.75	7.85	6.92	0.001508256	0.008018757
8140852	219557	*chromosome 7 open reading frame 62*	*C7orf62*	5.48	5.47	5.24	0.001508113	0.008018757
8009631	350383	*G protein-coupled receptor 142*	*GPR142*	7.33	7.31	6.75	0.001509733	0.008025129
8068478	8208	*chromatin assembly factor 1, subunit B (p60)*	*CHAF1B*	7.83	8.12	9.62	0.001511199	0.008030485
8007826	162540	*intramembrane protease 5*	*IMP5*	8.14	8.19	7.54	0.001511297	0.008030485
8003679	6117	*replication protein A1, 70kDa*	*RPA1*	7.99	8.34	9.43	0.001512319	0.008034254
7906786	84824	*Fc receptor-like A*	*FCRLA*	6.17	6.08	5.58	0.001512563	0.008034254
7952046	10205	*myelin protein zero-like 2*	*MPZL2*	5.46	5.81	6.55	0.001516226	0.008052234
8012787	4621	*myosin, heavy chain 3, skeletal muscle, embryonic*	*MYH3*	6.28	6.30	5.89	0.001519148	0.008064167
8045030	4648	*myosin VIIB*	*MYO7B*	6.77	6.87	6.39	0.001519056	0.008064167
8066493	6590	*secretory leukocyte peptidase inhibitor*	*SLPI*	6.90	6.47	5.91	0.001520488	0.008064167
8064790	9770	*Ras association (RalGDS/AF-6) domain family member 2*	*RASSF2*	11.12	9.00	8.47	0.001520708	0.008064167
8042588	10199	*M-phase phosphoprotein 10 (U3 small nucleolar ribonucleoprotein)*	*MPHOSPH10*	6.47	6.57	7.83	0.001519779	0.008064167
8112306	57399	*uncharacterized gastric protein ZA52P*	*LOC57399*	4.74	4.73	4.62	0.001520144	0.008064167
8015511	79132	*DEXH (Asp-Glu-X-His) box polypeptide 58*	*DHX58*	8.06	7.56	6.79	0.001520293	0.008064167
8047854	151195	*cyclin Y-like 1*	*CCNYL1*	7.47	7.83	9.54	0.001520539	0.008064167
8094688	11019	*lipoic acid synthetase*	*LIAS*	6.27	6.43	8.73	0.00152166	0.008067735

8088065	51460	*Scm-like with four mbt domains 1*	*SFMBT1*	6.65	6.69	7.99	0.001522081	0.008068481
8148671	286076	*breast cancer estrogen-induced apoptosis 2*	*BREA2*	6.40	6.33	5.94	0.001523503	0.00807454
7945349	51272	*blocked early in transport 1 homolog (S. cerevisiae)-like*	*BET1L*	10.54	10.40	8.91	0.00152443	0.008077969
7988380	90527, 405753	*dual oxidase maturation factor 1 | dual oxidase maturation factor 2*	*DUOXA1| DUOXA2*	7.95	7.92	7.34	0.001525195	0.008080539
8117020	29116	*myosin regulatory light chain interacting protein*	*MYLIP*	8.55	7.86	9.18	0.001525815	0.008082341
7906995	7371	*uridine-cytidine kinase 2*	*UCK2*	9.78	10.34	10.81	0.001531392	0.008110392
8034420	4125	*mannosidase, alpha, class 2B, member 1*	*MAN2B1*	11.03	10.63	9.57	0.001532379	0.008111157
7940959	56834	*G protein-coupled receptor 137*	*GPR137*	9.24	9.19	7.77	0.001532297	0.008111157
8100893	285521		*COX18*	8.24	8.18	9.31	0.001531842	0.008111157
7946781	144100	*pleckstrin homology domain containing, family A member 7*	*PLEKHA7*	6.63	6.59	7.84	0.001532665	0.008111184
8029193	23152	*capicua homolog (Drosophila)*	*CIC*	9.61	9.55	8.11	0.001536306	0.008128961
8141358				6.43	6.64	5.74	0.001536787	0.008130016
8041508	25797	*glutaminyl-peptide cyclotransferase*	*QPCT*	6.98	8.72	8.20	0.001538739	0.008138852
8008277	6442	*sarcoglycan, alpha (50kDa dystrophin-associated glycoprotein)*	*SGCA*	7.54	7.75	6.73	0.001539345	0.008139297
7917942	400765		*FLJ35409*	6.92	8.00	6.25	0.001539387	0.008139297
8005483	10517, 374286	*F-box and WD repeat domain containing 10 | CMT1A duplicated region transcript 1*	*FBXW10|C DRT1*	6.12	6.00	5.39	0.001540106	0.008141605
8099581	254251, 64151	*ligand dependent nuclear receptor corepressor-like | non-SMC condensin I complex, subunit G*	*LCORL|N CAPG*	7.03	6.82	8.57	0.001540486	0.008142124
7913665				8.27	8.22	7.38	0.001542189	0.008149632
8078619	3680	*integrin, alpha 9*	*ITGA9*	6.72	6.81	7.92	0.001545056	0.00816329
8062603	7150	*topoisomerase (DNA) I*	*TOP1*	9.40	9.20	11.20	0.001546321	0.008168478
7962623	10411	*Rap guanine nucleotide exchange factor (GEF) 3*	*RAPGEF3*	7.35	7.26	6.87	0.001547261	0.008171951
8043218	10713	*ubiquitin specific peptidase 39*	*USP39*	9.32	9.34	10.10	0.001548552	0.008177275
7983290	10169	*small EDRK-rich factor 2*	*SERF2*	8.89	8.86	7.93	0.001548882	0.008177521
8039524	163033	*zinc finger protein 579*	*ZNF579*	8.39	8.52	7.60	0.001550495	0.008184539
7915147	2275	*four and a half LIM domains 3*	*FHL3*	10.77	11.14	9.35	0.001553329	0.0081965

8083221	5089	*pre-B-cell leukemia homeobox 2*	*PBX2*	10.21	9.95	10.79	0.001553208	0.0081965
8024056	1991	*elastase, neutrophil expressed*	*ELANE*	8.69	8.58	7.80	0.001553859	0.008197797
7942342	3636	*inositol polyphosphate phosphatase-like 1*	*INPPL1*	9.87	9.94	8.42	0.001554815	0.008201342
8132642	5478	*peptidylprolyl isomerase A (cyclophilin A)*	*PPIA*	8.36	8.30	8.90	0.001555327	0.008202545
8165319	54461	*F-box and WD repeat domain containing 5*	*FBXW5*	10.09	10.18	8.68	0.001556546	0.008206505
8110920	79072	*FAST kinase domains 3*	*FASTKD3*	6.56	6.58	7.49	0.001556646	0.008206505
8045816	2820	*glycerol-3-phosphate dehydrogenase 2 (mitochondrial)*	*GPD2*	7.32	7.10	9.15	0.00155833	0.008210884
8031196	3904	*leukocyte-associated immunoglobulin-like receptor 2*	*LAIR2*	7.17	7.02	6.47	0.001557983	0.008210884
7923005				5.32	5.47	5.15	0.001558051	0.008210884
7969703	3843	*importin 5*	*IPO5*	10.14	10.71	11.64	0.001559349	0.008214752
7977371	23241	*phosphofurin acidic cluster sorting protein 2*	*PACS2*	7.97	7.90	7.15	0.001559932	0.008214931
7985522	57188	*ADAMTS-like 3*	*ADAMTSL 3*	6.03	6.08	6.56	0.001559952	0.008214931
7984436	390598	*SKI family transcriptional corepressor 1*	*SKOR1*	8.08	8.12	7.52	0.001560479	0.00821621
7941004	283234	*coiled-coil domain containing 88B*	*CCDC88B*	7.27	7.38	6.80	0.001561134	0.008216661
8171350	170082, 645769	*transcription elongation factor A (SII) N-terminal and central domain containing | hypothetical LOC645769*	*TCEANC|LOC645769*	6.94	6.92	6.11	0.001561129	0.008216661
8044440	129804	*fibulin 7*	*FBLN7*	9.19	9.32	7.05	0.001561652	0.008217888
7974835	5583	*protein kinase C, eta*	*PRKCH*	6.61	6.43	8.04	0.00156374	0.008227059
7916836	26135	*SERPINE1 mRNA binding protein 1*	*SERBP1*	7.05	6.87	10.20	0.001563965	0.008227059
8045889	85461	*tetratricopeptide repeat, ankyrin repeat and coiled-coil containing 1*	*TANC1*	9.26	8.28	7.62	0.001564529	0.008228527
8134890	2783	*guanine nucleotide binding protein (G protein), beta polypeptide 2*	*GNB2*	10.71	10.75	9.49	0.001565572	0.008231013
8061685	9777	*transmembrane 9 superfamily protein member 4*	*TM9SF4*	11.39	11.32	10.43	0.001565362	0.008231013
7945944	391	*ras homolog gene family, member G (rho G)*	*RHOG*	11.30	11.49	9.87	0.001566365	0.008233119
8004400	201243	*chromosome 17 open reading frame 74*	*C17orf74*	6.92	6.90	6.48	0.001566583	0.008233119
7945660	387742	*family with sequence similarity 99, member A*	*FAM99A*	6.70	6.93	6.12	0.001566828	0.008233119
8134777	79037, 10734	*poliovirus receptor related immunoglobulin domain containing | stromal antigen 3*	*PVRIG|STAG3*	7.32	7.43	6.98	0.00156752	0.008235254

8049682	406941	*microRNA 149*	*MIR149*	7.93	7.96	7.21	0.00156785	0.00823549
7995322				9.06	9.28	8.30	0.001568795	0.008238956
8047161	64859	*oligonucleotide/oligosaccharide-binding fold containing 2A*	*OBFC2A*	10.14	9.84	8.25	0.001569082	0.008238963
8035793	100129842	*zinc finger protein 737*	*ZNF737*	8.49	8.00	10.66	0.001570315	0.008243269
8133902				6.55	6.54	8.59	0.001570473	0.008243269
7981601	3494, 28396, 3495, 3507, 3493, 3500, 3502, 3501, 3503, 28442, 100126583, 652102, 100290415, 100133862, 100293211, 100290006	*immunoglobulin heavy constant alpha 2 (A2m marker) | immunoglobulin heavy variable 4-31 | immunoglobulin heavy constant delta | immunoglobulin heavy constant mu | immunoglobulin heavy constant alpha 1 | immunoglobulin heavy constant gamma 1 (G1m marker) | immunoglobulin heavy constant gamma 3 (G3m marker) | immunoglobulin heavy constant gamma 2 (G2m marker) | immunoglobulin heavy constant gamma 4 (G4m marker) | immunoglobulin heavy variable 3-23 | hypothetical LOC100126583 | similar to Ig heavy chain V-I region HG3 precursor | IgG VH | ig heavy chain V-I region V35-like | ig heavy chain V-III region VH26-like | similar to hCG2038941*	*IGHA2|IGHV4-31|IGHD|IGHM|IGHA1|IGHG1|IGHG3|IGHG2|IGHG4|IGHV3-23|LOC100126583|LOC652102|LOC100290415|LOC100133862|LOC100293211|LOC100290006*	6.65	6.70	6.26	0.001570882	0.008243915
8114843				7.33	7.41	6.64	0.001572029	0.00824844
7998129	51728, 79622	*polymerase (RNA) III (DNA directed) polypeptide K, 12.3 kDa | small nuclear ribonucleoprotein 25kDa (U11/U12)*	*POLR3K|SNRNP25*	9.46	9.32	10.48	0.001572862	0.008251309
8110569	8878	*sequestosome 1*	*SQSTM1*	11.82	11.52	10.74	0.001573904	0.008252618
8076644	25830	*sulfotransferase family 4A, member 1*	*SULT4A1*	6.16	6.27	7.20	0.001573969	0.008252618
8073013				8.98	9.12	8.29	0.001573511	0.008252618
7993108	440337		*LOC440337*	5.68	5.86	5.58	0.00157467	0.00825454

8013450	284194, 654346, 3965	*lectin, galactoside-binding, soluble, 9B | lectin, galactoside-binding, soluble, 9C | lectin, galactoside-binding, soluble, 9*	*LGALS9B| LGALS9C*	7.49	7.76	6.56	0.001574908	0.00825454
7962185	196394, 254013	*antagonist of mitotic exit network 1 homolog (S. cerevisiae) | chromosome 12 open reading frame 72*	*AMN1|C12 orf72*	6.83	6.91	7.93	0.001575787	0.008257652
7901993	57685	*cache domain containing 1*	*CACHD1*	8.42	7.87	10.48	0.00157665	0.008259173
8160346	401494, 54914	*protein tyrosine phosphatase-like A domain containing 2 | KIAA1797*	*PTPLAD2| KIAA1797*	9.29	7.89	7.16	0.001576449	0.008259173
8086222	6331	*sodium channel, voltage-gated, type V, alpha subunit*	*SCN5A*	6.56	6.41	7.10	0.001577158	0.008260336
7984475	10391	*coronin, actin binding protein, 2B*	*CORO2B*	8.62	8.71	6.93	0.001577928	0.008262873
8129666	154091	*solute carrier family 2 (facilitated glucose transporter), member 12*	*SLC2A12*	9.24	5.88	8.32	0.001579892	0.008271656
8017867	54757	*family with sequence similarity 20, member A*	*FAM20A*	7.14	8.89	7.36	0.0015807	0.008274385
8155661	286380, 653404, 2298, 200350, 100036519, 349334, 653427	*forkhead box D4-like 3 | forkhead box D4-like 6 | forkhead box D4 | forkhead box D4-like 1 | forkhead box D4-like 2 | forkhead box D4-like 4 | forkhead box D4-like 5*	*FOXD4L3| FOXD4L6| FOXD4L1| FOXD4L2| FOXD4L4| FOXD4L5*	7.78	7.78	7.33	0.001581445	0.008276782
8000948	9274	*B-cell CLL/lymphoma 7C*	*BCL7C*	9.42	9.65	8.37	0.00158189	0.008277614
8148715	8733	*glycosylphosphatidylinositol anchor attachment protein 1 homolog (yeast)*	*GPAA1*	11.68	11.85	10.23	0.001583532	0.0082832
7921155	3645, 4914	*insulin receptor-related receptor | neurotrophic tyrosine kinase, receptor, type 1*	*INSRR|NT RK1*	6.50	6.44	5.97	0.001583394	0.0082832
7992191	23430	*tryptase delta 1*	*TPSD1*	8.40	8.44	7.54	0.001584077	0.008284551
8037231	5671	*pregnancy specific beta-1-glycoprotein 3*	*PSG3*	8.05	7.66	5.72	0.001585181	0.008288821
8037422	284348	*LY6/PLAUR domain containing 5*	*LYPD5*	7.59	7.45	6.92	0.001585704	0.008290053
7911170	149134		*LOC14913 4*	6.37	6.15	6.04	0.001586454	0.008290975
8109618				5.52	5.79	5.08	0.001586188	0.008290975
8178512	4050	*lymphotoxin beta (TNF superfamily, member 3)*	*LTB*	8.52	8.60	7.86	0.001587128	0.008291672
8092905	55341	*large subunit GTPase 1 homolog (S. cerevisiae)*	*LSG1*	8.24	8.71	10.58	0.001587162	0.008291672

8008802	284161	*glycerophosphodiester phosphodiesterase domain containing 1*	*GDPD1*	6.10	6.19	6.63	0.001588504	0.008296799
8074606	11274, 373856	*ubiquitin specific peptidase 18 | ubiquitin specific peptidase 41*	*USP18|USP41*	9.65	8.22	8.15	0.001588933	0.008296799
7990892				5.29	5.22	5.11	0.001589006	0.008296799
7904869	81926, 100289252	*family with sequence similarity 108, member A1 | family with sequence similarity 108, member A11, pseudogene*	*FAM108A1|FAM108A11P*	10.69	10.64	9.37	0.001591647	0.008309085
7936083	84108	*polycomb group ring finger 6*	*PCGF6*	8.87	9.04	10.09	0.001592249	0.008310728
8035506	9244	*cytokine receptor-like factor 1*	*CRLF1*	11.48	10.53	9.13	0.001593706	0.008313822
8159318	26086	*G-protein signaling modulator 1*	*GPSM1*	8.84	8.79	7.37	0.001593665	0.008313822
8026763	376497	*solute carrier family 27 (fatty acid transporter), member 1*	*SLC27A1*	8.19	8.92	7.27	0.001593171	0.008313822
8025621	7087	*intercellular adhesion molecule 5, telencephalin*	*ICAM5*	7.58	7.63	7.04	0.001594422	0.008316054
7967230	56616	*diablo homolog (Drosophila)*	*DIABLO*	8.72	8.75	9.61	0.001595743	0.008321441
8157949	9649	*Ral GEF with PH domain and SH3 binding motif 1*	*RALGPS1*	7.01	6.87	7.91	0.001596514	0.008323958
8038587	84258	*synaptotagmin III*	*SYT3*	6.83	6.79	7.81	0.001598521	0.008331413
8139270	10156, 401331, 100271927	*RAS p21 protein activator 4 | RAS p21 protein activator 4 pseudogene | RAS p21 protein activator 4B*	*RASA4|RASA4P|RASA4B*	9.04	9.76	7.52	0.001598477	0.008331413
7904948	81926, 100289252	*family with sequence similarity 108, member A1 | family with sequence similarity 108, member A11, pseudogene*	*FAM108A1|FAM108A11P*	10.68	10.64	9.37	0.001599407	0.008334529
7983677				6.37	6.28	5.82	0.001600679	0.008339648
7958410	11153	*FIC domain containing*	*FICD*	8.90	9.38	8.75	0.001601995	0.008345001
8024909	23030	*lysine (K)-specific demethylase 4B*	*KDM4B*	8.98	9.22	8.16	0.001602669	0.008347005
7910372	149603	*ring finger protein 187*	*RNF187*	11.22	11.38	9.78	0.001603235	0.008348445
8138799	9865	*TLR4 interactor with leucine-rich repeats*	*TRIL*	7.52	7.30	6.86	0.001604074	0.008351311
7915286	5538	*palmitoyl-protein thioesterase 1*	*PPT1*	10.08	9.89	11.87	0.001604466	0.008351843
8178211	5698	*proteasome (prosome, macropain) subunit, beta type, 9 (large multifunctional peptidase 2)*	*PSMB9*	8.86	8.06	7.05	0.001604968	0.00835295
8042310	6509	*solute carrier family 1 (glutamate/neutral amino acid transporter), member 4*	*SLC1A4*	9.42	10.51	6.70	0.001605531	0.008354376

7949931	51111	*suppressor of variegation 4-20 homolog 1 (Drosophila)*	*SUV420H1*	7.47	7.46	9.37	0.001606294	0.008356839
8031499	284297	*scavenger receptor cysteine-rich glycoprotein*	*SSC5D*	9.21	9.38	7.86	0.001606755	0.008357731
8059770	200765	*tigger transposable element derived 1*	*TIGD1*	8.36	8.04	9.36	0.001607309	0.008359107
7911643	339457	*chromosome 1 open reading frame 222*	*C1orf222*	6.39	6.42	5.93	0.001608251	0.008362502
7981740	3493	*immunoglobulin heavy constant alpha 1*	*IGHA1*	6.71	6.86	6.30	0.001611406	0.008377397
8114050	23176	*septin 8*	*8-Sep*	10.04	9.92	9.18	0.001612019	0.008379072
8021150	51124	*immediate early response 3 interacting protein 1*	*IER3IP1*	5.41	5.91	6.62	0.001613118	0.008383276
8121685	285761	*discoidin, CUB and LCCL domain containing 1*	*DCBLD1*	7.54	8.81	7.50	0.001614191	0.008387343
8139281	27434	*polymerase (DNA directed), mu*	*POLM*	8.35	8.40	7.37	0.001614573	0.008387818
8100507	84525	*HOP homeobox*	*HOPX*	6.20	6.25	5.88	0.001615048	0.008388778
7926979				8.48	5.90	6.65	0.001615955	0.008391977
8136832	135927	*chromosome 7 open reading frame 34*	*C7orf34*	6.39	6.46	5.94	0.00161651	0.008393349
8009243	284021	*chromosome 17 open reading frame 60*	*C17orf60*	6.32	7.01	5.47	0.001618689	0.008403151
7942796	283219, 100289388	*potassium channel tetramerisation domain containing 21 | hypothetical LOC100289388*	*KCTD21|LOC100289388*	6.88	6.98	6.30	0.001620125	0.008409094
8043278	51318	*mitochondrial ribosomal protein L35*	*MRPL35*	7.85	8.12	9.80	0.001620589	0.008409991
8118571	5698	*proteasome (prosome, macropain) subunit, beta type, 9 (large multifunctional peptidase 2)*	*PSMB9*	8.87	8.06	7.05	0.001623378	0.008422949
7953166	10867	*tetraspanin 9*	*TSPAN9*	8.45	7.85	7.43	0.001624074	0.00842505
8044613	55871, 150472, 445571, 220869, 644019, 728013, 653510, 100507355	*COBW domain containing 1 | COBW domain containing 2 | COBW domain containing 3 | COBW domain containing 5 | COBW domain containing 6 | COBW domain containing 7 | COBW domain-containing protein 5-like | hypothetical LOC100507355*	*CBWD1|CBWD2|CBWD3|CBWD5|CBWD6|CBWD7|LOC653510|LOC100507355*	7.88	8.02	9.78	0.001624399	0.008425217
8110022	8992	*ATPase, H+ transporting, lysosomal 9kDa, V0 subunit e1*	*ATP6V0E1*	12.81	12.84	12.39	0.001625151	0.008426348
7952321	390259	*brain-specific homeobox*	*BSX*	7.66	7.72	7.06	0.0016252	0.008426348

8037563	10848	*protein phosphatase 1, regulatory (inhibitor) subunit 13 like*	*PPP1R13L*	9.16	9.81	8.14	0.001625708	0.008427468
7986943				8.24	8.35	7.30	0.001627226	0.008433091
8023059				8.07	8.20	7.21	0.001627377	0.008433091
8179768	4050	*lymphotoxin beta (TNF superfamily, member 3)*	*LTB*	8.52	8.60	7.86	0.001627912	0.008434347
7970439	2700	*gap junction protein, alpha 3, 46kDa*	*GJA3*	7.33	7.33	6.84	0.001628806	0.008435402
8030789	27036	*sialic acid binding Ig-like lectin 7*	*SIGLEC7*	6.95	6.94	6.48	0.001629858	0.008435402
8137257	55340	*GTPase, IMAP family member 5*	*GIMAP5*	5.89	5.85	5.51	0.001629869	0.008435402
8157463	203197	*chromosome 9 open reading frame 91*	*C9orf91*	8.53	8.91	7.47	0.001629522	0.008435402
7999614	5310, 339044, 9284, 399491, 100288332, 642778	*polycystic kidney disease 1 (autosomal dominant) | polycystic kidney disease 1 (autosomal dominant) pseudogene 1 | nuclear pore complex interacting protein | GPS, PLAT and transmembrane domain-containing protein | GPS, PLAT and transmembrane domain-containing protein FLJ00285-like | nuclear pore complex-interacting protein-like 1-like*	*PKD1|PKD1P1|NPIP|LOC399491|LOC100288332|LOC642778*	11.53	11.57	10.27	0.001628447	0.008435402
7948085	7299, 7300	*tyrosinase (oculocutaneous albinism IA) | tyrosinase-like (pseudogene)*	*TYR|TYRL*	4.97	4.95	4.72	0.001629476	0.008435402
8124059	9972	*nucleoporin 153kDa*	*NUP153*	9.09	9.00	11.17	0.001632372	0.008444496
8041553	79833	*gem (nuclear organelle) associated protein 6*	*GEMIN6*	6.42	6.43	7.16	0.001632796	0.008444496
7952626	219833	*chromosome 11 open reading frame 45*	*C11orf45*	6.64	6.20	5.75	0.001632534	0.008444496
8123802	63915, 81567, 9521	*muted homolog (mouse) | thioredoxin domain containing 5 (endoplasmic reticulum) | eukaryotic translation elongation factor 1 epsilon 1*	*MUTED|TXNDC5|EEF1E1*	9.43	9.62	10.41	0.001632542	0.008444496
8161537	445571, 644019, 55871, 150472, 220869, 728013, 653510, 100507355	*COBW domain containing 3 | COBW domain containing 6 | COBW domain containing 1 | COBW domain containing 2 | COBW domain containing 5 | COBW domain containing 7 | COBW domain-containing protein 5-like | hypothetical LOC100507355*	*CBWD3|CBWD6|CBWD1|CBWD2|CBWD5|CBWD7|LOC653510|LOC100507355*	7.34	7.53	9.31	0.001634393	0.008451243

8042059	344405	*prolyl-tRNA synthetase associated domain containing 1, pseudogene*	*PRORSD1P*	6.81	6.84	7.26	0.001635371	0.008454788
8176709	84663	*chromosome Y open reading frame 15B*	*CYorf15B*	6.11	5.61	8.14	0.001635976	0.008456399
8019574	85294, 81872, 728279, 730755, 85297	*keratin associated protein 2-4 | keratin associated protein 2-1 | keratin associated protein 2-2 | keratin associated protein 2-4-like | keratin associated protein 2.1B*	*KRTAP2-4|KRTAP2-1|KRTAP2-2|LOC730755|KAP2.1B*	8.67	9.50	7.83	0.001636576	0.008457989
7935707	1147	*conserved helix-loop-helix ubiquitous kinase*	*CHUK*	7.77	7.75	8.96	0.001637946	0.008463549
8082673	55	*acid phosphatase, prostate*	*ACPP*	5.76	5.74	6.26	0.001638755	0.008466218
7987097	406993	*microRNA 211*	*MIR211*	7.62	7.53	7.18	0.001639642	0.008468649
8170418				6.49	6.52	8.37	0.001639813	0.008468649
8000910	146542	*zinc finger protein 688*	*ZNF688*	8.28	8.33	7.15	0.001640628	0.008470331
8111118				5.21	5.13	4.91	0.001640725	0.008470331
8001102				6.71	6.90	6.31	0.001641444	0.008472529
8008228	1748	*distal-less homeobox 4*	*DLX4*	7.89	7.96	7.29	0.001642746	0.00847773
8015214	85294, 81872, 728279, 730755, 85297	*keratin associated protein 2-4 | keratin associated protein 2-1 | keratin associated protein 2-2 | keratin associated protein 2-4-like | keratin associated protein 2.1B*	*KRTAP2-4|KRTAP2-1|KRTAP2-2|LOC730755|KAP2.1B*	8.67	9.51	7.83	0.001643382	0.008479499
8144643	2626	*GATA binding protein 4*	*GATA4*	7.35	7.47	6.88	0.001643814	0.008479749
8020349				4.77	4.72	5.39	0.001644018	0.008479749
8117685	80317	*zinc finger with KRAB and SCAN domains 3*	*ZKSCAN3*	6.32	6.21	7.32	0.001645441	0.008485572
8027006	170463	*single stranded DNA binding protein 4*	*SSBP4*	9.71	9.69	8.52	0.001646146	0.008487692
8113130	79772	*multiple C2 domains, transmembrane 1*	*MCTP1*	5.33	5.35	6.68	0.001646946	0.008490301
8124950	4050	*lymphotoxin beta (TNF superfamily, member 3)*	*LTB*	8.52	8.60	7.86	0.001648169	0.008492057

8104760	6897	*threonyl-tRNA synthetase*	*TARS*	9.22	9.30	11.23	0.001647638	0.008492057
8033744	26651	*olfactory receptor, family 7, subfamily E, member 19 pseudogene*	*OR7E19P*	6.08	6.18	5.73	0.001648113	0.008492057
7971222	9617	*mitochondrial translational release factor 1*	*MTRF1*	6.88	6.58	7.61	0.001648685	0.00849247
8176574	652811	*matrix-remodeling-associated protein 5-like*	*LOC65281 1*	7.51	8.59	6.35	0.001648838	0.00849247
8012270				5.86	6.00	7.40	0.001649141	0.008492518
8046279				8.29	8.46	9.09	0.001649699	0.008493874
8134880	64598	*motile sperm domain containing 3*	*MOSPD3*	10.82	10.88	9.47	0.001651192	0.008500048
8019263	396	*Rho GDP dissociation inhibitor (GDI) alpha*	*ARHGDIA*	11.20	11.39	9.71	0.001651787	0.008501596
8143708				8.07	8.09	7.70	0.001652991	0.008506271
7949570	89792	*galactose-3-O-sulfotransferase 3*	*GAL3ST3*	7.21	7.31	8.40	0.00165755	0.008528214
8139966	375593, 378108	*tripartite motif-containing 73 | tripartite motif-containing 74*	*TRIM73|T RIM74*	7.41	7.39	6.74	0.001658826	0.008533261
7904287	914		*CD2*	5.84	5.79	5.60	0.00166206	0.008546876
8157677				5.33	5.47	4.95	0.001662065	0.008546876
8064175	56731, 140685	*SLC2A4 regulator | zinc finger and BTB domain containing 46*	*SLC2A4R G|ZBTB46*	8.94	8.93	7.47	0.001662452	0.008547343
7977046	7127	*tumor necrosis factor, alpha-induced protein 2*	*TNFAIP2*	9.11	8.98	7.36	0.001663333	0.008550351
7932964	10438		*C1D*	8.46	8.91	9.46	0.001663664	0.008550528
8041987	129852	*chromosome 2 open reading frame 73*	*C2orf73*	5.20	5.07	4.91	0.00166467	0.008554177
8057933	9330	*general transcription factor IIIC, polypeptide 3, 102kDa*	*GTF3C3*	7.60	7.45	9.62	0.001666423	0.008561657
7927288	439965, 414241, 54537	*family with sequence similarity 35, member A pseudogene*	*FAM35B2| FAM35A*	8.66	8.73	9.99	0.001666782	0.008561981
8171885	139425	*DDB1 and CUL4 associated factor 8-like 1*	*DCAF8L1*	5.59	5.64	5.40	0.001667978	0.008566601
7971541	8803	*succinate-CoA ligase, ADP-forming, beta subunit*	*SUCLA2*	9.09	8.93	10.48	0.001669441	0.00857259
8044107				4.64	4.60	4.50	0.001670107	0.008574485
8021528	8792	*tumor necrosis factor receptor superfamily, member 11a, NFKB activator*	*TNFRSF11 A*	6.79	6.82	7.66	0.001670593	0.008574548
8022559	147463	*ankyrin repeat domain 29*	*ANKRD29*	8.68	7.13	6.61	0.001670714	0.008574548

7945475	53841	*cadherin-related family member 5*	*CDHR5*	7.18	7.43	6.88	0.001671604	0.008576066
8087250	494337	*microRNA 425*	*MIR425*	6.47	6.65	6.15	0.001671564	0.008576066
7904865				5.78	5.65	5.03	0.001672144	0.008577316
7981720	28424	*immunoglobulin heavy variable 3-48*	*IGHV3-48*	7.08	7.15	6.66	0.001673833	0.008582784
8153652	81858	*SHANK-associated RH domain interactor*	*SHARPIN*	10.62	10.54	9.20	0.001674102	0.008582784
8058496				6.41	6.51	5.97	0.00167402	0.008582784
7932510				7.08	6.96	6.54	0.001674777	0.008584719
8140971	219285	*sterile alpha motif domain containing 9-like*	*SAMD9L*	9.14	8.15	5.28	0.001677572	0.008597519
7938880	10553	*HIV-1 Tat interactive protein 2, 30kDa*	*HTATIP2*	7.97	8.15	9.58	0.001678003	0.008598203
8103722	441051	*heat shock protein 90kDa alpha (cytosolic), class A member 6 (pseudogene)*	*HSP90AA6P*	7.49	7.40	8.81	0.001679463	0.008604156
8179495	5698	*proteasome (prosome, macropain) subunit, beta type, 9 (large multifunctional peptidase 2)*	*PSMB9*	8.87	8.06	7.06	0.001679801	0.008604362
8043474				9.33	9.43	8.36	0.001680475	0.008606288
8083569	25976	*TCDD-inducible poly(ADP-ribose) polymerase*	*TIPARP*	10.36	9.72	8.58	0.001681886	0.008611434
8157828	81873	*actin related protein 2/3 complex, subunit 5-like*	*ARPC5L*	7.19	7.44	7.89	0.001682077	0.008611434
8116664	8899	*PRP4 pre-mRNA processing factor 4 homolog B (yeast)*	*PRPF4B*	7.58	7.18	9.34	0.001683652	0.008617968
7991104				8.57	8.62	7.99	0.001683974	0.00861809
7942465	51642	*mitochondrial ribosomal protein L48*	*MRPL48*	8.64	8.55	9.14	0.001685241	0.008623044
8109697	900	*cyclin G1*	*CCNG1*	12.12	11.81	13.02	0.001687037	0.008630696
8168045	1947	*ephrin-B1*	*EFNB1*	9.14	8.84	7.17	0.001687351	0.008630696
8123129	9589	*Wilms tumor 1 associated protein*	*WTAP*	9.41	9.44	10.67	0.001687633	0.008630696
7966189	121643	*forkhead box N4*	*FOXN4*	7.12	7.17	6.57	0.001688436	0.008633275
7905862	149095	*DC-STAMP domain containing 1*	*DCST1*	6.98	7.02	6.53	0.001689389	0.008636073
8070867				6.87	6.89	6.42	0.001689582	0.008636073
7904209				6.87	6.84	6.49	0.001690533	0.008639405
7926875	25805	*BMP and activin membrane-bound inhibitor homolog (Xenopus laevis)*	*BAMBI*	7.13	7.79	9.98	0.001691704	0.008643858
8028950	22952	*cytochrome P450, family 2, subfamily G, polypeptide 1 pseudogene*	*CYP2G1P*	4.97	5.14	4.73	0.001693431	0.008647068

7979548	122876	*glycoprotein hormone beta 5*	*GPHB5*	7.19	7.32	6.75	0.001692967	0.008647068
8030908	147657	*zinc finger protein 480*	*ZNF480*	8.39	8.18	10.73	0.001692983	0.008647068
7986661				8.24	8.36	7.31	0.00169353	0.008647068
8151032	8836	*gamma-glutamyl hydrolase (conjugase, folylpolygammaglutamyl hydrolase)*	*GGH*	6.88	6.55	7.91	0.001694046	0.008648173
7939946	219438	*olfactory receptor, family 5, subfamily D, member 18*	*OR5D18*	5.87	6.00	5.49	0.001695969	0.008656458
7938348	7465		*WEE1*	10.16	9.34	10.46	0.001696435	0.008657307
7948656	2495	*ferritin, heavy polypeptide 1*	*FTH1*	11.94	11.93	11.28	0.001697011	0.008658716
8147970	9166	*estrogen receptor binding site associated, antigen, 9*	*EBAG9*	7.77	8.02	8.56	0.001699428	0.008660474
7944825	26541	*olfactory receptor, family 10, subfamily D, member 1 pseudogene*	*OR10D1P*	6.94	6.97	6.82	0.001700116	0.008660474
7918235	254268	*AKNA domain containing 1*	*AKNAD1*	5.72	5.69	5.36	0.001700327	0.008660474
7925687	388759	*chromosome 1 open reading frame 229*	*C1orf229*	7.73	7.75	7.06	0.001699031	0.008660474
8122144	594839	*small nucleolar RNA, H/ACA box 33*	*SNORA33*	5.72	5.53	6.77	0.001698595	0.008660474
8034448	51398, 84292	*chromosome 19 open reading frame 56 | WD repeat domain 83*	*C19orf56| WDR83*	9.87	9.97	8.83	0.001698758	0.008660474
7905077				6.15	6.18	7.51	0.001700245	0.008660474
7916851				5.61	5.56	5.13	0.001700356	0.008660474
7982152				8.25	8.36	7.31	0.001698999	0.008660474
8074192				5.70	5.85	5.43	0.001698268	0.008660474
8178988	9278	*zinc finger and BTB domain containing 22*	*ZBTB22*	8.58	8.65	7.54	0.001700821	0.008661317
7940391	923		*CD6*	6.95	7.03	6.52	0.001701481	0.008663151
7900931	8704	*UDP-Gal:betaGlcNAc beta 1,4- galactosyltransferase, polypeptide 2*	*B4GALT2*	10.98	11.37	9.94	0.00170208	0.008663267
7995282	24150, 729355	*TP53 target 3 | TP53 target 3B*	*TP53TG3| TP53TG3B*	5.99	6.05	5.56	0.001702104	0.008663267
8004842	22899	*Rho guanine nucleotide exchange factor (GEF) 15*	*ARHGEF1 5*	7.98	7.95	7.35	0.001702738	0.008664414
8090960	51163	*debranching enzyme homolog 1 (S. cerevisiae)*	*DBR1*	6.23	6.20	7.25	0.00170293	0.008664414
7931108	1755	*deleted in malignant brain tumors 1*	*DMBT1*	6.30	6.36	5.84	0.001703592	0.00866481

8037775	5739	*prostaglandin I2 (prostacyclin) receptor (IP)*	*PTGIR*	8.17	8.83	6.67	0.001703608	0.00866481
8154872	51271	*ubiquitin associated protein 1*	*UBAP1*	9.89	9.99	9.47	0.001707866	0.008683404
8046086	253782	*LAG1 homolog, ceramide synthase 6*	*LASS6*	9.10	9.06	10.92	0.001707666	0.008683404
8114805	2246	*fibroblast growth factor 1 (acidic)*	*FGF1*	7.47	8.61	6.39	0.001708208	0.008683614
8003204	51659	*GINS complex subunit 2 (Psf2 homolog)*	*GINS2*	8.41	9.09	11.75	0.001709711	0.008689729
7945110	6484	*ST3 beta-galactoside alpha-2,3-sialyltransferase 4*	*ST3GAL4*	8.41	8.03	6.94	0.001710164	0.008690499
8011797	388324	*inhibitor of CDK, cyclin A1 interacting protein 1*	*INCA1*	8.14	8.20	7.24	0.001710762	0.008692008
8155636	445571, 55871, 150472, 644019, 220869, 728013, 653510, 100507355	*COBW domain containing 3 | COBW domain containing 1 | COBW domain containing 2 | COBW domain containing 6 | COBW domain containing 5 | COBW domain containing 7 | COBW domain-containing protein 5-like | hypothetical LOC100507355*	*CBWD3|CBWD1|CBWD2|CBWD6|CBWD5|CBWD7|LOC653510|LOC100507355*	7.30	7.49	9.26	0.001711369	0.008693564
7942409	5031	*pyrimidinergic receptor P2Y, G-protein coupled, 6*	*P2RY6*	7.30	7.07	6.62	0.001711694	0.008693681
8173493	2833	*chemokine (C-X-C motif) receptor 3*	*CXCR3*	7.42	7.37	6.71	0.001712998	0.008698774
8135319				7.65	7.67	7.08	0.001716634	0.008715707
7953569	5777	*protein tyrosine phosphatase, non-receptor type 6*	*PTPN6*	7.29	7.34	8.39	0.001717861	0.008716275
7919898	10500	*sema domain, transmembrane domain (TM), and cytoplasmic domain, (semaphorin) 6C*	*SEMA6C*	7.83	7.76	7.26	0.001717954	0.008716275
7941843	54961	*slingshot homolog 3 (Drosophila)*	*SSH3*	9.63	9.09	8.02	0.001717428	0.008716275
8168391	653687	*non-protein coding RNA 246B*	*NCRNA00246B*	6.06	6.03	6.65	0.001717946	0.008716275
8066198				7.10	7.14	6.45	0.001718372	0.008716865
8106250				6.45	6.65	5.94	0.001718797	0.008717489
8002713	497190, 283971, 348174	*C-type lectin domain family 18, member B | C-type lectin domain family 18, member C | C-type lectin domain family 18, member A*	*CLEC18B|CLEC18C|CLEC18A*	7.04	7.11	6.62	0.001719527	0.008719659

7994565	54700, 730092, 653390	*RRN3 RNA polymerase I transcription factor homolog (S. cerevisiae) | RNA polymerase I transcription factor homolog (S. cerevisiae) pseudogene 1 | RNA polymerase I transcription factor homolog (S. cerevisiae) pseudogene 2*	*RRN3|RRN3P1|RRN3P2*	9.24	9.09	10.88	0.001720524	0.008721991
7991169				8.57	8.62	7.99	0.001720591	0.008721991
8043491	150483	*tektin 4*	*TEKT4*	8.03	8.13	7.33	0.001724279	0.008739154
7992889	4993	*olfactory receptor, family 2, subfamily C, member 1*	*OR2C1*	6.68	6.56	6.23	0.00172578	0.008743688
8159239	157927	*chromosome 9 open reading frame 62*	*C9orf62*	6.93	7.03	6.62	0.001725533	0.008743688
8006433	6347	*chemokine (C-C motif) ligand 2*	*CCL2*	10.30	10.47	8.03	0.001726282	0.008744644
8145097	55124	*piwi-like 2 (Drosophila)*	*PIWIL2*	5.56	5.35	7.32	0.001726835	0.008744644
7977331	122616	*chromosome 14 open reading frame 79*	*C14orf79*	7.37	7.60	6.33	0.001726877	0.008744644
7953100	2288	*FK506 binding protein 4, 59kDa*	*FKBP4*	10.07	10.25	12.08	0.001728453	0.008748138
7975191	161142	*family with sequence similarity 71, member D*	*FAM71D*	5.54	5.49	5.24	0.001728243	0.008748138
8173766	254065	*bromodomain and WD repeat domain containing 3*	*BRWD3*	7.27	7.00	9.67	0.001728476	0.008748138
7921516	89886	*SLAM family member 9*	*SLAMF9*	6.05	6.12	5.61	0.001729545	0.008752013
7928019	3098	*hexokinase 1*	*HK1*	8.86	9.20	9.87	0.001730941	0.008754472
8157038	23446	*solute carrier family 44, member 1*	*SLC44A1*	8.24	9.16	10.55	0.001730841	0.008754472
7927267	414241, 439965, 54537	*family with sequence similarity 35, member A pseudogene*	*FAM35B|FAM35B2|FAM35A*	8.08	8.19	9.45	0.0017309	0.008754472
8010061	8877	*sphingosine kinase 1*	*SPHK1*	8.28	9.40	7.07	0.001732096	0.008758407
7953626	9746	*calsyntenin 3*	*CLSTN3*	8.47	7.96	7.80	0.001732326	0.008758407
7952897	440073	*IQ motif and Sec7 domain 3*	*IQSEC3*	7.39	7.39	6.78	0.001733701	0.008763827
8177658	8293, 728492	*small EDRK-rich factor 1A (telomeric) | small EDRK-rich factor 1B (centromeric)*	*SERF1A|SERF1B*	7.55	7.75	8.32	0.001734056	0.008764084
7959957	8408	*unc-51-like kinase 1 (C. elegans)*	*ULK1*	10.07	10.03	8.58	0.001734421	0.008764397
7997491	3294	*hydroxysteroid (17-beta) dehydrogenase 2*	*HSD17B2*	8.27	6.56	6.18	0.001735043	0.008765363
8090314	10840	*aldehyde dehydrogenase 1 family, member L1*	*ALDH1L1*	6.99	6.97	6.37	0.001735523	0.008765363
8053722	654342	*lymphocyte-specific protein 1 pseudogene*	*LOC654342*	9.97	10.30	9.15	0.001735477	0.008765363

8132406	5898	*v-ral simian leukemia viral oncogene homolog A (ras related)*	*RALA*	8.91	9.20	10.13	0.00173614	0.008766945
8144810				6.67	6.38	6.17	0.001736563	0.008767546
8048749	23704	*potassium voltage-gated channel, Isk-related family, member 4*	*KCNE4*	8.40	9.34	5.98	0.001736986	0.008768151
8126163	89822	*potassium channel, subfamily K, member 17*	*KCNK17*	6.26	6.31	6.65	0.001737759	0.008768983
8151209				6.09	6.13	5.66	0.001737682	0.008768983
7916135	200014	*coiled-coil and C2 domain containing 1B*	*CC2D1B*	9.15	9.23	8.02	0.001738852	0.008772969
8013641	94005	*phosphatidylinositol glycan anchor biosynthesis, class S*	*PIGS*	11.22	11.12	9.98	0.001740484	0.00877727
8035795	199777	*zinc finger protein 626*	*ZNF626*	8.30	8.35	10.05	0.001740617	0.00877727
8119124	221476	*peptidase inhibitor 16*	*PI16*	8.85	10.38	7.02	0.001740568	0.00877727
8103684	54969	*chromosome 4 open reading frame 27*	*C4orf27*	6.65	6.82	7.70	0.001742712	0.008786301
8054437	64682	*anaphase promoting complex subunit 1*	*ANAPC1*	6.83	6.84	9.81	0.001743511	0.008788795
7945991	81285	*olfactory receptor, family 51, subfamily E, member 2*	*OR51E2*	5.74	5.97	5.61	0.001744561	0.008792551
8069985	677846	*small nucleolar RNA, H/ACA box 80*	*SNORA80*	8.19	8.27	7.66	0.001745524	0.008794333
8058388				5.39	5.31	6.62	0.001745487	0.008794333
7980744	9252	*ribosomal protein S6 kinase, 90kDa, polypeptide 5*	*RPS6KA5*	5.65	5.63	6.70	0.001747559	0.008803052
8032751				7.65	7.69	7.19	0.001748181	0.008804645
8008491				5.25	5.29	4.98	0.001749644	0.008810476
8105949	8293, 728492	*small EDRK-rich factor 1A (telomeric) | small EDRK-rich factor 1B (centromeric)*	*SERF1A|SERF1B*	7.55	7.76	8.31	0.001750502	0.008812452
8123891				7.55	7.69	6.69	0.001750646	0.008812452
7930148	118980	*sideroflexin 2*	*SFXN2*	7.51	7.38	9.06	0.001752843	0.00881988
7951163				5.78	5.75	5.32	0.001752851	0.00881988
7961418				6.62	7.41	5.71	0.001753039	0.00881988
7992067				7.01	7.09	6.46	0.001753408	0.008820202
7978376	29091	*syntaxin binding protein 6 (amisyn)*	*STXBP6*	6.59	6.92	7.51	0.001754438	0.00882383
8008321	80221	*acyl-CoA synthetase family member 2*	*ACSF2*	8.89	8.53	7.57	0.001755658	0.00882383
8062782	84969	*TOX high mobility group box family member 2*	*TOX2*	8.93	8.48	6.90	0.001755603	0.00882383

7994683	124446	*transmembrane protein 219*	*TMEM219*	8.95	8.76	7.65	0.001755483	0.00882383
8074644				10.71	10.66	9.68	0.001754981	0.00882383
7897066	100128003, 199990	*hypothetical LOC100128003 | chromosome 1 open reading frame 86*	*LOC100128003|C1orf86*	7.84	7.77	6.60	0.001756348	0.008825763
8169640	292	*solute carrier family 25 (mitochondrial carrier; adenine nucleotide translocator), member 5*	*SLC25A5*	11.54	11.71	12.70	0.001757587	0.008828914
8104656	729862	*striatin, calmodulin binding protein pseudogene*	*LOC729862*	6.48	6.63	6.09	0.00175756	0.008828914
8045768	151188	*ADP-ribosylation-like factor 6 interacting protein 6*	*ARL6IP6*	7.60	7.98	9.15	0.001759269	0.008834289
8149192	140596, 503618	*defensin, beta 104A | defensin, beta 104B*	*DEFB104A|DEFB104B*	5.51	5.58	5.25	0.00175918	0.008834289
7922889	10625	*influenza virus NS1A binding protein*	*IVNS1ABP*	7.31	7.85	10.05	0.001760531	0.00883909
7959696	196385	*dynein, axonemal, heavy chain 10*	*DNAH10*	6.47	6.54	6.02	0.001760931	0.008839561
8073662	29780	*parvin, beta*	*PARVB*	10.32	10.77	9.02	0.001762077	0.008843774
7925434				7.16	6.94	6.37	0.001762886	0.008846297
8078014	6533	*solute carrier family 6 (neurotransmitter transporter, taurine), member 6*	*SLC6A6*	8.65	8.70	10.34	0.001764519	0.008852954
8132188	23080		*AVL9*	7.45	7.50	9.01	0.001766152	0.008857262
7985248	23251		*KIAA1024*	7.15	7.35	6.69	0.001766024	0.008857262
8120826	134728	*interleukin-1 receptor-associated kinase 1 binding protein 1*	*IRAK1BP1*	7.28	6.98	8.60	0.001766298	0.008857262
8016400	406943	*microRNA 152*	*MIR152*	7.24	7.32	6.39	0.001767088	0.008859686
7906069	339403	*relaxin/insulin-like family peptide receptor 4*	*RXFP4*	6.78	6.86	6.29	0.00176834	0.00886442
8031999	8612	*phosphatidic acid phosphatase type 2C*	*PPAP2C*	8.81	8.80	9.81	0.001770163	0.00887202
7951752	57646	*ubiquitin specific peptidase 28*	*USP28*	7.66	8.05	11.14	0.00177069	0.008873119
7980065				4.74	4.74	4.57	0.001771485	0.008875565
8075375	266629	*SEC14-like 3 (S. cerevisiae)*	*SEC14L3*	5.81	5.99	5.54	0.0017725	0.008879107
8119088	1026	*cyclin-dependent kinase inhibitor 1A (p21, Cip1)*	*CDKN1A*	11.49	11.77	9.05	0.001773652	0.008882782
8165171	56623	*inositol polyphosphate-5-phosphatase, 72 kDa*	*INPP5E*	9.00	8.95	7.76	0.001774407	0.008882782
8144586	66036	*myotubularin related protein 9*	*MTMR9*	7.93	8.16	9.88	0.00177407	0.008882782

8176429	50858, 100101116, 60439, 100101117	*testis-specific transcript, Y-linked 1 (non-protein coding) | testis-specific transcript, Y-linked 1B (non-protein coding) | testis-specific transcript, Y-linked 2 (non-protein coding) | testis-specific transcript, Y-linked 2B (non-protein coding)*	*TTTY1|TTTY1B|TTTY2|TTTY2B*	6.26	6.26	5.80	0.001774772	0.008882782
7911047				8.50	8.55	8.11	0.001774521	0.008882782
7962384	80070	*ADAM metallopeptidase with thrombospondin type 1 motif, 20*	*ADAMTS20*	5.50	5.46	6.05	0.001775452	0.008884644
7965156	8825	*lin-7 homolog A (C. elegans)*	*LIN7A*	7.86	6.90	8.11	0.001776764	0.008887308
8087731	51161	*chromosome 3 open reading frame 18*	*C3orf18*	8.70	8.70	7.65	0.001776907	0.008887308
7926150	9712, 439951	*USP6 N-terminal like | hypothetical LOC439951*	*USP6NL|LOC439951*	8.23	8.25	7.51	0.001776788	0.008887308
8154386				5.78	5.73	5.10	0.001777621	0.008889338
8086669	377047, 29122	*protease, serine, 45 | protease, serine, 50*	*PRSS45|PRSS50*	6.75	6.81	6.45	0.001778711	0.008893248
8103743				7.39	7.45	6.97	0.001781615	0.008906225
8131970	2128	*even-skipped homeobox 1*	*EVX1*	7.50	7.49	6.78	0.001782112	0.008907165
8170562	4101, 266740	*melanoma antigen family A, 2 | melanoma antigen family A, 2B*	*MAGEA2|MAGEA2B*	6.84	6.94	6.41	0.001783165	0.008910888
8076547	25809	*tubulin tyrosine ligase-like family, member 1*	*TTLL1*	9.21	9.05	7.86	0.001783714	0.008912086
7995793	4500	*metallothionein 1L (gene/pseudogene)*	*MT1L*	10.99	10.71	9.19	0.001785842	0.008918732
7967304	65117	*arginine/serine-rich coiled-coil 2*	*RSRC2*	7.53	7.36	8.99	0.001785798	0.008918732
8055486				8.81	8.78	7.68	0.001785971	0.008918732
7953351	9918	*non-SMC condensin I complex, subunit D2*	*NCAPD2*	9.98	9.90	11.15	0.001787518	0.008924916
8148808	727957	*HEAT repeat containing 7A*	*HEATR7A*	9.18	8.88	7.65	0.001788472	0.008928132
8159581				5.76	6.00	5.67	0.001788998	0.008929214
8047059	3183	*heterogeneous nuclear ribonucleoprotein C (C1/C2)*	*HNRNPC*	10.42	10.59	12.04	0.001791281	0.008936376
8013989	6532	*solute carrier family 6 (neurotransmitter transporter, serotonin), member 4*	*SLC6A4*	6.22	6.24	5.88	0.001791671	0.008936376
8157383	85301	*collagen, type XXVII, alpha 1*	*COL27A1*	8.58	8.45	7.84	0.001791075	0.008936376

8036981	1549, 1550	*cytochrome P450, family 2, subfamily A, polypeptide 7 | cytochrome P450, family 2, subfamily A, polypeptide 7 pseudogene 1*	*CYP2A7|CYP2A7P1*	6.78	7.24	6.46	0.001791458	0.008936376
8026214	342977	*nanos homolog 3 (Drosophila)*	*NANOS3*	8.34	8.38	7.68	0.001792949	0.008941206
8116635	670, 100130927	*biphenyl hydrolase-like (serine hydrolase) | hypothetical protein LOC100130927*	*BPHL|LOC100130927*	8.17	8.09	9.67	0.001793561	0.008941896
8116400				5.92	5.98	5.44	0.001793707	0.008941896
7973629	9985		*REC8*	7.39	7.40	8.08	0.001794367	0.008943645
8131140	100288594		*LOC100288594*	7.17	7.32	6.70	0.001794996	0.00894369
7992843				8.11	8.23	7.31	0.001794786	0.00894369
7907861	9213	*xenotropic and polytropic retrovirus receptor 1*	*XPR1*	8.56	8.52	10.22	0.001797089	0.008949014
8014755	619505	*small nucleolar RNA, H/ACA box 21*	*SNORA21*	8.13	8.42	9.80	0.001797615	0.008949014
8028607	282616, 282617	*interleukin 28A (interferon, lambda 2) | interleukin 28B (interferon, lambda 3)*	*IL28A|IL28B*	7.55	7.55	6.86	0.001797091	0.008949014
8105997	8293, 728492	*small EDRK-rich factor 1A (telomeric) | small EDRK-rich factor 1B (centromeric)*	*SERF1A|SERF1B*	7.55	7.76	8.31	0.001796411	0.008949014
7985039				7.20	7.03	6.27	0.001797479	0.008949014
7976496	12	*serpin peptidase inhibitor, clade A (alpha-1 antiproteinase, antitrypsin), member 3*	*SERPINA3*	8.30	7.16	5.55	0.001801371	0.008964623
7977249	64423	*inverted formin, FH2 and WH2 domain containing*	*INF2*	9.18	9.30	7.90	0.00180131	0.008964623
8160546	158038	*leucine rich repeat and Ig domain containing 2*	*LINGO2*	5.36	5.20	5.90	0.00180299	0.008971132
8030470	160	*adaptor-related protein complex 2, alpha 1 subunit*	*AP2A1*	11.24	11.03	9.89	0.001807002	0.008988525
7950555	2615	*leucine rich repeat containing 32*	*LRRC32*	8.93	9.83	7.40	0.001807108	0.008988525
8084923	401109		*LOC401109*	6.05	6.07	5.59	0.001809194	0.00899735
7995310	162137		*MGC34800*	7.56	7.48	6.96	0.001810082	0.009000216
8162449	83744	*zinc finger protein 484*	*ZNF484*	7.76	7.35	8.58	0.001810465	0.009000571
8178439	29113	*chromosome 6 open reading frame 15*	*C6orf15*	7.02	7.02	6.32	0.00181088	0.009001084
7961198	10748	*killer cell lectin-like receptor subfamily A pseudogene 1*	*KLRAP1*	5.39	5.35	6.92	0.001811508	0.009002653

8067985	4685	*neural cell adhesion molecule 2*	*NCAM2*	7.17	6.08	5.73	0.001813958	0.009012803
8013112	51655	*RAS, dexamethasone-induced 1*	*RASD1*	8.21	8.04	7.36	0.001814174	0.009012803
8043244	84913	*atonal homolog 8 (Drosophila)*	*ATOH8*	7.55	7.62	6.25	0.001815446	0.009017568
8130394	26575	*regulator of G-protein signaling 17*	*RGS17*	6.45	6.38	6.95	0.001817752	0.009019817
8127987	26799	*small nucleolar RNA, C/D box 50A*	*SNORD50A*	8.20	8.33	9.04	0.001818184	0.009019817
8065280	57186	*Ral GTPase activating protein, alpha subunit 2 (catalytic)*	*RALGAPA2*	6.98	6.83	8.73	0.001818542	0.009019817
8021614	89778	*serpin peptidase inhibitor, clade B (ovalbumin), member 11 (gene/pseudogene)*	*SERPINB11*	5.28	5.28	5.01	0.001817458	0.009019817
8050497	130497	*odd-skipped related 1 (Drosophila)*	*OSR1*	10.32	9.36	7.57	0.001818128	0.009019817
7896985	142678	*mindbomb homolog 2 (Drosophila)*	*MIB2*	8.37	8.35	7.52	0.001817028	0.009019817
8069841	337973	*keratin associated protein 19-6*	*KRTAP19-6*	5.28	5.13	4.88	0.001817431	0.009019817
7927153				6.62	6.47	5.87	0.001818711	0.009019817
8113701				6.27	6.20	5.61	0.001816289	0.009019817
8088844				5.54	5.45	4.77	0.001819758	0.009023461
8066786	23613	*zinc finger, MYND-type containing 8*	*ZMYND8*	7.72	8.07	9.99	0.001822359	0.009034139
8141768	10156, 401331, 100271927	*RAS p21 protein activator 4 | RAS p21 protein activator 4 pseudogene | RAS p21 protein activator 4B*	*RASA4|RASA4P|RASA4B*	9.05	9.60	7.57	0.001822537	0.009034139
8068496	6493	*single-minded homolog 2 (Drosophila)*	*SIM2*	6.95	7.68	6.87	0.001823495	0.009037333
8035477	8178	*elongation factor RNA polymerase II*	*ELL*	8.79	8.90	8.13	0.001826439	0.009048821
8120184	83741	*transcription factor AP-2 delta (activating enhancer binding protein 2 delta)*	*TFAP2D*	5.23	5.15	4.93	0.001826317	0.009048821
7993404	339044, 9284, 5310, 399491, 100288332, 642778	*polycystic kidney disease 1 (autosomal dominant) pseudogene 1 | nuclear pore complex interacting protein | GPS, PLAT and transmembrane domain-containing protein | GPS, PLAT and transmembrane domain-containing protein FLJ00285-like | nuclear pore complex-interacting protein-like 1-like*	*PKD1P1|NPIP|LOC399491|LOC100288332|LOC642778*	11.78	11.82	10.39	0.00182724	0.009051235
8065013	728450	*chromosome 20 open reading frame 61*	*C20orf61*	6.20	6.30	5.86	0.001827647	0.009051698
7933840				5.48	5.50	5.25	0.001828056	0.009052169

8166065	51311	*toll-like receptor 8*	*TLR8*	5.05	5.12	4.80	0.00183005	0.009060487
7982248				5.77	5.63	7.01	0.001830797	0.009062636
8114185	91368	*CDKN2A interacting protein N-terminal like*	*CDKN2AIPNL*	6.47	6.47	7.48	0.001832671	0.009070353
7937852	5002	*solute carrier family 22, member 18*	*SLC22A18*	9.34	9.45	7.61	0.001833515	0.009072978
8012028	433	*asialoglycoprotein receptor 2*	*ASGR2*	6.70	6.86	6.37	0.00183555	0.009073712
7927285	6009	*Ras homolog enriched in brain*	*RHEB*	9.84	10.34	10.82	0.001834757	0.009073712
8094778	7345	*ubiquitin carboxyl-terminal esterase L1 (ubiquitin thiolesterase)*	*UCHL1*	11.13	11.36	12.08	0.001835187	0.009073712
8012883	9955	*heparan sulfate (glucosamine) 3-O-sulfotransferase 3A1*	*HS3ST3A1*	8.80	9.06	7.98	0.001835286	0.009073712
7959657	23545	*ATPase, H+ transporting, lysosomal V0 subunit a2*	*ATP6V0A2*	8.40	8.76	9.84	0.001835035	0.009073712
8003848				4.80	4.75	4.63	0.001835112	0.009073712
8053429	2677	*gamma-glutamyl carboxylase*	*GGCX*	9.09	9.17	8.18	0.001837418	0.009081163
7896882	6339	*sodium channel, nonvoltage-gated 1, delta*	*SCNN1D*	7.64	7.62	6.84	0.001838	0.009081163
8097685				6.12	6.02	5.79	0.001837902	0.009081163
8024936	6294	*scaffold attachment factor B*	*SAFB*	8.42	8.37	9.54	0.001838343	0.0090813
8050594	64342	*HCLS1 binding protein 3*	*HS1BP3*	8.42	8.16	7.38	0.001839007	0.009083026
8116530	619571	*small nucleolar RNA, C/D box 96A*	*SNORD96A*	7.55	7.40	9.14	0.001840655	0.009089611
7924863	84033, 574407	*obscurin, cytoskeletal calmodulin and titin-interacting RhoGEF | chromosome 1 open reading frame 145*	*OBSCN|C1orf145*	6.74	6.72	6.03	0.001841976	0.009094577
7907286	2326	*flavin containing monooxygenase 1*	*FMO1*	6.47	5.20	4.97	0.001843602	0.009100972
7940781	10313	*reticulon 3*	*RTN3*	7.60	7.69	8.48	0.001844532	0.009100972
8027330	84108	*polycomb group ring finger 6*	*PCGF6*	8.40	8.76	10.42	0.001844242	0.009100972
8162247				7.56	7.68	7.18	0.001844394	0.009100972
7898607				8.22	8.30	7.80	0.001845738	0.009105367
7980352	58157	*neuroglobin*	*NGB*	7.43	7.44	6.95	0.001846054	0.009105369
8105647	80006	*chromosome 5 open reading frame 44*	*C5orf44*	6.74	6.86	7.82	0.001846471	0.009105871
7917232	2787	*guanine nucleotide binding protein (G protein), gamma 5*	*GNG5*	10.32	10.79	9.47	0.00184709	0.009107367

7983391				4.81	4.75	4.57	0.001847534	0.009108002
8054766	49	*acrosin*	*ACR*	7.15	7.27	6.68	0.00184813	0.009109385
8054611	112597, 541471	*non-protein coding RNA 152 | hypothetical LOC541471*	*NCRNA00152|LOC541471*	11.37	11.69	10.07	0.001848456	0.009109438
8083737	406949	*microRNA 15b*	*MIR15B*	5.47	5.04	5.89	0.001849813	0.009114568
7967034	5319	*phospholipase A2, group IB (pancreas)*	*PLA2G1B*	7.40	6.98	6.18	0.001850642	0.009115279
8022085	9229	*discs, large (Drosophila) homolog-associated protein 1*	*DLGAP1*	7.06	7.09	7.66	0.001850511	0.009115279
7949264	10938	*EH-domain containing 1*	*EHD1*	10.25	9.86	8.27	0.001850905	0.009115279
8027860	2865	*free fatty acid receptor 3*	*FFAR3*	6.07	5.98	5.52	0.001853562	0.009125252
7908732	55705	*importin 9*	*IPO9*	9.86	10.09	11.18	0.001853391	0.009125252
7969959	267012	*D-amino acid oxidase activator*	*DAOA*	4.85	4.76	4.67	0.001856012	0.009135755
8039389	5794	*protein tyrosine phosphatase, receptor type, H*	*PTPRH*	6.33	6.56	6.01	0.001858469	0.009146289
7905233	54507	*ADAMTS-like 4*	*ADAMTSL4*	9.74	8.80	6.93	0.001859651	0.009150548
8098496				5.34	5.07	5.08	0.001860059	0.009150995
8059710	6082	*small nucleolar RNA, C/D box 20*	*SNORD20*	4.65	4.54	5.91	0.001861988	0.009157363
7939005				5.99	6.24	5.27	0.001861908	0.009157363
7931951	57713	*Scm-like with four mbt domains 2*	*SFMBT2*	6.38	6.42	7.67	0.001863943	0.009163612
8074577	375133, 728233	*phosphatidylinositol 4-kinase, catalytic, alpha pseudogene 2 | phosphatidylinositol 4-kinase, catalytic, alpha pseudogene 1*	*PI4KAP2|PI4KAP1*	10.29	10.27	9.37	0.001863591	0.009163612
8000200	730092, 54700, 653390	*RNA polymerase I transcription factor homolog (S. cerevisiae) pseudogene 1 | RRN3 RNA polymerase I transcription factor homolog (S. cerevisiae) | RNA polymerase I transcription factor homolog (S. cerevisiae) pseudogene 2*	*RRN3P1|RRN3P2*	9.13	9.03	10.77	0.001864211	0.009163612
8003719	3090	*hypermethylated in cancer 1*	*HIC1*	8.62	8.80	7.48	0.001865629	0.009169024
7951224	9313	*matrix metallopeptidase 20*	*MMP20*	5.71	5.78	5.31	0.001866221	0.009169052
7925611	64216	*transcription factor B2, mitochondrial*	*TFB2M*	8.74	8.84	10.19	0.00186627	0.009169052
8154981	10497	*unc-13 homolog B (C. elegans)*	*UNC13B*	8.39	8.18	9.41	0.001867026	0.009170727

8053139	388963	*chromosome 2 open reading frame 81*	*C2orf81*	8.51	8.44	7.38	0.001867246	0.009170727
8148261				4.98	5.09	4.71	0.001872814	0.009196508
8032839	10501	*sema domain, transmembrane domain (TM), and cytoplasmic domain, (semaphorin) 6B*	*SEMA6B*	7.28	7.29	7.64	0.001873746	0.009197956
8173848	139324	*highly divergent homeobox*	*HDX*	6.34	6.23	7.43	0.00187373	0.009197956
8168852	3188	*heterogeneous nuclear ribonucleoprotein H2 (H')*	*HNRNPH2*	8.44	8.20	10.23	0.001874097	0.009198112
8081941				5.49	5.62	5.24	0.001875241	0.009202166
8177344	83863	*testis-specific transcript, Y-linked 5 (non-protein coding)*	*TTTY5*	6.77	6.67	6.28	0.001876322	0.009205907
8014485				8.01	7.95	7.28	0.001877959	0.009212369
8062064	57644	*myosin, heavy chain 7B, cardiac muscle, beta*	*MYH7B*	7.04	7.10	6.39	0.001878335	0.009212651
7918359	57463	*adhesion molecule with Ig-like domain 1*	*AMIGO1*	8.96	8.53	7.71	0.001883514	0.009236483
8124423	8339	*histone cluster 1, H2bg*	*HIST1H2BG*	7.02	7.55	9.17	0.001884394	0.009239228
7987634				5.77	5.88	5.52	0.001885906	0.009245073
8054945	274	*bridging integrator 1*	*BIN1*	10.66	10.41	8.97	0.001887082	0.009249263
7995843	9688	*nucleoporin 93kDa*	*NUP93*	9.38	9.53	11.45	0.001891452	0.009269112
8163731				8.98	8.99	8.21	0.001893137	0.00927579
8001030	29108	*PYD and CARD domain containing*	*PYCARD*	10.21	9.23	8.97	0.001893638	0.009276671
8169085	139231	*family with sequence similarity 199, X-linked*	*FAM199X*	7.96	7.93	9.02	0.001896122	0.009287267
8074701	6545	*solute carrier family 7 (cationic amino acid transporter, y+ system), member 4*	*SLC7A4*	7.31	7.20	6.78	0.00189739	0.009291901
8089291				4.85	4.91	5.55	0.001899623	0.009301255
8047738	8828	*neuropilin 2*	*NRP2*	7.36	8.97	8.18	0.001900305	0.009303018
8007757	752	*formin-like 1*	*FMNL1*	8.59	8.30	7.25	0.001901777	0.009308648
8117547	10279	*protease, serine, 16 (thymus)*	*PRSS16*	6.72	6.58	7.19	0.001904145	0.009316744
8067798	54345	*SRY (sex determining region Y)-box 18*	*SOX18*	8.74	8.57	7.99	0.001903975	0.009316744
8050253	100302743	*small nucleolar RNA, H/ACA box 80B*	*SNORA80B*	7.84	8.22	7.31	0.0019044	0.009316744
8016735	55018	*chromosome 17 open reading frame 73*	*C17orf73*	6.36	6.36	5.97	0.001906412	0.009317892

8061831	149954	*chromosome 20 open reading frame 186*	*C20orf186*	6.33	6.43	5.96	0.001905663	0.009317892
7970793	283537	*solute carrier family 46, member 3*	*SLC46A3*	7.89	7.14	5.56	0.001906893	0.009317892
8010560	100129503		*LOC100129503*	6.95	6.70	5.50	0.001905907	0.009317892
8048077				4.45	4.46	4.37	0.00190629	0.009317892
8102869				6.24	6.23	5.63	0.001905848	0.009317892
8128308				6.70	6.83	6.10	0.001906687	0.009317892
7989924	10302	*small nuclear RNA activating complex, polypeptide 5, 19kDa*	*SNAPC5*	7.55	7.53	8.67	0.001908333	0.009323347
8042925	10505	*sema domain, immunoglobulin domain (Ig), transmembrane domain (TM) and short cytoplasmic domain, (semaphorin) 4F*	*SEMA4F*	8.14	8.00	6.95	0.001909288	0.009324858
8005739	284123	*family with sequence similarity 27-like*	*FAM27L*	7.11	7.12	6.62	0.001909034	0.009324858
8165538	377841	*ectonucleoside triphosphate diphosphohydrolase 8*	*ENTPD8*	7.29	7.53	6.75	0.001912411	0.009338021
8126301				8.80	8.89	7.99	0.00191263	0.009338021
8117840	135644	*tripartite motif-containing 40*	*TRIM40*	6.42	6.48	5.99	0.001912999	0.009338242
8121532	8838	*WNT1 inducible signaling pathway protein 3*	*WISP3*	5.84	5.86	5.53	0.001914158	0.009342322
7933290	728053, 399761, 728127, 642517	*BMS1 pseudogene 1 | BMS1 pseudogene 5 | ArfGAP with GTPase domain, ankyrin repeat and PH domain 10 | ArfGAP with GTPase domain, ankyrin repeat and PH domain 9*	*BMS1P1|BMS1P5|AGAP10|AGAP9*	7.44	7.33	9.24	0.0019152	0.009345824
8126312	7942	*transcription factor EB*	*TFEB*	7.81	7.97	6.90	0.001917258	0.00935008
7942032	55291	*protein phosphatase 6, regulatory subunit 3*	*PPP6R3*	8.79	8.71	10.65	0.001917367	0.00935008
8009873	80022	*myosin XVB pseudogene*	*MYO15B*	7.38	7.01	6.33	0.001916817	0.00935008
8035297	83483	*plasmalemma vesicle associated protein*	*PLVAP*	7.58	7.75	7.01	0.00191732	0.00935008
8164833	26301, 5900	*globoside alpha-1,3-N-acetylgalactosaminyltransferase 1 | ral guanine nucleotide dissociation stimulator*	*GBGT1|RALGDS*	9.16	8.50	7.63	0.001919977	0.009361225
7915392	59269	*human immunodeficiency virus type I enhancer binding protein 3*	*HIVEP3*	6.76	7.28	6.22	0.001921599	0.00936123
7904796	148741	*ankyrin repeat domain 35*	*ANKRD35*	6.63	6.61	6.15	0.001921526	0.00936123
7936516	259217	*heat shock 70kDa protein 12A*	*HSPA12A*	7.57	8.06	7.38	0.001920943	0.00936123

8068044				5.05	4.99	4.64	0.001921487	0.00936123
8081337				4.75	4.81	4.57	0.001920559	0.00936123
8125825	11165	*nudix (nucleoside diphosphate linked moiety X)-type motif 3*	*NUDT3*	9.95	10.14	10.89	0.001921951	0.009361365
7924619	55740	*enabled homolog (Drosophila)*	*ENAH*	9.76	10.24	11.42	0.00192235	0.009361727
8104856	79925	*sperm flagellar 2*	*SPEF2*	5.11	5.14	4.77	0.001926458	0.009378834
8006606				5.69	5.43	5.21	0.001926512	0.009378834
8114658	1729	*diaphanous homolog 1 (Drosophila)*	*DIAPH1*	8.48	8.62	9.36	0.00192944	0.00938992
7963244	7024	*transcription factor CP2*	*TFCP2*	7.80	7.85	9.46	0.00192944	0.00938992
8168399	53344	*cysteine-rich hydrophobic domain 1*	*CHIC1*	7.87	7.53	8.64	0.001933905	0.00941006
8071758	4320	*matrix metallopeptidase 11 (stromelysin 3)*	*MMP11*	8.73	8.45	7.84	0.001935641	0.00941689
8074617	91179, 84861	*scavenger receptor class F, member 2 | kelch-like 22 (Drosophila)*	*SCARF2|KLHL22*	9.70	10.09	8.09	0.001935961	0.00941689
8092409	55486	*presenilin associated, rhomboid-like*	*PARL*	8.14	8.28	8.87	0.001936386	0.009417375
8067551	140865	*chromosome 20 open reading frame 90*	*C20orf90*	8.01	8.07	7.35	0.001937827	0.009422792
7914202	85028	*small nucleolar RNA host gene 12 (non-protein coding)*	*SNHG12*	8.99	9.36	10.43	0.001938613	0.00942406
8002344	118460	*exosome component 6*	*EXOSC6*	10.06	10.18	9.54	0.00193874	0.00942406
8100347	152579	*sec1 family domain containing 2*	*SCFD2*	9.86	9.98	8.95	0.001939119	0.009424316
8151376	6129	*ribosomal protein L7*	*RPL7*	12.02	11.92	12.43	0.001939707	0.009425584
7933204	11067	*chromosome 10 open reading frame 10*	*C10orf10*	9.22	8.66	6.63	0.001940611	0.009426413
8121300	55278	*glutaminyl-tRNA synthase (glutamine-hydrolyzing)-like 1*	*QRSL1*	7.29	7.35	9.62	0.001940798	0.009426413
7965589	120935	*coiled-coil domain containing 38*	*CCDC38*	5.19	5.19	4.90	0.001940857	0.009426413
8114225	9555	*H2A histone family, member Y*	*H2AFY*	9.58	9.66	8.99	0.001941822	0.009429512
7994487	930		*CD19*	7.21	7.34	6.79	0.001942331	0.009429777
7903079	1810	*down-regulator of transcription 1, TBP-binding (negative cofactor 2)*	*DR1*	9.67	9.84	10.74	0.00194253	0.009429777
8090462				7.13	7.11	6.56	0.001943683	0.009433792
8114628	3035	*histidyl-tRNA synthetase*	*HARS*	10.49	10.50	11.10	0.001944863	0.009436347
8133155	8460	*tyrosylprotein sulfotransferase 1*	*TPST1*	10.48	10.14	9.10	0.001944733	0.009436347

7969438	4008	*LIM domain 7*	*LMO7*	9.55	9.48	7.24	0.001945237	0.009436574
8033825	50509	*collagen, type V, alpha 3*	*COL5A3*	8.11	8.35	7.26	0.001947491	0.00944592
8078214	5868		*RAB5A*	10.06	10.10	11.36	0.001948489	0.009447838
7941274	57410, 4054	*SCY1-like 1 (S. cerevisiae) | latent transforming growth factor beta binding protein 3*	*SCYL1|LTBP3*	11.57	11.45	10.25	0.001948541	0.009447838
7899424	93974	*ATPase inhibitory factor 1*	*ATPIF1*	7.86	7.86	8.44	0.001948877	0.009447883
8138670	3181	*heterogeneous nuclear ribonucleoprotein A2/B1*	*HNRNPA2B1*	8.86	8.67	11.74	0.001949311	0.009448398
8033912	1786	*DNA (cytosine-5-)-methyltransferase 1*	*DNMT1*	9.36	9.36	10.53	0.001949676	0.009448581
8075992	6663	*SRY (sex determining region Y)-box 10*	*SOX10*	7.24	7.37	7.01	0.001951803	0.009454128
7933312	728113, 653145, 244	*annexin A8-like 1 | annexin A8 | annexin A8-like 2*	*ANXA8L1|ANXA8L2*	6.77	6.68	6.32	0.001951413	0.009454128
8022610				6.55	6.52	6.04	0.001951563	0.009454128
8029107	1084	*carcinoembryonic antigen-related cell adhesion molecule 3*	*CEACAM3*	7.76	7.78	6.98	0.001952966	0.009458173
8175234	2719	*glypican 3*	*GPC3*	10.17	6.39	11.00	0.001956842	0.009475355
8043782	1261	*cyclic nucleotide gated channel alpha 3*	*CNGA3*	6.64	6.92	6.04	0.001957476	0.009476835
8095187	9662	*centrosomal protein 135kDa*	*CEP135*	6.20	6.21	7.86	0.001958937	0.00947756
8094271	80306	*mediator complex subunit 28*	*MED28*	9.24	9.16	10.68	0.001958698	0.00947756
8105741	375449	*microtubule associated serine/threonine kinase family member 4*	*MAST4*	7.76	7.50	7.03	0.001959267	0.00947756
8161442	724094	*family with sequence similarity 27, member D1*	*FAM27D1*	8.45	8.75	7.85	0.001959094	0.00947756
8162462	23196, 445577	*family with sequence similarity 120A | chromosome 9 open reading frame 129*	*FAM120A|C9orf129*	11.33	11.36	10.57	0.001958582	0.00947756
7913206	54546	*ring finger protein 186*	*RNF186*	7.32	7.45	6.76	0.001959812	0.009478606
7967698				5.10	5.11	5.98	0.001961959	0.009487402
7967002	5829	*paxillin*	*PXN*	9.83	9.78	8.90	0.00196289	0.009488723
8095251				6.59	6.59	5.98	0.001962777	0.009488723
7917672				7.28	7.33	6.91	0.001964414	0.009494503
7967794	23141	*ankyrin repeat and LEM domain containing 2*	*ANKLE2*	9.35	9.98	9.45	0.001966569	0.009503327
8057719	26275	*3-hydroxyisobutyryl-CoA hydrolase*	*HIBCH*	6.68	6.18	8.42	0.001967854	0.009507946

8063345	26765	*small nucleolar RNA, C/D box 12C*	*SNORD12C*	7.82	7.76	9.36	0.001968436	0.009509166
8012598	5957	*recoverin*	*RCVRN*	7.52	6.86	5.95	0.001971977	0.009523988
8164632	84929	*fibrinogen C domain containing 1*	*FIBCD1*	7.95	8.03	7.22	0.001972164	0.009523988
7997072	197258	*fucokinase*	*FUK*	7.46	7.45	6.75	0.001974766	0.009532885
8126937	245927	*defensin, beta 113*	*DEFB113*	6.10	6.26	5.65	0.001974997	0.009532885
7898858	148898 80818	*chromosome 1 open reading frame 213 | zinc finger protein 436*	*C1orf213|ZNF436*	6.86	6.75	6.05	0.001974975	0.009532885
8137783	202915	*transmembrane protein 184A*	*TMEM184A*	7.31	7.39	6.91	0.001975463	0.009533539
8062545	79913	*ARP5 actin-related protein 5 homolog (yeast)*	*ACTR5*	7.51	7.62	8.37	0.001977895	0.009543682
8105523	3796	*kinesin heavy chain member 2A*	*KIF2A*	9.26	9.34	11.13	0.001978864	0.00954676
7997767	161882	*zinc finger protein, multitype 1*	*ZFPM1*	8.73	8.76	7.78	0.001979683	0.009549118
8063549				9.07	9.43	8.19	0.001980088	0.009549474
7939897	2346	*folate hydrolase (prostate-specific membrane antigen) 1*	*FOLH1*	7.77	7.92	6.41	0.001984039	0.009566932
7930162	54838	*chromosome 10 open reading frame 26*	*C10orf26*	9.34	8.97	8.23	0.001986903	0.009579141
7905881	8751	*ADAM metallopeptidase domain 15*	*ADAM15*	9.24	9.08	7.67	0.001988546	0.009583601
8175616	51402, 100130086	*heat shock transcription factor family, X linked 1 | heat shock transcription factor family, X linked 2*	*HSFX1|HSFX2*	7.03	6.91	6.43	0.001988431	0.009583601
7903115				4.98	5.06	4.76	0.001988824	0.009583601
8055478	11249	*neurexophilin 2*	*NXPH2*	7.17	7.24	7.95	0.001989308	0.009584335
7985577	54993	*zinc finger and SCAN domain containing 2*	*ZSCAN2*	7.24	7.22	8.41	0.001991378	0.009592708
8073824	406884, 400931	*microRNA let-7b | hypothetical LOC400931*	*MIRLET7B|LOC400931*	8.21	8.21	7.34	0.001992773	0.009597823
8015545	5878		*RAB5C*	12.90	12.87	12.09	0.001993316	0.009597977
8029937				6.76	6.83	6.45	0.001993469	0.009597977
8024888	284424	*chromosome 19 open reading frame 30*	*C19orf30*	6.18	6.27	5.92	0.001994947	0.009603491
7908125	23179	*ral guanine nucleotide dissociation stimulator-like 1*	*RGL1*	9.34	9.10	7.55	0.001995677	0.009603979
8091546	645843	*transmembrane protein 14E*	*TMEM14E*	5.89	5.88	5.63	0.001995714	0.009603979

8132376	83930	*STARD3 N-terminal like*	*STARD3NL*	8.50	8.66	9.35	0.001997878	0.009612788
8131519	9678	*PHD finger protein 14*	*PHF14*	8.24	7.93	9.95	0.001999442	0.009617107
8031939	201514	*zinc finger protein 584*	*ZNF584*	8.34	8.29	7.36	0.001999344	0.009617107
8083318	646903		*LOC646903*	6.15	6.15	6.60	0.00200007	0.009618525
8074842	81926, 100289252	*family with sequence similarity 108, member A1 | family with sequence similarity 108, member A11, pseudogene*	*FAM108A1 |FAM108A11P*	10.51	10.44	9.19	0.002000759	0.00962024
8046604	8540	*alkylglycerone phosphate synthase*	*AGPS*	7.94	7.84	9.69	0.002002591	0.009627442
8062728	10110	*serum/glucocorticoid regulated kinase 2*	*SGK2*	6.60	6.55	6.14	0.002004989	0.009636697
7979524	161291	*transmembrane protein 30B*	*TMEM30B*	7.63	7.17	8.19	0.002005183	0.009636697
7990511	5780	*protein tyrosine phosphatase, non-receptor type 9*	*PTPN9*	10.11	10.21	9.61	0.002005892	0.009638499
8117165	6659	*SRY (sex determining region Y)-box 4*	*SOX4*	8.48	8.52	9.07	0.002007217	0.009640955
8160487	9373	*phospholipase A2-activating protein*	*PLAA*	8.86	9.00	10.41	0.002007497	0.009640955
7922108	23432	*G protein-coupled receptor 161*	*GPR161*	8.18	8.26	7.58	0.002007739	0.009640955
7919761				7.12	7.13	8.70	0.00200761	0.009640955
8125993	51513	*ets variant 7*	*ETV7*	7.14	6.93	6.39	0.002008421	0.009642624
8059387	79843	*family with sequence similarity 124B*	*FAM124B*	6.49	6.41	6.86	0.002010098	0.009649071
7941927	9049	*aryl hydrocarbon receptor interacting protein*	*AIP*	9.07	8.83	7.76	0.002011009	0.009651838
8039905	56900	*transmembrane protein 167B*	*TMEM167B*	10.59	10.46	9.71	0.002013317	0.009657542
8111925	389289	*chromosome 5 open reading frame 39*	*C5orf39*	5.79	5.81	5.31	0.00201316	0.009657542
7978664				6.31	6.40	5.77	0.002013535	0.009657542
8176779				6.04	6.01	5.56	0.002013132	0.009657542
7980537	85439	*stonin 2*	*STON2*	6.25	5.93	7.07	0.002014627	0.009661173
8001072	24150, 729355	*TP53 target 3 | TP53 target 3B*	*TP53TG3| TP53TG3B*	6.00	6.06	5.57	0.002015154	0.009662095
8053480				8.06	8.09	7.45	0.002015524	0.009662265
8066683	64405	*cadherin 22, type 2*	*CDH22*	8.06	8.06	7.51	0.002016287	0.009663737

7925182	677802	*small nucleolar RNA, H/ACA box 14B*	*SNORA14B*	5.67	5.94	6.97	0.002016501	0.009663737
8137986	54476	*ring finger protein 216*	*RNF216*	8.83	8.83	8.29	0.002017726	0.009667232
8106401				6.32	6.02	5.66	0.002017899	0.009667232
8076046	25829	*transmembrane protein 184B*	*TMEM184B*	11.87	11.72	10.27	0.002019458	0.009673094
8103378	51802	*amiloride-sensitive cation channel 5, intestinal*	*ACCN5*	4.96	4.96	4.76	0.002020158	0.009674842
8159531	401562	*lipocalin-like 1*	*LCNL1*	7.79	7.78	7.14	0.002023101	0.00968572
8067111				5.08	5.18	4.91	0.002023045	0.00968572
8107992	134548	*ankyrin repeat domain 43*	*ANKRD43*	8.19	8.22	7.74	0.002023456	0.009685816
8001918	6236	*Ras-related associated with diabetes*	*RRAD*	6.56	6.54	5.90	0.002023843	0.009686062
8165817	8908	*glycogenin 2*	*GYG2*	8.52	7.82	9.45	0.00202515	0.009690711
8060803				6.50	6.62	6.09	0.002026506	0.009695591
8006820	3927	*LIM and SH3 protein 1*	*LASP1*	11.95	11.95	10.82	0.002026952	0.009696117
8026363				6.58	6.50	6.02	0.002030765	0.009711138
8115366				4.58	4.96	4.55	0.002030691	0.009711138
8069620	54148	*mitochondrial ribosomal protein L39*	*MRPL39*	8.80	8.79	10.73	0.002032229	0.009716534
8178554	80741	*lymphocyte antigen 6 complex, locus G5C*	*LY6G5C*	7.92	7.34	6.64	0.002033113	0.009719146
8076533	11252	*protein kinase C and casein kinase substrate in neurons 2*	*PACSIN2*	9.76	9.74	10.26	0.002035501	0.009722516
7903586	56900	*transmembrane protein 167B*	*TMEM167B*	10.58	10.46	9.71	0.002035233	0.009722516
7984892	79748	*lectin, mannose-binding, 1 like*	*LMAN1L*	6.84	6.93	6.27	0.002035243	0.009722516
8146388	401459	*hypothetical LOC401459*	*FLJ46365*	6.26	6.41	5.75	0.002035004	0.009722516
8179007	442194	*olfactory receptor, family 10, subfamily C, member 1*	*OR10C1*	6.13	6.19	5.47	0.002034496	0.009722516
7949058	3338	*DnaJ (Hsp40) homolog, subfamily C, member 4*	*DNAJC4*	7.30	7.23	6.55	0.002036507	0.009725709
8175998	4204	*methyl CpG binding protein 2 (Rett syndrome)*	*MECP2*	9.40	9.34	8.81	0.002039844	0.009740034
8166104	8481	*oral-facial-digital syndrome 1*	*OFD1*	6.78	6.84	8.08	0.00204028	0.009740504
8138757	3206	*homeobox A10*	*HOXA10*	6.97	7.45	6.62	0.002041427	0.009741131
8059996	8864	*period homolog 2 (Drosophila)*	*PER2*	8.53	8.76	7.53	0.002041761	0.009741131

8129273	221322	*chromosome 6 open reading frame 170*	*C6orf170*	6.09	6.02	7.32	0.002041636	0.009741131
8159549	2529, 401563	*fucosyltransferase 7 (alpha (1,3) fucosyltransferase) | chromosome 9 open reading frame 139*	*FUT7|C9orf139*	7.20	7.18	6.51	0.002040858	0.009741131
8123893	4117	*male germ cell-associated kinase*	*MAK*	5.32	5.30	5.49	0.002042984	0.009745358
8165705	4541	*NADH dehydrogenase, subunit 6 (complex I)*	*ND6*	7.76	7.56	8.42	0.002048258	0.00976729
8035435	80726		*KIAA1683*	7.59	7.55	6.81	0.002048069	0.00976729
8013399	9706	*unc-51-like kinase 2 (C. elegans)*	*ULK2*	8.64	8.48	7.49	0.002049231	0.009770315
8078262	7324	*ubiquitin-conjugating enzyme E2E 1 (UBC4/5 homolog, yeast)*	*UBE2E1*	9.13	9.34	9.93	0.002050157	0.009773113
8108633	56135, 56134, 56137, 56142, 56141, 9752, 56147, 56139, 56138, 56144, 56136, 56146, 56145, 56140, 56143	*protocadherin alpha subfamily C, 1 | protocadherin alpha subfamily C, 2 | protocadherin alpha 12 | protocadherin alpha 6 | protocadherin alpha 7 | protocadherin alpha 9 | protocadherin alpha 1 | protocadherin alpha 10 | protocadherin alpha 11 | protocadherin alpha 4 | protocadherin alpha 13 | protocadherin alpha 2 | protocadherin alpha 3 | protocadherin alpha 8 | protocadherin alpha 5*	*PCDHAC1|PCDHAC2|PCDHA12|PCDHA6|PCDHA7|PCDHA9|PCDHA10|PCDHA11|PCDHA4|PCDHA13|PCDHA2|PCDHA3|PCDHA8|PCDHA5*	6.31	6.32	6.77	0.00205447	0.009792056
8042696	6697	*sepiapterin reductase (7,8-dihydrobiopterin:NADP+ oxidoreductase)*	*SPR*	9.51	8.93	8.00	0.002055022	0.009793074
7949540	83638	*chromosome 11 open reading frame 68*	*C11orf68*	9.82	9.84	8.45	0.002055871	0.0097955
7966345	160760	*PTC7 protein phosphatase homolog (S. cerevisiae)*	*PPTC7*	7.78	7.47	8.98	0.002056249	0.009795687
8018793	23210	*jumonji domain containing 6*	*JMJD6*	9.15	9.64	8.45	0.002058007	0.009800823
8153336	57152	*secreted LY6/PLAUR domain containing 1*	*SLURP1*	7.28	7.32	6.76	0.002057882	0.009800823
8034043	53637	*sphingosine-1-phosphate receptor 5*	*S1PR5*	8.38	8.38	7.84	0.002058349	0.009800839
8121757	3298	*heat shock transcription factor 2*	*HSF2*	8.06	8.28	9.27	0.002059191	0.009803229
8149084	100131112		*LOC100131112*	5.65	5.76	5.41	0.002059841	0.009804709

8071179	8214	*DiGeorge syndrome critical region gene 6*	*DGCR6*	7.95	7.99	7.24	0.002062047	0.009806732
7984819	80125	*coiled-coil domain containing 33*	*CCDC33*	6.73	6.76	6.44	0.002061989	0.009806732
8143127	389558	*family with sequence similarity 180, member A*	*FAM180A*	8.32	9.35	6.44	0.002061224	0.009806732
8072316	550631	*coiled-coil domain containing 157*	*CCDC157*	7.29	7.42	6.78	0.002061922	0.009806732
8153426	642475	*chromosome 8 open reading frame 73*	*C8orf73*	9.16	9.17	8.36	0.002062304	0.009806732
7968928	5954, 100190939	*reticulocalbin 1, EF-hand calcium binding domain | hypothetical LOC100190939*	*RCN1|LOC100190939*	9.27	9.17	8.34	0.002062018	0.009806732
7990965	6218	*ribosomal protein S17*	*RPS17*	13.00	13.05	13.20	0.002067588	0.009830235
8128409	85015	*ubiquitin specific peptidase 45*	*USP45*	7.24	7.45	8.88	0.002067992	0.009830536
7927998	80201	*hexokinase domain containing 1*	*HKDC1*	6.53	6.53	6.31	0.00206918	0.009834565
8050007	7837	*peroxidasin homolog (Drosophila)*	*PXDN*	12.25	12.25	11.02	0.002070512	0.009839277
7972977				6.67	6.81	6.03	0.002071681	0.009843214
8013384	218	*aldehyde dehydrogenase 3 family, member A1*	*ALDH3A1*	7.38	7.33	6.58	0.002073119	0.009848426
7981439	9529	*BCL2-associated athanogene 5*	*BAG5*	7.47	7.47	8.48	0.002077265	0.009864872
8030429	83596, 3661	*BCL2-like 12 (proline rich) | interferon regulatory factor 3*	*BCL2L12|IRF3*	7.52	7.66	8.55	0.002077165	0.009864872
8051241	238	*anaplastic lymphoma receptor tyrosine kinase*	*ALK*	6.28	6.39	6.11	0.002079538	0.009874043
8094848	10463	*solute carrier family 30 (zinc transporter), member 9*	*SLC30A9*	9.20	9.01	10.42	0.002081504	0.009881751
8125750	6136	*ribosomal protein L12*	*RPL12*	12.74	12.70	12.90	0.002082449	0.009884614
8098554				4.87	4.78	4.63	0.002083102	0.009886086
7962183	205	*adenylate kinase 4*	*AK4*	7.03	9.67	10.03	0.002086984	0.009900999
7949440	254102	*EH domain binding protein 1-like 1*	*EHBP1L1*	7.51	7.56	6.76	0.002087616	0.009900999
7952795				6.92	6.92	7.53	0.002087275	0.009900999
8052667				7.15	6.86	6.50	0.002087041	0.009900999
7976755	283600	*solute carrier family 25, member 47*	*SLC25A47*	7.44	7.60	6.89	0.002089289	0.009907304
7925677	79862	*zinc finger protein 669*	*ZNF669*	6.59	6.83	7.66	0.0020901	0.009909524
8101489	84142, 51023	*family with sequence similarity 175, member A | mitochondrial ribosomal protein S18C*	*FAM175A|MRPS18C*	7.01	7.05	9.69	0.002090473	0.009909667

8095705	441024	*methylenetetrahydrofolate dehydrogenase (NADP+ dependent) 2-like*	*MTHFD2L*	5.89	5.96	6.67	0.002092519	0.009917735
7936762	11101	*arginyltransferase 1*	*ATE1*	8.37	8.19	9.54	0.002095616	0.009930784
8106170	134285	*transmembrane protein 171*	*TMEM171*	8.83	9.35	7.59	0.002096753	0.009934542
8061982	434	*agouti signaling protein*	*ASIP*	7.05	7.10	6.51	0.002098127	0.009939013
8010614	25794	*fascin homolog 2, actin-bundling protein, retinal (Strongylocentrotus purpuratus)*	*FSCN2*	7.78	8.03	7.08	0.002098385	0.009939013
8139881				6.87	6.82	5.96	0.002099197	0.009941228
8069083	755	*chromosome 21 open reading frame 2*	*C21orf2*	8.25	8.28	7.29	0.00209992	0.009941388
7990700	80349	*WD repeat domain 61*	*WDR61*	8.03	8.21	8.97	0.002099763	0.009941388
8072413	6525	*smoothelin*	*SMTN*	9.82	10.01	7.97	0.002100916	0.009944472
8011832	4927	*nucleoporin 88kDa*	*NUP88*	9.04	9.64	11.43	0.002103076	0.009953065
7988082	146050	*zinc finger and SCAN domain containing 29*	*ZSCAN29*	7.32	7.52	8.52	0.002103682	0.009954302
8107897	8572	*PDZ and LIM domain 4*	*PDLIM4*	9.40	9.53	7.61	0.002104714	0.009954974
8031576	147945	*NLR family, pyrin domain containing 4*	*NLRP4*	5.79	5.70	5.44	0.002104859	0.009954974
8174281				5.12	5.15	6.65	0.002104688	0.009954974
7967021	5829	*paxillin*	*PXN*	8.29	8.18	7.82	0.00210579	0.009955195
8063043	11065	*ubiquitin-conjugating enzyme E2C*	*UBE2C*	7.74	8.26	9.53	0.00210594	0.009955195
8090507				8.22	8.42	7.22	0.0021057	0.009955195
7987572				12.85	12.90	12.22	0.002106735	0.009957322
7982290				6.78	6.54	9.08	0.002107772	0.009960585
8072582				6.51	6.55	7.80	0.002108115	0.009960585
8104129				5.78	5.88	5.41	0.002109527	0.009965624
7950490	442900	*microRNA 326*	*MIR326*	9.10	9.18	8.66	0.002110169	0.009967028
8053165	116540, 84865	*mitochondrial ribosomal protein L53 | coiled-coil domain containing 142*	*MRPL53|CCDC142*	11.50	11.37	10.27	0.002110706	0.00996793
7903519	55119	*PRP38 pre-mRNA processing factor 38 (yeast) domain containing B*	*PRPF38B*	8.05	7.72	9.38	0.002113545	0.009979709
8083240	185	*angiotensin II receptor, type 1*	*AGTR1*	6.80	7.28	6.23	0.002114654	0.009982327
8078759	9390	*solute carrier family 22 (organic anion transporter), member 13*	*SLC22A13*	6.44	6.45	6.30	0.002114791	0.009982327

8014282	79148	*matrix metallopeptidase 28*	*MMP28*	7.52	7.59	6.95	0.002115334	0.009983256
8089785	64091, 10063	*popeye domain containing 2 | COX17 cytochrome c oxidase assembly homolog (S. cerevisiae)*	*POPDC2| COX17*	5.78	6.36	5.45	0.00211909	0.00999838
8036479	90522, 64073	*Yip1 interacting factor homolog B (S. cerevisiae) | chromosome 19 open reading frame 33*	*YIF1B|C19 orf33*	8.15	8.21	7.67	0.002119231	0.00999838
8159501	286256	*lipocalin 12*	*LCN12*	7.39	7.54	7.06	0.002120283	0.010000072
8020491				6.09	6.25	6.67	0.002120156	0.010000072
8119427	285852	*triggering receptor expressed on myeloid cells-like 4*	*TREML4*	6.54	6.60	6.10	0.002123188	0.010010788
7938625				7.48	7.76	6.61	0.002123248	0.010010788
8114491	9963	*solute carrier family 23 (nucleobase transporters), member 1*	*SLC23A1*	7.45	7.37	6.96	0.002123825	0.010011873
7949650	8722	*cathepsin F*	*CTSF*	9.84	9.86	8.23	0.002124483	0.01001334
8141533				6.07	6.08	5.66	0.002125864	0.010018214
7946245	8642	*dachsous 1 (Drosophila)*	*DCHS1*	9.65	9.03	7.42	0.002126896	0.010019803
8026915	27106	*arrestin domain containing 2*	*ARRDC2*	8.38	8.56	7.50	0.002126589	0.010019803
8159734	774	*calcium channel, voltage-dependent, N type, alpha 1B subunit*	*CACNA1B*	7.02	7.23	7.89	0.002129455	0.010028497
7906355	913	*CD1e molecule*	*CD1E*	6.17	6.24	5.79	0.002130406	0.010028497
8054862	79134	*transmembrane protein 185B (pseudogene)*	*TMEM185 B*	9.98	10.13	8.91	0.002129655	0.010028497
7951157	79780	*coiled-coil domain containing 82*	*CCDC82*	6.85	6.76	7.39	0.002130073	0.010028497
7950197	116985	*ArfGAP with RhoGAP domain, ankyrin repeat and PH domain 1*	*ARAP1*	9.56	9.19	8.08	0.002130478	0.010028497
7968062	479	*ATPase, H+/K+ transporting, nongastric, alpha polypeptide*	*ATP12A*	5.74	5.87	6.12	0.002131803	0.0100331
8035449	25804		*LSM4*	10.04	10.01	11.44	0.002134104	0.01004229
7916356	51668	*heat shock protein family B (small), member 11*	*HSPB11*	7.43	7.28	8.04	0.002135343	0.01004648
8167965	4478	*moesin*	*MSN*	11.96	12.19	11.19	0.002135962	0.010047755
8116722	100129033	*QIQN5815*	*LOC10012 9033*	6.61	6.72	5.97	0.00213684	0.010050247
8033300	56927	*G protein-coupled receptor 108*	*GPR108*	10.81	10.86	9.60	0.002138445	0.010056159
8081111				5.11	5.01	4.79	0.002139001	0.010057136

8022277				7.10	7.17	6.55	0.002139902	0.010059737
8073546	339674		*LOC339674*	7.66	7.74	7.03	0.002141189	0.010064147
7905283	8416, 55793	*annexin A9 | family with sequence similarity 63, member A*	*ANXA9|FAM63A*	6.74	6.84	7.87	0.002143311	0.010072479
7910446	22796	*component of oligomeric golgi complex 2*	*COG2*	7.14	7.20	8.47	0.002143724	0.010072782
8179112	23	*ATP-binding cassette, sub-family F (GCN20), member 1*	*ABCF1*	9.32	9.56	10.39	0.00214471	0.010074137
7917649	7049	*transforming growth factor, beta receptor III*	*TGFBR3*	10.21	9.16	7.48	0.002144453	0.010074137
8156240	392360	*cathepsin L family member 3*	*CTSL3*	6.24	6.41	5.92	0.002146456	0.010080696
7926368	7431	*vimentin*	*VIM*	13.32	13.45	11.74	0.002148848	0.010089547
7962930	23109	*dendrin*	*DDN*	6.98	6.99	6.35	0.00214904	0.010089547
8035234				5.70	5.80	5.51	0.00215093	0.010096778
7912701	348487	*family with sequence similarity 131, member C*	*FAM131C*	8.26	8.34	7.39	0.00215383	0.010107106
8045279				6.94	7.01	6.26	0.002153585	0.010107106
7929779	1244	*ATP-binding cassette, sub-family C (CFTR/MRP), member 2*	*ABCC2*	6.31	6.36	6.08	0.002154832	0.010110165
8097120				5.04	5.14	4.68	0.002155407	0.01011122
7907568				6.26	6.25	5.89	0.002157608	0.010118254
8010768				9.03	8.93	8.39	0.002157438	0.010118254
8044882	57669	*erythrocyte membrane protein band 4.1 like 5*	*EPB41L5*	8.04	7.26	9.24	0.002158199	0.010119384
7920303	6284	*S100 calcium binding protein A13*	*S100A13*	8.18	8.23	7.02	0.002159698	0.010124765
8081676	29083	*GTP-binding protein 8 (putative)*	*GTPBP8*	7.86	7.69	8.93	0.002160811	0.010128343
8177797	23	*ATP-binding cassette, sub-family F (GCN20), member 1*	*ABCF1*	9.32	9.56	10.39	0.002161524	0.010128396
8003465				8.14	8.10	7.88	0.002161222	0.010128396
7973158	55701	*Rho guanine nucleotide exchange factor (GEF) 40*	*ARHGEF40*	8.59	8.82	7.31	0.00216228	0.010130293
8117929	23	*ATP-binding cassette, sub-family F (GCN20), member 1*	*ABCF1*	9.32	9.56	10.39	0.002163513	0.010132985
8081348	57092	*PEST proteolytic signal containing nuclear protein*	*PCNP*	10.45	10.58	11.36	0.002163557	0.010132985
8097687	5458	*POU class 4 homeobox 2*	*POU4F2*	6.87	6.95	6.31	0.002164174	0.010134231

7915919	6886	*T-cell acute lymphocytic leukemia 1*	*TAL1*	6.60	6.65	6.00	0.002165371	0.010137055
7955170	7471	*wingless-type MMTV integration site family, member 1*	*WNT1*	8.10	8.19	7.48	0.00216583	0.010137055
7919564				9.83	9.87	8.99	0.002165568	0.010137055
8117696	1353		*COX11*	6.50	6.70	8.09	0.002166476	0.010138433
8060528	128653	*chromosome 20 open reading frame 141*	*C20orf141*	7.04	7.08	6.25	0.002167798	0.010142977
7970577	4285	*mitochondrial intermediate peptidase*	*MIPEP*	7.10	7.16	7.91	0.002170433	0.010153659
8039316	54776	*protein phosphatase 1, regulatory (inhibitor) subunit 12C*	*PPP1R12C*	9.42	9.37	8.43	0.002171089	0.01015508
7979916	55333	*synaptojanin 2 binding protein*	*SYNJ2BP*	8.96	8.89	9.88	0.002172397	0.010159553
8014865	4761	*neurogenic differentiation 2*	*NEUROD2*	7.16	7.30	6.74	0.002173747	0.010162775
8109802	5917	*arginyl-tRNA synthetase*	*RARS*	9.46	9.23	10.56	0.002174707	0.010162775
8070097	54943	*DnaJ (Hsp40) homolog, subfamily C, member 28*	*DNAJC28*	6.13	6.11	7.03	0.002175198	0.010162775
7971866	81624	*diaphanous homolog 3 (Drosophila)*	*DIAPH3*	8.50	7.29	9.27	0.002174876	0.010162775
8062480	128439, 677821, 677837	*small nucleolar RNA host gene 11 (non-protein coding) | small nucleolar RNA, H/ACA box 39 | small nucleolar RNA, H/ACA box 60*	*SNHG11|SNORA39|SNORA60*	7.80	7.95	7.16	0.002174352	0.010162775
8036025				4.90	4.92	4.79	0.00217486	0.010162775
7931393	10539	*glutaredoxin 3*	*GLRX3*	8.04	8.36	9.06	0.002176814	0.01016868
8016858	8161	*coilin*	*COIL*	8.90	8.94	10.62	0.002177262	0.01016913
7996569				6.78	7.04	8.14	0.00217773	0.010169665
7999936	7369	*uromodulin*	*UMOD*	6.80	6.84	6.31	0.002179784	0.010177614
7988177	161497	*stereocilin*	*STRC*	6.66	6.74	6.24	0.002181537	0.010184149
8041383	4052	*latent transforming growth factor beta binding protein 1*	*LTBP1*	11.34	9.89	8.85	0.002183002	0.010189341
8149652	55806	*hairless homolog (mouse)*	*HR*	9.01	8.72	7.37	0.002183539	0.010190201
8001185	10294	*DnaJ (Hsp40) homolog, subfamily A, member 2*	*DNAJA2*	8.18	8.36	9.72	0.002184154	0.010191422
8171311				5.60	5.92	5.03	0.0021858	0.010197456
7903893	963		*CD53*	6.06	6.00	6.30	0.002186184	0.010197599
8114213				5.12	5.12	4.71	0.002187097	0.010200213

8012054	1742	*discs, large homolog 4 (Drosophila)*	*DLG4*	9.36	9.31	8.33	0.002188743	0.010205714
8064418	27111	*syndecan binding protein (syntenin) 2*	*SDCBP2*	7.54	7.66	6.99	0.002189338	0.010205714
8149820	137814	*NK2 transcription factor related, locus 6 (Drosophila)*	*NKX2-6*	7.10	8.60	6.70	0.002189246	0.010205714
8037071	10567	*Rab acceptor 1 (prenylated)*	*RABAC1*	11.24	11.42	9.92	0.002190626	0.010209568
8083757	51068		*NMD3*	8.87	8.81	9.44	0.002191225	0.010209568
8151401	55284	*ubiquitin-conjugating enzyme E2W (putative)*	*UBE2W*	9.06	9.09	10.13	0.002191044	0.010209568
8170362				5.55	6.03	6.91	0.002194292	0.010222209
8152668	29028	*ATPase family, AAA domain containing 2*	*ATAD2*	7.07	7.15	8.59	0.00219562	0.010226742
8015759	10493	*vesicle amine transport protein 1 homolog (T. californica)*	*VAT1*	12.13	12.07	11.03	0.002197278	0.010232814
7937263	113746	*outer dense fiber of sperm tails 3*	*ODF3*	6.69	6.71	6.00	0.002199055	0.010239436
8039937	80772	*glycolipid transfer protein domain containing 1*	*GLTPD1*	8.23	8.31	6.53	0.00219978	0.010241161
8104163	389257	*leucine rich repeat containing 14B*	*LRRC14B*	6.85	7.06	6.54	0.002200478	0.010242758
8122174				8.53	8.54	7.65	0.002201256	0.010244729
8013509	256302	*chromosome 17 open reading frame 103*	*C17orf103*	8.62	8.59	7.40	0.002202532	0.010249018
7932041	26019		*UPF2*	7.21	6.96	9.18	0.00220437	0.010255546
8010583	388428		*LOC388428*	7.66	7.70	7.04	0.002204646	0.010255546
8076331	10766	*transducer of ERBB2, 2*	*TOB2*	10.52	10.19	9.32	0.002206312	0.010261645
8085815	7155	*topoisomerase (DNA) II beta 180kDa*	*TOP2B*	8.06	7.21	10.13	0.002208271	0.010269102
8052125	51130, 100302652, 10936	*ankyrin repeat and SOCS box-containing 3 | GPR75-ASB3 | G protein-coupled receptor 75*	*ASB3|LOC100302652|GPR75*	9.05	8.87	10.48	0.002211143	0.010280801
8041636				4.91	4.74	6.40	0.00221345	0.010289869
7942839	51585		*PCF11*	8.06	7.58	9.58	0.00221539	0.010291115
7951781	54494	*chromosome 11 open reading frame 71*	*C11orf71*	7.76	7.47	8.29	0.002214375	0.010291115
8094876	60558		*GUF1*	7.51	7.44	9.66	0.002214491	0.010291115
7923037				6.16	6.33	5.80	0.002215376	0.010291115
8100782				9.91	10.01	9.47	0.002215501	0.010291115
8116658	26240	*family with sequence similarity 50, member B*	*FAM50B*	8.55	8.40	7.42	0.002216641	0.010294758

8166289	6792	*cyclin-dependent kinase-like 5*	*CDKL5*	7.12	6.64	9.46	0.002217714	0.010298085
8102440	79642	*arylsulfatase family, member J*	*ARSJ*	8.21	8.24	6.33	0.002218279	0.010299052
7906597	4807	*nescient helix loop helix 1*	*NHLH1*	6.64	6.74	6.32	0.002219722	0.010304092
8025328	115704	*ecotropic viral integration site 5-like*	*EVI5L*	8.80	8.69	7.40	0.00222228	0.01031419
8168375	63947, 728656, 653687	*DMRT-like family C1 | DMRT-like family C1B | non-protein coding RNA 246B*	*DMRTC1| DMRTC1B |NCRNA00 246B*	7.01	6.86	6.43	0.002222612	0.01031419
8055279				6.19	6.11	5.39	0.002223553	0.010316902
7933945				8.45	8.60	7.81	0.002224255	0.010318498
8035494	23770	*FK506 binding protein 8, 38kDa*	*FKBP8*	11.74	11.71	10.63	0.002224884	0.010319759
8114468	26785	*small nucleolar RNA, C/D box 63*	*SNORD63*	5.42	5.83	5.83	0.002226759	0.010326278
8091342	84107	*Zic family member 4*	*ZIC4*	6.08	6.18	5.62	0.002227005	0.010326278
8163485	138065	*ring finger protein 183*	*RNF183*	6.69	6.84	6.44	0.002228974	0.010327115
7900540	284716	*ribosomal modification protein rimK-like family member A*	*RIMKLA*	7.30	7.45	8.31	0.002228337	0.010327115
8168412	554203	*non-protein coding RNA 183*	*NCRNA00 183*	5.88	6.32	7.43	0.002228803	0.010327115
8146717	23678, 56260	*serum/glucocorticoid regulated kinase family, member 3 | chromosome 8 open reading frame 44*	*SGK3|C8o rf44*	7.01	6.75	9.09	0.002228293	0.010327115
8066292				5.34	5.36	5.14	0.002227687	0.010327115
7964303	6866	*tachykinin 3*	*TAC3*	6.16	6.20	7.12	0.002230231	0.010331282
7989307				4.99	5.03	5.78	0.002231566	0.010335804
7977773				6.48	6.53	6.18	0.002232964	0.010340624
8167476	89801	*protein phosphatase 1, regulatory (inhibitor) subunit 3F*	*PPP1R3F*	7.71	7.84	7.16	0.002233902	0.010343305
8148888	84988	*protein phosphatase 1, regulatory (inhibitor) subunit 16A*	*PPP1R16A*	8.60	8.64	7.77	0.002235019	0.010346821
8171148	414	*arylsulfatase D*	*ARSD*	9.03	9.04	7.51	0.00223576	0.01034859
8109226	1044	*caudal type homeobox 1*	*CDX1*	8.06	8.05	7.31	0.00223691	0.010352252
8167784				5.36	5.43	5.08	0.002241086	0.010369919
7974387	6815	*serine/threonine/tyrosine interacting protein*	*STYX*	8.95	8.68	10.83	0.002242209	0.01037345

8039006	162967	*zinc finger protein 320*	*ZNF320*	6.59	6.62	8.58	0.002243488	0.010377705
7948424	54948	*mitochondrial ribosomal protein L16*	*MRPL16*	8.94	8.94	10.10	0.002249042	0.010398927
7899310	84065	*transmembrane protein 222*	*TMEM222*	9.89	10.12	8.26	0.002248712	0.010398927
7956856	253827	*methionine sulfoxide reductase B3*	*MSRB3*	8.77	8.78	6.24	0.002249157	0.010398927
8044640	5239, 595135	*phosphoglucomutase 5 | phosphoglucomutase 5 pseudogene 2*	*PGM5|PGM5P2*	7.98	7.94	6.97	0.002253105	0.010415514
8137474	57180	*ARP3 actin-related protein 3 homolog B (yeast)*	*ACTR3B*	7.35	7.65	8.60	0.002254065	0.010418283
8055309				7.70	7.64	7.46	0.002255302	0.01042233
8092162	100128046	*hypothetical LOC100128046*	*PP13439*	6.56	6.43	5.93	0.002255784	0.010422892
8006655	79154	*dehydrogenase/reductase (SDR family) member 11*	*DHRS11*	8.21	8.08	9.81	0.002256701	0.01042546
7998427	25823, 8912	*tryptase gamma 1 | calcium channel, voltage-dependent, T type, alpha 1H subunit*	*TPSG1|CACNA1H*	7.38	7.33	6.78	0.002260112	0.010439548
7960253	4815	*ninjurin 2*	*NINJ2*	7.96	8.09	7.06	0.002263252	0.010451182
7914516	339483	*myotubularin related protein 9-like, pseudogene*	*MTMR9LP*	8.72	8.28	8.00	0.002263355	0.010451182
8035829	6164	*ribosomal protein L34*	*RPL34*	12.78	12.86	13.22	0.002269241	0.010476687
8074931	7621	*zinc finger protein 70*	*ZNF70*	8.80	8.67	7.43	0.002274973	0.010501366
7917470				6.41	6.55	6.10	0.002275649	0.010501366
8044764				6.17	6.46	5.92	0.002275678	0.010501366
7933469	58504	*Rho GTPase activating protein 22*	*ARHGAP22*	7.73	7.94	7.11	0.00227625	0.010502328
7994308	23247		*KIAA0556*	8.80	8.62	8.02	0.002276886	0.010503585
7912928	6390	*succinate dehydrogenase complex, subunit B, iron sulfur (Ip)*	*SDHB*	10.48	10.70	11.00	0.002278166	0.010507809
8146393	492307	*chromosome 8 open reading frame 22*	*C8orf22*	5.28	5.21	5.07	0.002279938	0.010514299
8074712	25812, 29797, 646074, 29774	*POM121 membrane glycoprotein-like 1, pseudogene | POM121 membrane glycoprotein-like 8 pseudogene | POM121 membrane glycoprotein-like 10, pseudogene | POM121 membrane glycoprotein-like 9, pseudogene*	*POM121L1P|POM121L8P|POM121L10P|POM121L9P*	7.43	7.49	6.91	0.002284086	0.010531749
8036890	57716	*periaxin*	*PRX*	7.50	7.62	7.06	0.002286719	0.010542203

8155699	2395	*frataxin*	*FXN*	9.36	9.31	10.46	0.002288799	0.010547945
8133335	8326	*frizzled homolog 9 (Drosophila)*	*FZD9*	8.40	8.38	7.84	0.002289791	0.010547945
8078155	117248	*UDP-N-acetyl-alpha-D-galactosamine:polypeptide N-acetylgalactosaminyltransferase-like 2*	*GALNTL2*	6.29	8.25	5.76	0.002289786	0.010547945
8099924				9.57	9.64	9.02	0.002289564	0.010547945
8136078				5.75	4.96	6.96	0.002289626	0.010547945
8174340	55285	*RNA binding motif protein 41*	*RBM41*	6.24	5.98	7.47	0.002291048	0.010551685
8076732				8.47	8.64	8.09	0.002291334	0.010551685
8043413	22934	*ribose 5-phosphate isomerase A*	*RPIA*	6.91	7.04	7.63	0.002293198	0.010558587
8042356	4211	*Meis homeobox 1*	*MEIS1*	7.48	8.43	6.14	0.002295097	0.010562276
8035318	23025	*unc-13 homolog A (C. elegans)*	*UNC13A*	6.25	6.48	7.76	0.002294534	0.010562276
8081055	25978	*chromatin modifying protein 2B*	*CHMP2B*	7.63	8.11	9.42	0.002295018	0.010562276
7907904	51278	*immediate early response 5*	*IER5*	9.09	9.21	8.25	0.002296802	0.010568436
8147057	92421	*chromatin modifying protein 4C*	*CHMP4C*	6.14	6.20	7.02	0.002298825	0.010576059
8038890	3036	*hyaluronan synthase 1*	*HAS1*	7.86	7.93	7.47	0.002299393	0.010576989
7981233				7.36	7.40	6.63	0.002301632	0.010585601
8016402	10951	*chromobox homolog 1*	*CBX1*	8.98	9.24	11.01	0.00230228	0.010586041
8112896	340120	*ankyrin repeat domain 34B*	*ANKRD34 B*	4.84	4.75	5.51	0.002302657	0.010586041
8080964	727936	*glucoside xylosyltransferase 2*	*GXYLT2*	10.18	9.94	7.56	0.002302828	0.010586041
8146482	96764, 137695	*trimethylguanosine synthase 1 | transmembrane protein 68*	*TGS1|TME M68*	7.54	7.52	9.70	0.002304191	0.010590619
8026989				7.16	7.37	6.41	0.002305601	0.010595415
8124716	10107	*tripartite motif-containing 10*	*TRIM10*	6.11	6.18	5.78	0.002306903	0.010598025
8087545				5.92	6.13	6.84	0.002306608	0.010598025
7944463	79671	*NLR family member X1*	*NLRX1*	8.80	8.65	7.70	0.002307989	0.010601327
7936891	399818	*methyltransferase like 10*	*METTL10*	7.93	7.96	8.81	0.002308391	0.010601487
7905507	353139	*late cornified envelope 2A*	*LCE2A*	7.85	7.88	7.40	0.002311049	0.010610315
8040290				8.32	8.43	7.66	0.00231091	0.010610315
7992568	1877	*E4F transcription factor 1*	*E4F1*	8.23	8.30	7.66	0.002311754	0.010611868

8140433	22932, 7784	*POM121 and ZP3 fusion | zona pellucida glycoprotein 3 (sperm receptor)*	*POMZP3*	8.13	8.10	7.30	0.002312494	0.010613575
7913805	864	*runt-related transcription factor 3*	*RUNX3*	8.88	9.33	7.52	0.002315638	0.010616832
8090546	7555	*CCHC-type zinc finger, nucleic acid binding protein*	*CNBP*	9.43	9.49	11.03	0.00231572	0.010616832
8093500	10460	*transforming, acidic coiled-coil containing protein 3*	*TACC3*	7.49	7.93	9.54	0.002314365	0.010616832
7949490	91056		*DKFZp761 E198*	8.18	8.11	7.22	0.002315778	0.010616832
7896865	254173	*tubulin tyrosine ligase-like family, member 10*	*TTLL10*	8.16	8.35	7.63	0.002315056	0.010616832
7993825	440352, 84127, 400509	*RUN domain containing 2C | RUN domain containing 2A | RUN domain containing 2B*	*RUNDC2C |RUNDC2 A|RUNDC 2B*	8.71	8.44	7.09	0.002313993	0.010616832
8115166				5.98	6.19	5.68	0.002314533	0.010616832
7957560	8738	*CASP2 and RIPK1 domain containing adaptor with death domain*	*CRADD*	8.16	8.10	7.62	0.002316812	0.010619884
8032699	23217	*zinc finger RNA binding protein 2*	*ZFR2*	7.36	7.40	6.74	0.002319183	0.010625691
7954985	84216	*transmembrane protein 117*	*TMEM117*	8.61	8.40	6.85	0.002318821	0.010625691
8171403	140456	*ankyrin repeat and SOCS box-containing 11*	*ASB11*	5.28	5.35	5.13	0.002318831	0.010625691
8050336	1876	*E2F transcription factor 6*	*E2F6*	7.29	7.53	8.69	0.002320373	0.010629457
8010320	84733	*chromobox homolog 2*	*CBX2*	7.60	7.70	8.78	0.00232096	0.010630461
7952036	196264	*myelin protein zero-like 3*	*MPZL3*	5.86	6.08	6.87	0.002321469	0.010631104
8161884	158471	*prune homolog 2 (Drosophila)*	*PRUNE2*	5.88	6.49	7.63	0.00232245	0.010633911
8151587	401466	*chromosome 8 open reading frame 59*	*C8orf59*	7.48	7.53	8.25	0.002323207	0.010635689
7902367	34	*acyl-CoA dehydrogenase, C-4 to C-12 straight chain*	*ACADM*	8.97	8.79	10.52	0.002324063	0.010636237
8132617	83637	*zinc finger, MIZ-type containing 2*	*ZMIZ2*	9.10	9.18	8.26	0.002323698	0.010636237
8000192	730092, 653390, 100131998, 54700	*RNA polymerase I transcription factor homolog (S. cerevisiae) pseudogene 1 | RNA polymerase I transcription factor homolog (S. cerevisiae) pseudogene 2 | RNA polymerase I transcription factor homolog (S. cerevisiae) pseudogene 3 | RRN3 RNA polymerase I transcription factor homolog (S. cerevisiae)*	*RRN3P1|R RN3P2|RR N3P3*	9.18	9.13	10.37	0.002324461	0.01063637

8126486	9820	*cullin 7*	*CUL7*	9.08	9.43	7.60	0.002325256	0.010638325
7927658	7321	*ubiquitin-conjugating enzyme E2D 1 (UBC4/5 homolog, yeast)*	*UBE2D1*	8.50	8.89	9.95	0.00232636	0.010641689
8103094	4306	*nuclear receptor subfamily 3, group C, member 2*	*NR3C2*	6.08	6.18	6.82	0.002327003	0.010642773
7897960	126767	*arylacetamide deacetylase-like 3*	*AADACL3*	5.90	6.06	7.10	0.002327335	0.010642773
8126387	2979	*guanylate cyclase activator 1B (retina)*	*GUCA1B*	6.36	6.49	6.06	0.002327836	0.010643378
7905349	79626	*tumor necrosis factor, alpha-induced protein 8-like 2*	*TNFAIP8L 2*	6.60	6.70	6.19	0.002329415	0.010647226
7985037				5.43	5.45	5.09	0.00232933	0.010647226
7946041	3045	*hemoglobin, delta*	*HBD*	6.32	6.34	5.70	0.002331132	0.010650651
8046536	3236	*homeobox D10*	*HOXD10*	6.12	6.24	5.96	0.002332151	0.010650651
8175457	4168	*MCF.2 cell line derived transforming sequence*	*MCF2*	5.24	5.32	5.78	0.002332025	0.010650651
7967325	27198	*G protein-coupled receptor 81*	*GPR81*	5.76	5.31	5.26	0.002330698	0.010650651
8145989	203100	*HtrA serine peptidase 4*	*HTRA4*	6.94	7.15	6.43	0.002332378	0.010650651
8066161	140699, 6185	*chromosome 20 open reading frame 132 | ribophorin II*	*C20orf132| RPN2*	6.89	6.65	6.08	0.002332195	0.010650651
7916024	22996	*tetratricopeptide repeat domain 39A*	*TTC39A*	6.40	6.45	6.93	0.002333292	0.010653142
7969828				6.84	6.81	6.54	0.002334657	0.010657687
8174304	6906	*serpin peptidase inhibitor, clade A (alpha-1 antiproteinase, antitrypsin), member 7*	*SERPINA7*	4.94	5.02	4.84	0.002336135	0.010662749
8048319	7429	*villin 1*	*VIL1*	6.45	6.55	7.03	0.002337186	0.010665857
8099008	57732	*zinc finger, FYVE domain containing 28*	*ZFYVE28*	7.11	7.36	6.51	0.002338245	0.010667318
7995976	221184	*copine II*	*CPNE2*	10.79	10.76	8.91	0.002338113	0.010667318
8137183	23145	*SCO-spondin homolog (Bos taurus)*	*SSPO*	7.88	7.89	7.10	0.002340447	0.010675675
7965231	25834	*mannosyl (alpha-1,3-)-glycoprotein beta-1,4-N-acetylglucosaminyltransferase, isozyme C (putative)*	*MGAT4C*	5.06	5.03	5.48	0.002342133	0.010679995
8170402	51402, 100130086	*heat shock transcription factor family, X linked 1 | heat shock transcription factor family, X linked 2*	*HSFX1|HS FX2*	7.02	6.91	6.43	0.002341808	0.010679995
7984846	1198, 646748	*CDC-like kinase 3 | hypothetical protein LOC646748*	*CLK3|FLJ 42022*	8.57	8.64	7.64	0.002342845	0.010681555
7994620	3835	*kinesin family member 22*	*KIF22*	8.13	8.58	10.62	0.002343755	0.010684016
8177704	442194	*olfactory receptor, family 10, subfamily C, member 1*	*OR10C1*	6.13	6.20	5.48	0.002345302	0.010689377

8071861	56241	*sushi domain containing 2*	*SUSD2*	8.73	7.47	7.12	0.002347366	0.010697097
8103372				5.38	5.31	4.83	0.002348067	0.010698603
8078412	25904	*CCR4-NOT transcription complex, subunit 10*	*CNOT10*	7.42	7.48	9.00	0.002350112	0.010706232
8164398	2801	*golgin A2*	*GOLGA2*	8.30	8.45	7.55	0.00235049	0.010706264
8066905	57169	*zinc finger, NFX1-type containing 1*	*ZNFX1*	10.07	10.16	8.17	0.002353936	0.010720267
7944035	345	*apolipoprotein C-III*	*APOC3*	6.89	7.00	6.48	0.002356063	0.010726568
8166899	60506	*nyctalopin*	*NYX*	7.78	7.82	7.01	0.00235584	0.010726568
8114550	9542	*neuregulin 2*	*NRG2*	8.14	8.02	7.48	0.002357815	0.010732853
7969263	144983	*heterogeneous nuclear ribonucleoprotein A1-like 2*	*HNRNPA1 L2*	6.24	6.26	7.77	0.002362019	0.010750294
8165183	9919	*SEC16 homolog A (S. cerevisiae)*	*SEC16A*	9.70	9.64	8.08	0.002364049	0.010757114
7997569	161931	*adenosine deaminase domain containing 2*	*ADAD2*	8.29	8.46	7.79	0.002364262	0.010757114
7953150	7004	*TEA domain family member 4*	*TEAD4*	8.68	8.66	9.79	0.002365937	0.010761491
7969677	10150	*muscleblind-like 2 (Drosophila)*	*MBNL2*	10.66	10.39	8.22	0.00236597	0.010761491
8174654	90293	*kelch-like 13 (Drosophila)*	*KLHL13*	6.89	5.43	7.32	0.002367119	0.01076502
8086077	23122	*cytoplasmic linker associated protein 2*	*CLASP2*	7.75	7.65	9.85	0.002372315	0.010786953
7899795				7.90	8.06	7.10	0.002376942	0.01080629
7972923	496	*ATPase, H+/K+ exchanging, beta polypeptide*	*ATP4B*	6.92	6.95	6.31	0.002381355	0.010820102
7970763	2321	*fms-related tyrosine kinase 1 (vascular endothelial growth factor/vascular permeability factor receptor)*	*FLT1*	8.33	6.22	9.39	0.002380986	0.010820102
7902205	3595	*interleukin 12 receptor, beta 2*	*IL12RB2*	5.22	5.24	5.64	0.002381479	0.010820102
7953949	3824	*killer cell lectin-like receptor subfamily D, member 1*	*KLRD1*	4.99	4.96	4.78	0.002380915	0.010820102
8112731	2151	*coagulation factor II (thrombin) receptor-like 2*	*F2RL2*	10.44	6.60	6.74	0.002383835	0.010825693
7948599	26070	*hypothetical LOC26070*	*DKFZP43 4K028*	7.11	6.92	6.52	0.002383726	0.010825693
8107859	90624	*Lyrm7 homolog (mouse)*	*LYRM7*	5.82	5.85	7.20	0.002383668	0.010825693
8148597	286128	*zinc finger protein 41 homolog (mouse)*	*ZFP41*	7.89	7.82	7.03	0.002384557	0.010827269
8163491	114987	*WD repeat domain 31*	*WDR31*	7.03	6.94	6.12	0.002385574	0.010830185
8067812	198437	*chromosome 20 open reading frame 201*	*C20orf201*	8.03	7.95	7.21	0.002386539	0.01083286
8036318	84924	*zinc finger protein 566*	*ZNF566*	6.57	6.51	8.29	0.002387878	0.010837234

7979824	87	*actinin, alpha 1*	*ACTN1*	12.69	12.99	11.92	0.002389758	0.01084236
7949668	55231	*coiled-coil domain containing 87*	*CCDC87*	7.04	7.02	6.50	0.002389616	0.01084236
7961514	4256	*matrix Gla protein*	*MGP*	8.66	6.71	5.20	0.002393366	0.010857022
8061324	22803	*5'-3' exoribonuclease 2*	*XRN2*	8.12	8.12	10.19	0.002395928	0.010866937
8117178	140767	*neurensin 1*	*NRSN1*	6.33	6.42	6.18	0.002397215	0.010871064
7990916	6218	*ribosomal protein S17*	*RPS17*	13.00	13.05	13.19	0.002398163	0.010873657
7960865	6515	*solute carrier family 2 (facilitated glucose transporter), member 3*	*SLC2A3*	10.14	10.60	12.83	0.002399054	0.010875987
7992732	124220	*zymogen granule protein 16 homolog B (rat)*	*ZG16B*	6.98	7.13	6.46	0.002399523	0.010876407
8141708	24146	*claudin 15*	*CLDN15*	8.55	8.73	7.64	0.002401088	0.01088179
7977987	4624	*myosin, heavy chain 6, cardiac muscle, alpha*	*MYH6*	6.50	6.47	5.96	0.00240571	0.010897649
7927474	118461	*chromosome 10 open reading frame 71*	*C10orf71*	6.37	6.51	6.08	0.00240572	0.010897649
8137802	401296		*LOC401296*	7.04	7.13	6.54	0.00240518	0.010897649
8111913				6.37	6.36	5.79	0.002406221	0.010898209
8096251	53343	*nudix (nucleoside diphosphate linked moiety X)-type motif 9*	*NUDT9*	7.26	7.24	8.09	0.002407068	0.010900334
8019083	440465	*hypothetical LOC440465*	*FLJ90757*	8.09	7.98	7.33	0.002407808	0.010901977
7901309	2301	*forkhead box E3*	*FOXE3*	8.99	8.99	8.30	0.002409238	0.010903993
8167334	7454	*Wiskott-Aldrich syndrome (eczema-thrombocytopenia)*	*WAS*	7.21	7.24	6.82	0.002409764	0.010903993
8134318	64921	*CAS1 domain containing 1*	*CASD1*	8.11	8.16	9.72	0.00240951	0.010903993
7943126	85459, 79101	*KIAA1731 | TATA box binding protein (TBP)-associated factor, RNA polymerase I, D, 41kDa*	*KIAA1731| TAF1D*	6.58	6.47	8.28	0.002409259	0.010903993
7913727	127294	*myomesin family, member 3*	*MYOM3*	6.08	6.16	5.79	0.002410454	0.010905404
7934156	4838	*nodal homolog (mouse)*	*NODAL*	6.18	6.22	7.20	0.002410881	0.010905629
8117128	1871	*E2F transcription factor 3*	*E2F3*	7.04	7.43	8.30	0.002412588	0.010911639
7930205	729020	*rcRPE*	*LOC729020*	6.29	6.45	7.33	0.002414224	0.010917328
8042720	10574	*chaperonin containing TCP1, subunit 7 (eta)*	*CCT7*	10.70	10.88	11.92	0.002419198	0.01093811
8077958	79885	*histone deacetylase 11*	*HDAC11*	8.71	8.51	7.47	0.002422703	0.01095224

8037045	1089	*carcinoembryonic antigen-related cell adhesion molecule 4*	*CEACAM4*	6.98	7.00	6.34	0.002423772	0.010953677
8058516				7.31	7.22	6.64	0.002423779	0.010953677
8173261	55906	*zinc finger, C4H2 domain containing*	*ZC4H2*	8.84	8.51	9.81	0.002424945	0.01095723
7932014	399717	*hypothetical LOC399717*	*FLJ45983*	6.37	6.58	5.97	0.002430783	0.010980334
8039010	91661, 126017, 55762, 7576, 170958	*zinc finger protein 765 | zinc finger protein 813 | zinc finger protein 701 | zinc finger protein 28 | zinc finger protein 525*	*ZNF765|ZNF813|ZNF701|ZNF28|ZNF525*	10.33	10.15	11.92	0.002430819	0.010980334
8037679	1762	*dystrophia myotonica, WD repeat containing*	*DMWD*	9.61	9.69	8.41	0.002431733	0.010982472
8037579	2067	*excision repair cross-complementing rodent repair deficiency, complementation group 1 (includes overlapping antisense sequence)*	*ERCC1*	9.74	9.77	8.59	0.002432433	0.010982472
7948293	219541	*mediator complex subunit 19*	*MED19*	7.33	7.37	6.83	0.002432211	0.010982472
8073573	150368	*family with sequence similarity 109, member B*	*FAM109B*	9.84	9.77	8.28	0.002433584	0.010985948
8150872	6224	*ribosomal protein S20*	*RPS20*	12.83	12.91	13.14	0.002435083	0.010989961
8125007	80741	*lymphocyte antigen 6 complex, locus G5C*	*LY6G5C*	7.76	7.23	6.62	0.002435234	0.010989961
8009301	5578	*protein kinase C, alpha*	*PRKCA*	10.58	10.22	8.22	0.002436657	0.010993443
8140028	135892	*tripartite motif-containing 50*	*TRIM50*	6.72	7.09	6.39	0.002436767	0.010993443
8087739	1154	*cytokine inducible SH2-containing protein*	*CISH*	7.81	7.68	7.11	0.002437793	0.010996265
7905826	1163	*CDC28 protein kinase regulatory subunit 1B*	*CKS1B*	8.01	8.24	8.99	0.002438155	0.010996265
8115410	25929	*gem (nuclear organelle) associated protein 5*	*GEMIN5*	8.26	8.35	10.16	0.002438801	0.010997463
8074591	85376, 440804, 150221	*RIMS binding protein 3 | RIMS binding protein 3B | RIMS binding protein 3C*	*RIMBP3|RIMBP3B|RIMBP3C*	6.55	6.47	5.89	0.002441139	0.011006284
8001576	92922	*coiled-coil domain containing 102A*	*CCDC102A*	8.44	8.25	7.47	0.002443417	0.011014835
8075981	84645	*chromosome 22 open reading frame 23*	*C22orf23*	6.68	6.77	6.19	0.002445443	0.011018808
8021546	54877, 400653	*zinc finger, CCHC domain containing 2 | chromosome 18 open reading frame 49*	*ZCCHC2|C18orf49*	6.72	6.71	6.99	0.00244478	0.011018808
8056728				8.54	8.77	9.70	0.002445164	0.011018808

8141803	10156, 401331, 100271927	*RAS p21 protein activator 4 | RAS p21 protein activator 4 pseudogene | RAS p21 protein activator 4B*	*RASA4|RASA4P|RASA4B*	8.72	9.17	7.37	0.002447837	0.011027872
7974054	8487	*survival of motor neuron protein interacting protein 1*	*SIP1*	5.89	6.04	7.22	0.00245007	0.011031451
8156022	642947		*LOC642947*	6.13	5.79	7.44	0.00245016	0.011031451
7985364	3603, 80765	*interleukin 16 (lymphocyte chemoattractant factor) | StAR-related lipid transfer (START) domain containing 5*	*IL16|STARD5*	7.39	7.28	6.56	0.002449668	0.011031451
7969143				7.47	7.48	7.03	0.00245008	0.011031451
8156278	1903, 286223	*sphingosine-1-phosphate receptor 3 | chromosome 9 open reading frame 47*	*S1PR3|C9orf47*	8.92	7.80	7.47	0.002450926	0.01103146
7958759				7.09	6.92	5.95	0.002450572	0.01103146
8044375	10018	*BCL2-like 11 (apoptosis facilitator)*	*BCL2L11*	7.16	6.53	8.44	0.00245219	0.011031989
8119357	23500	*dishevelled associated activator of morphogenesis 2*	*DAAM2*	8.61	8.76	6.44	0.002451817	0.011031989
7904480				5.46	5.34	6.70	0.002451628	0.011031989
7936419	55088, 100302224	*chromosome 10 open reading frame 118 | microRNA 2110*	*C10orf118|MIR2110*	6.73	6.55	9.05	0.002452797	0.011033001
8125220	1797	*dom-3 homolog Z (C. elegans)*	*DOM3Z*	9.33	9.31	8.07	0.002456057	0.011045527
7993774				4.75	4.68	5.58	0.002456347	0.011045527
7972745	8660	*insulin receptor substrate 2*	*IRS2*	9.16	9.37	8.14	0.002456946	0.011045885
7947165	159963	*solute carrier family 5 (sodium/glucose cotransporter), member 12*	*SLC5A12*	6.24	6.48	6.98	0.002457192	0.011045885
7991779	83986	*integrin alpha FG-GAP repeat containing 3*	*ITFG3*	10.81	10.69	9.76	0.002458609	0.011050536
8019347				6.82	6.83	6.28	0.002462213	0.01106501
7922550	89866, 730102	*SEC16 homolog B (S. cerevisiae) | quinone oxidoreductase-like protein 2 pseudogene*	*SEC16B|LOC730102*	6.63	6.63	5.98	0.002463174	0.011066493
7997489				13.77	13.78	13.57	0.00246331	0.011066493
8048014	6120	*ribulose-5-phosphate-3-epimerase*	*RPE*	7.59	7.41	9.29	0.002464153	0.011068558
8045381	905	*cyclin T2*	*CCNT2*	8.56	8.44	10.12	0.00246541	0.011069939
7945462	3665	*interferon regulatory factor 7*	*IRF7*	8.37	8.43	7.64	0.00246561	0.011069939
8078544	4292	*mutL homolog 1, colon cancer, nonpolyposis type 2 (E. coli)*	*MLH1*	8.72	8.96	10.60	0.002465144	0.011069939

7914182				5.06	5.08	4.81	0.002466088	0.01107036
8108832	9812		*KIAA0141*	9.77	9.73	9.05	0.002466772	0.011071708
7971998				8.55	8.75	7.91	0.002469319	0.011081418
8123104	84624	*fibronectin type III domain containing 1*	*FNDC1*	8.66	11.07	6.18	0.00247068	0.011085575
7997226				5.68	5.38	5.24	0.002471013	0.011085575
8097749				5.11	5.10	4.82	0.002472668	0.011091276
8167314	4943	*TBC1 domain family, member 25*	*TBC1D25*	8.92	9.09	7.71	0.002473206	0.011091965
7950140	220077	*dedicator of cytokinesis 1 pseudogene*	*LOC220077*	7.74	8.00	7.20	0.002474331	0.011095287
7996034	6361	*chemokine (C-C motif) ligand 17*	*CCL17*	7.82	7.85	7.19	0.002475131	0.011097151
8161499	100131997, 100289124, 100287333	*family with sequence similarity 27, member E3 | family with sequence similarity 27, member E2 | family with sequence similarity 27, member E1*	*FAM27E3|FAM27E2|FAM27E1*	8.49	9.03	7.31	0.002475997	0.011099311
8175558	171489, 64648	*SPANX family, member E | SPANX family, member D*	*SPANXE|SPANXD*	6.72	6.84	6.02	0.002476809	0.011101225
8020551	3909	*laminin, alpha 3*	*LAMA3*	6.98	6.69	6.47	0.002478181	0.011105653
7915015	29889	*guanine nucleotide binding protein-like 2 (nucleolar)*	*GNL2*	8.54	8.40	10.72	0.002479937	0.011106749
8162216	53358	*SHC (Src homology 2 domain containing) transforming protein 3*	*SHC3*	9.64	7.71	7.97	0.002479965	0.011106749
8152976	137835	*transmembrane protein 71*	*TMEM71*	5.26	5.62	5.04	0.00247965	0.011106749
8073705	55615, 553158, 23779	*proline rich 5 (renal) | PRR5-ARHGAP8 readthrough | Rho GTPase activating protein 8*	*PRR5|PRR5-ARHGAP8*	9.60	9.35	8.67	0.002479685	0.011106749
8070933	10841	*formiminotransferase cyclodeaminase*	*FTCD*	7.91	7.95	7.42	0.002482431	0.01111607
8121704	116150, 11049	*nuclear undecaprenyl pyrophosphate synthase 1 homolog (S. cerevisiae) | nuclear undecaprenyl pyrophosphate synthase 1 homolog (S. cerevisiae) pseudogene 3*	*NUS1|NUS1P3*	10.11	10.20	11.67	0.002483447	0.011118896
8015460	47	*ATP citrate lyase*	*ACLY*	10.88	11.16	11.41	0.00248408	0.011120006
8050255	79954	*nucleolar protein 10*	*NOL10*	7.97	8.09	9.62	0.00248542	0.011124279
8112959				6.24	6.29	6.67	0.002486673	0.011128161
8068610				5.74	5.25	5.34	0.002487926	0.011128593

8094772				6.23	6.03	5.58	0.002487259	0.011128593
8161737				5.75	5.67	6.93	0.002487722	0.011128593
8044049	8807	*interleukin 18 receptor accessory protein*	*IL18RAP*	5.03	5.10	4.92	0.002488868	0.011131081
7923967	55432		*YOD1*	8.16	8.09	9.07	0.002491277	0.011140131
8143885	2636	*gastrulation brain homeobox 1*	*GBX1*	6.83	6.91	6.58	0.002492294	0.01114295
8114119	23105	*follistatin-like 4*	*FSTL4*	6.42	6.45	7.00	0.002493822	0.011148055
8108753	56121	*protocadherin beta 15*	*PCDHB15*	6.56	6.86	8.39	0.002495003	0.01115161
8090420	131601	*transmembrane protein, adipocyte asscociated 1*	*TPRA1*	11.00	10.79	9.83	0.002496799	0.011157909
8035842	7644	*zinc finger protein 91*	*ZNF91*	6.82	6.51	9.19	0.002499242	0.011163644
8019177	284186	*transmembrane protein 105*	*TMEM105*	7.32	7.51	6.81	0.002498952	0.011163644
7960338				8.78	8.49	7.62	0.002498699	0.011163644
7924230	81926, 100289252, 648359	*family with sequence similarity 108, member A1 | family with sequence similarity 108, member A11, pseudogene | family with sequence similarity 108, member A4*	*FAM108A1 |FAM108A 11P|FAM1 08A4*	10.32	10.31	9.07	0.00250018	0.011166105
8117861	3139	*major histocompatibility complex, class I, L, pseudogene*	*HLA-L*	7.82	7.70	7.11	0.002501337	0.011169542
8088718	9039	*ubiquitin-like modifier activating enzyme 3*	*UBA3*	9.36	9.04	10.46	0.002504259	0.011179332
8124574	9753	*zinc finger and SCAN domain containing 12*	*ZSCAN12*	6.16	6.07	7.23	0.002504543	0.011179332
8153273	389690		*FLJ43860*	6.68	6.65	6.21	0.002504691	0.011179332
8106818				6.77	6.66	7.68	0.002505408	0.011180805
8040292	9687	*growth regulation by estrogen in breast cancer 1*	*GREB1*	6.19	6.33	7.03	0.002506436	0.011181933
7901110	10327	*aldo-keto reductase family 1, member A1 (aldehyde reductase)*	*AKR1A1*	10.14	10.02	11.48	0.002506262	0.011181933
8049702	11132, 2859	*calpain 10 | G protein-coupled receptor 35*	*CAPN10|G PR35*	7.55	7.67	7.26	0.00250685	0.011182053
7975482	9628	*regulator of G-protein signaling 6*	*RGS6*	6.14	6.06	6.36	0.002508556	0.011187936
7966534	6128	*ribosomal protein L6*	*RPL6*	12.15	12.26	12.66	0.002509244	0.011189274
8059277				8.19	8.32	7.25	0.002509864	0.01119031
8011990	83659	*tektin 1*	*TEKT1*	6.39	6.53	5.84	0.00251194	0.011197837
7951420				5.97	5.76	5.53	0.00251261	0.011199094

8086842	4134	*microtubule-associated protein 4*	*MAP4*	10.00	9.92	9.29	0.002514063	0.01120384
8176644	9087	*thymosin beta 4, Y-linked*	*TMSB4Y*	6.95	7.03	7.23	0.002515578	0.011205712
8080847	57415	*chromosome 3 open reading frame 14*	*C3orf14*	7.23	7.59	8.49	0.002515647	0.011205712
8069800	643803	*keratin associated protein 24-1*	*KRTAP24-1*	6.24	6.29	5.57	0.002515167	0.011205712
8178699	1797	*dom-3 homolog Z (C. elegans)*	*DOM3Z*	9.33	9.31	8.07	0.002517138	0.01120751
8023259	26791	*small nucleolar RNA, C/D box 58A*	*SNORD58A*	7.03	7.08	8.62	0.002516935	0.01120751
8086498	57456		*KIAA1143*	8.65	8.72	9.97	0.002517215	0.01120751
8130013	7957	*epilepsy, progressive myoclonus type 2A, Lafora disease (laforin)*	*EPM2A*	6.65	6.75	7.28	0.002518492	0.011211464
7935746	282991	*biogenesis of lysosomal organelles complex-1, subunit 2*	*BLOC1S2*	7.99	8.45	9.40	0.002521867	0.011224755
8096845	1950	*epidermal growth factor*	*EGF*	5.71	5.68	6.67	0.002525004	0.011232161
8066513	6385	*syndecan 4*	*SDC4*	11.38	11.34	10.52	0.002524436	0.011232161
8084146	8087	*fragile X mental retardation, autosomal homolog 1*	*FXR1*	9.13	8.71	11.22	0.002524866	0.011232161
7987565	54866	*protein phosphatase 1, regulatory (inhibitor) subunit 14D*	*PPP1R14D*	6.33	6.42	5.92	0.002525087	0.011232161
7930025	83401	*elongation of very long chain fatty acids (FEN1/Elo2, SUR4/Elo3, yeast)-like 3*	*ELOVL3*	6.43	6.26	5.81	0.002525637	0.011232878
8048108				7.45	7.32	6.60	0.002527528	0.011239559
7911273	127074	*olfactory receptor, family 2, subfamily T, member 4*	*OR2T4*	5.79	5.50	5.12	0.002528766	0.011240042
8142878	246184	*cell division cycle 26 homolog (S. cerevisiae)*	*CDC26*	8.03	7.82	8.67	0.002528026	0.011240042
7998538	390667	*pentraxin 4, long*	*PTX4*	7.41	7.44	6.83	0.002528912	0.011240042
7969368				8.50	8.66	7.75	0.002529194	0.011240042
7902565	23266	*latrophilin 2*	*LPHN2*	9.79	7.62	10.96	0.002530059	0.011242155
7997746	57338	*junctophilin 3*	*JPH3*	6.67	6.50	7.19	0.002531254	0.011242677
7931469	170394	*PWWP domain containing 2B*	*PWWP2B*	7.69	7.88	7.01	0.002531345	0.011242677
8173166				6.39	5.71	5.36	0.002531106	0.011242677
7932985	8829	*neuropilin 1*	*NRP1*	10.32	10.25	7.85	0.002533641	0.011247815
8135323	60561	*RAD50 interactor 1*	*RINT1*	7.47	7.43	8.46	0.002533083	0.011247815

8027128	284439	*solute carrier family 25, member 42*	*SLC25A42*	9.22	9.07	8.10	0.002533671	0.011247815
8068664				5.90	5.91	5.62	0.002535802	0.011255544
8175252	406899	*microRNA 106a*	*MIR106A*	4.59	4.50	4.94	0.002536778	0.011256414
8113274				5.46	5.50	4.77	0.0025364	0.011256414
8058524	1420	*crystallin, gamma C*	*CRYGC*	6.95	7.05	6.47	0.00253804	0.011257671
7971134	80209	*chromosome 13 open reading frame 23*	*C13orf23*	9.76	9.68	10.99	0.002538231	0.011257671
8130142	84918	*low density lipoprotein receptor-related protein 11*	*LRP11*	8.12	8.04	7.43	0.002537715	0.011257671
8045795	3760	*potassium inwardly-rectifying channel, subfamily J, member 3*	*KCNJ3*	6.23	6.31	6.13	0.002541208	0.011262656
8032623	6915	*thromboxane A2 receptor*	*TBXA2R*	8.46	8.44	7.01	0.002541305	0.011262656
7992071	10232	*mesothelin*	*MSLN*	7.43	7.25	6.58	0.002540244	0.011262656
8074916	51233	*chromosome 22 open reading frame 43*	*C22orf43*	5.13	5.03	4.75	0.002540118	0.011262656
8053183	85474	*ladybird homeobox 2*	*LBX2*	7.84	7.96	7.03	0.002540954	0.011262656
8141374	563	*alpha-2-glycoprotein 1, zinc-binding*	*AZGP1*	5.37	5.54	5.22	0.002544534	0.011275235
7946635	58486	*zinc finger, BED-type containing 5*	*ZBED5*	8.25	7.99	9.14	0.002546103	0.01127865
8098916	92305	*transmembrane protein 129*	*TMEM129*	9.39	9.26	8.26	0.002546477	0.01127865
8133209	441251, 442590, 285955, 441273, 100310812	*speedy homolog E7 (Xenopus laevis), pseudogene | speedy homolog E5 (Xenopus laevis) | speedy homolog E1 (Xenopus laevis) | speedy homolog E2 (Xenopus laevis) | WBSCR19-like protein 3*	*SPDYE7P|SPDYE5|SPDYE1|SPDYE2|SPDYE2L*	6.90	6.87	6.45	0.002546344	0.01127865
8176972	3581	*interleukin 9 receptor*	*IL9R*	7.13	7.22	6.95	0.002548277	0.011279786
8169240	5631	*phosphoribosyl pyrophosphate synthetase 1*	*PRPS1*	9.45	10.43	10.92	0.002548296	0.011279786
7966600	80024	*solute carrier family 24 (sodium/potassium/calcium exchanger), member 6*	*SLC24A6*	9.35	9.08	7.72	0.002547517	0.011279786
7932860				8.15	8.16	7.21	0.00254767	0.011279786
8169028	27018	*nerve growth factor receptor (TNFRSF16) associated protein 1*	*NGFRAP1*	9.66	8.68	9.35	0.00255156	0.011292502
7967418				5.61	5.67	5.12	0.002552715	0.011295881
8120990				5.75	6.01	5.45	0.002554572	0.011302368

7985444	100133144, 388152, 80154, 727849, 100134869, 388165	*ubiquitin-conjugating enzyme E2Q family member 2 pseudogene 3 | hypothetical LOC388152 | hypothetical LOC80154 | golgin A2 pseudogene | ubiquitin-conjugating enzyme E2Q family member 2 pseudogene 2 | ubiquitin-conjugating enzyme E2Q family member 2 pseudogene 1*	*UBE2Q2P3|LOC388152|LOC80154|LOC727849|UBE2Q2P2|UBE2Q2P1*	6.63	6.84	7.13	0.002558209	0.011316723
8007058	147179	*WAS/WASL interacting protein family, member 2*	*WIPF2*	8.37	8.60	7.56	0.002559833	0.011322173
8030630	7376	*nuclear receptor subfamily 1, group H, member 2*	*NR1H2*	10.18	10.27	8.85	0.002561382	0.011327287
8097790				4.50	4.56	5.38	0.002562437	0.011330216
8062823	3172	*hepatocyte nuclear factor 4, alpha*	*HNF4A*	7.24	7.19	6.74	0.002564048	0.011332135
8153175	83696	*trafficking protein particle complex 9*	*TRAPPC9*	9.54	9.09	8.26	0.002563707	0.011332135
7950423				6.94	7.20	6.29	0.002563767	0.011332135
8155234	84186	*zinc finger, CCHC domain containing 7*	*ZCCHC7*	7.68	7.57	8.24	0.002566986	0.011341647
8116494	643836	*zinc finger protein 62 homolog (mouse)*	*ZFP62*	6.30	6.35	8.17	0.002566599	0.011341647
8146921	157506	*retinol dehydrogenase 10 (all-trans)*	*RDH10*	12.73	11.32	8.16	0.002569576	0.011351353
8133872				5.74	5.81	5.48	0.002572319	0.011361729
8179080	3139	*major histocompatibility complex, class I, L, pseudogene*	*HLA-L*	7.82	7.69	7.11	0.002573888	0.011365707
7938078	160298	*chromosome 11 open reading frame 42*	*C11orf42*	6.08	6.20	5.64	0.002574007	0.011365707
8164177	79109	*mitogen-activated protein kinase associated protein 1*	*MAPKAP1*	10.10	10.12	10.70	0.002574569	0.011366454
8107282	134430	*WD repeat domain 36*	*WDR36*	7.88	7.60	9.89	0.002576794	0.011372795
8059648				6.60	6.08	7.87	0.002576545	0.011372795
8068510				7.65	7.63	7.08	0.002577213	0.011372907
8144557	4482	*methionine sulfoxide reductase A*	*MSRA*	8.67	8.62	7.48	0.002580719	0.01138664
7974257	27109	*ATP synthase, H+ transporting, mitochondrial Fo complex, subunit s (factor B)*	*ATP5S*	6.72	6.56	7.40	0.002581564	0.011388625
8059878	79781	*IQ motif containing with AAA domain 1*	*IQCA1*	6.08	6.11	7.27	0.002583588	0.011395812
8115918	1212	*clathrin, light chain B*	*CLTB*	11.01	11.08	9.34	0.002583987	0.011395831
8139816				9.04	8.93	7.68	0.002584945	0.011398315

7996720	5681, 1506	*protein serine kinase H1 | chymotrypsin-like*	*PSKH1|CTRL*	9.72	9.63	8.68	0.002585398	0.011398572
7931216	23172	*family with sequence similarity 175, member B*	*FAM175B*	8.58	8.59	10.06	0.002586135	0.011400083
7950082	100133315	*transient receptor potential cation channel, subfamily C, member 2-like*	*LOC100133315*	7.62	7.38	9.39	0.002587377	0.011403815
7971727	220112	*CTAGE family, member 3, pseudogene*	*CTAGE3P*	4.77	4.90	4.73	0.002588239	0.011405873
8168817	1821	*dystrophin related protein 2*	*DRP2*	6.77	6.31	5.83	0.002591084	0.011416665
7985555	79631, 648809	*elongation factor Tu GTP binding domain containing 1 | elongation factor Tu GTP-binding domain-containing protein 1 pseudogene*	*EFTUD1|LOC648809*	5.87	5.78	8.30	0.002594348	0.011429302
7982985	23005	*mitogen-activated protein kinase binding protein 1*	*MAPKBP1*	8.30	8.21	7.32	0.002598772	0.011447048
8016590	81558	*family with sequence similarity 117, member A*	*FAM117A*	8.90	8.55	9.42	0.002602005	0.011459538
7920333				7.77	8.20	6.41	0.002604039	0.011466749
7979387	341880	*solute carrier family 35, member F4*	*SLC35F4*	5.83	5.86	5.44	0.002604581	0.011466981
7925364	55127, 3964	*HEAT repeat containing 1 | lectin, galactoside-binding, soluble, 8*	*HEATR1|LGALS8*	8.43	8.35	11.00	0.002604886	0.011466981
7970381	729171, 644249	*ankyrin repeat domain 20B | hypothetical LOC644249*	*ANKRD20B|LOC644249*	4.88	4.66	5.04	0.002606203	0.011471029
8075263	8508	*nipsnap homolog 1 (C. elegans)*	*NIPSNAP1*	10.81	10.33	11.51	0.002607154	0.011471717
8037355	126299	*zinc finger protein 428*	*ZNF428*	7.13	7.09	6.36	0.002606849	0.011471717
8164810	5900	*ral guanine nucleotide dissociation stimulator*	*RALGDS*	8.80	8.98	7.48	0.002608003	0.011473373
8089954	9657	*IQ motif containing B1*	*IQCB1*	7.50	7.08	9.72	0.002608539	0.011473373
8031526	29903	*coiled-coil domain containing 106*	*CCDC106*	8.67	8.68	7.96	0.002608723	0.011473373
8176006	1527	*testis expressed 28*	*TEX28*	6.64	6.71	6.15	0.002609403	0.011473599
8026350	388512, 84658	*C-type lectin domain family 17, member A | egf-like module containing, mucin-like, hormone receptor-like 3*	*CLEC17A|EMR3*	7.32	7.39	6.86	0.002609886	0.011473599
8043375				11.26	11.26	10.47	0.002609967	0.011473599
8054254	3899	*AF4/FMR2 family, member 3*	*AFF3*	8.50	8.21	5.97	0.002611349	0.011473742
8000998	79001	*vitamin K epoxide reductase complex, subunit 1*	*VKORC1*	12.57	12.89	11.61	0.002610568	0.011473742

8023528	115701	*alpha-kinase 2*	*ALPK2*	8.33	8.68	5.82	0.002611589	0.011473742
7984759	161514	*TBC1 domain family, member 21*	*TBC1D21*	6.80	6.90	6.32	0.002611279	0.011473742
8031600	126206	*NLR family, pyrin domain containing 5*	*NLRP5*	5.91	6.17	5.66	0.002612493	0.01147422
7984488	246777, 79400	*sperm equatorial segment protein 1 | NADPH oxidase, EF-hand calcium binding domain 5*	*SPESP1|NOX5*	6.22	6.16	5.58	0.002612104	0.01147422
7949882	222	*aldehyde dehydrogenase 3 family, member B2*	*ALDH3B2*	6.64	6.68	6.14	0.002613331	0.011476154
8003583	3835	*kinesin family member 22*	*KIF22*	8.09	8.55	10.59	0.002616036	0.011486289
8068361	6526	*solute carrier family 5 (sodium/myo-inositol cotransporter), member 3*	*SLC5A3*	8.47	7.96	10.07	0.002616686	0.011487396
8171105	64109	*cytokine receptor-like factor 2*	*CRLF2*	5.12	5.31	4.88	0.002618633	0.011494195
8163202	79987	*sushi, von Willebrand factor type A, EGF and pentraxin domain containing 1*	*SVEP1*	10.71	9.10	5.77	0.002619633	0.011496835
7944516				6.40	6.57	6.07	0.002623051	0.011510087
7904963				5.46	5.33	6.70	0.002624095	0.011512919
8176012	1527	*testis expressed 28*	*TEX28*	6.64	6.71	6.14	0.002625009	0.011515178
7901038	6202	*ribosomal protein S8*	*RPS8*	12.13	12.09	12.58	0.002627628	0.011521942
7920123	6281	*S100 calcium binding protein A10*	*S100A10*	13.23	13.24	12.76	0.002627748	0.011521942
7983365	140691	*tripartite motif-containing 69*	*TRIM69*	6.16	6.06	5.51	0.002627209	0.011521942
7972257				6.43	6.28	6.07	0.0026311	0.011534887
8170159	680	*bombesin-like receptor 3*	*BRS3*	5.20	5.19	4.93	0.002633673	0.011544415
7991469	283777	*family with sequence similarity 169, member B*	*FAM169B*	6.35	6.43	6.04	0.002634899	0.011548032
7929288	54536	*exocyst complex component 6*	*EXOC6*	7.16	6.13	7.38	0.002635366	0.011548327
7928855	657	*bone morphogenetic protein receptor, type IA*	*BMPR1A*	9.61	9.15	11.34	0.002636711	0.011550715
8075316	5008	*oncostatin M*	*OSM*	6.53	6.70	6.03	0.002636638	0.011550715
8041281	84272	*Yip1 domain family, member 4*	*YIPF4*	10.11	10.17	9.76	0.002637126	0.011550779
7988963	10776	*cAMP-regulated phosphoprotein, 19kDa*	*ARPP19*	8.59	8.95	10.54	0.002637666	0.011551392
8045332	339742, 100134240	*family with sequence similarity 201, member B | hypothetical protein LOC100134240*	*FAM201B|LOC100134240*	8.51	8.58	7.93	0.002639324	0.011556899
8104654				6.99	6.97	6.55	0.002640488	0.011560245

8034393	10224	*zinc finger protein 443*	*ZNF443*	4.97	4.76	5.63	0.002641075	0.01156106
8054740	7849	*paired box 8*	*PAX8*	7.62	7.64	6.94	0.002641703	0.011562057
8059139	79065	*ATG9 autophagy related 9 homolog A (S. cerevisiae)*	*ATG9A*	10.34	10.43	9.08	0.002643083	0.011566343
8157922	4010	*LIM homeobox transcription factor 1, beta*	*LMX1B*	7.64	7.76	6.87	0.002644805	0.011570372
8106950	153643	*family with sequence similarity 81, member B*	*FAM81B*	5.79	5.89	5.52	0.00264445	0.011570372
8011354	162514	*transient receptor potential cation channel, subfamily V, member 3*	*TRPV3*	6.64	6.76	6.23	0.00264574	0.011571881
8069863	337879	*keratin associated protein 8-1*	*KRTAP8-1*	6.66	6.76	6.18	0.002645952	0.011571881
8041592	27436	*echinoderm microtubule associated protein like 4*	*EML4*	9.28	8.75	10.97	0.002647161	0.011575417
8139746	5260	*phosphorylase kinase, gamma 1 (muscle)*	*PHKG1*	6.12	6.09	5.58	0.002648589	0.011579907
8030982	91661, 388561, 126017	*zinc finger protein 765 | zinc finger protein 761 | zinc finger protein 813*	*ZNF765|ZNF761|ZNF813*	8.71	8.55	9.70	0.002650679	0.011587287
7912155				10.20	10.28	8.97	0.002653228	0.011596673
8098740	22947, 728410, 653548, 653545, 653544, 441056, 653543, 26583	*double homeobox 4 | double homeobox 4 like 2 | double homeobox 4 like 3 | double homeobox 4 like 5 | double homeobox 4 like 6 | double homeobox 4 like 4 | double homeobox 4 like 7 | double homeobox 2*	*DUX4|DUX4L2|DUX4L3|DUX4L5|DUX4L6|DUX4L4|DUX4L7|DUX2*	8.57	8.62	7.89	0.002655174	0.011603423
8178811	3126, 3123	*major histocompatibility complex, class II, DR beta 4 | major histocompatibility complex, class II, DR beta 1*	*HLA-DRB4|HLA-DRB1*	6.58	6.52	7.69	0.002657109	0.011608991
7996423	55336, 3299	*F-box and leucine-rich repeat protein 8 | heat shock transcription factor 4*	*FBXL8|HSF4*	8.53	8.50	7.84	0.002657252	0.011608991
8101788	8633	*unc-5 homolog C (C. elegans)*	*UNC5C*	5.68	5.70	5.48	0.00266025	0.011620329
8175977	3654	*interleukin-1 receptor-associated kinase 1*	*IRAK1*	8.81	9.06	7.68	0.002661046	0.01162205
7997230	5713	*proteasome (prosome, macropain) 26S subunit, non-ATPase, 7*	*PSMD7*	10.05	10.29	11.43	0.002661458	0.011622091
7901634	374977	*chromosome 1 open reading frame 175*	*C1orf175*	6.83	6.89	6.53	0.002663888	0.011630941
8014269	246176	*growth arrest-specific 2 like 2*	*GAS2L2*	7.22	7.35	6.65	0.00266485	0.01163275

7938293	677826	*small nucleolar RNA, H/ACA box 45*	*SNORA45*	7.89	8.15	9.30	0.002665108	0.01163275
7940493	220004	*chromosome 11 open reading frame 66*	*C11orf66*	7.92	7.75	6.94	0.002665994	0.011634857
8140107	84277	*DnaJ (Hsp40) homolog, subfamily C, member 30*	*DNAJC30*	9.60	9.54	8.55	0.002667047	0.011637694
7950425	25884	*chordin-like 2*	*CHRDL2*	6.87	6.77	6.04	0.002668419	0.01164192
7901662	7268, 374977	*tetratricopeptide repeat domain 4 | chromosome 1 open reading frame 175*	*TTC4|C1orf175*	8.02	8.07	8.97	0.002670371	0.011648677
8074934	29802	*pre-B lymphocyte 3*	*VPREB3*	6.91	7.05	6.64	0.002671733	0.011650282
7941610	246330	*pellino homolog 3 (Drosophila)*	*PELI3*	7.67	7.73	6.96	0.00267195	0.011650282
8123332				6.45	6.42	5.99	0.002671852	0.011650282
8035445	3727	*jun D proto-oncogene*	*JUND*	10.04	10.13	8.83	0.002675811	0.011665049
8157605				8.06	7.22	6.27	0.002676145	0.011665049
8012473	643904	*ring finger protein 222*	*RNF222*	8.22	8.28	7.56	0.002676648	0.011665482
8029914	27202	*G protein-coupled receptor 77*	*GPR77*	6.56	6.37	5.99	0.002677273	0.011666443
8062377	6714	*v-src sarcoma (Schmidt-Ruppin A-2) viral oncogene homolog (avian)*	*SRC*	9.00	9.07	7.86	0.002682036	0.011679081
7999427	7142	*transition protein 2 (during histone to protamine replacement)*	*TNP2*	6.59	6.56	5.86	0.002681516	0.011679081
8073858	51512	*G-2 and S-phase expressed 1*	*GTSE1*	7.46	7.95	8.62	0.002682048	0.011679081
7934527	338599	*dual specificity phosphatase and pro isomerase domain containing 1*	*DUPD1*	7.07	7.24	6.59	0.002682196	0.011679081
8161446	100131997, 100287333, 100289124	*family with sequence similarity 27, member E3 | family with sequence similarity 27, member E1 | family with sequence similarity 27, member E2*	*FAM27E3|FAM27E1|FAM27E2*	8.45	8.97	7.27	0.002680883	0.011679081
7897728	26270	*F-box protein 6*	*FBXO6*	8.41	8.23	7.22	0.002682821	0.011680043
7920077	339398, 6097	*leucine rich repeat and Ig domain containing 4 | RAR-related orphan receptor C*	*LINGO4|RORC*	7.23	7.11	6.77	0.002685766	0.011691099
8070160	539	*ATP synthase, H+ transporting, mitochondrial F1 complex, O subunit*	*ATP5O*	10.07	10.42	10.49	0.002687489	0.011696673
8157638	92399	*mitochondrial ribosome recycling factor*	*MRRF*	8.69	9.00	9.83	0.002687857	0.011696673
8013259				8.32	8.55	7.90	0.002689725	0.011703039
8130408	26034	*interaction protein for cytohesin exchange factors 1*	*IPCEF1*	5.97	5.80	5.71	0.002693645	0.011718329
8159448	158056	*MAM domain containing 4*	*MAMDC4*	7.83	8.04	7.05	0.00269487	0.011720126

7950012	595, 220064, 440049	*cyclin D1 | oral cancer overexpressed 1 | FLJ42258 protein*	*CCND1|ORAOV1|FLJ42258*	7.59	7.68	6.95	0.002694538	0.011720126
8039062	79788	*zinc finger protein 665*	*ZNF665*	5.38	5.32	6.07	0.002697914	0.011731197
8038139	126147	*netrin 5*	*NTN5*	7.13	7.17	6.68	0.002698228	0.011731197
8163437	57864	*solute carrier family 46, member 2*	*SLC46A2*	6.91	7.07	6.45	0.00269973	0.011735959
8140556	3082	*hepatocyte growth factor (hepapoietin A; scatter factor)*	*HGF*	7.98	5.85	5.49	0.002701168	0.011740443
7913850	56181, 646471	*family with sequence similarity 54, member B | hypothetical LOC646471*	*FAM54B|LOC646471*	7.93	7.99	7.22	0.002703983	0.011750907
7983350	8669	*eukaryotic translation initiation factor 3, subunit J*	*EIF3J*	8.10	8.22	9.43	0.002704803	0.011752701
8044733				5.70	5.47	5.17	0.002705923	0.0117558
8065563	245934	*defensin, beta 121*	*DEFB121*	5.39	5.33	5.13	0.00270699	0.011757818
8075542	253143	*chromosome 22 open reading frame 30*	*C22orf30*	7.78	7.60	9.94	0.002707202	0.011757818
8176018	1527	*testis expressed 28*	*TEX28*	6.71	6.77	6.22	0.002707664	0.011758055
8007259				8.36	8.44	7.71	0.002709959	0.011766251
7939507	84680	*1-aminocyclopropane-1-carboxylate synthase homolog (Arabidopsis)(non-functional)*	*ACCS*	7.74	7.39	6.68	0.002711021	0.011769093
8063057	90203	*sorting nexin family member 21*	*SNX21*	9.11	8.87	7.41	0.002711623	0.011769938
7959102	26353	*heat shock 22kDa protein 8*	*HSPB8*	8.74	8.67	7.63	0.002713354	0.011775677
8171834	6133	*ribosomal protein L9*	*RPL9*	11.90	12.02	12.34	0.002715101	0.01178149
8062174	51614	*ERGIC and golgi 3*	*ERGIC3*	12.59	12.61	11.11	0.002716165	0.011784337
8040103	3398	*inhibitor of DNA binding 2, dominant negative helix-loop-helix protein*	*ID2*	10.34	11.16	9.80	0.002717998	0.011785202
8124391	8335	*histone cluster 1, H2ab*	*HIST1H2AB*	6.89	7.44	9.71	0.002717978	0.011785202
8106709	83734		*ATG10*	7.62	7.69	8.72	0.00271771	0.011785202
8160805	203259	*chromosome 9 open reading frame 25*	*C9orf25*	8.40	8.65	7.73	0.002717388	0.011785202
7935521	60370	*arginine vasopressin-induced 1*	*AVPI1*	10.18	10.18	8.91	0.002718912	0.011785404
8042859	83444	*INO80 complex subunit B*	*INO80B*	8.26	8.35	7.46	0.002719269	0.011785404
8162132	389766	*chromosome 9 open reading frame 153*	*C9orf153*	5.36	5.28	5.08	0.002718595	0.011785404

8108861	80762	*Nedd4 family interacting protein 1*	*NDFIP1*	9.94	9.93	9.23	0.002720234	0.011786047
7952335	85391	*small nucleolar RNA, C/D box 14E*	*SNORD14 E*	7.44	7.32	8.38	0.002719864	0.011786047
8060533	100288797		*LOC10028 8797*	7.65	7.81	7.21	0.002721195	0.011788442
8052583				6.95	6.99	6.57	0.002722579	0.011792667
8061815	359710	*chromosome 20 open reading frame 185*	*C20orf185*	7.18	7.29	6.66	0.002725272	0.011802563
8156573	407012, 100422842, 84909	*microRNA 24-1 | microRNA 3074 | chromosome 9 open reading frame 3*	*MIR24-1|MIR3074 |C9orf3*	5.35	5.42	4.67	0.002726652	0.011806765
8008350	84073	*MYCBP associated protein*	*MYCBPAP*	6.85	6.64	6.35	0.00272983	0.011816985
8148655	84948	*tigger transposable element derived 5*	*TIGD5*	8.55	8.59	7.65	0.00272945	0.011816985
7964677	144577	*chromosome 12 open reading frame 66*	*C12orf66*	6.89	7.08	7.88	0.002730256	0.011817053
7940112	219957	*olfactory receptor, family 9, subfamily Q, member 2*	*OR9Q2*	6.21	6.47	5.74	0.00273174	0.011821481
8127421				6.40	6.50	5.79	0.002732098	0.011821481
7952404	26493	*olfactory receptor, family 8, subfamily B, member 8*	*OR8B8*	6.01	5.84	5.53	0.002733248	0.011824688
7964739	84298	*LLP homolog, long-term synaptic facilitation (Aplysia)*	*LLPH*	7.28	7.29	7.79	0.002733793	0.011825272
8076909	85378	*tubulin, gamma complex associated protein 6*	*TUBGCP6*	7.92	7.89	7.22	0.002734921	0.011828378
8020898	10778	*zinc finger protein 271*	*ZNF271*	6.80	6.90	8.32	0.002736456	0.011833246
8144667				6.80	6.93	7.35	0.002739365	0.011844051
8036584	3963, 653499	*lectin, galactoside-binding, soluble, 7 | lectin, galactoside-binding, soluble, 7B*	*LGALS7|L GALS7B*	7.59	7.61	7.14	0.002742017	0.011853742
8051119	5496	*protein phosphatase, Mg2+/Mn2+ dependent, 1G*	*PPM1G*	10.08	10.06	11.20	0.002743797	0.01185966
7919743	54507, 574406	*ADAMTS-like 4 | chromosome 1 open reading frame 138*	*ADAMTSL 4|C1orf138*	6.73	6.64	6.21	0.002744575	0.011861247
8175016	8862	*apelin*	*APLN*	7.38	7.88	6.96	0.002745547	0.011863672
8041644	130271	*pleckstrin homology domain containing, family H (with MyTH4 domain) member 2*	*PLEKHH2*	6.04	6.47	7.40	0.002746805	0.011867331
8076815	100128818	*LPEQ6126*	*LOC10012 8818*	6.63	6.55	6.27	0.002749445	0.011876959
8115840	1482	*NK2 transcription factor related, locus 5 (Drosophila)*	*NKX2-5*	9.11	9.11	8.45	0.002749926	0.011877263

7976243	53981	*cleavage and polyadenylation specific factor 2, 100kDa*	*CPSF2*	8.77	8.53	10.81	0.002752342	0.011885917
8150881	5324	*pleiomorphic adenoma gene 1*	*PLAG1*	6.93	7.01	8.69	0.002754826	0.011894864
8030999	55422	*zinc finger protein 331*	*ZNF331*	7.24	7.28	8.31	0.002755681	0.011896779
7964484	2583	*beta-1,4-N-acetyl-galactosaminyl transferase 1*	*B4GALNT 1*	8.29	8.17	6.91	0.002758355	0.011906542
8017825				6.33	6.22	5.79	0.002759462	0.01190954
8126303	54210	*triggering receptor expressed on myeloid cells 1*	*TREM1*	5.77	5.89	5.44	0.002760337	0.011911533
8155563	25902, 100133920, 286297, 100506095	*methylenetetrahydrofolate dehydrogenase (NADP+ dependent) 1-like | hypothetical LOC100133920 | hypothetical LOC286297 | monofunctional C1-tetrahydrofolate synthase, mitochondrial-like*	*MTHFD1L |LOC100133920|LOC286297|LOC100506095*	7.86	8.18	7.71	0.002761698	0.011915628
8052947	56603	*cytochrome P450, family 26, subfamily B, polypeptide 1*	*CYP26B1*	7.41	7.43	6.57	0.002762598	0.011917728
8110415	64426	*suppressor of defective silencing 3 homolog (S. cerevisiae)*	*SUDS3*	8.11	8.08	9.53	0.002763223	0.011918643
8043197	8673	*vesicle-associated membrane protein 8 (endobrevin)*	*VAMP8*	8.29	7.57	10.47	0.00276427	0.011921377
8031784	7693, 10520	*zinc finger protein 134 | zinc finger protein 211*	*ZNF134|ZNF211*	7.93	7.78	10.15	0.002765983	0.011926986
7980616	11099	*protein tyrosine phosphatase, non-receptor type 21*	*PTPN21*	9.32	9.21	7.87	0.002767938	0.01193363
8039294	51206	*glycoprotein VI (platelet)*	*GP6*	7.64	7.71	6.99	0.00276885	0.011935781
7936798	50624	*CUB and zona pellucida-like domains 1*	*CUZD1*	5.87	6.07	7.68	0.002773454	0.011950277
8100714	91746	*YTH domain containing 1*	*YTHDC1*	8.37	8.42	10.48	0.002772823	0.011950277
8067380	149986		*LSM14B*	5.66	5.78	5.39	0.002773188	0.011950277
7954789	144245, 84920	*asparagine-linked glycosylation 10, alpha-1,2-glucosyltransferase homolog B (yeast) | asparagine-linked glycosylation 10, alpha-1,2-glucosyltransferase homolog (S. pombe)*	*ALG10B*	7.07	7.12	8.69	0.002774824	0.011954391
7962441	5756	*twinfilin, actin-binding protein, homolog 1 (Drosophila)*	*TWF1*	10.46	10.49	9.70	0.002776336	0.011959122
8154627	3439. 3447	*interferon, alpha 1 | interferon, alpha 13*	*IFNA1|IFNA13*	5.52	5.46	5.25	0.002777238	0.011961225

8166826	8239	*ubiquitin specific peptidase 9, X-linked*	*USP9X*	8.92	8.62	11.80	0.002777743	0.011961613
8161919	7088	*transducin-like enhancer of split 1 (E(sp1) homolog, Drosophila)*	*TLE1*	9.70	8.32	10.14	0.002778633	0.011963664
8032576	166	*amino-terminal enhancer of split*	*AES*	11.46	11.40	10.28	0.002782171	0.011973537
8020423	57534	*mindbomb homolog 1 (Drosophila)*	*MIB1*	7.46	7.17	8.67	0.002781997	0.011973537
7994576	440352, 84127, 400509	*RUN domain containing 2C | RUN domain containing 2A | RUN domain containing 2B*	*RUNDC2C |RUNDC2 A|RUNDC 2B*	8.98	8.70	7.31	0.002781498	0.011973537
8154973	138724	*chromosome 9 open reading frame 131*	*C9orf131*	6.12	6.16	5.80	0.002785425	0.011985754
7946078	50613	*ubiquilin 3*	*UBQLN3*	5.64	5.67	5.27	0.002786957	0.011990562
8044669	11159, 11158	*RAB, member of RAS oncogene family-like 2A | RAB, member of RAS oncogene family-like 2B*	*RABL2A|R ABL2B*	10.39	10.37	9.25	0.002788372	0.011994862
8161265	347252	*insulin-like growth factor binding protein-like 1*	*IGFBPL1*	8.32	8.49	9.15	0.002789739	0.011998953
8020226	10650	*slowmo homolog 1 (Drosophila)*	*SLMO1*	7.29	7.34	6.82	0.002790638	0.011999243
8132539	28988	*drebrin-like*	*DBNL*	10.36	10.49	8.84	0.002790256	0.011999243
8086985	1294, 100313843	*collagen, type VII, alpha 1 | microRNA 711*	*COL7A1| MIR711*	8.24	8.21	7.31	0.00279459	0.012014447
8039961	52	*acid phosphatase 1, soluble*	*ACP1*	9.34	9.44	11.11	0.002795598	0.012016993
7922382	339416	*ankyrin repeat domain 45*	*ANKRD45*	5.21	5.07	5.45	0.002798156	0.012026195
8051361	6716	*steroid-5-alpha-reductase, alpha polypeptide 2 (3-oxo-5 alpha-steroid delta 4-dehydrogenase alpha 2)*	*SRD5A2*	6.16	6.14	5.75	0.002803163	0.012038305
8068612	7485	*tryptophan rich basic protein*	*WRB*	9.29	9.50	10.17	0.002803475	0.012038305
8078772	9389	*solute carrier family 22, member 14*	*SLC22A14*	6.19	6.28	5.70	0.002801795	0.012038305
8127903	22832		*KIAA1009*	5.90	5.72	6.72	0.002802024	0.012038305
8161484	25902, 100133920, 286297, 100506095	*methylenetetrahydrofolate dehydrogenase (NADP+ dependent) 1-like | hypothetical LOC100133920 | hypothetical LOC286297 | monofunctional C1-tetrahydrofolate synthase, mitochondrial-like*	*MTHFD1L |LOC1001 33920|LO C286297|L OC100506 095*	7.85	8.18	7.71	0.002802521	0.012038305
8114918				5.94	5.93	5.58	0.002803122	0.012038305
8137336	9311	*amiloride-sensitive cation channel 3*	*ACCN3*	7.59	7.68	6.97	0.002808769	0.012057449

7980773	440193	*coiled-coil domain containing 88C*	*CCDC88C*	6.97	7.22	8.07	0.00280841	0.012057449
7907859				9.01	9.56	10.20	0.002809814	0.012060141
8127511	80759, 100129128	*KH homology domain containing 1 | KH homology domain containing 1-like*	*KHDC1|KHDC1L*	6.63	6.52	6.09	0.002810808	0.012060824
7974247				5.02	5.06	4.73	0.002810633	0.012060824
8002303	1728	*NAD(P)H dehydrogenase, quinone 1*	*NQO1*	12.52	11.77	10.50	0.002811313	0.012061196
8044008	8808	*interleukin 1 receptor-like 2*	*IL1RL2*	5.86	5.93	5.47	0.002815617	0.01207428
7931140	375940	*deleted in malignant brain tumors 1 pseudogene*	*FLJ46361*	6.86	6.88	6.32	0.002814829	0.01207428
8147373				5.08	5.25	6.37	0.00281551	0.01207428
8158939	8328	*growth factor independent 1B transcription repressor*	*GFI1B*	7.48	7.49	7.03	0.002816389	0.012075797
8012625	8735	*myosin, heavy chain 13, skeletal muscle*	*MYH13*	5.68	5.93	5.49	0.002817445	0.012078528
7930181	57412, 119032	*arsenic (+3 oxidation state) methyltransferase | chromosome 10 open reading frame 32*	*AS3MT|C10orf32*	8.73	8.32	10.22	0.002819509	0.012085583
7982187	321	*amyloid beta (A4) precursor protein-binding, family A, member 2*	*APBA2*	7.81	6.91	7.45	0.002821984	0.012090985
8064168	54923	*Lck interacting transmembrane adaptor 1*	*LIME1*	8.47	8.53	7.61	0.002822025	0.012090985
8156848	8013, 2130	*nuclear receptor subfamily 4, group A, member 3 | Ewing sarcoma breakpoint region 1*	*NR4A3|EWSR1*	9.67	7.45	6.54	0.002821999	0.012090985
7907222	5396	*paired related homeobox 1*	*PRRX1*	12.67	10.81	8.08	0.002822986	0.012093306
8135688	51691	*N(alpha)-acetyltransferase 38, NatC auxiliary subunit*	*NAA38*	7.02	7.10	8.07	0.002825551	0.012098909
8028624	55095	*sterile alpha motif domain containing 4B*	*SAMD4B*	9.97	10.18	9.07	0.002825336	0.012098909
8026503	148231	*hypothetical LOC148231*	*FLJ25328*	6.36	6.33	5.67	0.002825114	0.012098909
8084694	1974	*eukaryotic translation initiation factor 4A2*	*EIF4A2*	10.38	10.15	11.50	0.002826993	0.012103286
8165669	4514, 4509	*cytochrome c oxidase III | ATP synthase F0 subunit 8*	*COX3|ATP8*	13.31	13.20	13.47	0.002827905	0.012105397
8162404	1842	*extracellular matrix protein 2, female organ and adipocyte specific*	*ECM2*	7.92	7.63	5.94	0.002833309	0.012117569
8046564	3231	*homeobox D1*	*HOXD1*	7.88	8.04	7.18	0.002833151	0.012117569
7997192	3250	*haptoglobin-related protein*	*HPR*	5.75	5.71	5.40	0.002833062	0.012117569

8155422	220869, 728013, 150472, 644019, 445571, 653510, 100507355	*COBW domain containing 5 | COBW domain containing 7 | COBW domain containing 2 | COBW domain containing 6 | COBW domain containing 3 | COBW domain-containing protein 5-like | hypothetical LOC100507355*	*CBWD5|CBWD7|CBWD2|CBWD6|CBWD3|LOC653510|LOC100507355*	7.15	7.40	9.27	0.002833939	0.012117569
8074330	729461, 26080	*hypothetical LOC729461 | non-protein coding RNA 281*	*LOC729461|NCRNA00281*	6.19	6.37	5.77	0.002832894	0.012117569
7957534				5.74	5.61	5.38	0.002833395	0.012117569
8133221				7.63	7.73	6.86	0.002834107	0.012117569
8167198				6.79	6.87	6.06	0.002833426	0.012117569
7903393	1901	*sphingosine-1-phosphate receptor 1*	*S1PR1*	6.32	7.31	6.35	0.002839426	0.012135655
7932530	5305	*phosphatidylinositol-5-phosphate 4-kinase, type II, alpha*	*PIP4K2A*	8.78	9.00	10.15	0.002839425	0.012135655
7996160	388282		*LOC388282*	6.66	6.71	6.14	0.002840438	0.012135655
8018445	85451, 100129946	*unkempt homolog (Drosophila) | hypothetical LOC100129946*	*UNK|LOC100129946*	6.36	6.39	5.93	0.002839883	0.012135655
7940491				7.26	7.24	6.84	0.002840022	0.012135655
7921420	81448	*olfactory receptor, family 6, subfamily K, member 2*	*OR6K2*	5.22	5.25	5.03	0.002841395	0.012137946
8103812	140458	*ankyrin repeat and SOCS box-containing 5*	*ASB5*	5.35	5.41	5.19	0.002841853	0.012138104
7910241	2987	*guanylate kinase 1*	*GUK1*	9.90	10.24	8.68	0.002842558	0.012139319
8098193				5.02	4.87	6.33	0.002844959	0.012147776
7999727	368	*ATP-binding cassette, sub-family C (CFTR/MRP), member 6*	*ABCC6*	7.09	7.11	6.57	0.002845478	0.012148195
8005202	26800	*small nucleolar RNA, C/D box 49A*	*SNORD49A*	6.89	7.02	7.97	0.002846391	0.012150299
8005707	5606	*mitogen-activated protein kinase kinase 3*	*MAP2K3*	9.67	10.18	8.15	0.002847282	0.012152305
8116760	83732	*RIO kinase 1 (yeast)*	*RIOK1*	7.70	7.82	9.36	0.002851809	0.012169826

8004784	247	*arachidonate 15-lipoxygenase, type B*	*ALOX15B*	7.23	7.34	6.54	0.002853868	0.012176813
8144712	57604	*chromosome 8 open reading frame 79*	*C8orf79*	6.09	5.61	5.26	0.002855014	0.012179904
8173513	6191	*ribosomal protein S4, X-linked*	*RPS4X*	12.55	12.66	12.99	0.002858327	0.012192235
8035380	3594	*interleukin 12 receptor, beta 1*	*IL12RB1*	6.90	6.77	6.31	0.002858931	0.012193007
8031550	29924	*epsin 1*	*EPN1*	10.41	10.44	9.01	0.002859812	0.012193164
8144230	169270	*zinc finger protein 596*	*ZNF596*	6.30	6.28	5.64	0.002859501	0.012193164
8078136				8.54	8.60	7.66	0.002860502	0.012194306
8032473	374872	*chromosome 19 open reading frame 35*	*C19orf35*	7.19	7.21	6.56	0.002862478	0.012199125
7954777	84920, 144245	*asparagine-linked glycosylation 10, alpha-1,2-glucosyltransferase homolog (S. pombe) | asparagine-linked glycosylation 10, alpha-1,2-glucosyltransferase homolog B (yeast)*	*ALG10|ALG10B*	7.10	7.15	8.84	0.002862429	0.012199125
7900365	84879	*major facilitator superfamily domain containing 2A*	*MFSD2A*	7.30	8.08	8.88	0.002863724	0.012200832
7942267	387273	*keratin associated protein 5-10*	*KRTAP5-10*	8.28	8.30	7.86	0.002863574	0.012200832
7920903	6746	*signal sequence receptor, beta (translocon-associated protein beta)*	*SSR2*	13.37	13.36	12.92	0.002865185	0.012205258
8126337	10817	*fibroblast growth factor receptor substrate 3*	*FRS3*	8.68	8.71	7.53	0.002866113	0.01220741
8128767	9841, 64780	*zinc finger and BTB domain containing 24 | microtubule associated monoxygenase, calponin and LIM domain containing 1*	*ZBTB24|MICAL1*	7.40	7.20	9.34	0.002871283	0.012227626
7991106	114817	*chondroitin sulfate proteoglycan 4 pseudogene 5*	*CSPG4P5*	7.51	7.46	6.65	0.002878104	0.012253057
8154392				5.62	5.59	5.37	0.002878052	0.012253057
8034379	79973	*zinc finger protein 442*	*ZNF442*	7.06	6.85	7.32	0.002880751	0.01226252
8154951	2752	*glutamate-ammonia ligase*	*GLUL*	11.75	11.00	12.08	0.00288186	0.012265431
7905481				5.65	5.65	6.84	0.002884481	0.012274776
8115014	27190	*interleukin 17B*	*IL17B*	7.13	7.24	6.81	0.002885728	0.012278275
7949592	9610	*Ras and Rab interactor 1*	*RIN1*	8.87	8.87	7.14	0.002888578	0.012288589
7990345	8482	*semaphorin 7A, GPI membrane anchor (John Milton Hagen blood group)*	*SEMA7A*	8.53	10.10	8.41	0.002890728	0.012295924
8160441	1029	*cyclin-dependent kinase inhibitor 2A (melanoma, p16, inhibits CDK4)*	*CDKN2A*	9.38	9.49	7.98	0.00289201	0.012299562

8099395	9364		*RAB28*	8.17	8.57	9.69	0.002892941	0.01230171
8027920	2116	*ets variant 2*	*ETV2*	7.09	7.23	6.68	0.002893751	0.012303344
8001830	146223	*CKLF-like MARVEL transmembrane domain containing 4*	*CMTM4*	7.62	7.73	8.84	0.002895462	0.012308804
8071434	1399	*v-crk sarcoma virus CT10 oncogene homolog (avian)-like*	*CRKL*	9.49	9.39	10.00	0.002896971	0.012310019
7997453	5336	*phospholipase C, gamma 2 (phosphatidylinositol-specific)*	*PLCG2*	6.66	6.67	7.11	0.002898732	0.012310019
8114511	51237	*plasma cell-induced ER protein 1*	*MGC2950 6*	6.27	6.22	5.73	0.002898593	0.012310019
7912750	54455	*F-box protein 42*	*FBXO42*	9.17	9.43	8.76	0.002898573	0.012310019
8047078	54842	*major facilitator superfamily domain containing 6*	*MFSD6*	8.28	7.81	7.10	0.00289722	0.012310019
8020668	55364	*Impact homolog (mouse)*	*IMPACT*	8.15	7.67	9.89	0.00289747	0.012310019
8074070	55586	*myo-inositol oxygenase*	*MIOX*	8.20	8.35	7.55	0.002898139	0.012310019
8027947	27033	*zinc finger and BTB domain containing 32*	*ZBTB32*	7.18	7.09	6.67	0.002903044	0.012324704
8075817				8.80	8.68	7.98	0.002902875	0.012324704
8088299	201625	*dynein, axonemal, heavy chain 12*	*DNAH12*	4.76	4.84	4.67	0.002905255	0.012328652
8155510	548321, 100133121, 100132948	*family with sequence similarity 27, member A | family with sequence similarity 27, member B | family with sequence similarity 27, member C*	*FAM27A|FAM27B|FAM27C*	8.63	8.88	7.96	0.002905066	0.012328652
8046586				4.73	4.79	5.03	0.002904597	0.012328652
8071646	7441	*pre-B lymphocyte 1*	*VPREB1*	5.92	5.90	5.42	0.002905877	0.012329478
8118124	401250	*mitochondrial coiled-coil domain 1*	*MCCD1*	7.92	7.99	7.55	0.002908062	0.012336933
7937944	119772	*olfactory receptor, family 52, subfamily M, member 1*	*OR52M1*	6.52	6.48	6.19	0.002908555	0.012337213
8031374	2204	*Fc fragment of IgA, receptor for*	*FCAR*	6.38	6.29	6.03	0.002909411	0.012339029
8147516	4147	*matrilin 2*	*MATN2*	7.52	7.44	8.34	0.002910463	0.012341679
7990825				6.11	6.24	5.91	0.002911131	0.012342699
7974566	55745	*MU-2/AP1M2 domain containing, death-inducing*	*MUDENG*	8.17	7.89	8.97	0.002912022	0.01234285
8043438	28299, 3514	*immunoglobulin kappa variable 1-5 | immunoglobulin kappa constant*	*IGKV1-5|IGKC*	9.71	9.62	8.88	0.002911686	0.01234285
8079370	10803	*chemokine (C-C motif) receptor 9*	*CCR9*	5.75	5.83	5.50	0.002913964	0.012349272

7936706	79892	*chromosome 10 open reading frame 119*	*C10orf119*	9.11	9.50	10.63	0.002917201	0.012361172
7937782	10077	*tetraspanin 32*	*TSPAN32*	7.61	7.59	7.03	0.002918713	0.012365767
8115806	92181	*ubiquitin domain containing 2*	*UBTD2*	8.91	8.91	9.78	0.002921388	0.012375284
8061247	54453	*Ras and Rab interactor 2*	*RIN2*	9.58	9.39	7.75	0.002924839	0.012386266
8099144	132884	*Ellis van Creveld syndrome 2*	*EVC2*	7.16	7.31	6.71	0.002924501	0.012386266
7934812	23063	*wings apart-like homolog (Drosophila)*	*WAPAL*	7.98	7.83	9.94	0.002926321	0.012390723
8127128	26268	*F-box protein 9*	*FBXO9*	8.34	8.45	7.84	0.002928	0.012396015
7968242	2971	*general transcription factor IIIA*	*GTF3A*	8.99	9.54	9.75	0.002929531	0.012399791
7973149	84659	*ribonuclease, RNase A family, 7*	*RNASE7*	6.26	6.28	5.72	0.002929751	0.012399791
7922462	63931	*mitochondrial ribosomal protein S14*	*MRPS14*	9.09	9.02	9.49	0.002930318	0.012400375
8008544	84643	*kinesin family member 2B*	*KIF2B*	6.71	6.80	6.17	0.002933489	0.012411129
8041638				5.35	5.18	6.42	0.002933719	0.012411129
8117529				4.54	4.69	5.27	0.00293559	0.012417221
8020162	83650, 146861, 643664, 646000	*acyl-malonyl condensing enzyme 1-like 2 | acyl-malonyl condensing enzyme 1 | acyl-malonyl condensing enzyme 1-like 3 | acyl-malonyl condensing enzyme 1-like 1*	*AMAC1L2| AMAC1L3| AMAC1L1*	7.75	7.75	7.19	0.002938292	0.012426833
7909896				6.71	6.76	6.25	0.002939368	0.012429562
8053030	51471	*N-acetyltransferase 8B (GCN5-related, putative, gene/pseudogene)*	*NAT8B*	6.75	6.77	6.21	0.002940473	0.012432411
7958913	4939	*2'-5'-oligoadenylate synthetase 2, 69/71kDa*	*OAS2*	7.22	6.72	5.71	0.002943399	0.012440539
7928189	219699	*unc-5 homolog B (C. elegans)*	*UNC5B*	9.39	11.24	9.17	0.002943042	0.012440539
8003700	1801, 124641	*DPH1 homolog (S. cerevisiae) | ovarian tumor suppressor candidate 2*	*DPH1|OV CA2*	9.38	9.44	8.15	0.002943688	0.012440539
7944656	6309	*sterol-C5-desaturase (ERG3 delta-5-desaturase homolog, S. cerevisiae)-like*	*SC5DL*	9.12	8.60	10.84	0.002944429	0.01244185
8131135	55501	*carbohydrate (chondroitin 4) sulfotransferase 12*	*CHST12*	9.98	9.97	8.86	0.002945192	0.012443254
8087825	84836	*abhydrolase domain containing 14B*	*ABHD14B*	10.30	10.30	8.51	0.002948602	0.012455837
8059951				6.73	6.77	6.18	0.002950347	0.012461386
8083053	287015	*tripartite motif-containing 42*	*TRIM42*	5.94	5.94	5.60	0.00295447	0.012476973
8021777	400661		*FLJ44881*	5.24	5.30	4.95	0.002954923	0.01247706

7958582	84260, 9815	*trichoplein, keratin filament binding | G protein-coupled receptor kinase interacting ArfGAP 2*	*TCHP|GIT 2*	7.69	7.84	7.29	0.002956904	0.012483601
7982587	400359	*chromosome 15 open reading frame 53*	*C15orf53*	6.86	6.88	6.50	0.002962723	0.012506337
8157516	22954	*tripartite motif-containing 32*	*TRIM32*	10.13	9.90	9.19	0.00296327	0.01250682
7901549	1376	*carnitine palmitoyltransferase 2*	*CPT2*	7.79	7.75	8.67	0.00296555	0.012511281
8110865	50805	*iroquois homeobox 4*	*IRX4*	6.93	6.90	6.47	0.002965628	0.012511281
7952341	79827	*adipocyte-specific adhesion molecule*	*ASAM*	11.69	11.67	9.96	0.002965382	0.012511281
7973393	64806	*interleukin 25*	*IL25*	6.58	6.64	6.19	0.002967995	0.01251944
7971311	55068	*ecto-NOX disulfide-thiol exchanger 1*	*ENOX1*	7.69	7.39	5.84	0.002970728	0.012528357
8136891				4.70	4.64	5.12	0.002970977	0.012528357
8071745	150248	*chromosome 22 open reading frame 15*	*C22orf15*	6.63	6.70	6.06	0.002972536	0.0125331
7900699	991	*cell division cycle 20 homolog (S. cerevisiae)*	*CDC20*	8.92	9.50	11.59	0.002977224	0.012539281
7999532	2935	*G1 to S phase transition 1*	*GSPT1*	10.47	10.59	11.73	0.002977477	0.012539281
8081362	79598	*centrosomal protein 97kDa*	*CEP97*	7.19	6.78	8.71	0.002974944	0.012539281
7924465	79802	*HHIP-like 2*	*HHIPL2*	6.36	6.47	5.95	0.00297621	0.012539281
7933877	221037	*jumonji domain containing 1C*	*JMJD1C*	8.15	7.95	11.10	0.002975907	0.012539281
8060484	10528, 26793	*NOP56 ribonucleoprotein homolog (yeast) | small nucleolar RNA, C/D box 56*	*NOP56|SN ORD56*	7.89	7.90	9.17	0.002977158	0.012539281
8023184	652991, 100508378	*SKI family transcriptional corepressor 2 | SKI family transcriptional corepressor 2-like*	*SKOR2|LO C1005083 78*	7.79	7.89	7.11	0.002976753	0.012539281
7981775				7.33	7.47	6.68	0.002975479	0.012539281
8156167	60560	*N(alpha)-acetyltransferase 35, NatC auxiliary subunit*	*NAA35*	8.36	8.57	10.01	0.002977917	0.012539307
8105040	9180	*oncostatin M receptor*	*OSMR*	10.69	10.77	7.02	0.002979306	0.012543326
8113491	134429	*StAR-related lipid transfer (START) domain containing 4*	*STARD4*	6.82	6.43	8.59	0.002980236	0.012545413
7952305	657	*bone morphogenetic protein receptor, type IA*	*BMPR1A*	9.39	8.95	11.13	0.002981361	0.012547628
8080619				6.66	6.89	6.44	0.002981632	0.012547628
7955845	3229	*homeobox C13*	*HOXC13*	6.76	6.72	6.16	0.002983216	0.012548805
7984319	5604, 10302	*mitogen-activated protein kinase kinase 1 | small nuclear RNA activating complex, polypeptide 5, 19kDa*	*MAP2K1|S NAPC5*	9.37	10.04	9.75	0.002983081	0.012548805

7995326				8.20	8.15	7.47	0.002982584	0.012548805
8179628	10107	*tripartite motif-containing 10*	*TRIM10*	6.12	6.17	5.78	0.002984134	0.012550839
8037444	9310	*zinc finger protein 235*	*ZNF235*	6.44	6.00	7.09	0.002986647	0.012556367
7949344	10004	*N-acetylated alpha-linked acidic dipeptidase-like 1*	*NAALADL 1*	8.43	8.22	7.30	0.002988058	0.012556367
8175302	26071	*family with sequence similarity 127, member B*	*FAM127B*	12.10	12.17	11.03	0.002987635	0.012556367
7967175	84678	*lysine (K)-specific demethylase 2B*	*KDM2B*	7.58	7.81	8.96	0.002987884	0.012556367
8006321	406968	*microRNA 193a*	*MIR193A*	7.81	7.87	7.13	0.002986671	0.012556367
8116996				6.03	6.11	5.63	0.002987938	0.012556367
8051075	2976	*general transcription factor IIIC, polypeptide 2, beta 110kDa*	*GTF3C2*	9.96	9.85	10.73	0.002990227	0.012558172
8133625	375593, 378108	*tripartite motif-containing 73 | tripartite motif-containing 74*	*TRIM73|TRIM74*	7.57	7.58	6.93	0.002989481	0.012558172
7995419				6.37	6.47	5.76	0.002989965	0.012558172
8156525				4.67	4.58	4.56	0.002989004	0.012558172
7957962	121599	*Spi-C transcription factor (Spi-1/PU.1 related)*	*SPIC*	4.82	5.00	4.65	0.002991109	0.012560047
7915955	54558	*spermatogenesis associated 6*	*SPATA6*	8.10	7.72	8.71	0.002991978	0.012561868
8103244	285533	*ring finger protein 175*	*RNF175*	6.44	6.48	7.08	0.002993002	0.01256434
8075564	10739	*ret finger protein-like 2*	*RFPL2*	5.29	5.43	5.12	0.002993482	0.012564528
8049079	165100	*chromosome 2 open reading frame 57*	*C2orf57*	6.62	6.61	6.16	0.002994382	0.012566478
8000692	552900, 654483, 440354, 595101	*bolA homolog 2 (E. coli) | bolA homolog 2B (E. coli) | PI-3-kinase-related kinase SMG-1 pseudogene*	*BOLA2|BOLA2B|LOC440354|LOC595101*	11.18	11.13	12.29	0.002996573	0.012573846
7971150	10186	*lipoma HMGIC fusion partner*	*LHFP*	11.84	11.92	9.74	0.00299752	0.012575991
8070567	7033	*trefoil factor 3 (intestinal)*	*TFF3*	7.02	7.05	6.59	0.00300135	0.012588404
7928944	9060	*3'-phosphoadenosine 5'-phosphosulfate synthase 2*	*PAPSS2*	11.77	11.27	10.08	0.003001112	0.012588404
8015189	81850, 81851	*keratin associated protein 1-3 | keratin associated protein 1-1*	*KRTAP1-3|KRTAP1-1*	8.01	8.40	7.36	0.003002638	0.012591974
8075057				6.62	5.99	5.45	0.003003169	0.012592373

8048112	402117	*von Willebrand factor C domain-containing protein 2-like*	*VWC2L*	5.43	5.46	5.06	0.00300403	0.012594153
7937251	441581, 448831, 100288801	*FSHD region gene 2 family, member B | FSHD region gene 2 family, member C*	*FRG2B|FRG2C*	6.27	6.22	5.82	0.003004978	0.012596301
8168058	27112	*family with sequence similarity 155, member B*	*FAM155B*	7.56	7.61	8.25	0.003009579	0.01261054
7941746	254439	*chromosome 11 open reading frame 86*	*C11orf86*	7.49	7.47	7.00	0.003009528	0.01261054
8019754	348262	*family with sequence similarity 195, member B*	*FAM195B*	9.22	9.43	8.31	0.003009686	0.01261054
7963386	3888	*keratin 82*	*KRT82*	6.44	6.34	5.84	0.003012264	0.012617679
8016168	113026	*phospholipase C, delta 3*	*PLCD3*	10.41	10.40	8.51	0.003012202	0.012617679
8087513	10293	*TRAF interacting protein*	*TRAIP*	6.77	6.88	8.30	0.003015512	0.012629451
8170794	554	*arginine vasopressin receptor 2*	*AVPR2*	6.85	6.93	6.43	0.003017075	0.012633807
8146544	137886	*UBX domain protein 2B*	*UBXN2B*	7.14	6.79	8.99	0.003017427	0.012633807
8164742	64794	*DEAD (Asp-Glu-Ala-Asp) box polypeptide 31*	*DDX31*	7.42	7.78	8.35	0.003019311	0.01263803
7983469	79029	*spermatogenesis associated 5-like 1*	*SPATA5L1*	6.73	6.70	7.26	0.00301899	0.01263803
8107373				6.55	6.38	5.76	0.003020768	0.012642295
7977161	122402	*tudor domain containing 9*	*TDRD9*	5.32	5.18	5.04	0.003024302	0.012653645
7947742				6.19	6.33	6.17	0.003024357	0.012653645
8052861	6637	*small nuclear ribonucleoprotein polypeptide G*	*SNRPG*	6.43	6.47	7.25	0.003025438	0.012656337
7923386	25802	*leiomodin 1 (smooth muscle)*	*LMOD1*	8.86	8.53	6.31	0.003026087	0.012657218
8095907	80144	*Fraser syndrome 1*	*FRAS1*	7.35	7.71	9.85	0.003027715	0.012658523
8005289	83450	*leucine rich repeat containing 48*	*LRRC48*	7.07	7.01	5.99	0.003026987	0.012658523
7986541	374666, 653635, 375260, 100287171, 375690, 100288778, 100134445	*WAS protein family homolog 3 pseudogene | WAS protein family homolog 7 pseudogene | WAS protein family homolog 2 pseudogene | WAS protein family homolog 1 | WAS protein family homolog 5 pseudogene | WAS protein family homolog 1 pseudogene | hypothetical LOC100134445*	*WASH3P|WASH7P|WASH2P|WASH1|WASH5P|LOC100288778|LOC100134445*	9.60	9.38	8.58	0.003027603	0.012658523
8039180	10990	*leukocyte immunoglobulin-like receptor, subfamily B (with TM and ITIM domains), member 5*	*LILRB5*	6.77	6.87	6.23	0.00303175	0.012673559

8076673	84247	*leucine zipper, down-regulated in cancer 1-like*	*LDOC1L*	9.44	9.52	8.44	0.003036159	0.012690151
8099811				6.56	6.55	5.85	0.00304018	0.01270328
8104319				7.22	7.24	6.62	0.003039825	0.01270328
8028397	147965	*family with sequence similarity 98, member C*	*FAM98C*	9.09	9.05	7.88	0.003040804	0.012704048
8004802	54785	*chromosome 17 open reading frame 59*	*C17orf59*	6.99	7.00	6.37	0.003041508	0.012705149
7954701	254013	*chromosome 12 open reading frame 72*	*C12orf72*	5.87	6.04	5.52	0.003043592	0.012712019
7954310	100008588	*RNA, 18S ribosomal 1*	*RN18S1*	13.92	13.85	13.77	0.003046742	0.01272333
8053427				6.19	5.84	5.84	0.003050034	0.012735237
7898371	644634	*UPF0627 protein ENSP00000358171-like*	*LOC644634*	6.78	6.76	6.41	0.003056143	0.012758898
8089072	1371	*coproporphyrinogen oxidase*	*CPOX*	7.65	7.51	8.38	0.00305695	0.0127602
8136045	3663	*interferon regulatory factor 5*	*IRF5*	6.48	6.46	6.12	0.003057338	0.0127602
8138527	256227		*MGC87042*	8.49	7.17	8.65	0.003061043	0.012773817
7970735				8.34	8.52	7.66	0.003063456	0.012782035
7918487	79961	*DENN/MADD domain containing 2D*	*DENND2D*	8.11	7.81	7.02	0.003064478	0.012784453
8005894	8456	*forkhead box N1*	*FOXN1*	6.36	6.40	5.78	0.003065826	0.012786381
8000716	26470	*seizure related 6 homolog (mouse)-like 2*	*SEZ6L2*	9.88	9.79	8.98	0.003065415	0.012786381
8109572	100190949	*chromosome 5 open reading frame 52*	*C5orf52*	6.00	5.84	5.51	0.00306864	0.012794148
8005636				8.37	8.31	6.87	0.003068931	0.012794148
8084122				7.68	7.78	7.13	0.003069018	0.012794148
8088485	55079	*FEZ family zinc finger 2*	*FEZF2*	7.49	7.46	6.96	0.003070307	0.012795828
8071530	25812, 29797, 646074, 29774	*POM121 membrane glycoprotein-like 1, pseudogene | POM121 membrane glycoprotein-like 8 pseudogene | POM121 membrane glycoprotein-like 10, pseudogene | POM121 membrane glycoprotein-like 9, pseudogene*	*POM121L1P|POM121L8P|POM121L10P|POM121L9P*	7.41	7.48	6.90	0.003069922	0.012795828
8091032	668	*forkhead box L2*	*FOXL2*	7.89	7.95	6.75	0.003072612	0.012803586
7947815	53	*acid phosphatase 2, lysosomal*	*ACP2*	10.77	10.69	9.28	0.003077367	0.012817849

8041122	5500	*protein phosphatase 1, catalytic subunit, beta isozyme*	*PPP1CB*	10.33	10.22	10.92	0.003076651	0.012817849
7901140	23139	*microtubule associated serine/threonine kinase 2*	*MAST2*	10.17	10.37	9.43	0.003077365	0.012817849
8178193	3122	*major histocompatibility complex, class II, DR alpha*	*HLA-DRA*	6.21	6.13	7.38	0.003080374	0.012821124
8167887	28986	*melanoma antigen family H, 1*	*MAGEH1*	8.15	8.03	7.20	0.003080125	0.012821124
8146115	56892	*chromosome 8 open reading frame 4*	*C8orf4*	7.40	6.37	6.13	0.00308009	0.012821124
7967091	121665	*signal peptide peptidase 3*	*SPPL3*	10.61	10.50	10.09	0.003079471	0.012821124
8023218				6.75	6.59	6.04	0.003080203	0.012821124
8069252	54039	*poly(rC) binding protein 3*	*PCBP3*	9.54	9.24	7.45	0.003082357	0.01282568
7982287	89839	*Rho GTPase activating protein 11B*	*ARHGAP1 1B*	6.77	6.81	8.18	0.003082051	0.01282568
8150599	5591	*protein kinase, DNA-activated, catalytic polypeptide*	*PRKDC*	8.19	7.97	10.78	0.003084058	0.012828297
8083709	10051	*structural maintenance of chromosomes 4*	*SMC4*	8.86	8.19	10.36	0.003084319	0.012828297
8042079	114800	*coiled-coil domain containing 85A*	*CCDC85A*	7.39	7.87	6.74	0.003083968	0.012828297
8154916	2592	*galactose-1-phosphate uridylyltransferase*	*GALT*	9.14	8.69	7.87	0.00308537	0.012829152
8073397	23264	*zinc finger CCCH-type containing 7B*	*ZC3H7B*	11.21	10.40	10.00	0.003085414	0.012829152
8137118	100289678, 155060	*zinc finger family member 783 | AI894139 pseudogene*	*ZNF783|L OC155060*	7.77	7.83	7.10	0.003087226	0.01283484
7988605	9318	*COP9 constitutive photomorphogenic homolog subunit 2 (Arabidopsis)*	*COPS2*	10.72	10.92	11.36	0.003091126	0.012844641
7989708	123263	*mitochondrial methionyl-tRNA formyltransferase*	*MTFMT*	8.94	9.05	10.18	0.003090958	0.012844641
8036702	282617	*interleukin 28B (interferon, lambda 3)*	*IL28B*	9.04	9.18	8.07	0.00309137	0.012844641
8019585	81850, 81851	*keratin associated protein 1-3 | keratin associated protein 1-1*	*KRTAP1-3|KRTAP1-1*	8.01	8.40	7.36	0.003091808	0.012844641
8157123				5.19	5.33	4.94	0.003091414	0.012844641
8174594				5.11	5.21	6.14	0.003092297	0.012844823
8179391	8859	*serine/threonine kinase 19*	*STK19*	8.67	8.67	7.77	0.003095157	0.012854852
7937198				7.26	7.30	6.74	0.00309605	0.012856714
8080938	4286	*microphthalmia-associated transcription factor*	*MITF*	7.83	6.80	6.69	0.003096954	0.012858618
8162533	5727	*patched 1*	*PTCH1*	10.03	8.55	10.76	0.0030983	0.012862356

7961252	11272	*proline rich 4 (lacrimal)*	*PRR4*	8.32	8.41	7.27	0.003099872	0.012867032
8037043				6.87	6.86	6.38	0.003101425	0.012871628
8016088	124808	*coiled-coil domain containing 43*	*CCDC43*	7.62	7.54	9.55	0.003103075	0.012876624
8162373	4969	*osteoglycin*	*OGN*	6.98	5.99	5.26	0.003103796	0.012877764
7942809				5.69	5.89	5.40	0.003104766	0.012879939
7931778	10531	*pitrilysin metallopeptidase 1*	*PITRM1*	11.06	11.09	10.29	0.003108241	0.01289065
8017133	348235	*spindle and kinetochore associated complex subunit 2*	*SKA2*	7.94	7.81	10.20	0.003107824	0.01289065
8059674	9290	*G protein-coupled receptor 55*	*GPR55*	6.08	6.14	5.82	0.003109881	0.012891894
8011062	51763	*inositol polyphosphate-5-phosphatase K*	*INPP5K*	9.57	9.51	8.30	0.003109567	0.012891894
8097811	54553		*DKFZP43 4I0714*	6.97	7.11	7.37	0.003109863	0.012891894
7970716	222484	*ligand of numb-protein X 2*	*LNX2*	6.98	7.37	9.23	0.003111159	0.012895342
8036155	58510	*proline dehydrogenase (oxidase) 2*	*PRODH2*	6.34	6.46	6.04	0.003115097	0.012909808
7912496	4524	*methylenetetrahydrofolate reductase (NAD(P)H)*	*MTHFR*	8.50	7.87	7.15	0.00311563	0.012910164
7967039	4440	*musashi homolog 1 (Drosophila)*	*MSI1*	7.34	7.36	8.51	0.003116907	0.012910328
7994074	6338	*sodium channel, nonvoltage-gated 1, beta*	*SCNN1B*	7.06	7.13	6.74	0.003117079	0.012910328
8001658	29105	*chromosome 16 open reading frame 80*	*C16orf80*	9.95	10.26	11.06	0.003116644	0.012910328
7919157	644591, 164022, 5478	*peptidylprolyl isomerase A (cyclophilin A)-like 4G | peptidylprolyl isomerase A (cyclophilin A)-like 4A | peptidylprolyl isomerase A (cyclophilin A)*	*PPIAL4G| PPIAL4A*	7.03	7.02	7.25	0.003117458	0.012910328
7992998	23295	*mahogunin, ring finger 1*	*MGRN1*	10.17	10.19	9.16	0.003118258	0.012911786
7930777	5406, 1278	*pancreatic lipase | collagen, type I, alpha 2*	*PNLIP|CO L1A2*	5.19	5.26	5.06	0.003123263	0.012930657
7972665	196541	*chromosome 13 open reading frame 39*	*C13orf39*	5.10	5.07	4.87	0.003124472	0.012931822
8060080	389090	*olfactory receptor, family 6, subfamily B, member 2*	*OR6B2*	5.35	5.40	4.85	0.003124888	0.012931822
7933190	100131195		*LOC10013 1195*	6.04	6.03	5.58	0.003124182	0.012931822
7983777				4.84	4.81	4.58	0.003125589	0.01293287
8027431	9745	*zinc finger protein 536*	*ZNF536*	6.58	6.39	5.74	0.003129154	0.012945766
8130495				4.51	4.54	4.84	0.003129986	0.012947353

8120378	57691		*KIAA1586*	7.73	7.70	8.83	0.003133427	0.012959729
8137537	2020	*engrailed homeobox 2*	*EN2*	7.53	7.66	7.12	0.003134005	0.012960261
7931181	340784	*H6 family homeobox 3*	*HMX3*	8.22	8.39	7.49	0.003134613	0.0129607
8113784				6.40	6.60	6.02	0.003135009	0.0129607
7939093	120526	*DnaJ (Hsp40) homolog, subfamily C, member 24*	*DNAJC24*	6.68	6.75	7.97	0.003136542	0.012965183
8174026	392510	*YWHAQ pseudogene 8*	*YWHAQP8*	9.32	9.52	10.04	0.003137292	0.012966427
8069852	337966	*keratin associated protein 6-1*	*KRTAP6-1*	7.28	7.27	6.68	0.003138126	0.012968016
7949275	406970	*microRNA 194-2*	*MIR194-2*	6.94	6.99	6.44	0.003140107	0.012974344
8069037	326	*autoimmune regulator*	*AIRE*	7.92	7.98	7.33	0.003141607	0.012978686
8087433	84276, 275	*nicolin 1 | aminomethyltransferase*	*NICN1|AMT*	9.04	8.90	7.97	0.003142566	0.01298079
7984684				6.23	6.25	5.95	0.003146489	0.012995133
7982564	161742	*sprouty-related, EVH1 domain containing 1*	*SPRED1*	8.61	8.60	9.95	0.003147877	0.012999007
8177108	50858, 100101116, 60439, 100101117	*testis-specific transcript, Y-linked 1 (non-protein coding) | testis-specific transcript, Y-linked 1B (non-protein coding) | testis-specific transcript, Y-linked 2 (non-protein coding) | testis-specific transcript, Y-linked 2B (non-protein coding)*	*TTTY1|TTTY1B|TTTY2|TTTY2B*	6.28	6.28	5.82	0.003148643	0.013000312
7915910	10158	*PDZK1 interacting protein 1*	*PDZK1IP1*	7.60	7.53	6.79	0.003149782	0.013003156
8124859	29113	*chromosome 6 open reading frame 15*	*C6orf15*	6.99	6.99	6.34	0.003151059	0.013006567
8074780	29799, 23759	*yippee-like 1 (Drosophila) | peptidylprolyl isomerase (cyclophilin)-like 2*	*YPEL1|PPIL2*	6.94	7.08	7.90	0.003151911	0.013008224
7994237	51451	*leucine carboxyl methyltransferase 1*	*LCMT1*	9.91	9.80	10.57	0.0031524	0.013008379
8074061				10.06	10.11	9.28	0.00315449	0.013009569
8129454				7.03	7.39	6.33	0.003153632	0.013009569
8151540				9.95	10.07	9.21	0.00315448	0.013009569
8176373				5.05	5.13	4.89	0.003153866	0.013009569
8098877	100130872, 10417	*hypothetical LOC100130872 | spondin 2, extracellular matrix protein*	*LOC100130872|SPON2*	7.48	7.43	6.79	0.00315545	0.013010595

8007112	100132476, 100132386, 653240, 728224	*keratin associated protein 4-7 | keratin associated protein 4-9 | keratin associated protein 4-11 | keratin associated protein 4-8*	*KRTAP4-7|KRTAP4-9|KRTAP4-11|KRTAP 4-8*	8.65	8.62	7.91	0.003155641	0.013010595
8096959	287	*ankyrin 2, neuronal*	*ANK2*	6.79	7.34	8.35	0.003157653	0.013017033
8023977	3004	*granzyme M (lymphocyte met-ase 1)*	*GZMM*	7.70	7.62	7.15	0.003158137	0.013017169
7946860	6833	*ATP-binding cassette, sub-family C (CFTR/MRP), member 8*	*ABCC8*	7.08	7.06	6.73	0.003159935	0.013019004
8059854	10123	*ADP-ribosylation factor-like 4C*	*ARL4C*	7.23	8.37	7.79	0.003159917	0.013019004
8046099	115677	*nitric oxide synthase trafficker*	*NOSTRIN*	5.54	5.23	5.04	0.003159576	0.013019004
7978801	161357	*MAM domain containing glycosylphosphatidylinositol anchor 2*	*MDGA2*	5.57	5.53	6.49	0.003161046	0.013021723
8060020	401039		*FLJ43879*	6.32	6.44	5.96	0.003164491	0.013032196
8061138	57325, 100303755	*CSRP2 binding protein | cytochrome c oxidase assembly factor-like*	*CSRP2BP|PET117*	7.22	7.13	8.12	0.003164323	0.013032196
7951309	4322	*matrix metallopeptidase 13 (collagenase 3)*	*MMP13*	5.49	5.56	5.10	0.00316606	0.013036798
7935058	26509	*myoferlin*	*MYOF*	11.42	11.46	8.32	0.003167681	0.013041611
7937391	115399	*leucine rich repeat containing 56*	*LRRC56*	7.62	7.49	6.89	0.003170999	0.01305341
8070782	386680	*keratin associated protein 10-5*	*KRTAP10-5*	7.27	7.25	6.75	0.00317229	0.013055002
7918876				7.52	7.64	7.07	0.003171852	0.013055002
8100547				6.43	6.20	5.62	0.003176857	0.013071935
7947744	4038	*low density lipoprotein receptor-related protein 4*	*LRP4*	7.42	8.16	8.10	0.003178148	0.013072682
8092931				8.01	7.97	7.60	0.003178397	0.013072682
8162568				6.71	6.82	6.06	0.003177569	0.013072682
8059339				7.31	7.55	6.71	0.00317924	0.013074285
7927599	56624, 653308, 653365	*N-acylsphingosine amidohydrolase (non-lysosomal ceramidase) 2 | N-acylsphingosine amidohydrolase (non-lysosomal ceramidase) 2B | N-acylsphingosine amidohydrolase (non-lysosomal ceramidase) 2C*	*ASAH2|ASAH2B|ASAH2C*	7.14	8.00	9.62	0.003180492	0.01307757

8175288	56180	*motile sperm domain containing 1*	*MOSPD1*	7.32	7.51	8.37	0.003181585	0.0130802
8173926				5.72	5.95	5.41	0.003182759	0.013083165
7949364	113130	*cell division cycle associated 5*	*CDCA5*	6.80	7.30	9.58	0.003186252	0.013095658
8144481	1673	*defensin, beta 4A*	*DEFB4A*	5.91	5.91	5.43	0.003187514	0.013098573
8043480				5.90	6.08	6.35	0.003187869	0.013098573
8139330	816	*calcium/calmodulin-dependent protein kinase II beta*	*CAMK2B*	6.38	6.44	6.00	0.003189671	0.01310321
8089436				5.73	5.71	5.36	0.003190359	0.01310321
8121743				9.30	9.24	8.68	0.003190052	0.01310321
7956639	10102	*Ts translation elongation factor, mitochondrial*	*TSFM*	7.85	8.08	8.56	0.003191945	0.01310786
8004497	1973	*eukaryotic translation initiation factor 4A1*	*EIF4A1*	11.20	11.48	12.28	0.003196542	0.013123386
8163013				7.60	7.83	7.02	0.003196635	0.013123386
7971511				6.98	7.15	6.39	0.00319742	0.013124742
8083794	55892	*myoneurin*	*MYNN*	7.41	7.54	8.23	0.003198817	0.013128611
7984001	9245	*glucosaminyl (N-acetyl) transferase 3, mucin type*	*GCNT3*	6.79	6.74	6.11	0.003199864	0.013131039
7920182	353145	*late cornified envelope 3E*	*LCE3E*	6.66	6.84	6.52	0.003200915	0.013132082
7945418				9.29	9.43	8.13	0.003201027	0.013132082
7900510	1503	*CTP synthase*	*CTPS*	8.34	9.09	10.52	0.003203732	0.013139282
8030171	2512	*ferritin, light polypeptide*	*FTL*	13.65	13.41	13.01	0.003204148	0.013139282
7950753	60492	*coiled-coil domain containing 90B*	*CCDC90B*	8.28	8.67	9.23	0.003203364	0.013139282
8114068	134549	*shroom family member 1*	*SHROOM1*	8.62	8.75	7.63	0.003206842	0.013148462
8135172	80228	*ORAI calcium release-activated calcium modulator 2*	*ORAI2*	9.97	10.17	8.48	0.003207418	0.013148955
7905553	6699	*small proline-rich protein 1B*	*SPRR1B*	6.06	6.09	5.64	0.003208971	0.013153443
7966389	144717	*family with sequence similarity 109, member A*	*FAM109A*	8.06	8.06	7.32	0.003209424	0.013153443
8085164	9901	*SLIT-ROBO Rho GTPase activating protein 3*	*SRGAP3*	7.04	6.96	7.40	0.003212682	0.01316493
8081667	55032	*solute carrier family 35, member A5*	*SLC35A5*	9.19	8.94	10.13	0.00321718	0.01318149
8159036	11093	*ADAM metallopeptidase with thrombospondin type 1 motif, 13*	*ADAMTS13*	7.39	7.33	6.89	0.003222197	0.013191164
8026780	25796	*6-phosphogluconolactonase*	*PGLS*	9.42	9.78	7.82	0.003221756	0.013191164

7914630	55223	*tripartite motif-containing 62*	*TRIM62*	8.38	8.25	7.64	0.003220054	0.013191164
8015655	162427	*family with sequence similarity 134, member C*	*FAM134C*	10.52	10.49	9.63	0.003220978	0.013191164
8172415	6759, 548313	*synovial sarcoma, X breakpoint 4 | synovial sarcoma, X breakpoint 4B*	*SSX4|SSX4B*	5.87	5.89	6.57	0.003220936	0.013191164
8166948				9.99	10.50	8.95	0.003222283	0.013191164
8063729	1908	*endothelin 3*	*EDN3*	6.74	6.78	6.35	0.003223131	0.013192766
7985099	9051	*proline-serine-threonine phosphatase interacting protein 1*	*PSTPIP1*	6.83	6.84	6.49	0.003224965	0.013198399
7990165	56965	*poly (ADP-ribose) polymerase family, member 6*	*PARP6*	10.39	10.32	9.41	0.003226063	0.013201021
7944418	9854, 1798	*C2CD2-like | dolichyl-phosphate (UDP-N-acetylglucosamine) N-acetylglucosaminephosphotransferase 1 (GlcNAc-1-P transferase)*	*C2CD2L|DPAGT1*	8.42	8.59	7.23	0.003229896	0.013211088
7936994				5.79	6.04	5.36	0.003228991	0.013211088
8006319				7.14	7.49	6.71	0.003229552	0.013211088
8028389	399473	*sprouty-related, EVH1 domain containing 3*	*SPRED3*	7.52	7.76	7.18	0.00323118	0.013212596
8020027				6.69	6.74	6.26	0.0032308	0.013212596
8021113	147339	*chromosome 18 open reading frame 25*	*C18orf25*	6.73	6.68	7.97	0.003233683	0.013220224
7972921				6.53	6.58	7.43	0.003233961	0.013220224
8062557	26051	*protein phosphatase 1, regulatory (inhibitor) subunit 16B*	*PPP1R16B*	6.93	7.03	7.61	0.00323449	0.013220514
7984626	123228	*SUMO/sentrin specific peptidase family member 8*	*SENP8*	7.02	6.99	8.30	0.003235764	0.013223849
8129120	221303	*family with sequence similarity 162, member B*	*FAM162B*	10.40	9.17	9.68	0.003238114	0.013231579
8077204	345	*apolipoprotein C-III*	*APOC3*	6.89	7.00	6.45	0.00323988	0.013234332
7959473	8562	*density-regulated protein*	*DENR*	7.47	7.53	8.97	0.003240621	0.013234332
8000375	55114	*Rho GTPase activating protein 17*	*ARHGAP17*	10.03	9.81	8.98	0.003239444	0.013234332
8096919	80216	*alpha-kinase 1*	*ALPK1*	6.93	7.20	5.40	0.003240617	0.013234332
8053842	150763	*glycerol-3-phosphate acyltransferase 2, mitochondrial*	*GPAT2*	8.28	8.17	7.35	0.00324125	0.013235026
8132667	107	*adenylate cyclase 1 (brain)*	*ADCY1*	7.21	7.05	8.18	0.003242574	0.01323856
7941761	29984	*ras homolog gene family, member D*	*RHOD*	10.92	10.64	9.08	0.003245457	0.013248457

7916541	1600	*disabled homolog 1 (Drosophila)*	*DAB1*	6.50	8.73	8.19	0.003251297	0.013268543
8102988	2996. 2993	*glycophorin E (MNS blood group) | glycophorin A (MNS blood group)*	*GYPE|GYPA*	6.84	6.82	5.62	0.003251008	0.013268543
8176091	158960	*hypothetical protein BC009467*	*LOC158960*	8.27	8.14	7.62	0.003252044	0.01326972
8111814	6167, 619564	*ribosomal protein L37 | small nucleolar RNA, C/D box 72*	*RPL37|SNORD72*	7.28	7.30	7.93	0.003253032	0.013271872
7919384				6.60	6.57	6.03	0.003254001	0.013273953
7999279	51172	*N-acetylglucosamine-1-phosphodiester alpha-N-acetylglucosaminidase*	*NAGPA*	8.92	8.66	7.42	0.003255111	0.013274726
8031973	65996	*hypothetical LOC65996*	*MGC2752*	9.12	9.36	8.09	0.003254697	0.013274726
8015349	3880	*keratin 19*	*KRT19*	8.95	8.86	10.03	0.003255861	0.013275911
8027556	4037	*low density lipoprotein receptor-related protein 3*	*LRP3*	9.68	9.90	8.50	0.003258136	0.013277684
7929911	84445	*leucine zipper, putative tumor suppressor 2*	*LZTS2*	10.03	10.10	9.07	0.003257228	0.013277684
8053944	93082	*neuralized homolog 3 (Drosophila) pseudogene*	*NEURL3*	7.62	7.70	7.12	0.003257848	0.013277684
8019243	116729	*dysferlin interacting protein 1*	*DYSFIP1*	6.93	7.02	6.29	0.003257598	0.013277684
7917944	406928	*microRNA 137*	*MIR137*	5.60	6.31	5.14	0.003259603	0.013281789
7938750	494141	*mitochondrial carrier triple repeat 1 pseudogene*	*LOC494141*	6.51	6.60	6.24	0.00326188	0.013288167
8063547				6.19	6.20	5.74	0.003262089	0.013288167
7921936	4009	*LIM homeobox transcription factor 1, alpha*	*LMX1A*	6.77	6.90	6.37	0.00326352	0.01329212
7950764	1740	*discs, large homolog 2 (Drosophila)*	*DLG2*	5.75	5.81	6.52	0.00326666	0.013301158
8177046				8.19	8.61	9.44	0.003266574	0.013301158
8114861	51520	*leucyl-tRNA synthetase*	*LARS*	8.05	8.10	10.87	0.003269458	0.013310673
7920633	54344	*dolichyl-phosphate mannosyltransferase polypeptide 3*	*DPM3*	9.85	9.91	8.98	0.003270169	0.013311691
7943969	3359	*5-hydroxytryptamine (serotonin) receptor 3A*	*HTR3A*	6.82	6.83	7.89	0.003273212	0.013322201
8026456	4051	*cytochrome P450, family 4, subfamily F, polypeptide 3*	*CYP4F3*	5.97	6.07	5.57	0.003274157	0.013324165
8014008	642	*bleomycin hydrolase*	*BLMH*	8.27	8.31	9.70	0.003275689	0.013326642
8053057				5.61	5.43	6.13	0.003275479	0.013326642
8108205	26223	*F-box and leucine-rich repeat protein 21 (gene/pseudogene)*	*FBXL21*	5.20	5.16	5.49	0.003276166	0.013326705

8043512	7549	*zinc finger protein 2*	*ZNF2*	7.28	7.33	6.62	0.003278379	0.01333383
8052598	51057	*chromosome 2 open reading frame 86*	*C2orf86*	7.24	7.28	8.51	0.003279196	0.013335275
8151447	3574	*interleukin 7*	*IL7*	5.41	6.02	5.09	0.003281869	0.013339867
8126710	8464	*suppressor of Ty 3 homolog (S. cerevisiae)*	*SUPT3H*	7.58	7.66	9.04	0.003282972	0.013339867
7933537	55753	*oxoglutarate dehydrogenase-like*	*OGDHL*	6.68	6.72	7.43	0.003283098	0.013339867
7896917	80772	*glycolipid transfer protein domain containing 1*	*GLTPD1*	8.40	8.52	7.03	0.003282679	0.013339867
7927641				4.54	4.56	5.22	0.003282858	0.013339867
8055348				6.10	6.21	5.74	0.003281907	0.013339867
8035859				7.24	7.20	6.81	0.003284088	0.013342011
8029273	1954	*multiple EGF-like-domains 8*	*MEGF8*	7.65	7.51	6.74	0.003285769	0.013346963
8010260	332	*baculoviral IAP repeat-containing 5*	*BIRC5*	7.92	8.25	9.57	0.003287148	0.013348809
8099193	389197	*chromosome 4 open reading frame 50*	*C4orf50*	6.36	6.78	6.30	0.003287135	0.013348809
7986820				6.81	6.75	6.27	0.003287934	0.013350122
7952223				5.49	5.72	5.07	0.003289184	0.013353318
8125870	6954	*t-complex 11 homolog (mouse)*	*TCP11*	6.42	6.33	6.01	0.003290332	0.013354516
8037651	147912	*SIX homeobox 5*	*SIX5*	9.04	9.11	8.05	0.003290404	0.013354516
7984908	594855, 79748	*complexin 3 | lectin, mannose-binding, 1 like*	*CPLX3|LMAN1L*	6.88	6.91	6.27	0.003292852	0.013362573
8150126	5516	*protein phosphatase 2, catalytic subunit, beta isozyme*	*PPP2CB*	8.82	9.17	9.83	0.003297034	0.013371774
8124280	9750	*family with sequence similarity 65, member B*	*FAM65B*	6.45	7.66	8.72	0.003296122	0.013371774
8123728	57128	*LYR motif containing 4*	*LYRM4*	7.73	7.83	8.52	0.003297435	0.013371774
7950005	116535	*MAS-related GPR, member F*	*MRGPRF*	10.42	9.86	8.25	0.003296719	0.013371774
8093150				6.31	6.18	5.72	0.003297261	0.013371774
7984524	54852	*progestin and adipoQ receptor family member V*	*PAQR5*	6.31	6.43	7.24	0.003299901	0.013378015
7956038	4327, 440104	*matrix metallopeptidase 19 | 1110012D08Rik pseudogene*	*MMP19|LOC440104*	9.96	9.32	8.60	0.003299768	0.013378015
8021058	8170	*solute carrier family 14 (urea transporter), member 2*	*SLC14A2*	6.34	6.48	6.00	0.003300489	0.013378519
8065278	643659	*hCG2019139*	*LOC643659*	6.15	6.23	5.86	0.003301643	0.01338132
7997676	197196	*transmembrane protein 148*	*TMEM148*	6.55	6.72	6.27	0.003302434	0.013382646

8142747	5078	*paired box 4*	*PAX4*	7.02	7.14	6.75	0.003304686	0.013389893
8119444	116113	*forkhead box P4*	*FOXP4*	9.19	9.24	7.94	0.003305815	0.013390708
8168578	100131816	*ubiquitin-conjugating enzyme E2D N-terminal like (pseudogene)*	*UBE2DNL*	7.29	7.33	6.59	0.003305685	0.013390708
7918504	5016	*oviductal glycoprotein 1, 120kDa*	*OVGP1*	6.69	6.80	6.29	0.003306764	0.01339267
7902102				4.68	4.62	4.49	0.003308855	0.013399259
7905496	388699	*chromosome 1 open reading frame 46*	*C1orf46*	6.25	6.12	5.78	0.003310773	0.013405146
8096415	22915	*multimerin 1*	*MMRN1*	5.63	5.57	5.17	0.003311891	0.013405914
8039166	79143	*membrane bound O-acyltransferase domain containing 7*	*MBOAT7*	11.01	11.26	9.96	0.003311872	0.013405914
8112041				8.02	8.84	7.27	0.003312381	0.013406016
8076302	150356	*chondroadherin-like*	*CHADL*	8.20	8.10	7.43	0.003314924	0.01341443
8070173	3753	*potassium voltage-gated channel, Isk-related family, member 1*	*KCNE1*	7.36	7.11	6.63	0.003316165	0.013415317
7981387	9578	*CDC42 binding protein kinase beta (DMPK-like)*	*CDC42BPB*	10.76	10.75	10.10	0.003316103	0.013415317
8177191				7.13	7.20	6.62	0.003316538	0.013415317
8116504	81786	*tripartite motif-containing 7*	*TRIM7*	7.17	7.22	6.43	0.003318777	0.013422495
8039884	57292, 553128	*killer cell immunoglobulin-like receptor, two domains, long cytoplasmic tail, 5A | killer cell immunoglobulin-like receptor, two domains, long cytoplasmic tail, 5B*	*KIR2DL5A |KIR2DL5B*	6.51	6.56	5.86	0.003321806	0.013432862
8103894	133121	*ectonucleotide pyrophosphatase/phosphodiesterase 6*	*ENPP6*	5.65	5.79	5.34	0.003324648	0.013442473
7903239	178	*amylo-alpha-1, 6-glucosidase, 4-alpha-glucanotransferase*	*AGL*	7.30	6.86	8.61	0.003327843	0.013445338
8046020	6326	*sodium channel, voltage-gated, type II, alpha subunit*	*SCN2A*	5.26	5.28	5.59	0.003327581	0.013445338
7930980	196051	*phosphatidic acid phosphatase type 2 domain containing 1A*	*PPAPDC1A*	7.42	9.52	7.08	0.003328278	0.013445338
8041561	729967	*MORN repeat containing 2*	*MORN2*	9.85	9.73	8.84	0.003326374	0.013445338
8155514	100131997, 100289124, 100287333	*family with sequence similarity 27, member E3 | family with sequence similarity 27, member E2 | family with sequence similarity 27, member E1*	*FAM27E3|FAM27E2|FAM27E1*	8.43	8.98	7.26	0.003328397	0.013445338

8043363	112597, 541471	*non-protein coding RNA 152 | hypothetical LOC541471*	*NCRNA00 152|LOC5 41471*	11.29	11.60	10.17	0.003328617	0.013445338
7953098				5.30	5.30	4.86	0.003327178	0.013445338
8056823				5.23	5.28	4.78	0.003329492	0.013446991
7939056	8424	*butyrobetaine (gamma), 2-oxoglutarate dioxygenase (gamma-butyrobetaine hydroxylase) 1*	*BBOX1*	5.09	5.06	4.87	0.00333332	0.013460569
8032273				6.38	6.48	5.91	0.003334639	0.013464011
7936835	119587	*carboxypeptidase X (M14 family), member 2*	*CPXM2*	8.84	9.22	7.22	0.003335481	0.013465528
8139826	168474	*selenophosphate synthetase pseudogene*	*LOC16847 4*	6.28	6.13	6.89	0.003336227	0.013466187
8159646	441476	*chromosome 9 open reading frame 173*	*C9orf173*	7.33	7.40	6.90	0.003336577	0.013466187
7952475	220296, 641654	*hepatocyte cell adhesion molecule | HEPACAM opposite strand 1*	*HEPACAM |HEPN1*	6.45	6.37	5.91	0.003341884	0.01348572
8036840	208	*v-akt murine thymoma viral oncogene homolog 2*	*AKT2*	11.89	11.82	10.72	0.003342657	0.013486956
8161024	6023	*RNA component of mitochondrial RNA processing endoribonuclease*	*RMRP*	12.92	12.98	12.24	0.003344168	0.013490675
7983938	100289060	*guanine nucleotide binding protein (G protein), gamma 10 pseudogene*	*LOC10028 9060*	8.41	8.46	7.89	0.003344514	0.013490675
7933010	56288	*par-3 partitioning defective 3 homolog (C. elegans)*	*PARD3*	8.20	8.23	9.56	0.003346612	0.013495576
8112855				5.38	5.92	4.53	0.003346663	0.013495576
8106107	79810	*pentatricopeptide repeat domain 2*	*PTCD2*	7.33	7.15	8.67	0.003347869	0.013498551
8171052	3581	*interleukin 9 receptor*	*IL9R*	7.12	7.22	6.95	0.003350005	0.013504397
8026182	84245	*methylthioribose-1-phosphate isomerase homolog (S. cerevisiae)*	*MRI1*	9.44	9.26	8.54	0.003350254	0.013504397
7924499	7100	*toll-like receptor 5*	*TLR5*	5.95	5.96	5.69	0.0033516	0.013506048
8039645	400720	*zinc finger protein 772*	*ZNF772*	6.81	6.52	7.66	0.003351538	0.013506048
8026361				8.23	8.23	7.45	0.003357343	0.013527303
8068130	140258	*keratin associated protein 13-1*	*KRTAP13-1*	4.99	4.93	4.79	0.003363009	0.013548243
8047487	8324	*frizzled homolog 7 (Drosophila)*	*FZD7*	10.04	9.62	10.90	0.003364173	0.013550772
7951826	84811		*BUD13*	7.56	7.50	8.26	0.003364576	0.013550772

8019796	4097	*v-maf musculoaponeurotic fibrosarcoma oncogene homolog G (avian)*	*MAFG*	9.46	9.69	8.67	0.003366345	0.013552224
8135945	84691	*family with sequence similarity 71, member F1*	*FAM71F1*	6.29	6.27	7.90	0.003365661	0.013552224
7910377				7.77	8.11	6.71	0.003365983	0.013552224
8111153	4651	*myosin X*	*MYO10*	9.21	8.73	10.73	0.003369293	0.013553547
8019939	7050	*TGFB-induced factor homeobox 1*	*TGIF1*	8.13	8.21	8.92	0.003369686	0.013553547
8041360	55622	*tetratricopeptide repeat domain 27*	*TTC27*	7.66	7.33	9.13	0.003367579	0.013553547
7958532	89910	*ubiquitin protein ligase E3B*	*UBE3B*	10.07	9.96	8.96	0.003368204	0.013553547
8130422	154043	*CNKSR family member 3*	*CNKSR3*	8.85	7.17	6.83	0.003369817	0.013553547
8175269	159090	*family with sequence similarity 122B*	*FAM122B*	7.70	7.59	8.29	0.003370429	0.013553547
7957478	160418	*transmembrane and tetratricopeptide repeat containing 3*	*TMTC3*	10.16	9.92	11.02	0.003370032	0.013553547
8148748	340390		*KIAA1875*	7.02	6.95	6.54	0.003368916	0.013553547
7977340	90135, 2972	*BTB (POZ) domain containing 6 | BRF1 homolog, subunit of RNA polymerase III transcription initiation factor IIIB (S. cerevisiae)*	*BTBD6|BRF1*	9.02	9.28	7.66	0.00337143	0.013555684
7978666	51562	*MAP3K12 binding inhibitory protein 1*	*MBIP*	9.73	9.76	10.86	0.003373359	0.013560359
8101224				7.80	8.09	8.58	0.003373532	0.013560359
7928909	54537	*family with sequence similarity 35, member A*	*FAM35A*	8.03	8.09	9.12	0.003374999	0.013564365
7974447	122809	*suppressor of cytokine signaling 4*	*SOCS4*	6.87	6.94	8.94	0.003375956	0.013564437
7938170	144125	*olfactory receptor, family 2, subfamily AG, member 1*	*OR2AG1*	6.29	6.21	5.84	0.003375887	0.013564437
8095402	79799	*UDP glucuronosyltransferase 2 family, polypeptide A3*	*UGT2A3*	5.27	5.02	4.95	0.003377309	0.013567985
7920707	10712	*family with sequence similarity 189, member B*	*FAM189B*	9.75	9.63	8.63	0.003377816	0.013568134
7942798	57558	*ubiquitin specific peptidase 35*	*USP35*	8.45	8.27	7.38	0.003379255	0.013572024
7991860	9727	*RAB11 family interacting protein 3 (class II)*	*RAB11FIP3*	8.84	8.81	7.94	0.003380568	0.013575409
7898249	284723	*solute carrier family 25, member 34*	*SLC25A34*	7.29	7.35	6.70	0.003381104	0.013575671
8013622	124923	*uncharacterized serine/threonine-protein kinase SgK494*	*SGK494*	6.92	6.75	7.91	0.003382594	0.013579767
8020267	55125	*centrosomal protein 192kDa*	*CEP192*	7.43	7.17	9.07	0.003386556	0.013593781

8041696	64241	*ATP-binding cassette, sub-family G (WHITE), member 8*	*ABCG8*	6.35	6.47	6.02	0.003389004	0.013601717
8083030				8.63	8.67	7.60	0.003389518	0.013601891
7944739	56253	*cytotoxic and regulatory T cell molecule*	*CRTAM*	5.57	5.54	5.24	0.003391064	0.013606202
8006786	57636	*Rho GTPase activating protein 23*	*ARHGAP23*	9.04	9.04	7.49	0.003393968	0.01361596
8082552	6010	*rhodopsin*	*RHO*	6.54	6.76	6.11	0.003398547	0.013630545
8039759	162968, 1	*zinc finger protein 497 | alpha-1-B glycoprotein*	*ZNF497|A1BG*	7.86	7.99	7.14	0.003398351	0.013630545
7916562	3178, 729423	*heterogeneous nuclear ribonucleoprotein A1 | heterogeneous nuclear ribonucleoprotein A1 pseudogene 6*	*HNRNPA1|HNRNPA1P6*	10.65	10.39	12.85	0.003402545	0.013644683
8090772	11073	*topoisomerase (DNA) II binding protein 1*	*TOPBP1*	8.36	8.24	10.50	0.003404134	0.013647271
8038962	162962	*zinc finger protein 836*	*ZNF836*	7.13	6.96	8.12	0.003404609	0.013647271
7955863	406973	*microRNA 196a-2*	*MIR196A2*	5.75	6.98	5.57	0.00340455	0.013647271
7935855	10660	*ladybird homeobox 1*	*LBX1*	7.86	8.07	7.19	0.003406704	0.013653776
8005399	54890	*alkB, alkylation repair homolog 5 (E. coli)*	*ALKBH5*	10.36	10.46	9.40	0.003409314	0.013662339
8012958	27338	*ubiquitin-conjugating enzyme E2S*	*UBE2S*	8.51	8.94	10.22	0.003411895	0.013670784
8027448	147991	*dpy-19-like 3 (C. elegans)*	*DPY19L3*	7.47	7.19	8.94	0.003413663	0.013675971
8151496	619279	*zinc finger protein 704*	*ZNF704*	6.14	6.54	7.16	0.003416917	0.013685206
7912861	644634	*UPF0627 protein ENSP00000358171-like*	*LOC644634*	6.77	6.76	6.41	0.00341691	0.013685206
7958425	1610	*D-amino-acid oxidase*	*DAO*	5.96	5.92	5.57	0.003417691	0.013686296
8078729	4615	*myeloid differentiation primary response gene (88)*	*MYD88*	11.02	10.60	9.83	0.003418137	0.013686296
7907058	5451	*POU class 2 homeobox 1*	*POU2F1*	8.55	8.33	10.94	0.003419269	0.01368893
7911506	1855	*dishevelled, dsh homolog 1 (Drosophila)*	*DVL1*	9.44	9.54	8.67	0.003420869	0.013690326
8078690	9940	*deleted in lung and esophageal cancer 1*	*DLEC1*	6.75	6.79	6.49	0.003420512	0.013690326
8098848	10861, 3425	*solute carrier family 26 (sulfate transporter), member 1 | iduronidase, alpha-L-*	*SLC26A1|IDUA*	7.18	7.20	6.61	0.003421989	0.013690326
8061169				7.07	7.08	6.32	0.003421641	0.013690326
8122259				7.19	7.47	6.77	0.003421402	0.013690326

7970033	1284	*collagen, type IV, alpha 2*	*COL4A2*	10.36	10.21	8.66	0.003424209	0.013697311
8139697				5.96	5.96	5.68	0.00342959	0.013716933
8031865				8.59	8.61	8.26	0.003430706	0.013719498
7927732	84159	*AT rich interactive domain 5B (MRF1-like)*	*ARID5B*	10.84	10.17	7.55	0.003433762	0.013728655
8041540				5.22	5.14	4.92	0.003433947	0.013728655
8077042	6305	*SET binding factor 1*	*SBF1*	9.79	9.79	8.34	0.003435394	0.013732539
8107887	1437	*colony stimulating factor 2 (granulocyte-macrophage)*	*CSF2*	5.77	5.75	5.27	0.003437095	0.013737435
7938008	390066	*olfactory receptor, family 52, subfamily D, member 1*	*OR52D1*	6.14	6.20	5.77	0.00343921	0.013743986
7985240	23423	*transmembrane emp24 protein transport domain containing 3*	*TMED3*	10.74	10.67	9.44	0.003439696	0.013744026
8004428	643664, 83650, 146861, 646000	*acyl-malonyl condensing enzyme 1-like 3 | acyl-malonyl condensing enzyme 1-like 2 | acyl-malonyl condensing enzyme 1 | acyl-malonyl condensing enzyme 1-like 1*	*AMAC1L3|AMAC1L2|AMAC1L1*	7.36	7.36	6.78	0.003440657	0.013745962
7911309	653635, 374666, 375260, 100287171, 375690, 100288778, 100134445	*WAS protein family homolog 7 pseudogene | WAS protein family homolog 3 pseudogene | WAS protein family homolog 2 pseudogene | WAS protein family homolog 1 | WAS protein family homolog 5 pseudogene | WAS protein family homolog 1 pseudogene | hypothetical LOC100134445*	*WASH7P|WASH3P|WASH2P|WASH1|WASH5P|LOC100288778|LOC100134445*	8.98	8.80	8.25	0.003442447	0.013751211
8071877	29774	*POM121 membrane glycoprotein-like 9, pseudogene*	*POM121L9P*	7.41	7.35	6.79	0.003443428	0.013751322
7910503	440730	*tripartite motif-containing 67*	*TRIM67*	7.02	7.08	6.46	0.003442985	0.013751322
7901951	5236	*phosphoglucomutase 1*	*PGM1*	11.20	11.27	9.94	0.003445947	0.013759481
8016016				7.99	8.13	7.74	0.003446462	0.013759634
8173444	3561	*interleukin 2 receptor, gamma*	*IL2RG*	6.37	6.24	5.93	0.003448193	0.013764643
7925523				7.51	7.42	8.91	0.003449876	0.013769455
7998749	283870, 64223	*chromosome 16 open reading frame 79 | MTOR associated protein, LST8 homolog (S. cerevisiae)*	*C16orf79|MLST8*	8.24	8.26	7.52	0.003450431	0.013769768
8012528	388333	*speedy homolog E4 (Xenopus laevis)*	*SPDYE4*	6.15	6.24	5.67	0.003451674	0.013771192
8077511				5.42	5.67	5.12	0.003451742	0.013771192

7974146	55015	*PRP39 pre-mRNA processing factor 39 homolog (S. cerevisiae)*	*PRPF39*	6.69	6.42	8.22	0.003452438	0.013772063
8062527	149685	*adipogenin*	*ADIG*	7.13	7.02	6.56	0.003456896	0.013787942
8056491	6335	*sodium channel, voltage-gated, type IX, alpha subunit*	*SCN9A*	9.21	6.77	6.78	0.00345879	0.013793466
8118111	10866	*HLA complex P5*	*HCP5*	6.58	6.19	7.18	0.003460192	0.013793466
8006504	54752, 54475	*fibronectin type III domain containing 8 | notchless homolog 1 (Drosophila)*	*FNDC8|NLE1*	6.38	6.58	5.87	0.003459964	0.013793466
7986336				6.87	6.94	6.34	0.003459277	0.013793466
8033190	79085	*solute carrier family 25 (mitochondrial carrier; phosphate carrier), member 23*	*SLC25A23*	9.78	9.39	8.71	0.003463214	0.013803606
7903884	84432	*prokineticin 1*	*PROK1*	6.44	6.43	5.94	0.003465298	0.013808388
8157231				5.61	5.50	6.45	0.00346537	0.013808388
7973902	6729	*signal recognition particle 54kDa*	*SRP54*	8.46	8.23	9.97	0.003466432	0.013808807
7981787	283767, 727832, 100132025, 645202, 440243	*golgin A6 family-like 1 | golgin A6 family-like 6 | transmembrane domain-containing protein ENSP00000320207-like | Putative golgin subfamily A member 6-like protein 6*	*GOLGA6L1|GOLGA6L6|LOC100132025|LOC645202|LOC440243*	5.93	5.93	5.64	0.003466339	0.013808807
8092222				6.11	6.01	5.60	0.003477025	0.013849094
8109326	85027	*chromosome 5 open reading frame 62*	*C5orf62*	7.84	8.56	7.41	0.003478428	0.013851072
8169808	100130613	*chromosome X open reading frame 64*	*CXorf64*	6.02	6.22	5.68	0.003478481	0.013851072
7995868	6559	*solute carrier family 12 (sodium/chloride transporters), member 3*	*SLC12A3*	6.84	7.06	6.46	0.003480172	0.013852966
7927767	84890	*2-aminoethanethiol (cysteamine) dioxygenase*	*ADO*	8.95	9.23	9.38	0.003479989	0.013852966
8150217	728024	*chromosome X open reading frame 56 pseudogene*	*LOC728024*	7.74	7.69	8.40	0.003480397	0.013852966
8018902	8632	*dynein, axonemal, heavy chain 17*	*DNAH17*	6.21	6.40	5.93	0.003480917	0.013853128
8008289	162461	*transmembrane protein 92*	*TMEM92*	7.66	7.82	8.50	0.003482539	0.013857671
8022745				4.63	4.65	4.48	0.003483656	0.013860208
8170288				5.82	5.95	5.43	0.003484354	0.013861073

8019532	10966		*RAB40B*	8.51	8.48	7.79	0.003488031	0.013873789
7940409	921		*CD5*	6.88	7.01	6.40	0.003491552	0.013874413
7964413	22864	*R3H domain containing 2*	*R3HDM2*	10.27	10.13	8.95	0.003491311	0.013874413
8039526	84922	*FLT3-interacting zinc finger 1*	*FIZ1*	8.70	8.83	7.94	0.003489397	0.013874413
8130720	113402	*SFT2 domain containing 1*	*SFT2D1*	7.96	8.19	9.03	0.0034908	0.013874413
8160472	286319	*tumor suppressor candidate 1*	*TUSC1*	7.67	7.68	7.08	0.003491525	0.013874413
8172749	9502, 728242	*X antigen family, member 2 | X antigen family, member 2B*	*XAGE2|XAGE2B*	6.02	5.94	5.56	0.003489449	0.013874413
8172717				4.89	4.95	4.78	0.003491377	0.013874413
8028112	79414	*leucine rich repeat and fibronectin type III domain containing 3*	*LRFN3*	8.22	8.25	7.14	0.003495421	0.013887875
7902317	51086	*TNNI3 interacting kinase*	*TNNI3K*	5.25	5.17	5.01	0.003496752	0.013889341
7984736	283677	*chromosome 15 open reading frame 60*	*C15orf60*	5.47	5.39	5.16	0.003496368	0.013889341
7958346	90488	*chromosome 12 open reading frame 23*	*C12orf23*	9.88	9.64	8.39	0.003498901	0.013894053
7950195	406931	*microRNA 139*	*MIR139*	8.83	8.81	8.19	0.00349876	0.013894053
7955613	3855	*keratin 7*	*KRT7*	9.95	10.58	7.46	0.003501987	0.013904397
8110562	4056, 9794	*leukotriene C4 synthase | mastermind-like 1 (Drosophila)*	*LTC4S|MAML1*	8.50	8.48	7.53	0.003502915	0.013904949
7934810				6.34	6.51	6.01	0.00350309	0.013904949
8063019	391253	*serine peptidase inhibitor, Kunitz type 4*	*SPINT4*	4.86	4.83	4.69	0.003504031	0.013906774
8059864	2637	*gastrulation brain homeobox 2*	*GBX2*	8.76	8.83	8.13	0.003505355	0.013908472
7961111	51267	*C-type lectin domain family 1, member A*	*CLEC1A*	5.10	5.21	5.00	0.003505423	0.013908472
8021653	5271	*serpin peptidase inhibitor, clade B (ovalbumin), member 8*	*SERPINB8*	8.28	9.17	6.86	0.003508143	0.013915443
8065321				5.28	5.17	4.90	0.003508014	0.013915443
8144577	203076	*chromosome 8 open reading frame 74*	*C8orf74*	6.97	7.21	6.42	0.003508864	0.013916389
8000799	79153	*glycerophosphodiester phosphodiesterase domain containing 3*	*GDPD3*	8.03	7.34	6.41	0.003510152	0.013919585
8130628	56895	*1-acylglycerol-3-phosphate O-acyltransferase 4 (lysophosphatidic acid acyltransferase, delta)*	*AGPAT4*	7.95	7.88	8.54	0.003513826	0.013932242
7912412	2475	*mechanistic target of rapamycin (serine/threonine kinase)*	*MTOR*	9.15	9.08	10.24	0.003517343	0.013938526

7932637	22852	*ankyrin repeat domain 26*	*ANKRD26*	6.24	6.07	7.23	0.003516557	0.013938526
7946742	120227	*cytochrome P450, family 2, subfamily R, polypeptide 1*	*CYP2R1*	7.55	7.78	9.16	0.003517317	0.013938526
8049195				8.37	8.44	7.72	0.003516163	0.013938526
8090770				7.94	7.89	7.26	0.00351915	0.013941861
8176667				7.13	7.20	6.63	0.003518866	0.013941861
8144317	9920	*kelch repeat and BTB (POZ) domain containing 11*	*KBTBD11*	7.47	7.39	6.69	0.003521656	0.013949459
8090395	114112, 645840	*thioredoxin reductase 3 | thioredoxin reductase 3 intronic transcript 1*	*TXNRD3|TXNRD3IT1*	7.39	7.22	6.75	0.003522034	0.013949459
7932826	57608		*KIAA1462*	8.20	9.24	6.69	0.003523882	0.013954862
7902038	205	*adenylate kinase 4*	*AK4*	8.37	8.69	8.00	0.003524509	0.013955432
7996264	1003	*cadherin 5, type 2 (vascular endothelium)*	*CDH5*	6.56	6.60	6.04	0.003527271	0.013964453
7975876	2103	*estrogen-related receptor beta*	*ESRRB*	6.22	6.14	5.93	0.003529758	0.01397093
8039044	55786	*zinc finger protein 415*	*ZNF415*	7.78	7.44	8.09	0.003530493	0.01397093
7957386	79611	*acyl-CoA synthetase short-chain family member 3*	*ACSS3*	8.25	7.17	9.36	0.003530843	0.01397093
7997032	497190, 283971, 348174	*C-type lectin domain family 18, member B | C-type lectin domain family 18, member C | C-type lectin domain family 18, member A*	*CLEC18B|CLEC18C|CLEC18A*	7.14	7.20	6.70	0.003529904	0.01397093
8006573	8148		*TAF15*	8.41	8.76	10.32	0.00353657	0.013987278
8039491	27338	*ubiquitin-conjugating enzyme E2S*	*UBE2S*	8.47	8.92	10.04	0.003536912	0.013987278
7995338				10.05	10.04	9.35	0.003536265	0.013987278
8175568				6.62	6.71	5.83	0.00353594	0.013987278
7955211	79962	*DnaJ (Hsp40) homolog, subfamily C, member 22*	*DNAJC22*	6.41	6.23	5.78	0.003539066	0.013991961
7918847	80143	*suppressor of IKBKE 1*	*SIKE1*	7.92	7.88	9.80	0.003538942	0.013991961
8064451	284759	*signal-regulatory protein beta 2*	*SIRPB2*	6.14	6.16	5.71	0.003540115	0.01399419
8162231	10507	*sema domain, immunoglobulin domain (Ig), transmembrane domain (TM) and short cytoplasmic domain, (semaphorin) 4D*	*SEMA4D*	7.04	6.81	6.43	0.003540892	0.013995345
7991809	398, 64714	*Rho GDP dissociation inhibitor (GDI) gamma | protein disulfide isomerase family A, member 2*	*ARHGDIG |PDIA2*	7.56	7.84	7.15	0.003542805	0.01400099

8070716	100129890	*similar to hCG1750329*	*LOC100129890*	7.07	7.10	6.46	0.00354414	0.014004349
8048733	2181	*acyl-CoA synthetase long-chain family member 3*	*ACSL3*	8.31	7.75	9.78	0.003545828	0.0140091
8160168	158326	*FRAS1 related extracellular matrix 1*	*FREM1*	6.08	5.96	6.68	0.003546629	0.01401035
8064613	83959	*solute carrier family 4, sodium borate transporter, member 11*	*SLC4A11*	8.98	8.85	10.09	0.003549069	0.01401807
7907396	92346	*chromosome 1 open reading frame 105*	*C1orf105*	5.43	5.41	5.17	0.003549964	0.014019685
8090277	114885	*oxysterol binding protein-like 11*	*OSBPL11*	6.96	6.91	8.45	0.003550943	0.014021633
7996256				7.91	8.01	7.65	0.003552094	0.014024263
7982527	54989	*zinc finger protein 770*	*ZNF770*	5.84	5.74	6.35	0.003553225	0.014026808
8099860	5981	*replication factor C (activator 1) 1, 145kDa*	*RFC1*	7.96	7.67	10.54	0.003554125	0.014028445
7985898	56964	*WD repeat domain 93*	*WDR93*	5.93	5.88	5.52	0.003557046	0.014038055
8142345	9732	*dedicator of cytokinesis 4*	*DOCK4*	8.23	6.48	8.62	0.003558658	0.014042496
8093230	9711		*KIAA0226*	8.57	8.87	7.97	0.003559749	0.014044882
8088384	5162	*pyruvate dehydrogenase (lipoamide) beta*	*PDHB*	8.44	8.88	10.24	0.003561902	0.014049535
7986536	100272148		*GPCRLTM7*	5.45	5.37	5.34	0.003561589	0.014049535
8141180	25851	*tectonin beta-propeller repeat containing 1*	*TECPR1*	7.59	7.70	6.67	0.003563093	0.014052311
8128865				7.97	8.16	7.25	0.003563672	0.014052678
8111666				5.31	4.86	6.12	0.003566939	0.014063639
8155440	100036519, 349334, 653404, 653427, 200350, 286380, 2298	*forkhead box D4-like 2 | forkhead box D4-like 4 | forkhead box D4-like 6 | forkhead box D4-like 5 | forkhead box D4-like 1 | forkhead box D4-like 3 | forkhead box D4*	*FOXD4L2|FOXD4L4|FOXD4L6|FOXD4L5|FOXD4L1|FOXD4L3*	7.73	7.78	7.39	0.00357088	0.014077255
8063473	6136	*ribosomal protein L12*	*RPL12*	12.91	12.87	13.03	0.003574712	0.014088514
8170260	139081	*melanoma antigen family C, 3*	*MAGEC3*	6.07	5.99	5.60	0.003574258	0.014088514
8066754	128506	*chromosome 20 open reading frame 123*	*C20orf123*	7.15	7.16	6.75	0.003581464	0.01411127
8031799	201516	*zinc finger and SCAN domain containing 4*	*ZSCAN4*	5.05	5.05	4.94	0.003581215	0.01411127

7993458	89927	*chromosome 16 open reading frame 45*	*C16orf45*	9.95	9.87	8.71	0.003583609	0.014113945
8106006	6606, 6607	*survival of motor neuron 1, telomeric | survival of motor neuron 2, centromeric*	*SMN1|SMN2*	8.63	9.09	10.36	0.003582813	0.014113945
8023462				5.13	5.75	4.93	0.003583533	0.014113945
8031489	89887	*zinc finger protein 628*	*ZNF628*	8.20	8.39	7.50	0.0035845	0.014115529
8100977	6374	*chemokine (C-X-C motif) ligand 5*	*CXCL5*	7.11	7.07	7.76	0.003585004	0.014115587
7941587	254263	*cornichon homolog 2 (Drosophila)*	*CNIH2*	7.90	8.00	9.25	0.003586215	0.014118428
8082465	79825	*coiled-coil domain containing 48*	*CCDC48*	8.40	8.39	7.78	0.003587378	0.014121084
8044760				7.47	7.42	6.89	0.003590281	0.014130582
7938519	84953	*MICAL C-terminal like*	*MICALCL*	6.04	6.22	5.80	0.003591962	0.014135269
7915261	54802	*tRNA isopentenyltransferase 1*	*TRIT1*	7.81	8.07	9.57	0.003594617	0.014141863
7962829	677794	*small nucleolar RNA, H/ACA box 2B*	*SNORA2B*	4.82	4.73	5.60	0.003594442	0.014141863
8114797	81848	*sprouty homolog 4 (Drosophila)*	*SPRY4*	8.17	8.33	9.68	0.003597262	0.014150342
7911371	84808	*chromosome 1 open reading frame 170*	*C1orf170*	7.20	7.26	6.61	0.003601523	0.014163597
8173208	139886	*spindlin family, member 4*	*SPIN4*	8.47	8.13	9.61	0.003601613	0.014163597
7979743	51109	*retinol dehydrogenase 11 (all-trans/9-cis/11-cis)*	*RDH11*	8.29	8.71	10.52	0.003603324	0.014168397
8065359	22918		*CD93*	6.20	6.18	5.73	0.003609088	0.014185265
8001163	91807	*myosin light chain kinase 3*	*MYLK3*	7.17	7.20	6.68	0.003608769	0.014185265
8036309	284406	*zinc finger protein 82 homolog (mouse)*	*ZFP82*	7.07	6.80	8.42	0.003608612	0.014185265
7982333	100499221		*LOC100499221*	6.48	6.58	6.20	0.003610243	0.014187873
8039791	84878	*zinc finger and BTB domain containing 45*	*ZBTB45*	9.43	9.53	8.15	0.003611723	0.014191755
8075720	23780	*apolipoprotein L, 2*	*APOL2*	8.02	7.55	7.01	0.003614447	0.014199631
8019565	83755, 85289, 85291, 728224	*keratin associated protein 4-12 | keratin associated protein 4-5 | keratin associated protein 4-2 | keratin associated protein 4-8*	*KRTAP4-12|KRTAP4-5|KRTAP4-2|KRTAP4-8*	8.06	8.55	7.50	0.003615182	0.014199631
8077526				9.74	9.90	9.09	0.003615203	0.014199631

7975976	10598	*AHA1, activator of heat shock 90kDa protein ATPase homolog 1 (yeast)*	*AHSA1*	9.39	9.26	10.32	0.003616183	0.01420155
7957221	29953, 283392	*thyrotropin-releasing hormone degrading enzyme | hypothetical LOC283392*	*TRHDE|LOC283392*	8.56	6.05	7.48	0.003616739	0.014201802
8175492	286410	*ATPase, class VI, type 11C*	*ATP11C*	7.63	7.50	9.55	0.003619366	0.014210182
7947270	3739	*potassium voltage-gated channel, shaker-related subfamily, member 4*	*KCNA4*	6.35	6.43	6.04	0.003622992	0.014222486
7976681	10858	*cytochrome P450, family 46, subfamily A, polypeptide 1*	*CYP46A1*	7.51	7.56	6.98	0.003623639	0.01422309
7964660	552	*arginine vasopressin receptor 1A*	*AVPR1A*	6.12	6.06	5.70	0.003624908	0.014226137
8144701	100131581		*LOC100131581*	5.51	5.34	6.46	0.003629519	0.014242299
7926609	648		*BMI1*	8.81	8.97	7.94	0.00363204	0.014247301
8025868	126075	*coiled-coil domain containing 159*	*CCDC159*	9.01	8.79	7.59	0.003632175	0.014247301
8179028	554223		*LOC554223*	8.24	8.47	7.28	0.003632275	0.014247301
8094556	55276	*phosphoglucomutase 2*	*PGM2*	8.12	8.29	9.79	0.003633033	0.01424834
7917199	79739	*tubulin tyrosine ligase-like family, member 7*	*TTLL7*	6.01	6.56	7.16	0.00363794	0.014263711
8006227				4.91	4.95	4.66	0.003637762	0.014263711
8037788	147906	*dapper, antagonist of beta-catenin, homolog 3 (Xenopus laevis)*	*DACT3*	8.85	8.78	7.80	0.003640252	0.014270837
8174937	10178	*odz, odd Oz/ten-m homolog 1(Drosophila)*	*ODZ1*	5.85	5.93	6.67	0.003641988	0.014275702
8123080	94120	*synaptotagmin-like 3*	*SYTL3*	6.80	7.17	6.14	0.003643569	0.014278022
8025984	284391	*zinc finger protein 844*	*ZNF844*	6.73	6.48	7.65	0.003643201	0.014278022
7955535	91	*activin A receptor, type IB*	*ACVR1B*	8.57	8.74	8.31	0.003644074	0.014278063
8062444	671	*bactericidal/permeability-increasing protein*	*BPI*	5.83	5.77	5.33	0.003645549	0.014279965
8113064	116068	*LysM, putative peptidoglycan-binding, domain containing 3*	*LYSMD3*	8.97	8.83	9.61	0.003645228	0.014279965
7925972	810	*calmodulin-like 3*	*CALML3*	6.91	6.93	6.37	0.003646269	0.014280848
7977234	400258	*chromosome 14 open reading frame 180*	*C14orf180*	8.18	8.13	7.57	0.003647866	0.014283229

8161583	100036519, 349334, 653404, 653427, 200350, 286380, 2298	*forkhead box D4-like 2 | forkhead box D4-like 4 | forkhead box D4-like 6 | forkhead box D4-like 5 | forkhead box D4-like 1 | forkhead box D4-like 3 | forkhead box D4*	*FOXD4L2| FOXD4L4| FOXD4L6| FOXD4L5| FOXD4L1| FOXD4L3*	7.73	7.78	7.39	0.003647759	0.014283229
8155327	219	*aldehyde dehydrogenase 1 family, member B1*	*ALDH1B1*	10.42	11.43	11.44	0.003651161	0.014286444
8116484	4245	*mannosyl (alpha-1,3-)-glycoprotein beta-1,2-N-acetylglucosaminyltransferase*	*MGAT1*	8.39	8.27	7.33	0.003649824	0.014286444
8158568	10868	*ubiquitin specific peptidase 20*	*USP20*	8.32	8.15	7.19	0.003650658	0.014286444
8019010	57332	*chromobox homolog 8*	*CBX8*	8.05	7.98	7.34	0.003649491	0.014286444
7938561				5.54	5.54	5.29	0.003650794	0.014286444
7944493	867	*Cas-Br-M (murine) ecotropic retroviral transforming sequence*	*CBL*	8.82	8.98	10.61	0.003653188	0.014291194
8177176				5.32	5.34	5.11	0.003653366	0.014291194
8072216	10634	*growth arrest-specific 2 like 1*	*GAS2L1*	8.73	8.75	7.44	0.003655895	0.014299152
8175696	2556	*gamma-aminobutyric acid (GABA) A receptor, alpha 3*	*GABRA3*	5.81	5.74	8.00	0.00366231	0.0143223
8077116	440836	*outer dense fiber of sperm tails 3B*	*ODF3B*	7.81	7.72	6.87	0.003663071	0.014323335
7913138	246181	*aldo-keto reductase family 7-like*	*AKR7L*	6.01	5.99	5.60	0.003663778	0.014324161
8037123	5452	*POU class 2 homeobox 2*	*POU2F2*	8.00	8.11	7.43	0.003666017	0.014327718
7952768	399979	*sorting nexin 19*	*SNX19*	9.63	9.56	8.41	0.003665452	0.014327718
8132690				7.51	7.43	8.92	0.003666673	0.014327718
8173671				6.37	6.52	5.73	0.003666194	0.014327718
8128394	25957	*splicing factor, arginine/serine-rich 18*	*SFRS18*	7.83	7.34	10.19	0.003667857	0.014328466
8080578	55799	*calcium channel, voltage-dependent, alpha 2/delta subunit 3*	*CACNA2D 3*	6.54	6.28	7.09	0.003667827	0.014328466
7990827	100133746		*LOC10013 3746*	6.68	6.43	6.17	0.003668563	0.014329284
7962238				6.07	6.29	5.71	0.003670618	0.014335372
8040034				7.08	6.99	6.77	0.003672774	0.01434185
7960370	84766	*EF-hand calcium binding domain 4B*	*EFCAB4B*	7.35	7.13	6.72	0.003675896	0.014350159

8023948	91978	*chromosome 19 open reading frame 20*	*C19orf20*	8.53	8.62	7.97	0.003675656	0.014350159
7978208	26277	*TERF1 (TRF1)-interacting nuclear factor 2*	*TINF2*	10.25	10.26	8.94	0.003679061	0.014356692
8124588	257202	*glutathione peroxidase 6 (olfactory)*	*GPX6*	5.76	5.73	5.37	0.003678236	0.014356692
8099233	100133131		*LOC100133131*	7.10	7.00	6.65	0.003678708	0.014356692
8175732	4101, 266740	*melanoma antigen family A, 2 | melanoma antigen family A, 2B*	*MAGEA2|MAGEA2B*	6.82	6.87	6.38	0.003679669	0.014357125
8117389	8344	*histone cluster 1, H2be*	*HIST1H2BE*	6.09	6.29	7.90	0.003681693	0.014363079
7976844	406924	*microRNA 134*	*MIR134*	7.90	7.60	7.05	0.003682191	0.01436308
7960280	144699	*F-box and leucine-rich repeat protein 14*	*FBXL14*	8.09	8.28	7.71	0.003684231	0.014369099
7996934	51388	*nuclear import 7 homolog (S. cerevisiae)*	*NIP7*	8.40	9.18	10.72	0.003684959	0.014369995
8125798	4295	*motilin*	*MLN*	7.39	7.38	6.78	0.003685681	0.014370869
8098654	3818	*kallikrein B, plasma (Fletcher factor) 1*	*KLKB1*	5.67	5.65	7.42	0.003686638	0.014370971
7992632	4917	*netrin 3*	*NTN3*	7.80	7.86	7.25	0.003686703	0.014370971
8004152	23302	*WSC domain containing 1*	*WSCD1*	7.01	6.88	7.43	0.003687457	0.014371972
8042537	525	*ATPase, H+ transporting, lysosomal 56/58kDa, V1 subunit B1*	*ATP6V1B1*	7.38	7.56	6.72	0.003688198	0.014372918
7927056				4.98	5.04	4.85	0.003689746	0.014377012
7923027	51022	*glutaredoxin 2*	*GLRX2*	7.25	7.57	7.08	0.003691227	0.01438084
8134814	29992	*paired immunoglobin-like type 2 receptor alpha*	*PILRA*	6.64	6.68	6.07	0.00369514	0.014394145
8099670	91050	*coiled-coil domain containing 149*	*CCDC149*	7.85	7.54	6.83	0.003696039	0.014395703
8012140	51087	*Y box binding protein 2*	*YBX2*	8.27	8.38	8.96	0.003706179	0.014430157
8044844	80775	*transmembrane protein 177*	*TMEM177*	8.49	8.53	9.84	0.003706385	0.014430157
8001271	85320, 83752	*ATP-binding cassette, sub-family C (CFTR/MRP), member 11 | lon peptidase 2, peroxisomal*	*ABCC11|LONP2*	6.27	6.49	6.12	0.003706105	0.014430157
7924551				7.50	7.41	8.90	0.003707694	0.014433309
7911049	200159	*chromosome 1 open reading frame 100*	*C1orf100*	5.88	5.91	5.57	0.003713039	0.014452072
7971373				8.10	8.35	9.02	0.003713515	0.014452072

8005097	9953	*heparan sulfate (glucosamine) 3-O-sulfotransferase 3B1*	*HS3ST3B1*	8.84	8.97	7.60	0.003714623	0.014454434
8170326	2332	*fragile X mental retardation 1*	*FMR1*	7.52	7.31	9.25	0.003715884	0.014456225
8103503	84076	*transketolase-like 2*	*TKTL2*	5.92	6.13	5.49	0.003716084	0.014456225
7908488	3078	*complement factor H-related 1*	*CFHR1*	5.35	5.21	4.61	0.003717811	0.014460994
7923958	79098	*chromosome 1 open reading frame 116*	*C1orf116*	6.37	6.50	6.93	0.003718897	0.014463269
7953284	4908	*neurotrophin 3*	*NTF3*	6.55	8.19	5.94	0.003719551	0.014463862
8037878	56917	*Meis homeobox 3*	*MEIS3*	8.33	8.30	7.21	0.003721356	0.014468933
8044745	8886	*DEAD (Asp-Glu-Ala-Asp) box polypeptide 18*	*DDX18*	8.06	8.19	10.32	0.003723454	0.014475142
8133072	677803	*small nucleolar RNA, H/ACA box 15*	*SNORA15*	5.28	5.30	5.68	0.003725505	0.014481162
8045835	11227	*UDP-N-acetyl-alpha-D-galactosamine:polypeptide N-acetylgalactosaminyltransferase 5 (GalNAc-T5)*	*GALNT5*	7.84	8.24	5.36	0.003727777	0.014488045
7978542	57456		*KIAA1143*	9.58	9.61	10.97	0.003730116	0.014495186
7934731	10438		*C1D*	7.79	8.08	8.46	0.003733055	0.01450465
8136811	2051	*EPH receptor B6*	*EPHB6*	9.82	7.87	7.62	0.003736767	0.014513216
8049657	51665	*ankyrin repeat and SOCS box-containing 1*	*ASB1*	8.92	9.32	8.57	0.003735798	0.014513216
8098723				7.09	7.24	6.37	0.0037364	0.014513216
8121768	5570	*protein kinase (cAMP-dependent, catalytic) inhibitor beta*	*PKIB*	5.26	5.28	7.22	0.00373928	0.014521022
8045736	114793	*formin-like 2*	*FMNL2*	7.65	7.18	9.51	0.003740891	0.014525322
7956076	1017	*cyclin-dependent kinase 2*	*CDK2*	8.45	8.68	9.75	0.00374167	0.014526392
8051993	5281	*phosphatidylinositol glycan anchor biosynthesis, class F*	*PIGF*	6.27	6.42	7.58	0.003749411	0.014554489
8076461				11.02	11.07	10.32	0.003754393	0.014571871
8140367	6369	*chemokine (C-C motif) ligand 24*	*CCL24*	6.41	6.53	6.05	0.003755672	0.014574874
8065330				8.45	8.45	7.58	0.003757373	0.014579517
7905530	4184	*sperm mitochondria-associated cysteine-rich protein*	*SMCP*	5.66	5.73	5.42	0.003759496	0.014585792
7945958	143496	*olfactory receptor, family 52, subfamily B, member 4*	*OR52B4*	5.82	5.95	5.65	0.003762991	0.014597392
8029353	7673	*zinc finger protein 222*	*ZNF222*	6.52	6.32	6.88	0.003766599	0.014609425
8124380	3024	*histone cluster 1, H1a*	*HIST1H1A*	8.14	8.33	12.00	0.00377049	0.014616589

7948630	3995	*fatty acid desaturase 3*	*FADS3*	11.16	11.49	9.78	0.003774158	0.014616589
8065136	6238	*ribosome binding protein 1 homolog 180kDa (dog)*	*RRBP1*	9.40	9.36	8.53	0.003769843	0.014616589
8052845	7072		*TIA1*	8.97	8.34	11.02	0.003774656	0.014616589
7904086	9860	*leucine-rich repeats and immunoglobulin-like domains 2*	*LRIG2*	7.76	7.74	9.54	0.003769226	0.014616589
8143949	155051	*crystallin, gamma N*	*CRYGN*	7.87	7.99	7.43	0.003772931	0.014616589
8003861	201305	*spinster homolog 3 (Drosophila)*	*SPNS3*	6.62	6.75	6.20	0.003773482	0.014616589
8040338	6029, 378706	*RNA, 7SL, cytoplasmic 1 | RNA, 7SL, cytoplasmic 2*	*RN7SL1|RN7SL2*	13.71	13.76	13.41	0.003774654	0.014616589
7907349				5.44	5.36	6.23	0.003774361	0.014616589
7972733				6.78	6.76	6.53	0.003775028	0.014616589
8094142				4.74	4.87	4.70	0.003771664	0.014616589
8114111				7.17	7.56	6.39	0.003774012	0.014616589
8146894				5.58	5.62	5.05	0.003770308	0.014616589
8044813	140738	*transmembrane protein 37*	*TMEM37*	8.27	7.71	9.39	0.003782366	0.014639563
8023816	220158	*Gilles de la Tourette syndrome chromosome region, candidate 1*	*GTSCR1*	5.09	5.08	4.88	0.003782483	0.014639563
7898256	388595	*transmembrane protein 82*	*TMEM82*	7.12	7.15	6.58	0.003782423	0.014639563
8138765	3207	*homeobox A11*	*HOXA11*	6.02	6.81	5.68	0.003783107	0.014640017
8164843	29989	*odorant binding protein 2B*	*OBP2B*	6.81	7.01	6.21	0.003794028	0.014680309
7967060	8683	*serine/arginine-rich splicing factor 9*	*SRSF9*	8.86	8.85	9.26	0.003796551	0.014688104
8171579	6247	*retinoschisin 1*	*RS1*	5.95	6.08	5.76	0.003799188	0.014696335
8044611				6.96	6.87	6.51	0.003801676	0.014703992
8061940	128864, 63941	*chromosome 20 open reading frame 144 | N-terminal EF-hand calcium binding protein 3*	*C20orf144|NECAB3*	8.74	8.76	8.17	0.003804067	0.014711268
8007931	3690	*integrin, beta 3 (platelet glycoprotein IIIa, antigen CD61)*	*ITGB3*	8.40	8.60	5.95	0.003808144	0.014725061
8135114	1523	*cut-like homeobox 1*	*CUX1*	9.19	9.29	8.75	0.003809076	0.014726693
7912968	80834	*taste receptor, type 1, member 2*	*TAS1R2*	7.20	7.21	6.59	0.003809625	0.014726842
7977432	80757	*transmembrane protein 121*	*TMEM121*	8.55	8.51	7.74	0.003810445	0.014727499

8124495	94026	*POM121 membrane glycoprotein-like 2*	*POM121L 2*	5.28	5.37	5.11	0.003811186	0.014727499
8176249	474382, 83740, 474381	*H2A histone family, member B1 | H2A histone family, member B3 | H2A histone family, member B2*	*H2AFB1|H 2AFB3|H2 AFB2*	6.42	6.45	6.11	0.003811835	0.014727499
8115164				6.24	6.34	7.67	0.003811544	0.014727499
8126948	2509	*ferritin, heavy polypeptide 1 pseudogene 5*	*FTH1P5*	6.60	6.49	5.74	0.003814948	0.014737554
8047339	9689	*basic leucine zipper and W2 domains 1*	*BZW1*	9.81	9.78	10.93	0.003820367	0.014756094
8014574	11056	*DEAD (Asp-Glu-Ala-Asp) box polypeptide 52*	*DDX52*	8.62	8.60	10.72	0.00382077	0.014756094
8133314	653361, 654817, 654816	*neutrophil cytosolic factor 1 | neutrophil cytosolic factor 1C pseudogene | neutrophil cytosolic factor 1B pseudogene*	*NCF1|NC F1C|NCF1 B*	7.23	7.27	6.58	0.003823815	0.014765881
7965390	1833	*epiphycan*	*EPYC*	5.72	5.72	5.42	0.003826262	0.014773354
8113726	5480	*peptidylprolyl isomerase C (cyclophilin C)*	*PPIC*	9.17	9.28	8.10	0.003829515	0.014779981
7944845	116337	*pannexin 3*	*PANX3*	5.99	6.12	5.69	0.00382852	0.014779981
7956229	283375	*solute carrier family 39 (metal ion transporter), member 5*	*SLC39A5*	6.66	6.65	6.29	0.003829472	0.014779981
8000957	283932	*non-protein coding RNA 95*	*NCRNA00 095*	6.15	6.19	5.70	0.00383003	0.014779994
8107234	4124	*mannosidase, alpha, class 2A, member 1*	*MAN2A1*	8.42	9.28	10.26	0.003834138	0.014791894
7929932	81621	*Kazal-type serine peptidase inhibitor domain 1*	*KAZALD1*	8.73	8.58	7.50	0.003833672	0.014791894
7954279	121536	*AE binding protein 2*	*AEBP2*	8.76	8.50	10.27	0.003836761	0.014800033
8003156				6.98	7.13	6.56	0.003837558	0.014801133
8166872				6.94	6.76	5.86	0.003838653	0.014803377
7937952	143503	*olfactory receptor, family 51, subfamily E, member 1*	*OR51E1*	5.92	6.02	5.57	0.003839623	0.014803754
8172086				8.84	8.90	8.08	0.003839777	0.014803754
7940891	3338	*DnaJ (Hsp40) homolog, subfamily C, member 4*	*DNAJC4*	8.88	8.54	7.59	0.003840944	0.014806278
8038852	89858	*sialic acid binding Ig-like lectin 12*	*SIGLEC12*	7.10	7.08	6.60	0.00384183	0.014807716
7988833	256586	*LysM, putative peptidoglycan-binding, domain containing 2*	*LYSMD2*	7.60	7.53	6.80	0.003842507	0.014808347
7929168	80351	*tankyrase, TRF1-interacting ankyrin-related ADP-ribose polymerase 2*	*TNKS2*	8.55	8.38	10.12	0.003845983	0.014819766

7989750	8483	*cartilage intermediate layer protein, nucleotide pyrophosphohydrolase*	*CILP*	6.30	6.38	5.88	0.003848458	0.014827324
8049847	9855	*FERM, RhoGEF and pleckstrin domain protein 2*	*FARP2*	8.92	8.55	8.10	0.003849282	0.014828518
8103240	303	*annexin A2 pseudogene 1*	*ANXA2P1*	8.64	8.90	8.05	0.003849991	0.014829272
7929132	84333	*polycomb group ring finger 5*	*PCGF5*	7.70	8.03	6.26	0.003851814	0.014834315
8076744				7.22	7.40	6.43	0.003852543	0.014835141
7897509	64802	*nicotinamide nucleotide adenylyltransferase 1*	*NMNAT1*	6.64	6.58	6.08	0.003853349	0.014836268
8077376	3708	*inositol 1,4,5-triphosphate receptor, type 1*	*ITPR1*	6.56	6.64	6.82	0.003857458	0.014845368
7897295	80835	*taste receptor, type 1, member 1*	*TAS1R1*	7.16	7.18	6.38	0.003856649	0.014845368
8009183	4215, 80774	*mitogen-activated protein kinase kinase kinase 3 | LIM domain containing 2*	*MAP3K3|LIMD2*	9.25	9.07	8.12	0.003856903	0.014845368
8043449	50802, 28876, 28875, 100510044	*immunoglobulin kappa locus | immunoglobulin kappa variable 3D-11 | immunoglobulin kappa variable 3D-15 (gene/pseudogene) | immunoglobulin kappa locus-like*	*IGK@|IGKV3D-11|IGKV3D-15|LOC100510044*	6.98	6.94	6.46	0.00385777	0.014845368
7916018				5.33	5.30	6.78	0.003858953	0.014847943
7964089	9924		*PAN2*	8.37	8.25	9.11	0.003863349	0.014860895
8081853	56983	*protein O-glucosyltransferase 1*	*POGLUT1*	7.06	7.42	8.17	0.003863125	0.014860895
8118632	407002	*microRNA 219-1*	*MIR219-1*	6.80	6.98	6.05	0.00386657	0.014871304
7923360	7135	*troponin I type 1 (skeletal, slow)*	*TNNI1*	7.46	7.42	6.86	0.003870513	0.014884485
8146633	643763		*LOC643763*	5.76	5.70	6.56	0.003871109	0.014884795
8069818	337960	*keratin associated protein 13-3*	*KRTAP13-3*	5.57	5.66	5.39	0.003873479	0.014889942
7914232	399474, 2035	*transmembrane protein 200B | erythrocyte membrane protein band 4.1 (elliptocytosis 1, RH-linked)*	*TMEM200B|EPB41*	9.05	9.00	8.29	0.003873039	0.014889942
8048889	130888	*F-box protein 36*	*FBXO36*	7.47	7.36	8.29	0.003874473	0.014891778
8174596				5.60	5.64	6.34	0.003875755	0.014894726
8012218				9.87	10.39	8.45	0.003878356	0.014902734
8112622	84340	*G elongation factor, mitochondrial 2*	*GFM2*	7.56	7.14	8.60	0.003879088	0.014903199

8071597	100423062, 96610, 28775, 28825, 28816	*immunoglobulin lambda-like polypeptide 5 | BMS1 homolog, ribosome assembly protein (yeast) pseudogene | immunoglobulin lambda variable 7-46 (gene/pseudogene) | immunoglobulin lambda variable 1-40 | immunoglobulin lambda variable 2-11*	*IGLL5|LOC96610|IGLV7-46|IGLV1-40|IGLV2-11*	7.10	7.06	6.78	0.003879509	0.014903199
8038904	84765	*zinc finger protein 577*	*ZNF577*	7.11	6.84	8.65	0.003880086	0.014903434
7998872	124221	*protease, serine, 30 homolog (mouse), pseudogene*	*PRSS30P*	7.65	7.71	7.14	0.003881621	0.014905361
8097062				7.00	6.78	8.26	0.003881367	0.014905361
7919197	84265	*polymerase (RNA) III (DNA directed) polypeptide G (32kD)-like*	*POLR3GL*	6.79	6.72	6.09	0.003884574	0.014912176
7911237	284521	*olfactory receptor, family 2, subfamily L, member 13*	*OR2L13*	6.08	5.94	5.64	0.003884391	0.014912176
8165833	347527	*arylsulfatase family, member H*	*ARSH*	6.75	6.75	6.37	0.003884945	0.014912176
8159017	169436	*chromosome 9 open reading frame 96*	*C9orf96*	7.13	7.33	6.66	0.00388719	0.014918811
8117368	8364	*histone cluster 1, H4c*	*HIST1H4C*	9.69	10.32	11.76	0.003887946	0.014919729
7971602	55213	*regulator of chromosome condensation (RCC1) and BTB (POZ) domain containing protein 1*	*RCBTB1*	8.42	7.81	9.59	0.003888891	0.014921372
8121487				5.09	4.95	4.72	0.003890576	0.014925853
7923885	9641, 574431	*inhibitor of kappa light polypeptide gene enhancer in B-cells, kinase epsilon | chromosome 1 open reading frame 147*	*IKBKE|C1orf147*	6.04	5.95	5.61	0.003891513	0.014927464
8010285				7.64	7.75	7.15	0.003896223	0.014943546
8015223	83755, 85289, 85291, 728224	*keratin associated protein 4-12 | keratin associated protein 4-5 | keratin associated protein 4-2 | keratin associated protein 4-8*	*KRTAP4-12|KRTAP4-5|KRTAP4-2|KRTAP4-8*	8.07	8.55	7.51	0.003898297	0.014949513
8074878				8.59	8.74	8.00	0.003899053	0.014950426
8119918	221416	*chromosome 6 open reading frame 223*	*C6orf223*	6.33	6.40	6.07	0.003901694	0.014958565
7901054	1263, 343521	*polo-like kinase 3 | Tctex1 domain containing 4*	*PLK3|TCTEX1D4*	8.15	9.04	7.42	0.003903467	0.014963375

8055624	9839	*zinc finger E-box binding homeobox 2*	*ZEB2*	8.92	8.97	6.43	0.003904291	0.01496455
8127370	202559	*KH domain containing, RNA binding, signal transduction associated 2*	*KHDRBS2*	5.88	5.94	7.08	0.003904984	0.014965218
8118127	4795	*nuclear factor of kappa light polypeptide gene enhancer in B-cells inhibitor-like 1*	*NFKBIL1*	8.48	8.49	7.73	0.003908226	0.01497322
7939265	55226	*N-acetyltransferase 10 (GCN5-related)*	*NAT10*	8.94	9.03	10.16	0.003908249	0.01497322
7916667				5.89	5.65	5.92	0.003908628	0.01497322
8172345	2002		*ELK1*	9.56	9.85	8.84	0.003911444	0.014979321
8125360	5089	*pre-B-cell leukemia homeobox 2*	*PBX2*	9.96	9.72	10.46	0.00391097	0.014979321
8046726	6744	*sperm specific antigen 2*	*SSFA2*	10.25	9.18	7.79	0.003912067	0.014979321
8148220	654463	*fer-1-like 6 (C. elegans)*	*FER1L6*	6.56	6.47	6.06	0.003912296	0.014979321
8163063	8727	*catenin (cadherin-associated protein), alpha-like 1*	*CTNNAL1*	8.54	8.53	9.41	0.003914743	0.014980732
8092418	10057	*ATP-binding cassette, sub-family C (CFTR/MRP), member 5*	*ABCC5*	7.22	7.38	8.53	0.003915259	0.014980732
8048980	51719	*calcium binding protein 39*	*CAB39*	9.65	9.33	10.80	0.003914804	0.014980732
8051226	55006, 245711	*tRNA methyltransferase 61 homolog B (S. cerevisiae) | speedy homolog A (Xenopus laevis)*	*TRMT61B| SPDYA*	7.70	7.80	9.49	0.003914157	0.014980732
8117241				5.97	6.10	5.68	0.003914771	0.014980732
8176698	246126	*chromosome Y open reading frame 15A*	*CYorf15A*	7.03	6.21	8.62	0.003916049	0.014981767
7907368				7.30	7.59	6.61	0.003918223	0.014986113
8042970				5.98	6.23	7.91	0.00391776	0.014986113
7970317	7027	*transcription factor Dp-1*	*TFDP1*	11.60	11.89	11.13	0.003919392	0.014988598
7971461	3936	*lymphocyte cytosolic protein 1 (L-plastin)*	*LCP1*	5.64	5.79	7.73	0.003920084	0.014989261
7907146	9095	*T-box 19*	*TBX19*	7.60	8.07	6.85	0.003921588	0.014993024
8102751	132320	*sodium channel and clathrin linker 1*	*SCLT1*	7.07	6.85	7.39	0.003923125	0.014996912
8064514	79175	*zinc finger protein 343*	*ZNF343*	7.83	7.57	6.62	0.00392524	0.015002459
8040516	388931	*major facilitator superfamily domain containing 2B*	*MFSD2B*	6.05	6.20	5.64	0.003925615	0.015002459
8144742	286097	*EF-hand domain family, member A2*	*EFHA2*	7.87	6.78	7.34	0.00392823	0.015010465
8119801	89845	*ATP-binding cassette, sub-family C (CFTR/MRP), member 10*	*ABCC10*	9.01	9.15	7.91	0.003929323	0.015010801

8060400	642636, 9751	*RAD21-like 1 (S. pombe) | syntaphilin*	*RAD21L1|SNPH*	6.61	6.61	6.24	0.003929358	0.015010801
8106393	2149	*coagulation factor II (thrombin) receptor*	*F2R*	9.71	7.93	8.72	0.003930427	0.0150129
7978706	3169	*forkhead box A1*	*FOXA1*	7.22	7.25	6.57	0.003932782	0.015016548
7998643	6123	*ribosomal protein L3-like*	*RPL3L*	7.39	7.46	6.94	0.003932255	0.015016548
7902043	9829, 645195	*DnaJ (Hsp40) homolog, subfamily C, member 6 | hypothetical LOC645195*	*DNAJC6|LOC645195*	7.93	6.65	7.03	0.003932943	0.015016548
8125072	80737	*chromosome 6 open reading frame 27*	*C6orf27*	7.33	7.36	6.73	0.003933903	0.015018228
7939215	25758	*chromosome 11 open reading frame 41*	*C11orf41*	7.89	8.68	6.90	0.003935217	0.015020836
7996198	29070	*coiled-coil domain containing 113*	*CCDC113*	6.32	6.69	8.32	0.003936044	0.015020836
7933723	253430	*inositol polyphosphate multikinase*	*IPMK*	8.90	9.09	9.85	0.003936147	0.015020836
8003939	9032	*transmembrane 4 L six family member 5*	*TM4SF5*	7.49	7.62	6.95	0.003939542	0.015031803
7960637	57121	*lysophosphatidic acid receptor 5*	*LPAR5*	7.58	7.70	7.17	0.003941724	0.015038144
8061529	28954	*RAS (RAD and GEM)-like GTP-binding 1*	*REM1*	7.36	7.47	6.96	0.00394274	0.015040033
8087653	51364	*zinc finger, MYND-type containing 10*	*ZMYND10*	7.15	7.28	6.61	0.003953338	0.015076716
7915682	57643	*zinc finger, SWIM-type containing 5*	*ZSWIM5*	7.00	6.73	7.68	0.003953402	0.015076716
8157696	392391	*olfactory receptor, family 5, subfamily C, member 1*	*OR5C1*	7.07	7.10	6.52	0.003954324	0.015078241
7970251	8858	*protein Z, vitamin K-dependent plasma glycoprotein*	*PROZ*	5.67	5.77	5.42	0.003955416	0.015080218
8041000	11321, 84450	*GPN-loop GTPase 1 | zinc finger protein 512*	*GPN1|ZNF512*	8.40	8.32	9.58	0.003955887	0.015080218
8172225	80258	*EF-hand domain (C-terminal) containing 2*	*EFHC2*	5.45	5.37	6.11	0.003956864	0.015081951
7960438	57101	*anoctamin 2*	*ANO2*	5.92	5.86	6.49	0.003959744	0.015085816
7973875	64067	*neuronal PAS domain protein 3*	*NPAS3*	6.16	6.32	5.72	0.003960491	0.015085816
8093756	345222	*chromosome 4 open reading frame 44*	*C4orf44*	6.13	6.09	5.73	0.003959035	0.015085816
7995222	6524, 64755	*solute carrier family 5 (sodium/glucose cotransporter), member 2 | chromosome 16 open reading frame 58*	*SLC5A2|C16orf58*	7.43	7.45	6.91	0.003958643	0.015085816
8159965				5.80	5.83	4.96	0.003960436	0.015085816
8129773	9774	*BCL2-associated transcription factor 1*	*BCLAF1*	9.06	8.92	11.00	0.003962707	0.015088285
8149955	55872	*PDZ binding kinase*	*PBK*	6.21	6.59	7.46	0.003962427	0.015088285

8062964	90196, 767557	*SYS1 Golgi-localized integral membrane protein homolog (S. cerevisiae) | SYS1-DBNDD2 read-through transcript*	*SYS1|SYS1-DBNDD2*	9.99	9.81	8.99	0.003962073	0.015088285
7949124	5837	*phosphorylase, glycogen, muscle*	*PYGM*	7.30	6.65	8.02	0.003965926	0.015093826
8110894	54888	*NOP2/Sun domain family, member 2*	*NSUN2*	8.25	8.40	10.10	0.003966027	0.015093826
8031617	55311	*zinc finger protein 444*	*ZNF444*	8.37	8.46	7.61	0.003965962	0.015093826
8137133	84626	*KRAB-A domain containing 1*	*KRBA1*	8.18	8.23	7.66	0.003966253	0.015093826
8113350				4.39	4.36	4.34	0.003967204	0.015095455
8024655	100128569	*chromosome 19 open reading frame 71*	*C19orf71*	7.83	8.03	7.24	0.003968102	0.01509688
8078395				5.33	5.37	5.12	0.003969693	0.015100943
8096147	404201	*non-protein coding RNA 247*	*NCRNA00247*	7.29	7.24	7.00	0.003971097	0.015104296
8076851	79087	*asparagine-linked glycosylation 12, alpha-1,6-mannosyltransferase homolog (S. cerevisiae)*	*ALG12*	9.92	9.77	8.86	0.003971658	0.015104437
7958749	10019	*SH2B adaptor protein 3*	*SH2B3*	9.44	9.26	8.77	0.003981369	0.015139376
8112892				9.42	9.26	11.17	0.003984762	0.015150283
8119016	5603	*mitogen-activated protein kinase 13*	*MAPK13*	7.74	7.50	8.66	0.003985611	0.015151514
8028613	282618	*interleukin 29 (interferon, lambda 1)*	*IL29*	7.32	7.44	6.71	0.003986413	0.015152569
8170027	203522	*DEAD/H (Asp-Glu-Ala-Asp/His) box polypeptide 26B*	*DDX26B*	6.44	6.51	7.35	0.003988395	0.015158107
7975292	5890	*RAD51-like 1 (S. cerevisiae)*	*RAD51L1*	6.92	7.04	7.98	0.003990674	0.015158783
8122222	27115	*phosphodiesterase 7B*	*PDE7B*	7.47	7.09	5.88	0.003990369	0.015158783
8065372	63908	*N-ethylmaleimide-sensitive factor attachment protein, beta*	*NAPB*	6.64	6.70	7.63	0.003990195	0.015158783
8112331	81689	*iron-sulfur cluster assembly 1 homolog (S. cerevisiae)*	*ISCA1*	9.50	9.59	10.68	0.003990131	0.015158783
8132302	402481	*hypothetical LOC402481*	*PP13004*	6.56	6.74	6.24	0.003992816	0.015164928
8118682	5252	*PHD finger protein 1*	*PHF1*	10.01	10.25	8.77	0.003994703	0.015169924
8048926	93349	*SP140 nuclear body protein-like*	*SP140L*	7.55	7.54	5.61	0.003995183	0.015169924
8137715	79778	*MICAL-like 2*	*MICALL2*	8.75	8.52	7.72	0.003996718	0.015173758
8084607	59343	*SUMO1/sentrin/SMT3 specific peptidase 2*	*SENP2*	7.42	7.34	9.48	0.003999205	0.015179331
8063209	100131496		*LOC100131496*	6.28	6.51	6.93	0.003999237	0.015179331

8083935				6.67	6.73	6.22	0.004000073	0.015180507
8114455	3313	*heat shock 70kDa protein 9 (mortalin)*	*HSPA9*	9.75	10.55	11.96	0.004000916	0.015181708
7961300	5544	*proline-rich protein BstNI subfamily 3*	*PRB3*	8.48	8.58	7.96	0.004001756	0.015182902
7917000				6.22	6.30	5.84	0.004003649	0.015188086
8050557	6382	*syndecan 1*	*SDC1*	10.01	9.66	8.84	0.004004676	0.015189989
8175217	2239	*glypican 4*	*GPC4*	7.41	7.85	11.02	0.00400579	0.015192217
8117219	55856	*acyl-CoA thioesterase 13*	*ACOT13*	10.36	10.20	11.47	0.004007397	0.015196316
8011220	406994	*microRNA 212*	*MIR212*	7.94	8.01	7.38	0.004009549	0.015202477
8020308	753	*chromosome 18 open reading frame 1*	*C18orf1*	6.52	6.71	6.11	0.004012345	0.015207781
7922689	2752	*glutamate-ammonia ligase*	*GLUL*	10.43	9.81	10.90	0.004011849	0.015207781
8058456				5.61	5.60	5.19	0.004012528	0.015207781
7938834	89797	*neuron navigator 2*	*NAV2*	8.34	8.04	9.65	0.00401574	0.01521596
8093137	255798	*chromosome 3 open reading frame 43*	*C3orf43*	5.52	5.51	5.25	0.004015328	0.01521596
8074890				6.32	6.47	6.19	0.00401896	0.015226162
8000409	3151	*high-mobility group nucleosomal binding domain 2*	*HMGN2*	11.71	11.46	12.06	0.004023891	0.015235463
8078600	6917	*transcription elongation factor A (SII), 1*	*TCEA1*	10.21	10.46	11.32	0.004024237	0.015235463
8087201	51447	*inositol hexakisphosphate kinase 2*	*IP6K2*	8.93	8.77	9.67	0.004024991	0.015235463
7947358	100190939		*LOC100190939*	9.25	9.29	8.48	0.004023346	0.015235463
8035831	148198, 57615	*zinc finger protein 98 | zinc finger protein 492*	*ZNF98|ZNF492*	6.14	6.08	8.07	0.004026165	0.015235463
8051061	7349, 57159	*urocortin | tripartite motif-containing 54*	*UCN|TRIM54*	8.18	8.20	7.59	0.00402598	0.015235463
8057561				5.97	5.91	6.35	0.004025849	0.015235463
8146896				4.81	4.88	4.61	0.004024425	0.015235463
8168079				8.23	8.57	7.01	0.004023018	0.015235463
8042413	10887	*prokineticin receptor 1*	*PROKR1*	6.75	6.95	7.69	0.004028873	0.015243712
8077171	11158, 11159	*RAB, member of RAS oncogene family-like 2B | RAB, member of RAS oncogene family-like 2A*	*RABL2B|RABL2A*	10.13	10.15	9.09	0.004029671	0.015244734
8145568				7.41	7.61	6.90	0.004031772	0.015250684

8068866	5316, 4731	*PBX/knotted 1 homeobox 1 | NADH dehydrogenase (ubiquinone) flavoprotein 3, 10kDa*	*PKNOX1|NDUFV3*	7.32	7.73	8.43	0.004033599	0.015255597
8062409	56259	*catenin, beta like 1*	*CTNNBL1*	7.85	7.93	8.35	0.004034925	0.015258614
8027352				12.19	12.02	13.38	0.004039053	0.015272224
8063755	63939	*chromosome 20 open reading frame 177*	*C20orf177*	7.69	7.41	9.12	0.004043163	0.015280101
8088803	317649	*eukaryotic translation initiation factor 4E family member 3*	*EIF4E3*	7.76	8.14	9.08	0.004043512	0.015280101
8167201	347344	*zinc finger protein 81*	*ZNF81*	6.59	6.65	7.39	0.004041952	0.015280101
8141477	352954		*GATS*	8.34	8.18	7.66	0.004043783	0.015280101
8071951				8.54	8.61	7.44	0.004043163	0.015280101
7980463	22938	*SNW domain containing 1*	*SNW1*	8.20	7.91	9.35	0.004046333	0.015282302
8104580	90268	*family with sequence similarity 105, member B*	*FAM105B*	8.16	8.33	8.77	0.004046483	0.015282302
8056215				7.08	7.22	6.61	0.004044979	0.015282302
8136159				9.44	10.22	7.94	0.004046185	0.015282302
7925257	1130	*lysosomal trafficking regulator*	*LYST*	7.46	6.94	5.64	0.004047625	0.015284615
8177647	6606, 6607	*survival of motor neuron 1, telomeric | survival of motor neuron 2, centromeric*	*SMN1|SMN2*	8.25	8.68	9.88	0.004048314	0.01528522
7902754	9629	*chloride channel accessory 3 (pseudogene)*	*CLCA3P*	5.41	5.36	5.14	0.004050704	0.015290242
7902493				7.77	7.77	7.19	0.004050431	0.015290242
7999634	9284, 339044, 399491, 100288332, 642778	*nuclear pore complex interacting protein | polycystic kidney disease 1 (autosomal dominant) pseudogene 1 | GPS, PLAT and transmembrane domain-containing protein | GPS, PLAT and transmembrane domain-containing protein FLJ00285-like | nuclear pore complex-interacting protein-like 1-like*	*NPIP|PKD1P1|LOC399491|LOC100288332|LOC642778*	11.50	11.56	10.53	0.004051455	0.015291077
8111885				5.41	5.48	5.19	0.004052114	0.015291565
8041820	9655	*suppressor of cytokine signaling 5*	*SOCS5*	10.27	10.65	9.39	0.004053851	0.015292125
8163678	23245	*astrotactin 2*	*ASTN2*	7.05	6.94	7.54	0.004053383	0.015292125
8174103	2710, 2713	*glycerol kinase | glycerol kinase 3 pseudogene*	*GK|GK3P*	8.60	7.79	9.41	0.004053763	0.015292125
8018107	23580	*CDC42 effector protein (Rho GTPase binding) 4*	*CDC42EP4*	8.98	8.36	7.51	0.004056461	0.015299971
8148331				5.51	5.45	4.75	0.00405803	0.01530389

8024754	84699	*cAMP responsive element binding protein 3-like 3*	*CREB3L3*	7.08	7.16	6.76	0.004060626	0.015311677
8133108	677803	*small nucleolar RNA, H/ACA box 15*	*SNORA15*	5.28	5.29	5.68	0.004062732	0.015317618
8105958	6606, 6607	*survival of motor neuron 1, telomeric | survival of motor neuron 2, centromeric*	*SMN1|SMN2*	8.25	8.68	9.87	0.004064527	0.015322385
8003892	342527	*smoothelin-like 2*	*SMTNL2*	7.84	7.93	7.27	0.004066083	0.015326249
8014916	94103	*ORM1-like 3 (S. cerevisiae)*	*ORMDL3*	8.59	8.55	7.83	0.004074674	0.015356627
7953723	50856	*C-type lectin domain family 4, member A*	*CLEC4A*	5.69	5.62	5.31	0.004084348	0.015390322
7924055	148304	*chromosome 1 open reading frame 74*	*C1orf74*	8.05	8.15	9.21	0.004084681	0.015390322
7899016	6392	*succinate dehydrogenase complex, subunit D, integral membrane protein*	*SDHD*	10.24	10.37	11.34	0.004088227	0.015395647
8103011	54726	*OTU domain containing 4*	*OTUD4*	6.85	6.87	9.34	0.004087035	0.015395647
8141669	375607	*chromosome 7 open reading frame 52*	*C7orf52*	6.13	6.23	5.66	0.004087434	0.015395647
7992181	7177, 64499	*tryptase alpha/beta 1 | tryptase beta 2 (gene/pseudogene)*	*TPSAB1|TPSB2*	7.49	7.55	6.79	0.004088087	0.015395647
8156838	10952	*Sec61 beta subunit*	*SEC61B*	11.45	11.82	11.42	0.00408929	0.015397642
8110916	442132	*golgin A6 family-like 1 pseudogene*	*LOC442132*	9.12	9.33	8.35	0.004089968	0.015398187
7973564	5720	*proteasome (prosome, macropain) activator subunit 1 (PA28 alpha)*	*PSME1*	11.28	10.79	10.19	0.004091371	0.01540146
8073645	25813	*sorting and assembly machinery component 50 homolog (S. cerevisiae)*	*SAMM50*	10.74	10.64	11.36	0.004093551	0.015407656
8011245	55720, 63826	*TSR1, 20S rRNA accumulation, homolog (S. cerevisiae) | serine racemase*	*TSR1|SRR*	7.20	7.74	9.58	0.004095038	0.015411242
8171874				5.94	6.08	5.31	0.004098475	0.01542217
8121136				5.53	5.54	5.23	0.004099795	0.015425123
8149169	1673	*defensin, beta 4A*	*DEFB4A*	5.92	5.92	5.44	0.004101572	0.01542779
8049620	151176	*family with sequence similarity 132, member B*	*FAM132B*	8.15	8.10	7.09	0.004101377	0.01542779
8154635	4507, 100048912	*methylthioadenosine phosphorylase | CDKN2B antisense RNA (non-protein coding)*	*MTAP|CDKN2B-AS*	7.02	7.18	8.65	0.004102131	0.015427881
7935421	23401	*frequently rearranged in advanced T-cell lymphomas 2*	*FRAT2*	7.99	8.09	8.92	0.004103424	0.015430733
7984217	80301	*pleckstrin homology domain containing, family O member 2*	*PLEKHO2*	9.60	9.65	8.28	0.004105571	0.015436798
8160670	360	*aquaporin 3 (Gill blood group)*	*AQP3*	8.07	7.63	7.20	0.004107401	0.015439656

8178338	10107	*tripartite motif-containing 10*	*TRIM10*	6.48	6.50	6.01	0.004107153	0.015439656
7950804	220388	*coiled-coil domain containing 89*	*CCDC89*	6.05	5.98	5.49	0.004109704	0.0154463
7931519	85442	*kinase non-catalytic C-lobe domain (KIND) containing 1*	*KNDC1*	7.86	7.84	7.40	0.004111504	0.015451056
7977241	64423	*inverted formin, FH2 and WH2 domain containing*	*INF2*	10.45	10.41	8.96	0.004115673	0.015463649
8071532	85376, 440804, 150221	*RIMS binding protein 3 | RIMS binding protein 3B | RIMS binding protein 3C*	*RIMBP3|RIMBP3B|RIMBP3C*	6.85	6.77	6.26	0.004115927	0.015463649
8165512	54932	*exonuclease 3'-5' domain containing 3*	*EXD3*	7.84	7.77	7.20	0.004118295	0.015470533
8001564	55715	*docking protein 4*	*DOK4*	9.56	9.18	8.27	0.004119752	0.015473994
8041447	51232	*cysteine rich transmembrane BMP regulator 1 (chordin-like)*	*CRIM1*	11.26	11.27	9.47	0.004124822	0.015491021
8177967	4795	*nuclear factor of kappa light polypeptide gene enhancer in B-cells inhibitor-like 1*	*NFKBIL1*	8.49	8.49	7.74	0.004126989	0.015493115
8144047	22976	*PAX interacting (with transcription-activation domain) protein 1*	*PAXIP1*	7.17	7.45	9.22	0.004126335	0.015493115
7898707	401944, 3339	*low density lipoprotein receptor class A domain containing 2 | heparan sulfate proteoglycan 2*	*LDLRAD2|HSPG2*	8.00	7.96	7.18	0.004126851	0.015493115
8122598				7.37	7.31	6.92	0.004127597	0.01549338
7951497	4863	*nuclear protein, ataxia-telangiectasia locus*	*NPAT*	8.44	7.78	10.00	0.004129523	0.015498597
8128079	135295	*serine/arginine-rich splicing factor 12*	*SRSF12*	6.23	6.36	7.46	0.004130288	0.01549945
8003180	55625	*zinc finger, DHHC-type containing 7*	*ZDHHC7*	10.54	10.29	9.79	0.004136	0.015518871
7924923	55746	*nucleoporin 133kDa*	*NUP133*	7.48	7.24	9.67	0.004144169	0.0155475
8140227	653361, 654817, 654816	*neutrophil cytosolic factor 1 | neutrophil cytosolic factor 1C pseudogene | neutrophil cytosolic factor 1B pseudogene*	*NCF1|NCF1C|NCF1B*	7.25	7.28	6.58	0.00414684	0.015555497
8046527	3238	*homeobox D12*	*HOXD12*	6.23	6.36	5.60	0.004150276	0.015562467
8044225	27233	*sulfotransferase family, cytosolic, 1C, member 4*	*SULT1C4*	5.12	5.09	5.61	0.004149585	0.015562467
8013602	645832, 7448	*SEBOX homeobox | vitronectin*	*SEBOX|VTN*	6.30	6.36	6.04	0.004150315	0.015562467
8139367	29881	*NPC1 (Niemann-Pick disease, type C1, gene)-like 1*	*NPC1L1*	6.88	6.62	6.08	0.004151682	0.01556355
7948314	219960	*olfactory receptor, family 10, subfamily Q, member 1*	*OR10Q1*	6.97	7.14	6.68	0.004151143	0.01556355

8104321	170690	*ADAM metallopeptidase with thrombospondin type 1 motif, 16*	*ADAMTS16*	6.92	6.84	7.71	0.004152278	0.015563765
8173551	5255	*phosphorylase kinase, alpha 1 (muscle)*	*PHKA1*	6.83	6.66	8.41	0.004154371	0.015569586
8104930	6507	*solute carrier family 1 (glial high affinity glutamate transporter), member 3*	*SLC1A3*	6.45	7.80	7.68	0.004159845	0.015577967
8173201	7789	*zinc finger, X-linked, duplicated A*	*ZXDA*	9.95	10.00	9.14	0.004158273	0.015577967
8177706	7932	*olfactory receptor, family 2, subfamily H, member 2*	*OR2H2*	6.11	6.06	5.63	0.004159353	0.015577967
7972805	55647		*RAB20*	7.29	7.33	8.39	0.00415833	0.015577967
8029117	63946	*DMRT-like family C2*	*DMRTC2*	6.82	6.89	6.20	0.004159282	0.015577967
8047377				5.56	5.39	5.93	0.004159465	0.015577967
8169009	56271	*brain expressed, X-linked 4*	*BEX4*	9.19	8.59	10.27	0.004163867	0.01559101
7937977				5.69	5.64	5.23	0.004164941	0.015593008
7951565	57569	*Rho GTPase activating protein 20*	*ARHGAP20*	7.48	6.43	5.58	0.00416796	0.015602287
8106978	317671, 83890	*Rieske (Fe-S) domain containing | spermatogenesis associated 9*	*RFESD|SPATA9*	5.93	5.72	6.61	0.00416931	0.015605317
8071194				6.28	6.37	5.80	0.004170678	0.015608412
7914923	127700	*organic solute carrier partner 1*	*OSCP1*	7.65	7.46	6.62	0.004171661	0.015608576
8067506	140893	*chromosome 20 open reading frame 151*	*C20orf151*	7.59	7.59	7.05	0.004171803	0.015608576
7985001	79661	*nei endonuclease VIII-like 1 (E. coli)*	*NEIL1*	7.79	7.35	6.86	0.00417267	0.015609799
8019765	396	*Rho GDP dissociation inhibitor (GDI) alpha*	*ARHGDIA*	11.17	11.40	9.73	0.004175677	0.015619021
7931079				6.50	6.41	5.84	0.0041769	0.015621573
8064676	64096	*GDNF family receptor alpha 4*	*GFRA4*	8.46	8.52	7.70	0.004178021	0.015621717
8098163				6.26	5.40	5.32	0.004177753	0.015621717
7972723	55082	*arginine and glutamate rich 1*	*ARGLU1*	8.71	8.77	10.71	0.00417991	0.015624774
7943552	60496, 143879	*aminoadipate-semialdehyde dehydrogenase-phosphopantetheinyl transferase | kelch repeat and BTB (POZ) domain containing 3*	*AASDHPPT|KBTBD3*	8.66	8.39	9.63	0.004179921	0.015624774
8150842				6.66	6.64	6.25	0.004182757	0.015633354
8024003	5064	*paralemmin*	*PALM*	9.17	8.98	8.23	0.004185638	0.015642093
8151547				5.73	5.50	6.53	0.004186936	0.01564492

8143714	27153	*zinc finger protein 777*	*ZNF777*	8.40	8.43	7.36	0.004189543	0.01565258
8081053				6.35	6.38	5.82	0.004190071	0.01565258
7930311	119391	*glutathione S-transferase omega 2*	*GSTO2*	7.43	7.33	7.90	0.004190782	0.015653212
7921786	5202	*prefoldin subunit 2*	*PFDN2*	9.43	9.63	9.93	0.004195223	0.01565903
7909271	11009	*interleukin 24*	*IL24*	5.90	5.98	5.61	0.004196025	0.01565903
7906810	11266	*dual specificity phosphatase 12*	*DUSP12*	7.86	8.24	8.71	0.004193497	0.01565903
8031827	84914	*zinc finger protein 587*	*ZNF587*	8.24	7.75	9.01	0.004195313	0.01565903
8076882	164714	*tubulin tyrosine ligase-like family, member 8*	*TTLL8*	6.71	6.93	6.26	0.004196136	0.01565903
7922887				5.76	6.04	5.13	0.004195584	0.01565903
8167871				4.73	4.65	4.57	0.004195723	0.01565903
7897404	11315	*Parkinson disease (autosomal recessive, early onset) 7*	*PARK7*	10.86	10.88	11.39	0.004197182	0.015660906
8030753	354	*kallikrein-related peptidase 3*	*KLK3*	6.77	6.81	6.18	0.004199029	0.015665775
8019273	10189	*THO complex 4*	*THOC4*	10.89	11.09	12.52	0.004202601	0.015671002
8118564	3118, 3117	*major histocompatibility complex, class II, DQ alpha 2 | major histocompatibility complex, class II, DQ alpha 1*	*HLA-DQA2|HLA-DQA1*	6.24	6.27	5.76	0.004202212	0.015671002
7940259	58475, 84689	*membrane-spanning 4-domains, subfamily A, member 7 | membrane-spanning 4-domains, subfamily A, member 14*	*MS4A7|MS4A14*	5.66	5.67	5.35	0.004202471	0.015671002
7983478	84419, 100126311	*chromosome 15 open reading frame 48 | microRNA 147b*	*C15orf48|MIR147B*	5.12	5.09	4.82	0.004201643	0.015671002
8019772	10189	*THO complex 4*	*THOC4*	11.07	11.25	12.71	0.004203992	0.015674165
8116195	91522	*collagen, type XXIII, alpha 1*	*COL23A1*	8.32	8.40	7.93	0.004207467	0.015685093
7985662	5151	*phosphodiesterase 8A*	*PDE8A*	8.04	8.41	6.97	0.004209814	0.015691815
8078442	1233	*chemokine (C-C motif) receptor 4*	*CCR4*	5.90	5.80	5.58	0.00421171	0.015692474
8180093	3111	*major histocompatibility complex, class II, DO alpha*	*HLA-DOA*	7.62	7.66	8.46	0.004211471	0.015692474
8098611	7098	*toll-like receptor 3*	*TLR3*	8.05	6.68	6.35	0.004210559	0.015692474
7910164	56997	*aarF domain containing kinase 3*	*ADCK3*	7.98	7.83	8.84	0.004212165	0.015692474
7956018	5959	*retinol dehydrogenase 5 (11-cis/9-cis)*	*RDH5*	8.15	8.05	6.99	0.004212796	0.0156928
8008644				8.08	8.20	7.29	0.004213496	0.015693381

8179009	7932	*olfactory receptor, family 2, subfamily H, member 2*	*OR2H2*	6.10	6.06	5.63	0.004217694	0.015706994
8000034	55567	*dynein, axonemal, heavy chain 3*	*DNAH3*	5.77	5.84	5.54	0.004218798	0.015709078
8007123	83899, 83901, 81870, 83900, 85280	*keratin associated protein 9-2 | keratin associated protein 9-8 | keratin associated protein 9-9 | keratin associated protein 9-3 | keratin associated protein 9-4*	*KRTAP9-2|KRTAP9-8|KRTAP9-9|KRTAP9-3|KRTAP9-4*	6.43	6.52	6.22	0.004220335	0.015712775
8124134	7172	*thiopurine S-methyltransferase*	*TPMT*	7.08	7.80	8.63	0.004221801	0.015714178
8071042	100288884		*LOC100288884*	5.99	6.22	5.52	0.004221558	0.015714178
7979260	2764	*glia maturation factor, beta*	*GMFB*	9.34	9.49	10.40	0.004223897	0.015719954
7970565				8.70	8.74	8.28	0.004229862	0.015740125
8156043	29968	*phosphoserine aminotransferase 1*	*PSAT1*	10.77	11.33	11.84	0.004233609	0.01575204
8094056	94031	*HtrA serine peptidase 3*	*HTRA3*	9.69	9.14	7.40	0.004235383	0.015756607
8137271	26	*amiloride binding protein 1 (amine oxidase (copper-containing))*	*ABP1*	7.30	7.21	6.84	0.004237311	0.015761746
7952408	54414	*sialic acid acetylesterase*	*SIAE*	8.70	8.45	6.80	0.004239445	0.01576611
8064203	140701	*chromosome 20 open reading frame 135*	*C20orf135*	7.02	7.24	6.52	0.004239576	0.01576611
8083034	64084	*calsyntenin 2*	*CLSTN2*	7.22	6.81	6.52	0.004240665	0.01576813
8010086	124512	*chromosome 17 open reading frame 95*	*C17orf95*	9.03	9.11	9.69	0.00424405	0.015774619
7981777	400968		*LOC400968*	6.31	6.27	5.89	0.004244035	0.015774619
8067963				4.65	4.64	6.18	0.004243353	0.015774619
8062852	11142	*protein kinase (cAMP-dependent, catalytic) inhibitor gamma*	*PKIG*	9.76	9.27	8.22	0.004244855	0.01577558
8171516	5931	*retinoblastoma binding protein 7*	*RBBP7*	11.21	11.28	12.18	0.004245459	0.015775794
8179249	4795	*nuclear factor of kappa light polypeptide gene enhancer in B-cells inhibitor-like 1*	*NFKBIL1*	8.49	8.50	7.74	0.004248268	0.0157842
8154620				7.24	7.34	6.90	0.004251228	0.015793165

7992854	7760	*zinc finger protein 213*	*ZNF213*	8.09	8.27	7.13	0.004255008	0.01580314
8090660				4.57	4.57	4.46	0.004254737	0.01580314
8179224	394263	*mucin 21, cell surface associated*	*MUC21*	7.41	7.47	6.89	0.004257136	0.015809013
8125383	4855, 63940	*notch 4 | G-protein signaling modulator 3*	*NOTCH4| GPSM3*	7.54	7.54	6.99	0.004257921	0.015809892
7914665				6.38	6.44	5.65	0.004259105	0.015812257
8112072	10309	*cyclin O*	*CCNO*	7.82	7.95	7.17	0.004260447	0.015815204
8055265				7.10	7.08	6.70	0.004261089	0.015815552
7914334	9410	*small nuclear ribonucleoprotein 40kDa (U5)*	*SNRNP40*	9.81	9.82	11.18	0.004263479	0.015817061
7997662	9764		*KIAA0513*	8.72	8.51	7.43	0.004263836	0.015817061
7915130	10946	*splicing factor 3a, subunit 3, 60kDa*	*SF3A3*	9.60	9.81	11.96	0.004263293	0.015817061
7909661	26750	*ribosomal protein S6 kinase, 52kDa, polypeptide 1*	*RPS6KC1*	7.62	7.44	8.57	0.004264733	0.015817061
7956795	29110	*TANK-binding kinase 1*	*TBK1*	8.12	7.83	8.68	0.004262153	0.015817061
7979804				5.51	5.75	5.32	0.004264783	0.015817061
7939494	390110	*1-aminocyclopropane-1-carboxylate synthase homolog (Arabidopsis)(non-functional)-like*	*ACCSL*	5.91	5.81	5.20	0.004268387	0.015828395
7967874				5.60	5.59	5.39	0.004271836	0.01583915
8116033	2161	*coagulation factor XII (Hageman factor)*	*F12*	7.13	7.23	6.63	0.004274263	0.015846113
7905544	6698	*small proline-rich protein 1A*	*SPRR1A*	7.41	7.29	6.81	0.004278221	0.015856717
8163444	7539	*zinc finger protein 37 homolog (mouse)*	*ZFP37*	6.48	6.03	7.04	0.004278066	0.015856717
7981383				8.45	8.67	7.93	0.004279741	0.015860314
8042701	2016	*empty spiracles homeobox 1*	*EMX1*	8.25	8.33	7.55	0.004281938	0.015864383
8010080	677849	*small nucleolar RNA, C/D box 1B*	*SNORD1B*	4.94	4.93	5.48	0.004281735	0.015864383
7902269	9295	*serine/arginine-rich splicing factor 11*	*SRSF11*	10.73	10.58	12.26	0.004284907	0.015871307
8102606				4.66	4.87	4.58	0.004284664	0.015871307
7990307				6.22	6.26	5.97	0.00428576	0.01587243
7997520	3281	*heat shock factor binding protein 1*	*HSBP1*	8.87	9.34	9.58	0.004288109	0.015879093
7899244	55650	*phosphatidylinositol glycan anchor biosynthesis, class V*	*PIGV*	9.30	9.29	8.79	0.004290342	0.015885327
8051443	6801	*striatin, calmodulin binding protein*	*STRN*	8.02	7.76	10.63	0.004291763	0.015888548

8178771	177	*advanced glycosylation end product-specific receptor*	*AGER*	7.23	7.28	6.90	0.004293412	0.015888866
7923503	51094	*adiponectin receptor 1*	*ADIPOR1*	12.25	12.37	11.89	0.0042935	0.015888866
8048075				7.91	7.73	7.09	0.004293178	0.015888866
7930561	3026	*hyaluronan binding protein 2*	*HABP2*	6.71	6.75	6.11	0.004294778	0.015889524
8110589	57472	*CCR4-NOT transcription complex, subunit 6*	*CNOT6*	7.18	7.09	8.93	0.004294274	0.015889524
8094301	9353	*slit homolog 2 (Drosophila)*	*SLIT2*	10.17	10.59	7.54	0.004299935	0.015906563
7998434	7177 64499	*tryptase alpha/beta 1 | tryptase beta 2 (gene/pseudogene)*	*TPSAB1|TPSB2*	7.45	7.49	6.75	0.004302518	0.01591408
7921088	10763	*nestin*	*NES*	7.92	7.50	8.52	0.004307391	0.015930064
8084064	10797	*methylenetetrahydrofolate dehydrogenase (NADP+ dependent) 2, methenyltetrahydrofolate cyclohydrolase*	*MTHFD2*	9.96	10.50	11.61	0.004308884	0.015933542
8038150	284358	*MEF2 activating motif and SAP domain containing transcriptional regulator*	*MAMSTR*	7.77	7.36	6.73	0.004309524	0.015933868
8001531	4495	*metallothionein 1G*	*MT1G*	12.00	12.20	10.76	0.004318612	0.015964389
7967863	100289635	*zinc finger protein 605*	*ZNF605*	6.72	6.57	7.93	0.004318885	0.015964389
7999326	7874	*ubiquitin specific peptidase 7 (herpes virus-associated)*	*USP7*	9.25	9.19	11.15	0.004319728	0.015965464
8085094	285362	*sulfatase modifying factor 1*	*SUMF1*	7.72	7.69	7.15	0.004320411	0.01596594
7984987				6.25	6.26	5.97	0.004326846	0.015987676
8133518	653361, 654817, 654816	*neutrophil cytosolic factor 1 | neutrophil cytosolic factor 1C pseudogene | neutrophil cytosolic factor 1B pseudogene*	*NCF1|NCF1C|NCF1B*	7.25	7.29	6.64	0.004327882	0.015989456
7973084	283, 6038	*angiogenin, ribonuclease, RNase A family, 5 | ribonuclease, RNase A family, 4*	*ANG|RNASE4*	8.13	7.96	6.64	0.004328949	0.015991354
8062658	64900, 90187	*lipin 3 | elastin microfibril interfacer 3*	*LPIN3|EMILIN3*	7.77	7.93	6.86	0.004335116	0.016012086
8054020	51252	*family with sequence similarity 178, member B*	*FAM178B*	6.88	7.03	6.41	0.004335903	0.016012942
8130151	135250	*retinoic acid early transcript 1E*	*RAET1E*	5.23	5.34	4.94	0.004337908	0.016018301
8103411				6.54	6.43	5.85	0.004339312	0.016021434
8014047	79736	*chromosome 17 open reading frame 42*	*C17orf42*	6.50	6.45	7.47	0.004343703	0.016033133
7930703	142940	*TruB pseudouridine (psi) synthase homolog 1 (E. coli)*	*TRUB1*	8.03	8.05	9.19	0.004343329	0.016033133
7991184				4.66	4.66	4.60	0.004344147	0.016033133

8008029	30009	*T-box 21*	*TBX21*	7.47	7.56	6.89	0.00434724	0.016038397
8050480	93034	*5'-nucleotidase, cytosolic IB*	*NT5C1B*	5.54	5.59	5.22	0.004347092	0.016038397
8175232				6.59	6.01	5.90	0.004346175	0.016038397
8040068				4.79	4.55	4.47	0.004348801	0.016042106
8087782	132141	*IQ motif containing F1*	*IQCF1*	5.58	5.54	5.32	0.004349404	0.016042281
7961339	4040	*low density lipoprotein receptor-related protein 6*	*LRP6*	8.73	7.97	10.53	0.004350896	0.016045736
7984983				6.25	6.26	5.96	0.004351964	0.016046716
8102037				5.91	5.89	6.68	0.004352273	0.016046716
8056730	440925		*LOC44092 5*	6.34	6.31	5.89	0.004354716	0.016053671
8140995	799	*calcitonin receptor*	*CALCR*	5.44	5.40	5.93	0.004357025	0.016060133
8070744	755	*chromosome 21 open reading frame 2*	*C21orf2*	7.51	7.58	6.89	0.004357638	0.016060343
7899643	3061	*hypocretin (orexin) receptor 1*	*HCRTR1*	7.38	7.35	6.70	0.004361173	0.016071318
7925174	9804	*translocase of outer mitochondrial membrane 20 homolog (yeast)*	*TOMM20*	9.21	9.14	10.76	0.004361773	0.016071478
8134737	255374	*metallo-beta-lactamase domain containing 1*	*MBLAC1*	7.27	7.30	6.85	0.004363511	0.016075829
8012953	10626	*tripartite motif-containing 16*	*TRIM16*	10.58	9.92	8.04	0.004368221	0.016091127
8030736	93650	*acid phosphatase, testicular*	*ACPT*	7.45	7.59	6.92	0.004372086	0.016103312
8029413	7770	*zinc finger protein 227*	*ZNF227*	7.30	7.10	7.97	0.004373074	0.01610346
8064059	57642	*collagen, type XX, alpha 1*	*COL20A1*	7.44	7.52	6.90	0.004373242	0.01610346
7959251	5027	*purinergic receptor P2X, ligand-gated ion channel, 7*	*P2RX7*	6.47	6.34	5.61	0.004374994	0.016104411
8080773	11102	*ribonuclease P/MRP 14kDa subunit*	*RPP14*	7.87	8.25	9.81	0.004376285	0.016104411
8100798	27284	*sulfotransferase family, cytosolic, 1B, member 1*	*SULT1B1*	6.61	5.64	5.07	0.004376388	0.016104411
8144505	137075	*claudin 23*	*CLDN23*	8.03	8.09	7.44	0.004376184	0.016104411
8014326	201299	*RAD52 motif 1*	*RDM1*	5.90	6.01	7.17	0.004376847	0.016104411
7933931				8.68	8.79	7.95	0.004374445	0.016104411
7912863	11223, 11209, 4485	*macrophage stimulating 1 (hepatocyte growth factor-like) pseudogene 9 | macrophage stimulating 1 (hepatocyte growth factor-like) pseudogene 2*	*MST1P9|M ST1P2*	7.46	7.32	6.89	0.004377814	0.016105914
7974914				6.32	6.38	5.94	0.004381195	0.016116299

8031669	388566	*zinc finger protein 470*	*ZNF470*	6.45	6.34	8.12	0.004387443	0.016137226
8159992	79956	*endoplasmic reticulum metallopeptidase 1*	*ERMP1*	7.11	6.76	8.91	0.004388308	0.016138352
8133504	2969	*general transcription factor IIi*	*GTF2I*	9.18	8.74	10.45	0.004389426	0.016138699
7992670	5170	*3-phosphoinositide dependent protein kinase-1*	*PDPK1*	9.28	9.49	8.86	0.00439008	0.016138699
8139909				6.55	6.47	5.83	0.004389618	0.016138699
8046542	3235	*homeobox D9*	*HOXD9*	7.43	8.56	6.95	0.004391194	0.016140742
8084488	5437	*polymerase (RNA) II (DNA directed) polypeptide H*	*POLR2H*	11.53	11.54	12.32	0.004392054	0.016141847
8171427	2277	*c-fos induced growth factor (vascular endothelial growth factor D)*	*FIGF*	6.53	5.34	5.18	0.004393003	0.016143281
7920616	127579	*DC-STAMP domain containing 2*	*DCST2*	7.02	6.96	6.45	0.004394988	0.016146464
8153862	441381, 9684	*leucine rich repeat containing 24 | leucine rich repeat containing 14*	*LRRC24|LRRC14*	8.66	8.71	7.80	0.004394628	0.016146464
8067113	7764	*zinc finger protein 217*	*ZNF217*	8.13	8.38	9.99	0.004395582	0.01614659
8005473	10605	*poly(A) binding protein interacting protein 1*	*PAIP1*	7.90	8.13	9.12	0.004396593	0.01614825
8140140	1365	*claudin 3*	*CLDN3*	8.22	8.25	8.98	0.004397476	0.016149439
7975696	338917	*visual system homeobox 2*	*VSX2*	7.31	7.39	6.77	0.004400011	0.016156692
8027510	91442	*chromosome 19 open reading frame 40*	*C19orf40*	7.57	7.34	7.91	0.004403232	0.016166463
8061780	80341	*bactericidal/permeability-increasing protein-like 1*	*BPIL1*	6.84	6.97	6.45	0.004409262	0.016186543
8065124	631	*beaded filament structural protein 1, filensin*	*BFSP1*	6.49	6.40	7.07	0.004416651	0.016211607
8150559	55145	*THAP domain containing, apoptosis associated protein 1*	*THAP1*	8.22	8.56	8.68	0.004418439	0.01621611
8175098	139760	*G protein-coupled receptor 119*	*GPR119*	6.09	6.19	5.77	0.004421131	0.016223928
8096070	651	*bone morphogenetic protein 3*	*BMP3*	6.43	6.41	5.96	0.004423411	0.016227556
8078330	27303	*RNA binding motif, single stranded interacting protein 3*	*RBMS3*	9.66	9.81	7.15	0.004423806	0.016227556
8091629	285315	*chromosome 3 open reading frame 33*	*C3orf33*	6.21	6.35	6.94	0.004423031	0.016227556
8056966	100129455		*LOC100129455*	11.95	11.92	11.50	0.004424727	0.01622887
7982204	3151	*high-mobility group nucleosomal binding domain 2*	*HMGN2*	11.75	11.52	12.11	0.004429266	0.016239328
7946288	7761	*zinc finger protein 214*	*ZNF214*	6.19	5.77	6.57	0.004428787	0.016239328
8011808	55065	*G protein-coupled receptor 172B*	*GPR172B*	7.10	7.22	6.67	0.004429172	0.016239328

8001048	64755, 6524	*chromosome 16 open reading frame 58 | solute carrier family 5 (sodium/glucose cotransporter), member 2*	*C16orf58|SLC5A2*	10.13	10.04	8.76	0.004430138	0.016240463
8067495	81928	*Cdk5 and Abl enzyme substrate 2*	*CABLES2*	8.19	8.24	7.36	0.004432769	0.016248045
8157668	26740	*olfactory receptor, family 1, subfamily J, member 2*	*OR1J2*	4.66	4.85	4.58	0.00443343	0.016248407
8114158	5515, 51265	*protein phosphatase 2, catalytic subunit, alpha isozyme | cyclin-dependent kinase-like 3*	*PPP2CA|CDKL3*	9.80	10.05	11.90	0.004434957	0.016251938
8127534	115004	*chromosome 6 open reading frame 150*	*C6orf150*	7.26	7.18	7.97	0.004437469	0.016259082
7959314	23067	*SET domain containing 1B*	*SETD1B*	9.27	9.16	7.88	0.004440019	0.01626636
7951422	84437		*KIAA1826*	7.03	6.81	8.26	0.004442238	0.016272424
7915657				6.20	6.18	5.65	0.004444774	0.016279649
7994570				6.86	6.75	6.25	0.004447472	0.016287465
7968577	26960	*neurobeachin*	*NBEA*	7.21	6.89	8.67	0.004448648	0.016287636
8099235	114932	*Morf4 family associated protein 1-like 1*	*MRFAP1L1*	8.42	8.31	9.33	0.00444861	0.016287636
8138950	6100	*retinitis pigmentosa 9 (autosomal dominant)*	*RP9*	7.71	8.03	7.48	0.004451116	0.016288409
8012285	9196	*potassium voltage-gated channel, shaker-related subfamily, beta member 3*	*KCNAB3*	6.27	6.19	5.79	0.004450972	0.016288409
7999025	10131	*TNF receptor-associated protein 1*	*TRAP1*	10.01	9.90	11.59	0.004450939	0.016288409
8005458	654346, 284194, 3965	*lectin, galactoside-binding, soluble, 9C | lectin, galactoside-binding, soluble, 9B | lectin, galactoside-binding, soluble, 9*	*LGALS9C|LGALS9B*	7.43	7.65	6.60	0.004450052	0.016288409
7903742	2948, 2946	*glutathione S-transferase mu 4 | glutathione S-transferase mu 2 (muscle)*	*GSTM4|GSTM2*	10.99	10.25	8.54	0.00445295	0.016293056
8042495	11017	*small nuclear ribonucleoprotein 27kDa (U4/U6.U5)*	*SNRNP27*	9.60	10.14	10.00	0.004459702	0.016315695
8077851	3269	*histamine receptor H1*	*HRH1*	6.90	6.96	6.09	0.004463902	0.016328989
8140739	11257	*TP53 target 1 (non-protein coding)*	*TP53TG1*	7.97	7.23	6.84	0.004468299	0.016334867
8019964	79822	*Rho GTPase activating protein 28*	*ARHGAP28*	6.27	7.73	8.02	0.004466513	0.016334867
8013068	201164	*phospholipase D family, member 6*	*PLD6*	7.79	7.88	7.32	0.004468338	0.016334867

8098725	441056, 653543, 22947, 728410, 653548, 653545, 653544	*double homeobox 4 like 4 | double homeobox 4 like 7 | double homeobox 4 like 2 | double homeobox 4 like 3 | double homeobox 4 like 5 | double homeobox 4 like 6*	*DUX4L4|DUX4L7|DUX4L2|DUX4L3|DUX4L5|DUX4L6*	8.58	8.65	7.92	0.004468205	0.016334867
7968798				6.88	6.72	6.22	0.004467137	0.016334867
7980309	11161	*chromosome 14 open reading frame 1*	*C14orf1*	12.61	12.05	12.69	0.004469	0.01633522
8132911				6.02	5.86	5.41	0.004469639	0.016335487
7958056	55576	*stabilin 2*	*STAB2*	5.96	5.98	5.61	0.00447103	0.016338501
7966052	1407	*cryptochrome 1 (photolyase-like)*	*CRY1*	7.31	7.46	8.60	0.004473037	0.016343765
8004241	440400, 124944	*ribonuclease, RNase K | chromosome 17 open reading frame 49*	*RNASEK|C17orf49*	11.29	11.47	10.55	0.004476007	0.016352547
8081217	2838	*G protein-coupled receptor 15*	*GPR15*	5.03	4.97	5.37	0.004479204	0.016356533
8116439	92304	*secretoglobin, family 3A, member 1*	*SCGB3A1*	7.34	7.38	6.89	0.004478048	0.016356533
8016336	114881	*oxysterol binding protein-like 7*	*OSBPL7*	8.23	7.93	7.34	0.004479364	0.016356533
7984569	54839, 56906	*leucine rich repeat containing 49 | THAP domain containing 10*	*LRRC49|THAP10*	8.79	8.33	9.32	0.004479063	0.016356533
8164062	613206	*myeloproliferative disease associated tumor antigen 5*	*LOC613206*	7.00	7.00	6.55	0.004483807	0.01637069
8042115				5.54	5.59	5.32	0.004487664	0.016382698
8140166				5.35	5.58	5.19	0.004491296	0.016393883
7951089				6.42	6.42	5.98	0.004493658	0.016400432
8038981	81856	*zinc finger protein 611*	*ZNF611*	6.28	6.06	7.40	0.004500149	0.016422046
8018449	201294	*unc-13 homolog D (C. elegans)*	*UNC13D*	7.18	7.11	6.82	0.004501462	0.01642476
7954245	54477	*pleckstrin homology domain containing, family A member 5*	*PLEKHA5*	9.09	7.43	10.05	0.004507781	0.016441582
8070482	150135	*chromosome 21 open reading frame 129*	*C21orf129*	6.68	6.71	6.09	0.004507741	0.016441582
7957476				5.59	5.59	5.20	0.004507302	0.016441582
7911207	81470	*olfactory receptor, family 2, subfamily G, member 2*	*OR2G2*	5.62	5.87	5.44	0.004510123	0.016448047
8123843	266553	*orofacial cleft 1 candidate 1*	*OFCC1*	5.05	5.05	4.90	0.004512117	0.016453242

8005726	3768, 100134444	*potassium inwardly-rectifying channel, subfamily J, member 12 | potassium inwardly-rectifying channel, subfamily J, member 18*	*KCNJ12|KCNJ18*	8.07	8.23	7.38	0.004513093	0.01645472
8132694	3484	*insulin-like growth factor binding protein 1*	*IGFBP1*	8.13	8.20	7.44	0.004516827	0.016464492
7961820	144360	*chromosome 12 open reading frame 67*	*C12orf67*	6.15	6.18	5.84	0.004516914	0.016464492
7928069	1305	*collagen, type XIII, alpha 1*	*COL13A1*	7.51	7.69	6.96	0.004518662	0.016464628
7994109	5347	*polo-like kinase 1*	*PLK1*	9.49	10.08	12.06	0.004518397	0.016464628
8143863	10922	*Fas-activated serine/threonine kinase*	*FASTK*	9.83	9.83	8.32	0.004518032	0.016464628
8157610	153090	*DAB2 interacting protein*	*DAB2IP*	9.22	8.68	7.99	0.00452023	0.016468262
7904830	27246	*ring finger protein 115*	*RNF115*	10.45	10.74	9.86	0.004524356	0.016481215
7979269	2643	*GTP cyclohydrolase 1*	*GCH1*	6.19	6.19	7.38	0.004528454	0.016486458
8178867	6890	*transporter 1, ATP-binding cassette, sub-family B (MDR/TAP)*	*TAP1*	9.86	9.61	8.19	0.004528482	0.016486458
7962811	54934	*chromosome 12 open reading frame 41*	*C12orf41*	7.90	8.01	8.84	0.004529222	0.016486458
8002087	57610	*RAN binding protein 10*	*RANBP10*	9.37	9.21	8.61	0.004528649	0.016486458
8036830	148014	*tetratricopeptide repeat domain 9B*	*TTC9B*	7.12	7.23	6.67	0.004527391	0.016486458
8025982				5.73	5.57	6.79	0.004528846	0.016486458
8099140				4.77	4.88	4.60	0.004530503	0.016489046
8090388	152065	*chromosome 3 open reading frame 22*	*C3orf22*	6.56	6.65	6.18	0.004531131	0.01648925
8180061	6890	*transporter 1, ATP-binding cassette, sub-family B (MDR/TAP)*	*TAP1*	9.86	9.61	8.19	0.00453347	0.016495682
7984014				6.94	6.92	6.15	0.004534681	0.016498009
7932962				6.14	6.22	5.72	0.004538803	0.016510926
8090503				6.50	6.52	6.05	0.004539796	0.016512457
7996341	57546	*pyruvate dehyrogenase phosphatase catalytic subunit 2*	*PDP2*	8.24	7.84	9.00	0.004545278	0.016530313
8125512	6890	*transporter 1, ATP-binding cassette, sub-family B (MDR/TAP)*	*TAP1*	9.86	9.61	8.19	0.00454724	0.016534753
8061869	128861	*chromosome 20 open reading frame 71*	*C20orf71*	6.17	6.17	5.64	0.004547644	0.016534753
8121257	639	*PR domain containing 1, with ZNF domain*	*PRDM1*	8.95	8.52	6.85	0.0045493	0.016536608
8113403	54826	*gypsy retrotransposon integrase 1*	*GIN1*	6.18	6.34	6.85	0.004549116	0.016536608

7978824	6235	*ribosomal protein S29*	*RPS29*	8.00	7.96	8.40	0.004550631	0.016539363
7968274	255967		*PAN3*	8.95	8.71	10.40	0.004552189	0.01654086
8059012				6.02	5.92	5.61	0.004551711	0.01654086
8074212	128954	*GRB2-associated binding protein family, member 4*	*GAB4*	7.08	7.05	6.60	0.004554288	0.016546404
8053713				9.64	9.59	8.94	0.004555071	0.01654717
8060325	400830	*defensin, beta 132*	*DEFB132*	7.06	7.09	6.49	0.004557183	0.016552757
8006592				6.74	6.67	6.31	0.004558664	0.016556052
7932118	222389	*BEN domain containing 7*	*BEND7*	6.93	7.40	6.31	0.004561575	0.01656454
8084917	440993		*LOC440993*	6.64	6.55	5.71	0.004562624	0.016566267
8117589	8357	*histone cluster 1, H3h*	*HIST1H3H*	6.79	7.22	9.01	0.004564133	0.016567579
7925446				7.23	7.59	6.50	0.00456379	0.016567579
8052010	805	*calmodulin 2 (phosphorylase kinase, delta)*	*CALM2*	10.28	10.58	10.89	0.004569555	0.016584644
8117727	442194	*olfactory receptor, family 10, subfamily C, member 1*	*OR10C1*	5.83	5.85	5.26	0.004569983	0.016584644
8139921	83698	*calneuron 1*	*CALN1*	6.08	6.08	7.05	0.004572074	0.016590144
8033043	2528	*fucosyltransferase 6 (alpha (1,3) fucosyltransferase)*	*FUT6*	7.03	7.02	6.63	0.004576547	0.016602201
7908328				8.48	8.52	7.79	0.004576179	0.016602201
7975203	64398	*membrane protein, palmitoylated 5 (MAGUK p55 subfamily member 5)*	*MPP5*	7.53	7.22	8.65	0.004577302	0.016602855
8117120	3400	*inhibitor of DNA binding 4, dominant negative helix-loop-helix protein*	*ID4*	7.54	7.93	8.84	0.004580376	0.016611919
8046895	165215	*family with sequence similarity 171, member B*	*FAM171B*	11.00	10.07	10.42	0.004581621	0.01661432
8091037	729627, 389151, 56945	*proline rich 23A | proline rich 23B | mitochondrial ribosomal protein S22*	*PRR23A|PRR23B|MRPS22*	7.19	7.26	6.46	0.004582765	0.01661432
8162868				8.55	8.59	8.21	0.004582429	0.01661432
8100306				7.28	7.43	6.69	0.004584312	0.016617842
8063351	23315	*solute carrier family 9 (sodium/hydrogen exchanger), member 8*	*SLC9A8*	9.61	9.65	8.76	0.004584948	0.016618062
8160663	364	*aquaporin 7*	*AQP7*	7.04	6.89	6.61	0.004585586	0.016618287

7965112	5074	*PRKC, apoptosis, WT1, regulator*	*PAWR*	9.02	9.13	11.06	0.004586272	0.016618688
8107005	831	*calpastatin*	*CAST*	10.56	10.61	8.79	0.00458968	0.016626863
7952426	23584, 90952	*V-set and immunoglobulin domain containing 2 | endothelial cell adhesion molecule*	*VSIG2|ESAM*	7.74	7.64	7.19	0.004589371	0.016626863
7986444	145757		*LOC145757*	6.98	7.02	6.53	0.004590681	0.016628403
7900159	7802	*dynein, axonemal, light intermediate chain 1*	*DNALI1*	9.27	8.05	9.22	0.004592579	0.016633192
8142646	154865	*IQ motif and ubiquitin domain containing*	*IQUB*	4.66	4.64	4.93	0.004594115	0.016636668
8023261	10449	*acetyl-CoA acyltransferase 2*	*ACAA2*	8.48	8.57	10.14	0.004594704	0.016636712
8106479	9522	*secretory carrier membrane protein 1*	*SCAMP1*	9.60	9.56	10.65	0.004596094	0.016637105
7981742	28387	*immunoglobulin heavy variable 5-78 pseudogene*	*IGHV5-78*	6.98	7.07	6.62	0.004596542	0.016637105
7896929	64856	*von Willebrand factor A domain containing 1*	*VWA1*	8.58	8.48	7.96	0.004596359	0.016637105
8066091	140710	*chromosome 20 open reading frame 117*	*C20orf117*	7.80	7.77	6.93	0.004598079	0.016640585
7995834	644314, 4501	*metallothionein 1I (pseudogene) | metallothionein 1X*	*MT1IP|MT1X*	7.28	7.29	6.56	0.004601604	0.016651256
8173360				6.39	6.29	5.93	0.004605956	0.016664915
8160870	10850, 730098	*chemokine (C-C motif) ligand 27 | hypothetical LOC730098*	*CCL27|LOC730098*	7.17	7.26	6.63	0.004607245	0.016667487
7992379	197342	*essential meiotic endonuclease 1 homolog 2 (S. pombe)*	*EME2*	8.47	8.43	7.81	0.004608229	0.016668959
8098146	4889	*neuropeptide Y receptor Y5*	*NPY5R*	4.96	4.99	6.68	0.004609187	0.016670337
8039664	55659	*zinc finger protein 416*	*ZNF416*	8.51	8.46	9.24	0.004612107	0.016678808
7915170	26292	*c-myc binding protein*	*MYCBP*	8.70	8.99	9.64	0.00461421	0.016684322
7984245	123264, 51285	*organic solute transporter beta | RAS-like, family 12*	*OSTBETA|RASL12*	6.96	6.81	6.37	0.00461563	0.016687365
8065776	23054	*nuclear receptor coactivator 6*	*NCOA6*	9.23	9.04	7.85	0.004616761	0.016689366
8125936	1208	*colipase, pancreatic*	*CLPS*	6.87	6.86	6.48	0.004617418	0.01668965
8065427	92086	*gamma-glutamyltransferase light chain 1*	*GGTLC1*	6.51	6.50	5.94	0.004618157	0.016690232
8168362	392490	*TPT1-like protein*	*FLJ44635*	7.23	7.28	7.01	0.004619774	0.016693985
8118100	100507436	*MHC class I polypeptide-related sequence A*	*MICA*	9.10	8.82	7.81	0.00462246	0.016701601
7940000	390181	*olfactory receptor, family 5, subfamily AK, member 2*	*OR5AK2*	5.71	5.48	5.36	0.004628406	0.016719932

8068145	337968, 337975	*keratin associated protein 6-3 | keratin associated protein 20-1*	*KRTAP6-3|KRTAP20-1*	7.27	7.32	6.99	0.004628691	0.016719932
8103106	987	*LPS-responsive vesicle trafficking, beach and anchor containing*	*LRBA*	8.36	7.37	10.71	0.004632696	0.016732302
8079107	131377	*kelch repeat and BTB (POZ) domain containing 5*	*KBTBD5*	7.05	7.06	6.39	0.004635186	0.0167392
7963142	23017	*Fas apoptotic inhibitory molecule 2*	*FAIM2*	10.75	8.96	6.57	0.004637798	0.016746541
8079117	1238	*chemokine binding protein 2*	*CCBP2*	6.47	6.48	6.11	0.004639925	0.016752124
7922058	55811	*adenylate cyclase 10 (soluble)*	*ADCY10*	5.54	5.58	5.32	0.00464692	0.016771087
8042556				5.92	6.04	5.43	0.004646256	0.016771087
8069764				6.70	6.86	6.14	0.004646723	0.016771087
7978833	6166	*ribosomal protein L36a-like*	*RPL36AL*	9.48	9.22	10.18	0.004648726	0.016771317
8043630	10865	*AT rich interactive domain 5A (MRF1-like)*	*ARID5A*	7.62	7.77	7.00	0.004648308	0.016771317
7910638				8.52	8.39	7.54	0.004647711	0.016771317
8140113	6804	*syntaxin 1A (brain)*	*STX1A*	7.52	7.80	6.91	0.004652392	0.016782444
7908940	493	*ATPase, Ca++ transporting, plasma membrane 4*	*ATP2B4*	10.52	10.55	8.66	0.004655789	0.016786972
8154765	3301	*DnaJ (Hsp40) homolog, subfamily A, member 1*	*DNAJA1*	8.55	8.40	9.68	0.00465597	0.016786972
7985741	123624	*ATP/GTP binding protein-like 1*	*AGBL1*	5.68	5.68	5.27	0.004655158	0.016786972
7937438	283232	*transmembrane protein 80*	*TMEM80*	9.05	8.90	7.74	0.004655973	0.016786972
7956005	121129	*olfactory receptor, family 2, subfamily AP, member 1*	*OR2AP1*	5.18	5.12	4.93	0.004656636	0.016787266
7959205	9761	*malectin*	*MLEC*	10.69	10.65	11.38	0.004657586	0.01678803
7917283	255231	*mucolipin 2*	*MCOLN2*	6.08	6.08	6.86	0.004658011	0.01678803
8009322				6.97	7.00	6.45	0.004659498	0.016791292
8001776				5.66	5.84	6.49	0.004660904	0.016794264
7999791	408050, 23420, 283820	*NODAL modulator 3 | NODAL modulator 1 | NODAL modulator 2*	*NOMO3|NOMO1|NOMO2*	12.59	12.56	12.02	0.004662006	0.016796138
7993511	408050, 23420, 283820	*NODAL modulator 3 | NODAL modulator 1 | NODAL modulator 2*	*NOMO3|NOMO1|NOMO2*	12.59	12.56	12.02	0.004663636	0.016799912
8074384	2928	*goosecoid homeobox 2*	*GSC2*	8.31	8.51	7.67	0.004669697	0.016813355

7911383	54991	*chromosome 1 open reading frame 159*	*C1orf159*	7.92	7.98	7.23	0.004669583	0.016813355
8061490	284800, 728882	*family with sequence similarity 182, member A | family with sequence similarity 182, member B*	*FAM182A|FAM182B*	6.58	6.56	6.12	0.004668868	0.016813355
8122732				6.40	6.56	7.02	0.004668008	0.016813355
7952671				7.24	7.34	6.84	0.004675295	0.016831411
7917612	164045		*HFM1*	4.77	4.85	5.43	0.00467724	0.016834216
7998629				8.00	8.01	7.33	0.004677094	0.016834216
8013771	116238	*TLC domain containing 1*	*TLCD1*	7.70	7.78	9.05	0.004679822	0.016841408
7993371	408050, 23420, 283820	*NODAL modulator 3 | NODAL modulator 1 | NODAL modulator 2*	*NOMO3|NOMO1|NOMO2*	12.59	12.56	12.02	0.004680405	0.016841408
7940851	23769	*fibronectin leucine rich transmembrane protein 1*	*FLRT1*	7.38	7.62	6.52	0.004684947	0.016851451
7977771	26534	*olfactory receptor, family 10, subfamily G, member 2*	*OR10G2*	5.90	5.90	5.64	0.004684419	0.016851451
7943787	3316, 91894	*heat shock 27kDa protein 2 | chromosome 11 open reading frame 52*	*HSPB2|C11orf52*	8.08	8.03	7.07	0.004684358	0.016851451
8069148	386681	*keratin associated protein 10-8*	*KRTAP10-8*	8.14	8.05	7.37	0.004688892	0.016861439
8040111				5.41	5.11	4.98	0.004688657	0.016861439
8028104	10870	*hematopoietic cell signal transducer*	*HCST*	7.24	7.37	6.53	0.00469051	0.016864058
8032601	84839	*retina and anterior neural fold homeobox 2*	*RAX2*	7.49	7.67	7.04	0.004690789	0.016864058
7967969	53342	*interleukin 17D*	*IL17D*	8.40	8.46	7.77	0.004694242	0.01687437
8028984	1572	*cytochrome P450, family 2, subfamily F, polypeptide 1*	*CYP2F1*	6.07	6.14	5.63	0.004695558	0.016877
8179481	3122	*major histocompatibility complex, class II, DR alpha*	*HLA-DRA*	6.16	6.05	6.99	0.004696497	0.016878273
8160086				6.57	6.56	5.90	0.004697337	0.01687919
8033996	7297	*tyrosine kinase 2*	*TYK2*	8.82	8.79	7.65	0.00469833	0.01688066
8040908	9784	*sorting nexin 17*	*SNX17*	11.43	11.30	10.05	0.004699078	0.016881242
8179258	4049	*lymphotoxin alpha (TNF superfamily, member 1)*	*LTA*	6.41	6.45	6.15	0.004701	0.016886048
8148553	54742	*lymphocyte antigen 6 complex, locus K*	*LY6K*	7.88	7.58	6.54	0.004703476	0.01689284
8078920	4336	*myelin-associated oligodendrocyte basic protein*	*MOBP*	5.97	5.98	5.60	0.004705239	0.01689707
7975995	283579	*chromosome 14 open reading frame 178*	*C14orf178*	7.43	7.41	6.79	0.004708284	0.0169059

7963084	84070	*family with sequence similarity 186, member B*	*FAM186B*	6.27	6.32	5.82	0.00471209	0.016917463
8007497				4.62	4.51	4.48	0.004715305	0.016926901
8112584	167465	*zinc finger protein 366*	*ZNF366*	6.28	6.37	5.98	0.00471924	0.016938921
8171066	100288778, 100287171, 653635, 375260, 374666	*WAS protein family homolog 1 pseudogene | WAS protein family homolog 7 pseudogene | WAS protein family homolog 2 pseudogene | WAS protein family homolog 3 pseudogene*	*LOC100288778|WASH1|WASH7P|WASH2P|WASH3P*	9.78	9.56	8.80	0.004720596	0.01694043
8112174				8.08	8.14	7.45	0.004720834	0.01694043
8131203				5.14	5.18	4.81	0.00472487	0.016952803
7969426				4.66	4.74	4.55	0.004728841	0.016964944
7897561	23095	*kinesin family member 1B*	*KIF1B*	8.01	8.15	9.89	0.004729661	0.016965777
8072894	11078, 79159	*TRIO and F-actin binding protein | nucleolar protein 12*	*TRIOBP|NOL12*	8.81	8.62	7.79	0.004731211	0.016969232
7912250	407040	*microRNA 34a*	*MIR34A*	9.56	9.51	8.79	0.004731951	0.016969776
8168958	64860	*armadillo repeat containing, X-linked 5*	*ARMCX5*	7.48	7.26	8.04	0.004735082	0.016978893
7976669	84439	*HHIP-like 1*	*HHIPL1*	8.94	8.60	7.95	0.004736005	0.016980097
8048523	3300	*DnaJ (Hsp40) homolog, subfamily B, member 2*	*DNAJB2*	8.23	8.19	7.19	0.004738312	0.01698178
8121632	3841	*karyopherin alpha 5 (importin alpha 6)*	*KPNA5*	7.15	7.10	8.11	0.004738781	0.01698178
8130567	6950	*t-complex 1*	*TCP1*	10.77	10.93	12.38	0.004738828	0.01698178
7934653	11128	*polymerase (RNA) III (DNA directed) polypeptide A, 155kDa*	*POLR3A*	8.07	8.21	9.32	0.004738208	0.01698178
8012349	5187	*period homolog 1 (Drosophila)*	*PER1*	8.33	8.49	7.71	0.004745369	0.017001
7941039	8986	*ribosomal protein S6 kinase, 90kDa, polypeptide 4*	*RPS6KA4*	8.67	8.78	7.31	0.004745227	0.017001
8002845	440387, 1504	*chymotrypsinogen B2 | chymotrypsinogen B1*	*CTRB2|CTRB1*	7.54	7.51	7.07	0.004746189	0.017001828
7905503	353140	*late cornified envelope 2C*	*LCE2C*	5.84	6.00	5.19	0.004747604	0.017004784
7977344	23241	*phosphofurin acidic cluster sorting protein 2*	*PACS2*	9.70	9.65	8.55	0.004748499	0.017005883
8088956				4.99	5.05	4.80	0.00474961	0.017007751
8000411	79831	*jumonji domain containing 5*	*JMJD5*	6.41	6.32	5.75	0.004754216	0.017022133

8113286	55781	*RIO kinase 2 (yeast)*	*RIOK2*	8.42	8.59	9.61	0.004758683	0.017036013
8150590				5.79	5.97	6.38	0.004759987	0.017038569
7941457	11007	*coiled-coil domain containing 85B*	*CCDC85B*	9.15	9.13	8.18	0.004761329	0.017038692
8063955	11054	*opioid growth factor receptor*	*OGFR*	9.33	9.44	8.34	0.004761367	0.017038692
8052149	23198	*proteasome (prosome, macropain) activator subunit 4*	*PSME4*	8.87	8.60	10.72	0.004762114	0.017038692
8092638				4.97	4.62	4.48	0.004762382	0.017038692
8043028				5.74	5.57	5.39	0.004763373	0.017040124
7945631	81532	*Mps one binder kinase activator-like 2*	*MOB2*	7.17	7.30	6.75	0.004765868	0.017046938
8141311	221786	*family with sequence similarity 200, member A*	*FAM200A*	6.27	6.36	7.37	0.004766735	0.017047927
8035566	9454	*homer homolog 3 (Drosophila)*	*HOMER3*	9.79	9.77	8.70	0.004767886	0.017049931
7955551	341405	*ankyrin repeat domain 33*	*ANKRD33*	6.62	6.79	6.29	0.004771754	0.01706165
7938154	3611	*integrin-linked kinase*	*ILK*	11.71	11.75	10.54	0.004773746	0.01706666
8019622	113277, 728772	*transmembrane protein 106A | hypothetical LOC728772*	*TMEM106A|FLJ77644*	9.88	9.44	7.78	0.00477764	0.017078465
7952453	54538	*roundabout homolog 4, magic roundabout (Drosophila)*	*ROBO4*	7.62	7.46	6.83	0.004781396	0.017089775
8079532	25930	*protein tyrosine phosphatase, non-receptor type 23*	*PTPN23*	9.52	9.54	8.31	0.004783001	0.017093396
8064967	8195	*McKusick-Kaufman syndrome*	*MKKS*	5.85	5.91	7.05	0.004783667	0.01709366
7946972	6290	*serum amyloid A3 pseudogene*	*SAA3P*	5.42	5.44	5.12	0.004787691	0.017105924
8076355	5372	*phosphomannomutase 1*	*PMM1*	8.76	8.83	7.09	0.004789868	0.01710735
8118093	253018	*HLA complex group 27*	*HCG27*	7.19	7.12	6.61	0.004789775	0.01710735
8135929				7.55	7.47	8.85	0.004788984	0.01710735
8019699	653240, 100132476, 728224	*keratin associated protein 4-11 | keratin associated protein 4-7 | keratin associated protein 4-8*	*KRTAP4-11|KRTAP4-7|KRTAP4-8*	7.32	7.45	6.71	0.004790841	0.017108707
8077185	335	*apolipoprotein A-I*	*APOA1*	7.63	7.67	7.11	0.004792497	0.017111914

8102636	132332, 100192379	*transmembrane protein 155 | hypothetical LOC100192379*	*TMEM155| LOC100192379*	6.15	6.39	5.78	0.004793517	0.017111914
7942877				6.72	6.74	6.13	0.004793465	0.017111914
8117614	8348	*histone cluster 1, H2bo*	*HIST1H2BO*	6.50	6.86	7.87	0.004794538	0.017113442
8064261	4987	*opiate receptor-like 1*	*OPRL1*	7.21	7.27	6.50	0.004796657	0.01711889
8131965	441204		*LOC441204*	7.01	7.04	6.57	0.004799382	0.017126499
8088642	26018	*leucine-rich repeats and immunoglobulin-like domains 1*	*LRIG1*	8.21	8.34	9.66	0.004801659	0.017132506
7998414	100128785	*hypothetical LOC100128785*	*GS85*	7.34	7.28	6.61	0.004805182	0.01713872
8107090				8.71	8.84	8.27	0.004805123	0.01713872
8171491				5.99	6.05	6.62	0.004804037	0.01713872
7932885	94134	*Rho GTPase activating protein 12*	*ARHGAP12*	8.66	8.42	9.72	0.004809648	0.017150776
8158918	9329, 64794	*general transcription factor IIIC, polypeptide 4, 90kDa | DEAD (Asp-Glu-Ala-Asp) box polypeptide 31*	*GTF3C4|DDX31*	7.93	7.88	9.34	0.004810344	0.017150776
8081107				4.71	4.75	4.59	0.004810193	0.017150776
8136963	26211	*olfactory receptor, family 2, subfamily F, member 1*	*OR2F1*	6.54	6.48	5.95	0.004812361	0.017154556
7956819	283349	*Ras association (RalGDS/AF-6) domain family member 3*	*RASSF3*	8.03	7.29	8.36	0.004812916	0.017154556
8144947				4.65	4.69	4.52	0.004813187	0.017154556
7903461	22854	*netrin G1*	*NTNG1*	6.77	7.58	7.96	0.004817636	0.017164055
8028737	29124	*lectin, galactoside-binding, soluble, 13*	*LGALS13*	4.94	5.17	4.87	0.004817233	0.017164055
7989953	79719	*alpha- and gamma-adaptin binding protein*	*AAGAB*	7.24	7.44	8.51	0.004817168	0.017164055
8038117	1628	*D site of albumin promoter (albumin D-box) binding protein*	*DBP*	9.60	9.27	8.37	0.004818376	0.017164571
8041900				8.19	8.37	7.49	0.004819155	0.017165229
8167749	29057, 727866	*family with sequence similarity 156, member A | family with sequence similarity 156, member B*	*FAM156A| FAM156B*	8.78	8.78	7.84	0.004820211	0.01716565

8075024	646074, 25812, 29774, 29797, 100294020	*POM121 membrane glycoprotein-like 10, pseudogene | POM121 membrane glycoprotein-like 1, pseudogene | POM121 membrane glycoprotein-like 9, pseudogene | POM121 membrane glycoprotein-like 8 pseudogene | putative POM121-like protein 1-like*	*POM121L10P|POM121L1P|POM121L9P|POM121L8P|LOC100294020*	7.00	6.97	6.34	0.004820462	0.01716565
8128459	6492	*single-minded homolog 1 (Drosophila)*	*SIM1*	5.96	6.61	5.81	0.004822209	0.017169753
7936552	11023	*ventral anterior homeobox 1*	*VAX1*	6.43	6.63	6.04	0.00482403	0.017169882
8049088	64708	*COP9 constitutive photomorphogenic homolog subunit 7B (Arabidopsis)*	*COPS7B*	9.25	9.21	10.10	0.004823023	0.017169882
8011112				6.33	6.19	5.88	0.004823488	0.017169882
7979437	112849	*chromosome 14 open reading frame 149*	*C14orf149*	9.28	8.96	8.18	0.004830124	0.017185219
8043570				7.14	7.18	6.71	0.004829935	0.017185219
8107472				4.92	4.94	4.74	0.004829937	0.017185219
7909603	29937	*neuron derived neurotrophic factor*	*NENF*	10.09	10.19	8.89	0.004830771	0.0171854
7989013	51187	*ribosomal L24 domain containing 1*	*RSL24D1*	8.49	8.74	9.42	0.004831797	0.017185623
8065388	140880	*cystatin 11*	*CST11*	5.51	5.50	5.21	0.004832024	0.017185623
8159096	1621	*dopamine beta-hydroxylase (dopamine beta-monooxygenase)*	*DBH*	7.43	7.49	6.74	0.004834219	0.017191313
8069057	5211	*phosphofructokinase, liver*	*PFKL*	9.85	10.35	8.98	0.004838216	0.017203405
7958398	9671	*WSC domain containing 2*	*WSCD2*	6.99	6.93	6.58	0.004840749	0.017206056
8157141	10881	*actin-like 7A*	*ACTL7A*	5.94	5.88	5.62	0.004839872	0.017206056
8097476				6.76	7.00	6.17	0.00484067	0.017206056
7918725	51592	*tripartite motif-containing 33*	*TRIM33*	8.61	8.20	10.52	0.004845558	0.01722103
8161642				7.02	7.08	6.50	0.00484663	0.017222717
8010696	256933	*neuropeptide B*	*NPB*	8.52	8.54	7.86	0.004848771	0.017228205
8035903				6.70	6.91	6.10	0.00485067	0.017232833
8107671				7.83	7.87	7.05	0.004851812	0.017234767
8130464	729515	*chromosome 6 open reading frame 35*	*C6orf35*	7.48	7.62	8.63	0.004853872	0.017239965

8057574				5.61	5.57	5.17	0.004859432	0.01725759
8070876	23275	*protein O-fucosyltransferase 2*	*POFUT2*	9.37	9.20	8.21	0.004862939	0.017265795
8008511	124857	*WAP, follistatin/kazal, immunoglobulin, kunitz and netrin domain containing 2*	*WFIKKN2*	7.21	7.35	6.80	0.004862602	0.017265795
7970524	253832	*zinc finger, DHHC-type containing 20*	*ZDHHC20*	8.56	8.47	9.25	0.004867788	0.017280884
7983763	5597	*mitogen-activated protein kinase 6*	*MAPK6*	8.19	8.31	10.51	0.004875352	0.017303558
7922504	64326	*ring finger and WD repeat domain 2*	*RFWD2*	7.75	7.91	8.95	0.004875972	0.017303558
8121130				5.82	5.32	7.85	0.00487571	0.017303558
8167165	369	*v-raf murine sarcoma 3611 viral oncogene homolog*	*ARAF*	10.67	10.61	9.41	0.004882886	0.017317384
8031893	7554	*zinc finger protein 8*	*ZNF8*	8.41	8.38	9.31	0.004883468	0.017317384
7984190	9325	*thyroid hormone receptor interactor 4*	*TRIP4*	7.81	7.93	8.58	0.004882897	0.017317384
7961983	51768	*transmembrane 7 superfamily member 3*	*TM7SF3*	10.24	10.03	11.64	0.004881845	0.017317384
7913918	91544	*UBX domain protein 11*	*UBXN11*	8.01	7.81	6.93	0.004882561	0.017317384
7972442				6.82	6.88	6.34	0.004882712	0.017317384
7970404	93492	*transmembrane phosphoinositide 3-phosphatase and tensin homolog 2*	*TPTE2*	5.43	5.54	5.19	0.004886827	0.017327169
8154856	54926	*ubiquitin-conjugating enzyme E2R 2*	*UBE2R2*	11.81	11.78	11.00	0.00489039	0.017337672
8075332	83874	*TBC1 domain family, member 10A*	*TBC1D10 A*	8.92	9.31	7.78	0.00489163	0.017339941
8008052	6668	*Sp2 transcription factor*	*SP2*	8.76	8.82	7.61	0.004894889	0.017347232
7981106	145258	*goosecoid homeobox*	*GSC*	7.87	8.13	7.23	0.004894743	0.017347232
8168650	140886	*poly(A) binding protein, cytoplasmic 5*	*PABPC5*	6.99	7.14	6.49	0.004904664	0.017377608
7937612	727897	*mucin 5B, oligomeric mucus/gel-forming*	*MUC5B*	7.02	7.06	6.56	0.004904606	0.017377608
8101673				5.82	6.35	5.06	0.004906453	0.017381813
7906458	325	*amyloid P component, serum*	*APCS*	5.86	5.89	5.55	0.004908052	0.017382246
8087640	11186	*Ras association (RalGDS/AF-6) domain family member 1*	*RASSF1*	8.98	8.89	7.36	0.004908382	0.017382246
8011375	7442, 23729	*transient receptor potential cation channel, subfamily V, member 1 | sedoheptulokinase*	*TRPV1|SH PK*	7.73	7.69	7.29	0.004907443	0.017382246
8092358	23101	*MCF.2 cell line derived transforming sequence-like 2*	*MCF2L2*	5.91	5.86	6.71	0.00491296	0.017394193
7896859	406984	*microRNA 200b*	*MIR200B*	7.28	7.36	6.75	0.004912491	0.017394193

8042346	440867		*FLJ16124*	6.05	6.07	5.79	0.004914525	0.0173976
8155073	203260, 84904	*coiled-coil domain containing 107 | chromosome 9 open reading frame 100*	*CCDC107| C9orf100*	8.57	8.83	7.81	0.004915568	0.017399156
8116696	404220	*chromosome 6 open reading frame 201*	*C6orf201*	6.01	5.90	5.57	0.004916285	0.017399563
8051030	8884	*solute carrier family 5 (sodium-dependent vitamin transporter), member 6*	*SLC5A6*	8.48	8.41	10.04	0.004926581	0.017433863
7977841	54930	*HAUS augmin-like complex, subunit 4*	*HAUS4*	8.77	8.38	9.49	0.004929604	0.017442016
8097747				5.96	6.02	5.51	0.004930093	0.017442016
8173338				5.92	5.38	5.17	0.004931469	0.017444748
8032088	400668	*protease, serine-like 1*	*PRSSL1*	8.23	8.35	7.47	0.004932586	0.01744656
7949553	117144	*cation channel, sperm associated 1*	*CATSPER 1*	6.60	6.67	6.15	0.004933677	0.01744828
8112428	4064		*CD180*	4.97	4.98	4.78	0.004935432	0.017450213
8113301				4.69	4.71	4.57	0.004934873	0.017450213
7995352				9.07	9.09	8.70	0.004936215	0.017450843
8022043				8.20	7.81	8.01	0.004937086	0.017451787
8114287	6695	*sparc/osteonectin, cwcv and kazal-like domains proteoglycan (testican) 1*	*SPOCK1*	9.82	10.65	7.48	0.004939718	0.017458951
7966427	8315	*BRCA1 associated protein*	*BRAP*	8.48	8.71	9.42	0.004943693	0.017464165
7970806	728437	*hCG2020170*	*LOC72843 7*	6.46	6.45	5.98	0.0049428	0.017464165
7919193	440672, 11163	*nudix (nucleoside diphosphate linked moiety X)-type motif 4 pseudogene 1*	*NUDT4P1*	11.51	11.30	12.10	0.004943647	0.017464165
7982812	84936, 55192	*zinc finger, FYVE domain containing 19 | DnaJ (Hsp40) homolog, subfamily C, member 17*	*ZFYVE19| DNAJC17*	7.90	7.93	7.01	0.004944823	0.017464165
7943709				4.60	4.51	4.66	0.004941943	0.017464165
7974027				5.20	5.20	4.90	0.004944223	0.017464165
7973743	664	*BCL2/adenovirus E1B 19kDa interacting protein 3*	*BNIP3*	5.67	6.26	5.08	0.004947812	0.017470777
7899273	10726	*nuclear distribution gene C homolog (A. nidulans)*	*NUDC*	8.74	8.53	9.63	0.00494851	0.017470777
7994445	25970	*SH2B adaptor protein 1*	*SH2B1*	7.87	7.76	6.75	0.004948437	0.017470777
8092095	23043	*TRAF2 and NCK interacting kinase*	*TNIK*	7.23	6.36	8.91	0.004950035	0.017472407
7925902				5.39	5.40	5.01	0.004950183	0.017472407

7959282	80196	*ring finger protein 34*	*RNF34*	8.55	8.62	9.69	0.004952098	0.01747703
8149283	94137	*retinitis pigmentosa 1-like 1*	*RP1L1*	6.63	6.68	6.21	0.004954593	0.0174837
8060604	5020	*oxytocin, prepropeptide*	*OXT*	8.74	8.67	7.59	0.004957898	0.01748681
8051573	10602	*CDC42 effector protein (Rho GTPase binding) 3*	*CDC42EP 3*	11.18	11.49	9.31	0.004957123	0.01748681
8123637	4835, 401233	*NAD(P)H dehydrogenase, quinone 2 | HIV-1 Tat specific factor 1 pseudogene*	*NQO2|LO C401233*	6.86	7.10	6.49	0.004956566	0.01748681
7915594				8.81	8.88	9.24	0.004957649	0.01748681
8138789	221895	*JAZF zinc finger 1*	*JAZF1*	10.23	9.38	9.18	0.004960772	0.017494813
7945678	728008	*chromosome 11 open reading frame 89*	*C11orf89*	8.43	8.45	7.70	0.004962612	0.017499164
7997907				6.55	6.76	6.24	0.00496391	0.017501602
7931455	80313	*leucine rich repeat containing 27*	*LRRC27*	7.71	7.73	6.70	0.004966324	0.01750485
8175248	407049	*microRNA 92a-2*	*MIR92A2*	4.56	4.58	4.65	0.004965496	0.01750485
7972709				4.80	4.87	4.66	0.004966651	0.01750485
8133896				11.63	11.70	11.30	0.004968116	0.017507879
7984862	1544	*cytochrome P450, family 1, subfamily A, polypeptide 2*	*CYP1A2*	7.08	7.12	6.60	0.004969108	0.017509237
8148329				7.75	7.83	7.23	0.004972006	0.017517311
8056611	4036	*low density lipoprotein receptor-related protein 2*	*LRP2*	5.72	5.78	6.67	0.004973229	0.017519278
7998055	4157, 10381	*melanocortin 1 receptor (alpha melanocyte stimulating hormone receptor) | tubulin, beta 3*	*MC1R|TU BB3*	7.89	7.83	7.20	0.004973779	0.017519278
8144151				4.94	4.79	5.34	0.004975807	0.017524285
8105013	133584	*EGF-like, fibronectin type III and laminin G domains*	*EGFLAM*	6.08	6.07	5.75	0.004977085	0.017526649
8169645	7319	*ubiquitin-conjugating enzyme E2A (RAD6 homolog)*	*UBE2A*	10.71	10.93	10.40	0.004981888	0.017541422
7979241	652	*bone morphogenetic protein 4*	*BMP4*	10.16	7.43	9.50	0.004983475	0.01754487
7952116	283149	*B-cell CLL/lymphoma 9-like*	*BCL9L*	10.50	10.54	9.03	0.0049842	0.017545283
8162311				6.91	6.71	6.30	0.004985211	0.017546702
8151296	51110	*lactamase, beta 2*	*LACTB2*	7.68	7.18	8.42	0.004987832	0.01754951
8152656	11244, 84933	*zinc fingers and homeoboxes 1 | chromosome 8 open reading frame 76*	*ZHX1|C8o rf76*	9.09	8.67	7.78	0.004987208	0.01754951
7901714				6.28	6.20	5.90	0.0049874	0.01754951

7923956				6.72	6.91	6.39	0.004988652	0.017550254
8109926	2568	*gamma-aminobutyric acid (GABA) A receptor, pi*	*GABRP*	6.23	6.19	7.39	0.004990886	0.017554944
8053890	56910	*StAR-related lipid transfer (START) domain containing 7*	*STARD7*	10.63	10.69	11.58	0.004991201	0.017554944
7947425	966		*CD59*	11.63	11.69	10.14	0.00499241	0.017557058
8155537				6.29	6.31	5.80	0.004993315	0.017558102
8049799	50636	*anoctamin 7*	*ANO7*	7.31	7.42	6.85	0.004994619	0.017558778
8032023	51298	*Theg homolog (mouse)*	*THEG*	6.88	7.11	6.59	0.004995332	0.017558778
7976726	51466	*Enah/Vasp-like*	*EVL*	9.35	9.33	8.25	0.004995193	0.017558778
8019693	256933	*neuropeptide B*	*NPB*	8.51	8.54	7.86	0.004996407	0.017560419
7906527	480	*ATPase, Na+/K+ transporting, alpha 4 polypeptide*	*ATP1A4*	5.89	5.97	5.70	0.004999081	0.017561713
8146986	3174	*hepatocyte nuclear factor 4, gamma*	*HNF4G*	5.58	5.63	5.94	0.004999209	0.017561713
8111387	81792	*ADAM metallopeptidase with thrombospondin type 1 motif, 12*	*ADAMTS12*	10.02	8.47	8.28	0.004999199	0.017561713
8020254	81929	*SEH1-like (S. cerevisiae)*	*SEH1L*	8.12	8.26	9.80	0.004998585	0.017561713
7923837	148811	*peptidase M20 domain containing 1*	*PM20D1*	6.88	6.95	6.50	0.00500186	0.017568888
7917798	24	*ATP-binding cassette, sub-family A (ABC1), member 4*	*ABCA4*	6.10	6.24	5.73	0.005010592	0.017595278
7908812	9283	*G protein-coupled receptor 37 like 1*	*GPR37L1*	8.30	8.43	7.81	0.005010592	0.017595278
8159790	100287171, 375260, 374666, 653635, 375690, 100288778, 100134445	*WAS protein family homolog 1 | WAS protein family homolog 2 pseudogene | WAS protein family homolog 3 pseudogene | WAS protein family homolog 7 pseudogene | WAS protein family homolog 5 pseudogene | WAS protein family homolog 1 pseudogene | hypothetical LOC100134445*	*WASH1|WASH2P|WASH3P|WASH7P|WASH5P|LOC100288778|LOC100134445*	9.90	9.70	8.87	0.005011309	0.017595656
8066345				5.31	5.35	4.99	0.005012013	0.017595986
8116162	5626	*PROP paired-like homeobox 1*	*PROP1*	8.08	8.07	7.19	0.005015719	0.017606856
8043791	3631	*inositol polyphosphate-4-phosphatase, type I, 107kDa*	*INPP4A*	8.44	8.45	9.11	0.005016848	0.01760868
7915331				6.72	6.80	6.20	0.005018754	0.017611112
7919560				7.86	7.85	9.76	0.005019115	0.017611112

7973054				9.42	9.68	8.72	0.005019371	0.017611112
8044263	5903	*RAN binding protein 2*	*RANBP2*	7.49	7.49	10.10	0.005020388	0.01761254
8136248	4232	*mesoderm specific transcript homolog (mouse)*	*MEST*	8.62	10.04	12.09	0.00502135	0.017613772
7992758	79412	*kringle containing transmembrane protein 2*	*KREMEN2*	8.88	8.97	8.31	0.005025142	0.017624233
7997228				5.50	5.51	5.02	0.005025553	0.017624233
8161026	84904, 203260	*chromosome 9 open reading frame 100 | coiled-coil domain containing 107*	*C9orf100| CCDC107*	7.13	7.30	8.05	0.005028076	0.017628796
8176663				5.33	5.33	5.12	0.005027844	0.017628796
8056812	29789	*Obg-like ATPase 1*	*OLA1*	10.13	9.96	11.24	0.005031199	0.017637605
7977033	81693	*amnionless homolog (mouse)*	*AMN*	8.17	8.24	7.60	0.005031854	0.01763776
8086057	7342	*upstream binding protein 1 (LBP-1a)*	*UBP1*	9.51	9.60	10.47	0.005035101	0.017646998
8094830	55161	*transmembrane protein 33*	*TMEM33*	9.10	9.19	10.18	0.005036027	0.0176481
7933842	219623	*transmembrane protein 26*	*TMEM26*	7.26	5.86	5.69	0.005037373	0.017650677
8065185	58495	*ovo-like 2 (Drosophila)*	*OVOL2*	6.42	6.45	6.86	0.005040069	0.01765798
7982957	23269	*MAX gene associated*	*MGA*	7.41	7.28	9.42	0.0050431	0.017665183
7918034	51611		*DPH5*	7.79	7.78	9.30	0.005043349	0.017665183
7937560	4583	*mucin 2, oligomeric mucus/gel-forming*	*MUC2*	7.17	7.24	6.64	0.005045879	0.017670467
7925028	375061	*family with sequence similarity 89, member A*	*FAM89A*	7.59	7.54	6.99	0.005046694	0.017670467
7927367	653145, 728113 244	*annexin A8 | annexin A8-like 1 | annexin A8-like 2*	*ANXA8|ANXA8L1|ANXA8L2*	6.80	6.74	6.38	0.005046661	0.017670467
8151952	79815	*NIPA-like domain containing 2*	*NIPAL2*	9.90	9.12	6.49	0.005048989	0.01767636
8007483	113277, 728772	*transmembrane protein 106A | hypothetical LOC728772*	*TMEM106A|FLJ77644*	9.87	9.43	7.82	0.005051551	0.017683186

8044649	375260, 374666, 653635, 100287171, 375690, 100288778, 100134445	*WAS protein family homolog 2 pseudogene | WAS protein family homolog 3 pseudogene | WAS protein family homolog 7 pseudogene | WAS protein family homolog 1 | WAS protein family homolog 5 pseudogene | WAS protein family homolog 1 pseudogene | hypothetical LOC100134445*	*WASH2P|WASH3P|WASH7P|WASH1|WASH5P|LOC100288778|LOC100134445*	9.86	9.65	8.84	0.005054965	0.017691176
7914260	4146, 100129196	*matrilin 1, cartilage matrix protein | hypothetical LOC100129196*	*MATN1|LOC100129196*	6.43	6.52	5.96	0.00505506	0.017691176
8036004	1050	*CCAAT/enhancer binding protein (C/EBP), alpha*	*CEBPA*	7.26	7.43	6.76	0.00505728	0.017696802
7915200				5.47	5.31	5.11	0.005058771	0.017699873
7898679	84224	*neuroblastoma breakpoint family, member 3*	*NBPF3*	8.75	8.05	7.37	0.005068597	0.017732104
8027665	126374	*Wilms tumor 1 interacting protein*	*WTIP*	9.07	9.15	7.93	0.005069575	0.017733376
8110084	4488	*msh homeobox 2*	*MSX2*	8.26	7.64	7.09	0.005072536	0.017741583
8094872				6.92	6.88	6.32	0.005073755	0.0177437
7952490	9638	*fasciculation and elongation protein zeta 1 (zygin I)*	*FEZ1*	9.65	9.26	8.37	0.005077019	0.017750813
8059580	92737	*delta/notch-like EGF repeat containing*	*DNER*	6.82	6.73	7.75	0.005076558	0.017750813
8062213	51230	*PHD finger protein 20*	*PHF20*	7.40	7.41	9.08	0.005080984	0.017762525
7908351	5321	*phospholipase A2, group IVA (cytosolic, calcium-dependent)*	*PLA2G4A*	5.57	5.34	5.95	0.005082136	0.0177644
8165637	727800	*ring finger protein 208*	*RNF208*	8.49	8.24	7.46	0.00508745	0.017780824
7896878	126792	*UDP-Gal:betaGal beta 1,3-galactosyltransferase polypeptide 6*	*B3GALT6*	9.01	9.12	8.03	0.005088479	0.017782265
8142795	3614	*IMP (inosine 5'-monophosphate) dehydrogenase 1*	*IMPDH1*	9.31	9.60	8.10	0.005093537	0.01779348
8057615	116093	*disrupted in renal carcinoma 1*	*DIRC1*	6.21	6.80	5.58	0.005093061	0.01779348
7979806				5.20	5.21	4.95	0.005093222	0.01779348
8053668	9451	*eukaryotic translation initiation factor 2-alpha kinase 3*	*EIF2AK3*	7.37	7.14	8.49	0.005099503	0.017812166
8109383	2890	*glutamate receptor, ionotropic, AMPA 1*	*GRIA1*	6.43	8.55	7.60	0.005100186	0.017812395
8045882	92196	*death associated protein-like 1*	*DAPL1*	5.87	5.91	5.57	0.005100868	0.017812623

8034249	115948	*coiled-coil domain containing 151*	*CCDC151*	7.08	7.08	6.70	0.005101551	0.017812856
8035765	7561	*zinc finger protein 14*	*ZNF14*	5.88	5.78	6.74	0.0051035	0.017817505
7955055	55652	*solute carrier family 48 (heme transporter), member 1*	*SLC48A1*	9.86	9.60	8.38	0.005108244	0.01782368
8148615	79943	*zinc finger protein 696*	*ZNF696*	7.35	7.46	7.01	0.005106261	0.01782368
8101916	93587	*RNA (guanine-9-) methyltransferase domain containing 2*	*RG9MTD2*	6.16	6.16	6.45	0.005107909	0.01782368
8059965	394261	*chromosome 2 open reading frame 19*	*C2orf19*	5.80	5.92	5.18	0.005106912	0.01782368
8125772	401253	*chromosome 6 open reading frame 227*	*C6orf227*	7.12	7.21	6.57	0.005108356	0.01782368
8106025	55814	*B double prime 1, subunit of RNA polymerase III transcription initiation factor IIIB*	*BDP1*	7.79	7.62	9.43	0.005109855	0.017826756
7981714				7.68	7.61	6.70	0.00511265	0.017834352
8033414	126006	*Purkinje cell protein 2*	*PCP2*	7.94	7.93	7.40	0.005114792	0.017839668
7967493	9612	*nuclear receptor corepressor 2*	*NCOR2*	10.29	10.11	8.81	0.005121439	0.017860694
8105456	133383	*chromosome 5 open reading frame 35*	*C5orf35*	6.06	6.46	6.96	0.005125132	0.01786943
8174853	728096, 653282, 728036, 255313, 728090, 728082, 728075, 728072, 728049, 728042, 728062	*cancer/testis antigen family 47, member A1 | cancer/testis antigen family 47, member A7 | cancer/testis antigen family 47, member A10 | cancer/testis antigen family 47, member A11 | cancer/testis antigen family 47, member A2 | cancer/testis antigen family 47, member A3 | cancer/testis antigen family 47, member A4 | cancer/testis antigen family 47, member A5 | cancer/testis antigen family 47, member A8 | cancer/testis antigen family 47, member A9 | cancer/testis antigen family 47, member A6*	*CT47A1|CT47A7|CT47A10|CT47A11|CT47A2|CT47A3|CT47A4|CT47A5|CT47A8|CT47A9|CT47A6*	7.14	7.25	6.82	0.005125182	0.01786943
7930833	338567	*potassium channel, subfamily K, member 18*	*KCNK18*	6.43	6.52	6.03	0.00512668	0.017872494
8113489				6.18	5.91	5.83	0.005127483	0.017873134
7919251	51463, 653519, 728932	*G protein-coupled receptor 89B | G protein-coupled receptor 89A | G protein-coupled receptor 89C*	*GPR89B|GPR89A|GPR89C*	7.73	7.76	9.11	0.005129377	0.017877366
8116571				7.13	7.24	7.80	0.005129935	0.017877366

7928882	10974, 119385	*chromosome 10 open reading frame 116 | ankyrin repeat and GTPase domain Arf GTPase activating protein 11*	*C10orf116|AGAP11*	10.18	9.25	7.13	0.005130651	0.017877702
8141206	55971	*BAI1-associated protein 2-like 1*	*BAIAP2L1*	9.31	9.49	10.71	0.005134373	0.01788851
8085220	10474	*transcriptional adaptor 3*	*TADA3*	10.29	10.45	9.23	0.005137254	0.01789639
7963121	57701	*NCK-associated protein 5-like*	*NCKAP5L*	8.41	8.72	7.57	0.005139551	0.017902233
7958784	217	*aldehyde dehydrogenase 2 family (mitochondrial)*	*ALDH2*	11.33	10.02	10.15	0.005143994	0.017911224
7958379	11137		*PWP1*	9.09	9.10	10.08	0.005143638	0.017911224
7944972				6.49	6.53	6.04	0.005142759	0.017911224
7962625	10411	*Rap guanine nucleotide exchange factor (GEF) 3*	*RAPGEF3*	7.30	7.36	6.64	0.005145261	0.017913474
8150818	6917	*transcription elongation factor A (SII), 1*	*TCEA1*	10.12	10.37	11.14	0.005147867	0.017920388
8137332	11194	*ATP-binding cassette, sub-family B (MDR/TAP), member 8*	*ABCB8*	7.90	7.85	7.07	0.005152972	0.017931912
7975901	145497	*chromosome 14 open reading frame 166B*	*C14orf166B*	5.95	5.91	5.62	0.005153041	0.017931912
8092295	339829	*coiled-coil domain containing 39*	*CCDC39*	4.82	4.90	5.16	0.005152213	0.017931912
8169676	84528, 727940	*Rhox homeobox family, member 2 | Rhox homeobox family, member 2B*	*RHOXF2|RHOXF2B*	5.86	5.84	5.60	0.005153835	0.017932512
8085026	6165	*ribosomal protein L35a*	*RPL35A*	12.89	12.91	13.19	0.005155429	0.017933283
7932498	387640, 219688	*chromosome 10 open reading frame 140 | hypothetical protein LOC219688*	*C10orf140|LOC219688*	7.10	7.08	7.72	0.005155416	0.017933283
7926403	653567, 100289299	*family with sequence similarity 23, member A | family with sequence similarity 23, member B*	*FAM23A|FAM23B*	6.00	6.01	5.92	0.00515592	0.017933283
7905691	6232	*ribosomal protein S27*	*RPS27*	12.74	12.77	12.54	0.00515819	0.017937224
7980720	145567	*tetratricopeptide repeat domain 7B*	*TTC7B*	9.87	10.11	8.98	0.005158295	0.017937224
8147030	11075	*stathmin-like 2*	*STMN2*	5.85	10.76	7.12	0.005162785	0.01794635
7926674	256297	*pancreas specific transcription factor, 1a*	*PTF1A*	7.71	7.71	7.21	0.005162565	0.01794635
8040221	100131506		*LOC100131506*	6.51	6.48	6.05	0.005161873	0.01794635
7968746	11193	*WW domain binding protein 4 (formin binding protein 21)*	*WBP4*	6.71	6.67	7.58	0.005169633	0.017963665

7964388	56901	*NADH dehydrogenase (ubiquinone) 1 alpha subcomplex, 4-like 2*	*NDUFA4L 2*	9.21	9.38	7.77	0.005168692	0.017963665
7937868	283303	*chromosome 11 open reading frame 36*	*C11orf36*	6.06	6.14	5.67	0.005169149	0.017963665
7914950	1441	*colony stimulating factor 3 receptor (granulocyte)*	*CSF3R*	6.24	6.20	5.76	0.005171308	0.017967323
8151711	4683	*nibrin*	*NBN*	8.45	8.24	9.19	0.005173854	0.017972035
7951008	79101, 677805, 684959, 85459	*TATA box binding protein (TBP)-associated factor, RNA polymerase I, D, 41kDa | small nucleolar RNA, H/ACA box 18 | small nucleolar RNA, H/ACA box 25 | KIAA1731*	*TAF1D|SNORA18|SNORA25|KIAA1731*	9.52	9.52	10.80	0.00517391	0.017972035
8076978	5600	*mitogen-activated protein kinase 11*	*MAPK11*	7.57	7.68	6.94	0.005176899	0.017980257
8058866	7141	*transition protein 1 (during histone to protamine replacement)*	*TNP1*	6.36	6.36	5.79	0.005179914	0.0179864
8106532				6.08	6.43	5.54	0.005179695	0.0179864
8165492	727800	*ring finger protein 208*	*RNF208*	8.49	8.24	7.46	0.005184337	0.017997857
8013531				4.76	5.06	4.81	0.00518446	0.017997857
8039504	729956	*shisa homolog 7 (Xenopus laevis)*	*SHISA7*	8.87	8.98	8.14	0.005185734	0.018000113
8155110	9827, 692094	*RGP1 retrograde golgi transport homolog (S. cerevisiae) | microseminoprotein, prostate associated*	*RGP1|MSMP*	9.22	9.24	8.57	0.005186428	0.01800036
7904930	51463, 653519, 728932	*G protein-coupled receptor 89B | G protein-coupled receptor 89A | G protein-coupled receptor 89C*	*GPR89B|GPR89A|GPR89C*	7.78	7.81	9.14	0.005187165	0.018000754
7951865	335	*apolipoprotein A-I*	*APOA1*	7.61	7.63	7.06	0.005189742	0.01800489
8174710	6170	*ribosomal protein L39*	*RPL39*	11.74	11.88	11.70	0.005189814	0.01800489
7937713	90019	*synaptotagmin VIII*	*SYT8*	7.98	7.96	7.37	0.005190228	0.01800489
8032380	55643	*BTB (POZ) domain containing 2*	*BTBD2*	11.88	11.65	10.70	0.005192554	0.018010793
7970162	23250	*ATPase, class VI, type 11A*	*ATP11A*	7.54	7.66	8.55	0.005195156	0.018017653
8040173	9014	*TATA box binding protein (TBP)-associated factor, RNA polymerase I, B, 63kDa*	*TAF1B*	8.32	8.23	9.25	0.005197791	0.01801892
8120402	9532	*BCL2-associated athanogene 2*	*BAG2*	9.78	9.99	9.07	0.005197943	0.01801892
8100292	285527	*FRY-like*	*FRYL*	6.85	6.60	8.30	0.005198018	0.01801892
8126946				6.62	6.28	6.11	0.005198011	0.01801892
8174092	9823	*armadillo repeat containing, X-linked 2*	*ARMCX2*	7.94	8.03	8.79	0.005199873	0.01802319

8067270	164284	*adenomatosis polyposis coli down-regulated 1-like*	*APCDD1L*	8.65	9.31	7.81	0.005203437	0.018033374
7950906	1075	*cathepsin C*	*CTSC*	11.29	10.05	12.50	0.005208293	0.01804804
8136839	5304	*prolactin-induced protein*	*PIP*	5.74	5.59	5.46	0.005210096	0.01805212
8133788	5782	*protein tyrosine phosphatase, non-receptor type 12*	*PTPN12*	8.90	9.12	10.79	0.005212374	0.018055294
8103206	55294	*F-box and WD repeat domain containing 7*	*FBXW7*	6.62	6.66	7.71	0.00521263	0.018055294
7993453	255027	*MPV17 mitochondrial membrane protein-like*	*MPV17L*	6.91	6.82	8.23	0.005212888	0.018055294
7998466	64718	*unkempt homolog (Drosophila)-like*	*UNKL*	8.31	8.38	7.78	0.005213679	0.018055866
7899504	10691	*glucocorticoid modulatory element binding protein 1*	*GMEB1*	8.32	8.35	9.34	0.005219787	0.018070517
7963333	144501	*keratin 80*	*KRT80*	6.38	6.59	6.01	0.005219604	0.018070517
8046522				5.29	5.35	5.04	0.005218835	0.018070517
8110183	54825	*cadherin-related family member 2*	*CDHR2*	6.56	6.50	6.07	0.005221984	0.018075956
8060094	150677	*otospiralin*	*OTOS*	8.20	8.27	7.83	0.005225143	0.01808472
8039120	126014	*osteoclast associated, immunoglobulin-like receptor*	*OSCAR*	7.18	7.33	6.58	0.005229896	0.018099
7977058	1983	*eukaryotic translation initiation factor 5*	*EIF5*	9.56	9.82	10.93	0.005232515	0.018102935
7983051	51332, 100289090	*spectrin, beta, non-erythrocytic 5 | hypothetical protein LOC100289090*	*SPTBN5|LOC100289090*	6.40	6.34	5.81	0.005232914	0.018102935
7957275				10.38	10.51	9.50	0.005232757	0.018102935
8121782				10.80	10.83	10.23	0.005234187	0.01810517
8066009	9584	*RNA binding motif protein 39*	*RBM39*	9.74	9.57	11.08	0.005240657	0.018118866
7923516	51706	*cytochrome b5 reductase 1*	*CYB5R1*	9.32	9.23	7.84	0.005240421	0.018118866
8007208	94086	*heat shock protein, alpha-crystallin-related, B9*	*HSPB9*	6.19	6.29	5.76	0.005239436	0.018118866
8058462	130752	*malate dehydrogenase 1B, NAD (soluble)*	*MDH1B*	5.67	5.45	5.92	0.005240212	0.018118866
8119338	2740	*glucagon-like peptide 1 receptor*	*GLP1R*	6.95	7.03	7.44	0.005242146	0.018120421
8090938	199221	*DAZ interacting protein 1-like*	*DZIP1L*	6.99	7.03	6.57	0.005242362	0.018120421
7946676				7.45	7.31	6.75	0.005243888	0.018123526
7976874				5.09	5.00	4.67	0.005244596	0.018123804
8015236	84616	*keratin associated protein 4-4*	*KRTAP4-4*	7.87	8.08	7.29	0.005246659	0.01812876
7897753	284498	*chromosome 1 open reading frame 167*	*C1orf167*	7.43	7.40	6.88	0.005248162	0.018131785

7992861				6.49	6.51	8.15	0.005251303	0.018140467
7948864	6811	*syntaxin 5*	*STX5*	10.18	10.24	8.91	0.005254834	0.018148068
7998033	22980	*transcription factor 25 (basic helix-loop-helix)*	*TCF25*	10.11	9.95	8.95	0.00525539	0.018148068
8170553	4105, 4102	*melanoma antigen family A, 6 | melanoma antigen family A, 3*	*MAGEA6| MAGEA3*	7.82	7.90	7.36	0.005254635	0.018148068
7981573	2972		*BRF1*	8.69	8.47	7.68	0.005256632	0.01814899
8096839	345193	*leucine-rich repeat, immunoglobulin-like and transmembrane domains 3*	*LRIT3*	5.32	5.37	5.15	0.005256914	0.01814899
7948470	11251	*G protein-coupled receptor 44*	*GPR44*	8.08	8.10	7.41	0.005260828	0.01816033
8102311	839	*caspase 6, apoptosis-related cysteine peptidase*	*CASP6*	6.58	6.79	7.88	0.005262126	0.01816094
7925419	57829	*zona pellucida glycoprotein 4*	*ZP4*	5.75	5.83	5.46	0.005262263	0.01816094
8157058	83856	*fibronectin type III and SPRY domain containing 1-like*	*FSD1L*	5.43	5.50	6.15	0.005263151	0.018161302
8077877	100130924		*LOC10013 0924*	6.47	6.39	6.05	0.005263626	0.018161302
8065683	6640	*syntrophin, alpha 1 (dystrophin-associated protein A1, 59kDa, acidic component)*	*SNTA1*	8.67	9.10	7.57	0.00526691	0.018170461
7926661	22921	*methionine sulfoxide reductase B2*	*MSRB2*	10.73	10.79	9.44	0.005272146	0.018186351
8126629	54676	*GTP binding protein 2*	*GTPBP2*	10.23	11.10	9.72	0.005273191	0.018187786
8132962	677803	*small nucleolar RNA, H/ACA box 15*	*SNORA15*	5.38	5.38	5.74	0.005275053	0.01819203
8016213				7.34	7.61	6.11	0.005276942	0.018196374
7970096	3621	*inhibitor of growth family, member 1*	*ING1*	7.60	7.57	8.00	0.005279009	0.018199326
8027760	5348	*FXYD domain containing ion transport regulator 1*	*FXYD1*	9.04	8.39	7.29	0.005279059	0.018199326
8074714	25812, 646074, 29797, 29774, 100294020	*POM121 membrane glycoprotein-like 1, pseudogene | POM121 membrane glycoprotein-like 10, pseudogene | POM121 membrane glycoprotein-like 8 pseudogene | POM121 membrane glycoprotein-like 9, pseudogene | putative POM121-like protein 1-like*	*POM121L 1P|POM12 1L10P|PO M121L8P| POM121L 9P|LOC10 0294020*	6.95	6.89	6.31	0.005284557	0.018213928

8149361	441317, 389633, 55138, 441315, 441324, 441327, 441328, 645572	*family with sequence similarity 90, member A7 | family with sequence similarity 90 pseudogene | family with sequence similarity 90, member A1 | family with sequence similarity 90, member A5 | family with sequence similarity 90, member A8 | family with sequence similarity 90, member A9 | family with sequence similarity 90, member A10 | family with sequence similarity 90, member A23 pseudogene*	*FAM90A7|FAM90A25P|FAM90A1|FAM90A5|FAM90A8|FAM90A9|FAM90A10|FAM90A23*	7.19	7.18	6.65	0.005284337	0.018213928
7907211				8.04	8.38	7.40	0.005287008	0.018220201
7909104	5129	*cyclin-dependent kinase 18*	*CDK18*	7.32	7.42	8.26	0.005291015	0.01822966
8143461				8.09	7.86	7.54	0.005290392	0.01822966
8063761	60437	*cadherin 26*	*CDH26*	6.22	6.27	6.41	0.005292259	0.018231768
8143661				6.86	7.08	6.56	0.005297535	0.018247766
8172520	7030	*transcription factor binding to IGHM enhancer 3*	*TFE3*	10.82	10.72	9.45	0.005298832	0.01825006
8095074	170825	*GS homeobox 2*	*GSX2*	8.12	8.29	7.68	0.005301399	0.018256636
8006679				6.89	7.08	6.46	0.005302007	0.018256636
7979510	51804	*SIX homeobox 4*	*SIX4*	9.69	8.64	10.37	0.005302958	0.018257733
8133036				5.20	5.23	4.86	0.005304154	0.018259672
8083854	5584	*protein kinase C, iota*	*PRKCI*	8.43	8.04	10.74	0.005309207	0.018271638
8090664	28990	*asteroid homolog 1 (Drosophila)*	*ASTE1*	6.32	6.49	7.06	0.005309588	0.018271638
7945974	143501	*chromosome 11 open reading frame 40*	*C11orf40*	6.51	6.42	6.02	0.005309818	0.018271638
8069161	386683	*keratin associated protein 12-3*	*KRTAP12-3*	7.73	7.81	7.10	0.005310161	0.018271638
8030015	23546	*synaptogyrin 4*	*SYNGR4*	7.65	7.67	7.22	0.00531883	0.01829751
7942774	282679	*aquaporin 11*	*AQP11*	7.33	7.02	6.81	0.005318948	0.01829751
7954783				5.33	5.43	4.96	0.005324521	0.0183145
8155864	6096	*RAR-related orphan receptor B*	*RORB*	6.15	6.11	6.80	0.005327407	0.018322242
8170786	643736	*lung carcinoma-associated protein 10*	*LCA10*	7.39	7.48	6.78	0.005328064	0.01832232
8081254				6.00	6.02	6.56	0.005330447	0.018328331
8157751	9355	*LIM homeobox 2*	*LHX2*	6.93	6.96	6.35	0.005335896	0.018344883

8009713	347741	*otopetrin 3*	*OTOP3*	6.88	6.82	6.38	0.005344313	0.018371632
8037688	81492	*radial spoke head 6 homolog A (Chlamydomonas)*	*RSPH6A*	7.38	7.46	6.93	0.005346116	0.01837564
8146278	84197	*protein kinase-like protein SgK196*	*SGK196*	8.72	8.55	10.28	0.005347697	0.01837889
8056784	1746	*distal-less homeobox 2*	*DLX2*	8.14	7.87	6.54	0.005352408	0.018392455
8006477	91603	*zinc finger protein 830*	*ZNF830*	7.18	7.39	8.40	0.005353095	0.018392455
7961483	121504	*histone cluster 4, H4*	*HIST4H4*	6.15	6.13	6.89	0.005353556	0.018392455
8041172	165186	*family with sequence similarity 179, member A*	*FAM179A*	6.82	6.73	6.44	0.005355524	0.01839703
8127563	1303	*collagen, type XII, alpha 1*	*COL12A1*	11.76	12.29	8.74	0.005361315	0.01841035
8048432	1593	*cytochrome P450, family 27, subfamily A, polypeptide 1*	*CYP27A1*	9.24	8.14	6.88	0.005361158	0.01841035
7898448	23569	*peptidyl arginine deiminase, type IV*	*PADI4*	6.75	6.79	6.17	0.00536066	0.01841035
7919556				7.91	7.88	9.74	0.005368316	0.018432196
8153937	83482	*scratch homolog 1, zinc finger protein (Drosophila)*	*SCRT1*	7.05	7.01	6.60	0.005370137	0.018434066
8142971	136259	*Kruppel-like factor 14*	*KLF14*	8.15	8.34	7.34	0.005369574	0.018434066
8046502	79634	*secernin 3*	*SCRN3*	8.80	8.54	7.64	0.005377693	0.018453406
7973826	80224	*nucleotide binding protein-like*	*NUBPL*	6.72	6.68	8.19	0.005378328	0.018453406
8062016	83658	*dynein, light chain, roadblock-type 1*	*DYNLRB1*	10.32	10.24	9.65	0.00537802	0.018453406
8174750	84528, 727940	*Rhox homeobox family, member 2 | Rhox homeobox family, member 2B*	*RHOXF2| RHOXF2B*	5.86	5.84	5.59	0.005378165	0.018453406
8028656	64857	*pleckstrin homology domain containing, family G (with RhoGef domain) member 2*	*PLEKHG2*	9.45	9.41	8.33	0.005381852	0.018454527
8156404	84270	*chromosome 9 open reading frame 89*	*C9orf89*	9.37	9.67	8.32	0.005381522	0.018454527
8141522	402573	*chromosome 7 open reading frame 61*	*C7orf61*	7.43	7.44	6.75	0.005380159	0.018454527
7992136				7.24	7.23	6.74	0.005379504	0.018454527
8009524				5.92	5.98	5.58	0.005380943	0.018454527
8080621	285331	*coiled-coil domain containing 66*	*CCDC66*	6.97	6.90	7.71	0.005384739	0.018462237
7953094	775	*calcium channel, voltage-dependent, L type, alpha 1C subunit*	*CACNA1C*	7.69	7.14	6.23	0.005386564	0.018466301
8132413				5.85	5.31	6.65	0.005389725	0.018474942
8155598				5.28	5.27	4.92	0.005395237	0.018491643

7900538				8.25	8.31	7.55	0.005397603	0.018497555
8015681	2826	*chemokine (C-C motif) receptor 10*	*CCR10*	8.41	8.39	7.22	0.005401719	0.018505072
8144112	3110	*motor neuron and pancreas homeobox 1*	*MNX1*	8.21	8.25	7.59	0.005401393	0.018505072
8053710				7.94	7.98	7.25	0.005401444	0.018505072
8034983	23476	*bromodomain containing 4*	*BRD4*	10.37	10.31	9.25	0.005403693	0.018509638
7961875	160492	*intermediate filament tail domain containing 1*	*IFLTD1*	5.31	5.29	5.03	0.005407649	0.01852099
7921637	8832		*CD84*	5.64	5.64	5.40	0.005408479	0.018521637
7937039	253738	*early B-cell factor 3*	*EBF3*	7.08	7.68	6.36	0.005410736	0.018524969
8017346	440452, 729873, 84218, 414059, 414060, 654341, 729877, 100134348	*TBC1 domain family, member 3 pseudogene 2 | TBC1 domain family, member 3F | TBC1 domain family, member 3B | TBC1 domain family, member 3C | TBC1 domain family, member 3G | TBC1 domain family, member 3H | similar to TBC1 domain family member 3 (Rab GTPase-activating protein PRC17) (Prostate cancer gene 17 protein) (TRE17 alpha protein)*	*TBC1D3P2|TBC1D3F|TBC1D3B|TBC1D3C|TBC1D3G|TBC1D3H|LOC100134348*	7.97	7.97	7.38	0.005410303	0.018524969
8102592				5.36	5.37	5.03	0.005412027	0.018527193
8025368	6618	*small nuclear RNA activating complex, polypeptide 2, 45kDa*	*SNAPC2*	10.38	10.43	9.15	0.005413743	0.01852867
7977955	90673, 57594	*protein phosphatase 1, regulatory (inhibitor) subunit 3E | homeobox and leucine zipper encoding*	*PPP1R3E|HOMEZ*	7.98	8.05	7.26	0.005413333	0.01852867
7959946	4326	*matrix metallopeptidase 17 (membrane-inserted)*	*MMP17*	8.58	8.37	7.09	0.005418341	0.01854221
7903028				5.05	5.15	4.87	0.00542309	0.018556263
8082774	8419	*beaded filament structural protein 2, phakinin*	*BFSP2*	6.38	6.42	6.09	0.005423997	0.018557167
7925701				6.59	6.70	7.65	0.005425626	0.018560538
8029551	344	*apolipoprotein C-II*	*APOC2*	7.38	7.53	6.86	0.005428493	0.018566387
8070701	1476	*cystatin B (stefin B)*	*CSTB*	11.65	11.59	11.12	0.005428622	0.018566387
7915257	64064	*3-oxoacid CoA transferase 2*	*OXCT2*	7.63	7.58	7.24	0.00543233	0.018576868
8166079	25975	*EGF-like-domain, multiple 6*	*EGFL6*	8.96	6.46	6.66	0.005434764	0.018582992
8068413	874	*carbonyl reductase 3*	*CBR3*	8.33	8.42	7.56	0.00543637	0.018586282

8022814	3178, 664709, 144983	*heterogeneous nuclear ribonucleoprotein A1 | heterogeneous nuclear ribonucleoprotein A1 pseudogene 10 | heterogeneous nuclear ribonucleoprotein A1-like 2*	*HNRNPA1|HNRNPA1P10|HNRNPA1L2*	10.57	10.37	12.84	0.005437895	0.018589294
7967127	10645	*calcium/calmodulin-dependent protein kinase kinase 2, beta*	*CAMKK2*	9.25	9.19	8.30	0.005448022	0.018621705
8103802	132851	*spermatogenesis associated 4*	*SPATA4*	5.44	5.47	5.19	0.005448805	0.018622179
8172739	653219, 653220, 653048, 9503, 653067	*X antigen family, member 1A | X antigen family, member 1B | X antigen family, member 1C | X antigen family, member 1D | X antigen family, member 1E*	*XAGE1A|XAGE1B|XAGE1C|XAGE1D|XAGE1E*	6.08	6.20	5.82	0.005449748	0.018623197
7899955				11.15	11.42	10.59	0.005452089	0.018628992
8177560	55814	*B double prime 1, subunit of RNA polymerase III transcription initiation factor IIIB*	*BDP1*	7.76	7.61	9.43	0.0054539	0.018632976
7978066	8906	*adaptor-related protein complex 1, gamma 2 subunit*	*AP1G2*	8.27	7.74	8.59	0.005456318	0.01863903
7925361	3964, 100287902	*lectin, galactoside-binding, soluble, 8 | hypothetical LOC100287902*	*LGALS8|LOC100287902*	6.65	6.70	6.11	0.005457038	0.018639285
7934083	50674	*neurogenin 3*	*NEUROG3*	6.78	6.86	6.25	0.005459045	0.018643934
8073511				7.50	7.57	6.72	0.0054616	0.018650452
7927179	643236	*transmembrane protein 72*	*TMEM72*	7.31	7.49	6.78	0.005463621	0.018652942
7977294	100128927	*zinc finger and BTB domain containing 42*	*ZBTB42*	7.45	7.98	7.20	0.00546334	0.018652942
8010550	79643	*chromatin modifying protein 6*	*CHMP6*	8.47	8.71	7.48	0.005464958	0.018653095
7932883				7.66	7.60	7.15	0.005464885	0.018653095
8064575	551	*arginine vasopressin*	*AVP*	7.45	7.52	6.81	0.005465803	0.018653773
7919780	55204	*golgi phosphoprotein 3-like*	*GOLPH3L*	8.52	8.40	10.34	0.005470323	0.018666994
8069872	337880	*keratin associated protein 11-1*	*KRTAP11-1*	6.41	6.51	5.85	0.005471367	0.018668348
8158912	56751	*BarH-like homeobox 1*	*BARHL1*	7.52	7.51	6.95	0.005472194	0.018668965
8074473	7122	*claudin 5*	*CLDN5*	7.83	7.94	7.37	0.005474065	0.018673139
8019604	125115	*keratin 40*	*KRT40*	5.90	5.86	5.66	0.005479283	0.018685268

8031908	342945	*zinc finger and SCAN domain containing 22*	*ZSCAN22*	7.58	7.86	6.97	0.005480209	0.018685268
8045142	80097, 55627	*mitotic spindle organizing protein 2B | sphingomyelin phosphodiesterase 4, neutral membrane (neutral sphingomyelinase-3)*	*MZT2B|SMPD4*	11.54	11.64	10.74	0.005478328	0.018685268
8102858				6.94	6.86	6.35	0.005479595	0.018685268
8151413	6921	*transcription elongation factor B (SIII), polypeptide 1 (15kDa, elongin C)*	*TCEB1*	7.14	7.58	7.68	0.005485788	0.01869414
8034806	10212	*DEAD (Asp-Glu-Ala-Asp) box polypeptide 39*	*DDX39*	7.70	8.04	9.16	0.005485857	0.01869414
8002020	51673	*tubulin polymerization-promoting protein family member 3*	*TPPP3*	8.18	8.03	7.01	0.005484086	0.01869414
8007730	79777	*acyl-CoA binding domain containing 4*	*ACBD4*	7.89	7.80	7.14	0.005486049	0.01869414
8123340				6.64	6.66	6.33	0.005485016	0.01869414
7998601	3483	*insulin-like growth factor binding protein, acid labile subunit*	*IGFALS*	8.04	8.06	7.40	0.005489557	0.018703884
8116113	54540	*family with sequence similarity 193, member B*	*FAM193B*	9.03	8.98	8.18	0.005490791	0.01870588
8055173	90557, 91409	*coiled-coil domain containing 74A | coiled-coil domain containing 74B*	*CCDC74A|CCDC74B*	8.61	8.55	7.59	0.005492953	0.01871104
8064208	80331	*DnaJ (Hsp40) homolog, subfamily C, member 5*	*DNAJC5*	8.89	8.91	7.99	0.005494943	0.018713983
7985482	123720	*WAS protein homolog associated with actin, golgi membranes and microtubules*	*WHAMM*	8.36	8.23	7.12	0.005495113	0.018713983
8063389	284751		*LOC284751*	7.76	7.60	6.45	0.005496308	0.018715842
8032829	729359	*perilipin 4*	*PLIN4*	7.30	7.36	6.84	0.005497521	0.018717764
8070853	84536	*chromosome 21 open reading frame 67*	*C21orf67*	7.96	8.12	7.13	0.005501792	0.018730098
8144880	63898	*SH2 domain containing 4A*	*SH2D4A*	9.21	9.35	7.75	0.005503995	0.01873539
8131098	100128374, 100127955	*hypothetical LOC100128374 | hypothetical LOC100127955*	*LOC100128374|LOC100127955*	7.32	7.47	6.72	0.005505195	0.018737266
8060599	64949	*mitochondrial ribosomal protein S26*	*MRPS26*	9.22	9.07	10.03	0.005506437	0.018739283
8052669	9792	*SERTA domain containing 2*	*SERTAD2*	8.32	9.68	7.54	0.00551176	0.018754337
8074168	642477	*hypothetical LOC642477*	*FLJ39632*	7.29	7.20	6.65	0.00551216	0.018754337

7940869	83706	*fermitin family member 3*	*FERMT3*	7.24	7.71	6.80	0.005513395	0.018756326
8052438	339803		*LOC339803*	8.50	8.37	7.61	0.005514795	0.018758882
8001898	1014	*cadherin 16, KSP-cadherin*	*CDH16*	6.45	6.36	5.99	0.005516727	0.018761031
8110289	64324	*nuclear receptor binding SET domain protein 1*	*NSD1*	8.08	7.81	10.06	0.005516092	0.018761031
8059019	1412	*crystallin, beta A2*	*CRYBA2*	6.96	6.91	6.26	0.005520068	0.01877018
7940884	84304	*nudix (nucleoside diphosphate linked moiety X)-type motif 22*	*NUDT22*	8.49	8.53	7.70	0.005522078	0.018774806
8064762	1059	*centromere protein B, 80kDa*	*CENPB*	9.03	9.23	8.07	0.005526295	0.018783579
7928308	54541	*DNA-damage-inducible transcript 4*	*DDIT4*	10.37	10.89	8.57	0.00552661	0.018783579
8055664				6.01	6.04	5.86	0.005525398	0.018783579
7981278	123099	*degenerative spermatocyte homolog 2, lipid desaturase (Drosophila)*	*DEGS2*	8.54	8.60	8.01	0.005529359	0.018790709
7968035	221178	*spermatogenesis associated 13*	*SPATA13*	7.52	7.15	8.11	0.005531419	0.018795494
8100532	84273	*chromosome 4 open reading frame 14*	*C4orf14*	10.33	10.10	10.69	0.005534153	0.018796276
7917548	388646	*guanylate binding protein 7*	*GBP7*	6.09	6.16	5.79	0.00553366	0.018796276
7998119	100287171, 100288778, 375260, 374666, 653635	*WAS protein family homolog 1 | WAS protein family homolog 1 pseudogene | WAS protein family homolog 2 pseudogene | WAS protein family homolog 3 pseudogene | WAS protein family homolog 7 pseudogene*	*WASH1|LOC100288778|WASH2P|WASH3P|WASH7P*	10.15	9.97	9.09	0.005533197	0.018796276
8006377	7756, 693217	*zinc finger protein 207 | microRNA 632*	*ZNF207|MIR632*	9.87	9.86	11.73	0.005534253	0.018796276
7915614	8643	*patched 2*	*PTCH2*	7.61	7.51	6.98	0.005535611	0.018798335
7946073	390063	*olfactory receptor, family 51, subfamily I, member 1*	*OR51I1*	6.10	6.14	5.75	0.005536161	0.018798335
8025973	90592	*zinc finger protein 700*	*ZNF700*	7.27	7.18	7.80	0.005537638	0.018801136
8113658	206338, 100132014	*laeverin | hypothetical protein LOC100132014*	*AQPEP|LOC100132014*	6.71	6.52	6.08	0.005543113	0.018815301
8091554				9.70	9.80	9.00	0.005542649	0.018815301
8117535	8969	*histone cluster 1, H2ag*	*HIST1H2AG*	7.81	8.23	9.34	0.005543903	0.018815767

8179967	177	*advanced glycosylation end product-specific receptor*	*AGER*	7.20	7.26	6.90	0.005547845	0.018824104
8120043	860	*runt-related transcription factor 2*	*RUNX2*	9.31	9.45	7.50	0.005550271	0.018824104
7957633	10988	*methionyl aminopeptidase 2*	*METAP2*	8.96	8.81	10.36	0.005549754	0.018824104
7970999	23111	*spastic paraplegia 20 (Troyer syndrome)*	*SPG20*	8.85	8.34	10.07	0.005549098	0.018824104
8123558	135458	*HUS1 checkpoint homolog b (S. pombe)*	*HUS1B*	6.79	6.77	7.22	0.005547498	0.018824104
8059642	151473	*solute carrier family 16, member 14 (monocarboxylic acid transporter 14)*	*SLC16A14*	7.05	6.72	7.44	0.005548357	0.018824104
8081128	63899, 200895	*NOP2/Sun domain family, member 3 | dihydrofolate reductase-like 1*	*NSUN3|DHFRL1*	7.49	7.34	8.75	0.005556337	0.018842462
7957373	4618	*myogenic factor 6 (herculin)*	*MYF6*	5.81	5.97	5.61	0.005557669	0.018844768
7932792				6.13	6.40	5.46	0.005561221	0.018854598
8121249				5.11	5.02	4.80	0.005565651	0.018867401
8155946	23230	*vacuolar protein sorting 13 homolog A (S. cerevisiae)*	*VPS13A*	6.58	6.51	8.56	0.005567347	0.01887052
8159554	91373	*UDP-N-acteylglucosamine pyrophosphorylase 1-like 1*	*UAP1L1*	8.56	8.69	7.71	0.005568123	0.01887052
7929744	159296	*NK2 transcription factor related, locus 3 (Drosophila)*	*NKX2-3*	7.27	7.26	6.82	0.005568532	0.01887052
8170322				6.55	6.74	6.24	0.005569287	0.01887086
8026122	5886	*RAD23 homolog A (S. cerevisiae)*	*RAD23A*	10.76	10.86	9.43	0.005573929	0.018878404
8067903	29761	*ubiquitin specific peptidase 25*	*USP25*	9.78	9.82	10.65	0.005575437	0.018878404
7993973	55718	*polymerase (RNA) III (DNA directed) polypeptide E (80kD)*	*POLR3E*	8.74	8.79	9.66	0.005572802	0.018878404
7900352	64064	*3-oxoacid CoA transferase 2*	*OXCT2*	7.63	7.58	7.24	0.005574868	0.018878404
8070905	378832	*non-protein coding RNA 175*	*NCRNA00175*	6.92	6.95	6.35	0.005573012	0.018878404
8155397	441425, 441430, 84210, 728747, 729171	*ankyrin repeat domain 20 family, member A3 | ankyrin repeat domain 20 family, member A2 | ankyrin repeat domain 20 family, member A1 | ankyrin repeat domain 20 family, member A4 | ankyrin repeat domain 20B*	*ANKRD20A3|ANKRD20A2|ANKRD20A1|ANKRD20A4|ANKRD20B*	5.51	5.47	5.37	0.005574252	0.018878404
8173955	94121	*synaptotagmin-like 4*	*SYTL4*	6.37	6.37	6.00	0.005584044	0.01890533
8035007	10270	*A kinase (PRKA) anchor protein 8*	*AKAP8*	8.90	8.94	9.45	0.005588063	0.01891672

8023061				6.98	6.84	6.17	0.005590808	0.018923795
8164630	347148	*pyroglutamylated RFamide peptide*	*QRFP*	7.75	7.86	7.55	0.005593427	0.018930437
8089394	84666	*resistin like beta*	*RETNLB*	6.19	6.25	5.87	0.005599256	0.018947946
7910385				11.72	11.80	10.90	0.005600748	0.018950772
7957673	144535	*chromosome 12 open reading frame 55*	*C12orf55*	5.89	5.93	5.60	0.005601483	0.01895104
7995574	3178, 664709, 144983	*heterogeneous nuclear ribonucleoprotein A1 | heterogeneous nuclear ribonucleoprotein A1 pseudogene 10 | heterogeneous nuclear ribonucleoprotein A1-like 2*	*HNRNPA1|HNRNPA1P10|HNRNPA1L2*	10.61	10.42	12.86	0.005603936	0.018957114
7952200	283152	*coiled-coil domain containing 153*	*CCDC153*	7.30	7.10	6.99	0.005607412	0.01896665
8010379	79092	*caspase recruitment domain family, member 14*	*CARD14*	6.84	6.96	6.33	0.00560895	0.018968178
8002301				5.91	5.68	5.23	0.005609177	0.018968178
7933008				6.67	6.57	5.92	0.005609996	0.018968724
8114396	8697	*cell division cycle 23 homolog (S. cerevisiae)*	*CDC23*	7.82	8.03	9.72	0.005614337	0.018977279
7954940	51535	*periphilin 1*	*PPHLN1*	8.35	8.33	9.90	0.005615813	0.018977279
8002249	55512	*sphingomyelin phosphodiesterase 3, neutral membrane (neutral sphingomyelinase II)*	*SMPD3*	7.04	6.83	6.33	0.005615705	0.018977279
7918300	84722	*proline/serine-rich coiled-coil 1*	*PSRC1*	8.01	8.07	9.07	0.005614502	0.018977279
8027480	388531	*regulator of G protein signaling 9 binding protein*	*RGS9BP*	6.11	6.19	5.63	0.005613597	0.018977279
8120382	26036	*zinc finger protein 451*	*ZNF451*	7.90	7.64	9.29	0.005618025	0.018980311
8160317				6.70	6.88	6.21	0.005617615	0.018980311
8034199	26526	*tetraspanin 16*	*TSPAN16*	6.87	6.79	6.55	0.005622236	0.018983074
8023549	30062	*retina and anterior neural fold homeobox*	*RAX*	7.46	7.61	6.82	0.005621762	0.018983074
7963020	50846	*desert hedgehog*	*DHH*	7.04	7.02	6.56	0.00562312	0.018983074
8038427	60385	*testis-specific serine kinase substrate*	*TSKS*	7.39	7.34	6.73	0.005621831	0.018983074
7954965				4.81	4.71	5.38	0.00562251	0.018983074
8053664				5.93	5.71	5.46	0.005623445	0.018983074
8166657				5.90	5.98	5.46	0.005619723	0.018983074
7940287	931	*membrane-spanning 4-domains, subfamily A, member 1*	*MS4A1*	5.43	5.45	5.08	0.0056251	0.018984219

7908161	81563	*chromosome 1 open reading frame 21*	*C1orf21*	10.81	9.49	10.54	0.005624482	0.018984219
8129637	8875	*vanin 2*	*VNN2*	5.15	5.06	5.02	0.005625913	0.018984744
8077353	51095	*tRNA nucleotidyl transferase, CCA-adding, 1*	*TRNT1*	6.48	6.53	7.99	0.005627193	0.018986842
7951034	654320, 79101	*small nucleolar RNA, H/ACA box 8 | TATA box binding protein (TBP)-associated factor, RNA polymerase I, D, 41kDa*	*SNORA8|TAF1D*	9.61	9.71	10.31	0.005631776	0.018997867
7920337				6.73	6.67	6.20	0.005631237	0.018997867
7914003	126695	*chromosome 1 open reading frame 172*	*C1orf172*	7.15	7.13	7.82	0.005634185	0.019001553
7929589				5.61	5.69	5.44	0.00563412	0.019001553
7906146	84283	*transmembrane protein 79*	*TMEM79*	7.36	7.63	6.70	0.00564358	0.019031014
8048725	116255	*monoacylglycerol O-acyltransferase 1*	*MOGAT1*	5.51	5.52	5.19	0.005648914	0.019046778
8064464	128646	*signal-regulatory protein delta*	*SIRPD*	5.88	5.90	5.66	0.005652978	0.019058252
8034304	54	*acid phosphatase 5, tartrate resistant*	*ACP5*	7.12	6.96	7.93	0.005654941	0.01906194
8152244				4.75	4.71	4.53	0.005655392	0.01906194
8141728	64792	*RAB, member RAS oncogene family-like 5*	*RABL5*	9.86	9.77	9.20	0.005656176	0.019062355
7897172	127262	*tumor protein p63 regulated 1-like*	*TPRG1L*	10.38	10.20	9.44	0.005659021	0.01906972
7977786	9056	*solute carrier family 7 (cationic amino acid transporter, y+ system), member 7*	*SLC7A7*	8.01	7.12	6.99	0.00566059	0.019070918
7987426	161835	*fibrous sheath interacting protein 1*	*FSIP1*	5.91	5.94	5.48	0.005660698	0.019070918
7958577	84915	*chromosome 12 open reading frame 34*	*C12orf34*	7.87	7.90	7.29	0.005662415	0.019074477
8154416	203238	*chromosome 9 open reading frame 93*	*C9orf93*	6.22	6.15	7.10	0.005663726	0.019076666
8037018	80776, 641649	*B9 protein domain 2 | transmembrane protein 91*	*B9D2|TMEM91*	7.62	7.67	7.07	0.005666564	0.019084
8170282	441525	*SPANX family, member N4*	*SPANXN4*	5.83	5.81	5.48	0.005668504	0.019088306
7940654	7356	*secretoglobin, family 1A, member 1 (uteroglobin)*	*SCGB1A1*	6.78	6.85	6.36	0.005670758	0.019091444
8129410	387357	*thymocyte selection associated*	*THEMIS*	5.08	5.13	4.90	0.005670224	0.019091444
8095488	26952	*submaxillary gland androgen regulated protein 3A*	*SMR3A*	5.80	5.81	5.40	0.005676124	0.019107278
8092392	89857	*kelch-like 6 (Drosophila)*	*KLHL6*	6.75	6.81	6.34	0.005678235	0.019112157
7916248	6512	*solute carrier family 1 (glutamate transporter), member 7*	*SLC1A7*	6.92	6.75	6.21	0.005681444	0.019116271

8086752	692084, 80185, 6078	*small nucleolar RNA, C/D box 13 | chromosome 8 open reading frame 41 | small nucleolar RNA, C/D box 13 pseudogene 3*	*SNORD13|C8orf41|SNORD13P3*	13.58	13.53	13.16	0.00568029	0.019116271
8017829				6.08	6.04	5.78	0.005681391	0.019116271
7911349				5.42	5.07	6.62	0.005682726	0.019118357
8164343	399665	*family with sequence similarity 102, member A*	*FAM102A*	10.94	10.65	9.59	0.005689021	0.019137304
7957273				4.64	4.60	4.52	0.005690603	0.019140394
7964142	319	*apolipoprotein F*	*APOF*	5.75	5.77	5.51	0.005695678	0.019146744
8131573	10124	*ADP-ribosylation factor-like 4A*	*ARL4A*	7.52	7.16	7.78	0.00569417	0.019146744
7907370	26052	*dynamin 3*	*DNM3*	7.01	6.78	8.29	0.005694945	0.019146744
7983123	55142	*HAUS augmin-like complex, subunit 2*	*HAUS2*	7.37	7.86	9.07	0.005695806	0.019146744
7974647	387990, 26520	*translocase of outer mitochondrial membrane 20 homolog (yeast)-like | translocase of inner mitochondrial membrane 9 homolog (yeast)*	*TOMM20L|TIMM9*	5.89	5.88	5.48	0.005693781	0.019146744
7938225	283298	*olfactomedin-like 1*	*OLFML1*	9.76	8.42	5.06	0.005699011	0.019155284
8131056	54905	*cytochrome P450, family 2, subfamily W, polypeptide 1*	*CYP2W1*	7.70	7.78	7.02	0.005700015	0.019156428
8089652	80218	*N(alpha)-acetyltransferase 50, NatE catalytic subunit*	*NAA50*	10.28	10.17	12.28	0.005701864	0.019160412
8117140	54901	*CDK5 regulatory subunit associated protein 1-like 1*	*CDKAL1*	8.00	7.90	9.90	0.005702952	0.01916184
8042119	64895	*poly(A) polymerase gamma*	*PAPOLG*	9.22	8.78	10.58	0.005705246	0.019167315
8005857	147007	*transmembrane protein 199*	*TMEM199*	9.25	9.34	9.98	0.005710277	0.019181987
8144995	8822	*fibroblast growth factor 17*	*FGF17*	7.24	7.31	6.77	0.00571113	0.019182619
7971905	64881	*protocadherin 20*	*PCDH20*	5.85	5.95	5.57	0.005714901	0.01919082
8016825	1353, 10040	*COX11 cytochrome c oxidase assembly homolog (yeast) | target of myb1 (chicken)-like 1*	*COX11|TOM1L1*	8.15	8.45	8.84	0.005714764	0.01919082
8048611	55515	*amiloride-sensitive cation channel 4, pituitary*	*ACCN4*	5.75	5.79	5.33	0.005716176	0.019192869
8013906	5756	*twinfilin, actin-binding protein, homolog 1 (Drosophila)*	*TWF1*	10.44	10.47	9.65	0.005718087	0.019197052
7994541	27040, 83985	*linker for activation of T cells | spinster homolog 1 (Drosophila)*	*LAT|SPNS1*	7.55	7.52	6.91	0.005723786	0.01921395
8074892	150244	*zinc finger, DHHC-type containing 8 pseudogene 1*	*ZDHHC8P1*	7.81	7.83	7.11	0.005725176	0.019216383

7991772	3049	*hemoglobin, theta 1*	*HBQ1*	8.51	8.62	7.85	0.005730103	0.019230684
8067221	81030	*Z-DNA binding protein 1*	*ZBP1*	6.63	6.65	6.12	0.005743119	0.019272128
8100007	8929	*paired-like homeobox 2b*	*PHOX2B*	5.49	5.60	5.30	0.005745313	0.019276956
8061919	9139	*core-binding factor, runt domain, alpha subunit 2; translocated to, 2*	*CBFA2T2*	7.89	7.85	7.38	0.005745894	0.019276956
7905395	5692	*proteasome (prosome, macropain) subunit, beta type, 4*	*PSMB4*	11.22	10.95	11.77	0.005747754	0.019279245
8155600				5.27	5.27	4.92	0.005747912	0.019279245
8070771	386677	*keratin associated protein 10-1*	*KRTAP10-1*	8.29	8.35	7.70	0.005748902	0.019280326
7935780	25956	*SEC31 homolog B (S. cerevisiae)*	*SEC31B*	7.18	6.95	6.44	0.005756484	0.0192997
7953032	654429	*leucine-rich repeats and transmembrane domains 2*	*LRTM2*	7.40	7.32	6.68	0.005756867	0.0192997
8139456	677798, 285958	*small nucleolar RNA, H/ACA box 9 | chromosome 7 open reading frame 40*	*SNORA9|C7orf40*	7.39	7.72	8.61	0.005757353	0.0192997
7939938				5.56	5.56	5.44	0.005756905	0.0192997
8083656	64747	*major facilitator superfamily domain containing 1*	*MFSD1*	9.83	10.06	8.27	0.005759751	0.019305497
7927389	5599	*mitogen-activated protein kinase 8*	*MAPK8*	8.87	8.81	10.00	0.00576346	0.0193112
8146024	203102	*ADAM metallopeptidase domain 32*	*ADAM32*	6.73	6.92	5.84	0.005762241	0.0193112
8168657	27328, 83259	*protocadherin 11 X-linked | protocadherin 11 Y-linked*	*PCDH11X|PCDH11Y*	5.44	5.47	6.77	0.005763245	0.0193112
8010624	83882	*tetraspanin 10*	*TSPAN10*	7.29	7.47	6.52	0.005764416	0.019312166
8091696				6.45	6.37	6.16	0.005765264	0.019312765
7980044	9240	*paraneoplastic antigen MA1*	*PNMA1*	8.59	9.08	8.56	0.005770314	0.019327438
8092000	7012	*telomerase RNA component*	*TERC*	8.70	8.82	9.92	0.005771815	0.019328382
8143397	27147	*DENN/MADD domain containing 2A*	*DENND2A*	8.04	7.33	7.12	0.005772894	0.019328382
8160722	55833	*ubiquitin associated protein 2*	*UBAP2*	9.95	9.85	10.97	0.005773274	0.019328382
7928766	387695	*chromosome 10 open reading frame 99*	*C10orf99*	6.66	6.70	6.23	0.005772534	0.019328382
8082289	166012	*carbohydrate (chondroitin 4) sulfotransferase 13*	*CHST13*	7.77	7.92	7.07	0.005778895	0.01934496
7948794	221091	*LRRN4 C-terminal like*	*LRRN4CL*	8.86	8.86	6.65	0.005779671	0.019345313
7972190	64062	*RNA binding motif protein 26*	*RBM26*	8.63	8.41	10.12	0.005784234	0.01935834

7955317	41	*amiloride-sensitive cation channel 2, neuronal*	*ACCN2*	8.56	8.52	6.27	0.005788245	0.019364735
7987650	4058	*leukocyte receptor tyrosine kinase*	*LTK*	6.66	6.76	6.26	0.005788096	0.019364735
8164002	57684	*zinc finger and BTB domain containing 26*	*ZBTB26*	7.49	7.18	8.39	0.005787902	0.019364735
7899703	200081	*taxilin alpha*	*TXLNA*	10.48	9.98	9.27	0.005789974	0.019364735
8161632	375743	*protein prenyltransferase alpha subunit repeat containing 1*	*PTAR1*	10.02	9.52	11.79	0.005790169	0.019364735
7933261	100133130	*PRO1102*	*LOC100133130*	5.15	5.09	6.27	0.005789604	0.019364735
7964021	10193	*ring finger protein 41*	*RNF41*	9.86	9.98	9.17	0.005791339	0.019366404
7919055	3158	*3-hydroxy-3-methylglutaryl-CoA synthase 2 (mitochondrial)*	*HMGCS2*	6.21	6.31	5.89	0.005794922	0.019376142
7909175	23380	*SLIT-ROBO Rho GTPase activating protein 2*	*SRGAP2*	9.50	9.36	8.31	0.005800255	0.019390488
8175531	1038, 64848	*cerebellar degeneration-related protein 1, 34kDa | YTH domain containing 2*	*CDR1|YTHDC2*	9.09	8.75	7.97	0.005800556	0.019390488
8150862				5.43	5.57	6.02	0.005802851	0.019395914
8157151				6.15	6.14	5.54	0.005803979	0.019397436
7955624	3892, 3887, 3889	*keratin 86 | keratin 81 | keratin 83*	*KRT86|KRT81|KRT83*	7.50	7.82	6.93	0.005809806	0.019414665
8171725	256714	*MAP7 domain containing 2*	*MAP7D2*	5.98	5.92	6.23	0.005811579	0.01941616
8154848	5646, 5645	*protease, serine, 3 | protease, serine, 2 (trypsin 2)*	*PRSS3|PRSS2*	7.78	7.64	7.04	0.005811599	0.01941616
7897293				8.08	8.14	7.46	0.005812893	0.019418236
8153043	57623	*zinc finger and AT hook domain containing*	*ZFAT*	8.09	8.07	7.55	0.005813939	0.019419484
8177462	1022	*cyclin-dependent kinase 7*	*CDK7*	8.81	8.06	9.05	0.005815305	0.019419555
7950544	5612	*protein-kinase, interferon-inducible double stranded RNA dependent inhibitor, repressor of (P58 repressor)*	*PRKRIR*	8.00	8.09	9.06	0.005815029	0.019419555
8117334	8359	*histone cluster 1, H4a*	*HIST1H4A*	6.63	7.05	8.52	0.00581992	0.019432716
8149847				7.55	7.54	7.27	0.005821992	0.019437388
8103389	1519	*cathepsin O*	*CTSO*	7.86	7.55	5.72	0.005830242	0.01946043
8170994	474382, 83740, 474381	*H2A histone family, member B1 | H2A histone family, member B3 | H2A histone family, member B2*	*H2AFB1|H2AFB3|H2AFB2*	6.44	6.45	6.12	0.005829576	0.01946043

7924119	25896	*integrator complex subunit 7*	*INTS7*	7.61	7.53	9.50	0.00583264	0.019466182
8117429	8346	*histone cluster 1, H2bi*	*HIST1H2B I*	5.69	6.30	8.11	0.005836867	0.019466545
8048898	11262		*SP140*	6.19	6.14	5.93	0.005834899	0.019466545
8060447	343641	*transglutaminase 6*	*TGM6*	6.64	6.71	6.28	0.005837468	0.019466545
8105132	389289, 153684	*chromosome 5 open reading frame 39 | hypothetical LOC153684*	*C5orf39|L OC153684*	7.21	7.00	6.28	0.005835208	0.019466545
7910539	83932, 149371	*chromosome 1 open reading frame 124 | exocyst complex component 8*	*C1orf124| EXOC8*	7.83	7.92	8.84	0.005836663	0.019466545
8007499				4.76	4.69	4.58	0.005834243	0.019466545
8105739				7.19	7.35	6.65	0.005835773	0.019466545
7916493	8613	*phosphatidic acid phosphatase type 2B*	*PPAP2B*	13.07	12.75	11.73	0.005840546	0.019472308
8037170	5050, 284338	*platelet-activating factor acetylhydrolase 1b, catalytic subunit 3 (29kDa) | proline rich 19*	*PAFAH1B3 |PRR19*	9.87	10.07	11.55	0.005840288	0.019472308
8075363	85371		*KIAA1656*	5.94	6.12	5.51	0.005841657	0.019473763
8097813	27236	*ADP-ribosylation factor interacting protein 1*	*ARFIP1*	8.51	8.30	9.33	0.005843063	0.019476203
7929032	355	*Fas (TNF receptor superfamily, member 6)*	*FAS*	9.77	9.72	8.22	0.005846901	0.019484496
7917599	343472	*BarH-like homeobox 2*	*BARHL2*	7.91	7.98	7.36	0.005846473	0.019484496
8137330	11194	*ATP-binding cassette, sub-family B (MDR/TAP), member 8*	*ABCB8*	8.92	9.04	8.23	0.005849059	0.019489437
8128549				6.60	6.31	6.93	0.005851701	0.01949599
8033420	2208	*Fc fragment of IgE, low affinity II, receptor for (CD23)*	*FCER2*	6.43	6.45	6.13	0.005853097	0.019496143
7938313	10527	*importin 7*	*IPO7*	10.01	10.08	11.69	0.005852812	0.019496143

8049349	54658, 54578, 54575, 54600, 54657, 54659, 54579, 54577, 54576	*UDP glucuronosyltransferase 1 family, polypeptide A1 | UDP glucuronosyltransferase 1 family, polypeptide A6 | UDP glucuronosyltransferase 1 family, polypeptide A10 | UDP glucuronosyltransferase 1 family, polypeptide A9 | UDP glucuronosyltransferase 1 family, polypeptide A4 | UDP glucuronosyltransferase 1 family, polypeptide A3 | UDP glucuronosyltransferase 1 family, polypeptide A5 | UDP glucuronosyltransferase 1 family, polypeptide A7 | UDP glucuronosyltransferase 1 family, polypeptide A8*	*UGT1A1|UGT1A6|UGT1A10|UGT1A9|UGT1A4|UGT1A3|UGT1A5|UGT1A7|UGT1A8*	5.77	5.81	5.56	0.005855903	0.019503236
8055952	4929	*nuclear receptor subfamily 4, group A, member 2*	*NR4A2*	6.91	6.83	6.16	0.005858586	0.019506244
8068740	89766	*uromodulin-like 1*	*UMODL1*	6.65	6.77	6.23	0.005859508	0.019506244
7981161	161176	*chromosome 14 open reading frame 49*	*C14orf49*	8.06	7.68	6.59	0.005858806	0.019506244
7977452	642477	*hypothetical LOC642477*	*FLJ39632*	7.39	7.28	6.77	0.00585926	0.019506244
8029318	56269	*immunity-related GTPase family, cinema*	*IRGC*	6.15	6.23	5.60	0.00586077	0.019508192
7919168	9659	*phosphodiesterase 4D interacting protein*	*PDE4DIP*	9.91	9.51	7.89	0.005861931	0.019509597
8067802	10287	*regulator of G-protein signaling 19*	*RGS19*	9.32	9.41	8.36	0.005863219	0.019509597
8129573	26002	*monooxygenase, DBH-like 1*	*MOXD1*	8.51	10.46	6.64	0.005862829	0.019509597
8002051	181	*agouti related protein homolog (mouse)*	*AGRP*	7.53	7.60	7.12	0.005869929	0.019529674
7911796	440556	*hypothetical LOC440556*	*FLJ42875*	8.21	8.21	7.46	0.005873481	0.019534737
8172757	653219, 653220, 653048, 9503, 653067	*X antigen family, member 1A | X antigen family, member 1B | X antigen family, member 1C | X antigen family, member 1D | X antigen family, member 1E*	*XAGE1A|XAGE1B|XAGE1C|XAGE1D|XAGE1E*	6.07	6.18	5.81	0.005872665	0.019534737
8040477				7.36	7.43	6.63	0.005873344	0.019534737
7951859	337	*apolipoprotein A-IV*	*APOA4*	6.95	7.04	6.57	0.005874327	0.0195353
7968331	7332	*ubiquitin-conjugating enzyme E2L 3*	*UBE2L3*	12.35	12.36	12.67	0.005878529	0.019547023
7933105	219749	*zinc finger protein 25*	*ZNF25*	7.43	7.49	6.14	0.005886814	0.019572316
7915498	100131756		*LOC100131756*	5.53	5.52	5.35	0.00588756	0.019572545
8003087				7.09	6.87	7.60	0.005894151	0.019592198

7963698	11016	*activating transcription factor 7*	*ATF7*	9.66	9.63	8.35	0.005897169	0.019595584
8051238	388939	*chromosome 2 open reading frame 71*	*C2orf71*	6.76	6.89	6.36	0.005897385	0.019595584
7913290	400745	*SH2 domain containing 5*	*SH2D5*	7.24	7.36	7.07	0.005897884	0.019595584
8076813	642757	*hypothetical LOC642757*	*FLJ32756*	5.85	5.77	5.50	0.005895906	0.019595584
7978866	9147	*serologically defined colon cancer antigen 1*	*SDCCAG1*	7.98	7.71	8.84	0.005899312	0.019595815
8029301	284346	*zinc finger protein 575*	*ZNF575*	8.42	8.16	7.21	0.005898998	0.019595815
8128698	27244	*sestrin 1*	*SESN1*	7.83	7.48	9.57	0.005901505	0.019600846
8163599	25861	*deafness, autosomal recessive 31*	*DFNB31*	7.64	7.75	7.12	0.005903688	0.019605841
8070389	3150	*high-mobility group nucleosome binding domain 1*	*HMGN1*	8.09	8.15	8.49	0.005905181	0.019608542
7897132	63976	*PR domain containing 16*	*PRDM16*	7.23	7.34	6.64	0.005908625	0.019617723
8023382	8932	*methyl-CpG binding domain protein 2*	*MBD2*	9.51	9.88	8.82	0.005909819	0.01961943
8066117	25939, 140711	*SAM domain and HD domain 1 | chromosome 20 open reading frame 118*	*SAMHD1|C20orf118*	7.83	7.31	9.92	0.005911697	0.01962115
7963410	286887, 3854, 3853	*keratin 6C | keratin 6B | keratin 6A*	*KRT6C|KRT6B|KRT6A*	7.95	7.91	7.50	0.005911408	0.01962115
8156718	4686	*nuclear cap binding protein subunit 1, 80kDa*	*NCBP1*	9.12	8.93	10.88	0.005918364	0.019641021
7905598	23557	*SNAP-associated protein*	*SNAPIN*	9.99	10.12	9.25	0.005920625	0.01964401
7950420	387787	*lipoyl(octanoyl) transferase 2 (putative)*	*LIPT2*	7.88	8.12	9.00	0.005920013	0.01964401
8010785				5.19	5.15	4.81	0.005929295	0.019670516
8023195	4087	*SMAD family member 2*	*SMAD2*	8.87	8.59	10.02	0.005933029	0.01967838
7904244	126868	*chromosome 1 open reading frame 161*	*C1orf161*	5.79	5.78	5.28	0.005932625	0.01967838
8070896	246704	*chromosome 21 open reading frame 93*	*C21orf93*	6.82	6.92	6.37	0.005934844	0.01968214
7944006	10179	*RNA binding motif protein 7*	*RBM7*	7.21	7.39	8.11	0.005935872	0.019683285
8155501				6.35	6.38	5.81	0.005938305	0.01968909
7965154				5.70	5.79	5.32	0.005948217	0.019719692

8161571	100036519, 349334, 653427, 653404, 286380, 200350, 2298	*forkhead box D4-like 2 | forkhead box D4-like 4 | forkhead box D4-like 5 | forkhead box D4-like 6 | forkhead box D4-like 3 | forkhead box D4-like 1 | forkhead box D4*	*FOXD4L2| FOXD4L4| FOXD4L5| FOXD4L6| FOXD4L3| FOXD4L1*	7.86	7.85	7.54	0.005949043	0.019720167
8174317	92129, 9075	*ripply1 homolog (zebrafish) | claudin 2*	*RIPPLY1| CLDN2*	6.60	6.59	6.31	0.005951385	0.019725662
8157092	55151	*transmembrane protein 38B*	*TMEM38B*	6.46	6.84	7.69	0.005953239	0.019729545
8078312	152100, 64343	*COX assembly mitochondrial protein homolog (S. cerevisiae) | 5-azacytidine induced 2*	*CMC1|AZI 2*	5.96	6.01	6.40	0.005954141	0.019730268
8137006				4.66	4.76	4.55	0.005957695	0.01973978
8111253				4.71	4.84	4.51	0.005959434	0.019743273
7974418	10668	*cell growth regulator with ring finger domain 1*	*CGRRF1*	6.64	6.84	7.11	0.005963268	0.01975371
8097282	10252	*sprouty homolog 1, antagonist of FGF signaling (Drosophila)*	*SPRY1*	8.37	8.39	9.42	0.005965012	0.019754952
8088979	389136	*vestigial like 3 (Drosophila)*	*VGLL3*	11.00	10.41	7.73	0.005964547	0.019754952
7975713	51077		*FCF1*	7.74	7.77	9.09	0.005966516	0.019757668
8168646	90316, 90655	*TGFB-induced factor homeobox 2-like, X-linked | TGFB-induced factor homeobox 2-like, Y-linked*	*TGIF2LX| TGIF2LY*	5.40	5.56	5.20	0.005967354	0.019758176
8144078	6469	*sonic hedgehog*	*SHH*	7.57	7.82	7.07	0.005969231	0.019762125
7928752	27069	*growth hormone inducible transmembrane protein*	*GHITM*	10.30	10.72	10.77	0.005972209	0.019768678
7991296	374654	*kinesin family member 7*	*KIF7*	7.86	7.94	7.27	0.00597258	0.019768678
7954124	55766	*H2A histone family, member J*	*H2AFJ*	7.49	7.57	7.19	0.00597392	0.019770848
8008493	51747	*LUC7-like 3 (S. cerevisiae)*	*LUC7L3*	7.03	6.85	8.76	0.005979854	0.019788217
8117408	3012	*histone cluster 1, H2ae*	*HIST1H2A E*	6.14	6.83	8.11	0.005981486	0.01979103
8077351				7.81	7.66	7.09	0.005982075	0.01979103
8151927				6.80	7.40	6.09	0.005982853	0.019791333
8006562	91608	*RAS-like, family 10, member B*	*RASL10B*	8.32	8.22	7.88	0.005989146	0.019809883
8120675	154288	*chromosome 6 open reading frame 221*	*C6orf221*	6.74	7.14	6.27	0.005997884	0.019836511

7963750	23468	*chromobox homolog 5*	*CBX5*	10.29	10.22	12.46	0.006011936	0.019878432
8078896				5.03	5.01	4.71	0.006011261	0.019878432
8158542	100128501		*LOC100128501*	6.35	6.28	6.01	0.006017606	0.019894902
8047765				6.01	6.07	5.62	0.006019285	0.019898172
8125341	177	*advanced glycosylation end product-specific receptor*	*AGER*	7.24	7.28	6.88	0.006021303	0.019902565
8073623	758	*metallophosphoesterase domain containing 1*	*MPPED1*	7.87	7.95	7.53	0.006023076	0.019905396
8042324				5.88	6.06	5.42	0.006023539	0.019905396
8034694	407013	*microRNA 24-2*	*MIR24-2*	6.09	6.70	5.26	0.006024949	0.019907778
8148783	727957	*HEAT repeat containing 7A*	*HEATR7A*	9.26	9.31	8.10	0.00602633	0.019910064
7965812	79158	*N-acetylglucosamine-1-phosphate transferase, alpha and beta subunits*	*GNPTAB*	7.86	7.96	9.87	0.00602923	0.019917361
7996761	23659	*phospholipase A2, group XV*	*PLA2G15*	9.66	9.42	8.09	0.006032353	0.0199254
7989277	4643	*myosin IE*	*MYO1E*	7.90	8.57	9.82	0.00603382	0.019926632
7996022	6367	*chemokine (C-C motif) ligand 22*	*CCL22*	6.92	6.89	6.49	0.006035487	0.019926632
8095230	6731	*signal recognition particle 72kDa*	*SRP72*	10.97	10.97	11.68	0.006035394	0.019926632
8035581	10147	*SURP and G patch domain containing 2*	*SUGP2*	8.07	8.37	9.04	0.006035173	0.019926632
8151149	10565	*ADP-ribosylation factor guanine nucleotide-exchange factor 1(brefeldin A-inhibited)*	*ARFGEF1*	8.27	7.77	10.31	0.006039072	0.019936189
7959131	92558	*coiled-coil domain containing 64*	*CCDC64*	6.66	6.93	7.04	0.006040282	0.019936662
8025850	57572, 55908	*dedicator of cytokinesis 6 | hepatocellular carcinoma-associated gene TD26*	*DOCK6|LOC55908*	7.48	7.42	6.97	0.006040596	0.019936662
7971692	26512	*integrator complex subunit 6*	*INTS6*	7.41	7.35	8.31	0.006043735	0.019944739
8022170	6128	*ribosomal protein L6*	*RPL6*	12.18	12.30	12.67	0.006044573	0.019945225
8133856				7.60	5.60	5.63	0.006046326	0.01994873
8135594	857	*caveolin 1, caveolae protein, 22kDa*	*CAV1*	12.78	12.74	11.08	0.006047139	0.019949133
7909862	79762	*chromosome 1 open reading frame 115*	*C1orf115*	6.78	7.32	7.05	0.006048607	0.019949414
8094516				6.05	6.12	5.54	0.006048236	0.019949414
7999431	58531	*protamine 3*	*PRM3*	7.70	7.72	7.07	0.006053957	0.019964779
7973867				9.72	9.30	8.78	0.006054719	0.019965012

8122805				4.70	4.60	4.53	0.006056225	0.019967696
7907353	51603	*methyltransferase like 13*	*METTL13*	7.92	7.96	8.97	0.006060076	0.019978113
8067671	6725	*src-related kinase lacking C-terminal regulatory tyrosine and N-terminal myristylation sites*	*SRMS*	7.52	7.65	6.96	0.006061578	0.019980783
7987225	55505		*NOP10*	11.30	11.61	11.90	0.006064785	0.019989071
7928514				5.98	5.97	5.52	0.006065538	0.01998927
8061706	23509	*protein O-fucosyltransferase 1*	*POFUT1*	9.35	9.33	8.24	0.006066855	0.019991329
7989657	53944	*casein kinase 1, gamma 1*	*CSNK1G1*	6.24	6.05	7.67	0.00606826	0.019992054
8031815	284309, 84914	*zinc finger protein 776 | zinc finger protein 587*	*ZNF776|ZNF587*	7.31	7.15	8.85	0.00606846	0.019992054
7919751	4170	*myeloid cell leukemia sequence 1 (BCL2-related)*	*MCL1*	10.16	10.28	11.55	0.006070405	0.019996181
8005029	6416	*mitogen-activated protein kinase kinase 4*	*MAP2K4*	8.55	8.50	9.39	0.006083748	0.020026844
8130674	10846	*phosphodiesterase 10A*	*PDE10A*	5.78	5.82	6.64	0.006082351	0.020026844
8000941	23361	*zinc finger protein 629*	*ZNF629*	7.74	7.78	7.10	0.006082156	0.020026844
7937112	54777	*chromosome 10 open reading frame 92*	*C10orf92*	6.73	6.80	6.28	0.006083876	0.020026844
7987526	90416, 359821	*chromosome 15 open reading frame 57 | mitochondrial ribosomal protein L42 pseudogene 5*	*C15orf57|MRPL42P5*	8.70	8.34	7.64	0.006082503	0.020026844
7981215				9.21	8.40	7.49	0.006082518	0.020026844
7987012	89832, 1139	*CHRNA7 (cholinergic receptor, nicotinic, alpha 7, exons 5-10) and FAM7A (family with sequence similarity 7A, exons A-E) fusion | cholinergic receptor, nicotinic, alpha 7*	*CHRFAM7A|CHRNA7*	7.30	7.05	8.21	0.006085375	0.020029495
8107194	90355	*chromosome 5 open reading frame 30*	*C5orf30*	7.86	7.85	8.72	0.006089548	0.020040944
7938047				5.93	5.77	5.74	0.006092668	0.020048926
8076734	7477	*wingless-type MMTV integration site family, member 7B*	*WNT7B*	6.96	7.08	6.32	0.006099689	0.020069744
8022882	7572	*zinc finger protein 24*	*ZNF24*	8.27	8.02	9.89	0.006101288	0.020071313
7944799	219870, 390265, 390264	*olfactory receptor, family 10, subfamily G, member 9 | olfactory receptor, family 10, subfamily G, member 7 | olfactory receptor, family 10, subfamily G, member 4*	*OR10G9|OR10G7|OR10G4*	6.30	6.47	5.96	0.006101557	0.020071313
8115871	441116		*FLJ16171*	7.58	7.40	6.76	0.006110812	0.020099467

7944876	4900	*neurogranin (protein kinase C substrate, RC3)*	*NRGN*	7.41	7.38	6.62	0.006114521	0.020106625
8090133	64770	*coiled-coil domain containing 14*	*CCDC14*	7.55	7.48	9.70	0.00611647	0.020106625
8035110	117286	*calcium and integrin binding family member 3*	*CIB3*	6.01	6.11	5.63	0.006115839	0.020106625
8123695	10455, 404220	*peroxisomal D3,D2-enoyl-CoA isomerase | chromosome 6 open reading frame 201*	*PECI|C6orf201*	8.75	8.58	9.40	0.006114077	0.020106625
8043682				7.94	8.00	7.20	0.006115485	0.020106625
8172766	653219, 653220, 653048, 9503, 653067	*X antigen family, member 1A | X antigen family, member 1B | X antigen family, member 1C | X antigen family, member 1D | X antigen family, member 1E*	*XAGE1A|XAGE1B|XAGE1C|XAGE1D|XAGE1E*	6.07	6.19	5.81	0.006117738	0.020108504
8107321	114915	*non-protein coding RNA 219*	*NCRNA00219*	8.83	9.20	9.50	0.006119075	0.020110609
7945767	1028	*cyclin-dependent kinase inhibitor 1C (p57, Kip2)*	*CDKN1C*	9.32	9.20	8.67	0.006122186	0.02011701
8050993	130106	*calcium and integrin binding family member 4*	*CIB4*	5.55	5.55	5.24	0.006122417	0.02011701
8063650	79716	*aminopeptidase-like 1*	*NPEPL1*	9.39	9.10	8.38	0.006123991	0.020119892
7992678	652276		*LOC652276*	7.49	7.77	7.39	0.006128723	0.020133149
8167710	653219, 653220, 653048, 9503, 653067	*X antigen family, member 1A | X antigen family, member 1B | X antigen family, member 1C | X antigen family, member 1D | X antigen family, member 1E*	*XAGE1A|XAGE1B|XAGE1C|XAGE1D|XAGE1E*	6.08	6.19	5.82	0.006129893	0.020134699
7936918	387718	*chromosome 10 open reading frame 122*	*C10orf122*	5.37	5.26	4.94	0.006131293	0.020134717
7993259	8303, 51061	*stannin | thioredoxin domain containing 11*	*SNN|TXNDC11*	8.54	8.45	9.06	0.006131183	0.020134717
7954481	8082	*sarcospan (Kras oncogene-associated gene)*	*SSPN*	8.57	8.24	6.90	0.006133596	0.020136973
7945162	8538	*BARX homeobox 2*	*BARX2*	7.36	7.48	6.85	0.006134073	0.020136973
8141946				4.51	4.51	4.42	0.00613278	0.020136973
8000856	26000	*TBC1 domain family, member 10B*	*TBC1D10B*	8.44	8.72	7.28	0.006135644	0.020139841
7926898				4.52	4.50	4.42	0.00614096	0.020155
8115907	51491		*NOP16*	7.64	7.99	9.15	0.006144373	0.020163909

7898713	23436	*chymotrypsin-like elastase family, member 3B*	*CELA3B*	7.67	7.71	7.13	0.006145418	0.020165045
8053533	10989	*inner membrane protein, mitochondrial*	*IMMT*	9.10	9.46	11.00	0.006146243	0.02016546
7945058	79607	*family with sequence similarity 118, member B*	*FAM118B*	9.99	9.88	11.50	0.006147552	0.020167464
8031949	388569	*zinc finger protein 324B*	*ZNF324B*	8.28	8.39	7.59	0.006149065	0.020170134
8020149	8774	*N-ethylmaleimide-sensitive factor attachment protein, gamma*	*NAPG*	7.92	7.95	9.28	0.006150398	0.020172216
8086400	131375	*lysozyme-like 4*	*LYZL4*	6.54	6.64	6.19	0.006164391	0.020215811
8063315	55661	*DEAD (Asp-Glu-Ala-Asp) box polypeptide 27*	*DDX27*	7.91	7.96	8.95	0.006167492	0.020221388
8126556	65989	*delta-like 2 homolog (Drosophila)*	*DLK2*	7.90	7.74	7.19	0.006167468	0.020221388
8069666				8.32	8.30	7.72	0.006168244	0.020221556
8122724	80329	*UL16 binding protein 1*	*ULBP1*	7.82	9.71	7.34	0.006170103	0.020225355
7938076	120787	*olfactory receptor, family 52, subfamily W, member 1*	*OR52W1*	6.36	6.37	5.96	0.006172033	0.020229384
8161745				6.02	6.03	5.46	0.006174224	0.020234266
8179926	1797	*dom-3 homolog Z (C. elegans)*	*DOM3Z*	9.91	9.92	8.69	0.00617689	0.020240707
8168026				6.51	6.53	5.75	0.006180757	0.02025108
8099037	10227	*major facilitator superfamily domain containing 10*	*MFSD10*	10.87	11.07	9.68	0.006187526	0.020270957
8001131				5.25	4.82	4.79	0.006188755	0.020272683
8035896	57616	*teashirt zinc finger homeobox 3*	*TSHZ3*	8.89	9.22	7.61	0.006193643	0.020284092
8023871	284273	*zinc binding alcohol dehydrogenase domain containing 2*	*ZADH2*	8.96	8.85	7.71	0.006193302	0.020284092
8123800				6.59	6.42	7.45	0.006198105	0.020296404
8044415				7.86	7.84	7.30	0.006199447	0.020298494
8061364	6171, 6172	*ribosomal protein L41 | ribosomal protein L41 pseudogene 5*	*RPL41|RPL41P5*	13.13	13.23	13.05	0.006203102	0.020305859
8005200	692087, 125144	*small nucleolar RNA, C/D box 49B | non-protein coding RNA 188*	*SNORD49B|NCRNA00188*	11.01	11.33	10.13	0.006202503	0.020305859
7998542	9742	*intraflagellar transport 140 homolog (Chlamydomonas)*	*IFT140*	8.35	8.05	7.47	0.006207438	0.020315442
7985767	64782	*apoptosis enhancing nuclease*	*AEN*	9.23	9.32	10.50	0.006207183	0.020315442
7904751	9939	*RNA binding motif protein 8A*	*RBM8A*	7.19	7.35	8.47	0.006211523	0.020326508

8079677	54681	*prolyl 4-hydroxylase, transmembrane (endoplasmic reticulum)*	*P4HTM*	9.04	8.88	7.94	0.006217194	0.020339228
8174239	84707	*brain expressed X-linked 2*	*BEX2*	6.11	6.14	6.73	0.00621653	0.020339228
8074867	25812, 646074, 29774, 29797, 100294020	*POM121 membrane glycoprotein-like 1, pseudogene | POM121 membrane glycoprotein-like 10, pseudogene | POM121 membrane glycoprotein-like 9, pseudogene | POM121 membrane glycoprotein-like 8 pseudogene | putative POM121-like protein 1-like*	*POM121L1P|POM121L10P|POM121L9P|POM121L8P|LOC100294020*	6.97	6.94	6.39	0.006217524	0.020339228
8119423	221442	*adenylate cyclase 10 pseudogene*	*LOC221442*	5.68	5.33	6.26	0.006221749	0.02034844
7989619	5479, 79856	*peptidylprolyl isomerase B (cyclophilin B) | sorting nexin 22*	*PPIB|SNX22*	13.25	13.29	12.97	0.006221488	0.02034844
7911902	261734	*nephronophthisis 4*	*NPHP4*	7.52	7.65	6.90	0.006223146	0.020350704
8088001	6787	*NIMA (never in mitosis gene a)-related kinase 4*	*NEK4*	7.39	7.38	8.57	0.006225161	0.020354986
8158081	138428, 286207	*peptidyl-tRNA hydrolase 1 homolog (S. cerevisiae) | chromosome 9 open reading frame 117*	*PTRH1|C9orf117*	6.86	7.10	6.39	0.006227861	0.020361507
8131091	7975, 202915	*v-maf musculoaponeurotic fibrosarcoma oncogene homolog K (avian) | transmembrane protein 184A*	*MAFK|TMEM184A*	8.04	8.15	7.40	0.00622946	0.02036443
8042601	27332	*zinc finger protein 638*	*ZNF638*	7.77	7.29	9.15	0.006235393	0.020381518
8002072	84080	*chromosome 16 open reading frame 48*	*C16orf48*	8.11	8.03	7.26	0.006236878	0.020384062
7948344	10249	*glycine-N-acyltransferase*	*GLYAT*	5.16	5.33	4.95	0.006238691	0.020387681
7935572	84795, 100302133	*pyridine nucleotide-disulphide oxidoreductase domain 2 | microRNA 1287*	*PYROXD2|MIR1287*	9.28	8.42	6.78	0.006239737	0.02038879
7911854	57212		*KIAA0495*	7.86	7.73	7.29	0.006242437	0.020395305
7936928	118856	*matrix metallopeptidase 21*	*MMP21*	6.24	6.23	5.80	0.006243981	0.020398041
7954511	23012	*serine/threonine kinase 38 like*	*STK38L*	8.74	8.62	10.97	0.006247541	0.020405052
8006415	26022	*transmembrane protein 98*	*TMEM98*	11.06	10.64	9.62	0.006246881	0.020405052
8023377	51320	*mex-3 homolog C (C. elegans)*	*MEX3C*	9.80	9.92	10.97	0.006250294	0.020411734
8147724	528	*ATPase, H+ transporting, lysosomal 42kDa, V1 subunit C1*	*ATP6V1C1*	8.02	7.90	9.77	0.006251865	0.020412248

7964685				4.54	4.59	4.43	0.006251334	0.020412248
7912743	79363	*chromosome 1 open reading frame 89*	*C1orf89*	8.26	8.12	7.12	0.006257559	0.02042622
8179238	100507436	*MHC class I polypeptide-related sequence A*	*MICA*	9.57	9.30	8.22	0.006257468	0.02042622
7981157	79686	*chromosome 14 open reading frame 139*	*C14orf139*	7.87	7.55	5.98	0.006260533	0.020433616
8048707	151278	*coiled-coil domain containing 140*	*CCDC140*	7.07	6.97	6.30	0.006263594	0.020441294
8160597	138474	*TAF1 RNA polymerase II, TATA box binding protein (TBP)-associated factor, 210kDa-like*	*TAF1L*	6.08	6.17	6.02	0.006264388	0.020441575
8128572	5550	*prolyl endopeptidase*	*PREP*	8.26	8.58	9.14	0.006267726	0.020449413
8044634	200350, 2298, 653404, 100036519, 349334, 653427	*forkhead box D4-like 1 | forkhead box D4 | forkhead box D4-like 6 | forkhead box D4-like 2 | forkhead box D4-like 4 | forkhead box D4-like 5*	*FOXD4L1|FOXD4L6|FOXD4L2|FOXD4L4|FOXD4L5*	7.19	7.25	6.92	0.006268207	0.020449413
8167701	653219, 653220, 653048, 9503, 653067	*X antigen family, member 1A | X antigen family, member 1B | X antigen family, member 1C | X antigen family, member 1D | X antigen family, member 1E*	*XAGE1A|XAGE1B|XAGE1C|XAGE1D|XAGE1E*	6.07	6.19	5.81	0.006269447	0.020451147
8109236	6534	*solute carrier family 6 (neurotransmitter transporter, L-proline), member 7*	*SLC6A7*	6.87	6.90	6.28	0.006272406	0.02045849
7919390				5.63	5.69	7.47	0.006274739	0.020463787
7962489	51054	*pleckstrin homology domain containing, family A (phosphoinositide binding specific) member 9*	*PLEKHA9*	6.70	6.77	7.67	0.006276958	0.0204664
7981181	677768, 283596	*small Cajal body-specific RNA 13 | small nucleolar RNA host gene 10 (non-protein coding)*	*SCARNA13|SNHG10*	9.55	9.60	8.72	0.006276783	0.0204664
7989476	255180, 100289289	*hypothetical FLJ38723 | hypothetical protein LOC100289289*	*FLJ38723|LOC100289289*	6.18	6.27	5.91	0.006280272	0.020474892
8032782	126259	*transmembrane and immunoglobulin domain containing 2*	*TMIGD2*	6.93	6.93	6.54	0.006285179	0.020486947
8001037	260434	*PYD (pyrin domain) containing 1*	*PYDC1*	6.30	6.33	5.87	0.006285389	0.020486947
8124440	8355	*histone cluster 1, H3g*	*HIST1H3G*	6.60	7.55	9.68	0.006294366	0.02051389

7929901	56652	*chromosome 10 open reading frame 2*	*C10orf2*	6.71	7.02	8.16	0.006301092	0.020531176
8075555	150297	*chromosome 22 open reading frame 42*	*C22orf42*	6.07	6.16	5.76	0.006300613	0.020531176
8014700	100170841	*chromosome 17 open reading frame 96*	*C17orf96*	7.40	7.65	6.66	0.006303102	0.020535408
8079796	63891	*ring finger protein 123*	*RNF123*	9.19	9.13	7.93	0.00630581	0.020541912
8047659	10152	*abl-interactor 2*	*ABI2*	9.59	9.55	10.50	0.006316645	0.020573243
7973306	63874	*abhydrolase domain containing 4*	*ABHD4*	11.49	11.32	10.01	0.006316853	0.020573243
8006590				5.23	5.40	5.11	0.006318367	0.020575851
7960283	93589	*calcium channel, voltage-dependent, alpha 2/delta subunit 4*	*CACNA2D 4*	7.09	7.18	6.69	0.006320226	0.020577652
8168018	286451	*Yip1 domain family, member 6*	*YIPF6*	9.11	8.98	10.09	0.006320345	0.020577652
7927550	728407, 100133130	*poly(ADP-ribose) glycohydrolase pseudogene | PRO1102*	*LOC72840 7|LOC100 133130*	5.25	5.15	6.39	0.00632552	0.020592177
7907962				6.77	6.80	6.16	0.006327041	0.020594807
7953844	440081, 1663, 642846	*DEAD/H (Asp-Glu-Ala-Asp/His) box polypeptide 12 | DEAD/H (Asp-Glu-Ala-Asp/His) box polypeptide 11 | DEAD/H (Asp-Glu-Ala-Asp/His) box polypeptide 11-like*	*DDX12|D DX11|LOC 642846*	7.78	7.76	9.40	0.006329413	0.020599168
7907970				8.31	8.49	7.56	0.006329808	0.020599168
8017421	57003	*coiled-coil domain containing 47*	*CCDC47*	7.27	7.45	9.01	0.006330891	0.020600371
7971444	1361	*carboxypeptidase B2 (plasma)*	*CPB2*	4.82	4.86	4.78	0.0063317	0.020600682
8105862	1022	*cyclin-dependent kinase 7*	*CDK7*	8.80	8.06	9.04	0.006334894	0.020606428
8046997	54529	*asparagine synthetase domain containing 1*	*ASNSD1*	8.29	8.18	8.86	0.006334779	0.020606428
8173176	139629		*XAGE-4*	6.13	6.31	5.97	0.006336712	0.020610021
7998927				8.06	8.11	10.40	0.006339526	0.02061685
8053610	64682, 285074	*anaphase promoting complex subunit 1 | anaphase promoting complex subunit 1 pseudogene*	*ANAPC1|L OC285074*	9.20	9.58	11.06	0.006345226	0.020633062
7994737	226	*aldolase A, fructose-bisphosphate*	*ALDOA*	11.30	11.47	10.62	0.006348165	0.020640293
7975404				5.42	5.30	5.19	0.006349075	0.020640928
7934244	54788	*DnaJ (Hsp40) homolog, subfamily B, member 12*	*DNAJB12*	8.60	8.68	7.58	0.00635089	0.020644505

8021900	26251	*potassium voltage-gated channel, subfamily G, member 2*	*KCNG2*	8.67	8.50	7.80	0.006351893	0.02064544
8028806	57731	*spectrin, beta, non-erythrocytic 4*	*SPTBN4*	6.67	6.79	6.32	0.006357107	0.020660061
7898483	55160	*Rho guanine nucleotide exchange factor (GEF) 10-like*	*ARHGEF10L*	8.24	8.45	7.68	0.006357976	0.020660559
8085571	131965	*methyltransferase like 6*	*METTL6*	8.28	8.49	9.14	0.006361818	0.02067072
8084732	64108	*receptor (chemosensory) transporter protein 4*	*RTP4*	6.75	6.26	5.73	0.006364657	0.020673867
8141459	79690	*galactose-3-O-sulfotransferase 4*	*GAL3ST4*	9.88	9.04	8.20	0.006363859	0.020673867
8091757	286827	*tripartite motif-containing 59*	*TRIM59*	7.48	6.95	9.28	0.006364936	0.020673867
8052121				4.67	4.71	4.58	0.006366272	0.02067588
7997801	27189	*interleukin 17C*	*IL17C*	8.15	8.29	7.59	0.006368073	0.020677216
8073449	27254	*cold shock domain containing C2, RNA binding*	*CSDC2*	7.98	8.06	7.28	0.006368867	0.020677216
8097066	57721	*methyltransferase like 14*	*METTL14*	8.47	7.79	9.47	0.006369548	0.020677216
8039054	84671	*zinc finger protein 347*	*ZNF347*	5.98	5.71	7.35	0.006369514	0.020677216
7899911	84970	*chromosome 1 open reading frame 94*	*C1orf94*	6.43	6.58	7.18	0.006371398	0.020680895
7965627	4048	*leukotriene A4 hydrolase*	*LTA4H*	9.08	8.89	11.38	0.006375692	0.020692509
7949946				6.74	6.73	6.09	0.006376532	0.020692907
8110546	9794, 4056	*mastermind-like 1 (Drosophila) | leukotriene C4 synthase*	*MAML1|LTC4S*	9.00	9.02	8.43	0.00637905	0.020698752
8104131	441058	*hypothetical LOC441058*	*MGC39584*	5.46	5.35	4.97	0.006386547	0.02072075
7969933	54841	*basic, immunoglobulin-like variable motif containing*	*BIVM*	7.98	8.24	7.29	0.006388924	0.020726131
8055688	390	*Rho family GTPase 3*	*RND3*	10.47	10.36	9.29	0.006396244	0.020744288
8000903	65988	*zinc finger protein 747*	*ZNF747*	8.64	8.70	7.91	0.006396676	0.020744288
7941350	399909	*pecanex-like 3 (Drosophila)*	*PCNXL3*	9.68	9.71	8.49	0.006396624	0.020744288
7934870	84896	*ATPase family, AAA domain containing 1*	*ATAD1*	8.39	8.31	9.44	0.006401986	0.020754514
8065327	644524	*NK2 homeobox 4*	*NKX2-4*	9.61	9.70	8.94	0.006400911	0.020754514
7913582	80818, 148898	*zinc finger protein 436 | chromosome 1 open reading frame 213*	*ZNF436|C1orf213*	8.60	9.18	6.87	0.006401746	0.020754514
7916592	114803	*Myb-like, SWIRM and MPN domains 1*	*MYSM1*	7.88	7.51	10.36	0.006405663	0.0207641
8148824	3297	*heat shock transcription factor 1*	*HSF1*	10.31	10.25	9.32	0.006409401	0.020771554

8139883				8.74	8.92	8.00	0.00640921	0.020771554
8061564	3397	*inhibitor of DNA binding 1, dominant negative helix-loop-helix protein*	*ID1*	9.42	9.87	11.89	0.006411032	0.020774506
7911351	79854, 169270	*non-protein coding RNA 115 | zinc finger protein 596*	*NCRNA00 115|ZNF59 6*	8.34	8.38	7.95	0.006414249	0.0207826
7928838	11155	*LIM domain binding 3*	*LDB3*	6.85	6.79	6.36	0.006416272	0.020786822
7997904	197320	*zinc finger protein 778*	*ZNF778*	8.93	8.77	11.05	0.006417541	0.020788599
8026991	54858, 23770	*pyroglutamyl-peptidase I | FK506 binding protein 8, 38kDa*	*PGPEP1|F KBP8*	9.12	9.03	8.09	0.006424384	0.020807832
8028117				7.73	7.83	7.09	0.00642492	0.020807832
8072687	4174	*minichromosome maintenance complex component 5*	*MCM5*	8.02	8.78	11.05	0.006426193	0.02080962
7921840	9970	*nuclear receptor subfamily 1, group I, member 3*	*NR1I3*	5.86	6.20	5.63	0.006429062	0.020815887
8080212	132158	*glycerate kinase*	*GLYCTK*	7.46	7.53	6.97	0.00642957	0.020815887
7926189	55176	*Sec61 alpha 2 subunit (S. cerevisiae)*	*SEC61A2*	8.50	8.55	9.51	0.006434144	0.020826021
8112256				5.53	5.61	5.35	0.006434066	0.020826021
8065016				10.45	10.45	9.94	0.006437474	0.020834465
8143597	9747	*family with sequence similarity 115, member A*	*FAM115A*	5.01	5.06	4.83	0.006445973	0.020859635
7951717	80975	*transmembrane protease, serine 5*	*TMPRSS5*	6.13	6.08	5.87	0.006451108	0.02087391
8019802	6066, 26855, 54663	*RNA, U2 small nuclear 1 | RNA, U2 small nuclear 2 | WD repeat domain 74*	*RNU2-1|RNU2-2|WDR74*	13.44	13.44	13.81	0.006454767	0.020883411
8078665	50853	*villin-like*	*VILL*	7.92	7.82	7.33	0.006456447	0.020886505
8093906	7466	*Wolfram syndrome 1 (wolframin)*	*WFS1*	9.85	9.41	8.46	0.006458179	0.020889767
8058535	1418	*crystallin, gamma A*	*CRYGA*	5.85	5.87	5.47	0.006460993	0.020892859
8172119	9282	*mediator complex subunit 14*	*MED14*	9.24	9.33	11.55	0.006461223	0.020892859
8174246	142684		*RAB40A*	7.08	6.98	6.53	0.006462662	0.020892859
8052902	344018	*folliculogenesis specific basic helix-loop-helix*	*FIGLA*	5.67	5.83	5.44	0.006462291	0.020892859
8073680				9.66	10.30	9.13	0.006462753	0.020892859
8104307	153571	*chromosome 5 open reading frame 38*	*C5orf38*	8.00	8.89	7.51	0.00646513	0.020898202
7993935				6.78	6.92	6.50	0.006466435	0.02090008

8105436	4214	*mitogen-activated protein kinase kinase kinase 1*	*MAP3K1*	8.08	7.50	8.74	0.00646728	0.020900473
8123405	26238, 441179	*chromosome 6 open reading frame 123 | hypothetical LOC441179*	*C6orf123| LOC441179*	6.03	6.08	5.79	0.006475131	0.020923499
8074565	85359	*DiGeorge syndrome critical region gene 6-like*	*DGCR6L*	9.56	9.40	8.62	0.006478026	0.020930514
8170166	27336	*HIV-1 Tat specific factor 1*	*HTATSF1*	6.92	6.92	8.27	0.006478982	0.020931257
8145175	64236	*PDZ and LIM domain 2 (mystique)*	*PDLIM2*	9.00	8.95	8.02	0.006479934	0.020931993
7972713	1948	*ephrin-B2*	*EFNB2*	5.94	8.00	7.36	0.006492478	0.020953756
8127526	6170	*ribosomal protein L39*	*RPL39*	11.77	11.92	11.73	0.00649226	0.020953756
7999419	28955	*Dexi homolog (mouse)*	*DEXI*	9.45	9.36	8.39	0.006491829	0.020953756
7991587	55829	*selenoprotein S*	*SELS*	8.94	9.32	8.52	0.006491796	0.020953756
8001014	146547	*protease, serine, 36*	*PRSS36*	7.78	7.76	7.26	0.006490502	0.020953756
8118498	1589, 1590	*cytochrome P450, family 21, subfamily A, polypeptide 2 | cytochrome P450, family 21, subfamily A, polypeptide 1 pseudogene*	*CYP21A2| CYP21A1P*	6.76	6.69	6.14	0.006488771	0.020953756
7912535				7.55	7.41	6.31	0.006490188	0.020953756
8045814				6.98	6.89	6.30	0.006489939	0.020953756
8078066	152273	*FYVE, RhoGEF and PH domain containing 5*	*FGD5*	6.32	6.09	6.64	0.006496186	0.02096338
7897257	388591	*ring finger protein 207*	*RNF207*	7.77	7.77	7.13	0.006500456	0.020974815
8000869	1731	*septin 1*	*1-Sep*	6.83	6.82	6.41	0.006503544	0.020979645
7974270	51062	*atlastin GTPase 1*	*ATL1*	7.14	6.98	8.01	0.006503825	0.020979645
7933572	100133130	*PRO1102*	*LOC100133130*	5.15	5.09	6.24	0.006504133	0.020979645
8071783	66035	*solute carrier family 2 (facilitated glucose transporter), member 11*	*SLC2A11*	7.61	7.60	7.14	0.006505075	0.02098034
7902235	57554	*leucine rich repeat containing 7*	*LRRC7*	5.83	5.80	5.48	0.006506669	0.020983135
8102695				7.01	7.16	6.77	0.006508336	0.020986168
8007607	10900	*RUN domain containing 3A*	*RUNDC3A*	6.83	6.82	7.20	0.006522182	0.021028467
8142084	646531	*Y box binding protein 1 pseudogene 2*	*YBX1P2*	10.52	10.57	11.07	0.006527995	0.021044858
8179593	116511	*MAS1 oncogene-like*	*MAS1L*	5.55	5.56	5.31	0.006532422	0.021056775

7908421	6738	*TROVE domain family, member 2*	*TROVE2*	7.92	7.85	9.65	0.006534076	0.02105924
7958202	50515	*carbohydrate (chondroitin 4) sulfotransferase 11*	*CHST11*	9.03	9.65	9.85	0.006534645	0.02105924
7899654	10657	*KH domain containing, RNA binding, signal transduction associated 1*	*KHDRBS1*	9.13	9.21	10.39	0.006543187	0.021075007
8179819	23564	*dimethylarginine dimethylaminohydrolase 2*	*DDAH2*	9.75	9.60	8.69	0.006542249	0.021075007
7953012	81029	*wingless-type MMTV integration site family, member 5B*	*WNT5B*	8.33	8.39	7.14	0.006541796	0.021075007
8110676	401166		*FLJ44896*	6.24	6.95	5.97	0.006542653	0.021075007
7953450	10536, 27239	*leprecan-like 2 | G protein-coupled receptor 162*	*LEPREL2| GPR162*	9.03	8.88	7.52	0.006542809	0.021075007
8115076	1436	*colony stimulating factor 1 receptor*	*CSF1R*	6.64	6.64	6.09	0.006547628	0.02108632
7961889				6.52	6.67	6.04	0.006548891	0.02108632
8114643				6.91	7.00	6.16	0.006548429	0.02108632
8124537	8356	*histone cluster 1, H3j*	*HIST1H3J*	7.49	8.65	11.27	0.006557669	0.02110519
8178590	23564	*dimethylarginine dimethylaminohydrolase 2*	*DDAH2*	9.75	9.61	8.69	0.006557124	0.02110519
7896779	339451	*kelch-like 17 (Drosophila)*	*KLHL17*	8.15	8.26	7.61	0.006557677	0.02110519
8053447				8.04	8.25	7.32	0.006556702	0.02110519
7999965	348158, 123876	*acyl-CoA synthetase medium-chain family member 2B | acyl-CoA synthetase medium-chain family member 2A*	*ACSM2B|ACSM2A*	5.84	5.82	5.66	0.006559868	0.02110989
7942647	387790		*LOC387790*	6.82	6.62	7.59	0.006566271	0.02112814
8059650	3431		*SP110*	8.20	7.58	6.90	0.006567879	0.02113096
8015412	3728	*junction plakoglobin*	*JUP*	9.06	8.69	9.85	0.006568742	0.02113138
8167862	389860	*P antigen family, member 2B*	*PAGE2B*	6.27	6.45	5.66	0.006569598	0.02113178
8169701	28985	*malignant T cell amplified sequence 1*	*MCTS1*	7.56	7.70	8.04	0.006580287	0.021163803
7934166	219793	*chromosome 10 open reading frame 27*	*C10orf27*	6.23	6.06	5.69	0.006582047	0.021167107
8083677	29970, 100505385	*schwannomin interacting protein 1 | IQ motif containing J-schwannomin interacting protein 1 read-through transcript*	*SCHIP1|IQCJ-SCHIP1*	8.45	9.33	9.47	0.00658375	0.021170225
7969488	1203	*ceroid-lipofuscinosis, neuronal 5*	*CLN5*	8.80	8.46	7.76	0.006587579	0.021180179
7998759	283871	*phosphoglycolate phosphatase*	*PGP*	9.01	9.30	10.31	0.006589562	0.021184195

7901611	619518	*chromosome 1 open reading frame 191*	*C1orf191*	6.91	6.94	6.47	0.006591215	0.02118715
7984743	80381		*CD276*	10.28	10.23	9.50	0.006594151	0.021194227
7973898				7.87	7.70	7.41	0.006594952	0.021194441
7993713	124152	*IQ motif containing K*	*IQCK*	8.94	8.31	7.99	0.006597768	0.02120113
8060526				6.30	6.29	5.91	0.006598565	0.021201331
7941269	100291851	*putative ubiquitin-like protein FUBI-like protein ENSP00000310146-like*	*LOC100291851*	8.68	9.11	7.51	0.006599433	0.021201763
7961031	440081, 1663, 642846	*DEAD/H (Asp-Glu-Ala-Asp/His) box polypeptide 12 | DEAD/H (Asp-Glu-Ala-Asp/His) box polypeptide 11 | DEAD/H (Asp-Glu-Ala-Asp/His) box polypeptide 11-like*	*DDX12|DDX11|LOC642846*	7.86	7.90	9.33	0.006600508	0.021202855
7959777	140707	*BRI3 binding protein*	*BRI3BP*	8.28	8.52	9.27	0.006605497	0.02121652
8040090	9781	*ring finger protein 144A*	*RNF144A*	6.48	6.58	7.34	0.006607018	0.021216748
8105144				7.40	7.11	9.19	0.006607038	0.021216748
8158866	10585	*protein-O-mannosyltransferase 1*	*POMT1*	9.54	9.34	8.31	0.00661063	0.021225924
7898721				5.45	5.74	5.24	0.006612817	0.021230586
8020549				7.22	6.93	6.28	0.00661585	0.021237958
7965484				6.45	6.55	6.10	0.006619676	0.021247879
8015821	4222	*mesenchyme homeobox 1*	*MEOX1*	7.26	7.36	6.89	0.00662047	0.021248065
8042830	10797	*methylenetetrahydrofolate dehydrogenase (NADP+ dependent) 2, methenyltetrahydrofolate cyclohydrolase*	*MTHFD2*	10.31	10.84	11.91	0.00662236	0.02125177
7942596	871	*serpin peptidase inhibitor, clade H (heat shock protein 47), member 1, (collagen binding protein 1)*	*SERPINH1*	13.15	13.24	12.29	0.006624242	0.021255447
8071989	1417	*crystallin, beta B3*	*CRYBB3*	7.79	7.91	7.35	0.006632061	0.021275805
7915991	63950	*DMRT-like family A2*	*DMRTA2*	7.39	7.44	6.95	0.006631667	0.021275805
8090416				5.39	5.51	5.00	0.006636448	0.021287512
8069153	353333	*keratin associated protein 10-10*	*KRTAP10-10*	8.00	8.12	7.37	0.006638552	0.021291897
7955112	341567	*H1 histone family, member N, testis-specific*	*H1FNT*	7.96	8.06	7.52	0.006639926	0.021293938
8001858	283847	*coiled-coil domain containing 79*	*CCDC79*	4.83	4.79	4.71	0.006642704	0.021300482
8036883	147746	*homeodomain interacting protein kinase 4*	*HIPK4*	7.17	7.08	6.60	0.006648355	0.021316234

7940002	219527	*leucine rich repeat containing 55*	*LRRC55*	6.89	6.93	6.44	0.0066517	0.02132459
8043157	284948	*SH2 domain containing 6*	*SH2D6*	6.91	6.95	6.39	0.006652975	0.021326307
8064978	182	*jagged 1*	*JAG1*	9.16	7.65	8.98	0.006654393	0.021328485
8139131	273	*amphiphysin*	*AMPH*	6.86	7.40	6.61	0.006657063	0.02133145
7981068	5265	*serpin peptidase inhibitor, clade A (alpha-1 antiproteinase, antitrypsin), member 1*	*SERPINA1*	6.92	6.53	6.32	0.006657534	0.02133145
7947694	9793	*cytoskeleton associated protein 5*	*CKAP5*	8.42	8.27	10.75	0.00665702	0.02133145
8160602	54840	*aprataxin*	*APTX*	7.17	7.12	7.87	0.006659283	0.021334684
8099242	257236	*coiled-coil domain containing 96*	*CCDC96*	6.36	6.41	5.84	0.006661682	0.021340001
8124413	8360	*histone cluster 1, H4d*	*HIST1H4D*	8.02	8.19	10.25	0.006667164	0.021355193
8004534	6462	*sex hormone-binding globulin*	*SHBG*	7.05	6.85	6.49	0.006674895	0.021363892
8026631	9002	*coagulation factor II (thrombin) receptor-like 3*	*F2RL3*	7.00	7.17	6.58	0.006674583	0.021363892
8020037	23255		*KIAA0802*	8.61	8.65	7.87	0.00667728	0.021363892
7983413	53905	*dual oxidase 1*	*DUOX1*	6.40	6.57	5.98	0.006673748	0.021363892
8126173	83795	*potassium channel, subfamily K, member 16*	*KCNK16*	7.09	7.11	6.54	0.006673174	0.021363892
8057418	151126	*zinc finger protein 385B*	*ZNF385B*	6.60	6.56	6.95	0.006674467	0.021363892
8070789	386684	*keratin associated protein 12-4*	*KRTAP12-4*	7.88	7.93	7.40	0.006675276	0.021363892
8109990	644994	*chromosome 5 open reading frame 50*	*C5orf50*	6.44	6.41	6.13	0.006677128	0.021363892
8175609				6.82	6.69	6.11	0.00667703	0.021363892
8176921				9.01	9.12	8.46	0.006676337	0.021363892
7914350				7.36	7.22	6.61	0.006678752	0.021366233
8139264	84820, 441273, 246721, 548644	*polymerase (RNA) II (DNA directed) polypeptide J4, pseudogene | speedy homolog E2 (Xenopus laevis) | polymerase (RNA) II (DNA directed) polypeptide J2 | polymerase (RNA) II (DNA directed) polypeptide J3*	*POLR2J4|SPDYE2|POLR2J2|POLR2J3*	12.16	12.48	11.24	0.00668097	0.021368591
7981728				6.96	7.31	6.49	0.00668085	0.021368591
8036444	126231	*zinc finger protein 573*	*ZNF573*	5.55	5.44	6.40	0.006683465	0.021374203
8029076	90273	*carcinoembryonic antigen-related cell adhesion molecule 21*	*CEACAM21*	6.39	6.41	6.06	0.006687321	0.021381801

8178205	3118, 3117	*major histocompatibility complex, class II, DQ alpha 2 | major histocompatibility complex, class II, DQ alpha 1*	*HLA-DQA2|HLA-DQA1*	6.22	6.23	5.77	0.006687318	0.021381801
7913864	3925	*stathmin 1*	*STMN1*	6.89	7.01	6.27	0.006691608	0.021393137
8175444	2258	*fibroblast growth factor 13*	*FGF13*	6.80	7.21	8.04	0.006694158	0.02139655
7943882	5805	*6-pyruvoyltetrahydropterin synthase*	*PTS*	8.25	8.91	8.53	0.00669364	0.02139655
7952227	114902, 83552	*C1q and tumor necrosis factor related protein 5 | membrane frizzled-related protein*	*C1QTNF5|MFRP*	7.53	7.64	7.03	0.006698265	0.021407306
8035956				8.39	8.49	7.68	0.006704222	0.021423973
8166006	265	*amelogenin, X-linked*	*AMELX*	5.46	5.52	5.20	0.006706024	0.021427361
8166632	2710	*glycerol kinase*	*GK*	7.91	7.12	8.58	0.006708549	0.021433057
8128472	10973	*activating signal cointegrator 1 complex subunit 3*	*ASCC3*	8.35	8.05	10.07	0.006709818	0.021434737
8030578	400710		*FLJ26850*	6.48	6.51	5.97	0.006718685	0.021460688
8112687	10087	*collagen, type IV, alpha 3 (Goodpasture antigen) binding protein*	*COL4A3BP*	7.79	7.71	9.32	0.006719461	0.021460796
8140500	85025	*transmembrane protein 60*	*TMEM60*	8.31	8.23	9.26	0.006723851	0.02147244
8081903	89876	*chromosome 3 open reading frame 15*	*C3orf15*	5.16	5.31	5.61	0.006726046	0.021477072
8068024	58494	*junctional adhesion molecule 2*	*JAM2*	8.15	10.22	9.34	0.006728163	0.02148043
8156620	441457, 54754, 728118	*family with sequence similarity 22, member G | family with sequence similarity 22, member F | family with sequence similarity 22, member A*	*FAM22G|FAM22F|FAM22A*	7.05	7.17	6.72	0.006728585	0.02148043
8085114				6.16	5.36	5.36	0.00673023	0.021483304
8162639	158431	*zinc finger protein 782*	*ZNF782*	6.14	5.89	6.89	0.006731905	0.021486275
8177424				9.01	9.12	8.46	0.006735	0.02149378
7937533	161	*adaptor-related protein complex 2, alpha 2 subunit*	*AP2A2*	9.46	9.65	8.74	0.006736091	0.021494882
8044499	6574	*solute carrier family 20 (phosphate transporter), member 1*	*SLC20A1*	9.00	9.45	10.20	0.006738558	0.021497235
7948282	219539	*yippee-like 4 (Drosophila)*	*YPEL4*	6.70	6.70	6.21	0.00673769	0.021497235
7946027				5.99	6.26	5.73	0.006739062	0.021497235
8108478	84418	*chromosome 5 open reading frame 32*	*C5orf32*	9.30	9.39	8.59	0.006743559	0.021509202

7993546	339044, 9284, 399491, 100288332, 642778	*polycystic kidney disease 1 (autosomal dominant) pseudogene 1 | nuclear pore complex interacting protein | GPS, PLAT and transmembrane domain-containing protein | GPS, PLAT and transmembrane domain-containing protein FLJ00285-like | nuclear pore complex-interacting protein-like 1-like*	*PKD1P1|NPIP|LOC399491|LOC100288332|LOC642778*	11.68	11.69	10.45	0.0067502	0.02152801
7972018	100288208		*LOC100288208*	5.37	5.32	5.26	0.006757513	0.02154895
8178177	1589, 1590	*cytochrome P450, family 21, subfamily A, polypeptide 2 | cytochrome P450, family 21, subfamily A, polypeptide 1 pseudogene*	*CYP21A2|CYP21A1P*	6.61	6.54	6.00	0.006759307	0.021552289
8100572				4.89	4.95	4.67	0.00676099	0.021555277
7917148	64853	*axin interactor, dorsalization associated*	*AIDA*	10.12	10.06	11.07	0.006762446	0.021557538
7904131	64858	*DNA cross-link repair 1B*	*DCLRE1B*	6.68	7.27	7.46	0.006764904	0.02156061
7976154				7.13	7.25	6.58	0.006764507	0.02156061
7999754	64131	*xylosyltransferase I*	*XYLT1*	9.00	9.88	9.02	0.006766507	0.02156334
8051956	400952		*UNQ6975*	5.09	5.11	4.85	0.006768868	0.021568483
8162047	3190	*heterogeneous nuclear ribonucleoprotein K*	*HNRNPK*	10.99	11.18	12.63	0.00677328	0.021577775
7917019	127254	*chromosome 1 open reading frame 173*	*C1orf173*	5.14	5.18	5.00	0.006772822	0.021577775
8074806	9647	*protein phosphatase, Mg2+/Mn2+ dependent, 1F*	*PPM1F*	8.50	8.60	7.58	0.006775034	0.021579709
8041197	51646	*yippee-like 5 (Drosophila)*	*YPEL5*	10.64	10.56	9.79	0.006775381	0.021579709
8068220	54067	*chromosome 21 open reading frame 49*	*C21orf49*	6.23	6.01	5.55	0.006776399	0.02158057
8102986	166752	*FRAS1 related extracellular matrix 3*	*FREM3*	5.41	5.33	5.19	0.006779164	0.021586994
8076584	150378		*FLJ30901*	7.73	7.86	7.30	0.006783841	0.021599503
7998251	84326	*chromosome 16 open reading frame 13*	*C16orf13*	9.35	9.39	8.60	0.006786579	0.021605838
8075285	84164	*activating signal cointegrator 1 complex subunit 2*	*ASCC2*	10.98	10.96	10.08	0.006789735	0.021613501
7934141	64106	*neuropeptide FF receptor 1*	*NPFFR1*	7.45	7.62	6.85	0.006793675	0.02161458
8156549	84909	*chromosome 9 open reading frame 3*	*C9orf3*	7.75	7.59	7.22	0.006794565	0.02161458
8023864	125704	*family with sequence similarity 69, member C*	*FAM69C*	6.17	6.08	5.74	0.00679441	0.02161458
8010017	100134934, 1018	*chromosome 17 open reading frame 106 | cyclin-dependent kinase 3*	*C17orf106|CDK3*	8.95	8.96	8.36	0.006792103	0.02161458

8024682	58509, 404665	*chromosome 19 open reading frame 29 | chromosome 19 open reading frame 29 opposite strand*	*C19orf29|C19orf29OS*	6.97	6.94	6.21	0.006792414	0.02161458
8034313	664709, 3178, 144983, 728643	*heterogeneous nuclear ribonucleoprotein A1 pseudogene 10 | heterogeneous nuclear ribonucleoprotein A1-like 2*	*HNRNPA1P10|HNRNPA1L2|LOC728643*	10.65	10.47	12.86	0.006794158	0.02161458
8097388	55132	*La ribonucleoprotein domain family, member 1B*	*LARP1B*	6.97	6.79	7.61	0.006795813	0.021616166
7990283	342096, 55889, 645752, 653641, 653643	*golgin A6 family, member A | golgin A6 family, member B | golgi autoantigen, golgin subfamily a, 6 pseudogene | golgin A6 family, member C | golgin A6 family, member D*	*GOLGA6A|GOLGA6B|LOC645752|GOLGA6C|GOLGA6D*	6.55	6.60	6.32	0.006801977	0.021631006
7968796				5.42	5.40	5.14	0.006801818	0.021631006
8063240				6.47	6.57	6.08	0.006805521	0.021639893
7962085	10526	*importin 8*	*IPO8*	8.01	7.38	9.53	0.006807409	0.021643512
7936923	228	*aldolase A, fructose-bisphosphate pseudogene 2*	*ALDOAP2*	8.03	8.03	7.39	0.006814665	0.021664195
7911767	79258, 127281	*membrane metallo-endopeptidase-like 1 | chromosome 1 open reading frame 93*	*MMEL1|C1orf93*	6.60	6.57	6.15	0.006815563	0.021664664
7918444	3744	*potassium voltage-gated channel, shaker-related subfamily, member 10*	*KCNA10*	7.22	7.27	6.79	0.006820956	0.02167942
7985166	3658	*iron-responsive element binding protein 2*	*IREB2*	7.99	7.79	10.50	0.006824206	0.021685906
7967702				5.59	5.52	5.02	0.006824499	0.021685906
7910111	2052	*epoxide hydrolase 1, microsomal (xenobiotic)*	*EPHX1*	11.95	11.08	10.39	0.006825863	0.021687852
8081407	131368	*zona pellucida-like domain containing 1*	*ZPLD1*	5.97	6.08	5.81	0.00683122	0.021702487
8015218	100132476, 100132386, 728224, 81871	*keratin associated protein 4-7 | keratin associated protein 4-9 | keratin associated protein 4-8 | keratin associated protein 4-6*	*KRTAP4-7|KRTAP4-9|KRTAP4-8|KRTAP4-6*	8.57	8.84	7.90	0.006837198	0.021719089

8058428	4719	*NADH dehydrogenase (ubiquinone) Fe-S protein 1, 75kDa (NADH-coenzyme Q reductase)*	*NDUFS1*	8.51	8.72	10.15	0.006839625	0.021724407
8038804	125875	*claudin domain containing 2*	*CLDND2*	7.84	7.90	7.34	0.00684426	0.021736737
8141380	7551, 7589	*zinc finger protein 3 | zinc finger and SCAN domain containing 21*	*ZNF3|ZSCAN21*	8.62	8.34	9.66	0.006846004	0.021739885
7940226	2206	*membrane-spanning 4-domains, subfamily A, member 2 (Fc fragment of IgE, high affinity I, receptor for; beta polypeptide)*	*MS4A2*	4.86	4.93	4.71	0.006850723	0.021750005
8081620	29114	*transgelin 3*	*TAGLN3*	6.47	6.53	7.27	0.006852203	0.021750005
7972743				6.12	6.04	5.99	0.006852205	0.021750005
8065325				11.55	11.58	12.12	0.006851632	0.021750005
8000567	246778	*interleukin 27*	*IL27*	7.36	7.49	7.02	0.006857631	0.021764837
7946069	79345	*olfactory receptor, family 51, subfamily B, member 2*	*OR51B2*	6.43	6.37	6.12	0.006858493	0.02176518
8112857	345778	*metaxin 3*	*MTX3*	7.25	6.75	9.01	0.00686087	0.02177033
8117422	8361	*histone cluster 1, H4f*	*HIST1H4F*	7.93	8.39	10.24	0.006861736	0.021770682
7977409	1396	*cysteine-rich protein 1 (intestinal)*	*CRIP1*	8.68	8.53	7.49	0.006863781	0.021774778
8169473	5358	*plastin 3*	*PLS3*	10.14	10.05	11.42	0.006866544	0.02178115
7947363	7490	*Wilms tumor 1*	*WT1*	6.81	6.86	6.25	0.006869566	0.021788342
7948744	23193	*glucosidase, alpha; neutral AB*	*GANAB*	12.14	12.18	11.46	0.006874866	0.02179923
7945071	55572	*FAD-dependent oxidoreductase domain containing 1*	*FOXRED1*	8.27	8.51	9.83	0.006875264	0.02179923
8169419	79868	*asparagine-linked glycosylation 13 homolog (S. cerevisiae)*	*ALG13*	6.70	6.67	8.30	0.006874947	0.02179923
8043347				6.76	7.09	5.93	0.006876565	0.02180096
7917472	2959	*general transcription factor IIB*	*GTF2B*	8.93	8.95	9.66	0.006881056	0.021806417
8012475	4628	*myosin, heavy chain 10, non-muscle*	*MYH10*	8.74	8.17	11.23	0.006881307	0.021806417
7946589	10335	*murine retrovirus integration site 1 homolog*	*MRVI1*	7.83	8.15	6.52	0.006881199	0.021806417
8056489				5.62	5.52	5.20	0.006880875	0.021806417
8040503	165324	*UBX domain protein 2A*	*UBXN2A*	7.05	7.27	8.02	0.006882321	0.021807237

8043360	50802, 28876, 28875	*immunoglobulin kappa locus | immunoglobulin kappa variable 3D-11 | immunoglobulin kappa variable 3D-15 (gene/pseudogene)*	*IGK@|IGKV3D-11|IGKV3D-15*	6.55	6.54	6.16	0.006887678	0.021821816
7932160	83641	*family with sequence similarity 107, member B*	*FAM107B*	8.77	7.72	8.44	0.006889633	0.021823218
8094456	389203	*chromosome 4 open reading frame 52*	*C4orf52*	8.14	8.06	8.87	0.006889424	0.021823218
8039409	255043	*transmembrane protein 86B*	*TMEM86B*	7.38	7.51	6.84	0.006899218	0.021851182
7936614	8661	*eukaryotic translation initiation factor 3, subunit A*	*EIF3A*	9.79	9.64	11.72	0.006900451	0.02185193
8051926				6.62	6.69	6.19	0.006900968	0.02185193
7998494	645811	*coiled-coil domain containing 154*	*CCDC154*	7.41	7.61	7.00	0.006902128	0.021853207
7978407	5587	*protein kinase D1*	*PRKD1*	8.14	9.68	7.72	0.006907682	0.021866906
8065370				6.02	6.00	5.77	0.00690797	0.021866906
7945436	3265	*v-Ha-ras Harvey rat sarcoma viral oncogene homolog*	*HRAS*	9.37	9.54	8.33	0.006911851	0.021871997
8116247	6940	*zinc finger protein 354A*	*ZNF354A*	6.77	6.66	7.67	0.006911725	0.021871997
7968226	6144, 10013,1205	*ribosomal protein L21 | ribosomal protein L21 pseudogene 28*	*RPL21|RPL21P28*	11.00	11.15	11.41	0.00691159	0.021871997
8126288	79865	*triggering receptor expressed on myeloid cells-like 2*	*TREML2*	6.68	6.92	6.34	0.006914716	0.021878665
8150864				7.54	7.70	7.04	0.00691988	0.021892605
8082305	84303	*coiled-coil-helix-coiled-coil-helix domain containing 6*	*CHCHD6*	8.44	8.39	9.20	0.006924922	0.021898957
8132805	375567	*von Willebrand factor C domain containing 2*	*VWC2*	8.42	8.39	7.73	0.006924154	0.021898957
8082163				7.64	7.62	7.13	0.006924592	0.021898957
8172191				7.40	7.42	6.82	0.006923589	0.021898957
8066444	79015		*LOC79015*	6.91	6.94	5.81	0.006929604	0.021908402
8123488	80069	*chromosome 6 open reading frame 208*	*C6orf208*	7.08	6.96	6.70	0.006930186	0.021908402
8176400	83259, 27328	*protocadherin 11 Y-linked | protocadherin 11 X-linked*	*PCDH11Y|PCDH11X*	5.56	5.55	6.86	0.006930165	0.021908402

8071170	29797, 646074, 29774, 25812	*POM121 membrane glycoprotein-like 8 pseudogene | POM121 membrane glycoprotein-like 10, pseudogene | POM121 membrane glycoprotein-like 9, pseudogene | POM121 membrane glycoprotein-like 1, pseudogene*	*POM121L8P|POM121L10P|POM121L9P|POM121L1P*	6.99	6.94	6.67	0.006935897	0.021924058
8065527	284800, 728882	*family with sequence similarity 182, member A | family with sequence similarity 182, member B*	*FAM182A|FAM182B*	6.83	6.90	6.34	0.006936994	0.021925125
8049934	64090, 129807	*galactose-3-O-sulfotransferase 2 | sialidase 4*	*GAL3ST2|NEU4*	7.62	7.75	7.17	0.006945943	0.021951005
8083968	22871	*neuroligin 1*	*NLGN1*	8.74	6.58	8.00	0.006946949	0.02195178
7911458	116983	*ArfGAP with coiled-coil, ankyrin repeat and PH domains 3*	*ACAP3*	8.32	8.36	7.35	0.006953533	0.02197018
8085867	152110	*NIMA (never in mitosis gene a)- related kinase 10*	*NEK10*	6.46	6.19	5.82	0.006954875	0.021972016
8128284	2045	*EPH receptor A7*	*EPHA7*	5.59	5.67	7.10	0.006959205	0.02198329
8079346	22908	*SAC1 suppressor of actin mutations 1-like (yeast)*	*SACM1L*	8.52	8.08	10.12	0.006961275	0.021985017
7897955	343066	*arylacetamide deacetylase-like 4*	*AADACL4*	5.59	5.56	5.21	0.006961169	0.021985017
8018993	146713	*RNA binding protein, fox-1 homolog (C. elegans) 3*	*RBFOX3*	7.15	7.29	7.99	0.006963278	0.021986533
8130462				5.68	5.57	5.24	0.006963265	0.021986533
8160792	84688	*chromosome 9 open reading frame 24*	*C9orf24*	7.02	6.90	6.34	0.006968093	0.02199933
7991386	10519, 390637	*calcium and integrin binding 1 (calmyrin) | chromosome 15 open reading frame 58*	*CIB1|C15orf58*	9.76	9.85	8.38	0.006972956	0.022012275
8132292	989	*septin 7*	*7-Sep*	9.77	9.41	9.86	0.006981568	0.022034641
8065798	2686	*gamma-glutamyltransferase 7*	*GGT7*	9.26	9.02	7.95	0.00698094	0.022034641
7991143	23478	*SEC11 homolog A (S. cerevisiae)*	*SEC11A*	8.99	8.96	9.34	0.006985247	0.022038417
7942476	80227	*proteasomal ATPase-associated factor 1*	*PAAF1*	7.73	7.32	8.42	0.006985817	0.022038417
7954655	1663, 440081, 642846	*DEAD/H (Asp-Glu-Ala-Asp/His) box polypeptide 11 | DEAD/H (Asp-Glu-Ala-Asp/His) box polypeptide 12 | DEAD/H (Asp-Glu-Ala-Asp/His) box polypeptide 11-like*	*DDX11|DDX12|LOC642846*	8.11	8.03	9.39	0.006984125	0.022038417
8013507				7.70	7.70	7.11	0.006984906	0.022038417
8022870				6.48	6.76	5.88	0.006988627	0.022044873
8115464	84868	*hepatitis A virus cellular receptor 2*	*HAVCR2*	5.26	5.34	5.13	0.006990076	0.022047034

8130715	285800	*proline rich 18*	*PRR18*	8.22	8.26	7.57	0.006991674	0.022049664
7968265	3651	*pancreatic and duodenal homeobox 1*	*PDX1*	7.56	7.56	7.07	0.006995556	0.022057172
8063497	57091	*Cas scaffolding protein family member 4*	*CASS4*	6.04	6.17	5.64	0.006996347	0.022057172
7939930	219431	*olfactory receptor, family 4, subfamily S, member 2*	*OR4S2*	5.11	5.14	4.92	0.006995843	0.022057172
8119408	4800	*nuclear transcription factor Y, alpha*	*NFYA*	7.90	8.19	9.72	0.0069979	0.02205966
8088889				7.52	7.64	7.00	0.007001579	0.022068849
8091558				4.39	4.43	4.35	0.007003402	0.022072185
8070786	386674, 386672, 386675, 353333, 386685	*keratin associated protein 10-6 | keratin associated protein 10-4 | keratin associated protein 10-7 | keratin associated protein 10-10 | keratin associated protein 10-12*	*KRTAP10-6|KRTAP10-4|KRTAP10-7|KRTAP10-10|KRTAP10-12*	7.63	7.65	7.27	0.007005004	0.022074822
8114767	5097	*protocadherin 1*	*PCDH1*	7.21	7.36	8.37	0.007007166	0.022079226
7898184	51032	*chymotrypsin-like elastase family, member 2B*	*CELA2B*	6.56	6.53	6.09	0.007009841	0.022085246
8001104				6.69	6.78	6.38	0.007012768	0.022092056
8143749	168544	*zinc finger protein 467*	*ZNF467*	7.41	7.50	6.79	0.007022289	0.022119636
7898616	64600	*phospholipase A2, group IIF*	*PLA2G2F*	7.15	7.15	6.66	0.00702689	0.022131715
8122409	8504	*peroxisomal biogenesis factor 3*	*PEX3*	6.89	6.79	7.93	0.007035472	0.022153419
8083599	152078	*chromosome 3 open reading frame 55*	*C3orf55*	8.57	7.38	7.02	0.007036083	0.022153419
8022100				6.25	6.14	5.75	0.007035189	0.022153419
8010638	9146	*hepatocyte growth factor-regulated tyrosine kinase substrate*	*HGS*	9.77	10.00	8.93	0.007037667	0.022155989
7990879	79631	*elongation factor Tu GTP binding domain containing 1*	*EFTUD1*	5.92	6.40	7.60	0.007040931	0.022163847
7927167				5.69	5.71	5.31	0.007042096	0.022165097
8117377	3008	*histone cluster 1, H1e*	*HIST1H1E*	13.07	13.28	13.85	0.007044435	0.022170043
8012403	9212	*aurora kinase B*	*AURKB*	8.54	9.26	11.42	0.00704627	0.022173401
8031847	284312	*zinc finger and SCAN domain containing 1*	*ZSCAN1*	8.10	8.06	7.46	0.007048873	0.022177171

7980113				6.03	5.39	5.34	0.007049005	0.022177171
7902799	339524, 9653	*hypothetical LOC339524 | heparan sulfate 2-O-sulfotransferase 1*	*LOC339524|HS2ST1*	6.52	6.63	7.21	0.007051603	0.022180552
8172252				6.29	6.27	5.78	0.007051616	0.022180552
7932447				4.78	4.72	4.59	0.007052491	0.022180887
7996241	79918	*SET domain containing 6*	*SETD6*	7.97	8.11	8.59	0.007053966	0.02218311
8067183				7.90	7.68	7.29	0.007057444	0.022191629
7905548	6707	*small proline-rich protein 3*	*SPRR3*	6.23	6.30	5.83	0.007058903	0.022193799
7977693	57680	*chromodomain helicase DNA binding protein 8*	*CHD8*	8.14	7.94	9.79	0.007060436	0.022196203
7908178	54823	*chromosome 1 open reading frame 26*	*C1orf26*	6.57	6.65	7.66	0.00706491	0.022207849
7937728	7136	*troponin I type 2 (skeletal, fast)*	*TNNI2*	7.37	7.38	6.85	0.0070705	0.022218162
8069146	386675	*keratin associated protein 10-7*	*KRTAP10-7*	6.73	6.99	6.31	0.007070314	0.022218162
8019071	57674, 100294362	*ring finger protein 213 | hypothetical LOC100294362*	*RNF213|LOC100294362*	6.80	6.87	6.25	0.007069079	0.022218162
8135705	3751	*potassium voltage-gated channel, Shal-related subfamily, member 2*	*KCND2*	5.71	6.78	8.38	0.007074604	0.022228641
8173428				7.48	7.52	7.27	0.007079226	0.022240743
8118721	221504	*zinc finger and BTB domain containing 9*	*ZBTB9*	7.83	7.70	8.69	0.007080453	0.022242175
7978222	7051	*transglutaminase 1 (K polypeptide epidermal type I, protein-glutamine-gamma-glutamyltransferase)*	*TGM1*	7.06	7.03	6.66	0.00708261	0.022246532
8062539	140679	*solute carrier family 32 (GABA vesicular transporter), member 1*	*SLC32A1*	6.86	7.10	6.62	0.007083961	0.022248354
8171867	170302	*aristaless related homeobox*	*ARX*	8.06	8.19	7.45	0.007088107	0.022258954
7940630	10647	*secretoglobin, family 1D, member 2*	*SCGB1D2*	5.23	5.19	5.04	0.007091681	0.022267753
8028643				7.48	7.47	7.05	0.007095383	0.022276955
7939150	79056	*proline rich Gla (G-carboxyglutamic acid) 4 (transmembrane)*	*PRRG4*	5.82	5.97	6.83	0.007096939	0.022276998
8118890	222663	*signal peptide, CUB domain, EGF-like 3*	*SCUBE3*	7.34	7.79	6.87	0.007096193	0.022276998
7903592	57535		*KIAA1324*	7.76	7.42	6.70	0.007100795	0.022286674
8107375	64848	*YTH domain containing 2*	*YTHDC2*	7.62	7.31	9.12	0.007103475	0.022290243

8101141				6.56	6.51	6.06	0.007103023	0.022290243
8080894				4.88	4.85	4.64	0.007104784	0.022291925
7995674	79190	*iroquois homeobox 6*	*IRX6*	7.62	7.45	7.09	0.007105882	0.02229295
8072242	4771	*neurofibromin 2 (merlin)*	*NF2*	10.28	9.96	8.86	0.007109353	0.022300318
8176155	100132967	*non-protein coding RNA 204*	*NCRNA00 204*	5.70	5.48	5.18	0.007109776	0.022300318
8115039				6.01	6.02	5.80	0.007113534	0.02230968
7971620	3839	*karyopherin alpha 3 (importin alpha 4)*	*KPNA3*	9.43	9.55	10.87	0.007116375	0.022316165
7913705	1269	*cannabinoid receptor 2 (macrophage)*	*CNR2*	6.45	6.52	5.99	0.007117797	0.022316514
8072962	85377	*MICAL-like 1*	*MICALL1*	8.95	9.38	7.95	0.007118032	0.022316514
8059955	64284		*RAB17*	6.88	7.09	7.90	0.007120148	0.022320723
7944363				5.11	5.30	4.85	0.007122605	0.022326004
8011823	84622	*zinc finger protein 594*	*ZNF594*	6.18	6.21	7.13	0.007124629	0.022329923
8065719	11264	*peroxisomal membrane protein 4, 24kDa*	*PXMP4*	8.84	8.72	7.62	0.0071257	0.022330856
8041536	285154	*chromosome 2 open reading frame 58*	*C2orf58*	5.79	5.89	5.42	0.007129962	0.022341786
8158825	84814	*phosphatidic acid phosphatase type 2 domain containing 3*	*PPAPDC3*	8.04	8.28	7.17	0.007134179	0.022352574
8043861	9669	*eukaryotic translation initiation factor 5B*	*EIF5B*	7.42	7.34	9.81	0.007138131	0.02236253
7920220	114771	*peptidoglycan recognition protein 3*	*PGLYRP3*	6.27	6.35	5.85	0.007142152	0.022372698
8072353	646513	*VLGN1945*	*LOC64651 3*	6.41	6.44	6.17	0.007146192	0.022382924
8021984	7525	*v-yes-1 Yamaguchi sarcoma viral oncogene homolog 1*	*YES1*	7.82	7.46	10.15	0.0071488	0.022388663
7997264	1504, 440387	*chymotrypsinogen B1 | chymotrypsinogen B2*	*CTRB1|CT RB2*	7.60	7.61	7.11	0.00715566	0.022407716
8057898	57520	*HECT, C2 and WW domain containing E3 ubiquitin protein ligase 2*	*HECW2*	7.67	6.76	6.83	0.007156712	0.02240858
7964466	10540	*dynactin 2 (p50)*	*DCTN2*	11.57	11.54	10.63	0.007158498	0.022411743
8145227	23221	*Rho-related BTB domain containing 2*	*RHOBTB2*	8.62	8.61	7.38	0.007163227	0.022421684
7896983	100288313	*similar to hCG1820414*	*LOC10028 8313*	7.20	7.48	6.67	0.007162743	0.022421684
8097096				8.90	8.75	8.03	0.00716826	0.022435004

7939465	51144	*hydroxysteroid (17-beta) dehydrogenase 12*	*HSD17B12*	9.41	9.40	10.36	0.007175352	0.022454768
8113071	729040		*LOC729040*	8.22	8.31	7.67	0.007179815	0.022466298
8135250	5701	*proteasome (prosome, macropain) 26S subunit, ATPase, 2*	*PSMC2*	9.94	9.85	11.01	0.007181311	0.022468545
7996164	374739	*testis, prostate and placenta expressed*	*TEPP*	7.40	7.48	6.75	0.007183739	0.022473704
8037433	7596	*zinc finger protein 45*	*ZNF45*	6.03	6.24	7.11	0.007188221	0.022485293
8179246	401250	*mitochondrial coiled-coil domain 1*	*MCCD1*	7.63	7.74	7.29	0.007190391	0.022489643
8064242	284739	*non-protein coding RNA 176*	*NCRNA00176*	7.63	7.69	6.96	0.007191419	0.02249042
8077513	25917	*THUMP domain containing 3*	*THUMPD3*	8.04	8.14	9.87	0.0071995	0.022509852
8009255	90799	*coiled-coil domain containing 45*	*CCDC45*	8.03	8.13	9.39	0.007199435	0.022509852
8170249	728712, 30014, 64663, 64648, 171489	*SPANX family, member A2 | sperm protein associated with the nucleus, X-linked, family member A1 | SPANX family, member C | SPANX family, member D | SPANX family, member E*	*SPANXA2|SPANXA1|SPANXC|SPANXD|SPANXE*	5.71	5.79	5.52	0.007199972	0.022509852
8178164	8859	*serine/threonine kinase 19*	*STK19*	8.55	8.51	7.72	0.007205619	0.022524353
8144600	83650	*acyl-malonyl condensing enzyme 1-like 2*	*AMAC1L2*	5.29	5.54	5.27	0.00720617	0.022524353
8144619				6.44	6.60	6.13	0.007210793	0.022536363
7955441	25840	*methyltransferase like 7A*	*METTL7A*	8.13	6.70	7.56	0.00721246	0.02253857
8033587	84106	*PML-RARA regulated adaptor molecule 1*	*PRAM1*	7.31	7.38	6.83	0.007213061	0.02253857
8161154	392307	*chromosome 9 open reading frame 128*	*C9orf128*	5.74	5.80	5.50	0.007216977	0.022548364
7964316	4640, 6866	*myosin IA | tachykinin 3*	*MYO1A|TAC3*	6.22	6.17	5.76	0.007218344	0.022550197
7991453	400451	*family with sequence similarity 174, member B*	*FAM174B*	8.88	8.35	9.09	0.007223764	0.022564687
7902203				6.61	6.62	6.02	0.007227239	0.022573099
7908637	2848	*G protein-coupled receptor 25*	*GPR25*	8.08	8.08	7.65	0.007229237	0.022574196
8045309	90557, 91409	*coiled-coil domain containing 74A | coiled-coil domain containing 74B*	*CCDC74A|CCDC74B*	8.65	8.58	7.61	0.00722911	0.022574196

8163253				7.20	6.00	4.79	0.007229936	0.022574196
8035801				7.71	7.82	7.22	0.007232074	0.022578431
8076007	80115	*BAI1-associated protein 2-like 2*	*BAIAP2L2*	8.15	7.94	7.54	0.007235621	0.02258517
7998265	100130285		*LOC100130285*	8.66	8.83	8.26	0.007235798	0.02258517
8101212	10983	*cyclin I*	*CCNI*	12.83	12.67	12.20	0.007236756	0.02258572
8093258	84223	*IQ motif containing G*	*IQCG*	7.10	7.10	7.71	0.007240296	0.022594325
8094032	54436	*SH3 domain and tetratricopeptide repeats 1*	*SH3TC1*	7.36	7.33	6.85	0.007241419	0.022595387
8036324	339324	*zinc finger protein 260*	*ZNF260*	8.04	7.63	10.05	0.007246471	0.022608707
7982254				5.53	5.46	5.84	0.007249547	0.02261586
8118395	8859	*serine/threonine kinase 19*	*STK19*	8.55	8.51	7.73	0.007254988	0.022628253
7904863				7.65	7.67	7.19	0.007255087	0.022628253
8059862				4.69	4.82	4.58	0.007257643	0.022633778
8026398	23581	*caspase 14, apoptosis-related cysteine peptidase*	*CASP14*	5.58	5.65	5.42	0.007260098	0.02263899
7939922				5.55	5.64	5.08	0.007271246	0.022671305
8068593	2114	*v-ets erythroblastosis virus E26 oncogene homolog 2 (avian)*	*ETS2*	9.53	8.45	9.28	0.007274361	0.022673642
8124594	114821	*SCAN domain containing 3*	*SCAND3*	6.17	6.25	6.37	0.007275138	0.022673642
7952388	390265	*olfactory receptor, family 10, subfamily G, member 7*	*OR10G7*	6.98	7.01	6.51	0.007275071	0.022673642
8065905	8200, 554250	*growth differentiation factor 5 | growth differentiation factor 5 opposite strand*	*GDF5|GDF5OS*	6.86	7.37	6.19	0.007272919	0.022673642
8102817	1998	*E74-like factor 2 (ets domain transcription factor)*	*ELF2*	8.06	7.93	8.89	0.007278841	0.022680638
8041886				6.46	6.44	6.17	0.007278954	0.022680638
8064462				4.81	4.86	4.67	0.007280789	0.022683907
8108080	23338	*PHD finger protein 15*	*PHF15*	8.45	8.30	9.27	0.007282589	0.022687068
8043114	83439	*transcription factor 7-like 1 (T-cell specific, HMG-box)*	*TCF7L1*	10.95	9.70	10.86	0.007288891	0.022704251
7933115	285961, 989	*septin 7-like | septin 7*	*SEPT7L*	8.12	7.82	8.72	0.007293692	0.022716397
8176427				7.34	7.34	6.85	0.007294364	0.022716397
7975602	10965	*acyl-CoA thioesterase 2*	*ACOT2*	8.97	9.31	7.81	0.00729518	0.022716487
7924450	11221	*dual specificity phosphatase 10*	*DUSP10*	9.05	9.08	7.50	0.007296735	0.02271888

8088772				6.02	6.03	5.72	0.007299893	0.022726258
8122452	153918	*chromosome 6 open reading frame 94*	*C6orf94*	5.35	5.46	4.92	0.007306339	0.02273897
8095451	401137	*chromosome 4 open reading frame 40*	*C4orf40*	5.39	5.38	5.07	0.007306309	0.02273897
8062842				7.17	7.10	6.47	0.007305218	0.02273897
8028206	25850	*zinc finger protein 345*	*ZNF345*	5.69	5.41	6.81	0.00730876	0.022741603
8045146	80097	*mitotic spindle organizing protein 2B*	*MZT2B*	7.63	7.89	7.39	0.007308724	0.022741603
7970467	221143	*N-6 adenine-specific DNA methyltransferase 2 (putative)*	*N6AMT2*	6.98	7.18	6.73	0.007312072	0.022749456
8125048	23564	*dimethylarginine dimethylaminohydrolase 2*	*DDAH2*	9.27	9.14	8.25	0.007318903	0.02276716
8174279	100507927	*histone H2B type W-T-like*	*LOC100507927*	7.26	7.31	6.86	0.00731934	0.02276716
8107474	1657	*Dmx-like 1*	*DMXL1*	7.78	7.35	9.58	0.007333268	0.022808028
8036622	126432	*Ras and Rab interactor-like*	*RINL*	7.16	7.17	6.86	0.007334748	0.022809906
7960172	677784	*family with sequence similarity 138, member D*	*FAM138D*	5.03	5.01	4.91	0.007335452	0.022809906
8090295	54946	*solute carrier family 41, member 3*	*SLC41A3*	9.20	9.29	8.27	0.007342102	0.022828124
7937975				5.14	5.15	4.98	0.007346165	0.0228383
8007188	1267	*2',3'-cyclic nucleotide 3' phosphodiesterase*	*CNP*	10.60	10.30	9.59	0.007350058	0.022847729
7969794	100132099	*FRSS1829*	*LOC100132099*	5.28	5.06	6.45	0.007350782	0.022847729
7918878	845	*calsequestrin 2 (cardiac muscle)*	*CASQ2*	5.90	5.83	5.48	0.007355484	0.022850046
8063739	116154	*phosphatase and actin regulator 3*	*PHACTR3*	6.59	7.13	6.03	0.007353564	0.022850046
7995344				7.83	7.91	7.36	0.007355307	0.022850046
8021468				7.37	7.46	6.78	0.007354813	0.022850046
8076509				6.88	6.72	6.39	0.007354849	0.022850046
8015152	125115	*keratin 40*	*KRT40*	5.93	5.87	5.67	0.007363192	0.022871528
7948937	9376	*solute carrier family 22 (organic anion transporter), member 8*	*SLC22A8*	6.32	6.43	5.99	0.007371619	0.022895243
7913415	84196	*ubiquitin specific peptidase 48*	*USP48*	7.11	6.79	8.50	0.0073726	0.022895824
8126379	647024	*chromosome 6 open reading frame 132*	*C6orf132*	8.47	8.47	7.91	0.007378935	0.022913033

8007115	100132386, 653240, 100132476, 83755, 728224	*keratin associated protein 4-9 | keratin associated protein 4-11 | keratin associated protein 4-7 | keratin associated protein 4-12 | keratin associated protein 4-8*	*KRTAP4-9|KRTAP4-11|KRTAP4-7|KRTAP4-12|KRTAP4-8*	8.66	8.78	7.94	0.007380895	0.022916656
8010161	10801	*septin 9*	*9-Sep*	11.41	11.18	10.24	0.007382407	0.022918884
7945573	5441	*polymerase (RNA) II (DNA directed) polypeptide L, 7.6kDa*	*POLR2L*	12.72	12.68	11.49	0.007383577	0.022920052
8143327	64761	*poly (ADP-ribose) polymerase family, member 12*	*PARP12*	9.70	9.00	8.79	0.007386025	0.022925185
8016718	1101	*chondroadherin*	*CHAD*	6.68	6.79	6.18	0.007387311	0.022925213
7963631	5916	*retinoic acid receptor, gamma*	*RARG*	10.14	9.50	8.45	0.007387988	0.022925213
8161238	441400		*RAB1C*	10.45	10.50	9.43	0.007388754	0.022925213
7926445	653567, 100289299	*family with sequence similarity 23, member A | family with sequence similarity 23, member B*	*FAM23A|FAM23B*	6.00	6.02	5.92	0.007390798	0.022925213
7966299				9.30	9.51	8.57	0.007390766	0.022925213
8093035				6.66	6.55	6.17	0.007390132	0.022925213
8165258	10555	*1-acylglycerol-3-phosphate O-acyltransferase 2 (lysophosphatidic acid acyltransferase, beta)*	*AGPAT2*	9.96	9.86	8.63	0.007394846	0.022927914
8002891	23563	*carbohydrate (N-acetylglucosamine 6-O) sulfotransferase 5*	*CHST5*	6.97	6.98	6.44	0.007394047	0.022927914
8125316	63943	*FK506 binding protein like*	*FKBPL*	6.95	7.08	6.58	0.007394568	0.022927914
8154731				5.92	5.95	5.48	0.007392569	0.022927914
7938082	1262	*cyclic nucleotide gated channel alpha 4*	*CNGA4*	6.34	6.41	5.94	0.007397668	0.0229342
8003578	653808	*zymogen granule protein 16 homolog (rat)*	*ZG16*	6.33	6.35	5.80	0.007400121	0.022939341
7918951				5.48	5.37	5.13	0.007403906	0.02294861
7944718				6.08	6.01	5.61	0.007411921	0.022970986
8058855				6.96	6.93	7.73	0.007413584	0.022973675
8152867	50807	*ArfGAP with SH3 domain, ankyrin repeat and PH domain 1*	*ASAP1*	10.83	10.50	9.41	0.007419176	0.022988535

8157580				4.77	4.87	4.63	0.00742434	0.023002068
7966150	54434	*slingshot homolog 1 (Drosophila)*	*SSH1*	8.80	8.91	7.65	0.007429034	0.023014143
7993664	9738		*CP110*	6.22	6.00	7.70	0.007432485	0.023021605
7913110	23065		*KIAA0090*	10.06	10.20	9.34	0.007433038	0.023021605
8033159	5623	*persephin*	*PSPN*	7.55	7.70	7.15	0.007434622	0.02302404
8044741				6.46	6.24	5.58	0.007438347	0.023033105
8132840	285877	*POM121 membrane glycoprotein-like 12*	*POM121L 12*	6.61	6.72	6.18	0.007441785	0.023038944
7945394	338440	*anoctamin 9*	*ANO9*	7.02	7.14	7.71	0.007441829	0.023038944
7992863	4992	*olfactory receptor, family 1, subfamily F, member 1*	*OR1F1*	7.36	7.22	6.97	0.00744952	0.023054281
7950606	51773	*remodeling and spacing factor 1*	*RSF1*	7.10	6.84	8.83	0.007449414	0.023054281
7916775				4.90	4.87	5.46	0.007447924	0.023054281
7939118				5.07	5.19	4.93	0.007449977	0.023054281
8037723	26145	*interferon regulatory factor 2 binding protein 1*	*IRF2BP1*	8.80	8.91	7.80	0.007454562	0.023065995
7993274	84127	*RUN domain containing 2A*	*RUNDC2A*	8.91	8.74	7.57	0.007462577	0.023088323
8027805	7392	*upstream transcription factor 2, c-fos interacting*	*USF2*	10.71	10.79	9.91	0.007470686	0.023108615
8107554	340069	*family with sequence similarity 170, member A*	*FAM170A*	6.26	6.32	5.76	0.007470737	0.023108615
7978351	1511	*cathepsin G*	*CTSG*	6.25	6.13	5.69	0.007481532	0.023130799
8024687	27134	*tight junction protein 3 (zona occludens 3)*	*TJP3*	7.39	7.40	8.23	0.007483517	0.023130799
7998457	64718	*unkempt homolog (Drosophila)-like*	*UNKL*	8.42	8.54	7.93	0.007482474	0.023130799
8172280	84679	*solute carrier family 9 (sodium/hydrogen exchanger), member 7*	*SLC9A7*	9.34	9.74	8.45	0.007483158	0.023130799
7981078	256394	*serpin peptidase inhibitor, clade A (alpha-1 antiproteinase, antitrypsin), member 11*	*SERPINA1 1*	6.91	6.98	6.77	0.007480037	0.023130799
7953689	404635	*Nanog homeobox pseudogene 1*	*NANOGP1*	5.38	5.58	6.82	0.007480674	0.023130799
8132034				4.69	4.75	4.54	0.007482243	0.023130799
8014487	31	*acetyl-CoA carboxylase alpha*	*ACACA*	7.96	7.90	9.59	0.007492098	0.023154842
7949995	219927	*mitochondrial ribosomal protein L21*	*MRPL21*	10.19	10.21	11.15	0.007495301	0.02316226
8057887	9262	*serine/threonine kinase 17b*	*STK17B*	10.74	9.04	9.83	0.007497928	0.02316542

8092888	93109	*transmembrane protein 44*	*TMEM44*	8.22	7.97	7.38	0.007497229	0.02316542
7953594	10436		*EMG1*	8.05	8.30	9.29	0.007506775	0.023190271
8048283	6556	*solute carrier family 11 (proton-coupled divalent metal ion transporters), member 1*	*SLC11A1*	6.52	6.43	6.13	0.007509335	0.023190737
7992795	79228, 54985	*THO complex 6 homolog (Drosophila) | host cell factor C1 regulator 1 (XPO1 dependent)*	*THOC6|HCFC1R1*	10.59	10.29	11.50	0.007508626	0.023190737
8016806				6.59	6.60	7.13	0.0075081	0.023190737
8015293	3882	*keratin 32*	*KRT32*	6.70	6.71	6.28	0.007513509	0.02319866
7931656	441056, 653543, 22947, 728410, 653548, 653545, 653544	*double homeobox 4 like 4 | double homeobox 4 like 7 | double homeobox 4 like 2 | double homeobox 4 like 3 | double homeobox 4 like 5 | double homeobox 4 like 6*	*DUX4L4|DUX4L7|DUX4L2|DUX4L3|DUX4L5|DUX4L6*	8.35	8.43	7.75	0.007513387	0.02319866
8010903	9501	*rabphilin 3A-like (without C2 domains)*	*RPH3AL*	7.54	7.74	6.87	0.007515613	0.023202676
8159283	57582	*potassium channel, subfamily T, member 1*	*KCNT1*	7.08	7.17	6.60	0.007519864	0.023212451
8112740	84327	*zinc finger, BED-type containing 3*	*ZBED3*	8.78	8.87	8.38	0.007520387	0.023212451
7931671	441056, 653543, 728410, 653548, 653545, 653544	*double homeobox 4 like 4 | double homeobox 4 like 7 | double homeobox 4 like 2 | double homeobox 4 like 3 | double homeobox 4 like 5 | double homeobox 4 like 6*	*DUX4L4|DUX4L7|DUX4L2|DUX4L3|DUX4L5|DUX4L6*	8.35	8.43	7.75	0.007522665	0.023217
8065084	613266		*LOC613266*	6.48	6.60	6.16	0.007523887	0.023218289
8147837	23414	*zinc finger protein, multitype 2*	*ZFPM2*	8.87	8.73	6.62	0.007525736	0.023221511
7952707	56980	*PR domain containing 10*	*PRDM10*	7.82	7.77	8.25	0.00752851	0.023227591
8058295	57679	*amyotrophic lateral sclerosis 2 (juvenile)*	*ALS2*	6.90	6.78	8.18	0.007530998	0.023232784
8026365	84449	*zinc finger protein 333*	*ZNF333*	7.23	7.22	6.53	0.007533103	0.023236793
8015078	353288	*keratin 26*	*KRT26*	5.18	5.21	5.01	0.007537871	0.023249017
8117330	8350	*histone cluster 1, H3a*	*HIST1H3A*	7.79	8.16	10.63	0.007541058	0.023252537
8009353	26207	*phosphatidylinositol transfer protein, cytoplasmic 1*	*PITPNC1*	9.45	9.20	10.37	0.007541429	0.023252537

7904879				9.36	9.32	8.50	0.007541176	0.023252537
8102619	308	*annexin A5*	*ANXA5*	12.95	13.27	12.59	0.007549421	0.023274692
8076865	400935	*interleukin 17 receptor E-like*	*IL17REL*	7.05	7.22	6.47	0.007551367	0.023278207
7937749	7140	*troponin T type 3 (skeletal, fast)*	*TNNT3*	7.88	7.89	7.38	0.007552327	0.02327868
8101904	131	*alcohol dehydrogenase 7 (class IV), mu or sigma polypeptide*	*ADH7*	5.54	5.60	5.23	0.00755413	0.023279268
7950796	58487	*CREB/ATF bZIP transcription factor*	*CREBZF*	7.49	7.16	8.50	0.007553432	0.023279268
8109436				4.87	4.92	6.51	0.007556913	0.02328536
8034783	22859	*latrophilin 1*	*LPHN1*	9.25	8.67	9.85	0.007563883	0.023304347
7924190	90806	*angel homolog 2 (Drosophila)*	*ANGEL2*	6.82	6.73	8.29	0.007565375	0.023306455
8135915	29923	*chromosome 7 open reading frame 68*	*C7orf68*	8.37	9.05	9.22	0.007566256	0.023306686
8111821	133558	*HEAT repeat family member 7B2*	*HEATR7B 2*	5.23	5.32	5.04	0.007568827	0.023312118
7905490	353144	*late cornified envelope 3C*	*LCE3C*	6.62	6.87	6.24	0.007583447	0.023354653
7899939	2701	*gap junction protein, alpha 4, 37kDa*	*GJA4*	7.58	7.74	7.21	0.00758618	0.023358088
8116932	221692	*phosphatase and actin regulator 1*	*PHACTR1*	8.20	7.06	7.49	0.007586034	0.023358088
8059413	55619	*dedicator of cytokinesis 10*	*DOCK10*	8.03	7.72	5.90	0.007588488	0.023362702
8056821				6.52	6.44	7.07	0.007590097	0.023365166
7931662	441056, 653543	*double homeobox 4 like 4 | double homeobox 4 like 7*	*DUX4L4|D UX4L7*	8.35	8.43	7.75	0.007595164	0.023378268
8037962	56344	*calcium binding protein 5*	*CABP5*	6.29	6.38	6.07	0.007600565	0.023384921
8120249	125050	*RNA, 7SK small nuclear*	*RN7SK*	13.98	13.98	13.84	0.007600376	0.023384921
8172813	29057, 727866	*family with sequence similarity 156, member A | family with sequence similarity 156, member B*	*FAM156A| FAM156B*	8.69	8.73	7.92	0.007598339	0.023384921
7962242				5.22	5.28	4.84	0.007599264	0.023384921
8084982	152217		*LOC15221 7*	7.83	8.01	8.96	0.00760141	0.023385027
8082928	51208	*claudin 18*	*CLDN18*	6.40	6.44	5.93	0.007602283	0.023385221
8006690	26574	*apoptosis antagonizing transcription factor*	*AATF*	9.64	9.80	10.30	0.00760992	0.023405215
8055291	408029	*chromosome 2 open reading frame 27B*	*C2orf27B*	9.07	8.99	8.50	0.007610404	0.023405215
8024446	56928	*signal peptide peptidase-like 2B*	*SPPL2B*	9.98	9.90	8.75	0.007613222	0.023408893

7907655				7.82	7.27	6.84	0.007612677	0.023408893
8092390				7.22	7.18	6.76	0.007616618	0.023416843
8023984	610	*hyperpolarization activated cyclic nucleotide-gated potassium channel 2*	*HCN2*	8.20	8.11	7.19	0.00761753	0.023417152
8053288	6936	*chromosome 2 open reading frame 3*	*C2orf3*	7.60	7.23	8.45	0.007620507	0.02342381
8151906	392255	*growth differentiation factor 6*	*GDF6*	6.79	8.33	6.37	0.007626022	0.023438266
8020211	1149	*cell death-inducing DFFA-like effector a*	*CIDEA*	6.57	6.68	6.17	0.007629899	0.023447687
7941608				6.63	6.80	6.49	0.007632112	0.023451988
8152291	137735	*actin-binding Rho activating protein*	*ABRA*	6.51	6.59	6.17	0.007640119	0.023474094
8179440	1589, 1590	*cytochrome P450, family 21, subfamily A, polypeptide 2 | cytochrome P450, family 21, subfamily A, polypeptide 1 pseudogene*	*CYP21A2|CYP21A1P*	6.60	6.54	6.00	0.007641631	0.023476243
8122773	25902	*methylenetetrahydrofolate dehydrogenase (NADP+ dependent) 1-like*	*MTHFD1L*	9.95	10.39	9.83	0.007643845	0.023480544
8161615	5568	*protein kinase, cAMP-dependent, catalytic, gamma*	*PRKACG*	6.71	6.76	6.26	0.007647496	0.023489261
7931659	441056, 653543, 22947, 728410, 653548, 653545, 653544	*double homeobox 4 like 4 | double homeobox 4 like 7 | double homeobox 4 like 2 | double homeobox 4 like 3 | double homeobox 4 like 5 | double homeobox 4 like 6*	*DUX4L4|DUX4L7|DUX4L2|DUX4L3|DUX4L5|DUX4L6*	8.35	8.43	7.75	0.007659936	0.023524966
8030416	58506	*SR-related CTD-associated factor 1*	*SCAF1*	8.99	9.05	8.08	0.00766876	0.023549562
8002133	5699	*proteasome (prosome, macropain) subunit, beta type, 10*	*PSMB10*	8.30	8.00	7.29	0.007673679	0.023562163
8159211	2220	*ficolin (collagen/fibrinogen domain containing lectin) 2 (hucolin)*	*FCN2*	7.24	7.37	6.59	0.00768311	0.023585772
7956261	5939	*RNA binding motif, single stranded interacting protein 2*	*RBMS2*	11.56	11.39	10.08	0.007684178	0.023585772
8171684	30011	*SH3-domain kinase binding protein 1*	*SH3KBP1*	10.27	10.34	9.36	0.007684636	0.023585772
8003806	8386, 653166	*olfactory receptor, family 1, subfamily D, member 5 | olfactory receptor, family 1, subfamily D, member 4 (gene/pseudogene)*	*OR1D5|OR1D4*	6.89	6.64	6.46	0.00768356	0.023585772
8036373	199704	*zinc finger protein 585A*	*ZNF585A*	6.89	6.72	7.87	0.007687906	0.023587229

8107119	345757	*family with sequence similarity 174, member A*	*FAM174A*	6.77	7.11	6.42	0.00768816	0.023587229
8043687	375248	*ankyrin repeat domain 36*	*ANKRD36*	6.73	6.12	7.23	0.007687688	0.023587229
8134552	10095 10552	*actin related protein 2/3 complex, subunit 1B, 41kDa | actin related protein 2/3 complex, subunit 1A, 41kDa*	*ARPC1B|ARPC1A*	12.32	11.98	11.37	0.007688379	0.023587229
7973724	57523	*NYN domain and retroviral integrase containing*	*NYNRIN*	10.03	8.62	7.72	0.007693055	0.023599064
7928471	132	*adenosine kinase*	*ADK*	9.29	8.73	9.51	0.007700825	0.023615373
8039593	63934	*zinc finger protein 667*	*ZNF667*	6.50	6.49	7.95	0.007700462	0.023615373
7911281	401992, 403244	*olfactory receptor, family 2, subfamily T, member 2 | olfactory receptor, family 2, subfamily T, member 35*	*OR2T2|OR2T35*	6.04	6.15	5.83	0.007700443	0.023615373
7999435	5620	*protamine 2*	*PRM2*	6.67	6.80	6.36	0.00770459	0.023616882
8011214	146760	*reticulon 4 receptor-like 1*	*RTN4RL1*	6.89	7.40	6.43	0.007703492	0.023616882
8060126	375318, 653437	*aquaporin 12A | aquaporin 12B*	*AQP12A|AQP12B*	7.86	7.93	7.44	0.007704208	0.023616882
7908610				4.96	4.78	5.84	0.007702281	0.023616882
7960365	84766	*EF-hand calcium binding domain 4B*	*EFCAB4B*	7.00	7.03	5.69	0.007705718	0.023617832
8055021	55677		*IWS1*	7.03	7.15	8.84	0.00770853	0.023622938
8095139	79644	*steroid 5 alpha-reductase 3*	*SRD5A3*	9.07	9.42	9.69	0.007709838	0.023622938
7920193	353133	*late cornified envelope 1C*	*LCE1C*	7.47	7.45	6.97	0.007709496	0.023622938
8019709	6066, 26855, 54663	*RNA, U2 small nuclear 1 | RNA, U2 small nuclear 2 | WD repeat domain 74*	*RNU2-1|RNU2-2|WDR74*	13.52	13.52	13.87	0.007719596	0.023650326
8045810				4.55	4.62	4.44	0.007724507	0.023662861
8130803	401286		*LOC401286*	5.97	5.95	5.65	0.007734469	0.023690522
8155630	25902, 100133920, 286297, 100506095	*methylenetetrahydrofolate dehydrogenase (NADP+ dependent) 1-like | hypothetical LOC100133920 | hypothetical LOC286297 | monofunctional C1-tetrahydrofolate synthase, mitochondrial-like*	*MTHFD1L|LOC100133920|LOC286297|LOC100506095*	7.99	8.24	7.80	0.007735178	0.023690522
8021442	55205	*zinc finger protein 532*	*ZNF532*	10.04	9.53	11.30	0.007736994	0.023693567
8169044	85012	*transcription elongation factor A (SII)-like 3*	*TCEAL3*	8.62	8.73	8.08	0.007744334	0.023713531

8100312	339977	*leucine rich repeat containing 66*	*LRRC66*	5.11	5.00	4.83	0.00774765	0.02372117
7958051	429	*achaete-scute complex homolog 1 (Drosophila)*	*ASCL1*	6.81	6.96	6.23	0.007750291	0.02372674
7905974	57657	*hyperpolarization activated cyclic nucleotide-gated potassium channel 3*	*HCN3*	7.87	8.05	7.25	0.007754061	0.023735765
7934789	2894	*glutamate receptor, ionotropic, delta 1*	*GRID1*	7.93	7.91	6.78	0.007755219	0.023736792
8138007				8.82	8.89	8.40	0.007768327	0.023774393
8074880	9609		*RAB36*	6.67	6.68	6.20	0.007769852	0.023776537
7898167	11330	*chymotrypsin C (caldecrin)*	*CTRC*	6.44	6.63	5.91	0.007773458	0.02378505
7995776	4504	*metallothionein 3*	*MT3*	8.31	8.37	7.63	0.007777139	0.02379379
8135229	83787	*armadillo repeat containing 10*	*ARMC10*	7.86	7.92	8.49	0.007780039	0.023800144
8084812				5.26	5.27	5.10	0.007782839	0.023806186
8170412	4107	*melanoma antigen family A, 8*	*MAGEA8*	8.34	8.45	7.82	0.007786567	0.023815066
8057677	30061	*solute carrier family 40 (iron-regulated transporter), member 1*	*SLC40A1*	7.78	6.43	5.92	0.00779095	0.023825945
8019842	7298	*thymidylate synthetase*	*TYMS*	8.96	9.28	10.79	0.007794645	0.02383472
8135931	402483	*hypothetical LOC402483*	*FLJ45340*	7.78	7.70	7.09	0.00779587	0.023835942
7910265	84033	*obscurin, cytoskeletal calmodulin and titin-interacting RhoGEF*	*OBSCN*	6.98	7.03	6.58	0.00779799	0.023839898
8029375				6.70	6.67	6.15	0.007799897	0.023843206
7989735	10845	*ClpX caseinolytic peptidase X homolog (E. coli)*	*CLPX*	7.76	7.55	9.28	0.007802301	0.023848029
8107328	54508		*FLJ11235*	7.62	7.75	7.24	0.007806223	0.023857491
7944603	2900	*glutamate receptor, ionotropic, kainate 4*	*GRIK4*	6.65	6.76	7.30	0.007811875	0.02386971
8178244	6015	*ring finger protein 1*	*RING1*	9.35	9.31	8.15	0.007811386	0.02386971
7971644	57213	*chromosome 13 open reading frame 1*	*C13orf1*	8.18	8.58	8.24	0.007813584	0.023872163
7955981	441639	*olfactory receptor, family 9, subfamily K, member 2*	*OR9K2*	5.20	5.23	4.96	0.007814332	0.023872163
8044541	56300	*interleukin 1 family, member 9*	*IL1F9*	5.88	6.10	5.61	0.007816366	0.023875851
8097541				5.99	5.95	5.59	0.007821986	0.02389049
8046238	140469	*myosin IIIB*	*MYO3B*	5.47	5.41	5.33	0.007825017	0.023895571

8161388	441425, 441430, 84210, 728747	*ankyrin repeat domain 20 family, member A3 | ankyrin repeat domain 20 family, member A2 | ankyrin repeat domain 20 family, member A1 | ankyrin repeat domain 20 family, member A4*	*ANKRD20A3|ANKRD20A2|ANKRD20A1|ANKRD20A4*	5.48	5.45	5.35	0.007825305	0.023895571
8158242	81605	*ubiquitin related modifier 1*	*URM1*	10.05	9.76	8.91	0.007829495	0.023905836
8027856	2865	*free fatty acid receptor 3*	*FFAR3*	6.10	6.01	5.69	0.007830724	0.023907062
8177114				7.34	7.35	6.84	0.007832847	0.023911012
7975987				4.85	5.10	5.37	0.007833982	0.02391195
8118634	6015	*ring finger protein 1*	*RING1*	9.30	9.25	8.10	0.007836269	0.023914048
7992905	79903	*N-acetyltransferase 15 (GCN5-related, putative)*	*NAT15*	10.23	10.42	9.24	0.007836326	0.023914048
7903719	271	*adenosine monophosphate deaminase 2*	*AMPD2*	9.58	9.53	8.32	0.007838061	0.023916814
7931668	441056, 653543, 22947, 728410, 653548, 653545, 653544	*double homeobox 4 like 4 | double homeobox 4 like 7 | double homeobox 4 like 2 | double homeobox 4 like 3 | double homeobox 4 like 5 | double homeobox 4 like 6*	*DUX4L4|DUX4L7|DUX4L2|DUX4L3|DUX4L5|DUX4L6*	8.35	8.43	7.75	0.007839834	0.023919698
8128007	375519	*gap junction protein, beta 7, 25kDa*	*GJB7*	5.12	5.12	5.45	0.007846369	0.023937104
7948667	79026		*AHNAK*	11.08	10.99	8.65	0.007850604	0.023947494
8103092	79658, 100129572	*Rho GTPase activating protein 10 | hypothetical protein LOC100129572*	*ARHGAP10|LOC100129572*	6.43	6.48	6.03	0.007852322	0.023950206
7971800	374500, 644623	*thrombospondin, type I, domain containing 1 pseudogene 1 | transmembrane phosphoinositide 3-phosphatase and tensin homolog 2 pseudogene 2*	*THSD1P1|TPTE2P2*	6.79	6.91	7.74	0.007854545	0.023954453
8079966	7869	*sema domain, immunoglobulin domain (Ig), short basic domain, secreted, (semaphorin) 3B*	*SEMA3B*	8.75	7.39	6.37	0.007855857	0.023955924
7938261	26343	*olfactory receptor, family 5, subfamily E, member 1 pseudogene*	*OR5E1P*	6.18	6.15	5.83	0.007859559	0.023964252
8004889	146845	*WD repeat domain 16*	*WDR16*	5.74	5.75	5.40	0.007860248	0.023964252
8085946	8320	*eomesodermin*	*EOMES*	7.27	7.31	7.02	0.007861377	0.023965161

8175621	84548	*transmembrane protein 185A*	*TMEM185 A*	9.32	8.91	8.32	0.00786635	0.023975259
7985030	100128285	*DNM1 pseudogene 35*	*DNM1P35*	7.81	7.98	7.44	0.007865884	0.023975259
7952549	84881	*RNA pseudouridylate synthase domain containing 4*	*RPUSD4*	7.94	7.69	8.37	0.00787193	0.023989731
8043552	30818	*Kv channel interacting protein 3, calsenilin*	*KCNIP3*	9.55	9.78	8.02	0.007878831	0.024004765
7948150	390168	*olfactory receptor, family 5, subfamily M, member 1*	*OR5M1*	4.91	4.90	4.69	0.007877773	0.024004765
8151743				5.51	5.66	5.29	0.007879357	0.024004765
8082250				6.37	6.28	5.76	0.007880857	0.024006803
8114612	929		*CD14*	7.11	6.94	5.97	0.007882237	0.024008472
8109821	6134	*ribosomal protein L10*	*RPL10*	14.09	14.05	14.01	0.007887601	0.024022276
8090891	2047	*EPH receptor B1*	*EPHB1*	8.13	8.23	7.64	0.007890921	0.024029853
8004057	10749	*kinesin family member 1C*	*KIF1C*	10.35	10.33	9.03	0.007893303	0.024032034
8113573	83942	*testis-specific serine kinase 1B*	*TSSK1B*	6.50	6.40	6.01	0.007892543	0.024032034
7909350	1380	*complement component (3d/Epstein Barr virus) receptor 2*	*CR2*	5.06	5.09	5.91	0.007897547	0.024033263
8132130	2692	*growth hormone releasing hormone receptor*	*GHRHR*	6.89	7.04	6.54	0.007897869	0.024033263
7943176	9440	*mediator complex subunit 17*	*MED17*	7.48	7.63	8.61	0.007896966	0.024033263
7973727	57523	*NYN domain and retroviral integrase containing*	*NYNRIN*	10.84	9.06	8.60	0.007897837	0.024033263
8130626				5.63	5.66	5.38	0.007897378	0.024033263
7948741	26229	*beta-1,3-glucuronyltransferase 3 (glucuronosyltransferase I)*	*B3GAT3*	9.22	9.34	7.90	0.007902869	0.024045944
8105323	8988	*heat shock 27kDa protein 3*	*HSPB3*	6.09	6.44	5.34	0.007907786	0.024058372
7901418	91408	*basic transcription factor 3-like 4*	*BTF3L4*	10.33	10.63	11.84	0.007910947	0.024065452
8044574	3557	*interleukin 1 receptor antagonist*	*IL1RN*	5.86	5.64	5.41	0.007914458	0.024068283
8025000	400673	*vimentin-type intermediate filament associated coiled-coil protein*	*VMAC*	7.80	7.74	7.00	0.007915213	0.024068283
8016615	201191, 5164	*sterile alpha motif domain containing 14 | pyruvate dehydrogenase kinase, isozyme 2*	*SAMD14|PDK2*	8.15	7.95	7.23	0.007914517	0.024068283
8020666				6.09	6.00	5.58	0.007914931	0.024068283
7945728				5.11	5.28	4.96	0.007923235	0.02409014
7985352	161502	*chromosome 15 open reading frame 26*	*C15orf26*	5.53	5.37	5.21	0.00792776	0.024101358

8100615	55236	*ubiquitin-like modifier activating enzyme 6*	*UBA6*	8.64	8.32	9.79	0.007929861	0.024105206
8067680	85441	*peroxisomal proliferator-activated receptor A interacting complex 285*	*PRIC285*	8.30	8.35	7.60	0.007931321	0.024107108
7970727	1045	*caudal type homeobox 2*	*CDX2*	7.63	7.67	7.18	0.007933023	0.024109744
8163402	9991		*ROD1*	8.73	8.77	10.46	0.007934296	0.024111072
7898594	3362	*5-hydroxytryptamine (serotonin) receptor 6*	*HTR6*	7.03	7.06	6.68	0.007935583	0.024112444
8054939				7.56	7.61	7.00	0.007942135	0.024129815
7909601	692157	*small nucleolar RNA, H/ACA box 16B*	*SNORA16B*	5.08	4.81	5.30	0.007948856	0.024147693
8093858	55351	*serine/threonine kinase 32B*	*STK32B*	8.84	8.34	6.78	0.007949732	0.024147814
8095680	3576	*interleukin 8*	*IL8*	6.68	7.31	6.51	0.007952136	0.024148265
8146462	64321	*SRY (sex determining region Y)-box 17*	*SOX17*	7.92	8.13	7.41	0.007952753	0.024148265
7987110				5.53	5.45	5.82	0.007950894	0.024148265
8053840				6.80	6.81	6.30	0.007953227	0.024148265
7946082	143630	*ubiquilin-like*	*UBQLNL*	6.34	6.21	5.82	0.007956152	0.024154605
7986687	339005, 440253	*WAS protein homolog associated with actin, golgi membranes and microtubules-like 1 (pseudogene) | WAS protein homolog associated with actin, golgi membranes and microtubules-like 2 (pseudogene)*	*WHAMML1|WHAMML2*	7.56	7.40	6.16	0.007979563	0.024223136
7949080	9379	*neurexin 2*	*NRXN2*	8.11	8.42	7.52	0.007986852	0.02424252
8048350	84812	*phospholipase C, delta 4*	*PLCD4*	6.69	6.51	5.68	0.007988469	0.02424252
8161377	25902, 100133920, 286297, 100506095	*methylenetetrahydrofolate dehydrogenase (NADP+ dependent) 1-like | hypothetical LOC100133920 | hypothetical LOC286297 | monofunctional C1-tetrahydrofolate synthase, mitochondrial-like*	*MTHFD1L|LOC100133920|LOC286297|LOC100506095*	7.99	8.24	7.80	0.007987931	0.02424252
8078984	9045	*ribosomal protein L14*	*RPL14*	9.03	9.16	9.60	0.007993752	0.024255967
7986975				5.33	5.42	5.04	0.00799458	0.024255967
8123876	221718	*chromosome 6 open reading frame 218*	*C6orf218*	5.61	5.64	5.36	0.007995761	0.024257
8058415	54891	*INO80 complex subunit D*	*INO80D*	8.48	8.15	10.41	0.007997203	0.024258826
8143144	5764	*pleiotrophin*	*PTN*	8.80	6.77	7.88	0.008005071	0.02428014

7990151	5315, 652797	*pyruvate kinase, muscle | pyruvate kinase isozymes M1/M2-like*	*PKM2|LOC652797*	12.79	12.97	12.36	0.00800622	0.024281075
8128894	5980	*REV3-like, catalytic subunit of DNA polymerase zeta (yeast)*	*REV3L*	9.94	8.53	7.67	0.008007869	0.024283526
7949843	51475	*calcium binding protein 2*	*CABP2*	8.37	8.55	7.89	0.00801014	0.02428786
7923483	5877	*RAB interacting factor*	*RABIF*	8.84	9.19	8.68	0.008012683	0.024293022
7939920	81330	*olfactory receptor, family 4, subfamily A, member 13 pseudogene*	*OR4A13P*	5.08	5.19	4.80	0.008014754	0.02429675
8146000	8754	*ADAM metallopeptidase domain 9*	*ADAM9*	11.46	10.53	9.29	0.008018762	0.024306346
8073088	60489	*apolipoprotein B mRNA editing enzyme, catalytic polypeptide-like 3G*	*APOBEC3G*	7.70	6.80	6.17	0.00802078	0.024307359
7967089				6.61	6.50	5.93	0.00802051	0.024307359
7903355	54112	*G protein-coupled receptor 88*	*GPR88*	8.04	8.03	7.43	0.00802969	0.024330495
8152759	83940	*TatD DNase domain containing 1*	*TATDN1*	6.78	6.79	7.50	0.008030317	0.024330495
8022572	114876	*oxysterol binding protein-like 1A*	*OSBPL1A*	8.59	7.55	9.05	0.008030943	0.024330495
8030749	284365	*hypothetical LOC284365*	*MGC45922*	6.84	7.01	6.41	0.008032723	0.024333335
8032197	255057	*chromosome 19 open reading frame 26*	*C19orf26*	8.90	9.06	8.29	0.008033977	0.02433458
7922200	6403	*selectin P (granule membrane protein 140kDa, antigen CD62)*	*SELP*	5.78	5.89	5.54	0.008036932	0.024340976
8051707	8491	*mitogen-activated protein kinase kinase kinase kinase 3*	*MAP4K3*	8.95	8.38	9.80	0.008037926	0.024341432
7943413	330	*baculoviral IAP repeat-containing 3*	*BIRC3*	5.15	5.07	4.93	0.008038896	0.024341818
8173869	79983	*premature ovarian failure, 1B*	*POF1B*	5.05	5.04	4.94	0.008042111	0.024348998
7969881	7174	*tripeptidyl peptidase II*	*TPP2*	8.71	8.47	10.58	0.00804648	0.024359671
8081631	344805	*transmembrane protease, serine 7*	*TMPRSS7*	5.86	5.85	5.58	0.00804841	0.024362959
8159667	54863	*chromosome 9 open reading frame 167*	*C9orf167*	8.61	8.17	7.57	0.008059298	0.02439336
7977732	319103	*small nucleolar RNA, C/D box 8*	*SNORD8*	6.64	6.63	7.13	0.008060453	0.0243943
8071259	5413, 2812	*septin 5 | glycoprotein Ib (platelet), beta polypeptide*	*SEPT5|GP1BB*	8.40	8.32	7.61	0.008069759	0.024419904
8017711	10672	*guanine nucleotide binding protein (G protein), alpha 13*	*GNA13*	9.08	9.16	10.37	0.008072173	0.024424648
7917792				5.15	5.06	4.79	0.008074508	0.024429152

8157686	254973	*olfactory receptor, family 1, subfamily L, member 4*	*OR1L4*	5.47	5.45	5.20	0.008075989	0.024431072
8012837	644139	*phosphoinositide-interacting regulator of transient receptor potential channels*	*PIRT*	6.90	7.10	6.53	0.008077994	0.024434578
8174670	57481		*KIAA1210*	5.67	5.62	5.40	0.008085704	0.024455339
8174005	79979	*TRM2 tRNA methyltransferase 2 homolog B (S. cerevisiae)*	*TRMT2B*	8.04	8.15	8.71	0.008088879	0.024460122
7998444	123787	*protease, serine, 29 pseudogene*	*PRSS29P*	8.62	8.70	8.21	0.008088981	0.024460122
8077572	151835	*copine family member IX*	*CPNE9*	6.25	6.33	6.61	0.008090065	0.024460837
8017365	162333	*membrane-associated ring finger (C3HC4) 10*	*10-Mar*	5.69	5.80	5.50	0.00809311	0.024463486
8088911	100131827, 100287163	*zinc finger protein 717 | zinc finger protein 717-like*	*ZNF717|LOC100287163*	9.27	8.98	10.39	0.008092294	0.024463486
8051666				5.67	5.39	5.20	0.008093483	0.024463486
7958819	10961	*endoplasmic reticulum protein 29*	*ERP29*	10.68	10.23	11.05	0.00809698	0.024471493
8023990	27006	*fibroblast growth factor 22*	*FGF22*	8.11	8.12	7.52	0.00809884	0.02447199
8009913	80022	*myosin XVB pseudogene*	*MYO15B*	7.30	6.86	6.56	0.008098275	0.02447199
8075865	164656	*transmembrane protease, serine 6*	*TMPRSS6*	6.87	6.91	6.48	0.008099969	0.02447284
8146429	2831	*neuropeptides B/W receptor 1*	*NPBWR1*	6.37	6.41	5.89	0.008103189	0.02448001
7967212	3242	*4-hydroxyphenylpyruvate dioxygenase*	*HPD*	6.87	6.34	6.44	0.008107073	0.02448918
8151217				4.42	4.45	4.37	0.00810844	0.024490746
8007641	728675	*UPF0443 protein C11orf75-like*	*LOC728675*	9.37	9.38	8.98	0.008111927	0.024498714
8007429	2538	*glucose-6-phosphatase, catalytic subunit*	*G6PC*	5.70	5.66	5.35	0.008112824	0.02449886
8002262				8.51	8.59	7.65	0.008114688	0.024501927
7986744	283685	*golgin A6 family-like 2*	*GOLGA6L2*	6.53	6.52	6.21	0.008122362	0.024522534
8093053	7037	*transferrin receptor (p90, CD71)*	*TFRC*	8.70	8.81	10.75	0.008127091	0.024534244
8160857	10280	*sigma non-opioid intracellular receptor 1*	*SIGMAR1*	11.15	11.12	9.98	0.008129245	0.024538182
7899990	23154	*neurochondrin*	*NCDN*	10.36	10.41	9.39	0.008132107	0.024540184
8076441	150372	*NFAT activating protein with ITAM motif 1*	*NFAM1*	8.00	8.11	7.50	0.008132718	0.024540184
7955985	254783	*olfactory receptor, family 6, subfamily C, member 74*	*OR6C74*	5.45	5.22	5.17	0.008132178	0.024540184

7915468	374969, 114625	*coiled-coil domain containing 23 | erythroblast membrane-associated protein (Scianna blood group)*	*CCDC23|ERMAP*	7.84	7.67	8.72	0.008134158	0.024540184
7959818				5.76	5.86	5.47	0.00813401	0.024540184
8082046	846	*calcium-sensing receptor*	*CASR*	6.30	6.37	6.01	0.008139439	0.024553405
7980477				6.15	6.14	6.86	0.008140658	0.024553405
8022007				6.46	6.42	5.96	0.008141092	0.024553405
8045224	205147	*family with sequence similarity 123C*	*FAM123C*	7.09	6.95	6.69	0.008144585	0.024561372
8069561	91408	*basic transcription factor 3-like 4*	*BTF3L4*	9.84	10.13	11.31	0.00814657	0.024563564
8007134	85280, 83899, 83901, 83900, 81870	*keratin associated protein 9-4 | keratin associated protein 9-2 | keratin associated protein 9-8 | keratin associated protein 9-3 | keratin associated protein 9-9*	*KRTAP9-4|KRTAP9-2|KRTAP9-8|KRTAP9-3|KRTAP9-9*	7.07	7.18	6.87	0.008147013	0.024563564
7899905	127540	*high-mobility group box 4*	*HMGB4*	5.94	6.01	5.63	0.008148297	0.024564868
8059067	3549	*Indian hedgehog*	*IHH*	7.33	7.32	6.77	0.008150936	0.02457026
7910591	84284	*chromosome 1 open reading frame 57*	*C1orf57*	9.81	9.15	10.18	0.008155362	0.024581034
7899600				6.52	6.69	6.04	0.008156288	0.024581257
8080445	3697	*inter-alpha (globulin) inhibitor H1*	*ITIH1*	6.26	6.33	5.89	0.008157724	0.02458302
8158446	23511	*nucleoporin 188kDa*	*NUP188*	9.22	9.61	10.27	0.008160016	0.02458736
8031223	10859	*leukocyte immunoglobulin-like receptor, subfamily B (with TM and ITIM domains), member 1*	*LILRB1*	6.73	6.70	6.06	0.008171406	0.024615398
8106976	285601	*G protein-coupled receptor 150*	*GPR150*	8.90	8.97	8.21	0.008171879	0.024615398
8141659				7.54	7.49	6.97	0.008170399	0.024615398
8160879	6363	*chemokine (C-C motif) ligand 19*	*CCL19*	6.82	6.92	6.33	0.008174057	0.02461939
8099541	5860	*quinoid dihydropteridine reductase*	*QDPR*	7.89	7.82	8.57	0.008179972	0.024629496
8099760	116984	*ArfGAP with RhoGAP domain, ankyrin repeat and PH domain 2*	*ARAP2*	4.95	5.00	5.32	0.008179629	0.024629496
7953733	440077	*zinc finger protein 705A*	*ZNF705A*	4.59	4.80	4.59	0.008178591	0.024629496

8005829				6.40	5.61	7.84	0.008180829	0.024629507
8140814	55972	*solute carrier family 25, member 40*	*SLC25A40*	8.72	8.72	9.70	0.00818723	0.024646208
8005749				6.58	6.52	6.03	0.008190902	0.024652189
8075668				6.82	7.02	6.34	0.008190924	0.024652189
8131438				8.65	8.76	7.86	0.008198772	0.024673235
8154124	169522	*potassium channel, subfamily V, member 2*	*KCNV2*	6.09	6.03	5.67	0.008203595	0.024685176
7924874	51127	*tripartite motif-containing 17*	*TRIM17*	7.46	7.39	7.04	0.00820634	0.02469086
8165866	412	*steroid sulfatase (microsomal), isozyme S*	*STS*	8.58	9.09	6.73	0.008208985	0.024694964
8003926	84225	*zinc finger, MYND-type containing 15*	*ZMYND15*	6.86	6.87	6.29	0.008209414	0.024694964
8064539	56265	*carboxypeptidase X (M14 family), member 1*	*CPXM1*	7.91	7.44	8.29	0.008217533	0.024716813
8001041	1339	*cytochrome c oxidase subunit VIa polypeptide 2*	*COX6A2*	8.25	8.30	7.62	0.008219645	0.02472059
7949577	10897	*Yip1 interacting factor homolog A (S. cerevisiae)*	*YIF1A*	10.67	10.95	9.55	0.008221027	0.02472217
8102004				6.14	6.25	5.85	0.008222547	0.024724167
7934477	818	*calcium/calmodulin-dependent protein kinase II gamma*	*CAMK2G*	10.40	10.01	9.43	0.008233011	0.024743756
8106689	1160	*creatine kinase, mitochondrial 2 (sarcomeric)*	*CKMT2*	6.32	6.30	6.94	0.008232142	0.024743756
7991186	4916	*neurotrophic tyrosine kinase, receptor, type 3*	*NTRK3*	6.25	6.50	6.99	0.008230163	0.024743756
8090817	80111	*chromosome 3 open reading frame 36*	*C3orf36*	6.14	6.37	5.83	0.008233348	0.024743756
8095802				4.71	4.77	5.24	0.008233093	0.024743756
7949619	3177	*solute carrier family 29 (nucleoside transporters), member 2*	*SLC29A2*	7.42	7.53	8.23	0.008245614	0.024775462
7906041	7818	*death associated protein 3*	*DAP3*	8.13	8.22	8.98	0.008245362	0.024775462
8049239	389084	*chromosome 2 open reading frame 82*	*C2orf82*	8.34	8.44	7.80	0.008249056	0.024783222
7926565	8028	*myeloid/lymphoid or mixed-lineage leukemia (trithorax homolog, Drosophila); translocated to, 10*	*MLLT10*	7.96	7.98	9.73	0.008251356	0.024784116
7897675	10218	*angiopoietin-like 7*	*ANGPTL7*	5.75	5.89	5.56	0.008250963	0.024784116
8049567	10267	*receptor (G protein-coupled) activity modifying protein 1*	*RAMP1*	8.85	8.77	7.87	0.008251929	0.024784116
7965673				7.43	7.45	6.96	0.008253453	0.024786117
7975687	91750	*lin-52 homolog (C. elegans)*	*LIN52*	7.64	7.79	9.19	0.008254936	0.02478799

8044548	27179	*interleukin 1 family, member 6 (epsilon)*	*IL1F6*	6.91	6.89	6.39	0.008258572	0.024791174
8159275	392399	*lipocalin 9*	*LCN9*	5.94	5.97	5.45	0.008258236	0.024791174
8097013				5.92	5.96	5.46	0.008258238	0.024791174
8027864	26330	*glyceraldehyde-3-phosphate dehydrogenase, spermatogenic*	*GAPDHS*	6.29	6.52	5.94	0.008260309	0.02479381
7997321	22879, 100130958	*MON1 homolog B (yeast) | synaptonemal complex central element protein 1-like*	*MON1B|SYCE1L*	8.74	8.64	8.20	0.008261409	0.024794532
8154449	54875	*centlein, centrosomal protein*	*CNTLN*	7.52	6.94	8.18	0.008263155	0.024797196
8037525	1158	*creatine kinase, muscle*	*CKM*	6.72	6.73	6.10	0.00826683	0.024805645
8030212	64130	*lin-7 homolog B (C. elegans)*	*LIN7B*	7.66	7.75	7.11	0.008270151	0.024813032
8165398	2529, 401563	*fucosyltransferase 7 (alpha (1,3) fucosyltransferase) | chromosome 9 open reading frame 139*	*FUT7|C9orf139*	6.57	6.48	6.28	0.008271303	0.02481391
8018620	23265	*exocyst complex component 7*	*EXOC7*	10.87	10.91	9.79	0.008273953	0.0248167
7900146	80149	*zinc finger CCCH-type containing 12A*	*ZC3H12A*	8.20	8.44	7.70	0.008273108	0.0248167
8020630	125488	*tetratricopeptide repeat domain 39C*	*TTC39C*	10.50	9.05	9.83	0.008279552	0.024829438
8074958				5.77	5.91	5.40	0.00827992	0.024829438
7994535	27040, 83985	*linker for activation of T cells | spinster homolog 1 (Drosophila)*	*LAT|SPNS1*	9.35	9.46	8.46	0.008284077	0.024839325
8142618	168433	*ring finger protein 133*	*RNF133*	4.70	4.66	5.05	0.008288194	0.024849087
8112918				8.57	8.34	8.89	0.008289685	0.024850976
8006681	3975	*LIM homeobox 1*	*LHX1*	7.43	7.47	6.98	0.008292764	0.024857627
7943958	9177	*5-hydroxytryptamine (serotonin) receptor 3B*	*HTR3B*	5.24	5.36	5.06	0.008299651	0.024864605
7940160	23220	*deltex homolog 4 (Drosophila)*	*DTX4*	8.73	7.82	8.60	0.008297209	0.024864605
7969129	51131	*PHD finger protein 11*	*PHF11*	8.08	7.90	7.15	0.008298207	0.024864605
7952350	55800	*sodium channel, voltage-gated, type III, beta*	*SCN3B*	6.62	6.84	6.33	0.008298658	0.024864605
8059361	57590	*WD repeat and FYVE domain containing 1*	*WDFY1*	9.43	9.20	10.77	0.00830026	0.024864605
7973458	317749, 10901, 728635	*dehydrogenase/reductase (SDR family) member 4 like 2 | dehydrogenase/reductase (SDR family) member 4 like 1*	*DHRS4L2|DHRS4L1*	9.14	8.83	8.05	0.008298535	0.024864605
8007493	379	*ADP-ribosylation factor-like 4D*	*ARL4D*	9.85	9.61	8.02	0.008307302	0.024878688
7896921	83756	*taste receptor, type 1, member 3*	*TAS1R3*	7.73	7.77	7.02	0.008307546	0.024878688

8084941				6.40	6.34	5.66	0.008306895	0.024878688
8084844	4976	*optic atrophy 1 (autosomal dominant)*	*OPA1*	8.35	8.01	10.16	0.008310359	0.02488453
7904921				5.34	5.34	5.22	0.008311522	0.024885433
8094596	10744	*pituitary tumor-transforming 2*	*PTTG2*	4.47	4.47	4.66	0.008315129	0.024893649
8100851				7.01	6.89	6.49	0.008317419	0.024897924
7948920	9356	*solute carrier family 22 (organic anion transporter), member 6*	*SLC22A6*	7.29	7.27	6.92	0.008318788	0.02489944
8010499	284131		*FLJ35220*	7.81	7.80	7.07	0.008322307	0.02490739
8160559	23586	*DEAD (Asp-Glu-Ala-Asp) box polypeptide 58*	*DDX58*	7.14	6.85	8.46	0.008332009	0.024932269
8076819	400932	*hypothetical LOC400932*	*FLJ46257*	6.45	6.45	6.10	0.008332348	0.024932269
8043105	56888	*potassium channel modulatory factor 1*	*KCMF1*	10.76	10.79	11.53	0.00833821	0.024947226
8178442	1041	*corneodesmosin*	*CDSN*	7.29	7.30	6.83	0.008343047	0.024951356
8130528	83861	*radial spoke 3 homolog (Chlamydomonas)*	*RSPH3*	7.92	8.29	6.99	0.008342586	0.024951356
8014259	342618	*schlafen family member 14*	*SLFN14*	5.50	5.42	5.12	0.008341162	0.024951356
7914628				6.51	6.39	7.08	0.008341666	0.024951356
7945645	387264	*keratin associated protein 5-1*	*KRTAP5-1*	9.19	9.21	8.55	0.008344046	0.024951756
8100456				6.97	6.58	8.22	0.008346865	0.024957601
8116384	255426	*RasGEF domain family, member 1C*	*RASGEF1C*	6.60	6.62	6.26	0.008348221	0.02495907
8078147	686	*biotinidase*	*BTD*	8.55	8.30	9.01	0.008354798	0.024973929
8119034	27154	*bromodomain and PHD finger containing, 3*	*BRPF3*	9.02	9.06	8.43	0.008354921	0.024973929
8108905	57528	*potassium channel tetramerisation domain containing 16*	*KCTD16*	7.68	6.80	6.17	0.008356708	0.024976686
8130038	257218	*SNF2 histone linker PHD RING helicase*	*SHPRH*	7.12	6.74	8.94	0.008358465	0.02497935
8096556	23173	*methionyl aminopeptidase 1*	*METAP1*	9.48	9.49	11.49	0.00836151	0.024985865
7905533	3713	*involucrin*	*IVL*	6.24	6.36	5.93	0.008365042	0.02499383
8099926	23244	*PDS5, regulator of cohesion maintenance, homolog A (S. cerevisiae)*	*PDS5A*	9.24	9.04	10.52	0.008368903	0.02500278
8089795	10063		*COX17*	8.45	9.12	9.50	0.008375898	0.02502109
7903851	164153	*ubiquitin-like 4B*	*UBL4B*	6.61	6.27	5.89	0.008381054	0.0250339

8048478	130617	*zinc finger, AN1-type domain 2B*	*ZFAND2B*	9.57	9.74	8.49	0.008383649	0.025039062
8073081	200316	*apolipoprotein B mRNA editing enzyme, catalytic polypeptide-like 3F*	*APOBEC3F*	9.26	8.38	7.90	0.008393153	0.025064852
8106122	3842	*transportin 1*	*TNPO1*	10.72	10.35	11.81	0.008395093	0.025068052
8019593	83896	*keratin associated protein 3-1*	*KRTAP3-1*	6.13	6.19	5.75	0.008397211	0.025069192
7959002	113675	*serine dehydratase-like*	*SDSL*	8.29	7.92	6.95	0.008396668	0.025069192
8113356	57678	*glycerol-3-phosphate acyltransferase, mitochondrial*	*GPAM*	6.29	6.15	5.41	0.008398142	0.025069378
8039353	7137	*troponin I type 3 (cardiac)*	*TNNI3*	6.54	6.56	7.04	0.008400069	0.025072537
7931665	441056, 653543, 653548, 653545, 653544	*double homeobox 4 like 4 | double homeobox 4 like 7 | double homeobox 4 like 3 | double homeobox 4 like 5 | double homeobox 4 like 6*	*DUX4L4|DUX4L7|DUX4L3|DUX4L5|DUX4L6*	8.34	8.42	7.75	0.008402306	0.025076622
8172658				5.63	5.72	5.14	0.008407638	0.02508994
7925244				7.66	7.72	7.04	0.008409511	0.025092939
8120411	5558	*primase, DNA, polypeptide 2 (58kDa)*	*PRIM2*	8.35	8.09	9.86	0.008411264	0.025095575
8152465	8667	*eukaryotic translation initiation factor 3, subunit H*	*EIF3H*	10.62	10.46	11.58	0.008414384	0.02510229
8030022	2906	*glutamate receptor, ionotropic, N-methyl D-aspartate 2D*	*GRIN2D*	8.09	8.14	7.17	0.00841705	0.025107648
7951596	5450	*POU class 2 associating factor 1*	*POU2AF1*	6.34	6.46	5.97	0.00842517	0.025129272
8135089	10467	*zinc finger, HIT-type containing 1*	*ZNHIT1*	7.61	7.54	7.02	0.008429145	0.025135115
7955983	121364	*olfactory receptor, family 10, subfamily A, member 7*	*OR10A7*	5.54	5.58	5.22	0.008428011	0.025135115
7926670				10.25	10.28	11.15	0.008429741	0.025135115
7969222	83446	*coiled-coil domain containing 70*	*CCDC70*	6.11	6.12	5.70	0.008439845	0.025162645
8081288	55076	*transmembrane protein 45A*	*TMEM45A*	9.70	9.81	8.02	0.008448888	0.025187002
7976744	7528		*YY1*	9.66	9.80	10.29	0.008451173	0.025191214
8011218	406921	*microRNA 132*	*MIR132*	6.40	6.54	5.89	0.00845705	0.025203528

7984662	55889, 342096, 645752, 653641, 653643	*golgin A6 family, member B | golgin A6 family, member A | golgi autoantigen, golgin subfamily a, 6 pseudogene | golgin A6 family, member C | golgin A6 family, member D*	*GOLGA6B |GOLGA6 A|LOC645 752|GOLG A6C|GOL GA6D*	6.49	6.53	6.25	0.008456334	0.025203528
7982366	6447	*secretogranin V (7B2 protein)*	*SCG5*	7.15	7.08	7.97	0.00845885	0.025203688
8150797	51606	*ATPase, H+ transporting, lysosomal 50/57kDa, V1 subunit H*	*ATP6V1H*	7.49	7.76	8.06	0.008458451	0.025203688
8097030				6.49	6.55	5.92	0.008461261	0.025208272
7920341	57459	*GATA zinc finger domain containing 2B*	*GATAD2B*	10.91	10.89	10.06	0.008467359	0.025223834
8174610	57631	*leucine-rich repeats and calponin homology (CH) domain containing 2*	*LRCH2*	6.78	6.15	7.47	0.00846919	0.025226684
8128258				7.98	7.98	7.49	0.008474103	0.025238717
7952813	22997	*immunoglobulin superfamily, member 9B*	*IGSF9B*	7.08	7.16	7.89	0.008475195	0.025238885
8148198	55093	*WDYHV motif containing 1*	*WDYHV1*	6.93	7.25	7.91	0.008475908	0.025238885
8122887	4988	*opioid receptor, mu 1*	*OPRM1*	5.92	5.94	5.58	0.008477691	0.025239028
8075016	388886	*chromosome 22 open reading frame 36*	*C22orf36*	8.52	8.74	7.66	0.008477705	0.025239028
8055104	79595	*Sin3A-associated protein, 130kDa*	*SAP130*	10.80	10.76	11.35	0.008479824	0.025240133
7960764	83758	*retinol binding protein 5, cellular*	*RBP5*	7.47	7.38	7.02	0.00847938	0.025240133
8085372				6.93	6.95	6.42	0.00848487	0.025252549
8045229	50649	*Rho guanine nucleotide exchange factor (GEF) 4*	*ARHGEF4*	7.37	7.26	6.86	0.008489256	0.025255186
8041061	151056	*phospholipase B1*	*PLB1*	6.37	6.35	6.04	0.008488502	0.025255186
7964033	283373	*ankyrin repeat domain 52*	*ANKRD52*	9.15	9.21	7.87	0.008489252	0.025255186
8166703	442444	*family with sequence similarity 47, member C*	*FAM47C*	6.67	6.90	6.33	0.008487723	0.025255186
8070891	257103	*non-protein coding RNA 205*	*NCRNA00 205*	7.31	7.46	6.75	0.008490699	0.025256878
7966878	11113	*citron (rho-interacting, serine/threonine kinase 21)*	*CIT*	7.63	7.53	8.92	0.008492879	0.025260758
7905299	58497	*prune homolog (Drosophila)*	*PRUNE*	8.86	8.60	9.46	0.008494997	0.025261851
7931348	399823	*forkhead box I2*	*FOXI2*	8.29	8.38	7.73	0.008494769	0.025261851
8044915				7.56	7.43	6.78	0.008500217	0.02527477

8154836	304	*annexin A2 pseudogene 2*	*ANXA2P2*	13.00	13.16	12.57	0.008506713	0.025291482
8006968	284110	*gasdermin A*	*GSDMA*	6.56	6.79	6.18	0.00850821	0.02529333
7910014	29097	*cornichon homolog 4 (Drosophila)*	*CNIH4*	10.66	10.84	11.48	0.008512409	0.025303204
8059393	8452	*cullin 3*	*CUL3*	9.79	9.66	10.81	0.008514021	0.02530539
8030925	400713	*zinc finger protein 880*	*ZNF880*	7.42	6.95	8.55	0.00851706	0.025311816
8012397	54785	*chromosome 17 open reading frame 59*	*C17orf59*	7.83	7.85	7.19	0.008520638	0.025319844
7956152	5036	*proliferation-associated 2G4, 38kDa*	*PA2G4*	11.81	12.00	12.70	0.008521644	0.02532023
8015133	25984	*keratin 23 (histone deacetylase inducible)*	*KRT23*	6.43	6.41	5.98	0.008525498	0.025326468
8090152	54763, 152015	*ropporin, rhophilin associated protein 1 | ropporin, rhophilin associated protein 1B*	*ROPN1|ROPN1B*	5.03	5.16	4.94	0.008524696	0.025326468
7946687	5741	*parathyroid hormone*	*PTH*	4.73	4.81	4.61	0.008527851	0.025330849
8119107				6.71	6.86	6.33	0.008529105	0.025331968
8135422	55973	*B-cell receptor-associated protein 29*	*BCAP29*	7.87	8.19	7.46	0.008530379	0.025332604
8096160	83478	*Rho GTPase activating protein 24*	*ARHGAP24*	7.41	8.18	6.30	0.008531074	0.025332604
8011430	3682	*integrin, alpha E (antigen CD103, human mucosal lymphocyte antigen 1; alpha polypeptide)*	*ITGAE*	6.83	6.81	6.52	0.008532983	0.025335668
8099841	10333	*toll-like receptor 6*	*TLR6*	6.04	6.04	5.18	0.00853894	0.025350746
7910587				5.07	4.95	4.88	0.008541745	0.025356466
7968970				5.35	4.96	4.87	0.008543228	0.025358262
8051370				8.40	8.63	7.82	0.008546106	0.025364194
7994889	10847	*Snf2-related CREBBP activator protein*	*SRCAP*	9.93	9.88	8.61	0.008551938	0.025376288
7918517	79084	*WD repeat domain 77*	*WDR77*	8.56	8.70	9.88	0.008551597	0.025376288
8045287				8.75	8.76	9.15	0.008553159	0.025377303
8168081				5.31	5.29	5.80	0.008554678	0.0253792
8019308	4097	*v-maf musculoaponeurotic fibrosarcoma oncogene homolog G (avian)*	*MAFG*	8.76	8.88	8.12	0.008555822	0.025379986
7917482	56267, 494115	*cysteine conjugate-beta lyase 2 | RNA binding motif protein, X-linked-like 1*	*CCBL2|RBMXL1*	9.20	8.78	10.28	0.008558065	0.025384031
7978911	283551	*chromosome 14 open reading frame 182*	*C14orf182*	6.38	6.59	5.89	0.008560983	0.025387468
7912207				6.21	6.15	5.65	0.0085604	0.025387468

8157229				4.54	4.48	4.41	0.008565755	0.02539901
8039474	284418	*family with sequence similarity 71, member E2*	*FAM71E2*	6.47	6.59	5.91	0.008568369	0.025401544
8175550	728712, 30014, 64663, 64648, 171489	*SPANX family, member A2 | sperm protein associated with the nucleus, X-linked, family member A1 | SPANX family, member C | SPANX family, member D | SPANX family, member E*	*SPANXA2| SPANXA1| SPANXC|SPANXD|SPANXE*	5.70	5.79	5.51	0.008567944	0.025401544
8049610	377007	*kelch-like 30 (Drosophila)*	*KLHL30*	7.14	7.18	6.46	0.008572364	0.025410779
8119782	10864	*solute carrier family 22 (organic anion transporter), member 7*	*SLC22A7*	6.35	6.28	5.99	0.00857628	0.025419775
8123685	222826	*chromosome 6 open reading frame 146*	*C6orf146*	5.56	5.56	5.27	0.00858025	0.025428932
8145894	9070	*ash2 (absent, small, or homeotic)-like (Drosophila)*	*ASH2L*	10.30	10.43	11.60	0.008583491	0.025433315
8028002				6.77	6.14	7.11	0.008582796	0.025433315
8044109	5455	*POU class 3 homeobox 3*	*POU3F3*	8.61	8.70	8.01	0.008587468	0.02544249
8040725	56896	*dihydropyrimidinase-like 5*	*DPYSL5*	6.44	6.61	7.30	0.008595075	0.025462411
8133339	155382	*vacuolar protein sorting 37 homolog D (S. cerevisiae)*	*VPS37D*	8.65	8.73	8.05	0.00859792	0.025468227
7954787				7.55	7.55	7.20	0.008607954	0.025495334
8090505				6.88	6.92	6.49	0.008610073	0.025498994
8130962	5134	*programmed cell death 2*	*PDCD2*	8.72	8.79	9.13	0.00861112	0.02549948
7913972				5.49	5.66	5.35	0.008612369	0.025500562
8112337	285672	*SREK1-interacting protein 1*	*SREK1IP1*	7.68	7.61	8.94	0.008613304	0.025500717
8052916	50489		*CD207*	6.09	6.19	5.65	0.008616096	0.025506366
8112763	23440	*orthopedia homeobox*	*OTP*	6.16	6.33	5.88	0.008617491	0.025507879
8000917	146540	*zinc finger protein 785*	*ZNF785*	8.72	8.73	7.99	0.008618672	0.025508761
8067727	10139	*ADP-ribosylation factor related protein 1*	*ARFRP1*	8.10	8.15	7.36	0.008622969	0.025518237
8055236	150527, 646743	*hypothetical LOC150527 | hypothetical LOC646743*	*LOC150527|LOC646743*	5.47	5.72	5.03	0.008623642	0.025518237
7951036	692072, 79101	*small nucleolar RNA, C/D box 5 | TATA box binding protein (TBP)-associated factor, RNA polymerase I, D, 41kDa*	*SNORD5|TAF1D*	10.67	10.62	12.16	0.008633677	0.025545312

7955305	363	*aquaporin 6, kidney specific*	*AQP6*	6.56	6.60	6.16	0.008637688	0.025554562
8002270				7.55	7.78	7.22	0.008639817	0.025558243
7971967	22894, 79866	*DIS3 mitotic control homolog (S. cerevisiae) | chromosome 13 open reading frame 34*	*DIS3|C13orf34*	7.66	7.53	8.77	0.008643041	0.025562542
7968329				6.36	6.00	5.71	0.008642426	0.025562542
7995350				8.23	8.29	7.68	0.008644431	0.025564032
7952797	219938	*spermatogenesis associated 19*	*SPATA19*	5.85	5.88	5.48	0.008647861	0.025571555
7943240	143689	*piwi-like 4 (Drosophila)*	*PIWIL4*	6.11	6.40	5.87	0.008649441	0.025573608
8095477	1448	*casein kappa*	*CSN3*	5.73	5.85	5.45	0.008656506	0.025591878
8117485	11120	*butyrophilin, subfamily 2, member A1*	*BTN2A1*	8.03	8.28	7.50	0.008660069	0.025599787
7992789	51330	*tumor necrosis factor receptor superfamily, member 12A*	*TNFRSF12A*	10.83	12.07	10.90	0.00866893	0.025623359
8143803	285973	*ATG9 autophagy related 9 homolog B (S. cerevisiae)*	*ATG9B*	7.41	7.38	7.06	0.008672384	0.025630942
8073194	9402	*GRB2-related adaptor protein 2*	*GRAP2*	6.02	6.06	5.75	0.008675736	0.025635602
8174494				4.44	4.41	4.30	0.008675686	0.025635602
8146788				5.72	5.31	7.06	0.008678332	0.025640648
8035069	114770	*peptidoglycan recognition protein 2*	*PGLYRP2*	6.34	6.28	5.87	0.008684883	0.025655365
8134349				6.98	6.84	6.71	0.008685091	0.025655365
8158998	26813, 26815, 6130	*small nucleolar RNA, C/D box 36C | small nucleolar RNA, C/D box 36A | ribosomal protein L7a*	*SNORD36C|SNORD36A|RPL7A*	8.12	8.04	9.00	0.008687117	0.025658725
8127024	112744	*interleukin 17F*	*IL17F*	6.39	6.57	6.01	0.008691161	0.025668044
7984188				5.68	5.75	5.44	0.008692474	0.025669295
8046573	10651	*metaxin 2*	*MTX2*	7.98	8.43	9.98	0.008696618	0.025678905
8031997				7.50	7.17	9.11	0.008700821	0.025686061
8142763				5.34	4.97	5.74	0.008699956	0.025686061
8052721	5534	*protein phosphatase 3, regulatory subunit B, alpha*	*PPP3R1*	8.71	8.66	9.62	0.008704108	0.025693139
7927405	57705	*WDFY family member 4*	*WDFY4*	6.03	6.09	5.55	0.008709417	0.025703553

8045423	677822, 79101	*small nucleolar RNA, H/ACA box 40 | TATA box binding protein (TBP)-associated factor, RNA polymerase I, D, 41kDa*	*SNORA40|TAF1D*	6.61	6.54	7.81	0.008709188	0.025703553
8162664	100130916	*HSAL5836*	*LOC100130916*	6.82	6.84	6.35	0.008711727	0.025707744
7996647	50855	*par-6 partitioning defective 6 homolog alpha (C. elegans)*	*PARD6A*	7.93	8.02	8.57	0.008713431	0.025710143
8050240	4953	*ornithine decarboxylase 1*	*ODC1*	10.29	10.55	11.11	0.008716803	0.025717463
8077786	3656	*interleukin-1 receptor-associated kinase 2*	*IRAK2*	7.19	7.32	6.56	0.008719869	0.02572388
7993195	4261	*class II, major histocompatibility complex, transactivator*	*CIITA*	7.26	7.36	6.93	0.008729587	0.025749918
7930956	11196	*SEC23 interacting protein*	*SEC23IP*	8.07	8.00	9.64	0.008732924	0.025754834
8045205	150527, 646743	*hypothetical LOC150527 | hypothetical LOC646743*	*LOC150527|LOC646743*	5.47	5.70	5.03	0.008733038	0.025754834
8144625	640	*B lymphoid tyrosine kinase*	*BLK*	6.60	6.79	6.33	0.008742448	0.025769424
8100154	10699	*corin, serine peptidase*	*CORIN*	6.33	6.38	6.05	0.008741063	0.025769424
8009552	55028	*chromosome 17 open reading frame 80*	*C17orf80*	7.54	7.58	8.80	0.008741556	0.025769424
7952950	100049716		*LOC100049716*	7.76	7.61	6.85	0.008740024	0.025769424
7911331				7.50	7.17	9.10	0.00874084	0.025769424
7984922	4351	*mannose phosphate isomerase*	*MPI*	10.33	10.42	9.44	0.008745423	0.02577556
7934755	4143	*methionine adenosyltransferase I, alpha*	*MAT1A*	6.52	6.44	6.95	0.008751607	0.025791153
8012908	64518	*tektin 3*	*TEKT3*	5.73	5.86	6.33	0.008752769	0.025791945
8075956	3957	*lectin, galactoside-binding, soluble, 2*	*LGALS2*	6.35	6.33	6.09	0.008760169	0.025808483
8056734	9874	*tousled-like kinase 1*	*TLK1*	9.51	8.82	10.43	0.008760149	0.025808483
7956013	2647	*biogenesis of lysosomal organelles complex-1, subunit 1*	*BLOC1S1*	10.28	10.19	9.36	0.008763419	0.025812788
8118142	7124	*tumor necrosis factor*	*TNF*	6.57	6.69	6.09	0.008762567	0.025812788
7942123	595	*cyclin D1*	*CCND1*	9.74	10.85	10.09	0.008768545	0.025825255
7927089				7.50	7.17	9.11	0.008772963	0.025835628
7970139	121793	*chromosome 13 open reading frame 16*	*C13orf16*	6.78	6.72	6.17	0.008775161	0.025839465

7946983	6289	*serum amyloid A2*	*SAA2*	6.08	6.14	5.49	0.008790843	0.025883004
7946221	6881		*TAF10*	12.49	12.53	11.88	0.008794201	0.025890248
8175153				6.46	6.49	7.62	0.008796784	0.025895214
8137435				5.02	4.98	4.77	0.008800405	0.02590323
7937275	60626	*resistance to inhibitors of cholinesterase 8 homolog A (C. elegans)*	*RIC8A*	10.72	10.88	9.43	0.008803461	0.025909584
7976336	55148	*ubiquitin protein ligase E3 component n-recognin 7 (putative)*	*UBR7*	7.09	7.33	8.45	0.008806218	0.025912417
7919584	440689, 337875	*histone cluster 2, H2bf | histone cluster 2, H2ba*	*HIST2H2BF|HIST2H2BA*	9.06	8.74	9.82	0.008806144	0.025912417
8165083	8022	*LIM homeobox 3*	*LHX3*	8.05	7.99	7.24	0.008807342	0.02591308
8126916	5232	*phosphoglycerate kinase 2*	*PGK2*	5.52	5.67	5.39	0.008815412	0.025934184
8037835	6510	*solute carrier family 1 (neutral amino acid transporter), member 5*	*SLC1A5*	12.56	12.52	11.52	0.008816765	0.02593552
8153320				7.62	7.98	7.04	0.008818624	0.025938343
8025515	50700	*retinol dehydrogenase 8 (all-trans)*	*RDH8*	6.53	6.63	6.33	0.008825443	0.025955757
7929719	27291	*chromosome 10 open reading frame 28*	*C10orf28*	6.95	6.61	8.17	0.00882865	0.025960963
7970842				10.64	10.80	10.19	0.008829012	0.025960963
8163569	80709	*AT-hook transcription factor*	*AKNA*	8.78	9.06	7.73	0.008831049	0.02596431
8091402	116441	*transmembrane 4 L six family member 18*	*TM4SF18*	5.51	5.56	7.05	0.008839928	0.025987465
7924463				7.49	7.17	9.10	0.008840725	0.025987465
7972487	23348	*dedicator of cytokinesis 9*	*DOCK9*	7.69	7.54	9.08	0.008841953	0.025988428
7945999	256892	*olfactory receptor, family 51, subfamily F, member 1*	*OR51F1*	5.27	5.38	5.04	0.008844639	0.025993677
8039269	94059, 114823	*leukocyte receptor cluster (LRC) member 9 | leukocyte receptor cluster (LRC) member 8*	*LENG9|LENG8*	8.63	8.39	7.67	0.008851002	0.02600973
8158864	26786, 692093	*small nucleolar RNA, C/D box 62A | small nucleolar RNA, C/D box 62B*	*SNORD62A|SNORD62B*	6.77	6.62	7.57	0.0088535	0.026014421
8158406	54662	*TBC1 domain family, member 13*	*TBC1D13*	10.55	10.39	9.30	0.008857491	0.026018206
7918467	55791	*chromosome 1 open reading frame 103*	*C1orf103*	7.77	7.65	8.92	0.008857281	0.026018206
8110587				4.43	4.41	4.37	0.008855791	0.026018206

7909745	51133	*potassium channel tetramerisation domain containing 3*	*KCTD3*	8.61	8.12	9.95	0.008859367	0.026021067
8157976	84253	*GTPase activating Rap/RanGAP domain-like 3*	*GARNL3*	6.67	6.36	7.12	0.008864683	0.026031384
8008980	388407	*chromosome 17 open reading frame 82*	*C17orf82*	6.86	6.83	6.39	0.008864465	0.026031384
7985999				6.16	6.19	5.65	0.008869674	0.02604339
7945347				7.50	7.17	9.10	0.008871272	0.026045434
7952103	1656	*DEAD (Asp-Glu-Ala-Asp) box polypeptide 6*	*DDX6*	9.26	9.07	11.21	0.008880422	0.026069649
7994615	653808	*zymogen granule protein 16 homolog (rat)*	*ZG16*	6.23	6.30	5.94	0.008882913	0.026074307
8005102				5.51	5.60	5.25	0.008887389	0.026084287
8102530				7.49	7.16	9.10	0.00888812	0.026084287
8179263	7124	*tumor necrosis factor*	*TNF*	6.57	6.70	6.09	0.008894361	0.026088113
7922807	23127	*glycosyltransferase 25 domain containing 2*	*GLT25D2*	7.81	7.05	6.56	0.00889161	0.026088113
8130191	55005	*required for meiotic nuclear division 1 homolog (S. cerevisiae)*	*RMND1*	6.08	6.33	7.37	0.008893912	0.026088113
8116372	55819	*ring finger protein 130*	*RNF130*	9.58	9.86	10.43	0.008895981	0.026088113
8141737	93408	*myosin, light chain 10, regulatory*	*MYL10*	6.24	6.31	5.89	0.008895636	0.026088113
8149358	641517, 245912	*defensin, beta 109, pseudogene 1B | defensin, beta 109, pseudogene 1*	*DEFB109P1B*	7.11	6.82	6.66	0.008895486	0.026088113
7998115				7.50	7.17	9.10	0.008893354	0.026088113
8137668				7.50	7.17	9.10	0.008896653	0.026088113
8066038	84174, 55969	*Src-like-adaptor 2 | chromosome 20 open reading frame 24*	*SLA2|C20orf24*	6.46	6.39	6.03	0.008899493	0.02609379
7904812	10401	*protein inhibitor of activated STAT, 3*	*PIAS3*	10.17	10.19	9.18	0.008902092	0.02609663
7948229	29015	*solute carrier family 43, member 3*	*SLC43A3*	10.97	10.88	9.90	0.008902269	0.02609663
8168990				6.20	5.75	7.28	0.00890718	0.026108375
7962479				6.78	6.91	5.85	0.008909427	0.026112309
7916969	9406	*zinc finger, RAN-binding domain containing 2*	*ZRANB2*	8.56	8.20	10.46	0.008911843	0.02611554
8146669	84675	*tripartite motif-containing 55*	*TRIM55*	6.13	6.15	5.79	0.008912339	0.02611554
8083978	254827	*N-acetylated alpha-linked acidic dipeptidase-like 2*	*NAALADL2*	7.64	7.37	5.39	0.008918724	0.026131598

8071268	2812, 5413	*glycoprotein Ib (platelet), beta polypeptide | septin 5*	*GP1BB|SEPT5*	8.42	8.36	7.64	0.008922407	0.026139736
7951385	838	*caspase 5, apoptosis-related cysteine peptidase*	*CASP5*	5.39	5.25	5.07	0.008924037	0.02614186
7941136	738	*chromosome 11 open reading frame 2*	*C11orf2*	10.43	10.40	9.76	0.008926161	0.026145427
8007904	9570	*golgi SNAP receptor complex member 2*	*GOSR2*	9.99	9.93	9.35	0.008929364	0.026149506
8016504				7.57	7.64	6.83	0.00892928	0.026149506
7954551	341346	*chromosome 12 open reading frame 70*	*C12orf70*	5.43	5.35	5.18	0.008932814	0.026156954
8155169	8434	*reversion-inducing-cysteine-rich protein with kazal motifs*	*RECK*	10.96	10.26	7.85	0.008933958	0.02615765
7952307	406911	*microRNA 125b-1*	*MIR125B1*	6.95	6.68	5.88	0.008942323	0.026179487
8029693	2354	*FBJ murine osteosarcoma viral oncogene homolog B*	*FOSB*	6.92	6.99	6.35	0.008947349	0.026191546
8039280	199713	*NLR family, pyrin domain containing 7*	*NLRP7*	5.80	6.04	6.79	0.008966631	0.02624533
8158862	26786, 692093	*small nucleolar RNA, C/D box 62A | small nucleolar RNA, C/D box 62B*	*SNORD62A|SNORD62B*	6.78	6.62	7.57	0.008970415	0.026253743
7949674	83759	*RNA binding motif protein 4B*	*RBM4B*	10.97	10.92	10.31	0.008973509	0.026260136
8133874				8.05	8.28	7.44	0.008974531	0.026260464
7953469	2784	*guanine nucleotide binding protein (G protein), beta polypeptide 3*	*GNB3*	7.91	7.73	7.08	0.008975643	0.026261056
8005955	26809	*small nucleolar RNA, C/D box 42A*	*SNORD42A*	7.51	7.85	8.41	0.008977591	0.026264094
7998249				6.50	6.53	6.17	0.008982086	0.02627458
8122807	9590	*A kinase (PRKA) anchor protein 12*	*AKAP12*	9.13	6.67	9.40	0.008985806	0.0262828
7907053	92235	*dual specificity phosphatase 27 (putative)*	*DUSP27*	6.31	6.44	6.01	0.008987223	0.026284281
8139941				5.77	5.88	5.46	0.008991104	0.026292967
8114249	9547	*chemokine (C-X-C motif) ligand 14*	*CXCL14*	9.87	7.84	7.85	0.008996329	0.026303807
8108166	79770	*thioredoxin domain containing 15*	*TXNDC15*	9.26	9.13	8.46	0.008996633	0.026303807
8096331				8.30	8.23	7.60	0.009000634	0.026312841
8063156	958		*CD40*	7.90	7.14	6.92	0.009007732	0.02632826
8024013	126353	*chromosome 19 open reading frame 21*	*C19orf21*	7.00	6.99	6.50	0.009007583	0.02632826
7963590	51380	*cysteine sulfinic acid decarboxylase*	*CSAD*	7.83	7.45	6.89	0.009010899	0.026334848

8139832	51351, 2086	*zinc finger protein 117 | endogenous retroviral sequence 3*	*ZNF117|ERV3*	6.32	5.88	8.17	0.00901204	0.026335517
8078350	7048	*transforming growth factor, beta receptor II (70/80kDa)*	*TGFBR2*	10.35	10.78	8.22	0.009015176	0.026342018
8135273				7.42	7.61	6.84	0.009019501	0.026351985
8113010	902	*cyclin H*	*CCNH*	6.85	7.20	7.75	0.009022709	0.026353357
8178749	63943	*FK506 binding protein like*	*FKBPL*	6.94	7.08	6.58	0.009021918	0.026353357
7900488	407034	*microRNA 30e*	*MIR30E*	6.25	5.94	7.10	0.009020984	0.026353357
8123148	29074	*mitochondrial ribosomal protein L18*	*MRPL18*	9.96	10.12	10.72	0.00902605	0.026360448
7945445	256329, 115399, 692247	*chromosome 11 open reading frame 35 | leucine rich repeat containing 56 | hypothetical locus LOC692247*	*C11orf35|LRRC56|LOC692247*	8.03	8.00	7.60	0.009028156	0.026361266
8138155				6.69	6.72	6.40	0.009028094	0.026361266
7931353	5791	*protein tyrosine phosphatase, receptor type, E*	*PTPRE*	6.83	7.16	6.41	0.009031715	0.02636899
8067593	54915	*YTH domain family, member 1*	*YTHDF1*	10.71	10.67	11.28	0.009035535	0.026369477
8020141	147495	*adenomatosis polyposis coli down-regulated 1*	*APCDD1*	11.19	9.45	6.83	0.009034629	0.026369477
8141829	246721, 100134938, 548644, 84820	*polymerase (RNA) II (DNA directed) polypeptide J2 | uroplakin 3B-like | polymerase (RNA) II (DNA directed) polypeptide J3 | polymerase (RNA) II (DNA directed) polypeptide J4, pseudogene*	*POLR2J2|UPK3BL|POLR2J3|POLR2J4*	10.80	11.21	9.77	0.009034281	0.026369477
8102210				5.70	5.85	5.35	0.009032873	0.026369477
8024111	1265	*calponin 2*	*CNN2*	12.92	13.06	12.29	0.0090378	0.02637342
8026217	54862	*coiled-coil and C2 domain containing 1A*	*CC2D1A*	8.65	8.65	7.77	0.00903998	0.026377117
8013908	57532	*nuclear fragile X mental retardation protein interacting protein 2*	*NUFIP2*	8.44	8.31	10.69	0.009041839	0.026379874
8139534	168507	*polycystic kidney disease 1 like 1*	*PKD1L1*	6.18	6.21	5.83	0.009043027	0.026380673
8172803				5.77	5.86	5.44	0.009049227	0.026396094
7905339	126626	*GA binding protein transcription factor, beta subunit 2*	*GABPB2*	7.70	8.03	6.83	0.009051864	0.026400626
7991512	196968	*dynamin 1 pseudogene*	*C15orf51*	7.26	6.81	6.33	0.00905261	0.026400626
7936673	6001	*regulator of G-protein signaling 10*	*RGS10*	9.15	8.34	8.05	0.009056082	0.026402751
7938269	7275	*tubby homolog (mouse)*	*TUB*	8.27	7.92	8.22	0.009054645	0.026402751

7977319	122618	*phospholipase D family, member 4*	*PLD4*	7.34	7.34	6.88	0.009055441	0.026402751
8005089	1352		*COX10*	9.01	8.94	9.71	0.00906706	0.026431594
8153625	26873	*5-oxoprolinase (ATP-hydrolysing)*	*OPLAH*	8.37	8.38	7.37	0.009067806	0.026431594
8025004	828	*calcyphosine*	*CAPS*	8.25	8.23	7.62	0.009073342	0.02644506
8064502	6628	*small nuclear ribonucleoprotein polypeptides B and B1*	*SNRPB*	11.05	11.15	12.27	0.00907893	0.026458675
8132097	84182, 358, 11185	*family with sequence similarity 188, member B | aquaporin 1 (Colton blood group) | indolethylamine N-methyltransferase*	*FAM188B|AQP1|INMT*	8.94	8.17	7.58	0.009085205	0.02647429
7987315	70	*actin, alpha, cardiac muscle 1*	*ACTC1*	7.09	9.69	11.78	0.009095989	0.02650304
8101304	153020	*RasGEF domain family, member 1B*	*RASGEF1B*	5.42	5.40	6.48	0.009097137	0.026503706
7907008				5.77	5.65	5.28	0.009100404	0.02651055
7957835	9971	*nuclear receptor subfamily 1, group H, member 4*	*NR1H4*	5.16	5.22	4.98	0.009103105	0.026515743
7979957	89919	*chromosome 14 open reading frame 56*	*C14orf56*	5.93	5.95	5.76	0.00910449	0.026517104
7945781	7262	*pleckstrin homology-like domain, family A, member 2*	*PHLDA2*	8.96	9.13	8.01	0.009109928	0.026530266
8117922	80742	*proline rich 3*	*PRR3*	8.81	9.17	8.44	0.009113198	0.026531758
8168892	140597	*transcription elongation factor A (SII)-like 2*	*TCEAL2*	6.35	6.49	7.00	0.009111396	0.026531758
7954995				5.00	5.14	4.67	0.009113009	0.026531758
8078187	23228	*phospholipase C-like 2*	*PLCL2*	7.29	6.58	7.80	0.009118942	0.026543126
8137381	116988	*ArfGAP with GTPase domain, ankyrin repeat and PH domain 3*	*AGAP3*	8.56	8.46	7.80	0.009118614	0.026543126
7919314	2330	*flavin containing monooxygenase 5*	*FMO5*	6.13	6.04	5.69	0.00912375	0.026549092
7918681	54665	*round spermatid basic protein 1*	*RSBN1*	8.07	7.93	8.55	0.009121968	0.026549092
7980306	646701	*developmental pluripotency associated 5*	*LOC646701*	6.03	6.10	5.45	0.009123107	0.026549092
7967084	51367	*processing of precursor 5, ribonuclease P/MRP subunit (S. cerevisiae)*	*POP5*	6.88	7.10	7.89	0.00912506	0.026550228
8080853				5.46	5.31	5.20	0.009128311	0.026554752
8094717				5.34	5.44	6.10	0.009128455	0.026554752
8098041	55314	*transmembrane protein 144*	*TMEM144*	5.43	5.48	6.37	0.009137637	0.026570754
8157945	403341	*zinc finger and BTB domain containing 34*	*ZBTB34*	8.24	8.27	9.36	0.009137074	0.026570754

8038200				6.24	6.20	5.86	0.009137436	0.026570754
8131867				9.73	10.95	11.10	0.00913556	0.026570754
8057599	7035	*tissue factor pathway inhibitor (lipoprotein-associated coagulation inhibitor)*	*TFPI*	8.69	6.95	6.13	0.009140809	0.026577301
8118137	4049	*lymphotoxin alpha (TNF superfamily, member 1)*	*LTA*	6.58	6.64	6.22	0.009143717	0.026583077
7956088	5869		*RAB5B*	11.16	10.99	10.00	0.009147502	0.026591403
8043456				4.90	4.83	4.73	0.00915486	0.026610114
8042168	84542		*KIAA1841*	6.21	6.29	6.76	0.009158888	0.026616462
8167457	28952, 50943	*coiled-coil domain containing 22 | forkhead box P3*	*CCDC22|FOXP3*	8.46	8.40	7.52	0.009158143	0.026616462
8174831	643311, 728072, 653282	*cancer/testis antigen family 147, member B1 | cancer/testis antigen family 47, member A5 | cancer/testis antigen family 47, member A7*	*CT47B1|CT47A5|CT47A7*	6.58	6.69	6.22	0.009161213	0.02662054
7906863	6675	*UDP-N-acteylglucosamine pyrophosphorylase 1*	*UAP1*	9.22	10.06	9.25	0.009163349	0.026621386
8153868	441381, 414919	*leucine rich repeat containing 24 | chromosome 8 open reading frame 82*	*LRRC24|C8orf82*	8.69	8.72	7.91	0.009162967	0.026621386
8164937	642968, 643750	*family with sequence similarity 163, member B | protein FAM163B-like*	*FAM163B|LOC643750*	7.95	7.97	7.58	0.009166756	0.026625926
8149146				6.95	7.12	6.35	0.009166346	0.026625926
8045368	130013	*aminocarboxymuconate semialdehyde decarboxylase*	*ACMSD*	5.48	5.46	5.22	0.009169352	0.026630787
8013071	201163	*folliculin*	*FLCN*	8.59	8.56	8.05	0.009171773	0.026635138
8053763	64969	*mitochondrial ribosomal protein S5*	*MRPS5*	9.17	9.21	9.94	0.009173401	0.026637185
8006121				6.44	6.45	5.99	0.009175224	0.026639802
8156160	55582	*kinesin family member 27*	*KIF27*	7.33	7.35	7.82	0.009178121	0.026640173
8146225	114926	*chromosome 8 open reading frame 40*	*C8orf40*	9.26	9.20	9.70	0.009176361	0.026640173
8116128	202181		*LOC202181*	6.93	6.86	6.36	0.009178107	0.026640173
8086627	259173	*ALS2 C-terminal like*	*ALS2CL*	7.04	6.99	6.49	0.009182301	0.02664963
8105432	6171, 6172	*ribosomal protein L41 | ribosomal protein L41 pseudogene 5*	*RPL41|RPL41P5*	13.16	13.25	13.08	0.009185859	0.026657274
8102024	7323	*ubiquitin-conjugating enzyme E2D 3 (UBC4/5 homolog, yeast)*	*UBE2D3*	8.83	8.90	9.34	0.009187236	0.026658589

8078569	2803	*golgin A4*	*GOLGA4*	8.00	7.48	9.67	0.009191586	0.026664145
8069831	337970, 337971	*keratin associated protein 19-3 | keratin associated protein 19-4*	*KRTAP19-3|KRTAP19-4*	5.95	5.79	5.36	0.009190172	0.026664145
7998392				6.38	6.50	5.96	0.009191921	0.026664145
8123274	4216	*mitogen-activated protein kinase kinase kinase 4*	*MAP3K4*	7.79	8.12	9.58	0.009199102	0.026679615
8093304	9034	*chemokine (C-C motif) receptor-like 2*	*CCRL2*	5.20	5.20	4.97	0.00919832	0.026679615
8115516	11063	*SRY (sex determining region Y)-box 30*	*SOX30*	6.14	6.04	6.33	0.009204468	0.026686821
7962794	121274	*zinc finger protein 641*	*ZNF641*	8.30	8.33	7.45	0.009205285	0.026686821
8065248	100130264		*LOC100130264*	6.51	6.44	6.06	0.009202801	0.026686821
8155591	729171, 644249	*ankyrin repeat domain 20B | hypothetical LOC644249*	*ANKRD20B|LOC644249*	4.75	4.68	4.94	0.009204048	0.026686821
8164438	407003	*microRNA 219-2*	*MIR219-2*	6.91	6.80	6.35	0.009209462	0.02669625
8146391				5.18	5.26	5.76	0.009215434	0.02671088
8099754	401123	*hypothetical LOC401123*	*FLJ45721*	5.53	5.58	5.35	0.009216518	0.026711341
8177983	7124	*tumor necrosis factor*	*TNF*	6.58	6.69	6.10	0.009238539	0.026772475
7923271	779	*calcium channel, voltage-dependent, L type, alpha 1S subunit*	*CACNA1S*	6.43	6.55	6.04	0.009240354	0.026775047
7953395	50813	*COP9 constitutive photomorphogenic homolog subunit 7A (Arabidopsis)*	*COPS7A*	11.88	11.79	10.79	0.009248736	0.026793955
7957715	121441	*neural precursor cell expressed, developmentally down-regulated 1*	*NEDD1*	8.29	8.57	9.59	0.009247832	0.026793955
8131087	340260	*UNC homeobox*	*UNCX*	9.19	9.36	8.56	0.009261293	0.02682764
7901969	4919	*receptor tyrosine kinase-like orphan receptor 1*	*ROR1*	9.21	8.57	10.85	0.009264444	0.026833465
7940824	79829	*N(alpha)-acetyltransferase 40, NatD catalytic subunit, homolog (S. cerevisiae)*	*NAA40*	8.98	8.89	9.61	0.009265163	0.026833465
8172858	23096	*IQ motif and Sec7 domain 2*	*IQSEC2*	9.15	8.76	7.82	0.009267972	0.026836216
8061666				7.01	6.75	6.25	0.00926742	0.026836216
8130837	79981	*FERM domain containing 1*	*FRMD1*	6.64	6.68	6.31	0.009269431	0.026837751
7995328				9.08	9.02	8.72	0.009272142	0.026842907

7989335	302	*annexin A2*	*ANXA2*	12.72	12.91	12.22	0.009275706	0.02685053
7964733	204010	*ribosomal protein SA pseudogene 52*	*RPSAP52*	5.35	5.22	4.96	0.009286569	0.02687928
7897860	943	*tumor necrosis factor receptor superfamily, member 8*	*TNFRSF8*	7.80	7.85	8.61	0.009288142	0.026879488
7954613	55297	*coiled-coil domain containing 91*	*CCDC91*	8.57	8.46	9.53	0.009288502	0.026879488
8022655	361	*aquaporin 4*	*AQP4*	5.50	5.45	5.95	0.009290489	0.026882544
8058344				5.92	5.95	5.60	0.009292574	0.02688588
8094751	55584	*cholinergic receptor, nicotinic, alpha 9*	*CHRNA9*	5.74	6.00	6.02	0.009293572	0.026886074
8085716	6304	*SATB homeobox 1*	*SATB1*	6.54	7.68	9.07	0.009296234	0.026888385
8104590	56172, 100130744	*ankylosis, progressive homolog (mouse) | hypothetical LOC100130744*	*ANKH|LOC100130744*	7.66	7.59	7.09	0.009295394	0.026888385
7937942	390036	*olfactory receptor, family 52, subfamily K, member 1*	*OR52K1*	5.33	5.37	5.11	0.009297926	0.026890585
8092468	94032	*calcium/calmodulin-dependent protein kinase II inhibitor 2*	*CAMK2N2*	8.68	8.72	8.09	0.009299793	0.026893292
8087839	25886	*POC1 centriolar protein homolog A (Chlamydomonas)*	*POC1A*	6.97	7.33	8.47	0.009301205	0.026894681
8130988	729515	*chromosome 6 open reading frame 35*	*C6orf35*	7.51	7.64	8.60	0.009302467	0.026895635
8158995	3933	*lipocalin 1 (tear prealbumin)*	*LCN1*	6.47	7.19	6.32	0.00930443	0.026898619
8048712				7.32	7.39	6.84	0.009309587	0.02691083
7937257	147199, 653486	*secretoglobin, family 1C, member 1 | secretoglobin, family 1C, member 1-like*	*SCGB1C1|LOC653486*	7.53	7.47	7.01	0.009310687	0.026911316
8178293	116511	*MAS1 oncogene-like*	*MAS1L*	5.54	5.56	5.30	0.009316917	0.026926626
8060432	7053	*transglutaminase 3 (E polypeptide, protein-glutamine-gamma-glutamyltransferase)*	*TGM3*	5.90	5.84	5.45	0.009318266	0.02692783
7921690	55600	*intelectin 1 (galactofuranose binding)*	*ITLN1*	5.43	5.44	5.19	0.009320227	0.026928104
7939994	390174, 504191	*olfactory receptor, family 9, subfamily G, member 1 | olfactory receptor, family 9, subfamily G, member 9*	*OR9G1|OR9G9*	5.62	5.68	5.36	0.009319765	0.026928104
7974425	23034	*sterile alpha motif domain containing 4A*	*SAMD4A*	8.16	8.93	8.17	0.009324872	0.02693883
7988442				5.37	5.61	5.16	0.009325953	0.026939258
8160889	6366	*chemokine (C-C motif) ligand 21*	*CCL21*	7.07	7.09	6.48	0.009330682	0.02695022
8057004	50940	*phosphodiesterase 11A*	*PDE11A*	5.78	6.64	5.42	0.009332845	0.026951075

7999769	339044, 9284, 399491, 100288332, 642778	*polycystic kidney disease 1 (autosomal dominant) pseudogene 1 | nuclear pore complex interacting protein | GPS, PLAT and transmembrane domain-containing protein | GPS, PLAT and transmembrane domain-containing protein FLJ00285-like | nuclear pore complex-interacting protein-like 1-like*	*PKD1P1|NPIP|LOC399491|LOC100288332|LOC642778*	11.66	11.66	10.55	0.009332342	0.026951075
8104369	6715	*steroid-5-alpha-reductase, alpha polypeptide 1 (3-oxo-5 alpha-steroid delta 4-dehydrogenase alpha 1)*	*SRD5A1*	8.96	9.18	9.57	0.009337045	0.026960509
8021461	2922	*gastrin-releasing peptide*	*GRP*	7.14	7.40	6.66	0.009339456	0.026962074
8063601	128602	*chromosome 20 open reading frame 85*	*C20orf85*	6.59	6.68	6.21	0.009338999	0.026962074
7999196	26048	*zinc finger protein 500*	*ZNF500*	7.66	7.64	6.89	0.009343243	0.026970312
8175666	2564	*gamma-aminobutyric acid (GABA) A receptor, epsilon*	*GABRE*	9.40	9.24	7.31	0.009349501	0.026983872
7973448	10901, 317749	*dehydrogenase/reductase (SDR family) member 4 | dehydrogenase/reductase (SDR family) member 4 like 2*	*DHRS4|DHRS4L2*	9.26	8.92	8.11	0.00934981	0.026983872
8005879	9058	*solute carrier family 13 (sodium-dependent dicarboxylate transporter), member 2*	*SLC13A2*	6.74	6.78	6.31	0.009353235	0.026991058
8050942	9381	*otoferlin*	*OTOF*	6.44	6.47	6.11	0.009354382	0.02699167
8076757	9620	*cadherin, EGF LAG seven-pass G-type receptor 1 (flamingo homolog, Drosophila)*	*CELSR1*	7.23	7.41	7.72	0.00936115	0.027007235
7925622	25909	*AT hook containing transcription factor 1*	*AHCTF1*	7.51	7.28	9.25	0.009361962	0.027007235
7945368	387733	*interferon induced transmembrane protein 5*	*IFITM5*	6.26	6.31	5.80	0.009362583	0.027007235
8015410				6.59	6.77	6.30	0.009367989	0.02702013
7927307	244, 653145, 728113	*annexin A8-like 2 | annexin A8 | annexin A8-like 1*	*ANXA8L2|ANXA8L1*	6.76	6.71	6.34	0.009375551	0.02703924
7938592	84188	*fatty acyl CoA reductase 1*	*FAR1*	7.80	8.10	9.62	0.009383652	0.0270599
8122670				5.37	5.78	5.01	0.009392334	0.02708223
8003633	727857	*basic helix-loop-helix family, member a9*	*BHLHA9*	8.81	8.94	8.05	0.009395823	0.027089585
8005529	125206, 644815	*solute carrier family 5 (sodium/glucose cotransporter), member 10 | family with sequence similarity 83, member G*	*SLC5A10|FAM83G*	7.19	7.34	6.69	0.009396869	0.027089894
8069503	441956	*similar to cDNA sequence BC021523*	*LOC441956*	7.80	7.50	6.66	0.009400406	0.02709468

7957568				7.53	7.83	7.10	0.009400157	0.02709468
8028924	8190	*melanoma inhibitory activity*	*MIA*	7.65	7.66	7.19	0.009401921	0.027096342
8056794				5.48	5.54	5.27	0.009409547	0.027115613
7997829	9780, 100289580	*family with sequence similarity 38, member A | hypothetical LOC100289580*	*FAM38A|LOC100289580*	6.44	6.60	5.93	0.009413496	0.027123284
8058693				4.94	4.91	4.70	0.009414088	0.027123284
8036969	1548, 1549, 1553	*cytochrome P450, family 2, subfamily A, polypeptide 6 | cytochrome P450, family 2, subfamily A, polypeptide 7 | cytochrome P450, family 2, subfamily A, polypeptide 13*	*CYP2A6|CYP2A7|CYP2A13*	6.68	6.74	6.26	0.009416093	0.027126353
8050608	60526	*chromosome 2 open reading frame 43*	*C2orf43*	6.30	6.06	7.87	0.009420386	0.027130598
7939492	387763	*chromosome 11 open reading frame 96*	*C11orf96*	7.10	7.45	6.83	0.00942015	0.027130598
7932834	55149, 729668	*mitochondrial poly(A) polymerase | golgi autoantigen, golgin subfamily a, 6 pseudogene*	*MTPAP|LOC729668*	6.62	6.83	7.44	0.009419036	0.027130598
8094936	1259, 280665	*cyclic nucleotide gated channel alpha 1 | anti-CNG alpha 1 cation channel translation product-like*	*CNGA1|LOC280665*	5.48	5.47	6.40	0.009421564	0.027131284
8082350	4171	*minichromosome maintenance complex component 2*	*MCM2*	7.89	8.13	9.93	0.009424649	0.027137462
8132725	7378	*uridine phosphorylase 1*	*UPP1*	7.26	8.13	8.27	0.009426441	0.027139915
7936096	22984	*programmed cell death 11*	*PDCD11*	7.30	7.35	7.00	0.009429479	0.027145954
7986769	7337	*ubiquitin protein ligase E3A*	*UBE3A*	8.04	8.10	8.88	0.009432175	0.027151009
7928789	5995	*retinal G protein coupled receptor*	*RGR*	6.65	6.65	6.22	0.009437105	0.02716249
8160769				7.10	7.18	6.86	0.009438103	0.027162656
8146241				4.63	4.66	4.49	0.009439335	0.027163494
7900228	4725, 6134	*NADH dehydrogenase (ubiquinone) Fe-S protein 5, 15kDa (NADH-coenzyme Q reductase) | ribosomal protein L10*	*NDUFS5|RPL10*	12.51	12.57	13.08	0.009441688	0.027164849
8060539	5786, 64601	*protein tyrosine phosphatase, receptor type, A | vacuolar protein sorting 16 homolog (S. cerevisiae)*	*PTPRA|VPS16*	9.01	8.94	8.53	0.009441632	0.027164849
7983663	9101	*ubiquitin specific peptidase 8*	*USP8*	8.20	7.96	9.93	0.009447712	0.02717947
7921930				5.95	6.08	5.62	0.009451021	0.027183663
7926354				5.05	4.99	4.70	0.009451052	0.027183663

8122969				4.74	4.89	4.60	0.009454856	0.027191894
8078834	57599	*WD repeat domain 48*	*WDR48*	7.71	7.80	9.26	0.009459658	0.027202994
8047505	165057		*FLJ39061*	6.07	6.05	5.62	0.009464475	0.027214138
8106986	22836	*Rho-related BTB domain containing 3*	*RHOBTB3*	10.75	9.01	7.36	0.009466028	0.02721589
7961702	3764	*potassium inwardly-rectifying channel, subfamily J, member 8*	*KCNJ8*	9.14	8.44	7.68	0.009480414	0.027254539
8059071	79840, 151295	*nonhomologous end-joining factor 1 | solute carrier family 23 (nucleobase transporters), member 3*	*NHEJ1|SLC23A3*	8.53	8.41	9.49	0.009484758	0.02726431
8035315	115861	*nucleoredoxin-like 1*	*NXNL1*	8.15	8.31	7.83	0.009487302	0.027268909
7904361	54855	*family with sequence similarity 46, member C*	*FAM46C*	8.35	6.58	6.49	0.009489331	0.027272027
7999468	9516	*lipopolysaccharide-induced TNF factor*	*LITAF*	11.66	10.82	11.88	0.009492788	0.027279247
8141363	89122	*tripartite motif-containing 4*	*TRIM4*	8.78	8.65	7.75	0.009505976	0.027314425
8125568	1302	*collagen, type XI, alpha 2*	*COL11A2*	7.62	7.73	7.30	0.009515668	0.027339553
8175195	90161	*heparan sulfate 6-O-sulfotransferase 2*	*HS6ST2*	5.92	5.99	6.51	0.009517873	0.027343165
8088830	55164		*SHQ1*	8.24	8.42	9.46	0.009522453	0.02734544
7923942	83953	*Fc receptor, IgA, IgM, high affinity*	*FCAMR*	6.75	6.80	6.35	0.009522035	0.02734544
8105411	133396	*interleukin 31 receptor A*	*IL31RA*	5.44	5.52	5.23	0.00952086	0.02734544
8174226				6.86	6.51	6.09	0.009520393	0.02734544
8064779	146	*adrenergic, alpha-1D-, receptor*	*ADRA1D*	8.59	8.96	7.32	0.009538476	0.02738873
8071375	729461, 653203	*hypothetical LOC729461 | similar to hypothetical gene supported by BC039313*	*LOC729461|LOC653203*	5.86	5.89	5.62	0.009539689	0.027389485
8129363	51020	*HD domain containing 2*	*HDDC2*	9.76	9.88	10.35	0.00954214	0.0273938
7903619	6301	*seryl-tRNA synthetase*	*SARS*	12.05	12.08	11.27	0.009543599	0.027395261
8120983	3354	*5-hydroxytryptamine (serotonin) receptor 1E*	*HTR1E*	5.97	6.06	5.62	0.009544631	0.0273955
7901311	2306	*forkhead box D2*	*FOXD2*	6.94	7.02	6.38	0.00954631	0.027397595
8131265	9907		*KIAA0415*	8.21	8.35	7.48	0.009548191	0.02740027
7925737	391189	*olfactory receptor, family 11, subfamily L, member 1*	*OR11L1*	6.53	6.36	6.08	0.009554222	0.027414853
8089851	3081	*homogentisate 1,2-dioxygenase*	*HGD*	5.34	5.50	5.19	0.009560465	0.027430039
8037389	56006	*chromosome 19 open reading frame 61*	*C19orf61*	10.12	10.14	9.05	0.009564484	0.027438844

7978676	7080	*NK2 homeobox 1*	*NKX2-1*	6.14	6.15	5.78	0.009573093	0.027460812
7938231	8495	*PTPRF interacting protein, binding protein 2 (liprin beta 2)*	*PPFIBP2*	8.81	8.48	7.68	0.00957683	0.02746737
7984799				7.18	7.48	6.86	0.009577282	0.02746737
8038547	256236	*napsin B aspartic peptidase pseudogene*	*NAPSB*	7.17	7.26	6.67	0.009579497	0.027470995
7925939	1109	*aldo-keto reductase family 1, member C4 (chlordecone reductase; 3-alpha hydroxysteroid dehydrogenase, type I; dihydrodiol dehydrogenase 4)*	*AKR1C4*	5.14	5.04	4.79	0.009584995	0.02747759
8151909	7381	*ubiquinol-cytochrome c reductase binding protein*	*UQCRB*	6.82	6.82	7.81	0.009585604	0.02747759
7897288	83715	*espin*	*ESPN*	8.17	8.18	7.56	0.009585473	0.02747759
8049950	100131763		*LOC100131763*	7.42	7.45	6.91	0.009582829	0.02747759
7929814				4.97	4.88	4.79	0.0095874	0.027480008
8053406	54884	*retinol saturase (all-trans-retinol 13,14-reductase)*	*RETSAT*	9.87	9.33	8.12	0.009592525	0.027491968
7905559	149018	*late cornified envelope-like proline-rich 1*	*LELP1*	7.05	7.18	6.65	0.009595585	0.027498009
8038747	43849	*kallikrein-related peptidase 12*	*KLK12*	6.30	6.31	6.05	0.009600812	0.02750854
8002005	146212	*potassium channel tetramerisation domain containing 19*	*KCTD19*	6.18	6.15	5.77	0.009601166	0.02750854
8093590	6047	*ring finger protein 4*	*RNF4*	9.06	9.18	9.71	0.009602393	0.027509326
8159491	733	*complement component 8, gamma polypeptide*	*C8G*	7.80	7.99	7.37	0.009610896	0.027530953
8037315	653583	*pleckstrin homology-like domain, family B, member 3*	*PHLDB3*	7.56	7.56	6.90	0.009614827	0.027539482
8124942	534, 7919	*ATPase, H+ transporting, lysosomal 13kDa, V1 subunit G2 | HLA-B associated transcript 1*	*ATP6V1G2 |BAT1*	8.63	7.93	8.08	0.00961672	0.027539682
8136471				8.15	8.25	7.37	0.009616804	0.027539682
8149942	55246	*coiled-coil domain containing 25*	*CCDC25*	8.31	8.12	9.27	0.009621705	0.027550982
8015490	7266	*DnaJ (Hsp40) homolog, subfamily C, member 7*	*DNAJC7*	8.34	8.43	9.85	0.009623999	0.027554817
8118548	3122	*major histocompatibility complex, class II, DR alpha*	*HLA-DRA*	6.12	6.04	6.93	0.009624974	0.027554875
8013465	644945, 400578, 3868, 729252	*keratin 16 pseudogene 3 | keratin 16 pseudogene 2 | keratin 16 pseudogene 1*	*KRT16P3| KRT16P2| KRT16P1*	7.18	7.25	6.82	0.009626077	0.0275553
7955297	362	*aquaporin 5*	*AQP5*	7.27	7.23	6.64	0.009640614	0.027591443

8151125	100129654	*transcription factor 24*	*TCF24*	9.46	9.40	8.82	0.009639815	0.027591443
8157522				5.67	5.87	5.48	0.009652256	0.027622024
8056022	130940	*coiled-coil domain containing 148*	*CCDC148*	5.70	5.27	5.34	0.009653864	0.027623886
7962831	904	*cyclin T1*	*CCNT1*	8.86	8.74	10.81	0.009655955	0.027624395
8018908	8632	*dynein, axonemal, heavy chain 17*	*DNAH17*	5.62	5.52	5.17	0.009655286	0.027624395
8075063	89781	*Hermansky-Pudlak syndrome 4*	*HPS4*	7.84	7.91	8.43	0.009657455	0.027625946
8126147	55776	*chromosome 6 open reading frame 64*	*C6orf64*	7.93	7.95	7.63	0.009659953	0.027630355
7928821				5.07	4.93	4.74	0.009667108	0.027648082
7926636				5.50	5.46	5.08	0.009671615	0.027658232
8080923	5700	*proteasome (prosome, macropain) 26S subunit, ATPase, 1*	*PSMC1*	9.40	9.68	10.56	0.009673808	0.027659023
8124365	10246	*solute carrier family 17 (sodium phosphate), member 2*	*SLC17A2*	6.29	6.24	5.85	0.009673096	0.027659023
8004271	37	*acyl-CoA dehydrogenase, very long chain*	*ACADVL*	10.12	10.42	9.39	0.009676762	0.02766473
7961767	9847		*KIAA0528*	8.53	7.95	10.07	0.009679133	0.027668769
8071390				4.79	4.72	4.56	0.009680448	0.027669787
8130976	401286		*LOC401286*	5.97	5.96	5.66	0.009683121	0.027673073
8046512				5.86	6.01	6.32	0.009683515	0.027673073
7910901	100130331	*actin, gamma-like*	*LOC100130331*	11.58	11.50	11.04	0.00969109	0.02769198
7978366	3002	*granzyme B (granzyme 2, cytotoxic T-lymphocyte-associated serine esterase 1)*	*GZMB*	6.49	6.53	6.32	0.009692224	0.027692478
8149256				7.60	7.61	7.18	0.009693688	0.027693922
8162514	2176	*Fanconi anemia, complementation group C*	*FANCC*	7.01	7.09	7.84	0.009698293	0.027701596
7971563	57092	*PEST proteolytic signal containing nuclear protein*	*PCNP*	9.98	10.16	10.70	0.00969734	0.027701596
8104912	6502	*S-phase kinase-associated protein 2 (p45)*	*SKP2*	10.49	9.56	11.57	0.009700993	0.027704287
8168424	6567	*solute carrier family 16, member 2 (monocarboxylic acid transporter 8)*	*SLC16A2*	10.60	10.58	9.37	0.009702114	0.027704287
7957452	8092	*ALX homeobox 1*	*ALX1*	6.21	5.84	6.61	0.009701624	0.027704287
8123562	2762	*GDP-mannose 4,6-dehydratase*	*GMDS*	8.60	8.06	9.23	0.009712629	0.027731568
8013364	146802	*solute carrier family 47, member 2*	*SLC47A2*	6.28	6.30	5.88	0.009716329	0.027739389

8000651	23049, 552900, 654483, 81893, 387254, 641298, 100271836, 595101, 440354	*SMG1 homolog, phosphatidylinositol 3-kinase-related kinase (C. elegans) | bolA homolog 2 (E. coli) | bolA homolog 2B (E. coli) | solute carrier family 7 (cationic amino acid transporter, y+ system), member 5 pseudogene 1 | solute carrier family 7 (cationic amino acid transporter, y+ system), member 5 pseudogene 2 | SMG1 homolog, phosphatidylinositol 3-kinase-related kinase pseudogene | PI-3-kinase-related kinase SMG-1 pseudogene*	*SMG1|BOLA2|BOLA2B|SLC7A5P1|SLC7A5P2|LOC641298|LOC100271836|LOC595101|LOC440354*	10.10	9.98	11.29	0.009718049	0.027741557
8139015				7.96	8.12	7.65	0.009720767	0.027746571
8117773	554223		*LOC554223*	7.47	7.43	6.85	0.009731748	0.027775168
7920191	353142	*late cornified envelope 3A*	*LCE3A*	7.69	7.71	7.39	0.009734648	0.027777955
8010915	359845	*family with sequence similarity 101, member B*	*FAM101B*	11.68	12.54	11.13	0.009734497	0.027777955
8173181	169981	*spindlin family, member 3*	*SPIN3*	7.20	7.30	8.29	0.009735731	0.027778296
8088895	692099	*family with sequence similarity 86, member D, pseudogene*	*FAM86DP*	8.40	9.26	8.40	0.009739261	0.027785623
8026390	126402	*coiled-coil domain containing 105*	*CCDC105*	7.13	7.26	6.70	0.009740393	0.027786106
8158177	79095	*chromosome 9 open reading frame 16*	*C9orf16*	9.20	9.11	8.44	0.009742633	0.027789753
7902909				7.05	6.92	6.52	0.009744591	0.02779259
7903703	2773	*guanine nucleotide binding protein (G protein), alpha inhibiting activity polypeptide 3*	*GNAI3*	8.91	8.85	9.85	0.009749509	0.027803743
8033460				6.67	6.32	6.03	0.009750428	0.027803743
7927964	5552	*serglycin*	*SRGN*	9.56	9.00	6.51	0.00975326	0.027809072
8168461				6.34	6.75	5.91	0.009760101	0.02782583
8074030	9701	*protein phosphatase 6, regulatory subunit 2*	*PPP6R2*	9.47	9.36	8.54	0.009764344	0.02783518
7976101	79882	*zinc finger CCCH-type containing 14*	*ZC3H14*	8.20	8.06	9.41	0.009774516	0.027861426
8056798	6670	*Sp3 transcription factor*	*SP3*	8.96	8.86	9.46	0.009778188	0.027863637
8172399	280660	*synovial sarcoma, X breakpoint 9*	*SSX9*	4.61	4.66	4.91	0.009777834	0.027863637
7933535				5.41	5.30	5.06	0.009777817	0.027863637

8015230	83755, 653240, 81871, 100132476	*keratin associated protein 4-12 | keratin associated protein 4-11 | keratin associated protein 4-6 | keratin associated protein 4-7*	*KRTAP4-12|KRTAP4-11|KRTAP4-6|KRTAP4-7*	8.42	8.79	7.83	0.009787338	0.027886957
8175802	11219	*three prime repair exonuclease 2*	*TREX2*	7.68	7.83	7.12	0.009788434	0.02788733
8174119	84460	*zinc finger, matrin-type 1*	*ZMAT1*	6.10	5.85	6.81	0.00978952	0.02788767
8065344	3170	*forkhead box A2*	*FOXA2*	7.38	7.44	6.79	0.009791763	0.027891306
8060660	116835	*heat shock 70kD protein 12B*	*HSPA12B*	7.90	7.79	7.34	0.009799012	0.02790645
8118235	259215, 58530	*lymphocyte antigen 6 complex, locus G6F | lymphocyte antigen 6 complex, locus G6D*	*LY6G6F|LY6G6D*	7.05	7.06	6.51	0.009798761	0.02790645
7975889	22846	*vasohibin 1*	*VASH1*	8.10	8.08	7.12	0.009800873	0.027908996
7951159				5.87	5.93	5.45	0.00980342	0.027911346
8078542				6.55	6.55	5.91	0.009803632	0.027911346
8147746				5.42	5.24	5.04	0.009812115	0.027932743
8012605	8522	*growth arrest-specific 7*	*GAS7*	8.82	8.50	7.90	0.009813899	0.027935067
7986092	5045	*furin (paired basic amino acid cleaving enzyme)*	*FURIN*	9.36	9.44	8.38	0.009820857	0.027952118
7936108	255022	*calcium homeostasis modulator 1*	*CALHM1*	6.77	6.88	6.36	0.009821987	0.027952576
8026638	4650	*myosin IXB*	*MYO9B*	9.73	9.93	8.90	0.009826904	0.027962165
8107942	10111		*RAD50*	8.72	8.27	9.93	0.009828262	0.027962165
8169859	54440	*SAM and SH3 domain containing 3*	*SASH3*	7.07	7.02	6.61	0.009827668	0.027962165
7939706	2147	*coagulation factor II (thrombin)*	*F2*	6.33	6.42	6.04	0.009830878	0.027966853
8141241	57154	*SMAD specific E3 ubiquitin protein ligase 1*	*SMURF1*	10.16	9.59	9.50	0.009834025	0.027973048
7984932	192683	*secretory carrier membrane protein 5*	*SCAMP5*	8.19	7.59	9.76	0.009837306	0.027976869
8106352				7.50	7.62	7.81	0.009837052	0.027976869
8076403	4668	*N-acetylgalactosaminidase, alpha-*	*NAGA*	10.32	9.74	8.60	0.00983996	0.027980357
7943562	55531	*ELMO/CED-12 domain containing 1*	*ELMOD1*	5.19	5.38	5.72	0.009840472	0.027980357
7956281	11318	*G protein-coupled receptor 182*	*GPR182*	6.76	6.74	6.29	0.009842756	0.027984098

8121794	10924	*sphingomyelin phosphodiesterase, acid-like 3A*	*SMPDL3A*	8.55	8.12	6.25	0.00984391	0.027984623
8179762	534, 7919	*ATPase, H+ transporting, lysosomal 13kDa, V1 subunit G2 | HLA-B associated transcript 1*	*ATP6V1G2 |BAT1*	8.63	7.93	8.08	0.009847106	0.02799095
7971243				6.73	6.60	6.54	0.009849562	0.027995177
8085283	115795, 2177	*chromosome 3 open reading frame 24 | Fanconi anemia, complementation group D2*	*C3orf24|FANCD2*	6.87	7.02	6.57	0.009850571	0.027995288
8014316	6352	*chemokine (C-C motif) ligand 5*	*CCL5*	6.51	6.43	6.08	0.009863156	0.02802002
8050658	54454	*ATPase family, AAA domain containing 2B*	*ATAD2B*	7.14	6.71	8.03	0.009861501	0.02802002
8036989				6.99	6.99	6.61	0.00986232	0.02802002
8082238				5.90	5.84	5.37	0.009862624	0.02802002
8161166	56656	*olfactory receptor, family 2, subfamily S, member 2*	*OR2S2*	7.28	7.47	6.95	0.00986423	0.028020313
7918394	79574	*EPS8-like 3*	*EPS8L3*	6.85	6.96	6.44	0.009866375	0.028023649
8057817				5.78	5.88	5.53	0.009870981	0.028033974
8015252	83902	*keratin associated protein 17-1*	*KRTAP17-1*	7.39	7.59	6.94	0.009874935	0.028040396
7979416	26520, 387990	*translocase of inner mitochondrial membrane 9 homolog (yeast) | translocase of outer mitochondrial membrane 20 homolog (yeast)-like*	*TIMM9|TOMM20L*	7.92	8.10	8.61	0.009875185	0.028040396
7966003	55198	*adaptor protein, phosphotyrosine interaction, PH domain and leucine zipper containing 2*	*APPL2*	9.43	10.28	8.58	0.009879	0.028048469
7936637	641451	*small nucleolar RNA, H/ACA box 19*	*SNORA19*	5.30	5.46	6.19	0.009881168	0.028050305
7911110				6.88	6.58	6.35	0.00988159	0.028050305
7905571	6280	*S100 calcium binding protein A9*	*S100A9*	6.62	6.82	6.43	0.009886419	0.028061256
8006445	6356	*chemokine (C-C motif) ligand 11*	*CCL11*	6.83	6.87	6.25	0.009894795	0.028082268
7925229	148789	*beta-1,3-N-acetylgalactosaminyltransferase 2*	*B3GALNT2*	8.21	8.24	9.32	0.009898438	0.028089847
8128522	57531	*HECT domain and ankyrin repeat containing, E3 ubiquitin protein ligase 1*	*HACE1*	8.09	7.65	9.49	0.009900204	0.028092096
7975863	55668	*chromosome 14 open reading frame 118*	*C14orf118*	8.65	8.85	10.28	0.009908583	0.028110534
8169156				5.60	5.87	5.11	0.009908649	0.028110534
7971669				5.35	5.36	6.06	0.009914781	0.028125165
8134091	9069	*claudin 12*	*CLDN12*	8.83	8.72	10.93	0.009920985	0.028134493

8090955	51146	*alpha-1,4-N-acetylglucosaminyltransferase*	*A4GNT*	5.61	5.52	5.22	0.009919766	0.028134493
8027429				5.73	5.56	6.69	0.009920993	0.028134493
7924760	3707	*inositol 1,4,5-trisphosphate 3-kinase B*	*ITPKB*	6.65	6.23	6.10	0.009922871	0.028137056
8169659	4694, 7737	*NADH dehydrogenase (ubiquinone) 1 alpha subcomplex, 1, 7.5kDa | ring finger protein 113A*	*NDUFA1|RNF113A*	8.78	8.79	9.21	0.009927685	0.028147941
8031145	79042	*tRNA splicing endonuclease 34 homolog (S. cerevisiae)*	*TSEN34*	8.93	9.05	8.20	0.009934074	0.028163292
7916526	732	*complement component 8, beta polypeptide*	*C8B*	5.48	5.59	5.31	0.009938063	0.028165042
7973105	6037	*ribonuclease, RNase A family, 3*	*RNASE3*	7.15	7.46	6.96	0.009938595	0.028165042
8015173	83896	*keratin associated protein 3-1*	*KRTAP3-1*	6.14	6.19	5.75	0.009937773	0.028165042
7919570				4.71	4.73	5.57	0.009935937	0.028165042
7906797	127943	*Fc receptor-like B*	*FCRLB*	7.36	7.53	6.99	0.009943347	0.028173944
7918550				6.21	6.18	5.80	0.009943687	0.028173944
8047561				4.38	4.40	4.34	0.009945047	0.028175032
7961386	55507	*G protein-coupled receptor, family C, group 5, member D*	*GPRC5D*	6.10	6.10	5.73	0.009955816	0.028202772
8147221	734	*oxidative stress induced growth inhibitor family member 2*	*OSGIN2*	7.17	6.76	7.90	0.009959739	0.028211117
7942715	4975	*olfactory marker protein*	*OMP*	7.62	7.65	7.22	0.009968095	0.028229412
7979367	10640	*exocyst complex component 5*	*EXOC5*	8.92	8.60	10.24	0.009968153	0.028229412
8173673	546	*alpha thalassemia/mental retardation syndrome X-linked*	*ATRX*	6.94	6.59	8.69	0.009971365	0.0282302
8107520	25816	*tumor necrosis factor, alpha-induced protein 8*	*TNFAIP8*	7.84	7.00	7.32	0.009970379	0.0282302
8006052	90313	*tumor protein p53 inducible protein 13*	*TP53I13*	8.33	8.48	7.55	0.009970771	0.0282302
8130916	55274	*PHD finger protein 10*	*PHF10*	8.42	8.29	9.04	0.009974931	0.028237527
8157283	9128	*PRP4 pre-mRNA processing factor 4 homolog (yeast)*	*PRPF4*	8.78	9.10	10.22	0.009977136	0.028240798
7916403	23648	*single stranded DNA binding protein 3*	*SSBP3*	10.65	10.44	11.08	0.009978044	0.028240798
8151512	55824	*phosphoprotein associated with glycosphingolipid microdomains 1*	*PAG1*	7.48	6.54	6.17	0.009979929	0.028242286
7973371	22985, 55017	*apoptotic chromatin condensation inducer 1 | chromosome 14 open reading frame 119*	*ACIN1|C14orf119*	9.95	10.17	10.65	0.009980526	0.028242286
8007197	28511	*NFKB inhibitor interacting Ras-like 2*	*NKIRAS2*	10.30	10.49	9.35	0.009990949	0.02826901

7966779	4842	*nitric oxide synthase 1 (neuronal)*	*NOS1*	6.41	6.54	6.87	0.009994323	0.028273016
8108301	10112, 8697	*kinesin family member 20A | cell division cycle 23 homolog (S. cerevisiae)*	*KIF20A|CDC23*	8.17	8.24	10.88	0.009993629	0.028273016
8007537	146894	*CD300 molecule-like family member g*	*CD300LG*	7.30	7.39	6.88	0.010002433	0.028293185
8083025	60467	*blepharophimosis, epicanthus inversus and ptosis, candidate 1 (non-protein coding)*	*BPESC1*	6.52	6.53	5.91	0.010007652	0.028305173
8139767				6.61	5.59	7.49	0.010011264	0.028312616
8095333	10640	*exocyst complex component 5*	*EXOC5*	7.07	6.88	8.28	0.010012956	0.028314628
7924309	2104	*estrogen-related receptor gamma*	*ESRRG*	5.58	5.63	6.04	0.01002243	0.028334204
8115851	8614	*stanniocalcin 2*	*STC2*	9.48	9.97	8.40	0.010023805	0.028334204
8083897	389177	*transmembrane protein 212*	*TMEM212*	5.42	5.57	5.30	0.010023485	0.028334204
8094154	391632	*olfactory receptor, family 7, subfamily E, member 35 pseudogene*	*OR7E35P*	7.02	7.11	6.48	0.010021551	0.028334204
8079138				5.53	5.61	5.43	0.010026247	0.028338334
8139411	222068	*transmembrane emp24 protein transport domain containing 4*	*TMED4*	11.71	11.60	11.14	0.010028222	0.028341139
7959220	35	*acyl-CoA dehydrogenase, C-2 to C-3 short chain*	*ACADS*	8.31	8.27	7.47	0.010037617	0.028362138
8136213				5.39	5.12	5.00	0.010036777	0.028362138
8076161	10126, 25777	*dynein, axonemal, light chain 4 | Sad1 and UNC84 domain containing 2*	*DNAL4|SUN2*	8.06	8.14	7.17	0.010038733	0.028362514
8033023	163154	*proline rich 22*	*PRR22*	7.59	7.58	7.01	0.01004383	0.028374137
8003903	409	*arrestin, beta 2*	*ARRB2*	8.95	8.62	10.17	0.01004487	0.028374301
8038952	90317	*zinc finger protein 616*	*ZNF616*	6.09	5.92	7.49	0.010050605	0.028387723
7957966	4604	*myosin binding protein C, slow type*	*MYBPC1*	5.57	5.65	5.33	0.010053674	0.028391853
7976598	10914	*poly(A) polymerase alpha*	*PAPOLA*	10.55	10.20	12.35	0.010055018	0.028391853
7985134	3419, 23205	*isocitrate dehydrogenase 3 (NAD+) alpha | acyl-CoA synthetase bubblegum family member 1*	*IDH3A|ACSBG1*	9.61	9.89	10.93	0.010054339	0.028391853
8092654	116832	*ribosomal protein L39-like*	*RPL39L*	7.03	7.08	8.18	0.010063404	0.02840241
8071301	128989	*chromosome 22 open reading frame 25*	*C22orf25*	9.12	9.27	8.19	0.010063883	0.02840241
7978795	140801	*ribosomal protein L10-like*	*RPL10L*	7.09	7.14	6.54	0.010060785	0.02840241
8021695	220164	*docking protein 6*	*DOK6*	6.58	6.70	5.69	0.010066026	0.02840241

8136391	346673	*stimulated by retinoic acid gene 8 homolog (mouse)*	*STRA8*	5.54	5.64	5.29	0.010064248	0.02840241
8010787	284004, 79415	*hexosaminidase (glycosyl hydrolase family 20, catalytic domain) containing | chromosome 17 open reading frame 62*	*HEXDC|C17orf62*	8.43	8.32	7.77	0.010066628	0.02840241
7989130				5.44	5.36	5.14	0.010065561	0.02840241
8121064				5.43	5.49	5.07	0.010065388	0.02840241
8066266	9935	*v-maf musculoaponeurotic fibrosarcoma oncogene homolog B (avian)*	*MAFB*	7.32	7.63	6.81	0.010074295	0.028418679
8035023	26993	*A kinase (PRKA) anchor protein 8-like*	*AKAP8L*	10.05	9.79	10.65	0.010075347	0.028418679
7980142	646658	*transmembrane protein 90A*	*TMEM90A*	6.51	6.48	6.10	0.010075338	0.028418679
8126566	221424	*chromosome 6 open reading frame 154*	*C6orf154*	6.84	6.98	6.24	0.010079483	0.028427567
7931062	118663	*BTB (POZ) domain containing 16*	*BTBD16*	6.03	6.11	5.63	0.010085812	0.02844264
8121216	59336	*PR domain containing 13*	*PRDM13*	7.50	7.64	7.06	0.01009274	0.028459398
8037991	3978	*ligase I, DNA, ATP-dependent*	*LIG1*	7.66	7.84	8.76	0.010097257	0.028469354
8018251	283985	*fatty acid desaturase domain family, member 6*	*FADS6*	7.33	7.46	6.88	0.010099794	0.028473726
8157444	619556	*microRNA 455*	*MIR455*	5.73	5.79	5.37	0.010104254	0.028483517
7907430	356	*Fas ligand (TNF superfamily, member 6)*	*FASLG*	6.24	6.31	5.85	0.010105519	0.028484302
8133030	2551	*GA binding protein transcription factor, alpha subunit 60kDa*	*GABPA*	7.80	7.33	8.90	0.010109792	0.028485091
8067662	5753		*PTK6*	7.39	7.44	6.89	0.010113515	0.028485091
8126360	9477	*mediator complex subunit 20*	*MED20*	8.67	8.87	9.89	0.01010942	0.028485091
8177964	401250	*mitochondrial coiled-coil domain 1*	*MCCD1*	7.64	7.73	7.30	0.010111293	0.028485091
7985253	283687, 400410	*chromosome 15 open reading frame 37 | suppressor of tumorigenicity 20*	*C15orf37|ST20*	7.81	7.86	7.11	0.010113732	0.028485091
7905517	353137, 353135	*late cornified envelope 1F | late cornified envelope 1E*	*LCE1F|LCE1E*	8.13	8.27	7.70	0.010111978	0.028485091
8007584	92591, 339201	*ankyrin repeat and SOCS box-containing 16 | chromosome 17 open reading frame 65*	*ASB16|C17orf65*	8.05	8.06	7.45	0.010111134	0.028485091
8017104				5.43	5.43	7.50	0.010108745	0.028485091
8046426				8.41	8.17	7.71	0.010114679	0.028485091
8167592	9506	*P antigen family, member 4 (prostate associated)*	*PAGE4*	5.67	5.68	5.38	0.010116507	0.02848746

8092501	7066	*thrombopoietin*	*THPO*	7.14	7.08	6.55	0.010117732	0.028488131
8064581	60493, 22888	*FAST kinase domains 5 | U-box domain containing 5*	*FASTKD5| UBOX5*	7.82	8.04	9.13	0.010118835	0.028488457
8039635	503835	*double homeobox A*	*DUXA*	4.95	4.99	4.87	0.010122426	0.028495789
7898102	23254, 200197	*kazrin | chromosome 1 open reading frame 126*	*KAZ|C1orf 126*	6.76	6.96	6.52	0.01013215	0.028520381
7976189	5700	*proteasome (prosome, macropain) 26S subunit, ATPase, 1*	*PSMC1*	9.22	9.49	10.35	0.010134188	0.028520843
8059177	7277	*tubulin, alpha 4a*	*TUBA4A*	8.77	9.18	10.47	0.010135715	0.028520843
8018774	55808	*ST6 (alpha-N-acetyl-neuraminyl-2,3-beta-galactosyl-1,3)-N-acetylgalactosaminide alpha-2,6-sialyltransferase 1*	*ST6GALN AC1*	6.66	6.73	6.37	0.010136044	0.028520843
7951091				7.37	7.63	6.70	0.010136448	0.028520843
8066818				4.45	4.52	4.40	0.010137253	0.028520843
8160392	3449	*interferon, alpha 16*	*IFNA16*	4.61	4.62	4.51	0.010139461	0.028521493
8069150	386676	*keratin associated protein 10-9*	*KRTAP10-9*	8.61	8.41	7.52	0.010139147	0.028521493
8018305	51155	*hematological and neurological expressed 1*	*HN1*	10.08	10.34	11.64	0.010144173	0.028531969
8012344	84667	*hairy and enhancer of split 7 (Drosophila)*	*HES7*	7.42	7.60	6.84	0.010148192	0.028540492
7943162	28970	*chromosome 11 open reading frame 54*	*C11orf54*	7.80	7.87	9.57	0.010159677	0.02857001
8070386	257357	*non-protein coding RNA 257*	*NCRNA00 257*	5.90	6.07	5.57	0.010169678	0.028595349
8051637	90957	*DEAH (Asp-Glu-Ala-Asp/His) box polypeptide 57*	*DHX57*	7.20	7.04	8.85	0.010173196	0.028602453
8047782				5.00	5.09	4.65	0.010175824	0.028607057
7972808				7.34	7.49	6.92	0.010185223	0.028630693
8165453	389816, 123803	*leucine rich repeat containing 26 | N-terminal asparagine amidase*	*LRRC26|N TAN1*	8.82	8.92	8.12	0.010187639	0.028634695
8170360	2495	*ferritin, heavy polypeptide 1*	*FTH1*	13.82	13.82	13.51	0.010192455	0.028644672
7945158				8.50	8.62	7.83	0.010193173	0.028644672
8037152	2931	*glycogen synthase kinase 3 alpha*	*GSK3A*	11.42	11.42	10.73	0.010194521	0.02864567
7900167	55143	*cell division cycle associated 8*	*CDCA8*	7.78	8.25	9.74	0.01019769	0.028651787
7973444				6.18	6.07	5.67	0.010201923	0.02866089

8006345	55288	*ras homolog gene family, member T1*	*RHOT1*	8.82	8.76	9.77	0.010203606	0.02866283
8002379				7.18	6.97	6.47	0.010207559	0.028671144
7987325	9716	*aquarius homolog (mouse)*	*AQR*	9.26	8.91	11.25	0.010214157	0.028678516
8054731	27177	*interleukin 1 family, member 8 (eta)*	*IL1F8*	5.56	5.61	5.34	0.010213641	0.028678516
8013860	399687, 9220	*myosin XVIIIA | TGFB1-induced anti-apoptotic factor 1*	*MYO18A|TIAF1*	8.77	8.48	7.70	0.01021366	0.028678516
8120783	4646, 23598	*myosin VI | POZ (BTB) and AT hook containing zinc finger 1*	*MYO6|PATZ1*	7.83	7.36	9.15	0.010214151	0.028678516
8037301	27076	*LY6/PLAUR domain containing 3*	*LYPD3*	7.97	7.74	7.58	0.010216157	0.028681342
7916853	9077	*DIRAS family, GTP-binding RAS-like 3*	*DIRAS3*	7.99	7.14	6.18	0.010220512	0.0286882
8024956	374875	*hydroxysteroid (11-beta) dehydrogenase 1-like*	*HSD11B1L*	7.68	7.74	7.19	0.010220587	0.0286882
7903119	5825	*ATP-binding cassette, sub-family D (ALD), member 3*	*ABCD3*	8.03	7.72	9.01	0.010223087	0.028692426
8027437				6.60	7.06	6.24	0.010229458	0.028707517
7918813	4893, 7812	*neuroblastoma RAS viral (v-ras) oncogene homolog | cold shock domain containing E1, RNA-binding*	*NRAS|CSDE1*	10.29	10.56	12.05	0.010232382	0.028712932
8035612	100271849, 4207, 729991	*myocyte enhancer factor 2B | LOC729991-MEF2B readthrough transcript | hypothetical protein LOC729991*	*MEF2B|LOC729991-MEF2B*	9.32	9.39	8.46	0.010243149	0.02874035
8004571	1949	*ephrin-B3*	*EFNB3*	7.67	7.16	7.37	0.010244285	0.028740743
8045349	4249	*mannosyl (alpha-1,6-)-glycoprotein beta-1,6-N-acetyl-glucosaminyltransferase*	*MGAT5*	8.75	7.99	9.01	0.010245774	0.028742129
8003875	124976	*spinster homolog 2 (Drosophila)*	*SPNS2*	7.74	7.82	7.38	0.010247579	0.0287444
8002347	16	*alanyl-tRNA synthetase*	*AARS*	11.43	11.41	11.77	0.010251344	0.028749375
8021169	9388	*lipase, endothelial*	*LIPG*	6.21	6.37	6.86	0.010251066	0.028749375
8065576	2307	*forkhead box S1*	*FOXS1*	7.70	7.79	7.15	0.010254737	0.028752847
7963328	401720	*fidgetin-like 2*	*FIGNL2*	6.97	6.95	7.87	0.010255571	0.028752847
8125857				6.73	6.02	7.63	0.010254441	0.028752847
7914834	5690	*proteasome (prosome, macropain) subunit, beta type, 2*	*PSMB2*	11.63	11.67	12.09	0.010259038	0.028759776
7948192	6749	*structure specific recognition protein 1*	*SSRP1*	9.36	9.48	10.22	0.010260523	0.028761147
7930378				5.87	5.90	5.60	0.010261993	0.028762473

7898377	9696	*ciliary rootlet coiled-coil, rootletin*	*CROCC*	7.36	7.27	6.79	0.010266467	0.028772047
8149646	79873	*nudix (nucleoside diphosphate linked moiety X)-type motif 18*	*NUDT18*	7.74	7.68	7.22	0.010267402	0.028772047
7954055	81575	*apolipoprotein L domain containing 1*	*APOLD1*	7.74	7.24	6.62	0.010269345	0.028774697
7905581	6271, 6284	*S100 calcium binding protein A1 | S100 calcium binding protein A13*	*S100A1|S100A13*	6.21	6.24	5.98	0.01027129	0.028777357
7906642	126823	*kelch domain containing 9*	*KLHDC9*	6.61	6.52	6.94	0.010273118	0.028779684
7963366	3891	*keratin 85*	*KRT85*	6.88	6.97	6.38	0.010274491	0.028780738
7908496	3080	*complement factor H-related 2*	*CFHR2*	4.87	4.72	4.58	0.010275591	0.028781027
8138922	25948	*kelch repeat and BTB (POZ) domain containing 2*	*KBTBD2*	7.57	8.10	8.56	0.010276642	0.028781177
8067877	150000	*ATP-binding cassette, sub-family C (CFTR/MRP), member 13, pseudogene*	*ABCC13*	5.09	5.13	5.00	0.010282228	0.028794028
7906764	3310	*heat shock 70kDa protein 6 (HSP70B')*	*HSPA6*	5.92	6.02	6.30	0.010289698	0.028812151
7926983	1390	*cAMP responsive element modulator*	*CREM*	6.37	6.41	6.66	0.010292511	0.028814439
8021091	284266	*sialic acid binding Ig-like lectin 15*	*SIGLEC15*	7.41	7.54	6.99	0.010291624	0.028814439
8113504	9315	*chromosome 5 open reading frame 13*	*C5orf13*	11.57	12.39	12.60	0.010294812	0.028815292
7910950	8564, 23596	*kynurenine 3-monooxygenase (kynurenine 3-hydroxylase) | opsin 3*	*KMO|OPN3*	4.79	4.74	4.64	0.01029382	0.028815292
7995340				9.58	9.82	9.10	0.010296951	0.028818486
8005685				5.66	5.76	5.39	0.010298773	0.02882079
7898211	84301, 6248	*DNA-damage inducible 1 homolog 2 (S. cerevisiae) | regulatory solute carrier protein, family 1, member 1*	*DDI2|RSC1A1*	7.87	7.90	9.52	0.010299949	0.028821288
7965431	574016	*chronic lymphocytic leukemia up-regulated 1 opposite strand*	*CLLU1OS*	5.99	6.00	5.68	0.010304494	0.02883121
8154622	3445	*interferon, alpha 8*	*IFNA8*	5.35	5.46	5.17	0.010309164	0.028841484
8071504	400891	*chromosome 14 open reading frame 166B pseudogene*	*LOC400891*	5.87	6.08	5.56	0.010316907	0.028858613
8042825				7.11	7.09	6.98	0.010317287	0.028858613
7923317	647219	*achaete-scute complex homolog 5 (Drosophila)*	*ASCL5*	6.80	7.15	6.32	0.010319885	0.028863084
8131195	155185	*archaelysin family metallopeptidase 1*	*AMZ1*	7.85	8.19	7.25	0.010323712	0.02887099
7912764	374955	*spermatogenesis associated 21*	*SPATA21*	6.69	6.83	6.31	0.010334629	0.028896071
7909780				6.14	6.23	5.86	0.010334682	0.028896071

8170753	26576	*SRSF protein kinase 3*	*SRPK3*	7.44	7.54	7.19	0.010338509	0.028901173
8095562				5.35	5.35	4.93	0.010338452	0.028901173
8155487	387628, 2252, 654466	*keratinocyte growth factor-like protein 1 | fibroblast growth factor 7 | keratinocyte growth factor-like protein 2*	*KGFLP1|FGF7|KGFLP2*	7.96	8.59	6.00	0.010347229	0.028922748
7942453	58473	*pleckstrin homology domain containing, family B (evectins) member 1*	*PLEKHB1*	6.76	6.85	7.49	0.010351423	0.028931672
8161455	387628, 654466, 2252	*keratinocyte growth factor-like protein 1 | keratinocyte growth factor-like protein 2 | fibroblast growth factor 7*	*KGFLP1|KGFLP2|FGF7*	7.95	8.58	6.00	0.010353765	0.028935418
8177976	4049	*lymphotoxin alpha (TNF superfamily, member 1)*	*LTA*	7.02	7.07	6.56	0.010357776	0.02894102
8012006	162515	*solute carrier family 16, member 11 (monocarboxylic acid transporter 11)*	*SLC16A11*	7.30	7.25	6.69	0.010357574	0.02894102
7932512	64215	*DnaJ (Hsp40) homolog, subfamily C, member 1*	*DNAJC1*	9.65	10.15	9.08	0.010361293	0.028948048
7968516	23047	*PDS5, regulator of cohesion maintenance, homolog B (S. cerevisiae)*	*PDS5B*	8.39	7.38	9.17	0.010365082	0.02895583
8047443	55437	*STE20-related kinase adaptor beta*	*STRADB*	10.74	10.50	11.04	0.010366738	0.028957654
7957604				8.93	9.06	8.56	0.010367787	0.028957784
8026954	5296	*phosphoinositide-3-kinase, regulatory subunit 2 (beta)*	*PIK3R2*	10.21	9.88	8.68	0.01037195	0.02896661
8067277	407022	*microRNA 296*	*MIR296*	7.15	7.14	6.79	0.010375376	0.028973373
7970546	221154	*EF-hand domain family, member A1*	*EFHA1*	6.91	6.87	8.17	0.010386331	0.02900116
8083777				7.00	7.02	6.63	0.010396113	0.029025665
8024934	148066	*zinc and ring finger 4*	*ZNRF4*	6.93	7.13	6.52	0.010403848	0.029044453
8133904	2913	*glutamate receptor, metabotropic 3*	*GRM3*	5.66	5.58	6.42	0.010410925	0.029056044
7973022	122742	*olfactory receptor, family 4, subfamily L, member 1*	*OR4L1*	5.51	5.44	5.21	0.010410765	0.029056044
8173752	203430	*zinc finger, CCHC domain containing 5*	*ZCCHC5*	5.33	5.48	5.01	0.010411019	0.029056044
8075569	6527	*solute carrier family 5 (low affinity glucose cotransporter), member 4*	*SLC5A4*	5.70	5.73	5.43	0.010414074	0.02906176
8057034	8575	*protein kinase, interferon-inducible double stranded RNA dependent activator*	*PRKRA*	9.07	9.39	10.14	0.010428664	0.029099662
8140170	84163, 389524	*GTF2I repeat domain containing 2 | GTF2I repeat domain containing 2B*	*GTF2IRD2|GTF2IRD2B*	8.16	8.09	6.47	0.01043508	0.029114751

8109254	6949	*Treacher Collins-Franceschetti syndrome 1*	*TCOF1*	8.32	8.46	9.36	0.010440783	0.029127846
8151238				6.06	6.26	5.57	0.010443958	0.029133892
8124865	170680	*psoriasis susceptibility 1 candidate 2*	*PSORS1C2*	7.41	7.38	6.97	0.010448157	0.029142786
8049876	666	*BCL2-related ovarian killer*	*BOK*	9.67	10.57	8.87	0.010452977	0.029153416
7945652	387266	*keratin associated protein 5-3*	*KRTAP5-3*	8.84	8.89	8.55	0.010456066	0.029156413
7932508				5.00	4.84	4.66	0.010456072	0.029156413
7991566	55180	*lines homolog (Drosophila)*	*LINS*	6.51	6.45	7.22	0.010458415	0.02916013
7995336				9.99	10.05	9.32	0.010462046	0.029167436
7971482				6.04	6.07	5.77	0.010466213	0.029176237
8161423	387628, 654466, 2252	*keratinocyte growth factor-like protein 1 | keratinocyte growth factor-like protein 2 | fibroblast growth factor 7*	*KGFLP1|KGFLP2|FGF7*	7.95	8.57	6.00	0.010469647	0.029182991
7924069				7.72	7.39	8.12	0.010472604	0.029188415
8017102				6.04	5.95	5.61	0.01047547	0.029190766
8164153				6.97	6.82	7.78	0.010475031	0.029190766
7955852	3228	*homeobox C12*	*HOXC12*	6.70	6.88	6.29	0.010481131	0.029192962
8173366	5030	*pyrimidinergic receptor P2Y, G-protein coupled, 4*	*P2RY4*	6.87	6.86	6.39	0.010479704	0.029192962
7898052	645425, 391001	*PRAME family member 20 | PRAME family member 21*	*PRAMEF20|PRAMEF21*	7.08	7.24	6.66	0.010480294	0.029192962
8106763				4.58	4.53	4.46	0.0104777	0.029192962
8154719				4.80	4.81	5.11	0.010481314	0.029192962
8035551	84757		*MGC10814*	7.57	7.80	7.15	0.010482731	0.029194092
8163629	944	*tumor necrosis factor (ligand) superfamily, member 8*	*TNFSF8*	6.38	6.26	5.77	0.010484001	0.029194811
8170196				5.95	6.09	5.45	0.0104851	0.029195055
7975324	57452	*UDP-N-acetyl-alpha-D-galactosamine:polypeptide N-acetylgalactosaminyltransferase-like 1*	*GALNTL1*	8.63	7.99	7.46	0.010490345	0.029206844
8118259	4439, 401251	*mutS homolog 5 (E. coli) | chromosome 6 open reading frame 26*	*MSH5|C6orf26*	7.17	7.14	7.98	0.010496854	0.029222148
8015737				8.59	8.67	7.93	0.010504627	0.029240968

8069676	9510	*ADAM metallopeptidase with thrombospondin type 1 motif, 1*	*ADAMTS1*	10.15	10.23	7.50	0.010515105	0.029261671
8061305	55857	*polo-like kinase 1 substrate 1*	*PLK1S1*	7.81	7.69	9.13	0.010513522	0.029261671
7923394	127829	*ADP-ribosylation factor-like 8A*	*ARL8A*	11.19	11.22	10.59	0.010514289	0.029261671
7992049	84264	*hydroxyacylglutathione hydrolase-like*	*HAGHL*	8.42	8.63	7.88	0.010520678	0.029274357
8114113	54819	*zinc finger, CCHC domain containing 10*	*ZCCHC10*	6.75	7.11	7.50	0.010529586	0.029293498
8033179	284427	*solute carrier family 25, member 41*	*SLC25A41*	6.53	6.51	6.18	0.010529246	0.029293498
8019930	103910	*myosin, light chain 12B, regulatory*	*MYL12B*	12.69	12.74	12.44	0.010533433	0.02929991
8090511	28976, 100132731, 57501	*acyl-CoA dehydrogenase family, member 9 | hypothetical protein LOC100132731 | KIAA1257*	*ACAD9|LOC100132731|KIAA1257*	5.99	6.01	5.69	0.010533921	0.02929991
8162194	23552	*cyclin-dependent kinase 20*	*CDK20*	8.01	8.05	6.91	0.01053586	0.029302482
7951363	120329	*caspase 12 (gene/pseudogene)*	*CASP12*	5.23	5.21	5.08	0.010540286	0.029311966
7976080	8477	*G protein-coupled receptor 65*	*GPR65*	5.01	4.88	4.72	0.010544959	0.029321829
7904048				5.40	5.19	6.54	0.010545864	0.029321829
8103951	2180	*acyl-CoA synthetase long-chain family member 1*	*ACSL1*	8.12	8.36	6.89	0.010559903	0.029341081
8126436	5961	*peripherin 2 (retinal degeneration, slow)*	*PRPH2*	7.57	6.76	6.20	0.010557054	0.029341081
7914847	127703	*chromosome 1 open reading frame 216*	*C1orf216*	7.50	7.49	6.87	0.010559784	0.029341081
8128638	256380	*sex comb on midleg-like 4 (Drosophila)*	*SCML4*	6.40	6.55	6.10	0.010558517	0.029341081
8100699	401135	*synaptotagmin XIV-like*	*SYT14L*	5.48	5.30	5.76	0.010554668	0.029341081
8062339	25939, 140711	*SAM domain and HD domain 1 | chromosome 20 open reading frame 118*	*SAMHD1|C20orf118*	7.33	7.16	6.79	0.010555666	0.029341081
8122142	594837, 594839	*small nucleolar RNA, C/D box 101 | small nucleolar RNA, H/ACA box 33*	*SNORD101|SNORA33*	5.56	5.46	6.35	0.010559267	0.029341081
7985233	5923	*Ras protein-specific guanine nucleotide-releasing factor 1*	*RASGRF1*	6.58	6.77	6.33	0.010561146	0.02934171
8021668	1005	*cadherin 7, type 2*	*CDH7*	5.26	5.39	5.61	0.010572654	0.029370857
8135568	29969	*MyoD family inhibitor domain containing*	*MDFIC*	10.11	9.36	8.39	0.010574747	0.029373845
8018600	6730	*signal recognition particle 68kDa*	*SRP68*	9.43	9.66	10.49	0.01057685	0.029376859

8121784	2173	*fatty acid binding protein 7, brain*	*FABP7*	5.83	5.89	6.53	0.010582141	0.0293859
8071168	29797, 646074, 29774, 25812	*POM121 membrane glycoprotein-like 8 pseudogene | POM121 membrane glycoprotein-like 10, pseudogene | POM121 membrane glycoprotein-like 9, pseudogene | POM121 membrane glycoprotein-like 1, pseudogene*	*POM121L8P|POM121L10P|POM121L9P|POM121L1P*	7.11	7.09	6.48	0.010581798	0.0293859
8093440	53834	*fibroblast growth factor receptor-like 1*	*FGFRL1*	9.43	10.21	8.84	0.010585495	0.029392388
8085999	54918	*CKLF-like MARVEL transmembrane domain containing 6*	*CMTM6*	7.99	7.80	9.10	0.010588763	0.029398635
7935180	9124	*PDZ and LIM domain 1*	*PDLIM1*	12.26	11.45	12.28	0.010591613	0.02940372
7921900	117157	*SH2 domain containing 1B*	*SH2D1B*	5.33	5.55	5.11	0.010595417	0.02941145
7973983				5.58	5.58	5.41	0.01059668	0.02941213
7979125				4.85	4.86	4.66	0.010599444	0.02941697
8067242	140731	*ankyrin repeat domain 60*	*ANKRD60*	7.47	7.52	6.95	0.010601933	0.029421052
7921434	9447	*absent in melanoma 2*	*AIM2*	4.97	4.92	5.12	0.010606572	0.029431004
7942830	360219	*GCRG-P224*	*GCRG224*	5.90	5.80	5.43	0.010607558	0.029431004
7918153				7.23	7.24	6.73	0.010608746	0.02943147
8088047	389125, 375346	*musculoskeletal, embryonic nuclear protein 1 | transmembrane protein 110*	*MUSTN1|TMEM110*	7.60	7.11	6.80	0.010618239	0.029454976
8167027	9104	*regucalcin (senescence marker protein-30)*	*RGN*	6.83	6.46	6.03	0.01062364	0.029462079
8037755	388555	*IGF-like family member 3*	*IGFL3*	6.00	6.14	5.84	0.010623861	0.029462079
8144687	613209	*defensin, beta 135*	*DEFB135*	4.85	4.88	4.66	0.010622146	0.029462079
8047467	65061	*cyclin-dependent kinase 15*	*CDK15*	8.05	7.62	5.71	0.01062665	0.029465584
8024467	360200, 26517	*transmembrane protease, serine 9 | translocase of inner mitochondrial membrane 13 homolog (yeast)*	*TMPRSS9|TIMM13*	7.00	7.08	6.61	0.010627166	0.029465584
8060082	643905	*proline rich 21*	*PRR21*	6.51	6.55	6.13	0.010629579	0.029469442
8093278	3081	*homogentisate 1,2-dioxygenase*	*HGD*	5.36	5.53	5.21	0.010634825	0.029478325
7909236	9261	*mitogen-activated protein kinase-activated protein kinase 2*	*MAPKAPK2*	9.79	10.09	9.22	0.010633828	0.029478325
8030804	945		*CD33*	7.02	7.05	6.59	0.010643855	0.02949769

8160377	3441, 3451, 3446, 3444	*interferon, alpha 4 | interferon, alpha 17 | interferon, alpha 10 | interferon, alpha 7*	*IFNA4|IFNA17|IFNA10|IFNA7*	5.30	5.20	5.00	0.010643405	0.02949769
7930775				4.55	4.47	4.41	0.010648061	0.029506514
7995161	3681	*integrin, alpha D*	*ITGAD*	6.87	6.92	6.50	0.010651263	0.029510282
8039859	3805, 57292	*killer cell immunoglobulin-like receptor, two domains, long cytoplasmic tail, 4 | killer cell immunoglobulin-like receptor, two domains, long cytoplasmic tail, 5A*	*KIR2DL4|KIR2DL5A*	5.95	5.94	5.63	0.010651465	0.029510282
7949046	83707, 83706	*tRNA phosphotransferase 1 | fermitin family member 3*	*TRPT1|FERMT3*	8.68	8.74	7.95	0.010667703	0.029552436
7980636	161436	*echinoderm microtubule associated protein like 5*	*EML5*	6.68	5.80	5.86	0.010676128	0.029572936
8086419	57467	*hedgehog acyltransferase-like*	*HHATL*	6.69	6.70	6.26	0.010679432	0.029573577
7946004	119692	*olfactory receptor, family 51, subfamily S, member 1*	*OR51S1*	7.34	7.27	6.74	0.010678593	0.029573577
7996651	388284	*chromosome 16 open reading frame 86*	*C16orf86*	7.82	7.81	7.24	0.010677897	0.029573577
8153373	1585	*cytochrome P450, family 11, subfamily B, polypeptide 2*	*CYP11B2*	6.84	7.07	6.42	0.010687885	0.029591305
7992893				6.19	6.60	8.42	0.010687004	0.029591305
7973110	6036	*ribonuclease, RNase A family, 2 (liver, eosinophil-derived neurotoxin)*	*RNASE2*	5.08	5.06	4.90	0.010691226	0.029597716
7909144	284581		*LOC284581*	6.85	6.65	6.21	0.010692633	0.029598774
8005699	25979	*dehydrogenase/reductase (SDR family) member 7B*	*DHRS7B*	9.10	9.56	8.68	0.010694649	0.029601172
8137906	55698	*Ras association and DIL domains*	*RADIL*	7.13	7.16	6.57	0.01069555	0.029601172
8079407	9034	*chemokine (C-C motif) receptor-like 2*	*CCRL2*	5.13	5.12	4.90	0.010711717	0.029643074
8060792	643406		*LOC643406*	6.35	6.49	6.01	0.01071956	0.029661937
7998634	64711	*heparan sulfate (glucosamine) 3-O-sulfotransferase 6*	*HS3ST6*	8.59	8.76	7.87	0.010722755	0.029667934
8176821	9083, 442867, 442868	*basic charge, Y-linked, 2 | basic charge, Y-linked, 2B | basic charge, Y-linked, 2C*	*BPY2|BPY2B|BPY2C*	5.55	5.55	5.31	0.010724753	0.029670618
8111101	56172	*ankylosis, progressive homolog (mouse)*	*ANKH*	9.93	9.43	8.41	0.010727269	0.029674733

8163023	8518	*inhibitor of kappa light polypeptide gene enhancer in B-cells, kinase complex-associated protein*	*IKBKAP*	8.21	8.29	9.90	0.010729408	0.029677808
8066273				5.05	5.08	4.95	0.01073048	0.02967793
8113299				5.19	5.10	5.63	0.01073204	0.0296794
7916185	23318	*zinc finger, CCHC domain containing 11*	*ZCCHC11*	7.99	7.75	9.46	0.010737668	0.029692119
8148194				5.64	5.31	5.27	0.010750745	0.029725432
7952488				6.51	6.41	5.98	0.010758305	0.029743489
7934531				5.75	5.75	6.71	0.010759654	0.029744368
8036676	115290, 54938	*F-box protein 17 | seryl-tRNA synthetase 2, mitochondrial*	*FBXO17|SARS2*	10.06	10.11	9.04	0.010761183	0.029745746
8075921	114794	*extracellular leucine-rich repeat and fibronectin type III domain containing 2*	*ELFN2*	6.90	7.26	6.45	0.010768862	0.029764123
8143188	64764	*cAMP responsive element binding protein 3-like 2*	*CREB3L2*	10.64	10.51	9.35	0.010770505	0.029765816
7920217	6706, 6702	*small proline-rich protein 2G | small proline-rich protein 2C (pseudogene)*	*SPRR2G|SPRR2C*	7.71	7.73	7.06	0.010774354	0.0297736
7950372				5.56	5.55	5.27	0.01077822	0.029781437
8019988	5797	*protein tyrosine phosphatase, receptor type, M*	*PTPRM*	11.00	10.45	8.58	0.010784997	0.029796273
8010050	283991	*family with sequence similarity 100, member B*	*FAM100B*	9.59	9.89	8.90	0.010785654	0.029796273
7896822	375790	*agrin*	*AGRN*	9.31	9.39	8.57	0.010787354	0.029798117
8044965	7247	*translin*	*TSN*	7.03	7.35	9.06	0.01078919	0.029800337
8122840	2099	*estrogen receptor 1*	*ESR1*	5.94	5.95	5.65	0.010791034	0.029802578
8157036				5.48	5.52	5.27	0.010793283	0.029805938
8064351	1457, 283106	*casein kinase 2, alpha 1 polypeptide | casein kinase 2, alpha 1 polypeptide pseudogene*	*CSNK2A1|CSNK2A1P*	10.31	10.50	12.02	0.010798207	0.029816683
8020717	147429	*chromosome 18 open reading frame 16*	*C18orf16*	5.33	5.53	5.04	0.01080237	0.029822474
8132803	168448	*CDC14 cell division cycle 14 homolog C (S. cerevisiae)*	*CDC14C*	5.42	5.39	4.85	0.010801837	0.029822474
8114245	4762	*neurogenin 1*	*NEUROG1*	6.96	7.05	6.42	0.010805795	0.029829077
7904717	148738	*hemochromatosis type 2 (juvenile)*	*HFE2*	6.31	6.38	5.80	0.01080765	0.029831346

8069855	337978	*keratin associated protein 21-2*	*KRTAP21-2*	5.69	5.65	5.39	0.010812183	0.029841004
8144874	100131275	*nervous system abundant protein 11*	*NSAP11*	5.63	5.76	5.31	0.010813458	0.02984167
8071361	729444, 729461, 26080, 653203	*hypothetical LOC729444 | hypothetical LOC729461 | non-protein coding RNA 281 | similar to hypothetical gene supported by BC039313*	*LOC729444|LOC729461|NCRNA00281|LOC653203*	5.69	5.85	5.40	0.010815648	0.029844863
8117608	8332	*histone cluster 1, H2al*	*HIST1H2AL*	7.24	7.95	9.68	0.010818283	0.02984928
8066964	387521, 387522, 7335	*transmembrane protein 189 | TMEM189-UBE2V1 readthrough | ubiquitin-conjugating enzyme E2 variant 1*	*TMEM189|TMEM189-UBE2V1*	9.59	9.84	9.16	0.010819398	0.029849503
8167951				4.75	4.78	4.54	0.010828678	0.02987225
8070579	7031	*trefoil factor 1*	*TFF1*	7.12	7.26	6.55	0.010830625	0.029874766
8178063	259215, 58530	*lymphocyte antigen 6 complex, locus G6F | lymphocyte antigen 6 complex, locus G6D*	*LY6G6F|LY6G6D*	7.04	7.05	6.51	0.010832616	0.029877406
8010212	147138	*transmembrane channel-like 8*	*TMC8*	7.42	7.57	7.12	0.010833735	0.029877635
7898939	57185	*NIPA-like domain containing 3*	*NIPAL3*	8.88	8.77	8.12	0.01083586	0.029877787
8057416				4.97	4.92	4.84	0.010835147	0.029877787
7898556	54896	*PQ loop repeat containing 2*	*PQLC2*	8.82	8.95	7.93	0.010837672	0.029878775
7897026	163688	*calmodulin-like 6*	*CALML6*	6.41	6.58	6.15	0.010838288	0.029878775
8011011	7531, 5048	*tyrosine 3-monooxygenase/tryptophan 5-monooxygenase activation protein, epsilon polypeptide | platelet-activating factor acetylhydrolase 1b, regulatory subunit 1 (45kDa)*	*YWHAE|PAFAH1B1*	11.84	12.07	12.77	0.010845115	0.029894743
7906993				7.70	7.62	7.34	0.010850772	0.02990748
7913226	26279	*phospholipase A2, group IID*	*PLA2G2D*	7.51	7.61	7.09	0.010852089	0.029908255
8040630				5.17	4.93	6.32	0.010854451	0.029911907
7917468				6.75	7.18	6.32	0.010856127	0.029913671
8014882	84299	*chromosome 17 open reading frame 37*	*C17orf37*	10.57	10.71	9.50	0.010865332	0.029930463
8172369	7569, 57232	*zinc finger protein 182 | zinc finger protein 630*	*ZNF182|ZNF630*	6.96	6.76	7.28	0.010864557	0.029930463

7904967				8.28	8.59	9.98	0.010863796	0.029930463
7998407	390664	*C1q and tumor necrosis factor related protein 8*	*C1QTNF8*	7.07	7.06	6.61	0.010872127	0.029945146
8031346	3805, 57292	*killer cell immunoglobulin-like receptor, two domains, long cytoplasmic tail, 4 | killer cell immunoglobulin-like receptor, two domains, long cytoplasmic tail, 5A*	*KIR2DL4|KIR2DL5A*	6.06	6.06	5.75	0.010872737	0.029945146
7970945				5.46	5.45	5.02	0.010874716	0.029947741
8031726	57343	*zinc finger protein 304*	*ZNF304*	6.73	6.93	8.07	0.010876928	0.029950975
8076209	6122	*ribosomal protein L3*	*RPL3*	13.36	13.33	13.62	0.010896892	0.030001633
8004184	54739	*XIAP associated factor 1*	*XAF1*	8.32	8.46	6.17	0.010897403	0.030001633
8160419	3447, 3439	*interferon, alpha 13 | interferon, alpha 1*	*IFNA13*	5.26	5.12	4.91	0.01090229	0.030012226
7899829	64766, 8565	*S100P binding protein | tyrosyl-tRNA synthetase*	*S100PBP|YARS*	7.96	8.03	9.42	0.010911497	0.030034708
7976065				5.12	4.99	4.78	0.010918701	0.030051671
7973770	23256	*sec1 family domain containing 1*	*SCFD1*	8.45	8.24	9.32	0.010930578	0.030081494
8079079	4820	*natural killer-tumor recognition sequence*	*NKTR*	7.36	6.88	8.82	0.010934423	0.030089205
7967420	55206	*strawberry notch homolog 1 (Drosophila)*	*SBNO1*	7.82	7.35	9.99	0.010938326	0.030097077
7905085	333932, 126961, 653604	*histone cluster 2, H3a | histone cluster 2, H3c | histone cluster 2, H3d*	*HIST2H3A|HIST2H3C|HIST2H3D*	10.06	10.68	12.16	0.010940791	0.030098123
8062555				6.26	5.93	6.63	0.01094035	0.030098123
7942489	374407	*DnaJ (Hsp40) homolog, subfamily B, member 13*	*DNAJB13*	6.26	6.35	5.65	0.010946291	0.030107513
8175747	4102, 4105	*melanoma antigen family A, 3 | melanoma antigen family A, 6*	*MAGEA3|MAGEA6*	6.65	6.72	6.29	0.01094535	0.030107513
7945132	2313, 2130	*Friend leukemia virus integration 1 | Ewing sarcoma breakpoint region 1*	*FLI1|EWSR1*	7.89	7.99	6.47	0.010948411	0.030110478
7982927	3706	*inositol 1,4,5-trisphosphate 3-kinase A*	*ITPKA*	7.43	7.56	6.81	0.010956335	0.030129043
7938299				6.23	6.21	5.81	0.010958291	0.030129043
8160906				5.25	5.26	5.09	0.010958293	0.030129043
8124388	8358	*histone cluster 1, H3b*	*HIST1H3B*	9.04	10.29	12.37	0.010980064	0.03018599
8027416	8725	*chromosome 19 open reading frame 2*	*C19orf2*	10.04	10.31	10.81	0.010981096	0.03018599

7985620	57538	*alpha-kinase 3*	*ALPK3*	6.75	6.91	7.46	0.010982734	0.030187618
8056005	90	*activin A receptor, type I*	*ACVR1*	9.08	9.41	8.12	0.010987431	0.030191166
8148902	2875	*glutamic-pyruvate transaminase (alanine aminotransferase)*	*GPT*	6.96	7.23	6.56	0.01098569	0.030191166
8014115	4642	*myosin ID*	*MYO1D*	9.62	10.87	9.25	0.010987529	0.030191166
7963689	8620	*neuropeptide FF-amide peptide precursor*	*NPFF*	6.76	6.67	6.18	0.010988208	0.030191166
8096897				6.12	5.95	5.60	0.010994613	0.030203015
8148147				6.19	6.11	5.63	0.010994162	0.030203015
7944335	643	*chemokine (C-X-C motif) receptor 5*	*CXCR5*	6.52	6.45	5.82	0.01100073	0.030216943
8037408	3783	*potassium intermediate/small conductance calcium-activated channel, subfamily N, member 4*	*KCNN4*	6.81	7.12	6.48	0.011008124	0.030234376
8082473	2815	*glycoprotein IX (platelet)*	*GP9*	7.36	7.39	6.86	0.011013079	0.030242233
8018220	2905	*glutamate receptor, ionotropic, N-methyl D-aspartate 2C*	*GRIN2C*	7.10	7.15	6.73	0.011012135	0.030242233
8033248	8740	*tumor necrosis factor (ligand) superfamily, member 14*	*TNFSF14*	7.36	7.32	6.76	0.011015895	0.030247087
8084755	407020	*microRNA 28*	*MIR28*	4.81	4.82	4.66	0.011018548	0.030250471
8136065	407835	*mitogen-activated protein kinase kinase 2 pseudogene*	*LOC407835*	8.56	8.59	8.04	0.011019223	0.030250471
7925504	440738	*microtubule-associated protein 1 light chain 3 gamma*	*MAP1LC3C*	7.58	7.35	6.86	0.011023543	0.030257262
7966148				4.91	4.73	4.50	0.011023793	0.030257262
7953252	26290	*UDP-N-acetyl-alpha-D-galactosamine:polypeptide N-acetylgalactosaminyltransferase 8 (GalNAc-T8)*	*GALNT8*	5.79	5.84	6.41	0.01102687	0.03026283
8067295	51012	*slowmo homolog 2 (Drosophila)*	*SLMO2*	8.46	8.73	9.05	0.011030205	0.030266227
8072355	284904	*SEC14-like 4 (S. cerevisiae)*	*SEC14L4*	5.73	5.69	5.33	0.011030072	0.030266227
8072744	4689	*neutrophil cytosolic factor 4, 40kDa*	*NCF4*	6.60	6.54	6.18	0.01103874	0.03028677
8044080	6549	*solute carrier family 9 (sodium/hydrogen exchanger), member 2*	*SLC9A2*	5.38	5.38	5.25	0.011041206	0.030290658
8062766	4605	*v-myb myeloblastosis viral oncogene homolog (avian)-like 2*	*MYBL2*	8.07	8.73	10.95	0.011042574	0.030291531
8025402	51129	*angiopoietin-like 4*	*ANGPTL4*	7.30	8.33	6.84	0.011050432	0.030310206
7904969	81552	*vesicular, overexpressed in cancer, prosurvival protein 1*	*VOPP1*	10.35	10.84	9.85	0.011056468	0.03031812

7930994	404216	*chromosome 10 open reading frame 85*	*C10orf85*	6.81	6.97	6.55	0.011056342	0.03031812
7997502				6.43	6.37	5.99	0.011054403	0.03031812
8086341	165904	*xin actin-binding repeat containing 1*	*XIRP1*	6.95	7.06	6.58	0.011060296	0.030325737
7951807	23705, 100132764	*cell adhesion molecule 1 | hypothetical LOC100132764*	*CADM1|LOC100132764*	7.34	8.26	8.43	0.011071584	0.030353805
8008922	8493	*protein phosphatase, Mg2+/Mn2+ dependent, 1D*	*PPM1D*	8.74	8.65	9.53	0.011075228	0.03035803
7939158	79832	*glutamine and serine rich 1*	*QSER1*	9.00	8.61	11.21	0.011074773	0.03035803
8072124	1413	*crystallin, beta A4*	*CRYBA4*	6.85	6.93	6.56	0.011079249	0.030366167
7897426	765	*carbonic anhydrase VI*	*CA6*	6.10	6.31	5.92	0.011085602	0.030380696
8061605	85366	*myosin light chain kinase 2*	*MYLK2*	6.77	6.86	6.26	0.011088794	0.03038656
7922152	57821	*chromosome 1 open reading frame 114*	*C1orf114*	5.83	5.81	6.20	0.011095144	0.030398188
8154043	58524	*doublesex and mab-3 related transcription factor 3*	*DMRT3*	6.74	6.87	6.53	0.011094951	0.030398188
8171180				7.49	7.44	7.02	0.011107237	0.030428434
8052908	165530	*C-type lectin domain family 4, member F*	*CLEC4F*	6.30	6.42	5.98	0.011109852	0.030432709
8091764	3840	*karyopherin alpha 4 (importin alpha 3)*	*KPNA4*	8.60	8.57	10.14	0.011112087	0.030432863
7965979	160428	*aldehyde dehydrogenase 1 family, member L2*	*ALDH1L2*	9.95	9.87	7.27	0.011112025	0.030432863
7917370	255631	*collagen, type XXIV, alpha 1*	*COL24A1*	6.28	6.34	5.93	0.011113071	0.030432863
8159624	375791	*chromosome 9 open reading frame 169*	*C9orf169*	7.77	7.86	7.32	0.011116366	0.030438999
7984008				5.66	5.79	6.21	0.011117557	0.030439373
8158666	256158	*hemicentin 2*	*HMCN2*	7.51	7.58	7.13	0.011121288	0.0304467
8163273	26248	*olfactory receptor, family 2, subfamily K, member 2*	*OR2K2*	5.72	5.74	5.44	0.011123782	0.030450642
8170803	8269	*transmembrane protein 187*	*TMEM187*	7.62	7.52	6.88	0.011124955	0.030450964
8102350				6.80	7.00	7.02	0.011128771	0.030458523
7908879	8497, 100507405	*protein tyrosine phosphatase, receptor type, f polypeptide (PTPRF), interacting protein (liprin), alpha 4 | liprin-alpha-1-like*	*PPFIA4|LOC100507405*	6.77	6.96	6.35	0.011133594	0.030468833
8027398	79156	*pleckstrin homology domain containing, family F (with FYVE domain) member 1*	*PLEKHF1*	8.64	8.93	7.73	0.011141031	0.030486297
7941662	89	*actinin, alpha 3*	*ACTN3*	7.10	7.27	8.61	0.011148065	0.030502653

7906613	57823	*SLAM family member 7*	*SLAMF7*	5.09	5.02	4.70	0.011156983	0.030521268
7903414	279, 276, 277, 278, 280	*amylase, alpha 2A (pancreatic) | amylase, alpha 1A (salivary) | amylase, alpha 1B (salivary) | amylase, alpha 1C (salivary) | amylase, alpha 2B (pancreatic)*	*AMY2A|AMY1A|AMY1B|AMY1C|AMY2B*	5.63	6.11	5.48	0.011156241	0.030521268
8033662	284382	*actin-like 9*	*ACTL9*	7.75	7.83	7.25	0.011159211	0.030521577
7919614	333932, 126961, 653604	*histone cluster 2, H3a | histone cluster 2, H3c | histone cluster 2, H3d*	*HIST2H3A|HIST2H3C|HIST2H3D*	10.07	10.69	12.16	0.011159016	0.030521577
8003722	63826, 55720	*serine racemase | TSR1, 20S rRNA accumulation, homolog (S. cerevisiae)*	*SRR|TSR1*	9.14	9.33	7.95	0.011168015	0.030542765
8153486	100130274	*coiled-coil domain containing 121-like*	*LOC100130274*	7.24	7.48	6.88	0.011172053	0.030550916
7929653	26287	*ankyrin repeat domain 2 (stretch responsive muscle)*	*ANKRD2*	7.45	7.54	6.94	0.011179638	0.03056876
8089701	26137	*zinc finger and BTB domain containing 20*	*ZBTB20*	8.92	8.34	6.59	0.011184786	0.03057994
7902883	55144	*leucine rich repeat containing 8 family, member D*	*LRRC8D*	9.03	9.70	11.01	0.011187274	0.030583845
8139712	81552	*vesicular, overexpressed in cancer, prosurvival protein 1*	*VOPP1*	9.99	10.47	9.48	0.011200224	0.03061635
7997832	339059		*FLJ40448*	7.10	7.22	6.77	0.011202133	0.03061867
8169636				5.42	5.29	5.01	0.011203268	0.03061887
7918323	6272	*sortilin 1*	*SORT1*	9.55	9.44	11.10	0.011207208	0.03062674
8162631	22869	*zinc finger protein 510*	*ZNF510*	7.30	7.04	8.43	0.011210186	0.030631978
8018377				7.08	7.04	6.47	0.011216555	0.03064648
8076511	6125	*ribosomal protein L5*	*RPL5*	13.16	13.14	13.63	0.011221837	0.030658012
7930750	374355	*chromosome 10 open reading frame 96*	*C10orf96*	4.94	4.89	5.50	0.011226517	0.030667895
8062695	6431	*serine/arginine-rich splicing factor 6*	*SRSF6*	9.72	9.65	10.83	0.011229908	0.030674256
7972840	10426	*tubulin, gamma complex associated protein 3*	*TUBGCP3*	7.97	8.02	8.98	0.011237653	0.030688025
7927028	219770	*gap junction protein, delta 4, 40.1kDa*	*GJD4*	6.62	6.81	6.28	0.011238046	0.030688025
7998351	401827, 724032	*mesothelin-like | microRNA 662*	*MSLNL|MIR662*	8.32	8.46	7.81	0.011238862	0.030688025
8095543				4.77	4.92	4.66	0.011239201	0.030688025

7943293	23052	*endonuclease domain containing 1*	*ENDOD1*	7.57	7.69	6.71	0.011242907	0.030690864
7997135	92154	*metastasis suppressor 1-like*	*MTSS1L*	7.84	7.97	7.33	0.011243531	0.030690864
7997861				6.18	6.20	5.63	0.011244493	0.030690864
8101061				7.09	7.37	7.72	0.011241717	0.030690864
8032214	2593	*guanidinoacetate N-methyltransferase*	*GAMT*	10.01	9.75	8.99	0.011261596	0.030728715
7987163	342184, 100506959	*formin 1 | formin-1-like*	*FMN1|LOC100506959*	6.15	6.39	5.51	0.011262012	0.030728715
8023708				6.14	6.09	5.53	0.011260685	0.030728715
8045347				5.42	5.22	6.03	0.011262618	0.030728715
7991283	51458	*Rh family, C glycoprotein*	*RHCG*	6.29	6.32	5.99	0.011267893	0.030739931
8121193	114792	*kelch-like 32 (Drosophila)*	*KLHL32*	5.13	5.27	5.31	0.011269666	0.030739931
8104838	134218	*DnaJ (Hsp40) homolog, subfamily C, member 21*	*DNAJC21*	8.14	7.97	9.22	0.011269924	0.030739931
7917739				6.87	7.97	5.66	0.011275242	0.030751532
8168733				6.10	6.22	5.74	0.011283682	0.030771643
8049123	250	*alkaline phosphatase, placental*	*ALPP*	6.06	6.26	5.77	0.01128657	0.030776521
7926819				5.11	5.21	4.97	0.011288669	0.030776521
8039769				6.68	6.88	6.24	0.011288289	0.030776521
7913682	3155	*3-hydroxymethyl-3-methylglutaryl-CoA lyase*	*HMGCL*	8.61	8.52	7.61	0.011294102	0.030788427
7960464	7450	*von Willebrand factor*	*VWF*	6.36	6.31	5.99	0.011298719	0.030798104
8132399	57002	*chromosome 7 open reading frame 36*	*C7orf36*	9.65	9.63	10.51	0.011304052	0.03080973
8044154	926	*CD8b molecule*	*CD8B*	6.50	6.61	6.16	0.011307607	0.030816512
8131803	3569	*interleukin 6 (interferon, beta 2)*	*IL6*	8.87	9.36	6.20	0.011331738	0.03087936
8167305	10682	*emopamil binding protein (sterol isomerase)*	*EBP*	8.14	7.93	9.42	0.011334361	0.030880792
8055967				5.87	5.82	5.66	0.011335473	0.030880792
8143142				4.45	4.42	4.36	0.011334679	0.030880792
8121749	2697	*gap junction protein, alpha 1, 43kDa*	*GJA1*	10.29	10.79	12.83	0.01134767	0.030905738
8048940	6672		*SP100*	8.21	7.99	5.58	0.011347842	0.030905738
8118147				7.15	7.20	6.77	0.011346758	0.030905738

8129043				5.11	5.26	4.95	0.011362185	0.030941883
8102730	256471	*major facilitator superfamily domain containing 8*	*MFSD8*	7.63	7.65	8.89	0.011367622	0.030950852
7995342				8.69	8.89	8.09	0.011367161	0.030950852
7952384	219873	*olfactory receptor, family 10, subfamily S, member 1*	*OR10S1*	6.64	6.52	6.23	0.011369061	0.030951025
8001064	317702	*vomeronasal 1 receptor 3 (gene/pseudogene)*	*VN1R3*	5.72	5.62	5.34	0.01136983	0.030951025
8050278	10130	*protein disulfide isomerase family A, member 6*	*PDIA6*	10.99	11.16	12.32	0.01138789	0.030997159
7998374	51764	*guanine nucleotide binding protein (G protein), gamma 13*	*GNG13*	7.47	7.64	7.06	0.011388925	0.030997159
8048445	7475	*wingless-type MMTV integration site family, member 6*	*WNT6*	8.09	8.21	7.65	0.011397435	0.031014474
8131292	57786	*RB-associated KRAB zinc finger*	*RBAK*	7.11	6.68	7.98	0.011396391	0.031014474
7905848	55891	*lens epithelial protein*	*LENEP*	7.12	7.15	6.78	0.011402471	0.031025253
8089986				5.25	5.18	4.96	0.011408691	0.031039253
7999360	6144, 10013,1205	*ribosomal protein L21 | ribosomal protein L21 pseudogene 28*	*RPL21|RP L21P28*	10.38	10.55	10.86	0.011413554	0.031049555
8169158	55086	*chromosome X open reading frame 57*	*CXorf57*	4.94	5.04	5.41	0.011421351	0.031067839
7941917	57010, 390212	*calcium binding protein 4 | G protein-coupled receptor 152*	*CABP4|GP R152*	7.80	7.84	7.30	0.011422563	0.03106821
7920600	6464	*SHC (Src homology 2 domain containing) transforming protein 1*	*SHC1*	11.82	11.66	11.01	0.011426192	0.031072224
7946128	84067	*family with sequence similarity 160, member A2*	*FAM160A2*	8.56	8.67	8.00	0.011425852	0.031072224
7995348				10.09	10.10	9.42	0.011427335	0.031072404
8156770	79695	*UDP-N-acetyl-alpha-D-galactosamine:polypeptide N-acetylgalactosaminyltransferase 12 (GalNAc-T12)*	*GALNT12*	8.11	7.69	9.57	0.01142956	0.03107553
7978653	253959	*Ral GTPase activating protein, alpha subunit 1 (catalytic)*	*RALGAPA 1*	7.70	7.16	9.27	0.011432362	0.03108022
8175319	7626	*zinc finger protein 75D*	*ZNF75D*	6.28	6.22	7.17	0.011438357	0.031090684
8100530				5.57	5.80	5.03	0.011438365	0.031090684
7939977	219473	*olfactory receptor, family 8, subfamily K, member 3*	*OR8K3*	5.10	5.24	5.05	0.011448908	0.031113587
8043018				6.42	6.40	6.14	0.011448947	0.031113587

7904853	653519, 51463, 728932	*G protein-coupled receptor 89A | G protein-coupled receptor 89B | G protein-coupled receptor 89C*	*GPR89A|GPR89B|GPR89C*	7.40	7.41	8.75	0.011451054	0.031116387
8037032	374907	*UDP-GlcNAc:betaGal beta-1,3-N-acetylglucosaminyltransferase 8*	*B3GNT8*	7.74	7.69	6.95	0.011452973	0.03111867
8040614				6.47	6.48	6.12	0.011465234	0.031149052
8064495	5173	*prodynorphin*	*PDYN*	5.90	6.11	5.79	0.011467484	0.031149304
8040486				6.98	7.18	6.68	0.011466607	0.031149304
8077270	10752	*cell adhesion molecule with homology to L1CAM (close homolog of L1)*	*CHL1*	5.42	5.37	5.17	0.011470656	0.031152057
8059475				6.80	6.91	6.33	0.011469587	0.031152057
8074593	85376, 440804, 150221	*RIMS binding protein 3 | RIMS binding protein 3B | RIMS binding protein 3C*	*RIMBP3|RIMBP3B|RIMBP3C*	7.33	7.22	6.68	0.011478907	0.031171532
8018673	84074	*glutamine rich 2*	*QRICH2*	7.29	7.45	6.54	0.011481612	0.031175945
7919625	337873, 8349	*histone cluster 2, H2bc | histone cluster 2, H2be*	*HIST2H2BC|HIST2H2BE*	11.32	11.56	12.32	0.011483209	0.031177351
7981320				6.39	6.54	5.90	0.011487193	0.031185234
8058024	23451	*splicing factor 3b, subunit 1, 155kDa*	*SF3B1*	9.82	9.23	11.35	0.011490357	0.03119089
8004804	5198	*phosphoribosylformylglycinamidine synthase*	*PFAS*	8.25	8.50	9.85	0.011491626	0.031191401
7939007	338645	*leucine zipper protein 2*	*LUZP2*	6.50	6.53	7.78	0.011493831	0.031194357
8150159				4.55	4.53	4.78	0.011494876	0.031194357
8072636	91464	*intestine-specific homeobox*	*ISX*	6.86	7.09	6.62	0.011499658	0.031200977
7927383	118738	*zinc finger protein 488*	*ZNF488*	6.94	6.87	7.26	0.011500558	0.031200977
7947228				5.03	5.12	4.65	0.011499669	0.031200977
8155530	654466, 387628, 2252, 403323	*keratinocyte growth factor-like protein 2 | keratinocyte growth factor-like protein 1 | fibroblast growth factor 7 | hypothetical protein FLJ20444*	*KGFLP2|KGFLP1|FGF7|FLJ20444*	6.91	7.20	6.35	0.011502492	0.031203294
7922523				7.51	7.92	8.42	0.011513019	0.031228915
8002904	23536	*adenosine deaminase, tRNA-specific 1*	*ADAT1*	7.66	7.96	8.99	0.011518251	0.031240173

7957746	5250	*solute carrier family 25 (mitochondrial carrier; phosphate carrier), member 3*	*SLC25A3*	11.80	11.69	11.98	0.011520186	0.031242486
8068305	6453	*intersectin 1 (SH3 domain protein)*	*ITSN1*	8.33	8.40	9.52	0.011522927	0.031246984
8146643	406908	*microRNA 124-2*	*MIR124-2*	6.83	6.90	6.24	0.011524684	0.031248812
8075971	129138	*ankyrin repeat domain 54*	*ANKRD54*	8.56	8.78	7.57	0.011527426	0.031250376
8131301				5.36	5.22	5.04	0.011526397	0.031250376
8115997	53917		*RAB24*	9.48	9.44	8.71	0.011530754	0.031254742
7978260	115817	*dehydrogenase/reductase (SDR family) member 1*	*DHRS1*	9.32	9.51	8.34	0.011531201	0.031254742
8075709	80832	*apolipoprotein L, 4*	*APOL4*	6.70	6.30	6.06	0.011541347	0.031279303
8172254	79742	*chromosome X open reading frame 36*	*CXorf36*	5.66	5.72	5.45	0.011549369	0.031298105
8093298	1234	*chemokine (C-C motif) receptor 5*	*CCR5*	5.94	5.99	5.61	0.011551445	0.031300794
7952599				5.16	5.12	4.89	0.01155348	0.031303372
8094028	84740, 60312	*AFAP1 antisense RNA (non-protein coding) | actin filament associated protein 1*	*AFAP1-AS*	6.32	6.48	5.93	0.01155661	0.031308915
7956910	55832	*cullin-associated and neddylation-dissociated 1*	*CAND1*	9.21	8.65	10.66	0.011565822	0.03132994
8102875				6.39	6.57	6.96	0.011566542	0.03132994
7927505	282966, 1103	*chromosome 10 open reading frame 53 | choline O-acetyltransferase*	*C10orf53| CHAT*	6.29	6.31	6.00	0.011570965	0.03133898
7921677	51744		*CD244*	5.70	5.74	5.40	0.011573011	0.031341583
8018579	2125	*envoplakin*	*EVPL*	7.78	7.73	7.41	0.011574258	0.03134202
7967072	84274	*coenzyme Q5 homolog, methyltransferase (S. cerevisiae)*	*COQ5*	9.12	9.17	10.12	0.01157535	0.031342033
7909225	8444	*dual-specificity tyrosine-(Y)-phosphorylation regulated kinase 3*	*DYRK3*	7.40	7.88	7.08	0.0115813	0.031355206
8123372	1235	*chemokine (C-C motif) receptor 6*	*CCR6*	7.64	7.80	7.26	0.011588755	0.0313721
8031084	59283, 100126325	*calcium channel, voltage-dependent, gamma subunit 8 | microRNA 935*	*CACNG8| MIR935*	8.65	8.78	8.15	0.011589714	0.0313721
7976957	91833	*WD repeat domain 20*	*WDR20*	7.21	7.33	7.60	0.011595967	0.031386085
8117172	154150	*hepatoma derived growth factor-like 1*	*HDGFL1*	7.11	7.06	6.72	0.01160776	0.031415056
7976846	574436	*microRNA 485*	*MIR485*	7.16	6.74	6.47	0.011617367	0.031438112
8112570	23107	*mitochondrial ribosomal protein S27*	*MRPS27*	10.29	9.80	11.07	0.011631108	0.031472344

8029884	10055	*SUMO1 activating enzyme subunit 1*	*SAE1*	10.55	10.57	11.56	0.011633793	0.03147371
8148003	169026	*solute carrier family 30 (zinc transporter), member 8*	*SLC30A8*	5.85	5.80	5.60	0.011632893	0.03147371
8049375	339766	*HEAT repeat containing 7B1*	*HEATR7B 1*	6.72	6.66	6.36	0.011639132	0.031485204
8156098	389761, 389762, 347127	*family with sequence similarity 75, member D4 | family with sequence similarity 75, member D3 | FAM75-like protein FLJ46321 pseudogene*	*FAM75D4| FAM75D3| FAM75D5*	5.38	5.33	5.19	0.011641295	0.031488106
7901253	374973	*chromosome 1 open reading frame 223*	*C1orf223*	5.48	5.53	5.24	0.01165485	0.031519007
8062204				6.75	6.76	6.32	0.011654903	0.031519007
8133273				10.05	9.91	9.46	0.011667979	0.031551413
8063476	4159	*melanocortin 3 receptor*	*MC3R*	6.42	6.37	6.01	0.011673895	0.03156445
8137352	6522	*solute carrier family 4, anion exchanger, member 2 (erythrocyte membrane protein band 3-like 1)*	*SLC4A2*	9.45	9.66	8.73	0.01167779	0.031572025
8130087	85313	*peptidylprolyl isomerase (cyclophilin)-like 4*	*PPIL4*	8.13	7.96	9.11	0.011680003	0.031575054
8103630	57630	*SH3 domain containing ring finger 1*	*SH3RF1*	8.75	9.67	8.56	0.011681614	0.031576455
7945521	347862	*Parkinson disease 7 domain containing 1*	*PDDC1*	9.74	9.62	8.43	0.011687945	0.031590607
8023220	4092	*SMAD family member 7*	*SMAD7*	7.75	8.37	7.67	0.011693418	0.03159061
8159354	51162	*EGF-like-domain, multiple 7*	*EGFL7*	8.36	8.40	7.83	0.011690671	0.03159061
7966631	64211	*LIM homeobox 5*	*LHX5*	6.97	7.09	6.55	0.011693071	0.03159061
7914809	79932	*KIAA0319-like*	*KIAA0319 L*	9.51	9.34	9.84	0.011689807	0.03159061
8082246				8.05	8.11	7.46	0.011691659	0.03159061
8176867	9083, 442867, 442868	*basic charge, Y-linked, 2 | basic charge, Y-linked, 2B | basic charge, Y-linked, 2C*	*BPY2|BPY 2B|BPY2C*	5.56	5.55	5.32	0.011696532	0.031593114
8110461				7.06	6.95	6.68	0.011696475	0.031593114

8015221	653240, 100132386, 83755, 728224, 100132476, 85289	*keratin associated protein 4-11 | keratin associated protein 4-9 | keratin associated protein 4-12 | keratin associated protein 4-8 | keratin associated protein 4-7 | keratin associated protein 4-5*	*KRTAP4-11|KRTAP4-9|KRTAP4-12|KRTAP4-8|KRTAP4-7|KRTAP4-5*	8.53	8.70	7.86	0.011716851	0.031645034
8095504	4589	*mucin 7, secreted*	*MUC7*	6.12	5.93	5.62	0.011720136	0.031650946
8024637	51343	*fizzy/cell division cycle 20 related 1 (Drosophila)*	*FZR1*	9.16	9.16	8.02	0.011725123	0.03166145
8094030	60312	*actin filament associated protein 1*	*AFAP1*	7.60	7.60	7.11	0.01172626	0.03166156
8145766	3084, 100507358	*neuregulin 1 | hypothetical protein LOC100507358*	*NRG1|LOC100507358*	7.02	8.85	5.99	0.011731471	0.031672668
7989617				5.88	6.05	5.43	0.011733507	0.0316752
7957404	84190	*chromosome 12 open reading frame 26*	*C12orf26*	7.30	7.34	7.76	0.011739126	0.031684447
8121525	91749		*KIAA1919*	7.37	7.62	8.78	0.011738761	0.031684447
7961891	79365	*basic helix-loop-helix family, member e41*	*BHLHE41*	7.70	8.34	7.35	0.011743479	0.03169323
8068788	53347	*ubiquitin associated and SH3 domain containing A*	*UBASH3A*	5.62	5.52	5.26	0.011752087	0.031713497
8037246	5675	*pregnancy specific beta-1-glycoprotein 6*	*PSG6*	8.25	7.88	7.01	0.011754463	0.031716943
8004372	8711	*tyrosine kinase, non-receptor, 1*	*TNK1*	7.57	7.32	7.11	0.01176238	0.031732373
8055273				4.55	4.50	4.45	0.011762114	0.031732373
8157454	9550	*ATPase, H+ transporting, lysosomal 13kDa, V1 subunit G1*	*ATP6V1G1*	10.00	10.18	10.80	0.011769995	0.031749953
8083063	55186	*solute carrier family 25, member 36*	*SLC25A36*	8.64	8.37	9.90	0.011772387	0.031753436
8082974	83850	*extended synaptotagmin-like protein 3*	*ESYT3*	6.12	6.33	6.98	0.011778375	0.031760685
7941863				7.14	7.24	6.63	0.01177688	0.031760685
8139158				7.38	7.51	6.94	0.011777286	0.031760685
8008237	3675	*integrin, alpha 3 (antigen CD49C, alpha 3 subunit of VLA-3 receptor)*	*ITGA3*	9.60	9.84	8.31	0.011787412	0.03177615

8106722	92270	*ATPase, H+ transporting, lysosomal accessory protein 1-like*	*ATP6AP1L*	7.27	7.48	7.07	0.011786081	0.03177615
7940177	390197	*olfactory receptor, family 4, subfamily D, member 10*	*OR4D10*	5.68	5.81	5.36	0.011787077	0.03177615
8109901	2299	*forkhead box I1*	*FOXI1*	7.20	7.28	6.76	0.011795851	0.031795934
8067839	387628, 2252	*keratinocyte growth factor-like protein 1 | fibroblast growth factor 7*	*KGFLP1|FGF7*	8.08	8.72	6.18	0.011798208	0.031799313
8034276	115950	*zinc finger protein 653*	*ZNF653*	8.43	8.61	7.92	0.011805203	0.031815197
8112033	54622	*ADP-ribosylation factor-like 15*	*ARL15*	8.46	7.77	8.91	0.011813683	0.031835083
7935639	81894	*solute carrier family 25, member 28*	*SLC25A28*	9.08	9.14	8.29	0.011815543	0.03183712
7948834	10629	*TAF6-like RNA polymerase II, p300/CBP-associated factor (PCAF)-associated factor, 65kDa*	*TAF6L*	7.53	7.62	7.01	0.011818402	0.031837367
8070847	84536	*chromosome 21 open reading frame 67*	*C21orf67*	7.72	7.92	6.75	0.01181689	0.031837367
8036589				4.50	4.55	4.69	0.011818943	0.031837367
7931500	84435	*G protein-coupled receptor 123*	*GPR123*	7.19	7.15	6.73	0.011821527	0.031841356
8148559	51337	*chromosome 8 open reading frame 55*	*C8orf55*	8.72	8.29	7.32	0.011825337	0.031845678
7982060	100036564		*HBII-52-27*	4.49	4.51	4.39	0.011824334	0.031845678
8099524	9079	*LIM domain binding 2*	*LDB2*	10.30	8.74	9.65	0.011828746	0.031851884
8032137	91304	*chromosome 19 open reading frame 6*	*C19orf6*	11.31	11.27	10.42	0.011834734	0.031865038
7971422	23091	*zinc finger CCCH-type containing 13*	*ZC3H13*	7.57	7.59	9.43	0.011838379	0.03187041
8061539	140875	*non-protein coding RNA 28*	*NCRNA00028*	6.10	6.04	5.75	0.011838937	0.03187041
7936727	6144, 10013,1205	*ribosomal protein L21 | ribosomal protein L21 pseudogene 28*	*RPL21|RPL21P28*	11.02	11.15	11.38	0.011840601	0.03187192
8133549	389524, 84163	*GTF2I repeat domain containing 2B | GTF2I repeat domain containing 2*	*GTF2IRD2B*	8.24	8.11	6.37	0.011842487	0.031874023
8117402	8367	*histone cluster 1, H4e*	*HIST1H4E*	9.65	9.72	10.61	0.011857979	0.031908765
8095456	54959	*odontogenic, ameloblast asssociated*	*ODAM*	4.75	4.75	4.64	0.011859817	0.031908765
8012931	374286, 10517	*CMT1A duplicated region transcript 1 | F-box and WD repeat domain containing 10*	*CDRT1|FBXW10*	6.88	6.66	6.04	0.011859618	0.031908765
8076382				5.87	5.92	5.52	0.011858897	0.031908765
7911108				7.33	7.21	7.76	0.01186474	0.031919036

7925511	200150	*phospholipase D family, member 5*	*PLD5*	6.42	6.61	7.43	0.011873158	0.031938706
7946149	322	*amyloid beta (A4) precursor protein-binding, family B, member 1 (Fe65)*	*APBB1*	8.63	8.60	7.45	0.011886148	0.03196471
7958895	4940	*2'-5'-oligoadenylate synthetase 3, 100kDa*	*OAS3*	7.70	7.38	6.49	0.01188591	0.03196471
7996883	3038, 54921	*hyaluronan synthase 3 | CTF8, chromosome transmission fidelity factor 8 homolog (S. cerevisiae)*	*HAS3|CHTF8*	7.32	7.38	8.06	0.011885917	0.03196471
7926239	10133	*optineurin*	*OPTN*	9.22	9.04	7.76	0.011891009	0.031965036
8026679	55850	*unconventional SNARE in the ER 1 homolog (S. cerevisiae)*	*USE1*	9.57	9.71	8.20	0.011890477	0.031965036
8011343	84690	*spermatogenesis associated 22*	*SPATA22*	5.45	5.59	5.27	0.011888944	0.031965036
7910261	200205	*chromosome 1 open reading frame 69*	*C1orf69*	6.75	7.01	6.62	0.011891804	0.031965036
8133032				5.07	5.13	4.86	0.011888877	0.031965036
8001764	2806	*glutamic-oxaloacetic transaminase 2, mitochondrial (aspartate aminotransferase 2)*	*GOT2*	12.03	11.93	12.77	0.011898506	0.03197114
7949075	51504	*tRNA methyltransferase 11-2 homolog (S. cerevisiae)*	*TRMT112*	12.62	12.52	13.32	0.011895915	0.03197114
7960383	57097	*poly (ADP-ribose) polymerase family, member 11*	*PARP11*	6.17	6.08	7.01	0.011898068	0.03197114
8054354				7.51	7.55	6.90	0.011898361	0.03197114
8018196	146722	*CD300 molecule-like family member f*	*CD300LF*	6.71	6.64	6.21	0.011903085	0.03198047
8083792				8.93	9.08	9.55	0.011906553	0.03198681
8103341	79884	*microtubule-associated protein 9*	*MAP9*	5.75	5.38	6.36	0.011907763	0.031987086
8084035	51193	*zinc finger protein 639*	*ZNF639*	7.01	7.03	7.96	0.011909159	0.03198786
8079590	820	*cathelicidin antimicrobial peptide*	*CAMP*	6.74	6.74	6.38	0.011912532	0.03199222
8161133	26206, 4882	*sperm associated antigen 8 | natriuretic peptide receptor B/guanylate cyclase B (atrionatriuretic peptide receptor B)*	*SPAG8|NPR2*	6.45	6.51	6.01	0.011912999	0.03199222
7923662	5287	*phosphoinositide-3-kinase, class 2, beta polypeptide*	*PIK3C2B*	7.95	6.84	7.93	0.01191528	0.031995367
8065652	140732	*Sad1 and UNC84 domain containing 5*	*SUN5*	5.97	5.91	5.73	0.011917176	0.031997483
7897128	140625	*actin-related protein T2*	*ACTRT2*	7.24	7.36	6.72	0.011924013	0.032009885
7937089	282974	*serine/threonine kinase 32C*	*STK32C*	8.32	8.63	7.65	0.01192377	0.032009885
7980213	283571	*prospero homeobox 2*	*PROX2*	5.63	5.46	5.29	0.011928445	0.032018807
7900129	79729	*chromosome 1 open reading frame 113*	*C1orf113*	7.63	8.00	7.20	0.011934135	0.032031104

7963427	3852	*keratin 5*	*KRT5*	6.43	6.50	6.11	0.011937602	0.03203359
7901050	94162	*small nucleolar RNA, C/D box 38A*	*SNORD38 A*	6.40	6.41	7.31	0.011938389	0.03203359
8047709	117583	*par-3 partitioning defective 3 homolog B (C. elegans)*	*PARD3B*	8.90	7.79	7.43	0.011937093	0.03203359
8003249	79791	*F-box protein 31*	*FBXO31*	9.15	9.20	8.12	0.011940379	0.032035947
8166195	8233	*zinc finger (CCCH type), RNA-binding motif and serine/arginine rich 2*	*ZRSR2*	7.47	7.73	7.78	0.011948202	0.03203736
8093156	22916	*nuclear cap binding protein subunit 2, 20kDa*	*NCBP2*	7.42	7.26	8.85	0.011943529	0.03203736
7974902	57381	*ras homolog gene family, member J*	*RHOJ*	6.78	9.52	6.41	0.011948949	0.03203736
7969955	121952		*LOC12195 2*	5.97	5.76	5.47	0.011949224	0.03203736
8170488	139135	*PAS domain containing 1*	*PASD1*	5.20	5.22	5.01	0.011949783	0.03203736
8067521	140628	*GATA binding protein 5*	*GATA5*	8.73	8.81	8.21	0.011944688	0.03203736
7988763	388121	*tumor necrosis factor, alpha-induced protein 8-like 3*	*TNFAIP8L 3*	6.56	6.73	6.25	0.011947311	0.03203736
8060609				7.80	7.90	7.26	0.011947398	0.03203736
8089584	25871	*chromosome 3 open reading frame 17*	*C3orf17*	8.87	8.66	10.17	0.011952169	0.032040782
8041168	26796	*small nucleolar RNA, C/D box 53*	*SNORD53*	9.32	9.49	8.80	0.011964627	0.0320712
8025895	5589	*protein kinase C substrate 80K-H*	*PRKCSH*	12.60	12.55	11.86	0.011969087	0.032077197
8013533				8.03	8.12	7.61	0.011968345	0.032077197
8048763	57624		*KIAA1486*	6.04	6.17	5.75	0.011975907	0.03208654
8171026	474382, 83740, 474381	*H2A histone family, member B1 | H2A histone family, member B3 | H2A histone family, member B2*	*H2AFB1|H 2AFB3|H2 AFB2*	6.16	6.17	5.86	0.01197455	0.03208654
8163105				5.69	5.63	7.15	0.011975249	0.03208654
8161739	138240	*chromosome 9 open reading frame 57*	*C9orf57*	5.68	5.96	5.47	0.011980631	0.03209622
7951966	486	*FXYD domain containing ion transport regulator 2*	*FXYD2*	7.79	7.77	7.23	0.011986028	0.032107696
7928147	140766	*ADAM metallopeptidase with thrombospondin type 1 motif, 14*	*ADAMTS1 4*	7.87	7.43	6.69	0.011988536	0.032111432
7952386	79490	*olfactory receptor, family 10, subfamily G, member 6*	*OR10G6*	6.55	6.47	6.12	0.012005052	0.03215269
8094393	29063	*zinc finger, CCHC domain containing 4*	*ZCCHC4*	6.85	6.93	8.20	0.012010696	0.032164823

8096528	5161	*pyruvate dehydrogenase (lipoamide) alpha 2*	*PDHA2*	6.42	6.37	6.20	0.012011854	0.03216494
8016320	388394	*reprimo-like*	*RPRML*	8.69	8.83	8.24	0.012015449	0.03217158
8115681	79646	*pantothenate kinase 3*	*PANK3*	8.47	8.41	9.94	0.012020661	0.032175012
8085772				4.63	4.59	4.48	0.012021189	0.032175012
8099324				5.40	5.48	4.98	0.012018807	0.032175012
8167161				5.61	5.61	5.23	0.012020452	0.032175012
8141795	548644, 246721, 84820, 5439, 441259	*polymerase (RNA) II (DNA directed) polypeptide J3 | polymerase (RNA) II (DNA directed) polypeptide J2 | polymerase (RNA) II (DNA directed) polypeptide J4, pseudogene | polymerase (RNA) II (DNA directed) polypeptide J, 13.3kDa | PMS2 postmeiotic segregation increased 2 (S. cerevisiae)-like*	*POLR2J3| POLR2J2| POLR2J4| LOC441259*	11.08	11.29	10.55	0.012023757	0.032178905
7923596	55224	*ethanolamine kinase 2*	*ETNK2*	9.63	8.07	9.49	0.012030638	0.032192174
8008110	404266		*LOC404266*	7.29	8.04	6.85	0.012030945	0.032192174
7912994	126917	*intermediate filament family orphan 2*	*IFFO2*	7.69	8.09	7.25	0.012044684	0.032219976
7920873	677823	*small nucleolar RNA, H/ACA box 42*	*SNORA42*	9.25	9.23	8.33	0.012044555	0.032219976
8132243				5.81	5.53	5.09	0.012044541	0.032219976
7986765	6125	*ribosomal protein L5*	*RPL5*	12.90	12.89	13.40	0.012055652	0.03224633
7923929	5284	*polymeric immunoglobulin receptor*	*PIGR*	6.27	6.43	6.04	0.012058583	0.03225065
8073698	388910	*non-protein coding RNA 207*	*NCRNA00207*	5.94	6.04	5.71	0.012059502	0.03225065
7935834	79955	*PDZ domain containing 7*	*PDZD7*	7.40	7.42	6.80	0.012064531	0.03226111
7951351	80310	*platelet derived growth factor D*	*PDGFD*	9.09	7.87	6.72	0.012066476	0.032263122
7975705	122961	*iron-sulfur cluster assembly 2 homolog (S. cerevisiae)*	*ISCA2*	8.75	8.70	9.31	0.012067518	0.032263122
8073596	253190	*serine hydrolase-like 2*	*SERHL2*	6.61	6.69	5.67	0.012070926	0.032269243
8108470				6.86	7.05	6.64	0.012073147	0.032272194
7986767				5.98	6.07	5.98	0.012088597	0.0323105
8071642	28778	*immunoglobulin lambda variable 6-57*	*IGLV6-57*	6.83	6.93	6.54	0.012092461	0.032317836

8070799	7327, 6612	*ubiquitin-conjugating enzyme E2G 2 (UBC7 homolog, yeast) | SMT3 suppressor of mif two 3 homolog 3 (S. cerevisiae)*	*UBE2G2|SUMO3*	8.93	9.22	9.76	0.012099867	0.032334633
8010841	64122	*fructosamine 3 kinase*	*FN3K*	8.77	8.25	7.84	0.012109058	0.032356203
8099721	23231	*sel-1 suppressor of lin-12-like 3 (C. elegans)*	*SEL1L3*	8.20	8.55	10.30	0.012111561	0.032359894
7907394				4.75	4.70	4.49	0.012116065	0.032368932
7933394	143162, 728798, 728603	*FERM and PDZ domain containing 2 | FERM and PDZ domain containing 2 pseudogene 1 | FERM and PDZ domain containing 2 pseudogene 2*	*FRMPD2|FRMPD2P1|FRMPDP2*	5.82	5.84	5.70	0.01211814	0.03237148
7942261	3846	*keratin associated protein 5-9*	*KRTAP5-9*	7.46	7.20	6.90	0.01212546	0.032377303
7943984	7704	*zinc finger and BTB domain containing 16*	*ZBTB16*	7.18	7.22	6.87	0.012125826	0.032377303
7898296	149563	*chromosome 1 open reading frame 64*	*C1orf64*	6.61	6.57	6.14	0.012125928	0.032377303
8019635	6066, 26855, 54663	*RNA, U2 small nuclear 1 | RNA, U2 small nuclear 2 | WD repeat domain 74*	*RNU2-1|RNU2-2|WDR74*	13.55	13.53	13.84	0.012125571	0.032377303
8037003				7.20	7.04	6.56	0.012121632	0.032377303
8134599	10898	*cleavage and polyadenylation specific factor 4, 30kDa*	*CPSF4*	9.34	9.90	10.60	0.012129361	0.03238048
8019707	6066, 26855, 54663	*RNA, U2 small nuclear 1 | RNA, U2 small nuclear 2 | WD repeat domain 74*	*RNU2-1|RNU2-2|WDR74*	13.55	13.53	13.84	0.012128476	0.03238048
8131324				6.21	6.22	6.44	0.012136008	0.03239523
7999476				7.58	6.55	8.37	0.012143248	0.03241156
7949496	1072	*cofilin 1 (non-muscle)*	*CFL1*	13.15	13.29	12.62	0.012145208	0.032413796
7942232	55191	*NAD synthetase 1*	*NADSYN1*	9.79	9.69	8.96	0.012159501	0.03244594
8166565	286514	*melanoma antigen family B, 18*	*MAGEB18*	5.73	5.62	5.36	0.012159037	0.03244594
7901913	27022	*forkhead box D3*	*FOXD3*	7.00	7.10	7.45	0.012171286	0.032469675
8019641	6066, 26855, 54663	*RNA, U2 small nuclear 1 | RNA, U2 small nuclear 2 | WD repeat domain 74*	*RNU2-1|RNU2-2|WDR74*	13.55	13.53	13.84	0.012172552	0.032469675
8019703	6066, 26855, 54663	*RNA, U2 small nuclear 1 | RNA, U2 small nuclear 2 | WD repeat domain 74*	*RNU2-1|RNU2-2|WDR74*	13.55	13.53	13.84	0.012172358	0.032469675

8176284				6.35	6.75	5.92	0.012172895	0.032469675
8049534	9208	*leucine rich repeat (in FLII) interacting protein 1*	*LRRFIP1*	8.11	8.17	7.35	0.012176871	0.032477282
8019633	6066, 26855, 54663	*RNA, U2 small nuclear 1 | RNA, U2 small nuclear 2 | WD repeat domain 74*	*RNU2-1|RNU2-2|WDR74*	13.55	13.53	13.84	0.012179517	0.03248052
8143365				6.28	5.73	6.03	0.012180336	0.03248052
8091656	196074	*methyltransferase 5 domain containing 1*	*METT5D1*	7.44	6.90	9.17	0.012192132	0.032508973
8082261	152015, 54763	*ropporin, rhophilin associated protein 1B | ropporin, rhophilin associated protein 1*	*ROPN1B*	5.08	5.17	4.92	0.012194183	0.03251144
8164293	203	*adenylate kinase 1*	*AK1*	9.09	9.47	8.00	0.012209018	0.032547984
8042501				5.21	5.05	5.51	0.012221299	0.03257772
7963534	3851	*keratin 4*	*KRT4*	7.02	7.02	6.46	0.012234638	0.032610264
8042464				5.30	5.25	4.99	0.012237777	0.03261562
7954029	1027	*cyclin-dependent kinase inhibitor 1B (p27, Kip1)*	*CDKN1B*	9.81	9.19	9.19	0.012241578	0.032616694
8160163	9350	*cerberus 1, cysteine knot superfamily, homolog (Xenopus laevis)*	*CER1*	5.98	6.02	7.18	0.012242699	0.032616694
8019637	6066, 26855, 54663	*RNA, U2 small nuclear 1 | RNA, U2 small nuclear 2 | WD repeat domain 74*	*RNU2-1|RNU2-2|WDR74*	13.55	13.53	13.84	0.012241841	0.032616694
8019639	6066, 26855, 54663	*RNA, U2 small nuclear 1 | RNA, U2 small nuclear 2 | WD repeat domain 74*	*RNU2-1|RNU2-2|WDR74*	13.55	13.53	13.84	0.012240554	0.032616694
8178324	80862	*ZNRD1 antisense RNA (non-protein coding)*	*ZNRD1-AS*	5.46	5.43	6.39	0.012248705	0.032626674
7956783				5.62	5.53	5.30	0.012248074	0.032626674
7917697	2672	*growth factor independent 1 transcription repressor*	*GFI1*	6.70	6.74	6.25	0.012255348	0.032638345
8143221	50617	*ATPase, H+ transporting, lysosomal V0 subunit a4*	*ATP6V0A4*	6.29	6.23	7.36	0.012255022	0.032638345
7943729	341032	*chromosome 11 open reading frame 53*	*C11orf53*	6.06	5.92	5.63	0.012256646	0.03263879
8019631	6066, 26855, 54663	*RNA, U2 small nuclear 1 | RNA, U2 small nuclear 2 | WD repeat domain 74*	*RNU2-1|RNU2-2|WDR74*	13.55	13.53	13.84	0.012265869	0.032660335
8045153				5.95	5.88	5.23	0.012271513	0.032672353

7994187	27327	*trinucleotide repeat containing 6A*	*TNRC6A*	8.43	8.05	10.42	0.012272741	0.032672606
8082517	339942	*chromosome 3 open reading frame 47*	*C3orf47*	7.19	7.27	6.77	0.012274547	0.032674402
7940914	2286	*FK506 binding protein 2, 13kDa*	*FKBP2*	8.19	8.42	7.80	0.012277712	0.032679815
8019705	6066, 26855, 54663	*RNA, U2 small nuclear 1 | RNA, U2 small nuclear 2 | WD repeat domain 74*	*RNU2-1|RNU2-2|WDR74*	13.55	13.53	13.84	0.01229106	0.032712325
7943226	390243	*folate receptor 4 (delta) homolog (mouse)*	*FOLR4*	7.59	7.48	6.99	0.0122922	0.032712344
7973116	64745	*methyltransferase 11 domain containing 1*	*METT11D1*	8.88	8.80	9.36	0.012297506	0.03272345
8032761	5605	*mitogen-activated protein kinase kinase 2*	*MAP2K2*	10.76	10.85	9.98	0.012299845	0.032726657
8069845	337974, 337973	*keratin associated protein 19-7 | keratin associated protein 19-6*	*KRTAP19-7|KRTAP19-6*	5.30	5.10	5.03	0.012304371	0.032735683
8125780	117283	*inositol hexakisphosphate kinase 3*	*IP6K3*	6.70	6.88	6.50	0.012310106	0.032747924
8008208	11248	*neurexophilin 3*	*NXPH3*	8.24	8.25	7.66	0.012316002	0.032760587
8080320	11188	*nischarin*	*NISCH*	9.81	9.62	8.96	0.012323236	0.032773856
8120441	23469	*PHD finger protein 3*	*PHF3*	8.84	8.27	10.36	0.01232326	0.032773856
8045563	55843	*Rho GTPase activating protein 15*	*ARHGAP15*	5.26	5.39	5.13	0.012325166	0.032775905
7953532	2026	*enolase 2 (gamma, neuronal)*	*ENO2*	8.46	9.89	9.32	0.012329648	0.032784805
8013616	113235, 23098, 147007	*solute carrier family 46 (folate transporter), member 1 | sterile alpha and TIR motif containing 1 | transmembrane protein 199*	*SLC46A1|SARM1|TMEM199*	8.81	7.99	7.63	0.012343895	0.032819662
8130403				9.34	9.19	9.74	0.01234933	0.03283109
8076836	23774	*bromodomain containing 1*	*BRD1*	8.08	8.12	7.82	0.012354208	0.032841034
8053004	84279	*chromosome 2 open reading frame 7*	*C2orf7*	7.32	7.54	6.74	0.012359668	0.032849498
7929003	340654	*lipase, family member M*	*LIPM*	5.75	5.85	5.53	0.012358564	0.032849498
8040465				7.59	7.86	6.69	0.012363888	0.03285769
8103975	643036	*proteoglycan 3 pseudogene*	*SLED1*	6.21	6.11	5.90	0.012377843	0.03289175
8048717	130367	*sphingosine-1-phosphate phosphatase 2*	*SGPP2*	5.65	5.70	5.39	0.012381568	0.032898616
8040578	79172, 109	*centromere protein O | adenylate cyclase 3*	*CENPO|ADCY3*	7.95	8.27	9.50	0.012383466	0.03290063

7921319	115350	*Fc receptor-like 1*	*FCRL1*	6.54	6.65	6.15	0.012390205	0.032915507
8175432	26787	*small nucleolar RNA, C/D box 61*	*SNORD61*	5.21	5.41	5.99	0.012393353	0.032917812
8024246	55009	*chromosome 19 open reading frame 24*	*C19orf24*	9.80	10.00	9.01	0.012393015	0.032917812
8020139				8.95	9.16	8.20	0.01239727	0.03292519
8051799	23498	*3-hydroxyanthranilate 3,4-dioxygenase*	*HAAO*	8.70	8.06	7.90	0.012405423	0.032943502
8080416	440957	*chromosome 3 open reading frame 78*	*C3orf78*	7.12	7.10	8.08	0.012406448	0.032943502
8014361	6368	*chemokine (C-C motif) ligand 23*	*CCL23*	5.33	5.44	5.20	0.012415782	0.032960396
8159008	6835	*surfeit 2*	*SURF2*	9.40	9.45	10.41	0.012418519	0.032960396
8067011	23394	*activity-dependent neuroprotector homeobox*	*ADNP*	9.27	8.98	10.31	0.012418481	0.032960396
7977203	374569	*asparaginase homolog (S. cerevisiae)*	*ASPG*	7.79	7.89	7.28	0.012418246	0.032960396
7905127				9.41	9.53	8.87	0.012416639	0.032960396
8133167	154881, 27342	*potassium channel tetramerisation domain containing 7 | RAB guanine nucleotide exchange factor (GEF) 1*	*KCTD7|RABGEF1*	9.15	8.66	8.08	0.012425237	0.032975197
8148607	2738	*GLI family zinc finger 4*	*GLI4*	7.76	7.71	7.07	0.012429269	0.032982863
8119595	285855	*ribosomal protein L7-like 1*	*RPL7L1*	8.42	8.94	10.33	0.01243979	0.03300775
7993872	7385	*ubiquinol-cytochrome c reductase core protein II*	*UQCRC2*	10.69	10.59	12.18	0.012443225	0.033010796
8155824	117531	*transmembrane channel-like 1*	*TMC1*	5.52	5.55	5.96	0.012442865	0.033010796
8023323	4152	*methyl-CpG binding domain protein 1*	*MBD1*	8.95	9.01	8.44	0.012454623	0.03302953
7939723	79096	*chromosome 11 open reading frame 49*	*C11orf49*	9.34	9.17	8.52	0.012452868	0.03302953
8039740	79673	*zinc finger protein 329*	*ZNF329*	6.57	6.68	7.41	0.012453663	0.03302953
8132978				9.42	9.51	8.88	0.012454864	0.03302953
7964631	338811	*family with sequence similarity 19 (chemokine (C-C motif)-like), member A2*	*FAM19A2*	5.77	6.73	6.57	0.012459962	0.033040013
7909446	3290	*hydroxysteroid (11-beta) dehydrogenase 1*	*HSD11B1*	5.43	5.60	5.23	0.012463686	0.033046853
8159919	55064	*chromosome 9 open reading frame 68*	*C9orf68*	5.66	5.67	5.91	0.012466762	0.033051975
8018264	283987	*chromosome 17 open reading frame 28*	*C17orf28*	7.69	7.16	7.83	0.012468024	0.033052284
7952069	56912	*intraflagellar transport 46 homolog (Chlamydomonas)*	*IFT46*	9.61	9.81	8.86	0.012474774	0.033067144
7985997				8.06	8.09	7.69	0.012504302	0.03314237
8144571	203074	*protease, serine, 55*	*PRSS55*	5.62	5.56	5.32	0.012507497	0.033147797

8130539	117289	*T-cell activation RhoGTPase activating protein*	*TAGAP*	5.77	5.81	5.54	0.012510853	0.033153646
8159078	11094	*chromosome 9 open reading frame 7*	*C9orf7*	8.48	8.39	7.88	0.012520967	0.033177402
7932495	399726	*chromosome 10 open reading frame 114*	*C10orf114*	6.70	6.88	7.65	0.0125234	0.033180803
8163999	10773	*zinc finger and BTB domain containing 6*	*ZBTB6*	7.54	7.44	8.12	0.012530037	0.033189252
7930376				7.57	7.55	6.92	0.012529548	0.033189252
8168456				5.39	5.31	5.12	0.012529736	0.033189252
8104074	4543	*melatonin receptor 1A*	*MTNR1A*	6.33	6.58	6.91	0.012534376	0.033197697
7945296	112937	*galactosidase, beta 1-like 3*	*GLB1L3*	6.01	6.10	7.58	0.012536835	0.033201165
7907260	388714	*flavin containing monooxygenase 6 pseudogene*	*FMO6P*	5.46	5.61	5.17	0.012540002	0.033206508
8125859	6882, 54887	*TAF11 RNA polymerase II, TATA box binding protein (TBP)-associated factor, 28kDa | UHRF1 binding protein 1*	*TAF11|UHRF1BP1*	7.39	7.56	9.01	0.012545049	0.033216823
8008049				6.06	6.09	5.66	0.012550516	0.033228252
8128714				5.47	5.39	5.18	0.012552989	0.03323175
8021741	84735	*carnosine dipeptidase 1 (metallopeptidase M20 family)*	*CNDP1*	5.72	5.65	5.32	0.012557183	0.033239808
7939424	8539	*apoptosis inhibitor 5*	*API5*	9.01	9.12	11.46	0.012567846	0.03326498
8104117				4.66	4.58	5.45	0.012573608	0.03327718
8101657				6.87	7.00	6.77	0.012575061	0.033277977
8052018	56660	*potassium channel, subfamily K, member 12*	*KCNK12*	7.63	7.70	8.35	0.012578683	0.033284508
7974483	3895	*kinectin 1 (kinesin receptor)*	*KTN1*	7.70	7.57	9.06	0.012581538	0.0332884
8102848	80854	*SET domain containing (lysine methyltransferase) 7*	*SETD7*	9.38	9.54	7.10	0.01258246	0.0332884
8043932	6160	*ribosomal protein L31*	*RPL31*	9.24	9.26	9.71	0.012593605	0.03331438
8148932	340385	*zinc finger protein 517*	*ZNF517*	7.85	7.88	7.21	0.012594587	0.03331438
8115064				4.56	4.65	4.48	0.012604276	0.033336952
7998910	146439	*coiled-coil domain containing 64B*	*CCDC64B*	7.36	7.53	6.98	0.012608766	0.03334272
8070791	353323	*keratin associated protein 12-2*	*KRTAP12-2*	7.57	7.55	7.09	0.012608407	0.03334272
7901416				5.34	5.22	5.15	0.012612672	0.03334999
8129134	6098	*c-ros oncogene 1 , receptor tyrosine kinase*	*ROS1*	5.14	5.25	5.07	0.012616066	0.03335286
7947563	60529	*ALX homeobox 4*	*ALX4*	7.32	7.16	6.69	0.012614995	0.03335286

8150976				4.87	4.96	4.73	0.012624075	0.033370975
8078971	956	*ectonucleoside triphosphate diphosphohydrolase 3*	*ENTPD3*	6.95	6.27	6.11	0.012629272	0.03337106
8015432	10609	*leprecan-like 4*	*LEPREL4*	8.21	8.36	6.88	0.012629887	0.03337106
7940333	219995	*membrane-spanning 4-domains, subfamily A, member 15*	*MS4A15*	6.40	6.47	6.10	0.012626988	0.03337106
7970392				6.76	6.39	5.93	0.012629597	0.03337106
7970426				6.36	6.56	7.33	0.012625584	0.03337106
8148070	7373	*collagen, type XIV, alpha 1*	*COL14A1*	7.21	7.03	8.41	0.012634582	0.03338041
8151250				4.95	5.02	4.77	0.012638203	0.033386923
8112020	4338	*molybdenum cofactor synthesis 2*	*MOCS2*	10.08	9.76	9.41	0.012641018	0.033391304
8073929	25817	*family with sequence similarity 19 (chemokine (C-C motif)-like), member A5*	*FAM19A5*	9.16	9.61	8.34	0.01264285	0.03339309
7931850				5.01	5.04	4.80	0.012652343	0.0334151
8019599	85293	*keratin associated protein 3-3*	*KRTAP3-3*	5.17	5.07	4.87	0.012654915	0.033418838
7963139	144233	*BCDIN3 domain containing*	*BCDIN3D*	7.01	7.17	6.38	0.012657051	0.033421423
8014071				5.97	6.14	5.49	0.012661259	0.033429474
8124534	8368	*histone cluster 1, H4l*	*HIST1H4L*	9.36	9.80	12.15	0.012667437	0.033440903
8174288	401612	*mitochondrial carrier triple repeat 6*	*MCART6*	6.88	6.92	7.05	0.012667904	0.033440903
7923917	9214	*Fas apoptotic inhibitory molecule 3*	*FAIM3*	6.36	6.41	6.00	0.012670183	0.03344386
8067007	7114, 7117, 55653, 7120	*thymosin beta 4, X-linked | thymosin-like 3 | breast carcinoma amplified sequence 4 | thymosin-like 6 (pseudogene)*	*TMSB4X|TMSL3|BCAS4|TMSL6*	11.55	11.99	11.18	0.012672472	0.033446845
8060461	117532	*transmembrane channel-like 2*	*TMC2*	5.29	5.14	4.88	0.012683081	0.033471785
8109620	7265	*tetratricopeptide repeat domain 1*	*TTC1*	7.14	7.26	8.69	0.012690418	0.033488087
8169447	3358	*5-hydroxytryptamine (serotonin) receptor 2C*	*HTR2C*	5.73	5.80	6.69	0.012699552	0.03350886
8101992	64116	*solute carrier family 39 (zinc transporter), member 8*	*SLC39A8*	8.77	7.31	9.79	0.01270063	0.03350886
7903507	284611	*family with sequence similarity 102, member B*	*FAM102B*	7.34	6.82	8.70	0.012701771	0.03350886
7960635	677780	*small Cajal body-specific RNA 11*	*SCARNA11*	6.75	6.93	6.38	0.012706053	0.03351709
8144044	346547	*hypothetical LOC346547*	*FLJ42291*	6.29	6.20	5.72	0.012710291	0.033522144

8165639	375791	*chromosome 9 open reading frame 169*	*C9orf169*	7.90	8.05	7.57	0.01270997	0.033522144
8131917				7.83	7.97	7.50	0.012711968	0.033523504
7937823	3784	*potassium voltage-gated channel, KQT-like subfamily, member 1*	*KCNQ1*	7.23	7.20	6.75	0.012720093	0.03354187
8144949				5.83	5.96	5.42	0.012723058	0.03354662
7958305	5992	*regulatory factor X, 4 (influences HLA class II expression)*	*RFX4*	5.39	5.44	5.15	0.012727761	0.033555955
7985587	54581	*SCAN domain containing 2 pseudogene*	*SCAND2*	7.04	7.10	6.70	0.012733066	0.03356418
7982185	28955, 100289656	*Dexi homolog (mouse) | Dexi homolog (mouse) pseudogene*	*DEXI|LOC 100289656*	11.60	11.40	10.62	0.012733205	0.03356418
8148327				6.29	6.45	5.90	0.012736916	0.033570897
8168638	53336	*CPX chromosome region, candidate 1*	*CPXCR1*	5.19	5.21	4.98	0.012739117	0.03357363
8030796	284367	*sialic acid binding Ig-like lectin, pseudogene 3*	*SIGLECP3*	5.90	5.99	5.59	0.012740525	0.033574276
8163930	4702	*NADH dehydrogenase (ubiquinone) 1 alpha subcomplex, 8, 19kDa*	*NDUFA8*	9.47	9.49	10.11	0.012748157	0.033591323
8114470	26045, 1495	*leucine rich repeat transmembrane neuronal 2 | catenin (cadherin-associated protein), alpha 1, 102kDa*	*LRRTM2|C TNNA1*	5.60	5.56	5.16	0.012758986	0.03361679
8115623	23120	*ATPase, class V, type 10B*	*ATP10B*	6.23	6.21	5.80	0.012763077	0.033622093
8025450	284383	*olfactory receptor, family 2, subfamily Z, member 1*	*OR2Z1*	6.69	6.72	6.17	0.012763329	0.033622093
8083429	4154	*muscleblind-like (Drosophila)*	*MBNL1*	10.63	10.92	9.24	0.012766303	0.033626862
7986685	28955, 100289656	*Dexi homolog (mouse) | Dexi homolog (mouse) pseudogene*	*DEXI|LOC 100289656*	11.60	11.40	10.62	0.012768059	0.03362842
8149264	79660	*protein phosphatase 1, regulatory (inhibitor) subunit 3B*	*PPP1R3B*	7.26	7.50	8.45	0.012782835	0.033664264
8148476	22898	*DENN/MADD domain containing 3*	*DENND3*	7.38	7.13	6.78	0.012784926	0.0336667
7907024	57645	*pogo transposable element with KRAB domain*	*POGK*	8.67	8.74	9.59	0.012787493	0.03367039
8102127	6870	*tachykinin receptor 3*	*TACR3*	5.13	5.16	5.89	0.012798599	0.03369656
7936023	79946	*chromosome 10 open reading frame 95*	*C10orf95*	7.61	7.59	7.14	0.012807772	0.033717632
8071981	85379		*KIAA1671*	7.71	7.68	8.43	0.012813969	0.033730872
7910727	88	*actinin, alpha 2*	*ACTN2*	5.99	6.11	6.30	0.012816847	0.033735372

8080685	7871	*sarcolemma associated protein*	*SLMAP*	8.53	8.27	9.57	0.012818118	0.03373564
8115346	475	*ATX1 antioxidant protein 1 homolog (yeast)*	*ATOX1*	9.56	9.75	8.55	0.012831971	0.033765946
8079719	339834	*coiled-coil domain containing 36*	*CCDC36*	7.05	6.95	5.71	0.012831332	0.033765946
7967117	8638	*2'-5'-oligoadenylate synthetase-like*	*OASL*	6.31	6.30	5.92	0.012834101	0.03376847
8013243	6470	*serine hydroxymethyltransferase 1 (soluble)*	*SHMT1*	6.58	6.66	7.85	0.012840503	0.033771798
8117054	10486	*CAP, adenylate cyclase-associated protein, 2 (yeast)*	*CAP2*	10.87	10.09	9.35	0.012842324	0.033771798
7920228	57115	*peptidoglycan recognition protein 4*	*PGLYRP4*	6.39	6.59	6.06	0.012840559	0.033771798
7953590	406985	*microRNA 200c*	*MIR200C*	5.80	5.75	6.19	0.012842385	0.033771798
7957271	100289143	*similar to nucleolar protein family A, member 3*	*LOC100289143*	6.99	7.39	7.58	0.012840478	0.033771798
8151609	254958, 100288527	*REX1, RNA exonuclease 1 homolog (S. cerevisiae)-like 1 | REX1, RNA exonuclease 1 homolog (S. cerevisiae)-like 2 (pseudogene)*	*REXO1L1|REXO1L2P*	7.33	7.48	6.80	0.012836676	0.033771798
8093993	93624	*transcriptional adaptor 2B*	*TADA2B*	9.31	9.19	8.52	0.012849508	0.033784375
7911683	199990, 100128003	*chromosome 1 open reading frame 86 | hypothetical LOC100128003*	*C1orf86|LOC100128003*	8.38	8.46	7.65	0.012849349	0.033784375
8164562	375759	*chromosome 9 open reading frame 50*	*C9orf50*	7.43	7.53	6.96	0.012851659	0.033786956
7972912	55208	*DCN1, defective in cullin neddylation 1, domain containing 2 (S. cerevisiae)*	*DCUN1D2*	6.79	7.14	8.20	0.012863912	0.033816088
8026548	8907	*adaptor-related protein complex 1, mu 1 subunit*	*AP1M1*	10.51	10.38	8.94	0.012871944	0.033834122
8075924	4242		*MFNG*	6.16	6.33	5.98	0.012875694	0.033840895
8060758	5621	*prion protein*	*PRNP*	11.99	11.61	10.06	0.012881785	0.03385382
8032996	125988	*chromosome 19 open reading frame 70*	*C19orf70*	9.08	9.03	7.85	0.012887355	0.033865377
7970954	9201	*doublecortin-like kinase 1*	*DCLK1*	7.96	7.46	10.52	0.012890872	0.033871535
8031213	11024	*leukocyte immunoglobulin-like receptor, subfamily A (with TM domain), member 1*	*LILRA1*	6.65	6.52	6.22	0.012894199	0.033877198
7963988	6601	*SWI/SNF related, matrix associated, actin dependent regulator of chromatin, subfamily c, member 2*	*SMARCC2*	10.38	10.12	9.48	0.012906804	0.033891805
7953229	8798	*dual-specificity tyrosine-(Y)-phosphorylation regulated kinase 4*	*DYRK4*	6.78	6.77	6.21	0.012906044	0.033891805
8144281	9639	*Rho guanine nucleotide exchange factor (GEF) 10*	*ARHGEF10*	8.04	8.15	8.81	0.012902625	0.033891805

8065668	51654	*CDK5 regulatory subunit associated protein 1*	*CDK5RAP 1*	7.91	7.98	8.68	0.012904494	0.033891805
8061406	79953	*transmembrane protein 90B*	*TMEM90B*	7.07	7.74	7.80	0.012906164	0.033891805
7935778				4.70	4.70	4.58	0.012903951	0.033891805
7930450				5.87	6.06	6.90	0.012914428	0.033902574
7983252				6.50	6.32	6.01	0.012914342	0.033902574
8149281				5.10	4.97	4.74	0.01291305	0.033902574
7928679	219348	*placenta-specific 9*	*PLAC9*	8.36	8.81	7.11	0.012916623	0.033905253
7957549				7.88	7.76	9.01	0.012920467	0.03391226
7925561	116228, 284702	*family with sequence similarity 36, member A | non-protein coding RNA 201*	*FAM36A|NCRNA00201*	6.83	6.28	8.43	0.012924143	0.033918828
8086878				5.24	5.29	4.95	0.012928117	0.03392617
7973694	56413, 1241, 27141, 161424	*leukotriene B4 receptor 2 | cell death-inducing DFFA-like effector b | chromosome 14 open reading frame 21*	*LTB4R2|CIDEB|C14orf21*	7.02	7.05	6.58	0.012932986	0.03393278
7991153				5.43	5.32	5.30	0.01293219	0.03393278
8134079	85865, 9069	*GTP-binding protein 10 (putative) | claudin 12*	*GTPBP10|CLDN12*	8.59	8.68	9.59	0.01293552	0.033936344
8174642				8.02	8.27	7.64	0.012941047	0.03394776
7918188	29957	*solute carrier family 25 (mitochondrial carrier; phosphate carrier), member 24*	*SLC25A24*	8.16	7.89	9.21	0.012946887	0.033959992
7947531	29099	*COMM domain containing 9*	*COMMD9*	8.94	9.05	8.26	0.012949505	0.033963773
8051987				5.72	5.96	5.43	0.012953307	0.033970658
8010783	2837	*urotensin 2 receptor*	*UTS2R*	7.52	7.77	6.94	0.012960964	0.033985175
8085340	9686	*vestigial like 4 (Drosophila)*	*VGLL4*	8.14	8.56	7.90	0.012963258	0.033985175
8126296	340206	*triggering receptor expressed on myeloid cells-like 3*	*TREML3*	6.48	6.26	5.96	0.012963551	0.033985175
7906486	56833, 284677	*SLAM family member 8 | chromosome 1 open reading frame 204*	*SLAMF8|C1orf204*	6.46	6.66	6.23	0.012961803	0.033985175
7970810	6541	*solute carrier family 7 (cationic amino acid transporter, y+ system), member 1*	*SLC7A1*	10.64	11.12	10.24	0.012968412	0.033991743

8103437	5481, 2110	*peptidylprolyl isomerase D | electron-transferring-flavoprotein dehydrogenase*	*PPID|ETFDH*	6.77	6.91	8.55	0.012967568	0.033991743
7976642				6.98	6.87	8.34	0.01297534	0.03400373
8100555				5.64	5.60	5.81	0.012975216	0.03400373
7952451	100130428	*IGYY565*	*LOC100130428*	7.00	6.42	9.12	0.012989363	0.03403739
8059799				4.78	4.79	4.63	0.012992584	0.03404274
7915949	388630	*UPF0632 protein A*	*LOC388630*	7.42	7.49	6.91	0.012995831	0.034048155
8161988	29979	*ubiquilin 1*	*UBQLN1*	10.54	10.74	11.36	0.013007792	0.03407462
8050227	400943	*AILT5830*	*UNQ5830*	6.23	6.26	5.88	0.013008292	0.03407462
8075846	400926		*FLJ90680*	6.11	6.02	5.75	0.013009856	0.034075625
7922482	7143	*tenascin R (restrictin, janusin)*	*TNR*	6.42	6.45	6.03	0.013015142	0.034086376
8138997	57057	*T-box 20*	*TBX20*	6.69	6.71	6.40	0.013021534	0.03409621
8085255	285368	*proline-rich transmembrane protein 3*	*PRRT3*	7.33	6.95	6.81	0.013022187	0.03409621
8094874				8.08	7.98	8.38	0.01302244	0.03409621
7967287	55596	*zinc finger, CCHC domain containing 8*	*ZCCHC8*	8.08	8.04	9.18	0.013031203	0.03411606
8084045	55669	*mitofusin 1*	*MFN1*	8.22	7.91	9.31	0.01303284	0.03411725
7955419				6.18	5.79	6.62	0.013034249	0.034117848
8069519				7.85	7.93	7.26	0.013039275	0.03412791
8113639				5.61	5.71	5.43	0.013045604	0.03414138
7969995	23026	*myosin XVI*	*MYO16*	6.79	6.79	6.42	0.013048843	0.034143664
8056220	25909	*AT hook containing transcription factor 1*	*AHCTF1*	7.59	7.31	9.38	0.013048805	0.034143664
7937106	84504	*NK6 homeobox 2*	*NKX6-2*	7.58	7.40	7.14	0.01305455	0.0341555
8083166	7220	*transient receptor potential cation channel, subfamily C, member 1*	*TRPC1*	8.08	7.91	8.90	0.01306032	0.034164406
7935968	8861	*LIM domain binding 1*	*LDB1*	12.07	11.69	11.50	0.013059695	0.034164406
8098441	55714	*odz, odd Oz/ten-m homolog 3 (Drosophila)*	*ODZ3*	7.27	9.54	10.38	0.013070495	0.034186423
7945859	116534	*MAS-related GPR, member E*	*MRGPRE*	7.62	7.68	7.16	0.013071105	0.034186423
8158671	445	*argininosuccinate synthase 1*	*ASS1*	12.85	12.42	11.57	0.013072784	0.034187716

8149574	55790	*chondroitin sulfate N-acetylgalactosaminyltransferase 1*	*CSGALNACT1*	6.62	6.12	5.83	0.013079273	0.03419849
8106929				4.61	4.61	4.51	0.013079064	0.03419849
7972682	79070	*KDEL (Lys-Asp-Glu-Leu) containing 1*	*KDELC1*	7.40	7.67	8.34	0.01308172	0.03420179
8143684	9601	*protein disulfide isomerase family A, member 4*	*PDIA4*	10.74	11.01	11.32	0.013092802	0.034225475
8024255	84939	*melanoma associated antigen (mutated) 1*	*MUM1*	8.48	8.66	7.93	0.013093149	0.034225475
8016866				6.60	6.69	6.00	0.013099546	0.034239095
8040080	91543	*radical S-adenosyl methionine domain containing 2*	*RSAD2*	5.64	5.58	5.21	0.013110396	0.034264352
7960682	196500	*chromosome 12 open reading frame 53*	*C12orf53*	8.77	8.31	7.83	0.01311279	0.034267504
8014369	6348	*chemokine (C-C motif) ligand 3*	*CCL3*	6.81	6.99	6.50	0.01311867	0.034276668
8104014	256309	*coiled-coil domain containing 110*	*CCDC110*	5.25	5.14	5.73	0.013118526	0.034276668
8100603	1060	*centromere protein C 1*	*CENPC1*	7.12	6.94	8.10	0.013122445	0.03428109
7925565	3192	*heterogeneous nuclear ribonucleoprotein U (scaffold attachment factor A)*	*HNRNPU*	8.39	8.39	10.56	0.013122738	0.03428109
7947881	5913	*receptor-associated protein of the synapse*	*RAPSN*	7.55	7.48	7.03	0.013136425	0.03431374
8063903	6227	*ribosomal protein S21*	*RPS21*	8.29	8.36	9.01	0.013146007	0.034329448
8124437	8968	*histone cluster 1, H3f*	*HIST1H3F*	8.29	9.30	11.01	0.013145606	0.034329448
7931859	51806	*calmodulin-like 5*	*CALML5*	7.20	7.29	6.74	0.013145307	0.034329448
7997875	283860	*chromosome 16 open reading frame 81*	*C16orf81*	6.90	6.91	6.45	0.013148686	0.03433334
8170185				4.57	4.67	4.59	0.013150019	0.034333713
8092845	2814	*glycoprotein V (platelet)*	*GP5*	7.57	7.63	7.04	0.013155018	0.034340557
7996976	11060	*WW domain containing E3 ubiquitin protein ligase 2*	*WWP2*	8.68	8.65	7.97	0.013154706	0.034340557
7973034	390442	*olfactory receptor, family 11, subfamily H, member 4*	*OR11H4*	4.69	4.79	4.61	0.013160344	0.034351353
7995193	79798	*armadillo repeat containing 5*	*ARMC5*	7.69	7.87	7.14	0.013171469	0.034377284
7998668	124056	*NADPH oxidase organizer 1*	*NOXO1*	7.53	7.65	6.93	0.013178079	0.03439143
7925735	284532	*olfactory receptor, family 14, subfamily A, member 16*	*OR14A16*	5.41	5.54	5.23	0.01318097	0.034395862
7900395	6018	*rearranged L-myc fusion*	*RLF*	8.45	8.47	9.81	0.01318428	0.03440139
8170286				5.40	5.31	5.24	0.013188026	0.034408055
8012469	6154	*ribosomal protein L26*	*RPL26*	10.29	10.52	10.76	0.013195229	0.034423735

7920852	22889		*KIAA0907*	8.50	8.66	9.60	0.013216623	0.034476433
8072358				7.00	7.09	6.48	0.013228136	0.03450335
8152701				6.62	6.61	6.19	0.013236109	0.034521025
7949971	1374	*carnitine palmitoyltransferase 1A (liver)*	*CPT1A*	9.98	9.92	8.81	0.013252793	0.034560543
8116710	9425	*chromodomain protein, Y-like*	*CDYL*	8.74	8.99	9.52	0.013253654	0.034560543
7968632				5.67	5.54	6.59	0.013258118	0.03456906
8159004	26820, 6130	*small nucleolar RNA, C/D box 24 | ribosomal protein L7a*	*SNORD24|RPL7A*	9.91	10.07	10.63	0.013269027	0.034594376
8092800	84239	*ATPase type 13A4*	*ATP13A4*	5.55	5.70	5.47	0.013273452	0.03460279
7984353	4091	*SMAD family member 6*	*SMAD6*	8.56	8.29	7.59	0.013280912	0.034609735
7977018	7187	*TNF receptor-associated factor 3*	*TRAF3*	8.12	8.00	7.73	0.01328045	0.034609735
7994655	79447	*chromosome 16 open reading frame 53*	*C16orf53*	8.38	8.31	9.16	0.013279134	0.034609735
8154979				5.78	5.64	5.26	0.013279022	0.034609735
8059985	151174		*LOC151174*	6.58	6.57	6.28	0.013285601	0.03461883
7910257	57165	*gap junction protein, gamma 2, 47kDa*	*GJC2*	8.84	8.86	8.30	0.013287627	0.034620985
8126244	57497	*leucine rich repeat and fibronectin type III domain containing 2*	*LRFN2*	6.31	6.41	6.03	0.013291449	0.034624692
7899251				5.04	5.07	4.76	0.013290777	0.034624692
8075691	6171, 6172	*ribosomal protein L41 | ribosomal protein L41 pseudogene 5*	*RPL41|RPL41P5*	13.20	13.29	13.12	0.013292714	0.034624863
8137464	5723	*phosphoserine phosphatase*	*PSPH*	9.42	10.30	10.58	0.013296768	0.0346323
8117339	8352	*histone cluster 1, H3c*	*HIST1H3C*	7.26	7.74	9.49	0.013301724	0.03464208
8096461	474	*atonal homolog 1 (Drosophila)*	*ATOH1*	7.03	7.05	7.85	0.01330654	0.034648668
8058258	65062	*amyotrophic lateral sclerosis 2 (juvenile) chromosome region, candidate 4*	*ALS2CR4*	8.23	8.90	8.79	0.013306653	0.034648668
7955855	3227	*homeobox C11*	*HOXC11*	6.28	6.68	5.98	0.013317518	0.03467383
8014391	6349, 414062, 6348	*chemokine (C-C motif) ligand 3-like 1 | chemokine (C-C motif) ligand 3-like 3 | chemokine (C-C motif) ligand 3*	*CCL3L1|CCL3L3*	7.93	7.95	7.40	0.013322542	0.034683783
8035646	404037	*hyaluronan and proteoglycan link protein 4*	*HAPLN4*	9.59	9.64	9.13	0.013333393	0.034708902

8153896	80778	*zinc finger protein 34*	*ZNF34*	7.12	7.28	7.50	0.013345998	0.03473858
8013947	84940	*coronin 6*	*CORO6*	7.85	7.88	7.06	0.013349278	0.034743987
8165498	10383, 401565	*tubulin, beta 2C | family with sequence similarity 166, member A*	*TUBB2C|FAM166A*	7.00	7.27	6.56	0.013353115	0.03475084
8112070				6.79	6.31	7.80	0.013355701	0.03475444
8024038	566	*azurocidin 1*	*AZU1*	7.53	7.56	6.93	0.01336139	0.03476321
8026442	11283	*cytochrome P450, family 4, subfamily F, polypeptide 8*	*CYP4F8*	6.09	6.10	5.58	0.01336148	0.03476321
7901732	731	*complement component 8, alpha polypeptide*	*C8A*	5.46	5.34	5.21	0.013362978	0.034763973
7968972	10063		*COX17*	8.21	8.79	9.25	0.013365356	0.034767028
8165667				10.68	10.68	10.03	0.01339131	0.0348314
8035905	84079	*ankyrin repeat domain 27 (VPS9 domain)*	*ANKRD27*	8.68	9.04	9.50	0.013396239	0.034841083
8054790				5.75	5.67	5.49	0.013406434	0.03486446
8163002	9314	*Kruppel-like factor 4 (gut)*	*KLF4*	8.65	8.55	7.28	0.013409307	0.034868788
8112668	51301	*glucosaminyl (N-acetyl) transferase 4, core 2*	*GCNT4*	7.78	6.44	8.92	0.013412025	0.034872714
8029784	64344	*hypoxia inducible factor 3, alpha subunit*	*HIF3A*	6.81	6.67	7.49	0.013413529	0.034872856
7984152	283807	*F-box and leucine-rich repeat protein 22*	*FBXL22*	6.98	7.12	6.25	0.013415077	0.034872856
7911092				9.16	9.29	8.47	0.013415703	0.034872856
7896752				10.68	10.68	10.03	0.013421438	0.03488462
7992140				7.35	7.39	6.89	0.013428278	0.034899257
8037970	8605	*phospholipase A2, group IVC (cytosolic, calcium-independent)*	*PLA2G4C*	7.28	7.35	5.80	0.013432049	0.03490027
8153939	8694	*diacylglycerol O-acyltransferase 1*	*DGAT1*	9.83	9.82	8.92	0.013431716	0.03490027
8144089	64327	*limb region 1 homolog (mouse)*	*LMBR1*	8.61	8.56	10.15	0.013432294	0.03490027
8065889	55245	*ubiquinol-cytochrome c reductase complex chaperone*	*UQCC*	8.18	8.15	8.73	0.01344625	0.03493339
8056363	151258	*solute carrier family 38, member 11*	*SLC38A11*	5.47	5.62	5.20	0.013448821	0.034936924
8153684	8694	*diacylglycerol O-acyltransferase 1*	*DGAT1*	9.83	9.81	8.92	0.013459212	0.03496077
8063921	406904	*microRNA 1-1*	*MIR1-1*	4.70	4.81	4.58	0.01346478	0.034972087
8174979	139741	*actin-related protein T1*	*ACTRT1*	5.86	5.79	5.65	0.01348764	0.03502831
8010184				6.39	6.04	5.50	0.013491229	0.035034478

7968199	1024	*cyclin-dependent kinase 8*	*CDK8*	8.62	8.69	9.99	0.013492749	0.035035275
7941444	1521	*cathepsin W*	*CTSW*	6.72	6.78	6.13	0.013497323	0.035040848
7945125	283165	*non-protein coding RNA 288*	*NCRNA00 288*	6.96	7.15	6.60	0.013496763	0.035040848
7948324	441608	*olfactory receptor, family 5, subfamily B, member 3*	*OR5B3*	5.13	5.07	4.91	0.013498849	0.035041656
8091327	5359	*phospholipid scramblase 1*	*PLSCR1*	7.66	7.45	9.10	0.013500189	0.035041984
8134374				7.57	7.51	7.90	0.013501653	0.035042632
8016086				7.28	7.49	6.90	0.013505738	0.035050087
7929945				6.67	6.78	6.41	0.013507513	0.035051543
8103906	728175		*LOC72817 5*	6.42	6.47	6.15	0.013514431	0.03506634
7896798	84069	*pleckstrin homology domain containing, family N member 1*	*PLEKHN1*	8.22	8.27	7.69	0.013518508	0.035073765
7947338	5080	*paired box 6*	*PAX6*	6.44	6.87	6.28	0.013532008	0.035102405
7914218	6883		*TAF12*	8.55	8.57	9.03	0.013533194	0.035102405
7984588	79875	*thrombospondin, type I, domain containing 4*	*THSD4*	8.75	7.73	7.33	0.013530917	0.035102405
8141791	548644, 246721, 100134938	*polymerase (RNA) II (DNA directed) polypeptide J3 | polymerase (RNA) II (DNA directed) polypeptide J2 | uroplakin 3B-like*	*POLR2J3| POLR2J2| UPK3BL*	11.14	11.84	9.65	0.013535596	0.03510548
7922969	5132	*phosducin*	*PDC*	5.33	5.17	5.08	0.013540265	0.035111204
8004431	5430	*polymerase (RNA) II (DNA directed) polypeptide A, 220kDa*	*POLR2A*	11.47	11.21	10.10	0.013540717	0.035111204
8159808	2298, 200350, 653404, 286380	*forkhead box D4 | forkhead box D4-like 1 | forkhead box D4-like 6 | forkhead box D4-like 3*	*FOXD4|F OXD4L1|F OXD4L6|F OXD4L3*	7.18	7.27	6.89	0.013541451	0.035111204
8134581	10552	*actin related protein 2/3 complex, subunit 1A, 41kDa*	*ARPC1A*	11.18	11.21	10.88	0.013543983	0.035114616
8081115	200894	*ADP-ribosylation factor-like 13B*	*ARL13B*	7.29	7.34	8.16	0.013558535	0.035149187
8116545	642316	*hypothetical LOC642316*	*FLJ43763*	5.67	5.75	5.32	0.01356137	0.035152663
8015946	339201, 92591	*chromosome 17 open reading frame 65 | ankyrin repeat and SOCS box-containing 16*	*C17orf65| ASB16*	9.68	9.36	8.75	0.013562311	0.035152663
8059576	55022	*phosphotyrosine interaction domain containing 1*	*PID1*	10.00	10.04	9.47	0.013564766	0.035152715

8002237	84138, 9057	*solute carrier family 7, member 6 opposite strand | solute carrier family 7 (cationic amino acid transporter, y+ system), member 6*	*SLC7A6OS*	8.36	8.38	8.78	0.013564209	0.035152715
8138224	4697	*NADH dehydrogenase (ubiquinone) 1 alpha subcomplex, 4, 9kDa*	*NDUFA4*	8.26	8.50	9.14	0.013566155	0.035153158
8000217	23049, 100271836, 641298, 440354, 595101	*SMG1 homolog, phosphatidylinositol 3-kinase-related kinase (C. elegans) | SMG1 homolog, phosphatidylinositol 3-kinase-related kinase pseudogene | PI-3-kinase-related kinase SMG-1 pseudogene*	*SMG1|LOC100271836|LOC641298|LOC440354|LOC595101*	11.39	11.28	12.60	0.01357047	0.035158027
8156897	91283, 8577	*chromosome 9 open reading frame 30 | transmembrane protein with EGF-like and two follistatin-like domains 1*	*C9orf30|TMEFF1*	9.96	10.24	9.95	0.013569735	0.035158027
8167947				5.67	5.63	4.67	0.013575194	0.03516711
8091875				5.53	5.22	4.98	0.013584049	0.03518689
7998940	4210	*Mediterranean fever*	*MEFV*	6.81	6.79	6.39	0.013588869	0.035191216
8066195	10904	*bladder cancer associated protein*	*BLCAP*	11.12	11.20	10.42	0.013588157	0.035191216
8014414	6349, 414062, 6348	*chemokine (C-C motif) ligand 3-like 1 | chemokine (C-C motif) ligand 3-like 3 | chemokine (C-C motif) ligand 3*	*CCL3L1|CCL3L3*	7.93	7.95	7.39	0.013589376	0.035191216
7994345	388228	*SH3-binding domain kinase 1*	*SBK1*	7.63	7.69	8.42	0.013601067	0.035218332
8091190	545	*ataxia telangiectasia and Rad3 related*	*ATR*	6.81	6.67	8.15	0.013605092	0.035225596
7975924				6.70	6.86	6.15	0.013612799	0.03524239
8064866	149837, 643406	*hypothetical LOC149837 | hypothetical LOC643406*	*LOC149837|LOC643406*	8.94	8.74	7.36	0.013620919	0.035257086
7974194				7.21	7.16	6.69	0.013619934	0.035257086
8110688	6550	*solute carrier family 9 (sodium/hydrogen exchanger), member 3*	*SLC9A3*	6.59	6.63	6.10	0.013624748	0.03526384
8061382	128817	*cystatin-like 1*	*CSTL1*	5.53	5.53	5.27	0.01363147	0.03527807
8167742	170627	*X antigen family, member 5*	*XAGE5*	4.91	4.95	4.68	0.013636023	0.03528669
8145685				5.97	5.39	8.07	0.013642311	0.0352998

7979378	55195	*chromosome 14 open reading frame 105*	*C14orf105*	5.00	4.99	4.87	0.013647013	0.03530723
7942284	245911, 503694	*defensin, beta 108B | defensin, beta 108, pseudogene 1*	*DEFB108B|DEFB108P1*	5.78	5.76	5.34	0.013647628	0.03530723
8092021	79782	*leucine rich repeat containing 31*	*LRRC31*	4.94	5.02	4.81	0.01365023	0.035307635
7967987	78988, 221150	*mitochondrial ribosomal protein 63 | spindle and kinetochore associated complex subunit 3*	*MRP63|SKA3*	7.39	7.53	8.12	0.013649308	0.035307635
8110090	94081	*sideroflexin 1*	*SFXN1*	9.40	9.46	10.50	0.013656367	0.03531925
8169965				4.65	4.66	4.79	0.013657169	0.03531925
8093829	166793	*zinc finger and BTB domain containing 49*	*ZBTB49*	7.03	7.04	7.73	0.013666342	0.035336643
8104122	22947, 728410, 653548, 653545, 653544	*double homeobox 4 | double homeobox 4 like 2 | double homeobox 4 like 3 | double homeobox 4 like 5 | double homeobox 4 like 6*	*DUX4|DUX4L2|DUX4L3|DUX4L5|DUX4L6*	8.58	8.71	8.06	0.01366563	0.035336643
7988767	1588	*cytochrome P450, family 19, subfamily A, polypeptide 1*	*CYP19A1*	5.76	5.84	5.38	0.013672193	0.035348605
8175524	347487	*chromosome X open reading frame 66*	*CXorf66*	4.91	4.88	4.71	0.013673552	0.035348956
8037236	440533	*pregnancy specific beta-1-glycoprotein 8*	*PSG8*	5.44	5.40	5.15	0.013682654	0.035369318
7952225				5.97	5.88	5.36	0.01369296	0.03539279
8028645	55588	*mediator complex subunit 29*	*MED29*	9.59	9.43	8.77	0.013702708	0.035414815
7949763				5.40	5.29	5.00	0.013707281	0.03542346
7962895	51303	*FK506 binding protein 11, 19 kDa*	*FKBP11*	9.06	9.48	8.31	0.013713243	0.035435695
8072482				5.20	5.22	5.53	0.013725009	0.035462927
8147994	84294		*UTP23*	7.03	7.21	8.60	0.013733201	0.035480917
8025488	7730	*zinc finger protein 177*	*ZNF177*	6.34	6.34	7.36	0.013734583	0.03548131
7916590	204	*adenylate kinase 2*	*AK2*	10.58	11.06	11.15	0.013739567	0.03549062
8066393	57158	*junctophilin 2*	*JPH2*	7.48	7.93	6.72	0.013740645	0.03549062
7981309	57596	*brain-enriched guanylate kinase-associated homolog (rat)*	*BEGAIN*	7.01	6.84	6.57	0.013743042	0.03549364
8154844				4.73	4.72	4.52	0.013745982	0.035498057
8059477	1286	*collagen, type IV, alpha 4*	*COL4A4*	6.54	6.67	6.22	0.013754367	0.03550933

8153101	169044	*collagen, type XXII, alpha 1*	*COL22A1*	7.39	7.47	6.99	0.01375475	0.03550933
8046276	389058	*Sp5 transcription factor*	*SP5*	8.51	8.60	7.69	0.013755268	0.03550933
8019731	6349, 414062, 6348	*chemokine (C-C motif) ligand 3-like 1 | chemokine (C-C motif) ligand 3-like 3 | chemokine (C-C motif) ligand 3*	*CCL3L1|CCL3L3*	7.93	7.95	7.40	0.013752316	0.03550933
8099850	80008	*transmembrane protein 156*	*TMEM156*	5.02	4.85	4.78	0.013757798	0.035509508
7986501				5.37	5.35	5.10	0.013757631	0.035509508
7959039	26259	*F-box and WD repeat domain containing 8*	*FBXW8*	9.27	9.47	8.75	0.013762837	0.035516165
8148867	90990, 8928	*kinesin family member C2 | forkhead box H1*	*KIFC2|FOXH1*	8.26	8.20	7.73	0.013762735	0.035516165
7977418	283643	*chromosome 14 open reading frame 80*	*C14orf80*	7.78	7.85	7.30	0.01377162	0.035535652
8065244				5.21	5.28	4.92	0.013772891	0.035535756
7934570	3778	*potassium large conductance calcium-activated channel, subfamily M, alpha member 1*	*KCNMA1*	8.57	8.79	7.32	0.013775188	0.035538506
8063369	55905	*ring finger protein 114*	*RNF114*	10.94	10.43	11.03	0.013784144	0.035557546
7915238	84618	*5'-nucleotidase, cytosolic IA*	*NT5C1A*	7.41	7.39	6.83	0.013786262	0.035557546
8144448	441328, 441317, 389633, 441315, 441324, 441327, 55138, 645572, 441332, 645558, 100287045	*family with sequence similarity 90, member A10 | family with sequence similarity 90, member A7 | family with sequence similarity 90 pseudogene | family with sequence similarity 90, member A5 | family with sequence similarity 90, member A8 | family with sequence similarity 90, member A9 | family with sequence similarity 90, member A1 | family with sequence similarity 90, member A23 pseudogene | family with sequence similarity 90, member A24 (pseudogene) | family with sequence similarity 90, member A22 pseudogene | family with sequence similarity 90, member A-like*	*FAM90A10|FAM90A7|FAM90A25P|FAM90A5|FAM90A8|FAM90A9|FAM90A23|FAM90A24P|FAM90A22|LOC100287045*	7.17	7.17	6.64	0.013785434	0.035557546
7963061	338761	*complement component 1, q subcomponent-like 4*	*C1QL4*	7.55	7.67	7.00	0.013797744	0.03558398
8126952	9589	*Wilms tumor 1 associated protein*	*WTAP*	11.30	11.43	12.39	0.013810012	0.035609256
8027439	22847	*zinc finger protein 507*	*ZNF507*	7.57	7.21	9.32	0.013809834	0.035609256
8086255	6336	*sodium channel, voltage-gated, type X, alpha subunit*	*SCN10A*	5.87	5.86	5.65	0.013815236	0.035619546
7940600	3619	*inner centromere protein antigens 135/155kDa*	*INCENP*	7.88	7.98	8.43	0.013829149	0.035650898

8153823	50626	*cysteine/histidine-rich 1*	*CYHR1*	8.66	8.53	7.62	0.013829866	0.035650898
7909586	5525	*protein phosphatase 2, regulatory subunit B', alpha*	*PPP2R5A*	8.97	8.52	9.45	0.013832632	0.035654847
8115397	10826	*chromosome 5 open reading frame 4*	*C5orf4*	10.54	9.49	8.40	0.013834659	0.035656884
8011331	4995	*olfactory receptor, family 3, subfamily A, member 2*	*OR3A2*	6.71	6.55	6.24	0.013840089	0.03566451
7999591	730013, 368, 653190	*ATP-binding cassette, sub-family C, member 6 pseudogene 2 | ATP-binding cassette, sub-family C (CFTR/MRP), member 6 | ATP-binding cassette, sub-family C, member 6 pseudogene 1*	*ABCC6P2| ABCC6P1*	8.20	8.10	7.74	0.01383922	0.03566451
8145555	51435	*scavenger receptor class A, member 3*	*SCARA3*	10.03	9.90	8.43	0.013850595	0.0356884
8067792	140700	*sterile alpha motif domain containing 10*	*SAMD10*	8.56	8.49	7.95	0.013853647	0.035693076
7983910	366	*aquaporin 9*	*AQP9*	5.41	5.54	5.27	0.013858653	0.035702787
8153762	55630	*solute carrier family 39 (zinc transporter), member 4*	*SLC39A4*	8.46	8.52	8.05	0.01386431	0.035706397
8024373	113177	*IZUMO family member 4*	*IZUMO4*	7.32	7.43	6.94	0.013864643	0.035706397
7937483	619565	*small nucleolar RNA, H/ACA box 52*	*SNORA52*	9.29	9.52	8.85	0.013865002	0.035706397
8132838				5.86	5.91	5.29	0.013862762	0.035706397
8017476	1443	*chorionic somatomammotropin hormone 2*	*CSH2*	7.36	7.31	6.54	0.013870111	0.03571637
8110631	153579	*butyrophilin-like 9*	*BTNL9*	6.86	6.90	6.43	0.013873114	0.03572092
7970569	26278	*spastic ataxia of Charlevoix-Saguenay (sacsin)*	*SACS*	8.26	7.93	10.16	0.013880944	0.03573789
8023828	81832	*neuropilin (NRP) and tolloid (TLL)-like 1*	*NETO1*	7.62	5.72	8.03	0.013887343	0.03575118
7956668				9.28	9.14	8.83	0.013891489	0.035758663
8065460	30813	*visual system homeobox 1*	*VSX1*	6.81	6.91	6.33	0.013894073	0.035762124
8004545	482	*ATPase, Na+/K+ transporting, beta 2 polypeptide*	*ATP1B2*	7.53	7.68	9.31	0.013902846	0.035779387
8114977	3360	*5-hydroxytryptamine (serotonin) receptor 4*	*HTR4*	5.80	5.87	5.57	0.013904497	0.035779387
8019088	100131170		*LOC100131170*	6.75	6.89	6.34	0.013904396	0.035779387
8077099	9997	*SCO cytochrome oxidase deficient homolog 2 (yeast)*	*SCO2*	8.15	8.28	7.59	0.013907732	0.03578452
8039730	65982	*zinc finger and SCAN domain containing 18*	*ZSCAN18*	8.47	8.23	7.86	0.013917196	0.03580568
8031176	114823	*leukocyte receptor cluster (LRC) member 8*	*LENG8*	10.07	9.80	9.24	0.013922421	0.03581593
8079401	1234	*chemokine (C-C motif) receptor 5*	*CCR5*	5.94	5.98	5.60	0.013923911	0.035816573

8177053	60439, 100101117, 50858, 100101116	*testis-specific transcript, Y-linked 2 (non-protein coding) | testis-specific transcript, Y-linked 2B (non-protein coding) | testis-specific transcript, Y-linked 1 (non-protein coding) | testis-specific transcript, Y-linked 1B (non-protein coding)*	*TTTY2|TTTY2B|TTTY1|TTTY1B*	5.40	5.52	5.16	0.013926181	0.03581922
8154229	3641	*insulin-like 4 (placenta)*	*INSL4*	5.14	5.16	5.02	0.013930241	0.03582647
8061883	51297	*palate, lung and nasal epithelium associated*	*PLUNC*	6.11	6.19	5.76	0.013936487	0.03583325
8047767				4.64	5.05	4.55	0.013935058	0.03583325
8117533				6.70	6.85	7.44	0.013936601	0.03583325
7965152				6.88	7.25	7.68	0.013943397	0.035847533
8088371	1776	*deoxyribonuclease I-like 3*	*DNASE1L3*	5.34	5.46	5.08	0.013945923	0.035850834
7965384	196477	*chromosome 12 open reading frame 12*	*C12orf12*	5.84	5.94	5.49	0.013963081	0.035891745
7941111	29907	*sorting nexin 15*	*SNX15*	8.50	8.60	7.64	0.013967507	0.035899926
7924233	7399	*Usher syndrome 2A (autosomal recessive, mild)*	*USH2A*	5.62	5.66	5.53	0.013972999	0.035901256
8101609	345274	*solute carrier family 10 (sodium/bile acid cotransporter family), member 6*	*SLC10A6*	5.99	6.23	5.89	0.013971836	0.035901256
7977642	440163	*ribonuclease, RNase A family, 13 (non-active)*	*RNASE13*	5.80	5.80	5.51	0.013972576	0.035901256
8071541	84222, 728229, 645426	*transmembrane protein 191A | transmembrane protein 191B | transmembrane protein 191C*	*TMEM191A|TMEM191B|TMEM191C*	10.17	10.05	9.41	0.013971207	0.035901256
8112302	643155	*chromosome 5 open reading frame 43*	*C5orf43*	7.62	7.56	8.48	0.013980158	0.03591645
7997740	81631	*microtubule-associated protein 1 light chain 3 beta*	*MAP1LC3B*	7.85	7.80	7.34	0.013991158	0.035941515
7950136	401	*paired-like homeobox 2a*	*PHOX2A*	7.05	7.22	6.56	0.013993165	0.03594347
8068202	59271	*chromosome 21 open reading frame 63*	*C21orf63*	7.95	7.75	7.33	0.013997663	0.035951827
8175685	4104	*melanoma antigen family A, 5*	*MAGEA5*	7.39	7.50	7.06	0.01400739	0.035973523
8044512	284958	*5'-nucleotidase domain containing 4*	*NT5DC4*	5.81	5.89	5.66	0.014008602	0.035973523
8015607	6774	*signal transducer and activator of transcription 3 (acute-phase response factor)*	*STAT3*	12.40	12.01	11.45	0.014012255	0.035976503
8029147	9138	*Rho guanine nucleotide exchange factor (GEF) 1*	*ARHGEF1*	8.59	8.56	7.71	0.014011356	0.035976503

8011724	284013	*vitelline membrane outer layer 1 homolog (chicken)*	*VMO1*	6.48	6.48	6.08	0.014019875	0.035992865
8064928	54363	*hydroxyacid oxidase (glycolate oxidase) 1*	*HAO1*	5.14	5.05	4.85	0.014022617	0.0359967
8113800	2201	*fibrillin 2*	*FBN2*	12.38	8.80	7.82	0.014030882	0.036000438
8109969	30012	*T-cell leukemia homeobox 3*	*TLX3*	7.02	6.97	6.41	0.014027353	0.036000438
8025183	57192	*mucolipin 1*	*MCOLN1*	10.25	10.25	9.37	0.014027819	0.036000438
7955974	58158	*neurogenic differentiation 4*	*NEUROD4*	6.28	6.22	5.85	0.014027411	0.036000438
8167880	90737	*P antigen family, member 5 (prostate associated)*	*PAGE5*	7.04	7.18	6.62	0.014030242	0.036000438
8174761	158801	*NFKB activating protein pseudogene 1*	*NKAPP1*	6.92	6.93	7.12	0.014031554	0.036000438
8178330	11074	*tripartite motif-containing 31*	*TRIM31*	6.17	6.35	5.85	0.014035285	0.036006812
8149104	1671	*defensin, alpha 6, Paneth cell-specific*	*DEFA6*	5.80	5.91	5.68	0.01404329	0.036014546
8098344	79804	*Nbla00301*	*NBLA0030 1*	6.71	6.82	5.67	0.014042417	0.036014546
7931582	196743	*polyamine oxidase (exo-N4-amino)*	*PAOX*	7.74	7.72	7.42	0.014041008	0.036014546
8038925	284370, 9668	*zinc finger protein 615 | zinc finger protein 432*	*ZNF615|Z NF432*	6.28	6.01	7.10	0.01404065	0.036014546
7946180	10612	*tripartite motif-containing 3*	*TRIM3*	7.95	8.04	7.40	0.014050526	0.03602977
8026696				6.03	6.21	5.60	0.014051723	0.03602977
8008172	124872	*beta-1,4-N-acetyl-galactosaminyl transferase 2*	*B4GALNT 2*	6.63	6.71	6.28	0.014063398	0.036056504
8103254	6423	*secreted frizzled-related protein 2*	*SFRP2*	8.77	12.08	10.30	0.014071746	0.0360747
7920047	65005	*mitochondrial ribosomal protein L9*	*MRPL9*	10.57	10.64	11.29	0.014074943	0.036079697
8125887	7005	*TEA domain family member 3*	*TEAD3*	9.97	10.22	9.21	0.014083257	0.036097802
8162254	54769	*DIRAS family, GTP-binding RAS-like 2*	*DIRAS2*	6.57	6.67	6.16	0.014088321	0.036105596
8046116	57818	*glucose-6-phosphatase, catalytic, 2*	*G6PC2*	5.55	5.48	5.32	0.01408922	0.036105596

7986642	100132979, 390535, 729786, 283768, 100132565, 653061, 100510725	*golgin A8 family, member D (pseudogene) | golgin A8 family, member E | golgin A8 family, member C | golgin A8 family, member G | golgin A8 family, member F | Golgin subfamily A member 8-like protein 2-like | putative golgin subfamily A member 8C-like*	*GOLGA8DP|GOLGA8E|GOLGA8C|GOLGA8G|GOLGA8F|LOC653061|LOC100510725*	6.68	6.67	6.56	0.01409005	0.036105596
8042942	3099	*hexokinase 2*	*HK2*	8.56	9.13	9.45	0.014093007	0.03610997
7939240				6.07	6.01	5.74	0.014094682	0.036111053
8101852	127	*alcohol dehydrogenase 4 (class II), pi polypeptide*	*ADH4*	4.96	4.86	4.79	0.014108809	0.03613504
8062927	5266	*peptidase inhibitor 3, skin-derived*	*PI3*	6.62	6.69	6.17	0.014107785	0.03613504
8072784	79734	*potassium channel tetramerisation domain containing 17*	*KCTD17*	9.10	9.22	8.34	0.014109051	0.03613504
8067585	128408	*basic helix-loop-helix family, member e23*	*BHLHE23*	8.44	8.45	7.73	0.014108874	0.03613504
8037847	1175	*adaptor-related protein complex 2, sigma 1 subunit*	*AP2S1*	10.55	10.66	9.60	0.01411116	0.03613618
8168366	5303	*protein (peptidylprolyl cis/trans isomerase) NIMA-interacting, 4 (parvulin)*	*PIN4*	6.86	6.76	7.51	0.014112283	0.03613618
8033674	94025	*mucin 16, cell surface associated*	*MUC16*	6.38	6.43	6.03	0.014113251	0.03613618
8016487	10481	*homeobox B13*	*HOXB13*	6.61	6.70	6.24	0.014116293	0.036140766
8172110	159013	*chromosome X open reading frame 38*	*CXorf38*	6.94	7.29	7.82	0.014121605	0.03615116
8006466	124842	*transmembrane protein 132E*	*TMEM132E*	7.24	7.30	6.60	0.014123778	0.036153514
7977928	1053	*CCAAT/enhancer binding protein (C/EBP), epsilon*	*CEBPE*	7.13	7.25	6.54	0.014136863	0.0361838
7902223				6.84	6.82	6.54	0.014146734	0.036205858
8044143	84417	*chromosome 2 open reading frame 40*	*C2orf40*	6.45	6.46	6.94	0.014165211	0.036249932
7947450	4005	*LIM domain only 2 (rhombotin-like 1)*	*LMO2*	6.67	6.66	6.24	0.014167844	0.036253456
7939329	8050	*pyruvate dehydrogenase complex, component X*	*PDHX*	9.65	9.19	10.09	0.014185522	0.036295474
8033362	3643	*insulin receptor*	*INSR*	7.88	8.19	9.00	0.014189268	0.036301844
8048551	10290		*SPEG*	7.58	8.18	7.12	0.014197706	0.036319576
7943777				5.06	5.15	4.86	0.014198716	0.036319576

8150860				7.71	7.81	7.39	0.01420286	0.036326956
8087380	7375	*ubiquitin specific peptidase 4 (proto-oncogene)*	*USP4*	10.56	10.56	10.10	0.014206276	0.03633183
8021275	11201	*polymerase (DNA directed) iota*	*POLI*	8.21	8.30	9.39	0.014209471	0.03633183
8078461	25827	*F-box and leucine-rich repeat protein 2*	*FBXL2*	8.60	7.15	7.62	0.014209722	0.03633183
8025035	84266	*alkB, alkylation repair homolog 7 (E. coli)*	*ALKBH7*	8.58	8.57	7.80	0.014209799	0.03633183
8077192	337	*apolipoprotein A-IV*	*APOA4*	7.06	7.11	6.64	0.014215117	0.036338992
8158240	7114, 7117, 7120	*thymosin beta 4, X-linked | thymosin-like 3 | thymosin-like 6 (pseudogene)*	*TMSB4X|TMSL3|TMSL6*	10.38	10.95	10.12	0.014214976	0.036338992
7902789	9653	*heparan sulfate 2-O-sulfotransferase 1*	*HS2ST1*	8.26	7.49	8.92	0.014236558	0.03639058
8000346	10595	*endoplasmic reticulum to nucleus signaling 2*	*ERN2*	6.84	6.84	6.50	0.014243475	0.036405038
7921298	79368	*Fc receptor-like 2*	*FCRL2*	5.40	5.43	5.24	0.014247824	0.03641293
8161319	26149, 401509, 653501	*zinc finger protein 658 | zinc finger protein 658B, pseudogene | zinc finger protein 658 pseudogene*	*ZNF658|ZNF658B|LOC653501*	6.26	6.04	6.85	0.014249324	0.036413535
8005836	23614	*pancreatic polypeptide 2*	*PPY2*	6.43	6.52	5.99	0.014253183	0.03641695
8145272	203069	*R3H domain and coiled-coil containing 1*	*R3HCC1*	9.99	9.77	8.92	0.014252511	0.03641695
7952735				4.69	4.55	4.54	0.014255308	0.036419157
8043981	7850	*interleukin 1 receptor, type II*	*IL1R2*	6.31	6.29	5.91	0.014273913	0.03644581
8083119	9616	*ring finger protein 7*	*RNF7*	8.60	8.81	9.09	0.014267756	0.03644581
8150846	137695	*transmembrane protein 68*	*TMEM68*	6.68	6.70	8.08	0.014274578	0.03644581
7918755	163259	*DENN/MADD domain containing 2C*	*DENND2C*	5.91	5.91	5.53	0.0142733	0.03644581
8037502	284353	*NTPase, KAP family P-loop domain containing 1*	*NKPD1*	7.74	7.77	7.29	0.014272673	0.03644581
8152314	340419	*R-spondin 2 homolog (Xenopus laevis)*	*RSPO2*	8.03	6.34	6.05	0.014272716	0.03644581
8162479	54754, 441457, 728118	*family with sequence similarity 22, member F | family with sequence similarity 22, member G | family with sequence similarity 22, member A*	*FAM22F|FAM22G|FAM22A*	6.90	7.04	6.66	0.014268857	0.03644581
7941879	374403	*TBC1 domain family, member 10C*	*TBC1D10C*	7.51	7.54	6.95	0.014281358	0.036456674
8062404				6.74	6.79	6.37	0.014280279	0.036456674

7979694				12.03	12.11	12.49	0.014284645	0.03646184
8124855	389376	*surfactant associated 2*	*SFTA2*	6.54	6.57	6.10	0.014286937	0.036464468
8167195	392459	*CXXC finger protein 1 pseudogene 1*	*CXXC1P1*	6.44	6.36	5.99	0.014294606	0.036480814
8012326	59344	*arachidonate lipoxygenase 3*	*ALOXE3*	6.53	6.55	6.08	0.014297547	0.036485095
8080896	115286	*solute carrier family 25, member 26*	*SLC25A26*	8.47	8.26	9.24	0.014299841	0.036487725
8092578	2119	*ets variant 5*	*ETV5*	8.12	9.56	8.31	0.014305618	0.03649924
7974799	4331	*menage a trois homolog 1, cyclin H assembly factor (Xenopus laevis)*	*MNAT1*	9.09	9.16	9.80	0.014311561	0.036511175
8015884	5539	*pancreatic polypeptide*	*PPY*	5.92	5.90	5.52	0.014317811	0.03651778
8144721	157773	*chromosome 8 open reading frame 48*	*C8orf48*	6.94	6.85	6.24	0.014317945	0.03651778
8082585	256076	*collagen, type VI, alpha 5*	*COL6A5*	4.95	5.04	4.77	0.014317694	0.03651778
7937709	440023	*keratin associated protein 5-6*	*KRTAP5-6*	6.93	6.94	6.61	0.0143209	0.03652209
8162352	55035	*nucleolar protein 8*	*NOL8*	7.48	7.19	8.41	0.014325761	0.036531262
8001327				4.79	4.78	4.63	0.014329234	0.03653689
7997179	1723	*dihydroorotate dehydrogenase*	*DHODH*	6.98	7.24	7.95	0.014335887	0.036550622
8006440	6354	*chemokine (C-C motif) ligand 7*	*CCL7*	5.89	5.97	5.64	0.014341733	0.0365623
7908529	56956	*LIM homeobox 9*	*LHX9*	6.58	9.23	6.07	0.014351925	0.03658182
8161346	26149, 401509, 653501	*zinc finger protein 658 | zinc finger protein 658B, pseudogene | zinc finger protein 658 pseudogene*	*ZNF658|ZNF658B|LOC653501*	6.21	6.00	6.79	0.014350692	0.03658182
7899774	3065	*histone deacetylase 1*	*HDAC1*	10.07	9.73	11.63	0.01436216	0.03660468
8140686	223117	*sema domain, immunoglobulin domain (Ig), short basic domain, secreted, (semaphorin) 3D*	*SEMA3D*	9.87	7.93	7.79	0.014368288	0.036617067
8038839	27181	*sialic acid binding Ig-like lectin 8*	*SIGLEC8*	6.38	6.51	5.97	0.014371044	0.036620587
8108483	83697	*solute carrier family 4, sodium bicarbonate cotransporter, member 9*	*SLC4A9*	6.46	6.47	6.21	0.014372207	0.036620587
7950409	10008	*potassium voltage-gated channel, Isk-related family, member 3*	*KCNE3*	6.53	6.45	6.08	0.014379081	0.036631953
7902400	26804, 5876	*small nucleolar RNA, C/D box 45B | Rab geranylgeranyltransferase, beta subunit*	*SNORD45B|RABGGTB*	7.86	8.14	8.49	0.014379206	0.036631953
8043995	3554	*interleukin 1 receptor, type I*	*IL1R1*	9.02	9.46	5.64	0.014386371	0.036640715

8112081	54505	*DEAH (Asp-Glu-Ala-His) box polypeptide 29*	*DHX29*	8.98	8.70	9.94	0.014386453	0.036640715
8146892				5.75	5.72	5.34	0.014385517	0.036640715
7981488	100288144		*LOC100288144*	7.94	7.91	7.39	0.014389203	0.036644485
7907572	60676	*pappalysin 2*	*PAPPA2*	6.29	7.83	6.10	0.014392509	0.036649674
7971239				7.24	7.31	6.37	0.014400551	0.03666692
8060320	140881	*defensin, beta 129*	*DEFB129*	5.82	5.73	5.40	0.014402753	0.036669288
8111136	54463	*family with sequence similarity 134, member B*	*FAM134B*	6.03	6.16	6.73	0.014407763	0.036678184
8104648				4.70	4.65	4.48	0.014408788	0.036678184
8154130				5.65	5.64	5.46	0.014422733	0.03671045
8098177	11275	*kelch-like 2, Mayven (Drosophila)*	*KLHL2*	6.18	6.29	7.28	0.01442506	0.03671313
8049544	9208	*leucine rich repeat (in FLII) interacting protein 1*	*LRRFIP1*	7.00	6.85	8.44	0.014428915	0.036719706
7972438	140432	*ring finger protein 113B*	*RNF113B*	6.58	6.73	6.19	0.014436999	0.036734167
8148930				7.96	7.94	7.60	0.014437143	0.036734167
8096075				4.38	4.37	4.35	0.014445493	0.036752176
8120300	90523	*chromosome 6 open reading frame 142*	*C6orf142*	5.20	5.25	4.93	0.014449118	0.03675816
8035648	53345	*transmembrane 6 superfamily member 2*	*TM6SF2*	9.84	9.08	7.89	0.014459601	0.036781587
7921462	391123, 284677	*V-set and immunoglobulin domain containing 8 | chromosome 1 open reading frame 204*	*VSIG8|C1orf204*	7.36	7.29	7.02	0.014474079	0.03681517
8152096	7534	*tyrosine 3-monooxygenase/tryptophan 5-monooxygenase activation protein, zeta polypeptide*	*YWHAZ*	11.30	11.34	12.31	0.014478665	0.03682359
7936937	7390	*uroporphyrinogen III synthase*	*UROS*	10.92	10.80	10.37	0.014486336	0.036839854
8157698	392392	*olfactory receptor, family 1, subfamily K, member 1*	*OR1K1*	6.56	6.52	6.14	0.01449063	0.036847025
8031568				7.56	7.65	6.98	0.014491708	0.036847025
8018494	91107	*tripartite motif-containing 47*	*TRIM47*	8.39	8.51	7.65	0.0144949	0.036851894
8061447	5834	*phosphorylase, glycogen; brain*	*PYGB*	10.09	10.25	9.19	0.014496414	0.036852118
8035408	5143, 729966	*phosphodiesterase 4C, cAMP-specific | hypothetical LOC729966*	*PDE4C|LOC729966*	7.28	7.45	6.99	0.014497541	0.036852118
8140398	7532	*tyrosine 3-monooxygenase/tryptophan 5-monooxygenase activation protein, gamma polypeptide*	*YWHAG*	9.85	10.31	10.93	0.014502775	0.03685893

8117580	8329	*histone cluster 1, H2ai*	*HIST1H2AI*	7.54	8.09	10.08	0.014501843	0.03685893
8076561	85368		*KIAA1654*	7.51	7.53	7.14	0.014507766	0.03686837
7898263	54751	*filamin binding LIM protein 1*	*FBLIM1*	8.49	8.65	7.71	0.014510832	0.036872916
7942717	4647	*myosin VIIA*	*MYO7A*	7.11	7.16	6.73	0.014517169	0.03687613
8110666	84851	*tripartite motif-containing 52*	*TRIM52*	7.19	7.20	9.27	0.014515042	0.03687613
8164627	401554		*FLJ46836*	7.10	7.10	6.68	0.014517206	0.03687613
8049729	375318, 653437	*aquaporin 12A | aquaporin 12B*	*AQP12A|AQP12B*	7.91	7.94	7.48	0.014517113	0.03687613
7983157	80021	*transmembrane protein 62*	*TMEM62*	8.93	9.06	8.17	0.014534371	0.036916483
8069168	386685	*keratin associated protein 10-12*	*KRTAP10-12*	8.30	8.22	7.61	0.014539609	0.036926538
8055287	653784	*mitotic spindle organizing protein 2A*	*MZT2A*	10.69	10.73	9.98	0.014540916	0.03692654
7924144	100131205, 6144	*ribosomal protein L21 pseudogene 28*	*RPL21P28*	10.92	11.07	11.30	0.014542168	0.03692654
7923608	5972	*renin*	*REN*	6.12	6.29	5.72	0.014546808	0.036931675
8051396	58484	*NLR family, CARD domain containing 4*	*NLRC4*	5.17	5.26	5.15	0.014549307	0.036931675
8094101	8532, 27201	*carboxypeptidase Z | G protein-coupled receptor 78*	*CPZ|GPR78*	9.72	9.18	8.37	0.014548761	0.036931675
7899287				9.09	9.09	8.54	0.014547458	0.036931675
7998307	124093	*coiled-coil domain containing 78*	*CCDC78*	7.24	7.33	6.80	0.01455874	0.03695237
8145193	541565	*chromosome 8 open reading frame 58*	*C8orf58*	8.99	8.97	8.13	0.014569874	0.036974695
8150868				5.28	5.48	4.94	0.014570097	0.036974695
7900585	4904	*Y box binding protein 1*	*YBX1*	12.63	12.67	13.07	0.014581944	0.037001505
7993265	51061	*thioredoxin domain containing 11*	*TXNDC11*	7.63	7.55	7.03	0.014586373	0.037009493
8028963	1555	*cytochrome P450, family 2, subfamily B, polypeptide 6*	*CYP2B6*	5.99	6.02	7.11	0.014588789	0.03701237
8131515				5.49	5.44	5.20	0.014599732	0.037036877
7974576	122830	*N(alpha)-acetyltransferase 30, NatC catalytic subunit*	*NAA30*	8.15	7.92	8.80	0.014615867	0.03707455
8157671	26219, 26740	*olfactory receptor, family 1, subfamily J, member 4 | olfactory receptor, family 1, subfamily J, member 2*	*OR1J4|OR1J2*	5.25	5.86	5.13	0.014620076	0.037081968
8095697	2919	*chemokine (C-X-C motif) ligand 1 (melanoma growth stimulating activity, alpha)*	*CXCL1*	7.13	7.63	6.95	0.014621832	0.037083168

8108472	5813	*purine-rich element binding protein A*	*PURA*	10.46	10.50	9.51	0.014624484	0.037086632
8170963	100132967	*non-protein coding RNA 204*	*NCRNA00 204*	5.74	5.57	5.23	0.014630592	0.037098862
8026051	10535	*ribonuclease H2, subunit A*	*RNASEH2 A*	8.04	8.51	9.84	0.01463509	0.037100494
8094974	54940	*OCIA domain containing 1*	*OCIAD1*	9.04	9.23	10.03	0.014634784	0.037100494
8032518	148252	*DIRAS family, GTP-binding RAS-like 1*	*DIRAS1*	8.91	9.42	8.28	0.014634047	0.037100494
7903889				5.40	5.64	5.49	0.014643268	0.037117966
7943195				6.49	6.50	5.96	0.014645812	0.037121154
7923907	3586	*interleukin 10*	*IL10*	5.96	5.97	5.71	0.014654139	0.03713574
8113660	6230	*ribosomal protein S25*	*RPS25*	9.23	9.20	9.54	0.01465298	0.03713574
8004385	57555	*neuroligin 2*	*NLGN2*	9.62	9.37	8.63	0.014662225	0.03715046
8128447	84539	*melanin-concentrating hormone receptor 2*	*MCHR2*	5.93	6.04	5.66	0.014662522	0.03715046
8018754	114757	*cytoglobin*	*CYGB*	9.04	8.73	7.63	0.014665792	0.037152883
8037363	199731	*cell adhesion molecule 4*	*CADM4*	7.45	7.55	8.15	0.014666052	0.037152883
7905512	100129271	*chromosome 1 open reading frame 68*	*C1orf68*	7.41	7.49	6.91	0.014671281	0.03716287
8106096				13.39	13.40	12.86	0.014675584	0.037170507
7917532	2634	*guanylate binding protein 2, interferon-inducible*	*GBP2*	6.98	6.73	5.06	0.014676958	0.037170727
8071595				7.20	7.14	6.73	0.01468029	0.0371759
8045972				4.94	4.88	4.70	0.014693614	0.03720638
8160712	441394	*suppressor of G2 allele of SKP1 (S. cerevisiae) pseudogene 1*	*SUGT1P1*	5.24	5.01	4.95	0.014695497	0.037207887
8124492	85236	*histone cluster 1, H2bk*	*HIST1H2B K*	11.21	11.13	10.22	0.014701297	0.037219305
8039975	100128185	*PNAS-19*	*LOC10012 8185*	4.87	4.79	4.68	0.01470527	0.0372261
7947861	6688	*spleen focus forming virus (SFFV) proviral integration oncogene spi1*	*SPI1*	7.37	7.50	6.98	0.014711555	0.037235495
8059014	54738		*FEV*	7.45	7.60	7.03	0.014711561	0.037235495
7940996	25824	*peroxiredoxin 5*	*PRDX5*	11.13	11.04	10.20	0.014724748	0.037261635
8129837	53832	*interleukin 20 receptor, alpha*	*IL20RA*	6.06	6.23	6.48	0.014725651	0.037261635

8005632				5.32	5.18	5.02	0.014725761	0.037261635
7998002	92822	*zinc finger protein 276*	*ZNF276*	7.89	8.09	7.25	0.01472961	0.037268106
7900597	79078	*chromosome 1 open reading frame 50*	*C1orf50*	7.58	7.87	7.99	0.014736256	0.037281655
8113591	5229	*protein geranylgeranyltransferase type I, beta subunit*	*PGGT1B*	7.32	7.30	8.92	0.014742797	0.03729167
8116031	345456	*profilin 3*	*PFN3*	7.52	7.57	7.20	0.014742636	0.03729167
8040053	55821	*allantoicase*	*ALLC*	5.60	5.63	5.37	0.014745506	0.037295252
8120860	594	*branched chain keto acid dehydrogenase E1, beta polypeptide*	*BCKDHB*	8.37	8.79	9.83	0.014754852	0.037315622
7950036	2248	*fibroblast growth factor 3*	*FGF3*	7.31	7.44	6.79	0.014758588	0.0373218
8139828				7.52	7.60	7.19	0.014763045	0.0373298
7982096	100033822	*small nucleolar RNA, C/D box 115-48*	*SNORD115-48*	4.51	4.52	4.48	0.014765133	0.037331812
8132286				5.65	5.63	5.27	0.014767178	0.037333712
7981051	51156	*serpin peptidase inhibitor, clade A (alpha-1 antiproteinase, antitrypsin), member 10*	*SERPINA10*	6.32	6.31	6.04	0.014772616	0.03734419
8059345	7857	*secretogranin II*	*SCG2*	5.32	5.50	5.12	0.014774878	0.03734664
7960575	4839		*NOP2*	8.36	8.57	9.50	0.01477762	0.0373503
7966266				6.70	6.70	6.08	0.014779004	0.037350528
8178802	3125, 3123	*major histocompatibility complex, class II, DR beta 3 | major histocompatibility complex, class II, DR beta 1*	*HLA-DRB3|HLA-DRB1*	6.66	6.55	7.64	0.01478544	0.03736352
7971039	55578	*family with sequence similarity 48, member A*	*FAM48A*	9.49	9.13	11.30	0.014795141	0.037384763
8179564	3833	*kinesin family member C1*	*KIFC1*	6.97	7.65	9.00	0.014806245	0.0374058
8058203	130540	*amyotrophic lateral sclerosis 2 (juvenile) chromosome region, candidate 12*	*ALS2CR12*	6.30	6.17	5.50	0.01480956	0.0374058
8040374	151354	*family with sequence similarity 84, member A*	*FAM84A*	7.17	7.54	6.90	0.01481124	0.0374058
7903854	388662	*solute carrier family 6, member 17*	*SLC6A17*	7.28	6.77	6.30	0.01481025	0.0374058
7933279	143162, 728798, 728603	*FERM and PDZ domain containing 2 | FERM and PDZ domain containing 2 pseudogene 1 | FERM and PDZ domain containing 2 pseudogene 2*	*FRMPD2|FRMPD2P1|FRMPDP2*	5.82	5.83	5.69	0.014805819	0.0374058
7982348				6.94	7.10	6.59	0.014810452	0.0374058

7946275	387751	*GTPase, very large interferon inducible pseudogene 1*	*GVINP1*	5.75	5.86	5.59	0.014815042	0.03741213
8086540	54716	*solute carrier family 6 (proline IMINO transporter), member 20*	*SLC6A20*	6.63	6.85	6.35	0.014829117	0.037444394
8058552	3417	*isocitrate dehydrogenase 1 (NADP+), soluble*	*IDH1*	10.62	10.03	12.18	0.014834049	0.0374503
7991695	100132979, 729786, 100132565, 283768, 390535, 653061, 100510725	*golgin A8 family, member D (pseudogene) | golgin A8 family, member C | golgin A8 family, member F | golgin A8 family, member G | golgin A8 family, member E | Golgin subfamily A member 8-like protein 2-like | putative golgin subfamily A member 8C-like*	*GOLGA8DP|GOLGA8C|GOLGA8F|GOLGA8G|GOLGA8E|LOC653061|LOC100510725*	6.96	6.95	6.85	0.014833796	0.0374503
8053715				4.90	4.84	4.69	0.014844184	0.03747261
7955635				6.54	6.68	6.14	0.014847578	0.0374779
7981750	400968		*LOC400968*	6.56	6.46	6.17	0.01484912	0.037478514
8010949	51031	*glyoxalase domain containing 4*	*GLOD4*	7.94	8.25	9.01	0.014853927	0.03748421
7976578	122481	*adenylate kinase 7*	*AK7*	5.39	5.30	5.87	0.014853974	0.03748421
8134915	7455	*zonadhesin*	*ZAN*	6.76	6.73	6.29	0.014855333	0.037484363
8124527	3009	*histone cluster 1, H1b*	*HIST1H1B*	8.52	10.08	12.16	0.014862211	0.037498444
7915156	5453	*POU class 3 homeobox 1*	*POU3F1*	8.60	8.58	8.07	0.01486813	0.037500266
7976506	388007	*serpin peptidase inhibitor, clade A (alpha-1 antiproteinase, antitrypsin), member 13 (pseudogene)*	*SERPINA13*	6.11	6.34	5.78	0.014864652	0.037500266
8080001	51409, 51161	*HemK methyltransferase family member 1 | chromosome 3 open reading frame 18*	*HEMK1|C3orf18*	8.45	8.17	7.56	0.014867003	0.037500266
8104447				6.33	6.49	5.84	0.014866246	0.037500266
7916120	51060	*thioredoxin domain containing 12 (endoplasmic reticulum)*	*TXNDC12*	11.73	11.94	12.56	0.014873101	0.037509527
8061572	84701	*cytochrome c oxidase subunit IV isoform 2 (lung)*	*COX4I2*	6.80	6.92	6.31	0.014876349	0.03751444
7995040	10295	*branched chain ketoacid dehydrogenase kinase*	*BCKDK*	10.13	10.28	9.26	0.014901426	0.037565336
8150364	83877	*TM2 domain containing 2*	*TM2D2*	7.92	7.95	8.79	0.014904339	0.037565336
7911301	267002	*piggyBac transposable element derived 2*	*PGBD2*	6.07	5.78	6.83	0.014903137	0.037565336

8110882	401172		*FLJ33360*	5.86	5.90	5.58	0.014900189	0.037565336
8011396	23729, 7442	*sedoheptulokinase | transient receptor potential cation channel, subfamily V, member 1*	*SHPK|TRP V1*	8.41	8.26	9.14	0.014901781	0.037565336
7950269				4.58	4.54	4.83	0.014898887	0.037565336
7925509				7.17	7.20	6.47	0.014907718	0.037570573
8116829				4.71	4.74	4.53	0.014909069	0.037570696
7962197				5.64	5.68	5.26	0.014910494	0.03757101
7947127				4.78	4.75	4.67	0.01492341	0.03760027
8178653	80736, 4758	*solute carrier family 44, member 4 | sialidase 1 (lysosomal sialidase)*	*SLC44A4| NEU1*	6.97	6.96	7.52	0.014932489	0.037619863
7984562	6176	*ribosomal protein, large, P1*	*RPLP1*	13.36	13.34	12.91	0.014934942	0.03762276
8010078	677850	*small nucleolar RNA, C/D box 1C*	*SNORD1C*	8.70	9.01	9.32	0.014948388	0.037653346
7942372	2350	*folate receptor 2 (fetal)*	*FOLR2*	5.80	5.89	5.51	0.014954981	0.037666667
7943943	255239	*ankyrin repeat and kinase domain containing 1*	*ANKK1*	7.26	7.35	6.90	0.014956967	0.037668385
7998878	64063	*protease, serine, 22*	*PRSS22*	7.57	7.58	7.17	0.014977053	0.037715677
7927482	1103	*choline O-acetyltransferase*	*CHAT*	6.89	6.76	6.47	0.014994024	0.037755124
7945130				8.02	8.08	7.61	0.014996452	0.037757944
8030813	284369	*chromosome 19 open reading frame 75*	*C19orf75*	5.75	5.75	5.36	0.015006137	0.037779033
8067029	3755	*potassium voltage-gated channel, subfamily G, member 1*	*KCNG1*	8.28	9.40	7.56	0.015009788	0.03778493
8082745	51554	*chemokine (C-C motif) receptor-like 1*	*CCRL1*	6.03	6.09	4.99	0.015015598	0.03779626
8005943	6147	*ribosomal protein L23a*	*RPL23A*	11.88	11.91	12.04	0.01502293	0.037811417
8135601	4233	*met proto-oncogene (hepatocyte growth factor receptor)*	*MET*	6.84	7.60	8.29	0.01503151	0.037829716
8114002	8974	*prolyl 4-hydroxylase, alpha polypeptide II*	*P4HA2*	6.42	6.33	6.15	0.015042968	0.037855253
8134431				10.00	10.10	9.45	0.015048477	0.037865814
8136932				4.95	4.93	5.21	0.01505605	0.037881568
8045664	130576	*LY6/PLAUR domain containing 6B*	*LYPD6B*	8.92	9.79	7.74	0.015059209	0.037886214
8076113	646851	*similar to OTTHUMP00000028720*	*LOC64685 1*	7.07	6.73	7.84	0.015072659	0.03791344

8179861	80736, 4758	*solute carrier family 44, member 4 | sialidase 1 (lysosomal sialidase)*	*SLC44A4| NEU1*	6.97	6.95	7.52	0.015072124	0.03791344
8143790	28959	*transmembrane protein 176B*	*TMEM176 B*	9.15	8.38	7.46	0.015075272	0.037916712
8117849	89870	*tripartite motif-containing 15*	*TRIM15*	7.47	7.45	7.08	0.015080562	0.03792341
7948399	219988	*protein associated with topoisomerase II homolog 1 (yeast)*	*PATL1*	11.01	11.24	10.16	0.015079692	0.03792341
8157804	169611	*olfactomedin-like 2A*	*OLFML2A*	8.59	8.18	7.61	0.015088405	0.037939828
8070286	10281	*Down syndrome critical region gene 4*	*DSCR4*	6.27	6.35	5.97	0.01509626	0.03794689
7946332	26496	*olfactory receptor, family 10, subfamily A, member 3*	*OR10A3*	5.37	5.40	5.20	0.01509604	0.03794689
8002975	124359	*chromodomain protein, Y-like 2*	*CDYL2*	7.92	7.91	7.28	0.015097787	0.03794689
7946113	390084	*olfactory receptor, family 56, subfamily A, member 5*	*OR56A5*	5.29	5.39	5.15	0.015097688	0.03794689
7980003				6.61	6.55	6.07	0.015092869	0.03794689
8105714	140890	*splicing regulatory glutamine/lysine-rich protein 1*	*SREK1*	6.96	6.66	8.11	0.015106721	0.037966043
7978239	5875	*Rab geranylgeranyltransferase, alpha subunit*	*RABGGTA*	8.98	9.05	8.02	0.015128974	0.03801866
7935647	1355		*COX15*	8.71	8.63	9.47	0.015138003	0.038034726
8094136	245911, 503694	*defensin, beta 108B | defensin, beta 108, pseudogene 1*	*DEFB108 B|DEFB10 8P1*	5.78	5.76	5.35	0.015137423	0.038034726
8166593	11141	*interleukin 1 receptor accessory protein-like 1*	*IL1RAPL1*	5.92	6.10	5.68	0.015139767	0.038035847
7997839	81620	*chromatin licensing and DNA replication factor 1*	*CDT1*	7.19	7.81	8.48	0.015141234	0.038036224
8062948	11317, 8785	*recombination signal binding protein for immunoglobulin kappa J region-like | matrilin 4*	*RBPJL|MA TN4*	7.14	7.12	6.59	0.015155011	0.038064208
8177449	9083, 442867, 442868	*basic charge, Y-linked, 2 | basic charge, Y-linked, 2B | basic charge, Y-linked, 2C*	*BPY2|BPY 2B|BPY2C*	5.54	5.55	5.32	0.015154119	0.038064208
8076424	1565	*cytochrome P450, family 2, subfamily D, polypeptide 6*	*CYP2D6*	7.84	7.96	7.55	0.015168744	0.03809539
8143879	83590	*transmembrane and ubiquitin-like domain containing 1*	*TMUB1*	9.91	9.91	9.13	0.015170377	0.038096175
8111998	348980	*hyperpolarization activated cyclic nucleotide-gated potassium channel 1*	*HCN1*	10.82	7.45	9.16	0.015175668	0.038106143
8166925	4128	*monoamine oxidase A*	*MAOA*	5.99	6.00	6.43	0.015178553	0.038110074
8031744	7565	*zinc finger protein 17*	*ZNF17*	7.24	7.26	8.19	0.015185002	0.038122952

7898161	79180	*EF-hand domain family, member D2*	*EFHD2*	9.03	9.15	7.92	0.01518997	0.03813211
7922912	7175	*translocated promoter region (to activated MET oncogene)*	*TPR*	7.41	7.13	9.40	0.015193149	0.038136773
8005117	353149, 254272, 57335	*TBC1 domain family, member 26 | TBC1 domain family, member 28 | zinc finger protein 286A*	*TBC1D26| TBC1D28| ZNF286A*	6.81	6.89	6.35	0.015196958	0.038143016
7994813	197407	*zinc finger protein 48*	*ZNF48*	6.68	6.78	7.76	0.015204386	0.038158342
7901256	1580	*cytochrome P450, family 4, subfamily B, polypeptide 1*	*CYP4B1*	6.12	6.24	5.95	0.015208658	0.038165722
7998931	7752	*zinc finger protein 200*	*ZNF200*	7.36	7.54	7.61	0.015210664	0.038165722
7948364	219972	*macrophage expressed 1*	*MPEG1*	6.30	6.22	5.75	0.015211293	0.038165722
7907308				4.65	4.69	4.50	0.015220233	0.038184837
8111569	202151	*RAN binding protein 3-like*	*RANBP3L*	4.98	5.29	4.92	0.015231979	0.038201027
8093453	401114		*FLJ35816*	6.10	6.03	5.79	0.015229971	0.038201027
7922418	619498	*small nucleolar RNA, C/D box 74*	*SNORD74*	11.62	11.68	11.93	0.015231179	0.038201027
8040415				8.99	9.02	8.28	0.015229828	0.038201027
7963845	117159	*dermcidin*	*DCD*	6.94	6.98	6.58	0.015236732	0.038209625
7979196	64841	*glucosamine-phosphate N-acetyltransferase 1*	*GNPNAT1*	8.31	8.52	9.62	0.015243801	0.038224034
7910229	375	*ADP-ribosylation factor 1*	*ARF1*	12.37	12.31	11.83	0.015250717	0.03823388
8147717	4956	*outer dense fiber of sperm tails 1*	*ODF1*	5.79	5.68	5.52	0.015251702	0.03823388
8112980	10085	*EGF-like repeats and discoidin I-like domains 3*	*EDIL3*	7.10	9.96	11.00	0.015251372	0.03823388
8007397	10197	*proteasome (prosome, macropain) activator subunit 3 (PA28 gamma; Ki)*	*PSME3*	10.76	10.95	11.61	0.015258557	0.038247745
8173232	139285	*family with sequence similarity 123B*	*FAM123B*	7.48	7.43	8.35	0.015270649	0.038274735
8110399	11285	*xylosylprotein beta 1,4-galactosyltransferase, polypeptide 7 (galactosyltransferase I)*	*B4GALT7*	9.73	9.63	8.81	0.015274255	0.038280446
8053107	1639	*dynactin 1*	*DCTN1*	10.86	10.72	10.02	0.015276649	0.038283125

8017173	440452, 729873, 84218, 414060, 654341, 729877, 653645, 414059, 100134348	*TBC1 domain family, member 3 pseudogene 2 | TBC1 domain family, member 3F | TBC1 domain family, member 3C | TBC1 domain family, member 3G | TBC1 domain family, member 3H | DEAH (Asp-Glu-Ala-His) box polypeptide 40 pseudogene 1 | TBC1 domain family, member 3B | similar to TBC1 domain family member 3 (Rab GTPase-activating protein PRC17) (Prostate cancer gene 17 protein) (TRE17 alpha protein)*	*TBC1D3P2|TBC1D3F|TBC1D3C|TBC1D3G|TBC1D3H|DHX40P1|TBC1D3B|LOC100134348*	8.02	7.92	7.31	0.015286824	0.038305297
8017511	974	*CD79b molecule, immunoglobulin-associated beta*	*CD79B*	7.72	7.04	6.71	0.015288613	0.038306452
8169638				5.88	5.79	5.10	0.015290995	0.038309097
7961182	3822, 3823	*killer cell lectin-like receptor subfamily C, member 2 | killer cell lectin-like receptor subfamily C, member 3*	*KLRC2|KLRC3*	4.55	4.58	4.43	0.015298942	0.038325682
8143040	84912	*solute carrier family 35, member B4*	*SLC35B4*	7.89	8.34	9.53	0.015312991	0.03835755
8125149	80736, 4758	*solute carrier family 44, member 4 | sialidase 1 (lysosomal sialidase)*	*SLC44A4|NEU1*	6.97	6.95	7.53	0.015315265	0.038359918
7980859	650662	*chromosome 14 open reading frame 184*	*C14orf184*	7.23	7.48	6.85	0.015318911	0.038365718
7923700	10446	*leucine rich repeat neuronal 2*	*LRRN2*	7.10	6.88	6.58	0.015321916	0.038369916
8085138	5021	*oxytocin receptor*	*OXTR*	8.96	8.50	7.09	0.015328473	0.038383007
8172274	641339	*zinc finger protein 674*	*ZNF674*	6.86	7.17	7.41	0.015331851	0.038388137
7930577	840	*caspase 7, apoptosis-related cysteine peptidase*	*CASP7*	7.86	7.95	7.08	0.015333832	0.03838977
8029939	29998	*glioma tumor suppressor candidate region gene 1*	*GLTSCR1*	8.57	8.42	7.55	0.015342603	0.038408395
8042986	5967	*regenerating islet-derived 1 alpha*	*REG1A*	5.37	5.57	5.20	0.015352393	0.03842957
8040238	3241	*hippocalcin-like 1*	*HPCAL1*	9.86	9.61	8.53	0.015358162	0.038437344
7904955				8.04	8.24	7.80	0.015357822	0.038437344
7901336	55437	*STE20-related kinase adaptor beta*	*STRADB*	10.88	10.56	11.15	0.015363201	0.038446624
8057744	6772	*signal transducer and activator of transcription 1, 91kDa*	*STAT1*	11.21	11.06	9.63	0.015388249	0.03850597
7987928	146059	*congenital dyserythropoietic anemia, type I*	*CDAN1*	8.08	8.06	7.35	0.015392133	0.03850901
8040514				6.62	6.86	6.82	0.015391273	0.03850901
8148692				6.44	6.51	6.08	0.015398518	0.038521647

8122843	2099	*estrogen receptor 1*	*ESR1*	6.38	6.47	6.01	0.015403718	0.03853132
7940358	22917	*zona pellucida glycoprotein 1 (sperm receptor)*	*ZP1*	6.63	6.63	6.19	0.01541139	0.03854717
8121209	5454	*POU class 3 homeobox 2*	*POU3F2*	7.47	7.69	7.07	0.015414455	0.038548157
7984660				10.39	10.69	9.79	0.015413621	0.038548157
7958275	55703	*polymerase (RNA) III (DNA directed) polypeptide B*	*POLR3B*	7.55	7.57	8.94	0.015427051	0.03857297
8050474	57665	*retinol dehydrogenase 14 (all-trans/9-cis/11-cis)*	*RDH14*	8.50	8.55	8.89	0.015426045	0.03857297
7898723				6.44	6.38	6.06	0.015439693	0.03860124
7908863				6.31	6.41	5.92	0.015451659	0.03862781
8066822	55959	*sulfatase 2*	*SULF2*	10.77	10.67	9.62	0.015463674	0.0386545
7906177	164118	*tetratricopeptide repeat domain 24*	*TTC24*	7.38	7.50	7.06	0.015472568	0.038670033
8117138				5.09	5.16	4.88	0.015471774	0.038670033
8027239	7608	*zinc finger protein 56*	*ZNF56*	5.32	5.27	6.53	0.015477068	0.03867793
7992021	10273	*STIP1 homology and U-box containing protein 1, E3 ubiquitin protein ligase*	*STUB1*	9.27	9.32	8.37	0.015481985	0.038685154
8136977				6.15	6.16	5.84	0.015482638	0.038685154
8128327				7.15	7.11	6.49	0.015493054	0.03870448
8152551				6.05	5.96	5.43	0.015492103	0.03870448
7949227	55561	*CDC42 binding protein kinase gamma (DMPK-like)*	*CDC42BPG*	7.38	7.43	7.13	0.015500965	0.038710844
8069822	337882	*keratin associated protein 19-1*	*KRTAP19-1*	5.79	5.78	5.40	0.015500167	0.038710844
8132493	100127950		*LOC100127950*	5.71	5.78	6.33	0.015498366	0.038710844
8173977				5.50	5.70	5.93	0.015497793	0.038710844
8080762	57406	*abhydrolase domain containing 6*	*ABHD6*	8.00	8.31	7.51	0.015512673	0.038736604
8084810				5.76	5.88	5.27	0.015513964	0.038736604
8010071	15	*aralkylamine N-acetyltransferase*	*AANAT*	6.97	7.11	6.47	0.015523072	0.038747996
8016980	406934	*microRNA 142*	*MIR142*	5.29	5.04	4.96	0.015523896	0.038747996
7945177	440072	*non-protein coding RNA 167*	*NCRNA00167*	6.48	6.60	6.04	0.015523526	0.038747996
7921425				5.91	6.07	5.57	0.015521401	0.038747996

8108949	5459	*POU class 4 homeobox 3*	*POU4F3*	7.26	7.28	6.71	0.015530593	0.038761362
8006594	6362	*chemokine (C-C motif) ligand 18 (pulmonary and activation-regulated)*	*CCL18*	6.41	6.45	6.13	0.015535201	0.03876616
8082165	8997	*kalirin, RhoGEF kinase*	*KALRN*	6.08	6.57	6.34	0.015534389	0.03876616
8137151	23145	*SCO-spondin homolog (Bos taurus)*	*SSPO*	7.84	7.81	7.30	0.015541016	0.03877732
8061799	128859	*bactericidal/permeability-increasing protein-like 3*	*BPIL3*	6.73	6.76	6.34	0.015543547	0.038780283
7923628	127845	*golgi transport 1A*	*GOLT1A*	6.95	6.99	7.32	0.015549383	0.038791493
8169492	186	*angiotensin II receptor, type 2*	*AGTR2*	4.98	5.09	4.82	0.015552837	0.038796756
8030980	170958, 91664, 91661	*zinc finger protein 525 | zinc finger protein 845 | zinc finger protein 765*	*ZNF525|ZNF845|ZNF765*	10.22	9.84	11.66	0.015555578	0.03880024
7983594	1854	*deoxyuridine triphosphatase*	*DUT*	7.88	7.96	8.62	0.015565193	0.03882087
7929373	9211	*leucine-rich, glioma inactivated 1*	*LGI1*	5.47	5.43	5.08	0.015575105	0.038842235
7965964	84102	*solute carrier family 41, member 2*	*SLC41A2*	6.81	7.73	6.72	0.015577989	0.03884607
8108453	84249	*pleckstrin and Sec7 domain containing 2*	*PSD2*	6.58	6.73	6.25	0.015587605	0.03886669
8086406	9117	*SEC22 vesicle trafficking protein homolog C (S. cerevisiae)*	*SEC22C*	9.56	9.80	9.13	0.015589382	0.038867764
8060772	8760	*CDP-diacylglycerol synthase (phosphatidate cytidylyltransferase) 2*	*CDS2*	10.23	10.21	10.64	0.015590983	0.0388684
7994091				5.35	5.38	5.07	0.015592459	0.038868725
7920725	10067	*secretory carrier membrane protein 3*	*SCAMP3*	9.66	9.77	8.62	0.015600267	0.03888483
8089259				5.55	5.53	5.36	0.015608541	0.038902096

8149204	441317, 441328, 728430, 389633, 441315, 441327, 55138, 441324, 645572, 441332, 645558, 100287045	*family with sequence similarity 90, member A7 | family with sequence similarity 90, member A10 | family with sequence similarity 90, member A20 | family with sequence similarity 90 pseudogene | family with sequence similarity 90, member A5 | family with sequence similarity 90, member A9 | family with sequence similarity 90, member A1 | family with sequence similarity 90, member A8 | family with sequence similarity 90, member A23 pseudogene | family with sequence similarity 90, member A24 (pseudogene) | family with sequence similarity 90, member A22 pseudogene | family with sequence similarity 90, member A-like*	*FAM90A7|FAM90A10|FAM90A20|FAM90A25P|FAM90A5|FAM90A9|FAM90A8|FAM90A23|FAM90A24P|FAM90A22|LOC100287045*	7.26	7.29	6.75	0.015611257	0.038905505
8033151	125981	*alkaline ceramidase 1*	*ACER1*	7.13	7.11	6.34	0.015616704	0.03891572
8149383	389633, 441317, 55138, 441315, 441324, 441327, 441328, 645572	*family with sequence similarity 90 pseudogene | family with sequence similarity 90, member A7 | family with sequence similarity 90, member A1 | family with sequence similarity 90, member A5 | family with sequence similarity 90, member A8 | family with sequence similarity 90, member A9 | family with sequence similarity 90, member A10 | family with sequence similarity 90, member A23 pseudogene*	*FAM90A25P|FAM90A7|FAM90A1|FAM90A5|FAM90A8|FAM90A9|FAM90A10|FAM90A23*	7.17	7.20	6.75	0.015620373	0.038921505
7941597				5.85	5.72	5.46	0.015627576	0.03893609
7915404	2980	*guanylate cyclase activator 2A (guanylin)*	*GUCA2A*	7.11	7.24	6.63	0.015635105	0.038938046
7927972	9559	*vacuolar protein sorting 26 homolog A (S. pombe)*	*VPS26A*	8.38	8.38	8.86	0.015633892	0.038938046
7970696	219333	*ubiquitin specific peptidase 12*	*USP12*	8.10	7.68	8.88	0.015631476	0.038938046
7897978	65121, 400736, 729528, 65122	*PRAME family member 1 | PRAME family member 13 | PRAME family member 14 | PRAME family member 2*	*PRAMEF1|PRAMEF13|PRAMEF14|PRAMEF2*	5.14	5.18	5.02	0.015630761	0.038938046
7936062				7.28	6.97	6.75	0.015633529	0.038938046

8019711	147199, 653486	*secretoglobin, family 1C, member 1 | secretoglobin, family 1C, member 1-like*	*SCGB1C1|LOC653486*	7.30	7.15	6.79	0.015639493	0.038945615
8028546	3963, 653499	*lectin, galactoside-binding, soluble, 7 | lectin, galactoside-binding, soluble, 7B*	*LGALS7|LGALS7B*	7.67	7.73	7.25	0.015641753	0.038947884
8058063	130132	*raftlin family member 2*	*RFTN2*	8.36	7.83	5.93	0.015644	0.038950123
8042391	5341	*pleckstrin*	*PLEK*	4.94	4.91	4.80	0.015646847	0.03895385
7971163				7.64	7.64	8.55	0.015650516	0.038959622
7931623	619207	*scavenger receptor protein family member*	*LOC619207*	7.04	7.13	6.81	0.015653903	0.038964693
8033780	79088	*zinc finger protein 426*	*ZNF426*	8.28	8.15	9.95	0.015661662	0.038980648
7968270	432369	*ATP synthase, H+ transporting, mitochondrial F1 complex, epsilon subunit pseudogene 2*	*ATP5EP2*	10.86	10.96	10.70	0.015663365	0.038981523
8024282	26528	*DAZ associated protein 1*	*DAZAP1*	9.25	9.38	10.32	0.015665503	0.038983487
8008040	90506	*leucine rich repeat containing 46*	*LRRC46*	6.78	6.89	6.02	0.015671195	0.03899429
8074771	85376, 440804, 150221	*RIMS binding protein 3 | RIMS binding protein 3B | RIMS binding protein 3C*	*RIMBP3|RIMBP3B|RIMBP3C*	7.33	7.25	6.71	0.015673548	0.038996782
7977820	10419	*protein arginine methyltransferase 5*	*PRMT5*	10.51	10.62	11.52	0.01568145	0.039013077
7952406	219858	*olfactory receptor, family 8, subfamily B, member 12*	*OR8B12*	5.70	5.72	5.46	0.015683277	0.03901426
8039453	284417	*transmembrane protein 150B*	*TMEM150B*	7.31	7.45	6.88	0.015693765	0.03903699
8082347	285311	*chromosome 3 open reading frame 56*	*C3orf56*	5.97	5.89	5.58	0.015707163	0.03906695
8019181	284187		*DKFZp761P0212*	7.82	7.94	7.35	0.015708627	0.039067227
8110606	79908	*butyrophilin-like 8*	*BTNL8*	5.74	5.93	5.49	0.015710058	0.039067417
7992010	9028	*rhomboid, veinlet-like 1 (Drosophila)*	*RHBDL1*	7.90	7.88	7.56	0.01571565	0.039074592
8047563				5.20	5.12	5.51	0.015715363	0.039074592
7924832	7483	*wingless-type MMTV integration site family, member 9A*	*WNT9A*	8.03	8.06	7.42	0.015720867	0.039084196
8126394	55173	*mitochondrial ribosomal protein S10*	*MRPS10*	8.34	8.57	9.51	0.015740383	0.039125543
7930861	255082	*cancer susceptibility candidate 2*	*CASC2*	7.08	7.12	6.56	0.015741562	0.039125543
8161169				11.25	11.31	11.54	0.015741125	0.039125543

7942779				8.65	8.77	7.44	0.015744865	0.039130382
8012539	146850	*phosphoinositide-3-kinase, regulatory subunit 6*	*PIK3R6*	6.71	6.75	6.14	0.01576058	0.039160468
7908525	388722	*chromosome 1 open reading frame 53*	*C1orf53*	8.43	8.97	7.68	0.015758393	0.039160468
7969770				6.04	6.09	5.63	0.01576104	0.039160468
8003733	9905	*small G protein signaling modulator 2*	*SGSM2*	8.83	8.52	7.96	0.015790189	0.039229516
8101624	57563	*kelch-like 8 (Drosophila)*	*KLHL8*	6.23	6.51	7.22	0.015792282	0.03923134
7927215	240	*arachidonate 5-lipoxygenase*	*ALOX5*	6.77	6.94	7.79	0.015799588	0.039235983
8125328	10554	*1-acylglycerol-3-phosphate O-acyltransferase 1 (lysophosphatidic acid acyltransferase, alpha)*	*AGPAT1*	11.17	11.11	10.31	0.01579571	0.039235983
8072039	84700	*myosin XVIIIB*	*MYO18B*	6.62	6.68	6.29	0.015797425	0.039235983
8074249				5.19	5.15	4.98	0.015799234	0.039235983
8170695	1852	*dual specificity phosphatase 9*	*DUSP9*	7.95	7.92	7.51	0.015803393	0.039238695
7989245	664618	*heat shock protein 90kDa alpha (cytosolic), class B member 4 (pseudogene)*	*HSP90AB4P*	5.99	6.11	6.52	0.015803399	0.039238695
8083032				13.62	13.75	13.57	0.015807195	0.039244745
8115205	134466	*zinc finger protein 300 pseudogene 1*	*ZNF300P1*	5.60	5.35	6.70	0.015819056	0.03927082
8060294	5133	*programmed cell death 1*	*PDCD1*	7.83	7.97	7.48	0.015822044	0.03927148
8160295	677776	*small Cajal body-specific RNA 8*	*SCARNA8*	6.66	6.91	7.10	0.015821	0.03927148
7942569	11309	*solute carrier organic anion transporter family, member 2B1*	*SLCO2B1*	6.07	6.24	5.70	0.015824562	0.039274354
8070239	3141	*holocarboxylase synthetase (biotin-(proprionyl-CoA-carboxylase (ATP-hydrolysing)) ligase)*	*HLCS*	8.70	9.06	8.32	0.015827172	0.039277453
8117707	729583	*chromosome 6 open reading frame 100*	*C6orf100*	5.84	6.01	5.68	0.015836326	0.039296795
8120501	1310	*collagen, type XIX, alpha 1*	*COL19A1*	6.21	6.32	5.86	0.01584637	0.039318338
8124510	8340	*histone cluster 1, H2bl*	*HIST1H2BL*	6.97	7.38	8.53	0.015850263	0.0393193
8130447	50508	*NADPH oxidase 3*	*NOX3*	5.90	5.94	5.52	0.015850844	0.0393193
8103362				4.88	4.89	4.68	0.015850166	0.0393193
8178762	10554	*1-acylglycerol-3-phosphate O-acyltransferase 1 (lysophosphatidic acid acyltransferase, alpha)*	*AGPAT1*	11.17	11.11	10.31	0.015855672	0.039327897
8050813	1788	*DNA (cytosine-5-)-methyltransferase 3 alpha*	*DNMT3A*	8.21	8.01	8.45	0.015867412	0.039350256

7905677	148327	*cAMP responsive element binding protein 3-like 4*	*CREB3L4*	6.83	6.96	7.47	0.015866442	0.039350256
7928823	94233	*opsin 4*	*OPN4*	7.44	7.44	7.08	0.015873043	0.039359514
8093578	339983	*N-acetyltransferase 8-like (GCN5-related, putative)*	*NAT8L*	7.62	7.57	7.15	0.015873872	0.039359514
8080084	7873	*mesencephalic astrocyte-derived neurotrophic factor*	*MANF*	9.43	9.81	9.92	0.015879689	0.039367173
7943345				6.45	6.53	6.11	0.015878774	0.039367173
7898030	645425, 391001	*PRAME family member 20 | PRAME family member 21*	*PRAMEF20|PRAMEF21*	7.00	7.26	6.65	0.0158837	0.03937233
8079999				5.39	5.34	5.08	0.015884496	0.03937233
7987466				7.25	7.30	6.48	0.015888164	0.03937804
8017378	1534	*cytochrome b-561*	*CYB561*	8.05	7.48	6.65	0.015899513	0.03939805
8128626	57107	*prenyl (decaprenyl) diphosphate synthase, subunit 2*	*PDSS2*	6.50	6.39	7.34	0.015900332	0.03939805
8177760	89870	*tripartite motif-containing 15*	*TRIM15*	7.47	7.44	7.08	0.01589898	0.03939805
7955464	9802	*DAZ associated protein 2*	*DAZAP2*	11.56	11.74	10.11	0.01590554	0.039407574
8150318	2260	*fibroblast growth factor receptor 1*	*FGFR1*	11.16	10.49	10.72	0.015910618	0.03941677
7981059	866	*serpin peptidase inhibitor, clade A (alpha-1 antiproteinase, antitrypsin), member 6*	*SERPINA6*	5.74	5.80	5.58	0.015927251	0.039454594
8165011	2219	*ficolin (collagen/fibrinogen domain containing) 1*	*FCN1*	7.15	7.11	6.67	0.015929475	0.039456718
8005747				6.91	6.85	6.11	0.015936049	0.03946961
7941685	9973	*copper chaperone for superoxide dismutase*	*CCS*	8.85	8.75	8.18	0.01593926	0.039474178
8161326	26149, 401509, 653501	*zinc finger protein 658 | zinc finger protein 658B, pseudogene | zinc finger protein 658 pseudogene*	*ZNF658|ZNF658B|LOC653501*	6.42	6.21	7.01	0.01594848	0.039493624
8023696	6317	*serpin peptidase inhibitor, clade B (ovalbumin), member 3*	*SERPINB3*	5.38	5.31	6.95	0.015955051	0.03950399
8117583	8329, 8357	*histone cluster 1, H2ai | histone cluster 1, H3h*	*HIST1H2AI|HIST1H3H*	7.68	8.36	10.17	0.015957085	0.03950399
7934991				4.49	4.43	4.45	0.015957376	0.03950399
7998053				6.59	6.43	6.15	0.01595814	0.03950399
8071715				6.44	6.52	6.11	0.015959518	0.039504014

7989473	388125	*C2 calcium-dependent domain containing 4B*	*C2CD4B*	8.15	8.37	7.73	0.015963178	0.039509688
8026729	79016	*DET1 and DDB1 associated 1*	*DDA1*	11.33	11.43	10.62	0.015967827	0.039517805
7925976				6.48	6.39	5.89	0.015990306	0.039570045
8095430	3347	*histatin 3*	*HTN3*	4.86	4.76	4.81	0.015999625	0.03958971
8179958	10554	*1-acylglycerol-3-phosphate O-acyltransferase 1 (lysophosphatidic acid acyltransferase, alpha)*	*AGPAT1*	11.17	11.11	10.31	0.016002532	0.03959012
8008566	252983	*syntaxin binding protein 4*	*STXBP4*	7.31	6.88	8.47	0.01600118	0.03959012
7971208	89890	*kelch repeat and BTB (POZ) domain containing 6*	*KBTBD6*	7.58	7.49	7.93	0.01600753	0.03959909
7952785	4978	*opioid binding protein/cell adhesion molecule-like*	*OPCML*	6.05	7.90	6.98	0.016014554	0.039606284
7987475	5330	*phospholipase C, beta 2*	*PLCB2*	6.26	6.33	6.66	0.016013946	0.039606284
7903404	55599	*RNA-binding region (RNP1, RRM) containing 3*	*RNPC3*	8.71	8.35	9.43	0.016013999	0.039606284
8114030	11127	*kinesin family member 3A*	*KIF3A*	6.69	6.52	7.54	0.016016463	0.03960761
8015376	3868	*keratin 16*	*KRT16*	7.23	7.44	6.94	0.016019577	0.03961192
8104781	51289	*relaxin/insulin-like family peptide receptor 3*	*RXFP3*	7.90	7.96	7.35	0.01602185	0.03961415
7991323	8800	*peroxisomal biogenesis factor 11 alpha*	*PEX11A*	6.90	6.99	6.56	0.016023343	0.039614446
7947894	10658	*CUGBP, Elav-like family member 1*	*CELF1*	9.78	9.93	11.06	0.01602876	0.039624017
8005501	201158, 51030	*family with sequence similarity 18, member B2 | family with sequence similarity 18, member B1*	*FAM18B2| FAM18B1*	8.65	8.52	8.93	0.01602996	0.039624017
8120943	51167	*cytochrome b5 reductase 4*	*CYB5R4*	7.96	7.82	8.46	0.016041415	0.03964894
8140371	83862	*transmembrane protein 120A*	*TMEM120 A*	9.59	9.56	8.36	0.016049799	0.039666265
7977782				6.39	6.41	6.20	0.016058704	0.039684877
8100990	10895	*pro-platelet basic protein-like 2*	*PPBPL2*	5.91	5.83	5.62	0.016068181	0.039704897
8066697	51006	*solute carrier family 35, member C2*	*SLC35C2*	9.57	9.48	8.82	0.016083768	0.03974001
7946641	374378	*UDP-N-acetyl-alpha-D-galactosamine:polypeptide N-acetylgalactosaminyltransferase-like 4*	*GALNTL4*	6.43	6.44	6.74	0.016091842	0.03975656
7997556	123872, 9013	*leucine rich repeat containing 50 | TATA box binding protein (TBP)-associated factor, RNA polymerase I, C, 110kDa*	*LRRC50|T AF1C*	6.54	6.51	6.24	0.016096897	0.039765645
8120431	7803	*protein tyrosine phosphatase type IVA, member 1*	*PTP4A1*	9.61	9.88	10.29	0.01609962	0.039767694
8036686	115290	*F-box protein 17*	*FBXO17*	8.88	8.92	7.99	0.016100481	0.039767694

8049959	401045		*FLJ41327*	6.86	6.84	6.47	0.01610535	0.039776314
8021993				4.93	4.78	5.05	0.016107963	0.039779365
8071161	729461, 26080, 653203	*hypothetical LOC729461 | non-protein coding RNA 281 | similar to hypothetical gene supported by BC039313*	*LOC729461|NCRNA00281|LOC653203*	5.71	5.90	5.36	0.016112423	0.039786976
7961865	3845, 144363	*v-Ki-ras2 Kirsten rat sarcoma viral oncogene homolog | LYR motif containing 5*	*KRAS|LYRM5*	7.52	7.29	9.40	0.016116513	0.03979367
8087145	1951	*cadherin, EGF LAG seven-pass G-type receptor 3 (flamingo homolog, Drosophila)*	*CELSR3*	7.23	7.24	7.75	0.016125727	0.039799403
8051762	6546	*solute carrier family 8 (sodium/calcium exchanger), member 1*	*SLC8A1*	8.43	8.02	6.21	0.016124688	0.039799403
8022941	25941	*chromosome 18 open reading frame 10*	*C18orf10*	12.01	11.94	11.72	0.016125064	0.039799403
7916783				4.54	4.60	4.47	0.016120689	0.039799403
8022623				7.64	7.58	7.14	0.016121613	0.039799403
7974653	9786		*KIAA0586*	7.67	7.42	8.86	0.016131856	0.039811123
7940508	747	*diacylglycerol lipase, alpha*	*DAGLA*	8.58	8.50	7.43	0.016134225	0.039813567
8078046	84077	*chromosome 3 open reading frame 20*	*C3orf20*	6.15	6.31	5.81	0.016140094	0.039824646
8100476				4.94	5.05	4.63	0.016142853	0.03982805
7957861	121601	*anoctamin 4*	*ANO4*	5.94	6.15	6.75	0.016145315	0.039830722
8004880	9423	*netrin 1*	*NTN1*	9.58	9.30	8.83	0.016148519	0.039835222
8026483	26538	*olfactory receptor, family 10, subfamily H, member 2*	*OR10H2*	5.71	5.83	5.33	0.01615345	0.039841503
8119132	221472	*FYVE, RhoGEF and PH domain containing 2*	*FGD2*	6.74	6.82	6.34	0.016153825	0.039841503
8083129	131890	*G protein-coupled receptor kinase 7*	*GRK7*	6.17	6.35	5.77	0.016156677	0.03984513
8015163	85293	*keratin associated protein 3-3*	*KRTAP3-3*	5.17	5.06	4.87	0.016163414	0.03985805
8178508	534, 7919	*ATPase, H+ transporting, lysosomal 13kDa, V1 subunit G2 | HLA-B associated transcript 1*	*ATP6V1G2|BAT1*	8.19	7.66	7.88	0.016164677	0.03985805
8167103	5127	*cyclin-dependent kinase 16*	*CDK16*	11.19	11.52	10.30	0.016166376	0.039858837
7992038	79006	*meteorin, glial cell differentiation regulator*	*METRN*	8.60	8.69	7.88	0.016175648	0.03987829
7912784	114819	*ciliary rootlet coiled-coil, rootletin pseudogene 3*	*CROCCP3*	7.20	7.21	6.94	0.016193429	0.03991262
8138392	155465	*anterior gradient homolog 3 (Xenopus laevis)*	*AGR3*	4.54	4.57	4.46	0.016193125	0.03991262

8176558	60439, 100101117, 50858, 100101116	*testis-specific transcript, Y-linked 2 (non-protein coding) | testis-specific transcript, Y-linked 2B (non-protein coding) | testis-specific transcript, Y-linked 1 (non-protein coding) | testis-specific transcript, Y-linked 1B (non-protein coding)*	*TTTY2|TTTY2B|TTTY1|TTTY1B*	5.40	5.52	5.18	0.01619372	0.03991262
7976200	801	*calmodulin 1 (phosphorylase kinase, delta)*	*CALM1*	9.18	9.45	10.02	0.016198585	0.0399212
7953333	939		*CD27*	7.11	7.07	6.64	0.016202856	0.039928317
8084986	84248	*forty-two-three domain containing 1*	*FYTTD1*	7.85	7.92	7.17	0.016205076	0.03993038
7950628	92105	*integrator complex subunit 4*	*INTS4*	8.43	8.56	9.56	0.016209234	0.039937217
7916654	163782	*KN motif and ankyrin repeat domains 4*	*KANK4*	6.62	6.77	6.31	0.016215464	0.039949156
7993898	146177	*von Willebrand factor A domain containing 3A*	*VWA3A*	6.09	6.01	5.85	0.01622152	0.039960667
7898869	259283	*myelodysplastic syndrome 2 translocation associated*	*MDS2*	6.72	6.92	6.31	0.016225915	0.039968085
8025321	440508	*C-type lectin domain family 4, member G pseudogene 1*	*CLEC4GP1*	8.33	8.38	8.06	0.0162298	0.039970834
8034871				9.59	9.69	8.83	0.016229782	0.039970834
7901443				7.47	7.32	7.10	0.016240733	0.039994344
8074737	729461, 26080	*hypothetical LOC729461 | non-protein coding RNA 281*	*LOC729461|NCRNA00281*	5.96	6.07	5.63	0.016251542	0.04001755
7905020	1816	*dopamine receptor D5*	*DRD5*	7.20	7.30	6.85	0.016254004	0.0400202
7903945	27159	*chitinase, acidic*	*CHIA*	6.08	6.14	5.67	0.016257212	0.04002127
7973873				5.33	5.28	5.08	0.016256195	0.04002127
8168274	2705	*gap junction protein, beta 1, 32kDa*	*GJB1*	6.44	6.53	6.20	0.01626102	0.040025022
8101721				5.20	4.87	4.71	0.016261509	0.040025022
8150962	9760	*thymocyte selection-associated high mobility group box*	*TOX*	10.33	8.24	9.61	0.016282383	0.040063843
8045933	10010	*TRAF family member-associated NFKB activator*	*TANK*	8.46	8.05	7.38	0.016282832	0.040063843
8117243	55604	*leucine rich repeat containing 16A*	*LRRC16A*	9.43	8.57	10.78	0.016278978	0.040063843
7948330	219968	*olfactory receptor, family 5, subfamily B, member 21*	*OR5B21*	5.70	5.53	5.46	0.016281666	0.040063843
8158987	1057	*carboxyl ester lipase pseudogene*	*CELP*	7.63	7.81	7.06	0.016291587	0.040081967
8151236				5.83	5.66	5.36	0.016296022	0.040089462
7950532				4.60	4.53	4.46	0.016298447	0.040092014

8142687	2861	*G protein-coupled receptor 37 (endothelin receptor type B-like)*	*GPR37*	6.34	7.54	7.18	0.016300466	0.040093563
8016433	3211	*homeobox B1*	*HOXB1*	6.90	7.00	6.60	0.016306093	0.040103987
8111339	54545	*myotubularin related protein 12*	*MTMR12*	8.42	8.40	10.42	0.016310617	0.0401117
8121275				6.71	7.40	5.74	0.016315173	0.040119484
7990839	80765, 3603	*StAR-related lipid transfer (START) domain containing 5 | interleukin 16 (lymphocyte chemoattractant factor)*	*STARD5|IL16*	9.30	9.13	7.59	0.016325302	0.04014097
8161415	644249		*LOC644249*	5.18	5.01	5.82	0.016333187	0.040156938
8021228	4089	*SMAD family member 4*	*SMAD4*	10.18	10.25	11.29	0.016340002	0.040170275
7953687				6.15	6.35	5.67	0.016352061	0.040196497
7913547	54361	*wingless-type MMTV integration site family, member 4*	*WNT4*	7.37	7.50	6.80	0.016354688	0.04019953
8045155				4.45	4.47	4.38	0.016360207	0.040209673
7919642	317772	*histone cluster 2, H2ab*	*HIST2H2AB*	10.46	11.02	12.37	0.016376184	0.040243104
8123763				7.10	7.04	6.83	0.016376598	0.040243104
8063097	63935	*PDX1 C-terminal inhibiting factor 1*	*PCIF1*	9.86	10.02	8.97	0.016380565	0.04024943
7964555	10677	*advillin*	*AVIL*	7.04	6.99	6.08	0.016382204	0.04024989
8145225				5.62	5.67	5.36	0.016383542	0.04024989
8085429	100128644	*LMNE6487*	*LOC100128644*	6.77	6.82	6.50	0.016387654	0.040256567
8136971	346525	*olfactory receptor, family 2, subfamily A, member 12*	*OR2A12*	5.35	5.37	5.01	0.016390556	0.04026027
7899075	2134	*exostoses (multiple)-like 1*	*EXTL1*	7.09	7.23	6.41	0.016400728	0.04028183
8164452	84885	*zinc finger, DHHC-type containing 12*	*ZDHHC12*	10.73	10.50	9.71	0.016404048	0.040286556
8019570	653240, 100132476, 728224, 100132386, 85289	*keratin associated protein 4-11 | keratin associated protein 4-7 | keratin associated protein 4-8 | keratin associated protein 4-9 | keratin associated protein 4-5*	*KRTAP4-11|KRTAP4-7|KRTAP4-8|KRTAP4-9|KRTAP4-5*	6.45	6.57	6.07	0.016412608	0.04030415

8163383	64420	*sushi domain containing 1*	*SUSD1*	8.38	7.96	7.50	0.016416715	0.040310808
7897277	148645	*chromosome 1 open reading frame 211*	*C1orf211*	6.77	6.66	7.36	0.016430741	0.040341817
8016245	55073, 374819, 474170	*leucine rich repeat containing 37, member A4 (pseudogene) | leucine rich repeat containing 37, member A3 | leucine rich repeat containing 37, member A2*	*LRRC37A4 |LRRC37A 3|LRRC37 A2*	8.29	7.93	10.29	0.016438665	0.04035784
7973108	643332	*ribonuclease, RNase A family, 2 (liver, eosinophil-derived neurotoxin) pseudogene*	*ECRP*	6.59	6.64	6.25	0.016444456	0.04036862
7973510	9362	*copine VI (neuronal)*	*CPNE6*	6.91	6.95	6.54	0.01645169	0.040382948
8039378	6861	*synaptotagmin V*	*SYT5*	7.25	7.50	6.86	0.016466616	0.040416148
8107613	6643	*sorting nexin 2*	*SNX2*	9.58	9.02	11.23	0.016470384	0.04042196
8028030	115703	*Rho GTPase activating protein 33*	*ARHGAP3 3*	7.32	7.45	6.98	0.01647237	0.040423397
8164034	57706	*DENN/MADD domain containing 1A*	*DENND1A*	9.18	9.19	8.47	0.016476138	0.04042921
8030044	3770	*potassium inwardly-rectifying channel, subfamily J, member 14*	*KCNJ14*	6.91	6.88	6.32	0.016484736	0.040443428
7913211	30814	*phospholipase A2, group IIE*	*PLA2G2E*	7.37	7.56	6.96	0.016484672	0.040443428
8124524	8330, 8341	*histone cluster 1, H2ak | histone cluster 1, H2bn*	*HIST1H2A K|HIST1H 2BN*	9.13	9.13	11.59	0.0164871	0.04044579
8092201	79718	*transducin (beta)-like 1 X-linked receptor 1*	*TBL1XR1*	10.69	9.75	10.65	0.016491534	0.040453233
7940279	64232	*membrane-spanning 4-domains, subfamily A, member 5*	*MS4A5*	4.79	4.75	4.68	0.016495492	0.040456068
7944797	390264	*olfactory receptor, family 10, subfamily G, member 4*	*OR10G4*	5.96	6.33	5.83	0.016494507	0.040456068
8179709	389376	*surfactant associated 2*	*SFTA2*	6.91	6.94	6.44	0.016503708	0.04047278
7914667	114784	*CUB and Sushi multiple domains 2*	*CSMD2*	6.94	7.39	7.28	0.016507551	0.040478766
7907090	100128751	*INM04*	*LOC10012 8751*	6.79	6.69	6.38	0.01651118	0.040484224
7946031	283111	*olfactory receptor, family 51, subfamily V, member 1*	*OR51V1*	6.24	6.17	5.94	0.016517216	0.040495586
8079305	7123	*C-type lectin domain family 3, member B*	*CLEC3B*	8.76	8.76	7.50	0.016526356	0.04051147
7957631	442903	*microRNA 331*	*MIR331*	6.15	6.18	5.82	0.016526502	0.04051147

7982339	100130857		*LOC100130857*	6.94	7.02	6.59	0.016533116	0.04052424
8117426	8345	*histone cluster 1, H2bh*	*HIST1H2BH*	10.23	10.80	12.59	0.016541148	0.040536236
8074856	23532	*preferentially expressed antigen in melanoma*	*PRAME*	5.89	5.89	5.65	0.01654362	0.040536236
8089559	344807	*CD200 receptor 1-like*	*CD200R1L*	4.87	4.85	4.66	0.016544862	0.040536236
8072133	284900, 23331	*TTC28 antisense RNA (non-protein coding) | tetratricopeptide repeat domain 28*	*TTC28-AS*	6.51	6.42	6.15	0.016543984	0.040536236
8149245	641517, 245912	*defensin, beta 109, pseudogene 1B | defensin, beta 109, pseudogene 1*	*DEFB109P1B*	5.89	5.61	5.42	0.01654503	0.040536236
7925492	23596, 8564	*opsin 3 | kynurenine 3-monooxygenase (kynurenine 3-hydroxylase)*	*OPN3|KMO*	9.03	7.99	8.21	0.0165478	0.04053958
8028524	81	*actinin, alpha 4*	*ACTN4*	13.22	13.32	12.73	0.016550923	0.04054379
8030946	388558	*zinc finger protein 808*	*ZNF808*	8.16	7.87	9.89	0.016557438	0.04054943
7916393	606495	*cytochrome b5 reductase-like*	*CYB5RL*	7.11	7.04	7.48	0.016556358	0.04054943
8127507				6.02	5.95	5.64	0.016554726	0.04054943
7944185	917	*CD3g molecule, gamma (CD3-TCR complex)*	*CD3G*	5.57	5.65	5.31	0.016568838	0.04056702
7962367	85437	*zinc finger CCHC-type and RNA binding motif 1*	*ZCRB1*	9.77	9.50	10.50	0.01656739	0.04056702
8106720				5.52	5.65	6.06	0.016568413	0.04056702
8089062	56650, 1371	*claudin domain containing 1 | coproporphyrinogen oxidase*	*CLDND1|CPOX*	10.50	10.57	11.30	0.016571261	0.040569518
8132832	1644, 100129427	*dopa decarboxylase (aromatic L-amino acid decarboxylase) | hypothetical LOC100129427*	*DDC|LOC100129427*	7.16	7.11	6.60	0.01657355	0.04057168
8071523	729461, 26080	*hypothetical LOC729461 | non-protein coding RNA 281*	*LOC729461|NCRNA00281*	5.98	6.11	5.65	0.01657702	0.040576734
8012830	388335	*transmembrane protein 220*	*TMEM220*	7.43	7.21	6.59	0.016578794	0.040577635
7945742	29850	*transient receptor potential cation channel, subfamily M, member 5*	*TRPM5*	7.16	7.25	6.60	0.01658102	0.040579643
8099099	85013	*transmembrane protein 128*	*TMEM128*	7.55	7.69	8.40	0.0165868	0.040586904
8003991	50488, 1145	*misshapen-like kinase 1 | cholinergic receptor, nicotinic, epsilon*	*MINK1|CHRNE*	10.29	9.87	9.29	0.016586456	0.040586904

7989315	2958	*general transcription factor IIA, 2, 12kDa*	*GTF2A2*	9.73	9.86	10.44	0.016590875	0.040593438
8054451	10913	*ectodysplasin A receptor*	*EDAR*	6.60	6.68	6.22	0.016592592	0.040594198
8035787				5.02	4.86	5.65	0.016601568	0.040612716
7981525	55038	*cell division cycle associated 4*	*CDCA4*	8.83	9.19	10.34	0.016605081	0.040615954
8104268	340024	*solute carrier family 6 (neutral amino acid transporter), member 19*	*SLC6A19*	6.87	6.92	6.27	0.016605705	0.040615954
8066641	63925	*zinc finger protein 335*	*ZNF335*	8.84	8.82	8.09	0.016615868	0.040635604
8049394	79054	*transient receptor potential cation channel, subfamily M, member 8*	*TRPM8*	5.58	5.66	5.36	0.016616555	0.040635604
8157941	403341	*zinc finger and BTB domain containing 34*	*ZBTB34*	7.29	7.31	8.22	0.016621323	0.040643822
7902452	26289	*adenylate kinase 5*	*AK5*	8.62	9.64	8.07	0.01662467	0.04064766
8025551	55337, 83854	*chromosome 19 open reading frame 66 | angiopoietin-like 6*	*C19orf66| ANGPTL6*	9.81	9.62	8.90	0.016625708	0.04064766
7948161				5.27	5.24	5.04	0.016630933	0.04065699
8160308	6194	*ribosomal protein S6*	*RPS6*	12.23	12.39	12.70	0.01663423	0.040661607
7900833	9682	*lysine (K)-specific demethylase 4A*	*KDM4A*	9.26	9.23	9.96	0.016644305	0.04068279
7899377	5511	*protein phosphatase 1, regulatory (inhibitor) subunit 8*	*PPP1R8*	8.62	8.91	10.06	0.016653882	0.040702753
8065396	128821	*cystatin 9-like*	*CST9L*	6.30	6.38	5.99	0.016657894	0.04070911
8061944	170487	*chromosome 20 open reading frame 134*	*C20orf134*	7.63	7.73	7.11	0.01666094	0.04071311
8020197	342850	*ankyrin repeat domain 62*	*ANKRD62*	5.09	5.02	4.96	0.016666055	0.04072216
8168205	4303	*forkhead box O4*	*FOXO4*	8.71	8.60	10.00	0.016674906	0.04073689
8118726	449520, 401253	*gametogenetin binding protein 1 | chromosome 6 open reading frame 227*	*GGNBP1| C6orf227*	7.20	7.33	6.93	0.016674807	0.04073689
7983173	23582	*cyclin D-type binding-protein 1*	*CCNDBP1*	8.73	8.89	7.95	0.01668059	0.040743884
7902850	163351	*guanylate binding protein family, member 6*	*GBP6*	5.38	5.29	5.07	0.016679598	0.040743884
8099551	27146	*family with sequence similarity 184, member B*	*FAM184B*	6.99	7.03	6.65	0.01670139	0.040791236
8064302	55251	*protein-L-isoaspartate (D-aspartate) O-methyltransferase domain containing 2*	*PCMTD2*	9.61	9.16	10.85	0.016704462	0.04079529
8043405				4.77	4.78	4.62	0.016719084	0.040827546
8029489	4059	*basal cell adhesion molecule (Lutheran blood group)*	*BCAM*	8.59	7.82	8.56	0.016721178	0.040829204
7930627	153	*adrenergic, beta-1-, receptor*	*ADRB1*	8.38	8.59	7.68	0.016722744	0.040829577

8178199	3117	*major histocompatibility complex, class II, DQ alpha 1*	*HLA-DQA1*	7.07	7.14	6.64	0.01672972	0.040839303
8066921	3745	*potassium voltage-gated channel, Shab-related subfamily, member 1*	*KCNB1*	6.76	6.72	6.26	0.016731469	0.040839303
8051413	25940	*family with sequence similarity 98, member A*	*FAM98A*	9.65	9.30	11.19	0.01673203	0.040839303
7906386	149628	*pyrin and HIN domain family, member 1*	*PYHIN1*	6.19	6.08	5.90	0.016732387	0.040839303
8139460	23148	*NAC alpha domain containing*	*NACAD*	8.35	8.15	7.32	0.016736979	0.040847056
7949015	65998	*chromosome 11 open reading frame 95*	*C11orf95*	9.28	9.26	8.83	0.016741758	0.04085506
8049942	129807	*sialidase 4*	*NEU4*	7.34	7.54	6.90	0.016744504	0.04085506
8088908				5.82	5.62	5.12	0.016743736	0.04085506
8179987	63940, 4855	*G-protein signaling modulator 3 | notch 4*	*GPSM3|NOTCH4*	7.92	7.81	7.18	0.016750181	0.04086546
8107574				4.76	4.69	4.56	0.01675628	0.040876884
7974253				6.78	6.79	6.39	0.016758697	0.040879328
7931914	3559	*interleukin 2 receptor, alpha*	*IL2RA*	6.27	6.23	5.92	0.016760899	0.040880393
8005576	5598	*mitogen-activated protein kinase 7*	*MAPK7*	8.84	9.23	8.02	0.016761966	0.040880393
7964983				5.81	5.74	5.41	0.016764577	0.040883306
7991451				6.74	6.73	6.05	0.016775478	0.040906433
8104998	55100	*WD repeat domain 70*	*WDR70*	7.37	7.55	8.46	0.016781397	0.040909857
7933501	644168	*dorsal root ganglia homeobox*	*DRGX*	7.25	7.37	6.79	0.016778514	0.040909857
8144496	389616, 403296	*olfactory receptor, family 7, subfamily E, member 125 pseudogene | olfactory receptor, family 7, subfamily E, member 154 pseudogene*	*OR7E125P|OR7E154P*	7.54	7.50	7.03	0.016782416	0.040909857
7926936	79741, 221016	*chromosome 10 open reading frame 68 | coiled-coil domain containing 7*	*C10orf68|CCDC7*	6.27	6.27	5.69	0.01678255	0.040909857
8025414	9230		*RAB11B*	11.58	11.53	10.91	0.01679374	0.04093368
8074020	83642	*selenoprotein O*	*SELO*	9.52	9.48	8.49	0.016824277	0.041004647
8016733				7.65	7.79	7.28	0.016827226	0.04100837
8085253				6.24	6.21	5.87	0.01683604	0.041026387
8062108	10544	*protein C receptor, endothelial*	*PROCR*	8.76	8.24	7.93	0.016837483	0.041026443
8073799	25814	*ataxin 10*	*ATXN10*	11.10	10.85	11.53	0.016844545	0.041040186

7937813	10078	*tumor suppressing subtransferable candidate 4*	*TSSC4*	7.74	7.81	7.10	0.016848259	0.04104577
8080306	51533	*PHD finger protein 7*	*PHF7*	7.23	7.36	7.91	0.016859319	0.04106925
8006877	10948	*StAR-related lipid transfer (START) domain containing 3*	*STARD3*	10.20	10.25	9.30	0.016865263	0.041080263
8041257	55676	*solute carrier family 30 (zinc transporter), member 6*	*SLC30A6*	8.78	8.85	10.81	0.01687209	0.041093424
8026170	81576	*coiled-coil domain containing 130*	*CCDC130*	9.34	9.54	8.35	0.016881337	0.04111248
8025828	3949	*low density lipoprotein receptor*	*LDLR*	11.13	10.10	10.73	0.016888881	0.041124947
8157524	7099	*toll-like receptor 4*	*TLR4*	8.34	8.02	5.79	0.016889306	0.041124947
7901187	541468, 55624	*chromosome 1 open reading frame 190 | protein O-linked mannose beta1,2-N-acetylglucosaminyltransferase*	*C1orf190| POMGNT1*	7.56	7.50	7.08	0.016899494	0.041146286
7945979	55128	*tripartite motif-containing 68*	*TRIM68*	9.92	9.22	8.79	0.016908122	0.04116382
7912659	79814	*agmatine ureohydrolase (agmatinase)*	*AGMAT*	7.81	7.60	8.90	0.016911898	0.04116954
8075963	23616	*SH3-domain binding protein 1*	*SH3BP1*	7.48	7.60	7.02	0.016919775	0.04118524
8077929				6.04	6.10	6.87	0.016928444	0.04120287
8152815	641384	*transmembrane protein 75*	*TMEM75*	5.95	6.03	5.76	0.016932553	0.041205924
8100013				5.28	5.32	5.09	0.016931126	0.041205924
8065756				6.32	6.20	6.73	0.01693401	0.041205995
7906107	64218	*sema domain, immunoglobulin domain (Ig), transmembrane domain (TM) and short cytoplasmic domain, (semaphorin) 4A*	*SEMA4A*	6.96	6.83	7.22	0.016941309	0.041218575
8096333				4.73	4.77	4.51	0.016942035	0.041218575
8078962	10289	*eukaryotic translation initiation factor 1B*	*EIF1B*	9.06	9.03	9.39	0.016947957	0.04122951
8176397	90655, 90316	*TGFB-induced factor homeobox 2-like, Y-linked | TGFB-induced factor homeobox 2-like, X-linked*	*TGIF2LY| TGIF2LX*	5.38	5.54	5.19	0.016955214	0.04124021
8059341				6.32	6.36	5.99	0.01695441	0.04124021
7936360	4892	*nebulin-related anchoring protein*	*NRAP*	6.22	6.33	6.00	0.016961468	0.04124757
8105077	84674	*caspase recruitment domain family, member 6*	*CARD6*	6.33	6.52	5.15	0.016962525	0.04124757
7929315	340665	*cytochrome P450, family 26, subfamily C, polypeptide 1*	*CYP26C1*	7.83	7.95	7.38	0.016960336	0.04124757
7914112	2268	*Gardner-Rasheed feline sarcoma viral (v-fgr) oncogene homolog*	*FGR*	7.43	7.43	7.09	0.016967654	0.041253958

8177118				7.46	7.51	6.68	0.01696801	0.041253958
7997827	9780	*family with sequence similarity 38, member A*	*FAM38A*	7.35	7.52	6.78	0.016983777	0.041287474
7968703	387921	*NHL repeat containing 3*	*NHLRC3*	7.08	7.02	8.29	0.016984656	0.041287474
7904025	7482	*wingless-type MMTV integration site family, member 2B*	*WNT2B*	7.50	7.24	6.76	0.01699453	0.041307997
7930074	4791	*nuclear factor of kappa light polypeptide gene enhancer in B-cells 2 (p49/p100)*	*NFKB2*	8.87	8.92	7.94	0.016998846	0.04131501
7915982	79656	*BEN domain containing 5*	*BEND5*	6.26	6.35	6.62	0.01700471	0.041323826
8000616	79874	*rabaptin, RAB GTPase binding effector protein 2*	*RABEP2*	7.47	7.58	7.07	0.017005336	0.041323826
8173615	51132	*ring finger protein, LIM domain interacting*	*RLIM*	7.40	7.26	9.24	0.017014738	0.041343194
8003467	5119	*chromatin modifying protein 1A*	*CHMP1A*	9.38	9.49	8.53	0.017016372	0.04134368
8174141	340542	*brain expressed, X-linked 5*	*BEX5*	5.93	6.04	6.32	0.017022582	0.04135529
8028778	4294	*mitogen-activated protein kinase kinase kinase 10*	*MAP3K10*	8.06	8.22	7.41	0.01702589	0.041359846
8010512	57521	*regulatory associated protein of MTOR, complex 1*	*RPTOR*	8.70	8.71	7.95	0.017031157	0.041369162
8089519	151888	*B and T lymphocyte associated*	*BTLA*	6.00	5.90	5.72	0.017035786	0.041376922
7955045	91523	*family with sequence similarity 113, member B*	*FAM113B*	7.76	7.94	6.65	0.017037692	0.04137807
7994692	9344	*TAO kinase 2*	*TAOK2*	10.17	10.09	9.06	0.017041977	0.041385
8174893	57187	*THO complex 2*	*THOC2*	8.19	7.82	9.86	0.017054	0.041407228
7984016	145741	*C2 calcium-dependent domain containing 4A*	*C2CD4A*	7.67	7.86	7.42	0.017053075	0.041407228
8000834	23049, 595101, 440354, 641298, 100271836	*SMG1 homolog, phosphatidylinositol 3-kinase-related kinase (C. elegans) | PI-3-kinase-related kinase SMG-1 pseudogene | SMG1 homolog, phosphatidylinositol 3-kinase-related kinase pseudogene*	*SMG1|LOC595101|LOC440354|LOC641298|LOC100271836*	10.04	9.89	11.27	0.017058011	0.041413482
8140955	1021	*cyclin-dependent kinase 6*	*CDK6*	8.21	7.82	9.37	0.017060226	0.04141538
7924327	55105	*G patch domain containing 2*	*GPATCH2*	8.04	7.90	8.73	0.017064469	0.041422196
8057563	151112	*zinc finger, SWIM-type containing 2*	*ZSWIM2*	4.82	4.87	4.62	0.017068878	0.04142941
8098769	84179, 4636	*major facilitator superfamily domain containing 7 | myosin, light chain 5, regulatory*	*MFSD7|MYL5*	8.77	8.98	7.72	0.017074978	0.041440737
8145822				4.70	4.67	4.52	0.017087402	0.041467402

7998902	54985, 79228	*host cell factor C1 regulator 1 (XPO1 dependent) | THO complex 6 homolog (Drosophila)*	*HCFC1R1| THOC6*	9.35	9.05	8.43	0.017089305	0.041468535
8028233	284459		*HKR1*	7.25	7.12	7.88	0.017090842	0.041468777
8178586	80740	*lymphocyte antigen 6 complex, locus G6C*	*LY6G6C*	6.92	6.90	6.61	0.017095018	0.041475426
8138189	6119	*replication protein A3, 14kDa*	*RPA3*	7.37	7.45	7.78	0.017100222	0.041484565
8137931	221938	*monocyte to macrophage differentiation-associated 2*	*MMD2*	6.90	6.79	6.44	0.017102478	0.04148655
8064277	4661	*myelin transcription factor 1*	*MYT1*	6.77	6.90	6.60	0.017109951	0.041497704
7997281	54386, 100288358	*telomeric repeat binding factor 2, interacting protein | hypothetical LOC100288358*	*TERF2IP| LOC10028 8358*	8.98	9.03	8.16	0.017108746	0.041497704
7923438	6232	*ribosomal protein S27*	*RPS27*	12.36	12.39	12.20	0.017111761	0.04149861
8081989	503582	*arginine-fifty homeobox*	*ARGFX*	5.21	5.26	4.81	0.017117176	0.041508254
8133914	9988	*cyclin D binding myb-like transcription factor 1*	*DMTF1*	8.16	7.88	10.28	0.017119499	0.0415104
8136281	286023		*FLJ40288*	5.51	5.60	5.22	0.01712437	0.04151872
7943098	4544	*melatonin receptor 1B*	*MTNR1B*	6.99	7.12	6.67	0.01713344	0.04153186
8152148	51366	*ubiquitin protein ligase E3 component n-recognin 5*	*UBR5*	8.47	7.61	10.17	0.017135888	0.04153186
7985147	55466	*DnaJ (Hsp40) homolog, subfamily A, member 4*	*DNAJA4*	7.83	7.68	8.24	0.017135248	0.04153186
8139256	55665	*upregulator of cell proliferation*	*URGCP*	8.10	8.00	7.38	0.017132597	0.04153186
7942255	440050, 57830	*keratin associated protein 5-7 | keratin associated protein 5-8*	*KRTAP5-7|KRTAP5-8*	8.77	8.86	8.16	0.017136982	0.04153186
7951217	4316	*matrix metallopeptidase 7 (matrilysin, uterine)*	*MMP7*	5.21	5.19	5.10	0.017142719	0.04153879
7980690	90141	*chromosome 14 open reading frame 143*	*C14orf143*	7.26	7.36	8.00	0.01714226	0.04153879
7972902	113622	*ADP-ribosylhydrolase like 1*	*ADPRHL1*	6.83	7.01	6.32	0.017148793	0.041546535
7991047	100131860		*LOC10013 1860*	7.83	7.39	8.13	0.017148418	0.041546535
8022570				4.90	5.03	4.73	0.017150452	0.041547067
7945498	6343	*secretin*	*SCT*	7.97	8.01	7.61	0.017153759	0.04155159
8172088	54880	*BCL6 corepressor*	*BCOR*	8.23	8.22	9.49	0.017155899	0.04155329
8128890	442245	*glutathione S-transferase mu 2 (muscle) pseudogene 1*	*GSTM2P1*	6.24	6.41	5.75	0.017158296	0.04155561

7921652	6504	*signaling lymphocytic activation molecule family member 1*	*SLAMF1*	5.47	5.56	5.27	0.01717811	0.041596618
8167690	401589, 100129860	*hypothetical LOC401589 | hypothetical LOC100129860*	*LOC401589|LOC100129860*	6.02	6.21	5.76	0.01717739	0.041596618
7902127	84251	*SH3-domain GRB2-like (endophilin) interacting protein 1*	*SGIP1*	7.54	6.92	5.84	0.017181806	0.04159915
8073960	415116	*pim-3 oncogene*	*PIM3*	7.89	7.84	7.58	0.01718204	0.04159915
8113003	55338		*FLJ11292*	6.27	6.39	5.95	0.01720793	0.041658342
7991313	5346	*perilipin 1*	*PLIN1*	7.32	7.45	6.91	0.017213413	0.041668124
7920687	2630, 2629	*glucosidase, beta, acid pseudogene 1*	*GBAP1*	8.46	8.70	7.60	0.017217347	0.041670658
8052087				8.35	8.10	7.79	0.01721625	0.041670658
8012949	374286, 10517	*CMT1A duplicated region transcript 1 | F-box and WD repeat domain containing 10*	*CDRT1|FBXW10*	6.59	5.99	5.10	0.01722039	0.041674525
8039996	7173	*thyroid peroxidase*	*TPO*	6.07	6.01	5.66	0.017226966	0.041683637
8093601	8603	*family with sequence similarity 193, member A*	*FAM193A*	9.17	9.08	8.34	0.017227042	0.041683637
8172561	4007	*prickle homolog 3 (Drosophila)*	*PRICKLE3*	9.72	9.53	8.61	0.017233456	0.04169566
7949904	81622	*unc-93 homolog B1 (C. elegans)*	*UNC93B1*	9.50	9.36	8.39	0.017248282	0.04171704
7941364	399909	*pecanex-like 3 (Drosophila)*	*PCNXL3*	10.30	10.24	9.20	0.017249065	0.04171704
8165674	30011, 4537	*SH3-domain kinase binding protein 1 | NADH dehydrogenase, subunit 3 (complex I)*	*SH3KBP1|ND3*	12.94	12.87	12.75	0.017246539	0.04171704
7917528				5.09	4.94	5.51	0.017249279	0.04171704
7937798				7.03	6.96	6.60	0.017250963	0.04171704
7979355				7.09	7.21	6.86	0.017249536	0.04171704
8114896	134391	*G protein-coupled receptor 151*	*GPR151*	6.11	6.18	5.80	0.017259363	0.04173386
8121319	55084	*sine oculis binding protein homolog (Drosophila)*	*SOBP*	7.35	6.72	7.25	0.01726687	0.041748513
8177951	253018	*HLA complex group 27*	*HCG27*	7.43	7.42	6.92	0.017274288	0.041762955
8153835	84988	*protein phosphatase 1, regulatory (inhibitor) subunit 16A*	*PPP1R16A*	7.15	7.14	6.52	0.017294735	0.041798383
8022488	171586	*abhydrolase domain containing 3*	*ABHD3*	6.31	6.28	7.19	0.017292451	0.041798383

8133600	389517, 442590, 441272, 729597	*speedy homolog E8 (Xenopus laevis), pseudogene | speedy homolog E5 (Xenopus laevis) | speedy homolog E3 (Xenopus laevis) | speedy homolog E6 (Xenopus laevis)*	*SPDYE8P|SPDYE5|SPDYE3|SPDYE6*	6.78	6.82	6.60	0.01729464	0.041798383
7993349	9284, 339044, 100288332, 642778	*nuclear pore complex interacting protein | polycystic kidney disease 1 (autosomal dominant) pseudogene 1 | GPS, PLAT and transmembrane domain-containing protein FLJ00285-like | nuclear pore complex-interacting protein-like 1-like*	*NPIP|PKD1P1|LOC100288332|LOC642778*	11.61	11.66	10.78	0.017291268	0.041798383
8117510	10473	*high mobility group nucleosomal binding domain 4*	*HMGN4*	9.63	9.81	10.37	0.01729712	0.04180065
8124648	116511	*MAS1 oncogene-like*	*MAS1L*	5.56	5.58	5.31	0.0173012	0.04180701
8084000				4.51	4.54	4.44	0.017304154	0.041810647
8097507	83452		*RAB33B*	7.71	7.84	9.09	0.017305853	0.041811254
8021594	89777	*serpin peptidase inhibitor, clade B (ovalbumin), member 12*	*SERPINB12*	5.07	5.00	4.82	0.017312357	0.04182347
7993606				6.07	5.97	6.34	0.017324654	0.041849673
7968307	23281	*microtubule associated tumor suppressor candidate 2*	*MTUS2*	6.18	6.24	5.96	0.017331205	0.04185149
8010924	55275	*vacuolar protein sorting 53 homolog (S. cerevisiae)*	*VPS53*	10.03	10.12	9.65	0.017328965	0.04185149
8082786				4.42	4.41	4.36	0.017330486	0.04185149
8161000				5.49	5.65	5.23	0.017329354	0.04185149
7920297	57402	*S100 calcium binding protein A14*	*S100A14*	6.92	6.97	6.66	0.017336732	0.041861333
8071206	64976	*mitochondrial ribosomal protein L40*	*MRPL40*	9.37	9.48	9.06	0.017339073	0.041863486
7967789	5827	*peroxisomal membrane protein 2, 22kDa*	*PXMP2*	8.45	8.59	7.69	0.017356629	0.041897617
8049425	6694	*secreted phosphoprotein 2, 24kDa*	*SPP2*	5.10	5.04	4.90	0.017356815	0.041897617
8179235	253018	*HLA complex group 27*	*HCG27*	7.37	7.28	6.70	0.017357564	0.041897617
8012726	4619	*myosin, heavy chain 1, skeletal muscle, adult*	*MYH1*	4.95	4.93	4.87	0.017359553	0.041898917
8108683	56133	*protocadherin beta 2*	*PCDHB2*	8.11	8.25	9.86	0.017361088	0.04189912
8103452	152940	*chromosome 4 open reading frame 45*	*C4orf45*	4.89	4.94	4.68	0.017364852	0.0419047
8124082				8.79	8.81	8.51	0.017366592	0.041905396
8116537	79908	*butyrophilin-like 8*	*BTNL8*	5.83	6.00	5.53	0.017371358	0.041913394

7969228	440138, 9724	*asparagine-linked glycosylation 11, alpha-1,2-mannosyltransferase homolog (yeast) | UTP14, U3 small nucleolar ribonucleoprotein, homolog C (yeast)*	*ALG11|UTP14C*	7.27	7.19	9.23	0.017401475	0.04198255
7913256	1650	*dolichyl-diphosphooligosaccharide--protein glycosyltransferase*	*DDOST*	10.90	10.76	10.29	0.017408097	0.041995015
8179489	3117	*major histocompatibility complex, class II, DQ alpha 1*	*HLA-DQA1*	7.08	7.15	6.64	0.017420532	0.0420215
7997504	1012	*cadherin 13, H-cadherin (heart)*	*CDH13*	10.20	11.13	8.88	0.017435858	0.04205496
8120204				5.97	6.07	5.59	0.01743899	0.042058997
8171271	171483	*family with sequence similarity 9, member B*	*FAM9B*	5.68	5.62	5.37	0.017441515	0.042061575
8111915	6414	*selenoprotein P, plasma, 1*	*SEPP1*	9.15	7.35	9.18	0.017448304	0.04207443
8015287	8687	*keratin 38*	*KRT38*	6.34	6.50	6.07	0.017453749	0.042081703
7927060				4.77	4.69	4.54	0.017454235	0.042081703
8049166	1146	*cholinergic receptor, nicotinic, gamma*	*CHRNG*	7.94	7.98	7.43	0.017463183	0.042089216
8025452	85509	*methyl-CpG binding domain protein 3-like 1*	*MBD3L1*	5.00	5.14	4.88	0.017462255	0.042089216
7933421				5.21	5.29	4.89	0.017460909	0.042089216
8071804				8.71	8.60	7.75	0.017460607	0.042089216
8089036				6.80	7.10	6.96	0.017470682	0.042103775
8081202	79295	*olfactory receptor, family 5, subfamily H, member 6*	*OR5H6*	5.08	5.18	4.88	0.017477449	0.042116567
7967792				6.71	6.71	6.38	0.017495073	0.04215552
7939932	219432	*olfactory receptor, family 4, subfamily C, member 6*	*OR4C6*	5.53	5.53	5.35	0.017502889	0.04217083
7903561	642864	*spermatogenesis-related protein 7*	*LOC642864*	4.96	4.96	4.76	0.01750794	0.042179484
7920744	5313	*pyruvate kinase, liver and RBC*	*PKLR*	7.22	7.35	6.81	0.01751169	0.042183366
7976158				9.15	9.16	8.46	0.017512474	0.042183366
8079896	6405	*sema domain, immunoglobulin domain (Ig), short basic domain, secreted, (semaphorin) 3F*	*SEMA3F*	9.17	7.83	7.94	0.017520748	0.04219977
7959850	11211	*frizzled homolog 10 (Drosophila)*	*FZD10*	7.51	7.54	7.08	0.017537503	0.042236604
7923332	7139	*troponin T type 2 (cardiac)*	*TNNT2*	6.98	6.88	7.55	0.01754177	0.042243358
8092748				4.69	4.64	4.57	0.017547896	0.042254586
7934966				7.04	7.01	6.61	0.01755585	0.042267382

8062337				8.42	8.48	8.06	0.017556138	0.042267382
8030437	3276	*protein arginine methyltransferase 1*	*PRMT1*	10.34	10.48	11.41	0.01756778	0.04227111
8037742	8993	*peptidoglycan recognition protein 1*	*PGLYRP1*	7.42	7.57	6.88	0.017560972	0.04227111
8006779	57636	*Rho GTPase activating protein 23*	*ARHGAP23*	10.82	10.84	9.47	0.017561961	0.04227111
8106702	84240	*zinc finger, CCHC domain containing 9*	*ZCCHC9*	8.85	8.73	9.54	0.017570578	0.04227111
8179070	89870	*tripartite motif-containing 15*	*TRIM15*	7.47	7.45	7.08	0.017575594	0.04227111
8126674	202500	*t-complex-associated-testis-expressed 1*	*TCTE1*	7.08	7.06	6.48	0.017571267	0.04227111
7978107	4901, 80344	*neural retina leucine zipper | DDB1 and CUL4 associated factor 11*	*NRL|DCAF11*	7.61	7.87	7.21	0.017576722	0.04227111
8043032				6.51	6.60	5.90	0.017573453	0.04227111
8053404				4.53	4.49	4.42	0.017572433	0.04227111
8094026				9.07	9.14	8.39	0.017563708	0.04227111
8116954				4.44	4.42	4.35	0.017571388	0.04227111
8131412				6.65	6.88	6.16	0.017575212	0.04227111
8136786				4.64	4.64	4.47	0.017571073	0.04227111
8125373	63940, 4855	*G-protein signaling modulator 3 | notch 4*	*GPSM3|NOTCH4*	7.92	7.81	7.17	0.017588831	0.042296708
8119076				5.76	5.53	5.22	0.01759367	0.04230482
8130939	28514	*delta-like 1 (Drosophila)*	*DLL1*	7.57	7.31	7.13	0.017597275	0.042309962
7953564	113246	*chromosome 12 open reading frame 57*	*C12orf57*	11.89	12.03	11.05	0.017605938	0.042323712
8149685	203190	*leucine-rich repeat LGI family, member 3*	*LGI3*	7.12	7.10	6.58	0.01760739	0.042323712
7946015	401666	*olfactory receptor, family 51, subfamily A, member 4*	*OR51A4*	5.66	5.49	5.11	0.017604746	0.042323712
8000399	342357	*zinc finger with KRAB and SCAN domains 2*	*ZKSCAN2*	6.89	6.92	7.53	0.017615657	0.042340055
7897286	100130071	*GSQS6193*	*LOC100130071*	7.59	7.71	7.16	0.017618213	0.042340674
7946655	283106, 1457	*casein kinase 2, alpha 1 polypeptide pseudogene*	*CSNK2A1P*	8.79	8.81	10.19	0.017618848	0.042340674
7915297	1298	*collagen, type IX, alpha 2*	*COL9A2*	7.81	7.97	7.27	0.017624503	0.042347215
7949894				7.03	6.87	6.72	0.017623043	0.042347215
8041027	9553	*mitochondrial ribosomal protein L33*	*MRPL33*	7.29	7.66	7.76	0.017627416	0.042350687

7967707				6.68	6.87	6.21	0.017632436	0.042359225
8140009	389517, 441272, 442590	*speedy homolog E8 (Xenopus laevis), pseudogene | speedy homolog E3 (Xenopus laevis) | speedy homolog E5 (Xenopus laevis)*	*SPDYE8P|SPDYE3|SPDYE5*	6.77	6.82	6.60	0.017643837	0.042383086
7972888	55795	*PCI domain containing 2*	*PCID2*	7.35	6.93	8.46	0.017657626	0.04241268
8168107	158833	*acyl-CoA wax alcohol acyltransferase 1*	*AWAT1*	6.00	6.09	5.60	0.017664574	0.04242584
8135734	79974	*chromosome 7 open reading frame 58*	*C7orf58*	8.10	7.40	5.70	0.017683016	0.0424666
7911339				12.08	12.17	11.25	0.01768523	0.042468384
7978405				5.51	5.16	5.11	0.017691247	0.0424793
7912166	473	*arginine-glutamic acid dipeptide (RE) repeats*	*RERE*	10.30	10.09	9.39	0.017704045	0.04250401
8165698				12.08	12.17	11.25	0.017704483	0.04250401
7949033	28992	*MACRO domain containing 1*	*MACROD1*	8.45	8.58	7.94	0.017707678	0.042508144
8115594	114898	*C1q and tumor necrosis factor related protein 2*	*C1QTNF2*	9.75	8.16	7.58	0.01771441	0.04252077
8120758	26054	*SUMO1/sentrin specific peptidase 6*	*SENP6*	8.38	8.21	9.44	0.017717015	0.04252349
7946006	401663	*olfactory receptor, family 51, subfamily H, member 1 pseudogene*	*OR51H1P*	6.00	6.01	5.72	0.017742723	0.04258165
7912473	26232, 93611	*F-box protein 2 | F-box protein 44*	*FBXO2|FBXO44*	8.30	8.22	8.79	0.017744549	0.042582493
8053594	926	*CD8b molecule*	*CD8B*	4.92	5.06	4.75	0.01774894	0.042589493
8082442				7.14	7.14	6.74	0.017759383	0.042611007
7967028	26834	*RNA, U4 small nuclear 2*	*RNU4-2*	7.11	7.62	6.92	0.01776562	0.0426215
8114625	373863, 54853, 3035	*dead end homolog 1 (zebrafish) | WD repeat domain 55 | histidyl-tRNA synthetase*	*DND1|WDR55|HARS*	8.78	8.92	8.14	0.01776671	0.0426215
8172197	80316	*protein phosphatase 1, regulatory (inhibitor) subunit 2 pseudogene 9*	*PPP1R2P9*	6.66	6.81	6.13	0.017782366	0.042655516
7960874	719	*complement component 3a receptor 1*	*C3AR1*	5.63	5.70	5.31	0.017790664	0.042671874
8132439	79783	*chromosome 7 open reading frame 10*	*C7orf10*	7.64	6.84	6.87	0.017795904	0.042680897
8174684	55922	*NFKB repressing factor*	*NKRF*	6.87	6.99	7.68	0.017803565	0.042692177
8120937	134701	*ripply2 homolog (zebrafish)*	*RIPPLY2*	6.70	6.78	6.39	0.01780215	0.042692177
8131512				6.66	6.49	7.04	0.017840123	0.042772736

8148640				7.93	8.06	7.27	0.017839061	0.042772736
7923978	947		*CD34*	7.19	6.78	6.61	0.017844617	0.042777665
8148737	84232		*MAF1*	10.32	10.26	9.50	0.017845143	0.042777665
7917561	115361	*guanylate binding protein 4*	*GBP4*	6.06	5.53	5.26	0.017852066	0.042790707
8054281	164832	*LON peptidase N-terminal domain and ring finger 2*	*LONRF2*	6.42	6.48	6.06	0.017854258	0.04279241
7931387				6.42	6.55	6.07	0.017865162	0.04281499
7966227				5.21	5.23	5.02	0.017870253	0.042823635
8009382	2186	*bromodomain PHD finger transcription factor*	*BPTF*	8.64	8.17	10.32	0.017872918	0.042826466
7911989	6146	*ribosomal protein L22*	*RPL22*	6.48	6.58	7.43	0.017875014	0.042827934
8017098				6.54	6.60	7.60	0.017882785	0.042842995
8178445	170680	*psoriasis susceptibility 1 candidate 2*	*PSORS1C2*	7.44	7.46	7.02	0.017900288	0.042881373
7898413	29943	*peptidyl arginine deiminase, type I*	*PADI1*	6.54	6.70	6.21	0.017904894	0.042888846
8095819	100129583, 8987	*family with sequence similarity 47, member E | starch binding domain 1*	*FAM47E|STBD1*	5.19	5.03	5.62	0.017918624	0.042918175
8099218	5522	*protein phosphatase 2, regulatory subunit B, gamma*	*PPP2R2C*	6.37	6.46	6.90	0.01793078	0.04294003
8100382	26511	*cysteine-rich hydrophobic domain 2*	*CHIC2*	10.11	10.42	9.38	0.017931392	0.04294003
7967700	100128840		*LOC100128840*	6.19	6.05	5.97	0.017932212	0.04294003
8050363				5.23	5.35	5.04	0.017935097	0.04294338
8034469	84261	*F-box and WD repeat domain containing 9*	*FBXW9*	8.31	8.37	7.79	0.017943565	0.042956527
8052624	7360, 647115	*UDP-glucose pyrophosphorylase 2 | hypothetical LOC647115*	*UGP2|FLJ36848*	7.33	7.42	6.87	0.017942542	0.042956527
8051569				4.58	4.56	4.46	0.01794801	0.042963605
8023497	5205	*ATPase, aminophospholipid transporter, class I, type 8B, member 1*	*ATP8B1*	9.38	9.01	7.03	0.017952446	0.04297066
7934868				5.21	5.28	4.90	0.017957615	0.04297947
7972867	55795	*PCI domain containing 2*	*PCID2*	8.61	8.41	9.70	0.017969547	0.0430009
8083901	64778	*fibronectin type III domain containing 3B*	*FNDC3B*	10.94	10.87	9.55	0.017968873	0.0430009
8124862	1041	*corneodesmosin*	*CDSN*	7.17	7.18	6.72	0.017971652	0.04300237
7913235	391013	*phospholipase A2, group IIC*	*PLA2G2C*	6.80	6.81	6.50	0.017973173	0.04300245

8003066				6.94	7.07	6.25	0.017992424	0.04304494
7938907	9152	*solute carrier family 6 (neurotransmitter transporter, glycine), member 5*	*SLC6A5*	5.82	5.79	5.41	0.017994868	0.04304722
8114415	2676	*GDNF family receptor alpha 3*	*GFRA3*	6.15	6.17	6.66	0.017997483	0.043049905
8068786				6.10	6.17	5.82	0.018009134	0.043074206
8098730	22947, 728410, 653548, 653545, 653544, 441056, 653543	*double homeobox 4 | double homeobox 4 like 2 | double homeobox 4 like 3 | double homeobox 4 like 5 | double homeobox 4 like 6 | double homeobox 4 like 4 | double homeobox 4 like 7*	*DUX4|DUX4L2|DUX4L3|DUX4L5|DUX4L6|DUX4L4|DUX4L7*	8.46	8.61	7.89	0.018013969	0.043082204
7932788				6.65	6.53	6.20	0.018022252	0.043098442
8170816	2652, 728458, 5956	*opsin 1 (cone pigments), medium-wave-sensitive | opsin 1 (cone pigments), medium-wave-sensitive 2 | opsin 1 (cone pigments), long-wave-sensitive*	*OPN1MW|OPN1MW2|OPN1LW*	6.04	6.09	5.78	0.018028287	0.043109305
8157362	114991	*zinc finger protein 618*	*ZNF618*	8.78	8.19	8.53	0.018030033	0.043109905
7953390	171017	*zinc finger protein 384*	*ZNF384*	6.85	7.09	6.46	0.018034024	0.04311588
8017599	5175	*platelet/endothelial cell adhesion molecule*	*PECAM1*	5.90	5.92	5.55	0.018040935	0.04312883
8025958	126070	*zinc finger protein 440*	*ZNF440*	5.94	5.73	6.59	0.018048987	0.043144505
8128282				5.90	5.90	5.48	0.018064838	0.043178823
8032871	91039	*dipeptidyl-peptidase 9*	*DPP9*	9.80	10.11	8.71	0.018077424	0.04320533
8072529	9681	*DEP domain containing 5*	*DEPDC5*	7.97	7.98	8.46	0.018084487	0.043215275
7979328	22863		*ATG14*	8.25	8.19	8.88	0.01808458	0.043215275
7986757	4692	*necdin homolog (mouse)*	*NDN*	7.20	6.94	6.63	0.01809448	0.043234117
7962112	65981	*caprin family member 2*	*CAPRIN2*	7.04	7.19	8.41	0.01809546	0.043234117
8095260				6.85	7.07	6.41	0.01809755	0.043235533
7957379	4617	*myogenic factor 5*	*MYF5*	5.72	5.76	5.38	0.018103356	0.043245822
7962588				6.32	6.32	5.96	0.018110562	0.04325946
7967736	5426	*polymerase (DNA directed), epsilon*	*POLE*	7.88	7.92	9.18	0.018117933	0.043273482

8115734	3937	*lymphocyte cytosolic protein 2 (SH2 domain containing leukocyte protein of 76kDa)*	*LCP2*	6.44	6.47	6.17	0.018124433	0.04328543
7982054	100036563	*small nucleolar RNA, C/D box 115-24*	*SNORD115-24*	4.54	4.64	4.53	0.01812621	0.043286093
8109086	154	*adrenergic, beta-2-, receptor, surface*	*ADRB2*	6.92	6.77	6.38	0.018128129	0.04328709
7936011	79004	*CUE domain containing 2*	*CUEDC2*	10.24	10.10	9.11	0.01814296	0.043318924
8032371	81926	*family with sequence similarity 108, member A1*	*FAM108A1*	9.19	9.36	8.71	0.018146645	0.04332414
7965956	4801	*nuclear transcription factor Y, beta*	*NFYB*	7.55	6.99	8.05	0.018154327	0.043338895
7912591	441871, 391002	*PRAME family member 7 | PRAME family member 8*	*PRAMEF7 |PRAMEF8*	7.42	7.38	6.88	0.018161528	0.0433525
7929988	79803	*Hermansky-Pudlak syndrome 6*	*HPS6*	7.96	7.98	7.29	0.018165685	0.04335884
8108161				5.37	5.39	6.11	0.018177057	0.043382395
8131296	389458		*LOC389458*	7.51	7.58	7.10	0.018180728	0.04338757
8153380	4062	*lymphocyte antigen 6 complex, locus H*	*LY6H*	7.84	7.96	7.29	0.01818487	0.04339028
8022310	63895	*family with sequence similarity 38, member B*	*FAM38B*	6.92	7.82	6.63	0.018184412	0.04339028
7995332				8.56	8.56	8.23	0.018187813	0.043393716
8131583	28969	*basic leucine zipper and W2 domains 2*	*BZW2*	9.75	9.81	11.62	0.018197225	0.043408997
8021243				4.66	4.65	4.49	0.018196682	0.043408997
8166705	5638	*proline rich Gla (G-carboxyglutamic acid) 1*	*PRRG1*	8.58	7.39	8.44	0.018206153	0.043426707
7979888	6547	*solute carrier family 8 (sodium/calcium exchanger), member 3*	*SLC8A3*	5.49	5.71	5.46	0.018211326	0.043435454
8038770	43847	*kallikrein-related peptidase 14*	*KLK14*	6.98	7.04	6.78	0.01821499	0.043440606
8104613				6.55	6.62	6.31	0.018228883	0.043468274
8168422				5.12	4.97	4.75	0.018229602	0.043468274

8098743	22947, 728410, 653548, 653545, 653544, 441056, 653543	*double homeobox 4 | double homeobox 4 like 2 | double homeobox 4 like 3 | double homeobox 4 like 5 | double homeobox 4 like 6 | double homeobox 4 like 4 | double homeobox 4 like 7*	*DUX4|DUX4L2|DUX4L3|DUX4L5|DUX4L6|DUX4L4|DUX4L7*	8.46	8.61	7.89	0.018239357	0.04348794
8050089	7116	*thymosin beta 4, X-linked pseudogene 2*	*TMSB4XP2*	8.94	9.24	8.53	0.018245373	0.04349869
8123259	5340	*plasminogen*	*PLG*	6.03	6.13	5.64	0.018248105	0.043501616
7997010	348174, 497190, 283971	*C-type lectin domain family 18, member A | C-type lectin domain family 18, member B | C-type lectin domain family 18, member C*	*CLEC18A|CLEC18B|CLEC18C*	7.31	7.38	7.04	0.018253418	0.043510687
8133326	9883, 100101267	*POM121 membrane glycoprotein | POM121 membrane glycoprotein C*	*POM121|POM121C*	11.09	11.24	9.90	0.018266214	0.043535847
7954291				4.70	4.68	4.51	0.018266989	0.043535847
7939087	120534	*chromosome 11 open reading frame 46*	*C11orf46*	8.40	8.27	9.02	0.018274345	0.04354978
8158708	59335	*PR domain containing 12*	*PRDM12*	7.80	7.99	7.29	0.018279362	0.043558143
8102938	57484	*ring finger protein 150*	*RNF150*	9.97	8.90	8.46	0.018284332	0.04356639
8150165	84750	*fucosyltransferase 10 (alpha (1,3) fucosyltransferase)*	*FUT10*	7.60	7.47	8.61	0.01829142	0.043576084
8092741	257313	*urotensin 2 domain containing*	*UTS2D*	5.92	5.28	4.99	0.018290322	0.043576084
8073422	7008	*thyrotrophic embryonic factor*	*TEF*	8.28	8.11	7.55	0.018310277	0.04361741
8080810	5793	*protein tyrosine phosphatase, receptor type, G*	*PTPRG*	8.68	8.79	9.81	0.01831258	0.04361867
7977978	10278	*embryonal Fyn-associated substrate*	*EFS*	9.22	8.99	7.87	0.018313829	0.04361867
7953670	360030	*NANOG neighbor homeobox*	*NANOGNB*	5.26	5.18	4.93	0.01832415	0.043636054
7991939	197335, 100507167	*WD repeat domain 90 | hypothetical LOC100507167*	*WDR90|LOC100507167*	7.69	7.74	7.23	0.018323807	0.043636054
8061562				7.88	7.88	7.32	0.018326374	0.04363775
7952436	90952	*endothelial cell adhesion molecule*	*ESAM*	6.93	6.78	6.26	0.018331533	0.043646436
8012309	242	*arachidonate 12-lipoxygenase, 12R type*	*ALOX12B*	6.79	6.86	6.42	0.0183378	0.043657754
8071147	51807	*tubulin, alpha 8*	*TUBA8*	7.25	7.19	6.65	0.018340155	0.04365976

7992043	65990	*family with sequence similarity 173, member A*	*FAM173A*	8.48	8.62	7.87	0.018343799	0.043662757
7966596	115811	*IQ motif containing D*	*IQCD*	6.40	6.30	6.20	0.018347463	0.043662757
8148850	203054	*aarF domain containing kinase 5*	*ADCK5*	7.96	8.09	7.24	0.018346405	0.043662757
8121009	206412	*chromosome 6 open reading frame 163*	*C6orf163*	5.83	5.54	6.11	0.018347425	0.043662757
8091469	131831	*family with sequence similarity 194, member A*	*FAM194A*	5.39	5.51	5.30	0.018356452	0.04368055
7998965				6.30	6.19	5.79	0.01835932	0.04368377
8138662	64111	*neuropeptide VF precursor*	*NPVF*	5.18	5.22	4.93	0.018369408	0.043704174
7909188	9641	*inhibitor of kappa light polypeptide gene enhancer in B-cells, kinase epsilon*	*IKBKE*	8.41	8.50	7.51	0.018371306	0.043705087
8049016	5707	*proteasome (prosome, macropain) 26S subunit, non-ATPase, 1*	*PSMD1*	8.64	9.02	11.08	0.01838105	0.043723207
7963502	374454	*keratin 77*	*KRT77*	6.58	6.63	6.39	0.018383466	0.043723207
8110585				5.10	5.11	4.85	0.01838298	0.043723207
7967337	23457	*ATP-binding cassette, sub-family B (MDR/TAP), member 9*	*ABCB9*	7.64	7.56	6.79	0.018390909	0.043737303
8008139	65264	*ubiquitin-conjugating enzyme E2Z*	*UBE2Z*	11.68	11.83	11.32	0.018393083	0.04373887
7919088	11085	*ADAM metallopeptidase domain 30*	*ADAM30*	5.36	5.44	5.19	0.018394953	0.043739717
8004580	146754	*dynein, axonemal, heavy chain 2*	*DNAH2*	6.22	6.29	5.95	0.018405253	0.043758243
8104607				5.10	4.98	6.10	0.018405776	0.043758243
8099388	9957	*heparan sulfate (glucosamine) 3-O-sulfotransferase 1*	*HS3ST1*	7.25	7.28	6.87	0.018407509	0.043758757
8163086	23731	*chromosome 9 open reading frame 5*	*C9orf5*	9.21	9.07	10.29	0.018445445	0.043845333
8045793				6.01	6.15	5.69	0.018447798	0.043847315
8116313	285679, 100289470	*chromosome 5 open reading frame 60 | chromosome 5 open reading frame 60 pseudogene*	*C5orf60|LOC100289470*	6.46	6.49	6.93	0.018468967	0.043894015
8175844	3421	*isocitrate dehydrogenase 3 (NAD+) gamma*	*IDH3G*	12.01	12.12	11.24	0.018470896	0.043894988
8152094				5.43	5.60	5.18	0.018473102	0.043896616
8162902	84302	*chromosome 9 open reading frame 125*	*C9orf125*	8.15	7.70	7.71	0.018479744	0.043902617

7993359	9284, 339044, 100288332, 642778	*nuclear pore complex interacting protein | polycystic kidney disease 1 (autosomal dominant) pseudogene 1 | GPS, PLAT and transmembrane domain-containing protein FLJ00285-like | nuclear pore complex-interacting protein-like 1-like*	*NPIP|PKD1P1|LOC100288332|LOC642778*	11.62	11.67	10.80	0.01847867	0.043902617
8126310				5.63	5.62	5.24	0.01848019	0.043902617
8009929	643008		*LOC643008*	7.00	7.19	6.74	0.01849194	0.043926917
8128312				6.86	6.85	6.59	0.018500406	0.043943413
8098170	166655	*tripartite motif-containing 60*	*TRIM60*	6.46	6.46	6.14	0.018507374	0.043956347
7926037	5209	*6-phosphofructo-2-kinase/fructose-2,6-biphosphatase 3*	*PFKFB3*	9.94	9.90	9.06	0.018513788	0.043962896
7998157	8131	*nitrogen permease regulator-like 3 (S. cerevisiae)*	*NPRL3*	10.28	10.32	9.23	0.0185147	0.043962896
8154357				10.01	10.01	9.65	0.018512655	0.043962896
8126658	347734	*solute carrier family 35, member B2*	*SLC35B2*	10.54	10.65	9.48	0.018524984	0.04398008
8026265	117579, 9466	*relaxin 3 | interleukin 27 receptor, alpha*	*RLN3|IL27RA*	6.95	6.98	6.56	0.018524427	0.04398008
8165334	9022	*chloride intracellular channel 3*	*CLIC3*	7.82	8.22	7.03	0.018534092	0.043994468
8001691				4.75	4.74	4.63	0.01853284	0.043994468
8053482				5.06	4.95	4.78	0.018568484	0.04407248
8173373	51248	*PDZ domain containing 11*	*PDZD11*	7.60	7.78	7.10	0.01858519	0.044108506
7971573	1102	*regulator of chromosome condensation (RCC1) and BTB (POZ) domain containing protein 2*	*RCBTB2*	7.45	6.98	7.94	0.018597651	0.044130042
7929732	26507	*cyclin M1*	*CNNM1*	7.10	6.96	6.31	0.018597752	0.044130042
7944869	53340, 54414	*sperm autoantigenic protein 17 | sialic acid acetylesterase*	*SPA17|SIAE*	8.93	8.90	8.37	0.01859885	0.044130042
7931473	170393	*chromosome 10 open reading frame 91*	*C10orf91*	6.07	6.17	5.80	0.018600607	0.044130582
8176578	8287	*ubiquitin specific peptidase 9, Y-linked*	*USP9Y*	7.37	6.32	9.04	0.018606728	0.044141475
8036451	83889	*WD repeat domain 87*	*WDR87*	6.21	6.13	5.82	0.018609265	0.044143867
8074153	49	*acrosin*	*ACR*	7.43	7.47	7.04	0.018616887	0.04415832
8138686	9808		*KIAA0087*	5.75	5.72	5.44	0.018623922	0.044171378
8154670	80173	*intraflagellar transport 74 homolog (Chlamydomonas)*	*IFT74*	7.35	6.93	7.71	0.01864197	0.04421055

8179451	9374, 80864	*palmitoyl-protein thioesterase 2 | EGF-like-domain, multiple 8*	*PPT2|EGF L8*	8.79	8.13	7.92	0.01865228	0.044227738
7919602				6.37	6.37	5.96	0.018651418	0.044227738
8032478	645191	*leucine rich repeat and Ig domain containing 3*	*LINGO3*	7.70	7.88	7.34	0.018656688	0.04423455
8095564				4.48	4.48	4.43	0.018663425	0.044246893
8126446	441150	*chromosome 6 open reading frame 226*	*C6orf226*	8.20	8.38	7.34	0.018673178	0.04426638
8068383	54102	*chloride intracellular channel 6*	*CLIC6*	8.38	7.14	7.23	0.01867784	0.04426823
8177261	246119	*testis-specific transcript, Y-linked 10 (non-protein coding)*	*TTTY10*	5.28	5.31	5.09	0.018678559	0.04426823
8075918				6.38	6.48	5.91	0.018676689	0.04426823
8137310	11194	*ATP-binding cassette, sub-family B (MDR/TAP), member 8*	*ABCB8*	8.98	9.12	7.88	0.018685477	0.04428099
8086457	1582	*cytochrome P450, family 8, subfamily B, polypeptide 1*	*CYP8B1*	5.63	5.53	5.38	0.018695394	0.04429722
8110681	116349	*chromosome 5 open reading frame 55*	*C5orf55*	7.27	7.31	6.71	0.018694121	0.04429722
7991815	64714	*protein disulfide isomerase family A, member 2*	*PDIA2*	6.90	7.02	6.52	0.01870418	0.0443144
8114709	89848, 285613	*FCH and double SH3 domains 1 | RELT-like 2*	*FCHSD1|RELL2*	8.44	8.43	7.66	0.018706001	0.044315077
7912606	441871, 391002	*PRAME family member 7 | PRAME family member 8*	*PRAMEF7 |PRAMEF 8*	7.42	7.38	6.89	0.018709775	0.044316746
8103238				5.07	4.98	4.73	0.018708842	0.044316746
8015827	50964	*sclerostin*	*SOST*	7.67	7.82	7.35	0.018716354	0.044328693
7982341	1139	*cholinergic receptor, nicotinic, alpha 7*	*CHRNA7*	7.23	7.46	8.09	0.018727966	0.04434409
7989770	9543	*immunoglobulin superfamily, DCC subclass, member 3*	*IGDCC3*	7.45	7.56	8.06	0.018727915	0.04434409
8036103	374897	*suprabasin*	*SBSN*	7.18	7.49	6.53	0.018725319	0.04434409
8055206				4.40	4.40	4.36	0.018729	0.04434409
8098581	83891	*sorting nexin 25*	*SNX25*	8.07	8.59	9.18	0.018734897	0.044354413
7952274	5818	*poliovirus receptor-related 1 (herpesvirus entry mediator C)*	*PVRL1*	7.09	7.26	7.94	0.018750612	0.044387978
7998336	64428	*nuclear prelamin A recognition factor-like*	*NARFL*	7.80	7.90	7.08	0.018765835	0.044420376
8037535				7.75	7.85	7.28	0.018785825	0.044464048

8096290	56955	*matrix extracellular phosphoglycoprotein*	*MEPE*	5.01	5.04	4.87	0.018803453	0.04450212
8032553	7089	*transducin-like enhancer of split 2 (E(sp1) homolog, Drosophila)*	*TLE2*	8.34	8.85	8.03	0.018816143	0.04452353
8068891	400870		*FLJ41733*	6.37	6.57	6.19	0.018814778	0.04452353
8032922				6.80	6.77	6.19	0.018817125	0.04452353
7942697	726	*calpain 5*	*CAPN5*	9.89	9.53	8.71	0.018821672	0.044530638
8039674	7710	*zinc finger protein 154*	*ZNF154*	7.30	7.12	7.89	0.018845323	0.04457929
8079742				7.04	6.35	5.04	0.018844152	0.04457929
8155083	768	*carbonic anhydrase IX*	*CA9*	7.33	7.44	6.94	0.018855322	0.044596475
7908437	79577	*cell division cycle 73, Paf1/RNA polymerase II complex component, homolog (S. cerevisiae)*	*CDC73*	9.30	9.22	10.35	0.018855678	0.044596475
7913814	25949		*SYF2*	7.43	7.31	7.87	0.018857868	0.044598002
7958466	32	*acetyl-CoA carboxylase beta*	*ACACB*	7.96	7.30	7.44	0.01886395	0.044605076
8079740	474171		*STGC3*	7.21	7.17	6.15	0.018862842	0.044605076
8044067	389015	*solute carrier family 9 (sodium/hydrogen exchanger), member 4*	*SLC9A4*	5.65	5.86	5.61	0.018868126	0.044611298
8016382	80320	*Sp6 transcription factor*	*SP6*	6.96	6.94	6.38	0.018883249	0.044643395
8036902	29950	*SERTA domain containing 1*	*SERTAD1*	7.94	8.36	7.25	0.018887624	0.044650085
7922420	462	*serpin peptidase inhibitor, clade C (antithrombin), member 1*	*SERPINC1*	6.07	6.07	5.69	0.018896166	0.044666618
7897006	8510, 8511	*matrix metallopeptidase 23B | matrix metallopeptidase 23A (pseudogene)*	*MMP23B| MMP23A*	8.38	8.34	7.84	0.018910898	0.044697784
8131661	6144, 10013,1205	*ribosomal protein L21 | ribosomal protein L21 pseudogene 28*	*RPL21|RP L21P28*	10.95	11.07	11.29	0.018917467	0.04470965
8012013	10462	*C-type lectin domain family 10, member A*	*CLEC10A*	6.66	6.64	6.21	0.018921796	0.04471355
8143557	89883	*olfactory receptor, family 6, subfamily W, member 1 pseudogene*	*OR6W1P*	5.58	5.60	5.45	0.018922215	0.04471355
7937878	7221	*transient receptor potential cation channel, subfamily C, member 2, pseudogene*	*TRPC2*	6.27	6.24	5.81	0.018928083	0.044723753
8156024				5.57	5.70	5.35	0.01893444	0.04473511
8086482	353274	*zinc finger protein 445*	*ZNF445*	7.84	7.58	9.12	0.01894144	0.04474799
7984914	594855, 79748	*complexin 3 | lectin, mannose-binding, 1 like*	*CPLX3|LM AN1L*	6.38	6.45	6.10	0.018958127	0.04478375

8076002	23539	*solute carrier family 16, member 8 (monocarboxylic acid transporter 3)*	*SLC16A8*	8.04	8.10	7.61	0.01895999	0.044784483
8015031	1236	*chemokine (C-C motif) receptor 7*	*CCR7*	7.13	6.91	6.65	0.018976588	0.044818625
8023401	80323	*coiled-coil domain containing 68*	*CCDC68*	4.93	4.93	5.68	0.018977549	0.044818625
7897179	7161	*tumor protein p73*	*TP73*	7.41	7.45	7.08	0.018979933	0.044820588
7901565	1733	*deiodinase, iodothyronine, type I*	*DIO1*	5.89	5.91	5.63	0.018983318	0.04482164
8085370				8.10	8.16	7.48	0.018983483	0.04482164
7912224	6518	*solute carrier family 2 (facilitated glucose/fructose transporter), member 5*	*SLC2A5*	7.05	7.43	6.39	0.018989611	0.044822242
8009796	9772		*KIAA0195*	9.96	9.64	8.92	0.018989949	0.044822242
8121916	84870	*R-spondin 3 homolog (Xenopus laevis)*	*RSPO3*	5.77	5.92	5.51	0.018987715	0.044822242
8164560				6.19	6.36	6.01	0.018987486	0.044822242
7935910	10724	*meningioma expressed antigen 5 (hyaluronidase)*	*MGEA5*	9.08	8.72	10.39	0.018997822	0.04483716
8088415	11170	*family with sequence similarity 107, member A*	*FAM107A*	7.98	7.89	7.55	0.019005582	0.044851806
8076056	1454, 400927	*casein kinase 1, epsilon | TPTE and PTEN homologous inositol lipid phosphatase pseudogene*	*CSNK1E|LOC400927*	10.80	10.82	11.07	0.019011773	0.04486275
8038716	284366, 11202	*kallikrein-related peptidase 9 | kallikrein-related peptidase 8*	*KLK9|KLK8*	7.13	7.35	6.76	0.019018563	0.044875104
8007130	83901, 83899, 81870, 85280	*keratin associated protein 9-8 | keratin associated protein 9-2 | keratin associated protein 9-9 | keratin associated protein 9-4*	*KRTAP9-8|KRTAP9-2|KRTAP9-9|KRTAP9-4*	6.99	7.13	6.74	0.019021524	0.044878423
8161915				4.79	4.77	4.67	0.019033268	0.044902463
7942679	55331	*alkaline ceramidase 3*	*ACER3*	7.87	7.55	8.87	0.019043194	0.044922207
8030181	114336, 1082, 93659, 94115, 114335	*chorionic gonadotropin, beta polypeptide 2 | chorionic gonadotropin, beta polypeptide 5 | chorionic gonadotropin, beta polypeptide 8 | chorionic gonadotropin, beta polypeptide 1*	*CGB2|CGB5|CGB8|CGB1*	7.37	7.64	7.00	0.019051846	0.044938944

7901175	10103	*tetraspanin 1*	*TSPAN1*	6.17	6.25	5.67	0.019063445	0.044961706
7964499	116986	*ArfGAP with GTPase domain, ankyrin repeat and PH domain 2*	*AGAP2*	7.05	7.26	6.78	0.01906461	0.044961706
7969472	729420		*LOC729420*	7.12	6.90	6.67	0.019076847	0.04498689
8029136	973	*CD79a molecule, immunoglobulin-associated alpha*	*CD79A*	7.04	7.08	6.52	0.019079195	0.04498875
8166607				6.40	6.67	5.91	0.019083856	0.044996064
8101728	10144	*family with sequence similarity 13, member A*	*FAM13A*	7.43	6.81	7.87	0.019088779	0.045000322
8153935	286101	*zinc finger protein 252*	*ZNF252*	6.42	6.38	7.77	0.019087432	0.045000322
8034390	90576	*zinc finger protein 799*	*ZNF799*	6.52	6.50	7.13	0.019096699	0.045015316
7933047	8453	*cullin 2*	*CUL2*	8.23	8.25	9.35	0.019104281	0.045027178
7956978	55508	*solute carrier family 35, member E3*	*SLC35E3*	8.27	8.87	8.86	0.01910485	0.045027178
7929711	401647	*golgin A7 family, member B*	*GOLGA7B*	6.14	6.22	5.87	0.019108761	0.045029044
7954434				8.41	8.53	7.93	0.019108083	0.045029044
7922846	116496	*family with sequence similarity 129, member A*	*FAM129A*	9.09	9.43	8.39	0.019114526	0.045038953
8003812	8392	*olfactory receptor, family 3, subfamily A, member 3*	*OR3A3*	6.44	6.56	6.24	0.019122165	0.045053273
8163714				10.40	10.45	9.98	0.019138498	0.04508808
8084889				6.31	6.32	5.73	0.019160852	0.045137055
7925589	64754	*SET and MYND domain containing 3*	*SMYD3*	7.57	8.34	7.86	0.019165166	0.045143533
8113481				6.40	6.44	6.05	0.019169034	0.045148965
8165430	89958	*chromosome 9 open reading frame 140*	*C9orf140*	7.43	7.40	6.95	0.019172344	0.045153078
7983826				6.71	6.77	6.37	0.019181581	0.045171145
8000928				5.66	5.66	5.30	0.019191526	0.04519088
8094378	55300	*phosphatidylinositol 4-kinase type 2 beta*	*PI4K2B*	7.01	7.51	7.95	0.019193873	0.04519272
8080998				8.74	9.03	8.23	0.019197673	0.04519798
8121613	51389	*RWD domain containing 1*	*RWDD1*	9.32	9.55	9.10	0.019199507	0.045198612
8036813	163131	*zinc finger protein 780B*	*ZNF780B*	7.25	6.81	8.35	0.019231282	0.045269724
8149762	8797	*tumor necrosis factor receptor superfamily, member 10a*	*TNFRSF10A*	6.89	7.05	7.22	0.01924079	0.045277346
7948511	220001	*von Willebrand factor C and EGF domains*	*VWCE*	7.21	7.37	7.79	0.019237848	0.045277346

7916491				6.28	6.71	6.11	0.019238207	0.045277346
8035863				6.38	6.27	6.02	0.019240795	0.045277346
7926330	644890	*meiosis expressed gene 1 homolog (mouse)*	*MEIG1*	4.73	4.61	4.56	0.019248813	0.045292526
8072733				5.23	5.19	4.90	0.019255957	0.045301948
8141281				6.15	5.94	5.59	0.01925512	0.045301948
7904293	5738	*prostaglandin F2 receptor negative regulator*	*PTGFRN*	9.98	8.85	8.91	0.019265117	0.045312423
8018652	114804	*ring finger protein 157*	*RNF157*	8.29	7.42	8.76	0.019264808	0.045312423
7971690				5.84	6.12	6.70	0.019263864	0.045312423
7981530	29933	*G protein-coupled receptor 132*	*GPR132*	6.40	6.39	6.01	0.019274512	0.045330826
8060850	650	*bone morphogenetic protein 2*	*BMP2*	7.18	6.95	6.64	0.01928906	0.045361344
7980940	4287	*ataxin 3*	*ATXN3*	7.50	7.46	9.07	0.019294359	0.04537011
7927527				7.64	7.32	8.83	0.019297607	0.045374054
8030823	402665	*IgLON family member 5*	*IGLON5*	6.60	7.00	7.73	0.019304447	0.045386437
8066451	140730	*regulating synaptic membrane exocytosis 4*	*RIMS4*	7.28	7.44	7.78	0.019306518	0.04538761
8107977	3565	*interleukin 4*	*IL4*	5.36	5.38	5.19	0.019309472	0.04539086
7943853	6392	*succinate dehydrogenase complex, subunit D, integral membrane protein*	*SDHD*	8.42	8.41	9.04	0.019315735	0.045401882
7979878	6554	*solute carrier family 10 (sodium/bile acid cotransporter family), member 1*	*SLC10A1*	5.87	5.89	5.68	0.019320665	0.045409776
7977571	55644	*O-sialoglycoprotein endopeptidase*	*OSGEP*	7.17	7.33	8.19	0.019328423	0.045424312
7998978	146434	*zinc finger protein 597*	*ZNF597*	7.32	7.40	8.54	0.019349467	0.045470066
8136662	8972	*maltase-glucoamylase (alpha-glucosidase)*	*MGAM*	5.27	5.32	5.01	0.019354017	0.04547706
8040831	51374	*chromosome 2 open reading frame 28*	*C2orf28*	11.97	11.95	11.53	0.019360553	0.04548871
8079392	729230	*chemokine (C-C motif) receptor 2*	*CCR2*	5.32	5.42	5.14	0.019364208	0.045493595
8052742	554251	*F-box protein 48*	*FBXO48*	6.01	6.33	6.54	0.019377176	0.045520358
8086434	152206	*coiled-coil domain containing 13*	*CCDC13*	7.29	7.27	6.91	0.019380344	0.045524098
8123714				6.16	6.15	5.74	0.01938626	0.045534287
7914103	27245	*AT hook, DNA binding motif, containing 1*	*AHDC1*	8.39	8.36	7.36	0.019391395	0.045542646
7912133	100129476		*LOC100129476*	6.58	6.54	6.06	0.019395448	0.04554506

7932451				4.76	4.65	4.61	0.019395579	0.04554506
8006161	26083	*TBC1 domain family, member 29*	*TBC1D29*	6.72	6.92	6.24	0.019411156	0.045577932
8143459				6.32	6.34	5.89	0.019416425	0.045586597
7964234	506	*ATP synthase, H+ transporting, mitochondrial F1 complex, beta polypeptide*	*ATP5B*	12.72	12.72	13.24	0.019421374	0.045594506
8090364	79364	*ZXD family zinc finger C*	*ZXDC*	5.95	6.19	6.25	0.019428119	0.04560663
7948662	404552	*secretoglobin, family 1D, member 4*	*SCGB1D4*	6.16	6.05	5.64	0.019437367	0.045624632
8153704	83482	*scratch homolog 1, zinc finger protein (Drosophila)*	*SCRT1*	6.43	6.47	6.03	0.01944502	0.045637656
8133571	389517, 442590, 441272	*speedy homolog E8 (Xenopus laevis), pseudogene | speedy homolog E5 (Xenopus laevis) | speedy homolog E3 (Xenopus laevis)*	*SPDYE8P|SPDYE5|SPDYE3*	6.80	6.85	6.62	0.019446682	0.045637656
8045167				6.89	6.89	6.57	0.019447658	0.045637656
8129231	79632	*family with sequence similarity 184, member A*	*FAM184A*	5.73	5.69	6.05	0.019450406	0.045640394
7929958	8945	*beta-transducin repeat containing*	*BTRC*	7.82	7.61	9.02	0.019453684	0.045644376
8115490	8728	*ADAM metallopeptidase domain 19*	*ADAM19*	9.83	9.43	8.14	0.019458063	0.04565094
8027719	6324	*sodium channel, voltage-gated, type I, beta*	*SCN1B*	7.82	7.96	7.22	0.019462656	0.045654297
8007919	55073, 374819, 474170	*leucine rich repeat containing 37, member A4 (pseudogene) | leucine rich repeat containing 37, member A3 | leucine rich repeat containing 37, member A2*	*LRRC37A4|LRRC37A3|LRRC37A2*	8.43	8.07	10.42	0.019461505	0.045654297
7906753				7.45	7.65	8.43	0.019467425	0.04566177
8135235	9512	*peptidase (mitochondrial processing) beta*	*PMPCB*	9.32	9.24	10.66	0.019472083	0.045668986
7901135	51249	*transmembrane protein 69*	*TMEM69*	9.26	9.03	10.40	0.019483402	0.04569182
8108977				6.50	6.67	6.30	0.019490797	0.045705453
7914496				6.51	6.81	6.15	0.019496359	0.04571478
7946697	22800	*related RAS viral (r-ras) oncogene homolog 2*	*RRAS2*	10.17	10.58	10.81	0.019510819	0.04574497
8087405	2876	*glutathione peroxidase 1*	*GPX1*	10.71	10.51	10.25	0.019517208	0.045752518
7918558	3752	*potassium voltage-gated channel, Shal-related subfamily, member 3*	*KCND3*	7.74	7.31	6.00	0.019516112	0.045752518
8029671	147700, 2068	*kinesin light chain 3 | excision repair cross-complementing rodent repair deficiency, complementation group 2*	*KLC3|ERCC2*	7.46	7.47	7.08	0.019519178	0.045753423

8165508	441478	*NOTCH-regulated ankyrin repeat protein*	*NRARP*	7.08	7.20	8.10	0.019527787	0.045769885
7993223	23274	*C-type lectin domain family 16, member A*	*CLEC16A*	9.73	9.59	9.27	0.019530984	0.045773663
7944401	3145	*hydroxymethylbilane synthase*	*HMBS*	9.65	9.85	11.63	0.019534271	0.04577765
8058075	66037	*bol, boule-like (Drosophila)*	*BOLL*	5.25	5.36	5.13	0.01954354	0.045795653
8171161	415	*arylsulfatase E (chondrodysplasia punctata 1)*	*ARSE*	9.15	7.46	8.10	0.019558763	0.045827605
8103235	201798	*tigger transposable element derived 4*	*TIGD4*	5.79	5.70	5.99	0.01956245	0.045832522
8170232				5.52	5.42	6.11	0.019572584	0.045852546
8007850	474170, 9884, 374819, 55073	*leucine rich repeat containing 37, member A2 | leucine rich repeat containing 37A | leucine rich repeat containing 37, member A3 | leucine rich repeat containing 37, member A4 (pseudogene)*	*LRRC37A2 |LRRC37A 3|LRRC37 A4*	8.41	8.27	10.04	0.019577429	0.045856453
8151494				5.96	6.06	5.64	0.019576376	0.045856453
7970158	400165	*chromosome 13 open reading frame 35*	*C13orf35*	5.94	6.06	5.60	0.019580986	0.045857344
8043129				5.01	4.75	5.79	0.019580051	0.045857344
7940737	387775	*solute carrier family 22, member 10*	*SLC22A10*	5.26	5.17	4.97	0.019583838	0.0458603
8037482	125931	*carcinoembryonic antigen-related cell adhesion molecule 20*	*CEACAM2 0*	6.18	6.17	5.80	0.019589212	0.045869168
7907232	80133	*chromosome 1 open reading frame 129*	*C1orf129*	5.04	4.67	4.54	0.019593019	0.04587436
8045247	55041	*pleckstrin homology domain containing, family B (evectins) member 2*	*PLEKHB2*	8.49	8.85	10.43	0.019606823	0.045902956
8017634	1655	*DEAD (Asp-Glu-Ala-Asp) box polypeptide 5*	*DDX5*	9.97	9.76	11.70	0.019637022	0.04596993
8057463	375298, 3676	*ceramide kinase-like | integrin, alpha 4 (antigen CD49D, alpha 4 subunit of VLA-4 receptor)*	*CERKL|IT GA4*	5.66	5.74	5.43	0.019641273	0.045976155
8177096	286557, 5940, 378949, 378948	*RNA binding motif protein, Y-linked, family 1, member A3 pseudogene | RNA binding motif protein, Y-linked, family 1, member A1 | RNA binding motif protein, Y-linked, family 1, member D | RNA binding motif protein, Y-linked, family 1, member B*	*RBMY1A3 P|RBMY1A 1|RBMY1D |RBMY1B*	5.04	5.07	4.90	0.019643733	0.045976363
8074309	729461, 26080	*hypothetical LOC729461 | non-protein coding RNA 281*	*LOC72946 1|NCRNA0 0281*	5.47	5.54	5.27	0.019644547	0.045976363
8047736				9.02	9.04	8.53	0.019653335	0.0459932
7967193	283385	*MORN repeat containing 3*	*MORN3*	6.71	6.92	6.40	0.019658128	0.045997303

7960700				5.76	5.63	5.37	0.019659868	0.045997303
8005630				4.84	4.85	4.70	0.01965962	0.045997303
7946561				6.09	5.96	5.59	0.019661848	0.04599821
7948881	54663	*WD repeat domain 74*	*WDR74*	10.72	10.90	11.86	0.019673474	0.046021678
8150206	124801		*LSM12*	12.19	12.39	12.82	0.019675469	0.046022616
8072266	164633, 55954	*calcium binding protein 7 | zinc finger, matrin-type 5*	*CABP7|ZMAT5*	7.02	7.08	6.77	0.019677749	0.04602422
7931479	3632	*inositol polyphosphate-5-phosphatase, 40kDa*	*INPP5A*	10.57	10.30	9.66	0.019679593	0.046024803
7945033	160065	*prostate and testis expressed 1*	*PATE1*	5.82	5.90	5.51	0.019683544	0.046030313
8072721	80831	*apolipoprotein L, 5*	*APOL5*	5.55	5.68	5.42	0.019691966	0.04604628
8147206	8767	*receptor-interacting serine-threonine kinase 2*	*RIPK2*	9.16	9.17	9.76	0.019697228	0.046054855
8038998	90333	*zinc finger protein 468*	*ZNF468*	7.18	7.08	8.58	0.019707082	0.046074163
8059739	4880	*natriuretic peptide C*	*NPPC*	8.91	8.94	8.52	0.019710155	0.0460761
8123006	8871	*synaptojanin 2*	*SYNJ2*	8.61	8.48	7.83	0.019711303	0.0460761
7966760	23014	*F-box protein 21*	*FBXO21*	8.53	8.53	9.54	0.0197127	0.0460761
7947221	55327	*lin-7 homolog C (C. elegans)*	*LIN7C*	9.50	9.36	10.88	0.019717561	0.046083733
8177072	23180	*raftlin, lipid raft linker 1*	*RFTN1*	5.70	5.73	5.50	0.019721873	0.04609008
7970262	8451	*cullin 4A*	*CUL4A*	8.61	8.64	9.45	0.019727847	0.046100307
8069517				6.40	6.34	6.09	0.019739937	0.046124827
8062908	6789	*serine/threonine kinase 4*	*STK4*	7.93	7.76	9.50	0.019755987	0.046158597
8144397	377630, 391627, 392188, 401447, 645836	*ubiquitin specific peptidase 17-like 2 | ubiquitin specific peptidase 17 | ubiquitin specific peptidase 17-like 8 | ubiquitin specific peptidase 17-like 1 (pseudogene) | ubiquitin specific peptidase 17-like 3*	*USP17L2|USP17L8|USP17L1P|USP17L3*	6.59	6.49	6.20	0.019763965	0.046173498
7992716	360226	*protease, serine, 41*	*PRSS41*	7.44	7.61	7.14	0.01978823	0.0462212
8071061	387590	*TPTE pseudogene*	*psiTPTE22*	6.89	6.99	7.70	0.019791618	0.0462212

8007867	474170, 9884, 374819, 55073	*leucine rich repeat containing 37, member A2 | leucine rich repeat containing 37A | leucine rich repeat containing 37, member A3 | leucine rich repeat containing 37, member A4 (pseudogene)*	*LRRC37A2 |LRRC37A 3|LRRC37 A4*	8.44	8.30	10.04	0.019792227	0.0462212
7951347				5.85	5.74	5.37	0.019792331	0.0462212
8053735				6.04	6.20	6.42	0.01979239	0.0462212
7943760	23235	*salt-inducible kinase 2*	*SIK2*	9.29	9.13	8.43	0.01980388	0.046236042
8091432	51122	*COMM domain containing 2*	*COMMD2*	8.93	9.45	11.07	0.019802708	0.046236042
8036936	100130713		*LOC10013 0713*	9.07	8.60	7.95	0.01980257	0.046236042
8016787				7.23	7.12	6.91	0.01980515	0.046236042
8111677	3977	*leukemia inhibitory factor receptor alpha*	*LIFR*	6.60	6.73	8.50	0.019816147	0.046254233
8068192	56246, 9875	*melanocortin 2 receptor accessory protein | URB1 ribosome biogenesis 1 homolog (S. cerevisiae)*	*MRAP|UR B1*	6.50	6.49	6.18	0.01981572	0.046254233
7955129	341568	*olfactory receptor, family 8, subfamily S, member 1*	*OR8S1*	6.03	6.06	5.69	0.019819958	0.04625939
7980096				5.83	5.78	6.64	0.019822406	0.04626136
7964177	4284	*major intrinsic protein of lens fiber*	*MIP*	6.48	6.66	6.18	0.019835448	0.046286866
8021664				5.43	5.49	5.29	0.019836541	0.046286866
8039131	29844, 26121	*TCF3 (E2A) fusion partner (in childhood Leukemia) | PRP31 pre-mRNA processing factor 31 homolog (S. cerevisiae)*	*TFPT|PRP F31*	8.64	8.56	8.09	0.019838436	0.046287544
8170353	158521	*fragile X mental retardation 1 neighbor*	*FMR1NB*	6.63	6.72	6.35	0.019846737	0.04630317
7978712	161198	*C-type lectin domain family 14, member A*	*CLEC14A*	6.72	7.31	6.26	0.019848507	0.04630356
7937016	119467	*clarin 3*	*CLRN3*	5.31	5.34	5.07	0.019862037	0.04633138
8133331	9883, 100101267	*POM121 membrane glycoprotein | POM121 membrane glycoprotein C*	*POM121|P OM121C*	10.45	10.51	9.65	0.01986815	0.046341896
7929047	3433	*interferon-induced protein with tetratricopeptide repeats 2*	*IFIT2*	6.32	6.71	5.05	0.019876717	0.046350386
7925978	54906	*chromosome 10 open reading frame 18*	*C10orf18*	8.11	7.94	9.85	0.019875536	0.046350386
8082583	442092	*hypothetical LOC442092*	*ARVP6125*	5.54	5.71	5.39	0.019874368	0.046350386
7956838				5.15	5.15	4.88	0.019878212	0.046350386
8172204	4129	*monoamine oxidase B*	*MAOB*	6.06	6.32	6.53	0.019883763	0.04635584

8038505	114132	*sialic acid binding Ig-like lectin 11*	*SIGLEC11*	6.55	6.60	6.17	0.01988219	0.04635584
8118669	3833	*kinesin family member C1*	*KIFC1*	7.06	7.62	8.80	0.019891087	0.046365425
8147461	6383	*syndecan 2*	*SDC2*	9.85	10.51	10.38	0.019890025	0.046365425
8140297	100101267, 9883, 100292653	*POM121 membrane glycoprotein C | nuclear envelope pore membrane protein POM 121-like*	*POM121C|LOC100292653*	10.96	11.06	10.02	0.019899406	0.046377327
8004175	84817, 51003, 9851	*thioredoxin domain containing 17 | mediator complex subunit 31 | KIAA0753*	*TXNDC17|MED31|KIAA0753*	7.32	7.64	7.91	0.01989938	0.046377327
8060303	245938	*defensin, beta 125*	*DEFB125*	5.75	5.64	5.23	0.019912394	0.04640385
8155596				9.31	9.52	8.13	0.019914234	0.04640439
7998774	10921	*RNA binding protein S1, serine-rich domain*	*RNPS1*	11.10	11.31	11.81	0.019917458	0.04640816
8027592	26065		*LSM14A*	9.14	9.09	10.69	0.019924844	0.04642162
8104378	11044	*PAP associated domain containing 7*	*PAPD7*	8.97	9.04	8.39	0.01993204	0.04643089
7942594	114599	*small nucleolar RNA, C/D box 15B*	*SNORD15B*	7.88	7.86	8.53	0.01993123	0.04643089
8127841	5238	*phosphoglucomutase 3*	*PGM3*	8.42	8.24	9.81	0.019945325	0.04645809
8020090	57045	*twisted gastrulation homolog 1 (Drosophila)*	*TWSG1*	10.28	10.02	9.23	0.019969303	0.04651019
8115730	407001	*microRNA 218-2*	*MIR218-2*	7.54	7.04	5.90	0.019976655	0.04652356
8068620	6450	*SH3 domain binding glutamic acid-rich protein*	*SH3BGR*	5.99	6.23	6.65	0.019983435	0.046535596
8134745	10734, 352954	*stromal antigen 3 | GATS, stromal antigen 3 opposite strand*	*STAG3|GATS*	6.91	6.82	7.36	0.019998102	0.046565995
8039226	11026	*leukocyte immunoglobulin-like receptor, subfamily A (without TM domain), member 3*	*LILRA3*	6.55	6.73	6.18	0.02001006	0.046590082
7925732	26188	*olfactory receptor, family 1, subfamily C, member 1*	*OR1C1*	5.49	5.54	5.15	0.020017857	0.046604477
7896756	157693	*family with sequence similarity 87, member A*	*FAM87A*	6.56	6.43	6.04	0.020036247	0.046636008
7931726				6.25	6.24	5.96	0.02003372	0.046636008
8111668				5.49	5.46	5.09	0.020034868	0.046636008
8149307	286046	*XK, Kell blood group complex subunit-related family, member 6*	*XKR6*	6.82	6.75	7.02	0.020038983	0.046638615
8118069	394263	*mucin 21, cell surface associated*	*MUC21*	6.96	6.98	6.58	0.02005066	0.046662033
7930996	55717	*WD repeat domain 11*	*WDR11*	7.79	7.74	9.36	0.020052666	0.046662938

7961757	6489	*ST8 alpha-N-acetyl-neuraminide alpha-2,8-sialyltransferase 1*	*ST8SIA1*	6.70	6.77	6.22	0.020058455	0.046672646
8168280	4841	*non-POU domain containing, octamer-binding*	*NONO*	11.86	11.60	12.51	0.020060124	0.04667277
7960362	100128816	*ACAH3104*	*LOC100128816*	7.97	7.22	6.30	0.020065386	0.046681248
7932326	8029	*cubilin (intrinsic factor-cobalamin receptor)*	*CUBN*	6.45	6.65	5.71	0.020070516	0.04668942
8116846				5.81	5.90	5.62	0.02007391	0.046693552
8148674	225689	*mitogen-activated protein kinase 15*	*MAPK15*	7.62	7.58	7.27	0.020076182	0.046695076
8040469	100131373		*LOC100131373*	6.77	6.85	6.49	0.020077959	0.04669545
8129880	64065		*PERP*	11.10	10.13	10.85	0.02008681	0.04671227
7984961	55889, 342096, 645752, 653641, 653643	*golgin A6 family, member B | golgin A6 family, member A | golgi autoantigen, golgin subfamily a, 6 pseudogene | golgin A6 family, member C | golgin A6 family, member D*	*GOLGA6B |GOLGA6A|LOC645752|GOLGA6C|GOLGA6D*	6.68	6.70	6.41	0.02009544	0.04672481
8173915				4.47	4.56	4.39	0.020094607	0.04672481
7933593				7.64	7.33	8.83	0.020101096	0.0467342
8076365	27351	*PPPDE peptidase domain containing 2*	*PPPDE2*	11.42	11.59	10.48	0.020106776	0.04674364
8144422	389616, 403296	*olfactory receptor, family 7, subfamily E, member 125 pseudogene | olfactory receptor, family 7, subfamily E, member 154 pseudogene*	*OR7E125P |OR7E154P*	7.45	7.38	6.89	0.020110385	0.046748266
8179814	80740	*lymphocyte antigen 6 complex, locus G6C*	*LY6G6C*	6.92	6.90	6.58	0.02012084	0.046765037
8122658				6.37	6.32	5.15	0.020119451	0.046765037
8063857	26164	*GTP binding protein 5 (putative)*	*GTPBP5*	8.42	8.47	7.93	0.020133495	0.046790686
8106633	4437, 1719	*mutS homolog 3 (E. coli) | dihydrofolate reductase*	*MSH3|DHFR*	8.16	8.38	9.32	0.020135455	0.04679147
8117594	8342	*histone cluster 1, H2bm*	*HIST1H2BM*	11.43	12.10	13.91	0.020139696	0.046793792
8133500				6.57	6.56	6.15	0.020139517	0.046793792
7945241	283171	*chromosome 11 open reading frame 44*	*C11orf44*	6.41	6.46	6.00	0.020147877	0.046805266

8143494	730441	*trypsin X3 pseudogene*	*LOC730441*	5.36	5.44	5.22	0.02014707	0.046805266
8116740	6239	*ras responsive element binding protein 1*	*RREB1*	8.60	8.77	8.23	0.02016375	0.046838373
8067530	253868	*chromosome 20 open reading frame 200*	*C20orf200*	7.99	7.94	7.62	0.020175932	0.046853684
7902265				7.50	7.70	7.02	0.020176833	0.046853684
8073941				7.29	7.35	6.77	0.020176396	0.046853684
8119239				4.45	4.45	4.51	0.020173607	0.046853684
7944147	486	*FXYD domain containing ion transport regulator 2*	*FXYD2*	6.78	6.86	6.34	0.020181267	0.04686021
7900119	9967	*thyroid hormone receptor associated protein 3*	*THRAP3*	8.89	9.17	10.51	0.020187233	0.04687029
8029476	23624	*Cas-Br-M (murine) ecotropic retroviral transforming sequence c*	*CBLC*	7.10	7.27	7.67	0.02019778	0.04689101
7986639				6.75	6.77	6.22	0.020215908	0.046929322
8011322	4991	*olfactory receptor, family 1, subfamily D, member 2*	*OR1D2*	6.03	5.92	5.71	0.020220466	0.04693613
8142852	23534	*transportin 3*	*TNPO3*	9.02	9.10	11.12	0.02023046	0.04695224
8156581	375748	*chromosome 9 open reading frame 102*	*C9orf102*	7.57	7.10	8.59	0.02023066	0.04695224
8149273				5.84	5.70	6.02	0.020234557	0.04695751
8028916	6626	*small nuclear ribonucleoprotein polypeptide A*	*SNRPA*	11.27	11.25	12.16	0.020255	0.04700117
7950067	1717	*7-dehydrocholesterol reductase*	*DHCR7*	10.61	9.53	10.82	0.020258328	0.04700282
7938301	56672	*chromosome 11 open reading frame 17*	*C11orf17*	9.40	9.36	8.56	0.020258965	0.04700282
8101065	5470	*protein phosphatase, EF-hand calcium binding domain 2*	*PPEF2*	6.15	6.18	5.81	0.020265345	0.04701384
8054846	6344	*secretin receptor*	*SCTR*	6.60	6.74	6.28	0.020278221	0.047038544
7942071				5.93	6.01	5.36	0.020279251	0.047038544
7972215				6.44	6.00	7.60	0.020286692	0.047052022
8048847	3267	*ArfGAP with FG repeats 1*	*AGFG1*	11.11	10.93	12.25	0.02028876	0.04705304
8054227	51455		*REV1*	7.46	7.52	8.65	0.020294894	0.047053546
8167866	203569	*P antigen family, member 2 (prostate associated)*	*PAGE2*	4.96	5.01	4.74	0.020295499	0.047053546
7951341				6.29	6.24	5.55	0.020292072	0.047053546
8105479				4.79	4.79	4.55	0.020294726	0.047053546

8144392	641517, 245912	*defensin, beta 109, pseudogene 1B | defensin, beta 109, pseudogene 1*	*DEFB109 P1B*	5.89	5.59	5.41	0.020302696	0.0470644
8141746				5.22	5.34	4.89	0.020303441	0.0470644
8008723	8288	*eosinophil peroxidase*	*EPX*	6.69	6.73	6.28	0.020306364	0.047067396
7979721	161145	*transmembrane protein 229B*	*TMEM229 B*	6.83	6.22	6.12	0.02031035	0.047072854
7906417	57863	*cell adhesion molecule 3*	*CADM3*	7.04	7.10	7.54	0.020313138	0.04707554
8082270	348807	*coiled-coil domain containing 37*	*CCDC37*	6.46	6.49	6.14	0.020355634	0.04716645
8046833	401024	*fibrous sheath interacting protein 2*	*FSIP2*	5.16	5.10	5.66	0.020354342	0.04716645
8017212	84669	*ubiquitin specific peptidase 32*	*USP32*	8.86	8.69	10.50	0.02036098	0.04717505
8071212	8318	*cell division cycle 45 homolog (S. cerevisiae)*	*CDC45*	7.08	7.83	9.54	0.020376364	0.047206905
7997582	58189	*WAP four-disulfide core domain 1*	*WFDC1*	10.34	9.03	7.19	0.020387132	0.04722806
8073334	9978, 63929	*ring-box 1, E3 ubiquitin protein ligase | X-prolyl aminopeptidase (aminopeptidase P) 3, putative*	*RBX1|XPN PEP3*	10.72	11.08	11.51	0.020393604	0.047239266
7963614	3695	*integrin, beta 7*	*ITGB7*	6.80	6.91	6.38	0.020399157	0.047248337
7946436	56676	*achaete-scute complex homolog 3 (Drosophila)*	*ASCL3*	5.88	6.07	5.68	0.020413017	0.047276646
8122099	5167	*ectonucleotide pyrophosphatase/phosphodiesterase 1*	*ENPP1*	9.89	8.24	11.07	0.020419978	0.047288973
7899790	81629	*testis-specific serine kinase 3*	*TSSK3*	6.88	6.64	6.37	0.020423979	0.047294445
8036913	645	*biliverdin reductase B (flavin reductase (NADPH))*	*BLVRB*	9.74	9.33	8.53	0.02042792	0.047299776
8060427	140901	*serine/threonine kinase 35*	*STK35*	8.93	8.99	9.67	0.020440275	0.04732459
8109732	27430	*methionine adenosyltransferase II, beta*	*MAT2B*	8.23	8.31	9.38	0.020442167	0.047325175
7984802	57611	*immunoglobulin superfamily containing leucine-rich repeat 2*	*ISLR2*	6.72	6.85	6.14	0.020446245	0.04733082
8015014				4.39	4.40	4.42	0.020462928	0.047365643
8150584				5.06	4.95	4.78	0.020468393	0.047374494
7965767				5.06	5.49	4.54	0.020471953	0.04737893
7910022	149111	*cornichon homolog 3 (Drosophila)*	*CNIH3*	6.43	6.60	6.74	0.020474557	0.047381163
8046524	3239	*homeobox D13*	*HOXD13*	7.52	7.54	7.08	0.020483356	0.047397725
8172447	27344	*proprotein convertase subtilisin/kexin type 1 inhibitor*	*PCSK1N*	8.99	9.17	8.41	0.020487895	0.047404427
8154660				5.39	5.34	5.27	0.020492708	0.047411766

7908155				5.47	5.30	5.06	0.020495005	0.04741328
7979982				8.33	8.27	7.76	0.020497195	0.047414545
8137040				5.37	5.23	4.95	0.020501066	0.0474197
8149638	9046	*docking protein 2, 56kDa*	*DOK2*	6.74	6.84	6.33	0.020513378	0.047444377
7903090				4.40	4.58	4.41	0.020530814	0.0474809
8010328	55036	*coiled-coil domain containing 40*	*CCDC40*	7.30	7.23	7.00	0.020541899	0.04750273
7996945				4.76	4.72	4.56	0.020545226	0.047506616
7912102	9903	*kelch-like 21 (Drosophila)*	*KLHL21*	7.90	7.85	7.20	0.020548712	0.047510874
7904281	388667	*chromosome 1 open reading frame 137*	*C1orf137*	4.95	5.01	4.84	0.020551832	0.047514282
8086330	64651	*cysteine-serine-rich nuclear protein 1*	*CSRNP1*	7.38	8.04	7.23	0.020554716	0.047517143
8085206	8536, 4968	*calcium/calmodulin-dependent protein kinase I | 8-oxoguanine DNA glycosylase*	*CAMK1|OGG1*	9.13	9.24	8.07	0.020564143	0.04753513
8133959				5.54	5.58	5.34	0.020576708	0.047560368
8046759	54431	*DnaJ (Hsp40) homolog, subfamily C, member 10*	*DNAJC10*	8.94	8.70	10.13	0.020586768	0.04757981
8151042	7274	*tocopherol (alpha) transfer protein*	*TTPA*	5.50	5.52	5.37	0.020588836	0.04758078
8020347				5.63	4.96	5.19	0.020627728	0.047666844
8004922	9340	*glucagon-like peptide 2 receptor*	*GLP2R*	6.09	5.96	5.68	0.020633081	0.047675397
7929649	80019	*ubiquitin domain containing 1*	*UBTD1*	9.64	9.78	8.93	0.020635508	0.04767719
8035684	83983, 51079	*testis-specific serine kinase 6 | NADH dehydrogenase (ubiquinone) 1 alpha subcomplex, 13*	*TSSK6|NDUFA13*	6.73	6.72	6.32	0.020637857	0.0476788
8051866	64240	*ATP-binding cassette, sub-family G (WHITE), member 5*	*ABCG5*	5.83	5.85	5.51	0.020640876	0.04768196
7921449	1401	*C-reactive protein, pentraxin-related*	*CRP*	5.50	5.59	5.23	0.020652154	0.0477038
7935983	5309	*paired-like homeodomain 3*	*PITX3*	7.50	7.56	7.03	0.020653635	0.0477038
8071368	84222, 728229, 645426	*transmembrane protein 191A | transmembrane protein 191B | transmembrane protein 191C*	*TMEM191A|TMEM191B|TMEM191C*	9.73	9.63	8.96	0.020660589	0.047716044
7901287	199974	*cytochrome P450, family 4, subfamily Z, polypeptide 1*	*CYP4Z1*	6.16	6.15	5.78	0.020666815	0.047726605
8119396	222642	*translocator protein 2*	*TSPO2*	6.54	6.63	6.32	0.020679262	0.04774771
7942820				7.56	7.54	6.93	0.020677637	0.04774771

7992584	1775	*deoxyribonuclease I-like 2*	*DNASE1L 2*	6.85	6.99	6.49	0.02068263	0.047751665
8160771	347240	*kinesin family member 24*	*KIF24*	6.18	6.30	6.53	0.020687137	0.04775825
7979611	7597	*zinc finger and BTB domain containing 25*	*ZBTB25*	7.60	7.82	8.55	0.020696152	0.047775246
8019494	79701, 284004	*chromosome 17 open reading frame 101 | hexosaminidase (glycosyl hydrolase family 20, catalytic domain) containing*	*C17orf101| HEXDC*	8.26	8.24	7.36	0.020697871	0.04777539
8148985	157697	*glutamate-rich 1*	*ERICH1*	7.89	8.05	8.66	0.020706149	0.04779068
8002370	6483	*ST3 beta-galactoside alpha-2,3-sialyltransferase 2*	*ST3GAL2*	9.77	9.83	8.77	0.020716358	0.0478066
8069156	386678	*keratin associated protein 10-11*	*KRTAP10-11*	6.92	6.93	6.55	0.020715252	0.0478066
8037872	27113	*BCL2 binding component 3*	*BBC3*	8.69	8.87	8.21	0.020722467	0.04781525
8000974	79759	*zinc finger protein 668*	*ZNF668*	7.98	8.18	7.19	0.02072342	0.04781525
8093112	26043	*UBX domain protein 7*	*UBXN7*	9.29	8.96	11.01	0.02073084	0.047826607
8053648	51315	*lysine-rich coiled-coil 1*	*KRCC1*	8.50	8.66	7.57	0.020731656	0.047826607
7989491	7168, 100128979	*tropomyosin 1 (alpha) | hypothetical LOC100128979*	*TPM1|LOC100128979*	7.53	7.58	7.06	0.02074234	0.04784743
8124636				4.89	4.91	4.68	0.020744175	0.04784784
8144444	441324, 441327, 441317, 441328, 441326, 728753, 389633, 441315, 645572, 389611, 441332, 645558, 100287045	*family with sequence similarity 90, member A8 | family with sequence similarity 90, member A9 | family with sequence similarity 90, member A7 | family with sequence similarity 90, member A10 | family with sequence similarity 90, member A18 | family with sequence similarity 90, member A19 | family with sequence similarity 90 pseudogene | family with sequence similarity 90, member A5 | family with sequence similarity 90, member A23 pseudogene | family with sequence similarity 90, member A3 | family with sequence similarity 90, member A24 (pseudogene) | family with sequence similarity 90, member A22 pseudogene | family with sequence similarity 90, member A-like*	*FAM90A8| FAM90A9| FAM90A7| FAM90A10 |FAM90A18|FAM90A19|FAM90A25P|FAM90A5|FAM90A23|FAM90A3|FAM90A24P| FAM90A22 |LOC100287045*	7.24	7.26	6.74	0.020748867	0.04785484

7981752	729786, 283768, 100132979, 390535, 100132565, 653061, 100510725	*golgin A8 family, member C | golgin A8 family, member G | golgin A8 family, member D (pseudogene) | golgin A8 family, member E | golgin A8 family, member F | Golgin subfamily A member 8-like protein 2-like | putative golgin subfamily A member 8C-like*	*GOLGA8C|GOLGA8G|GOLGA8DP|GOLGA8E|GOLGA8F|LOC653061|LOC100510725*	6.55	6.52	6.42	0.020750696	0.047855236
8074108	23542	*mitogen-activated protein kinase 8 interacting protein 2*	*MAPK8IP2*	7.32	7.44	6.90	0.020759001	0.04786292
7973403	116173	*CKLF-like MARVEL transmembrane domain containing 5*	*CMTM5*	6.76	6.70	6.29	0.020757651	0.04786292
7993017				6.94	7.01	6.54	0.02075682	0.04786292
8007427	8639	*amine oxidase, copper containing 3 (vascular adhesion protein 1)*	*AOC3*	8.32	8.32	7.79	0.020766964	0.047877453
7980485	1734	*deiodinase, iodothyronine, type II*	*DIO2*	6.33	5.46	5.26	0.020776834	0.047893513
8163784	26190	*F-box and WD repeat domain containing 2*	*FBXW2*	9.22	9.36	10.30	0.020777248	0.047893513
7949444	10089	*potassium channel, subfamily K, member 7*	*KCNK7*	7.56	7.65	7.18	0.020785235	0.0479081
8118509	9374, 80864	*palmitoyl-protein thioesterase 2 | EGF-like-domain, multiple 8*	*PPT2|EGFL8*	8.80	8.13	7.93	0.020790596	0.04791663
7899436	83667	*sestrin 2*	*SESN2*	9.66	10.29	8.61	0.020802097	0.04793931
7969390	10464, 22894	*progesterone immunomodulatory binding factor 1 | DIS3 mitotic control homolog (S. cerevisiae)*	*PIBF1|DIS3*	7.02	7.09	7.51	0.02080545	0.047943212
8099255	768239	*prosaposin-like 1 (gene/pseudogene)*	*PSAPL1*	6.79	6.93	6.36	0.020821823	0.047975555
8088977				5.21	5.22	4.99	0.02082281	0.047975555
8021645	5273	*serpin peptidase inhibitor, clade B (ovalbumin), member 10*	*SERPINB10*	5.52	5.68	5.31	0.02085308	0.048033796
8068898	85236, 8344, 54145	*histone cluster 1, H2bk | histone cluster 1, H2be | H2B histone family, member S*	*HIST1H2BK|HIST1H2BE|H2BFS*	11.47	11.74	12.43	0.020851353	0.048033796
8052678				4.52	4.56	4.45	0.020852119	0.048033796
8057821	56171	*dynein, axonemal, heavy chain 7*	*DNAH7*	5.19	5.20	5.02	0.02085541	0.04803533

8145334	8756	*ADAM metallopeptidase domain 7*	*ADAM7*	4.93	4.95	4.85	0.02086415	0.048051182
7956426	83729	*inhibin, beta E*	*INHBE*	6.66	8.50	7.04	0.020865621	0.048051182
7925058	645339	*small nuclear ribonucleoprotein D2 pseudogene 2*	*SNRPD2P 2*	5.69	5.65	5.43	0.020870669	0.04805897
8158418	2021, 51490	*endonuclease G | chromosome 9 open reading frame 114*	*ENDOG|C 9orf114*	8.41	8.51	8.04	0.020882092	0.048081335
8129375				8.34	8.53	7.62	0.020883711	0.048081335
7918008	1629	*dihydrolipoamide branched chain transacylase E2*	*DBT*	7.34	6.82	9.29	0.020901265	0.048113313
8103867	152641	*chromosome 4 open reading frame 38*	*C4orf38*	7.08	6.96	6.53	0.0209026	0.048113313
7962199				4.71	4.80	4.70	0.020901175	0.048113313
8024582	8698	*sphingosine-1-phosphate receptor 4*	*S1PR4*	6.81	6.93	6.18	0.020911265	0.048126303
7976976	9895	*tectonin beta-propeller repeat containing 2*	*TECPR2*	8.56	8.36	7.67	0.020911578	0.048126303
8084912	727956, 6389, 255812	*succinate dehydrogenase complex, subunit A, flavoprotein pseudogene 2 | succinate dehydrogenase complex, subunit A, flavoprotein (Fp) | succinate dehydrogenase complex, subunit A, flavoprotein pseudogene 1*	*SDHAP2|S DHAP1*	9.81	9.34	9.16	0.02092228	0.048147097
8079368				5.75	5.67	5.95	0.020926226	0.048152335
8155574	84210, 441425, 441430, 728747	*ankyrin repeat domain 20 family, member A1 | ankyrin repeat domain 20 family, member A3 | ankyrin repeat domain 20 family, member A2 | ankyrin repeat domain 20 family, member A4*	*ANKRD20 A1|ANKR D20A3|AN KRD20A2| ANKRD20 A4*	5.48	5.46	5.38	0.020930754	0.04815508
8172713				9.55	9.59	8.82	0.020930754	0.04815508
8090893	55167	*male-specific lethal 2 homolog (Drosophila)*	*MSL2*	10.84	10.69	11.78	0.020954713	0.04820252
8169439				6.08	5.95	5.66	0.020953625	0.04820252
8101011				4.88	4.91	4.52	0.020957895	0.048205998
7991661				7.34	7.41	6.68	0.020959755	0.048206437
8104115				7.87	7.68	8.39	0.020963196	0.048210505
8146230	1142	*cholinergic receptor, nicotinic, beta 3*	*CHRNB3*	5.21	5.26	5.08	0.020966737	0.048214808
7980454	8846	*alkB, alkylation repair homolog 1 (E. coli)*	*ALKBH1*	8.03	8.11	9.26	0.020974182	0.048224248

8003621	55178	*RNA methyltransferase like 1*	*RNMTL1*	7.89	7.84	8.58	0.020973753	0.048224248
7926127	10659	*CUGBP, Elav-like family member 2*	*CELF2*	7.19	7.19	8.50	0.020980174	0.048233904
7930631	56165	*tudor domain containing 1*	*TDRD1*	5.32	5.18	5.77	0.020981723	0.048233904
8170520	1260	*cyclic nucleotide gated channel alpha 2*	*CNGA2*	6.17	6.29	5.93	0.020983968	0.048234157
7912567	343068, 440561, 653619, 343070, 400735, 440560, 645359	*PRAME family member 5 | PRAME family member 6 | PRAME family member 15 | PRAME family member 9 | PRAME family member 4 | PRAME family member 11*	*PRAMEF5 |PRAMEF 6|PRAME F15|PRAM EF9|PRA MEF4|PR AMEF11|L OC645359*	5.75	5.84	5.61	0.020986846	0.048234157
7983405	405753, 90527	*dual oxidase maturation factor 2 | dual oxidase maturation factor 1*	*DUOXA2| DUOXA1*	6.54	6.65	6.05	0.020985771	0.048234157
8089185	50939	*interphotoreceptor matrix proteoglycan 2*	*IMPG2*	5.68	5.56	5.36	0.02099024	0.048238117
8092769	344905	*ATPase type 13A5*	*ATP13A5*	5.33	5.40	5.19	0.02099582	0.0482471
7951112	8898	*myotubularin related protein 2*	*MTMR2*	9.72	9.70	11.03	0.020999985	0.048252832
8069532	6782	*heat shock protein 70kDa family, member 13*	*HSPA13*	8.71	9.55	9.52	0.021025589	0.048303973
7979730				4.38	4.39	4.35	0.021024281	0.048303973
7965873	3479	*insulin-like growth factor 1 (somatomedin C)*	*IGF1*	6.90	7.14	5.82	0.021033362	0.048317984
8043937	55571	*chromosome 2 open reading frame 29*	*C2orf29*	9.94	9.87	10.34	0.021041516	0.04833287
8126240	401260	*hypothetical LOC401260*	*FLJ41649*	6.12	5.92	5.83	0.021053417	0.048356358
7937882	417	*ADP-ribosyltransferase 1*	*ART1*	6.66	6.77	6.28	0.021065362	0.0483684
8029854	6510	*solute carrier family 1 (neutral amino acid transporter), member 5*	*SLC1A5*	7.58	7.82	6.79	0.02106076	0.0483684
7978756	84075	*fibrous sheath CABYR binding protein*	*FSCB*	5.07	5.09	4.84	0.02106528	0.0483684
8133219				8.60	8.64	8.12	0.021065021	0.0483684
7924987	183	*angiotensinogen (serpin peptidase inhibitor, clade A, member 8)*	*AGT*	7.15	7.10	6.31	0.021072093	0.048372317
7896817	9636		*ISG15*	7.46	7.57	7.03	0.021070048	0.048372317
8010313	339221	*ectonucleotide pyrophosphatase/phosphodiesterase 7*	*ENPP7*	7.94	7.98	7.34	0.02107165	0.048372317

8095422	6779	*statherin*	*STATH*	6.03	5.14	4.95	0.02108432	0.048396535
8176719	9086	*eukaryotic translation initiation factor 1A, Y-linked*	*EIF1AY*	8.22	6.58	9.43	0.021091444	0.04840904
8019170	284185	*chromosome 17 open reading frame 55*	*C17orf55*	8.01	8.28	7.51	0.021102019	0.04842561
7932019	414196	*chromosome 10 open reading frame 31*	*C10orf31*	6.02	6.02	5.71	0.021101609	0.04842561
7995438				6.42	6.46	6.18	0.021114208	0.048449732
7944042	5049	*platelet-activating factor acetylhydrolase 1b, catalytic subunit 2 (30kDa)*	*PAFAH1B2*	9.83	10.14	11.58	0.021116126	0.048450284
8019937				7.13	7.16	6.64	0.021119323	0.048453767
8034827	10755	*GIPC PDZ domain containing family, member 1*	*GIPC1*	9.05	9.13	8.17	0.021122567	0.048457358
8129924	167838	*taxilin beta*	*TXLNB*	6.03	5.89	5.70	0.021145107	0.048505213
8101507	4825	*NK6 homeobox 1*	*NKX6-1*	7.54	7.85	7.07	0.021153538	0.048512988
8178790	63940	*G-protein signaling modulator 3*	*GPSM3*	8.86	8.75	7.95	0.021150235	0.048512988
8149241	377630, 391627, 392188, 401447, 645836	*ubiquitin specific peptidase 17-like 2 | ubiquitin specific peptidase 17 | ubiquitin specific peptidase 17-like 8 | ubiquitin specific peptidase 17-like 1 (pseudogene) | ubiquitin specific peptidase 17-like 3*	*USP17L2| USP17L8| USP17L1P |USP17L3*	6.58	6.49	6.19	0.02115334	0.048512988
8038809	4818	*natural killer cell group 7 sequence*	*NKG7*	7.76	7.85	7.16	0.02116488	0.04853246
8039236	353514	*leukocyte immunoglobulin-like receptor, subfamily A (with TM domain), member 5*	*LILRA5*	5.84	6.01	5.68	0.021165391	0.04853246
8104746	4883	*natriuretic peptide receptor C/guanylate cyclase C (atrionatriuretic peptide receptor C)*	*NPR3*	7.47	7.05	5.42	0.0211772	0.048551828
7913835	6006, 6007	*Rh blood group, CcEe antigens | Rh blood group, D antigen*	*RHCE|RH D*	5.74	6.10	5.54	0.02117623	0.048551828
7916384	200008, 606495	*CUB domain containing protein 2 | cytochrome b5 reductase-like*	*CDCP2|C YB5RL*	6.29	6.41	6.02	0.021181507	0.048557844
7911278	26696	*olfactory receptor, family 2, subfamily T, member 1*	*OR2T1*	6.13	6.14	5.86	0.02118673	0.04856596
7898894	57095	*chromosome 1 open reading frame 128*	*C1orf128*	8.57	8.59	9.32	0.021200554	0.048593793
8003077	146167	*solute carrier family 38, member 8*	*SLC38A8*	6.99	7.03	6.60	0.021208944	0.048608396
8161913				5.82	5.81	5.10	0.021210294	0.048608396
7949792	5790	*protein tyrosine phosphatase, receptor type, C-associated protein*	*PTPRCAP*	7.90	7.94	7.50	0.02121746	0.048620958

8080645	26060	*adaptor protein, phosphotyrosine interaction, PH domain and leucine zipper containing 1*	*APPL1*	8.83	8.57	9.87	0.021222368	0.048628345
8089743	8702	*UDP-Gal:betaGlcNAc beta 1,4- galactosyltransferase, polypeptide 4*	*B4GALT4*	7.15	7.39	8.50	0.021226753	0.048634533
8089112	11259	*filamin A interacting protein 1-like*	*FILIP1L*	7.48	7.20	5.89	0.021230632	0.048639562
8109651	2559	*gamma-aminobutyric acid (GABA) A receptor, alpha 6*	*GABRA6*	5.68	5.76	5.42	0.021235116	0.048645973
8015273	3881	*keratin 31*	*KRT31*	6.81	6.96	6.46	0.02124259	0.04865453
7902992	79871	*RNA polymerase II associated protein 2*	*RPAP2*	8.12	7.86	8.90	0.021243908	0.04865453
8095467	260436	*chromosome 4 open reading frame 7*	*C4orf7*	5.43	5.36	5.13	0.021243647	0.04865453
7934842	79812	*multimerin 2*	*MMRN2*	6.57	6.63	6.27	0.021246945	0.04865763
7946504	440026	*transmembrane protein 41B*	*TMEM41B*	8.14	8.55	9.52	0.021251515	0.04866423
8175215				5.67	5.56	5.30	0.021253208	0.04866425
8135821	442721	*leiomodin 2 (cardiac)*	*LMOD2*	6.29	6.19	5.93	0.021260804	0.04867778
7954810	120892	*leucine-rich repeat kinase 2*	*LRRK2*	6.45	5.82	5.10	0.02126536	0.04868049
7998405	146336		*LOC146336*	8.06	7.98	7.46	0.021264158	0.04868049
8017310				4.45	4.48	4.37	0.021282095	0.04871494
7973006	161271	*olfactory receptor, family 4, subfamily H, member 12 pseudogene*	*OR4H12P*	4.76	4.69	4.64	0.021287197	0.048722755
8161701	23670	*transmembrane protein 2*	*TMEM2*	7.34	7.64	9.49	0.021290528	0.048726514
7966397	6311	*ataxin 2*	*ATXN2*	11.64	11.37	10.77	0.021308431	0.04876362
7948157	283189	*olfactory receptor, family 9, subfamily G, member 4*	*OR9G4*	5.63	5.73	5.44	0.021321386	0.0487894
8123959				5.49	5.45	5.05	0.021350019	0.04885105
8121365	2309	*forkhead box O3*	*FOXO3*	7.19	7.57	6.77	0.021353852	0.04885595
8106271	10412		*NSA2*	10.92	10.82	11.29	0.021382987	0.048914853
7969341	81550	*tudor domain containing 3*	*TDRD3*	8.77	8.72	8.39	0.021382436	0.048914853
8049582	51540, 140739	*selenocysteine lyase | ubiquitin-conjugating enzyme E2F (putative)*	*SCLY|UBE2F*	8.72	8.73	9.88	0.021387402	0.048921075
8046048	80034	*cysteine-serine-rich nuclear protein 3*	*CSRNP3*	6.48	6.58	7.46	0.021389434	0.04892185
7924340				5.86	5.73	5.53	0.021393059	0.048922386
8143710				6.69	6.91	5.97	0.021391647	0.048922386

8148463	56988	*chromosome 8 open reading frame 17*	*C8orf17*	6.51	6.57	6.17	0.02139831	0.04893052
7973968				5.73	5.97	5.60	0.021405421	0.048939817
8170235				6.70	6.85	6.32	0.021405766	0.048939817
8103289	5356	*pleiotropic regulator 1 (PRL1 homolog, Arabidopsis)*	*PLRG1*	9.66	9.69	10.59	0.0214181	0.048959002
7963134	57701	*NCK-associated protein 5-like*	*NCKAP5L*	7.74	7.88	6.93	0.02142094	0.048959002
8136934	259287	*taste receptor, type 2, member 41*	*TAS2R41*	6.31	6.42	6.18	0.021420073	0.048959002
7957602				4.88	4.92	4.68	0.021420134	0.048959002
8103834	175	*aspartylglucosaminidase*	*AGA*	8.89	8.98	8.14	0.02142468	0.04896367
8104443				4.90	4.71	4.64	0.021429203	0.048970133
8088476				4.63	5.45	4.41	0.021431308	0.048971064
7982938	7301		*TYRO3*	7.92	8.57	9.63	0.021437902	0.048978377
8021540				5.60	5.84	5.39	0.021436578	0.048978377
8115664				7.08	7.33	6.45	0.02144229	0.048984528
8090464	165721	*DnaJ (Hsp40) homolog, subfamily B, member 8*	*DNAJB8*	6.62	6.61	6.31	0.021447632	0.048992854
8129087				5.69	5.68	5.41	0.021452878	0.04900096
8149289	83595	*SRY (sex determining region Y)-box 7*	*SOX7*	7.21	7.35	6.78	0.021461321	0.049016368
8096098	51138	*COP9 constitutive photomorphogenic homolog subunit 4 (Arabidopsis)*	*COPS4*	9.40	9.48	10.07	0.021472191	0.049037315
8007118	728318	*keratin associated protein 9-1*	*KRTAP9-1*	7.14	6.94	6.41	0.021475183	0.049040265
8019517				6.00	6.12	5.74	0.021486044	0.049061187
8081462	84692	*coiled-coil domain containing 54*	*CCDC54*	5.25	5.15	4.99	0.021494081	0.049071778
8028940	112398	*egl nine homolog 2 (C. elegans)*	*EGLN2*	8.21	8.06	7.68	0.021492595	0.049071778
7905374	5710	*proteasome (prosome, macropain) 26S subunit, non-ATPase, 4*	*PSMD4*	8.26	7.89	8.48	0.02152111	0.0491296
8038919	59348	*zinc finger protein 350*	*ZNF350*	7.37	7.25	7.97	0.021527067	0.049139313
8095566	92597	*MOB1, Mps One Binder kinase activator-like 1A (yeast)*	*MOBKL1A*	7.72	7.53	8.65	0.021537583	0.049155544
7948371				5.27	5.33	4.91	0.02153636	0.049155544
7970448	10804	*gap junction protein, beta 6, 30kDa*	*GJB6*	6.17	6.24	5.74	0.021544997	0.049164753

8161943	644714		*LOC644714*	7.29	7.46	8.25	0.021545025	0.049164753
7940005	5024	*purinergic receptor P2X, ligand-gated ion channel, 3*	*P2RX3*	6.65	6.65	6.40	0.021552593	0.04917814
7939477	221120, 729799	*alkB, alkylation repair homolog 3 (E. coli) | SEC14-like 1 pseudogene*	*ALKBH3|LOC729799*	9.27	9.32	8.76	0.02156778	0.0492089
8121622	345895	*radial spoke head 4 homolog A (Chlamydomonas)*	*RSPH4A*	5.35	5.38	5.67	0.021569775	0.049209565
8048466				5.33	5.34	5.13	0.02157816	0.0492248
8151816	2669	*GTP binding protein overexpressed in skeletal muscle*	*GEM*	7.89	7.79	7.13	0.021589978	0.049247872
7957159				5.47	5.50	5.30	0.021597056	0.049260125
8016609	1747	*distal-less homeobox 3*	*DLX3*	6.46	6.50	6.12	0.02160675	0.049274452
8039257	3903	*leukocyte-associated immunoglobulin-like receptor 1*	*LAIR1*	6.40	6.43	6.17	0.021605426	0.049274452
7980523	2957	*general transcription factor IIA, 1, 19/37kDa*	*GTF2A1*	8.87	8.54	10.46	0.021609496	0.049274497
8102214	9061	*3'-phosphoadenosine 5'-phosphosulfate synthase 1*	*PAPSS1*	7.74	7.91	9.65	0.021614194	0.049274497
7954752	10059	*dynamin 1-like*	*DNM1L*	7.79	7.82	9.97	0.021612883	0.049274497
7940182	390197, 219986, 390199	*olfactory receptor, family 4, subfamily D, member 10 | olfactory receptor, family 4, subfamily D, member 11 | olfactory receptor, family 4, subfamily D, member 9*	*OR4D10|OR4D11|OR4D9*	7.08	7.18	6.56	0.021610364	0.049274497
8093128				6.34	6.45	6.16	0.021615304	0.049274497
8097553	3600	*interleukin 15*	*IL15*	6.43	6.06	5.46	0.021628411	0.049300484
8011337	4994	*olfactory receptor, family 3, subfamily A, member 1*	*OR3A1*	5.96	6.09	5.56	0.02165176	0.049343806
8091306	57088	*phospholipid scramblase 4*	*PLSCR4*	8.49	8.10	6.54	0.021649914	0.049343806
8036270	199745	*THAP domain containing 8*	*THAP8*	8.74	9.04	8.04	0.021652546	0.049343806
7946986	6288	*serum amyloid A1*	*SAA1*	4.78	4.90	4.64	0.021656206	0.049348254
8054297	9486	*carbohydrate sulfotransferase 10*	*CHST10*	9.24	9.02	9.74	0.021669684	0.04937507
8094342	133015	*PARK2 co-regulated-like*	*PACRGL*	7.94	8.15	9.23	0.021674257	0.049381588
7954010				5.51	5.46	5.04	0.021679783	0.049390282
8172719	401589, 100129860	*hypothetical LOC401589 | hypothetical LOC100129860*	*LOC401589|LOC100129860*	6.02	6.21	5.78	0.021687724	0.049404472

7903565	29899, 23155	*G-protein signaling modulator 2 | chloride channel CLIC-like 1*	*GPSM2|CLCC1*	7.08	7.41	8.11	0.021693321	0.049409427
8161362	654466, 387628, 2252, 403323	*keratinocyte growth factor-like protein 2 | keratinocyte growth factor-like protein 1 | fibroblast growth factor 7 | hypothetical protein FLJ20444*	*KGFLP2|KGFLP1|FGF7|FLJ20444*	6.93	7.13	6.24	0.021691784	0.049409427
7966621	10993	*serine dehydratase*	*SDS*	6.53	6.54	6.27	0.021707935	0.049434878
7994647	112476	*proline-rich transmembrane protein 2*	*PRRT2*	8.73	8.21	7.86	0.021709632	0.049434878
7924386				8.14	8.02	7.63	0.021709187	0.049434878
7900888	9670	*importin 13*	*IPO13*	9.38	9.56	8.41	0.021719404	0.049453225
8160276				5.85	5.90	5.34	0.021744775	0.04950709
7916616	1573	*cytochrome P450, family 2, subfamily J, polypeptide 2*	*CYP2J2*	5.87	5.85	6.20	0.021749923	0.049514905
7986426	196968	*dynamin 1 pseudogene*	*C15orf51*	6.01	6.19	5.52	0.021757392	0.049528006
7902738	22802	*chloride channel accessory 4*	*CLCA4*	5.12	5.04	4.98	0.021764833	0.049537133
8110668				7.23	6.71	8.62	0.02176326	0.049537133
8007290	6945	*MAX-like protein X*	*MLX*	10.58	10.75	9.93	0.02177129	0.04954792
8004195	162517	*F-box protein 39*	*FBXO39*	6.59	6.43	6.23	0.021773743	0.049549595
8136645	50832	*taste receptor, type 2, member 4*	*TAS2R4*	5.79	5.53	6.94	0.02178441	0.049569964
7985605	9640	*zinc finger protein 592*	*ZNF592*	10.06	10.15	9.21	0.021798026	0.04959581
8149320	157740	*hypothetical protein C8orf9*	*LOC157740*	6.58	6.46	7.39	0.021799205	0.04959581
8123362	1235	*chemokine (C-C motif) receptor 6*	*CCR6*	6.92	6.90	6.45	0.021814328	0.049626306
8105146	167359	*serine/threonine-protein kinase NIM1*	*MGC42105*	6.29	6.63	6.06	0.021826832	0.04965084
7964248	26789	*small nucleolar RNA, C/D box 59A*	*SNORD59A*	5.72	5.81	6.58	0.021835268	0.04966594
8086572	79443	*FYVE and coiled-coil domain containing 1*	*FYCO1*	8.85	7.90	7.80	0.02183691	0.04966594
7902645	64173, 2787	*spermatogenesis associated 1 | guanine nucleotide binding protein (G protein), gamma 5*	*SPATA1|GNG5*	5.20	5.20	4.94	0.021841528	0.04967253
8020889	84307	*zinc finger protein 397*	*ZNF397*	7.11	6.95	7.94	0.021845236	0.049674995
7977854	84962	*jub, ajuba homolog (Xenopus laevis)*	*JUB*	10.40	10.37	9.48	0.021846054	0.049674995

8144432	441324, 441327, 441317, 441328, 441326, 728753, 441314, 441315, 645572, 441332, 389611, 389633	*family with sequence similarity 90, member A8*	*FAM90A8*	7.23	7.25	6.74	0.021876434	0.049740158
8110858	728613	*programmed cell death 6 pseudogene*	*LOC728613*	9.24	9.28	8.99	0.021878216	0.049740292
8051462				5.34	5.03	6.85	0.021881672	0.04974423
8011341	8388	*olfactory receptor, family 1, subfamily E, member 2*	*OR1E2*	4.80	4.90	4.55	0.02189738	0.049776018
8132660	10268	*receptor (G protein-coupled) activity modifying protein 3*	*RAMP3*	8.27	8.33	7.81	0.021900065	0.049778204
8107330	324	*adenomatous polyposis coli*	*APC*	6.48	6.10	7.58	0.02191943	0.049814373
8073474	150365	*meiosis inhibitor 1*	*MEI1*	6.17	6.10	5.77	0.021918112	0.049814373
7960177	6539	*solute carrier family 6 (neurotransmitter transporter, betaine/GABA), member 12*	*SLC6A12*	6.59	6.63	6.35	0.021926349	0.049819212
8136591	90956	*aarF domain containing kinase 2*	*ADCK2*	7.79	7.84	6.81	0.021926735	0.049819212
8054511	205251	*non-protein coding RNA 116*	*NCRNA00116*	7.08	7.13	6.35	0.021923903	0.049819212
8149500	57509	*microtubule associated tumor suppressor 1*	*MTUS1*	5.82	5.43	6.08	0.021959893	0.049890622
7942228	220070	*chromosome 11 open reading frame 76*	*C11orf76*	7.41	7.30	7.01	0.021963358	0.049894568
7918313	343263	*myosin binding protein H-like*	*MYBPHL*	6.33	6.34	5.87	0.021978507	0.049925055
7958942	387885	*coiled-coil domain containing 42B*	*CCDC42B*	7.23	7.28	6.72	0.021981362	0.04992761
8033385	92960	*peroxisomal biogenesis factor 11 gamma*	*PEX11G*	8.34	8.39	7.74	0.021983614	0.0499288
7928316				5.06	5.07	4.80	0.021997565	0.049956553
7902911				6.38	6.49	5.91	0.02201122	0.04998363

Supplementary Table S2. Gene list in Figure 3D.

Entrez GeneID	*Symbol*	*Gene name*
196883	*ADCY4*	*adenylate cyclase 4*
115	*ADCY9*	*adenylate cyclase 9*
147	*ADRA1B*	*adrenoceptor alpha 1B*
408	*ARRB1*	*arrestin, beta 1*
624	*BDKRB2*	*bradykinin receptor B2*
652	*BMP4*	*bone morphogenetic protein 4*
718	*C3*	*complement component 3*
720	*C4A*	*complement component 4A (Rodgers blood group)*
1675	*CFD*	*complement factor D (adipsin)*
1129	*CHRM2*	*cholinergic receptor, muscarinic 2*
9547	*CXCL14*	*chemokine (C-X-C motif) ligand 14*
58191	*CXCL16*	*chemokine (C-X-C motif) ligand 16*
81624	*DIAPH3*	*diaphanous-related formin 3*
9829	*DNAJC6*	*DnaJ (Hsp40) homolog, subfamily C, member 6*
1909	*EDNRA*	*endothelin receptor type A*
1910	*EDNRB*	*endothelin receptor type B*
1956	*EGFR*	*epidermal growth factor receptor*
30845	*EHD3*	*EH-domain containing 3*
2044	*EPHA5*	*EPH receptor A5*
2051	*EPHB6*	*EPH receptor B6*
2159	*F10*	*coagulation factor X*
2149	*F2R*	*coagulation factor II (thrombin) receptor*
8817	*FGF18*	*fibroblast growth factor 18*
2277	*FIGF*	*c-fos induced growth factor (vascular endothelial growth factor D)*
2321	*FLT1*	*fms-related tyrosine kinase 1*
2869	*GRK5*	*G protein-coupled receptor kinase 5*
3082	*HGF*	*hepatocyte growth factor (hepapoietin A; scatter factor)*
3481	*IGF2*	*insulin-like growth factor 2*
8516	*ITGA8*	*integrin, alpha 8*
3949	*LDLR*	*low density lipoprotein receptor*
4286	*MITF*	*microphthalmia-associated transcription factor*
5228	*PGF*	*placental growth factor*
5468	*PPARG*	*peroxisome proliferator-activated receptor gamma*
5727	*PTCH1*	*patched 1*
2185	*PTK2B*	*protein tyrosine kinase 2 beta*
6092	*ROBO2*	*roundabout, axon guidance receptor, homolog 2 (Drosophila)*
223117	*SEMA3D*	*sema domain, immunoglobulin domain (Ig), short basic domain, secreted, (semaphorin) 3D*
6405	*SEMA3F*	*sema domain, immunoglobulin domain (Ig), short basic domain, secreted, (semaphorin) 3F*
10507	*SEMA4D*	*sema domain, immunoglobulin domain (Ig), transmembrane domain (TM) and short cytoplasmic domain, (semaphorin) 4D*
53358	*SHC3*	*SHC (Src homology 2 domain containing) transforming protein 3*
4088	*SMAD3*	*SMAD family member 3*
83439	*TCF7L1*	*transcription factor 7-like 1 (T-cell specific, HMG-box)*
7035	*TFPI*	*tissue factor pathway inhibitor (lipoprotein-associated coagulation inhibitor)*
7042	*TGFB2*	*transforming growth factor, beta 2*
7448	*VTN*	*vitronectin*
51384	*WNT16*	*wingless-type MMTV integration site family, member 16*

Supplementary Table S3. Gene list in Figure 3E.

Entrez GeneID	*Symbol*	*Gene name*
595	*CCND1*	*cyclin D1*
1311	*COMP*	*cartilage oligomeric matrix protein*
4616	*GADD45B*	*growth arrest and DNA-damage-inducible, beta*
3486	*IGFBP3*	*insulin-like growth factor binding protein 3*
22801	*ITGA11*	*integrin, alpha 11*
3655	*ITGA6*	*integrin, alpha 6*
56034	*PDGFC*	*platelet derived growth factor C*
5054	*SERPINE1*	*serpin peptidase inhibitor, clade E (plasminogen activator inhibitor type 1), member 1*
3371	*TNC*	*tenascin C*

Supplementary Table S4. Gene list in G1 categorized in Figure 4A.

[hOFs = hOF-iPSCs > hDFs (58 genes)]

Entrez GeneID	*Symbol*	*Gene name*	hOFs*	hDFs*	hOF-iPSCs*
**ECM/Secreted**					
652	*BMP4*	*bone morphogenetic protein 4*	1,145.64	171.93	723.07
5764	*PTN*	*pleiotrophin*	445.93	108.86	236.02
6414	*SEPP1*	*selenoprotein P, plasma, 1*	566.50	162.79	581.10
**Membrane protein**					
928	*CD9*	*CD9 molecule*	1,765.96	302.50	2,569.37
54805	*CNNM2*	*cyclin M2*	621.14	260.96	473.88
286097	*EFHA2*	*EF-hand domain family, member A2*	233.76	110.00	161.69
2719	*GPC3*	*glypican 3*	1,148.59	83.85	2,052.12
182	*JAG1*	*jagged 1*	572.88	201.35	506.70
154141	*MBOAT1*	*membrane bound O-acyltransferase domain containing 1*	723.38	191.11	1,087.47
81832	*NETO1*	*neuropilin (NRP) and tolloid (TLL)-like 1*	197.37	52.57	261.25
22871	*NLGN1*	*neuroligin 1*	428.97	95.37	256.63
10507	*SEMA4D*	*sema domain, immunoglobulin domain (Ig), transmembrane domain (TM) and short cytoplasmic domain, (semaphorin) 4D*	322.63	92.17	609.81
**Receptor**					
1909	*EDNRA*	*endothelin receptor type A*	627.40	101.39	318.24
1910	*EDNRB*	*endothelin receptor type B*	830.53	48.39	777.82
2149	*F2R*	*coagulation factor II (thrombin) receptor*	840.23	243.10	422.44
51704	*GPRC5B*	*G protein-coupled receptor, family C, group 5, member B*	3,062.22	331.04	4,258.31
3949	*LDLR*	*low density lipoprotein receptor*	2,242.41	1,100.47	1,697.41
10200	*MPHOSPH6*	*M-phase phosphoprotein 6*	199.15	96.47	380.36
23596	*OPN3*	*opsin 3*	522.00	254.57	295.57
5638	*PRRG1*	*proline rich Gla (G-carboxyglutamic acid) 1*	382.29	167.54	348.33
5727	*PTCH1*	*patched 1*	1,042.34	373.70	1,736.94
154091	*SLC2A12*	*solute carrier family 2 (facilitated glucose transporter), member 12*	603.62	58.76	318.58
**Enzyme**					
51363	*CHST15*	*carbohydrate (N-acetylgalactosamine 4-sulfate 6-O) sulfotransferase 15*	530.65	190.96	309.00
1717	*DHCR7*	*7-dehydrocholesterol reductase*	1,567.48	737.66	1,807.00
2053	*EPHX2*	*epoxide hydrolase 2, cytoplasmic*	133.74	58.30	90.70
55224	*ETNK2*	*ethanolamine kinase 2*	793.70	268.64	718.60
8630	*HSD17B6*	*hydroxysteroid (17-beta) dehydrogenase 6 homolog (mouse)*	146.90	48.68	115.49
25840	*METTL7A*	*methyltransferase like 7A*	279.46	103.78	188.35
221692	*PHACTR1*	*phosphatase and actin regulator 1*	293.74	133.44	179.63

9262	*STK17B*	*serine/threonine kinase 17b*	1,707.03	525.32	908.08
**Signaling**					
9590	*AKAP12*	*A kinase (PRKA) anchor protein 12*	558.88	101.52	675.64
408	*ARRB1*	*arrestin, beta 1*	1,816.62	464.48	1,062.94
9829	*DNAJC6*	*DnaJ (Hsp40) homolog, subfamily C, member 6*	244.47	100.57	131.13
9732	*DOCK4*	*dedicator of cytokinesis 4*	299.23	89.40	394.20
25827	*FBXL2*	*F-box and leucine-rich repeat protein 2*	387.80	142.04	196.52
3485	*IGFBP2*	*insulin-like growth factor binding protein 2, 36kDa*	3,214.92	345.69	1,837.77
90293	*KLHL13*	*kelch-like 13 (Drosophila)*	118.70	43.03	159.32
9079	*LDB2*	*LIM domain binding 2*	1,260.05	428.25	800.85
114876	*OSBPL1A*	*oxysterol binding protein-like 1A*	385.91	187.71	530.29
5287	*PIK3C2B*	*phosphoinositide-3-kinase, class 2, beta polypeptide*	248.11	114.91	243.96
54477	*PLEKHA5*	*pleckstrin homology domain containing, family A member 5*	544.34	173.03	1,062.80
5997	*RGS2*	*regulator of G-protein signaling 2, 24kDa*	606.02	96.03	456.93
**Transcriptional regulation**					
2114	*ETS2*	*v-ets erythroblastosis virus E26 oncogene homolog 2 (avian)*	740.82	348.60	621.02
51804	*SIX4*	*SIX homeobox 4*	825.37	399.69	1,327.50
56849	*TCEAL7*	*transcription elongation factor A (SII)-like 7*	353.00	175.74	221.59
83439	*TCF7L1*	*transcription factor 7-like 1 (T-cell specific, HMG-box)*	1,981.90	829.81	1,863.20
7088	*TLE1*	*transducin-like enhancer of split 1 (E(sp1) homolog, Drosophila)*	830.79	319.75	1,129.28
9760	*TOX*	*thymocyte selection-associated high mobility group box*	1,289.94	301.64	782.68
**Others**					
81563	*C1orf21*	*chromosome 1 open reading frame 21*	1,799.54	718.36	1,488.53
81624	*DIAPH3*	*diaphanous homolog 3 (Drosophila)*	361.94	156.90	617.43
7802	*DNALI1*	*dynein, axonemal, light intermediate chain 1*	617.56	265.48	597.25
54536	*EXOC6*	*exocyst complex component 6*	143.21	70.12	166.65
83641	*FAM107B*	*family with sequence similarity 107, member B*	437.79	211.14	347.81
221303	*FAM162B*	*family with sequence similarity 162, member B*	1,349.86	576.11	818.38
256227	*STEAP1B*	*STEAP family member 1B*	359.74	144.26	402.06
54970	*TTC12*	*tetratricopeptide repeat domain 12*	241.10	95.59	175.52
125488	*TTC39C*	*tetratricopeptide repeat domain 39C*	1,445.40	530.95	908.84
57507	*ZNF608*	*zinc finger protein 608*	473.56	147.91	815.54

***The numbers indicate average signal values in each cell type.**

Supplementary Table S5. Gene list in G2 categorized in Figure 4A.

[hOFs > hOF-iPSCs = hDFs (103 genes)]

Entrez GeneID	*Symbol*	*Gene name*	hOFs*	hDFs*	hOF-iPSCs*
**ECM/Secreted**					
170689	*ADAMTS15*	*ADAM metallopeptidase with thrombospondin type 1 motif, 15*	1,002.73	147.39	119.07
9547	*CXCL14*	*chemokine (C-X-C motif) ligand 14*	935.32	229.85	231.33
58191	*CXCL16*	*chemokine (C-X-C motif) ligand 16*	371.76	125.23	126.61
25975	*EGFL6*	*EGF-like-domain, multiple 6*	497.45	88.14	101.46
2201	*FBN2*	*fibrillin 2*	5,342.37	446.65	225.84
8817	*FGF18*	*fibroblast growth factor 18*	1,140.65	270.03	164.82
2277	*FIGF*	*c-fos induced growth factor (vascular endothelial growth factor D)*	92.12	40.63	36.24
342035	*GLDN*	*gliomedin*	164.76	65.80	54.88
3082	*HGF*	*hepatocyte growth factor (hepapoietin A; scatter factor)*	252.34	57.54	44.97
4685	*NCAM2*	*neural cell adhesion molecule 2*	143.67	67.59	52.89
5179	*PENK*	*proenkephalin*	2,914.14	175.21	103.79
5228	*PGF*	*placental growth factor*	1,484.07	214.93	119.91
284654	*RSPO1*	*R-spondin 1*	698.57	291.67	148.91
340419	*RSPO2*	*R-spondin 2*	260.65	81.29	66.31
6405	*SEMA3F*	*sema domain, immunoglobulin domain (Ig), short basic domain, secreted, (semaphorin) 3F*	575.91	228.16	245.48
51384	*WNT16*	*wingless-type MMTV integration site family, member 16*	211.59	99.22	69.88
**Membrane protein**					
54805	*CNNM2*	*cyclin M2*	621.14	260.96	473.88
10584	*COLEC10*	*collectin sub-family member 10 (C-type lectin)*	137.68	59.88	48.13
2039	*EPB49*	*erythrocyte membrane protein band 4.9 (dematin)*	310.87	133.16	77.72
3383	*ICAM1*	*intercellular adhesion molecule 1*	579.56	246.95	160.73
223117	*SEMA3D*	*sema domain, immunoglobulin domain (Ig), short basic domain, secreted, (semaphorin) 3D*	932.65	244.67	221.81
56241	*SUSD2*	*sushi domain containing 2*	424.90	177.73	138.88
222865	*TMEM130*	*transmembrane protein 130*	1,224.19	358.83	218.42
219623	*TMEM26*	*transmembrane protein 26*	153.13	58.15	51.67
11045	*UPK1A*	*uroplakin 1A*	270.13	108.42	107.83
**Receptor**					
2044	*EPHA5*	*EPH receptor A5*	195.17	52.39	46.50
2051	*EPHB6*	*EPH receptor B6*	901.41	234.08	196.97
2149	*F2R*	*coagulation factor II (thrombin) receptor*	840.23	243.10	422.44
2151	*F2RL2*	*coagulation factor II (thrombin) receptor-like 2*	1,388.20	97.04	106.69
2828	*GPR4*	*G protein-coupled receptor 4*	339.03	168.19	109.05

3949	*LDLR*	*low density lipoprotein receptor*	2,242.41	1,100.47	1,697.41
8013	*NR4A3*	*nuclear receptor subfamily 4, group A, member 3*	812.63	175.44	93.30
23596	*OPN3*	*opsin 3*	522.00	254.57	295.57
6092	*ROBO2*	*roundabout, axon guidance receptor, homolog 2 (Drosophila)*	871.92	72.79	81.39
1903	*S1PR3*	*sphingosine-1-phosphate receptor 3*	486.04	222.20	177.63
57559	*STAMBPL1*	*STAM binding protein-like 1*	1,331.74	246.44	151.22
7098	*TLR3*	*toll-like receptor 3*	264.38	102.71	81.30
**Channel**					
54102	*CLIC6*	*chloride intracellular channel 6*	333.86	140.65	149.92
286097	*EFHA2 (=MICU3)*	*EF-hand domain family, member A2*	233.76	110.00	161.69
3788	*KCNS2*	*potassium voltage-gated channel, delayed-rectifier, subfamily S, member 2*	384.36	157.62	107.72
6335	*SCN9A*	*sodium channel, voltage-gated, type IX, alpha subunit*	594.07	109.09	109.71
**Transport**					
9829	*DNAJC6 (auxilin)*	*DnaJ (Hsp40) homolog, subfamily C, member 6*	244.47	100.57	131.13
30845	*EHD3*	*EH-domain containing 3*	1,716.78	695.62	358.06
30061	*SLC40A1*	*solute carrier family 40 (iron-regulated transporter), member 1*	220.16	86.22	60.44
28232	*SLCO3A1*	*solute carrier organic anion transporter family, member 3A1*	1,871.92	630.79	683.84
64220	*STRA6*	*stimulated by retinoic acid gene 6 homolog (mouse)*	426.14	170.43	142.68
**Enzyme**					
81792	*ADAMTS12*	*ADAM metallopeptidase with thrombospondin type 1 motif, 12*	1,039.43	355.23	310.06
126	*ADH1C*	*alcohol dehydrogenase 1C (class I), gamma polypeptide*	196.14	82.88	43.81
217	*ALDH2*	*aldehyde dehydrogenase 2 family (mitochondrial)*	2,581.97	1,040.36	1,137.16
415	*ARSE*	*arylsulfatase E (chondrodysplasia punctata 1)*	567.49	176.13	273.43
834	*CASP1*	*caspase 1, apoptosis-related cysteine peptidase*	139.16	47.90	26.82
51363	*CHST15*	*carbohydrate (N-acetylgalactosamine 4-sulfate 6-O) sulfotransferase 15*	530.65	190.96	309.00
2053	*EPHX2*	*epoxide hydrolase 2, cytoplasmic*	133.74	58.30	90.70
2138	*EYA1*	*eyes absent homolog 1 (Drosophila)*	693.37	84.87	117.43
2326	*FMO1*	*flavin containing monooxygenase 1*	88.92	36.71	31.44
3294	*HSD17B2*	*hydroxysteroid (17-beta) dehydrogenase 2*	309.54	94.48	72.46
11185	*INMT*	*indolethylamine N-methyltransferase*	499.36	206.20	109.09
57134	*MAN1C1*	*mannosidase, alpha, class 1C, member 1*	813.28	304.28	206.89
25840	*METTL7A*	*methyltransferase like 7A*	279.46	103.78	188.35
2185	*PTK2B*	*PTK2B protein tyrosine kinase 2 beta*	1,029.69	234.43	124.39
401494	*PTPLAD2*	*protein tyrosine phosphatase-like A domain containing 2*	625.76	236.72	143.25

9262	*STK17B*	*serine/threonine kinase 17b*	1,707.03	525.32	908.08
11274	*USP18*	*ubiquitin specific peptidase 18*	800.73	298.52	284.62
**Signaling**					
57569	*ARHGAP20*	*Rho GTPase activating protein 20*	178.88	86.49	47.74
9411	*ARHGAP29*	*Rho GTPase activating protein 29*	627.56	260.05	150.02
56892	*C8orf4*	*chromosome 8 open reading frame 4*	168.84	82.94	69.87
154043	*CNKSR3*	*CNKSR family member 3*	460.36	144.04	113.63
25827	*FBXL2=SCF*	*F-box and leucine-rich repeat protein 2*	387.80	142.04	196.52
10586	*MAB21L2*	*mab-21-like 2 (C. elegans)*	1,108.96	87.40	58.62
117583	*PARD3B*	*par-3 partitioning defective 3 homolog B (C. elegans)*	478.38	221.64	172.10
221692	*PHACTR1*	*phosphatase and actin regulator 1*	293.74	133.44	179.63
5738	*PTGFRN*	*prostaglandin F2 receptor negative regulator*	1,011.31	460.16	482.20
5920	*RARRES3*	*retinoic acid receptor responder (tazarotene induced) 3*	822.85	183.22	122.87
387496	*RASL11A*	*RAS-like, family 11, member A*	562.03	109.31	110.51
9770	*RASSF2*	*Ras association (RalGDS/AF-6) domain family member 2*	2,222.80	511.12	353.93
83937	*RASSF4*	*Ras association (RalGDS/AF-6) domain family member 4*	844.54	133.25	179.19
6000	*RGS7*	*regulator of G-protein signaling 7*	341.54	76.01	104.05
83853	*ROPN1L*	*rhophilin associated tail protein 1-like*	763.00	225.85	123.32
8991	*SELENBP1*	*selenium binding protein 1*	2,774.32	878.88	659.08
53358	*SHC3*	*SHC (Src homology 2 domain containing) transforming protein 3*	797.94	209.13	250.88
7035	*TFPI*	*tissue factor pathway inhibitor (lipoprotein-associated coagulation inhibitor)*	411.99	123.24	69.91
**Transcriptional regulation**					
1749	*DLX5*	*distal-less homeobox 5*	418.74	106.42	81.89
2114	*ETS2*	*v-ets erythroblastosis virus E26 oncogene homolog 2 (avian)*	740.82	348.60	621.02
2294	*FOXF1*	*forkhead box F1*	768.86	182.83	101.53
9079	*LDB2*	*LIM domain binding 2*	1,260.05	428.25	800.85
431707	*LHX8*	*LIM homeobox 8*	735.18	68.93	57.84
4286	*MITF*	*microphthalmia-associated transcription factor*	227.47	111.40	103.55
5083	*PAX9*	*paired box 9*	4,117.50	102.03	75.43
5307	*PITX1*	*paired-like homeodomain 1*	1,039.93	281.68	163.81
5468	*PPARG*	*peroxisome proliferator-activated receptor gamma*	524.44	100.81	62.96
56849	*TCEAL7*	*transcription elongation factor A (SII)-like 7*	353.00	175.74	221.59
**Others**					
147463	*ANKRD29*	*ankyrin repeat domain 29*	410.47	139.90	97.52
83938	*C10orf11*	*chromosome 10 open reading frame 11*	323.62	108.12	60.18
152078	*C3orf55*	*chromosome 3 open reading frame 55*	379.27	166.05	130.17

83641	*FAM107B*	*family with sequence similarity 107, member B*	437.79	211.14	347.81
221303	*FAM162B*	*family with sequence similarity 162, member B*	1,349.86	576.11	818.38
54855	*FAM46C*	*family with sequence similarity 46, member C*	326.17	95.40	89.89
144363	*LYRM5*	*LYR motif containing 5*	1,026.89	437.41	289.32
57523	*NYNRIN*	*NYN domain and retroviral integrase containing*	1,838.88	532.23	388.31
22874	*PLEKHA6*	*pleckstrin homology domain containing, family A member 6*	723.64	253.54	247.55
54970	*TTC12*	*tetratricopeptide repeat domain 12*	241.10	95.59	175.52
125488	*TTC39C*	*tetratricopeptide repeat domain 39C*	1,445.40	530.95	908.84
51186	*WBP5*	*WW domain binding protein 5*	550.84	181.33	200.36

***The numbers indicate average signal values in each cell type.**

Supplementary Table S6. Gene list in G3 categorized in Figure 4A.

[hDFs > hOFs = hOF-iPSCs (70 genes)]

Entrez GeneID	*Symbol*	*Gene name*	hOFs*	hDFs*	hOF-iPSCs*
**ECM/Secreted**					
51129	*ANGPTL4*	*angiopoietin-like 4*	157.23	322.41	114.71
392255	*GDF6*	*growth differentiation factor 6*	110.65	320.69	82.52
83729	*INHBE*	*inhibin, beta E*	101.29	361.81	131.59
3885	*KRT34*	*keratin 34*	96.51	396.44	49.84
56034	*PDGFC*	*platelet derived growth factor C*	175.70	502.13	123.82
11341	*SCRG1*	*stimulator of chondrogenesis 1*	66.24	143.64	45.59
7292	*TNFSF4*	*tumor necrosis factor (ligand) superfamily, member 4*	73.65	407.97	73.64
**Membrane protein**					
3084	*NRG1*	*neuregulin 1*	194.47	401.57	123.72
8828	*NRP2*	*neuropilin 2*	163.95	502.96	290.66
4908	*NTF3*	*neurotrophin 3*	93.99	292.12	61.57
4978	*OPCML*	*opioid binding protein/cell adhesion molecule-like*	66.32	239.11	126.11
8482	*SEMA7A*	*semaphorin 7A, GPI membrane anchor (John Milton Hagen blood group)*	368.52	1096.90	339.94
80329	*ULBP1*	*UL16 binding protein 1*	226.29	838.48	161.63
**Receptor**					
150	*ADRA2A*	*adrenoceptor alpha 2A*	358.04	807.06	253.66
9568	*GABBR2*	*gamma-aminobutyric acid (GABA) B receptor, 2*	190.81	439.48	170.06
2861	*GPR37*	*G protein-coupled receptor 37 (endothelin receptor type B-like)*	81.05	186.10	145.51
2898	*GRIK2*	*glutamate receptor, ionotropic, kainate 2*	56.26	126.45	60.08
5798	*PTPRN*	*protein tyrosine phosphatase, receptor type, N*	167.46	864.93	171.57
51330	*TNFRSF12A*	*tumor necrosis factor receptor superfamily, member 12A*	1814.14	4302.70	1907.53
219699	*UNC5B*	*unc-5 homolog B (C. elegans)*	668.99	2425.98	574.30
**Enzyme**					
978	*CDA*	*cytidine deaminase*	134.37	474.77	238.77
9023	*CH25H*	*cholesterol 25-hydroxylase*	130.93	1883.66	160.70
11072	*DUSP14*	*dual specificity phosphatase 14*	437.06	1031.69	322.69
2026	*ENO2*	*enolase 2 (gamma, neuronal)*	351.59	948.42	640.23
3037	*HAS2*	*hyaluronan synthase 2*	306.76	1542.76	217.43
60676	*PAPPA2*	*pappalysin 2*	77.98	227.62	68.48
5587	*PRKD1*	*protein kinase D1*	281.49	821.26	210.26
8710	*SERPINB7*	*serpin peptidase inhibitor, clade B (ovalbumin), member 7*	40.55	161.10	29.01
**Signaling**					
9472	*AKAP6*	*A kinase (PRKA) anchor protein 6*	96.41	228.78	64.73
10123	*ARL4C*	*ADP-ribosylation factor-like 4C*	150.13	331.18	220.70
595	*CCND1*	*cyclin D1*	854.39	1845.44	1093.25
117248	*GALNTL2*	*UDP-N-acetyl-alpha-D-galactosamine:polypeptide N-acetylgalactosaminyltransferase-like 2*	78.33	304.28	54.37

80000	*GREB1L*	*growth regulation by estrogen in breast cancer-like*	68.39	149.22	49.06
81851	*KRTAP1-1*	*keratin associated protein 1-1*	407.75	1030.52	212.39
81872	*KRTAP2-1*	*keratin associated protein 2-1*	963.09	2000.59	488.66
29995	*LMCD1*	*LIM and cysteine-rich domains 1*	470.47	1885.54	467.76
4642	*MYO1D*	*myosin ID*	787.51	1867.04	610.42
57451	*ODZ2*	*odz, odd Oz/ten-m homolog 2 (Drosophila)*	120.85	2989.18	82.22
57381	*RHOJ*	*ras homolog family member J*	110.27	733.84	85.10
**Transcriptional regulation**					
646	*BNC1*	*basonuclin 1*	110.92	302.91	115.19
2119	*ETV5*	*ets variant 5*	278.62	753.87	316.87
3201	*HOXA4*	*homeobox A4*	56.22	197.06	47.74
3203	*HOXA6*	*homeobox A6*	124.56	251.45	94.94
3204	*HOXA7*	*homeobox A7*	97.80	443.06	94.46
3205	*HOXA9*	*homeobox A9*	109.45	453.34	76.16
3212	*HOXB2*	*homeobox B2*	104.86	949.20	92.03
3213	*HOXB3*	*homeobox B3*	95.23	504.78	72.78
3215	*HOXB5*	*homeobox B5*	61.76	186.66	49.10
3216	*HOXB6*	*homeobox B6*	66.54	375.16	50.91
3217	*HOXB7*	*homeobox B7*	56.39	125.71	42.66
3218	*HOXB8*	*homeobox B8*	109.84	301.85	90.79
3219	*HOXB9*	*homeobox B9*	115.73	759.22	87.43
3226	*HOXC10*	*homeobox C10*	80.33	1175.04	59.24
3222	*HOXC5*	*homeobox C5*	46.06	231.32	41.30
3223	*HOXC6*	*homeobox C6*	72.55	771.85	59.02
3224	*HOXC8*	*homeobox C8*	119.99	1081.51	77.57
3225	*HOXC9*	*homeobox C9*	62.23	432.30	51.29
3234	*HOXD8*	*homeobox D8*	138.92	341.93	75.36
3235	*HOXD9*	*homeobox D9*	172.26	377.58	123.78
56956	*LHX9*	*LIM homeobox 9*	95.70	601.44	67.02
137814	*NKX2-6*	*NK2 homeobox 6*	137.03	388.34	103.74
9792	*SERTAD2*	*SERTA domain containing 2*	320.00	822.43	186.38
6910	*TBX5*	*T-box 5*	118.86	770.39	76.33
**miRNA**					
400765	*MIR137HG*	*MIR137 host gene (non-protein coding)*	121.41	256.82	76.12
406973	*MIR196A2*	*microRNA 196a-2*	53.73	126.24	47.60
**Others**					
25841	*ABTB2*	*ankyrin repeat and BTB (POZ) domain containing 2*	186.86	390.65	186.25
79870	*BAALC*	*brain and acute leukemia, cytoplasmic*	112.21	237.19	88.72
285761	*DCBLD1*	*discoidin, CUB and LCCL domain containing 1*	186.00	447.59	181.15
54757	*FAM20A*	*family with sequence similarity 20, member A*	140.73	475.58	163.94
196051	*PPAPDC1A*	*phosphatidic acid phosphatase type 2 domain containing 1A*	171.52	736.63	135.56

***The numbers indicate average signal values in each cell type.**

Supplementary Table S7. Gene list in G4 categorized in Figure 4A.

[hDFs = hOF-iPSCs > hOFs (22 genes)]

Entrez GeneID	*Symbol*	*Gene name*	hOFs*	hDFs*	hOF-iPSCs*
**Membrane protein**					
1948	*EFNB2*	*ephrin-B2*	61.51	255.89	164.79
2861	*GPR37*	*G protein-coupled receptor 37 (endothelin receptor type B-like)*	81.05	186.10	145.51
2890	*GRIA1*	*glutamate receptor, ionotropic, AMPA 1*	86.27	375.72	194.07
58494	*JAM2*	*junctional adhesion molecule 2*	284.11	1189.57	649.12
4978	*OPCML*	*opioid binding protein/cell adhesion molecule-like*	66.32	239.11	126.11
84898	*PLXDC2*	*plexin domain containing 2*	114.84	831.44	1644.58
79845	*RNF122*	*ring finger protein 122*	226.50	469.62	509.37
6507	*SLC1A3*	*solute carrier family 1 (glial high affinity glutamate transporter), member 3*	87.69	223.11	205.04
**Receptor**					
8828	*NRP2*	*neuropilin 2*	163.95	502.96	290.66
114880	*OSBPL6*	*oxysterol binding protein-like 6*	100.30	204.24	265.24
**Enzyme**					
205	*AK4*	*adenylate kinase 4*	130.69	816.18	1043.56
219	*ALDH1B1*	*aldehyde dehydrogenase 1 family, member B1*	1367.53	2750.02	2787.63
481	*ATP1B1*	*ATPase, Na+/K+ transporting, beta 1 polypeptide*	154.65	326.04	580.63
978	*CDA*	*cytidine deaminase*	134.37	474.77	238.77
2026	*ENO2*	*enolase 2 (gamma, neuronal)*	351.59	948.42	640.23
25797	*QPCT*	*glutaminyl-peptide cyclotransferase*	126.52	422.84	293.24
**Signaling**					
79822	*ARHGAP28*	*Rho GTPase activating protein 28*	77.13	212.41	259.33
10123	*ARL4C*	*ADP-ribosylation factor-like 4C*	150.13	331.18	220.70
595	*CCND1*	*cyclin D1*	854.39	1845.44	1093.25
1600	*DAB1*	*disabled homolog 1 (Drosophila)*	90.64	424.81	291.18
55714	*ODZ3*	*odz, odd Oz/ten-m homolog 3 (Drosophila)*	154.87	746.81	1330.60
**Others**					
90768	*MGC45800*	*uncharacterized LOC90768*	137.47	344.63	381.19

***The numbers indicate average signal values in each cell type.**

Supplementary Table S8. Gene list in Figure 4D.

Entrez GeneID	*Symbol*	*Gene name*
2103	*ESRRB*	*estrogen-related receptor beta*
8928	*FOXH1*	*forkhead box H1*
148979	*GLIS1*	*GLIS family zinc finger 1*
79727	*LIN28A*	*lin-28 homolog A (C. elegans)*
53615	*MBD3*	*methyl-CpG binding domain protein 3*
55384	*MEG3*	*maternally expressed 3 (non-protein coding)*
79923	*NANOG*	*Nanog homeobox*
2494	*NR5A2*	*nuclear receptor subfamily 5, group A, member 2*
63978	*PRDM14*	*PR domain containing 14*
5916	*RARG*	*retinoic acid receptor, gamma*
57167	*SALL4*	*spalt-like transcription factor 4*
6926	*TBX3*	*T-box 3*
